# Genome-wide identification of long non-coding RNAs in pigs and their roles in muscle development

**DOI:** 10.5713/ab.260444

**Published:** 2026-07-27

**Authors:** Hang Liu, Hao Wang, Ligang Wang, Yuedong Zhang, Chao Guo, Lin Tao, Minsheng Peng, Zhongyin Zhou, Ruifei Yang, Ya-Ping Zhang

**Affiliations:** 1 State Key Laboratory of Genetic Evolution & Animal Models, Kunming Institute of Zoology, Chinese Academy of Sciences, Kunming, China; 2 Kunming College of Life Science, University of Chinese Academy of Sciences, Kunming, China; 3 School of Life Sciences, Division of Life Sciences and Medicine, University of Science and Technology of China, Hefei, China; 4 Institute of Animal Science, Chinese Academy of Agricultural Sciences, Beijing, China; 5 College of Animal Science and Technology, Yunnan Agricultural University, Kunming, China; 6 Bio-X Center for Interdisciplinary Innovation, Yunnan University, Kunming, China

**Keywords:** Enhancer, Expression Quantitative Trait Locus, Long Non-coding RNA, Muscle Development, Pig

## Abstract

**Objective:**

Long non-coding RNAs (lncRNAs) regulates complex traits in pigs and the systematic annotation and functional roles of these genes remain poorly understood. This study aimed to construct a comprehensive porcine lncRNA atlas and explore the regulatory role of lncRNAs in muscle development.

**Methods:**

We used 8,871 strand-specific RNA-seq datasets to identify porcine lncRNAs. Their transcriptional characteristics, tissue specificity, evolutionary conservation, and expression quantitative trait locus (eQTL)-supported regulatory relationships were esamined. Single-cell RNA-seq, Hi-C, H3K27ac ChIP-seq, and population differentiation analyses were further incorporated to investigate candidate lncRNAs involved in muscle development.

**Results:**

We identified 50,203 porcine lncRNA genes with greater tissue specificity and lower expression levels than protein-coding genes. A subset of lncRNAs showed sequence conservation across mammals. Integration with pigGTEx data identified extensive eQTL-supported lncRNA-mRNA candidate regulatory pairs enriched in active promoter and enhancer chromatin states. Single-cell and multi-omics analyses highlighted *PRANCR* as a candidate regulator of postnatal muscle growth in Laiwu pigs. A highly distinct enhancer variation (chr5:g.34155013T>C; Sscrofa11.1) could affect SOX8 binding and regulate *PRANCR* expression in satellite stem cells.

**Conclusion:**

The current study provides the most comprehensive porcine lncRNA resource currently available and proposes a potential enhancer-lncRNA regulatory mechanism that contributes to muscle development in pigs.

## INTRODUCTION

Long-term natural selection and domestication have driven the drastic diversification of phenotypic traits in pigs (*Sus scrofa*) [1,2]. Genome-wide association studies (GWAS) have mapped genetic variants for diverse porcine quantitative traits, with the vast majority of the significant signals located in non-coding regions, such as intergenic regions and introns [3,4]. These findings indicate that most causal genetic variants do not alter amino acid sequences but instead drive phenotypic divergence by affecting the regulatory functions of non-coding regions. For example, a causal mutation in intron 3 of *IGF2* influences muscle growth [5], and a 6-bp deletion in the promoter region of *MYH3* controls muscle fiber composition [6]. Notably, the non-coding genome can also be transcribed to produce a large number of long non-coding RNAs (lncRNAs). As important regulatory factors, lncRNAs have been shown to play critical regulatory roles in shaping variations for economic traits agricultural animals [7–9]. Therefore, the systematic identification and functional characterization of these lncRNAs is of great significance for elucidating the molecular mechanisms underlying variation of economic traits in pigs.

lncRNAs represent one of the most important classes of transcripts. Compared with mRNAs, lncRNAs are usually expressed at lower levels but exhibit stronger tissue-, cell type-, and developmental stage-specificity [10]. By interacting with DNA, RNA, or proteins, lncRNAs can regulate chromatin modification, transcription of nearby genes, RNA stability, and cell-state transitions [11–13]. In pigs, studies have revealed the regulatory functions of lncRNAs in multiple economic traits. For example, during muscle development, *lnc-RMG* regulates porcine skeletal myogenesis through a highly conserved mechanism [14]. *LOC106504303* promotes energy metabolism by participating in mitochondrial oxidative phosphorylation in liver, while *LOC102160805* enhances digestion and absorption by regulating lipid and cholesterol metabolism in ileum, thereby collectively and positively regulating feed efficiency [15]. Although considerable progress has been made in understanding the functions and mechanisms of lncRNAs over the past decade, the relevance of the vast majority of lncRNAs to complex traits in pigs remains unexplored.

In this study, we integrated large-scale strand-specific transcriptomic datasets to construct a high-confidence lncRNA annotation atlas for the domestic pig. We then systematically characterized the transcriptional features, tissue-specific expression patterns, and cross-species homology conservation of these lncRNAs. We further evaluated their potential roles in local gene regulation by integrating expression quantitative trait locus (eQTL) data. Subsequently, we focused on *MSTRG.33011*, a core candidate lncRNA that is differentially expressed in the myogenic lineage between Laiwu pigs and wild boars. We propose that a highly differentiated genetic variant located in its upstream enhancer may contribute to the molecular regulation underlying the increased proportion of postnatal myogenic progenitor cells and the rapid muscle growth observed in Laiwu pigs. Collectively, these findings provide a robust resource for systematic functional studies of porcine lncRNAs, using this resource, we revealed noncoding regulation of lncRNAs as potential key modulators of economic traits and phenotypic diversity in pigs.

## MATERIALS AND METHODS

### Animals, tissue collection, and sample processing

Pigs used for sequencing were healthy male purebred Canadian Large White pigs obtained from the breeding farm of Chifeng, Inner Mongolia, China. Samples were collected at postnatal day 21, day 50, day 100, and day 180, with two biological replicates. The sampled tissues included brain (prefrontal cortex), heart (apex), liver, spleen, lung, kidney, *longissimus dorsi*, dorsal subcutaneous fat, middle colon, and middle ileum, testis. Additionally, longissimus dorsi muscle samples were obtained from 733 Beijing Black pigs raised at Beijing Heiliu Animal Husbandry Technology. All animals were slaughtered at age 210±40 days. After slaughtering, longissimus dorsi muscle of the last rib was sampled. Approximately 200 mg of tissue was collected from each site and cut into ~50 mg pieces. Tissue samples were immediately immersed in 1 ml of RNAlater stabilization solution (AM7024; Invitrogen) after dissection and incubated overnight at 4°C. After incubation, samples were transferred to a −80°C freezer for long-term storage.

### Total RNA extraction, library construction, and sequencing

Total RNA was extracted from porcine tissue samples using TRIzol Reagent (Invitrogen) according to the manufacturer’s instructions. RNA quality and concentration were assessed using a NanoDrop 2000 spectrophotometer and an Agilent 2100 Bioanalyzer. Only samples with RNA integrity number (RIN)≥7.0 were used for library construction.

For the Canadian Large White pigs and two PK15 cell line samples, strand-specific RNA-seq libraries were prepared using the Ribo-Zero Gold rRNA Removal Kit (Illumina) and sequenced on the Illumina NovaSeq 6000 platform in paired-end 150-bp mode (PE150). For the longissimus dorsi muscle samples from 733 Beijing Black pigs, strand-specific RNA-seq libraries were prepared using the TruSeq Stranded mRNA Library Prep Kit (Illumina). In total, this study generated a total of 823 new RNA-seq datasets (Supplement 1).

### Processing of public RNA-seq data, transcript assembly, and long non-coding RNA identification

In total, 23,152 public RNA-seq datasets (16,456 biological samples) were downloaded from NCBI SRA (Supplements 1, 2). To eliminate the influence of sequencing technology, only Illumina data were retained. Raw SRA files were converted to FASTQ using SRA Toolkit (v3.1.1) and trimmed with Trimmomatic (v0.39).

Strand-specific RNA-seq is essential for lncRNA annotation, as it uniquely resolves overlapping transcripts in dense genomic regions. To evaluate the strand specificity of public data, strand specificity was determined using known orientation samples (733 porcine muscle samples from this study and 36 pig brain samples [16]), as shown in Supplement 1. For each sample, read1 and read2 were treated as single-end reads and independently aligned to the reference transcriptome using Bowtie2 (v2.3.4.3). Sense/antisense read counts were obtained with SAMtools (v1.17). A sample was defined as having clear orientation if ≥80% of reads aligned to one strand. Others were classified as non-strand-specific or ambiguous.

Only samples with clear orientation were retained. Clean reads were aligned to the Sus scrofa 11.1 genome using Hisat2 (v2.2.1). Gene expression levels were quantified with StringTie (v2.1.2) based on Ensembl v104 annotation. Quality control criteria: ≥500,000 clean reads, uniquely mapped read rate ≥60%, and ≥20% of annotated genes with transcripts per million (TPM)≥0.1. Passing samples were further subjected to tissue-specific t-SNE clustering to remove outliers, yielding 8,871 high-quality samples for assembly and lncRNA identification (Supplement 3).

A multi-stage pipeline was established for high-confidence lncRNA identification from multi-tissue RNA-seq data [17]. StringTie (v2.1.2) performed per-sample assembly; RegTools verified junction strand orientations, retaining only junctions with consistent direction across samples and ≥3 spanning reads. Transcripts were merged within and across tissues. Expression filtering (TPM≥0.1 in ≥1 sample) and Cuffcompare class-code selection (u, i, j, x, o, s) were applied. A Transcript Confidence Score (TCS) was calculated using artificial pseudogene controls. Coding potential was assessed by CPC2 (v0.1), CNCI (v2), and PLEK (v1.2); only consensus non-coding transcripts were retained, followed by Pfam scanning and removal of same-strand coding-gene overlaps. Final annotations were merged using StringTie merge. Gene expression levels were quantified by StringTie based on final annotations.

### Sample clustering analysis based on gene expression profiles

Gene expression matrices for all samples were transformed using log_2_ (TPM+1). Batch effects were corrected using the removeBatchEffect function in the limma package, with sequencing project ID specified as the batch variable. Pearson correlation coefficients were used to measure expression similarity between samples. t-SNE dimensionality reduction was then performed to visualize the global relationships among samples (input_perplexity = 30).

### Comparison with existing porcine long non-coding RNA annotations

Cuffcompare (v2.2.1) was used to analyze the genomic positional relationships between lncRNAs and protein-coding genes. Our assembled lncRNA transcripts were compared with previously published porcine lncRNA datasets generated by the next-generation sequencing [18] and the third-generation sequencing [19], and matched according to the class codes reported by Cuffcompare. For intronic lncRNAs with class code “i”, comparisons were restricted to transcripts also classified as “i” in the external datasets. If the corresponding transcript pair showed a class code of “=”, “c”, “j”, “e”, or “o”, the lncRNA was considered to be recapitulated by our annotation.

### Analysis of epigenetic signals around long non-coding RNA transcription start sites

Porcine ATAC-seq, H3K27ac ChIP-seq, and H3K4me3 ChIP-seq data (GSE143288) [20] were integrated for epigenetic analysis. The transcription start site (TSS) of each lncRNA transcript was extracted, and a ±10 kb window centered on the TSS was generated. Overlap between epigenetic peaks and TSS windows was calculated using bedtools intersect (v2.29.0). 10,000 random TSS positions were generated across the genome as background intervals.

### Quantification of tissue expression levels and calculation of the tissue specificity index

The expression level of a gene in a given tissue was defined as the mean TPM value across samples in which the gene was detected (TPM≥0.1) in that tissue. The same criterion was applied to both lncRNAs and protein-coding genes. Only tissues represented by at least 20% of the total sample number were included in the analysis. The tissue specificity index, τ, was calculated based on mean expression levels across tissues [21].

### Enrichment analysis of protein-coding genes neighboring long non-coding RNAs

Based on the expression profiles of the 10 core tissues described above, lncRNAs were divided into tissue-specific lncRNAs (expression breadth = 1) and broadly expressed lncRNAs (expression breadth = 10). Protein-coding genes located within ±1 Mb of each lncRNA TSS were used. The protein-coding genes were further grouped into categories corresponding to expression in 0–10 tissues. Their distributions were then summarized across six distance intervals: <1 kb, 1–10 kb, 10–100 kb, 100–200 kb, 200–500 kb, and 500 kb–1 Mb. A hypergeometric test was used to evaluate whether tissue-specific lncRNAs or broadly expressed lncRNAs were enriched for neighboring protein-coding genes from particular expression-breadth categories. The background set was defined as all protein-coding genes located within ±1 Mb of lncRNA TSSs.

### Identification of homologous long non-coding RNAs

Cross-species genomic coordinate conversion was performed using the UCSC *liftOver* tool together with the corresponding chain files (*susScr11ToHg38.over.chain.gz* and *susScr11ToMm10.over.chain.gz*) to map porcine lncRNA genomic coordinates from *Sus scrofa* 11.1 to the human (hg38, GENCODE v48) and mouse (mm10, GENCODE v48) genomes. Exon regions of lncRNAs were converted. The *liftOver* parameter minMatch was set to 0.1. Within the set of positionally homologous candidates, further sequence-level validation was performed using BLASTN (v2.10.0). Exonic sequences of porcine and human lncRNAs were extracted, and local alignment mode was used for sequence homology analysis. The E-value threshold was set to 1e-5.

### Long non-coding RNA conservation analysis

PhastCons conservation scores for pig exon regions that had been lifted over to the human genome (hg38) were obtained from the UCSC Genome Browser. Mean exonic PhastCons values were then calculated. Intergenic regions were defined as genomic segments that did not overlap any annotated genes, and 10,000 fixed-length (400 bp) fragments were generated by unbiased random sampling to serve as background.

### Analysis of pigGTEx expression quantitative trait locus-supported long non-coding RNA-mRNA pairs

mRNA eQTL SNPs from pigGTEx across 21 tissues were integrated [22]. A lncRNA-mRNA pair was defined as eQTL-linked if an eQTL SNP for the mRNA overlapped the genomic region of the lncRNA. Two types of random controls were generated for each eQTL-linked pair: (1) trans random controls, by selecting the same number of lncRNA-mRNA combinations from different chromosomes; (2) matched cis random controls, by selecting on the same chromosome of lncRNA-mRNA pair without eQTL linkage but matched for linear distance and transcriptional orientation. We downloaded the topologically associating domains (TADs) data of 11 tissues from the PigMatrix database ( https://pigmatrix.kiz.ac.cn/).

### Long non-coding RNA-mRNA co-expression analysis and evaluation of expression quantitative trait locus support strength

Spearman rank correlation analysis was used to calculate the expression correlation coefficient for each pair in each tissue, and co-expression strength was represented by the absolute Spearman correlation coefficient (absolute Spearman’s ρ). According to the physical distance between the TSSs of each lncRNA and its paired mRNA, all pairs were divided into six distance bins: 1 kb, 10 kb, 100 kb, 200 kb, 500 kb, and 1 Mb.

For each eQTL-linked pair, we further counted the number of eQTL SNPs located within the lncRNA region that supported the same mRNA. Pairs were then grouped into six categories according to the number of supporting SNPs: 1, 5, 10, 15, 20, and ≥25. Significance was evaluated using independent Wilcoxon rank-sum tests.

### Functional annotation and analysis of long non-coding RNAs based on epigenetic chromatin states

Five tissues (adipose, liver, lung, muscle, and spleen) in GSE158430 were integrated to classify eQTL-supported lncRNAs into functional categories [23]. If the full-length lncRNA region overlapped a TssA or TssBiv state, it was defined as a p-lncRNA. If it overlapped an EnhA or EnhAMe state, it was defined as an e-lncRNA. All remaining lncRNAs were classified as other lncRNAs.

### Preprocessing of single-cell transcriptomic data and annotation of skeletal muscle cell subpopulations

Public single-cell RNA-seq data from postnatal day 2 longissimus dorsi muscle of Laiwu pigs and wild boars (three biological replicates per group; Supplement 1) were analyzed [24]. Raw data were aligned and quantified with CellRanger (v7.1.0) using our expanded Sus scrofa 11.1 annotation. Doublets were removed with Scrublet. Quality control in Seurat (v5.0) retained cells with 200–5,000 detected gene and <10% mitochondrial reads.

After LogNormalize normalization, highly variable gene identification, and scaling, PCA, nearest-neighbor graph construction, Louvain clustering, and UMAP reduction were performed sequentially. Skeletal muscle-related cells were extracted from the full dataset and re-annotated based on known myogenic lineage markers (Supplement 4). Cell-type differential expression analysis was performed using muscat (v1.16.0). Cells were grouped by breed (Laiwu pig vs. wild boar), and raw counts were aggregated by cell type and biological sample to generate pseudobulk profiles. Differential expression was tested using edgeR via pbDS, with significance defined as p<0.05. Candidate gene expression across subpopulations was visualized with DotPlot.

### Integration of H3K27ac, H3K4me3 and Hi-C data to localize candidate regulatory regions of *PRANCR*

H3K27ac and H3K4me3 data from muscle tissue [20] and Hi-C data ( https://pigmatrix.kiz.ac.cn/) were integrated. H3K27ac peaks defined active enhancers/promoters; H3K4me3 peaks specifically marked promoter regions. Hi-C identified chromatin interactions between candidate SNP-containing regions and lncRNA promoters/gene bodies. Whole-genome resequencing data from domestic pigs were further integrated. Potential regulatory SNPs met three criteria: |ΔAF|>0.8, located within an H3K27ac peak, and connected to the target lncRNA region by a Hi-C loop. Two qualified sites (chr5:g.34154984 C>T and chr5:g.34155013 T>C) were identified in the upstream enhancer of PRANCR (MSTRG.33011). These SNPs, H3K27ac peaks, H3K4me3 peaks, Hi-C loops, and strand-specific RNA-seq signals were visualized using pyGenomeTracks (v3.9).

For each variant, 41 bp upstream/downstream sequences (reference vs. alternative) were extracted. Motif binding analysis used JASPAR 2024 (scan function), restricting to transcription factors with expression proportion <5% in satellite cells and >10% in satellite stem cells.

## RESULTS

### Assessment and characterization of the porcine long non-coding RNAs

We used 8,871 strand-specific RNA-seq datasets (823 were generated for this study) spanning 32 tissues and cell types, including 823 newly generated in this study (see Methods), and evaluated the reliability of these newly annotated lncRNAs (Figures 1A, 1B and Supplement 3). t-SNE clustering based on the lncRNA expression matrix clearly separated samples by tissue and cell type (Figure 1C). This tissue-specific clustering pattern was highly consistent with that obtained using either all genes or protein-coding genes alone (Supplements 5A, 5B). Compared with previous annotations [18,19], we found that our study not only recapitulated approximately 20%–50% of previously reported lncRNAs, but also substantially expanded the current annotation across multiple genomic categories. Additionally, the TSSs of the newly annotated lncRNAs showed active chromatin enrichment (ATAC, H3K27ac, H3K4me3), similar to known lncRNAs and above background, though lower than protein-coding genes (Figure 1E).

Based on this high-confidence annotation set, we further performed a systematic characterization of porcine lncRNAs. At the sequence and gene-structure levels, compared with protein-coding genes, lncRNAs generally exhibited fewer isoforms, fewer exons, shorter transcript length, and lower GC content (Supplements 5C–5F). At the transcriptional level, the overall expression abundance of lncRNAs was significantly lower than that of protein-coding genes (Supplement 5G). Conversely, the tissue specificity index (τ) showed that lncRNAs displayed much stronger tissue-specific expression than protein-coding genes (Supplement 5H). In addition, lncRNAs exhibited greater expression variability across samples (Supplement 5I), and the number of detectable lncRNA genes declined much more rapidly than that of protein-coding genes as the reproducibility threshold increased (Supplement 5J), indicating lower cross-sample reproducibility for lncRNA detection.

### Expression breadth of porcine long non-coding RNAs and their potential local regulatory modes

To infer the potential biological functions of lncRNAs from their tissue expression breadth, we first systematically analyzed the expression breadth of 50,203 porcine lncRNA genes across different tissues. We selected RNA-seq datasets from Canadian Large White pigs to ensure consistency in sequencing depth, library preparation, and tissue processing across samples, thereby improving the comparability of gene expression estimates across tissues. The results showed that the majority of the lncRNAs (75.2%) were detectable in at least one tissue. Further analysis revealed that the expression breadth of the lncRNAs followed an u-shaped distribution (Figure 2A, left). One group of lncRNAs was expressed in only a single tissue, whereas another group was expressed in most or even all examined tissues (Figure 2A, left). In contrast, protein-coding genes showed a similar overall pattern, but were more strongly biased toward broad expression across all tissues (Figure 2A, right).

As the number of expressing tissues increased, the expression levels of both lncRNAs and protein-coding genes showed an overall upward trend, but more rapidly for the latter (Figure 2B). Further analysis of tissue-specific lncRNAs showed that, among the 10 examined tissues, the brain contained the largest number of enriched tissue-specific lncRNAs (2,352 in total), whereas relatively few tissue-specific lncRNAs were detected in muscle (Figure 2C).

Previous studies have shown that some lncRNA loci can regulate the expression of neighboring genes in cis within a local genomic region [25]. Therefore, we further used the hypergeometric test to analyze the enrichment of protein-coding genes with different expression breadths. The results showed that tissue-specific lncRNAs were more likely to be located in close proximity (<100 kb) to protein-coding genes that are broadly expressed across many tissues (≥8 tissues) (Figure 2D). In contrast, no similarly pronounced pattern was observed for broadly expressed lncRNAs.

### Cross-species homology and conservation analysis of porcine long non-coding RNAs

We next conducted cross-species homology and conservation analyses of porcine lncRNAs with them of human and mouse. Of 50,203 porcine lncRNAs, 17,020 (33.9%) mapped to positionally homologous human regions (12,425 human lncRNAs), and 8,732 (17.4%) to mouse regions (6,994 mouse lncRNAs). Among the porcine lncRNAs successfully mapped to the human genome, a substantial proportion showed overlap at the exon level (Figure 3A). Specifically, approximately two-thirds of the gene-body overlap events extended to exonic overlap, and the numbers of lncRNAs involved in exon-overlapping relationships were comparable between pigs and humans (8,329 and 8,199, respectively). Further BLASTN alignment showed that about 60% of these exon-overlapping cases had direct sequence-level support, corresponding to 5,207 porcine lncRNAs and 5,239 human lncRNAs (Figure 3A). On this basis, we further compared PhastCons conservation scores. Both known and newly annotated lncRNAs exhibited markedly lower PhastCons scores than protein-coding genes, but consistently higher scores than intergenic regions (Figure 3B).

We further examined the relationship between tissue expression breadth and conservation of porcine lncRNAs. As the number of expressing tissues increased, the proportion of porcine lncRNAs with exon-homologous sequence support in the human genome, as identified by BLASTN, showed an overall upward trend (Figure 3C). Likewise, PhastCons conservation scores also increased with expression breadth (Figure 3D). In contrast, tissue-specific lncRNAs showed lower overall conservation, but their conservation levels remained higher than those of intergenic background sequences (Figure 3D).

### mRNA expression quantitative trait locus-overlap-based prioritization of candidate local long non-coding RNA–mRNA associations

To identify such candidate local lncRNA–mRNA associations in pigs, we integrated the eQTL resource across 21 tissues [22] and identified lncRNAs overlapping mRNA eQTL SNPs. In total, we detected 16,495 lncRNAs linked to mRNA eQTL SNPs, accounting for 33.27% of all porcine lncRNAs (Figure 4A). To further assess the spatial context of these candidate associations, we integrated Hi-C chromatin interaction data and found that, on average, 54.9% of the eQTL-overlap-based lncRNA–mRNA pairs were located within the same TAD (range: 47.6%–60.3% across tissues, Supplement 6), indicating that these candidate pairs largely reside in shared 3D chromatin regulatory units. To evaluate whether the eQTL-based pairing strategy was superior to inference based solely on linear proximity, we designed two types of reference controls. We then compared multi-tissue expression correlations between eQTL-linked pairs and these two control sets. The results showed that, at very short distances, the expression correlation of eQTL-linked pairs did not differ significantly from that of matched cis random pairs. However, once the linear distance exceeded 10 kb, eQTL-linked pairs showed significantly stronger correlations than the matched cis random controls (Figure 4B). Furthermore, among eQTL-linked pairs, the greater the number of SNPs supporting the same lncRNA-mRNA connection, the stronger the expression correlation between the two genes (Figure 4C).

Because different classes of lncRNAs may exhibit distinct regulatory potentials, we further classified eQTL-anchored lncRNAs according to active chromatin states (Figure 4D) [23]. We then compared the expression correlations between different lncRNA classes and their eQTL-linked mRNAs. Overall, p-lncRNA-linked pairs showed the strongest expression correlations, followed by e-lncRNA-linked pairs, whereas other lncRNA classes showed the weakest correlations. This difference remained evident even across longer cis distances (Figure 4E). A similar pattern was observed when examining the relationship between the number of supporting SNPs and expression correlation, as the number of supporting SNPs increased, p-lncRNA-linked pairs consistently showed the highest correlations, followed by e-lncRNA-linked pairs, while other lncRNA classes remained weaker overall (Figure 4F).

### *PRANCR* may regulate rapid postnatal muscle growth in Laiwu pigs

Compared with wild boars, domestic pigs show a pattern of skeletal muscle development with relatively delayed prenatal myogenesis but accelerated postnatal muscle growth [26,27]. To further determine whether these differences in cellular composition are associated with genetic regulation, we performed differential expression analysis of lncRNAs across eight cell types in the postnatal day 2 longissimus dorsi of Laiwu pigs and wild boars (Supplement 7A and Table 1). Across these eight cell types, a total of 12–211 differentially expressed lncRNAs were identified (Supplement 8). Notably, among them, 2–60 differentially expressed lncRNAs had eQTL-linked protein-coding genes that were also differentially expressed in the same cell type, suggesting that these lncRNAs may participate in regulating the expression of nearby protein-coding genes through cis-acting mechanism.

On this basis, we further integrated Hi-C and H3K27ac data to examine whether highly differentiated SNPs between Laiwu pigs and wild boars (ΔAF: allele frequency difference>0.8) might be involved in regulating these differentially expressed lncRNAs. The integrated analyses suggested that two highly differentiated SNPs on chr5 (Sscrofa11.1 chr5: g.34154984C>T and chr5:g.34155013T>C) may influence local enhancer activity and, through a chromatin loop, may regulate the lncRNA *PRANCR* (*MSTRG.33011*) (Figures 5A, 5B, Supplement 7B). Thus, highly differentiated variants may contribute to altered *PRANCR* expression, which in turn may contribute to increased postnatal progenitor cells and contributing to rapid muscle growth in Laiwu pigs.

To further assess whether *PRANCR* may be involved in regulating postnatal progenitor cell abundance in pigs, we compared its expression patterns across the eight cell types and further analyzed potential differences in transcription factor binding at chr5:g.34154984 and chr5:g.34155013. *PRANCR* was expressed in two satellite cells, two myogenic cells, and satellite stem cells, but showed significant differential expression between Laiwu pigs and wild boars only in satellite stem cells. This suggests that the regulatory differences at this locus may be related to muscle stem cell-specific transcription factor binding (Figure 5C). Among transcription factors meeting expression criteria (<5% in satellite cells, >10% in satellite stem cells) and predicted to bind either SNP, *SOX8* (involved in myogenesis) was predicted to bind chr5:g.34155013, with genotype affecting binding affinity (Figure 5D).

## DISCUSSION

In this study, we integrated 16,456 public RNA-seq samples (SRA, up to May 2023) and applied stringent strand-specificity filters, yielding 8,871 high-quality samples across 32 tissues/cell types. This provided, to our knowledge, the most comprehensive coverage currently available for the porcine transcriptome. Based on this large-scale resource, and following the general strategy used for lncRNA identification in the human GTEx project [17], we annotated 50,203 lncRNA genes encoding 95,385 transcripts in the domestic pig genome. It approaches the scale of current human lncRNA annotations (GENCODE v49: 35,899 lncRNAs; Ensembl release 115: 35,042; NONCODE v7: 96,411). We further validated the reliability of this annotation through t-SNE clustering of the global transcriptomic landscape, overlap analysis with previously reported lncRNA annotations, and enrichment analysis of activating epigenetic marks around TSSs. Together, these results support the high reliability of our annotation set.

This study therefore provides the most comprehensive porcine lncRNA resource to date, covering both genomic-position-based categories and function-based categories, including e-lncRNAs and p-lncRNAs. Beyond substantially expanding the noncoding annotation of the pig genome, this resource also provides an important foundation for subsequent prioritization of functional lncRNAs. Human GTEx and related multi-tissue studies have shown that expression level alone, or simple annotation based on neighboring genes, is insufficient to identify truly functional lncRNAs from the vast number of candidates [28,29]. Instead, integrating tissue-specific expression, genetic regulatory information, the activity of nearby cis-regulatory elements, and trait-associated signals provides a more comprehensive strategy for prioritizing candidate lncRNAs [28,30]. This resource enables future integration with epigenomic, chromatin interaction, and selection/QTL data to prioritize candidate lncRNAs [20,31,32]. It should be noted that the e-lncRNAs and p-lncRNAs that show tissue- or cell-specific expression, reside in active cis-regulatory regions, display co-expression or spatial interaction with target genes, and colocalize with economically important traits or selection signals, followed by functional validation.

LncRNAs showed lower cross-tissue reproducibility and higher expression variability than protein-coding genes, indicating stronger spatiotemporal specificity, consistent with previous reports [33–36]. This reinforces the view that lncRNAs play indispensable roles in maintaining cell identity and cellular state diversity. When examining the relationship between lncRNA conservation and expression breadth, we found that lncRNAs expressed in fewer tissue types generally showed lower sequence conservation. This suggests that lncRNAs involved in fundamental cellular processes or housekeeping regulation are subject to stronger evolutionary constraint and are therefore more conserved, in agreement with the general patterns of lncRNA expression evolution reported in humans and other tetrapods [37]. Taken together, these two observations suggest that rapidly evolving and weakly conserved lncRNAs may represent the very molecules that contribute to pig-specific cell types and physiological states. Accordingly, future efforts to identify important functional lncRNAs in pigs should not rely solely on cross-species sequence conservation as a primary criterion, but should instead prioritize lineage-specific lncRNAs active in key developmental windows, critical cell lineages, and phenotype-relevant tissues.

As one of the world’s most important agricultural livestock species, domestic pigs are a major source of high-quality animal protein, and muscle growth and meat-production traits therefore represent some of the most important economic traits in pig breeding. Previous studies have demonstrated that several key lncRNAs, including *H19* [38], *MEG3* [39], and *lnc-RMG* [14], directly regulate porcine muscle development. Single-cell sequencing now enables resolving cellular heterogeneity of muscle development at high resolution, facilitating precise investigation of the molecular basis of muscle-trait evolution. Here, we used the single-cell dataset generated by Xu et al from Laiwu pigs and wild boars to focus on subtle differences during early muscle development [24]. Our analysis suggested that lncRNAs differentially expressed in myogenic lineages, such as *MSTRG.33011*, the porcine homolog of human *PRANCR*, may be associated with the increased proportion of postnatal progenitor cells in Laiwu pigs, thus providing a potential cellular explanation for rapid postnatal muscle growth in domestic pigs. Through integrative multi-omics analysis, we further proposed a model in which noncoding genetic variation drives phenotypic divergence. A highly differentiated variant (chr5:g.34155013T>C) located within an upstream enhancer region of a specific lncRNA (*PRANCR*) may alter the binding affinity of the key myogenic transcription factor *SOX8*, thereby affecting the transcriptional activity and expression of this lncRNA. Although this putative regulatory model linking noncoding variation, cis-regulatory elements, lncRNA activity, and cell state still requires definitive validation through future molecular experiments, the analytical strategy adopted in this study is highly consistent with current mainstream multi-omics research frameworks [6,40]. This single-cell-based strategy for multi-omics integration and candidate regulatory inference not only provides valuable candidate targets for future functional investigation of noncoding elements involved in economically important traits in pigs, but also offers a general analytical framework for prioritizing candidate regulatory mechanisms of complex traits in agricultural animals.

Nevertheless, several limitations should be acknowledged. First, the single-cell analyses presented here were based on a single publicly available dataset, and independent validation in additional cohorts will be required to evaluate the generalizability of these findings. Second, the proposed regulatory mechanism linking the enhancer variant, SOX8 binding, and PRANCR expression is currently supported by integrative computational analyses, including chromatin interaction, epigenomic annotation, and transcription factor motif prediction. Although these complementary datasets provide converging evidence, direct experimental validation using approaches such as ChIP-qPCR, EMSA, and luciferase reporter assays will be necessary to establish the underlying molecular mechanism.

## CONCLUSION

In this study, we integrated large-scale strand-specific RNA-seq data to establish a comprehensive porcine lncRNA annotation comprising 50,203 genes. These lncRNAs show higher tissue specificity and lower expression abundance than protein-coding genes, with a subset conserved across mammals. Integration with pigGTEx eQTL data revealed extensive lncRNA-mRNA regulatory pairs enriched in active promoter and enhancer states. Using single-cell transcriptomics, multi-omics, and population differentiation analyses, we identified *PRANCR* (*MSTRG.33011*) as a candidate regulator of postnatal muscle growth in Laiwu pigs, where a highly differentiated enhancer variant (chr5:g.34155013T>C) may alter *SOX8* binding and modulate *PRANCR* expression in satellite stem cells. Together, this work provides a high-quality lncRNA atlas and a multi-omics framework for dissecting the roles of non-coding variants and lncRNAs in pig economic traits.

## Figures and Tables

**Figure 1:**
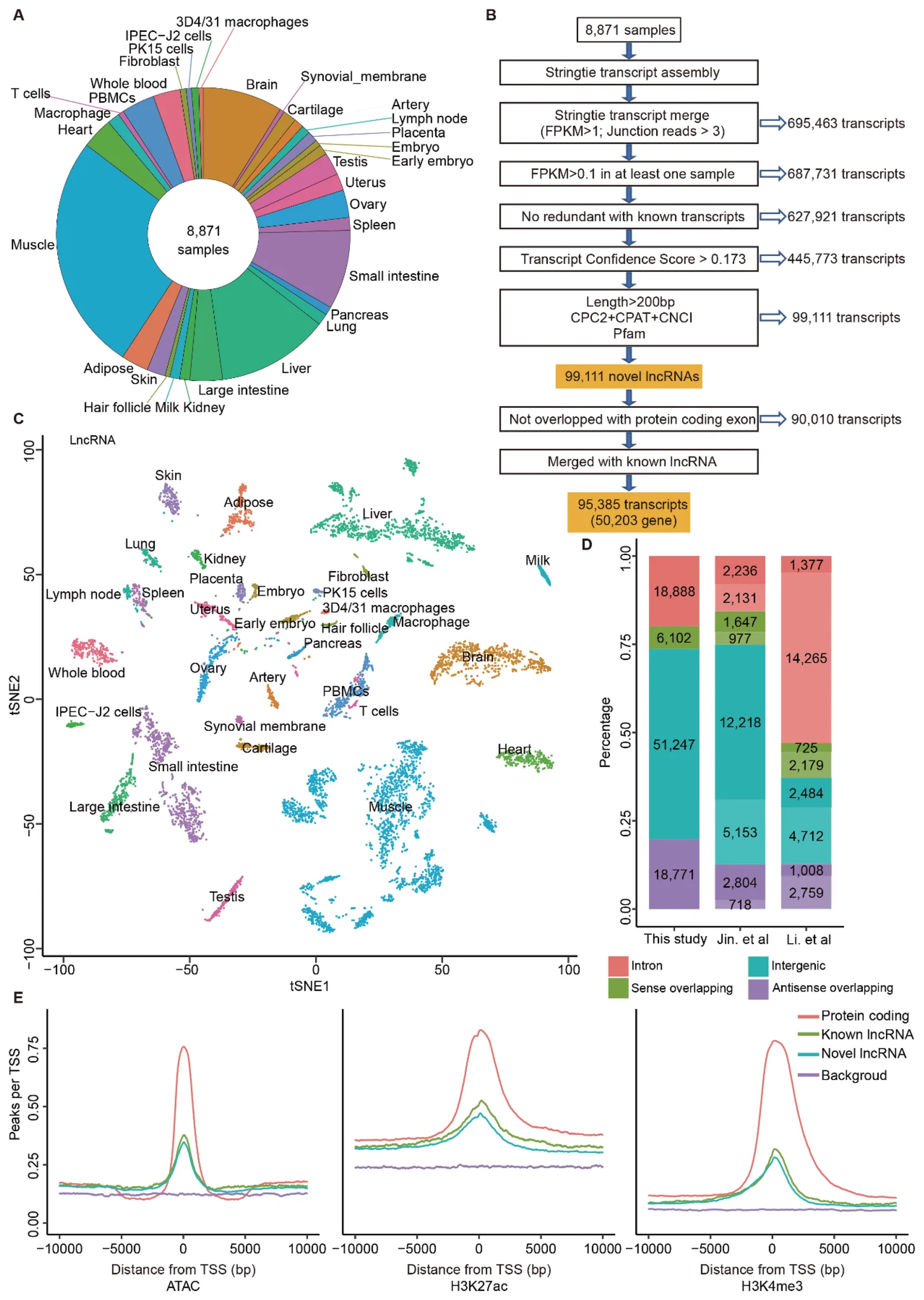
Annotation and reliability analysis of lncRNAs. (A) Number of samples per tissue. (B) Workflow of lncRNA annotation and filtering. (C) Tissue clustering based on lncRNA expression levels. (D) Comparison with previously published annotations, where lighter colors indicate the number of non-reproduced transcripts. (E) Enrichment analysis of activating epigenetic modifications within the 10 kb regions upstream and downstream of lncRNA and protein TSSs. lncRNA, long non-coding RNA; TSS, transcription start site.

**Figure 2:**
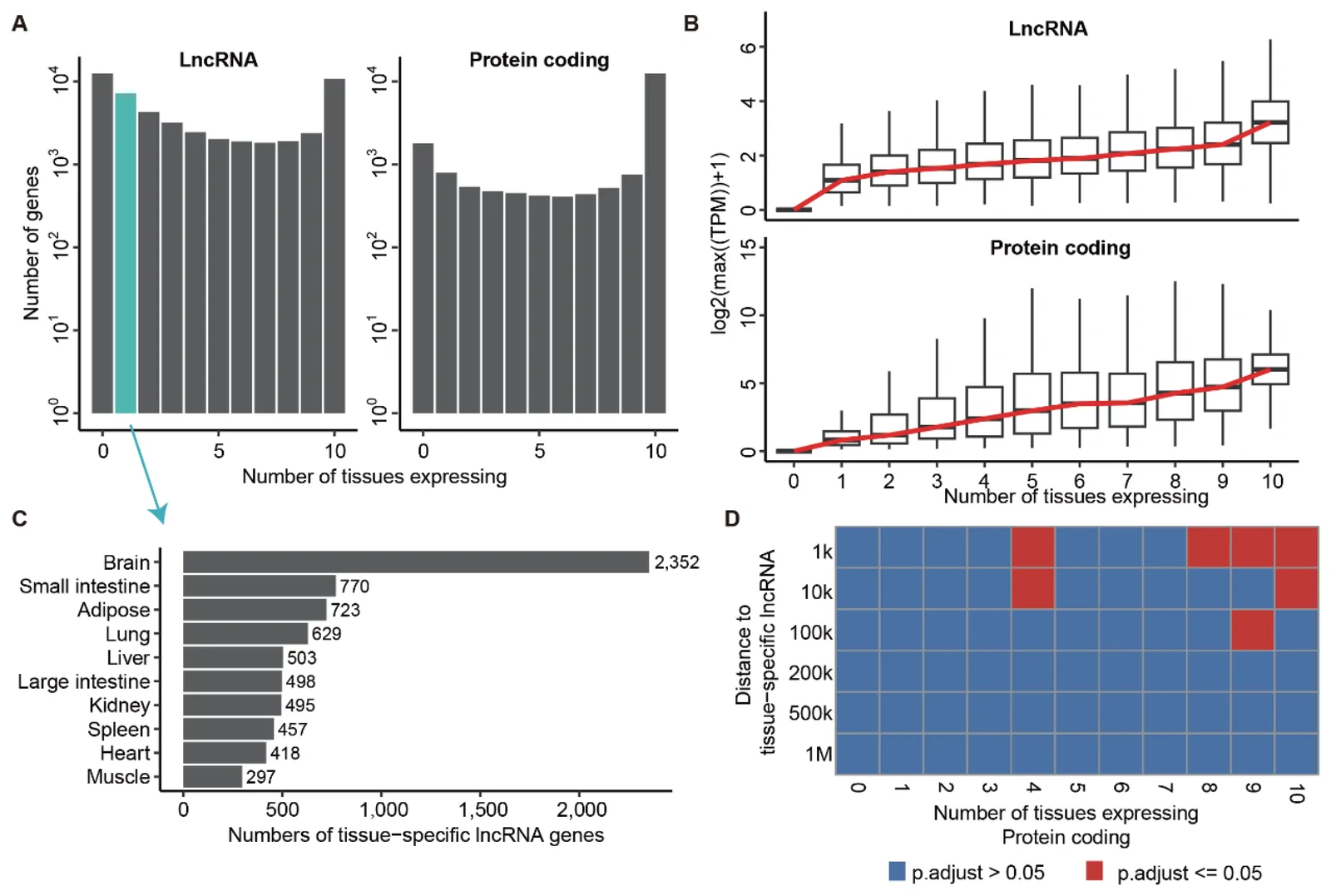
Tissue expression patterns of lncRNAs. (A) Distribution of expression breadth across tissues for lncRNAs (left) and protein-coding genes (right). (B) Relationship between expression breadth and maximum expression level. Spearman correlation analysis reveals an overall positive correlation. (C) Number of tissue-specific lncRNAs in each tissue. (D) Enrichment of protein-coding genes with varying expression breadths within the 1 Mb regions upstream and downstream of tissue-specific lncRNAs. lncRNA, long non-coding RNA.

**Figure 3:**
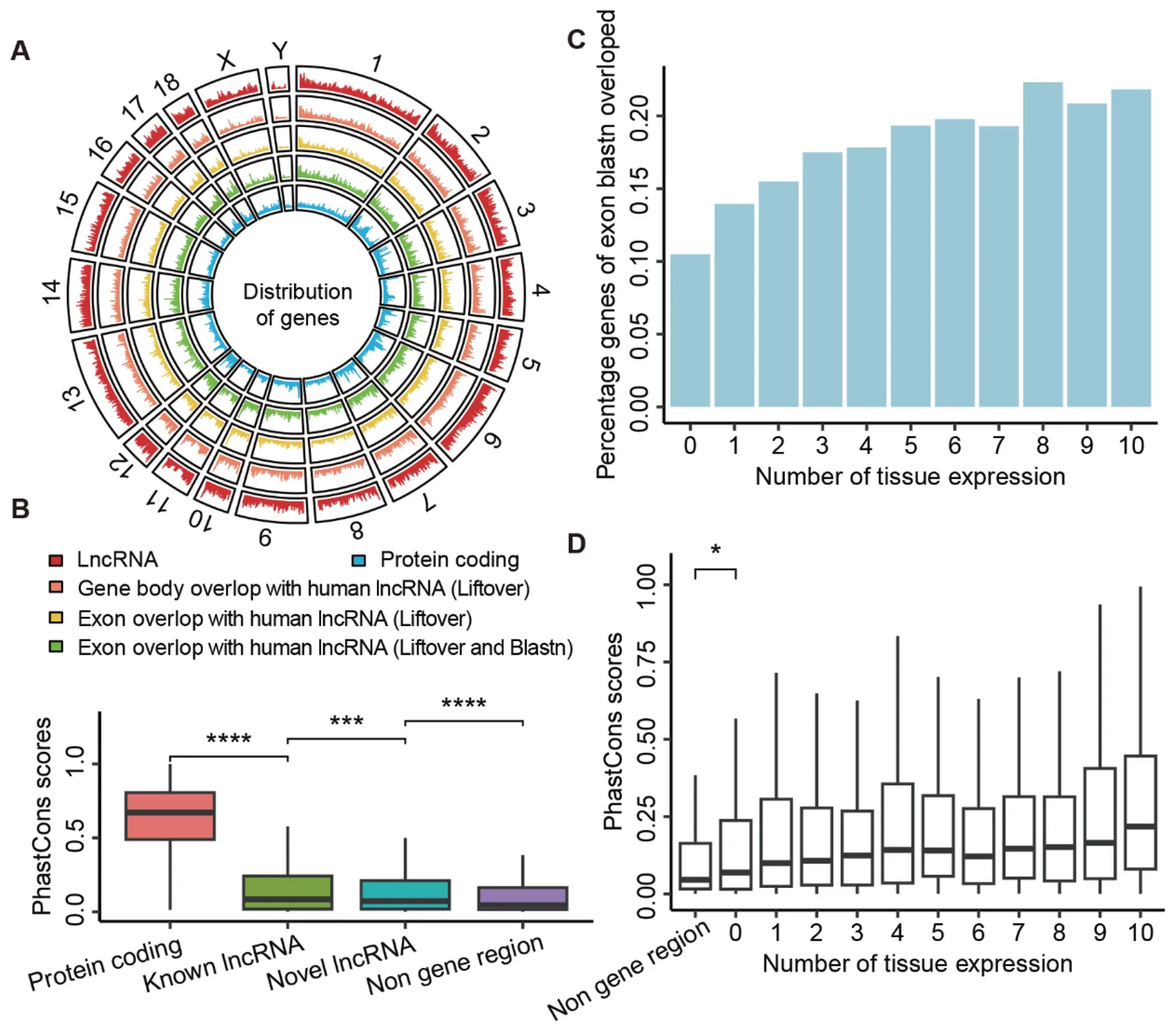
Cross-species conservation of lncRNAs. (A) Genomic distribution of lncRNAs and protein-coding genes. From inner to outer rings: protein-coding genes; lncRNAs with both exon position and sequence conservation; lncRNAs with only exon position conservation; lncRNAs with only gene locus conservation; and all lncRNAs. (B) Comparison of conservation levels between lncRNAs and protein-coding genes. (C) Relationship between the proportion of lncRNAs with conserved exon positions and sequences and their expression breadth. (D) Relationship between conservation level and expression breadth. * p<0.05, *** p<0.001, **** p<0.0001; ns indicates p>0.05. Significance was determined by Wilcoxon rank-sum test. lncRNA, long non-coding RNA.

**Figure 4:**
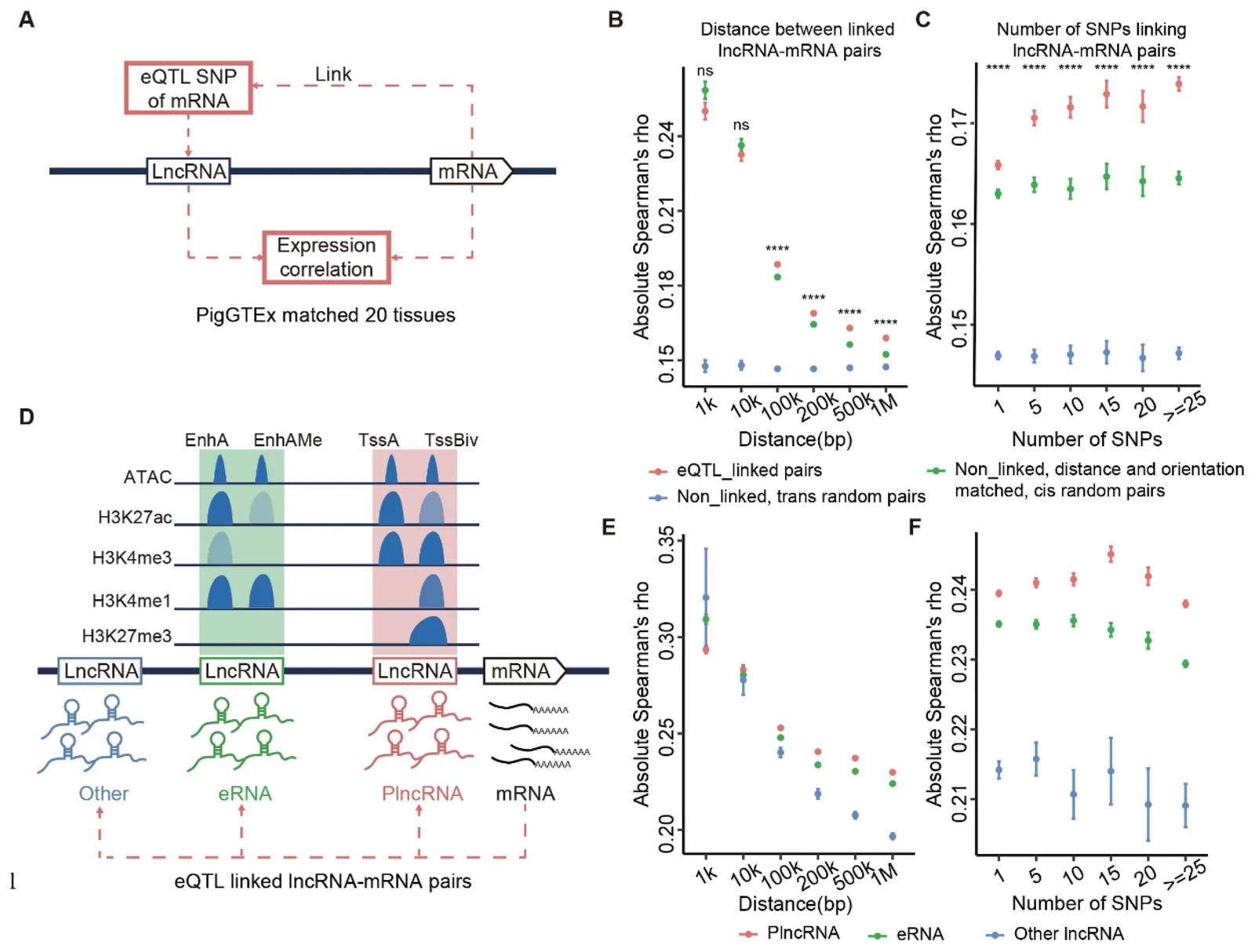
mRNA eQTL-overlap-based prioritization of candidate local lncRNA–mRNA associations. (A) Rationale of the analysis. (B, C) Expression correlation of lncRNA-mRNA pairs binned based on the distance between the pair (B) and the number of eQTL-associated SNPs linking the pair (C). (D) Rationale for lncRNA classification. (E, F) Expression correlation of different types of lncRNA-mRNA pairs, binned based on the distance between the pair (E) and the number of eQTL-associated SNPs linking the pair (F), respectively. Circles represent the mean absolute Spearman’s rho, and error bars indicate their 99.99% confidence intervals. **** p<0.0001, ns indicates p>0.05. Significance was determined by t-test. eQTL, expression quantitative trait locus; lncRNA, long non-coding RNA.

**Figure 5:**
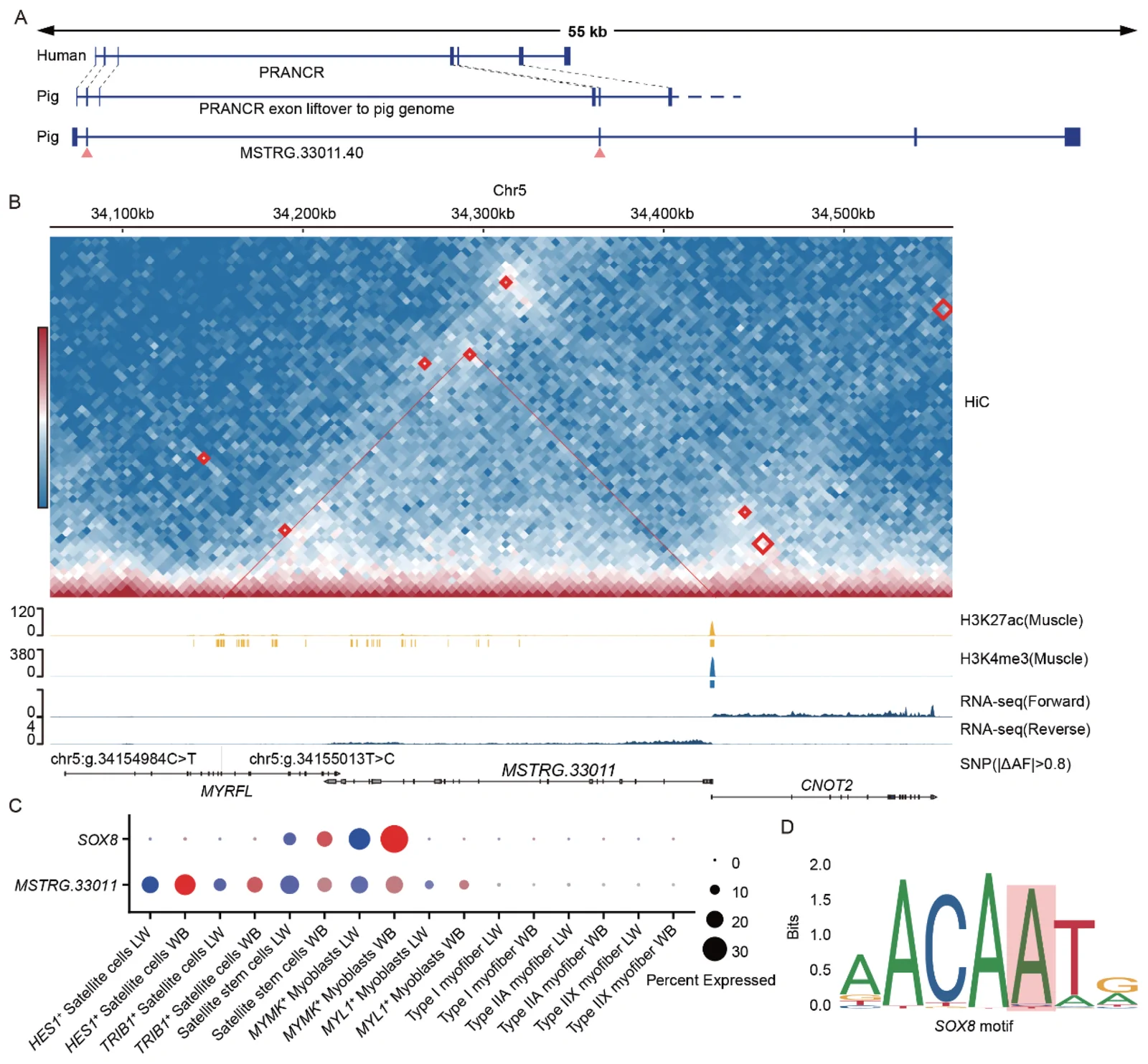
Functional analysis of *PRANCR*. (A) Syntenic comparison of *MSTRG.33011* and *PRANCR*. The three gene structures are shown from top to bottom: human *PRANCR*, human *PRANCR* lifted over to the pig genome, and *MSTRG.33011*. Red triangles indicate two exons with identical exon structures, highlighting their conservation across species. (B) Epigenetic modification enrichment at the *PRANCR* locus (from top to bottom): 3D genomic architecture; H3K27ac and H3K4me3 modifications together with transcriptional activity; high-differentiation-associated sites; gene structure. (C) Comparison of *SOX8* and *PRANCR* expression levels in different myogenic cells between Laiwu pig and wild boar. (D) The *SOX8* binding motif, with red-shaded regions indicating high-differentiation-associated sites.

**Table 1:** The number of different cells in the muscle tissue of Laiwu pigs and wild boars

Cell name	Cell number in Laiwu pigs	Cell number in wild boars
*HES1*^+^ satellite cells	2,188	1,927
*TRIB1*^+^ satellite cells	2,593	1,880
Satellite stem cells	546	366
*MYL1*^+^ myoblasts	338	219
*MYMK*^+^ myoblasts	184	148
I myofibers	276	590
IIA myofibers	829	2,070
IIX myofibers	799	2,550

## Data Availability

Public dataset accession numbers are provided in Supplement 3.

## SUPPLEMENTARY MATERIAL

**Supplement 1**. Summary of data and its applications.

**Supplement 2**. NCBI SRA SRR run accessions.

**Supplement 3**. Sample information for lncRNA annotation.

**Supplement 4**. Marker genes used for cell type annotation.

**Supplement 5**. Characteristics of lncRNA.

**Supplement 6**. Percentage of eQTL linked lncRNA-mRNA pairs in the same TAD.

**Supplement 7**. (A) Single-cell clustering of myogenic cell populations. (B) The results of BLASTN analysis of the second homologous exon of *MSTRG.33011* and *PRANCR*.

**Supplement 8**. Differentially expressed genes across different cell types.

## References

[1] Lai D Qu X Wang Z Integrative genomic, transcriptomic and epigenomic analysis reveals cis-regulatory contributions to high-altitude adaptation in Tibetan pigs Mol Biol Evol 2025 42 msaf169 10.1093/molbev/msaf169 PMC12290512 40644385

[2] Cao M Yuan T Li D Whole-genome resequencing reveals positive selection and introgression signatures and genetic loci associated with early puberty traits in Chinese indigenous pigs Genet Sel Evol 2025 57 29 10.1186/s12711-025-00975-1 PMC12150574 40495150

[3] Guo X Su G Christensen OF Janss L Lund MS Genome-wide association analyses using a Bayesian approach for litter size and piglet mortality in Danish Landrace and Yorkshire pigs BMC Genom 2016 17 468 10.1186/s12864-016-2806-z PMC4912826 27317562

[4] Zhang Z Chen Z Ye S Genome-wide association study for reproductive traits in a Duroc pig population Animals 2019 9 732 10.3390/ani9100732 PMC6826494 31561612

[5] Van Laere AS Nguyen M Braunschweig M A regulatory mutation in IGF2 causes a major QTL effect on muscle growth in the pig Nature 2003 425 832 6 10.1038/nature02064 14574411

[6] Cho IC Park HB Ahn JS A functional regulatory variant of MYH3 influences muscle fiber-type composition and intramuscular fat content in pigs PLOS Genet 2019 15 e1008279 10.1371/journal.pgen.1008279 PMC6788688 31603892

[7] Liu Q Li M Xie S MYOD induced lnc-MEG3 promotes porcine satellite cell differentiation via interacting with DLST Epigenetics 2023 18 2237789 10.1080/15592294.2023.2237789 PMC10392761 37506369

[8] Zhou ZY Li AM Adeola AC Genome-wide identification of long intergenic noncoding RNA genes and their potential association with domestication in pigs Genome Biol Evol 2014 6 1387 92 10.1093/gbe/evu113 PMC4079208 24891613

[9] Zhao J Zhang J Chen Z Xiao M Zhao Y Whole-transcriptome RNA sequencing reveals global expression dynamics and ceRNA regulatory networks related to hair follicle development and melanogenesis in goats Anim Biosci 2025 38 1841 57 10.5713/ab.24.0617 PMC12415471 40211852

[10] Derrien T Johnson R Bussotti G The GENCODE v7 catalog of human long noncoding RNAs: analysis of their gene structure, evolution, and expression Genome Res 2012 22 1775 89 10.1101/gr.132159.111 PMC3431493 22955988

[11] Allou L Balzano S Magg A Non-coding deletions identify Maenli lncRNA as a limb-specific En1 regulator Nature 2021 592 93 8 10.1038/s41586-021-03208-9 33568816

[12] Priyanka P Sharma M Das S Saxena S The lncRNA HMS recruits RNA-binding protein HuR to stabilize the 3′-UTR of HOXC10 mRNA J Biol Chem 2021 297 100997 10.1016/j.jbc.2021.100997 PMC8363838 34302808

[13] Dror I Chitiashvili T Tan SYX XIST directly regulates X-linked and autosomal genes in naive human pluripotent cells Cell 2024 187 110 29.E31 10.1016/j.cell.2023.11.033 PMC10783549 38181737

[14] Yao Y Zhou R Yan C LncRNA RMG controls liquid-liquid phase separation of MEIS2 to regulate myogenesis Int J Biol Macromol 2025 310 143309 10.1016/j.ijbiomac.2025.143309 40252346

[15] Zhao Z Wang S Wang K Transcriptome analysis of liver and ileum reveals potential regulation of long non-coding RNA in pigs with divergent feed efficiency Anim Biosci 2024 38 588 99 10.5713/ab.24.0434 PMC11917451 39483020

[16] Li YJ Liu H Zhang YD Genome wide analysis of allele-specific circular RNAs in mammals and their role in cell proliferation Gene 2025 946 149317 10.1016/j.gene.2025.149317 39921049

[17] Jiang S Cheng SJ Ren LC An expanded landscape of human long noncoding RNA Nucleic Acids Res 2019 47 7842 56 10.1093/nar/gkz621 PMC6735957 31350901

[18] Jin L Tang Q Hu S A pig BodyMap transcriptome reveals diverse tissue physiologies and evolutionary dynamics of transcription Nat Commun 2021 12 3715 10.1038/s41467-021-23560-8 PMC8211698 34140474

[19] Li J Guan D Halstead MM Transcriptome annotation of 17 porcine tissues using nanopore sequencing technology Anim Genet 2023 54 35 44 10.1111/age.13274 36385508

[20] Zhao Y Hou Y Xu Y A compendium and comparative epigenomics analysis of cis-regulatory elements in the pig genome Nat Commun 2021 12 2217 10.1038/s41467-021-22448-x PMC8044108 33850120

[21] Yanai I Benjamin H Shmoish M Genome-wide midrange transcription profiles reveal expression level relationships in human tissue specification Bioinformatics 2005 21 650 9 10.1093/bioinformatics/bti042 15388519

[22] Teng J Gao Y Yin H A compendium of genetic regulatory effects across pig tissues Nat Genet 2024 56 112 23 10.1038/s41588-023-01585-7 PMC10786720 38177344

[23] Pan Z Yao Y Yin H Pig genome functional annotation enhances the biological interpretation of complex traits and human disease Nat Commun 2021 12 5848 10.1038/s41467-021-26153-7 PMC8494738 34615879

[24] Xu D Wan B Qiu K Single-cell RNA-sequencing provides insight into skeletal muscle evolution during the selection of muscle characteristics Adv Sci 2023 10 2305080 10.1002/advs.202305080 PMC10724408 37870215

[25] Engreitz JM Haines JE Perez EM Local regulation of gene expression by lncRNA promoters, transcription and splicing Nature 2016 539 452 5 10.1038/nature20149 PMC6853796 27783602

[26] Rehfeldt C Henning M Fiedler I Consequences of pig domestication for skeletal muscle growth and cellularity Livest Sci 2008 116 30 41 10.1016/j.livsci.2007.08.017

[27] Lösel D Franke A Kalbe C Comparison of different skeletal muscles from growing domestic pigs and wild boars Arch Anim Breed 2013 56 766 77 10.7482/0003-9438-56-076

[28] de Goede OM Nachun DC Ferraro NM Population-scale tissue transcriptomics maps long non-coding RNAs to complex disease Cell 2021 184 2633 48.E19 10.1016/j.cell.2021.03.050 PMC8651477 33864768

[29] The GTEX Consortium The GTEx Consortium atlas of genetic regulatory effects across human tissues Science 2020 369 1318 30 10.1126/science.aaz1776 PMC7737656 32913098

[30] Ruan X Li P Chen Y In vivo functional analysis of non-conserved human lncRNAs associated with cardiometabolic traits Nat Commun 2020 11 45 10.1038/s41467-019-13688-z PMC6940387 31896749

[31] Zhang YD Guo C Liu H Repressor elements provide insights into tissue development and phenotypes in pigs Zool Res 2024 45 1313 26 10.24272/j.issn.2095-8137.2024.043 PMC11668954 39479997

[32] Wu Y Zhang Y Liu H Genome-wide identification of functional enhancers and their potential roles in pig breeding J Anim Sci Biotechnol 2022 13 75 10.1186/s40104-022-00726-y PMC9252078 35781353

[33] Kornienko AE Dotter CP Guenzl PM Long non-coding RNAs display higher natural expression variation than protein-coding genes in healthy humans Genome Biol 2016 17 14 10.1186/s13059-016-0873-8 PMC4731934 26821746

[34] Sarropoulos I Marin R Cardoso-Moreira M Kaessmann H Developmental dynamics of lncRNAs across mammalian organs and species Nature 2019 571 510 4 10.1038/s41586-019-1341-x PMC6660317 31243368

[35] Tang Z Wu Y Yang Y Comprehensive analysis of long non-coding RNAs highlights their spatio-temporal expression patterns and evolutional conservation in Sus scrofa Sci Rep 2017 7 43166 10.1038/srep43166 PMC5324117 28233874

[36] Liu SJ Nowakowski TJ Pollen AA Single-cell analysis of long non-coding RNAs in the developing human neocortex Genome Biol 2016 17 67 10.1186/s13059-016-0932-1 PMC4831157 27081004

[37] Necsulea A Soumillon M Warnefors M The evolution of lncRNA repertoires and expression patterns in tetrapods Nature 2014 505 635 40 10.1038/nature12943 24463510

[38] Liu Q Zhou H Fu C MAZ-induced lncRNA H19 regulates proliferation and differentiation of porcine skeletal muscle satellite cells via sponge miR-935/miR-296-5p and the p38 MAPK pathway Int J Biol Macromol 2025 308 142675 10.1016/j.ijbiomac.2025.142675 40164245

[39] Cheng X Li L Shi G MEG3 promotes differentiation of porcine satellite cells by sponging miR-423-5p to relieve inhibiting effect on SRF Cells 2020 9 449 10.3390/cells9020449 PMC7072828 32075310

[40] Sakaue S Weinand K Isaac S Tissue-specific enhancer–gene maps from multimodal single-cell data identify causal disease alleles Nat Genet 2024 56 615 26 10.1038/s41588-024-01682-1 PMC11456345 38594305

