# Supplementary material for: Genome-wide identification of long non-coding RNAs in pigs and their roles in muscle development
Source: Anim Biosci. 2026 Jul 27;39(10):260444. doi: 10.5713/ab.260444 (PMC13662941; doi:10.5713/ab.260444)
Supplement: Supplementary file 2 [file ab-260444-Supplementary-2.pdf]

Supplement 2. NCBI SRA SRR run accessions

Run ID	Run ID	Run ID	Run ID	Run ID	Run ID	Run ID	Run ID
DRR003381	DRR003382	DRR054945	DRR054946	DRR054947	DRR054948	DRR054949	DRR054950
DRR054951	DRR054952	DRR124548	DRR124546	DRR124547	DRR161384	DRR161385	DRR161386
DRR072340	DRR072341	DRR169422	DRR169423	DRR169424	DRR169425	DRR169426	DRR169427
ERR972391	ERR972392	ERR972393	ERR972394	ERR977006	ERR977007	ERR977008	ERR977009
ERR977010	ERR977011	ERR977013	ERR977012	ERR977015	ERR977014	ERR977017	ERR977016
ERR977019	ERR977018	ERR977020	ERR977021	ERR977022	ERR977023	ERR977024	ERR977025
ERR1104677	ERR1104678	ERR1104679	ERR1104680	ERR1104681	ERR1104682	ERR1104683	ERR1104684
ERR1104685	ERR1625938	ERR1625939	ERR1625940	ERR1625941	ERR1625942	ERR1625943	ERR1625944
ERR1199492	ERR1199493	ERR1199494	ERR1199495	ERR1199496	ERR1199497	ERR1199498	ERR1199499
ERR1199500	ERR1199501	ERR1199502	ERR1199503	ERR1199504	ERR1199505	ERR1199506	ERR1199507
ERR1199508	ERR1199509	ERR1199510	ERR1199511	ERR1199512	ERR1199513	ERR1199514	ERR1199515
ERR1199516	ERR1199517	ERR1199518	ERR1199519	ERR1199520	ERR1199521	ERR1199522	ERR1223303
ERR1223304	ERR1223305	ERR1223306	ERR1223307	ERR1223308	ERR1223309	ERR1223310	ERR1223311
ERR1223312	ERR1223313	ERR1223314	ERR1223315	ERR1223316	ERR1223317	ERR1223318	ERR1223319
ERR1223320	ERR1331666	ERR1331726	ERR1331727	ERR1464205	ERR1464206	ERR1464216	ERR1464215
ERR1464210	ERR1464208	ERR1464204	ERR1464214	ERR1464201	ERR1464202	ERR1464212	ERR1464203
ERR1464207	ERR1464209	ERR1464213	ERR1464211	ERR1629879	ERR1629880	ERR1629881	ERR1629882
ERR1629883	ERR1629884	ERR1629885	ERR1629886	ERR1629887	ERR1629888	ERR1629889	ERR1629890
ERR1629891	ERR1629892	ERR1629893	ERR1815469	ERR1815470	ERR1815471	ERR1815472	ERR1815476
ERR1815473	ERR1815474	ERR1815475	ERR1815477	ERR1815478	ERR1815479	ERR1815480	ERR1815481
ERR1815482	ERR1815483	ERR1815484	ERR1815485	ERR1815486	ERR1824415	ERR1824416	ERR1824495
ERR1824496	ERR1824463	ERR1824464	ERR1824563	ERR1824564	ERR1824266	ERR1824314	ERR1824315
ERR1824354	ERR1824355	ERR1824380	ERR1824393	ERR1824394	ERR1824473	ERR1824474	ERR1824533
ERR1824534	ERR1824443	ERR1824444	ERR1824523	ERR1824524	ERR1824273	ERR1824328	ERR1824329
ERR1824368	ERR1824369	ERR1824386	ERR1824288	ERR1824289	ERR1824312	ERR1824313	ERR1824352
ERR1824353	ERR1824379	ERR1824423	ERR1824424	ERR1824505	ERR1824506	ERR1824427	ERR1824428
ERR1824497	ERR1824498	ERR1824268	ERR1824318	ERR1824319	ERR1824358	ERR1824359	ERR1824395
ERR1824396	ERR1824475	ERR1824476	ERR1824535	ERR1824536	ERR1824391	ERR1824392	ERR1824471
ERR1824472	ERR1824531	ERR1824532	ERR1824276	ERR1824277	ERR1824298	ERR1824299	ERR1824338
ERR1824339	ERR1824389	ERR1824390	ERR1824469	ERR1824470	ERR1824529	ERR1824530	ERR1824435
ERR1824436	ERR1824515	ERR1824516	ERR1824453	ERR1824454	ERR1824553	ERR1824554	ERR1824459
ERR1824460	ERR1824559	ERR1824560	ERR1824409	ERR1824410	ERR1824489	ERR1824490	ERR1824274
ERR1824275	ERR1824296	ERR1824297	ERR1824336	ERR1824337	ERR1824373	ERR1824387	ERR1824388
ERR1824467	ERR1824468	ERR1824527	ERR1824528	ERR1824425	ERR1824426	ERR1824507	ERR1824508
ERR1824437	ERR1824438	ERR1824517	ERR1824518	ERR1824447	ERR1824448	ERR1824547	ERR1824548
ERR1824431	ERR1824432	ERR1824511	ERR1824512	ERR1824269	ERR1824320	ERR1824321	ERR1824360
ERR1824361	ERR1824382	ERR1824397	ERR1824398	ERR1824477	ERR1824478	ERR1824537	ERR1824538
ERR1824455	ERR1824456	ERR1824555	ERR1824556	ERR1824265	ERR1824306	ERR1824307	ERR1824346
ERR1824347	ERR1824376	ERR1824272	ERR1824326	ERR1824327	ERR1824366	ERR1824367	ERR1824385
ERR1824419	ERR1824420	ERR1824501	ERR1824502	ERR1824262	ERR1824290	ERR1824291	ERR1824330
ERR1824331	ERR1824370	ERR1824417	ERR1824418	ERR1824499	ERR1824500	ERR1824403	ERR1824404
ERR1824483	ERR1824484	ERR1824543	ERR1824544	ERR1824407	ERR1824408	ERR1824487	ERR1824488
ERR1824284	ERR1824285	ERR1824308	ERR1824309	ERR1824348	ERR1824349	ERR1824377	ERR1824445
ERR1824446	ERR1824525	ERR1824526	ERR1824411	ERR1824412	ERR1824491	ERR1824492	ERR1824449
ERR1824450	ERR1824549	ERR1824550	ERR1824280	ERR1824281	ERR1824302	ERR1824303	ERR1824342
ERR1824343	ERR1824374	ERR1824433	ERR1824434	ERR1824513	ERR1824514	ERR1824439	ERR1824440
ERR1824519	ERR1824520	ERR1824465	ERR1824466	ERR1824565	ERR1824566	ERR1824429	ERR1824430
ERR1824509	ERR1824510	ERR1824413	ERR1824414	ERR1824493	ERR1824494	ERR1824457	ERR1824458
ERR1824557	ERR1824558	ERR1824270	ERR1824322	ERR1824323	ERR1824362	ERR1824363	ERR1824383
ERR1824399	ERR1824400	ERR1824479	ERR1824480	ERR1824539	ERR1824540	ERR1824461	ERR1824462
ERR1824561	ERR1824562	ERR1824264	ERR1824294	ERR1824295	ERR1824334	ERR1824335	ERR1824372
ERR1824267	ERR1824316	ERR1824317	ERR1824356	ERR1824357	ERR1824381	ERR1824278	ERR1824279
ERR1824300	ERR1824301	ERR1824340	ERR1824341	ERR1824286	ERR1824287	ERR1824310	ERR1824311
ERR1824350	ERR1824351	ERR1824378	ERR1824441	ERR1824442	ERR1824521	ERR1824522	ERR1824263
ERR1824292	ERR1824293	ERR1824332	ERR1824333	ERR1824371	ERR1824421	ERR1824422	ERR1824503
ERR1824504	ERR1824451	ERR1824452	ERR1824551	ERR1824552	ERR1898446	ERR1898447	ERR1898448

ERR1898449	ERR1898450	ERR1898451	ERR1898452	ERR1898453	ERR1898454	ERR1898455	ERR1898456
ERR1898457	ERR1898458	ERR1898459	ERR1898460	ERR1898461	ERR1898462	ERR1898463	ERR1898464
ERR1898465	ERR1898466	ERR1898467	ERR1898468	ERR1898469	ERR1898470	ERR1898471	ERR1898472
ERR1898473	ERR1898474	ERR1898475	ERR1898476	ERR1898477	ERR2059850	ERR2059851	ERR2059852
ERR2059853	ERR2059854	ERR2059855	ERR2059856	ERR2059857	ERR2059858	ERR2059859	ERR2059860
ERR2059861	ERR2059862	ERR2059863	ERR2059864	ERR2059865	ERR2059866	ERR2059867	ERR2059868
ERR2059869	ERR2059870	ERR2059871	ERR2059872	ERR2059873	ERR2059874	ERR2059875	ERR2059876
ERR2059877	ERR2059878	ERR2059879	ERR2059880	ERR2059881	ERR2059882	ERR2059883	ERR2059884
ERR2059885	ERR2059886	ERR2059887	ERR2059888	ERR2059889	ERR2059890	ERR2059891	ERR2188804
ERR2188805	ERR2188806	ERR2188807	ERR2188808	ERR2188809	ERR2188810	ERR2188811	ERR2188812
ERR2188813	ERR2188815	ERR2188814	ERR2188816	ERR2188817	ERR2188818	ERR2188819	ERR2188820
ERR2188821	ERR2188822	ERR2188823	ERR2205062	ERR2205063	ERR2205064	ERR2205065	ERR2205066
ERR2205067	ERR2205068	ERR2205069	ERR2205070	ERR2205071	ERR2205072	ERR2205073	ERR2205075
ERR2205074	ERR2205076	ERR2205077	ERR2205078	ERR2205079	ERR2205081	ERR2205080	ERR2206066
ERR2206067	ERR2206068	ERR2206069	ERR2206070	ERR2206071	ERR2206072	ERR2206073	ERR2206074
ERR2206075	ERR2206077	ERR2206076	ERR2206078	ERR2206079	ERR2206080	ERR2206081	ERR2206082
ERR2206083	ERR2206085	ERR2206084	ERR2206824	ERR2206825	ERR2206826	ERR2206827	ERR2206828
ERR2206829	ERR2206830	ERR2206831	ERR2206832	ERR2206833	ERR2206834	ERR2206835	ERR2206836
ERR2206837	ERR2206838	ERR2206839	ERR2208521	ERR2208522	ERR2208523	ERR2208524	ERR2208525
ERR2208526	ERR2208527	ERR2208528	ERR2208529	ERR2208530	ERR2208531	ERR2208532	ERR2208533
ERR2208534	ERR2208535	ERR2208536	ERR2208537	ERR2208538	ERR2208539	ERR2208540	ERR2208541
ERR2208542	ERR2208543	ERR2208544	ERR2208545	ERR2208546	ERR2208547	ERR2208548	ERR2208549
ERR2208550	ERR2208551	ERR2208552	ERR2208553	ERR2208554	ERR2208555	ERR2208556	ERR2240042
ERR2240043	ERR2240044	ERR2240048	ERR2240049	ERR2240050	ERR2239979	ERR2239980	ERR2239981
ERR2240003	ERR2240004	ERR2240005	ERR2240012	ERR2240013	ERR2240014	ERR2240009	ERR2240010
ERR2240011	ERR2240036	ERR2240037	ERR2240038	ERR2240039	ERR2240040	ERR2240041	ERR2240027
ERR2240028	ERR2240029	ERR2240021	ERR2240022	ERR2240023	ERR2240006	ERR2240007	ERR2240008
ERR2239991	ERR2239992	ERR2239993	ERR2239988	ERR2239989	ERR2239990	ERR2240030	ERR2240031
ERR2240032	ERR2240015	ERR2240016	ERR2240017	ERR2240045	ERR2240046	ERR2240047	ERR2239982
ERR2239983	ERR2239984	ERR2239994	ERR2239995	ERR2239996	ERR2239997	ERR2239998	ERR2239999
ERR2239985	ERR2239986	ERR2239987	ERR2240000	ERR2240001	ERR2240002	ERR2240018	ERR2240019
ERR2240020	ERR2240024	ERR2240025	ERR2240026	ERR2240033	ERR2240034	ERR2240035	ERR2508834
ERR2508835	ERR2508836	ERR2508837	ERR2508838	ERR2508839	ERR2508840	ERR2508841	ERR2508842
ERR2508843	ERR2508844	ERR2508845	ERR2508846	ERR2508847	ERR2508848	ERR2508849	ERR2508850
ERR2508851	ERR2508852	ERR2508853	ERR2508854	ERR2508855	ERR2508856	ERR2508857	ERR2508858
ERR2508859	ERR2508860	ERR2508861	ERR2508862	ERR2508863	ERR2508864	ERR2508865	ERR2508866
ERR2508867	ERR2508868	ERR2508869	ERR2508870	ERR2508871	ERR2508872	ERR2508873	ERR2508874
ERR2508875	ERR2534319	ERR2534320	ERR2534321	ERR2534322	ERR2534323	ERR2534324	ERR2534325
ERR2534326	ERR2534327	ERR2534328	ERR2534329	ERR2534330	ERR2534331	ERR2534332	ERR2534333
ERR2534334	ERR2534335	ERR2534336	ERR2664195	ERR2664193	ERR2664189	ERR2664191	ERR2664186
ERR2664199	ERR2664200	ERR2664188	ERR2664201	ERR2743091	ERR2743092	ERR2743093	ERR2743094
ERR2743095	ERR2743096	ERR2743097	ERR2743098	ERR2743100	ERR2743101	ERR2743099	ERR2743104
ERR2743102	ERR2743103	ERR2743105	ERR2743106	ERR2743108	ERR2743107	ERR2743109	ERR2743110
ERR2743111	ERR2743112	ERR2743113	ERR2743115	ERR2743114	ERR2743116	ERR2743117	ERR2743118
ERR2743119	ERR2743120	ERR2743121	ERR2743122	ERR2743123	ERR2743124	ERR2743125	ERR2743126
ERR2743127	ERR2743128	ERR2743129	ERR2743132	ERR2743130	ERR2743131	ERR2743133	ERR2743134
ERR2743136	ERR2743135	ERR2743137	ERR2743138	ERR2743139	ERR2743140	ERR2743141	ERR2743142
ERR2743144	ERR2743143	ERR2743145	ERR2743146	ERR2743147	ERR2743148	ERR2743149	ERR2743150
ERR2743151	ERR2743152	ERR2743154	ERR2743153	ERR2743156	ERR2743155	ERR2743157	ERR2743158
ERR2743159	ERR2743160	ERR2743161	ERR2743162	ERR2743163	ERR2743164	ERR2743166	ERR2743165
ERR2743167	ERR2743168	ERR2743171	ERR2743169	ERR2743170	ERR2743173	ERR2743172	ERR2743175
ERR2743174	ERR2743176	ERR2743177	ERR2743178	ERR2743179	ERR2743180	ERR2743181	ERR2743182
ERR2743184	ERR2743183	ERR2743185	ERR2743186	ERR2743187	ERR2743188	ERR2743189	ERR2743191
ERR2743190	ERR2775643	ERR2775644	ERR2775645	ERR2775646	ERR2775647	ERR2775648	ERR2775649
ERR2775650	ERR2775651	ERR2775652	ERR2775653	ERR2775654	ERR2775655	ERR2775656	ERR2775657
ERR2775658	ERR2775659	ERR2775660	ERR2775661	ERR2775662	ERR2775663	ERR2775664	ERR2775665
ERR2775666	ERR2775667	ERR2775668	ERR2775669	ERR2775670	ERR2775671	ERR2775672	ERR2775673
ERR2775674	ERR2775675	ERR2775676	ERR2775677	ERR2775678	ERR2775679	ERR2775680	ERR2775681

ERR2775682	ERR2775683	ERR2775684	ERR2775685	ERR2775686	ERR2775687	ERR2775688	ERR2775689
ERR2775690	ERR2930321	ERR2930322	ERR2930324	ERR2930323	ERR2930325	ERR2930326	ERR2930327
ERR2930328	ERR2930329	ERR2930330	ERR2930331	ERR2930332	ERR2930333	ERR2930334	ERR2930335
ERR2930336	ERR2930337	ERR2930338	ERR2930339	ERR2930340	ERR2930341	ERR2930342	ERR2930344
ERR2930343	ERR2930345	ERR2930346	ERR2930347	ERR2930348	ERR2930350	ERR2930349	ERR2930352
ERR2930351	ERR2930353	ERR2930354	ERR2930355	ERR2930356	ERR2930357	ERR2930358	ERR2930359
ERR2930360	ERR2930362	ERR2930361	ERR2930363	ERR2930364	ERR2930366	ERR2930365	ERR2930367
ERR2930368	ERR2930369	ERR2930370	ERR2930371	ERR2930372	ERR2930373	ERR2930374	ERR2930375
ERR2930376	ERR2930378	ERR2930377	ERR2930380	ERR2930379	ERR2930381	ERR2930382	ERR2930383
ERR2930384	ERR2930385	ERR2930386	ERR2930387	ERR2930388	ERR2930390	ERR2930389	ERR2930391
ERR2930392	ERR2930393	ERR2930394	ERR2930396	ERR2930395	ERR2930397	ERR2930398	ERR2930399
ERR2930400	ERR2930401	ERR2930402	ERR2930404	ERR2930403	ERR2930405	ERR2930406	ERR2930407
ERR2930408	ERR2930409	ERR2930410	ERR2930411	ERR2930412	ERR2930413	ERR2930414	ERR2930415
ERR2930416	ERR2935138	ERR2935139	ERR2935140	ERR2935141	ERR2935142	ERR2935143	ERR2935144
ERR2935145	ERR2935146	ERR2935147	ERR2935148	ERR2935149	ERR2983233	ERR2983234	ERR2983235
ERR2983236	ERR2983237	ERR2983238	ERR2983239	ERR2983240	ERR2983241	ERR2983242	ERR2983243
ERR2983244	ERR2983245	ERR2983246	ERR2983247	ERR2983248	ERR2983249	ERR2984913	ERR2984914
ERR2984915	ERR2984916	ERR2984917	ERR2984918	ERR2984919	ERR2984920	ERR2984921	ERR2984922
ERR2984923	ERR2984924	ERR2984925	ERR2984926	ERR2984927	ERR2984928	ERR2984929	ERR2984930
ERR2984931	ERR2984932	ERR2984933	ERR2984934	ERR2984935	ERR2984936	ERR2984937	ERR2984938
ERR2984939	ERR2984940	ERR2984941	ERR2984942	ERR2984943	ERR2984944	ERR2984945	ERR2984946
ERR2984947	ERR2984948	ERR2984949	ERR2984950	ERR2984951	ERR2984952	ERR2984953	ERR2984954
ERR2984955	ERR2984956	ERR2984957	ERR2984958	ERR2984959	ERR2984960	ERR2984961	ERR2984962
ERR2984963	ERR2984964	ERR2984965	ERR2984966	ERR2984967	ERR2984968	ERR2984969	ERR2984970
ERR2984971	ERR2984972	ERR2984973	ERR2984974	ERR2984975	ERR2984976	ERR2984977	ERR2984978
ERR2984979	ERR2984980	ERR2984981	ERR2984982	ERR2984983	ERR2984984	ERR2984985	ERR2984986
ERR2984987	ERR2984988	ERR2984989	ERR2984990	ERR2984991	ERR2984992	ERR2984993	ERR2984994
ERR2984995	ERR2984996	ERR2984997	ERR2984998	ERR2984999	ERR2985000	ERR2985001	ERR2985002
ERR2985003	ERR2985004	ERR2985005	ERR2985006	ERR2985007	ERR2985008	ERR3010918	ERR3010919
ERR3010920	ERR3010921	ERR3010922	ERR3010923	ERR3010924	ERR3010925	ERR3010926	ERR3010927
ERR3010928	ERR3010929	ERR3010930	ERR3010931	ERR3010932	ERR3010933	ERR3010934	ERR3010935
ERR3010936	ERR3010937	ERR3010938	ERR3010939	ERR3010940	ERR3010941	ERR3010942	ERR3010943
ERR3010944	ERR3010945	ERR3010946	ERR3010947	ERR3010948	ERR3010949	ERR3010950	ERR3010951
ERR3010952	ERR3010953	ERR3010954	ERR3010955	ERR3010956	ERR3010957	ERR3010958	ERR3010959
ERR3010960	ERR3010961	ERR3010962	ERR3010963	ERR3010964	ERR3010965	ERR3010966	ERR3010967
ERR3010968	ERR3010969	ERR3010970	ERR3010971	ERR3010972	ERR3010973	ERR3010974	ERR3010975
ERR3010976	ERR3010977	ERR3010978	ERR3010979	ERR3010980	ERR3010981	ERR3010982	ERR3010983
ERR3010984	ERR3010985	ERR3010986	ERR3010987	ERR3010988	ERR3010989	ERR3010990	ERR3010991
ERR3010992	ERR3010993	ERR3010994	ERR3010995	ERR3010996	ERR3010997	ERR3010998	ERR3010999
ERR3011000	ERR3011001	ERR3011002	ERR3011003	ERR3011004	ERR3011005	ERR3011006	ERR3011007
ERR3011008	ERR3011009	ERR3011010	ERR3011011	ERR3011012	ERR3011013	ERR3011014	ERR3011015
ERR3011016	ERR3011017	ERR3011018	ERR3011019	ERR3011020	ERR3011021	ERR3011022	ERR3011023
ERR3011024	ERR3011025	ERR3011026	ERR3011027	ERR3011028	ERR3011029	ERR3011030	ERR3011031
ERR3011032	ERR3011033	ERR3011034	ERR3011035	ERR3011036	ERR3011037	ERR3011038	ERR3011039
ERR3011040	ERR3011041	ERR3011042	ERR3011043	ERR3011044	ERR3011045	ERR3011046	ERR3011047
ERR3011048	ERR3011049	ERR3011050	ERR3011051	ERR3011052	ERR3011053	ERR3011054	ERR3011055
ERR3011056	ERR3011057	ERR3011058	ERR3011059	ERR3011060	ERR3011061	ERR3011062	ERR3011063
ERR3011064	ERR3011065	ERR3011066	ERR3011067	ERR3011068	ERR3011069	ERR3011070	ERR3011071
ERR3011072	ERR3011073	ERR3011074	ERR3011075	ERR3011076	ERR3011077	ERR3011078	ERR3011079
ERR3153923	ERR3153924	ERR3153955	ERR3153956	ERR3153957	ERR3153958	ERR3153959	ERR3153960
ERR3153961	ERR3153962	ERR3153963	ERR3153964	ERR3153965	ERR3153966	ERR3184684	ERR3184693
ERR3184698	ERR3184697	ERR3184699	ERR3184682	ERR3184691	ERR3184677	ERR3184681	ERR3184687
ERR3184690	ERR3184696	ERR3184688	ERR3184685	ERR3184678	ERR3184695	ERR3184689	ERR3184676
ERR3184694	ERR3184683	ERR3184692	ERR3184679	ERR3184680	ERR3184686	ERR162216	ERR162223
ERR162265	ERR162254	ERR162220	ERR162247	ERR162255	ERR162233	ERR162241	ERR162231
ERR266405	ERR266361	ERR266387	ERR266362	ERR266406	ERR266392	ERR266396	ERR266379
ERR266390	ERR266363	ERR3341405	ERR3341406	ERR3341407	ERR3341408	ERR3341409	ERR3341410
ERR3341411	ERR3341412	ERR3415742	ERR3415753	ERR3415755	ERR3417904	ERR3417916	ERR3417918

ERR3417924	ERR3417935	ERR3417937	ERR3417952	ERR3417965	ERR3417993	ERR3418012	ERR3418014
ERR3418018	ERR3440518	ERR3440519	ERR3440520	ERR3440521	ERR3440522	ERR3440523	ERR3440524
ERR3440525	ERR3440526	ERR3440527	ERR3440528	ERR3440529	ERR3440530	ERR3440531	ERR3440532
ERR3440533	ERR3440534	ERR3440535	ERR3440536	ERR3440537	ERR3440538	ERR3440539	ERR3440540
ERR3440541	ERR3440542	ERR3440543	ERR3440544	ERR3440545	ERR3440546	ERR3440547	ERR3440548
ERR3440549	ERR3440550	ERR3440551	ERR3440552	ERR3440553	ERR3440554	ERR3440555	ERR3440556
ERR3440557	ERR3440558	ERR3440559	ERR3440560	ERR3440561	ERR3440562	ERR3440563	ERR3440564
ERR3440565	ERR3440566	ERR3440567	ERR3440568	ERR3440569	ERR3440570	ERR3440571	ERR3440572
ERR3440573	ERR3440574	ERR3440575	ERR3440576	ERR3440577	ERR3440578	ERR3440579	ERR3440580
ERR3440581	ERR192387	ERR192380	ERR192439	ERR192385	ERR192450	ERR192396	ERR192412
ERR192438	ERR192386	ERR192410	ERR192429	ERR192379	ERR192362	ERR192397	ERR192413
ERR192437	ERR192364	ERR192436	ERR192414	ERR192426	ERR192411	ERR192389	ERR192428
ERR192406	ERR192374	ERR192415	ERR192370	ERR192445	ERR192372	ERR192395	ERR192442
ERR192407	ERR192409	ERR192425	ERR192447	ERR192394	ERR192449	ERR192403	ERR192424
ERR192443	ERR192420	ERR192398	ERR192383	ERR192433	ERR192432	ERR192408	ERR192404
ERR192375	ERR192377	ERR192435	ERR192373	ERR192416	ERR192371	ERR192402	ERR192369
ERR192431	ERR192384	ERR192422	ERR192400	ERR192382	ERR192444	ERR192393	ERR192448
ERR192423	ERR192388	ERR192390	ERR192421	ERR192368	ERR192391	ERR192418	ERR192392
ERR192440	ERR192366	ERR192365	ERR192401	ERR192441	ERR192434	ERR192430	ERR192427
ERR192399	ERR192417	ERR192378	ERR192405	ERR192363	ERR192367	ERR192446	ERR192376
ERR192419	ERR192381	ERR3772540	ERR3772541	ERR3772542	ERR3772543	ERR3772544	ERR3772545
ERR3772546	ERR3772547	ERR3772548	ERR3772549	ERR3978720	ERR3978721	ERR3978722	ERR3978723
ERR3978724	ERR3978725	ERR3978726	ERR3978727	ERR3978728	ERR3978729	ERR3978730	ERR3978731
ERR3978732	ERR3978733	ERR3978734	ERR3978735	ERR3978736	ERR3978737	ERR3978738	ERR3978739
ERR3978740	ERR3978741	ERR3978742	ERR3978743	ERR3978744	ERR3978745	ERR3978746	ERR3978747
ERR3978748	ERR3978749	ERR3978750	ERR3978751	ERR3991152	ERR3991153	ERR3991154	ERR3991149
ERR3991150	ERR3991151	ERR4057010	ERR4057011	ERR4057012	ERR4057013	ERR4057014	ERR4057015
ERR4057016	ERR4057017	ERR4057018	ERR4057019	ERR4057020	ERR4057021	ERR4057022	ERR4057023
ERR4057024	ERR4057025	ERR4057026	ERR4057027	ERR4057028	ERR4057029	ERR4057030	ERR4057031
ERR4057032	ERR4057033	ERR4057034	ERR4057035	ERR4057036	ERR4057037	ERR4057038	ERR4057039
ERR4057040	ERR4057041	ERR4057042	ERR4057043	ERR4057044	ERR4057045	ERR4057046	ERR4057047
ERR4057048	ERR4057049	ERR4057050	ERR4057051	ERR4057052	ERR4057053	ERR4057054	ERR4057055
ERR4057056	ERR4057057	ERR4083453	ERR4083454	ERR4083455	ERR4083456	ERR4083457	ERR4083458
ERR4083459	ERR4083460	ERR4083461	ERR4083462	ERR4083463	ERR4083464	ERR4083465	ERR4083466
ERR4083467	ERR4083468	ERR4083469	ERR4083470	ERR4083471	ERR4083472	ERR4083473	ERR4083474
ERR4083475	ERR4083476	ERR4083477	ERR4083478	ERR4083479	ERR4083480	ERR4083481	ERR4083482
ERR4083483	ERR4083484	ERR4083485	ERR4083486	ERR4083487	ERR4083488	ERR4234128	ERR4234129
ERR4234134	ERR4234137	ERR4234135	ERR4234131	ERR4234136	ERR4234130	ERR4234132	ERR4234133
ERR4234138	ERR4234139	ERR4145477	ERR4145478	ERR4167313	ERR4167314	ERR4167315	ERR4167316
ERR4167317	ERR4167318	ERR4167319	ERR4167320	ERR4167321	ERR4167322	ERR4167323	ERR4167324
ERR4167325	ERR4167326	ERR4167327	ERR4167328	ERR4167329	ERR4167330	ERR4167331	ERR4167332
ERR4167333	ERR4167334	ERR4167335	ERR4167336	ERR4167337	ERR4167338	ERR4167339	ERR4167340
ERR4167358	ERR4167359	ERR4167360	ERR4167361	ERR4167362	ERR4167363	ERR4167364	ERR4167365
ERR4167366	ERR4167367	ERR4167368	ERR4167369	ERR4167370	ERR4167371	ERR4167372	ERR4167373
ERR4167374	ERR4167375	ERR4167376	ERR4167377	ERR4167378	ERR4167379	ERR4167380	ERR4167381
ERR4167382	ERR4167383	ERR4167384	ERR4167385	ERR4167386	ERR4167387	ERR4167388	ERR4167389
ERR4167390	ERR4167391	ERR4167392	ERR4167393	ERR4167394	ERR4167395	ERR4167396	ERR4167397
ERR4167398	ERR4167399	ERR4167400	ERR4167401	ERR4167402	ERR4167403	ERR4167404	ERR4167405
ERR4419506	ERR4419507	ERR4419508	ERR4419509	ERR4419510	ERR4419511	ERR4419512	ERR4419513
ERR4419514	ERR4421732	ERR4421733	ERR4421734	ERR4421735	ERR4421736	ERR4421737	ERR4421738
ERR4421739	ERR4421740	ERR4467692	ERR4467693	ERR4467694	ERR4467695	ERR4467696	ERR4467697
ERR4467698	ERR4467699	ERR4467700	ERR4467710	ERR4467711	ERR4467712	ERR4467713	ERR4467714
ERR4467715	ERR4467716	ERR4467717	ERR4467718	ERR4467701	ERR4467702	ERR4467703	ERR4467704
ERR4467705	ERR4467706	ERR4467707	ERR4467708	ERR4467709	ERR4551685	ERR4551686	ERR4551687
ERR4551688	ERR4868983	ERR4868982	ERR4868981	ERR4868976	ERR4868979	ERR4868975	ERR4868974
ERR4868972	ERR4868973	ERR4868980	ERR4868958	ERR4868968	ERR4868969	ERR4868963	ERR4868978
ERR4868970	ERR4868959	ERR4868960	ERR4868966	ERR4868961	ERR4868962	ERR4868964	ERR4868977
ERR4868965	ERR4868967	ERR4868971	ERR4873346	ERR4873347	ERR4873348	ERR4873349	ERR4873350

ERR4873351	ERR4873352	ERR4873353	ERR4873354	ERR4873355	ERR4873356	ERR4873357	ERR4873358
ERR4873359	ERR4873360	ERR4873361	ERR4873362	ERR4873363	ERR4873364	ERR4873365	ERR4873366
ERR4873367	ERR4873368	ERR4873369	ERR4873370	ERR4873371	ERR4873372	ERR4873373	ERR4873374
ERR4873375	ERR4873376	ERR4873377	ERR4873378	ERR4873379	ERR4873380	ERR4873381	ERR4873382
ERR4873383	ERR4873384	ERR4873385	ERR4873386	ERR4873387	ERR4873388	ERR4873389	ERR4873390
ERR4873391	ERR4873392	ERR4873393	ERR4873394	ERR4873395	ERR4873396	ERR4873397	ERR4873398
ERR4873399	ERR4873400	ERR4873401	ERR4873402	ERR4873403	ERR4873404	ERR4873405	ERR4873406
ERR4873407	ERR4873408	ERR4873409	ERR4873410	ERR4873411	ERR4873412	ERR4873413	ERR4873414
ERR4873415	ERR4873416	ERR4873417	ERR4873418	ERR4873419	ERR4873420	ERR4873421	ERR4873422
ERR4873423	ERR4873424	ERR4873425	ERR4873426	ERR4873427	ERR4873428	ERR4873429	ERR4873430
ERR4873431	ERR4873432	ERR4873433	ERR4873434	ERR4873435	ERR4873436	ERR4873437	ERR4873438
ERR4873439	ERR4873440	ERR4873441	ERR4982154	ERR4982155	ERR4982156	ERR4982157	ERR4982158
ERR4982159	ERR4982160	ERR4982161	ERR4982162	ERR4982163	ERR4982164	ERR4982165	ERR4982178
ERR4982179	ERR4982180	ERR4982181	ERR4982174	ERR4982175	ERR4982176	ERR4982177	ERR4982166
ERR4982167	ERR4982168	ERR4982169	ERR4982170	ERR4982171	ERR4982172	ERR4982173	ERR4982182
ERR4982183	ERR4982184	ERR4982185	ERR4982194	ERR4982195	ERR4982196	ERR4982197	ERR4982186
ERR4982187	ERR4982188	ERR4982189	ERR4982202	ERR4982203	ERR4982204	ERR4982205	ERR4982190
ERR4982191	ERR4982192	ERR4982193	ERR4982198	ERR4982199	ERR4982200	ERR4982201	ERR4982206
ERR4982207	ERR4982208	ERR4982209	ERR4982126	ERR4982130	ERR4982134	ERR4982150	ERR4982138
ERR4982142	ERR4982146	ERR4982127	ERR4982131	ERR4982135	ERR4982151	ERR4982139	ERR4982143
ERR4982147	ERR4982128	ERR4982132	ERR4982136	ERR4982152	ERR4982140	ERR4982144	ERR4982148
ERR4982129	ERR4982133	ERR4982137	ERR4982153	ERR4982141	ERR4982145	ERR4982149	ERR5101085
ERR5101084	ERR5101086	ERR5101125	ERR5101123	ERR5101124	ERR5101141	ERR5101143	ERR5101142
ERR5101122	ERR5101121	ERR5101120	ERR5101104	ERR5101103	ERR5101102	ERR5101136	ERR5101137
ERR5101135	ERR5101106	ERR5101107	ERR5101105	ERR5101082	ERR5101083	ERR5101081	ERR5101112
ERR5101113	ERR5101111	ERR5101115	ERR5101116	ERR5101114	ERR5101100	ERR5101101	ERR5101099
ERR5101110	ERR5101108	ERR5101109	ERR5101088	ERR5101087	ERR5101089	ERR5101126	ERR5101128
ERR5101127	ERR5101093	ERR5101094	ERR5101095	ERR5101129	ERR5101131	ERR5101130	ERR5101145
ERR5101146	ERR5101144	ERR5101096	ERR5101097	ERR5101098	ERR5101090	ERR5101091	ERR5101092
ERR5101133	ERR5101134	ERR5101132	ERR5101117	ERR5101118	ERR5101119	ERR5101140	ERR5101138
ERR5101139	ERR5416409	ERR5416410	ERR5416411	ERR5416412	ERR5416413	ERR5416414	ERR6061942
ERR6061943	ERR6061944	ERR6061945	ERR6061946	ERR6061947	ERR6061948	ERR6061949	ERR6061950
ERR6061951	ERR6061952	ERR6061953	ERR6061954	ERR6061955	ERR6061956	ERR6061957	ERR6061958
ERR6061959	ERR6061960	ERR6061961	ERR6061962	ERR6061963	ERR6061964	ERR6061965	ERR6061966
ERR6061967	ERR6061968	ERR6061969	ERR5523693	ERR5523694	ERR5523695	ERR5523696	ERR5523697
ERR5523698	ERR5523699	ERR5523700	ERR5523701	ERR5523702	ERR5523703	ERR5523704	ERR5523705
ERR5523706	ERR5523707	ERR6576431	ERR6576432	ERR6576433	ERR6576434	ERR6576435	ERR6576436
ERR6576437	ERR6576438	ERR6576439	ERR6576440	ERR6576450	ERR6576441	ERR6576442	ERR6576443
ERR6576444	ERR6576445	ERR6576446	ERR6576447	ERR6576448	ERR6576449	ERR6559211	ERR6559212
ERR6559213	ERR6559214	ERR6559215	ERR6559216	ERR6559217	ERR6559218	ERR7732396	ERR7732397
ERR7732398	ERR7732400	ERR7732401	ERR7732402	ERR7732403	ERR7732399	ERR7732404	ERR7732405
ERR7732406	ERR7732407	ERR7732417	ERR7732418	ERR7732416	ERR7732419	ERR7732408	ERR7732409
ERR7732410	ERR7732411	ERR7732412	ERR7732413	ERR7732414	ERR7732415	ERR7732476	ERR7732477
ERR7732479	ERR7732480	ERR7732481	ERR7732482	ERR7732483	ERR7732484	ERR7732485	ERR7732486
ERR7732487	ERR7732478	ERR7732488	ERR7732489	ERR7732490	ERR7732491	ERR7732492	ERR7732493
ERR7732494	ERR7732495	ERR7732496	ERR7732497	ERR7732498	ERR7732499	ERR7732512	ERR7732514
ERR7732515	ERR7732516	ERR7732517	ERR7732518	ERR7732519	ERR7732520	ERR7732521	ERR7732522
ERR7732523	ERR7732513	ERR7732500	ERR7732502	ERR7732503	ERR7732504	ERR7732505	ERR7732506
ERR7732507	ERR7732508	ERR7732509	ERR7732510	ERR7732511	ERR7732501	ERR7732372	ERR7732373
ERR7732374	ERR7732375	ERR7732376	ERR7732377	ERR7732378	ERR7732379	ERR7732381	ERR7732382
ERR7732383	ERR7732380	ERR7732392	ERR7732393	ERR7732394	ERR7732395	ERR7732384	ERR7732385
ERR7732386	ERR7732387	ERR7732388	ERR7732389	ERR7732390	ERR7732391	ERR7732440	ERR7732441
ERR7732442	ERR7732443	ERR7732444	ERR7732445	ERR7732446	ERR7732447	ERR7732449	ERR7732450
ERR7732451	ERR7732452	ERR7732453	ERR7732454	ERR7732455	ERR7732448	ERR7732465	ERR7732467
ERR7732468	ERR7732469	ERR7732470	ERR7732471	ERR7732472	ERR7732474	ERR7732475	ERR7732473
ERR7732466	ERR7732464	ERR7732456	ERR7732457	ERR7732458	ERR7732459	ERR7732460	ERR7732461
ERR7732462	ERR7732463	ERR7732348	ERR7732349	ERR7732350	ERR7732351	ERR7732352	ERR7732353
ERR7732354	ERR7732355	ERR7732356	ERR7732358	ERR7732357	ERR7732359	ERR7732368	ERR7732369

ERR7732370	ERR7732371	ERR7732360	ERR7732361	ERR7732362	ERR7732363	ERR7732364	ERR7732365
ERR7732366	ERR7732367	ERR7732525	ERR7732526	ERR7732527	ERR7732528	ERR7732529	ERR7732530
ERR7732532	ERR7732533	ERR7732534	ERR7732535	ERR7732524	ERR7732531	ERR7732536	ERR7732537
ERR7732538	ERR7732539	ERR7732540	ERR7732541	ERR7732542	ERR7732543	ERR7732552	ERR7732553
ERR7732554	ERR7732555	ERR7732556	ERR7732557	ERR7732558	ERR7732559	ERR7732560	ERR7732561
ERR7732562	ERR7732563	ERR7732544	ERR7732545	ERR7732546	ERR7732547	ERR7732548	ERR7732549
ERR7732551	ERR7732550	ERR7732420	ERR7732421	ERR7732422	ERR7732423	ERR7732424	ERR7732425
ERR7732426	ERR7732427	ERR7732436	ERR7732437	ERR7732438	ERR7732439	ERR7732431	ERR7732428
ERR7732429	ERR7732430	ERR7732432	ERR7732433	ERR7732434	ERR7732435	ERR7732564	ERR7732565
ERR7732566	ERR7732567	ERR7732568	ERR7732569	ERR7732570	ERR7732571	ERR7732572	ERR7732573
ERR7732574	ERR7732575	ERR7732576	ERR7732577	ERR7732578	ERR7732579	ERR7732580	ERR7732581
ERR7732582	ERR7732583	ERR7732596	ERR7732597	ERR7732598	ERR7732599	ERR7732600	ERR7732601
ERR7732602	ERR7732603	ERR7732604	ERR7732605	ERR7732606	ERR7732607	ERR7732608	ERR7732610
ERR7732611	ERR7732609	ERR7732588	ERR7732584	ERR7732585	ERR7732586	ERR7732587	ERR7732589
ERR7732590	ERR7732591	ERR7732592	ERR7732593	ERR7732594	ERR7732595	ERR7996892	ERR7996893
ERR7996894	ERR7996895	ERR7996896	ERR7996897	ERR8523366	ERR8523367	ERR8523368	ERR8523369
ERR8523370	ERR8523371	ERR8523372	ERR8523373	ERR8523374	ERR8523375	ERR8523376	ERR8523377
ERR8523378	ERR8523379	ERR8523380	ERR8523381	ERR8523382	ERR8523383	ERR8523384	ERR8523385
ERR8523386	ERR8523387	ERR8523388	ERR8523389	ERR8523390	ERR8523391	ERR8523392	ERR8523393
ERR8523394	ERR8523395	ERR8523396	ERR8523397	ERR8523398	ERR8523399	ERR8523400	ERR8523401
ERR8523436	ERR8523402	ERR8523403	ERR8523404	ERR8523405	ERR8523406	ERR8523407	ERR8523408
ERR8523409	ERR8523410	ERR8523411	ERR8523412	ERR8523413	ERR8523414	ERR8523415	ERR8523416
ERR8523417	ERR8523418	ERR8523419	ERR8523420	ERR8523421	ERR8523422	ERR8523423	ERR8523424
ERR8523425	ERR8523426	ERR8523427	ERR8523428	ERR8523429	ERR8523430	ERR8523431	ERR8523432
ERR8523433	ERR8523434	ERR8523435	ERR8521105	ERR8521106	ERR8521107	ERR8521108	ERR8521109
ERR8521110	ERR8521111	ERR8521112	ERR8521113	ERR8521114	ERR413339	ERR413345	ERR413328
ERR413333	ERR413324	ERR413337	ERR413325	ERR413317	ERR413329	ERR413340	ERR413334
ERR413322	ERR413332	ERR413344	ERR413330	ERR413335	ERR413321	ERR413336	ERR413327
ERR413338	ERR413341	ERR413342	ERR413320	ERR413326	ERR413323	ERR413315	ERR413343
ERR413331	ERR413319	ERR413346	ERR413318	ERR413316	ERR9692801	ERR9692802	ERR9692803
ERR9692804	ERR9692805	ERR9692806	ERR9692807	ERR9692808	ERR9692809	ERR9692810	ERR9692811
ERR9692812	ERR9692813	ERR9692814	ERR9692815	ERR9692816	ERR9692817	ERR9692818	ERR9692819
ERR9692820	ERR9692821	ERR9692822	ERR9692823	ERR9692824	ERR9692825	ERR9692826	ERR9692827
ERR9692828	ERR9692829	ERR9692830	ERR9692831	ERR9692832	ERR9692833	ERR9692834	ERR9692835
ERR9692836	ERR9692837	ERR9692838	ERR9692839	ERR9692840	ERR9692841	ERR9692842	ERR9692843
ERR9692844	ERR9692845	ERR9692846	ERR9692847	ERR9692848	ERR9692849	ERR9692850	ERR9692851
ERR9692852	ERR9692853	ERR9692854	ERR9692855	ERR9692856	ERR9692857	ERR9692858	ERR9692859
ERR9692860	ERR9692861	ERR9692862	ERR9692863	ERR9692864	ERR9692865	ERR9692866	ERR9692867
ERR9692868	ERR9692869	ERR9692870	ERR9692871	ERR9692872	ERR9726179	ERR9726180	ERR9726181
ERR9726182	ERR9726183	ERR9726184	ERR9726185	ERR9726186	ERR9726187	ERR9726188	ERR9726189
ERR9726190	ERR9726191	ERR9726192	ERR9726193	ERR9726194	ERR9726195	ERR9726196	ERR9726197
ERR9726198	ERR9726199	ERR9726200	ERR9726201	ERR9726202	ERR9726203	ERR9726204	ERR9726205
ERR9726206	ERR9726207	ERR9726208	ERR9726209	ERR9726210	ERR9726211	ERR9726212	ERR9726213
ERR9726214	ERR9726215	ERR9726216	ERR9726217	ERR9726218	ERR9726219	ERR9726220	ERR9726221
ERR9726222	ERR9726223	ERR9726224	ERR9726225	ERR9726226	ERR9726227	ERR9726228	ERR9726229
ERR9726230	ERR9726231	ERR9726232	ERR9726233	ERR9726234	ERR9726235	ERR9726236	ERR9726237
ERR9726487	ERR9726238	ERR9726239	ERR9726240	ERR9726241	ERR9726242	ERR9726243	ERR9726244
ERR9726245	ERR9726246	ERR9726247	ERR9726248	ERR9726488	ERR9726249	ERR9726250	ERR9726251
ERR9726252	ERR9726253	ERR9726254	ERR9726255	ERR9726256	ERR9726257	ERR9726258	ERR9726259
ERR9726260	ERR9726261	ERR9726262	ERR9726263	ERR9726264	ERR9726265	ERR9726266	ERR9726267
ERR9726268	ERR9726269	ERR9726270	ERR9726271	ERR9726272	ERR9726273	ERR9726274	ERR9726275
ERR9726276	ERR9726277	ERR9726278	ERR9726279	ERR9726280	ERR9726281	ERR9726282	ERR9726283
ERR9726284	ERR9726285	ERR9726286	ERR9726287	ERR9726288	ERR9726289	ERR9726489	ERR9726290
ERR9726291	ERR9726292	ERR9726293	ERR9726294	ERR9726295	ERR9726490	ERR9726296	ERR9726297
ERR9726298	ERR9726299	ERR9726300	ERR9726301	ERR9726302	ERR9726303	ERR9726304	ERR9726305
ERR9726306	ERR9726307	ERR9726308	ERR9726309	ERR9726310	ERR9726311	ERR9726312	ERR9726313
ERR9726314	ERR9726315	ERR9726316	ERR9726317	ERR9726318	ERR9726319	ERR9726320	ERR9726321
ERR9726322	ERR9726323	ERR9726324	ERR9726325	ERR9726326	ERR9726327	ERR9726328	ERR9726329

ERR9726330	ERR9726331	ERR9726332	ERR9726333	ERR9726334	ERR9726335	ERR9726336	ERR9726337
ERR9726338	ERR9726339	ERR9726340	ERR9726341	ERR9726342	ERR9726343	ERR9726344	ERR9726345
ERR9726346	ERR9726347	ERR9726348	ERR9726349	ERR9726350	ERR9726351	ERR9726352	ERR9726353
ERR9726354	ERR9726355	ERR9726356	ERR9726357	ERR9726358	ERR9726359	ERR9726360	ERR9726361
ERR9726362	ERR9726363	ERR9726364	ERR9726365	ERR9726366	ERR9726367	ERR9726368	ERR9726369
ERR9726370	ERR9726371	ERR9726372	ERR9726373	ERR9726374	ERR9726375	ERR9726376	ERR9726377
ERR9726378	ERR9726379	ERR9726380	ERR9726381	ERR9726382	ERR9726383	ERR9726384	ERR9726385
ERR9726386	ERR9726387	ERR9726388	ERR9726389	ERR9726390	ERR9726391	ERR9726392	ERR9726393
ERR9726394	ERR9726395	ERR9726396	ERR9726397	ERR9726398	ERR9726399	ERR9726400	ERR9726401
ERR9726402	ERR9726403	ERR9726404	ERR9726405	ERR9726406	ERR9726407	ERR9726408	ERR9726409
ERR9726410	ERR9726411	ERR9726412	ERR9726413	ERR9726414	ERR9726415	ERR9726416	ERR9726417
ERR9726418	ERR9726419	ERR9726420	ERR9726421	ERR9726422	ERR9726423	ERR9726424	ERR9726425
ERR9726426	ERR9726427	ERR9726428	ERR9726429	ERR9726430	ERR9726431	ERR9726432	ERR9726433
ERR9726434	ERR9726435	ERR9726436	ERR9726437	ERR9726438	ERR9726439	ERR9726440	ERR9726441
ERR9726442	ERR9726443	ERR9726444	ERR9726445	ERR9726446	ERR9726447	ERR9726448	ERR9726449
ERR9726450	ERR9726451	ERR9726452	ERR9726453	ERR9726454	ERR9726455	ERR9726456	ERR9726457
ERR9726458	ERR9726459	ERR9726460	ERR9726461	ERR9726462	ERR9726463	ERR9726464	ERR9726465
ERR9726466	ERR9726467	ERR9726468	ERR9726469	ERR9726470	ERR9726471	ERR9726472	ERR9726473
ERR9726474	ERR9726475	ERR9726476	ERR9726477	ERR9726478	ERR9726479	ERR9726480	ERR9726481
ERR9726482	ERR9726483	ERR9726484	ERR9726485	ERR9726486	ERR9969607	ERR9969610	ERR9969611
ERR9969617	ERR9969613	ERR9969626	ERR9969627	ERR9969628	ERR9969612	ERR9969614	ERR9969619
ERR9969630	ERR9969618	ERR9969621	ERR9969622	ERR9969625	ERR9969608	ERR9969615	ERR9969620
ERR9969624	ERR9969609	ERR9969616	ERR9969623	ERR9969629	ERR9966314	ERR9966315	ERR9966316
ERR9966317	ERR9966318	ERR9966319	ERR9966320	ERR9966321	ERR9966322	ERR9966323	ERR9966324
ERR9966325	ERR9966326	ERR9966327	ERR9966328	ERR9966329	ERR9966330	ERR9966331	ERR9966332
ERR9966333	ERR9966334	ERR9966335	ERR9966336	ERR9966337	ERR10030011	ERR10030012	ERR10030013
ERR10030014	ERR10030015	ERR10030016	ERR10030017	ERR10030018	ERR10030019	ERR10030020	ERR10030021
ERR10030022	ERR10030023	ERR10030024	ERR10030025	ERR10030026	ERR10030027	ERR10030028	ERR10030029
ERR10030030	ERR10156025	ERR10156026	ERR10156027	ERR10156028	ERR10156029	ERR10156030	ERR10156031
ERR10156032	ERR10156033	ERR10156034	ERR10156035	ERR10156036	ERR10368082	ERR10368083	ERR10368084
ERR10368085	ERR10368086	ERR10368087	ERR10368088	ERR10368089	ERR10368090	ERR10368091	ERR10385979
ERR10385980	ERR10385981	ERR10385982	ERR10385983	ERR10385984	ERR10385985	ERR10385986	ERR10385987
ERR10385988	ERR10463979	ERR10492766	ERR10492767	ERR10492768	ERR10492769	ERR10492770	ERR10492771
ERR10492772	ERR10492773	ERR10492774	ERR10492775	ERR10492776	ERR10492777	ERR10492778	ERR10492779
ERR10492780	ERR10492781	ERR10492782	ERR10492783	ERR10492784	ERR10492785	ERR10492786	ERR10492787
ERR10492788	ERR10492789	ERR10492790	ERR10492791	ERR10492792	ERR10492793	ERR10492794	ERR10492795
ERR10492796	ERR10492797	ERR10492798	ERR10492799	ERR10492800	ERR10492801	ERR10492802	ERR10492803
ERR10492804	ERR10492805	ERR10492806	ERR10492807	ERR10492808	ERR10492809	ERR10492810	ERR10492811
ERR10492812	ERR10492813	ERR10492814	ERR10492815	ERR10492816	ERR10492817	ERR10567630	ERR10567704
ERR10567741	ERR10567778	ERR10567667	ERR10567815	ERR10567852	ERR10567889	ERR10565311	ERR10565309
ERR10565310	ERR10565312	ERR10569238	ERR10569334	ERR10569950	ERR10569430	ERR10569526	ERR10569662
ERR10569758	ERR10569854	ERR10567116	ERR10567110	ERR10567112	ERR10567114	ERR10567118	ERR10567150
ERR10567182	ERR10567214	ERR10566018	ERR10566031	ERR10566057	ERR10566070	ERR10566083	ERR10566005
ERR10566044	ERR10566096	ERR10569431	ERR10569527	ERR10569663	ERR10569239	ERR10569335	ERR10569759
ERR10569855	ERR10569951	ERR10567111	ERR10567113	ERR10567117	ERR10567119	ERR10567183	ERR10567215
ERR10567115	ERR10567151	ERR10565430	ERR10565405	ERR10565455	ERR10565480	ERR10569336	ERR10569528
ERR10569760	ERR10569856	ERR10569952	ERR10569240	ERR10569432	ERR10569664	ERR10567742	ERR10567816
ERR10567631	ERR10567668	ERR10567705	ERR10567779	ERR10567853	ERR10567890	ERR10565431	ERR10565481
ERR10565406	ERR10565456	ERR10569761	ERR10569857	ERR10569953	ERR10569241	ERR10569337	ERR10569433
ERR10569529	ERR10569665	ERR10567434	ERR10567452	ERR10567470	ERR10567488	ERR10567524	ERR10567542
ERR10567560	ERR10567506	ERR10565407	ERR10565457	ERR10565432	ERR10565482	ERR10569434	ERR10569530
ERR10569666	ERR10569762	ERR10569954	ERR10569242	ERR10569338	ERR10569858	ERR10567632	ERR10567706
ERR10567854	ERR10567669	ERR10567743	ERR10567780	ERR10567817	ERR10567891	ERR10565621	ERR10565699
ERR10565712	ERR10565634	ERR10565647	ERR10565660	ERR10565673	ERR10565686	ERR10569243	ERR10569435
ERR10569763	ERR10569955	ERR10569339	ERR10569531	ERR10569667	ERR10569859	ERR10567435	ERR10567453
ERR10567489	ERR10567525	ERR10567543	ERR10567561	ERR10567471	ERR10567507	ERR10565408	ERR10565433
ERR10565458	ERR10565483	ERR10569532	ERR10569668	ERR10569860	ERR10569956	ERR10569244	ERR10569340
ERR10569436	ERR10569764	ERR10567633	ERR10567670	ERR10567855	ERR10567707	ERR10567744	ERR10567781

ERR10567818	ERR10567892	ERR10565434	ERR10565484	ERR10565409	ERR10565459	ERR10569245	ERR10569341
ERR10569437	ERR10569533	ERR10569632	ERR10569642	ERR10569669	ERR10569957	ERR10570056	ERR10570066
ERR10570076	ERR10569622	ERR10569652	ERR10569765	ERR10569861	ERR10570046	ERR10567634	ERR10567671
ERR10567708	ERR10567745	ERR10567819	ERR10567893	ERR10567782	ERR10567856	ERR10566006	ERR10566019
ERR10566032	ERR10566045	ERR10566058	ERR10566071	ERR10566084	ERR10566097	ERR10569534	ERR10569623
ERR10569633	ERR10569643	ERR10569766	ERR10569862	ERR10569958	ERR10570047	ERR10570057	ERR10570067
ERR10569246	ERR10569342	ERR10569438	ERR10569653	ERR10569670	ERR10570077	ERR10567436	ERR10567454
ERR10567472	ERR10567490	ERR10567508	ERR10567544	ERR10567562	ERR10567526	ERR10566007	ERR10566020
ERR10566033	ERR10566059	ERR10566046	ERR10566072	ERR10566085	ERR10566098	ERR10569247	ERR10569343
ERR10569671	ERR10569767	ERR10569959	ERR10569439	ERR10569535	ERR10569863	ERR10567417	ERR10567437
ERR10567455	ERR10567527	ERR10567545	ERR10567563	ERR10567366	ERR10567383	ERR10567400	ERR10567473
ERR10567491	ERR10567509	ERR10565410	ERR10565460	ERR10565435	ERR10565485	ERR10569344	ERR10569440
ERR10569536	ERR10569672	ERR10569768	ERR10569248	ERR10569864	ERR10569960	ERR10567709	ERR10567746
ERR10567857	ERR10567635	ERR10567672	ERR10567783	ERR10567820	ERR10567894	ERR10565622	ERR10565635
ERR10565648	ERR10565661	ERR10565687	ERR10565713	ERR10565674	ERR10565700	ERR10569345	ERR10569441
ERR10569537	ERR10569673	ERR10569249	ERR10569769	ERR10569865	ERR10569961	ERR10567636	ERR10567673
ERR10567710	ERR10567747	ERR10567784	ERR10567821	ERR10567858	ERR10567895	ERR10566099	ERR10566008
ERR10566021	ERR10566034	ERR10566047	ERR10566060	ERR10566073	ERR10566086	ERR10569442	ERR10569674
ERR10569962	ERR10569250	ERR10569346	ERR10569538	ERR10569770	ERR10569866	ERR10567637	ERR10567859
ERR10567674	ERR10567711	ERR10567748	ERR10567785	ERR10567822	ERR10567896	ERR10566009	ERR10566035
ERR10566048	ERR10566061	ERR10566022	ERR10566074	ERR10566087	ERR10566100	ERR10569251	ERR10569347
ERR10569443	ERR10569539	ERR10569675	ERR10569867	ERR10569963	ERR10569771	ERR10567438	ERR10567510
ERR10567528	ERR10567546	ERR10567456	ERR10567474	ERR10567492	ERR10567564	ERR10565411	ERR10565461
ERR10565436	ERR10565486	ERR10569348	ERR10569540	ERR10569676	ERR10569252	ERR10569444	ERR10569772
ERR10569868	ERR10569964	ERR10567367	ERR10567384	ERR10567418	ERR10567439	ERR10567475	ERR10567401
ERR10567457	ERR10567493	ERR10567511	ERR10567529	ERR10567547	ERR10567565	ERR10566023	ERR10566036
ERR10566062	ERR10566075	ERR10566101	ERR10566010	ERR10566049	ERR10566088	ERR10569253	ERR10569445
ERR10569541	ERR10569677	ERR10569773	ERR10569869	ERR10569349	ERR10569965	ERR10567638	ERR10567675
ERR10567749	ERR10567860	ERR10567897	ERR10567712	ERR10567786	ERR10567823	ERR10565623	ERR10565636
ERR10565649	ERR10565662	ERR10565701	ERR10565714	ERR10565675	ERR10565688	ERR10569254	ERR10569350
ERR10569542	ERR10569774	ERR10569870	ERR10569446	ERR10569678	ERR10569966	ERR10567385	ERR10567402
ERR10567440	ERR10567512	ERR10567548	ERR10567368	ERR10567419	ERR10567458	ERR10567476	ERR10567494
ERR10567530	ERR10567566	ERR10565605	ERR10565613	ERR10565617	ERR10565624	ERR10565650	ERR10565663
ERR10565676	ERR10565689	ERR10565702	ERR10565715	ERR10565609	ERR10565637	ERR10569447	ERR10569543
ERR10569679	ERR10569967	ERR10569255	ERR10569351	ERR10569775	ERR10569871	ERR10567120	ERR10567184
ERR10567246	ERR10567306	ERR10567152	ERR10567216	ERR10567276	ERR10567336	ERR10565487	ERR10565412
ERR10565437	ERR10565462	ERR10569256	ERR10569352	ERR10569448	ERR10569776	ERR10569544	ERR10569680
ERR10569872	ERR10569968	ERR10567639	ERR10567713	ERR10567750	ERR10567787	ERR10567676	ERR10567824
ERR10567861	ERR10567898	ERR10565488	ERR10565413	ERR10565438	ERR10565463	ERR10569353	ERR10569449
ERR10569545	ERR10569681	ERR10569873	ERR10569969	ERR10569257	ERR10569777	ERR10567677	ERR10567714
ERR10567751	ERR10567788	ERR10567825	ERR10567862	ERR10567899	ERR10567640	ERR10565414	ERR10565464
ERR10565439	ERR10565489	ERR10569354	ERR10569682	ERR10569258	ERR10569450	ERR10569546	ERR10569778
ERR10569874	ERR10569970	ERR10567441	ERR10567477	ERR10567531	ERR10567567	ERR10567459	ERR10567495
ERR10567513	ERR10567549	ERR10565789	ERR10565816	ERR10565843	ERR10565897	ERR10565924	ERR10565978
ERR10565870	ERR10565951	ERR10569259	ERR10569355	ERR10569451	ERR10569683	ERR10569779	ERR10569875
ERR10569971	ERR10569547	ERR10567442	ERR10567550	ERR10567568	ERR10567460	ERR10567478	ERR10567496
ERR10567514	ERR10567532	ERR10565440	ERR10565490	ERR10565415	ERR10565465	ERR10569260	ERR10569356
ERR10569684	ERR10569780	ERR10569452	ERR10569548	ERR10569876	ERR10569972	ERR10567678	ERR10567715
ERR10567752	ERR10567789	ERR10567641	ERR10567826	ERR10567863	ERR10567900	ERR10565466	ERR10565416
ERR10565441	ERR10565491	ERR10569357	ERR10569453	ERR10569624	ERR10569634	ERR10569644	ERR10569877
ERR10570058	ERR10570068	ERR10570078	ERR10569261	ERR10569549	ERR10569654	ERR10569685	ERR10569781
ERR10569973	ERR10570048	ERR10567679	ERR10567716	ERR10567753	ERR10567790	ERR10567642	ERR10567827
ERR10567864	ERR10567901	ERR10566011	ERR10566024	ERR10566050	ERR10566076	ERR10566102	ERR10566037
ERR10566063	ERR10566089	ERR10569358	ERR10569782	ERR10569974	ERR10569262	ERR10569454	ERR10569550
ERR10569686	ERR10569878	ERR10567369	ERR10567386	ERR10567403	ERR10567443	ERR10567461	ERR10567479
ERR10567533	ERR10567551	ERR10567420	ERR10567497	ERR10567515	ERR10567569	ERR10565313	ERR10565359
ERR10565336	ERR10565382	ERR10565417	ERR10565442	ERR10565467	ERR10565492	ERR10569455	ERR10569263
ERR10569359	ERR10569551	ERR10569687	ERR10569879	ERR10569975	ERR10569783	ERR10567121	ERR10567153

ERR10567277	ERR10567307	ERR10567337	ERR10567185	ERR10567217	ERR10567247	ERR10565844	ERR10565898
ERR10565952	ERR10565979	ERR10565790	ERR10565817	ERR10565871	ERR10565925	ERR10569264	ERR10569456
ERR10569552	ERR10569688	ERR10569784	ERR10569880	ERR10569976	ERR10569360	ERR10567717	ERR10567643
ERR10567680	ERR10567754	ERR10567791	ERR10567828	ERR10567865	ERR10567902	ERR10565606	ERR10565614
ERR10565625	ERR10565638	ERR10565677	ERR10565690	ERR10565703	ERR10565716	ERR10565610	ERR10565618
ERR10565651	ERR10565664	ERR10569977	ERR10569265	ERR10569361	ERR10569457	ERR10569553	ERR10569689
ERR10569785	ERR10569881	ERR10567444	ERR10567462	ERR10567498	ERR10567516	ERR10567534	ERR10567570
ERR10567480	ERR10567552	ERR10565314	ERR10565360	ERR10565383	ERR10565443	ERR10565468	ERR10565493
ERR10565337	ERR10565418	ERR10569266	ERR10569362	ERR10569458	ERR10569554	ERR10569786	ERR10569978
ERR10569690	ERR10569882	ERR10567445	ERR10567463	ERR10567481	ERR10567499	ERR10567517	ERR10567535
ERR10567553	ERR10567571	ERR10565725	ERR10565757	ERR10565773	ERR10565791	ERR10565818	ERR10565845
ERR10565899	ERR10565953	ERR10565980	ERR10565741	ERR10565872	ERR10565926	ERR10569555	ERR10569883
ERR10569979	ERR10569267	ERR10569363	ERR10569459	ERR10569691	ERR10569787	ERR10567446	ERR10567464
ERR10567482	ERR10567500	ERR10567518	ERR10567536	ERR10567554	ERR10567572	ERR10565607	ERR10565611
ERR10565615	ERR10565619	ERR10565652	ERR10565665	ERR10565678	ERR10565691	ERR10565704	ERR10565717
ERR10565626	ERR10565639	ERR10569268	ERR10569364	ERR10569460	ERR10569692	ERR10569980	ERR10569556
ERR10569788	ERR10569884	ERR10567681	ERR10567718	ERR10567829	ERR10567644	ERR10567755	ERR10567792
ERR10567866	ERR10567903	ERR10565384	ERR10565444	ERR10565494	ERR10565315	ERR10565338	ERR10565361
ERR10565419	ERR10565469	ERR10569365	ERR10569557	ERR10569693	ERR10569789	ERR10569885	ERR10569269
ERR10569461	ERR10569981	ERR10567645	ERR10567793	ERR10567830	ERR10567867	ERR10567682	ERR10567719
ERR10567756	ERR10567904	ERR10565627	ERR10565640	ERR10565679	ERR10565705	ERR10565653	ERR10565666
ERR10565692	ERR10565718	ERR10569366	ERR10569462	ERR10569694	ERR10569982	ERR10569270	ERR10569558
ERR10569790	ERR10569886	ERR10567683	ERR10567720	ERR10567868	ERR10567905	ERR10567646	ERR10567757
ERR10567794	ERR10567831	ERR10565316	ERR10565339	ERR10565495	ERR10565362	ERR10565385	ERR10565420
ERR10565445	ERR10565470	ERR10569367	ERR10569791	ERR10569271	ERR10569463	ERR10569559	ERR10569695
ERR10569887	ERR10569983	ERR10567248	ERR10567308	ERR10567338	ERR10567122	ERR10567154	ERR10567186
ERR10567218	ERR10567278	ERR10565628	ERR10565667	ERR10565706	ERR10565641	ERR10565654	ERR10565680
ERR10565693	ERR10565719	ERR10569272	ERR10569464	ERR10569888	ERR10569984	ERR10569368	ERR10569560
ERR10569696	ERR10569792	ERR10567684	ERR10567721	ERR10567758	ERR10567832	ERR10567869	ERR10567906
ERR10567647	ERR10567795	ERR10565340	ERR10565421	ERR10565446	ERR10565317	ERR10565363	ERR10565386
ERR10565471	ERR10565496	ERR10569369	ERR10569697	ERR10569889	ERR10569273	ERR10569465	ERR10569561
ERR10569793	ERR10569985	ERR10567465	ERR10567519	ERR10567555	ERR10567573	ERR10567447	ERR10567483
ERR10567501	ERR10567537	ERR10566025	ERR10566051	ERR10566090	ERR10566012	ERR10566038	ERR10566064
ERR10566077	ERR10566103	ERR10569466	ERR10569562	ERR10569698	ERR10569794	ERR10569890	ERR10569986
ERR10569274	ERR10569370	ERR10567448	ERR10567466	ERR10567502	ERR10567556	ERR10567574	ERR10567484
ERR10567520	ERR10567538	ERR10565726	ERR10565742	ERR10565758	ERR10565774	ERR10565792	ERR10565819
ERR10565846	ERR10565900	ERR10565927	ERR10565954	ERR10565873	ERR10565981	ERR10569275	ERR10569371
ERR10569467	ERR10569563	ERR10569699	ERR10569795	ERR10569891	ERR10569987	ERR10567685	ERR10567722
ERR10567648	ERR10567759	ERR10567796	ERR10567833	ERR10567870	ERR10567907	ERR10565743	ERR10565759
ERR10565793	ERR10565847	ERR10565928	ERR10565727	ERR10565775	ERR10565820	ERR10565874	ERR10565901
ERR10565955	ERR10565982	ERR10569276	ERR10569468	ERR10569564	ERR10569700	ERR10569796	ERR10569892
ERR10569372	ERR10569988	ERR10567723	ERR10567871	ERR10567649	ERR10567686	ERR10567760	ERR10567797
ERR10567834	ERR10567908	ERR10565728	ERR10565744	ERR10565776	ERR10565794	ERR10565821	ERR10565902
ERR10565956	ERR10565760	ERR10565848	ERR10565875	ERR10565929	ERR10565983	ERR10569277	ERR10569373
ERR10569625	ERR10569645	ERR10569655	ERR10569701	ERR10569797	ERR10569893	ERR10570049	ERR10570069
ERR10570079	ERR10569469	ERR10569565	ERR10569635	ERR10569989	ERR10570059	ERR10567449	ERR10567467
ERR10567485	ERR10567539	ERR10567557	ERR10567575	ERR10567503	ERR10567521	ERR10565745	ERR10565777
ERR10565795	ERR10565822	ERR10565849	ERR10565876	ERR10565903	ERR10565729	ERR10565761	ERR10565930
ERR10565957	ERR10565984	ERR10569278	ERR10569470	ERR10569566	ERR10569702	ERR10569798	ERR10569374
ERR10569894	ERR10569990	ERR10567909	ERR10567687	ERR10567761	ERR10567650	ERR10567724	ERR10567798
ERR10567835	ERR10567872	ERR10566013	ERR10566026	ERR10566052	ERR10566065	ERR10566039	ERR10566078
ERR10566091	ERR10566104	ERR10569375	ERR10569567	ERR10569703	ERR10569895	ERR10569279	ERR10569471
ERR10569799	ERR10569991	ERR10567468	ERR10567486	ERR10567504	ERR10567540	ERR10567558	ERR10567576
ERR10567450	ERR10567522	ERR10565608	ERR10565612	ERR10565616	ERR10565620	ERR10565629	ERR10565668
ERR10565681	ERR10565694	ERR10565707	ERR10565642	ERR10565655	ERR10565720	ERR10569376	ERR10569704
ERR10569800	ERR10569992	ERR10569280	ERR10569472	ERR10569568	ERR10569896	ERR10567688	ERR10567799
ERR10567910	ERR10567651	ERR10567725	ERR10567762	ERR10567836	ERR10567873	ERR10565630	ERR10565656
ERR10565682	ERR10565695	ERR10565708	ERR10565643	ERR10565669	ERR10565721	ERR10569281	ERR10569377

ERR10569801	ERR10569897	ERR10569473	ERR10569569	ERR10569705	ERR10569993	ERR10567652	ERR10567689
ERR10567726	ERR10567763	ERR10567800	ERR10567837	ERR10567874	ERR10567911	ERR10565364	ERR10565387
ERR10565318	ERR10565341	ERR10565422	ERR10565447	ERR10565472	ERR10565497	ERR10569282	ERR10569378
ERR10569570	ERR10569706	ERR10569802	ERR10569898	ERR10569474	ERR10569994	ERR10567653	ERR10567727
ERR10567801	ERR10567912	ERR10567690	ERR10567764	ERR10567838	ERR10567875	ERR10565365	ERR10565388
ERR10565473	ERR10565498	ERR10565319	ERR10565342	ERR10565423	ERR10565448	ERR10569283	ERR10569475
ERR10569571	ERR10569803	ERR10569899	ERR10569995	ERR10569379	ERR10569707	ERR10567451	ERR10567487
ERR10567505	ERR10567523	ERR10567541	ERR10567559	ERR10567577	ERR10567469	ERR10565730	ERR10565746
ERR10565762	ERR10565796	ERR10565850	ERR10565904	ERR10565931	ERR10565778	ERR10565823	ERR10565877
ERR10565958	ERR10565985	ERR10569572	ERR10569804	ERR10569996	ERR10569284	ERR10569380	ERR10569476
ERR10569708	ERR10569900	ERR10567123	ERR10567187	ERR10567249	ERR10567279	ERR10567309	ERR10567339
ERR10567155	ERR10567219	ERR10566027	ERR10566092	ERR10566105	ERR10566014	ERR10566040	ERR10566053
ERR10566066	ERR10566079	ERR10569381	ERR10569901	ERR10569285	ERR10569477	ERR10569573	ERR10569709
ERR10569805	ERR10569997	ERR10567280	ERR10567340	ERR10567124	ERR10567156	ERR10567188	ERR10567220
ERR10567250	ERR10567310	ERR10565366	ERR10565389	ERR10565424	ERR10565474	ERR10565499	ERR10565320
ERR10565343	ERR10565449	ERR10569478	ERR10569902	ERR10569998	ERR10569286	ERR10569382	ERR10569574
ERR10569710	ERR10569806	ERR10567125	ERR10567251	ERR10567157	ERR10567189	ERR10567221	ERR10567281
ERR10567311	ERR10567341	ERR10565747	ERR10565824	ERR10565905	ERR10565959	ERR10565986	ERR10565731
ERR10565763	ERR10565779	ERR10565797	ERR10565851	ERR10565878	ERR10565932	ERR10569287	ERR10569383
ERR10569575	ERR10569711	ERR10569807	ERR10569903	ERR10569479	ERR10569999	ERR10567370	ERR10567387
ERR10567404	ERR10567578	ERR10567591	ERR10567617	ERR10567421	ERR10567604	ERR10565732	ERR10565798
ERR10565825	ERR10565879	ERR10565906	ERR10565960	ERR10565748	ERR10565764	ERR10565780	ERR10565852
ERR10565933	ERR10565987	ERR10569480	ERR10569576	ERR10569712	ERR10569904	ERR10570000	ERR10569288
ERR10569384	ERR10569808	ERR10567190	ERR10567282	ERR10567312	ERR10567342	ERR10567126	ERR10567158
ERR10567222	ERR10567252	ERR10565733	ERR10565781	ERR10565799	ERR10565934	ERR10565988	ERR10565749
ERR10565765	ERR10565826	ERR10565853	ERR10565880	ERR10565907	ERR10565961	ERR10569577	ERR10569289
ERR10569385	ERR10569481	ERR10569713	ERR10569809	ERR10569905	ERR10570001	ERR10567127	ERR10567253
ERR10567313	ERR10567343	ERR10567159	ERR10567191	ERR10567223	ERR10567283	ERR10565734	ERR10565750
ERR10565827	ERR10565854	ERR10565881	ERR10565766	ERR10565782	ERR10565800	ERR10565908	ERR10565935
ERR10565962	ERR10565989	ERR10569290	ERR10569482	ERR10569714	ERR10569810	ERR10569906	ERR10569386
ERR10569578	ERR10570002	ERR10567314	ERR10567128	ERR10567160	ERR10567192	ERR10567224	ERR10567254
ERR10567284	ERR10567344	ERR10565735	ERR10565751	ERR10565767	ERR10565783	ERR10565828	ERR10565855
ERR10565936	ERR10565963	ERR10565801	ERR10565882	ERR10565909	ERR10565990	ERR10569715	ERR10569811
ERR10569907	ERR10569291	ERR10569387	ERR10569483	ERR10569579	ERR10570003	ERR10567654	ERR10567691
ERR10567728	ERR10567802	ERR10567765	ERR10567839	ERR10567876	ERR10567913	ERR10566080	ERR10566015
ERR10566028	ERR10566041	ERR10566054	ERR10566067	ERR10566093	ERR10566106	ERR10569292	ERR10569484
ERR10569580	ERR10569716	ERR10569908	ERR10569388	ERR10569812	ERR10570004	ERR10567371	ERR10567405
ERR10567579	ERR10567605	ERR10567618	ERR10567388	ERR10567422	ERR10567592	ERR10565736	ERR10565856
ERR10565883	ERR10565910	ERR10565937	ERR10565964	ERR10565991	ERR10565752	ERR10565768	ERR10565784
ERR10565802	ERR10565829	ERR10569389	ERR10569485	ERR10569581	ERR10569293	ERR10569717	ERR10569813
ERR10569909	ERR10570005	ERR10567655	ERR10567692	ERR10567729	ERR10567803	ERR10567766	ERR10567840
ERR10567877	ERR10567914	ERR10565737	ERR10565753	ERR10565769	ERR10565785	ERR10565803	ERR10565830
ERR10565884	ERR10565938	ERR10565965	ERR10565992	ERR10565857	ERR10565911	ERR10569486	ERR10569718
ERR10569814	ERR10569910	ERR10570006	ERR10569294	ERR10569390	ERR10569582	ERR10567656	ERR10567693
ERR10567730	ERR10567878	ERR10567915	ERR10567767	ERR10567804	ERR10567841	ERR10565644	ERR10565657
ERR10565683	ERR10565696	ERR10565631	ERR10565670	ERR10565709	ERR10565722	ERR10569391	ERR10569487
ERR10569719	ERR10569815	ERR10569911	ERR10570007	ERR10569295	ERR10569583	ERR10567842	ERR10567879
ERR10567916	ERR10567657	ERR10567694	ERR10567731	ERR10567768	ERR10567805	ERR10565378	ERR10565401
ERR10565332	ERR10565355	ERR10569296	ERR10569392	ERR10569488	ERR10569816	ERR10569912	ERR10569584
ERR10569720	ERR10570008	ERR10567372	ERR10567389	ERR10567406	ERR10567423	ERR10567580	ERR10567593
ERR10567606	ERR10567619	ERR10566016	ERR10566055	ERR10566094	ERR10566029	ERR10566042	ERR10566068
ERR10566081	ERR10566107	ERR10569489	ERR10569721	ERR10569913	ERR10570009	ERR10569297	ERR10569393
ERR10569585	ERR10569817	ERR10567129	ERR10567285	ERR10567161	ERR10567193	ERR10567225	ERR10567255
ERR10567315	ERR10567345	ERR10565738	ERR10565754	ERR10565786	ERR10565804	ERR10565858	ERR10565912
ERR10565939	ERR10565966	ERR10565993	ERR10565770	ERR10565831	ERR10565885	ERR10569394	ERR10569586
ERR10569818	ERR10570010	ERR10569298	ERR10569490	ERR10569722	ERR10569914	ERR10567695	ERR10567732
ERR10567769	ERR10567843	ERR10567880	ERR10567658	ERR10567806	ERR10567917	ERR10565755	ERR10565771
ERR10565787	ERR10565805	ERR10565886	ERR10565913	ERR10565940	ERR10565967	ERR10565994	ERR10565739

ERR10565832	ERR10565859	ERR10569395	ERR10569491	ERR10569587	ERR10569819	ERR10569915	ERR10569299
ERR10569723	ERR10570011	ERR10567194	ERR10567316	ERR10567130	ERR10567162	ERR10567226	ERR10567256
ERR10567286	ERR10567346	ERR10566082	ERR10566017	ERR10566030	ERR10566043	ERR10566056	ERR10566069
ERR10566095	ERR10566108	ERR10569300	ERR10569492	ERR10570012	ERR10569396	ERR10569588	ERR10569724
ERR10569820	ERR10569916	ERR10567733	ERR10567844	ERR10567918	ERR10567659	ERR10567696	ERR10567770
ERR10567807	ERR10567881	ERR10565645	ERR10565658	ERR10565684	ERR10565723	ERR10565632	ERR10565671
ERR10565697	ERR10565710	ERR10569821	ERR10569301	ERR10569397	ERR10569493	ERR10569589	ERR10569725
ERR10569917	ERR10570013	ERR10567660	ERR10567734	ERR10567771	ERR10567808	ERR10567882	ERR10567697
ERR10567845	ERR10567919	ERR10565740	ERR10565756	ERR10565772	ERR10565788	ERR10565806	ERR10565833
ERR10565860	ERR10565914	ERR10565941	ERR10565887	ERR10565968	ERR10565995	ERR10569398	ERR10569494
ERR10570014	ERR10569302	ERR10569590	ERR10569726	ERR10569822	ERR10569918	ERR10567661	ERR10567735
ERR10567698	ERR10567772	ERR10567809	ERR10567846	ERR10567883	ERR10567920	ERR10565646	ERR10565659
ERR10565672	ERR10565685	ERR10565724	ERR10565633	ERR10565698	ERR10565711	ERR10569495	ERR10569727
ERR10569823	ERR10569303	ERR10569399	ERR10569591	ERR10569919	ERR10570015	ERR10567131	ERR10567163
ERR10567195	ERR10567257	ERR10567287	ERR10567317	ERR10567227	ERR10567347	ERR10565861	ERR10565888
ERR10565807	ERR10565834	ERR10565915	ERR10565942	ERR10565969	ERR10565996	ERR10569304	ERR10569400
ERR10569592	ERR10569728	ERR10570016	ERR10569496	ERR10569824	ERR10569920	ERR10567132	ERR10567228
ERR10567258	ERR10567288	ERR10567348	ERR10567164	ERR10567196	ERR10567318	ERR10565367	ERR10565475
ERR10565321	ERR10565344	ERR10565390	ERR10565425	ERR10565450	ERR10565500	ERR10569593	ERR10569729
ERR10569921	ERR10570017	ERR10569305	ERR10569401	ERR10569497	ERR10569825	ERR10567197	ERR10567229
ERR10567259	ERR10567289	ERR10567319	ERR10567349	ERR10567133	ERR10567165	ERR10565368	ERR10565391
ERR10565426	ERR10565451	ERR10565476	ERR10565501	ERR10565322	ERR10565345	ERR10569306	ERR10569402
ERR10569594	ERR10569730	ERR10569826	ERR10569922	ERR10570018	ERR10569498	ERR10567134	ERR10567230
ERR10567260	ERR10567320	ERR10567166	ERR10567198	ERR10567290	ERR10567350	ERR10565369	ERR10565392
ERR10565323	ERR10565346	ERR10565427	ERR10565452	ERR10565477	ERR10565502	ERR10569595	ERR10570019
ERR10569307	ERR10569403	ERR10569499	ERR10569731	ERR10569827	ERR10569923	ERR10567373	ERR10567390
ERR10567407	ERR10567424	ERR10567581	ERR10567594	ERR10567607	ERR10567620	ERR10565835	ERR10565916
ERR10565943	ERR10565970	ERR10565997	ERR10565808	ERR10565862	ERR10565889	ERR10569308	ERR10569404
ERR10569500	ERR10569596	ERR10569732	ERR10569828	ERR10569924	ERR10570020	ERR10567135	ERR10567199
ERR10567231	ERR10567261	ERR10567167	ERR10567291	ERR10567321	ERR10567351	ERR10565809	ERR10565836
ERR10565863	ERR10565890	ERR10565971	ERR10565917	ERR10565944	ERR10565998	ERR10569405	ERR10569501
ERR10569829	ERR10569309	ERR10569597	ERR10569733	ERR10569925	ERR10570021	ERR10567374	ERR10567595
ERR10567391	ERR10567408	ERR10567425	ERR10567582	ERR10567608	ERR10567621	ERR10565505	ERR10565531
ERR10565544	ERR10565569	ERR10565581	ERR10565593	ERR10565518	ERR10565557	ERR10569310	ERR10569406
ERR10569502	ERR10569830	ERR10569598	ERR10569734	ERR10569926	ERR10570022	ERR10567168	ERR10567352
ERR10567136	ERR10567200	ERR10567232	ERR10567262	ERR10567292	ERR10567322	ERR10565519	ERR10565545
ERR10565570	ERR10565582	ERR10565594	ERR10565506	ERR10565532	ERR10565558	ERR10569636	ERR10569646
ERR10569656	ERR10569735	ERR10569831	ERR10569927	ERR10570050	ERR10570060	ERR10570080	ERR10569311
ERR10569407	ERR10569503	ERR10569599	ERR10569626	ERR10570023	ERR10570070	ERR10567375	ERR10567392
ERR10567409	ERR10567583	ERR10567609	ERR10567426	ERR10567596	ERR10567622	ERR10565533	ERR10565546
ERR10565507	ERR10565520	ERR10565559	ERR10565571	ERR10565583	ERR10565595	ERR10569312	ERR10569408
ERR10569627	ERR10569637	ERR10569647	ERR10569657	ERR10569832	ERR10569928	ERR10570061	ERR10569504
ERR10569600	ERR10569736	ERR10570024	ERR10570051	ERR10570071	ERR10570081	ERR10567137	ERR10567293
ERR10567323	ERR10567169	ERR10567201	ERR10567233	ERR10567263	ERR10567353	ERR10565508	ERR10565521
ERR10565547	ERR10565572	ERR10565534	ERR10565560	ERR10565584	ERR10565596	ERR10569505	ERR10569601
ERR10569833	ERR10570025	ERR10569313	ERR10569409	ERR10569737	ERR10569929	ERR10567662	ERR10567699
ERR10567736	ERR10567847	ERR10567884	ERR10567773	ERR10567810	ERR10567921	ERR10565333	ERR10565402
ERR10565428	ERR10565356	ERR10565379	ERR10565453	ERR10565478	ERR10565503	ERR10569314	ERR10569410
ERR10569506	ERR10569628	ERR10570052	ERR10570082	ERR10569602	ERR10569638	ERR10569648	ERR10569658
ERR10569738	ERR10569834	ERR10569930	ERR10570026	ERR10570062	ERR10570072	ERR10567138	ERR10567170
ERR10567234	ERR10567264	ERR10567294	ERR10567324	ERR10567354	ERR10567202	ERR10565535	ERR10565585
ERR10565597	ERR10565509	ERR10565522	ERR10565548	ERR10565561	ERR10565573	ERR10569507	ERR10569603
ERR10569739	ERR10569835	ERR10569931	ERR10570027	ERR10569315	ERR10569411	ERR10567139	ERR10567203
ERR10567325	ERR10567355	ERR10567171	ERR10567235	ERR10567265	ERR10567295	ERR10565549	ERR10565574
ERR10565586	ERR10565510	ERR10565523	ERR10565536	ERR10565562	ERR10565598	ERR10569836	ERR10569932
ERR10569316	ERR10569412	ERR10569508	ERR10569604	ERR10569740	ERR10570028	ERR10567700	ERR10567663
ERR10567737	ERR10567774	ERR10567811	ERR10567848	ERR10567885	ERR10567922	ERR10565537	ERR10565550
ERR10565563	ERR10565587	ERR10565599	ERR10565511	ERR10565524	ERR10565575	ERR10569509	ERR10569605

ERR10569837	ERR10569933	ERR10570029	ERR10569317	ERR10569413	ERR10569741	ERR10567775	ERR10567812
ERR10567849	ERR10567664	ERR10567701	ERR10567738	ERR10567886	ERR10567923	ERR10565538	ERR10565551
ERR10565564	ERR10565588	ERR10565600	ERR10565512	ERR10565525	ERR10565576	ERR10569318	ERR10569742
ERR10570030	ERR10569414	ERR10569510	ERR10569606	ERR10569838	ERR10569934	ERR10567393	ERR10567410
ERR10567427	ERR10567584	ERR10567610	ERR10567623	ERR10567376	ERR10567597	ERR10565526	ERR10565565
ERR10565577	ERR10565589	ERR10565513	ERR10565539	ERR10565552	ERR10565601	ERR10569415	ERR10569511
ERR10569839	ERR10570031	ERR10569319	ERR10569607	ERR10569743	ERR10569935	ERR10567172	ERR10567236
ERR10567296	ERR10567326	ERR10567140	ERR10567204	ERR10567266	ERR10567356	ERR10565527	ERR10565566
ERR10565578	ERR10565602	ERR10565514	ERR10565540	ERR10565553	ERR10565590	ERR10569320	ERR10569416
ERR10569744	ERR10569840	ERR10569936	ERR10570032	ERR10569512	ERR10569608	ERR10567141	ERR10567237
ERR10567267	ERR10567327	ERR10567357	ERR10567173	ERR10567205	ERR10567297	ERR10565864	ERR10565810
ERR10565945	ERR10565972	ERR10565999	ERR10565837	ERR10565891	ERR10565918	ERR10569513	ERR10569609
ERR10569745	ERR10569937	ERR10570033	ERR10569321	ERR10569417	ERR10569841	ERR10567377	ERR10567394
ERR10567411	ERR10567428	ERR10567598	ERR10567585	ERR10567611	ERR10567624	ERR10565838	ERR10565865
ERR10565919	ERR10565973	ERR10565811	ERR10565892	ERR10565946	ERR10566000	ERR10569322	ERR10569418
ERR10569514	ERR10569746	ERR10569842	ERR10569938	ERR10569610	ERR10570034	ERR10567378	ERR10567412
ERR10567429	ERR10567599	ERR10567612	ERR10567625	ERR10567395	ERR10567586	ERR10565866	ERR10565920
ERR10565974	ERR10565812	ERR10565839	ERR10565893	ERR10565947	ERR10566001	ERR10569419	ERR10569515
ERR10569611	ERR10569747	ERR10569843	ERR10570035	ERR10569323	ERR10569939	ERR10567379	ERR10567396
ERR10567600	ERR10567626	ERR10567413	ERR10567430	ERR10567587	ERR10567613	ERR10565813	ERR10565867
ERR10565894	ERR10565921	ERR10565975	ERR10566002	ERR10565840	ERR10565948	ERR10569612	ERR10569420
ERR10569629	ERR10569639	ERR10569748	ERR10569940	ERR10570036	ERR10570073	ERR10569324	ERR10569516
ERR10569649	ERR10569659	ERR10569844	ERR10570053	ERR10570063	ERR10570083	ERR10567702	ERR10567739
ERR10567850	ERR10567665	ERR10567776	ERR10567813	ERR10567887	ERR10567924	ERR10565515	ERR10565541
ERR10565567	ERR10565579	ERR10565591	ERR10565603	ERR10565528	ERR10565554	ERR10569613	ERR10569749
ERR10570037	ERR10569325	ERR10569421	ERR10569517	ERR10569845	ERR10569941	ERR10567397	ERR10567414
ERR10567601	ERR10567380	ERR10567431	ERR10567588	ERR10567614	ERR10567627	ERR10565324	ERR10565370
ERR10565393	ERR10565347	ERR10569518	ERR10569630	ERR10569640	ERR10569650	ERR10569750	ERR10569846
ERR10569942	ERR10570038	ERR10570074	ERR10570084	ERR10569326	ERR10569422	ERR10569614	ERR10569660
ERR10570054	ERR10570064	ERR10567142	ERR10567174	ERR10567298	ERR10567206	ERR10567238	ERR10567268
ERR10567328	ERR10567358	ERR10565325	ERR10565348	ERR10565394	ERR10565371	ERR10569327	ERR10569751
ERR10569847	ERR10569943	ERR10569423	ERR10569519	ERR10569615	ERR10570039	ERR10567381	ERR10567415
ERR10567589	ERR10567602	ERR10567615	ERR10567628	ERR10567398	ERR10567432	ERR10565568	ERR10565580
ERR10565592	ERR10565604	ERR10565334	ERR10565357	ERR10565380	ERR10565403	ERR10569424	ERR10569752
ERR10569848	ERR10569944	ERR10570040	ERR10569328	ERR10569520	ERR10569616	ERR10567207	ERR10567269
ERR10567299	ERR10567359	ERR10567143	ERR10567175	ERR10567239	ERR10567329	ERR10565349	ERR10565372
ERR10565326	ERR10565395	ERR10569753	ERR10569849	ERR10570041	ERR10569329	ERR10569425	ERR10569521
ERR10569617	ERR10569945	ERR10567144	ERR10567176	ERR10567208	ERR10567300	ERR10567330	ERR10567240
ERR10567270	ERR10567360	ERR10565814	ERR10565841	ERR10565895	ERR10565922	ERR10565949	ERR10565976
ERR10566003	ERR10565868	ERR10569330	ERR10569426	ERR10569631	ERR10569641	ERR10569651	ERR10569661
ERR10570042	ERR10570055	ERR10570065	ERR10570085	ERR10569522	ERR10569618	ERR10569754	ERR10569850
ERR10569946	ERR10570075	ERR10567399	ERR10567590	ERR10567603	ERR10567616	ERR10567382	ERR10567416
ERR10567433	ERR10567629	ERR10565373	ERR10565396	ERR10565542	ERR10565327	ERR10565350	ERR10565516
ERR10565529	ERR10565555	ERR10569619	ERR10569851	ERR10570043	ERR10569331	ERR10569427	ERR10569523
ERR10569755	ERR10569947	ERR10567666	ERR10567740	ERR10567777	ERR10567851	ERR10567703	ERR10567814
ERR10567888	ERR10567925	ERR10565815	ERR10565842	ERR10565923	ERR10565950	ERR10566004	ERR10565869
ERR10565896	ERR10565977	ERR10569332	ERR10569428	ERR10569524	ERR10569756	ERR10569852	ERR10569620
ERR10569948	ERR10570044	ERR10567177	ERR10567271	ERR10567301	ERR10567361	ERR10567145	ERR10567209
ERR10567241	ERR10567331	ERR10565351	ERR10565374	ERR10565397	ERR10565517	ERR10565543	ERR10565328
ERR10565530	ERR10565556	ERR10569429	ERR10569525	ERR10569621	ERR10569949	ERR10570045	ERR10569333
ERR10569757	ERR10569853	ERR10567210	ERR10567242	ERR10567272	ERR10567362	ERR10567146	ERR10567178
ERR10567302	ERR10567332	ERR10565375	ERR10565398	ERR10565329	ERR10565352	ERR10570098	ERR10570090
ERR10570094	ERR10570086	ERR10567179	ERR10567243	ERR10567303	ERR10567147	ERR10567211	ERR10567273
ERR10567333	ERR10567363	ERR10565330	ERR10565376	ERR10565353	ERR10565399	ERR10570091	ERR10570087
ERR10570095	ERR10570099	ERR10567148	ERR10567334	ERR10567180	ERR10567212	ERR10567244	ERR10567274
ERR10567304	ERR10567364	ERR10565331	ERR10565354	ERR10565377	ERR10565400	ERR10570088	ERR10570096
ERR10570100	ERR10570092	ERR10567181	ERR10567305	ERR10567335	ERR10567365	ERR10567149	ERR10567213
ERR10567245	ERR10567275	ERR10565358	ERR10565454	ERR10565335	ERR10565381	ERR10565404	ERR10565429

ERR10565479	ERR10565504	ERR10570093	ERR10570097	ERR10570089	ERR10570101	ERR10568514	ERR10568576
ERR10568638	ERR10568762	ERR10568802	ERR10568700	ERR10568842	ERR10568882	ERR10566606	ERR10566660
ERR10566633	ERR10566687	ERR10571327	ERR10571329	ERR10571390	ERR10571486	ERR10571278	ERR10571328
ERR10571342	ERR10571438	ERR10571290	ERR10571326	ERR10571302	ERR10571314	ERR10568843	ERR10568515
ERR10568577	ERR10568639	ERR10568701	ERR10568763	ERR10568803	ERR10568883	ERR10566688	ERR10566607
ERR10566634	ERR10566661	ERR10571343	ERR10571391	ERR10571291	ERR10571303	ERR10571439	ERR10571487
ERR10571279	ERR10571315	ERR10568516	ERR10568640	ERR10568844	ERR10568578	ERR10568702	ERR10568764
ERR10568804	ERR10568884	ERR10566608	ERR10566689	ERR10566635	ERR10566662	ERR10571344	ERR10571392
ERR10571488	ERR10571304	ERR10571440	ERR10571280	ERR10571292	ERR10571316	ERR10569006	ERR10569046
ERR10569066	ERR10569106	ERR10569126	ERR10569146	ERR10569026	ERR10569086	ERR10566609	ERR10566636
ERR10566663	ERR10566690	ERR10571345	ERR10571441	ERR10571489	ERR10571281	ERR10571393	ERR10571293
ERR10571305	ERR10571317	ERR10568970	ERR10568988	ERR10569166	ERR10569184	ERR10569220	ERR10568934
ERR10568952	ERR10569202	ERR10566691	ERR10566610	ERR10566637	ERR10566664	ERR10571346	ERR10571394
ERR10571442	ERR10571282	ERR10571294	ERR10571306	ERR10571490	ERR10571318	ERR10568935	ERR10568953
ERR10568989	ERR10569167	ERR10569185	ERR10569221	ERR10568971	ERR10569203	ERR10566611	ERR10566665
ERR10566638	ERR10566692	ERR10571319	ERR10571347	ERR10571395	ERR10571491	ERR10571283	ERR10571307
ERR10571443	ERR10571295	ERR10568936	ERR10568954	ERR10568990	ERR10569168	ERR10569186	ERR10569222
ERR10568972	ERR10569204	ERR10566612	ERR10566639	ERR10566666	ERR10566693	ERR10571492	ERR10571296
ERR10571396	ERR10571284	ERR10571320	ERR10571348	ERR10571444	ERR10571308	ERR10568517	ERR10568641
ERR10568765	ERR10568579	ERR10568703	ERR10568805	ERR10568845	ERR10568885	ERR10566640	ERR10566667
ERR10566694	ERR10566613	ERR10571321	ERR10571349	ERR10571397	ERR10571445	ERR10571297	ERR10571493
ERR10571285	ERR10571309	ERR10568949	ERR10568985	ERR10569217	ERR10569235	ERR10568967	ERR10569003
ERR10569181	ERR10569199	ERR10566614	ERR10566641	ERR10566668	ERR10566695	ERR10571350	ERR10571398
ERR10571446	ERR10571322	ERR10571286	ERR10571298	ERR10571310	ERR10571494	ERR10568973	ERR10569169
ERR10569187	ERR10569223	ERR10568937	ERR10568955	ERR10568991	ERR10569205	ERR10566615	ERR10566642
ERR10566669	ERR10566696	ERR10571323	ERR10571351	ERR10571399	ERR10571447	ERR10571495	ERR10571287
ERR10571299	ERR10571311	ERR10569007	ERR10569067	ERR10569107	ERR10569127	ERR10569147	ERR10569027
ERR10569047	ERR10569087	ERR10566670	ERR10566616	ERR10566643	ERR10566697	ERR10571324	ERR10571352
ERR10571400	ERR10571448	ERR10571496	ERR10571288	ERR10571312	ERR10571300	ERR10569028	ERR10569048
ERR10569068	ERR10569088	ERR10569148	ERR10569008	ERR10569108	ERR10569128	ERR10566617	ERR10566698
ERR10566644	ERR10566671	ERR10571353	ERR10571401	ERR10571301	ERR10571313	ERR10571289	ERR10571325
ERR10571449	ERR10571497	ERR10568766	ERR10568806	ERR10568846	ERR10568886	ERR10568518	ERR10568580
ERR10568642	ERR10568704	ERR10566854	ERR10566901	ERR10566948	ERR10566995	ERR10571354	ERR10571498
ERR10571026	ERR10571110	ERR10570942	ERR10571194	ERR10571402	ERR10571450	ERR10568643	ERR10568705
ERR10568807	ERR10568847	ERR10568887	ERR10568519	ERR10568581	ERR10568767	ERR10566996	ERR10566855
ERR10566902	ERR10566949	ERR10571403	ERR10571451	ERR10571499	ERR10571027	ERR10571195	ERR10571355
ERR10571111	ERR10570943	ERR10568520	ERR10568644	ERR10568768	ERR10568808	ERR10568582	ERR10568706
ERR10568848	ERR10568888	ERR10566903	ERR10566950	ERR10566997	ERR10566856	ERR10571356	ERR10571452
ERR10571500	ERR10570944	ERR10571028	ERR10571112	ERR10571196	ERR10571404	ERR10568938	ERR10568992
ERR10569170	ERR10569188	ERR10569224	ERR10568956	ERR10568974	ERR10569206	ERR10566904	ERR10566998
ERR10566857	ERR10566951	ERR10571405	ERR10571453	ERR10570945	ERR10571113	ERR10571357	ERR10571501
ERR10571029	ERR10571197	ERR10568521	ERR10568583	ERR10568707	ERR10568769	ERR10568809	ERR10568849
ERR10568645	ERR10568889	ERR10566999	ERR10566858	ERR10566905	ERR10566952	ERR10571406	ERR10571454
ERR10571502	ERR10570946	ERR10571030	ERR10571358	ERR10571114	ERR10571198	ERR10568522	ERR10568646
ERR10568770	ERR10568810	ERR10568890	ERR10568584	ERR10568708	ERR10568850	ERR10566859	ERR10566906
ERR10566953	ERR10567000	ERR10571359	ERR10571407	ERR10571455	ERR10571503	ERR10571199	ERR10570947
ERR10571031	ERR10571115	ERR10568993	ERR10569171	ERR10568939	ERR10568957	ERR10568975	ERR10569189
ERR10569207	ERR10569225	ERR10566860	ERR10567001	ERR10566907	ERR10566954	ERR10571408	ERR10571456
ERR10571504	ERR10571032	ERR10571200	ERR10571360	ERR10571116	ERR10570948	ERR10568523	ERR10568585
ERR10568647	ERR10568709	ERR10568811	ERR10568771	ERR10568851	ERR10568891	ERR10566861	ERR10566908
ERR10566955	ERR10567002	ERR10571361	ERR10571505	ERR10571201	ERR10571409	ERR10570949	ERR10571457
ERR10571033	ERR10571117	ERR10568586	ERR10568710	ERR10568524	ERR10568648	ERR10568772	ERR10568812
ERR10568852	ERR10568892	ERR10566862	ERR10566909	ERR10566956	ERR10567003	ERR10571362	ERR10571410
ERR10571118	ERR10571458	ERR10571506	ERR10570950	ERR10571034	ERR10571202	ERR10568649	ERR10568525
ERR10568587	ERR10568711	ERR10568773	ERR10568813	ERR10568853	ERR10568893	ERR10566863	ERR10566910
ERR10566957	ERR10567004	ERR10571507	ERR10570951	ERR10571203	ERR10571363	ERR10571459	ERR10571035
ERR10571411	ERR10571119	ERR10568588	ERR10568650	ERR10568774	ERR10568854	ERR10568526	ERR10568712
ERR10568814	ERR10568894	ERR10566958	ERR10566864	ERR10566911	ERR10567005	ERR10571364	ERR10571412

ERR10571204	ERR10571508	ERR10571460	ERR10570952	ERR10571036	ERR10571120	ERR10568527	ERR10568589
ERR10568775	ERR10568815	ERR10568895	ERR10568651	ERR10568713	ERR10568855	ERR10566912	ERR10566959
ERR10567006	ERR10566865	ERR10571365	ERR10571461	ERR10571509	ERR10571121	ERR10571413	ERR10570953
ERR10571037	ERR10571205	ERR10568958	ERR10568976	ERR10568994	ERR10569226	ERR10568940	ERR10569172
ERR10569190	ERR10569208	ERR10566618	ERR10566645	ERR10566699	ERR10567042	ERR10567066	ERR10566672
ERR10567054	ERR10567078	ERR10571366	ERR10571414	ERR10571510	ERR10570954	ERR10571206	ERR10571462
ERR10571038	ERR10571122	ERR10568528	ERR10568590	ERR10568714	ERR10568776	ERR10568856	ERR10568652
ERR10568816	ERR10568896	ERR10566619	ERR10567043	ERR10566646	ERR10566673	ERR10566700	ERR10567055
ERR10567067	ERR10567079	ERR10571367	ERR10571463	ERR10571511	ERR10571123	ERR10571415	ERR10571207
ERR10570955	ERR10571039	ERR10569009	ERR10569029	ERR10569109	ERR10569049	ERR10569069	ERR10569089
ERR10569129	ERR10569149	ERR10566620	ERR10566674	ERR10567044	ERR10567068	ERR10567080	ERR10566647
ERR10566701	ERR10567056	ERR10571416	ERR10571208	ERR10571040	ERR10571368	ERR10571464	ERR10571512
ERR10570956	ERR10571124	ERR10568529	ERR10568897	ERR10568591	ERR10568653	ERR10568715	ERR10568777
ERR10568817	ERR10568857	ERR10566621	ERR10566675	ERR10566702	ERR10567045	ERR10567069	ERR10566648
ERR10567057	ERR10567081	ERR10571417	ERR10571513	ERR10571041	ERR10571209	ERR10571369	ERR10571125
ERR10571465	ERR10570957	ERR10568530	ERR10568716	ERR10568592	ERR10568654	ERR10568778	ERR10568818
ERR10568858	ERR10568898	ERR10566622	ERR10567070	ERR10566649	ERR10566676	ERR10566703	ERR10567046
ERR10567058	ERR10567082	ERR10571514	ERR10571042	ERR10571370	ERR10571418	ERR10571210	ERR10571466
ERR10570958	ERR10571126	ERR10568593	ERR10568859	ERR10568531	ERR10568655	ERR10568717	ERR10568779
ERR10568819	ERR10568899	ERR10566623	ERR10566650	ERR10567059	ERR10567071	ERR10567083	ERR10566677
ERR10566704	ERR10567047	ERR10571419	ERR10571467	ERR10571515	ERR10570959	ERR10571371	ERR10571043
ERR10571127	ERR10571211	ERR10568594	ERR10568656	ERR10568780	ERR10568532	ERR10568718	ERR10568820
ERR10568860	ERR10568900	ERR10566624	ERR10566651	ERR10567048	ERR10567060	ERR10567084	ERR10566678
ERR10566705	ERR10567072	ERR10571420	ERR10571044	ERR10571468	ERR10571212	ERR10571372	ERR10571516
ERR10570960	ERR10571128	ERR10569070	ERR10569130	ERR10569150	ERR10569010	ERR10569030	ERR10569050
ERR10569090	ERR10569110	ERR10567049	ERR10567085	ERR10566625	ERR10566652	ERR10566679	ERR10566706
ERR10567061	ERR10567073	ERR10571517	ERR10571469	ERR10571045	ERR10571129	ERR10571373	ERR10571421
ERR10570961	ERR10571213	ERR10569051	ERR10569091	ERR10569111	ERR10569131	ERR10569011	ERR10569031
ERR10569071	ERR10569151	ERR10566626	ERR10566707	ERR10567050	ERR10567062	ERR10567074	ERR10566653
ERR10566680	ERR10567086	ERR10571374	ERR10571470	ERR10571518	ERR10571422	ERR10571046	ERR10570962
ERR10571130	ERR10571214	ERR10568533	ERR10568595	ERR10568821	ERR10568861	ERR10568657	ERR10568719
ERR10568781	ERR10568901	ERR10566627	ERR10566654	ERR10567063	ERR10566681	ERR10566708	ERR10567051
ERR10567075	ERR10567087	ERR10571375	ERR10571423	ERR10571519	ERR10571471	ERR10571215	ERR10570963
ERR10571047	ERR10571131	ERR10569012	ERR10569032	ERR10569052	ERR10569072	ERR10569092	ERR10569112
ERR10569132	ERR10569152	ERR10566655	ERR10566709	ERR10567064	ERR10567088	ERR10566628	ERR10566682
ERR10567052	ERR10567076	ERR10571424	ERR10571472	ERR10570964	ERR10571048	ERR10571376	ERR10571216
ERR10571520	ERR10571132	ERR10568995	ERR10569173	ERR10569191	ERR10569209	ERR10569227	ERR10568941
ERR10568959	ERR10568977	ERR10566629	ERR10566683	ERR10566710	ERR10567053	ERR10567077	ERR10567089
ERR10566656	ERR10567065	ERR10571377	ERR10571217	ERR10571425	ERR10571473	ERR10570965	ERR10571521
ERR10571049	ERR10571133	ERR10568960	ERR10569174	ERR10569192	ERR10569210	ERR10568942	ERR10568978
ERR10568996	ERR10569228	ERR10566714	ERR10566749	ERR10566960	ERR10567007	ERR10566784	ERR10566819
ERR10566866	ERR10566913	ERR10571378	ERR10571522	ERR10571050	ERR10571474	ERR10571426	ERR10570966
ERR10571134	ERR10571218	ERR10568534	ERR10568862	ERR10568596	ERR10568658	ERR10568720	ERR10568782
ERR10568822	ERR10568902	ERR10566914	ERR10567008	ERR10566715	ERR10566750	ERR10566785	ERR10566820
ERR10566867	ERR10566961	ERR10571475	ERR10571523	ERR10571379	ERR10570967	ERR10571427	ERR10571051
ERR10571135	ERR10571219	ERR10568535	ERR10568659	ERR10568721	ERR10568823	ERR10568597	ERR10568783
ERR10568863	ERR10568903	ERR10566716	ERR10566786	ERR10566915	ERR10566962	ERR10567009	ERR10566751
ERR10566821	ERR10566868	ERR10571380	ERR10571428	ERR10571524	ERR10570968	ERR10571052	ERR10571136
ERR10571476	ERR10571220	ERR10568598	ERR10568784	ERR10568904	ERR10568536	ERR10568660	ERR10568722
ERR10568824	ERR10568864	ERR10566717	ERR10566822	ERR10566963	ERR10567010	ERR10566752	ERR10566787
ERR10566869	ERR10566916	ERR10571381	ERR10571525	ERR10570969	ERR10571477	ERR10571429	ERR10571053
ERR10571137	ERR10571221	ERR10569073	ERR10569093	ERR10569133	ERR10569013	ERR10569033	ERR10569053
ERR10569113	ERR10569153	ERR10566870	ERR10566718	ERR10566753	ERR10566788	ERR10566823	ERR10566917
ERR10566964	ERR10567011	ERR10571382	ERR10571526	ERR10571054	ERR10571430	ERR10571138	ERR10571222
ERR10571478	ERR10570970	ERR10568979	ERR10569175	ERR10569211	ERR10569229	ERR10568943	ERR10568961
ERR10568997	ERR10569193	ERR10566754	ERR10566789	ERR10566965	ERR10566719	ERR10566824	ERR10566871
ERR10566918	ERR10567012	ERR10571527	ERR10571055	ERR10571139	ERR10571223	ERR10571383	ERR10571431
ERR10571479	ERR10570971	ERR10568537	ERR10568599	ERR10568661	ERR10568723	ERR10568785	ERR10568865

ERR10568905	ERR10568825	ERR10567013	ERR10566720	ERR10566755	ERR10566790	ERR10566825	ERR10566872
ERR10566919	ERR10566966	ERR10571384	ERR10571528	ERR10570972	ERR10571140	ERR10571432	ERR10571480
ERR10571056	ERR10571224	ERR10568600	ERR10568662	ERR10568724	ERR10568786	ERR10568866	ERR10568538
ERR10568826	ERR10568906	ERR10566873	ERR10566920	ERR10566967	ERR10567014	ERR10566721	ERR10566756
ERR10566791	ERR10566826	ERR10571385	ERR10571433	ERR10571141	ERR10571481	ERR10571529	ERR10570973
ERR10571057	ERR10571225	ERR10569014	ERR10569034	ERR10569074	ERR10569094	ERR10569114	ERR10569134
ERR10569154	ERR10569054	ERR10566722	ERR10566757	ERR10566792	ERR10566921	ERR10566968	ERR10566827
ERR10566874	ERR10567015	ERR10571386	ERR10571530	ERR10570974	ERR10571058	ERR10571434	ERR10571482
ERR10571142	ERR10571226	ERR10568601	ERR10568725	ERR10568827	ERR10568867	ERR10568907	ERR10568539
ERR10568663	ERR10568787	ERR10566723	ERR10566758	ERR10566793	ERR10566875	ERR10566828	ERR10566922
ERR10566969	ERR10567016	ERR10571387	ERR10571435	ERR10570975	ERR10571143	ERR10571483	ERR10571531
ERR10571059	ERR10571227	ERR10568602	ERR10568664	ERR10568726	ERR10568828	ERR10568868	ERR10568540
ERR10568788	ERR10568908	ERR10566759	ERR10566876	ERR10566724	ERR10566794	ERR10566829	ERR10566923
ERR10566970	ERR10567017	ERR10571532	ERR10571144	ERR10571228	ERR10571388	ERR10571436	ERR10571484
ERR10570976	ERR10571060	ERR10568968	ERR10568986	ERR10569182	ERR10569218	ERR10568950	ERR10569004
ERR10569200	ERR10569236	ERR10567096	ERR10567100	ERR10567102	ERR10567106	ERR10567094	ERR10567098
ERR10567104	ERR10567108	ERR10571389	ERR10571485	ERR10571533	ERR10571437	ERR10570977	ERR10571061
ERR10571145	ERR10571229	ERR10568869	ERR10568541	ERR10568603	ERR10568665	ERR10568727	ERR10568789
ERR10568829	ERR10568909	ERR10566725	ERR10566760	ERR10566877	ERR10566795	ERR10566830	ERR10566924
ERR10566971	ERR10567018	ERR10571146	ERR10570978	ERR10571062	ERR10571230	ERR10568542	ERR10568666
ERR10568728	ERR10568830	ERR10568870	ERR10568910	ERR10568604	ERR10568790	ERR10566726	ERR10566972
ERR10566761	ERR10566796	ERR10566831	ERR10566878	ERR10566925	ERR10567019	ERR10571147	ERR10571063
ERR10570979	ERR10571231	ERR10568667	ERR10568729	ERR10568791	ERR10568543	ERR10568605	ERR10568831
ERR10568871	ERR10568911	ERR10566727	ERR10566926	ERR10567020	ERR10566762	ERR10566797	ERR10566832
ERR10566879	ERR10566973	ERR10570980	ERR10571064	ERR10571148	ERR10571232	ERR10568944	ERR10568962
ERR10568980	ERR10569194	ERR10569212	ERR10569230	ERR10568998	ERR10569176	ERR10566763	ERR10566798
ERR10566833	ERR10566927	ERR10566728	ERR10566880	ERR10566974	ERR10567021	ERR10571149	ERR10571233
ERR10571065	ERR10570981	ERR10568606	ERR10568730	ERR10568832	ERR10568912	ERR10568544	ERR10568668
ERR10568792	ERR10568872	ERR10566729	ERR10566764	ERR10566799	ERR10566834	ERR10566928	ERR10566975
ERR10566881	ERR10567022	ERR10570982	ERR10571066	ERR10571150	ERR10571234	ERR10568545	ERR10568669
ERR10568833	ERR10568873	ERR10568913	ERR10568607	ERR10568731	ERR10568793	ERR10566730	ERR10566765
ERR10566835	ERR10566800	ERR10566882	ERR10566929	ERR10566976	ERR10567023	ERR10570983	ERR10571151
ERR10571067	ERR10571235	ERR10569015	ERR10569055	ERR10569135	ERR10569035	ERR10569075	ERR10569095
ERR10569115	ERR10569155	ERR10566766	ERR10566836	ERR10566930	ERR10566977	ERR10567024	ERR10566731
ERR10566801	ERR10566883	ERR10571068	ERR10571236	ERR10570984	ERR10571152	ERR10568969	ERR10569183
ERR10569201	ERR10568951	ERR10568987	ERR10569005	ERR10569219	ERR10569237	ERR10566767	ERR10566837
ERR10567025	ERR10566732	ERR10566802	ERR10566884	ERR10566931	ERR10566978	ERR10570985	ERR10571237
ERR10571153	ERR10571069	ERR10569016	ERR10569036	ERR10569056	ERR10569116	ERR10569156	ERR10569076
ERR10569096	ERR10569136	ERR10566733	ERR10566838	ERR10566885	ERR10567026	ERR10566768	ERR10566803
ERR10566932	ERR10566979	ERR10571238	ERR10571070	ERR10571154	ERR10570986	ERR10568608	ERR10568670
ERR10568732	ERR10568834	ERR10568914	ERR10568546	ERR10568794	ERR10568874	ERR10566769	ERR10566804
ERR10566839	ERR10566886	ERR10566933	ERR10566980	ERR10566734	ERR10567027	ERR10571155	ERR10571239
ERR10570987	ERR10571071	ERR10569017	ERR10569117	ERR10569137	ERR10569037	ERR10569057	ERR10569077
ERR10569097	ERR10569157	ERR10566770	ERR10566887	ERR10566934	ERR10566735	ERR10566805	ERR10566840
ERR10566981	ERR10567028	ERR10570988	ERR10571240	ERR10571072	ERR10571156	ERR10568671	ERR10568835
ERR10568875	ERR10568915	ERR10568547	ERR10568609	ERR10568733	ERR10568795	ERR10566736	ERR10566771
ERR10566806	ERR10566888	ERR10566982	ERR10566841	ERR10566935	ERR10567029	ERR10570989	ERR10571157
ERR10571241	ERR10571073	ERR10569038	ERR10569098	ERR10569138	ERR10569018	ERR10569058	ERR10569078
ERR10569118	ERR10569158	ERR10566936	ERR10566983	ERR10567030	ERR10566737	ERR10566772	ERR10566807
ERR10566842	ERR10566889	ERR10571074	ERR10570990	ERR10571158	ERR10571242	ERR10568548	ERR10568734
ERR10568836	ERR10568610	ERR10568672	ERR10568796	ERR10568876	ERR10568916	ERR10567092	ERR10567093
ERR10567090	ERR10567091	ERR10570991	ERR10571159	ERR10571243	ERR10571075	ERR10569019	ERR10569039
ERR10569079	ERR10569139	ERR10569159	ERR10569059	ERR10569099	ERR10569119	ERR10566890	ERR10567031
ERR10566738	ERR10566773	ERR10566808	ERR10566843	ERR10566937	ERR10566984	ERR10571076	ERR10571160
ERR10570992	ERR10571244	ERR10568549	ERR10568611	ERR10568735	ERR10568797	ERR10568877	ERR10568673
ERR10568837	ERR10568917	ERR10566739	ERR10566774	ERR10566844	ERR10566938	ERR10566985	ERR10567032
ERR10566809	ERR10566891	ERR10570993	ERR10571077	ERR10571161	ERR10571245	ERR10568448	ERR10568470
ERR10568612	ERR10568674	ERR10568736	ERR10568922	ERR10568924	ERR10568426	ERR10568492	ERR10568550

ERR10568926	ERR10568928	ERR10566740	ERR10566810	ERR10566845	ERR10567033	ERR10566775	ERR10566892
ERR10566939	ERR10566986	ERR10571246	ERR10570994	ERR10571078	ERR10571162	ERR10569020	ERR10569040
ERR10569060	ERR10569080	ERR10569100	ERR10569140	ERR10569120	ERR10569160	ERR10566776	ERR10566893
ERR10566940	ERR10566741	ERR10566811	ERR10566846	ERR10566987	ERR10567034	ERR10571163	ERR10571247
ERR10570995	ERR10571079	ERR10568449	ERR10568493	ERR10568613	ERR10568737	ERR10568427	ERR10568471
ERR10568551	ERR10568675	ERR10566742	ERR10566847	ERR10566894	ERR10566988	ERR10566777	ERR10566812
ERR10566941	ERR10567035	ERR10571164	ERR10570996	ERR10571080	ERR10571248	ERR10569021	ERR10569081
ERR10569101	ERR10569121	ERR10569041	ERR10569061	ERR10569141	ERR10569161	ERR10566743	ERR10566895
ERR10567036	ERR10566778	ERR10566813	ERR10566848	ERR10566942	ERR10566989	ERR10571081	ERR10571165
ERR10570997	ERR10571249	ERR10568428	ERR10568472	ERR10568552	ERR10568676	ERR10568738	ERR10568450
ERR10568614	ERR10566744	ERR10566779	ERR10566896	ERR10566990	ERR10566814	ERR10566849	ERR10566943
ERR10567037	ERR10570998	ERR10571082	ERR10571166	ERR10571250	ERR10569022	ERR10569042	ERR10569102
ERR10569122	ERR10569142	ERR10569062	ERR10569082	ERR10569162	ERR10566780	ERR10566850	ERR10566897
ERR10566944	ERR10567038	ERR10566745	ERR10566815	ERR10566991	ERR10571167	ERR10570999	ERR10571083
ERR10571251	ERR10568451	ERR10568615	ERR10568677	ERR10568739	ERR10568429	ERR10568473	ERR10568495
ERR10568553	ERR10566781	ERR10566816	ERR10566851	ERR10567039	ERR10566746	ERR10566898	ERR10566945
ERR10566992	ERR10571000	ERR10571168	ERR10571084	ERR10571252	ERR10568430	ERR10568452	ERR10568496
ERR10568616	ERR10568678	ERR10568474	ERR10568554	ERR10568740	ERR10566747	ERR10566852	ERR10566899
ERR10566946	ERR10566782	ERR10566817	ERR10566993	ERR10567040	ERR10571169	ERR10571253	ERR10571001
ERR10571085	ERR10568453	ERR10568475	ERR10568497	ERR10568555	ERR10568431	ERR10568617	ERR10568679
ERR10568741	ERR10566748	ERR10566818	ERR10566947	ERR10567041	ERR10566783	ERR10566853	ERR10566900
ERR10566994	ERR10571086	ERR10571254	ERR10571002	ERR10571170	ERR10568476	ERR10568498	ERR10568618
ERR10568742	ERR10568432	ERR10568454	ERR10568556	ERR10568680	ERR10566514	ERR10566560	ERR10566537
ERR10566583	ERR10571087	ERR10571255	ERR10571171	ERR10571003	ERR10568945	ERR10568981	ERR10568999
ERR10568963	ERR10569177	ERR10569195	ERR10569213	ERR10569231	ERR10566515	ERR10566538	ERR10566561
ERR10566584	ERR10571088	ERR10571172	ERR10571256	ERR10571004	ERR10568433	ERR10568455	ERR10568499
ERR10568557	ERR10568619	ERR10568743	ERR10568477	ERR10568681	ERR10566516	ERR10566539	ERR10566562
ERR10566585	ERR10571005	ERR10571173	ERR10571089	ERR10571257	ERR10569043	ERR10569123	ERR10569143
ERR10569023	ERR10569063	ERR10569083	ERR10569103	ERR10569163	ERR10566540	ERR10566586	ERR10566517
ERR10566563	ERR10571006	ERR10571174	ERR10571258	ERR10571090	ERR10568946	ERR10568982	ERR10569000
ERR10569178	ERR10569196	ERR10569232	ERR10568964	ERR10569214	ERR10566518	ERR10566541	ERR10566564
ERR10566587	ERR10571259	ERR10571007	ERR10571091	ERR10571175	ERR10569024	ERR10569064	ERR10569104
ERR10569124	ERR10569044	ERR10569084	ERR10569144	ERR10569164	ERR10566542	ERR10566565	ERR10566519
ERR10566588	ERR10571008	ERR10571092	ERR10571176	ERR10571260	ERR10568456	ERR10568478	ERR10568620
ERR10568682	ERR10568434	ERR10568500	ERR10568558	ERR10568744	ERR10566520	ERR10566543	ERR10566589
ERR10566566	ERR10571093	ERR10571009	ERR10571177	ERR10571261	ERR10569001	ERR10569179	ERR10568947
ERR10568965	ERR10568983	ERR10569197	ERR10569215	ERR10569233	ERR10566567	ERR10566521	ERR10566544
ERR10566590	ERR10571262	ERR10571010	ERR10571094	ERR10571178	ERR10569065	ERR10569085	ERR10569165
ERR10569025	ERR10569045	ERR10569105	ERR10569125	ERR10569145	ERR10566568	ERR10566522	ERR10566545
ERR10566591	ERR10571179	ERR10571011	ERR10571095	ERR10571263	ERR10568435	ERR10568479	ERR10568501
ERR10568745	ERR10568457	ERR10568559	ERR10568621	ERR10568683	ERR10566523	ERR10566592	ERR10566546
ERR10566569	ERR10571012	ERR10571180	ERR10571096	ERR10571264	ERR10568436	ERR10568480	ERR10568502
ERR10568458	ERR10568560	ERR10568622	ERR10568684	ERR10568746	ERR10566593	ERR10566524	ERR10566547
ERR10566570	ERR10571013	ERR10571097	ERR10571265	ERR10571181	ERR10568503	ERR10568561	ERR10568623
ERR10568747	ERR10568437	ERR10568459	ERR10568481	ERR10568685	ERR10566571	ERR10566594	ERR10566525
ERR10566548	ERR10571014	ERR10571098	ERR10571182	ERR10571266	ERR10568948	ERR10568966	ERR10568984
ERR10569180	ERR10569234	ERR10569002	ERR10569198	ERR10569216	ERR10566549	ERR10566595	ERR10566526
ERR10566572	ERR10571099	ERR10571183	ERR10571267	ERR10571015	ERR10568438	ERR10568460	ERR10568482
ERR10568504	ERR10568562	ERR10568624	ERR10568686	ERR10568748	ERR10566527	ERR10566596	ERR10566550
ERR10566573	ERR10571184	ERR10571268	ERR10571016	ERR10571100	ERR10568483	ERR10568563	ERR10568749
ERR10568439	ERR10568461	ERR10568505	ERR10568625	ERR10568687	ERR10566528	ERR10566551	ERR10566574
ERR10566597	ERR10571185	ERR10571017	ERR10571101	ERR10571269	ERR10568506	ERR10568440	ERR10568462
ERR10568484	ERR10568564	ERR10568626	ERR10568688	ERR10568750	ERR10566598	ERR10566529	ERR10566552
ERR10566575	ERR10571186	ERR10571018	ERR10571102	ERR10571270	ERR10568441	ERR10568485	ERR10568507
ERR10568627	ERR10568751	ERR10568463	ERR10568565	ERR10568689	ERR10566553	ERR10566599	ERR10566530
ERR10566576	ERR10571019	ERR10571103	ERR10571187	ERR10571271	ERR10568464	ERR10568508	ERR10568566
ERR10568925	ERR10568442	ERR10568486	ERR10568628	ERR10568690	ERR10568752	ERR10568923	ERR10568927
ERR10568929	ERR10566554	ERR10566577	ERR10566600	ERR10566531	ERR10571020	ERR10571104	ERR10571272

ERR10571188	ERR10568567	ERR10568629	ERR10568691	ERR10568753	ERR10568443	ERR10568465	ERR10568487
ERR10568509	ERR10566532	ERR10566555	ERR10566578	ERR10566601	ERR10571189	ERR10571021	ERR10571105
ERR10571273	ERR10568488	ERR10568510	ERR10568630	ERR10568692	ERR10568444	ERR10568466	ERR10568568
ERR10568754	ERR10566533	ERR10566602	ERR10566556	ERR10566579	ERR10571190	ERR10571274	ERR10571106
ERR10571022	ERR10568445	ERR10568489	ERR10568511	ERR10568569	ERR10568693	ERR10568467	ERR10568631
ERR10568755	ERR10566557	ERR10566580	ERR10566603	ERR10566534	ERR10571023	ERR10571191	ERR10571275
ERR10571107	ERR10568512	ERR10568632	ERR10568694	ERR10568756	ERR10568446	ERR10568468	ERR10568490
ERR10568570	ERR10566535	ERR10566581	ERR10566604	ERR10566558	ERR10571192	ERR10571024	ERR10571276
ERR10571108	ERR10568513	ERR10568930	ERR10568931	ERR10568447	ERR10568469	ERR10568491	ERR10568571
ERR10568633	ERR10568695	ERR10568757	ERR10568932	ERR10568933	ERR10566559	ERR10566582	ERR10566605
ERR10566536	ERR10571277	ERR10571109	ERR10571193	ERR10571025	ERR10568572	ERR10568696	ERR10568758
ERR10568798	ERR10568918	ERR10568634	ERR10568838	ERR10568878	ERR10567095	ERR10567099	ERR10567101
ERR10567105	ERR10567109	ERR10567097	ERR10567103	ERR10567107	ERR10571339	ERR10571330	ERR10571333
ERR10571336	ERR10568573	ERR10568697	ERR10568759	ERR10568799	ERR10568879	ERR10568919	ERR10568635
ERR10568839	ERR10566630	ERR10566657	ERR10566684	ERR10566711	ERR10571331	ERR10571334	ERR10571337
ERR10571340	ERR10568800	ERR10568840	ERR10568574	ERR10568636	ERR10568698	ERR10568760	ERR10568880
ERR10568920	ERR10566631	ERR10566658	ERR10566685	ERR10566712	ERR10571534	ERR10571537	ERR10571535
ERR10571536	ERR10568637	ERR10568699	ERR10568801	ERR10568841	ERR10568921	ERR10568575	ERR10568761
ERR10568881	ERR10566686	ERR10566632	ERR10566659	ERR10566713	ERR10571332	ERR10571335	ERR10571341
ERR10571338	ERR10567951	ERR10568001	ERR10568042	ERR10568138	ERR10568234	ERR10567926	ERR10567976
ERR10568330	ERR10566109	ERR10566167	ERR10566225	ERR10566283	ERR10570294	ERR10570198	ERR10570390
ERR10570558	ERR10570654	ERR10570750	ERR10570846	ERR10570102	ERR10567952	ERR10567977	ERR10568043
ERR10567927	ERR10568002	ERR10568139	ERR10568235	ERR10568331	ERR10566110	ERR10566168	ERR10566284
ERR10566226	ERR10570295	ERR10570847	ERR10570559	ERR10570103	ERR10570199	ERR10570391	ERR10570655
ERR10570751	ERR10567978	ERR10568044	ERR10568140	ERR10567928	ERR10567953	ERR10568003	ERR10568236
ERR10568332	ERR10566285	ERR10566111	ERR10566169	ERR10566227	ERR10570560	ERR10570200	ERR10570296
ERR10570392	ERR10570656	ERR10570752	ERR10570848	ERR10570104	ERR10567929	ERR10567979	ERR10568004
ERR10568045	ERR10568237	ERR10567954	ERR10568141	ERR10568333	ERR10566170	ERR10566112	ERR10566228
ERR10566286	ERR10570753	ERR10570393	ERR10570657	ERR10570201	ERR10570297	ERR10570561	ERR10570849
ERR10570105	ERR10567930	ERR10567955	ERR10568005	ERR10568046	ERR10568142	ERR10568334	ERR10567980
ERR10568238	ERR10566229	ERR10566113	ERR10566171	ERR10566287	ERR10570298	ERR10570394	ERR10570658
ERR10570850	ERR10570562	ERR10570202	ERR10570754	ERR10570106	ERR10567931	ERR10567981	ERR10568006
ERR10568047	ERR10568143	ERR10568335	ERR10567956	ERR10568239	ERR10566172	ERR10566288	ERR10566114
ERR10566230	ERR10570395	ERR10570851	ERR10570755	ERR10570107	ERR10570203	ERR10570299	ERR10570563
ERR10570659	ERR10567982	ERR10568007	ERR10568048	ERR10567932	ERR10567957	ERR10568144	ERR10568240
ERR10568336	ERR10566289	ERR10566115	ERR10566173	ERR10566231	ERR10570300	ERR10570564	ERR10570756
ERR10570204	ERR10570396	ERR10570660	ERR10570852	ERR10570108	ERR10567933	ERR10568008	ERR10568049
ERR10568145	ERR10568337	ERR10567958	ERR10567983	ERR10568241	ERR10566116	ERR10566174	ERR10566232
ERR10566290	ERR10570757	ERR10570853	ERR10570301	ERR10570397	ERR10570565	ERR10570661	ERR10570109
ERR10570205	ERR10568050	ERR10568242	ERR10567934	ERR10567959	ERR10567984	ERR10568009	ERR10568146
ERR10568338	ERR10566233	ERR10566117	ERR10566175	ERR10566291	ERR10570302	ERR10570758	ERR10570398
ERR10570566	ERR10570662	ERR10570854	ERR10570110	ERR10570206	ERR10567960	ERR10568147	ERR10568243
ERR10567935	ERR10567985	ERR10568010	ERR10568051	ERR10568339	ERR10566118	ERR10566234	ERR10566292
ERR10566176	ERR10570663	ERR10570759	ERR10570111	ERR10570207	ERR10570303	ERR10570399	ERR10570567
ERR10570855	ERR10568052	ERR10567936	ERR10567961	ERR10567986	ERR10568011	ERR10568148	ERR10568244
ERR10568340	ERR10566119	ERR10566177	ERR10566235	ERR10566293	ERR10570304	ERR10570400	ERR10570664
ERR10570208	ERR10570568	ERR10570760	ERR10570856	ERR10570112	ERR10568012	ERR10568149	ERR10568245
ERR10567937	ERR10567962	ERR10567987	ERR10568053	ERR10568341	ERR10566120	ERR10566178	ERR10566236
ERR10566294	ERR10570401	ERR10570761	ERR10570569	ERR10570665	ERR10570857	ERR10570113	ERR10570305
ERR10570209	ERR10568150	ERR10568246	ERR10568054	ERR10568342	ERR10566121	ERR10566179	ERR10566237
ERR10566295	ERR10570402	ERR10570500	ERR10570507	ERR10570528	ERR10570493	ERR10570514	ERR10570521
ERR10570210	ERR10570306	ERR10570486	ERR10570535	ERR10570570	ERR10570666	ERR10570762	ERR10570858
ERR10570114	ERR10568055	ERR10568247	ERR10568343	ERR10568151	ERR10566296	ERR10566122	ERR10566180
ERR10566238	ERR10570494	ERR10570501	ERR10570508	ERR10570571	ERR10570859	ERR10570115	ERR10570211
ERR10570307	ERR10570403	ERR10570487	ERR10570515	ERR10570522	ERR10570529	ERR10570536	ERR10570667
ERR10570763	ERR10568152	ERR10568056	ERR10568248	ERR10568344	ERR10566123	ERR10566181	ERR10566239
ERR10566297	ERR10570308	ERR10570404	ERR10570860	ERR10570212	ERR10570572	ERR10570668	ERR10570764
ERR10570116	ERR10568057	ERR10568345	ERR10568153	ERR10568249	ERR10566182	ERR10566240	ERR10566298

ERR10566124	ERR10570495	ERR10570502	ERR10570537	ERR10570669	ERR10570523	ERR10570117	ERR10570213
ERR10570309	ERR10570405	ERR10570488	ERR10570509	ERR10570516	ERR10570530	ERR10570573	ERR10570765
ERR10570861	ERR10568058	ERR10568154	ERR10568250	ERR10568346	ERR10566125	ERR10566183	ERR10566241
ERR10566299	ERR10570766	ERR10570406	ERR10570670	ERR10570118	ERR10570310	ERR10570574	ERR10570862
ERR10570214	ERR10568059	ERR10568155	ERR10568251	ERR10568347	ERR10566184	ERR10566242	ERR10566126
ERR10566300	ERR10570311	ERR10570863	ERR10570407	ERR10570575	ERR10570671	ERR10570767	ERR10570119
ERR10570215	ERR10568348	ERR10568060	ERR10568156	ERR10568252	ERR10566127	ERR10566185	ERR10566243
ERR10566301	ERR10570408	ERR10570312	ERR10570576	ERR10570672	ERR10570768	ERR10570864	ERR10570120
ERR10570216	ERR10568157	ERR10568253	ERR10568349	ERR10568061	ERR10566186	ERR10566244	ERR10566128
ERR10566302	ERR10570409	ERR10570313	ERR10570577	ERR10570673	ERR10570769	ERR10570865	ERR10570121
ERR10570217	ERR10568158	ERR10568254	ERR10568062	ERR10568350	ERR10566129	ERR10566245	ERR10566187
ERR10566303	ERR10570524	ERR10570531	ERR10570503	ERR10570770	ERR10570218	ERR10570314	ERR10570410
ERR10570489	ERR10570496	ERR10570510	ERR10570517	ERR10570538	ERR10570578	ERR10570674	ERR10570866
ERR10570122	ERR10568063	ERR10568255	ERR10568159	ERR10568351	ERR10566246	ERR10566304	ERR10566130
ERR10566188	ERR10570504	ERR10570511	ERR10570525	ERR10570518	ERR10570771	ERR10570123	ERR10570219
ERR10570315	ERR10570411	ERR10570490	ERR10570497	ERR10570532	ERR10570539	ERR10570579	ERR10570675
ERR10570867	ERR10568352	ERR10568064	ERR10568160	ERR10568256	ERR10566189	ERR10566131	ERR10566247
ERR10566305	ERR10570676	ERR10570868	ERR10570220	ERR10570316	ERR10570412	ERR10570580	ERR10570772
ERR10570124	ERR10568161	ERR10568065	ERR10568257	ERR10568353	ERR10566132	ERR10566190	ERR10566248
ERR10566306	ERR10570317	ERR10570773	ERR10570125	ERR10570221	ERR10570413	ERR10570581	ERR10570677
ERR10570869	ERR10568066	ERR10568162	ERR10568258	ERR10568354	ERR10566479	ERR10566361	ERR10566400
ERR10566440	ERR10570126	ERR10570222	ERR10570318	ERR10570414	ERR10570582	ERR10570678	ERR10570774
ERR10570870	ERR10568067	ERR10568163	ERR10568355	ERR10568259	ERR10566362	ERR10566441	ERR10566401
ERR10566480	ERR10570319	ERR10570871	ERR10570679	ERR10570775	ERR10570415	ERR10570583	ERR10570127
ERR10570223	ERR10568068	ERR10568356	ERR10568164	ERR10568260	ERR10566363	ERR10566402	ERR10566442
ERR10566481	ERR10570776	ERR10570224	ERR10570320	ERR10570416	ERR10570584	ERR10570680	ERR10570872
ERR10570128	ERR10568069	ERR10568165	ERR10568261	ERR10568357	ERR10566403	ERR10566482	ERR10566364
ERR10566443	ERR10570417	ERR10570777	ERR10570681	ERR10570129	ERR10570321	ERR10570585	ERR10570873
ERR10570225	ERR10568070	ERR10568166	ERR10568262	ERR10568358	ERR10566404	ERR10566365	ERR10566444
ERR10566483	ERR10570586	ERR10570418	ERR10570226	ERR10570322	ERR10570682	ERR10570778	ERR10570874
ERR10570130	ERR10568167	ERR10568359	ERR10568071	ERR10568263	ERR10566405	ERR10566366	ERR10566445
ERR10566484	ERR10570323	ERR10570683	ERR10570419	ERR10570587	ERR10570131	ERR10570227	ERR10570779
ERR10570875	ERR10568072	ERR10568168	ERR10568264	ERR10568360	ERR10566367	ERR10566446	ERR10566485
ERR10566406	ERR10570420	ERR10570876	ERR10570684	ERR10570780	ERR10570132	ERR10570324	ERR10570588
ERR10570228	ERR10568073	ERR10568169	ERR10568265	ERR10568361	ERR10566368	ERR10566407	ERR10566447
ERR10566486	ERR10570877	ERR10570781	ERR10570133	ERR10570229	ERR10570325	ERR10570421	ERR10570589
ERR10570685	ERR10568074	ERR10568170	ERR10568266	ERR10568362	ERR10566369	ERR10566408	ERR10566487
ERR10566448	ERR10570782	ERR10570686	ERR10570326	ERR10570422	ERR10570590	ERR10570878	ERR10570134
ERR10570230	ERR10568075	ERR10568267	ERR10568363	ERR10568171	ERR10566370	ERR10566409	ERR10566449
ERR10566488	ERR10570423	ERR10570783	ERR10570135	ERR10570231	ERR10570327	ERR10570591	ERR10570687
ERR10570879	ERR10568172	ERR10568364	ERR10568076	ERR10568268	ERR10566410	ERR10566371	ERR10566450
ERR10566489	ERR10570424	ERR10570784	ERR10570136	ERR10570232	ERR10570328	ERR10570592	ERR10570688
ERR10570880	ERR10568365	ERR10568077	ERR10568173	ERR10568269	ERR10566372	ERR10566411	ERR10566451
ERR10566490	ERR10570329	ERR10570425	ERR10570593	ERR10570785	ERR10570233	ERR10570689	ERR10570881
ERR10570137	ERR10568078	ERR10568174	ERR10568270	ERR10568366	ERR10566191	ERR10566307	ERR10566133
ERR10566249	ERR10570426	ERR10570786	ERR10570882	ERR10570138	ERR10570234	ERR10570330	ERR10570594
ERR10570690	ERR10568079	ERR10568271	ERR10568175	ERR10568367	ERR10566134	ERR10566192	ERR10566250
ERR10566308	ERR10570595	ERR10570691	ERR10570139	ERR10570331	ERR10570427	ERR10570787	ERR10570883
ERR10570235	ERR10568272	ERR10567938	ERR10567963	ERR10567988	ERR10568013	ERR10568080	ERR10568176
ERR10568368	ERR10566309	ERR10566135	ERR10566193	ERR10566251	ERR10570596	ERR10570140	ERR10570332
ERR10570428	ERR10570692	ERR10570788	ERR10570884	ERR10570236	ERR10568177	ERR10568273	ERR10568081
ERR10568369	ERR10566136	ERR10566194	ERR10566252	ERR10566310	ERR10570885	ERR10570141	ERR10570237
ERR10570333	ERR10570429	ERR10570597	ERR10570693	ERR10570789	ERR10568178	ERR10568082	ERR10568274
ERR10568370	ERR10566137	ERR10566195	ERR10566253	ERR10566311	ERR10570430	ERR10570790	ERR10570694
ERR10570142	ERR10570238	ERR10570334	ERR10570598	ERR10570886	ERR10568275	ERR10568083	ERR10568179
ERR10568371	ERR10566254	ERR10566312	ERR10566138	ERR10566196	ERR10570431	ERR10570599	ERR10570239
ERR10570335	ERR10570695	ERR10570791	ERR10570887	ERR10570143	ERR10568084	ERR10568180	ERR10568372
ERR10568276	ERR10566197	ERR10566255	ERR10566139	ERR10566313	ERR10570144	ERR10570336	ERR10570432

ERR10570600	ERR10570696	ERR10570792	ERR10570888	ERR10570240	ERR10568085	ERR10568181	ERR10568277
ERR10568373	ERR10566140	ERR10566198	ERR10566314	ERR10566256	ERR10570697	ERR10570433	ERR10570793
ERR10570145	ERR10570337	ERR10570601	ERR10570889	ERR10570241	ERR10568086	ERR10568374	ERR10568182
ERR10568278	ERR10566141	ERR10566199	ERR10566257	ERR10566315	ERR10570338	ERR10570602	ERR10570434
ERR10570890	ERR10570146	ERR10570242	ERR10570698	ERR10570794	ERR10568087	ERR10568183	ERR10568279
ERR10568375	ERR10566142	ERR10566316	ERR10566200	ERR10566258	ERR10570339	ERR10570891	ERR10570699
ERR10570795	ERR10570435	ERR10570603	ERR10570147	ERR10570243	ERR10568088	ERR10568280	ERR10568376
ERR10568184	ERR10566143	ERR10566259	ERR10566317	ERR10566201	ERR10570148	ERR10570244	ERR10570340
ERR10570436	ERR10570604	ERR10570700	ERR10570796	ERR10570892	ERR10568185	ERR10568281	ERR10568089
ERR10568377	ERR10566260	ERR10566202	ERR10566144	ERR10566318	ERR10570341	ERR10570437	ERR10570533
ERR10570540	ERR10570149	ERR10570491	ERR10570498	ERR10570505	ERR10570512	ERR10570519	ERR10570526
ERR10570605	ERR10570701	ERR10570797	ERR10570893	ERR10570245	ERR10568090	ERR10568186	ERR10568282
ERR10568378	ERR10566373	ERR10566412	ERR10566452	ERR10566491	ERR10570894	ERR10570438	ERR10570798
ERR10570246	ERR10570342	ERR10570606	ERR10570702	ERR10570150	ERR10568091	ERR10568187	ERR10568283
ERR10568379	ERR10566413	ERR10566453	ERR10566492	ERR10566374	ERR10570703	ERR10570799	ERR10570895
ERR10570151	ERR10570343	ERR10570439	ERR10570607	ERR10570247	ERR10568092	ERR10568284	ERR10568188
ERR10568380	ERR10566414	ERR10566375	ERR10566454	ERR10566493	ERR10570344	ERR10570896	ERR10570152
ERR10570440	ERR10570608	ERR10570704	ERR10570800	ERR10570248	ERR10568093	ERR10568189	ERR10568285
ERR10568381	ERR10566376	ERR10566415	ERR10566494	ERR10566455	ERR10570345	ERR10570441	ERR10570609
ERR10570705	ERR10570801	ERR10570897	ERR10570153	ERR10570249	ERR10568094	ERR10568190	ERR10568286
ERR10568382	ERR10566377	ERR10566416	ERR10566495	ERR10566456	ERR10570802	ERR10570706	ERR10570154
ERR10570346	ERR10570442	ERR10570610	ERR10570898	ERR10570250	ERR10568191	ERR10568287	ERR10568095
ERR10568383	ERR10566496	ERR10566378	ERR10566417	ERR10566457	ERR10570347	ERR10570611	ERR10570803
ERR10570251	ERR10570443	ERR10570707	ERR10570899	ERR10570155	ERR10568288	ERR10568096	ERR10568192
ERR10568384	ERR10566358	ERR10566397	ERR10566418	ERR10566437	ERR10566476	ERR10570708	ERR10570444
ERR10570612	ERR10570900	ERR10570156	ERR10570348	ERR10570804	ERR10570252	ERR10568385	ERR10568097
ERR10568193	ERR10568289	ERR10566458	ERR10566379	ERR10566419	ERR10566497	ERR10570445	ERR10570805
ERR10570157	ERR10570253	ERR10570349	ERR10570613	ERR10570709	ERR10570901	ERR10568194	ERR10568386
ERR10568098	ERR10568290	ERR10566380	ERR10566420	ERR10566459	ERR10566498	ERR10570446	ERR10570902
ERR10570158	ERR10570350	ERR10570614	ERR10570710	ERR10570806	ERR10570254	ERR10568099	ERR10568195
ERR10568291	ERR10568387	ERR10566421	ERR10566381	ERR10566460	ERR10566499	ERR10570351	ERR10570615
ERR10570159	ERR10570255	ERR10570447	ERR10570711	ERR10570807	ERR10570903	ERR10568292	ERR10568388
ERR10568100	ERR10568196	ERR10566422	ERR10566500	ERR10566382	ERR10566461	ERR10570352	ERR10570904
ERR10570448	ERR10570160	ERR10570616	ERR10570712	ERR10570808	ERR10570256	ERR10568197	ERR10568389
ERR10568101	ERR10568293	ERR10566383	ERR10566423	ERR10566462	ERR10566501	ERR10570353	ERR10570905
ERR10570617	ERR10570161	ERR10570449	ERR10570713	ERR10570809	ERR10570257	ERR10568390	ERR10568102
ERR10568198	ERR10568294	ERR10566424	ERR10566384	ERR10566463	ERR10566502	ERR10570354	ERR10570450
ERR10570810	ERR10570618	ERR10570714	ERR10570906	ERR10570162	ERR10570258	ERR10568103	ERR10568391
ERR10568199	ERR10568295	ERR10566425	ERR10566464	ERR10566503	ERR10566385	ERR10570451	ERR10570619
ERR10570163	ERR10570259	ERR10570355	ERR10570715	ERR10570811	ERR10570907	ERR10568296	ERR10568392
ERR10568104	ERR10568200	ERR10566386	ERR10566426	ERR10566465	ERR10566504	ERR10570716	ERR10570812
ERR10570164	ERR10570356	ERR10570452	ERR10570620	ERR10570908	ERR10570260	ERR10568105	ERR10568201
ERR10568297	ERR10568393	ERR10566387	ERR10566466	ERR10566427	ERR10566505	ERR10570357	ERR10570621
ERR10570813	ERR10570453	ERR10570717	ERR10570909	ERR10570165	ERR10570261	ERR10568394	ERR10568106
ERR10568202	ERR10568298	ERR10566467	ERR10566506	ERR10566388	ERR10566428	ERR10570454	ERR10570622
ERR10570718	ERR10570358	ERR10570814	ERR10570910	ERR10570166	ERR10570262	ERR10568203	ERR10568299
ERR10568395	ERR10568107	ERR10566389	ERR10566507	ERR10566429	ERR10566468	ERR10570815	ERR10570167
ERR10570263	ERR10570359	ERR10570455	ERR10570623	ERR10570719	ERR10570911	ERR10568204	ERR10568108
ERR10568300	ERR10568396	ERR10566390	ERR10566469	ERR10566508	ERR10566430	ERR10570360	ERR10570624
ERR10570456	ERR10570720	ERR10570816	ERR10570912	ERR10570168	ERR10570264	ERR10568397	ERR10568109
ERR10568205	ERR10568301	ERR10566509	ERR10566391	ERR10566431	ERR10566470	ERR10570817	ERR10570169
ERR10570361	ERR10570457	ERR10570625	ERR10570721	ERR10570913	ERR10570265	ERR10568110	ERR10568302
ERR10568206	ERR10568398	ERR10566392	ERR10566471	ERR10566510	ERR10566432	ERR10570818	ERR10570170
ERR10570266	ERR10570362	ERR10570458	ERR10570626	ERR10570722	ERR10570914	ERR10568207	ERR10568111
ERR10568303	ERR10568399	ERR10566393	ERR10566433	ERR10566472	ERR10566511	ERR10570267	ERR10570363
ERR10570459	ERR10570627	ERR10570723	ERR10570819	ERR10570915	ERR10570171	ERR10568208	ERR10568400
ERR10568112	ERR10568304	ERR10566394	ERR10566434	ERR10566473	ERR10566512	ERR10570724	ERR10570460
ERR10570172	ERR10570364	ERR10570628	ERR10570820	ERR10570916	ERR10570268	ERR10568305	ERR10568209

ERR10568113	ERR10568401	ERR10566395	ERR10566435	ERR10566474	ERR10566513	ERR10570917	ERR10570173
ERR10570269	ERR10570365	ERR10570461	ERR10570629	ERR10570725	ERR10570821	ERR10568114	ERR10568306
ERR10568210	ERR10568402	ERR10566261	ERR10566319	ERR10566145	ERR10566203	ERR10570270	ERR10570366
ERR10570462	ERR10570726	ERR10570918	ERR10570174	ERR10570630	ERR10570822	ERR10568115	ERR10568211
ERR10568403	ERR10568307	ERR10566204	ERR10566146	ERR10566262	ERR10566320	ERR10570367	ERR10570463
ERR10570919	ERR10570175	ERR10570271	ERR10570631	ERR10570727	ERR10570823	ERR10568308	ERR10568116
ERR10568212	ERR10568404	ERR10566147	ERR10566263	ERR10566321	ERR10566205	ERR10570272	ERR10570728
ERR10570368	ERR10570464	ERR10570632	ERR10570824	ERR10570920	ERR10570176	ERR10568117	ERR10568213
ERR10568309	ERR10568405	ERR10566206	ERR10566264	ERR10566148	ERR10566322	ERR10570729	ERR10570465
ERR10570177	ERR10570273	ERR10570369	ERR10570633	ERR10570825	ERR10570921	ERR10568118	ERR10568214
ERR10568310	ERR10568406	ERR10566323	ERR10566149	ERR10566207	ERR10566265	ERR10570274	ERR10570370
ERR10570466	ERR10570634	ERR10570730	ERR10570826	ERR10570922	ERR10570178	ERR10568311	ERR10568407
ERR10568119	ERR10568215	ERR10566150	ERR10566208	ERR10566266	ERR10566324	ERR10570731	ERR10570179
ERR10570275	ERR10570371	ERR10570467	ERR10570635	ERR10570827	ERR10570923	ERR10568120	ERR10568216
ERR10568312	ERR10568408	ERR10566151	ERR10566209	ERR10566325	ERR10566267	ERR10570276	ERR10570924
ERR10570468	ERR10570636	ERR10570180	ERR10570372	ERR10570732	ERR10570828	ERR10568121	ERR10568217
ERR10568313	ERR10568409	ERR10566359	ERR10566438	ERR10566398	ERR10566477	ERR10570277	ERR10570733
ERR10570373	ERR10570637	ERR10570181	ERR10570469	ERR10570829	ERR10570925	ERR10568122	ERR10568218
ERR10568314	ERR10568410	ERR10566268	ERR10566326	ERR10566152	ERR10566210	ERR10570734	ERR10570926
ERR10570374	ERR10570278	ERR10570470	ERR10570638	ERR10570830	ERR10570182	ERR10568123	ERR10568219
ERR10568315	ERR10568411	ERR10566211	ERR10566153	ERR10566269	ERR10566327	ERR10570492	ERR10570499
ERR10570534	ERR10570513	ERR10570520	ERR10570541	ERR10570735	ERR10570927	ERR10570279	ERR10570375
ERR10570471	ERR10570506	ERR10570527	ERR10570639	ERR10570183	ERR10568124	ERR10568412	ERR10568220
ERR10568316	ERR10566270	ERR10566328	ERR10566154	ERR10566212	ERR10570280	ERR10570736	ERR10570184
ERR10570376	ERR10570472	ERR10570640	ERR10570832	ERR10570928	ERR10568317	ERR10568125	ERR10568221
ERR10568413	ERR10566213	ERR10566155	ERR10566271	ERR10566329	ERR10570833	ERR10570185	ERR10570281
ERR10570377	ERR10570473	ERR10570641	ERR10570737	ERR10570929	ERR10567989	ERR10568014	ERR10568414
ERR10567939	ERR10567964	ERR10568126	ERR10568222	ERR10568318	ERR10566156	ERR10566214	ERR10566272
ERR10566330	ERR10570282	ERR10570378	ERR10570474	ERR10570642	ERR10570738	ERR10570834	ERR10570930
ERR10570186	ERR10568015	ERR10568127	ERR10568223	ERR10567940	ERR10567965	ERR10567990	ERR10568319
ERR10566273	ERR10566331	ERR10566157	ERR10566215	ERR10570283	ERR10570931	ERR10570475	ERR10570643
ERR10570835	ERR10570187	ERR10570379	ERR10570739	ERR10567966	ERR10568016	ERR10568416	ERR10567941
ERR10567991	ERR10568128	ERR10568224	ERR10568320	ERR10566158	ERR10566274	ERR10566216	ERR10566332
ERR10570380	ERR10570644	ERR10570740	ERR10570932	ERR10570284	ERR10570476	ERR10570836	ERR10570188
ERR10567992	ERR10568017	ERR10568225	ERR10568321	ERR10568417	ERR10567942	ERR10567967	ERR10568129
ERR10566159	ERR10566217	ERR10566275	ERR10566333	ERR10570381	ERR10570741	ERR10570285	ERR10570477
ERR10570645	ERR10570837	ERR10570933	ERR10570189	ERR10567968	ERR10568322	ERR10568418	ERR10567943
ERR10567993	ERR10568018	ERR10568130	ERR10568226	ERR10566160	ERR10566218	ERR10566276	ERR10566334
ERR10570286	ERR10570478	ERR10570742	ERR10570382	ERR10570934	ERR10570646	ERR10570838	ERR10570190
ERR10567994	ERR10568131	ERR10568227	ERR10568323	ERR10567944	ERR10567969	ERR10568019	ERR10568419
ERR10566219	ERR10566335	ERR10566161	ERR10566277	ERR10570935	ERR10570647	ERR10570743	ERR10570191
ERR10570287	ERR10570383	ERR10570479	ERR10570839	ERR10568132	ERR10568228	ERR10568420	ERR10567945
ERR10567970	ERR10567995	ERR10568020	ERR10568324	ERR10566162	ERR10566336	ERR10566220	ERR10566278
ERR10570288	ERR10570936	ERR10570648	ERR10570840	ERR10570384	ERR10570480	ERR10570744	ERR10570192
ERR10567996	ERR10568133	ERR10568229	ERR10568421	ERR10567946	ERR10567971	ERR10568021	ERR10568325
ERR10566163	ERR10566221	ERR10566279	ERR10566337	ERR10570385	ERR10570745	ERR10570289	ERR10570481
ERR10570649	ERR10570841	ERR10570937	ERR10570193	ERR10568230	ERR10568422	ERR10567947	ERR10567972
ERR10567997	ERR10568022	ERR10568134	ERR10568326	ERR10566222	ERR10566164	ERR10566280	ERR10566338
ERR10570290	ERR10570842	ERR10570386	ERR10570650	ERR10570194	ERR10570482	ERR10570746	ERR10570938
ERR10567973	ERR10567998	ERR10567948	ERR10568023	ERR10568135	ERR10568231	ERR10568327	ERR10568423
ERR10566165	ERR10566223	ERR10566281	ERR10566339	ERR10570747	ERR10570291	ERR10570387	ERR10570483
ERR10570651	ERR10570843	ERR10570939	ERR10570195	ERR10567949	ERR10568024	ERR10568136	ERR10568232
ERR10568424	ERR10567974	ERR10567999	ERR10568328	ERR10566166	ERR10566282	ERR10566340	ERR10566224
ERR10570292	ERR10570748	ERR10570940	ERR10570388	ERR10570484	ERR10570652	ERR10570844	ERR10570196
ERR10567950	ERR10568329	ERR10567975	ERR10568000	ERR10568025	ERR10568137	ERR10568233	ERR10568425
ERR10566399	ERR10566478	ERR10566360	ERR10566439	ERR10570293	ERR10570389	ERR10570653	ERR10570749
ERR10570845	ERR10570941	ERR10570485	ERR10570197	ERR10568030	ERR10568038	ERR10568026	ERR10568034
ERR10566341	ERR10566345	ERR10566349	ERR10566436	ERR10566353	ERR10566357	ERR10566396	ERR10566475

ERR10570546	ERR10570554	ERR10570542	ERR10570550	ERR10568027	ERR10568031	ERR10568039	ERR10568035
ERR10566354	ERR10566342	ERR10566346	ERR10566350	ERR10570543	ERR10570555	ERR10570547	ERR10570551
ERR10568028	ERR10568032	ERR10568036	ERR10568040	ERR10566351	ERR10566343	ERR10566347	ERR10566355
ERR10570544	ERR10570548	ERR10570552	ERR10570556	ERR10568033	ERR10568041	ERR10568029	ERR10568037
ERR10566356	ERR10566344	ERR10566348	ERR10566352	ERR10570545	ERR10570549	ERR10570553	ERR10570557
ERR11219000	ERR11219001	ERR11219002	ERR11219003	ERR11219004	ERR11219005	ERR11219006	ERR11219007
ERR11219008	ERR11219009	ERR11219010	ERR11219011	ERR11219012	ERR11219013	ERR11219014	ERR11219015
ERR11219016	ERR11219017	ERR11219018	ERR11219019	ERR11219020	ERR11219021	ERR11219022	ERR11219023
ERR11219024	ERR11219025	ERR11219026	ERR11219027	ERR11219028	ERR11219029	ERR11219030	ERR11219031
ERR11219032	ERR11219033	ERR11219034	ERR11219035	ERR11219036	ERR11219037	ERR11219038	ERR11219039
ERR11219040	ERR11219041	ERR11219042	ERR11219043	ERR11219044	ERR11219045	ERR11219046	ERR11219047
ERR11219048	ERR11219049	ERR11219050	ERR11219051	ERR11219052	ERR11219053	ERR11219055	ERR11219056
ERR11219057	ERR11219058	ERR11219059	ERR11219060	ERR11219061	ERR11219062	ERR11219063	ERR11219064
ERR11219065	ERR11219066	ERR11219067	ERR11219068	ERR11219069	ERR11219070	ERR11219071	ERR11219072
ERR11219073	ERR11219074	ERR11219075	ERR11219076	ERR11219077	ERR11219078	ERR11219079	ERR11219080
ERR11219081	ERR11219082	ERR11219083	ERR11219084	ERR11219085	ERR11219086	ERR11219087	ERR11219088
ERR11219089	ERR11219090	ERR11219091	ERR11219092	ERR11219093	ERR11219094	ERR11219095	ERR11219096
ERR11219097	ERR11219098	ERR11219099	ERR11219100	ERR11219101	ERR11219102	ERR11219103	ERR11219104
ERR11219106	ERR11219107	ERR11219108	ERR11219109	ERR11219110	ERR11219111	ERR11219112	ERR11219113
ERR11219114	ERR11219115	ERR11219116	ERR11219117	ERR11219118	ERR11219119	ERR566407	ERR566408
ERR566409	ERR566410	ERR566411	ERR566412	ERR566413	ERR566414	ERR566415	ERR566416
ERR566417	ERR566418	ERR566419	ERR566420	ERR566421	ERR566422	ERR566423	ERR566424
ERR566425	ERR566426	ERR566427	ERR566428	ERR566429	ERR566430	ERR566431	ERR566432
ERR566433	ERR629115	ERR629118	ERR629114	ERR629119	ERR629116	ERR629117	ERR712331
ERR712329	ERR712318	ERR712334	ERR712340	ERR712342	ERR712326	ERR712316	ERR712338
ERR712325	ERR712343	ERR712333	ERR712347	ERR712346	ERR712345	ERR712339	ERR712319
ERR712323	ERR712327	ERR712328	ERR712321	ERR712324	ERR712330	ERR712348	ERR712335
ERR712336	ERR712315	ERR712332	ERR712344	ERR712314	ERR712337	ERR712320	ERR712322
ERR712317	ERR712341	ERR712349	ERR1462495	ERR1462496	ERR1462497	ERR1462498	ERR1462499
ERR1462500	ERR1890429	ERR1890430	ERR1890431	ERR1890432	ERR1890433	ERR1890434	ERR1890435
ERR1890436	ERR1890437	ERR1890438	ERR1890439	ERR1890440	ERR1890441	ERR777787	ERR777781
ERR777784	ERR777783	ERR777785	ERR777786	ERR777782	ERR789427	ERR789435	ERR789443
ERR789428	ERR789436	ERR789444	ERR789429	ERR789437	ERR789445	ERR789430	ERR789438
ERR789446	ERR789431	ERR789439	ERR789447	ERR789432	ERR789440	ERR789448	ERR789433
ERR789441	ERR789449	ERR789434	ERR789442	ERR789450	ERR972389	ERR972387	ERR972388
ERR926727	ERR926729	ERR926728	ERR926731	ERR926732	ERR926730	ERR926734	ERR926735
ERR926733	ERR926737	ERR926736	ERR926738	ERR926739	ERR926740	ERR926741	ERR926742
ERR926743	ERR926744	ERR926747	ERR926745	ERR926746	ERR926749	ERR926748	ERR926750
ERR961026	ERR961027	ERR961028	ERR961029	ERR961030	ERR961031	ERR961032	ERR961033
ERR961034	ERR961035	ERR961036	ERR961037	ERR961038	ERR961039	ERR961040	ERR961041
ERR961042	ERR961043	ERR961044	ERR961045	SRR039170	SRR039171	SRR039169	SRR039199
SRR039200	SRR039201	SRR066365	SRR066366	SRR414980	SRR504819	SRR504820	SRR504821
SRR504822	SRR504823	SRR504824	SRR504825	SRR504826	SRR504827	SRR504818	SRR543739
SRR543893	SRR543897	SRR544922	SRR636881	SRR636882	SRR636928	SRR636929	SRR636973
SRR636974	SRR651712	SRR651713	SRR651714	SRR651715	SRR651716	SRR651717	SRR651718
SRR651719	SRR651720	SRR653843	SRR653844	SRR653845	SRR653846	SRR689211	SRR689212
SRR689213	SRR689214	SRR689215	SRR689216	SRR689217	SRR689218	SRR689219	SRR689220
SRR689221	SRR689222	SRR689223	SRR689224	SRR689225	SRR689226	SRR689227	SRR689228
SRR689229	SRR689230	SRR747926	SRR747924	SRR747925	SRR768448	SRR768468	SRR768469
SRR776497	SRR776498	SRR776499	SRR776500	SRR776501	SRR776502	SRR847962	SRR847963
SRR847964	SRR847965	SRR847966	SRR847967	SRR847968	SRR847969	SRR847970	SRR847971
SRR847972	SRR847973	SRR926619	SRR926620	SRR926621	SRR926622	SRR926623	SRR926624
SRR931786	SRR931788	SRR931785	SRR931787	SRR934623	SRR934626	SRR934625	SRR934628
SRR934629	SRR934627	SRR934624	SRR934630	SRR1006118	SRR1006119	SRR1006120	SRR1006121
SRR1006122	SRR1006123	SRR1006124	SRR1006125	SRR1006126	SRR1006127	SRR1006128	SRR1006129
SRR1006130	SRR1006131	SRR1006132	SRR1006133	SRR1004850	SRR1004851	SRR1014962	SRR1015059
SRR1015065	SRR1015070	SRR1015077	SRR1015079	SRR1022874	SRR1022875	SRR1022876	SRR1022877
SRR1022886	SRR1022887	SRR1022888	SRR1022889	SRR1022890	SRR1022891	SRR1022892	SRR1022893

SRR1022898	SRR1022899	SRR1022900	SRR1022901	SRR1022878	SRR1022879	SRR1022880	SRR1022881
SRR1022910	SRR1022911	SRR1022912	SRR1022913	SRR1022902	SRR1022903	SRR1022904	SRR1022905
SRR1022906	SRR1022907	SRR1022908	SRR1022909	SRR1022882	SRR1022883	SRR1022884	SRR1022885
SRR1022894	SRR1022895	SRR1022896	SRR1022897	SRR1047797	SRR1047809	SRR1048153	SRR1048200
SRR1048368	SRR1048422	SRR1051324	SRR1051323	SRR1051327	SRR1051322	SRR1051330	SRR1051326
SRR1051325	SRR1051329	SRR1051328	SRR1107684	SRR1107703	SRR1107709	SRR1107711	SRR1107716
SRR1107718	SRR1107727	SRR1107728	SRR1107730	SRR1107729	SRR1118244	SRR1118243	SRR2088056
SRR2088738	SRR5110497	SRR5112682	SRR5110983	SRR5110991	SRR5111004	SRR1184417	SRR1184418
SRR1184419	SRR1184420	SRR1184422	SRR1184421	SRR1184423	SRR1184424	SRR1184425	SRR1184426
SRR1184427	SRR1184428	SRR1185159	SRR1185160	SRR1185162	SRR1185152	SRR1185157	SRR1185156
SRR1185151	SRR1185165	SRR1185150	SRR1185168	SRR1185171	SRR1185174	SRR1185177	SRR1185158
SRR1185161	SRR1185164	SRR1185163	SRR1185180	SRR1185184	SRR1185169	SRR1185190	SRR1185175
SRR1185187	SRR1185178	SRR1185170	SRR1185173	SRR1185192	SRR1185181	SRR1185179	SRR1185176
SRR1185183	SRR1185167	SRR1185185	SRR1185195	SRR1185172	SRR1185186	SRR1185166	SRR1185182
SRR1185188	SRR1185194	SRR1185196	SRR1185193	SRR1185189	SRR1185191	SRR1185197	SRR1185198
SRR1185200	SRR1185199	SRR1185202	SRR1185207	SRR1185204	SRR1185201	SRR1185211	SRR1185210
SRR1185208	SRR1185124	SRR1185127	SRR1185130	SRR1185205	SRR1185213	SRR1185133	SRR1185203
SRR1185139	SRR1185136	SRR1185145	SRR1185206	SRR1185142	SRR1185209	SRR1185123	SRR1185128
SRR1185125	SRR1185212	SRR1185214	SRR1185126	SRR1185129	SRR1185132	SRR1185131	SRR1185134
SRR1185137	SRR1185141	SRR1185135	SRR1185138	SRR1185143	SRR1185144	SRR1185148	SRR1185146
SRR1185149	SRR1185140	SRR1185154	SRR1185153	SRR1185155	SRR1185147	SRR1282324	SRR1282322
SRR1282327	SRR1282330	SRR1282325	SRR1282326	SRR1282323	SRR1282329	SRR1282328	SRR1282332
SRR1282333	SRR1282331	SRR1519321	SRR1519322	SRR1566034	SRR1565997	SRR1572014	SRR1572008
SRR1572009	SRR1572010	SRR1572011	SRR1572012	SRR1572013	SRR1572015	SRR1572016	SRR1572017
SRR1572018	SRR1572019	SRR1572020	SRR1572021	SRR1572029	SRR1572022	SRR1572023	SRR1572024
SRR1572025	SRR1572026	SRR1572027	SRR1572028	SRR1572030	SRR1572031	SRR1572032	SRR1572033
SRR1572034	SRR1572035	SRR1572036	SRR1572037	SRR1572038	SRR1572039	SRR1572040	SRR1572041
SRR1572042	SRR1572043	SRR1628663	SRR1628662	SRR1628661	SRR1688258	SRR1951540	SRR1951539
SRR1736623	SRR1736624	SRR1737100	SRR1737101	SRR1737102	SRR1736622	SRR1781531	SRR1805952
SRR1805956	SRR1805950	SRR1805933	SRR1805954	SRR1805947	SRR1805955	SRR1805940	SRR1805957
SRR1805944	SRR1805930	SRR1805958	SRR1805961	SRR1805931	SRR1805948	SRR1805953	SRR1805946
SRR1805929	SRR1805941	SRR1805932	SRR1805945	SRR1805960	SRR1805964	SRR1805942	SRR1805943
SRR1805959	SRR1805962	SRR1805963	SRR1805951	SRR1805949	SRR1805938	SRR1805936	SRR1805939
SRR1805935	SRR1805934	SRR1805937	SRR2980118	SRR2980119	SRR2980120	SRR2980121	SRR2980122
SRR2980123	SRR2980124	SRR2980125	SRR2980126	SRR2980127	SRR2980128	SRR2980129	SRR2980130
SRR2980131	SRR2980132	SRR2980133	SRR2980134	SRR2980135	SRR2980136	SRR2980137	SRR2980138
SRR2980139	SRR2980140	SRR2980141	SRR2980142	SRR2980143	SRR2980144	SRR2980145	SRR2980146
SRR2980147	SRR2980148	SRR2980149	SRR2980150	SRR2980018	SRR2980019	SRR2980020	SRR2980021
SRR2980022	SRR2980023	SRR2980024	SRR2980025	SRR2980026	SRR2980027	SRR2980028	SRR2980029
SRR2980030	SRR2980031	SRR2980032	SRR2980065	SRR2980066	SRR2980067	SRR2980068	SRR2980069
SRR2980070	SRR1979629	SRR1979630	SRR1979631	SRR1979632	SRR1979633	SRR1979634	SRR1979635
SRR1979636	SRR1979637	SRR1979638	SRR1979639	SRR1979640	SRR1979641	SRR1979642	SRR1979643
SRR1979644	SRR1979645	SRR1979646	SRR1979647	SRR1979648	SRR2006107	SRR2006108	SRR2016644
SRR2029398	SRR2029399	SRR2029575	SRR2029576	SRR2029577	SRR2025257	SRR2025285	SRR2025299
SRR2029646	SRR2029647	SRR2029648	SRR2029649	SRR2029650	SRR2029651	SRR2029652	SRR2029653
SRR2035184	SRR2035185	SRR2035186	SRR2035187	SRR2035188	SRR2035189	SRR2035190	SRR2035191
SRR2035192	SRR7770492	SRR7770491	SRR7770496	SRR7770495	SRR7770494	SRR7770493	SRR2095545
SRR2095546	SRR2123960	SRR2123961	SRR2123963	SRR2123964	SRR2123965	SRR2123966	SRR2123967
SRR2123968	SRR2123962	SRR2123969	SRR2123970	SRR2123971	SRR2123972	SRR2123973	SRR2123974
SRR2123975	SRR2123976	SRR2123977	SRR2123978	SRR2123979	SRR2123980	SRR2123885	SRR2123886
SRR2123887	SRR2123888	SRR2123889	SRR2123890	SRR2123891	SRR2123892	SRR2123893	SRR2123894
SRR2123895	SRR2123896	SRR2123897	SRR2123898	SRR2123899	SRR2123900	SRR2123901	SRR2123902
SRR2123903	SRR2123904	SRR2123905	SRR2123906	SRR2123907	SRR2123908	SRR2123909	SRR2123910
SRR2123911	SRR2123912	SRR2123913	SRR2123914	SRR2123915	SRR2123916	SRR2123917	SRR2123918
SRR2123919	SRR2123920	SRR2123921	SRR2123922	SRR2123923	SRR2123924	SRR2123925	SRR2123926
SRR2123927	SRR2123928	SRR2123929	SRR2123930	SRR2123931	SRR2123932	SRR2123933	SRR2123934
SRR2123935	SRR2123936	SRR2123937	SRR2123938	SRR2123939	SRR2123940	SRR2123941	SRR2123942
SRR2123943	SRR2123944	SRR2123945	SRR2123946	SRR2123947	SRR2123948	SRR2123949	SRR2123950

SRR2123951	SRR2123952	SRR2123953	SRR2123954	SRR2123955	SRR2123956	SRR2123957	SRR2123958
SRR2123959	SRR2130359	SRR2130360	SRR5872577	SRR5872576	SRR5872575	SRR5872574	SRR5872573
SRR5872572	SRR5872571	SRR5872570	SRR5872569	SRR5872568	SRR5989241	SRR5872567	SRR5872565
SRR5872563	SRR5989240	SRR5872562	SRR5872561	SRR2069946	SRR2148913	SRR2148914	SRR2148915
SRR2148916	SRR2148917	SRR2148918	SRR2148923	SRR2148924	SRR2148925	SRR2148926	SRR2148919
SRR2148920	SRR2148921	SRR2148922	SRR2170106	SRR2170107	SRR2183559	SRR2183560	SRR2183561
SRR2183562	SRR2183563	SRR2183564	SRR2183566	SRR2183567	SRR2183568	SRR2183569	SRR2183570
SRR2183565	SRR2288563	SRR2288571	SRR2288565	SRR2288579	SRR2288782	SRR2288566	SRR2288783
SRR2292026	SRR2288784	SRR2288574	SRR2292020	SRR2292021	SRR2288584	SRR2288599	SRR2292024
SRR2292022	SRR2292025	SRR2292023	SRR2294735	SRR2307981	SRR2308102	SRR2536875	SRR2536876
SRR2536877	SRR2536878	SRR2536873	SRR2536874	SRR2536879	SRR2536880	SRR2536881	SRR2536882
SRR2536883	SRR2536884	SRR2537252	SRR2537253	SRR2537254	SRR2537255	SRR2537256	SRR2537257
SRR2550374	SRR2550375	SRR2550376	SRR2564757	SRR2564758	SRR2564759	SRR2564760	SRR2564761
SRR2564762	SRR2564763	SRR2564764	SRR2564765	SRR2564766	SRR2564767	SRR2564768	SRR2564769
SRR2564770	SRR2564771	SRR2564772	SRR2564773	SRR2564774	SRR2662909	SRR2662927	SRR2662928
SRR2663407	SRR2634171	SRR2640162	SRR2640267	SRR2640268	SRR2640269	SRR2640270	SRR2642730
SRR2642731	SRR2642732	SRR2640596	SRR2640600	SRR2640601	SRR2859026	SRR2859027	SRR2859028
SRR2859029	SRR2859030	SRR2859031	SRR2859032	SRR2859033	SRR2912674	SRR2912895	SRR2912896
SRR2912897	SRR2912898	SRR2920203	SRR2920204	SRR2920205	SRR2920206	SRR2920207	SRR2920208
SRR2920209	SRR2920210	SRR2920211	SRR2920212	SRR2920213	SRR2920214	SRR2920215	SRR2920216
SRR2920217	SRR2920218	SRR2920219	SRR2920220	SRR2920221	SRR2920222	SRR2920223	SRR2920224
SRR2920225	SRR2920226	SRR2920227	SRR2920228	SRR2920229	SRR2920230	SRR2920231	SRR2920232
SRR2920233	SRR2920234	SRR2920235	SRR2920236	SRR2920237	SRR2920238	SRR2920239	SRR2920240
SRR2920241	SRR2920242	SRR2920243	SRR2920244	SRR2920245	SRR2920246	SRR2920247	SRR2920248
SRR2920249	SRR2920250	SRR2920251	SRR2920252	SRR2920253	SRR2920254	SRR2920255	SRR2920256
SRR2920257	SRR2920258	SRR2920259	SRR2920260	SRR2920261	SRR2920267	SRR2920268	SRR2920269
SRR2920270	SRR2920271	SRR2920272	SRR2920273	SRR2920274	SRR2920275	SRR2920276	SRR2920277
SRR2920278	SRR2920279	SRR2920280	SRR2920281	SRR2920282	SRR2920283	SRR2920284	SRR2920285
SRR2920286	SRR2920287	SRR2920288	SRR2920289	SRR2920290	SRR2920291	SRR2920292	SRR2920293
SRR2920294	SRR2920295	SRR2920296	SRR2920262	SRR2920263	SRR2920264	SRR2920265	SRR2920266
SRR13291717	SRR13291716	SRR13291704	SRR13291700	SRR13291699	SRR13291698	SRR13291713	SRR13291697
SRR13291696	SRR13291695	SRR13291715	SRR13291714	SRR13291712	SRR13291711	SRR13291710	SRR13291709
SRR13291708	SRR13291707	SRR13291706	SRR13291705	SRR13291703	SRR13291702	SRR13291701	SRR2924899
SRR2924891	SRR2924905	SRR2924907	SRR2924909	SRR2919657	SRR2919658	SRR2919659	SRR2919660
SRR2919661	SRR2919662	SRR2924912	SRR2946530	SRR2946532	SRR2946533	SRR2946535	SRR2946536
SRR2946538	SRR2946539	SRR2946541	SRR2946542	SRR2946844	SRR2946889	SRR2924898	SRR2924906
SRR2924924	SRR2924926	SRR2924928	SRR2952735	SRR2952736	SRR2952751	SRR2952753	SRR2952765
SRR2952510	SRR2952511	SRR2952513	SRR2953733	SRR2953734	SRR2953735	SRR2953736	SRR2953737
SRR2953730	SRR2953731	SRR2953738	SRR2953739	SRR2953740	SRR2953741	SRR2953742	SRR2953743
SRR2953744	SRR2953745	SRR2953746	SRR2953747	SRR2953748	SRR2953749	SRR2953702	SRR2953703
SRR2953704	SRR2953705	SRR2953706	SRR2953707	SRR2953708	SRR2953709	SRR2953710	SRR2953711
SRR2953712	SRR2953713	SRR2953714	SRR2953715	SRR2953716	SRR2953717	SRR2953718	SRR2953719
SRR2953720	SRR2953721	SRR2953722	SRR2953723	SRR2953724	SRR2953725	SRR2953726	SRR2953727
SRR2953728	SRR2953729	SRR2953732	SRR2962734	SRR2966619	SRR2966620	SRR2966621	SRR2966622
SRR2966623	SRR2966624	SRR2966625	SRR2966626	SRR2966627	SRR2966628	SRR2966629	SRR2973855
SRR2973856	SRR2973857	SRR2973858	SRR2973859	SRR2973860	SRR2973861	SRR2973862	SRR2973863
SRR2973864	SRR2973865	SRR2973866	SRR2973867	SRR2973868	SRR2973869	SRR2973870	SRR2973871
SRR2973872	SRR2973873	SRR2973874	SRR2973875	SRR2973876	SRR2973877	SRR2973878	SRR2973879
SRR2973880	SRR2973881	SRR2973882	SRR2973883	SRR2973884	SRR2973885	SRR2973886	SRR2976589
SRR2983627	SRR2983628	SRR2983629	SRR2983630	SRR2983631	SRR2983632	SRR2983633	SRR2983634
SRR2983635	SRR2983636	SRR2983637	SRR2983638	SRR2983639	SRR2983640	SRR2983641	SRR2983642
SRR2983643	SRR2983644	SRR2983645	SRR2983646	SRR2983647	SRR2983648	SRR2983649	SRR2983650
SRR2983651	SRR2983652	SRR2983653	SRR2983654	SRR2983655	SRR2983656	SRR2983657	SRR2983658
SRR2994963	SRR2995031	SRR2995134	SRR2995139	SRR2995142	SRR2995144	SRR2995148	SRR2995151
SRR2995155	SRR2995140	SRR6134356	SRR6134355	SRR6134349	SRR6134346	SRR6134347	SRR6134345
SRR6134350	SRR6134359	SRR6134361	SRR3098385	SRR3066483	SRR3066484	SRR3091847	SRR3091848
SRR3091849	SRR3091850	SRR3091851	SRR3091852	SRR3091853	SRR3091854	SRR3091855	SRR3091856
SRR3091857	SRR3091858	SRR3105562	SRR3105563	SRR3105564	SRR3105565	SRR3105566	SRR3105567

SRR3105568	SRR3105569	SRR3105570	SRR3105571	SRR3105572	SRR3105573	SRR3105574	SRR3105576
SRR3105577	SRR3105578	SRR3105579	SRR3105580	SRR3107147	SRR3107144	SRR3107145	SRR3107146
SRR3107148	SRR3107149	SRR3107150	SRR3107162	SRR3107163	SRR3115003	SRR3147581	SRR3147562
SRR3147507	SRR3147511	SRR3147497	SRR3147520	SRR3147524	SRR3147518	SRR3147532	SRR3147549
SRR3147536	SRR3147554	SRR3147555	SRR3147561	SRR3147498	SRR3147517	SRR3147570	SRR3147544
SRR3147572	SRR3147585	SRR3147558	SRR3147504	SRR3147547	SRR3147525	SRR3147564	SRR3147506
SRR3147535	SRR3147577	SRR3147519	SRR3147578	SRR3147552	SRR3147503	SRR3147501	SRR3147512
SRR3147539	SRR3147527	SRR3147560	SRR3147541	SRR3147568	SRR3147574	SRR3147556	SRR3147580
SRR3147499	SRR3147567	SRR3147571	SRR3147505	SRR3147513	SRR3147579	SRR3147586	SRR3147502
SRR3147521	SRR3147516	SRR3147534	SRR3147533	SRR3147530	SRR3147551	SRR3147545	SRR3147548
SRR3147563	SRR3147553	SRR3147576	SRR3147565	SRR3147514	SRR3147569	SRR3147584	SRR3147528
SRR3147529	SRR3147583	SRR3147510	SRR3147546	SRR3147508	SRR3147522	SRR3147559	SRR3147526
SRR3147573	SRR3147537	SRR3160078	SRR3160079	SRR3160016	SRR3160017	SRR3160020	SRR3160021
SRR3160022	SRR3160023	SRR3160024	SRR3160025	SRR3160026	SRR3160027	SRR3160028	SRR3160029
SRR3160030	SRR3160031	SRR3160032	SRR3160033	SRR3160034	SRR3160035	SRR3160036	SRR3160037
SRR3160038	SRR3160039	SRR3160040	SRR3160041	SRR3160042	SRR3160048	SRR3160049	SRR3160050
SRR3160051	SRR3160043	SRR3159988	SRR3159989	SRR3159990	SRR3159991	SRR3159992	SRR3159993
SRR3159994	SRR3159995	SRR3159996	SRR3159997	SRR3159998	SRR3159999	SRR3160000	SRR3160001
SRR3160002	SRR3160003	SRR3160004	SRR3160005	SRR3160006	SRR3160007	SRR3160008	SRR3160009
SRR3160018	SRR3160019	SRR3160010	SRR3160011	SRR3160012	SRR3160013	SRR3160014	SRR3160015
SRR3160044	SRR3160045	SRR3160046	SRR3160047	SRR3160052	SRR3160053	SRR3160054	SRR3160055
SRR3160056	SRR3160057	SRR3160058	SRR3160059	SRR3160060	SRR3160061	SRR3160062	SRR3160063
SRR3160064	SRR3160065	SRR3160066	SRR3160067	SRR3160068	SRR3160069	SRR3160070	SRR3160071
SRR3160072	SRR3160073	SRR3160074	SRR3160075	SRR3160076	SRR3160077	SRR3178395	SRR3194646
SRR3194647	SRR3194648	SRR3194649	SRR3194650	SRR3194651	SRR3194652	SRR3194653	SRR3194654
SRR3194655	SRR3194656	SRR3194657	SRR3194658	SRR3194659	SRR3194660	SRR3194661	SRR3194662
SRR3194663	SRR3194664	SRR3194665	SRR3194666	SRR3194667	SRR3194668	SRR3194669	SRR3194670
SRR3194671	SRR3194672	SRR3194673	SRR3194674	SRR3194675	SRR3194764	SRR3194765	SRR3194768
SRR3194769	SRR3194770	SRR3194771	SRR3194772	SRR3194773	SRR3194774	SRR3194775	SRR3194776
SRR3194777	SRR3194778	SRR3194779	SRR3194780	SRR3194781	SRR3194782	SRR3194783	SRR3194784
SRR3194785	SRR3194786	SRR3194787	SRR3194788	SRR3194789	SRR3194790	SRR3194791	SRR3194792
SRR3194793	SRR3194794	SRR3194736	SRR3194737	SRR3194738	SRR3194739	SRR3194740	SRR3194741
SRR3194742	SRR3194743	SRR3194744	SRR3194745	SRR3194746	SRR3194747	SRR3194748	SRR3194749
SRR3194750	SRR3194751	SRR3194752	SRR3194753	SRR3194754	SRR3194755	SRR3194756	SRR3194757
SRR3194766	SRR3194767	SRR3194758	SRR3194759	SRR3194760	SRR3194761	SRR3194762	SRR3194763
SRR3194676	SRR3194677	SRR3194678	SRR3194679	SRR3194680	SRR3194681	SRR3194682	SRR3194683
SRR3194684	SRR3194685	SRR3194686	SRR3194687	SRR3194688	SRR3194689	SRR3194690	SRR3194691
SRR3194692	SRR3194693	SRR3194694	SRR3194695	SRR3194696	SRR3194697	SRR3194698	SRR3194699
SRR3194706	SRR3194707	SRR3194708	SRR3194709	SRR3194700	SRR3194701	SRR3194702	SRR3194703
SRR3194704	SRR3194705	SRR3194710	SRR3194711	SRR3194712	SRR3194713	SRR3194714	SRR3194715
SRR3194716	SRR3194717	SRR3194718	SRR3194719	SRR3194720	SRR3194721	SRR3194722	SRR3194723
SRR3194724	SRR3194725	SRR3194726	SRR3194727	SRR3194728	SRR3194729	SRR3194730	SRR3194731
SRR3194732	SRR3194733	SRR3194734	SRR3194735	SRR3194616	SRR3194617	SRR3194618	SRR3194619
SRR3194620	SRR3194621	SRR3194622	SRR3194623	SRR3194624	SRR3194625	SRR3194626	SRR3194627
SRR3194628	SRR3194629	SRR3194630	SRR3194631	SRR3194632	SRR3194633	SRR3194634	SRR3194635
SRR3194636	SRR3194637	SRR3194638	SRR3194639	SRR3194640	SRR3194641	SRR3194642	SRR3194643
SRR3194644	SRR3194645	SRR3280907	SRR3280908	SRR3280909	SRR3280910	SRR3280911	SRR3280912
SRR3292629	SRR3354390	SRR3390777	SRR3437128	SRR3437129	SRR3437130	SRR3437131	SRR3437132
SRR3437133	SRR3440108	SRR3440109	SRR5556386	SRR5556385	SRR5556388	SRR5556387	SRR5556390
SRR5556389	SRR3571535	SRR3571536	SRR3571537	SRR3571538	SRR3571539	SRR3571540	SRR3621730
SRR3621731	SRR3621732	SRR3621733	SRR3621734	SRR3621735	SRR3659816	SRR3659817	SRR3720831
SRR3720832	SRR3892839	SRR3892840	SRR3892842	SRR3892843	SRR3892844	SRR3892845	SRR3892846
SRR3892841	SRR3892835	SRR3892836	SRR3892837	SRR3892838	SRR4046569	SRR4046617	SRR4046619
SRR4046620	SRR4046621	SRR4046627	SRR4046630	SRR4046632	SRR4046633	SRR4046634	SRR4046635
SRR4046636	SRR4046637	SRR4046639	SRR4046645	SRR4046646	SRR4046647	SRR4046660	SRR4068061
SRR6938191	SRR4068060	SRR6938190	SRR4068065	SRR6938195	SRR4068064	SRR6938194	SRR4068063
SRR6938193	SRR4068062	SRR6938192	SRR4173987	SRR4173986	SRR4173985	SRR4174008	SRR4174007
SRR4174006	SRR4174005	SRR4174004	SRR4174003	SRR4174002	SRR4174001	SRR4174000	SRR4173999

SRR4173998	SRR4173997	SRR4173996	SRR4173995	SRR4173994	SRR4173993	SRR4173992	SRR4173991
SRR4173990	SRR4173989	SRR4173988	SRR4237534	SRR4242263	SRR4242264	SRR4253493	SRR4253494
SRR4289329	SRR4294896	SRR4294757	SRR4294899	SRR4294957	SRR4294782	SRR4296451	SRR4296450
SRR4296449	SRR4296448	SRR4296447	SRR4296446	SRR4299124	SRR4299125	SRR4299126	SRR4299127
SRR4299128	SRR4299130	SRR4299131	SRR4299134	SRR4299133	SRR4299135	SRR4299165	SRR4299209
SRR4299245	SRR4299246	SRR4299247	SRR4299248	SRR4299249	SRR4299250	SRR4299251	SRR4299252
SRR4299253	SRR4299254	SRR4299255	SRR4299256	SRR4299257	SRR4299258	SRR4299259	SRR4299260
SRR4299261	SRR4299262	SRR4299263	SRR4299264	SRR4299265	SRR4299266	SRR4299268	SRR4299269
SRR4299270	SRR4299271	SRR4299272	SRR4299273	SRR4299281	SRR4299302	SRR4299321	SRR4299338
SRR4299357	SRR4344101	SRR4344099	SRR4344097	SRR4344103	SRR4361753	SRR4407928	SRR4407927
SRR4407926	SRR4407925	SRR4407930	SRR4407929	SRR4412562	SRR4412561	SRR4412560	SRR4412565
SRR4412564	SRR4412563	SRR4415934	SRR4415933	SRR4449192	SRR4449197	SRR4449200	SRR4449203
SRR4449205	SRR4449207	SRR4449209	SRR4449211	SRR4998774	SRR5000321	SRR5000322	SRR5000323
SRR5000701	SRR5000703	SRR5000704	SRR5000705	SRR4915910	SRR4916228	SRR4916956	SRR4917363
SRR4917364	SRR4917683	SRR4918135	SRR4919131	SRR4919188	SRR4920041	SRR4920043	SRR4920045
SRR5010450	SRR5010461	SRR5010460	SRR5010459	SRR5010458	SRR5010457	SRR5010456	SRR5010455
SRR5010454	SRR5010453	SRR5010452	SRR5010451	SRR5027048	SRR5027063	SRR5027050	SRR5027061
SRR5027062	SRR5027051	SRR5027049	SRR5027064	SRR5027060	SRR5042828	SRR5042849	SRR5042851
SRR5042824	SRR5042830	SRR5042847	SRR5042832	SRR5042854	SRR5042855	SRR5043827	SRR5043828
SRR5043829	SRR5043824	SRR5043825	SRR5043826	SRR5043821	SRR5043822	SRR5043823	SRR5043818
SRR5043819	SRR5043820	SRR5054331	SRR5054336	SRR5054335	SRR5054334	SRR5054333	SRR5054332
SRR5070621	SRR5070627	SRR5070628	SRR5070629	SRR5091888	SRR5091889	SRR5091885	SRR5091886
SRR5091884	SRR5091887	SRR5121266	SRR5112953	SRR5112955	SRR5112954	SRR5115708	SRR5115709
SRR5115711	SRR5115713	SRR5115712	SRR5115710	SRR5565144	SRR5565143	SRR5565141	SRR5565139
SRR5565147	SRR5565137	SRR5565135	SRR5565145	SRR5565142	SRR5565140	SRR5565138	SRR5565146
SRR5565136	SRR5565134	SRR5565133	SRR5130133	SRR5130132	SRR5130131	SRR5130130	SRR5130129
SRR5130128	SRR5130127	SRR5130126	SRR5130125	SRR5130124	SRR5130123	SRR5130122	SRR5130121
SRR5130120	SRR5130119	SRR5130118	SRR5130117	SRR5130116	SRR5133954	SRR5133961	SRR5133960
SRR5133959	SRR5133958	SRR5133957	SRR5133956	SRR5133955	SRR5136328	SRR5136327	SRR5136326
SRR5136325	SRR5149568	SRR5149564	SRR5149562	SRR5149563	SRR5149560	SRR5149570	SRR5149573
SRR5149572	SRR5149566	SRR5149569	SRR5149571	SRR5149575	SRR5149567	SRR5149561	SRR5149574
SRR5149565	SRR5155450	SRR5155464	SRR5155485	SRR5155476	SRR5155461	SRR5155474	SRR5155459
SRR5155448	SRR5155484	SRR5155458	SRR5155473	SRR5155468	SRR5155482	SRR5155460	SRR5155483
SRR5155455	SRR5155477	SRR5155475	SRR5155454	SRR5155463	SRR5155453	SRR5155449	SRR5155478
SRR5155472	SRR5155481	SRR5155452	SRR5155457	SRR5155456	SRR5155462	SRR5155470	SRR5155480
SRR5155469	SRR5155479	SRR5155451	SRR5155467	SRR5155466	SRR5155465	SRR5155471	SRR6266864
SRR6266882	SRR6266897	SRR6266863	SRR6266881	SRR6266898	SRR6266866	SRR6266884	SRR6266899
SRR6266865	SRR6266883	SRR6266900	SRR6266860	SRR6266886	SRR6266890	SRR6266859	SRR6266885
SRR6266891	SRR6266862	SRR6266888	SRR6266902	SRR6266861	SRR6266887	SRR6266901	SRR6266858
SRR6266880	SRR6266892	SRR6266857	SRR6266879	SRR6266904	SRR6266872	SRR6266876	SRR6266893
SRR6266871	SRR6266875	SRR6266894	SRR6266870	SRR6266874	SRR6266895	SRR6266869	SRR6266873
SRR6266896	SRR6266868	SRR6266878	SRR6266903	SRR6266867	SRR6266877	SRR6266889	SRR5190482
SRR5190481	SRR5190480	SRR5190479	SRR5190478	SRR5190477	SRR5190470	SRR5190469	SRR5190468
SRR5190467	SRR5190466	SRR5190465	SRR5190449	SRR5190448	SRR5190416	SRR5190415	SRR5190414
SRR5190413	SRR5190412	SRR5190411	SRR5190410	SRR5190409	SRR5190408	SRR5190398	SRR5190397
SRR5190390	SRR5190391	SRR5190396	SRR5190395	SRR5190394	SRR5190393	SRR5190392	SRR5190506
SRR5190505	SRR5190504	SRR5190447	SRR5258689	SRR5272666	SRR5651315	SRR5651316	SRR5651317
SRR5332131	SRR5332130	SRR5332129	SRR5332128	SRR5332127	SRR5332126	SRR5332125	SRR5332124
SRR5332123	SRR5332122	SRR5332121	SRR5332120	SRR5332119	SRR5332118	SRR5344859	SRR5344858
SRR5344857	SRR5344856	SRR5344855	SRR5344854	SRR5345646	SRR5345688	SRR5345687	SRR5345686
SRR5345685	SRR5345684	SRR5345683	SRR5345682	SRR5345681	SRR5345680	SRR5345679	SRR5345678
SRR5345677	SRR5345676	SRR5345675	SRR5345674	SRR5345673	SRR5345672	SRR5345671	SRR5345670
SRR5345669	SRR5345668	SRR5345667	SRR5345666	SRR5345665	SRR5345664	SRR5345663	SRR5345662
SRR5345661	SRR5345660	SRR5345659	SRR5345658	SRR5345657	SRR5345656	SRR5345655	SRR5345654
SRR5345653	SRR5345652	SRR5345651	SRR5345650	SRR5345649	SRR5345643	SRR5345642	SRR5345644
SRR5345645	SRR5345647	SRR5345635	SRR5345634	SRR5345633	SRR5345632	SRR5345629	SRR5345628
SRR5345630	SRR5345631	SRR5345625	SRR5345626	SRR5345627	SRR5345636	SRR5345637	SRR5345638
SRR5345641	SRR5345639	SRR5345640	SRR5345648	SRR5406933	SRR5406934	SRR5434052	SRR5434053

SRR5434205	SRR5434216	SRR5434218	SRR5434219	SRR5434220	SRR5434221	SRR5434222	SRR5445530
SRR5445529	SRR5445528	SRR5445527	SRR5445533	SRR5445532	SRR5445531	SRR5470022	SRR5470021
SRR5470026	SRR5470025	SRR5470024	SRR5470023	SRR5507589	SRR5507588	SRR5507587	SRR5507590
SRR5507598	SRR5507597	SRR5507596	SRR5507595	SRR5507594	SRR5507593	SRR5507592	SRR5507591
SRR5755682	SRR5755681	SRR5755683	SRR5617597	SRR5617600	SRR5617596	SRR5617601	SRR5617595
SRR5617615	SRR5617614	SRR5617611	SRR5617616	SRR5617613	SRR5617591	SRR5617592	SRR5617590
SRR5617589	SRR5617594	SRR5617617	SRR5617603	SRR5617610	SRR5617612	SRR5617618	SRR5617626
SRR5617627	SRR5617622	SRR5617624	SRR5617625	SRR5617599	SRR5617593	SRR5617598	SRR5617602
SRR5617604	SRR5617623	SRR5617619	SRR5617620	SRR5617621	SRR5617605	SRR5617607	SRR5617609
SRR5617606	SRR5617608	SRR6761654	SRR6761653	SRR6761660	SRR6761657	SRR6761650	SRR6761649
SRR6761652	SRR6761661	SRR6761659	SRR6761658	SRR6761656	SRR6761655	SRR6761651	SRR6761662
SRR5577190	SRR5577189	SRR5577191	SRR5577193	SRR5577194	SRR5577192	SRR5643634	SRR5643635
SRR5643636	SRR5643637	SRR5643638	SRR5643639	SRR5643640	SRR5643641	SRR5651382	SRR5651381
SRR5651389	SRR5651388	SRR5651387	SRR5651386	SRR5651385	SRR5651384	SRR5651383	SRR5663677
SRR5663679	SRR5663675	SRR5663678	SRR5663672	SRR5663669	SRR5663668	SRR5663676	SRR5663664
SRR5663661	SRR5663671	SRR5663670	SRR5663673	SRR5663663	SRR5663662	SRR5663674	SRR5663667
SRR5663666	SRR5663665	SRR5665198	SRR5665199	SRR5665200	SRR5665201	SRR5665202	SRR5665193
SRR5665194	SRR5665195	SRR5665196	SRR5665197	SRR5665208	SRR5665203	SRR5665206	SRR5665207
SRR5665209	SRR5665204	SRR5665210	SRR5665205	SRR5665212	SRR5665211	SRR5665190	SRR5665192
SRR5665191	SRR5666720	SRR5666721	SRR5817984	SRR5817985	SRR5868416	SRR5868415	SRR5868414
SRR5868413	SRR5868417	SRR5868418	SRR5801198	SRR5801199	SRR5801704	SRR5801705	SRR5801715
SRR5801716	SRR5801910	SRR5801911	SRR5801912	SRR5801913	SRR5801923	SRR5801924	SRR5801925
SRR5801926	SRR5801938	SRR5801939	SRR5802407	SRR5802408	SRR5802409	SRR5802410	SRR5802415
SRR5802416	SRR5802417	SRR5802418	SRR5802423	SRR5802424	SRR5802425	SRR5802426	SRR5802555
SRR5802556	SRR5802557	SRR5802558	SRR5802567	SRR5802568	SRR5802569	SRR5802570	SRR5802577
SRR5802578	SRR5802579	SRR5802580	SRR5802612	SRR5802613	SRR5802614	SRR5802615	SRR5802617
SRR5802618	SRR5802619	SRR5802620	SRR5802700	SRR5802701	SRR5802702	SRR5802703	SRR5803010
SRR5803011	SRR5803012	SRR5803013	SRR5803014	SRR5803015	SRR5803016	SRR5803017	SRR5803018
SRR5803019	SRR5803020	SRR5803021	SRR5803022	SRR5803023	SRR5803024	SRR5803025	SRR5803026
SRR5803027	SRR5803028	SRR5803029	SRR5803071	SRR5803072	SRR5803073	SRR5803074	SRR5803236
SRR5803237	SRR5803238	SRR5803239	SRR5803803	SRR5803804	SRR5803805	SRR5803806	SRR5803826
SRR5803827	SRR5803828	SRR5803829	SRR5803840	SRR5803841	SRR5803842	SRR5803843	SRR5803848
SRR5803849	SRR5803850	SRR5803851	SRR5803856	SRR5803857	SRR5803858	SRR5803859	SRR5803862
SRR5803863	SRR5803864	SRR5803865	SRR5803880	SRR5803881	SRR5803891	SRR5803892	SRR5804040
SRR5804041	SRR5804123	SRR5804124	SRR5804133	SRR5804134	SRR5804148	SRR5804149	SRR5813848
SRR5813849	SRR5815991	SRR5815992	SRR5815993	SRR5815994	SRR5816152	SRR5816153	SRR5816154
SRR5816155	SRR5816156	SRR5816157	SRR5816158	SRR5816159	SRR5816160	SRR5816230	SRR5816231
SRR5816232	SRR5816233	SRR5816234	SRR5816235	SRR5816236	SRR5816237	SRR5816238	SRR5816148
SRR5816149	SRR5816239	SRR5816240	SRR5816241	SRR5816242	SRR5816243	SRR5816244	SRR5816245
SRR5816246	SRR5816247	SRR5816259	SRR5816261	SRR5816354	SRR5816355	SRR5816356	SRR5816357
SRR5816358	SRR5816359	SRR5816360	SRR5816361	SRR5816362	SRR5816363	SRR5816368	SRR5826457
SRR5826479	SRR5826478	SRR5826477	SRR5826476	SRR5826475	SRR5826474	SRR5826473	SRR5826472
SRR5826471	SRR5826470	SRR5826469	SRR5826468	SRR5826467	SRR5826466	SRR5826465	SRR5826464
SRR5826463	SRR5826462	SRR5826461	SRR5826460	SRR5826459	SRR5826458	SRR5830882	SRR5874347
SRR5874346	SRR5874349	SRR5874348	SRR5874351	SRR5874350	SRR5874353	SRR5874352	SRR5874355
SRR5874354	SRR5874345	SRR5874344	SRR5874343	SRR5874342	SRR5877117	SRR5877119	SRR5877116
SRR5877115	SRR5877114	SRR5877113	SRR5877112	SRR5877111	SRR5877110	SRR5877109	SRR5877108
SRR5877107	SRR5877106	SRR5877105	SRR5877118	SRR5939830	SRR5939835	SRR5939834	SRR5939833
SRR5939832	SRR5939831	SRR6023675	SRR6023676	SRR6023677	SRR6023678	SRR6023671	SRR6023672
SRR6023673	SRR6023674	SRR6023669	SRR6023670	SRR6023710	SRR6023711	SRR6023712	SRR6023713
SRR6023714	SRR6023715	SRR6023716	SRR6023717	SRR6023708	SRR6023709	SRR6023734	SRR6023733
SRR6023736	SRR6023735	SRR6023730	SRR6023729	SRR6023732	SRR6023731	SRR6023738	SRR6023737
SRR6023755	SRR6023756	SRR6023753	SRR6023754	SRR6023751	SRR6023752	SRR6023749	SRR6023750
SRR6023757	SRR6023758	SRR6023774	SRR6023773	SRR6023772	SRR6023771	SRR6023778	SRR6023777
SRR6023776	SRR6023775	SRR6023781	SRR6023780	SRR6023825	SRR6023826	SRR6023822	SRR6023697
SRR6023820	SRR6023821	SRR6023823	SRR6023824	SRR6023816	SRR6023817	SRR6023784	SRR6023783
SRR6023788	SRR6023785	SRR6023797	SRR6023782	SRR6023799	SRR6023798	SRR6023682	SRR6023681
SRR6023702	SRR6023703	SRR6023700	SRR6023701	SRR6023706	SRR6023707	SRR6023704	SRR6023705

SRR6023698	SRR6023699	SRR6023696	SRR6023695	SRR6023694	SRR6023800	SRR6023685	SRR6023779
SRR6023680	SRR6023679	SRR6023728	SRR6023727	SRR6023805	SRR6023726	SRR6023814	SRR6023815
SRR6023759	SRR6023770	SRR6023818	SRR6023819	SRR6023801	SRR6023802	SRR6023769	SRR6023768
SRR6023767	SRR6023766	SRR6023765	SRR6023764	SRR6023763	SRR6023762	SRR6023761	SRR6023760
SRR6023739	SRR6023740	SRR6023741	SRR6023742	SRR6023743	SRR6023744	SRR6023745	SRR6023746
SRR6023747	SRR6023748	SRR6023807	SRR6023806	SRR6023809	SRR6023808	SRR6023811	SRR6023810
SRR6023813	SRR6023812	SRR6023804	SRR6023803	SRR6023791	SRR6023792	SRR6023789	SRR6023790
SRR6023795	SRR6023796	SRR6023793	SRR6023794	SRR6023786	SRR6023787	SRR6023689	SRR6023688
SRR6023687	SRR6023686	SRR6023693	SRR6023692	SRR6023691	SRR6023690	SRR6023684	SRR6023683
SRR6023833	SRR6023834	SRR6023835	SRR6023836	SRR6023829	SRR6023830	SRR6023831	SRR6023832
SRR6023827	SRR6023828	SRR6023723	SRR6023722	SRR6023725	SRR6023724	SRR6023719	SRR6023718
SRR6023721	SRR6023720	SRR6043996	SRR6043997	SRR6043994	SRR6043995	SRR6043992	SRR6043993
SRR6043990	SRR6043991	SRR6047326	SRR6047337	SRR6047336	SRR6047335	SRR6047334	SRR6047333
SRR6047332	SRR6047331	SRR6047330	SRR6047329	SRR6047328	SRR6047327	SRR6746579	SRR6746578
SRR6746581	SRR6746580	SRR6746575	SRR6746574	SRR6746577	SRR6746576	SRR6746573	SRR6746572
SRR6746582	SRR6183024	SRR6183023	SRR6183022	SRR6183021	SRR6183020	SRR6183019	SRR6183018
SRR6183017	SRR6183026	SRR6183025	SRR6183008	SRR6183007	SRR6183010	SRR6183009	SRR6183004
SRR6183003	SRR6183006	SRR6183005	SRR6183002	SRR6183001	SRR6183027	SRR6183028	SRR6183029
SRR6183030	SRR6183031	SRR6183032	SRR6183033	SRR6183034	SRR6183035	SRR6183036	SRR6183016
SRR6183015	SRR6183014	SRR6183013	SRR6183012	SRR6183011	SRR6203153	SRR6203152	SRR6203155
SRR6203154	SRR6203156	SRR6203157	SRR6203158	SRR6203160	SRR6203159	SRR6203161	SRR6203140
SRR6203141	SRR6203143	SRR6203142	SRR6203138	SRR6203130	SRR6203147	SRR6203135	SRR6203137
SRR6203162	SRR6203132	SRR6203163	SRR6203150	SRR6203136	SRR6203139	SRR6203131	SRR6203148
SRR6203133	SRR6203151	SRR6203134	SRR6203146	SRR6203145	SRR6203144	SRR6203129	SRR6203149
SRR6203124	SRR6203125	SRR6203126	SRR6203127	SRR6203128	SRR6207744	SRR6207739	SRR6207742
SRR6207743	SRR6207748	SRR6207749	SRR6207746	SRR6207747	SRR6207740	SRR6207741	SRR6207738
SRR6207745	SRR6203227	SRR6203230	SRR6203229	SRR6203228	SRR6203221	SRR6203220	SRR6203219
SRR6203218	SRR6203217	SRR6203216	SRR6203222	SRR6203223	SRR6203226	SRR6203225	SRR6203224
SRR6217852	SRR6217854	SRR6217853	SRR6217850	SRR6217849	SRR6217851	SRR7072771	SRR7072772
SRR7072773	SRR7072774	SRR7072775	SRR7072776	SRR6236876	SRR6236879	SRR6236881	SRR6236882
SRR6236888	SRR6236889	SRR6240077	SRR6240076	SRR6240093	SRR6240092	SRR6240091	SRR6240090
SRR6240089	SRR6240088	SRR6240087	SRR6240086	SRR6240085	SRR6240084	SRR6240083	SRR6240082
SRR6240081	SRR6240080	SRR6240079	SRR6240078	SRR6253565	SRR6253564	SRR6253563	SRR6253562
SRR6253567	SRR6253566	SRR6261053	SRR6261052	SRR6261051	SRR6261050	SRR6261049	SRR6302585
SRR6302586	SRR6302587	SRR6302588	SRR6302589	SRR6302590	SRR6315754	SRR6315756	SRR6315757
SRR6315755	SRR6315758	SRR6315759	SRR6312393	SRR6312392	SRR6312391	SRR6312390	SRR6312389
SRR6312388	SRR6312387	SRR6312386	SRR6312385	SRR6312384	SRR6312383	SRR6312382	SRR6312381
SRR6321508	SRR6321507	SRR6321528	SRR6321527	SRR6321526	SRR6321525	SRR6321524	SRR6321523
SRR6321522	SRR6321521	SRR6321520	SRR6321519	SRR6321518	SRR6321517	SRR6321516	SRR6321515
SRR6321514	SRR6321513	SRR6321512	SRR6321511	SRR6321510	SRR6321509	SRR6328697	SRR6328708
SRR6328707	SRR6328706	SRR6328705	SRR6328704	SRR6328703	SRR6328702	SRR6328701	SRR6328700
SRR6328699	SRR6328698	SRR6339051	SRR6418064	SRR6418063	SRR6418066	SRR6418065	SRR6418068
SRR6418067	SRR6418070	SRR6418069	SRR6418062	SRR6418061	SRR6418060	SRR6418059	SRR6425101
SRR6425088	SRR6425087	SRR6425086	SRR6425085	SRR6425084	SRR6425083	SRR6425082	SRR6425081
SRR6425080	SRR6425079	SRR6425078	SRR6425077	SRR6425076	SRR6425075	SRR6425100	SRR6425099
SRR6425098	SRR6425130	SRR6425129	SRR6425128	SRR6425127	SRR6425126	SRR6425125	SRR6425124
SRR6425123	SRR6425122	SRR6425121	SRR6425120	SRR6425119	SRR6425118	SRR6425117	SRR6425116
SRR6425115	SRR6425114	SRR6425113	SRR6425112	SRR6425111	SRR6425104	SRR6425110	SRR6425109
SRR6425108	SRR6425107	SRR6425106	SRR6425105	SRR6425103	SRR6425102	SRR6425141	SRR6425134
SRR6425140	SRR6425139	SRR6425138	SRR6425137	SRR6425136	SRR6425135	SRR6425133	SRR6425132
SRR6425131	SRR6425097	SRR6425096	SRR6425095	SRR6425094	SRR6425093	SRR6425092	SRR6425091
SRR6425090	SRR6425089	SRR6854200	SRR6854199	SRR6854202	SRR6854201	SRR6854198	SRR6854197
SRR6447590	SRR6447591	SRR6451058	SRR6451057	SRR6451056	SRR6451055	SRR6451054	SRR6451053
SRR6451052	SRR6451051	SRR6451050	SRR6451049	SRR6451048	SRR6451047	SRR6451046	SRR6451045
SRR6451044	SRR6451043	SRR6451042	SRR6451041	SRR6451040	SRR6451039	SRR6451038	SRR6451037
SRR6451036	SRR6451035	SRR6451034	SRR6451033	SRR6451032	SRR6451031	SRR6451030	SRR6451029
SRR6451028	SRR6451027	SRR6451026	SRR6451025	SRR6451024	SRR6451023	SRR6451022	SRR6451021
SRR6451020	SRR6451019	SRR6466790	SRR6466789	SRR6466788	SRR6466787	SRR6466786	SRR6466785

SRR6466784	SRR6466783	SRR6466782	SRR6466781	SRR6466780	SRR6466779	SRR6505932	SRR6505933
SRR6505906	SRR6505907	SRR6505904	SRR6505905	SRR6505902	SRR6505903	SRR6505900	SRR6505901
SRR6505898	SRR6505899	SRR6505896	SRR6505897	SRR6505894	SRR6505895	SRR6505892	SRR6505893
SRR6505890	SRR6505891	SRR6505908	SRR6505909	SRR6505912	SRR6505913	SRR6505910	SRR6505911
SRR6505914	SRR6505915	SRR6505920	SRR6505921	SRR6505918	SRR6505919	SRR6505916	SRR6505917
SRR6505922	SRR6505923	SRR6505930	SRR6505931	SRR6505928	SRR6505929	SRR6505926	SRR6505927
SRR6505924	SRR6505925	SRR6669104	SRR6669105	SRR6669102	SRR6669103	SRR6669106	SRR6669131
SRR6669135	SRR6669134	SRR6669133	SRR6669132	SRR6703676	SRR6703675	SRR6703674	SRR6703673
SRR6703672	SRR6703671	SRR6703670	SRR6703669	SRR6703668	SRR6703677	SRR6705577	SRR6705576
SRR6705575	SRR6705574	SRR6708985	SRR6708986	SRR6708991	SRR6708992	SRR6708989	SRR6708990
SRR6708987	SRR6708988	SRR6799555	SRR6799554	SRR6799553	SRR6799552	SRR6799551	SRR6799550
SRR6799549	SRR6799548	SRR6799547	SRR6799546	SRR6799545	SRR6799544	SRR6799543	SRR6799542
SRR6799541	SRR6799540	SRR6806321	SRR6806316	SRR6806320	SRR6806317	SRR6806319	SRR6806318
SRR6836010	SRR6836011	SRR6836008	SRR6836009	SRR6836006	SRR6836007	SRR6836004	SRR6836005
SRR6836002	SRR6836003	SRR6836000	SRR6836001	SRR6835998	SRR6835999	SRR6835996	SRR6835997
SRR6835994	SRR6835995	SRR6835992	SRR6835993	SRR6835990	SRR6835991	SRR6835988	SRR6835989
SRR6835986	SRR6835987	SRR6835984	SRR6835985	SRR6835982	SRR6835983	SRR6853385	SRR6853384
SRR6853387	SRR6853386	SRR6853389	SRR6853388	SRR6875164	SRR6875165	SRR6875163	SRR6875166
SRR6875162	SRR6875161	SRR6875243	SRR6875248	SRR6875247	SRR6875246	SRR6875245	SRR6875244
SRR6906476	SRR6906475	SRR6906478	SRR6906477	SRR6906472	SRR6906471	SRR6906474	SRR6906473
SRR6906470	SRR6906469	SRR6917747	SRR6918606	SRR6921808	SRR6921806	SRR6921812	SRR6921860
SRR6918831	SRR6918847	SRR6918846	SRR6918845	SRR6918844	SRR6918843	SRR6918842	SRR6918841
SRR6918840	SRR6918839	SRR6918838	SRR6918837	SRR6918836	SRR6918835	SRR6918834	SRR6918833
SRR6918832	SRR6918851	SRR6918850	SRR6918849	SRR6918848	SRR7026134	SRR7026133	SRR7026136
SRR7026135	SRR7026130	SRR7026126	SRR7026125	SRR7026124	SRR7026123	SRR7026122	SRR7026113
SRR7026114	SRR7026111	SRR7026112	SRR7026109	SRR7014208	SRR7014207	SRR7014206	SRR7014205
SRR7014204	SRR7014203	SRR7039078	SRR7039075	SRR7039074	SRR7039073	SRR7039072	SRR7039071
SRR7039070	SRR7039069	SRR7039068	SRR7039067	SRR7039066	SRR7039065	SRR7039064	SRR7039063
SRR7039062	SRR7039061	SRR7039060	SRR7039059	SRR7039058	SRR7039057	SRR7039056	SRR7039055
SRR7039054	SRR7039053	SRR7039052	SRR7039077	SRR7039076	SRR7039081	SRR7039080	SRR7039079
SRR7041907	SRR7041906	SRR7041980	SRR7041979	SRR7041978	SRR7041977	SRR7041976	SRR7041975
SRR7041974	SRR7041973	SRR7041972	SRR7041971	SRR7041970	SRR7041969	SRR7041968	SRR7041967
SRR7041966	SRR7041965	SRR7041964	SRR7041963	SRR7041962	SRR7041961	SRR7042001	SRR7042000
SRR7041999	SRR7041998	SRR7041997	SRR7041994	SRR7041993	SRR7041992	SRR7041991	SRR7041990
SRR7041996	SRR7041930	SRR7041929	SRR7041928	SRR7041927	SRR7041926	SRR7041925	SRR7041924
SRR7041923	SRR7041922	SRR7041921	SRR7041920	SRR7041919	SRR7041918	SRR7041913	SRR7041912
SRR7041917	SRR7041916	SRR7041915	SRR7041914	SRR7041911	SRR7041910	SRR7041909	SRR7041908
SRR7041960	SRR7041959	SRR7041958	SRR7041957	SRR7041956	SRR7041955	SRR7041954	SRR7041953
SRR7041952	SRR7041951	SRR7041950	SRR7041949	SRR7041948	SRR7041947	SRR7041946	SRR7041945
SRR7041944	SRR7041943	SRR7041942	SRR7041941	SRR7041940	SRR7041934	SRR7041933	SRR7041939
SRR7041938	SRR7041937	SRR7041936	SRR7041935	SRR7041932	SRR7041931	SRR7041995	SRR7041989
SRR7041988	SRR7041987	SRR7041986	SRR7041985	SRR7041984	SRR7041983	SRR7041982	SRR7041981
SRR7056599	SRR7056598	SRR7056597	SRR7056596	SRR7056595	SRR7056594	SRR7056593	SRR7056592
SRR7056591	SRR7056590	SRR7056604	SRR7056603	SRR7056602	SRR7056601	SRR7056600	SRR7059835
SRR7059836	SRR7059837	SRR7059838	SRR7059839	SRR7059840	SRR7059841	SRR7059842	SRR7059843
SRR7059844	SRR7059845	SRR7076868	SRR7076869	SRR7076870	SRR7130581	SRR7130580	SRR7130579
SRR7130578	SRR7144120	SRR7144121	SRR7144122	SRR7144123	SRR7144118	SRR7144119	SRR7144126
SRR7144125	SRR7144124	SRR7144129	SRR7144128	SRR7138126	SRR7138151	SRR7138150	SRR7138149
SRR7138148	SRR7138147	SRR7138146	SRR7138145	SRR7138144	SRR7138143	SRR7138142	SRR7138141
SRR7138140	SRR7138139	SRR7138138	SRR7138137	SRR7138136	SRR7138135	SRR7138134	SRR7138133
SRR7138132	SRR7138131	SRR7138130	SRR7138129	SRR7138128	SRR7138127	SRR7138124	SRR7138123
SRR7138122	SRR7138121	SRR7138120	SRR7138119	SRR7138118	SRR7138117	SRR7138116	SRR7138115
SRR7138114	SRR7138113	SRR7138112	SRR7138111	SRR7138110	SRR7138109	SRR7138108	SRR7138107
SRR7138106	SRR7138105	SRR7138104	SRR7138103	SRR7138102	SRR7138101	SRR7138100	SRR7138099
SRR7138098	SRR7138153	SRR7138152	SRR7138125	SRR7138159	SRR7138158	SRR7138163	SRR7138162
SRR7138161	SRR7138160	SRR7178126	SRR7178127	SRR7178124	SRR7178125	SRR7178122	SRR7178123
SRR7178120	SRR7178121	SRR7191217	SRR7191216	SRR7191231	SRR7191230	SRR7191229	SRR7191228
SRR7191227	SRR7191226	SRR7191225	SRR7191224	SRR7191223	SRR7191222	SRR7191221	SRR7191220

SRR7191219	SRR7191218	SRR7191947	SRR7191945	SRR7191944	SRR7191941	SRR7191940	SRR7191946
SRR7191939	SRR7191938	SRR7191937	SRR7191936	SRR7191943	SRR7191942	SRR7207356	SRR7207355
SRR7207354	SRR7207353	SRR7207364	SRR7207363	SRR7207362	SRR7207361	SRR7207360	SRR7207359
SRR7207358	SRR7207357	SRR7410665	SRR7410664	SRR7410667	SRR7410666	SRR7410661	SRR7410660
SRR7410903	SRR7410663	SRR7410662	SRR7410659	SRR7410658	SRR7410657	SRR7267797	SRR7267796
SRR7267799	SRR7267798	SRR7267801	SRR7267800	SRR7267803	SRR7267802	SRR7267805	SRR7267804
SRR7267794	SRR7267793	SRR7267792	SRR7267791	SRR7267795	SRR7369740	SRR7369739	SRR7369738
SRR7369737	SRR7369736	SRR7369735	SRR7294241	SRR7294221	SRR7294220	SRR7294219	SRR7294218
SRR7294217	SRR7294216	SRR7294215	SRR7294214	SRR7294213	SRR7294212	SRR7294211	SRR7294240
SRR7294239	SRR7294246	SRR7294245	SRR7294244	SRR7294243	SRR7294242	SRR7294238	SRR7294237
SRR7294236	SRR7294235	SRR7294234	SRR7294233	SRR7294232	SRR7294231	SRR7294230	SRR7294229
SRR7294228	SRR7294227	SRR7294226	SRR7294225	SRR7294224	SRR7294223	SRR7294222	SRR7370249
SRR7370250	SRR7370248	SRR7381974	SRR7381975	SRR7381973	SRR7377681	SRR7377680	SRR7377682
SRR7385130	SRR7385131	SRR7385129	SRR7429151	SRR7429150	SRR7429149	SRR7429148	SRR7429157
SRR7429156	SRR7429155	SRR7429154	SRR7429153	SRR7429152	SRR7475984	SRR7475983	SRR7475982
SRR7475981	SRR7475980	SRR7475979	SRR7511292	SRR7511291	SRR7511290	SRR7511289	SRR7511288
SRR7511287	SRR7511286	SRR7511285	SRR7511284	SRR7511283	SRR7511282	SRR7511281	SRR8364651
SRR8275389	SRR8275392	SRR8275391	SRR8275388	SRR8275387	SRR7549163	SRR7549162	SRR7549165
SRR7549164	SRR7549159	SRR7549158	SRR7549161	SRR7549160	SRR7630955	SRR7630954	SRR7630957
SRR7630956	SRR7630959	SRR7630958	SRR7630961	SRR7630960	SRR7624150	SRR7624149	SRR7624148
SRR7624147	SRR7624146	SRR7624145	SRR7641814	SRR7641815	SRR7641816	SRR7641817	SRR7647439
SRR7647438	SRR7647437	SRR7647436	SRR7647435	SRR7647434	SRR7647433	SRR7647432	SRR7647431
SRR7647442	SRR7647441	SRR7647440	SRR7703968	SRR7703969	SRR7703972	SRR7703973	SRR7703962
SRR7703963	SRR7724848	SRR7724847	SRR7724846	SRR7724845	SRR7724852	SRR7724851	SRR7724850
SRR7724849	SRR7724855	SRR7724854	SRR7724894	SRR7724893	SRR7724896	SRR7724895	SRR7724898
SRR7724897	SRR7724900	SRR7724899	SRR7724902	SRR7724901	SRR7724861	SRR7724862	SRR7724863
SRR7724864	SRR7724857	SRR7724858	SRR7724859	SRR7724860	SRR7724869	SRR7724870	SRR7724904
SRR7724903	SRR7724867	SRR7724924	SRR7724908	SRR7724907	SRR7724906	SRR7724905	SRR7724923
SRR7724921	SRR7724871	SRR7724872	SRR7724884	SRR7724885	SRR7724873	SRR7724868	SRR7724865
SRR7724866	SRR7724853	SRR7724856	SRR7724918	SRR7724892	SRR7724888	SRR7724886	SRR7724922
SRR7724891	SRR7724879	SRR7724889	SRR7724925	SRR7724887	SRR7724890	SRR7724874	SRR7724875
SRR7724876	SRR7724877	SRR7724878	SRR7724917	SRR7724880	SRR7724881	SRR7724882	SRR7724916
SRR7724915	SRR7724914	SRR7724913	SRR7724912	SRR7724911	SRR7724910	SRR7724909	SRR7724920
SRR7724919	SRR7724883	SRR7779832	SRR7779831	SRR7779834	SRR7779833	SRR7779836	SRR7779835
SRR7779838	SRR7779837	SRR7779830	SRR7762350	SRR7762351	SRR7762348	SRR7762349	SRR7762346
SRR7762347	SRR7762344	SRR7762345	SRR7762342	SRR7762343	SRR7762340	SRR7762341	SRR7763659
SRR7763658	SRR7763657	SRR7763656	SRR7763663	SRR7763655	SRR7763661	SRR7763660	SRR7763665
SRR7763664	SRR7763668	SRR7763667	SRR7763670	SRR7763669	SRR7763672	SRR7763671	SRR7763674
SRR7763673	SRR7763676	SRR7763675	SRR7763629	SRR7763630	SRR7763631	SRR7763632	SRR7763625
SRR7763626	SRR7763627	SRR7763628	SRR7763637	SRR7763638	SRR7763678	SRR7763677	SRR7763691
SRR7763687	SRR7763682	SRR7763681	SRR7763680	SRR7763679	SRR7763662	SRR7763615	SRR7763639
SRR7763640	SRR7763618	SRR7763620	SRR7763635	SRR7763636	SRR7763633	SRR7763634	SRR7763616
SRR7763617	SRR7763623	SRR7763619	SRR7763624	SRR7763622	SRR7763646	SRR7763621	SRR7763614
SRR7763613	SRR7763653	SRR7763666	SRR7763641	SRR7763642	SRR7763643	SRR7763644	SRR7763645
SRR7763654	SRR7763647	SRR7763648	SRR7763649	SRR7763650	SRR7763690	SRR7763689	SRR7763688
SRR7763652	SRR7763686	SRR7763685	SRR7763684	SRR7763683	SRR7763693	SRR7763692	SRR7763651
SRR7800931	SRR7800930	SRR7800933	SRR7800932	SRR7790713	SRR7790714	SRR7777943	SRR7777942
SRR7777941	SRR7777940	SRR7777947	SRR7777946	SRR7777945	SRR7777944	SRR7777949	SRR7777948
SRR7777939	SRR7777938	SRR8613656	SRR8613657	SRR8613654	SRR8613655	SRR8613660	SRR8613661
SRR8613658	SRR8613659	SRR8613662	SRR8613663	SRR8613666	SRR8613667	SRR8613664	SRR8613665
SRR8613670	SRR8613671	SRR8613668	SRR8613669	SRR7778990	SRR7778987	SRR7778986	SRR7778985
SRR7778984	SRR7778983	SRR7778982	SRR7778981	SRR7778980	SRR7778979	SRR7778978	SRR7778977
SRR7778976	SRR7778975	SRR7778974	SRR7778973	SRR7778972	SRR7778971	SRR7778970	SRR7778969
SRR7778968	SRR7778967	SRR7778966	SRR7778965	SRR7778964	SRR7778963	SRR7778992	SRR7778991
SRR7778989	SRR7778988	SRR7778994	SRR7778993	SRR7779792	SRR7779793	SRR7779794	SRR7779795
SRR7779788	SRR7779789	SRR7779790	SRR7779791	SRR7779786	SRR7779787	SRR7779797	SRR7779798
SRR7779799	SRR7779800	SRR7779801	SRR7779802	SRR7779803	SRR7779804	SRR7779796	SRR7786323
SRR7786322	SRR7786321	SRR7786320	SRR7786319	SRR7786318	SRR7786317	SRR7786316	SRR7786315

SRR7786314	SRR7786313	SRR7786312	SRR7786311	SRR7786310	SRR7786309	SRR7786308	SRR7786307
SRR7786306	SRR7786305	SRR7786304	SRR7786303	SRR7786302	SRR7786301	SRR7786300	SRR7786299
SRR7786298	SRR7786329	SRR7786328	SRR7786327	SRR7786326	SRR7786325	SRR7786324	SRR7824853
SRR7824854	SRR7824855	SRR7824856	SRR7824857	SRR7824858	SRR7824859	SRR7824860	SRR7824861
SRR7876072	SRR7876073	SRR7876074	SRR7876075	SRR7876076	SRR7876077	SRR7876078	SRR7876079
SRR7876080	SRR7876081	SRR7876116	SRR7876117	SRR7876118	SRR7876119	SRR7876112	SRR7876113
SRR7876114	SRR7876115	SRR7876123	SRR7876124	SRR7876083	SRR7876082	SRR7876085	SRR7876084
SRR7876087	SRR7876086	SRR7876089	SRR7876088	SRR7876091	SRR7876090	SRR7876109	SRR7876110
SRR7876106	SRR7876107	SRR7876120	SRR7876125	SRR7876111	SRR7876108	SRR7876121	SRR7876122
SRR7876103	SRR7876102	SRR7876101	SRR7876100	SRR7876099	SRR7876098	SRR7876097	SRR7876096
SRR7876105	SRR7876104	SRR7876126	SRR7876127	SRR7876128	SRR7876129	SRR7876130	SRR7876131
SRR7876132	SRR7876133	SRR7876134	SRR7876135	SRR7876093	SRR7876092	SRR7876095	SRR7876094
SRR7851669	SRR7851668	SRR7851667	SRR7851666	SRR7851665	SRR7851664	SRR7851663	SRR7851662
SRR7851661	SRR7851660	SRR7851659	SRR7851658	SRR7947396	SRR7947397	SRR7947398	SRR7947399
SRR7947400	SRR7947395	SRR7903788	SRR7903789	SRR7903790	SRR7903784	SRR7903785	SRR7903791
SRR7903786	SRR7903787	SRR7903792	SRR7903770	SRR7903771	SRR7903793	SRR7903772	SRR7903773
SRR7903774	SRR7903775	SRR7903776	SRR7903777	SRR7903778	SRR7903779	SRR7903781	SRR7963450
SRR7963451	SRR7963452	SRR7963453	SRR7963454	SRR7963455	SRR7963456	SRR7963457	SRR7963458
SRR7963459	SRR7963460	SRR7963461	SRR7963462	SRR7963463	SRR7963464	SRR7963465	SRR7963466
SRR7963467	SRR7963468	SRR7963469	SRR7963470	SRR7963471	SRR7963472	SRR7963473	SRR7963474
SRR7967788	SRR7967799	SRR7967798	SRR7967797	SRR7967796	SRR7967795	SRR7967794	SRR7967793
SRR7967792	SRR7967791	SRR7967790	SRR7967789	SRR8039475	SRR8039477	SRR8039479	SRR8039474
SRR8039476	SRR8039478	SRR8052073	SRR8052072	SRR8052075	SRR8052074	SRR8052077	SRR8052076
SRR8052079	SRR8052078	SRR8052081	SRR8052080	SRR8052085	SRR8052084	SRR8052083	SRR8052082
SRR8052627	SRR8052626	SRR8052633	SRR8052630	SRR8052623	SRR8052622	SRR8052625	SRR8052624
SRR8052632	SRR8052631	SRR8052629	SRR8052628	SRR8117289	SRR8117290	SRR8117291	SRR8117292
SRR8117285	SRR8117286	SRR8117287	SRR8117288	SRR8117293	SRR8141428	SRR8141427	SRR8141426
SRR8141425	SRR8141424	SRR8141423	SRR8141422	SRR8141421	SRR8141420	SRR8141419	SRR8141418
SRR8141417	SRR8141416	SRR8141447	SRR8141446	SRR8141445	SRR8141444	SRR8141443	SRR8141442
SRR8141441	SRR8141440	SRR8141439	SRR8141438	SRR8141437	SRR8141436	SRR8141435	SRR8141434
SRR8141433	SRR8141432	SRR8141431	SRR8141430	SRR8141429	SRR8141481	SRR8141480	SRR8141479
SRR8141478	SRR8141477	SRR8141476	SRR8141475	SRR8141474	SRR8141473	SRR8173553	SRR8173552
SRR8173555	SRR8173554	SRR8173549	SRR8173548	SRR8177147	SRR8177146	SRR8177145	SRR8177160
SRR8177159	SRR8177158	SRR8177157	SRR8177156	SRR8177155	SRR8177154	SRR8177153	SRR8177152
SRR8177151	SRR8177150	SRR8177149	SRR8177148	SRR8177215	SRR8177214	SRR8177213	SRR8177212
SRR8177211	SRR8177210	SRR8177209	SRR8177208	SRR8177207	SRR8177206	SRR8177205	SRR8177204
SRR8177203	SRR8177202	SRR8177201	SRR8177200	SRR8187122	SRR8187121	SRR8187120	SRR8187119
SRR8187118	SRR8187117	SRR8187116	SRR8187115	SRR8187114	SRR8194961	SRR8194962	SRR8194963
SRR8194964	SRR8194965	SRR8194966	SRR8217162	SRR8217177	SRR8217176	SRR8217175	SRR8217174
SRR8217173	SRR8217172	SRR8217171	SRR8217170	SRR8217169	SRR8217168	SRR8217167	SRR8217166
SRR8217165	SRR8217164	SRR8217163	SRR8217148	SRR8217147	SRR8217146	SRR8217145	SRR8217144
SRR8217143	SRR8217161	SRR8217160	SRR8217159	SRR8217158	SRR8217157	SRR8217156	SRR8217155
SRR8217154	SRR8217153	SRR8217152	SRR8217151	SRR8217150	SRR8217149	SRR8224437	SRR8224436
SRR8224439	SRR8224438	SRR8224433	SRR8224432	SRR8224435	SRR8224434	SRR8224431	SRR8224430
SRR8224429	SRR8224428	SRR8237164	SRR8237163	SRR8237162	SRR8237161	SRR8237167	SRR8237166
SRR8237165	SRR8247557	SRR8247556	SRR8247555	SRR8247554	SRR8247553	SRR8247552	SRR8271213
SRR8271214	SRR8271215	SRR8271216	SRR8271209	SRR8271210	SRR8271211	SRR8271212	SRR8271544
SRR8271545	SRR8271542	SRR8271543	SRR8271540	SRR8271541	SRR8271538	SRR8271539	SRR8272598
SRR8272599	SRR8272596	SRR8272597	SRR8272594	SRR8272595	SRR8272592	SRR8272593	SRR8283957
SRR8283956	SRR8283955	SRR8283954	SRR8283953	SRR8283952	SRR8283951	SRR8283950	SRR8283958
SRR8310836	SRR8310839	SRR8310838	SRR8310837	SRR8306929	SRR8306930	SRR8306931	SRR8306932
SRR8306933	SRR8306934	SRR8306935	SRR8306936	SRR8306937	SRR8306938	SRR8306939	SRR8306940
SRR8318490	SRR8318489	SRR8318488	SRR8318487	SRR8318486	SRR8318485	SRR8318484	SRR8318483
SRR8318482	SRR8318481	SRR8327870	SRR8327869	SRR8327868	SRR8327867	SRR8327866	SRR8327865
SRR8327864	SRR8327863	SRR8327862	SRR8327861	SRR8366875	SRR8366874	SRR8366873	SRR8366872
SRR8366871	SRR8366870	SRR8366869	SRR8366868	SRR8366908	SRR8366907	SRR8366906	SRR8366905
SRR8366904	SRR8366903	SRR8366902	SRR8366901	SRR8366900	SRR8366899	SRR8366898	SRR8366897
SRR8366896	SRR8366895	SRR8366894	SRR8366893	SRR8366892	SRR8366891	SRR8366824	SRR8366823

SRR8366822	SRR8366821	SRR8366820	SRR8366819	SRR8366818	SRR8366817	SRR8366816	SRR8366815
SRR8366814	SRR8366813	SRR8366948	SRR8366947	SRR8366946	SRR8366945	SRR8366944	SRR8366943
SRR8366942	SRR8366941	SRR8366940	SRR8366939	SRR8366890	SRR8366889	SRR8366888	SRR8366887
SRR8366886	SRR8366885	SRR8366884	SRR8366883	SRR8366882	SRR8366881	SRR8366880	SRR8366879
SRR8366878	SRR8366877	SRR8366876	SRR8366860	SRR8366859	SRR8366858	SRR8366857	SRR8366856
SRR8366855	SRR8366854	SRR8366853	SRR8366847	SRR8366846	SRR8366852	SRR8366851	SRR8366850
SRR8366849	SRR8366848	SRR8366845	SRR8366844	SRR8366843	SRR8366842	SRR8366841	SRR8366840
SRR8366839	SRR8366838	SRR8366837	SRR8366836	SRR8366835	SRR8366834	SRR8366833	SRR8366832
SRR8366831	SRR8366830	SRR8366829	SRR8366828	SRR8366827	SRR8366826	SRR8366825	SRR8366952
SRR8366951	SRR8366950	SRR8366949	SRR8366938	SRR8366937	SRR8366936	SRR8366935	SRR8366934
SRR8366933	SRR8366932	SRR8366931	SRR8366930	SRR8366929	SRR8366928	SRR8366927	SRR8366926
SRR8366925	SRR8366924	SRR8366923	SRR8366922	SRR8388925	SRR8366921	SRR8366920	SRR8366915
SRR8366914	SRR8366919	SRR8366918	SRR8366917	SRR8366916	SRR8366913	SRR8366912	SRR8366911
SRR8366910	SRR8366909	SRR8366867	SRR8366866	SRR8366865	SRR8366864	SRR8366863	SRR8366862
SRR8366861	SRR8366799	SRR8366793	SRR8366798	SRR8366797	SRR8366796	SRR8366795	SRR8366794
SRR8366792	SRR8366791	SRR8366790	SRR8366789	SRR8366788	SRR8366787	SRR8366786	SRR8366785
SRR8366784	SRR8366783	SRR8366782	SRR8366781	SRR8366780	SRR8366779	SRR8366778	SRR8366777
SRR8366776	SRR8366775	SRR8366774	SRR8366773	SRR8366772	SRR8366771	SRR8366770	SRR8366769
SRR8366768	SRR8366767	SRR8366766	SRR8366765	SRR8366764	SRR8366812	SRR8366811	SRR8366810
SRR8366809	SRR8366808	SRR8366807	SRR8366806	SRR8366805	SRR8366804	SRR8366803	SRR8366802
SRR8366801	SRR8366800	SRR8379829	SRR8379834	SRR8379833	SRR8379832	SRR8379831	SRR8379830
SRR8392872	SRR8392871	SRR8392870	SRR8392869	SRR8392868	SRR8392867	SRR8392866	SRR8392865
SRR8392864	SRR8392863	SRR8392862	SRR8392861	SRR8392860	SRR8392859	SRR8392858	SRR8392857
SRR8392856	SRR8392855	SRR8392854	SRR8392853	SRR8417802	SRR8417801	SRR8417800	SRR8417799
SRR8417798	SRR8417797	SRR8417796	SRR8417795	SRR8417793	SRR8417794	SRR8417791	SRR8417792
SRR8417790	SRR8417788	SRR8417789	SRR8417786	SRR8417787	SRR8417784	SRR8417785	SRR8417782
SRR8417783	SRR8417780	SRR8417781	SRR8417778	SRR8417779	SRR8417776	SRR8417777	SRR8417774
SRR8417775	SRR8417772	SRR8417773	SRR8417770	SRR8417771	SRR8417768	SRR8417769	SRR8417766
SRR8417767	SRR8417764	SRR8417765	SRR8417762	SRR8417763	SRR8417761	SRR8417759	SRR8417760
SRR8417757	SRR8417758	SRR8417755	SRR8417756	SRR8417753	SRR8417754	SRR8417751	SRR8417752
SRR8417749	SRR8417750	SRR8417747	SRR8417748	SRR8417745	SRR8417746	SRR8417743	SRR8417744
SRR8417741	SRR8417742	SRR8417740	SRR8417739	SRR8417737	SRR8417738	SRR8417735	SRR8417736
SRR8417733	SRR8417734	SRR8417731	SRR8417732	SRR8417730	SRR8417729	SRR8417727	SRR8417728
SRR8417726	SRR8417725	SRR8417724	SRR8417723	SRR8417722	SRR8417721	SRR8417720	SRR8417719
SRR8417718	SRR8417717	SRR8417803	SRR8421360	SRR8421361	SRR8421362	SRR8421363	SRR8421355
SRR8421364	SRR8421353	SRR8421354	SRR8421351	SRR8421352	SRR8421356	SRR8421357	SRR8421358
SRR8421359	SRR8453478	SRR8453477	SRR8453476	SRR8453475	SRR8453480	SRR8453479	SRR8471345
SRR8471344	SRR8471339	SRR8471346	SRR8471341	SRR8471340	SRR8471343	SRR8471342	SRR8471338
SRR8478238	SRR8478243	SRR8478242	SRR8478241	SRR8478240	SRR8478239	SRR8492018	SRR8492040
SRR8492039	SRR8492038	SRR8492037	SRR8492036	SRR8492035	SRR8492034	SRR8492033	SRR8492032
SRR8492031	SRR8492030	SRR8492029	SRR8492028	SRR8492027	SRR8492026	SRR8492025	SRR8492024
SRR8492023	SRR8492022	SRR8492021	SRR8492020	SRR8492019	SRR8510414	SRR8510415	SRR8510418
SRR8510423	SRR8510426	SRR8510420	SRR8510424	SRR8510425	SRR8510421	SRR8510422	SRR8510410
SRR8510411	SRR8510412	SRR8510413	SRR8510427	SRR8510419	SRR8510416	SRR8510417	SRR8529787
SRR8529788	SRR8529785	SRR8529786	SRR8529791	SRR8529792	SRR8529789	SRR8529790	SRR8529793
SRR8529794	SRR8889061	SRR8889114	SRR8889113	SRR8889112	SRR8889111	SRR8889110	SRR8889109
SRR8889108	SRR8889063	SRR8889062	SRR8889075	SRR8889074	SRR8889077	SRR8889076	SRR8889071
SRR8889070	SRR8889073	SRR8889072	SRR8889069	SRR8889068	SRR8889098	SRR8889099	SRR8889100
SRR8889101	SRR8889102	SRR8889103	SRR8889104	SRR8889105	SRR8889106	SRR8889107	SRR8539527
SRR8539528	SRR8539525	SRR8539526	SRR8539523	SRR8539524	SRR8539521	SRR8539522	SRR8539520
SRR8539519	SRR8539517	SRR8539518	SRR8539515	SRR8539516	SRR8539513	SRR8539514	SRR8539511
SRR8539512	SRR8539507	SRR8539508	SRR8539505	SRR8539506	SRR8539509	SRR8539510	SRR8539503
SRR8539504	SRR8573570	SRR8573571	SRR8573572	SRR8573573	SRR8573574	SRR8573575	SRR8573576
SRR8573577	SRR8573568	SRR8573569	SRR8573567	SRR8573566	SRR8600346	SRR8600345	SRR8600344
SRR8600343	SRR8600350	SRR8600349	SRR8600348	SRR8600347	SRR8601588	SRR8601587	SRR8601586
SRR8601585	SRR8601592	SRR8601591	SRR8601590	SRR8601589	SRR8601584	SRR8601583	SRR8601594
SRR8601593	SRR8631871	SRR8631870	SRR8631869	SRR8631868	SRR8631867	SRR8631866	SRR8648494
SRR8648493	SRR8648496	SRR8648495	SRR8648490	SRR8648489	SRR8648492	SRR8648491	SRR8648488

SRR8648487	SRR8648502	SRR8648501	SRR8648500	SRR8648499	SRR8648498	SRR8648497	SRR8654743
SRR8654744	SRR8654745	SRR8654746	SRR8654747	SRR8654748	SRR8728056	SRR8728057	SRR8728054
SRR8728055	SRR8728058	SRR8728052	SRR8728051	SRR8728050	SRR8728049	SRR8728053	SRR8728060
SRR8728059	SRR8749613	SRR8749612	SRR8749611	SRR8749610	SRR8749609	SRR8749608	SRR8749607
SRR8749606	SRR8749605	SRR8749604	SRR8749619	SRR8749618	SRR8749621	SRR8749620	SRR8749615
SRR8749614	SRR8749617	SRR8749616	SRR8749623	SRR8749622	SRR8749596	SRR8749597	SRR8749598
SRR8749599	SRR8749600	SRR8749601	SRR8749602	SRR8749603	SRR8768400	SRR8768399	SRR8768398
SRR8768397	SRR8768404	SRR8768403	SRR8768402	SRR8768401	SRR8768406	SRR8768405	SRR8768388
SRR8768387	SRR8768390	SRR8768389	SRR8768392	SRR8768391	SRR8768394	SRR8768393	SRR8768396
SRR8768395	SRR8768411	SRR8768412	SRR8768413	SRR8768414	SRR8768407	SRR8768408	SRR8768409
SRR8768410	SRR8768415	SRR8768416	SRR8778536	SRR8778537	SRR8778538	SRR8778539	SRR8778532
SRR8778533	SRR8778534	SRR8778535	SRR8778530	SRR8778531	SRR8778522	SRR8778523	SRR8778524
SRR8778525	SRR8778526	SRR8778527	SRR8778528	SRR8778529	SRR8778520	SRR8778521	SRR8778545
SRR8778544	SRR8778547	SRR8778546	SRR8778541	SRR8778540	SRR8778543	SRR8778542	SRR8778549
SRR8778548	SRR8787459	SRR8787458	SRR8787457	SRR8787456	SRR8787455	SRR8787454	SRR8787453
SRR8787452	SRR8787461	SRR8787460	SRR8787449	SRR8787448	SRR8787451	SRR8787450	SRR8787445
SRR8787444	SRR8787447	SRR8787446	SRR8787443	SRR8787442	SRR8787462	SRR8787463	SRR8787464
SRR8787465	SRR8787466	SRR8787467	SRR8787468	SRR8787469	SRR8787470	SRR8787471	SRR8792416
SRR8792415	SRR8792414	SRR8792421	SRR8792413	SRR8792424	SRR8792423	SRR8792422	SRR8792420
SRR8792419	SRR8792418	SRR8792417	SRR8799725	SRR8799726	SRR8799723	SRR8799724	SRR8799721
SRR8799722	SRR8799719	SRR8799720	SRR8799717	SRR8799718	SRR8799713	SRR8799714	SRR8799711
SRR8799712	SRR8799709	SRR8799710	SRR8799707	SRR8799708	SRR8799715	SRR8799716	SRR8799736
SRR8799735	SRR8799734	SRR8799733	SRR8799732	SRR8799731	SRR8799730	SRR8799729	SRR8799728
SRR8799727	SRR8894173	SRR8894174	SRR8894175	SRR8894176	SRR8894177	SRR8894178	SRR8844238
SRR8844237	SRR8844236	SRR8844235	SRR8844234	SRR8844233	SRR8844232	SRR8844231	SRR8844230
SRR8844229	SRR8844228	SRR8844227	SRR8844226	SRR8844225	SRR8844239	SRR8844252	SRR8844266
SRR8844265	SRR8844264	SRR8844263	SRR8844262	SRR8844261	SRR8844260	SRR8844259	SRR8844258
SRR8844257	SRR8844256	SRR8844255	SRR8844254	SRR8844253	SRR8844251	SRR8844250	SRR8844249
SRR8844248	SRR8844247	SRR8844246	SRR8844245	SRR8844244	SRR8844243	SRR8844242	SRR8844241
SRR8844240	SRR17775329	SRR17775328	SRR17775285	SRR17775274	SRR17775263	SRR17775316	SRR17775305
SRR17775294	SRR17775251	SRR17775250	SRR17775327	SRR17775326	SRR17775293	SRR17775292	SRR17775291
SRR17775290	SRR17775289	SRR17775288	SRR17775287	SRR17775286	SRR17775284	SRR17775283	SRR17775282
SRR17775281	SRR17775280	SRR17775279	SRR17775278	SRR17775277	SRR17775276	SRR17775275	SRR17775273
SRR17775272	SRR17775271	SRR17775270	SRR17775269	SRR17775268	SRR17775267	SRR17775266	SRR17775265
SRR17775264	SRR17775262	SRR17775325	SRR17775324	SRR17775323	SRR17775322	SRR17775321	SRR17775320
SRR17775319	SRR17775318	SRR17775317	SRR17775315	SRR17775314	SRR17775313	SRR17775312	SRR17775311
SRR17775310	SRR17775309	SRR17775308	SRR17775307	SRR17775306	SRR17775304	SRR17775303	SRR17775302
SRR17775301	SRR17775300	SRR17775299	SRR17775298	SRR17775297	SRR17775296	SRR17775295	SRR17775261
SRR17775260	SRR17775259	SRR17775258	SRR17775257	SRR17775256	SRR17775255	SRR17775254	SRR17775253
SRR17775252	SRR19749979	SRR19749978	SRR19749970	SRR19749969	SRR19749968	SRR19749967	SRR19749966
SRR19749965	SRR19749964	SRR19749963	SRR19749977	SRR19749976	SRR19749975	SRR19749974	SRR19749973
SRR19749972	SRR19749971	SRR12805717	SRR12805724	SRR12805716	SRR12805723	SRR12805715	SRR12805719
SRR12805711	SRR12805728	SRR12805726	SRR12805727	SRR12805718	SRR12805725	SRR12805714	SRR12805722
SRR12805713	SRR12805721	SRR12805712	SRR12805720	SRR8885276	SRR8885266	SRR8885275	SRR8885274
SRR8885273	SRR8885272	SRR8885271	SRR8885270	SRR8885269	SRR8885268	SRR8885267	SRR8885277
SRR8921156	SRR8921157	SRR8921154	SRR8921155	SRR8921152	SRR8921153	SRR8925552	SRR8925551
SRR8925550	SRR8925549	SRR8925548	SRR8925547	SRR8931967	SRR8931968	SRR8931965	SRR8931966
SRR8931963	SRR8931964	SRR8948523	SRR8948522	SRR8948525	SRR8948524	SRR8948527	SRR8948526
SRR8948547	SRR8948546	SRR8948545	SRR8948544	SRR8948543	SRR8948542	SRR9030205	SRR9030206
SRR9030204	SRR9114668	SRR9114669	SRR9114670	SRR9114671	SRR9114672	SRR9114673	SRR9114674
SRR9114675	SRR9114676	SRR9114677	SRR9114678	SRR9114679	SRR9107322	SRR9107321	SRR9107320
SRR9137209	SRR9137208	SRR9137211	SRR9137205	SRR9137213	SRR9137212	SRR9137215	SRR9137214
SRR9137206	SRR9137207	SRR9137219	SRR9137218	SRR9137217	SRR9137216	SRR9137223	SRR9137222
SRR9137221	SRR9137220	SRR9137225	SRR9137224	SRR9137210	SRR9178049	SRR9178048	SRR9178051
SRR9178050	SRR9178065	SRR9178064	SRR9190120	SRR9190119	SRR9190118	SRR9190117	SRR9190116
SRR9190115	SRR9190114	SRR9190113	SRR9190112	SRR9190111	SRR9190106	SRR9190105	SRR9190104
SRR9190110	SRR9190109	SRR9190108	SRR9190107	SRR9190103	SRR9190102	SRR9190101	SRR9190100
SRR9190099	SRR9190098	SRR9190097	SRR9190096	SRR9190095	SRR12564393	SRR12564392	SRR12564391

SRR12564390	SRR12564389	SRR12564388	SRR12564387	SRR12564386	SRR12564385	SRR12564384	SRR12564395
SRR12564394	SRR9265666	SRR9265665	SRR9265664	SRR9265663	SRR9265658	SRR9265662	SRR9265661
SRR9265660	SRR9265659	SRR9265657	SRR9265656	SRR9265655	SRR9265670	SRR9265669	SRR9265668
SRR9265667	SRR9282918	SRR9282917	SRR9282916	SRR9282915	SRR9282914	SRR9282913	SRR9302356
SRR9302355	SRR9302354	SRR9302353	SRR9302352	SRR9302351	SRR9309021	SRR9309022	SRR9309019
SRR9308999	SRR9309000	SRR9309007	SRR9309008	SRR9309009	SRR9309010	SRR9309001	SRR9309015
SRR9309016	SRR9309013	SRR9309014	SRR9309017	SRR9309006	SRR9326114	SRR9326113	SRR9326112
SRR9326111	SRR9326110	SRR9326109	SRR9613393	SRR9613394	SRR9613395	SRR9613396	SRR9613391
SRR9613392	SRR9621331	SRR9621330	SRR9621329	SRR9621328	SRR9621327	SRR9621326	SRR9621325
SRR9621324	SRR9621323	SRR9621322	SRR9621321	SRR9621320	SRR9621319	SRR9621318	SRR9621317
SRR9621316	SRR9616600	SRR9616601	SRR9616598	SRR9616599	SRR9616602	SRR9616603	SRR9643863
SRR9643862	SRR9643865	SRR9643864	SRR9643867	SRR9643866	SRR9627725	SRR9627724	SRR9627723
SRR9627722	SRR9627721	SRR9627720	SRR9627717	SRR9627716	SRR9627715	SRR9627719	SRR9627718
SRR9654583	SRR9654584	SRR9654579	SRR9654580	SRR9654581	SRR9654582	SRR9657895	SRR9657894
SRR9657893	SRR9657892	SRR9657891	SRR9657890	SRR9657889	SRR9657888	SRR9657887	SRR9657886
SRR9657885	SRR9657884	SRR9657883	SRR9657882	SRR9657881	SRR9657880	SRR9673741	SRR9673742
SRR9673743	SRR9673744	SRR9673745	SRR9673746	SRR9673266	SRR9673267	SRR9673268	SRR9673269
SRR9673264	SRR9673265	SRR9668629	SRR9668628	SRR9668627	SRR9668626	SRR9668633	SRR9668632
SRR9668631	SRR9668630	SRR9668625	SRR9668622	SRR9668623	SRR9668624	SRR9669918	SRR9669917
SRR9669916	SRR9669942	SRR9669941	SRR9669940	SRR9669939	SRR9669938	SRR9669937	SRR9669936
SRR9669935	SRR9669934	SRR9669933	SRR9669932	SRR9669931	SRR9669930	SRR9669929	SRR9669928
SRR9669927	SRR9669926	SRR9669925	SRR9669924	SRR9669923	SRR9669922	SRR9669920	SRR9669919
SRR9669921	SRR9669955	SRR9669954	SRR9669953	SRR9669952	SRR9669951	SRR9669950	SRR9669949
SRR9669948	SRR9669947	SRR9669946	SRR9669945	SRR9669944	SRR9669943	SRR9669976	SRR9669975
SRR9669974	SRR9669972	SRR9669971	SRR9669973	SRR9669979	SRR9669978	SRR9669977	SRR9672896
SRR9672897	SRR9672898	SRR9672899	SRR9672900	SRR9672901	SRR9672902	SRR9672903	SRR9672895
SRR9695949	SRR9695950	SRR9695951	SRR9695952	SRR9695953	SRR9695954	SRR9729078	SRR9729079
SRR9729080	SRR9729081	SRR9729082	SRR9729083	SRR9735471	SRR9735470	SRR9735469	SRR9735468
SRR9735467	SRR9735466	SRR9735465	SRR9735464	SRR9735463	SRR9735462	SRR9735461	SRR9735460
SRR9735459	SRR9735458	SRR9735457	SRR9735456	SRR9735455	SRR9735454	SRR9735477	SRR9735476
SRR9735475	SRR9735474	SRR9735473	SRR9735472	SRR9829616	SRR9829617	SRR9829614	SRR9829615
SRR9829612	SRR9829613	SRR9829610	SRR9829611	SRR9829609	SRR9843080	SRR9849771	SRR9849772
SRR9945710	SRR9945711	SRR9945712	SRR9945713	SRR9945714	SRR9945715	SRR9909338	SRR9909337
SRR9909336	SRR9943945	SRR9943946	SRR9943947	SRR9943948	SRR9943949	SRR9943950	SRR10034591
SRR10034590	SRR10034589	SRR10034588	SRR10034587	SRR10034586	SRR10034673	SRR10034672	SRR10034671
SRR10034670	SRR10034669	SRR10034668	SRR10053323	SRR10053322	SRR10053321	SRR10053320	SRR10053319
SRR10053318	SRR10059417	SRR10059416	SRR10059415	SRR10059414	SRR10059413	SRR10059412	SRR10059411
SRR10059410	SRR10059409	SRR10059408	SRR10059407	SRR10061079	SRR10064651	SRR10064706	SRR10064746
SRR10071298	SRR10071297	SRR10071296	SRR10071295	SRR10071294	SRR10071293	SRR10071292	SRR10071291
SRR10071290	SRR10071289	SRR10071288	SRR10088582	SRR10088589	SRR10088588	SRR10088587	SRR10088586
SRR10088585	SRR10088584	SRR10088583	SRR10095873	SRR10095872	SRR10095865	SRR10095864	SRR10095863
SRR10095862	SRR10095861	SRR10095860	SRR10095859	SRR10095858	SRR10095871	SRR10095870	SRR10095869
SRR10095868	SRR10095867	SRR10095866	SRR10100268	SRR10100283	SRR10100282	SRR10100281	SRR10100280
SRR10100279	SRR10100278	SRR10100277	SRR10100276	SRR10100275	SRR10100274	SRR10100273	SRR10100272
SRR10100271	SRR10100270	SRR10100269	SRR10159669	SRR10159668	SRR10159657	SRR10159646	SRR10159635
SRR10159624	SRR10159616	SRR10159615	SRR10159614	SRR10159613	SRR10159667	SRR10159666	SRR10159665
SRR10159664	SRR10159663	SRR10159662	SRR10159661	SRR10159660	SRR10159659	SRR10159658	SRR10159656
SRR10159655	SRR10159654	SRR10159653	SRR10159652	SRR10159651	SRR10159650	SRR10159649	SRR10159648
SRR10159647	SRR10159645	SRR10159644	SRR10159643	SRR10159642	SRR10159641	SRR10159640	SRR10159639
SRR10159638	SRR10159637	SRR10159636	SRR10159634	SRR10159633	SRR10159632	SRR10159631	SRR10159630
SRR10159629	SRR10159628	SRR10159627	SRR10159626	SRR10159625	SRR10159623	SRR10159622	SRR10159621
SRR10159620	SRR10159619	SRR10159618	SRR10159617	SRR10230888	SRR10230887	SRR10230886	SRR10230885
SRR10230884	SRR10230883	SRR10230882	SRR10230881	SRR10230880	SRR10230879	SRR10230893	SRR10230892
SRR10230891	SRR10230890	SRR10230889	SRR10250952	SRR10250950	SRR10250951	SRR10250949	SRR10250957
SRR10250956	SRR10250955	SRR10250954	SRR10250953	SRR10258270	SRR10258269	SRR10258258	SRR10258247
SRR10258241	SRR10258240	SRR10258239	SRR10258238	SRR10258237	SRR10258236	SRR10258268	SRR10258267
SRR10258266	SRR10258265	SRR10258264	SRR10258263	SRR10258262	SRR10258261	SRR10258260	SRR10258259
SRR10258257	SRR10258256	SRR10258255	SRR10258254	SRR10258253	SRR10258252	SRR10258251	SRR10258250

SRR10258249	SRR10258248	SRR10258246	SRR10258245	SRR10258244	SRR10258243	SRR10258242	SRR10402457
SRR10402468	SRR10402471	SRR10402474	SRR10402475	SRR10402476	SRR10402480	SRR10402481	SRR10402482
SRR10402484	SRR10402455	SRR10402470	SRR10402479	SRR10402461	SRR10402483	SRR10402463	SRR10402472
SRR10402477	SRR10402469	SRR10402473	SRR10402460	SRR10402478	SRR10402459	SRR10402462	SRR10402458
SRR10402465	SRR10402456	SRR10402464	SRR10402467	SRR10402466	SRR10526967	SRR10526974	SRR10526976
SRR10526991	SRR10526965	SRR10526969	SRR10526972	SRR10526975	SRR10526977	SRR10526978	SRR10526983
SRR10526985	SRR10526986	SRR10526990	SRR10526966	SRR10526984	SRR10526989	SRR10526971	SRR10526982
SRR10526987	SRR10526979	SRR10526980	SRR10526973	SRR10526992	SRR10526964	SRR10526993	SRR10526963
SRR10526970	SRR10526988	SRR10526981	SRR10526968	SRR10548537	SRR10548529	SRR10548543	SRR10548542
SRR10548531	SRR10548536	SRR10548535	SRR10548545	SRR10548546	SRR10548539	SRR10548548	SRR10548544
SRR10548540	SRR10548541	SRR10548547	SRR10548530	SRR10548532	SRR10548533	SRR10548534	SRR10548538
SRR10419113	SRR10419111	SRR10419110	SRR10419109	SRR10419108	SRR10419112	SRR10305486	SRR10305485
SRR10305484	SRR10566883	SRR10566882	SRR10566881	SRR10315370	SRR10315369	SRR10315366	SRR10315365
SRR10315364	SRR10315363	SRR10315362	SRR10315361	SRR10315360	SRR10315359	SRR10315368	SRR10315367
SRR10341066	SRR10341067	SRR10341064	SRR10341065	SRR10341062	SRR10341063	SRR10341060	SRR10341061
SRR10341058	SRR10341059	SRR10341056	SRR10341057	SRR10343021	SRR10343020	SRR10343019	SRR10343058
SRR10343057	SRR10343056	SRR10343055	SRR10343054	SRR10343053	SRR10343052	SRR10343051	SRR10343050
SRR10343049	SRR10343040	SRR10343039	SRR10343038	SRR10343037	SRR10343036	SRR10343035	SRR10343034
SRR10343033	SRR10343032	SRR10343031	SRR10343030	SRR10343029	SRR10343028	SRR10343027	SRR10343026
SRR10343025	SRR10343024	SRR10343023	SRR10343022	SRR10343048	SRR10343047	SRR10343046	SRR10343045
SRR10343044	SRR10343043	SRR10343042	SRR10343041	SRR12442756	SRR12442755	SRR12442754	SRR12442753
SRR12442752	SRR12442751	SRR12442750	SRR12442749	SRR12442748	SRR12442746	SRR12442745	SRR12442744
SRR10446888	SRR10446887	SRR10446886	SRR10446885	SRR10446884	SRR10446883	SRR10446882	SRR10446881
SRR10446880	SRR10446908	SRR10446907	SRR10446906	SRR10446905	SRR10446904	SRR10446903	SRR10462014
SRR10462013	SRR10462012	SRR10462011	SRR10462010	SRR10462009	SRR10462008	SRR10462007	SRR10462006
SRR10462005	SRR10462004	SRR10462002	SRR10462003	SRR10462001	SRR10462000	SRR10461999	SRR10461998
SRR10462032	SRR10462031	SRR10462030	SRR10462029	SRR10462028	SRR10462027	SRR10462026	SRR10462025
SRR10462024	SRR10462022	SRR10462023	SRR10462021	SRR10462019	SRR10462018	SRR10462017	SRR10462016
SRR10462015	SRR10582015	SRR10582029	SRR10582028	SRR10582027	SRR10582026	SRR10582025	SRR10582024
SRR10582023	SRR10582022	SRR10582021	SRR10582020	SRR10582019	SRR10582018	SRR10582017	SRR10582016
SRR10688162	SRR10688161	SRR10688154	SRR10688153	SRR10688152	SRR10688151	SRR10688150	SRR10688149
SRR10688148	SRR10688147	SRR10688160	SRR10688159	SRR10688158	SRR10688157	SRR10597270	SRR10597271
SRR10597268	SRR10597269	SRR10597266	SRR10597267	SRR10597264	SRR10597265	SRR10597262	SRR10597263
SRR10597260	SRR10597261	SRR10597258	SRR10597259	SRR10597256	SRR10597257	SRR10597254	SRR10597255
SRR10597252	SRR10597253	SRR10597250	SRR10597251	SRR10597248	SRR10597249	SRR10597246	SRR10597247
SRR10597244	SRR10597245	SRR10597242	SRR10597243	SRR10597240	SRR10597241	SRR10597238	SRR10597239
SRR10597236	SRR10597237	SRR10597234	SRR10597235	SRR10597232	SRR10597233	SRR10597230	SRR10597231
SRR10598550	SRR10598551	SRR10611296	SRR10611295	SRR10611294	SRR10611293	SRR10611292	SRR10611297
SRR10677622	SRR10677621	SRR10677620	SRR10677619	SRR10677618	SRR10677617	SRR10677616	SRR10677615
SRR10677614	SRR10677613	SRR10677612	SRR10677611	SRR10720618	SRR10720626	SRR10720625	SRR10720624
SRR10720623	SRR10720622	SRR10720621	SRR10720620	SRR10720619	SRR10741270	SRR10741269	SRR10741268
SRR10741267	SRR10741266	SRR10741265	SRR10741264	SRR10741263	SRR10741262	SRR10741261	SRR10741260
SRR10741259	SRR10745663	SRR10745662	SRR10745661	SRR10764577	SRR10764576	SRR10764465	SRR10764654
SRR10764643	SRR10764632	SRR10764621	SRR10764610	SRR10764599	SRR10764588	SRR10764575	SRR10764564
SRR10764553	SRR10764542	SRR10764531	SRR10764520	SRR10764509	SRR10764498	SRR10764487	SRR10764476
SRR10764464	SRR10764453	SRR10764708	SRR10764696	SRR10764685	SRR10764674	SRR10764662	SRR10764657
SRR10764656	SRR10764655	SRR10764653	SRR10764652	SRR10764651	SRR10764650	SRR10764649	SRR10764648
SRR10764646	SRR10764645	SRR10764644	SRR10764647	SRR10764642	SRR10764641	SRR10764640	SRR10764638
SRR10764637	SRR10764636	SRR10764639	SRR10764635	SRR10764634	SRR10764633	SRR10764631	SRR10764630
SRR10769886	SRR10769887	SRR10769888	SRR10769883	SRR10769884	SRR10769885	SRR10799636	SRR10799635
SRR10799618	SRR10799607	SRR10799596	SRR10799631	SRR10799630	SRR10799629	SRR10799628	SRR10799627
SRR10799634	SRR10799633	SRR10799626	SRR10799625	SRR10799624	SRR10799623	SRR10799622	SRR10799621
SRR10799620	SRR10799619	SRR10799617	SRR10799616	SRR10799615	SRR10799614	SRR10799613	SRR10799612
SRR10799611	SRR10799610	SRR10799609	SRR10799608	SRR10799606	SRR10799605	SRR10799604	SRR10799603
SRR10799602	SRR10799601	SRR10799600	SRR10799599	SRR10799598	SRR10799597	SRR10799595	SRR10799632
SRR10821968	SRR10821967	SRR10821956	SRR10821951	SRR10821950	SRR10821949	SRR10821948	SRR10821947
SRR10821946	SRR10821945	SRR10821966	SRR10821965	SRR10821964	SRR10821963	SRR10821962	SRR10821961
SRR10821960	SRR10821959	SRR10821958	SRR10821957	SRR10821955	SRR10821954	SRR10821953	SRR10821952

SRR10822619	SRR10822621	SRR10822624	SRR10822622	SRR10822623	SRR10822620	SRR10829958	SRR10829957
SRR10829954	SRR10829953	SRR10829952	SRR10829951	SRR10829950	SRR10829949	SRR10829948	SRR10829947
SRR10829956	SRR10829955	SRR10850822	SRR10850821	SRR10850820	SRR10850819	SRR10850830	SRR10850828
SRR10850827	SRR10850826	SRR10850825	SRR10850824	SRR10850823	SRR10874314	SRR10874313	SRR10874312
SRR10874311	SRR10899673	SRR10899672	SRR10899661	SRR10899650	SRR10899639	SRR10899638	SRR10899637
SRR10899636	SRR10899635	SRR10899634	SRR10899671	SRR10899670	SRR10899669	SRR10899668	SRR10899667
SRR10899666	SRR10899665	SRR10899664	SRR10899663	SRR10899662	SRR10899660	SRR10899659	SRR10899658
SRR10899657	SRR10899656	SRR10899655	SRR10899654	SRR10899653	SRR10899652	SRR10899651	SRR10899649
SRR10899648	SRR10899647	SRR10899646	SRR10899645	SRR10899644	SRR10899643	SRR10899642	SRR10899641
SRR10899640	SRR10907229	SRR10907228	SRR10907227	SRR10907226	SRR10907225	SRR10909722	SRR10909721
SRR10909720	SRR10909719	SRR10909730	SRR10909729	SRR10909728	SRR10909727	SRR10909726	SRR10909725
SRR10909718	SRR10909717	SRR10909696	SRR10909695	SRR10909694	SRR10909724	SRR10909723	SRR10909708
SRR10909707	SRR10909706	SRR10909705	SRR10909704	SRR10909703	SRR10909702	SRR10909701	SRR10909700
SRR10909699	SRR10909698	SRR10909697	SRR10909716	SRR10909715	SRR10909714	SRR10909713	SRR10909712
SRR10909711	SRR10909710	SRR10909709	SRR10951686	SRR10951687	SRR10951684	SRR10951685	SRR10951682
SRR10951683	SRR10951681	SRR10951680	SRR10951678	SRR10951679	SRR10951676	SRR10951677	SRR10951674
SRR10951675	SRR10951672	SRR10951673	SRR10951670	SRR10951671	SRR10951668	SRR10951669	SRR10951666
SRR10951667	SRR10951665	SRR10951664	SRR10951662	SRR10951663	SRR10951660	SRR10951661	SRR10951659
SRR10951658	SRR10951657	SRR10951656	SRR10951654	SRR10951655	SRR10951652	SRR10951653	SRR10951650
SRR10951651	SRR10951648	SRR10951649	SRR10951647	SRR10951646	SRR10951645	SRR10951644	SRR10951642
SRR10951643	SRR10951640	SRR10951641	SRR10951638	SRR10951639	SRR10998733	SRR10998732	SRR10998729
SRR10998728	SRR10998727	SRR10998726	SRR10998725	SRR10998724	SRR10998723	SRR10998722	SRR10998731
SRR10998730	SRR11020929	SRR11020928	SRR11020940	SRR11020939	SRR11020938	SRR11020932	SRR11020931
SRR11020937	SRR11020936	SRR11020934	SRR11020933	SRR11020930	SRR11039333	SRR11039332	SRR11039331
SRR11039330	SRR11039326	SRR11039325	SRR11039329	SRR11039328	SRR11039327	SRR11039324	SRR11039323
SRR11039322	SRR11039321	SRR11039320	SRR11039319	SRR11039318	SRR11039317	SRR11039316	SRR11039336
SRR11039335	SRR11039334	SRR11040769	SRR11040768	SRR11040767	SRR11040766	SRR11040765	SRR11040764
SRR11040763	SRR11040762	SRR11040761	SRR11040760	SRR11040787	SRR11040786	SRR11040785	SRR11040784
SRR11040783	SRR11040782	SRR11040779	SRR11040778	SRR11040780	SRR11040777	SRR11040776	SRR11040775
SRR11040790	SRR11040789	SRR11040788	SRR11040774	SRR11040773	SRR11040772	SRR11040771	SRR11040770
SRR11091386	SRR11091385	SRR11091384	SRR11091383	SRR11091382	SRR11091381	SRR11091380	SRR11091379
SRR11091378	SRR11119412	SRR11119411	SRR11119410	SRR11119409	SRR11119408	SRR11119416	SRR11119415
SRR11119414	SRR11119413	SRR11147384	SRR11147383	SRR11147382	SRR11147381	SRR11147380	SRR11147379
SRR11147378	SRR11147377	SRR11185090	SRR11185089	SRR11185087	SRR11185088	SRR11185086	SRR11185085
SRR11185084	SRR11185083	SRR11185081	SRR11185082	SRR11185080	SRR11185079	SRR11185077	SRR11185078
SRR11185075	SRR11185076	SRR11185052	SRR11185051	SRR11185050	SRR11185049	SRR11185048	SRR11185047
SRR11185046	SRR11185045	SRR11185056	SRR11185055	SRR11185054	SRR11185053	SRR12887454	SRR12887453
SRR12887452	SRR12887451	SRR12887450	SRR12887449	SRR12887448	SRR12887447	SRR12887446	SRR11197091
SRR11197090	SRR11197087	SRR11197086	SRR11197085	SRR11197084	SRR11197083	SRR11197082	SRR11197081
SRR11197080	SRR11197089	SRR11197088	SRR11210475	SRR11210474	SRR11210465	SRR11210464	SRR11210463
SRR11210462	SRR11210461	SRR11210460	SRR11210459	SRR11210458	SRR11210473	SRR11210472	SRR11210471
SRR11210470	SRR11210469	SRR11210468	SRR11210467	SRR11210466	SRR11243418	SRR11243417	SRR11243416
SRR11243415	SRR11243414	SRR11243413	SRR11243412	SRR11243411	SRR11243410	SRR11243409	SRR11243408
SRR11243407	SRR11243406	SRR11243405	SRR11243404	SRR11243403	SRR11243402	SRR11280709	SRR11280708
SRR11280707	SRR11527008	SRR11526992	SRR11526970	SRR11526948	SRR11526926	SRR11526915	SRR11527006
SRR11527031	SRR11527020	SRR11527009	SRR11526998	SRR11526997	SRR11526996	SRR11526995	SRR11526994
SRR11526993	SRR11526991	SRR11526990	SRR11526989	SRR11526988	SRR11526987	SRR11526986	SRR11526985
SRR11526984	SRR11526983	SRR11526982	SRR11526980	SRR11526979	SRR11526978	SRR11526977	SRR11526976
SRR11526975	SRR11526974	SRR11526973	SRR11526972	SRR11526971	SRR11526969	SRR11526968	SRR11526967
SRR11526966	SRR11526965	SRR11526964	SRR11526963	SRR11526962	SRR11526961	SRR11526960	SRR11526958
SRR11526957	SRR11526955	SRR11526954	SRR11526952	SRR11526951	SRR11526950	SRR11526949	SRR11526947
SRR11526946	SRR11526944	SRR11526943	SRR11526942	SRR11526941	SRR11526940	SRR11526939	SRR11526938
SRR11526936	SRR11526935	SRR11526934	SRR11526932	SRR11526931	SRR11526930	SRR11526929	SRR11526928
SRR11526927	SRR11526925	SRR11526924	SRR11526923	SRR11526922	SRR11526921	SRR11526920	SRR11526919
SRR11526918	SRR11526917	SRR11526916	SRR11526914	SRR11526913	SRR11526912	SRR11526911	SRR11526910
SRR11526909	SRR11526908	SRR11526907	SRR11526906	SRR11526905	SRR11527005	SRR11527040	SRR11527039
SRR11527038	SRR11527037	SRR11527036	SRR11527035	SRR11527034	SRR11527033	SRR11527032	SRR11527030
SRR11527029	SRR11527028	SRR11527027	SRR11527026	SRR11527025	SRR11527024	SRR11527023	SRR11527022

SRR11527021	SRR11527019	SRR11527018	SRR11527017	SRR11527016	SRR11527015	SRR11527014	SRR11527013
SRR11527012	SRR11527011	SRR11527010	SRR11527004	SRR11527003	SRR11527002	SRR11527001	SRR11527000
SRR11526999	SRR11348430	SRR11348429	SRR11348428	SRR11348427	SRR11348426	SRR11348425	SRR11434028
SRR11434295	SRR11434294	SRR11434293	SRR11434292	SRR11434291	SRR11434290	SRR11434289	SRR11434288
SRR11434287	SRR11434286	SRR11434285	SRR11434284	SRR11434071	SRR11434070	SRR11434069	SRR11434068
SRR11434067	SRR11434066	SRR11434065	SRR11434064	SRR11434063	SRR11434062	SRR11434061	SRR11434060
SRR11434059	SRR11434058	SRR11434057	SRR11434047	SRR11434046	SRR11434045	SRR11434044	SRR11434043
SRR11434042	SRR11434041	SRR11434040	SRR11434039	SRR11434038	SRR11434037	SRR11434036	SRR11434035
SRR11434034	SRR11434033	SRR11434032	SRR11434026	SRR11434031	SRR11434030	SRR11434029	SRR11434009
SRR11434008	SRR11434007	SRR11434006	SRR11434005	SRR11434004	SRR11434003	SRR11434002	SRR11434001
SRR11434000	SRR11433999	SRR11433998	SRR11433997	SRR11434056	SRR11434055	SRR11434054	SRR11434053
SRR11434052	SRR11434051	SRR11434050	SRR11434049	SRR11434048	SRR11433898	SRR11433897	SRR11433896
SRR11433895	SRR11433894	SRR11434027	SRR11434025	SRR11434024	SRR11434023	SRR11434022	SRR11434021
SRR11434020	SRR11434019	SRR11434018	SRR11434017	SRR11434016	SRR11434015	SRR11434014	SRR11434013
SRR11434012	SRR11434011	SRR11434010	SRR11433994	SRR11433993	SRR11433992	SRR11433991	SRR11433990
SRR11433989	SRR11433988	SRR11433987	SRR11433986	SRR11433985	SRR11433984	SRR11433983	SRR11433982
SRR11433981	SRR11433980	SRR11433979	SRR11433978	SRR11433977	SRR11433903	SRR11433902	SRR11433901
SRR11433900	SRR11433899	SRR11434276	SRR11434275	SRR11434274	SRR11434273	SRR11434272	SRR11434271
SRR11434270	SRR11434269	SRR11434268	SRR11434267	SRR11434266	SRR11434265	SRR11434264	SRR11434263
SRR11434262	SRR11434261	SRR11434260	SRR11434259	SRR11434258	SRR11434257	SRR11434256	SRR11434255
SRR11434254	SRR11433996	SRR11433995	SRR11434240	SRR11434239	SRR11434238	SRR11434237	SRR11434236
SRR11434235	SRR11434234	SRR11434233	SRR11434232	SRR11434231	SRR11434230	SRR11434229	SRR11434228
SRR11434227	SRR11434226	SRR11434225	SRR11434224	SRR11434283	SRR11434282	SRR11434281	SRR11434280
SRR11434279	SRR11434278	SRR11434277	SRR11434204	SRR11434203	SRR11434202	SRR11434201	SRR11434200
SRR11434199	SRR11434198	SRR11434197	SRR11434196	SRR11434195	SRR11434194	SRR11434253	SRR11434252
SRR11434251	SRR11434250	SRR11434249	SRR11434248	SRR11434247	SRR11434246	SRR11434245	SRR11434244
SRR11434243	SRR11434242	SRR11434241	SRR11434107	SRR11434106	SRR11434105	SRR11434104	SRR11434103
SRR11434102	SRR11434223	SRR11434222	SRR11434221	SRR11434220	SRR11434219	SRR11434218	SRR11434217
SRR11434216	SRR11434215	SRR11434214	SRR11434213	SRR11434212	SRR11434211	SRR11434210	SRR11434209
SRR11434208	SRR11434207	SRR11434206	SRR11434205	SRR11434164	SRR11434163	SRR11434162	SRR11434161
SRR11434160	SRR11434159	SRR11434158	SRR11434121	SRR11434120	SRR11434119	SRR11434118	SRR11434117
SRR11434116	SRR11434115	SRR11434114	SRR11434113	SRR11434112	SRR11434111	SRR11434110	SRR11434109
SRR11434108	SRR11434185	SRR11434184	SRR11434183	SRR11434182	SRR11434181	SRR11434180	SRR11434179
SRR11434178	SRR11434177	SRR11434176	SRR11434175	SRR11434174	SRR11434173	SRR11434172	SRR11434171
SRR11434170	SRR11434169	SRR11434168	SRR11434167	SRR11434166	SRR11434165	SRR11434089	SRR11434088
SRR11434086	SRR11434085	SRR11434084	SRR11434083	SRR11434082	SRR11434087	SRR11434081	SRR11434080
SRR11434079	SRR11434078	SRR11434077	SRR11434076	SRR11434075	SRR11434074	SRR11434073	SRR11434072
SRR11434193	SRR11434192	SRR11434191	SRR11434190	SRR11434189	SRR11434188	SRR11434187	SRR11434186
SRR11434101	SRR11434100	SRR11434099	SRR11434098	SRR11434097	SRR11434096	SRR11434095	SRR11434094
SRR11434093	SRR11434092	SRR11434091	SRR11434090	SRR11433965	SRR11433963	SRR11433962	SRR11433961
SRR11433960	SRR11433959	SRR11433958	SRR11433957	SRR11433956	SRR11433955	SRR11434143	SRR11434142
SRR11434141	SRR11434140	SRR11434139	SRR11434138	SRR11434137	SRR11434136	SRR11434135	SRR11434134
SRR11434133	SRR11434132	SRR11434131	SRR11434130	SRR11434145	SRR11434144	SRR11434129	SRR11434128
SRR11434127	SRR11434126	SRR11434125	SRR11434124	SRR11434123	SRR11434122	SRR11433976	SRR11433975
SRR11433974	SRR11433973	SRR11433972	SRR11433971	SRR11433970	SRR11433969	SRR11433968	SRR11433964
SRR11433967	SRR11433966	SRR11434157	SRR11434156	SRR11434155	SRR11434154	SRR11434153	SRR11434152
SRR11434151	SRR11434150	SRR11434149	SRR11434148	SRR11434147	SRR11434146	SRR11433939	SRR11433938
SRR11433937	SRR11433936	SRR11433935	SRR11433934	SRR11433933	SRR11433932	SRR11433931	SRR11433930
SRR11433929	SRR11433928	SRR11433927	SRR11433926	SRR11433925	SRR11433924	SRR11433923	SRR11433922
SRR11433921	SRR11433920	SRR11433919	SRR11433918	SRR11433917	SRR11433916	SRR11433954	SRR11433953
SRR11433952	SRR11433951	SRR11433950	SRR11433949	SRR11433948	SRR11433947	SRR11433946	SRR11433945
SRR11433944	SRR11433943	SRR11433942	SRR11433941	SRR11433940	SRR11433915	SRR11433914	SRR11433913
SRR11433912	SRR11433911	SRR11433910	SRR11433909	SRR11433908	SRR11433907	SRR11433906	SRR11433905
SRR11433904	SRR11434301	SRR11434300	SRR11434299	SRR11434298	SRR11434297	SRR11434296	SRR11445603
SRR11445602	SRR11445601	SRR11458988	SRR11458987	SRR11497600	SRR11497599	SRR11497598	SRR11497597
SRR11497606	SRR11497608	SRR11497607	SRR11497605	SRR11497604	SRR11497603	SRR11497602	SRR11497601
SRR11611336	SRR11611335	SRR11611324	SRR11611313	SRR11611302	SRR11611291	SRR11611280	SRR11611269
SRR11611268	SRR11611267	SRR11611334	SRR11611333	SRR11611332	SRR11611331	SRR11611330	SRR11611329

SRR11611328	SRR11611327	SRR11611326	SRR11611325	SRR11611323	SRR11611322	SRR11611321	SRR11611320
SRR11611319	SRR11611318	SRR11611317	SRR11611316	SRR11611315	SRR11611314	SRR11611312	SRR11611311
SRR11611310	SRR11611309	SRR11611308	SRR11611307	SRR11611306	SRR11611305	SRR11611304	SRR11611303
SRR11611301	SRR11611300	SRR11611299	SRR11611298	SRR11611297	SRR11611296	SRR11611295	SRR11611294
SRR11611293	SRR11611292	SRR11611290	SRR11611289	SRR11611288	SRR11611287	SRR11611286	SRR11611285
SRR11611284	SRR11611283	SRR11611282	SRR11611281	SRR11611279	SRR11611278	SRR11611277	SRR11611276
SRR11611275	SRR11611274	SRR11611273	SRR11611272	SRR11611271	SRR11611270	SRR11576992	SRR11576996
SRR11577007	SRR11576990	SRR11576995	SRR11577003	SRR11577004	SRR11577008	SRR11577011	SRR11577005
SRR11577006	SRR11576994	SRR11576986	SRR11576988	SRR11576998	SRR11577009	SRR11577001	SRR11576987
SRR11576993	SRR11576997	SRR11577012	SRR11576991	SRR11577000	SRR11576999	SRR11577010	SRR11576989
SRR11577002	SRR11625162	SRR11625161	SRR11625160	SRR11625159	SRR11652949	SRR11652948	SRR11652947
SRR11652946	SRR11652945	SRR11652944	SRR11652943	SRR11652942	SRR11652941	SRR11652940	SRR11680296
SRR11680295	SRR11680294	SRR11680293	SRR11680292	SRR11680291	SRR11680290	SRR11680289	SRR11680288
SRR11680287	SRR11680275	SRR11680274	SRR11680273	SRR11680286	SRR11680285	SRR11680284	SRR11680283
SRR11680282	SRR11680281	SRR11680280	SRR11680279	SRR11680278	SRR11680277	SRR11680276	SRR11680482
SRR11680481	SRR11680480	SRR11680479	SRR11680478	SRR11680477	SRR11767718	SRR11767717	SRR11767716
SRR11767715	SRR11767714	SRR11767713	SRR11767724	SRR11767723	SRR11767722	SRR11767721	SRR11767720
SRR11767719	SRR12011936	SRR12011935	SRR12011934	SRR12011933	SRR12011932	SRR12011931	SRR11811931
SRR11811932	SRR11812322	SRR11812323	SRR11862672	SRR11862671	SRR11862673	SRR11862670	SRR11862859
SRR11862862	SRR11862861	SRR11862860	SRR11862896	SRR11862899	SRR11862897	SRR11862898	SRR11866744
SRR11866743	SRR11866745	SRR11866746	SRR11867838	SRR11867835	SRR11867837	SRR11867836	SRR11867996
SRR11867995	SRR11867997	SRR11867998	SRR11868217	SRR11868216	SRR11868218	SRR11868219	SRR11868368
SRR11868369	SRR11868400	SRR11868399	SRR11793935	SRR11793934	SRR11810843	SRR11810842	SRR11810839
SRR11810838	SRR11810837	SRR11810836	SRR11810835	SRR11810834	SRR11810833	SRR11810832	SRR11810841
SRR11810840	SRR11809918	SRR11809917	SRR11809916	SRR11809915	SRR11809924	SRR11809923	SRR11809922
SRR11809921	SRR11809920	SRR11809919	SRR11816531	SRR11816530	SRR11816529	SRR11816528	SRR11816527
SRR11816526	SRR11816539	SRR11816538	SRR11816537	SRR11816536	SRR11816535	SRR11816534	SRR11809756
SRR11809755	SRR11809754	SRR11809753	SRR11814558	SRR11814557	SRR11814556	SRR11814555	SRR11814554
SRR11814553	SRR11815061	SRR11815060	SRR11815059	SRR11815058	SRR11815057	SRR11815056	SRR11939300
SRR11939299	SRR11939298	SRR11939297	SRR11939296	SRR11939295	SRR11939294	SRR11939293	SRR11939291
SRR11939290	SRR11939289	SRR11939288	SRR11939287	SRR11939286	SRR11939285	SRR11939284	SRR11939283
SRR11939282	SRR11939280	SRR11939279	SRR11939278	SRR11939277	SRR11939276	SRR11939275	SRR11939274
SRR11939273	SRR11939272	SRR11939271	SRR11939269	SRR11939268	SRR11939267	SRR11939266	SRR11939265
SRR11939264	SRR11939263	SRR11939262	SRR11939261	SRR11939260	SRR11939258	SRR11939257	SRR11939256
SRR11939255	SRR11939254	SRR11939253	SRR11939252	SRR11939251	SRR11939250	SRR11939249	SRR11939247
SRR11939246	SRR11939245	SRR11939244	SRR11939243	SRR11939242	SRR11939241	SRR11939240	SRR11939239
SRR11939238	SRR11939235	SRR11939234	SRR11939233	SRR11939232	SRR11939231	SRR11939230	SRR11939229
SRR11939228	SRR11939227	SRR11939226	SRR11939224	SRR11939223	SRR11939222	SRR11939221	SRR11939220
SRR11939219	SRR11939218	SRR11939217	SRR11939216	SRR11939215	SRR11939213	SRR11939212	SRR11939211
SRR11939210	SRR11939209	SRR11939208	SRR11939207	SRR11939206	SRR11939205	SRR11939204	SRR11939202
SRR11939201	SRR11939200	SRR11939199	SRR11939198	SRR11939197	SRR11939196	SRR11939195	SRR11939194
SRR11939193	SRR11939191	SRR11939190	SRR11939189	SRR11939188	SRR11939187	SRR11939186	SRR11939185
SRR11939184	SRR11939183	SRR11939182	SRR11939180	SRR11939179	SRR11939178	SRR11939177	SRR11939176
SRR11939175	SRR11939174	SRR11939173	SRR11939172	SRR11939171	SRR11939169	SRR11939168	SRR11939167
SRR11939166	SRR11939165	SRR11939164	SRR11939163	SRR11939162	SRR11939161	SRR11939160	SRR11939158
SRR11939157	SRR11939156	SRR11939155	SRR11939154	SRR11939473	SRR11939472	SRR11939471	SRR11939470
SRR11939469	SRR11939467	SRR11939466	SRR11939465	SRR11939464	SRR11939463	SRR11939462	SRR11939461
SRR11939460	SRR11939459	SRR11939458	SRR11939456	SRR11939455	SRR11939454	SRR11939453	SRR11939452
SRR11939451	SRR11939450	SRR11939449	SRR11939448	SRR11939447	SRR11939444	SRR11939443	SRR11939442
SRR11939441	SRR11939440	SRR11939439	SRR11939438	SRR11939437	SRR11939436	SRR11939435	SRR11939433
SRR11939432	SRR11939431	SRR11939430	SRR11939429	SRR11939428	SRR11939427	SRR11939426	SRR11939425
SRR11939424	SRR11939918	SRR11939917	SRR11939910	SRR11939909	SRR11939908	SRR11939907	SRR11939906
SRR11939905	SRR11939904	SRR11939903	SRR11939916	SRR11939915	SRR11939914	SRR11939913	SRR11939912
SRR11939911	SRR12511406	SRR12511405	SRR13080432	SRR13080431	SRR12511401	SRR13080430	SRR11968744
SRR11968745	SRR11968742	SRR11968743	SRR11968740	SRR11968741	SRR11968755	SRR11968754	SRR11968752
SRR11968753	SRR11968751	SRR11968750	SRR11968748	SRR11968749	SRR11968746	SRR11968747	SRR12016210
SRR12016209	SRR12016208	SRR12016207	SRR12072288	SRR12072292	SRR12072293	SRR12072297	SRR12072286
SRR12072291	SRR12072287	SRR12072290	SRR12072289	SRR12072294	SRR12072295	SRR12072296	SRR12032693

SRR12032692	SRR12032691	SRR12032699	SRR12032698	SRR12032697	SRR12032696	SRR12032695	SRR12032694
SRR12044481	SRR12044480	SRR12044605	SRR12044594	SRR12044583	SRR12044572	SRR12044507	SRR12044496
SRR12044485	SRR12044564	SRR12044479	SRR12044544	SRR12044533	SRR12044522	SRR12044521	SRR12044520
SRR12044519	SRR12044518	SRR12044477	SRR12044606	SRR12044604	SRR12044603	SRR12044602	SRR12044601
SRR12044600	SRR12044599	SRR12044598	SRR12044597	SRR12044596	SRR12044595	SRR12044593	SRR12044592
SRR12044591	SRR12044590	SRR12044589	SRR12044588	SRR12044587	SRR12044586	SRR12044585	SRR12044584
SRR12044582	SRR12044581	SRR12044580	SRR12044579	SRR12044578	SRR12044577	SRR12044576	SRR12044575
SRR12044574	SRR12044573	SRR12044517	SRR12044516	SRR12044515	SRR12044514	SRR12044513	SRR12044512
SRR12044511	SRR12044510	SRR12044509	SRR12044508	SRR12044506	SRR12044505	SRR12044504	SRR12044503
SRR12044502	SRR12044501	SRR12044500	SRR12044499	SRR12044498	SRR12044497	SRR12044495	SRR12044494
SRR12044493	SRR12044492	SRR12044491	SRR12044490	SRR12044489	SRR12044488	SRR12044487	SRR12044486
SRR12044484	SRR12044483	SRR12044482	SRR12044571	SRR12044570	SRR12044569	SRR12044568	SRR12044567
SRR12044566	SRR12044565	SRR12044563	SRR12044562	SRR12044561	SRR12044560	SRR12044559	SRR12044558
SRR12044557	SRR12044556	SRR12044555	SRR12044553	SRR12044478	SRR12044554	SRR12044552	SRR12044551
SRR12044550	SRR12044549	SRR12044548	SRR12044547	SRR12044546	SRR12044545	SRR12044543	SRR12044542
SRR12044541	SRR12044540	SRR12044539	SRR12044538	SRR12044537	SRR12044536	SRR12044535	SRR12044534
SRR12044532	SRR12044531	SRR12044530	SRR12044529	SRR12044528	SRR12044527	SRR12044526	SRR12044525
SRR12044524	SRR12044523	SRR12072416	SRR12072415	SRR12072413	SRR12072411	SRR12072410	SRR12072409
SRR12072412	SRR12072408	SRR12072407	SRR12072406	SRR12072414	SRR12082071	SRR12082070	SRR12082069
SRR12082068	SRR12082067	SRR12082066	SRR12082917	SRR12082916	SRR12082915	SRR12082914	SRR12082913
SRR12082912	SRR12082911	SRR12082910	SRR12082909	SRR12082908	SRR12096695	SRR12096694	SRR12096693
SRR12172860	SRR12172859	SRR12172858	SRR12172857	SRR12172856	SRR12172855	SRR12179672	SRR12179671
SRR12179670	SRR12179669	SRR12179668	SRR12179667	SRR12179666	SRR12179665	SRR12179664	SRR12208195
SRR12208194	SRR12208193	SRR12208192	SRR12208199	SRR12208198	SRR12208197	SRR12208196	SRR12202389
SRR12202388	SRR12202387	SRR12202386	SRR12202385	SRR12202384	SRR12202383	SRR12202382	SRR12261481
SRR12261480	SRR12261479	SRR12261478	SRR12261477	SRR12261476	SRR12261475	SRR12261474	SRR12261473
SRR12245388	SRR12245387	SRR12245386	SRR12245385	SRR12245384	SRR12245383	SRR12269831	SRR12269830
SRR12269829	SRR12298332	SRR12298331	SRR12298330	SRR12298329	SRR12298328	SRR12298327	SRR12298326
SRR12298325	SRR12298324	SRR12298323	SRR12298322	SRR12298321	SRR12298318	SRR12298317	SRR12298316
SRR12298315	SRR12298314	SRR12298320	SRR12298319	SRR12298337	SRR12298336	SRR12298335	SRR12298334
SRR12298333	SRR12316591	SRR12316586	SRR12316585	SRR12316584	SRR12316579	SRR12316578	SRR12316577
SRR12316576	SRR12316583	SRR12316582	SRR12316581	SRR12316580	SRR12316575	SRR12316574	SRR12316573
SRR12316622	SRR12316621	SRR12316620	SRR12316619	SRR12316618	SRR12316617	SRR12316616	SRR12316615
SRR12316614	SRR12316613	SRR12316612	SRR12316611	SRR12316610	SRR12316609	SRR12316608	SRR12316607
SRR12316606	SRR12316605	SRR12316604	SRR12316603	SRR12316602	SRR12316601	SRR12316600	SRR12316599
SRR12316598	SRR12316597	SRR12316596	SRR12316595	SRR12316594	SRR12316593	SRR12316592	SRR12316590
SRR12316589	SRR12316588	SRR12316587	SRR12406636	SRR12406635	SRR12406634	SRR12406637	SRR12415909
SRR12415910	SRR12415908	SRR12415907	SRR12415906	SRR12415904	SRR12415905	SRR12415903	SRR12415902
SRR12415900	SRR12415901	SRR12415899	SRR12415898	SRR12415916	SRR12415915	SRR12415914	SRR12415913
SRR12415912	SRR12415911	SRR12433268	SRR12433266	SRR12433276	SRR12433274	SRR12433272	SRR12433270
SRR12441120	SRR12441119	SRR12441118	SRR12441117	SRR12441116	SRR12441115	SRR12441114	SRR12441113
SRR12441112	SRR12441099	SRR12441098	SRR12441097	SRR12441096	SRR12441095	SRR12441094	SRR12441093
SRR12441111	SRR12441110	SRR12441109	SRR12441108	SRR12441107	SRR12441106	SRR12441105	SRR12441104
SRR12441103	SRR12441102	SRR12441101	SRR12441100	SRR12480636	SRR12480635	SRR12480634	SRR12480633
SRR12480639	SRR12480638	SRR12480637	SRR12473526	SRR12473634	SRR12490847	SRR12490846	SRR12490845
SRR12490844	SRR12490843	SRR12490842	SRR12490841	SRR12490840	SRR12490839	SRR12478153	SRR12478166
SRR12478165	SRR12478167	SRR12478164	SRR12478163	SRR12478162	SRR12478161	SRR12478160	SRR12478159
SRR12478158	SRR12478157	SRR12478156	SRR12478155	SRR12478154	SRR12486894	SRR12486893	SRR12486912
SRR12486901	SRR12486900	SRR12486899	SRR12486898	SRR12486897	SRR12486896	SRR12486895	SRR12486892
SRR12486891	SRR12486920	SRR12486919	SRR12486918	SRR12486917	SRR12486916	SRR12486915	SRR12486914
SRR12486913	SRR12486911	SRR12486910	SRR12486909	SRR12486908	SRR12486907	SRR12486906	SRR12486905
SRR12486904	SRR12486903	SRR12486902	SRR12492184	SRR12492183	SRR12492182	SRR12492181	SRR12492180
SRR12492185	SRR12572669	SRR12572670	SRR12572671	SRR12572672	SRR12572673	SRR12572674	SRR12572675
SRR12572676	SRR12719550	SRR12719549	SRR12719558	SRR12719557	SRR12719556	SRR12719555	SRR12719554
SRR12719553	SRR12719552	SRR12719551	SRR12719548	SRR12719547	SRR12719559	SRR12608404	SRR12608403
SRR12608402	SRR12608401	SRR12608400	SRR12608399	SRR12622864	SRR12622863	SRR12622862	SRR12622861
SRR12622860	SRR12622859	SRR12622858	SRR12622857	SRR12622856	SRR12650254	SRR12650253	SRR12650242
SRR12650231	SRR12650220	SRR12650217	SRR12650216	SRR12650215	SRR12650214	SRR12650213	SRR12650252

SRR12650251	SRR12650250	SRR12650249	SRR12650248	SRR12650247	SRR12650246	SRR12650245	SRR12650244
SRR12650243	SRR12650241	SRR12650240	SRR12650239	SRR12650238	SRR12650237	SRR12650236	SRR12650235
SRR12650234	SRR12650233	SRR12650232	SRR12650230	SRR12650229	SRR12650228	SRR12650227	SRR12650226
SRR12650225	SRR12650224	SRR12650223	SRR12650222	SRR12650221	SRR12650219	SRR12650218	SRR12666276
SRR12666275	SRR12666264	SRR12666253	SRR12666243	SRR12666242	SRR12666241	SRR12666240	SRR12666239
SRR12666238	SRR12666274	SRR12666273	SRR12666272	SRR12666271	SRR12666270	SRR12666269	SRR12666268
SRR12666267	SRR12666266	SRR12666265	SRR12666263	SRR12666262	SRR12666261	SRR12666260	SRR12666259
SRR12666258	SRR12666257	SRR12666256	SRR12666255	SRR12666254	SRR12666252	SRR12666251	SRR12666250
SRR12666249	SRR12666248	SRR12666247	SRR12666246	SRR12666245	SRR12666244	SRR12672173	SRR12672172
SRR12672171	SRR12672170	SRR12672169	SRR12672168	SRR12672167	SRR12672166	SRR12672165	SRR12672164
SRR12672163	SRR12672162	SRR12672161	SRR12672184	SRR12672182	SRR12672181	SRR12672180	SRR12672179
SRR12672178	SRR12672177	SRR12672176	SRR12672175	SRR12672174	SRR12672146	SRR12672160	SRR12672159
SRR12672158	SRR12672157	SRR12672156	SRR12672155	SRR12672153	SRR12672152	SRR12672151	SRR12672150
SRR12672149	SRR12672148	SRR12672147	SRR12674047	SRR12674036	SRR12674048	SRR12674014	SRR12674015
SRR12674016	SRR12674042	SRR12674043	SRR12674044	SRR12674035	SRR12674037	SRR12674038	SRR12674024
SRR12674022	SRR12674023	SRR12674030	SRR12674031	SRR12674029	SRR12676649	SRR12676658	SRR12676657
SRR12676656	SRR12676655	SRR12676654	SRR12676653	SRR12676652	SRR12676651	SRR12676650	SRR12686527
SRR12686526	SRR12686523	SRR12686522	SRR12686521	SRR12686520	SRR12686519	SRR12686518	SRR12686517
SRR12686516	SRR12686525	SRR12686524	SRR12691812	SRR12691811	SRR12691810	SRR12691809	SRR12691808
SRR12691807	SRR12697109	SRR12697108	SRR12697103	SRR12697102	SRR12697097	SRR12697096	SRR12697121
SRR12697120	SRR12697114	SRR12697091	SRR12697090	SRR12697133	SRR12697132	SRR12697127	SRR12697126
SRR12697115	SRR12738855	SRR12758969	SRR12758968	SRR12758967	SRR12758966	SRR12758965	SRR12758964
SRR12758963	SRR12758962	SRR12758961	SRR12759012	SRR12759011	SRR12759010	SRR12759009	SRR12759008
SRR12759007	SRR12759006	SRR12759005	SRR12759004	SRR12759003	SRR12759002	SRR12759001	SRR12759000
SRR12758999	SRR12758998	SRR12758997	SRR12758996	SRR12758995	SRR12758994	SRR12758993	SRR12758992
SRR12758991	SRR12758990	SRR12758989	SRR12758988	SRR12758987	SRR12758986	SRR12758985	SRR12758984
SRR12758983	SRR12758982	SRR12758981	SRR12758980	SRR12758979	SRR12758978	SRR12758977	SRR12758976
SRR12758975	SRR12758974	SRR12758973	SRR12758972	SRR12758971	SRR12758970	SRR12810493	SRR12810492
SRR12810489	SRR12810488	SRR12810487	SRR12810486	SRR12810485	SRR12810484	SRR12810483	SRR12810482
SRR12810491	SRR12810490	SRR12835320	SRR12835323	SRR12835331	SRR12835330	SRR12835329	SRR12835328
SRR12835327	SRR12835326	SRR12835325	SRR12835324	SRR12835322	SRR12835321	SRR12835341	SRR12835339
SRR12835338	SRR12835337	SRR12835336	SRR12835335	SRR12835334	SRR12835333	SRR12835332	SRR12835354
SRR12835353	SRR12835352	SRR12835351	SRR12835350	SRR12835349	SRR12835348	SRR12835347	SRR12835346
SRR12835345	SRR12835344	SRR12835343	SRR12835342	SRR12919218	SRR12919217	SRR12907277	SRR12907276
SRR12907275	SRR12907274	SRR12907273	SRR12907272	SRR12907271	SRR12873772	SRR12873773	SRR12873770
SRR12873771	SRR12873768	SRR12873769	SRR12873766	SRR12873767	SRR12873764	SRR12873765	SRR12873762
SRR12873763	SRR12873760	SRR12873761	SRR12873758	SRR12873759	SRR12873757	SRR12873756	SRR12873754
SRR12873755	SRR12875345	SRR12875344	SRR12875343	SRR12875342	SRR12875341	SRR12897679	SRR12897673
SRR12897670	SRR12897669	SRR12897668	SRR12897678	SRR12897677	SRR12897676	SRR12897675	SRR12897674
SRR12897672	SRR12897671	SRR12897624	SRR12897623	SRR12897622	SRR12897621	SRR12897620	SRR12897619
SRR12897628	SRR12897627	SRR12904663	SRR12904662	SRR12904659	SRR12904658	SRR12904657	SRR12904656
SRR12904655	SRR12904654	SRR12904653	SRR12904652	SRR12904661	SRR12904660	SRR12936513	SRR12936512
SRR12936539	SRR12936538	SRR12936537	SRR12936536	SRR12936535	SRR12936534	SRR12936533	SRR12936532
SRR12936517	SRR12936516	SRR12936515	SRR12936514	SRR12936540	SRR12936531	SRR12936530	SRR12936529
SRR12936528	SRR12936527	SRR12936526	SRR12936525	SRR12936524	SRR12936523	SRR12936522	SRR12936521
SRR12936520	SRR12936519	SRR12936518	SRR12937444	SRR12937443	SRR12937442	SRR12937441	SRR12937439
SRR12937440	SRR12937437	SRR12937438	SRR12937449	SRR12937447	SRR12937448	SRR12937446	SRR12937445
SRR12937436	SRR12937435	SRR12937434	SRR12937433	SRR12937431	SRR12937432	SRR12937430	SRR12937429
SRR12937475	SRR12937428	SRR12937474	SRR12937473	SRR12937472	SRR12937471	SRR12937469	SRR12937470
SRR12937467	SRR12937468	SRR12937466	SRR12937465	SRR12937464	SRR12937463	SRR12937462	SRR12937461
SRR12937460	SRR12937459	SRR12937458	SRR12937457	SRR12937456	SRR12937455	SRR12937454	SRR12937453
SRR12937452	SRR12937451	SRR12937450	SRR12984014	SRR12984013	SRR12984012	SRR13009988	SRR13009987
SRR13009986	SRR13009985	SRR13066180	SRR13066179	SRR13066178	SRR13066177	SRR13066176	SRR13066175
SRR13066174	SRR13066173	SRR13066172	SRR13066171	SRR13066182	SRR13066181	SRR15076562	SRR15076561
SRR15076560	SRR13067767	SRR13067766	SRR13067765	SRR13067764	SRR13067763	SRR13067762	SRR13067761
SRR13067760	SRR13067759	SRR13067758	SRR13067757	SRR13067756	SRR13067755	SRR13067882	SRR13067880
SRR13067878	SRR13067877	SRR13067879	SRR13067881	SRR13172499	SRR13172500	SRR13172498	SRR13172506
SRR13172505	SRR13172504	SRR13172503	SRR13172502	SRR13172501	SRR13172494	SRR13172493	SRR13172492

SRR13172491	SRR13172490	SRR13172489	SRR13172488	SRR13172487	SRR13172486	SRR13172485	SRR13172484
SRR13172497	SRR13172496	SRR13172495	SRR13195561	SRR13195560	SRR13195559	SRR13195558	SRR13195557
SRR13195556	SRR13195555	SRR13195554	SRR13195553	SRR13195552	SRR13195551	SRR13195550	SRR13195549
SRR13195548	SRR13195547	SRR13195546	SRR13195545	SRR13195544	SRR13202232	SRR13202231	SRR13202220
SRR13202209	SRR13202198	SRR13202187	SRR13202176	SRR13202172	SRR13202171	SRR13202170	SRR13202230
SRR13202229	SRR13202228	SRR13202227	SRR13202226	SRR13202225	SRR13202224	SRR13202223	SRR13202222
SRR13202221	SRR13202219	SRR13202218	SRR13202217	SRR13202216	SRR13202215	SRR13202214	SRR13202213
SRR13202212	SRR13202211	SRR13202210	SRR13202208	SRR13202207	SRR13202206	SRR13202205	SRR13202204
SRR13202203	SRR13202202	SRR13202201	SRR13202200	SRR13202199	SRR13202197	SRR13202196	SRR13202195
SRR13202194	SRR13202193	SRR13202192	SRR13202191	SRR13202190	SRR13202189	SRR13202188	SRR13202186
SRR13202185	SRR13202184	SRR13202183	SRR13202182	SRR13202181	SRR13202180	SRR13202179	SRR13202178
SRR13202177	SRR13202175	SRR13202174	SRR13202173	SRR13259178	SRR13259177	SRR13259176	SRR13259175
SRR13259174	SRR13259179	SRR13235683	SRR13235682	SRR13235681	SRR13235680	SRR13235679	SRR13235678
SRR13252468	SRR13252467	SRR13252466	SRR13252465	SRR13252464	SRR13252463	SRR13252462	SRR13252461
SRR13252460	SRR13252459	SRR13252458	SRR13252446	SRR13252445	SRR13252444	SRR13252457	SRR13252456
SRR13252455	SRR13252454	SRR13252453	SRR13252452	SRR13252451	SRR13252450	SRR13252449	SRR13252443
SRR13252442	SRR13252441	SRR13252440	SRR13252448	SRR13252447	SRR13277483	SRR13277484	SRR13277485
SRR13277486	SRR13277478	SRR13277479	SRR13277480	SRR13277481	SRR13277482	SRR13282990	SRR13282989
SRR13282991	SRR13291689	SRR13291688	SRR13291687	SRR13291686	SRR13291685	SRR13291693	SRR13291692
SRR13291691	SRR13291690	SRR13297202	SRR13297201	SRR13297193	SRR13297192	SRR13297191	SRR13297190
SRR13297189	SRR13297188	SRR13297187	SRR13297186	SRR13297200	SRR13297199	SRR13297198	SRR13297197
SRR13297196	SRR13297195	SRR13297194	SRR13297172	SRR13297171	SRR13297180	SRR13297179	SRR13297178
SRR13297177	SRR13297176	SRR13297175	SRR13297174	SRR13297173	SRR13297170	SRR13297169	SRR13297185
SRR13297184	SRR13297183	SRR13297182	SRR13297181	SRR13273972	SRR13273971	SRR13273970	SRR13273969
SRR13273968	SRR13273967	SRR13273966	SRR13273965	SRR13273964	SRR13273963	SRR13273962	SRR13273961
SRR13273960	SRR13273959	SRR13273958	SRR13273957	SRR13273956	SRR13273955	SRR13273954	SRR13273953
SRR13273939	SRR13273938	SRR13273937	SRR13273936	SRR13273935	SRR13273974	SRR13273973	SRR13273952
SRR13273951	SRR13273950	SRR13273949	SRR13273948	SRR13273947	SRR13273946	SRR13273945	SRR13273944
SRR13273943	SRR13273942	SRR13273941	SRR13273940	SRR13275275	SRR13275274	SRR13275283	SRR13275282
SRR13275281	SRR13275280	SRR13275279	SRR13275278	SRR13275277	SRR13275276	SRR13275273	SRR13275272
SRR13275288	SRR13275287	SRR13275286	SRR13275285	SRR13275284	SRR13308297	SRR13308296	SRR13308287
SRR13308288	SRR13308289	SRR13308290	SRR13308291	SRR13308292	SRR13308293	SRR13308294	SRR13308295
SRR13308286	SRR13364296	SRR13364295	SRR13364294	SRR13364293	SRR13364292	SRR13364291	SRR13364290
SRR13364289	SRR13349253	SRR13349252	SRR13349251	SRR13349250	SRR13349249	SRR13349248	SRR13362306
SRR13362305	SRR13362299	SRR13362298	SRR13362297	SRR13362296	SRR13362295	SRR13362294	SRR13362293
SRR13362292	SRR13362304	SRR13362303	SRR13362302	SRR13362301	SRR13362300	SRR13399400	SRR13399399
SRR13399398	SRR13399397	SRR13399396	SRR13399395	SRR13377178	SRR13377177	SRR13377176	SRR13394635
SRR13394634	SRR13441378	SRR13441377	SRR13441376	SRR13449277	SRR13449278	SRR13449275	SRR13449276
SRR13449273	SRR13449274	SRR13449271	SRR13449272	SRR13449270	SRR13449269	SRR13449268	SRR13449267
SRR13449265	SRR13449266	SRR13449264	SRR13449263	SRR13449261	SRR13449262	SRR13449260	SRR13449259
SRR13449258	SRR13449257	SRR13449255	SRR13449256	SRR13449253	SRR13449254	SRR13449248	SRR13449247
SRR13449245	SRR13449246	SRR13449243	SRR13449244	SRR13449364	SRR13449363	SRR13449362	SRR13449361
SRR13449359	SRR13449360	SRR13449357	SRR13449358	SRR13449355	SRR13449356	SRR13449354	SRR13449353
SRR13449352	SRR13449351	SRR13449350	SRR13449349	SRR13449347	SRR13449348	SRR13449346	SRR13449345
SRR13449298	SRR13449297	SRR13449295	SRR13449296	SRR13449294	SRR13449293	SRR13449292	SRR13449291
SRR13449289	SRR13449290	SRR13449288	SRR13449287	SRR13449286	SRR13449285	SRR13449284	SRR13449283
SRR13449282	SRR13449281	SRR13449280	SRR13449279	SRR13449394	SRR13449393	SRR13449391	SRR13449392
SRR13449390	SRR13449389	SRR13449388	SRR13449387	SRR13449385	SRR13449386	SRR13449383	SRR13449384
SRR13449382	SRR13449381	SRR13449379	SRR13449380	SRR13449377	SRR13449378	SRR13449376	SRR13449375
SRR13449373	SRR13449374	SRR13449371	SRR13449372	SRR13449369	SRR13449370	SRR13449368	SRR13449367
SRR13449365	SRR13449366	SRR13449320	SRR13449319	SRR13449318	SRR13449317	SRR13449315	SRR13449316
SRR13449314	SRR13449313	SRR13449311	SRR13449312	SRR13449310	SRR13449309	SRR13449308	SRR13449307
SRR13449305	SRR13449306	SRR13449303	SRR13449304	SRR13449301	SRR13449302	SRR13449299	SRR13449300
SRR13449241	SRR13449242	SRR13449240	SRR13449239	SRR13449238	SRR13449237	SRR13449251	SRR13449252
SRR13449250	SRR13449249	SRR13449236	SRR13449235	SRR13449233	SRR13449234	SRR13449231	SRR13449232
SRR13449230	SRR13449229	SRR13449228	SRR13449227	SRR13449226	SRR13449225	SRR13449343	SRR13449344
SRR13449342	SRR13449341	SRR13449339	SRR13449340	SRR13449338	SRR13449337	SRR13449336	SRR13449335
SRR13449334	SRR13449333	SRR13449332	SRR13449331	SRR13449329	SRR13449330	SRR13449328	SRR13449327

SRR13449326	SRR13449325	SRR13449324	SRR13449323	SRR13449321	SRR13449322	SRR13503231	SRR13503230
SRR13503229	SRR13503228	SRR13503227	SRR13503226	SRR13559793	SRR13559792	SRR13559791	SRR13559790
SRR13559789	SRR13559788	SRR13577326	SRR13577325	SRR13577319	SRR13577318	SRR13577317	SRR13577316
SRR13577315	SRR13577314	SRR13577313	SRR13577312	SRR13577324	SRR13577323	SRR13577322	SRR13577321
SRR13577320	SRR13628080	SRR13628079	SRR13628078	SRR13628092	SRR13628091	SRR13628090	SRR13628089
SRR13628088	SRR13628087	SRR13628086	SRR13628085	SRR13628084	SRR13628083	SRR13628082	SRR13628081
SRR13628104	SRR13628103	SRR13628102	SRR13628101	SRR13628100	SRR13628099	SRR13628098	SRR13628097
SRR13628096	SRR13628095	SRR13628094	SRR13628093	SRR13628116	SRR13628115	SRR13628114	SRR13628113
SRR13628112	SRR13628111	SRR13628110	SRR13628109	SRR13628108	SRR13628107	SRR13628106	SRR13628105
SRR13670010	SRR13670009	SRR13670044	SRR13670033	SRR13670022	SRR13670015	SRR13670014	SRR13670013
SRR13670012	SRR13670011	SRR13670008	SRR13670007	SRR13670006	SRR13670051	SRR13670050	SRR13670049
SRR13670048	SRR13670047	SRR13670046	SRR13670045	SRR13670043	SRR13670042	SRR13670041	SRR13670040
SRR13670039	SRR13670038	SRR13670037	SRR13670036	SRR13670035	SRR13670034	SRR13670032	SRR13670031
SRR13670030	SRR13670029	SRR13670028	SRR13670027	SRR13670026	SRR13670025	SRR13670024	SRR13670023
SRR13670021	SRR13670020	SRR13670019	SRR13670018	SRR13670017	SRR13670016	SRR13651477	SRR13651476
SRR13651475	SRR13651474	SRR13651473	SRR13651472	SRR13665005	SRR13665006	SRR13665007	SRR13665008
SRR13665009	SRR13665010	SRR13665011	SRR13665012	SRR13665013	SRR13665014	SRR13665015	SRR13665016
SRR13665017	SRR13665018	SRR13665019	SRR13665020	SRR13665021	SRR13665022	SRR13665023	SRR13665024
SRR13665025	SRR13665026	SRR13665027	SRR13665028	SRR13665029	SRR13665030	SRR13665031	SRR13665032
SRR13665033	SRR13665034	SRR13665035	SRR13694668	SRR13694667	SRR13694666	SRR15015757	SRR15015758
SRR15015759	SRR15015760	SRR13694664	SRR15015753	SRR15015754	SRR15015755	SRR15015756	SRR15015749
SRR15015750	SRR15015751	SRR15015752	SRR13694661	SRR13694660	SRR13694659	SRR13694658	SRR13694657
SRR13760542	SRR13760543	SRR13760546	SRR13760539	SRR13760547	SRR13760538	SRR13760548	SRR13760540
SRR13760541	SRR13760544	SRR13760537	SRR13760545	SRR13786497	SRR13786496	SRR13786495	SRR13786494
SRR13791127	SRR13791126	SRR13791125	SRR13791124	SRR13791123	SRR13791122	SRR13826585	SRR13826584
SRR13826583	SRR13826582	SRR13826581	SRR13826580	SRR13826579	SRR13826578	SRR13826577	SRR13826576
SRR13826575	SRR13826574	SRR13970282	SRR13970281	SRR13970238	SRR13970227	SRR13970216	SRR13970337
SRR13970326	SRR13970315	SRR13970304	SRR13970293	SRR13970280	SRR13970269	SRR13970258	SRR13970247
SRR13970244	SRR13970243	SRR13970242	SRR13970241	SRR13970240	SRR13970239	SRR13970237	SRR13970236
SRR13970235	SRR13970234	SRR13970233	SRR13970232	SRR13970231	SRR13970230	SRR13970229	SRR13970228
SRR13970226	SRR13970225	SRR13970224	SRR13970223	SRR13970222	SRR13970221	SRR13970220	SRR13970219
SRR13970218	SRR13970217	SRR13970215	SRR13970346	SRR13970345	SRR13970344	SRR13970343	SRR13970342
SRR13970341	SRR13970340	SRR13970339	SRR13970338	SRR13970336	SRR13970335	SRR13970334	SRR13970333
SRR13970332	SRR13970331	SRR13970330	SRR13970329	SRR13970328	SRR13970327	SRR13970325	SRR13970324
SRR13970323	SRR13970322	SRR13970321	SRR13970320	SRR13970319	SRR13970318	SRR13970317	SRR13970316
SRR13970314	SRR13970313	SRR13970312	SRR13970311	SRR13970310	SRR13970309	SRR13970308	SRR13970307
SRR13970306	SRR13970305	SRR13970303	SRR13970302	SRR13970301	SRR13970300	SRR13970299	SRR13970298
SRR13970297	SRR13970296	SRR13970295	SRR13970294	SRR13970292	SRR13970291	SRR13970290	SRR13970289
SRR13970288	SRR13970287	SRR13970286	SRR13970285	SRR13970284	SRR13970283	SRR13970279	SRR13970278
SRR13970277	SRR13970276	SRR13970275	SRR13970274	SRR13970273	SRR13970272	SRR13970271	SRR13970270
SRR13970268	SRR13970267	SRR13970266	SRR13970265	SRR13970264	SRR13970263	SRR13970262	SRR13970261
SRR13970260	SRR13970259	SRR13970257	SRR13970256	SRR13970255	SRR13970254	SRR13970253	SRR13970252
SRR13970251	SRR13970250	SRR13970249	SRR13970248	SRR13970246	SRR13970245	SRR13913944	SRR13913943
SRR13913942	SRR13913941	SRR13913940	SRR13913939	SRR13913938	SRR13913937	SRR13913936	SRR13913935
SRR13924643	SRR13924642	SRR13924641	SRR13924640	SRR13924639	SRR13924638	SRR13924637	SRR13924636
SRR13924635	SRR13924634	SRR13924633	SRR13924632	SRR13924631	SRR13924630	SRR13924644	SRR13949084
SRR13949085	SRR13949068	SRR13949069	SRR13949070	SRR13949071	SRR13949080	SRR13949081	SRR13949082
SRR13949083	SRR13949086	SRR13949087	SRR13949072	SRR13949073	SRR13949074	SRR13949075	SRR13949076
SRR13949077	SRR13949078	SRR13949079	SRR13949365	SRR13949364	SRR13949353	SRR13949342	SRR13949331
SRR13949330	SRR13949329	SRR13949328	SRR13949327	SRR13949326	SRR13949363	SRR13949362	SRR13949361
SRR13949360	SRR13949359	SRR13949358	SRR13949357	SRR13949356	SRR13949355	SRR13949354	SRR13957251
SRR13957250	SRR13957249	SRR13957248	SRR13957247	SRR13957246	SRR13957245	SRR13957244	SRR13957243
SRR13957242	SRR13957241	SRR13957240	SRR13957239	SRR13957238	SRR13957237	SRR13957236	SRR13957235
SRR13957234	SRR13957233	SRR13957232	SRR13957231	SRR13957230	SRR13957252	SRR13989994	SRR13989993
SRR13989992	SRR13989991	SRR13989990	SRR13989989	SRR13989988	SRR13989987	SRR13989986	SRR13989985
SRR13989984	SRR13989983	SRR13996782	SRR13996781	SRR13996780	SRR13996779	SRR13996778	SRR13996777
SRR13996776	SRR13996775	SRR14089918	SRR14089917	SRR14089916	SRR14089915	SRR14089923	SRR14089922
SRR14089921	SRR14089920	SRR14089919	SRR14092565	SRR14092564	SRR14092561	SRR14092560	SRR14092559

SRR14092558	SRR14092557	SRR14092556	SRR14092555	SRR14092554	SRR14092563	SRR14092562	SRR14115420
SRR14115419	SRR14115418	SRR14117413	SRR14117412	SRR14117411	SRR14117410	SRR14117409	SRR14117408
SRR14117407	SRR14117406	SRR14140382	SRR14140381	SRR14140380	SRR14148300	SRR14148298	SRR14148297
SRR14148296	SRR14148295	SRR14148299	SRR14193624	SRR14193623	SRR14193622	SRR14193621	SRR14193620
SRR14193619	SRR14193618	SRR14193617	SRR14193616	SRR14193615	SRR14193614	SRR14193613	SRR14200500
SRR14200499	SRR14200498	SRR14200497	SRR14200496	SRR14200495	SRR14200494	SRR14200493	SRR14200492
SRR14200491	SRR14200490	SRR14200489	SRR14200514	SRR14200513	SRR14200512	SRR14200518	SRR14200517
SRR14200516	SRR14200515	SRR14200511	SRR14200510	SRR14200509	SRR14200508	SRR14200507	SRR14200506
SRR14200505	SRR14200504	SRR14200503	SRR14200502	SRR14200501	SRR14241032	SRR14241031	SRR14241026
SRR14241025	SRR14241024	SRR14241023	SRR14241022	SRR14241021	SRR14241020	SRR14241019	SRR14241030
SRR14241029	SRR14241028	SRR14241027	SRR14210735	SRR14210734	SRR14211801	SRR14211800	SRR14211789
SRR14211778	SRR14211771	SRR14211770	SRR14211769	SRR14211768	SRR14211767	SRR14211766	SRR14211799
SRR14211798	SRR14211797	SRR14211796	SRR14211795	SRR14211794	SRR14211793	SRR14211792	SRR14211791
SRR14211790	SRR14211788	SRR14211787	SRR14211786	SRR14211785	SRR14211784	SRR14211783	SRR14211782
SRR14211781	SRR14211780	SRR14211779	SRR14211777	SRR14211776	SRR14211775	SRR14211774	SRR14211773
SRR14211772	SRR14223600	SRR14223601	SRR14223602	SRR14223603	SRR14223604	SRR14223605	SRR14223606
SRR14223608	SRR14223609	SRR14223610	SRR14223611	SRR14223612	SRR14223613	SRR14223614	SRR14223615
SRR14223616	SRR14223617	SRR14223619	SRR14223620	SRR14223621	SRR14223622	SRR14223623	SRR14223547
SRR14223548	SRR14223549	SRR14223550	SRR14223551	SRR18156481	SRR18156480	SRR18156470	SRR18156469
SRR18156468	SRR18156467	SRR18156466	SRR18156463	SRR18156465	SRR18156479	SRR18156478	SRR18156464
SRR14238817	SRR14238816	SRR14238815	SRR14238814	SRR14238813	SRR14238835	SRR14238834	SRR14238833
SRR14238832	SRR14238831	SRR14238830	SRR14238829	SRR14238828	SRR14238827	SRR14238826	SRR14238825
SRR14238824	SRR14238823	SRR14238822	SRR14238821	SRR14238820	SRR14238819	SRR14238818	SRR14267934
SRR14267933	SRR14267932	SRR14267931	SRR14267930	SRR14267929	SRR14267928	SRR14267927	SRR14267926
SRR14267925	SRR14267924	SRR14267923	SRR14267922	SRR14267921	SRR14267920	SRR14267919	SRR14267918
SRR14267917	SRR14267916	SRR14267946	SRR14267945	SRR14267944	SRR14267943	SRR14267942	SRR14267941
SRR14267940	SRR14267939	SRR14267938	SRR14267937	SRR14267936	SRR14267935	SRR14285991	SRR14285992
SRR14285993	SRR14285994	SRR14285995	SRR14285986	SRR14285987	SRR14285988	SRR14285989	SRR14285990
SRR14285981	SRR14285982	SRR14285983	SRR14285984	SRR14285985	SRR14285976	SRR14285977	SRR14285978
SRR14285979	SRR14285980	SRR14285971	SRR14285972	SRR14285973	SRR14285974	SRR14285975	SRR14285966
SRR14285967	SRR14285968	SRR14285969	SRR14285970	SRR14285961	SRR14285962	SRR14285963	SRR14285964
SRR14285965	SRR14286001	SRR14286002	SRR14286003	SRR14286004	SRR14286005	SRR14285996	SRR14285997
SRR14285998	SRR14285999	SRR14286000	SRR14297046	SRR14297045	SRR14297034	SRR14297023	SRR14297012
SRR14297055	SRR14297050	SRR14297049	SRR14297048	SRR14297047	SRR14297044	SRR14297043	SRR14297042
SRR14297041	SRR14297040	SRR14297039	SRR14297038	SRR14297037	SRR14297036	SRR14297035	SRR14297033
SRR14297032	SRR14297031	SRR14297030	SRR14297029	SRR14297028	SRR14297027	SRR14297026	SRR14297025
SRR14297024	SRR14297022	SRR14297021	SRR14297020	SRR14297019	SRR14297018	SRR14297017	SRR14297016
SRR14297015	SRR14297014	SRR14297013	SRR14297011	SRR14297010	SRR14297009	SRR14297008	SRR14297007
SRR14297006	SRR14297059	SRR14297058	SRR14297057	SRR14297056	SRR14297054	SRR14297053	SRR14297052
SRR14297051	SRR14324674	SRR14324673	SRR14324672	SRR14324671	SRR14324670	SRR14324669	SRR14408077
SRR14408078	SRR14408075	SRR14408076	SRR14408073	SRR14408074	SRR14408071	SRR14408072	SRR14408069
SRR14408070	SRR14408067	SRR14408068	SRR14408065	SRR14408066	SRR14408033	SRR14408034	SRR14408063
SRR14408064	SRR14408061	SRR14408062	SRR14408059	SRR14408060	SRR14408057	SRR14408058	SRR14408055
SRR14408056	SRR14408053	SRR14408054	SRR14408051	SRR14408052	SRR14408049	SRR14408050	SRR14408047
SRR14408048	SRR14408045	SRR14408046	SRR14408043	SRR14408044	SRR14408041	SRR14408042	SRR14408039
SRR14408040	SRR14408031	SRR14408032	SRR14408037	SRR14408038	SRR14408035	SRR14408036	SRR14432726
SRR14432725	SRR14432724	SRR14432723	SRR14432722	SRR14432730	SRR14432729	SRR14432728	SRR14432727
SRR14432721	SRR14432720	SRR14432719	SRR14448086	SRR14448085	SRR14448084	SRR14448083	SRR14448082
SRR14448081	SRR14465195	SRR14465194	SRR14465193	SRR14465192	SRR14465191	SRR14465190	SRR14465189
SRR14465188	SRR14465187	SRR14465186	SRR14465185	SRR14465184	SRR14465183	SRR14465182	SRR14465204
SRR14465203	SRR14465202	SRR14465201	SRR14465200	SRR14465199	SRR14465198	SRR14465197	SRR14465196
SRR14521238	SRR14521237	SRR14521236	SRR14521235	SRR14521234	SRR14521233	SRR14521232	SRR14521231
SRR14521230	SRR14521229	SRR14521228	SRR14521227	SRR14552304	SRR14552303	SRR14552302	SRR14552301
SRR14552300	SRR14552299	SRR14552298	SRR14552297	SRR14552296	SRR14575907	SRR14575906	SRR14612102
SRR14612101	SRR14612100	SRR14612099	SRR14612098	SRR14612097	SRR14612096	SRR14612095	SRR14612094
SRR14612093	SRR14612092	SRR14612091	SRR14612090	SRR14612089	SRR14612088	SRR14612087	SRR14615892
SRR14615891	SRR14615890	SRR14615889	SRR14615888	SRR14615887	SRR14615886	SRR14615885	SRR14615884
SRR14615883	SRR14615882	SRR14631575	SRR14631574	SRR14631573	SRR14631572	SRR14631571	SRR14631570

SRR14672577	SRR14672575	SRR14672574	SRR14672573	SRR14672569	SRR14672568	SRR14672566	SRR14672565
SRR14672564	SRR14672561	SRR14672557	SRR14672556	SRR14672551	SRR14672547	SRR14672544	SRR14672543
SRR14672540	SRR14672538	SRR14672536	SRR14672534	SRR14664564	SRR14664563	SRR14664562	SRR14664561
SRR14664560	SRR14664559	SRR14667084	SRR14667083	SRR14667082	SRR14667081	SRR14752130	SRR14752129
SRR14752128	SRR14752127	SRR14752126	SRR14752125	SRR14752124	SRR14752123	SRR14752122	SRR14752121
SRR14765722	SRR14765721	SRR14765720	SRR14765719	SRR14765718	SRR14765717	SRR14765716	SRR14765715
SRR14765714	SRR14773913	SRR14773912	SRR14773911	SRR14773928	SRR14773927	SRR14773926	SRR14773925
SRR14773924	SRR14773923	SRR14773922	SRR14773921	SRR14773920	SRR14773919	SRR14773918	SRR14773917
SRR14773916	SRR14773915	SRR14773914	SRR14787865	SRR14787864	SRR14787863	SRR14787862	SRR14787861
SRR14787860	SRR14787859	SRR14787858	SRR350917	SRR350918	SRR14879193	SRR14879194	SRR14879202
SRR14879210	SRR14879209	SRR14879208	SRR14879207	SRR14879204	SRR14879205	SRR14879206	SRR14879195
SRR14879196	SRR14879197	SRR14879198	SRR14879199	SRR14879216	SRR14879215	SRR14879214	SRR14879200
SRR14879201	SRR14879203	SRR14879213	SRR14879212	SRR14879211	SRR14901944	SRR14901943	SRR14901942
SRR14901941	SRR14901940	SRR14901939	SRR14901938	SRR14901937	SRR14901936	SRR14901915	SRR14901914
SRR14901913	SRR14901911	SRR14901910	SRR14901909	SRR14901908	SRR14901907	SRR14901906	SRR14901884
SRR14901935	SRR14901933	SRR14901932	SRR14901931	SRR14901930	SRR14901929	SRR14901928	SRR14901927
SRR14901926	SRR14901905	SRR14901904	SRR14901903	SRR14901902	SRR14901900	SRR14901899	SRR14901898
SRR14901897	SRR14901896	SRR14901895	SRR14901883	SRR14901925	SRR14901924	SRR14901922	SRR14901921
SRR14901920	SRR14901919	SRR14901918	SRR14901917	SRR14901916	SRR14901894	SRR14901893	SRR14901892
SRR14901891	SRR14901889	SRR14901888	SRR14901887	SRR14901886	SRR14901885	SRR14901864	SRR14901863
SRR14916082	SRR14916081	SRR14916072	SRR14916080	SRR14916079	SRR14916078	SRR14916077	SRR14916076
SRR14916075	SRR14916074	SRR14916073	SRR14916071	SRR14924059	SRR14924058	SRR14924066	SRR14924065
SRR14924064	SRR14924072	SRR14924073	SRR14924071	SRR14924070	SRR14924069	SRR14924068	SRR14924067
SRR14924062	SRR14924063	SRR14924061	SRR14924060	SRR14924078	SRR14924079	SRR14924077	SRR14924076
SRR14924075	SRR14924074	SRR14933567	SRR14933566	SRR14933563	SRR14933562	SRR14933561	SRR14933560
SRR14933559	SRR14933558	SRR14933557	SRR14933556	SRR14933565	SRR14933564	SRR15039797	SRR15039796
SRR15039795	SRR15039794	SRR15039792	SRR15039791	SRR15043472	SRR15043471	SRR15043470	SRR15043469
SRR15043468	SRR15043467	SRR15043466	SRR15043465	SRR15043464	SRR15043463	SRR15043462	SRR15043461
SRR15089848	SRR15089847	SRR15089846	SRR15089845	SRR15089844	SRR15089843	SRR15089842	SRR15089841
SRR15089840	SRR15089839	SRR15089838	SRR15089837	SRR15095100	SRR15095099	SRR15095098	SRR15095097
SRR15095096	SRR15095101	SRR15100382	SRR15100381	SRR15100380	SRR15100379	SRR15100378	SRR15100377
SRR15168303	SRR15168302	SRR15168301	SRR15168300	SRR15168299	SRR15168298	SRR15168297	SRR15168296
SRR15168295	SRR15168294	SRR15168293	SRR15168292	SRR15168291	SRR15168290	SRR15168289	SRR15168288
SRR15168287	SRR15168286	SRR15183208	SRR15183207	SRR15183204	SRR15183203	SRR15183202	SRR15183201
SRR15183200	SRR15183199	SRR15183198	SRR15183197	SRR15183206	SRR15183205	SRR15184083	SRR15184082
SRR15184079	SRR15184078	SRR15184077	SRR15184076	SRR15184075	SRR15184074	SRR15184073	SRR15184072
SRR15184081	SRR15184080	SRR15184113	SRR15184112	SRR15184109	SRR15184108	SRR15184107	SRR15184106
SRR15184105	SRR15184104	SRR15184103	SRR15184102	SRR15184111	SRR15184110	SRR15195931	SRR15195930
SRR15195927	SRR15195926	SRR15195925	SRR15195924	SRR15195923	SRR15195922	SRR15195921	SRR15195920
SRR15195929	SRR15195928	SRR15210473	SRR15210472	SRR15210471	SRR15210470	SRR15210469	SRR15210468
SRR15210489	SRR15210488	SRR15210487	SRR15210486	SRR15210485	SRR15210484	SRR15210483	SRR15210482
SRR15210481	SRR15210480	SRR15210479	SRR15210478	SRR15210477	SRR15210476	SRR15210475	SRR15210474
SRR15215185	SRR15215184	SRR15215197	SRR15215196	SRR15215195	SRR15215194	SRR15215193	SRR15215192
SRR15249854	SRR15249853	SRR15249852	SRR15249851	SRR15249850	SRR15249855	SRR15275173	SRR15275172
SRR15275169	SRR15275168	SRR15275167	SRR15275166	SRR15275165	SRR15275164	SRR15275163	SRR15275162
SRR15275171	SRR15275170	SRR15291755	SRR15291754	SRR15291745	SRR15291744	SRR15291743	SRR15291742
SRR15291741	SRR15291740	SRR15291739	SRR15291738	SRR15291753	SRR15291752	SRR15291751	SRR15291750
SRR15291749	SRR15291748	SRR15291747	SRR15291746	SRR15312640	SRR15312639	SRR15312636	SRR15312635
SRR15312634	SRR15312633	SRR15312632	SRR15312631	SRR15312630	SRR15312629	SRR15312638	SRR15312637
SRR15327883	SRR15327877	SRR15327878	SRR15327879	SRR15327880	SRR15327884	SRR15327885	SRR15327886
SRR15327887	SRR15327888	SRR15327873	SRR15327874	SRR15327875	SRR15327876	SRR15327881	SRR15327882
SRR15327891	SRR15327892	SRR15327893	SRR15327894	SRR15327895	SRR15327896	SRR15327897	SRR15327898
SRR15327899	SRR15327900	SRR15327901	SRR15327902	SRR15327903	SRR15327904	SRR15327905	SRR15327906
SRR15327907	SRR15327908	SRR15327909	SRR15327910	SRR15327911	SRR15327912	SRR15327889	SRR15327890
SRR15412094	SRR15412093	SRR15412092	SRR15412091	SRR15412090	SRR15412089	SRR15372782	SRR15372781
SRR15372780	SRR15372779	SRR15372778	SRR15372777	SRR15372776	SRR15372775	SRR15372774	SRR15374734
SRR15374733	SRR15374722	SRR15374721	SRR15374720	SRR15374719	SRR15374718	SRR15374717	SRR15374716
SRR15374715	SRR15374732	SRR15374731	SRR15374730	SRR15374729	SRR15374728	SRR15374727	SRR15374726

SRR15374725	SRR15374724	SRR15374723	SRR15498085	SRR15498086	SRR15498087	SRR15498088	SRR15498089
SRR15498090	SRR15501831	SRR15501832	SRR15501833	SRR15501860	SRR15501861	SRR15501862	SRR15501863
SRR15501864	SRR15501847	SRR15501838	SRR15501839	SRR15501840	SRR15501841	SRR15501842	SRR15501843
SRR15501844	SRR15501845	SRR15501846	SRR15501848	SRR15501849	SRR15501850	SRR15501851	SRR15501852
SRR15501853	SRR15501854	SRR15501855	SRR15501856	SRR15501857	SRR15501858	SRR15501859	SRR15501829
SRR15501830	SRR15501834	SRR15501835	SRR15501836	SRR15501837	SRR15594027	SRR15594019	SRR15594020
SRR15594021	SRR15594022	SRR15594023	SRR15594024	SRR15594025	SRR15594026	SRR15619231	SRR15619226
SRR15619227	SRR15619228	SRR15619229	SRR15619230	SRR15694344	SRR15694343	SRR15694342	SRR15694341
SRR15694346	SRR15694345	SRR15700171	SRR15700172	SRR15700173	SRR15700174	SRR15700175	SRR15700176
SRR15700177	SRR15718863	SRR15718862	SRR15718861	SRR15718860	SRR15851437	SRR15851436	SRR15851425
SRR15851414	SRR15851403	SRR15851392	SRR15851381	SRR15851370	SRR15851367	SRR15851366	SRR15851435
SRR15851434	SRR15851433	SRR15851432	SRR15851431	SRR15851430	SRR15851429	SRR15851428	SRR15851427
SRR15851426	SRR15851424	SRR15851423	SRR15851422	SRR15851421	SRR15851420	SRR15851419	SRR15851418
SRR15851417	SRR15851416	SRR15851415	SRR15851413	SRR15851412	SRR15851411	SRR15851410	SRR15851409
SRR15851408	SRR15851407	SRR15851406	SRR15851405	SRR15851404	SRR15851402	SRR15851401	SRR15851400
SRR15851399	SRR15851398	SRR15851397	SRR15851396	SRR15851395	SRR15851394	SRR15851393	SRR15851391
SRR15851390	SRR15851389	SRR15851388	SRR15851387	SRR15851386	SRR15851385	SRR15851384	SRR15851383
SRR15851382	SRR15851380	SRR15851379	SRR15851378	SRR15851377	SRR15851376	SRR15851375	SRR15851374
SRR15851373	SRR15851372	SRR15851371	SRR15851369	SRR15851368	SRR15835811	SRR15835810	SRR15835809
SRR15835808	SRR15835807	SRR15835806	SRR15841156	SRR15841157	SRR15841158	SRR15841159	SRR15841160
SRR15841161	SRR15841162	SRR15841163	SRR15841126	SRR15841127	SRR15841128	SRR15841129	SRR15841130
SRR15841131	SRR15841132	SRR15841133	SRR15841134	SRR15841135	SRR15841136	SRR15841137	SRR15841138
SRR15841139	SRR15841140	SRR15841141	SRR15841142	SRR15841143	SRR15841144	SRR15841145	SRR15841152
SRR15841146	SRR15841147	SRR15841148	SRR15841149	SRR15841150	SRR15841151	SRR15841153	SRR15841154
SRR15841155	SRR15841124	SRR15841125	SRR15871875	SRR15871876	SRR15871877	SRR15871878	SRR15871879
SRR15871880	SRR15871881	SRR15871882	SRR15871883	SRR15871884	SRR15871885	SRR15871887	SRR15871886
SRR15871888	SRR15871889	SRR15871890	SRR15871871	SRR15871872	SRR15871873	SRR15871874	SRR15902243
SRR15902242	SRR15902263	SRR15902252	SRR15902249	SRR15902248	SRR15902247	SRR15902246	SRR15902245
SRR15902244	SRR15902241	SRR15902240	SRR15902271	SRR15902270	SRR15902269	SRR15902268	SRR15902267
SRR15902266	SRR15902265	SRR15902264	SRR15902262	SRR15902261	SRR15902260	SRR15902259	SRR15902258
SRR15902257	SRR15902256	SRR15902255	SRR15902254	SRR15902253	SRR15902251	SRR15902250	SRR15902211
SRR15902210	SRR15902231	SRR15902220	SRR15902217	SRR15902216	SRR15902215	SRR15902214	SRR15902213
SRR15902212	SRR15902209	SRR15902208	SRR15902239	SRR15902238	SRR15902237	SRR15902236	SRR15902235
SRR15902234	SRR15902233	SRR15902232	SRR15902230	SRR15902229	SRR15902228	SRR15902227	SRR15902226
SRR15902225	SRR15902224	SRR15902223	SRR15902222	SRR15902221	SRR15902219	SRR15902218	SRR16097994
SRR16097987	SRR16097988	SRR16097989	SRR16097990	SRR16097991	SRR16097992	SRR16097993	SRR16167564
SRR16167563	SRR16167562	SRR16167561	SRR16167560	SRR16167558	SRR16167557	SRR16167556	SRR16167555
SRR16167554	SRR16167553	SRR16167552	SRR16167551	SRR16167550	SRR16167549	SRR16204458	SRR16204459
SRR16204460	SRR16204461	SRR16204462	SRR16204463	SRR16290032	SRR16290031	SRR16290030	SRR16290029
SRR16290028	SRR16290027	SRR16290026	SRR16290025	SRR16350177	SRR16350176	SRR16350175	SRR16350174
SRR16350173	SRR16350172	SRR17088595	SRR17088594	SRR17088583	SRR17088572	SRR17088561	SRR17088550
SRR17088545	SRR17088544	SRR17088543	SRR17088542	SRR17088593	SRR17088592	SRR17088591	SRR17088590
SRR17088589	SRR17088588	SRR17088587	SRR17088586	SRR17088585	SRR17088584	SRR17088582	SRR17088581
SRR17088580	SRR17088579	SRR17088578	SRR17088577	SRR17088576	SRR17088575	SRR17088574	SRR17088573
SRR17088571	SRR17088570	SRR17088569	SRR17088568	SRR17088567	SRR17088566	SRR17088565	SRR17088564
SRR17088563	SRR17088562	SRR17088560	SRR17088559	SRR17088558	SRR17088557	SRR17088556	SRR17088555
SRR17088554	SRR17088553	SRR17088552	SRR17088551	SRR17088549	SRR17088548	SRR17088547	SRR17088546
SRR16362236	SRR16362235	SRR16362234	SRR16362233	SRR16362232	SRR16362231	SRR16591582	SRR16591583
SRR16550208	SRR16550207	SRR16550206	SRR16550205	SRR16550204	SRR16550203	SRR16550202	SRR16550201
SRR16643521	SRR16643520	SRR16643519	SRR16643518	SRR16706437	SRR16706438	SRR16706449	SRR16706451
SRR16706452	SRR16706445	SRR16706446	SRR16706447	SRR16706448	SRR16706450	SRR16815837	SRR16815846
SRR16815845	SRR16815843	SRR16815842	SRR16815841	SRR16815840	SRR16815839	SRR16815838	SRR16815848
SRR16815847	SRR16815844	SRR16912827	SRR16912825	SRR16912824	SRR16912823	SRR16912822	SRR16912826
SRR16938279	SRR16938280	SRR16938288	SRR16938285	SRR16938286	SRR16938287	SRR16938282	SRR16938283
SRR16938284	SRR16938277	SRR16938278	SRR16938281	SRR16953008	SRR16953007	SRR16953006	SRR16953005
SRR16953004	SRR16953003	SRR16953002	SRR16953001	SRR16953000	SRR16975863	SRR16975864	SRR16975865
SRR16975866	SRR16975867	SRR16975868	SRR16975869	SRR16975870	SRR16975871	SRR16975872	SRR16975873
SRR16986548	SRR16986547	SRR16986546	SRR16986545	SRR16986544	SRR16986543	SRR16986542	SRR16986541

SRR16986540	SRR16991086	SRR16991085	SRR16991084	SRR16991083	SRR16991082	SRR16991081	SRR17002446
SRR17002447	SRR17002448	SRR17002449	SRR17002450	SRR17002451	SRR17012357	SRR17012358	SRR17012359
SRR17012360	SRR17012361	SRR17012362	SRR17012363	SRR17012364	SRR17012365	SRR17012366	SRR17012367
SRR17012368	SRR17027046	SRR17027047	SRR17027048	SRR17027049	SRR17027050	SRR17027051	SRR17027052
SRR17027053	SRR17027054	SRR17027055	SRR17027056	SRR17027057	SRR17055239	SRR17055240	SRR17055241
SRR17055236	SRR17055237	SRR17055238	SRR17055382	SRR17055383	SRR17055384	SRR17055385	SRR17055386
SRR17055387	SRR17055388	SRR17055389	SRR17055390	SRR17055391	SRR17106042	SRR17106043	SRR17106044
SRR17106045	SRR17139373	SRR17139372	SRR17139371	SRR17139370	SRR17139369	SRR17139368	SRR17144013
SRR17144014	SRR17144015	SRR17144016	SRR17144017	SRR17144018	SRR17191931	SRR17191930	SRR17191929
SRR17191928	SRR17191927	SRR17191926	SRR17191925	SRR17191924	SRR18210216	SRR18210215	SRR18210214
SRR18210213	SRR18210212	SRR18210211	SRR17256774	SRR17256773	SRR17256772	SRR17256771	SRR17256770
SRR17256769	SRR17255120	SRR17265350	SRR17265351	SRR17265352	SRR17265353	SRR17265354	SRR17265355
SRR17265356	SRR17265357	SRR17265358	SRR17265359	SRR17265360	SRR17265361	SRR17265362	SRR17265363
SRR17265364	SRR17265365	SRR17265366	SRR17265367	SRR17265368	SRR17265369	SRR17265370	SRR17265371
SRR17265372	SRR17265373	SRR17282393	SRR17282394	SRR17282395	SRR17282396	SRR17282397	SRR17282398
SRR17282399	SRR17282400	SRR17282401	SRR17282402	SRR17282403	SRR17282404	SRR17282405	SRR17282406
SRR17282407	SRR17282408	SRR17282409	SRR17282410	SRR17282412	SRR17282411	SRR17282413	SRR17282414
SRR17282415	SRR17282416	SRR17282417	SRR17282418	SRR17282420	SRR17282419	SRR17282421	SRR17282422
SRR17282423	SRR17282424	SRR17282425	SRR17282426	SRR17282427	SRR17282428	SRR17282429	SRR17282430
SRR17282431	SRR17282432	SRR17282433	SRR17282434	SRR17282435	SRR17282436	SRR17282437	SRR17282438
SRR17282440	SRR17282439	SRR17318647	SRR17318646	SRR17318645	SRR17318644	SRR17318643	SRR17318642
SRR17297767	SRR17297768	SRR17297769	SRR17297770	SRR17297771	SRR17297772	SRR17331986	SRR17331987
SRR17331988	SRR17331993	SRR17331989	SRR17331992	SRR17331990	SRR17331991	SRR17331994	SRR17331995
SRR17534274	SRR17534269	SRR17534268	SRR17534267	SRR17534266	SRR17534273	SRR17534272	SRR17534271
SRR17534270	SRR17591868	SRR17591867	SRR17591864	SRR17591863	SRR17591862	SRR17591861	SRR17591860
SRR17591859	SRR17591858	SRR17591857	SRR17591866	SRR17591865	SRR17599574	SRR17599571	SRR17599572
SRR17599573	SRR17599575	SRR17599576	SRR17599577	SRR17599578	SRR17599582	SRR17599579	SRR17599580
SRR17599581	SRR17599583	SRR17599584	SRR17599585	SRR17599586	SRR17599590	SRR17599587	SRR17599588
SRR17599589	SRR17599591	SRR17599592	SRR17599593	SRR17599594	SRR17599987	SRR17599984	SRR17599985
SRR17599986	SRR17599988	SRR17599989	SRR17599990	SRR17599991	SRR17599995	SRR17599992	SRR17599993
SRR17599994	SRR17599996	SRR17599997	SRR17599998	SRR17599999	SRR17600003	SRR17600000	SRR17600001
SRR17600002	SRR17600004	SRR17600005	SRR17600006	SRR17600007	SRR17675193	SRR17675192	SRR17675189
SRR17675188	SRR17675187	SRR17675186	SRR17675185	SRR17675184	SRR17675183	SRR17675182	SRR17675191
SRR17675190	SRR17631831	SRR17631821	SRR17631827	SRR17631835	SRR17631836	SRR17631828	SRR17631829
SRR17631830	SRR17631837	SRR17631838	SRR17631822	SRR17631832	SRR17631833	SRR17631834	SRR17631823
SRR17631824	SRR17631825	SRR17631826	SRR17640024	SRR17640025	SRR17640026	SRR17640027	SRR17640028
SRR17640029	SRR17640030	SRR17640031	SRR17640032	SRR17640033	SRR17640034	SRR17640035	SRR17640036
SRR17640037	SRR17640038	SRR17640039	SRR17640040	SRR17640041	SRR17640042	SRR17640043	SRR17646261
SRR17646262	SRR17646263	SRR17646264	SRR17646265	SRR17646266	SRR17646267	SRR17646268	SRR17646269
SRR17651365	SRR17651364	SRR17651363	SRR17651362	SRR17651361	SRR17651360	SRR17664315	SRR17664316
SRR17664317	SRR17664322	SRR17664318	SRR17664321	SRR17664319	SRR17664320	SRR17664323	SRR17664324
SRR17711253	SRR17711252	SRR17711251	SRR17711250	SRR17714804	SRR17714803	SRR17714800	SRR17714799
SRR17714798	SRR17714797	SRR17714796	SRR17714795	SRR17714794	SRR17714793	SRR17714802	SRR17714801
SRR17779295	SRR17779296	SRR17779297	SRR17779298	SRR17779299	SRR17779300	SRR18028203	SRR18028202
SRR18028191	SRR18028183	SRR18028182	SRR18028181	SRR18028180	SRR18028179	SRR18028178	SRR18028177
SRR18028201	SRR18028200	SRR18028199	SRR18028198	SRR18028197	SRR18028196	SRR18028195	SRR18028194
SRR18028193	SRR18028192	SRR18028190	SRR18028189	SRR18028188	SRR18028187	SRR18028186	SRR18028185
SRR18028184	SRR17869080	SRR17869081	SRR17869082	SRR17869083	SRR17869084	SRR17869085	SRR17956008
SRR17956007	SRR17956006	SRR17956005	SRR17956014	SRR17956013	SRR17970844	SRR17970845	SRR17970846
SRR17970847	SRR17970848	SRR17970849	SRR17970850	SRR17970851	SRR17970852	SRR17970853	SRR17970854
SRR17970855	SRR17970856	SRR17970857	SRR17970858	SRR17970859	SRR18076842	SRR18076843	SRR18076844
SRR18076845	SRR18076846	SRR18076847	SRR18076848	SRR18076849	SRR18076850	SRR18076851	SRR18076852
SRR18076853	SRR18076854	SRR18076855	SRR18076856	SRR18076857	SRR18076858	SRR18076859	SRR18076860
SRR18087789	SRR18087788	SRR18087781	SRR18087780	SRR18087779	SRR18087778	SRR18087777	SRR18087776
SRR18087775	SRR18087774	SRR18087787	SRR18087786	SRR18087785	SRR18087784	SRR18087783	SRR18087782
SRR18090529	SRR18090530	SRR18090531	SRR18090532	SRR18090533	SRR18090534	SRR18090503	SRR18090504
SRR18090505	SRR18090506	SRR18090507	SRR18090508	SRR18090510	SRR18090512	SRR18090514	SRR18090515
SRR18090516	SRR18090517	SRR18090518	SRR18090519	SRR18090521	SRR18090523	SRR18090560	SRR18090561

SRR18090562	SRR18090563	SRR18090564	SRR18090565	SRR18090566	SRR18090567	SRR18090535	SRR18090536
SRR18090537	SRR18090538	SRR18090540	SRR18090542	SRR18090544	SRR18090546	SRR18099066	SRR18099067
SRR18099068	SRR18099069	SRR18099070	SRR18099071	SRR18099072	SRR18099073	SRR513212	SRR513213
SRR513214	SRR513215	SRR513216	SRR513217	SRR513218	SRR513219	SRR513229	SRR513230
SRR513231	SRR513232	SRR513233	SRR513234	SRR513235	SRR513236	SRR513237	SRR513238
SRR513239	SRR513240	SRR513241	SRR513242	SRR513243	SRR513244	SRR513245	SRR513246
SRR513247	SRR513248	SRR513249	SRR513250	SRR513251	SRR513252	SRR513253	SRR513254
SRR513255	SRR513256	SRR513257	SRR513258	SRR513259	SRR513260	SRR513261	SRR513262
SRR513263	SRR513264	SRR513265	SRR513266	SRR513267	SRR513268	SRR513269	SRR513270
SRR513271	SRR513272	SRR513273	SRR513274	SRR513275	SRR513276	SRR513277	SRR513278
SRR513279	SRR513280	SRR513281	SRR513282	SRR513283	SRR513284	SRR513285	SRR513286
SRR513287	SRR513288	SRR513289	SRR513290	SRR513291	SRR513292	SRR513293	SRR513294
SRR513295	SRR513296	SRR513297	SRR513298	SRR513299	SRR513300	SRR513301	SRR513302
SRR513303	SRR513304	SRR513305	SRR513306	SRR513307	SRR513308	SRR513309	SRR513310
SRR513328	SRR513329	SRR513330	SRR513331	SRR513332	SRR513333	SRR513334	SRR513335
SRR513336	SRR513337	SRR513338	SRR513339	SRR513340	SRR513341	SRR513342	SRR513343
SRR513344	SRR513345	SRR513346	SRR513347	SRR513348	SRR513349	SRR513350	SRR513351
SRR513352	SRR513353	SRR513354	SRR513355	SRR513356	SRR513357	SRR513358	SRR513359
SRR513360	SRR513361	SRR513362	SRR513363	SRR513364	SRR513365	SRR513366	SRR513367
SRR513368	SRR513369	SRR513370	SRR513879	SRR513882	SRR513883	SRR513886	SRR513889
SRR513890	SRR513894	SRR513895	SRR513896	SRR513899	SRR513900	SRR513902	SRR513905
SRR513912	SRR513915	SRR513929	SRR516752	SRR516753	SRR516754	SRR516755	SRR516756
SRR516757	SRR516758	SRR516759	SRR516760	SRR516761	SRR516762	SRR516763	SRR516764
SRR516765	SRR516766	SRR516767	SRR516768	SRR516769	SRR516770	SRR516771	SRR516772
SRR516773	SRR516774	SRR516775	SRR516776	SRR516777	SRR516778	SRR516779	SRR516780
SRR516781	SRR1610284	SRR1610285	SRR1610286	SRR1610287	SRR1610415	SRR1610288	SRR1610418
SRR1610425	SRR1612608	SRR1612610	SRR1610417	SRR1610419	SRR1614094	SRR1612622	SRR1612623
SRR1612624	SRR1612616	SRR1612621	SRR1612625	SRR1612830	SRR1612831	SRR1612832	SRR1612833
SRR1612834	SRR18156400	SRR18156396	SRR18156394	SRR18156392	SRR18156390	SRR18156398	SRR18246464
SRR18246463	SRR18246462	SRR18246461	SRR18246460	SRR18246459	SRR18246458	SRR18246457	SRR18246456
SRR18192011	SRR18192012	SRR18192013	SRR18192014	SRR18192015	SRR18192016	SRR18192017	SRR18192018
SRR18192019	SRR18192020	SRR18192021	SRR18192022	SRR18192023	SRR18192024	SRR18192025	SRR18192026
SRR18192027	SRR18192028	SRR18192029	SRR18192030	SRR18192031	SRR18192032	SRR18192033	SRR18192034
SRR18192035	SRR18192036	SRR18192037	SRR18192038	SRR18192039	SRR18192040	SRR18192041	SRR18192042
SRR18192043	SRR18192044	SRR18192045	SRR18192046	SRR18192047	SRR18192048	SRR18192007	SRR18192008
SRR18192009	SRR18192010	SRR18272534	SRR18272533	SRR18272532	SRR18280728	SRR18280727	SRR18280716
SRR18280705	SRR18280702	SRR18280701	SRR18280700	SRR18280699	SRR18280698	SRR18280697	SRR18280726
SRR18280725	SRR18280724	SRR18280723	SRR18280722	SRR18280721	SRR18280720	SRR18280719	SRR18280718
SRR18280717	SRR18280715	SRR18280714	SRR18280713	SRR18280712	SRR18280711	SRR18280710	SRR18280709
SRR18280708	SRR18280707	SRR18280706	SRR18280704	SRR18280703	SRR18493888	SRR18493887	SRR18493865
SRR18493854	SRR18493882	SRR18493879	SRR18493878	SRR18493877	SRR18493876	SRR18493874	SRR18493886
SRR18493875	SRR18493873	SRR18493872	SRR18493871	SRR18493870	SRR18493869	SRR18493868	SRR18493867
SRR18493866	SRR18493864	SRR18493863	SRR18493862	SRR18493861	SRR18493860	SRR18493859	SRR18493858
SRR18493857	SRR18493856	SRR18493855	SRR18493853	SRR18493852	SRR18493851	SRR18493850	SRR18493849
SRR18493848	SRR18493847	SRR18493885	SRR18493884	SRR18493883	SRR18493881	SRR18493880	SRR18494243
SRR18494242	SRR18494231	SRR18494220	SRR18494209	SRR18494198	SRR18494187	SRR18494176	SRR18494167
SRR18494166	SRR18494241	SRR18494240	SRR18494239	SRR18494238	SRR18494237	SRR18494236	SRR18494235
SRR18494234	SRR18494233	SRR18494232	SRR18494230	SRR18494229	SRR18494228	SRR18494227	SRR18494226
SRR18494225	SRR18494224	SRR18494223	SRR18494222	SRR18494221	SRR18494219	SRR18494218	SRR18494217
SRR18494216	SRR18494215	SRR18494214	SRR18494213	SRR18494212	SRR18494211	SRR18494210	SRR18494208
SRR18494207	SRR18494206	SRR18494205	SRR18494204	SRR18494203	SRR18494202	SRR18494201	SRR18494200
SRR18494199	SRR18494197	SRR18494196	SRR18494195	SRR18494194	SRR18494193	SRR18494192	SRR18494191
SRR18494190	SRR18494189	SRR18494188	SRR18494186	SRR18494185	SRR18494184	SRR18494183	SRR18494182
SRR18494181	SRR18494180	SRR18494179	SRR18494178	SRR18494177	SRR18494175	SRR18494174	SRR18494173
SRR18494172	SRR18494171	SRR18494170	SRR18494169	SRR18494168	SRR18399972	SRR18399973	SRR18399974
SRR18399975	SRR18399976	SRR18399977	SRR18399978	SRR18399979	SRR18399980	SRR18399981	SRR18399982
SRR18399983	SRR18399984	SRR18399985	SRR18399986	SRR18399987	SRR18399988	SRR18399989	SRR18474132
SRR18474133	SRR18474134	SRR18474135	SRR18474136	SRR18474137	SRR18474138	SRR18474139	SRR18474140

SRR18474141	SRR18474142	SRR18474143	SRR18531201	SRR18531200	SRR18531199	SRR18531198	SRR18531197
SRR18531196	SRR18531195	SRR18531194	SRR18531193	SRR18531192	SRR18531225	SRR18531224	SRR18531222
SRR18531221	SRR18531220	SRR18531219	SRR18531218	SRR18531217	SRR18531216	SRR18531215	SRR18531223
SRR18533981	SRR18533980	SRR18533979	SRR18533978	SRR18533977	SRR18533976	SRR18587417	SRR18587416
SRR18587415	SRR18587414	SRR18587413	SRR18587412	SRR18645190	SRR18645189	SRR18645178	SRR18645173
SRR18645172	SRR18645171	SRR18645170	SRR18645169	SRR18645168	SRR18645167	SRR18645188	SRR18645187
SRR18645186	SRR18645185	SRR18645184	SRR18645183	SRR18645182	SRR18645181	SRR18645180	SRR18645179
SRR18645177	SRR18645176	SRR18645175	SRR18645174	SRR18705627	SRR18705626	SRR18705601	SRR18705590
SRR18705579	SRR18705568	SRR18705557	SRR18705546	SRR18705535	SRR18705524	SRR18705625	SRR18705614
SRR18705609	SRR18705608	SRR18705607	SRR18705606	SRR18705605	SRR18705604	SRR18705603	SRR18705602
SRR18705600	SRR18705599	SRR18705598	SRR18705597	SRR18705596	SRR18705595	SRR18705594	SRR18705593
SRR18705592	SRR18705591	SRR18705589	SRR18705588	SRR18705587	SRR18705586	SRR18705585	SRR18705584
SRR18705583	SRR18705582	SRR18705581	SRR18705580	SRR18705578	SRR18705577	SRR18705576	SRR18705575
SRR18705574	SRR18705573	SRR18705572	SRR18705571	SRR18705570	SRR18705569	SRR18705567	SRR18705566
SRR18705565	SRR18705564	SRR18705563	SRR18705562	SRR18705561	SRR18705560	SRR18705559	SRR18705558
SRR18705556	SRR18705555	SRR18705554	SRR18705553	SRR18705552	SRR18705551	SRR18705550	SRR18705549
SRR18705548	SRR18705547	SRR18705545	SRR18705544	SRR18705543	SRR18705542	SRR18705541	SRR18705540
SRR18705539	SRR18705538	SRR18705537	SRR18705536	SRR18705534	SRR18705533	SRR18705532	SRR18705531
SRR18705530	SRR18705529	SRR18705528	SRR18705527	SRR18705526	SRR18705525	SRR18705523	SRR18705522
SRR18705521	SRR18705520	SRR18705519	SRR18705518	SRR18705517	SRR18705516	SRR18705515	SRR18705514
SRR18705624	SRR18705623	SRR18705622	SRR18705621	SRR18705620	SRR18705619	SRR18705618	SRR18705617
SRR18705616	SRR18705615	SRR18705613	SRR18705612	SRR18705611	SRR18705610	SRR18700715	SRR18700714
SRR18700721	SRR18700708	SRR18700697	SRR18700686	SRR18700675	SRR18700674	SRR18700673	SRR18700672
SRR18700731	SRR18700730	SRR18700729	SRR18700728	SRR18700727	SRR18700726	SRR18700725	SRR18700724
SRR18700723	SRR18700722	SRR18700720	SRR18700719	SRR18700718	SRR18700717	SRR18700716	SRR18700713
SRR18700712	SRR18700711	SRR18700710	SRR18700709	SRR18700707	SRR18700706	SRR18700705	SRR18700704
SRR18700703	SRR18700702	SRR18700701	SRR18700700	SRR18700699	SRR18700698	SRR18700696	SRR18700695
SRR18700694	SRR18700693	SRR18700692	SRR18700691	SRR18700690	SRR18700689	SRR18700688	SRR18700687
SRR18700685	SRR18700684	SRR18700683	SRR18700682	SRR18700681	SRR18700680	SRR18700679	SRR18700678
SRR18700677	SRR18700676	SRR18717090	SRR18717091	SRR18717092	SRR18717093	SRR18717094	SRR18717095
SRR18717096	SRR18717097	SRR18717098	SRR18717099	SRR18717100	SRR18717101	SRR18752452	SRR18752451
SRR18752450	SRR18752449	SRR18752454	SRR18752453	SRR18767125	SRR18767124	SRR18767123	SRR18767122
SRR18767121	SRR18767120	SRR18840440	SRR18840439	SRR18840428	SRR18840423	SRR18840422	SRR18840421
SRR18840420	SRR18840419	SRR18840418	SRR18840417	SRR18840438	SRR18840437	SRR18840436	SRR18840435
SRR18840434	SRR18840433	SRR18840432	SRR18840431	SRR18840430	SRR18840429	SRR18840427	SRR18840426
SRR18840425	SRR18840424	SRR18907625	SRR18907626	SRR18907609	SRR18907610	SRR18907611	SRR18907612
SRR18907613	SRR18907614	SRR18907615	SRR18907616	SRR18907617	SRR18907618	SRR18907619	SRR18907620
SRR18907621	SRR18907622	SRR18907623	SRR18907624	SRR18907627	SRR18907628	SRR18920769	SRR18920770
SRR18920771	SRR18920772	SRR18920773	SRR18920774	SRR18920775	SRR18920776	SRR18920777	SRR18920778
SRR18920779	SRR18920780	SRR18920781	SRR18920782	SRR18920783	SRR18920784	SRR18923152	SRR18923153
SRR18923154	SRR18923155	SRR18923156	SRR18923157	SRR18923158	SRR18923159	SRR18923160	SRR18923161
SRR18923162	SRR18923163	SRR18923164	SRR18923165	SRR18923166	SRR18923167	SRR18923168	SRR18923169
SRR18923170	SRR18923171	SRR18923172	SRR18923173	SRR18923174	SRR18923175	SRR18923176	SRR18923177
SRR18923178	SRR18923179	SRR18923180	SRR18923181	SRR18923182	SRR18923183	SRR18923184	SRR18923185
SRR18923186	SRR18923187	SRR18923188	SRR18923189	SRR18923190	SRR18923191	SRR18923192	SRR18923193
SRR18923194	SRR18922772	SRR18922773	SRR18922774	SRR18922775	SRR18922776	SRR18922777	SRR18922778
SRR18922779	SRR18922780	SRR18922781	SRR18922782	SRR18922783	SRR18922784	SRR18922785	SRR18922786
SRR18922787	SRR18922788	SRR18922789	SRR18922790	SRR18922791	SRR18922792	SRR18922793	SRR18922794
SRR18922795	SRR18922796	SRR18922797	SRR18922798	SRR18922799	SRR18922800	SRR18922801	SRR18922802
SRR18922803	SRR18922804	SRR18922805	SRR18922806	SRR18922807	SRR18922808	SRR18938186	SRR18938180
SRR18938169	SRR18938164	SRR18938163	SRR18938185	SRR18938184	SRR18938183	SRR18938182	SRR18938181
SRR18938179	SRR18938178	SRR18938177	SRR18938175	SRR18938176	SRR18938174	SRR18938173	SRR18938172
SRR18938171	SRR18938170	SRR18938168	SRR18938167	SRR18938166	SRR18938165	SRR19046514	SRR19046513
SRR19046512	SRR19046511	SRR19046510	SRR19046509	SRR19217640	SRR19217639	SRR19217638	SRR19217637
SRR19217636	SRR19217635	SRR19217634	SRR19217633	SRR19355466	SRR19355467	SRR19355468	SRR19355469
SRR19355470	SRR19355471	SRR19393067	SRR19393066	SRR19393059	SRR19393058	SRR19393057	SRR19393056
SRR19393055	SRR19393054	SRR19414715	SRR19414713	SRR19414712	SRR19414711	SRR19414710	SRR19414714
SRR19509839	SRR19509831	SRR19509838	SRR19509834	SRR19509837	SRR19509835	SRR19509836	SRR19509842

SRR19509873	SRR19509880	SRR19509874	SRR19509879	SRR19509875	SRR19509878	SRR19509876	SRR19509877
SRR19509881	SRR19509882	SRR19509896	SRR19509883	SRR19509895	SRR19509884	SRR19509894	SRR19509891
SRR19509893	SRR19509892	SRR19509897	SRR19509904	SRR19509898	SRR19509903	SRR19509899	SRR19509902
SRR19509900	SRR19509901	SRR19509905	SRR19509906	SRR19571482	SRR19571483	SRR19571484	SRR19571485
SRR19571489	SRR19571486	SRR19571487	SRR19571488	SRR19571490	SRR19571491	SRR19571492	SRR19571493
SRR19612823	SRR19612822	SRR19612817	SRR19612816	SRR19612815	SRR19612814	SRR19612813	SRR19612812
SRR19612811	SRR19612810	SRR19612821	SRR19612820	SRR19612819	SRR19612818	SRR19654616	SRR19654617
SRR19654618	SRR19654619	SRR19654620	SRR19654621	SRR19654622	SRR19654623	SRR19654624	SRR19654625
SRR19654626	SRR19654627	SRR19732943	SRR19732944	SRR19732945	SRR19732946	SRR19732947	SRR19732948
SRR19732949	SRR19732950	SRR19732951	SRR19732952	SRR19732953	SRR19732954	SRR19732955	SRR19732956
SRR19732957	SRR19732958	SRR19732959	SRR19732960	SRR19732961	SRR19732962	SRR19732963	SRR19732964
SRR19732965	SRR19757541	SRR19757540	SRR19757539	SRR19757538	SRR19757537	SRR19757536	SRR19907835
SRR19907836	SRR19907837	SRR19907838	SRR19907839	SRR19907840	SRR19913325	SRR19913324	SRR19913323
SRR19913322	SRR19913321	SRR19913320	SRR19913319	SRR19913318	SRR19913317	SRR19909239	SRR19909240
SRR19909241	SRR19909242	SRR19909243	SRR19909244	SRR19976736	SRR19976737	SRR19976738	SRR19976739
SRR19976740	SRR19976741	SRR20020633	SRR20020634	SRR20020635	SRR20020636	SRR20020637	SRR20020638
SRR20079700	SRR20079699	SRR20079694	SRR20079693	SRR20079692	SRR20079691	SRR20079690	SRR20079689
SRR20079688	SRR20079687	SRR20079698	SRR20079697	SRR20079696	SRR20079695	SRR20047268	SRR20047269
SRR20047270	SRR20047271	SRR20047272	SRR20047273	SRR20047274	SRR20047275	SRR20047276	SRR20290500
SRR20290499	SRR20290488	SRR20290477	SRR20290466	SRR20290455	SRR20290444	SRR20290433	SRR20290422
SRR20290417	SRR20290498	SRR20290497	SRR20290496	SRR20290495	SRR20290494	SRR20290493	SRR20290492
SRR20290491	SRR20290490	SRR20290489	SRR20290487	SRR20290486	SRR20290485	SRR20290484	SRR20290483
SRR20290482	SRR20290481	SRR20290480	SRR20290479	SRR20290478	SRR20290476	SRR20290475	SRR20290474
SRR20290473	SRR20290472	SRR20290471	SRR20290470	SRR20290469	SRR20290468	SRR20290467	SRR20290465
SRR20290464	SRR20073473	SRR20073472	SRR20073469	SRR20073468	SRR20073467	SRR20073466	SRR20073465
SRR20073464	SRR20073463	SRR20073462	SRR20073471	SRR20073470	SRR20738336	SRR20738335	SRR20738324
SRR20738316	SRR20738315	SRR20738314	SRR20738313	SRR20738312	SRR20738311	SRR20738310	SRR20738334
SRR20738333	SRR20738332	SRR20738331	SRR20738330	SRR20738329	SRR20738328	SRR20738327	SRR20738326
SRR20738325	SRR20738323	SRR20738322	SRR20738321	SRR20738320	SRR20738319	SRR20738318	SRR20738317
SRR20083851	SRR20083850	SRR20083849	SRR20083848	SRR20083847	SRR20083846	SRR20340069	SRR20340879
SRR20340888	SRR20647928	SRR20647927	SRR20647926	SRR20647925	SRR20647924	SRR20647923	SRR20647922
SRR20647921	SRR20647920	SRR20706677	SRR20706676	SRR20706665	SRR20706654	SRR20706643	SRR20706635
SRR20706634	SRR20706633	SRR20706632	SRR20706631	SRR20706675	SRR20706674	SRR20706673	SRR20706672
SRR20706671	SRR20706670	SRR20706669	SRR20706668	SRR20706667	SRR20706666	SRR20706664	SRR20706663
SRR20706662	SRR20706661	SRR20706660	SRR20706659	SRR20706658	SRR20706657	SRR20706656	SRR20706655
SRR20706653	SRR20706652	SRR20706651	SRR20706650	SRR20706649	SRR20706648	SRR20706647	SRR20706646
SRR20706645	SRR20706644	SRR20706642	SRR20706641	SRR20706640	SRR20706639	SRR20706638	SRR20706637
SRR20706636	SRR20701453	SRR20701452	SRR20701441	SRR20701430	SRR20701429	SRR20701428	SRR20701427
SRR20701426	SRR20701425	SRR20701424	SRR20701451	SRR20701450	SRR20701449	SRR20701448	SRR20701447
SRR20701446	SRR20701445	SRR20701444	SRR20701443	SRR20701442	SRR20701440	SRR20701439	SRR20701438
SRR20701437	SRR20701436	SRR20701435	SRR20701434	SRR20701433	SRR20701432	SRR20701431	SRR20689861
SRR20689863	SRR20830027	SRR20830028	SRR20830030	SRR20830029	SRR20830032	SRR20830031	SRR20830034
SRR20830033	SRR20830035	SRR20830036	SRR20830037	SRR20830038	SRR20830039	SRR20830040	SRR20830041
SRR20830042	SRR20830043	SRR20830044	SRR20830046	SRR20830045	SRR20830048	SRR20830047	SRR20830050
SRR20830049	SRR20830051	SRR20830052	SRR20830054	SRR20830053	SRR20830056	SRR20830055	SRR20830058
SRR20830057	SRR20830059	SRR20830060	SRR20830061	SRR20830062	SRR20830064	SRR20830063	SRR20830065
SRR20830066	SRR20830067	SRR20830068	SRR20830072	SRR20830071	SRR20830073	SRR20830074	SRR20830070
SRR20830069	SRR20830075	SRR20830076	SRR20830077	SRR20830078	SRR20830079	SRR20830080	SRR20982112
SRR20982111	SRR20982102	SRR20982101	SRR20982100	SRR20982099	SRR20982098	SRR20982097	SRR20982096
SRR21023585	SRR21023586	SRR21023587	SRR21023588	SRR21023589	SRR21023590	SRR21023591	SRR21023592
SRR21023593	SRR21023594	SRR21023595	SRR21023596	SRR21023597	SRR21023598	SRR21278554	SRR21278553
SRR21278550	SRR21278549	SRR21278548	SRR21278547	SRR21278546	SRR21278545	SRR21278544	SRR21278543
SRR21278552	SRR21278551	SRR21101581	SRR21101580	SRR21101582	SRR21101583	SRR21101585	SRR21101586
SRR21101584	SRR21101588	SRR21101587	SRR21101589	SRR21101590	SRR21315239	SRR21315240	SRR21315241
SRR21315242	SRR21315243	SRR21315244	SRR21384390	SRR21384389	SRR21384383	SRR21384382	SRR21384381
SRR21384380	SRR21384379	SRR21384378	SRR21384377	SRR21384376	SRR21384388	SRR21384387	SRR21384386
SRR21384385	SRR21384384	SRR21389694	SRR21389695	SRR21389696	SRR21389697	SRR21389698	SRR21389699
SRR21389700	SRR21389701	SRR21389702	SRR21389703	SRR21389704	SRR21389705	SRR21420622	SRR21420623

SRR21420624	SRR21420625	SRR21420626	SRR21420627	SRR21711052	SRR21711051	SRR21711040	SRR21711029
SRR21711018	SRR21711007	SRR21710996	SRR21710991	SRR21710990	SRR21710989	SRR21711050	SRR21711049
SRR21711048	SRR21711047	SRR21711046	SRR21711045	SRR21711044	SRR21711043	SRR21711042	SRR21711041
SRR21711039	SRR21711038	SRR21711037	SRR21711036	SRR21711035	SRR21711034	SRR21711033	SRR21711032
SRR21711031	SRR21711030	SRR21711028	SRR21711027	SRR21711026	SRR21711025	SRR21711024	SRR21711023
SRR21711022	SRR21711021	SRR21711020	SRR21711019	SRR21711017	SRR21711016	SRR21711015	SRR21711014
SRR21711013	SRR21711012	SRR21711011	SRR21711010	SRR21711009	SRR21711008	SRR21711006	SRR21711005
SRR21711004	SRR21711003	SRR21711002	SRR21711001	SRR21711000	SRR21710999	SRR21710998	SRR21710997
SRR21710995	SRR21710994	SRR21710993	SRR21710992	SRR21600995	SRR21600994	SRR21600993	SRR21600992
SRR21600991	SRR21600990	SRR21600989	SRR21600988	SRR21600987	SRR21669609	SRR21669608	SRR21669607
SRR21640142	SRR21640141	SRR21655996	SRR21655995	SRR21655926	SRR21655915	SRR21655904	SRR21655893
SRR21655882	SRR21655871	SRR21655953	SRR21655942	SRR21655994	SRR21655983	SRR21655972	SRR21655962
SRR21655961	SRR21655931	SRR21655930	SRR21655929	SRR21655928	SRR21655927	SRR21655925	SRR21655924
SRR21655923	SRR21655922	SRR21655921	SRR21655920	SRR21655919	SRR21655918	SRR21655917	SRR21655916
SRR21655914	SRR21655913	SRR21655912	SRR21655911	SRR21655910	SRR21655909	SRR21655908	SRR21655907
SRR21655906	SRR21655905	SRR21655903	SRR21655902	SRR21655901	SRR21655900	SRR21655899	SRR21655898
SRR21655897	SRR21655896	SRR21655895	SRR21655894	SRR21655892	SRR21655891	SRR21655890	SRR21655889
SRR21655888	SRR21655887	SRR21655886	SRR21655885	SRR21655884	SRR21655883	SRR21655881	SRR21655880
SRR21655879	SRR21655878	SRR21655877	SRR21655876	SRR21655875	SRR21655874	SRR21655873	SRR21655872
SRR21655870	SRR21655869	SRR21655868	SRR21655960	SRR21655959	SRR21655958	SRR21655957	SRR21655956
SRR21655955	SRR21655954	SRR21655952	SRR21655951	SRR21655950	SRR21655949	SRR21655948	SRR21655947
SRR21655946	SRR21655945	SRR21655944	SRR21655943	SRR21655941	SRR21655940	SRR21655939	SRR21655938
SRR21655937	SRR21655936	SRR21655935	SRR21655934	SRR21655933	SRR21655932	SRR21655993	SRR21655992
SRR21655991	SRR21655990	SRR21655989	SRR21655988	SRR21655987	SRR21655986	SRR21655985	SRR21655984
SRR21655982	SRR21655981	SRR21655980	SRR21655979	SRR21655978	SRR21655977	SRR21655976	SRR21655975
SRR21655974	SRR21655973	SRR21655971	SRR21655970	SRR21655969	SRR21655968	SRR21655967	SRR21655966
SRR21655965	SRR21655964	SRR21655963	SRR21674611	SRR21674612	SRR21674613	SRR21674614	SRR21674615
SRR21674616	SRR21674617	SRR21674618	SRR21674619	SRR21696927	SRR21696926	SRR21696915	SRR21696910
SRR21696909	SRR21696908	SRR21696907	SRR21696906	SRR21696905	SRR21696904	SRR21696925	SRR21696924
SRR21683757	SRR21683756	SRR21683755	SRR21683754	SRR21683753	SRR21683752	SRR21683751	SRR21683750
SRR21683749	SRR21706558	SRR21706559	SRR21706560	SRR21706561	SRR21706562	SRR21706563	SRR21779436
SRR21779435	SRR21779432	SRR21779431	SRR21779430	SRR21779429	SRR21779428	SRR21779427	SRR21779426
SRR21779425	SRR21779434	SRR21779433	SRR21783101	SRR21783100	SRR21783090	SRR21783089	SRR21783088
SRR21783087	SRR21783086	SRR21783085	SRR21783084	SRR21783083	SRR21783099	SRR21783098	SRR21783097
SRR21783096	SRR21783095	SRR21783094	SRR21783093	SRR21783092	SRR21783091	SRR21901320	SRR21901319
SRR21901316	SRR21901315	SRR21901314	SRR21901313	SRR21901312	SRR21901311	SRR21901310	SRR21901309
SRR21901318	SRR21901317	SRR21949253	SRR21949254	SRR21949255	SRR21949256	SRR21949257	SRR21949258
SRR21949259	SRR21949260	SRR21949261	SRR21949262	SRR21949263	SRR21949264	SRR21949265	SRR21949266
SRR21949267	SRR21949268	SRR21949269	SRR21949270	SRR21954039	SRR21954040	SRR21954042	SRR21954041
SRR21954043	SRR21954048	SRR21954044	SRR21954045	SRR21954046	SRR21954047	SRR21954050	SRR21954049
SRR21954051	SRR21954052	SRR21954053	SRR21954054	SRR21954056	SRR21954055	SRR22061909	SRR22061908
SRR22061907	SRR22137867	SRR22137866	SRR22137865	SRR22137864	SRR22137863	SRR22137862	SRR22199121
SRR22199122	SRR22199123	SRR22199124	SRR22199125	SRR22199126	SRR22199127	SRR22199128	SRR22199129
SRR22199130	SRR22199131	SRR22199132	SRR22306772	SRR22306771	SRR22306768	SRR22306767	SRR22306766
SRR22306765	SRR22306764	SRR22306763	SRR22306762	SRR22306761	SRR22306770	SRR22306769	SRR22362397
SRR22362398	SRR22362399	SRR22362400	SRR22393918	SRR22393919	SRR22393920	SRR22393921	SRR22393922
SRR22393923	SRR22393924	SRR22393925	SRR22393926	SRR22393927	SRR22393928	SRR22393929	SRR22450326
SRR22450327	SRR22450328	SRR22450329	SRR22450330	SRR22450331	SRR22461150	SRR22461149	SRR22461146
SRR22461145	SRR22461144	SRR22461143	SRR22461142	SRR22461141	SRR22461140	SRR22461139	SRR22461148
SRR22461147	SRR22514112	SRR22514111	SRR22514100	SRR22514089	SRR22514086	SRR22514085	SRR22514084
SRR22514083	SRR22514082	SRR22514081	SRR22514110	SRR22514109	SRR22514108	SRR22514107	SRR22514106
SRR22514105	SRR22514104	SRR22514103	SRR22514102	SRR22514101	SRR22514099	SRR22514098	SRR22514097
SRR22514096	SRR22514095	SRR22514094	SRR22514093	SRR22514092	SRR22514091	SRR22514090	SRR22514088
SRR22514087	SRR22585242	SRR22585241	SRR22585240	SRR22585239	SRR22585238	SRR22585237	SRR22585236
SRR22585235	SRR22585234	SRR22808115	SRR22808114	SRR22808113	SRR22808112	SRR22808111	SRR22808110
SRR22883067	SRR22883068	SRR22883069	SRR22883064	SRR22883065	SRR22883066	SRR23329178	SRR23329177
SRR22996527	SRR22996526	SRR22996515	SRR22996510	SRR22996509	SRR22996508	SRR22996507	SRR22996506
SRR22996505	SRR22996504	SRR22996525	SRR22996524	SRR22996523	SRR22996522	SRR22996521	SRR22996520

SRR22996519	SRR22996518	SRR22996517	SRR22996516	SRR22996514	SRR22996513	SRR22996512	SRR22996511
SRR23012415	SRR23012414	SRR23012413	SRR23012412	SRR23012411	SRR23012410	SRR23283388	SRR23283387
SRR23283382	SRR23283381	SRR23283380	SRR23283379	SRR23283378	SRR23283377	SRR23283376	SRR23283375
SRR23283386	SRR23283385	SRR23283384	SRR23283383	SRR23269694	SRR23269693	SRR23269692	SRR23269691
SRR23302757	SRR23302758	SRR23302759	SRR23302760	SRR23302761	SRR23302762	SRR23302787	SRR23302788
SRR23302789	SRR23302790	SRR23302791	SRR23302792	SRR23302793	SRR23302794	SRR23302795	SRR23302796
SRR23302797	SRR23302798	SRR23302799	SRR23302800	SRR23302801	SRR23302802	SRR23302803	SRR23302804
SRR23302805	SRR23302806	SRR23302807	SRR23302808	SRR23302809	SRR23302810	SRR23302763	SRR23302764
SRR23302765	SRR23302766	SRR23302767	SRR23302768	SRR23302769	SRR23302770	SRR23302771	SRR23302772
SRR23302773	SRR23302774	SRR23302775	SRR23302776	SRR23302777	SRR23302778	SRR23302779	SRR23302780
SRR23302781	SRR23302782	SRR23302783	SRR23302784	SRR23302785	SRR23302786	SRR23302811	SRR23302812
SRR23302813	SRR23330493	SRR23330498	SRR23330494	SRR23330497	SRR23330495	SRR23330496	SRR23330499
SRR23330500	SRR23427418	SRR23427417	SRR23434981	SRR23434982	SRR23434983	SRR23434984	SRR23434985
SRR23434986	SRR23565735	SRR23565738	SRR23565743	SRR23610231	SRR23610230	SRR23610219	SRR23610208
SRR23610201	SRR23610200	SRR23610199	SRR23610198	SRR23610197	SRR23610196	SRR23610229	SRR23610228
SRR23610227	SRR23610226	SRR23610225	SRR23610224	SRR23610223	SRR23610222	SRR23610221	SRR23610220
SRR23610218	SRR23610217	SRR23610216	SRR23610215	SRR23610214	SRR23610213	SRR23610212	SRR23610211
SRR23610210	SRR23610209	SRR23610207	SRR23610206	SRR23610205	SRR23610204	SRR23610203	SRR23610202
SRR23637453	SRR23637452	SRR23637451	SRR23637450	SRR23637449	SRR23637448	SRR23699633	SRR23699634
SRR23699635	SRR23699636	SRR23699644	SRR23699645	SRR23699630	SRR23699632	SRR23699631	SRR23699629
SRR23699643	SRR23699642	SRR23699639	SRR23699640	SRR23699641	SRR23699638	SRR23699637	SRR23892756
SRR23892755	SRR23892754	SRR23892753	SRR23892752	SRR23892751	SRR23839398	SRR23839399	SRR23839400
SRR23839401	SRR23839402	SRR23839403	SRR23839404	SRR23839405	SRR23839406	SRR23839407	SRR23839408
SRR23839409	SRR23967321	SRR23967322	SRR23967323	SRR23967324	SRR23967325	SRR23967326	SRR23967327
SRR23967328	SRR23967329	SRR23967330	SRR23978649	SRR23978650	SRR23978651	SRR23978652	SRR23978653
SRR23978654	SRR23998976	SRR23998975	SRR23998968	SRR23998967	SRR23998966	SRR23998965	SRR23998964
SRR23998963	SRR23998962	SRR23998961	SRR23998974	SRR23998973	SRR24055688	SRR24055607	SRR24055612
SRR24055608	SRR24055611	SRR24055609	SRR24055610	SRR24055613	SRR24055614	SRR24055619	SRR24055615
SRR24055618	SRR24055616	SRR24055617	SRR24055620	SRR24055635	SRR24055636	SRR24055637	SRR24055638
SRR24055639	SRR24055640	SRR24055641	SRR24055642	SRR24055643	SRR24055644	SRR24055645	SRR24055646
SRR24055661	SRR24055662	SRR24055663	SRR24055664	SRR24055665	SRR24055666	SRR24055667	SRR24055668
SRR24055669	SRR24055670	SRR24055671	SRR24055672	SRR24055689	SRR24055694	SRR24055690	SRR24055693
SRR24055691	SRR24055692	SRR24055695	SRR24055696	SRR24055701	SRR24055697	SRR24055700	SRR24055698
SRR24055699	SRR24055606	SRR24055621	SRR24055626	SRR24055622	SRR24055625	SRR24055623	SRR24055624
SRR24055627	SRR24055628	SRR24055633	SRR24055629	SRR24055632	SRR24055630	SRR24055631	SRR24055634
SRR24055647	SRR24055652	SRR24055648	SRR24055651	SRR24055649	SRR24055650	SRR24055653	SRR24055654
SRR24055659	SRR24055655	SRR24055658	SRR24055656	SRR24055657	SRR24055660	SRR24055673	SRR24055678
SRR24055674	SRR24055677	SRR24055675	SRR24055676	SRR24055679	SRR24055680	SRR24055685	SRR24055681
SRR24055684	SRR24055682	SRR24055683	SRR24055686	SRR24055687	SRR24101366	SRR24101367	SRR24101368
SRR24101369	SRR24101370	SRR24101371	SRR24101372	SRR24101373	SRR24101374	SRR24101375	SRR24101376
SRR24101377	SRR24182826	SRR24182825	SRR24182824	SRR24182823	SRR24182822	SRR24182821	SRR24182774
SRR24182773	SRR24182772	SRR24182771	SRR24182770	SRR24182769	SRR24266703	SRR24266704	SRR24266705
SRR24266706	SRR24266707	SRR24266708	SRR24266709	SRR24266710	SRR24266711	SRR24266712	SRR24266713
SRR24266714	SRR24266715	SRR24266716	SRR24266717	SRR24266718	SRR24266719	SRR24266720	SRR24266721
SRR24266722	SRR24266723	SRR24266724	SRR24266725	SRR24266726	SRR24266727	SRR24266728	SRR24266729
SRR24266730	SRR24266731	SRR24266732	SRR24266733	SRR24266734	SRR24266735	SRR24266736	SRR24266737
SRR24266738	SRR24266739	SRR24266740	SRR24266741	SRR24266766	SRR24266767	SRR24266768	SRR24266769
SRR24266770	SRR24266771	SRR24266772	SRR24266773	SRR24266774	SRR24266775	SRR24266776	SRR24266777
SRR24266778	SRR24266779	SRR24266780	SRR24266781	SRR24266782	SRR24266783	SRR24266784	SRR24266785
SRR24266786	SRR24266787	SRR24266788	SRR24266789	SRR24266742	SRR24266743	SRR24266744	SRR24266745
SRR24266746	SRR24266747	SRR24266748	SRR24266749	SRR24266750	SRR24266751	SRR24266752	SRR24266753
SRR24266754	SRR24266755	SRR24266756	SRR24266757	SRR24266758	SRR24266759	SRR24266760	SRR24266761
SRR24266762	SRR24266763	SRR24266764	SRR24266765	SRR24266790	SRR24266791	SRR24266792	SRR24322835
SRR24322834	SRR24322833	SRR24322832	SRR24322831	SRR24322830	SRR24322829	SRR24322828	SRR24322827
