# Supplementary material for: Genome-wide identification of long non-coding RNAs in pigs and their roles in muscle development
Source: Anim Biosci. 2026 Jul 27;39(10):260444. doi: 10.5713/ab.260444 (PMC13662941; doi:10.5713/ab.260444)
Supplement: Supplementary file 3 [file ab-260444-Supplementary-3.pdf]

Supplement 3. Sample information for lncRNA annotation.

Experiment	Experiment totall clean reads count	Layout	Read1 forward/ (forward+reverse)	Read2 forward/ (forward+reverse)	Strand specific	Aligned concordantly exactly 1 time (%)	Tissue	Strand condition
DKM1DNF4_2	58918534	Paired	0.0312368	0.966977	RF	77.37	Brain	Yes
DKM1DNF4_3	64840067	Paired	0.032699	0.965831	RF	79.81	Brain	Yes
DKM1DNF4_4	59935344	Paired	0.0448223	0.951957	RF	83.78	Brain	Yes
DKM1DNF6_1	62598143	Paired	0.0235068	0.976401	RF	83.64	Brain	Yes
DKM1DNF8_1	57605064	Paired	0.0217841	0.977624	RF	81.63	Brain	Yes
DKM2DNF3_1	54685696	Paired	0.0227417	0.976948	RF	77.28	Brain	Yes
DKM2DNF3_2	63290442	Paired	0.0223655	0.97769	RF	81.81	Brain	Yes
DKM2DNF3_3	55540203	Paired	0.0190938	0.980874	RF	79.96	Brain	Yes
DKM2DNF5_1	61612918	Paired	0.024089	0.975131	RF	78.97	Brain	Yes
DKM2DNF5_2	60216080	Paired	0.0241927	0.974859	RF	79.81	Brain	Yes
DKM2DNF5_3	55996697	Paired	0.0252315	0.973633	RF	71.14	Brain	Yes
DKM2DNF5_4	47390173	Paired	0.0265498	0.972477	RF	81.94	Brain	Yes
DKM2DNF7_4	56171724	Paired	0.0309807	0.967641	RF	82.74	Brain	Yes
DKM2DNF7_5	55217537	Paired	0.0295352	0.969055	RF	82.84	Brain	Yes
DKM2DNF7_6	53754633	Paired	0.0357121	0.962687	RF	78.18	Brain	Yes
DNM1_154001_1	61550380	Paired	0.0204165	0.97816	RF	61.48	Brain	Yes
DNM1_154001_2	59737323	Paired	0.036732	0.961248	RF	78.68	Brain	Yes
DNM1_154001_3	57379267	Paired	0.0283692	0.970958	RF	84.63	Brain	Yes
DNM1_154001_4	50086953	Paired	0.0240824	0.975142	RF	79.06	Brain	Yes
DNM1_154001_5	60558646	Paired	0.0276597	0.971593	RF	80.59	Brain	Yes
DNM1DKF2_5	54984516	Paired	0.0300802	0.968605	RF	82.2	Brain	Yes
DNM1DKF2_6	53985885	Paired	0.0290328	0.969677	RF	80.73	Brain	Yes
DNM1DKF2_7	50000698	Paired	0.0304693	0.968247	RF	83.77	Brain	Yes
DNM1DKF2_8	54364446	Paired	0.0319018	0.966818	RF	79.7	Brain	Yes
DNM2_118206_1	53240396	Paired	0.0206551	0.979354	RF	83.27	Brain	Yes
DNM2_118206_2	68635407	Paired	0.0192112	0.980722	RF	80.38	Brain	Yes
DNM2_118206_3	65870183	Paired	0.0263257	0.973138	RF	80.11	Brain	Yes
DNM2_118206_4	62229127	Paired	0.0235314	0.97638	RF	85.29	Brain	Yes
DNM2DKF1_1	72369036	Paired	0.0417235	0.955143	RF	81.25	Brain	Yes
DNM2DKF1_3	59808049	Paired	0.0497828	0.945052	RF	79.18	Brain	Yes
DNM2DKF1_5	55844999	Paired	0.0549888	0.941193	RF	80.24	Brain	Yes
DNM2DKF1_8	59039250	Paired	0.0330078	0.965571	RF	80.49	Brain	Yes
DNM2DKF3_1	59501156	Paired	0.0351017	0.963561	RF	85.14	Brain	Yes
DNM2DKF3_2	55996979	Paired	0.0400502	0.958593	RF	85.12	Brain	Yes
DNM2DKF3_3	54668724	Paired	0.0367264	0.9626	RF	86.55	Brain	Yes
DNM2DKF3_4	60463175	Paired	0.0377992	0.961359	RF	85.45	Brain	Yes
LR21A14DX105	21120064	Paired	0.0133364	0.987645	RF	81.96	Muscle	Yes
LR21A14DX106	18815246	Paired	0.0141762	0.986745	RF	82.44	Muscle	Yes
LR21A14DX107	19935431	Paired	0.0135397	0.987439	RF	83.23	Muscle	Yes
LR21A14DX108	23997738	Paired	0.0128606	0.988162	RF	83.2	Muscle	Yes
LR21A14DX109	19642144	Paired	0.0134236	0.987577	RF	80.85	Muscle	Yes
LR21A14DX110	18807938	Paired	0.0140981	0.986976	RF	82.25	Muscle	Yes
LR21A14DX111	18382512	Paired	0.0142048	0.986894	RF	82.21	Muscle	Yes
LR21A14DX112	20069234	Paired	0.0132911	0.987712	RF	82.38	Muscle	Yes
LR21A14DX113	22467459	Paired	0.0127917	0.988273	RF	82.75	Muscle	Yes
LR21A14DX114	23170370	Paired	0.0125466	0.988519	RF	82.38	Muscle	Yes
LR21A14DX115	23464003	Paired	0.0129649	0.987997	RF	81.22	Muscle	Yes
LR21A14DX116	19084303	Paired	0.0144022	0.986458	RF	83.81	Muscle	Yes
LR21A14DX117	21848434	Paired	0.013125	0.987928	RF	83.16	Muscle	Yes
LR21A14DX118	20720892	Paired	0.0133565	0.987586	RF	83.61	Muscle	Yes
LR21A14DX119	21748782	Paired	0.012594	0.988329	RF	82.93	Muscle	Yes
LR21A14DX120	22271481	Paired	0.0126331	0.988331	RF	82.43	Muscle	Yes
LR21A14DX121	22641404	Paired	0.0127025	0.988264	RF	83.06	Muscle	Yes
LR21A14DX122	22258239	Paired	0.0131627	0.987899	RF	83.34	Muscle	Yes
LR21A14DX123	20538600	Paired	0.012488	0.988421	RF	84.32	Muscle	Yes
LR21A14DX124	25756948	Paired	0.0129074	0.98806	RF	83.63	Muscle	Yes

LR21A14DX125	21108922	Paired	0.0130425	0.987948	RF	83.15	Muscle	Yes
LR21A14DX126	20028549	Paired	0.0128505	0.988187	RF	83.97	Muscle	Yes
LR21A14DX127	21961207	Paired	0.0137964	0.986842	RF	77.46	Muscle	Yes
LR21A14DX128	19283967	Paired	0.0135354	0.987418	RF	83.02	Muscle	Yes
LR21A14DX129	23088982	Paired	0.0127265	0.988372	RF	82.69	Muscle	Yes
LR21A14DX130	24264777	Paired	0.0118616	0.989159	RF	84.18	Muscle	Yes
LR21A14DX131	23817321	Paired	0.0122128	0.988937	RF	83.81	Muscle	Yes
LR21A14DX132	21233944	Paired	0.0135098	0.987417	RF	84.54	Muscle	Yes
LR21A14DX133	24507785	Paired	0.0120802	0.988901	RF	83.89	Muscle	Yes
LR21A14DX134	22412336	Paired	0.0136335	0.987312	RF	83.28	Muscle	Yes
LR21A14DX135	21885874	Paired	0.0125734	0.988372	RF	83.39	Muscle	Yes
LR21A14DX136	22699459	Paired	0.0134635	0.987382	RF	82.89	Muscle	Yes
LR21A14DX137	19027406	Paired	0.0142569	0.986765	RF	84.18	Muscle	Yes
LR21A14DX138	18673254	Paired	0.0130844	0.988025	RF	84.43	Muscle	Yes
LR21A14DX139	19131838	Paired	0.0127581	0.988352	RF	84.35	Muscle	Yes
LR21A14DX140	24115538	Paired	0.0125972	0.988472	RF	84.83	Muscle	Yes
LR21A14DX141	19910268	Paired	0.0130852	0.987923	RF	85.04	Muscle	Yes
LR21A14DX142	24796255	Paired	0.0129735	0.988246	RF	85.5	Muscle	Yes
LR21A14DX143	24355992	Paired	0.0129283	0.987883	RF	82.82	Muscle	Yes
LR21A14DX144	25044099	Paired	0.0124641	0.988585	RF	83.08	Muscle	Yes
LR21A14DX145	23527733	Paired	0.0119446	0.988838	RF	81.16	Muscle	Yes
LR21A14DX146	23766598	Paired	0.0127677	0.98828	RF	83.73	Muscle	Yes
LR21A14DX147	23965463	Paired	0.0148803	0.986136	RF	83.6	Muscle	Yes
LR21A14DX148	19024755	Paired	0.0125243	0.988453	RF	84.21	Muscle	Yes
LR21A14DX149	21241404	Paired	0.0131039	0.987968	RF	83.74	Muscle	Yes
LR21A14DX150	21483643	Paired	0.0136302	0.987648	RF	83.83	Muscle	Yes
LR21A14DX151	21971759	Paired	0.0153466	0.985684	RF	83.15	Muscle	Yes
LR21A14DX152	19637171	Paired	0.0133343	0.987716	RF	84.32	Muscle	Yes
LR21A14DX153	23099116	Paired	0.0130008	0.987859	RF	81.91	Muscle	Yes
LR21A14DX154	22685552	Paired	0.0124791	0.988533	RF	82.67	Muscle	Yes
LR21A14DX155	21983340	Paired	0.0126906	0.988182	RF	82.93	Muscle	Yes
LR21A14DX156	20191223	Paired	0.0128145	0.98813	RF	82.58	Muscle	Yes
LR21A14DX157	21737104	Paired	0.0133509	0.987661	RF	84.71	Muscle	Yes
LR21A14DX158	20269133	Paired	0.0127704	0.988078	RF	82.33	Muscle	Yes
LR21A14DX159	19760377	Paired	0.013966	0.986955	RF	83.94	Muscle	Yes
LR21A14DX160	21467771	Paired	0.0150331	0.986067	RF	81.9	Muscle	Yes
LR21A14DX161	23937217	Paired	0.0124217	0.988493	RF	81.99	Muscle	Yes
LR21A14DX162	23644119	Paired	0.0129272	0.987966	RF	82.22	Muscle	Yes
LR21A14DX163	23226746	Paired	0.0119071	0.989104	RF	83.33	Muscle	Yes
LR21A14DX164	18345413	Paired	0.0134628	0.987497	RF	84.16	Muscle	Yes
LR21A14DX165	23414931	Paired	0.0141479	0.987154	RF	81.92	Muscle	Yes
LR21A14DX166	22235002	Paired	0.0124593	0.988421	RF	83.32	Muscle	Yes
LR21A14DX167	20999690	Paired	0.012435	0.988501	RF	81.8	Muscle	Yes
LR21A14DX168	23767364	Paired	0.0130789	0.987836	RF	81.7	Muscle	Yes
LR21A14DX169	19725682	Paired	0.0135224	0.987644	RF	82.44	Muscle	Yes
LR21A14DX170	19382423	Paired	0.013112	0.987952	RF	82.51	Muscle	Yes
LR21A14DX171	19027577	Paired	0.0136406	0.98742	RF	82.61	Muscle	Yes
LR21A14DX172	25543621	Paired	0.0117598	0.989342	RF	82.86	Muscle	Yes
LR21A14DX173	18293072	Paired	0.0152453	0.985712	RF	83.85	Muscle	Yes
LR21A14DX174	18131573	Paired	0.013573	0.987338	RF	83.34	Muscle	Yes
LR21A14DX175	25245939	Paired	0.0128716	0.988197	RF	83.42	Muscle	Yes
LR21A14DX176	25585922	Paired	0.0121327	0.988871	RF	82.7	Muscle	Yes
LR21A14DX177	20176063	Paired	0.0148509	0.986208	RF	83.16	Muscle	Yes
LR21A14DX178	21360668	Paired	0.0143624	0.986606	RF	82.59	Muscle	Yes
LR21A14DX179	20505137	Paired	0.0137979	0.987179	RF	81.42	Muscle	Yes
LR21A14DX180	24851390	Paired	0.0132627	0.987612	RF	82.47	Muscle	Yes
LR21A14DX181	20540858	Paired	0.0140509	0.986856	RF	81.87	Muscle	Yes
LR21A14DX182	20553104	Paired	0.0130795	0.988012	RF	82.96	Muscle	Yes

LR21A14DX183	19565696	Paired	0.0136606	0.987205	RF	81.42	Muscle	Yes
LR21A14DX184	19428307	Paired	0.0132457	0.987686	RF	81.62	Muscle	Yes
LR21A14DX185	18340278	Paired	0.0137802	0.987048	RF	80.9	Muscle	Yes
LR21A14DX186	18840608	Paired	0.0137958	0.987188	RF	81.66	Muscle	Yes
LR21A14DX187	19487703	Paired	0.0130989	0.987933	RF	83.1	Muscle	Yes
LR21A14DX188	25636825	Paired	0.0125372	0.988563	RF	81.03	Muscle	Yes
LR21A14DX189	18732484	Paired	0.0129322	0.988002	RF	81.1	Muscle	Yes
LR21A14DX190	18570615	Paired	0.016349	0.984614	RF	80.18	Muscle	Yes
LR21A14DX191	17978396	Paired	0.0156236	0.985306	RF	81.5	Muscle	Yes
LR21A14DX192	24844537	Paired	0.0128605	0.988123	RF	81.3	Muscle	Yes
LR21A14DX193	21540648	Paired	0.0161039	0.984771	RF	81.33	Muscle	Yes
LR21A14DX194	20805146	Paired	0.0129453	0.987986	RF	81.62	Muscle	Yes
LR21A14DX195	20713031	Paired	0.013106	0.987818	RF	81.62	Muscle	Yes
LR21A14DX196	17739786	Paired	0.0137944	0.98715	RF	81.5	Muscle	Yes
LR21A14DX197	19313314	Paired	0.0133036	0.9876	RF	80.35	Muscle	Yes
LR21A14DX198	19513243	Paired	0.0139433	0.98694	RF	81.49	Muscle	Yes
LR21A14DX199	25038022	Paired	0.0141531	0.986773	RF	82.32	Muscle	Yes
LR21A14DX200	18144746	Paired	0.0133338	0.987729	RF	82.62	Muscle	Yes
LR21A14DX201	20692690	Paired	0.0145237	0.986455	RF	81.91	Muscle	Yes
LR21A14DX202	19603461	Paired	0.0135841	0.987395	RF	82.93	Muscle	Yes
LR21A14DX203	18554522	Paired	0.013251	0.987696	RF	81.73	Muscle	Yes
LR21A14DX204	21816138	Paired	0.0131262	0.987983	RF	82.67	Muscle	Yes
LR21A14DX205	18238501	Paired	0.0143606	0.986666	RF	82.72	Muscle	Yes
LR21A14DX206	19924266	Paired	0.0141427	0.986893	RF	82.32	Muscle	Yes
LR21A14DX207	18006489	Paired	0.0131286	0.987913	RF	81.69	Muscle	Yes
LR21A14DX208	24725037	Paired	0.0141381	0.98687	RF	82.04	Muscle	Yes
LR21A14DX209	25069672	Paired	0.0119244	0.989121	RF	81.64	Muscle	Yes
LR21A14DX210	22047618	Paired	0.0124325	0.988375	RF	81.36	Muscle	Yes
LR21A14DX211	19699265	Paired	0.0137062	0.987307	RF	82.69	Muscle	Yes
LR21A14DX212	21778827	Paired	0.0126313	0.988438	RF	82.18	Muscle	Yes
LR21A14DX213	20942428	Paired	0.0132968	0.987784	RF	83.07	Muscle	Yes
LR21A14DX214	20705727	Paired	0.0139573	0.987054	RF	82.9	Muscle	Yes
LR21A14DX215	21137923	Paired	0.0131261	0.987821	RF	82.82	Muscle	Yes
LR21A14DX216	21250214	Paired	0.0137293	0.987248	RF	82.23	Muscle	Yes
LR21A14DX217	24840850	Paired	0.0126697	0.98838	RF	82.27	Muscle	Yes
LR21A14DX218	22833467	Paired	0.0128744	0.987991	RF	82.52	Muscle	Yes
LR21A14DX219	21165316	Paired	0.01271	0.98846	RF	83.08	Muscle	Yes
LR21A14DX220	22884397	Paired	0.0139764	0.987069	RF	83.45	Muscle	Yes
LR21A14DX221	22880588	Paired	0.013206	0.987752	RF	82.53	Muscle	Yes
LR21A14DX222	22749751	Paired	0.0125632	0.988447	RF	81.93	Muscle	Yes
LR21A14DX223	21647092	Paired	0.013407	0.987756	RF	84.03	Muscle	Yes
LR21A14DX224	19917070	Paired	0.0125849	0.988342	RF	82.21	Muscle	Yes
LR21A14DX225	25546820	Paired	0.012524	0.9887	RF	82.69	Muscle	Yes
LR21A14DX226	25632405	Paired	0.016718	0.984434	RF	83.16	Muscle	Yes
LR21A14DX227	23708998	Paired	0.0125652	0.988446	RF	82.66	Muscle	Yes
LR21A14DX228	24894065	Paired	0.0126803	0.988301	RF	82.59	Muscle	Yes
LR21A14DX229	22408565	Paired	0.0136616	0.987377	RF	83.06	Muscle	Yes
LR21A14DX230	22679534	Paired	0.0130538	0.987941	RF	82.48	Muscle	Yes
LR21A14DX231	24553635	Paired	0.0124473	0.988531	RF	83.07	Muscle	Yes
LR21A14DX232	26531550	Paired	0.0135357	0.98783	RF	83.43	Muscle	Yes
LR21A14DX233	21738922	Paired	0.0147628	0.98631	RF	81.27	Muscle	Yes
LR21A14DX234	24053256	Paired	0.0118382	0.989195	RF	81.73	Muscle	Yes
LR21A14DX235	24695006	Paired	0.0148517	0.986182	RF	82.26	Muscle	Yes
LR21A14DX236	20468203	Paired	0.0126384	0.988254	RF	81.11	Muscle	Yes
LR21A14DX237	24957080	Paired	0.0138157	0.987131	RF	82.64	Muscle	Yes
LR21A14DX238	20675447	Paired	0.0145903	0.986492	RF	83.69	Muscle	Yes
LR21A14DX239	17939145	Paired	0.0138486	0.987137	RF	83.04	Muscle	Yes
LR21A14DX240	18749786	Paired	0.0132531	0.98787	RF	82.34	Muscle	Yes

LR21A14DX241	23051977	Paired	0.0118885	0.988966	RF	82.18	Muscle	Yes
LR21A14DX242	22883550	Paired	0.0136232	0.98742	RF	82.54	Muscle	Yes
LR21A14DX243	19063048	Paired	0.0137936	0.987225	RF	82.58	Muscle	Yes
LR21A14DX244	24412173	Paired	0.0119453	0.989105	RF	82.23	Muscle	Yes
LR21A14DX245	20181244	Paired	0.0129967	0.988006	RF	82.53	Muscle	Yes
LR21A14DX246	21705732	Paired	0.0132285	0.987823	RF	82.58	Muscle	Yes
LR21A14DX247	24854524	Paired	0.0129973	0.988051	RF	83.17	Muscle	Yes
LR21A14DX248	20578468	Paired	0.0151003	0.985994	RF	83.84	Muscle	Yes
LR21A14DX249	21361423	Paired	0.013735	0.987157	RF	81.6	Muscle	Yes
LR21A14DX250	24805184	Paired	0.0130867	0.987893	RF	82.19	Muscle	Yes
LR21A14DX251	18823225	Paired	0.0129986	0.988084	RF	81.49	Muscle	Yes
LR21A14DX252	24554114	Paired	0.0125653	0.988467	RF	82.53	Muscle	Yes
LR21A14DX253	20748385	Paired	0.0139	0.986982	RF	82.04	Muscle	Yes
LR21A14DX254	21969133	Paired	0.0126383	0.988347	RF	82.24	Muscle	Yes
LR21A14DX255	19439848	Paired	0.0136582	0.987397	RF	83.65	Muscle	Yes
LR21A14DX256	18075446	Paired	0.0138267	0.987156	RF	81.8	Muscle	Yes
LR21A14DX257	21091234	Paired	0.0141187	0.986928	RF	82.91	Muscle	Yes
LR21A14DX258	17725004	Paired	0.0150307	0.985979	RF	83.14	Muscle	Yes
LR21A14DX259	20141224	Paired	0.0123428	0.988692	RF	83.5	Muscle	Yes
LR21A14DX260	19978800	Paired	0.0142602	0.986567	RF	82.67	Muscle	Yes
LR21A14DX261	26247449	Paired	0.0116091	0.989364	RF	82.05	Muscle	Yes
LR21A14DX262	21432834	Paired	0.0135502	0.98765	RF	82.54	Muscle	Yes
LR21A14DX263	22512909	Paired	0.0136252	0.987366	RF	84.25	Muscle	Yes
LR21A14DX264	24051986	Paired	0.0131854	0.987632	RF	82.16	Muscle	Yes
LR21A14DX265	22297969	Paired	0.0133504	0.987581	RF	83	Muscle	Yes
LR21A14DX266	25535271	Paired	0.0120806	0.988862	RF	82.24	Muscle	Yes
LR21A14DX267	20395963	Paired	0.0137827	0.987203	RF	83.33	Muscle	Yes
LR21A14DX268	24833929	Paired	0.0120636	0.988885	RF	82.87	Muscle	Yes
LR21A14DX269	18738752	Paired	0.0144994	0.986486	RF	83.17	Muscle	Yes
LR21A14DX270	20257708	Paired	0.0130533	0.987958	RF	82.33	Muscle	Yes
LR21A14DX271	26230326	Paired	0.0117705	0.989179	RF	83.26	Muscle	Yes
LR21A14DX272	25168488	Paired	0.0130021	0.988099	RF	82.83	Muscle	Yes
LR21A14DX273	22041962	Paired	0.012329	0.988616	RF	81.01	Muscle	Yes
LR21A14DX274	24688153	Paired	0.0120713	0.98884	RF	81.34	Muscle	Yes
LR21A14DX275	23681860	Paired	0.0128091	0.988211	RF	82.64	Muscle	Yes
LR21A14DX276	19861884	Paired	0.0155231	0.985425	RF	83.09	Muscle	Yes
LR21A14DX277	19666137	Paired	0.0128994	0.988052	RF	81.83	Muscle	Yes
LR21A14DX278	26712547	Paired	0.0132828	0.987617	RF	82.48	Muscle	Yes
LR21A14DX279	19349635	Paired	0.0125628	0.988602	RF	82.93	Muscle	Yes
LR21A14DX280	19503274	Paired	0.0129619	0.987985	RF	82.2	Muscle	Yes
LR21A14DX281	24848385	Paired	0.0129217	0.98803	RF	82.13	Muscle	Yes
LR21A14DX282	22858815	Paired	0.0125095	0.988423	RF	81.83	Muscle	Yes
LR21A14DX283	26043169	Paired	0.012799	0.988476	RF	82.75	Muscle	Yes
LR21A14DX284	20221259	Paired	0.0134248	0.987509	RF	81.91	Muscle	Yes
LR21A14DX285	22268138	Paired	0.0138883	0.987153	RF	81	Muscle	Yes
LR21A14DX286	19481650	Paired	0.0127329	0.988332	RF	82.03	Muscle	Yes
LR21A14DX287	21082783	Paired	0.0135559	0.987388	RF	81.02	Muscle	Yes
LR21A14DX288	17813876	Paired	0.0138018	0.987199	RF	81.64	Muscle	Yes
LR21A14DX289	24414938	Paired	0.012978	0.988079	RF	82.04	Muscle	Yes
LR21A14DX290	23489077	Paired	0.0123851	0.988445	RF	80.65	Muscle	Yes
LR21A14DX291	20239812	Paired	0.0139009	0.987012	RF	82.32	Muscle	Yes
LR21A14DX292	21550781	Paired	0.0136495	0.987243	RF	81.84	Muscle	Yes
LR21A14DX293	20813090	Paired	0.0129319	0.988168	RF	82.36	Muscle	Yes
LR21A14DX294	20705919	Paired	0.0134811	0.987606	RF	83.39	Muscle	Yes
LR21A14DX295	21389148	Paired	0.013076	0.987905	RF	81.81	Muscle	Yes
LR21A14DX296	22085723	Paired	0.0157356	0.985433	RF	82.59	Muscle	Yes
LR21A14DX297	23475396	Paired	0.0126312	0.988304	RF	77.87	Muscle	Yes
LR21A14DX298	25634440	Paired	0.0124748	0.988897	RF	81.2	Muscle	Yes

LR21A14DX299	26144435	Paired	0.0130718	0.988054	RF	82	Muscle	Yes
LR21A14DX300	26921276	Paired	0.011637	0.989553	RF	82.65	Muscle	Yes
LR21A14DX301	20648862	Paired	0.0140538	0.987254	RF	80.77	Muscle	Yes
LR21A14DX302	22578561	Paired	0.013419	0.987591	RF	82.12	Muscle	Yes
LR21A14DX303	25105177	Paired	0.0128868	0.988175	RF	81.1	Muscle	Yes
LR21A14DX304	19192258	Paired	0.0158476	0.985156	RF	81.81	Muscle	Yes
LR21A14DX305	25196681	Paired	0.0127225	0.98827	RF	79.68	Muscle	Yes
LR21A14DX306	24827585	Paired	0.013574	0.987602	RF	80.63	Muscle	Yes
LR21A14DX307	20624811	Paired	0.0143936	0.986751	RF	81.98	Muscle	Yes
LR21A14DX308	21944806	Paired	0.0138968	0.987235	RF	80.99	Muscle	Yes
LR21A14DX309	26480600	Paired	0.0129833	0.988022	RF	81.23	Muscle	Yes
LR21A14DX310	21101879	Paired	0.0137946	0.98729	RF	83.23	Muscle	Yes
LR21A14DX311	26144965	Paired	0.0142928	0.986698	RF	82.41	Muscle	Yes
LR21A14DX312	26485325	Paired	0.0163893	0.984831	RF	81.73	Muscle	Yes
LR21A14DX313	21333589	Paired	0.0132074	0.987899	RF	80.58	Muscle	Yes
LR21A14DX314	20059458	Paired	0.012899	0.988042	RF	76.87	Muscle	Yes
LR21A14DX315	18732902	Paired	0.0145895	0.986411	RF	82.8	Muscle	Yes
LR21A14DX316	23671593	Paired	0.0129808	0.988078	RF	82.23	Muscle	Yes
LR21A14DX317	24739515	Paired	0.0136659	0.987397	RF	80.37	Muscle	Yes
LR21A14DX318	24619925	Paired	0.0132317	0.987787	RF	82.12	Muscle	Yes
LR21A14DX319	25561378	Paired	0.0131509	0.987882	RF	81.17	Muscle	Yes
LR21A14DX320	19091630	Paired	0.0136112	0.987461	RF	80.87	Muscle	Yes
LR21A14DX321	25558580	Paired	0.0141574	0.98725	RF	83.71	Muscle	Yes
LR21A14DX322	21951583	Paired	0.0152711	0.985935	RF	82.06	Muscle	Yes
LR21A14DX323	23849647	Paired	0.0145105	0.98651	RF	81.64	Muscle	Yes
LR21A14DX324	19622787	Paired	0.0127961	0.98822	RF	81.81	Muscle	Yes
LR21A14DX325	22965180	Paired	0.0122692	0.988731	RF	82.76	Muscle	Yes
LR21A14DX326	21110096	Paired	0.0146027	0.98644	RF	80.34	Muscle	Yes
LR21A14DX327	23395245	Paired	0.0155336	0.985611	RF	82	Muscle	Yes
LR21A14DX328	24134084	Paired	0.0137585	0.987528	RF	81.05	Muscle	Yes
LR21A14DX329	26429217	Paired	0.0129355	0.988317	RF	83.29	Muscle	Yes
LR21A14DX330	21820918	Paired	0.0133561	0.987793	RF	82.09	Muscle	Yes
LR21A14DX331	21471618	Paired	0.0127536	0.988349	RF	82.29	Muscle	Yes
LR21A14DX332	24181781	Paired	0.0123817	0.988605	RF	81.22	Muscle	Yes
LR21A14DX333	21320243	Paired	0.0159846	0.985216	RF	81.83	Muscle	Yes
LR21A14DX334	19033883	Paired	0.0145464	0.986564	RF	82.87	Muscle	Yes
LR21A14DX335	21703506	Paired	0.0155711	0.985606	RF	81.21	Muscle	Yes
LR21A14DX336	21104563	Paired	0.0142993	0.986887	RF	81.99	Muscle	Yes
LR21A14DX337	26447032	Paired	0.013594	0.987418	RF	81.39	Muscle	Yes
LR21A14DX338	25186659	Paired	0.0131987	0.98771	RF	78.44	Muscle	Yes
LR21A14DX339	25478132	Paired	0.0134534	0.98731	RF	78.6	Muscle	Yes
LR21A14DX340	21303655	Paired	0.01404	0.986977	RF	78.52	Muscle	Yes
LR21A14DX341	25612433	Paired	0.0139782	0.986951	RF	81.26	Muscle	Yes
LR21A14DX342	23636455	Paired	0.0130992	0.987841	RF	80.29	Muscle	Yes
LR21A14DX343	24038028	Paired	0.0126516	0.988349	RF	80.98	Muscle	Yes
LR21A14DX344	24607696	Paired	0.0143587	0.987051	RF	82.17	Muscle	Yes
LR21A14DX345	23471140	Paired	0.0133988	0.987573	RF	80.59	Muscle	Yes
LR21A14DX346	24851273	Paired	0.012948	0.98809	RF	79.38	Muscle	Yes
LR21A14DX347	23045104	Paired	0.0150305	0.985941	RF	79.59	Muscle	Yes
LR21A14DX348	18784941	Paired	0.0152709	0.98572	RF	77.78	Muscle	Yes
LR21A14DX349	23556949	Paired	0.0142685	0.98667	RF	78.22	Muscle	Yes
LR21A14DX350	19116854	Paired	0.0144679	0.986636	RF	82.87	Muscle	Yes
LR21A14DX351	20240331	Paired	0.0133329	0.987638	RF	80.76	Muscle	Yes
LR21A14DX352	20342064	Paired	0.0129782	0.988146	RF	80.6	Muscle	Yes
LR21A14DX353	25645042	Paired	0.0152748	0.985878	RF	81.91	Muscle	Yes
LR21A14DX354	23784213	Paired	0.0133563	0.987435	RF	75.03	Muscle	Yes
LR21A14DX355	24598467	Paired	0.0123256	0.988528	RF	79.19	Muscle	Yes
LR21A14DX356	21320525	Paired	0.0134952	0.987577	RF	80.03	Muscle	Yes

LR21A14DX357	24674762	Paired	0.0118705	0.989051	RF	81.27	Muscle	Yes
LR21A14DX358	22564297	Paired	0.0141925	0.986937	RF	82.04	Muscle	Yes
LR21A14DX359	22681441	Paired	0.0151767	0.985754	RF	80.77	Muscle	Yes
LR21A14DX360	20652345	Paired	0.0139707	0.987216	RF	81	Muscle	Yes
LR21A14DX361	23575648	Paired	0.013289	0.987771	RF	77.75	Muscle	Yes
LR21A14DX362	23388349	Paired	0.0162569	0.984913	RF	80.76	Muscle	Yes
LR21A14DX363	23794109	Paired	0.0127009	0.988309	RF	80.87	Muscle	Yes
LR21A14DX364	21073140	Paired	0.0139938	0.987084	RF	81.28	Muscle	Yes
LR21A14DX365	24189878	Paired	0.0132541	0.987823	RF	79.7	Muscle	Yes
LR21A14DX366	21275893	Paired	0.012592	0.988453	RF	81.27	Muscle	Yes
LR21A14DX367	21992276	Paired	0.0158336	0.985402	RF	82.53	Muscle	Yes
LR21A14DX368	21236240	Paired	0.0135083	0.987631	RF	81.14	Muscle	Yes
LR21A14DX369	22517922	Paired	0.0128561	0.988215	RF	80.29	Muscle	Yes
LR21A14DX370	23569121	Paired	0.0146179	0.986406	RF	80.08	Muscle	Yes
LR21A14DX371	22146959	Paired	0.0134861	0.987573	RF	82.53	Muscle	Yes
LR21A14DX372	18638974	Paired	0.01883	0.982462	RF	81.41	Muscle	Yes
LR21A14DX373	22044834	Paired	0.0137736	0.987358	RF	82.41	Muscle	Yes
LR21A14DX374	20324173	Paired	0.0148076	0.986397	RF	81.94	Muscle	Yes
LR21A14DX375	20155233	Paired	0.0135752	0.987496	RF	80.91	Muscle	Yes
LR21A14DX376	21144933	Paired	0.0139778	0.987138	RF	80.83	Muscle	Yes
LR21A14DX377	25053428	Paired	0.0129738	0.988137	RF	76.27	Muscle	Yes
LR21A14DX378	23712949	Paired	0.0169146	0.984245	RF	79.69	Muscle	Yes
LR21A14DX379	23978228	Paired	0.0128341	0.988243	RF	81.6	Muscle	Yes
LR21A14DX380	21429853	Paired	0.015995	0.98527	RF	80.75	Muscle	Yes
LR21A14DX381	24996987	Paired	0.0136444	0.98726	RF	80.18	Muscle	Yes
LR21A14DX382	21541469	Paired	0.0141139	0.987326	RF	82.49	Muscle	Yes
LR21A14DX383	22022592	Paired	0.0136205	0.987624	RF	80.45	Muscle	Yes
LR21A14DX384	22816023	Paired	0.0148874	0.98643	RF	79.31	Muscle	Yes
LR21A14DX385	24863007	Paired	0.0128788	0.988325	RF	80.38	Muscle	Yes
LR21A14DX386	27195209	Paired	0.0144389	0.986852	RF	80.9	Muscle	Yes
LR21A14DX387	19498824	Paired	0.0125598	0.988449	RF	81.08	Muscle	Yes
LR21A14DX388	26819618	Paired	0.0120653	0.989116	RF	81.74	Muscle	Yes
LR21A14DX389	19481585	Paired	0.0126088	0.988618	RF	80.15	Muscle	Yes
LR21A14DX390	25810199	Paired	0.0150749	0.986102	RF	79.7	Muscle	Yes
LR21A14DX391	19218534	Paired	0.0145654	0.986896	RF	80.05	Muscle	Yes
LR21A14DX392	24318364	Paired	0.0124164	0.988825	RF	78.56	Muscle	Yes
LR21A14DX393	24709211	Paired	0.0136683	0.987527	RF	76.86	Muscle	Yes
LR21A14DX394	19832972	Paired	0.0138086	0.98745	RF	78.17	Muscle	Yes
LR21A14DX395	18157028	Paired	0.0147033	0.986559	RF	79.43	Muscle	Yes
LR21A14DX396	22216329	Paired	0.0130459	0.987989	RF	81.55	Muscle	Yes
LR21A14DX397	22042319	Paired	0.0129168	0.988112	RF	78.54	Muscle	Yes
LR21A14DX398	18666292	Paired	0.0133678	0.987676	RF	80.61	Muscle	Yes
LR21A14DX399	19380401	Paired	0.0130537	0.988052	RF	80.65	Muscle	Yes
LR21A14DX400	24758082	Paired	0.0126808	0.988407	RF	76.83	Muscle	Yes
LR21A14DX401	25062041	Paired	0.0122224	0.988803	RF	78.06	Muscle	Yes
LR21A14DX402	26028326	Paired	0.0121159	0.988959	RF	78.68	Muscle	Yes
LR21A14DX403	25016657	Paired	0.0132715	0.987957	RF	80.15	Muscle	Yes
LR21A14DX404	26695615	Paired	0.01215	0.989035	RF	81.35	Muscle	Yes
LR21A14DX405	25127095	Paired	0.013196	0.987901	RF	78.36	Muscle	Yes
LR21A14DX406	22567451	Paired	0.0132819	0.988036	RF	81.16	Muscle	Yes
LR21A14DX407	22478529	Paired	0.0133542	0.988038	RF	81.16	Muscle	Yes
LR21A14DX408	25404428	Paired	0.0140509	0.987238	RF	81.02	Muscle	Yes
LR21A14DX409	20798691	Paired	0.0120948	0.989142	RF	82.01	Muscle	Yes
LR21A14DX410	25655033	Paired	0.0137926	0.987264	RF	82.16	Muscle	Yes
LR21A14DX411	26799526	Paired	0.0147823	0.986452	RF	79.9	Muscle	Yes
LR21A14DX412	24787735	Paired	0.0159923	0.985336	RF	81.05	Muscle	Yes
LR21A14DX413	24810291	Paired	0.0119991	0.989215	RF	81.2	Muscle	Yes
LR21A14DX414	25589435	Paired	0.0146083	0.986697	RF	83.06	Muscle	Yes

LR21A14DX415	25070256	Paired	0.0142311	0.98703	RF	82.58	Muscle	Yes
LR21A14DX416	26835669	Paired	0.014072	0.987278	RF	80.84	Muscle	Yes
LR21A14DX417	19791056	Paired	0.0145463	0.986676	RF	80.56	Muscle	Yes
LR21A14DX418	24495351	Paired	0.0124434	0.988569	RF	82.47	Muscle	Yes
LR21A14DX419	25911988	Paired	0.0136927	0.98747	RF	82.31	Muscle	Yes
LR21A14DX420	27108216	Paired	0.0132255	0.988123	RF	82.13	Muscle	Yes
LR21A14DX421	19009140	Paired	0.0138332	0.98729	RF	82.99	Muscle	Yes
LR21A14DX422	22383104	Paired	0.0131064	0.987997	RF	82.37	Muscle	Yes
LR21A14DX423	19958161	Paired	0.0150504	0.986245	RF	82.39	Muscle	Yes
LR21A14DX424	18730257	Paired	0.0147032	0.986379	RF	83.09	Muscle	Yes
LR21A14DX425	25817192	Paired	0.0134727	0.987958	RF	82.44	Muscle	Yes
LR21A14DX426	27024731	Paired	0.014745	0.986632	RF	83.87	Muscle	Yes
LR21A14DX427	25691654	Paired	0.0119517	0.98955	RF	84.94	Muscle	Yes
LR21A14DX428	27217106	Paired	0.0184041	0.983203	RF	84.39	Muscle	Yes
LR21A14DX429	26255310	Paired	0.0133406	0.988065	RF	84.1	Muscle	Yes
LR21A14DX430	26403893	Paired	0.0126094	0.988854	RF	82.05	Muscle	Yes
LR21A14DX431	25667273	Paired	0.0143114	0.987003	RF	82.17	Muscle	Yes
LR21A14DX432	27244777	Paired	0.0119039	0.989308	RF	82.66	Muscle	Yes
LR21A14DX433	25535320	Paired	0.0146033	0.986597	RF	78.96	Muscle	Yes
LR21A14DX434	26885688	Paired	0.0158897	0.98542	RF	83.78	Muscle	Yes
LR21A14DX435	25305192	Paired	0.0145605	0.98681	RF	81.1	Muscle	Yes
LR21A14DX436	26223969	Paired	0.0149831	0.986184	RF	80.66	Muscle	Yes
LR21A14DX437	26862606	Paired	0.014211	0.987111	RF	82.61	Muscle	Yes
LR21A14DX438	25725206	Paired	0.0141297	0.987003	RF	80.05	Muscle	Yes
LR21A14DX439	25383603	Paired	0.0178067	0.983591	RF	83.11	Muscle	Yes
LR21A14DX440	17840418	Paired	0.0144329	0.986951	RF	83.71	Muscle	Yes
LR21A14DX441	21831735	Paired	0.0162973	0.985237	RF	80.5	Muscle	Yes
LR21A14DX442	24802537	Paired	0.0137373	0.987456	RF	82.23	Muscle	Yes
LR21A14DX443	24794944	Paired	0.0148276	0.9865	RF	80.89	Muscle	Yes
LR21A14DX444	25663716	Paired	0.0151319	0.985954	RF	81.04	Muscle	Yes
LR21A14DX445	26624992	Paired	0.0131181	0.987971	RF	81.79	Muscle	Yes
LR21A14DX446	25164946	Paired	0.0136287	0.987523	RF	81.28	Muscle	Yes
LR21A14DX447	25089944	Paired	0.0154166	0.986056	RF	82.44	Muscle	Yes
LR21A14DX448	24918767	Paired	0.0139854	0.987305	RF	78.54	Muscle	Yes
LR21A14DX449	19321049	Paired	0.0126395	0.988682	RF	80.99	Muscle	Yes
LR21A14DX450	25310495	Paired	0.0138203	0.987385	RF	80.44	Muscle	Yes
LR21A14DX451	20885028	Paired	0.0134555	0.987736	RF	82.23	Muscle	Yes
LR21A14DX452	18689109	Paired	0.0140588	0.987253	RF	82.1	Muscle	Yes
LR21A14DX453	26023979	Paired	0.0135046	0.987685	RF	82.75	Muscle	Yes
LR21A14DX454	24837896	Paired	0.0131359	0.987985	RF	80.79	Muscle	Yes
LR21A14DX455	20841771	Paired	0.0144924	0.986658	RF	81.21	Muscle	Yes
LR21A14DX456	18140551	Paired	0.0174602	0.983763	RF	80.27	Muscle	Yes
LR21A14DX457	24904302	Paired	0.0164658	0.984944	RF	81.16	Muscle	Yes
LR21A14DX458	27055848	Paired	0.0167185	0.984523	RF	82.07	Muscle	Yes
LR21A14DX459	24429480	Paired	0.0151987	0.986069	RF	80.03	Muscle	Yes
LR21A14DX460	26024544	Paired	0.0130934	0.988253	RF	81.33	Muscle	Yes
LR21A14DX461	27011774	Paired	0.0159852	0.98532	RF	81.61	Muscle	Yes
LR21A14DX462	26996453	Paired	0.0126367	0.988715	RF	81.59	Muscle	Yes
LR21A14DX463	18760750	Paired	0.0143501	0.987065	RF	80.13	Muscle	Yes
LR21A14DX464	25737919	Paired	0.0138602	0.987521	RF	81.78	Muscle	Yes
LR21A14DX465	26683121	Paired	0.0146474	0.98657	RF	80.89	Muscle	Yes
LR21A14DX466	25589340	Paired	0.0157406	0.985555	RF	81.61	Muscle	Yes
LR21A14DX467	24904808	Paired	0.0133461	0.987861	RF	80.85	Muscle	Yes
LR21A14DX468	24779275	Paired	0.0131377	0.987956	RF	81.3	Muscle	Yes
LR21A14DX469	25426618	Paired	0.0128663	0.98814	RF	78.92	Muscle	Yes
LR21A14DX470	27200120	Paired	0.014331	0.987028	RF	82.81	Muscle	Yes
LR21A14DX471	25168461	Paired	0.0193589	0.982198	RF	80.77	Muscle	Yes
LR21A14DX472	25886185	Paired	0.0151271	0.98613	RF	79.89	Muscle	Yes

LR21A14DX473	19286111	Paired	0.0133465	0.988029	RF	79.96	Muscle	Yes
LR21A14DX474	26712965	Paired	0.0115925	0.989608	RF	80.45	Muscle	Yes
LR21A14DX475	24748165	Paired	0.0122028	0.98889	RF	80.75	Muscle	Yes
LR21A14DX476	19065977	Paired	0.0128289	0.988329	RF	82.26	Muscle	Yes
LR21A14DX477	26256371	Paired	0.0130454	0.988424	RF	80.45	Muscle	Yes
LR21A14DX478	17775486	Paired	0.0134761	0.987743	RF	80.14	Muscle	Yes
LR21A14DX479	18081797	Paired	0.0134839	0.987875	RF	77.32	Muscle	Yes
LR21A14DX480	26282880	Paired	0.0130075	0.988169	RF	76.61	Muscle	Yes
LR21A14DX481	23634549	Paired	0.012734	0.988254	RF	72.49	Muscle	Yes
LR21A14DX482	18138190	Paired	0.0130217	0.988221	RF	80.02	Muscle	Yes
LR21A14DX483	26829867	Paired	0.0138252	0.987288	RF	80	Muscle	Yes
LR21A14DX484	25213266	Paired	0.012742	0.988275	RF	79.25	Muscle	Yes
LR21A14DX485	19017455	Paired	0.0144471	0.986802	RF	77.95	Muscle	Yes
LR21A14DX486	18671300	Paired	0.0140964	0.987224	RF	77.06	Muscle	Yes
LR21A14DX487	26298662	Paired	0.0130535	0.988092	RF	77.5	Muscle	Yes
LR21A14DX488	25790044	Paired	0.0154764	0.985782	RF	76.98	Muscle	Yes
LR21A14DX489	24267779	Paired	0.0131183	0.987736	RF	79.62	Muscle	Yes
LR21A14DX490	20038009	Paired	0.0130914	0.987626	RF	78.7	Muscle	Yes
LR21A14DX491	21133523	Paired	0.0129176	0.987968	RF	80.03	Muscle	Yes
LR21A14DX492	23057872	Paired	0.0142701	0.986518	RF	80.06	Muscle	Yes
LR21A14DX493	23572643	Paired	0.0122336	0.988548	RF	80.2	Muscle	Yes
LR21A14DX494	22990283	Paired	0.0125454	0.98818	RF	80.11	Muscle	Yes
LR21A14DX495	24039858	Paired	0.0136528	0.987189	RF	80.53	Muscle	Yes
LR21A14DX496	20587520	Paired	0.012818	0.987975	RF	80.4	Muscle	Yes
LR21A14DX497	23916828	Paired	0.0127759	0.988003	RF	79.35	Muscle	Yes
LR21A14DX498	24073855	Paired	0.0120325	0.988809	RF	80.56	Muscle	Yes
LR21A14DX499	25127675	Paired	0.0126662	0.988111	RF	79.11	Muscle	Yes
LR21A14DX500	24569077	Paired	0.0121983	0.988532	RF	79.55	Muscle	Yes
LR21A14DX501	25960177	Paired	0.0114214	0.98947	RF	80.77	Muscle	Yes
LR21A14DX502	25770826	Paired	0.0126253	0.988268	RF	80.98	Muscle	Yes
LR21A14DX503	25710491	Paired	0.0123276	0.988674	RF	82.1	Muscle	Yes
LR21A14DX504	25632740	Paired	0.0126317	0.988254	RF	78.49	Muscle	Yes
LR21A14DX505	24556140	Paired	0.0138076	0.986802	RF	78.36	Muscle	Yes
LR21A14DX506	18760493	Paired	0.0154514	0.985148	RF	77.83	Muscle	Yes
LR21A14DX507	21161462	Paired	0.0133169	0.987338	RF	76.9	Muscle	Yes
LR21A14DX508	21340455	Paired	0.0127339	0.98781	RF	74.74	Muscle	Yes
LR21A14DX509	21857306	Paired	0.016014	0.984568	RF	78.36	Muscle	Yes
LR21A14DX510	22532836	Paired	0.012585	0.988034	RF	77.44	Muscle	Yes
LR21A14DX511	24525018	Paired	0.0132715	0.987306	RF	75.41	Muscle	Yes
LR21A14DX512	20934921	Paired	0.0129243	0.987698	RF	77.71	Muscle	Yes
LR21A14DX513	24898044	Paired	0.0128468	0.988188	RF	80.8	Muscle	Yes
LR21A14DX514	17088034	Paired	0.0132704	0.98745	RF	80.52	Muscle	Yes
LR21A14DX515	19626936	Paired	0.0129226	0.987761	RF	79.54	Muscle	Yes
LR21A14DX516	22765570	Paired	0.0127583	0.988017	RF	79.61	Muscle	Yes
LR21A14DX517	20804018	Paired	0.0133953	0.987408	RF	80.4	Muscle	Yes
LR21A14DX518	22023983	Paired	0.0141084	0.98682	RF	80.39	Muscle	Yes
LR21A14DX519	22479491	Paired	0.0136978	0.987132	RF	79.04	Muscle	Yes
LR21A14DX520	19746288	Paired	0.0127811	0.988167	RF	80.51	Muscle	Yes
LR21A14DX521	19872234	Paired	0.0136345	0.987339	RF	81.41	Muscle	Yes
LR21A14DX522	24690350	Paired	0.012843	0.988129	RF	81.12	Muscle	Yes
LR21A14DX523	23187119	Paired	0.013512	0.987374	RF	71.32	Muscle	Yes
LR21A14DX524	17866743	Paired	0.0123895	0.988629	RF	82.4	Muscle	Yes
LR21A14DX525	24225191	Paired	0.0117898	0.989146	RF	82.21	Muscle	Yes
LR21A14DX526	18615323	Paired	0.0129383	0.988139	RF	82.59	Muscle	Yes
LR21A14DX527	17386576	Paired	0.013357	0.987613	RF	82.24	Muscle	Yes
LR21A14DX528	25092324	Paired	0.0126851	0.988469	RF	82.74	Muscle	Yes
LR21A14DX529	23124381	Paired	0.0117771	0.989154	RF	79.99	Muscle	Yes
LR21A14DX530	18226537	Paired	0.0136107	0.987164	RF	80.03	Muscle	Yes

LR21A14DX531	21899719	Paired	0.0129429	0.987816	RF	79.31	Muscle	Yes
LR21A14DX532	21466237	Paired	0.0132415	0.987632	RF	80.61	Muscle	Yes
LR21A14DX533	21444646	Paired	0.0130292	0.987768	RF	81.08	Muscle	Yes
LR21A14DX534	23742799	Paired	0.0128678	0.988117	RF	80.77	Muscle	Yes
LR21A14DX535	21061560	Paired	0.0123566	0.988453	RF	80.66	Muscle	Yes
LR21A14DX536	20688729	Paired	0.0127029	0.988063	RF	79.58	Muscle	Yes
LR21A14DX537	22986715	Paired	0.0127709	0.988299	RF	80.7	Muscle	Yes
LR21A14DX538	18612724	Paired	0.0130006	0.987764	RF	79.78	Muscle	Yes
LR21A14DX539	18066738	Paired	0.013334	0.9874	RF	78.56	Muscle	Yes
LR21A14DX540	21760031	Paired	0.013031	0.987791	RF	79.83	Muscle	Yes
LR21A14DX541	23681632	Paired	0.012275	0.988505	RF	80.88	Muscle	Yes
LR21A14DX542	19821829	Paired	0.0133745	0.987354	RF	78.89	Muscle	Yes
LR21G9DX153	14477386	Paired	0.0220487	0.978625	RF	82.92	Muscle	Yes
LR21G9DX154	20062945	Paired	0.0322091	0.968584	RF	83.72	Muscle	Yes
LR21G9DX155	20574865	Paired	0.0925517	0.907278	RF	81.29	Muscle	Yes
LR21G9DX156	21628258	Paired	0.0762493	0.923775	RF	81.7	Muscle	Yes
LR21G9DX157	21543229	Paired	0.0777987	0.921916	RF	83.04	Muscle	Yes
LR21G9DX158	14487869	Paired	0.0749448	0.925022	RF	80.15	Muscle	Yes
LR21G9DX159	22370953	Paired	0.0486001	0.951591	RF	83.5	Muscle	Yes
LR21G9DX160	23105346	Paired	0.116936	0.881633	RF	82.58	Muscle	Yes
LR21G9DX161	17687319	Paired	0.0215308	0.979231	RF	82.88	Muscle	Yes
LR21G9DX162	15359585	Paired	0.028696	0.972056	RF	83.42	Muscle	Yes
LR21G9DX163	15008153	Paired	0.0760747	0.924088	RF	79.96	Muscle	Yes
LR21G9DX164	15636105	Paired	0.0455339	0.954929	RF	82.5	Muscle	Yes
LR21G9DX165	14063673	Paired	0.0873466	0.91277	RF	80.74	Muscle	Yes
LR21G9DX166	18622551	Paired	0.117955	0.881205	RF	81.81	Muscle	Yes
LR21G9DX167	17058810	Paired	0.105699	0.89341	RF	82.37	Muscle	Yes
LR21G9DX168	20177770	Paired	0.136659	0.86197	RF	82.13	Muscle	Yes
LR21G9DX169	14882216	Paired	0.0740115	0.926121	RF	81.54	Muscle	Yes
LR21G9DX170	14385925	Paired	0.0855903	0.914203	RF	82.25	Muscle	Yes
LR21G9DX171	15781922	Paired	0.0856394	0.914238	RF	81.23	Muscle	Yes
LR21G9DX172	17058902	Paired	0.0862896	0.913591	RF	82.12	Muscle	Yes
LR21G9DX173	14769738	Paired	0.0821009	0.917902	RF	81.12	Muscle	Yes
LR21G9DX174	17650494	Paired	0.0925972	0.907012	RF	81.61	Muscle	Yes
LR21G9DX175	15673959	Paired	0.0809596	0.919162	RF	76.23	Muscle	Yes
LR21G9DX176	19392188	Paired	0.106805	0.892379	RF	82.76	Muscle	Yes
LR21G9DX177	16222647	Paired	0.184528	0.813293	RF	78.82	Muscle	Yes
LR21G9DX178	19541463	Paired	0.144592	0.853648	RF	80.97	Muscle	Yes
LR21G9DX179	18725988	Paired	0.173707	0.82429	RF	78.65	Muscle	Yes
LR21G9DX180	23180946	Paired	0.183213	0.814724	RF	77.14	Muscle	Yes
LR21G9DX181	18957145	Paired	0.174171	0.823805	RF	79	Muscle	Yes
LR21G9DX182	14253015	Paired	0.139141	0.858911	RF	81.36	Muscle	Yes
LR21G9DX183	22384815	Paired	0.154577	0.843179	RF	81.14	Muscle	Yes
LR21G9DX184	23780225	Paired	0.158938	0.83875	RF	81.19	Muscle	Yes
LR21G9DX185	15392368	Paired	0.0504997	0.949712	RF	83.08	Muscle	Yes
LR21G9DX186	20124426	Paired	0.0649542	0.935137	RF	80.36	Muscle	Yes
LR21G9DX187	18529937	Paired	0.102096	0.896884	RF	82.63	Muscle	Yes
LR21G9DX188	18279252	Paired	0.0948935	0.904812	RF	76.6	Muscle	Yes
LR21G9DX189	15895897	Paired	0.0630516	0.936807	RF	82.83	Muscle	Yes
LR21G9DX190	15978323	Paired	0.0791838	0.92083	RF	81.99	Muscle	Yes
LR21G9DX191	18386054	Paired	0.089826	0.909919	RF	82.73	Muscle	Yes
LR21G9DX192	15770274	Paired	0.07439	0.925307	RF	82.55	Muscle	Yes
LR21G9DX193	21165712	Paired	0.135168	0.863846	RF	80.43	Muscle	Yes
LR21G9DX194	24868350	Paired	0.0677158	0.932223	RF	82.23	Muscle	Yes
LR21G9DX195	21439447	Paired	0.0573734	0.943147	RF	81.93	Muscle	Yes
LR21G9DX196	15304714	Paired	0.0935844	0.905754	RF	82.48	Muscle	Yes
LR21G9DX197	14861642	Paired	0.0646011	0.935232	RF	83.87	Muscle	Yes
LR21G9DX198	21262594	Paired	0.0629993	0.93685	RF	82.82	Muscle	Yes

LR21G9DX199	24550352	Paired	0.0824006	0.917105	RF	83.2	Muscle	Yes
LR21G9DX200	24307945	Paired	0.105364	0.893599	RF	83.32	Muscle	Yes
LR21G9DX201	20652264	Paired	0.137003	0.861405	RF	81.23	Muscle	Yes
LR21G9DX202	19789610	Paired	0.137967	0.860327	RF	81.33	Muscle	Yes
LR21G9DX203	21521737	Paired	0.14179	0.856341	RF	81.27	Muscle	Yes
LR21G9DX204	23044012	Paired	0.138887	0.859243	RF	81.72	Muscle	Yes
LR21G9DX205	15270163	Paired	0.127643	0.870789	RF	81.66	Muscle	Yes
LR21G9DX206	19010863	Paired	0.144043	0.85399	RF	80.75	Muscle	Yes
LR21G9DX207	22712686	Paired	0.139262	0.858769	RF	81.99	Muscle	Yes
LR21G9DX208	23451547	Paired	0.136695	0.861059	RF	82.16	Muscle	Yes
LR21G9DX209	24198632	Paired	0.10707	0.892005	RF	82.1	Muscle	Yes
LR21G9DX210	20687660	Paired	0.0899669	0.909469	RF	82.33	Muscle	Yes
LR21G9DX211	26436888	Paired	0.133047	0.864932	RF	82.94	Muscle	Yes
LR21G9DX212	19225071	Paired	0.0928832	0.906303	RF	83.11	Muscle	Yes
LR21G9DX213	23823361	Paired	0.131847	0.866703	RF	80.8	Muscle	Yes
LR21G9DX214	15380350	Paired	0.114264	0.885014	RF	77.77	Muscle	Yes
LR21G9DX215	17103937	Paired	0.0289622	0.971605	RF	81.82	Muscle	Yes
LR21G9DX216	21493656	Paired	0.101167	0.898225	RF	81.84	Muscle	Yes
LR21G9DX217	19418507	Paired	0.140228	0.858348	RF	78.47	Muscle	Yes
LR21G9DX218	17044667	Paired	0.138898	0.859309	RF	79.94	Muscle	Yes
LR21G9DX219	19234011	Paired	0.128409	0.870047	RF	81.16	Muscle	Yes
LR21G9DX220	18806554	Paired	0.166527	0.832427	RF	71.73	Muscle	Yes
LR21G9DX221	15542371	Paired	0.119741	0.879876	RF	80.42	Muscle	Yes
LR21G9DX222	16717832	Paired	0.0816016	0.918127	RF	82.85	Muscle	Yes
LR21G9DX223	17957129	Paired	0.086922	0.912813	RF	82.69	Muscle	Yes
LR21G9DX224	18101239	Paired	0.0653374	0.934654	RF	71.67	Muscle	Yes
LR21G9DX225	22895461	Paired	0.125306	0.873399	RF	80.29	Muscle	Yes
LR21G9DX226	20297712	Paired	0.163308	0.835691	RF	70.55	Muscle	Yes
LR21G9DX227	20718529	Paired	0.143861	0.855329	RF	71.87	Muscle	Yes
LR21G9DX228	23039472	Paired	0.127739	0.870901	RF	80.95	Muscle	Yes
LR21G9DX229	17611628	Paired	0.0783808	0.921718	RF	77.35	Muscle	Yes
LR21G9DX230	23491610	Paired	0.0888787	0.910665	RF	82.45	Muscle	Yes
LR21G9DX231	17192673	Paired	0.0696569	0.930427	RF	72.26	Muscle	Yes
LR21G9DX232	21468483	Paired	0.0882142	0.91187	RF	71.51	Muscle	Yes
LR21G9DX233	17169943	Paired	0.120622	0.878046	RF	80.59	Muscle	Yes
LR21G9DX234	19847592	Paired	0.12515	0.873548	RF	80.73	Muscle	Yes
LR21G9DX235	15978081	Paired	0.133922	0.864598	RF	80.71	Muscle	Yes
LR21G9DX236	14328819	Paired	0.148427	0.849939	RF	79.92	Muscle	Yes
LR21G9DX237	15521356	Paired	0.0815672	0.918402	RF	72.8	Muscle	Yes
LR21G9DX238	18348414	Paired	0.0779219	0.92212	RF	82.39	Muscle	Yes
LR21G9DX239	21462904	Paired	0.0672356	0.932851	RF	82.51	Muscle	Yes
LR21G9DX240	18513072	Paired	0.127142	0.872583	RF	72.38	Muscle	Yes
LR21G9DX241	20166866	Paired	0.0251225	0.975627	RF	81.65	Muscle	Yes
LR21G9DX242	18543265	Paired	0.0875416	0.91254	RF	71.64	Muscle	Yes
LR21G9DX243	24010926	Paired	0.0636293	0.936784	RF	78.65	Muscle	Yes
LR21G9DX244	14862882	Paired	0.0865843	0.913189	RF	81.98	Muscle	Yes
LR21G9DX245	22803601	Paired	0.0303007	0.97031	RF	73.47	Muscle	Yes
LR21G9DX246	14666195	Paired	0.0281234	0.972946	RF	83.15	Muscle	Yes
LR21G9DX247	24419992	Paired	0.0190368	0.981783	RF	84.34	Muscle	Yes
LR21G9DX248	24261414	Paired	0.0224443	0.978459	RF	84.85	Muscle	Yes
LR21G9DX249	23126216	Paired	0.0232197	0.977867	RF	82.71	Muscle	Yes
LR21G9DX250	22411909	Paired	0.0705039	0.929914	RF	81.62	Muscle	Yes
LR21G9DX251	20388690	Paired	0.0550282	0.945291	RF	82.63	Muscle	Yes
LR21G9DX252	18651268	Paired	0.0477317	0.952695	RF	82.57	Muscle	Yes
LR21G9DX253	19296560	Paired	0.0238661	0.976672	RF	75.07	Muscle	Yes
LR21G9DX254	20359227	Paired	0.0280086	0.972514	RF	74.24	Muscle	Yes
LR21G9DX255	18669424	Paired	0.0236409	0.976884	RF	78.35	Muscle	Yes
LR21G9DX256	19236458	Paired	0.0282227	0.97311	RF	83.76	Muscle	Yes

LR21G9DX257	17848893	Paired	0.0250799	0.976138	RF	81.05	Muscle	Yes
LR21G9DX258	18776740	Paired	0.0583027	0.942084	RF	71.76	Muscle	Yes
LR21G9DX259	20665600	Paired	0.087957	0.911964	RF	73.42	Muscle	Yes
LR21G9DX260	18753421	Paired	0.050477	0.949987	RF	82.49	Muscle	Yes
LR21G9DX261	14190505	Paired	0.0259039	0.974748	RF	79.51	Muscle	Yes
LR21G9DX262	16079878	Paired	0.029125	0.972601	RF	83.65	Muscle	Yes
LR21G9DX263	20344256	Paired	0.0295248	0.970825	RF	75.81	Muscle	Yes
LR21G9DX264	22336408	Paired	0.0274474	0.973114	RF	80.2	Muscle	Yes
LR21G9DX265	18134658	Paired	0.129175	0.86981	RF	80.34	Muscle	Yes
LR21G9DX266	17525322	Paired	0.143383	0.855311	RF	80.02	Muscle	Yes
LR21G9DX267	19933217	Paired	0.0413059	0.95922	RF	81.71	Muscle	Yes
LR21G9DX268	15583889	Paired	0.0536839	0.946593	RF	82.56	Muscle	Yes
LR21G9DX269	13889195	Paired	0.021413	0.979265	RF	81.2	Muscle	Yes
LR21G9DX270	18152201	Paired	0.0258778	0.974642	RF	74.66	Muscle	Yes
LR21G9DX271	23501415	Paired	0.0226895	0.978258	RF	77.18	Muscle	Yes
LR21G9DX272	18477594	Paired	0.0269907	0.973499	RF	74.26	Muscle	Yes
LR21I23DX357	25181942	Paired	0.121702	0.876779	RF	84.63	Muscle	Yes
LR21I23DX358	25440053	Paired	0.0908851	0.908311	RF	84.97	Muscle	Yes
LR21I23DX359	24296648	Paired	0.110556	0.888234	RF	83.89	Muscle	Yes
LR21I23DX360	22292888	Paired	0.113413	0.884937	RF	84.21	Muscle	Yes
LR21I23DX361	24676573	Paired	0.104649	0.894331	RF	84.81	Muscle	Yes
LR21I23DX362	22664689	Paired	0.12705	0.870783	RF	84.18	Muscle	Yes
LR21I23DX363	24425491	Paired	0.114821	0.883495	RF	85.41	Muscle	Yes
LR21I23DX364	25602239	Paired	0.106496	0.892102	RF	84.38	Muscle	Yes
LR21I23DX365	24140928	Paired	0.124528	0.873805	RF	84.5	Muscle	Yes
LR21I23DX366	20163105	Paired	0.114322	0.884159	RF	84.18	Muscle	Yes
LR21I23DX367	22991191	Paired	0.112578	0.885967	RF	84.78	Muscle	Yes
LR21I23DX368	22301272	Paired	0.116132	0.882049	RF	84.23	Muscle	Yes
LR21I23DX369	20359599	Paired	0.0985024	0.900222	RF	84.44	Muscle	Yes
LR21I23DX370	20248420	Paired	0.112282	0.886047	RF	84.59	Muscle	Yes
LR21I23DX371	20433601	Paired	0.107722	0.890937	RF	84.92	Muscle	Yes
LR21I23DX372	20818056	Paired	0.112848	0.885324	RF	84.72	Muscle	Yes
LR21I23DX373	19823487	Paired	0.122332	0.876324	RF	84.65	Muscle	Yes
LR21I23DX374	25605987	Paired	0.0990105	0.899849	RF	85.39	Muscle	Yes
LR21I23DX375	21377608	Paired	0.102477	0.896352	RF	84.08	Muscle	Yes
LR21I23DX376	20843914	Paired	0.101864	0.896944	RF	85.08	Muscle	Yes
LR21I23DX377	23054539	Paired	0.102199	0.896439	RF	85.07	Muscle	Yes
LR21I23DX378	22134424	Paired	0.121241	0.876812	RF	84.17	Muscle	Yes
LR21I23DX379	25127040	Paired	0.0957513	0.902987	RF	85.59	Muscle	Yes
LR21I23DX395	23250770	Paired	0.100517	0.898052	RF	84.96	Muscle	Yes
LR21I23DX396	23264439	Paired	0.10179	0.896806	RF	85.71	Muscle	Yes
LR21I23DX397	24917431	Paired	0.108952	0.889537	RF	84.69	Muscle	Yes
LR21I23DX398	21268832	Paired	0.0973092	0.901777	RF	84.83	Muscle	Yes
LR21I23DX399	20210450	Paired	0.100803	0.898313	RF	85.18	Muscle	Yes
LR21I23DX400	21605798	Paired	0.105853	0.892807	RF	84.47	Muscle	Yes
LR21I23DX401	19332212	Paired	0.106497	0.892187	RF	84.92	Muscle	Yes
LR21I23DX402	19686589	Paired	0.101886	0.896753	RF	85.07	Muscle	Yes
LR21I23DX403	19213296	Paired	0.109413	0.889337	RF	85.28	Muscle	Yes
LR21I23DX404	20904261	Paired	0.102655	0.896018	RF	85.06	Muscle	Yes
LR21I23DX405	24885372	Paired	0.116661	0.881672	RF	85.67	Muscle	Yes
LR21I23DX406	22702069	Paired	0.115416	0.882857	RF	85.23	Muscle	Yes
LR21I23DX407	23866015	Paired	0.109847	0.888492	RF	84.76	Muscle	Yes
LR21I23DX408	24997467	Paired	0.11276	0.885807	RF	85.03	Muscle	Yes
LR21I23DX409	23206937	Paired	0.131769	0.866132	RF	84.84	Muscle	Yes
LR21I23DX410	21623744	Paired	0.115967	0.882103	RF	85.7	Muscle	Yes
LR21I23DX411	25466526	Paired	0.112723	0.885684	RF	85.13	Muscle	Yes
LR21I23DX412	21936245	Paired	0.114305	0.884127	RF	84.99	Muscle	Yes
LR21I23DX413	26600451	Paired	0.0904295	0.908554	RF	86.1	Muscle	Yes

LR21I23DX414	24488192	Paired	0.103006	0.895637	RF	84.97	Muscle	Yes
LR21I23DX415	25559679	Paired	0.102519	0.89602	RF	85.27	Muscle	Yes
LR21I23DX416	24104503	Paired	0.0917606	0.907082	RF	85.69	Muscle	Yes
LR21I23DX417	23191653	Paired	0.109429	0.888858	RF	85.09	Muscle	Yes
LR21I23DX418	24109157	Paired	0.0933588	0.905421	RF	85.32	Muscle	Yes
LR21I23DX419	22444198	Paired	0.100409	0.898315	RF	84.96	Muscle	Yes
LR21I23DX420	23297594	Paired	0.115081	0.882913	RF	85.34	Muscle	Yes
LR21I23DX421	20511370	Paired	0.136089	0.862444	RF	84.87	Muscle	Yes
LR21I23DX422	20566065	Paired	0.108036	0.890425	RF	85.17	Muscle	Yes
LR21I23DX423	19813892	Paired	0.100511	0.898038	RF	85.55	Muscle	Yes
LR21I23DX424	19426251	Paired	0.119873	0.878676	RF	85.6	Muscle	Yes
LR21I23DX425	19836362	Paired	0.116945	0.881574	RF	85.86	Muscle	Yes
LR21I23DX426	19414322	Paired	0.100695	0.89797	RF	86.49	Muscle	Yes
LR21I23DX427	20155696	Paired	0.0988792	0.899991	RF	85.98	Muscle	Yes
LR21I23DX428	24446302	Paired	0.110432	0.888094	RF	85.54	Muscle	Yes
LR21I23DX429	26067216	Paired	0.104491	0.894298	RF	85.55	Muscle	Yes
LR21I23DX430	24228325	Paired	0.115017	0.883691	RF	84.47	Muscle	Yes
LR21I23DX431	22457413	Paired	0.105368	0.893193	RF	85.21	Muscle	Yes
LR21I23DX432	24250526	Paired	0.0923391	0.906666	RF	86.06	Muscle	Yes
LR21I23DX433	23923295	Paired	0.106475	0.892128	RF	85.29	Muscle	Yes
LR21I23DX434	23515240	Paired	0.0975408	0.901362	RF	85.34	Muscle	Yes
LR21I23DX435	20577098	Paired	0.117269	0.881594	RF	85.9	Muscle	Yes
LR21I23DX436	25891329	Paired	0.100119	0.898689	RF	85.83	Muscle	Yes
LR21I23DX437	24557778	Paired	0.120074	0.878271	RF	84.86	Muscle	Yes
LR21I23DX438	26313347	Paired	0.111924	0.886697	RF	84.41	Muscle	Yes
LR21I23DX439	25373292	Paired	0.113142	0.885177	RF	85.07	Muscle	Yes
LR21I23DX440	24142027	Paired	0.119618	0.879067	RF	85.09	Muscle	Yes
LR21I23DX441	23975786	Paired	0.130643	0.867343	RF	83.42	Muscle	Yes
LR21I23DX442	25916828	Paired	0.106866	0.891867	RF	85.31	Muscle	Yes
LR21I23DX443	25672292	Paired	0.0976385	0.901137	RF	85.53	Muscle	Yes
LR21I23DX444	25338375	Paired	0.128066	0.869784	RF	84.48	Muscle	Yes
LR21I23DX445	23991183	Paired	0.115209	0.882641	RF	87.52	Muscle	Yes
LR21I23DX446	24523197	Paired	0.112073	0.885915	RF	87.55	Muscle	Yes
LR21I23DX447	24141639	Paired	0.11	0.888764	RF	88.2	Muscle	Yes
LR21I23DX448	25665018	Paired	0.10568	0.892584	RF	88.48	Muscle	Yes
LR21I23DX449	25255313	Paired	0.119494	0.878338	RF	87.43	Muscle	Yes
LR21I23DX450	26858025	Paired	0.113129	0.884761	RF	87.9	Muscle	Yes
LR21I23DX451	24906390	Paired	0.117725	0.880352	RF	87.42	Muscle	Yes
LR21I23DX452	21787485	Paired	0.128493	0.869381	RF	87.34	Muscle	Yes
LR21I23DX453	27113217	Paired	0.128737	0.869142	RF	87.96	Muscle	Yes
LR21I23DX454	24793763	Paired	0.129045	0.868971	RF	88.41	Muscle	Yes
LR21I23DX455	20118512	Paired	0.124377	0.873764	RF	89.25	Muscle	Yes
LR21I23DX456	25068861	Paired	0.124968	0.873051	RF	88.66	Muscle	Yes
LR21I23DX457	21740054	Paired	0.122713	0.875116	RF	89.01	Muscle	Yes
LR21I23DX458	24588022	Paired	0.127309	0.871038	RF	88.31	Muscle	Yes
LR21I23DX459	26056492	Paired	0.125128	0.873089	RF	88.39	Muscle	Yes
LR21I23DX460	19542569	Paired	0.131837	0.866021	RF	87.93	Muscle	Yes
LR21I23DX461	21902274	Paired	0.122668	0.87518	RF	88.23	Muscle	Yes
LR21I23DX462	22126142	Paired	0.123674	0.874652	RF	88.56	Muscle	Yes
LR21I23DX463	20780392	Paired	0.126613	0.871523	RF	88.46	Muscle	Yes
LR21I23DX464	23432140	Paired	0.117767	0.880112	RF	88.05	Muscle	Yes
LR21I23DX465	23826424	Paired	0.115383	0.882389	RF	88.66	Muscle	Yes
LR21I23DX466	25197040	Paired	0.118245	0.879606	RF	88.37	Muscle	Yes
LR21I23DX467	25437735	Paired	0.120229	0.877995	RF	88.28	Muscle	Yes
LR21I23DX468	27020535	Paired	0.102845	0.895252	RF	88.73	Muscle	Yes
LR21I23DX469	24744649	Paired	0.114499	0.883774	RF	87.99	Muscle	Yes
LR21I23DX470	26361208	Paired	0.107977	0.890139	RF	88.01	Muscle	Yes
LR21I23DX471	26983288	Paired	0.107705	0.890442	RF	88.31	Muscle	Yes

LR21I23DX472	25054025	Paired	0.107685	0.890585	RF	88.36	Muscle	Yes
LR21I23DX473	22433263	Paired	0.104932	0.893353	RF	89.07	Muscle	Yes
LR21I23DX475	26596366	Paired	0.109269	0.888913	RF	88.36	Muscle	Yes
LR21I23DX476	25534072	Paired	0.11262	0.885936	RF	84.92	Muscle	Yes
LR21I23DX477	24751975	Paired	0.103201	0.895123	RF	88.75	Muscle	Yes
LR21I23DX478	22277843	Paired	0.103173	0.895054	RF	88.75	Muscle	Yes
LR21I23DX479	25267602	Paired	0.100913	0.897471	RF	88.39	Muscle	Yes
LR21I23DX480	25275038	Paired	0.104347	0.894319	RF	88.84	Muscle	Yes
LR21I23DX481	26646071	Paired	0.0899443	0.908531	RF	88.8	Muscle	Yes
LR21I23DX482	25310580	Paired	0.0935611	0.904763	RF	88.33	Muscle	Yes
LR21I23DX483	24996648	Paired	0.114669	0.883539	RF	88.86	Muscle	Yes
LR21I23DX484	22721705	Paired	0.106175	0.892035	RF	88.2	Muscle	Yes
LR21I23DX485	23315206	Paired	0.100067	0.89831	RF	89	Muscle	Yes
LR21I23DX486	23751398	Paired	0.112091	0.886239	RF	88.37	Muscle	Yes
LR21I23DX487	25519329	Paired	0.114281	0.883837	RF	88.58	Muscle	Yes
LR21I23DX488	25622184	Paired	0.110261	0.888027	RF	88.33	Muscle	Yes
LR21I23DX489	24765533	Paired	0.108907	0.889498	RF	88.26	Muscle	Yes
LR21I23DX490	25738036	Paired	0.101232	0.89706	RF	88.42	Muscle	Yes
LR21I23DX491	26406092	Paired	0.113221	0.885114	RF	88.88	Muscle	Yes
LR21I23DX492	26095418	Paired	0.0615857	0.938598	RF	89.11	Muscle	Yes
LR21I23DX493	25832440	Paired	0.0980234	0.900254	RF	88.37	Muscle	Yes
LR21I23DX494	25242868	Paired	0.111087	0.887002	RF	88.6	Muscle	Yes
LR21I23DX495	25121981	Paired	0.118081	0.880267	RF	88.99	Muscle	Yes
LR21I23DX496	26373704	Paired	0.105265	0.893057	RF	88.24	Muscle	Yes
LR21I23DX497	26525838	Paired	0.103014	0.895296	RF	88.41	Muscle	Yes
LR21I23DX498	26336969	Paired	0.105241	0.892941	RF	88.5	Muscle	Yes
LR21I23DX499	25317011	Paired	0.133654	0.863933	RF	87.96	Muscle	Yes
LR21I23DX500	22742227	Paired	0.130329	0.867355	RF	87.43	Muscle	Yes
LR21I23DX501	24007087	Paired	0.124209	0.873475	RF	87.65	Muscle	Yes
LR21I23DX502	26140603	Paired	0.120032	0.877826	RF	87.58	Muscle	Yes
LR21I23DX503	25204340	Paired	0.124342	0.873485	RF	88.06	Muscle	Yes
LR21I23DX504	25921321	Paired	0.117621	0.880299	RF	87.21	Muscle	Yes
LR21I23DX505	24723561	Paired	0.113185	0.884804	RF	88.07	Muscle	Yes
LR21I23DX506	24651133	Paired	0.107551	0.890565	RF	88.41	Muscle	Yes
LR21I23DX507	23236792	Paired	0.0332236	0.967465	RF	89.02	Muscle	Yes
LR21I23DX508	23989461	Paired	0.0849915	0.914511	RF	88.52	Muscle	Yes
LR21I23DX509	22579444	Paired	0.0926697	0.906213	RF	88.24	Muscle	Yes
LR21I23DX510	24075287	Paired	0.114552	0.883713	RF	87.77	Muscle	Yes
LR21I23DX511	24046569	Paired	0.115161	0.883136	RF	88.26	Muscle	Yes
LR21I23DX512	24682274	Paired	0.115844	0.882125	RF	87.98	Muscle	Yes
LR21I23DX513	24203001	Paired	0.114348	0.883658	RF	87.39	Muscle	Yes
LR21I23DX514	24699593	Paired	0.112179	0.885928	RF	88.26	Muscle	Yes
LR21I23DX515	24888019	Paired	0.125074	0.87255	RF	87.92	Muscle	Yes
LR21I23DX516	25661502	Paired	0.126635	0.871033	RF	88.3	Muscle	Yes
LR21I23DX517	25839735	Paired	0.117565	0.88012	RF	88.23	Muscle	Yes
LR21I23DX518	27112897	Paired	0.135328	0.862287	RF	87.9	Muscle	Yes
LR21I23DX519	26197701	Paired	0.131062	0.866512	RF	88.16	Muscle	Yes
LR21I23DX520	25426712	Paired	0.129714	0.867817	RF	87.45	Muscle	Yes
LR21I23DX521	25989151	Paired	0.108243	0.889929	RF	87.06	Muscle	Yes
LR21I23DX522	24980474	Paired	0.126209	0.871126	RF	79.47	Muscle	Yes
LR21I23DX523	25477736	Paired	0.0441349	0.956317	RF	88.92	Muscle	Yes
LR21I23DX524	25714214	Paired	0.114437	0.883876	RF	87.67	Muscle	Yes
LR21I23DX525	24315613	Paired	0.104702	0.893956	RF	88.44	Muscle	Yes
LR21I23DX526	25893781	Paired	0.114831	0.883839	RF	87.86	Muscle	Yes
LR21I23DX527	24833118	Paired	0.130906	0.866971	RF	88.05	Muscle	Yes
LR21I23DX528	26752404	Paired	0.145648	0.852085	RF	87.01	Muscle	Yes
LR21I23DX529	27022127	Paired	0.12848	0.869087	RF	87.66	Muscle	Yes
LR21I23DX530	23895516	Paired	0.131349	0.866169	RF	88.03	Muscle	Yes

LR21I23DX531	22666030	Paired	0.104149	0.894186	RF	88.32	Muscle	Yes
LR21I23DX532	23773273	Paired	0.10451	0.894199	RF	88.85	Muscle	Yes
LR21I23DX533	22780313	Paired	0.123154	0.874944	RF	88.22	Muscle	Yes
LR21I23DX534	23665075	Paired	0.114013	0.883944	RF	88.4	Muscle	Yes
LR21I23DX535	23550415	Paired	0.125443	0.872561	RF	88.26	Muscle	Yes
LR21I23DX536	24040770	Paired	0.120893	0.876888	RF	87.51	Muscle	Yes
LR21I23DX537	23999940	Paired	0.121677	0.876505	RF	88.35	Muscle	Yes
LR21I23DX538	22078978	Paired	0.118167	0.879645	RF	88.04	Muscle	Yes
LR21I23DX539	25215797	Paired	0.0481539	0.951928	RF	88.66	Muscle	Yes
LR21I23DX540	26403760	Paired	0.0858937	0.913092	RF	88.6	Muscle	Yes
LR21I23DX541	26777935	Paired	0.0720347	0.927527	RF	88.41	Muscle	Yes
LR21I23DX542	26245677	Paired	0.0760407	0.923156	RF	88.43	Muscle	Yes
LR21I23DX543	26993153	Paired	0.0865397	0.912837	RF	88.83	Muscle	Yes
LR21J12DX121	20276504	Paired	0.0391634	0.96213	RF	89.22	Muscle	Yes
LR21J12DX122	20270595	Paired	0.0402019	0.960776	RF	89.41	Muscle	Yes
LR21J12DX123	21734150	Paired	0.029234	0.971976	RF	89.53	Muscle	Yes
LR21J12DX124	20054377	Paired	0.0395487	0.961568	RF	89.35	Muscle	Yes
SRX3174434	29690619	Paired	0.0215641	0.979062	RF	86.76	Muscle	No
SRX3174435	23048987	Paired	0.97901	0.0216017	FR	85.7	Muscle	No
SRX3174436	26715694	Paired	0.0222362	0.978326	RF	88.86	Muscle	No
SRX3174437	25603870	Paired	0.0214013	0.979208	RF	88.46	Muscle	No
SRX3174438	24766126	Paired	0.0232834	0.977246	RF	88.52	Muscle	No
SRX3174439	15289879	Paired	0.0221109	0.978635	RF	84.69	Muscle	No
SRX8483892	45769752	Paired	0.0194105	0.981491	RF	89.35	Liver	No
SRX8483893	46253803	Paired	0.0245685	0.976141	RF	88.1	Liver	No
SRX8483895	40527288	Paired	0.0185361	0.98218	RF	87.3	Liver	No
SRX8483896	49085106	Paired	0.033238	0.967874	RF	88.06	Lung	No
SRX8483897	46735621	Paired	0.0359084	0.964913	RF	87.21	Lung	No
SRX8483898	49449751	Paired	0.036272	0.964199	RF	87.5	Lung	No
SRX8483899	49258055	Paired	0.0375598	0.961516	RF	75.38	Adipose	No
SRX3174440	26269616	Paired	0.982188	0.0185397	FR	86.75	Muscle	No
SRX3174441	18083387	Paired	0.02519	0.975149	RF	85.36	Muscle	No
SRX3174442	24487921	Paired	0.0242128	0.97645	RF	85.66	Muscle	No
SRX3174443	27445824	Paired	0.977695	0.0230555	FR	84.44	Muscle	No
SRX3174445	19718878	Paired	0.978451	0.0220774	FR	85.24	Muscle	No
SRX3174446	17747461	Paired	0.968415	0.0321091	FR	75.76	Muscle	No
SRX3174447	18878789	Paired	0.969542	0.0310282	FR	81.02	Muscle	No
ERX2987533	28759426	Paired	0.0159122	0.984784	RF	91.67	Liver	No
ERX2987534	19893398	Paired	0.0156601	0.985124	RF	93.28	Liver	No
ERX2987535	22533255	Paired	0.0158305	0.985033	RF	92.89	Liver	No
ERX2987536	17443655	Paired	0.0176256	0.983157	RF	92.89	Liver	No
ERX2987537	24191473	Paired	0.016405	0.984324	RF	92.96	Liver	No
ERX2987538	20323643	Paired	0.0147779	0.986049	RF	93.09	Liver	No
ERX2987539	20703772	Paired	0.0156277	0.985014	RF	92.86	Liver	No
SRX3174450	19410648	Paired	0.0251423	0.9755	RF	87.16	Muscle	No
SRX3174451	22003681	Paired	0.0244411	0.97611	RF	87.15	Muscle	No
SRX3174452	20458098	Paired	0.979894	0.0207015	FR	85.59	Muscle	No
SRX3174453	19088081	Paired	0.977309	0.023517	FR	86.57	Muscle	No
SRX3174454	16577742	Paired	0.978096	0.0228946	FR	86.46	Muscle	No
SRX3174455	19258810	Paired	0.979477	0.0212114	FR	86.41	Muscle	No
SRX3174456	19512483	Paired	0.975423	0.0252902	FR	83.97	Muscle	No
SRX3174457	22873522	Paired	0.97636	0.024313	FR	85.57	Muscle	No
SRX3174458	16998395	Paired	0.0287562	0.971597	RF	88.11	Muscle	No
SRX3174459	17645396	Paired	0.0243376	0.976291	RF	88.78	Muscle	No
SRX9854791	32706158	Paired	0.0212365	0.978864	RF	87.65	PK15	No
SRX9854792	93825291	Paired	0.016479	0.984141	RF	91.08	PK15	No
SRX9854793	88215156	Paired	0.0164051	0.984173	RF	90.59	PK15	No
ERX2987540	22863680	Paired	0.0150621	0.985587	RF	92.69	Liver	No

ERX2987541	17998552	Paired	0.0211649	0.980143	RF	91.45	Liver	No
ERX2987542	19813569	Paired	0.0179878	0.982648	RF	92.08	Liver	No
ERX2987543	30421475	Paired	0.0159891	0.984765	RF	92.3	Liver	No
ERX2987544	26270078	Paired	0.0209787	0.980261	RF	91.45	Liver	No
ERX2987545	25165881	Paired	0.0162081	0.984468	RF	92.17	Liver	No
ERX2987546	24335553	Paired	0.0164511	0.984241	RF	91.64	Liver	No
ERX2987547	22326439	Paired	0.0181462	0.98308	RF	91.9	Liver	No
ERX2987548	22725164	Paired	0.0156094	0.984922	RF	92.46	Liver	No
ERX2987549	27406215	Paired	0.0153248	0.985342	RF	92.72	Liver	No
SRX3174460	23143054	Paired	0.974207	0.0266036	FR	86.71	Muscle	No
SRX3174461	29030389	Paired	0.0304591	0.970829	RF	82.59	Muscle	No
SRX3174462	10678645	Paired	0.0357489	0.964364	RF	72.83	Muscle	No
SRX3174463	45466233	Paired	0.0222962	0.978007	RF	87.76	Muscle	No
SRX3174464	17464503	Paired	0.97162	0.0288434	FR	86.82	Muscle	No
SRX3174465	56121270	Paired	0.973387	0.0270945	FR	84.19	Muscle	No
SRX9818491	17133207	Single	0.0610381	NULL	RF	79.73	Fibroblast	No
SRX9818492	13244813	Single	0.0671428	NULL	RF	80.65	Fibroblast	No
SRX10560455	48755164	Paired	0.0248326	0.975265	RF	88.33	Testis	No
SRX3174467	33612104	Paired	0.975859	0.0245627	FR	86.73	Muscle	No
SRX9818493	14185286	Single	0.0604772	NULL	RF	80.06	Fibroblast	No
SRX10560456	46920466	Paired	0.0256055	0.974354	RF	89	Testis	No
SRX3174468	22774261	Paired	0.0150344	0.98589	RF	80.08	Muscle	No
SRX10560457	49470211	Paired	0.0254293	0.974817	RF	88.97	Testis	No
SRX3174469	12116310	Paired	0.975679	0.0250294	FR	86.85	Muscle	No
SRX9818495	14435709	Single	0.0543089	NULL	RF	80.22	Fibroblast	No
SRX10560458	43234602	Paired	0.0258016	0.974647	RF	90.55	Testis	No
SRX10560459	46078684	Paired	0.0240266	0.976614	RF	89.66	Testis	No
ERX2987550	23276932	Paired	0.0166766	0.984055	RF	92.5	Liver	No
ERX2987551	36545624	Paired	0.0153931	0.985266	RF	92.34	Liver	No
ERX2987552	19883742	Paired	0.0160494	0.984439	RF	92.28	Liver	No
ERX2987553	35732843	Paired	0.0144928	0.986396	RF	92.36	Liver	No
ERX2987554	20003015	Paired	0.0172458	0.983623	RF	92.71	Liver	No
ERX2987555	16901842	Paired	0.0164853	0.984365	RF	93.22	Liver	No
ERX2987556	38252548	Paired	0.0137011	0.987133	RF	93.05	Liver	No
ERX2987557	19288989	Paired	0.0158395	0.985085	RF	92.98	Liver	No
ERX2987558	31375597	Paired	0.0146584	0.986091	RF	92.68	Liver	No
ERX2987559	39313535	Paired	0.01427	0.986587	RF	92.36	Liver	No
SRX3174470	7249990	Paired	0.0342441	0.968136	RF	85.37	Muscle	No
SRX10560460	36217323	Paired	0.0263435	0.974092	RF	90.09	Testis	No
SRX3174472	33386339	Paired	0.0228873	0.977638	RF	86.53	Muscle	No
SRX10560461	48799731	Paired	0.0283926	0.971541	RF	89.37	Testis	No
SRX10560462	41820904	Paired	0.0298025	0.970104	RF	89.19	Testis	No
SRX3174474	27720188	Paired	0.975806	0.0248793	FR	87.35	Muscle	No
SRX10560463	43778226	Paired	0.021844	0.978448	RF	90.02	Testis	No
SRX3174475	27579472	Paired	0.973304	0.0274524	FR	86.27	Muscle	No
SRX10560464	47724048	Paired	0.0256532	0.974714	RF	90.62	Testis	No
SRX3174476	15898120	Paired	0.973915	0.026666	FR	82.88	Muscle	No
SRX10560465	44951226	Paired	0.0241039	0.976454	RF	90.43	Testis	No
SRX3174477	16531674	Paired	0.025464	0.975133	RF	85.01	Muscle	No
SRX10560466	40890683	Paired	0.0296242	0.97083	RF	91.01	Testis	No
SRX3174478	18359349	Paired	0.974491	0.0260429	FR	81.48	Muscle	No
SRX3174479	18203951	Paired	0.022297	0.978181	RF	83.95	Muscle	No
ERX2987560	13731294	Paired	0.0172306	0.983412	RF	92.81	Liver	No
ERX2987561	44997188	Paired	0.0137036	0.987116	RF	92.82	Liver	No
ERX2987562	39203774	Paired	0.0146672	0.985964	RF	92.52	Liver	No
ERX2987563	17324741	Paired	0.0181619	0.982618	RF	92.7	Liver	No
ERX2987564	16277121	Paired	0.0164901	0.984109	RF	92.91	Liver	No
ERX2987565	25966423	Paired	0.0156935	0.985588	RF	92.01	Liver	No

ERX2987566	17485511	Paired	0.017348	0.983221	RF	92.13	Liver	No
ERX2987567	32533930	Paired	0.0193232	0.982257	RF	91.79	Liver	No
ERX2987568	20940605	Paired	0.0167659	0.984157	RF	90.53	Liver	No
ERX2987569	22816871	Paired	0.0180713	0.982763	RF	89.65	Liver	No
SRX3174480	19441030	Paired	0.978144	0.0226861	FR	85.98	Muscle	No
SRX3174481	22348292	Paired	0.0203232	0.980207	RF	86.49	Muscle	No
SRX3174482	23047897	Paired	0.97509	0.0255695	FR	85.04	Muscle	No
SRX3174483	18051353	Paired	0.979336	0.0212148	FR	86.24	Muscle	No
SRX3174484	29146151	Paired	0.978059	0.0226371	FR	87.03	Muscle	No
SRX3174485	46402754	Paired	0.979491	0.0211255	FR	85.01	Muscle	No
SRX3174486	17978410	Paired	0.0242144	0.976314	RF	86.66	Muscle	No
SRX3174488	28227418	Paired	0.0264944	0.973941	RF	87.77	Muscle	No
SRX3174489	21671079	Paired	0.972686	0.0280905	FR	81.21	Muscle	No
SRX2826808	34518051	Paired	0.027682	0.971269	RF	87.13	Embryo	No
SRX2826809	40800358	Paired	0.024418	0.975484	RF	85.37	Placenta	No
ERX4193400	53070286	Paired	0.0390017	0.956205	RF	88.1	Small intestine	No
ERX4193402	41503270	Paired	0.0324438	0.965697	RF	87.89	Large intestine	No
ERX1184208	31215902	Paired	0.0283333	0.97242	RF	93.07	Brain	No
ERX4193408	61013083	Paired	0.0343003	0.963304	RF	86.93	Large intestine	No
ERX1184209	30345818	Paired	0.030461	0.970244	RF	93.18	Brain	No
ERX2987570	23004002	Paired	0.0167455	0.983735	RF	91.76	Liver	No
ERX2987571	17725676	Paired	0.0171181	0.983344	RF	92.67	Liver	No
ERX2987572	24094787	Paired	0.0161738	0.984379	RF	92.55	Liver	No
ERX2987573	29172291	Paired	0.0180286	0.983174	RF	92	Liver	No
ERX2987574	21702708	Paired	0.0160817	0.984804	RF	92.32	Liver	No
ERX2987575	18349817	Paired	0.0170166	0.983629	RF	92.76	Liver	No
ERX2987576	21068718	Paired	0.0169122	0.983828	RF	92.16	Liver	No
ERX2987577	29586504	Paired	0.0149516	0.985802	RF	93.19	Liver	No
ERX2987578	18737563	Paired	0.0187792	0.981957	RF	92.47	Liver	No
ERX2987579	30675303	Paired	0.0150928	0.985758	RF	92.69	Liver	No
SRX3174491	23179903	Paired	0.985897	0.0149992	FR	78.6	Muscle	No
SRX3174493	22296773	Paired	0.973349	0.0273256	FR	84	Muscle	No
SRX3174494	18960364	Paired	0.0302378	0.969957	RF	88.73	Muscle	No
SRX3174495	22666243	Paired	0.0282138	0.972344	RF	88.58	Muscle	No
SRX3174497	10257604	Paired	0.0294841	0.970977	RF	84.38	Muscle	No
SRX3174498	20938200	Paired	0.0234804	0.977107	RF	87.58	Muscle	No
SRX3174499	27759101	Paired	0.977517	0.0233262	FR	87.02	Muscle	No
SRX2826811	48184058	Paired	0.0252193	0.974449	RF	85.4	Embryo	No
SRX2826812	55219526	Paired	0.0258188	0.974009	RF	86.69	Embryo	No
SRX2826813	41847469	Paired	0.0245495	0.97551	RF	85.55	Placenta	No
SRX2826814	34934402	Paired	0.025312	0.97449	RF	85.57	Embryo	No
SRX2826815	58435761	Paired	0.0301625	0.969524	RF	85.3	Placenta	No
SRX2826816	38645867	Paired	0.0275659	0.972399	RF	86.86	Embryo	No
SRX2826817	45795485	Paired	0.0258194	0.974479	RF	86.62	Placenta	No
SRX2826818	35115302	Paired	0.0263455	0.972825	RF	87.79	Embryo	No
SRX2826819	57794364	Paired	0.0260943	0.9736	RF	86.33	Placenta	No
SRX8978707	43891530	Single	0.0240826	NULL	RF	91.56	Liver	No
SRX8978708	36607653	Single	0.0276899	NULL	RF	90.54	Liver	No
SRX8978709	41491917	Single	0.0231203	NULL	RF	91.18	Liver	No
ERX2787720	14144218	Paired	0.0160412	0.985863	RF	92.39	Muscle	No
ERX2787721	10834228	Paired	0.0170683	0.985365	RF	91.04	Muscle	No
ERX1184210	34422710	Paired	0.0288098	0.971916	RF	92.69	Brain	No
ERX2787722	18223676	Paired	0.0167005	0.984988	RF	90.7	Muscle	No
ERX1184211	37765521	Paired	0.0294124	0.971117	RF	93.19	Brain	No
ERX2787723	18733857	Paired	0.0166304	0.985271	RF	91.13	Muscle	No
ERX1184212	26026006	Paired	0.041917	0.959058	RF	88.68	Brain	No
ERX2787724	11442417	Paired	0.0173962	0.984253	RF	89.92	Muscle	No
ERX1184213	26029225	Paired	0.0307507	0.969928	RF	92.87	Brain	No

ERX2787725	11775444	Paired	0.017466	0.984234	RF	90.23	Muscle	No
ERX1184214	31938453	Paired	0.0309999	0.96996	RF	90.95	Brain	No
ERX2787726	11434630	Paired	0.0179251	0.983477	RF	90.53	Muscle	No
ERX1184215	31912069	Paired	0.0303507	0.970605	RF	91.94	Brain	No
ERX2787727	12659718	Paired	0.0165772	0.985393	RF	90.96	Muscle	No
ERX1184216	29838721	Paired	0.0283847	0.97226	RF	91.34	Brain	No
ERX2787728	14845048	Paired	0.0178201	0.984027	RF	91.02	Muscle	No
ERX2787729	12095804	Paired	0.0172047	0.984761	RF	90.44	Muscle	No
ERX2987580	20493897	Paired	0.0157766	0.984944	RF	92.31	Liver	No
SRX1295810	39788420	Paired	0.0457057	0.949392	RF	89.11	Ovary	No
SRX1295811	40407157	Paired	0.0529544	0.938012	RF	89.19	Ovary	No
SRX1295812	43460951	Paired	0.0681583	0.92361	RF	89.3	Ovary	No
SRX1295813	38190075	Paired	0.047143	0.947025	RF	88.36	Ovary	No
SRX1295814	40385930	Paired	0.945951	0.0478165	FR	89.03	Ovary	No
SRX1295815	40735105	Paired	0.0541477	0.939739	RF	88.78	Ovary	No
SRX2826820	39614825	Paired	0.026037	0.973688	RF	86.79	Embryo	No
SRX2826821	47406553	Paired	0.0224543	0.976806	RF	85.19	Placenta	No
SRX2826822	35118918	Paired	0.0251575	0.974866	RF	86.21	Placenta	No
SRX8978710	43506773	Single	0.0282817	NULL	RF	91.04	Liver	No
SRX8978711	40520279	Single	0.0262857	NULL	RF	90.19	Liver	No
SRX8978712	34487663	Single	0.0273905	NULL	RF	90.68	Liver	No
SRX8978713	31818229	Single	0.0243499	NULL	RF	91.14	Liver	No
SRX8978714	37050234	Single	0.0241738	NULL	RF	91.53	Liver	No
SRX8978715	32922754	Single	0.0244823	NULL	RF	92.38	Liver	No
SRX8978716	33851914	Single	0.0280063	NULL	RF	90.33	Liver	No
SRX8978717	31958722	Single	0.0224875	NULL	RF	91.16	Liver	No
SRX8978718	29305968	Single	0.0242846	NULL	RF	91.79	Liver	No
SRX8978719	40879466	Single	0.024251	NULL	RF	90.65	Liver	No
ERX2787730	15282412	Paired	0.0184181	0.98393	RF	91.12	Muscle	No
ERX2787731	15804198	Paired	0.0174711	0.984318	RF	91.15	Muscle	No
ERX2787732	16538467	Paired	0.0193843	0.982235	RF	90.48	Muscle	No
ERX2787733	18574025	Paired	0.0186928	0.983312	RF	90.15	Muscle	No
ERX2787734	14663001	Paired	0.0175596	0.98525	RF	90.87	Muscle	No
ERX2787735	10645556	Paired	0.0171904	0.984782	RF	89.97	Muscle	No
ERX2787736	14360999	Paired	0.0187348	0.983514	RF	92.03	Muscle	No
ERX2787737	16390808	Paired	0.0163186	0.985826	RF	91.32	Muscle	No
ERX2787738	12227090	Paired	0.0213285	0.980289	RF	90.96	Muscle	No
ERX2787739	14888603	Paired	0.0163583	0.985418	RF	90.83	Muscle	No
SRX8978720	37587188	Single	0.0224649	NULL	RF	91.61	Liver	No
SRX8978721	29567966	Single	0.0245702	NULL	RF	91.73	Liver	No
SRX8978722	38615686	Single	0.0225648	NULL	RF	91.61	Liver	No
SRX8978723	39324685	Single	0.0259191	NULL	RF	90.88	Liver	No
SRX8978724	31767363	Single	0.0244408	NULL	RF	92.16	Liver	No
SRX8978725	28790953	Single	0.0241478	NULL	RF	92.58	Liver	No
SRX8978726	31454819	Single	0.0236028	NULL	RF	92.46	Liver	No
SRX8978727	32420983	Single	0.0229671	NULL	RF	92.25	Liver	No
SRX8978728	24838204	Single	0.0231364	NULL	RF	92.03	Liver	No
SRX8978729	29623863	Single	0.0222002	NULL	RF	91.93	Liver	No
SRX10347948	25444341	Single	0.0285073	NULL	RF	91.41	Skin	No
SRX10347949	28678123	Single	0.0289846	NULL	RF	90.6	Skin	No
ERX2787740	13269935	Paired	0.0208451	0.981062	RF	90.68	Muscle	No
ERX2787741	16071971	Paired	0.0175481	0.984433	RF	90.56	Muscle	No
ERX2787742	10965560	Paired	0.0173737	0.98451	RF	90.6	Muscle	No
ERX2787743	16134878	Paired	0.016552	0.986136	RF	91.29	Muscle	No
ERX2787744	13386053	Paired	0.0178815	0.984765	RF	91.81	Muscle	No
ERX2787745	15999943	Paired	0.0179873	0.984244	RF	90.47	Muscle	No
ERX2787746	15661661	Paired	0.0190851	0.982728	RF	90.95	Muscle	No
ERX2787747	14125971	Paired	0.0191004	0.983074	RF	91.89	Muscle	No

ERX2787748	10498900	Paired	0.0182184	0.983893	RF	91.16	Muscle	No
ERX2787749	14095081	Paired	0.0177411	0.984727	RF	91.08	Muscle	No
SRX7692528	28968054	Single	0.0314559	NULL	RF	89.76	Pancreas	No
SRX7692529	28713474	Single	0.0304206	NULL	RF	91.57	Pancreas	No
SRX8978730	37509369	Single	0.0234087	NULL	RF	92.21	Liver	No
SRX8978731	34610903	Single	0.0241123	NULL	RF	92.5	Liver	No
SRX8978732	27746017	Single	0.0250916	NULL	RF	91.85	Liver	No
SRX10347950	34531967	Single	0.0273768	NULL	RF	91.65	Skin	No
SRX8978733	45639822	Single	0.0234145	NULL	RF	92.59	Liver	No
SRX10347951	32704311	Single	0.0205983	NULL	RF	92.74	Skin	No
SRX8978734	43436747	Single	0.022301	NULL	RF	92.08	Liver	No
SRX10347952	28631270	Single	0.0267423	NULL	RF	93.11	Skin	No
SRX8978735	37641745	Single	0.0220293	NULL	RF	92.19	Liver	No
SRX10347953	30319910	Single	0.0301856	NULL	RF	91.53	Skin	No
SRX8978736	37650842	Single	0.0268695	NULL	RF	90.59	Liver	No
SRX10347954	21726724	Single	0.0297444	NULL	RF	92.78	Skin	No
SRX10347955	24480741	Single	0.0234423	NULL	RF	92.48	Skin	No
SRX10347956	30319066	Single	0.0228806	NULL	RF	93.44	Skin	No
SRX10347957	26962262	Single	0.029836	NULL	RF	93.04	Skin	No
SRX10347958	35108781	Single	0.0240634	NULL	RF	91.74	Skin	No
SRX10347959	20815795	Single	0.0252403	NULL	RF	92.76	Skin	No
ERX2787750	11197632	Paired	0.0188267	0.983047	RF	90.25	Muscle	No
ERX2787751	11610402	Paired	0.0174426	0.984602	RF	91.25	Muscle	No
ERX2787752	12086927	Paired	0.0186973	0.983066	RF	91.46	Muscle	No
ERX2787753	13493852	Paired	0.0177219	0.985129	RF	91.39	Muscle	No
ERX2787754	15978124	Paired	0.0180505	0.984234	RF	91.01	Muscle	No
ERX2787755	16250562	Paired	0.0179511	0.984028	RF	91.48	Muscle	No
ERX2787756	15891351	Paired	0.0178406	0.984444	RF	90.84	Muscle	No
ERX2787757	10432299	Paired	0.0172327	0.985153	RF	90.15	Muscle	No
ERX2787758	14365191	Paired	0.0181922	0.983764	RF	91.62	Muscle	No
ERX2787759	16349391	Paired	0.0174993	0.984333	RF	91.86	Muscle	No
SRX7692530	30231292	Single	0.0338164	NULL	RF	91.18	Pancreas	No
SRX7692531	31653194	Single	0.0285434	NULL	RF	91.15	Pancreas	No
SRX3293200	23153834	Paired	0.0277392	0.970506	RF	88.64	Ovary	No
SRX7692532	27124373	Single	0.0283113	NULL	RF	92.91	Pancreas	No
SRX3293201	11017557	Paired	0.0294163	0.966515	RF	77.83	Ovary	No
SRX7692533	26717122	Single	0.0294065	NULL	RF	92.78	Pancreas	No
SRX3293202	27946141	Paired	0.0252802	0.973879	RF	89.97	Ovary	No
SRX7692534	4742122	Single	0.0445475	NULL	RF	90.78	Pancreas	No
SRX3293203	12023895	Paired	0.0288421	0.959096	RF	75.41	Ovary	No
SRX7692535	26530705	Single	0.032737	NULL	RF	91.14	Pancreas	No
SRX3293204	25222291	Paired	0.0272492	0.970217	RF	88.86	Ovary	No
SRX7692536	4194763	Single	0.0422619	NULL	RF	92.91	Pancreas	No
SRX3293205	4142625	Paired	0.0383969	0.803508	RF	60.74	Ovary	No
SRX7692537	23021116	Single	0.0294114	NULL	RF	93.21	Pancreas	No
SRX3293206	17880550	Paired	0.0272909	0.968281	RF	87.91	Ovary	No
SRX7692538	3905268	Single	0.0463917	NULL	RF	92.17	Pancreas	No
SRX3293207	10116023	Paired	0.0336224	0.948335	RF	75.53	Ovary	No
SRX7692539	25932388	Single	0.0318441	NULL	RF	92.64	Pancreas	No
SRX3293208	21752694	Paired	0.0301397	0.960041	RF	86.75	Ovary	No
SRX3293209	9168433	Paired	0.0292223	0.95716	RF	75.49	Ovary	No
SRX9176715	33278543	Paired	0.0199533	0.980789	RF	90.65	Adipose	No
SRX9176716	41504609	Paired	0.0173234	0.983505	RF	91.95	Adipose	No
SRX10347960	30703334	Single	0.0224833	NULL	RF	93.17	Skin	No
SRX10347961	30262153	Single	0.0251272	NULL	RF	92.78	Skin	No
SRX10347962	27099243	Single	0.0258926	NULL	RF	92.83	Skin	No
SRX10347963	26115431	Single	0.0266401	NULL	RF	92.61	Skin	No
SRX10347964	27139936	Single	0.0250037	NULL	RF	92.42	Skin	No

SRX10347965	18431292	Single	0.0250068	NULL	RF	93.3	Skin	No
SRX10347966	31776716	Single	0.0220879	NULL	RF	93.48	Skin	No
SRX10347967	28152569	Single	0.0214219	NULL	RF	92.56	Skin	No
SRX10347968	34274118	Single	0.0283942	NULL	RF	87.51	Skin	No
SRX10347969	29068718	Single	0.0257475	NULL	RF	92.4	Skin	No
ERX2787760	12871212	Paired	0.0186854	0.983031	RF	91.3	Muscle	No
ERX2787761	13170098	Paired	0.0195939	0.982345	RF	91.25	Muscle	No
ERX2787762	9587674	Paired	0.0213362	0.981016	RF	89.89	Muscle	No
SRX2163143	37750271	Paired	0.025647	0.973609	RF	82.02	Muscle	No
ERX2787763	14899335	Paired	0.0173738	0.985302	RF	91.29	Muscle	No
SRX2163144	37941148	Paired	0.02221	0.977256	RF	81.35	Muscle	No
ERX2787764	10872428	Paired	0.018753	0.983242	RF	90.6	Muscle	No
ERX2787765	17197303	Paired	0.01695	0.985216	RF	91.11	Muscle	No
ERX2787766	12119954	Paired	0.0183496	0.983518	RF	90.07	Muscle	No
ERX2787767	12941317	Paired	0.0174816	0.985169	RF	93.05	Muscle	No
SRX7692540	22299125	Single	0.0318237	NULL	RF	92.33	Pancreas	No
SRX3293210	20641407	Paired	0.0280184	0.971599	RF	89.17	Ovary	No
SRX7692541	3384796	Single	0.0482734	NULL	RF	91.97	Pancreas	No
SRX7692542	26332310	Single	0.0304225	NULL	RF	92.5	Pancreas	No
SRX7692543	27974940	Single	0.031059	NULL	RF	91.95	Pancreas	No
SRX7692544	27935409	Single	0.0289203	NULL	RF	92.37	Pancreas	No
SRX7692545	25726062	Single	0.0294992	NULL	RF	91.82	Pancreas	No
SRX7692546	3942575	Single	0.0439315	NULL	RF	91.66	Pancreas	No
SRX9176721	41045636	Paired	0.0238168	0.976579	RF	91.77	Brain	No
SRX7692547	26773164	Single	0.0322681	NULL	RF	92.65	Pancreas	No
SRX9176722	51225613	Paired	0.0243508	0.975927	RF	91.58	Brain	No
SRX7692548	24218264	Single	0.0302658	NULL	RF	92.27	Pancreas	No
SRX7692549	3707799	Single	0.0454156	NULL	RF	92.06	Pancreas	No
SRX9176727	35995035	Paired	0.0208547	0.979753	RF	90.4	Brain	No
SRX9176728	40635090	Paired	0.0235643	0.976977	RF	88.06	Brain	No
SRX10347970	33531853	Single	0.0259248	NULL	RF	90.95	Skin	No
SRX10347971	27065973	Single	0.0247983	NULL	RF	93.02	Skin	No
SRX10347972	29642199	Single	0.0239993	NULL	RF	92.75	Skin	No
SRX10347973	31080515	Single	0.0246259	NULL	RF	92.8	Skin	No
SRX10347974	18912250	Single	0.0256878	NULL	RF	93.33	Skin	No
SRX10347975	20333182	Single	0.0284683	NULL	RF	91.46	Skin	No
SRX10347976	30901783	Single	0.0234635	NULL	RF	93.08	Skin	No
SRX10347977	30245564	Single	0.0248504	NULL	RF	92.58	Skin	No
SRX10347979	25465047	Single	0.0277856	NULL	RF	93.53	Skin	No
ERX2244923	48777836	Paired	0.040946	0.959432	RF	60.98	Muscle	No
ERX2244924	46527379	Paired	0.0294112	0.970189	RF	63.34	Muscle	No
ERX2244925	47435465	Paired	0.0277107	0.971728	RF	64.62	Muscle	No
ERX2244926	52211949	Paired	0.0286153	0.970923	RF	64.66	Muscle	No
ERX2244928	44734880	Paired	0.0238537	0.975726	RF	70.74	Muscle	No
ERX2244929	53776406	Paired	0.0401666	0.960222	RF	62.83	Muscle	No
SRX7692550	28064340	Single	0.0297425	NULL	RF	92.72	Pancreas	No
SRX7692551	27260798	Single	0.0296401	NULL	RF	92.95	Pancreas	No
SRX7692552	26954194	Single	0.0285209	NULL	RF	92.38	Pancreas	No
SRX7692553	25039733	Single	0.0302106	NULL	RF	92.78	Pancreas	No
SRX7692554	26137355	Single	0.0311388	NULL	RF	92.14	Pancreas	No
SRX7692555	26190995	Single	0.02873	NULL	RF	92.78	Pancreas	No
SRX7692556	24920641	Single	0.0290149	NULL	RF	92.6	Pancreas	No
SRX9176731	41799419	Paired	0.0231487	0.97739	RF	90.88	Brain	No
SRX7692557	26064136	Single	0.0293274	NULL	RF	91.92	Pancreas	No
SRX9176732	38354729	Paired	0.0193908	0.981398	RF	90.8	Brain	No
SRX9176737	36345171	Paired	0.0242329	0.978253	RF	89.41	Liver	No
SRX9176738	27131143	Paired	0.0290271	0.973711	RF	88.76	Liver	No
SRX10347980	24126498	Single	0.0264538	NULL	RF	89.21	Skin	No

SRX10347981	30278707	Single	0.0195876	NULL	RF	93.6	Skin	No
SRX10347982	25368749	Single	0.0287399	NULL	RF	89.64	Skin	No
SRX10347983	29127983	Single	0.0297225	NULL	RF	91.64	Skin	No
SRX10347984	29670965	Single	0.0256004	NULL	RF	91.81	Skin	No
ERX1049364	29120800	Paired	0.0217377	0.980065	RF	84.98	Spleen	No
SRX10347985	34115685	Single	0.0228917	NULL	RF	91.54	Skin	No
ERX1049365	36953933	Paired	0.0140798	0.987244	RF	92.72	Liver	No
SRX10347986	27657456	Single	0.0248933	NULL	RF	92.21	Skin	No
ERX1049366	35173079	Paired	0.0163116	0.986182	RF	94.06	Muscle	No
SRX10347987	33301585	Single	0.022598	NULL	RF	92.69	Skin	No
SRX10347988	29115156	Single	0.0259694	NULL	RF	92.9	Skin	No
SRX10347989	30908856	Single	0.0286497	NULL	RF	91.83	Skin	No
SRX6976108	15164303	Paired	0.0197724	0.981012	RF	93	Embryo	No
SRX6976109	13689186	Paired	0.0199442	0.980944	RF	92.92	Embryo	No
ERX1403286	45875177	Paired	0.0267545	0.972664	RF	63.05	Liver	No
ERX2244930	54645963	Paired	0.0363688	0.964091	RF	63.61	Muscle	No
ERX2244934	50837694	Paired	0.0247124	0.975751	RF	65.29	Muscle	No
ERX2244937	42933822	Paired	0.0235564	0.976463	RF	67.67	Muscle	No
SRX9176743	59030061	Paired	0.024004	0.976394	RF	91.69	Lung	No
SRX9176744	62737119	Paired	0.0212394	0.979202	RF	91.73	Lung	No
SRX10347990	27480774	Single	0.026131	NULL	RF	94.02	Skin	No
SRX9176749	55152290	Paired	0.0137007	0.987339	RF	89.95	Muscle	No
SRX10347991	26426044	Single	0.0258743	NULL	RF	91.65	Skin	No
SRX10347992	31034663	Single	0.0238233	NULL	RF	93.34	Skin	No
SRX10347993	28526146	Single	0.0233582	NULL	RF	92.61	Skin	No
SRX10347994	28084628	Single	0.0274859	NULL	RF	92.5	Skin	No
SRX10347995	26158548	Single	0.0242575	NULL	RF	93.13	Skin	No
SRX10347996	27679942	Single	0.025651	NULL	RF	93.06	Skin	No
SRX10347997	25380096	Single	0.0250914	NULL	RF	92.38	Skin	No
SRX10347998	28751656	Single	0.0287105	NULL	RF	91.69	Skin	No
SRX10347999	33530145	Single	0.025589	NULL	RF	92.79	Skin	No
SRX6976117	13744791	Paired	0.0211783	0.979772	RF	93.61	Embryo	No
SRX6976118	13516149	Paired	0.0207163	0.980235	RF	93.36	Embryo	No
SRX6976119	13414749	Paired	0.0236855	0.976965	RF	92.29	Embryo	No
SRX3367382	56437832	Paired	0.0291835	0.970471	RF	89.12	Brain	No
SRX3367383	39840094	Paired	0.0357349	0.964069	RF	88.57	Heart	No
SRX3367384	46044939	Paired	0.0289093	0.97178	RF	90.63	Kidney	No
SRX3367385	46905535	Paired	0.0273165	0.972741	RF	89.05	Liver	No
SRX3367386	78381249	Paired	0.0143885	0.986776	RF	89.24	Muscle	No
SRX5888894	56429973	Paired	0.0412257	0.957622	RF	89.79	Lung	No
SRX5888895	63365529	Paired	0.0241677	0.974638	RF	79.93	Lung	No
SRX5888896	54297694	Paired	0.0270526	0.972201	RF	85.89	Lung	No
SRX5888897	56521567	Paired	0.0263298	0.972625	RF	78.91	Lung	No
ERX1534678	33278543	Paired	0.0199743	0.980798	RF	90.65	Adipose	No
SRX5888898	62497031	Paired	0.0277812	0.971381	RF	75.3	Lung	No
ERX1534679	41504609	Paired	0.017293	0.983485	RF	91.95	Adipose	No
SRX5888899	65973821	Paired	0.0386805	0.959084	RF	81.71	Lung	No
SRX4634906	21390047	Paired	0.0755874	0.922209	RF	80.62	Small intestine	No
SRX4634907	24785833	Paired	0.101136	0.900064	RF	77.4	Small intestine	No
SRX4634908	20126234	Paired	0.0788382	0.918473	RF	82.15	Small intestine	No
SRX4634909	24678960	Paired	0.149344	0.858453	RF	74.01	Small intestine	No
SRX8484394	34301410	Paired	0.185381	0.811591	RF	74.62	Muscle	No
SRX8484395	41487377	Paired	0.0304026	0.970642	RF	85.1	Muscle	No
SRX8484396	42858662	Paired	0.177061	0.819482	RF	81.11	Muscle	No
SRX8484397	54623256	Paired	0.0343646	0.966124	RF	87.59	Muscle	No
SRX8484398	51484203	Paired	0.0219273	0.980812	RF	85.36	Muscle	No
SRX9176750	39565401	Paired	0.0156741	0.985121	RF	88.81	Muscle	No
ERX2262879	25241599	Single	0.0224968	NULL	RF	93.86	Early embryo	No

SRX9176755	47521205	Paired	0.0262023	0.974809	RF	90.01	Spleen	No
SRX9176756	30622879	Paired	0.0251969	0.975556	RF	90.32	Spleen	No
SRX6976120	15365479	Paired	0.0218723	0.978984	RF	92.27	Embryo	No
SRX6976121	14145663	Paired	0.0201152	0.980777	RF	92.95	Embryo	No
SRX6976122	13767477	Paired	0.0220001	0.978879	RF	90.15	Embryo	No
SRX6976123	12938298	Paired	0.0224671	0.978358	RF	92.07	Embryo	No
SRX6976124	15006718	Paired	0.0198675	0.980844	RF	91.86	Embryo	No
SRX6976125	12419373	Paired	0.0221429	0.97862	RF	90.58	Embryo	No
SRX6976126	12664483	Paired	0.0231173	0.977549	RF	92.58	Embryo	No
ERX1534680	41045636	Paired	0.0238217	0.976599	RF	91.77	Brain	No
ERX1534681	51225613	Paired	0.0243618	0.975954	RF	91.58	Brain	No
SRX4254852	55885952	Paired	0.957357	0.043341	FR	83.52	Small intestine	No
ERX1534682	35995035	Paired	0.0208689	0.979777	RF	90.4	Brain	No
SRX4254853	61850440	Paired	0.0471532	0.95366	RF	82.74	Small intestine	No
ERX1534683	40635090	Paired	0.0235333	0.976979	RF	88.06	Brain	No
SRX4254854	59192826	Paired	0.895654	0.103719	FR	78.43	Small intestine	No
ERX1534684	41799419	Paired	0.0231426	0.977374	RF	90.88	Brain	No
ERX1534685	38354729	Paired	0.0194059	0.981398	RF	90.8	Brain	No
ERX1534686	36345171	Paired	0.0242187	0.978265	RF	89.41	Liver	No
ERX1534687	27131143	Paired	0.029042	0.973776	RF	88.76	Liver	No
ERX1534688	59030061	Paired	0.0239922	0.976375	RF	91.69	Lung	No
ERX1534689	62737119	Paired	0.0212304	0.979205	RF	91.73	Lung	No
SRX4634910	22196720	Paired	0.155874	0.854687	RF	71.52	Small intestine	No
SRX4634911	28129782	Paired	0.0825896	0.916011	RF	80.94	Small intestine	No
SRX4634912	21199821	Paired	0.0837586	0.917031	RF	84.52	Small intestine	No
SRX4634913	24515826	Paired	0.136798	0.864038	RF	74.05	Small intestine	No
SRX4634915	21890068	Paired	0.119582	0.885363	RF	76.26	Small intestine	No
SRX4634917	22044189	Paired	0.103119	0.898542	RF	80.97	Small intestine	No
SRX4634918	21478951	Paired	0.122977	0.888479	RF	74.97	Small intestine	No
SRX4634919	24116458	Paired	0.111998	0.891037	RF	76.01	Small intestine	No
ERX2262880	21806771	Single	0.0294029	NULL	RF	93	Early embryo	No
ERX2262881	24109302	Single	0.0271332	NULL	RF	91.57	Early embryo	No
ERX2262882	23998971	Single	0.0216009	NULL	RF	93.72	Early embryo	No
ERX2262883	32761292	Single	0.024438	NULL	RF	94.78	Early embryo	No
ERX2262884	28478298	Single	0.0245689	NULL	RF	94.62	Early embryo	No
ERX2262885	22540516	Single	0.0274288	NULL	RF	93.91	Early embryo	No
ERX2262886	24364312	Single	0.0213688	NULL	RF	94.61	Early embryo	No
ERX2262887	26066748	Single	0.0201715	NULL	RF	94.57	Early embryo	No
ERX2262888	24058610	Single	0.0242139	NULL	RF	94.55	Early embryo	No
ERX2262889	29268941	Single	0.0244615	NULL	RF	94.57	Early embryo	No
SRX6976131	14479211	Paired	0.0224909	0.97837	RF	93.12	Embryo	No
SRX6976137	14646201	Paired	0.0192598	0.981788	RF	90.62	Embryo	No
SRX6976138	14508785	Paired	0.0213059	0.979465	RF	91.69	Embryo	No
SRX6976139	14563410	Paired	0.0210738	0.979814	RF	93.01	Embryo	No
SRX2783296	10088450	Single	0.0256908	NULL	RF	94.3	Fibroblast	No
SRX2783297	9799173	Single	0.0235139	NULL	RF	94.49	Fibroblast	No
SRX2783298	9267733	Single	0.0245678	NULL	RF	93.86	Fibroblast	No
SRX2783299	10338062	Single	0.024518	NULL	RF	94.07	Fibroblast	No
ERX1534690	55152290	Paired	0.0136848	0.987332	RF	89.95	Muscle	No
ERX1534691	39565401	Paired	0.0156813	0.985093	RF	88.81	Muscle	No
ERX1534692	47521205	Paired	0.026192	0.974768	RF	90.01	Spleen	No
ERX1534693	30622879	Paired	0.0251849	0.975512	RF	90.32	Spleen	No
SRX4634920	21347191	Paired	0.180764	0.831823	RF	71.28	Small intestine	No
SRX4634921	20642408	Paired	0.172064	0.834754	RF	72.03	Small intestine	No
SRX4634923	21434373	Paired	0.152996	0.849835	RF	73.46	Small intestine	No
SRX4634924	27216775	Paired	0.149143	0.864145	RF	73.37	Small intestine	No
ERX2262890	25072300	Single	0.0221529	NULL	RF	94.17	Early embryo	No
ERX2262891	33847330	Single	0.0248217	NULL	RF	94.43	Early embryo	No

ERX2262892	20972764	Single	0.0278958	NULL	RF	92.21	Early embryo	No
SRX4994055	40198634	Paired	0.0199012	0.979243	RF	90.26	Skin	No
ERX2262893	24774347	Single	0.0235311	NULL	RF	94.07	Early embryo	No
SRX4994056	39117214	Paired	0.0858885	0.912458	RF	84.7	Skin	No
ERX2262894	27755351	Single	0.0233117	NULL	RF	93.88	Early embryo	No
SRX4994057	41438646	Paired	0.0910113	0.909109	RF	83.39	Skin	No
ERX2262895	30730010	Single	0.0217142	NULL	RF	94.14	Early embryo	No
SRX4994058	40333134	Paired	0.0692907	0.9306	RF	84.73	Skin	No
ERX2262896	26884949	Single	0.0232465	NULL	RF	94.55	Early embryo	No
ERX2262897	25161007	Single	0.0235564	NULL	RF	94.71	Early embryo	No
ERX2262898	27732470	Single	0.023763	NULL	RF	93.49	Early embryo	No
ERX2262899	24466712	Single	0.0211799	NULL	RF	95.1	Early embryo	No
SRX6976140	13201350	Paired	0.0225562	0.978142	RF	92.07	Embryo	No
SRX6976141	12744843	Paired	0.0240522	0.976788	RF	91.63	Embryo	No
SRX9352639	40394693	Paired	0.036379	0.956701	RF	84.6	Macrophage	No
SRX4994061	48711332	Paired	0.0705263	0.929344	RF	85.63	Skin	No
SRX4994062	38570601	Paired	0.0202953	0.978432	RF	90.03	Skin	No
SRX9352640	41352819	Paired	0.0382986	0.954236	RF	81.84	Macrophage	No
SRX9352641	43103550	Paired	0.0347182	0.958849	RF	85.96	Macrophage	No
SRX9352642	37566974	Paired	0.0393987	0.951837	RF	84.18	Macrophage	No
SRX9352643	37998572	Paired	0.0370586	0.954054	RF	82.57	Macrophage	No
SRX9352644	41294816	Paired	0.0357168	0.955329	RF	82.96	Macrophage	No
SRX9352645	39704500	Paired	0.0352966	0.95642	RF	83.13	Macrophage	No
SRX9352646	41331360	Paired	0.0343651	0.958524	RF	84.45	Macrophage	No
SRX9352647	34118484	Paired	0.0360409	0.955326	RF	85.04	Macrophage	No
ERX768400	79482442	Paired	0.018824	0.98272	RF	88.05	Muscle	No
ERX768401	80082418	Paired	0.0193195	0.982373	RF	86.75	Muscle	No
ERX768402	52116299	Paired	0.0191368	0.983252	RF	90.45	Muscle	No
SRX6769769	42005199	Paired	0.0483833	0.950089	RF	84.77	Ovary	No
SRX8573447	20446970	Paired	0.0237151	0.973464	RF	78.16	Milk	No
SRX8573449	14945211	Paired	0.0388773	0.95874	RF	66.48	Milk	No
ERX3441342	104914037	Paired	0.0613552	0.935285	RF	71.74	Testis	No
SRX6769770	24174654	Paired	0.0517439	0.946916	RF	81.77	Ovary	No
SRX6769771	22933813	Paired	0.0362665	0.963246	RF	83.8	Ovary	No
SRX6769772	18672389	Paired	0.0368727	0.962541	RF	84.3	Ovary	No
SRX6769773	21330478	Paired	0.0459316	0.95258	RF	83.68	Ovary	No
SRX6769774	30962570	Paired	0.0451082	0.952629	RF	84.9	Ovary	No
SRX8573450	19070712	Paired	0.0259997	0.970415	RF	65.31	Milk	No
SRX8573451	17016510	Paired	0.0494007	0.942505	RF	67.18	Milk	No
SRX8573453	18536388	Paired	0.0382692	0.959475	RF	72.07	Milk	No
SRX8573454	18748919	Paired	0.0254386	0.969412	RF	76.17	Milk	No
SRX8573455	18216072	Paired	0.043348	0.953539	RF	78.64	Milk	No
SRX8573456	17874144	Paired	0.0444922	0.949742	RF	66.57	Milk	No
SRX8573458	18834165	Paired	0.0335768	0.961806	RF	62.11	Milk	No
SRX7438550	41166663	Paired	0.0281806	0.971448	RF	82.95	Heart	No
ERX3441354	82970105	Paired	0.0796461	0.916761	RF	71.63	Brain	No
ERX3441356	45302062	Paired	0.0444358	0.954136	RF	78.47	Liver	No
SRX8573460	20360278	Paired	0.035606	0.963114	RF	76.71	Milk	No
SRX8573463	19226166	Paired	0.0505997	0.943614	RF	77.7	Milk	No
SRX10578413	29366224	Paired	0.0255917	0.975225	RF	81.52	Adipose	No
SRX10578414	29199924	Paired	0.0220347	0.978279	RF	78.48	Adipose	No
SRX8573466	18936257	Paired	0.0406535	0.960357	RF	81.88	Milk	No
SRX10578415	35203596	Paired	0.0420516	0.958966	RF	85.61	Adipose	No
SRX8573467	20119196	Paired	0.0688896	0.931311	RF	85.61	Milk	No
SRX10578416	32730212	Paired	0.0394449	0.961073	RF	84.65	Adipose	No
SRX8573468	18937880	Paired	0.0572596	0.943349	RF	82.3	Milk	No
SRX4108624	14128949	Paired	0.0201823	0.981174	RF	95.4	Artery	No
SRX10578417	34098870	Paired	0.0171355	0.984245	RF	87.21	Liver	No

SRX8573469	16922930	Paired	0.0385922	0.961222	RF	77.75	Milk	No
SRX4108625	20992834	Paired	0.0189049	0.982477	RF	95.37	Artery	No
SRX10578418	33319291	Paired	0.0173034	0.983647	RF	86.39	Liver	No
SRX4108626	22103642	Paired	0.0186534	0.982761	RF	95.56	Artery	No
SRX10578419	33866597	Paired	0.0212143	0.979956	RF	84.28	Liver	No
SRX4108627	15754860	Paired	0.0180873	0.983303	RF	95.37	Artery	No
SRX4108628	20735888	Paired	0.0173503	0.984199	RF	95.38	Artery	No
SRX4108629	20956663	Paired	0.0171788	0.984421	RF	95.37	Artery	No
SRX7805541	16933478	Paired	0.037183	0.961523	RF	91.22	Macrophage	No
SRX7805542	18248185	Paired	0.0338668	0.96478	RF	90.81	Macrophage	No
SRX7805543	20034407	Paired	0.0336085	0.965642	RF	90.84	Macrophage	No
SRX7805544	22885858	Paired	0.0289524	0.970267	RF	87.69	Macrophage	No
SRX5568600	33145670	Paired	0.0208313	0.979243	RF	81.61	Small intestine	No
SRX7805545	32548512	Paired	0.0314781	0.967603	RF	88.5	Macrophage	No
SRX5568601	35411982	Paired	0.0207313	0.977691	RF	81.78	Small intestine	No
SRX7805546	20844121	Paired	0.0319096	0.966728	RF	87.28	Macrophage	No
SRX5568602	33849585	Paired	0.0198826	0.979916	RF	83.06	Small intestine	No
SRX7805547	22845814	Paired	0.0354428	0.9611	RF	65.13	Macrophage	No
SRX5568603	34624595	Paired	0.0223053	0.976123	RF	79.3	Small intestine	No
SRX7805548	42216266	Paired	0.0332935	0.963586	RF	64.18	Macrophage	No
SRX5568604	34651737	Paired	0.021204	0.97873	RF	79.96	Small intestine	No
SRX7805549	18593930	Paired	0.0356589	0.961468	RF	65.04	Macrophage	No
SRX5568605	34244610	Paired	0.0206649	0.979354	RF	82.18	Small intestine	No
SRX5568607	36573652	Paired	0.021161	0.978766	RF	80.75	Small intestine	No
SRX5568608	34595711	Paired	0.0210142	0.977918	RF	80.62	Small intestine	No
SRX5568609	18939965	Paired	0.0232349	0.977538	RF	84.37	Small intestine	No
ERX3441362	56129943	Paired	0.0416341	0.957241	RF	79.63	Liver	No
SRX7438561	41114644	Paired	0.0217195	0.97796	RF	86.62	Liver	No
SRX10578420	32067938	Paired	0.0323712	0.969074	RF	84.91	Muscle	No
SRX8573472	18074016	Paired	0.0164765	0.983685	RF	82.59	Milk	No
SRX10578421	34172874	Paired	0.0341326	0.967709	RF	86.02	Muscle	No
SRX8573473	16450621	Paired	0.0281999	0.97125	RF	90.34	Milk	No
SRX10578422	32211558	Paired	0.0330531	0.969354	RF	85.74	Muscle	No
SRX8573474	16867215	Paired	0.0418138	0.956888	RF	60.19	Milk	No
SRX4108630	21215581	Paired	0.017435	0.983866	RF	95.36	Artery	No
SRX4108631	21467573	Paired	0.0172477	0.984028	RF	95.27	Artery	No
SRX8573476	22287555	Paired	0.0331337	0.966249	RF	85.66	Milk	No
SRX4108632	22351589	Paired	0.0170696	0.98418	RF	95.54	Artery	No
SRX10578425	27470279	Paired	0.0225376	0.978185	RF	85.13	Adipose	No
SRX8573477	22277288	Paired	0.0357687	0.963569	RF	71.81	Milk	No
SRX4108633	14923020	Paired	0.0217426	0.979707	RF	95.35	Artery	No
SRX8573478	20919437	Paired	0.0220413	0.977432	RF	80.8	Milk	No
SRX4108634	23748044	Paired	0.0204988	0.981161	RF	95.45	Artery	No
SRX8573479	21290452	Paired	0.0395471	0.960435	RF	80.78	Milk	No
SRX4108635	26238953	Paired	0.0205851	0.981039	RF	95.45	Artery	No
SRX7805550	23025423	Paired	0.0347997	0.961676	RF	60.38	Macrophage	No
SRX7805551	20578137	Paired	0.0361085	0.960257	RF	64.71	Macrophage	No
SRX7805552	18468353	Paired	0.0350473	0.962055	RF	60.91	Macrophage	No
SRX5568610	24120218	Paired	0.0199983	0.981776	RF	88.17	Small intestine	No
SRX5568611	19654399	Paired	0.0222344	0.978584	RF	73.29	Small intestine	No
SRX5568612	18483702	Paired	0.0235228	0.97722	RF	81.72	Small intestine	No
SRX5568613	19816055	Paired	0.0217318	0.979018	RF	80.68	Small intestine	No
SRX5568614	21163797	Paired	0.0210426	0.979394	RF	82.27	Small intestine	No
SRX5568615	10069098	Paired	0.0241203	0.975987	RF	83.37	Small intestine	No
SRX5568616	19512758	Paired	0.021742	0.97934	RF	74.9	Small intestine	No
SRX5568617	18935646	Paired	0.0218399	0.979123	RF	79.69	Small intestine	No
SRX5568618	19217412	Paired	0.0215102	0.979572	RF	77.54	Small intestine	No
SRX5568619	32589053	Paired	0.0213378	0.978157	RF	78.33	Small intestine	No

ERX3441373	39416490	Paired	0.0404418	0.95777	RF	83.06	Muscle	No
SRX7438572	38832630	Paired	0.0214628	0.978731	RF	68.79	Liver	No
ERX3441375	53558557	Paired	0.0427494	0.955414	RF	73.1	Liver	No
SRX8573480	19080655	Paired	0.0202231	0.979794	RF	77.99	Milk	No
SRX8573481	19777134	Paired	0.046781	0.950425	RF	71.41	Milk	No
SRX10578430	37349522	Paired	0.0217024	0.980789	RF	82.08	Muscle	No
SRX8573482	26516112	Paired	0.0284005	0.964055	RF	68.96	Milk	No
SRX10578431	40095699	Paired	0.0204308	0.981895	RF	82.74	Muscle	No
SRX8573483	22145244	Paired	0.034835	0.967089	RF	73.23	Milk	No
SRX10578432	36222468	Paired	0.0238424	0.980299	RF	80.55	Muscle	No
SRX8573484	35940137	Paired	0.0435594	0.957288	RF	82.33	Milk	No
SRX10578433	30476823	Paired	0.0287247	0.971916	RF	81.22	Adipose	No
SRX10578434	28539778	Paired	0.025297	0.975298	RF	82.23	Adipose	No
SRX8573486	23879516	Paired	0.0305497	0.970004	RF	79.1	Milk	No
SRX10578435	27386470	Paired	0.0342476	0.966642	RF	81.91	Adipose	No
SRX8573487	20069389	Paired	0.0480333	0.95135	RF	69.79	Milk	No
SRX10578436	29592611	Paired	0.034634	0.966535	RF	83.4	Liver	No
SRX8573488	28807046	Paired	0.044891	0.955155	RF	79.86	Milk	No
SRX10578437	34066722	Paired	0.0413439	0.95964	RF	82.52	Liver	No
SRX10578438	34725896	Paired	0.0361272	0.964199	RF	83.07	Liver	No
SRX10578439	33851101	Paired	0.0393665	0.960759	RF	84.19	Liver	No
SRX7805561	37778715	Paired	0.968264	0.0322556	FR	86.86	Adipose	No
SRX7805562	63326296	Paired	0.967518	0.0331456	FR	85.25	Adipose	No
SRX7805563	47697426	Paired	0.964495	0.0355892	FR	86.75	Adipose	No
SRX5568620	34823545	Paired	0.020403	0.979361	RF	79.85	Small intestine	No
SRX7805564	40301457	Paired	0.971902	0.0291633	FR	87.46	Adipose	No
SRX5568621	20062216	Paired	0.0221121	0.97862	RF	80.55	Small intestine	No
SRX7805565	43383066	Paired	0.955201	0.044287	FR	82.61	Adipose	No
SRX5568622	18097730	Paired	0.0219866	0.978468	RF	82.16	Small intestine	No
SRX7805566	60974354	Paired	0.954203	0.0448488	FR	84.29	Adipose	No
SRX5568623	18380091	Paired	0.0224052	0.978467	RF	79.2	Small intestine	No
SRX7805567	46677023	Paired	0.962323	0.0372883	FR	84.15	Adipose	No
SRX5568624	18906346	Paired	0.0216565	0.979755	RF	72.79	Small intestine	No
SRX7805568	64309493	Paired	0.96139	0.0386932	FR	88.81	Adipose	No
SRX5568625	20003757	Paired	0.0211164	0.979625	RF	78.56	Small intestine	No
SRX5568626	19945024	Paired	0.0225055	0.978114	RF	80.52	Small intestine	No
SRX5568627	34613757	Paired	0.0198742	0.980483	RF	81.51	Small intestine	No
SRX5568628	34631236	Paired	0.0202769	0.979692	RF	81.89	Small intestine	No
SRX7438583	48701171	Paired	0.0230186	0.977623	RF	80.08	Muscle	No
SRX7438589	37423328	Paired	0.0242282	0.976138	RF	83.66	Muscle	No
SRX8573491	24122465	Paired	0.0278247	0.973734	RF	79.86	Milk	No
SRX10578440	26508708	Paired	0.0276128	0.97731	RF	80.42	Muscle	No
SRX8573492	30905306	Paired	0.0339643	0.966904	RF	79.04	Milk	No
SRX10578441	28955765	Paired	0.0263826	0.978196	RF	79.74	Muscle	No
SRX8573493	28560710	Paired	0.0333859	0.969146	RF	79.24	Milk	No
SRX10578442	28509013	Paired	0.025316	0.979416	RF	79.75	Muscle	No
SRX10578443	31730335	Paired	0.040845	0.959534	RF	82.97	Liver	No
SRX10578444	30031099	Paired	0.0334783	0.967832	RF	86.01	Liver	No
SRX10578445	33813208	Paired	0.022592	0.980381	RF	83.6	Muscle	No
SRX8573497	20982572	Paired	0.0335235	0.967081	RF	77.79	Milk	No
SRX10578446	33416914	Paired	0.0289915	0.974771	RF	82.34	Muscle	No
SRX10578447	33759815	Paired	0.0307971	0.970092	RF	85.36	Muscle	No
SRX8573499	25790067	Paired	0.0381209	0.962071	RF	77.19	Milk	No
SRX4058971	21387380	Paired	0.017434	0.985371	RF	90.23	Liver	No
SRX10578448	32708154	Paired	0.0318129	0.968477	RF	82.67	Adipose	No
SRX4058972	22217237	Paired	0.0205655	0.982957	RF	81.85	Liver	No
SRX4058973	22924507	Paired	0.0168889	0.986129	RF	89.95	Liver	No
SRX4058974	19548256	Paired	0.0190667	0.984027	RF	90.64	Liver	No

SRX4058975	21796929	Paired	0.0276498	0.9746	RF	91.33	Liver	No
SRX4058976	21082019	Paired	0.0211762	0.980505	RF	86.93	Liver	No
SRX4058977	20206663	Paired	0.0206853	0.983647	RF	88.87	Liver	No
SRX4058978	23706395	Paired	0.0220885	0.980545	RF	84.31	Liver	No
SRX4058979	24718825	Paired	0.0166092	0.985417	RF	84.87	Liver	No
SRX3974000	14450168	Paired	0.0319025	0.968256	RF	85.69	Testis	No
SRX3974001	18468477	Paired	0.0383234	0.955183	RF	68.76	Testis	No
SRX3974002	19519686	Paired	0.0455626	0.950645	RF	80.37	Testis	No
SRX3974003	17254379	Paired	0.0260138	0.973996	RF	86.9	Testis	No
SRX3974004	8802293	Paired	0.0305869	0.969227	RF	83.61	Testis	No
SRX3974005	17670236	Paired	0.0268289	0.974071	RF	85.3	Testis	No
SRX3974006	17307018	Paired	0.0280525	0.971955	RF	88.73	Testis	No
SRX3974007	17094264	Paired	0.0273289	0.97275	RF	88.72	Testis	No
SRX3974009	15869222	Paired	0.0256858	0.974332	RF	85.19	Testis	No
ERX3441390	106395911	Paired	0.0639914	0.93397	RF	75.69	Testis	No
SRX7438590	38743805	Paired	0.043922	0.959069	RF	85.2	Spleen	No
SRX7438591	39908111	Paired	0.0435441	0.961891	RF	85.32	Spleen	No
SRX7438592	48593369	Paired	0.0321634	0.967678	RF	90.26	Heart	No
SRX7438593	44805772	Paired	0.0329476	0.966874	RF	85.88	Adipose	No
SRX7438594	47245176	Paired	0.0437186	0.955502	RF	83.5	Adipose	No
SRX7438595	41095630	Paired	0.0318925	0.968118	RF	90.02	Heart	No
SRX7438596	38769681	Paired	0.0350697	0.964586	RF	86.89	Heart	No
SRX7438597	38277801	Paired	0.0177863	0.98262	RF	89.15	Liver	No
SRX7438598	45194203	Paired	0.0215459	0.978668	RF	88.13	Liver	No
SRX7438599	47179676	Paired	0.0226511	0.978195	RF	82.67	Muscle	No
SRX4058980	24423194	Paired	0.0222451	0.981915	RF	85.29	Liver	No
SRX4058981	45004950	Paired	0.0219898	0.980993	RF	83.21	Liver	No
SRX4058982	16327938	Paired	0.0213543	0.98133	RF	84.46	Liver	No
SRX4058984	12701863	Paired	0.0289276	0.974899	RF	80.17	Liver	No
SRX4058985	22810312	Paired	0.0186212	0.984601	RF	82.96	Liver	No
SRX4058986	20259857	Paired	0.0175381	0.985242	RF	91.8	Liver	No
SRX4058987	10988863	Paired	0.0215894	0.982097	RF	90.62	Liver	No
SRX4058988	17157104	Paired	0.0184594	0.984213	RF	86.56	Liver	No
SRX4058989	36532593	Paired	0.0183276	0.984601	RF	81.72	Liver	No
SRX3974010	15027411	Paired	0.0274701	0.972427	RF	83.45	Testis	No
SRX3974011	13923055	Paired	0.0271168	0.972841	RF	81.97	Testis	No
SRX3974012	13408226	Paired	0.0287386	0.971432	RF	81.84	Testis	No
SRX3974013	18407473	Paired	0.027539	0.973078	RF	93.03	Testis	No
SRX3974014	16509950	Paired	0.0270624	0.973417	RF	91.86	Testis	No
SRX3974015	18526196	Paired	0.0339898	0.962252	RF	70.97	Testis	No
SRX3974016	19813596	Paired	0.0539051	0.9415	RF	66.44	Testis	No
SRX3974017	18597935	Paired	0.0308756	0.966856	RF	65.67	Testis	No
SRX3974018	22635924	Paired	0.0269318	0.973008	RF	87.2	Testis	No
SRX3974019	15390415	Paired	0.0287265	0.97142	RF	88.06	Testis	No
SRX4581356	44438638	Paired	0.0235745	0.977054	RF	70.84	Muscle	No
SRX4581357	54598242	Paired	0.030483	0.969983	RF	83.07	Muscle	No
SRX4581358	53478883	Paired	0.0278909	0.972062	RF	81.99	Muscle	No
SRX4581359	46871568	Paired	0.0267564	0.973417	RF	79.75	Muscle	No
SRX4058990	26486431	Paired	0.0183215	0.98488	RF	83.15	Liver	No
SRX4058991	19222104	Paired	0.0222807	0.979916	RF	82.67	Liver	No
SRX4058992	22337087	Paired	0.0249793	0.98062	RF	84.47	Liver	No
SRX4058993	22944579	Paired	0.0161035	0.9869	RF	91.84	Liver	No
SRX4058994	21592747	Paired	0.0201993	0.984501	RF	89.9	Liver	No
SRX4058995	7225778	Paired	0.035656	0.964352	RF	81.12	Testis	No
SRX4058996	18780273	Paired	0.0335724	0.967218	RF	91.71	Testis	No
SRX4058997	7370573	Paired	0.0342697	0.965429	RF	85.27	Testis	No
SRX4058998	6041981	Paired	0.0353386	0.965007	RF	88.19	Testis	No
SRX4058999	18768566	Paired	0.0318175	0.969939	RF	92.85	Testis	No

SRX3974020	28137263	Paired	0.0636251	0.930122	RF	64.41	Testis	No
SRX3974021	18137414	Paired	0.0268353	0.973011	RF	89.76	Testis	No
SRX3974022	15879583	Paired	0.0276448	0.972234	RF	85.57	Testis	No
SRX3974023	17824815	Paired	0.0266258	0.973633	RF	84.1	Testis	No
SRX3974024	17978487	Paired	0.0290784	0.970466	RF	87.55	Testis	No
SRX3974025	15421676	Paired	0.0268416	0.973449	RF	88.7	Testis	No
SRX3974026	17385847	Paired	0.0265731	0.973742	RF	85.91	Testis	No
SRX3974029	17421419	Paired	0.0476684	0.949292	RF	69.82	Testis	No
SRX4581360	45300731	Paired	0.0326934	0.967335	RF	83.43	Muscle	No
SRX4581361	44061095	Paired	0.0133677	0.986254	RF	66.09	Muscle	No
SRX4581362	48511263	Paired	0.0155675	0.983335	RF	64.21	Muscle	No
SRX4581363	47691264	Paired	0.0279688	0.972566	RF	83.79	Muscle	No
SRX4581364	46486465	Paired	0.0163389	0.984274	RF	63.16	Muscle	No
SRX4581365	49026612	Paired	0.0136433	0.986676	RF	64.57	Muscle	No
SRX4581366	39234136	Paired	0.0183285	0.981026	RF	70.53	Muscle	No
SRX4581367	44478572	Paired	0.018295	0.982214	RF	68.38	Muscle	No
SRX4581368	49272623	Paired	0.0160313	0.984988	RF	62.21	Muscle	No
SRX4581369	42798560	Paired	0.0217261	0.978604	RF	72.74	Muscle	No
SRX3974030	17247622	Paired	0.041025	0.956604	RF	64.4	Testis	No
SRX3974032	18334372	Paired	0.0418462	0.955076	RF	61	Testis	No
SRX4581370	53772451	Paired	0.0178851	0.982396	RF	68.94	Muscle	No
SRX4581371	41777430	Paired	0.022255	0.977624	RF	67.38	Muscle	No
SRX4581372	57622987	Paired	0.0144362	0.985874	RF	62.16	Muscle	No
SRX4581373	50029783	Paired	0.0378947	0.962302	RF	89.73	Muscle	No
SRX4581374	52091483	Paired	0.0242461	0.976514	RF	78.65	Muscle	No
SRX4581375	52689845	Paired	0.028588	0.971635	RF	84.27	Muscle	No
SRX4581376	54613499	Paired	0.0286502	0.971564	RF	86.82	Muscle	No
SRX4581377	55144245	Paired	0.0255849	0.974675	RF	65.15	Muscle	No
SRX4581378	52961459	Paired	0.029348	0.970821	RF	69.36	Muscle	No
SRX4581379	48975013	Paired	0.0254899	0.975641	RF	82.51	Muscle	No
SRX4581380	47009922	Paired	0.0219305	0.978834	RF	77.66	Muscle	No
SRX4581381	51990028	Paired	0.0288322	0.971942	RF	82.4	Muscle	No
SRX4581382	48213223	Paired	0.0271837	0.973608	RF	83.56	Muscle	No
SRX4581383	49951451	Paired	0.0290191	0.972412	RF	82.42	Muscle	No
SRX4581384	52693954	Paired	0.0269304	0.973679	RF	82.76	Muscle	No
SRX4581385	55940948	Paired	0.0249729	0.975448	RF	76.18	Muscle	No
SRX4581386	49507316	Paired	0.0280636	0.973467	RF	81.11	Muscle	No
SRX4581387	48109318	Paired	0.0284755	0.971793	RF	84.83	Muscle	No
SRX4581388	46933627	Paired	0.0237174	0.976664	RF	76.98	Muscle	No
SRX4581389	53048141	Paired	0.029164	0.970897	RF	79.72	Muscle	No
SRX9527195	41130788	Paired	0.0386773	0.960718	RF	90.29	Adipose	No
SRX9527196	29033153	Paired	0.0380086	0.960717	RF	75.5	Adipose	No
SRX9527197	41937430	Paired	0.048779	0.949148	RF	89.95	Adipose	No
SRX4581390	46840027	Paired	0.0199534	0.979622	RF	69.14	Muscle	No
SRX4581391	50761250	Paired	0.0179268	0.982713	RF	71.86	Muscle	No
SRX5202563	27598156	Single	0.0516257	NULL	RF	87.51	PBMCs	No
SRX4581392	51461537	Paired	0.0209452	0.980321	RF	70.44	Muscle	No
SRX5202564	26624867	Single	0.0526458	NULL	RF	87.1	PBMCs	No
SRX4581393	54828029	Paired	0.0211991	0.978905	RF	80	Muscle	No
SRX5202565	24613258	Single	0.0510583	NULL	RF	90.82	PBMCs	No
SRX4581394	48725281	Paired	0.0248597	0.975341	RF	70.87	Muscle	No
SRX5202566	26524039	Single	0.0474694	NULL	RF	90.69	PBMCs	No
SRX4581395	47915143	Paired	0.0228817	0.977251	RF	80.85	Muscle	No
SRX5202567	20901605	Single	0.049315	NULL	RF	90.54	PBMCs	No
SRX4581396	44897622	Paired	0.0353257	0.964908	RF	78.77	Muscle	No
SRX5202568	22632663	Single	0.0467927	NULL	RF	90.24	PBMCs	No
SRX4581397	50069217	Paired	0.025617	0.974703	RF	79.84	Muscle	No
SRX5202569	36491950	Single	0.0447812	NULL	RF	89.94	PBMCs	No

SRX4581398	39914398	Paired	0.018265	0.981321	RF	66.74	Muscle	No
SRX4581399	46375461	Paired	0.0139727	0.986432	RF	65.29	Muscle	No
SRX11632174	8946666	Paired	0.0252132	0.975771	RF	82.18	Small intestine	No
SRX11632175	15584572	Paired	0.0221245	0.97898	RF	86.87	Small intestine	No
SRX11632176	6284897	Paired	0.0280276	0.972929	RF	87.87	Small intestine	No
SRX11632178	11380018	Paired	0.0236658	0.977333	RF	84.68	Small intestine	No
SRX11632179	6917323	Paired	0.0289383	0.971991	RF	85.73	Small intestine	No
SRX5202570	32183962	Single	0.0468701	NULL	RF	89.75	PBMCs	No
SRX5202571	39312944	Single	0.0450796	NULL	RF	89.73	PBMCs	No
SRX5202572	15300699	Single	0.0494948	NULL	RF	85.07	PBMCs	No
ERX4132932	22851216	Paired	0.0266389	0.974716	RF	90.21	Small intestine	No
SRX5202573	37445171	Single	0.0449308	NULL	RF	88.81	PBMCs	No
ERX4132933	23312037	Paired	0.0195244	0.981544	RF	93.96	Kidney	No
SRX5202574	42336424	Single	0.0425134	NULL	RF	89.22	PBMCs	No
ERX4132934	29078436	Paired	0.0268405	0.974416	RF	91.45	Small intestine	No
SRX5202575	34254319	Single	0.0500065	NULL	RF	89.82	PBMCs	No
ERX4132935	33531252	Paired	0.0186831	0.982279	RF	93.73	Kidney	No
SRX5202576	46017475	Single	0.0475216	NULL	RF	88.93	PBMCs	No
ERX4132936	20879790	Paired	0.0259343	0.975293	RF	91.51	Small intestine	No
SRX5202577	45967506	Single	0.0460458	NULL	RF	89.69	PBMCs	No
ERX4132937	27576353	Paired	0.0197127	0.981218	RF	93.96	Kidney	No
SRX5202578	52882209	Single	0.0424355	NULL	RF	89.59	PBMCs	No
ERX4132938	53275192	Paired	0.0194469	0.981894	RF	90.77	Small intestine	No
SRX5202579	31589165	Single	0.0475879	NULL	RF	89.8	PBMCs	No
ERX4132939	20587143	Paired	0.0208996	0.980044	RF	93.04	Kidney	No
SRX11632180	7467971	Paired	0.0282152	0.972788	RF	87.85	Small intestine	No
SRX11632181	8272467	Paired	0.0262852	0.974593	RF	86.96	Small intestine	No
SRX11632182	8851449	Paired	0.0272152	0.973607	RF	86.59	Small intestine	No
SRX11632183	16491025	Paired	0.022732	0.978089	RF	83.5	Small intestine	No
SRX11632184	14088519	Paired	0.0232958	0.977803	RF	86.89	Small intestine	No
SRX11632185	17671412	Paired	0.0200854	0.981054	RF	86.73	Small intestine	No
SRX11632186	12764447	Paired	0.0231414	0.977632	RF	88.41	Small intestine	No
SRX11632187	12718792	Paired	0.0218846	0.978988	RF	86.86	Small intestine	No
SRX11632189	6859682	Paired	0.0284308	0.972375	RF	88.3	Small intestine	No
SRX12192844	26699399	Paired	0.0291516	0.969663	RF	92.75	Brain	No
SRX12192845	27720938	Paired	0.030175	0.968727	RF	93.1	Brain	No
SRX12192846	26237149	Paired	0.0300841	0.968979	RF	93.44	Brain	No
SRX12192847	28972979	Paired	0.0293801	0.968462	RF	91.44	Brain	No
SRX12192848	27904079	Paired	0.0309431	0.968869	RF	93.03	Brain	No
SRX12192849	25647215	Paired	0.030338	0.967465	RF	92.46	Brain	No
SRX5202580	25063091	Single	0.0462234	NULL	RF	88.3	PBMCs	No
ERX4132940	24552440	Paired	0.0206219	0.980751	RF	91.96	Small intestine	No
SRX5202581	23807106	Single	0.0480289	NULL	RF	90.43	PBMCs	No
ERX4132941	23814767	Paired	0.0210149	0.979828	RF	93.93	Kidney	No
SRX5202582	25060158	Single	0.0431789	NULL	RF	90.05	PBMCs	No
ERX4132942	22058046	Paired	0.0236652	0.97725	RF	91.16	Small intestine	No
ERX4132943	28795110	Paired	0.0187847	0.982292	RF	93.84	Kidney	No
SRX4250623	64987339	Paired	0.059247	0.940214	RF	83.61	Small intestine	No
ERX4132944	21396085	Paired	0.0234919	0.977742	RF	91.48	Small intestine	No
SRX4250624	64707607	Paired	0.907826	0.0897541	FR	85.61	Small intestine	No
ERX4132945	32009949	Paired	0.0190351	0.981923	RF	93.66	Kidney	No
SRX4250625	68647482	Paired	0.102044	0.894985	RF	83.4	Small intestine	No
ERX4132946	61999703	Paired	0.0191664	0.981785	RF	91.5	Small intestine	No
ERX4132947	28349873	Paired	0.0207191	0.980319	RF	93.83	Kidney	No
ERX4132948	35401509	Paired	0.0191987	0.982143	RF	91.47	Small intestine	No
ERX4132949	16426582	Paired	0.0208664	0.980038	RF	93.27	Kidney	No
ERX2292200	19374927	Paired	0.0321101	0.968979	RF	92.49	Brain	No
ERX2292201	18714557	Paired	0.0322509	0.968854	RF	92.48	Brain	No

SRX11632190	17615475	Paired	0.0212067	0.979745	RF	89.45	Small intestine	No
ERX2292202	12041990	Paired	0.0361992	0.964616	RF	92.97	Brain	No
SRX11632191	14346396	Paired	0.0237512	0.977449	RF	87.41	Small intestine	No
ERX2292203	12055773	Paired	0.0359044	0.965299	RF	93.55	Brain	No
SRX11632192	10622928	Paired	0.0240829	0.977087	RF	85.98	Small intestine	No
ERX2292204	12086787	Paired	0.0357049	0.965402	RF	93.47	Brain	No
SRX11632193	5265552	Paired	0.0311696	0.969754	RF	87.03	Small intestine	No
ERX2292205	13370338	Paired	0.0410847	0.959962	RF	92.9	Brain	No
SRX11632194	9286118	Paired	0.0261334	0.974755	RF	86.97	Small intestine	No
ERX2292206	13602209	Paired	0.0404939	0.960749	RF	93.45	Brain	No
SRX11632195	4256875	Paired	0.0345109	0.966703	RF	87.63	Small intestine	No
ERX2292207	13864382	Paired	0.0404175	0.960942	RF	93.55	Brain	No
SRX12192850	25402619	Paired	0.0295907	0.969969	RF	93.39	Brain	No
SRX11632196	4253837	Paired	0.0340468	0.966792	RF	87.6	Small intestine	No
ERX2292208	19432729	Paired	0.0283599	0.972275	RF	93.91	Brain	No
SRX12192851	41115822	Paired	0.0312239	0.967947	RF	64.47	Brain	No
ERX2292209	19534078	Paired	0.0281508	0.972472	RF	93.84	Brain	No
SRX12192852	28938505	Paired	0.028139	0.971431	RF	93.24	Brain	No
SRX11632198	8029944	Paired	0.0265136	0.97437	RF	84.85	Small intestine	No
SRX12192853	30054217	Paired	0.0286258	0.971037	RF	92.01	Brain	No
SRX11632199	11996987	Paired	0.023267	0.977593	RF	85.14	Small intestine	No
SRX12192854	26861698	Paired	0.0295736	0.96447	RF	91.8	Brain	No
SRX12192855	27792955	Paired	0.0287015	0.970163	RF	92.87	Brain	No
SRX12192856	29512186	Paired	0.0278704	0.971283	RF	92.46	Brain	No
SRX12192857	24859553	Paired	0.0318556	0.963073	RF	91	Brain	No
SRX12192858	54550872	Paired	0.0280756	0.971596	RF	90.51	Brain	No
SRX12192859	25124673	Paired	0.0288362	0.970215	RF	93.07	Brain	No
ERX4132950	44487851	Paired	0.018001	0.982948	RF	91.54	Small intestine	No
ERX4132951	22984854	Paired	0.0199997	0.980831	RF	93.5	Kidney	No
ERX4132952	22889165	Paired	0.0200995	0.98124	RF	91.23	Small intestine	No
ERX4132953	23980461	Paired	0.0205903	0.980287	RF	93.48	Kidney	No
ERX4132954	28380055	Paired	0.019755	0.981442	RF	90.68	Small intestine	No
ERX4132955	22846720	Paired	0.0212473	0.979742	RF	93.34	Kidney	No
ERX4132956	44308941	Paired	0.0186487	0.982459	RF	91	Small intestine	No
ERX4132957	55648425	Paired	0.0183808	0.982599	RF	93.56	Kidney	No
ERX4132958	31359148	Paired	0.0188096	0.982522	RF	90.35	Small intestine	No
ERX4132959	24443834	Paired	0.0197646	0.981177	RF	93.68	Kidney	No
ERX2292210	20065099	Paired	0.0281913	0.972294	RF	93.35	Brain	No
SRX12192860	27031793	Paired	0.0281581	0.96974	RF	92.05	Brain	No
SRX12192861	25368514	Paired	0.0329638	0.965327	RF	91.9	Brain	No
SRX12192862	26420871	Paired	0.0291383	0.970333	RF	92.33	Brain	No
SRX12192863	28458546	Paired	0.0279926	0.971433	RF	92.72	Brain	No
ERX4795802	147977597	Paired	0.0291031	0.968582	RF	90.75	Lung	No
SRX12192864	25313292	Paired	0.0309105	0.967623	RF	92.92	Brain	No
ERX4795803	164178112	Paired	0.0299351	0.967191	RF	91.06	Lung	No
SRX12192865	26374132	Paired	0.0288669	0.970708	RF	92.53	Brain	No
ERX4795804	144078669	Paired	0.0288209	0.968498	RF	90.8	Lung	No
SRX12192866	31256867	Paired	0.0283606	0.971412	RF	92.66	Brain	No
ERX4795805	148198382	Paired	0.026742	0.971228	RF	90.68	Lung	No
SRX12192867	31724919	Paired	0.0307279	0.966533	RF	93.09	Brain	No
ERX4795806	104422074	Paired	0.0258204	0.969391	RF	91.33	Brain	No
SRX12192868	30641859	Paired	0.0290339	0.971103	RF	92.82	Brain	No
ERX4795807	77259823	Paired	0.0265489	0.969275	RF	91.67	Brain	No
SRX12192869	26802503	Paired	0.0265579	0.972918	RF	91.31	Brain	No
ERX4795808	77580723	Paired	0.025762	0.970125	RF	91.77	Brain	No
ERX4795809	97384821	Paired	0.0245023	0.971191	RF	91.35	Brain	No
SRX7364993	59637873	Single	0.0804107	NULL	RF	75.89	Lung	No
SRX7364994	94433172	Single	0.0683998	NULL	RF	81.95	Lung	No

SRX12192870	31508300	Paired	0.0306382	0.966584	RF	91.56	Brain	No
ERX1696289	26668907	Paired	0.030559	0.970174	RF	91.65	Brain	No
ERX4795810	90419692	Paired	0.0213046	0.976658	RF	90.94	Skin	No
SRX12192871	27757788	Paired	0.0283559	0.971656	RF	93.78	Brain	No
ERX4795811	151623370	Paired	0.0254281	0.97254	RF	91.2	Skin	No
SRX12192872	32490576	Paired	0.027415	0.970217	RF	89.75	Brain	No
ERX4795812	127589199	Paired	0.022411	0.975375	RF	91.31	Skin	No
SRX12192873	27783867	Paired	0.0306159	0.967198	RF	91.65	Brain	No
ERX4795813	131752373	Paired	0.02125	0.976979	RF	91.18	Skin	No
SRX12192874	27642577	Paired	0.0299867	0.96866	RF	91.81	Brain	No
ERX4795814	85537096	Paired	0.0480851	0.93415	RF	90.09	Kidney	No
SRX12192875	28520770	Paired	0.0308222	0.967929	RF	93.26	Brain	No
ERX4795815	87652417	Paired	0.0408946	0.946943	RF	90.03	Kidney	No
SRX12192876	26467429	Paired	0.0280184	0.971702	RF	93.22	Brain	No
ERX4795816	93352426	Paired	0.0348675	0.954599	RF	91.14	Kidney	No
SRX12192877	28452266	Paired	0.0303729	0.969425	RF	92.38	Brain	No
ERX4795817	92739699	Paired	0.0419841	0.944259	RF	90.05	Kidney	No
SRX12192878	27978450	Paired	0.0294905	0.970361	RF	92.99	Brain	No
ERX4795818	73795577	Paired	0.0248995	0.970833	RF	91.09	Muscle	No
SRX12192879	27767392	Paired	0.0298004	0.970048	RF	92.52	Brain	No
ERX4795819	86214131	Paired	0.0234807	0.972754	RF	90.73	Muscle	No
ERX1696290	25911491	Paired	0.0292212	0.971659	RF	91.21	Brain	No
ERX1696291	32451287	Paired	0.0324832	0.968273	RF	90.71	Brain	No
ERX1696292	29710476	Paired	0.027293	0.973143	RF	91.76	Brain	No
ERX1696293	29940582	Paired	0.0358953	0.964202	RF	89.6	Brain	No
ERX1696294	33768633	Paired	0.0283543	0.972306	RF	91.07	Brain	No
ERX1696295	37375969	Paired	0.0298582	0.970628	RF	91.86	Brain	No
SRX12192880	45580130	Paired	0.0312106	0.967033	RF	91.39	Brain	No
ERX4795820	74093328	Paired	0.0272871	0.967736	RF	90.83	Muscle	No
SRX12192881	35259702	Paired	0.0306698	0.968461	RF	90.75	Brain	No
SRX5042940	19254574	Paired	0.0196607	0.980917	RF	87.1	Muscle	No
ERX4795821	96269267	Paired	0.0241574	0.971769	RF	90.79	Muscle	No
SRX12192882	29326267	Paired	0.032259	0.967317	RF	91.19	Brain	No
SRX5042941	22711066	Paired	0.0171157	0.983333	RF	88.89	Muscle	No
ERX4795822	86206743	Paired	0.0249382	0.974145	RF	89.88	Liver	No
SRX12192883	32831232	Paired	0.0315576	0.967682	RF	90.54	Brain	No
SRX5042942	31154454	Paired	0.0101944	0.990847	RF	85.72	Muscle	No
ERX4795823	106468531	Paired	0.027136	0.971105	RF	89.44	Liver	No
SRX12192884	26269837	Paired	0.0308681	0.967519	RF	91.51	Brain	No
SRX5042943	14404784	Paired	0.0160649	0.984473	RF	88.22	Muscle	No
ERX4795824	101464890	Paired	0.0274412	0.970341	RF	90.07	Liver	No
SRX12192885	28485500	Paired	0.0304922	0.968507	RF	90.25	Brain	No
SRX5042944	16456310	Paired	0.0140565	0.98679	RF	87.45	Muscle	No
ERX4795825	130192573	Paired	0.0244723	0.97471	RF	90.89	Liver	No
SRX12192886	22937688	Paired	0.0318261	0.967164	RF	89.73	Brain	No
SRX5042945	19367491	Paired	0.0124061	0.988487	RF	87.94	Muscle	No
ERX4795826	103784619	Paired	0.0287812	0.970019	RF	91.11	Lung	No
SRX12192887	26559228	Paired	0.0322124	0.966448	RF	91.12	Brain	No
SRX5042946	21941478	Paired	0.0137427	0.987134	RF	89.11	Muscle	No
ERX4795827	87426780	Paired	0.0291461	0.96907	RF	90.5	Lung	No
SRX12192888	26539153	Paired	0.0311924	0.967606	RF	90.48	Brain	No
SRX5042947	27259816	Paired	0.013622	0.987191	RF	86.3	Muscle	No
ERX4795828	139320041	Paired	0.0274268	0.971105	RF	91.07	Lung	No
SRX12192889	30126448	Paired	0.0290167	0.968576	RF	90.6	Brain	No
SRX5042948	32282523	Paired	0.0158316	0.98486	RF	88.64	Muscle	No
ERX4795829	131941932	Paired	0.0297793	0.968887	RF	90.85	Lung	No
SRX5042949	24271656	Paired	0.0161265	0.984507	RF	88.81	Muscle	No
SRX6492414	14325129	Single	0.0361179	NULL	RF	94.96	Brain	No

SRX6492415	16823636	Single	0.0314749	NULL	RF	94.14	Brain	No
SRX6492416	13599063	Single	0.0364072	NULL	RF	94.78	Brain	No
SRX6492417	15398721	Single	0.0348312	NULL	RF	94.78	Brain	No
SRX6492418	15245854	Single	0.0334566	NULL	RF	94.25	Brain	No
SRX6492419	15712696	Single	0.0330795	NULL	RF	93.98	Brain	No
SRX12192890	29090492	Paired	0.030415	0.967401	RF	90.48	Brain	No
SRX12192891	35197026	Paired	0.0309485	0.967321	RF	92.05	Brain	No
SRX5042950	24367657	Paired	0.0171606	0.983326	RF	88.79	Muscle	No
ERX4795830	88161785	Paired	0.0619316	0.910473	RF	89.43	Kidney	No
SRX12192892	32890230	Paired	0.0307323	0.966458	RF	90.5	Brain	No
SRX5042951	23944364	Paired	0.0180057	0.982822	RF	88.66	Muscle	No
ERX4795831	110715691	Paired	0.0676331	0.900971	RF	89.46	Kidney	No
SRX12192893	26476567	Paired	0.0387731	0.955062	RF	90.01	Brain	No
ERX4795832	117658091	Paired	0.0616931	0.908964	RF	89.64	Kidney	No
SRX12192894	28074488	Paired	0.0309552	0.964086	RF	90.44	Brain	No
ERX4795833	111391136	Paired	0.0576339	0.915574	RF	90.32	Kidney	No
SRX12192895	26374194	Paired	0.0298173	0.963103	RF	90.87	Brain	No
ERX4795834	81898964	Paired	0.0379765	0.950867	RF	90.16	Small intestine	No
SRX12192896	24814693	Paired	0.0311771	0.966646	RF	91.62	Brain	No
ERX4795835	77242833	Paired	0.0374648	0.95091	RF	90.79	Small intestine	No
SRX12192897	25252606	Paired	0.0295467	0.967601	RF	89.58	Brain	No
ERX4795836	85625158	Paired	0.0470148	0.93652	RF	89.89	Small intestine	No
SRX12192898	27762218	Paired	0.0301636	0.969011	RF	91.2	Brain	No
ERX4795837	136105408	Paired	0.0359515	0.954479	RF	90.44	Small intestine	No
SRX12192899	27904257	Paired	0.0330312	0.96611	RF	92.3	Brain	No
ERX4795838	95837755	Paired	0.0334171	0.957833	RF	91.54	Brain	No
ERX4795839	77821878	Paired	0.0394517	0.94766	RF	91.08	Brain	No
SRX6492420	15625457	Single	0.0322078	NULL	RF	94.14	Brain	No
SRX6492421	15053236	Single	0.031872	NULL	RF	94.54	Brain	No
SRX6492422	12913153	Single	0.0343675	NULL	RF	94.32	Brain	No
SRX6492423	17960341	Single	0.0327146	NULL	RF	95.08	Brain	No
SRX6492424	15278334	Single	0.0317117	NULL	RF	95.1	Brain	No
SRX6492425	14662012	Single	0.0315376	NULL	RF	94.43	Brain	No
SRX6492426	15149005	Single	0.035063	NULL	RF	95.02	Brain	No
SRX6492427	16590120	Single	0.0336487	NULL	RF	94.11	Brain	No
SRX6492428	14809981	Single	0.0361986	NULL	RF	94.87	Brain	No
SRX6492429	13304261	Single	0.034232	NULL	RF	94.74	Brain	No
ERX4795840	99906132	Paired	0.0420447	0.94345	RF	90.82	Brain	No
ERX4795841	135840997	Paired	0.0372762	0.951519	RF	91.64	Brain	No
ERX4795842	173344026	Paired	0.0296087	0.959781	RF	91.05	Muscle	No
ERX4795843	97816098	Paired	0.0243317	0.971835	RF	90.88	Muscle	No
ERX4795844	124106457	Paired	0.0263598	0.968105	RF	90.51	Muscle	No
ERX4795845	143545385	Paired	0.0244499	0.971429	RF	90.81	Muscle	No
ERX4795846	95725295	Paired	0.0359921	0.951129	RF	89.82	Skin	No
ERX4795847	121300779	Paired	0.0316578	0.958727	RF	89.21	Skin	No
ERX4795848	92150504	Paired	0.0297345	0.962238	RF	88.87	Skin	No
ERX4795849	116184360	Paired	0.0319119	0.959508	RF	89.44	Skin	No
SRX9198286	48266466	Paired	0.0891872	0.910943	RF	77.72	Lung	No
SRX6492430	16818177	Single	0.0333885	NULL	RF	94.18	Brain	No
SRX9198287	62023933	Paired	0.0816768	0.917816	RF	78.49	Lung	No
SRX6492431	16954470	Single	0.0323886	NULL	RF	94.37	Brain	No
SRX9198288	51993294	Paired	0.0952675	0.904664	RF	83.72	Lung	No
SRX6492432	17052916	Single	0.0308345	NULL	RF	94.4	Brain	No
SRX9198289	57496970	Paired	0.079065	0.921676	RF	84.12	Lung	No
SRX6492433	14252189	Single	0.0326224	NULL	RF	94.23	Brain	No
SRX6492434	17357221	Single	0.0307876	NULL	RF	94.56	Brain	No
SRX6492435	18464899	Single	0.0320144	NULL	RF	95.12	Brain	No
SRX6492436	18892238	Single	0.0316666	NULL	RF	95.16	Brain	No

SRX6492437	14912341	Single	0.0351246	NULL	RF	94.82	Brain	No
SRX9198290	52243068	Paired	0.0855167	0.915022	RF	79.63	Lung	No
SRX9198291	62356409	Paired	0.0956918	0.904555	RF	80.75	Lung	No
SRX9198292	52779813	Paired	0.0966632	0.903404	RF	78.87	Lung	No
SRX9198293	51794475	Paired	0.0837585	0.916359	RF	83.99	Lung	No
SRX9198294	44904974	Paired	0.0853869	0.915758	RF	81.67	Lung	No
SRX9198295	57584864	Paired	0.0897101	0.910491	RF	79.46	Lung	No
SRX9198296	59862042	Paired	0.0958535	0.904222	RF	81.47	Lung	No
SRX9198297	51822540	Paired	0.0799395	0.920836	RF	80.43	Lung	No
SRX9198298	54264981	Paired	0.0870901	0.913003	RF	84.25	Lung	No
SRX3194377	5978688	Paired	0.0696753	0.915122	RF	78.7	Brain	No
SRX3194378	9874348	Paired	0.0565389	0.937336	RF	81.69	Brain	No
SRX2353170	40701072	Paired	0.0215559	0.980056	RF	92.48	Muscle	No
SRX3194379	8279854	Paired	0.0578819	0.937473	RF	81.06	Brain	No
SRX2353172	55702737	Paired	0.0639753	0.936311	RF	93.66	Brain	No
SRX2353178	40563314	Paired	0.0281823	0.97191	RF	93.15	Brain	No
SRX2353179	72259718	Paired	0.0295048	0.97215	RF	89.83	Spleen	No
SRX3194380	9621120	Paired	0.0562359	0.939352	RF	82.31	Brain	No
SRX3194381	7281452	Paired	0.0635531	0.928251	RF	80.58	Brain	No
SRX3194382	9220386	Paired	0.0561109	0.939078	RF	81.19	Brain	No
SRX3194383	4419907	Paired	0.0676159	0.919684	RF	80.84	Brain	No
SRX3194384	7277045	Paired	0.0662157	0.929542	RF	82.24	Brain	No
SRX3194385	8164196	Paired	0.0614013	0.932568	RF	81.62	Brain	No
SRX3194386	9785016	Paired	0.06369	0.931562	RF	83.51	Brain	No
SRX3194387	7559072	Paired	0.0649278	0.925594	RF	80.55	Brain	No
SRX3194388	8990844	Paired	0.0566687	0.937599	RF	79.1	Brain	No
SRX2353180	63189709	Paired	0.0208795	0.980992	RF	91.77	Liver	No
SRX2353181	105892614	Paired	0.0398247	0.962314	RF	91.92	Brain	No
SRX2353182	33607039	Paired	0.0220033	0.978151	RF	91.64	Small intestine	No
ERX2260428	60801209	Paired	0.0222875	0.977517	RF	75.36	Liver	No
ERX2260429	42536639	Paired	0.0207236	0.979021	RF	75.59	Liver	No
SRX3971100	22966229	Single	0.0225444	NULL	RF	90.54	Embryo	No
SRX3971101	22178599	Single	0.021265	NULL	RF	93.77	Embryo	No
SRX3971102	27711722	Single	0.0196021	NULL	RF	94.42	Embryo	No
SRX3971103	25831372	Single	0.0201404	NULL	RF	94.21	Embryo	No
SRX3971104	27031395	Single	0.0202691	NULL	RF	88.87	Embryo	No
SRX3971105	25052953	Single	0.0200067	NULL	RF	93.17	Embryo	No
SRX3971106	28127800	Single	0.0206651	NULL	RF	94.34	Embryo	No
SRX3971107	33395551	Single	0.0194915	NULL	RF	93.38	Embryo	No
SRX3971108	24893822	Single	0.0195536	NULL	RF	95.01	Embryo	No
SRX3971109	35018956	Single	0.0183316	NULL	RF	91.86	Embryo	No
SRX7438704	33521469	Paired	0.0470024	0.951534	RF	82.44	Heart	No
ERX2260430	56688801	Paired	0.0211488	0.978571	RF	68.65	Liver	No
ERX2260431	49420558	Paired	0.021051	0.978463	RF	75.89	Liver	No
ERX2260432	52466649	Paired	0.0239473	0.975797	RF	72.24	Liver	No
ERX2260433	57612176	Paired	0.0201582	0.979343	RF	75.33	Liver	No
ERX2260434	58029185	Paired	0.0266014	0.973132	RF	64.84	Liver	No
ERX2260435	57387018	Paired	0.0294859	0.970057	RF	74.44	Liver	No
SRX3946651	48314104	Paired	0.928066	0.0717886	FR	63.54	Artery	No
ERX2260436	53143582	Paired	0.0193202	0.980895	RF	63.3	Liver	No
SRX3946652	46415652	Paired	0.899783	0.100272	FR	69.25	Artery	No
ERX2260437	42785049	Paired	0.016945	0.983023	RF	67.43	Liver	No
ERX2260438	48986987	Paired	0.0169947	0.982924	RF	66.99	Liver	No
ERX2260439	44555248	Paired	0.017278	0.982874	RF	63.43	Liver	No
SRX3971110	19502158	Single	0.025919	NULL	RF	89.94	Embryo	No
SRX3971111	28517717	Single	0.0252996	NULL	RF	92.69	Embryo	No
SRX3971112	26636235	Single	0.0212345	NULL	RF	93.13	Embryo	No
SRX3971113	37936878	Single	0.0179326	NULL	RF	94.6	Embryo	No

SRX3971114	19300604	Single	0.0186411	NULL	RF	94.93	Embryo	No
SRX3971115	24740834	Single	0.0179758	NULL	RF	94.56	Embryo	No
SRX3971116	31148637	Single	0.0179247	NULL	RF	92.91	Embryo	No
SRX3971117	32424831	Single	0.0172554	NULL	RF	94.43	Embryo	No
SRX3971118	39422597	Single	0.0165585	NULL	RF	95.16	Embryo	No
SRX3971119	27595026	Single	0.0197491	NULL	RF	94.97	Embryo	No
SRX7438715	53067292	Paired	0.018797	0.98167	RF	89.35	Liver	No
ERX2260440	45130558	Paired	0.017164	0.98289	RF	68.07	Liver	No
ERX2260442	47383566	Paired	0.0213665	0.97849	RF	67.91	Liver	No
ERX2260443	47352199	Paired	0.0245341	0.975214	RF	72.82	Liver	No
SRX7438726	37097827	Paired	0.0252496	0.974679	RF	83.04	Liver	No
SRX9372000	21445467	Paired	0.0318976	0.968746	RF	87.01	Brain	No
SRX12639673	24594778	Paired	0.157156	0.839623	RF	88.14	Brain	No
SRX7438737	57891738	Paired	0.0297617	0.97149	RF	88.12	Muscle	No
SRX12639675	23222634	Paired	0.175984	0.821158	RF	88.08	Brain	No
SRX12639676	23509088	Paired	0.184097	0.812805	RF	87.69	Brain	No
SRX12639677	23900514	Paired	0.175605	0.821169	RF	85.21	Brain	No
SRX2955861	6931037	Single	0.0290328	NULL	RF	93.98	Fibroblast	No
SRX2955862	5871174	Single	0.0279779	NULL	RF	94.1	Fibroblast	No
SRX2955863	9709289	Single	0.0243646	NULL	RF	94.39	Fibroblast	No
SRX2506325	22810168	Paired	0.121229	0.864631	RF	88.79	Kidney	No
SRX2506326	18064893	Paired	0.126389	0.862411	RF	88.92	Kidney	No
SRX7438748	33951329	Paired	0.0368488	0.963164	RF	82.3	Muscle	No
SRX7438759	49763204	Paired	0.044577	0.957513	RF	88.29	Spleen	No
SRX2365911	39321866	Paired	0.0226007	0.978406	RF	90.63	Liver	No
SRX2365915	48046391	Paired	0.0262283	0.974896	RF	91.39	Adipose	No
SRX2365917	21632434	Paired	0.0248275	0.975993	RF	90.39	Liver	No
SRX2365919	22841952	Paired	0.020969	0.980236	RF	86.73	Muscle	No
SRX11005659	24868893	Paired	0.0716919	0.92312	RF	88.25	Ovary	No
SRX11677230	24903285	Paired	0.0204438	0.978924	RF	86.57	Muscle	No
SRX11677231	21192959	Paired	0.0195573	0.979466	RF	87.17	Muscle	No
SRX11677232	31190246	Paired	0.0220951	0.980143	RF	86.12	Muscle	No
SRX11677233	26567959	Paired	0.0226639	0.979791	RF	84.56	Muscle	No
SRX11677234	23152636	Paired	0.0196872	0.982368	RF	87.24	Muscle	No
SRX11677235	22437806	Paired	0.0197758	0.98275	RF	85.2	Muscle	No
SRX11677236	25613093	Paired	0.0195124	0.982259	RF	86.04	Muscle	No
SRX11677237	26168593	Paired	0.0195144	0.982092	RF	85.17	Muscle	No
SRX11677238	26119594	Paired	0.0197861	0.981877	RF	85.04	Muscle	No
SRX11677239	26747692	Paired	0.0197798	0.982031	RF	87.65	Muscle	No
SRX7438770	37205238	Paired	0.0411552	0.96029	RF	83.65	Spleen	No
SRX11005660	23532185	Paired	0.0745163	0.920246	RF	87.66	Ovary	No
SRX11005661	23139795	Paired	0.0703295	0.927861	RF	89.55	Ovary	No
SRX11005662	20886503	Paired	0.0692204	0.926352	RF	87.24	Ovary	No
SRX11677240	26826916	Paired	0.0206626	0.980811	RF	84.96	Muscle	No
SRX11677241	27620105	Paired	0.0198136	0.981867	RF	83.9	Muscle	No
SRX11677242	31826257	Paired	0.0194089	0.980793	RF	87.79	Muscle	No
SRX11677243	31530626	Paired	0.0199932	0.979713	RF	86.56	Muscle	No
SRX11677244	23998073	Paired	0.0212541	0.981178	RF	86.8	Muscle	No
SRX11677245	23228073	Paired	0.0204045	0.981783	RF	87.41	Muscle	No
SRX11677246	28558132	Paired	0.0211922	0.980546	RF	86.4	Muscle	No
SRX11677247	31819412	Paired	0.0199053	0.981413	RF	84.62	Muscle	No
SRX11677248	43923506	Paired	0.0198228	0.982118	RF	85.74	Muscle	No
SRX11677249	27460187	Paired	0.0208702	0.981021	RF	87.3	Muscle	No
SRX2365930	21652997	Paired	0.0247371	0.976083	RF	90.57	Liver	No
SRX2365932	25586476	Paired	0.0283567	0.972648	RF	91.35	Adipose	No
SRX2365934	25062188	Paired	0.0284941	0.972468	RF	91.06	Adipose	No
SRX2365937	44773864	Paired	0.0182088	0.982917	RF	86.81	Muscle	No
SRX2365938	22921290	Paired	0.0209019	0.980313	RF	86.96	Muscle	No

SRX7438781	52655902	Paired	0.0270617	0.972813	RF	85.98	Heart	No
SRX7438782	38049595	Paired	0.0366071	0.962019	RF	84.67	Adipose	No
SRX1533923	63027878	Paired	0.0582526	0.937042	RF	91.39	Muscle	No
SRX1533924	85543697	Paired	0.0555798	0.93936	RF	90.36	Muscle	No
SRX1533925	112721563	Paired	0.0584082	0.936286	RF	88.35	Muscle	No
SRX1533926	66401545	Paired	0.0393091	0.959408	RF	90.42	Muscle	No
SRX1533927	64294907	Paired	0.0537304	0.939789	RF	90.68	Muscle	No
SRX1533928	112721563	Paired	0.0584079	0.936305	RF	88.35	Muscle	No
SRX1533929	83035279	Paired	0.0492465	0.945613	RF	88.91	Muscle	No
SRX4641289	7225778	Paired	0.035761	0.964351	RF	81.12	Testis	No
SRX7438793	42558220	Paired	0.0272847	0.972757	RF	82.62	Adipose	No
SRX1533930	79001881	Paired	0.0489234	0.944732	RF	70.83	Muscle	No
SRX1533932	92511536	Paired	0.049427	0.945065	RF	80.24	Muscle	No
SRX1533933	54765665	Paired	0.0607248	0.933381	RF	88.41	Muscle	No
SRX1533934	72960194	Paired	0.055904	0.937723	RF	89.2	Muscle	No
SRX1533935	72746563	Paired	0.0561858	0.937075	RF	90.35	Muscle	No
SRX1533936	55576690	Paired	0.0430735	0.952673	RF	88.32	Muscle	No
SRX1533937	74775829	Paired	0.049976	0.944278	RF	90.09	Muscle	No
SRX1533938	70709767	Paired	0.0493427	0.943187	RF	86.31	Muscle	No
SRX1533939	67919070	Paired	0.0539037	0.938677	RF	89.28	Muscle	No
SRX4641290	18780273	Paired	0.0336118	0.967303	RF	91.71	Testis	No
SRX2238915	46023048	Paired	0.0336544	0.966176	RF	86.54	Adipose	No
SRX4641291	7370573	Paired	0.0342611	0.965425	RF	85.27	Testis	No
SRX2238916	43074935	Paired	0.0351603	0.964306	RF	88.58	Adipose	No
SRX4641292	6041981	Paired	0.0352658	0.965007	RF	88.19	Testis	No
SRX4641293	18768566	Paired	0.0317835	0.969915	RF	92.85	Testis	No
SRX4641294	4521704	Paired	0.0457028	0.954211	RF	77.09	Testis	No
SRX4641295	5283264	Paired	0.037535	0.961958	RF	81.02	Testis	No
SRX4641296	6567665	Paired	0.0367141	0.963158	RF	76.67	Testis	No
SRX4641297	22133528	Paired	0.0366097	0.965292	RF	91.49	Testis	No
SRX4641298	3767917	Paired	0.0519178	0.949851	RF	74.94	Testis	No
SRX4641299	33998286	Paired	0.0290115	0.971599	RF	92.66	Testis	No
SRX9635607	26851291	Paired	0.0627112	0.945438	RF	78.08	Whole blood	No
SRX9635608	30992390	Paired	0.0504803	0.953748	RF	83.72	Whole blood	No
SRX9635609	30175445	Paired	0.0766332	0.93013	RF	69.76	Whole blood	No
SRX1533940	65472943	Paired	0.0603398	0.928088	RF	90.08	Muscle	No
SRX1533941	83126789	Paired	0.0612983	0.924058	RF	90.81	Muscle	No
SRX9635610	39804664	Paired	0.0675974	0.941284	RF	69.45	Whole blood	No
SRX9635611	33843069	Paired	0.0558397	0.950098	RF	80.62	Whole blood	No
SRX9635612	27314505	Paired	0.0665879	0.938695	RF	74.76	Whole blood	No
SRX9635613	42652300	Paired	0.0426768	0.959591	RF	87.61	Whole blood	No
SRX9635614	42608504	Paired	0.052619	0.953949	RF	84.67	Whole blood	No
SRX9635615	31092166	Paired	0.0395196	0.961015	RF	86.23	Whole blood	No
SRX9635616	39664504	Paired	0.0754177	0.938389	RF	70.26	Whole blood	No
SRX9635617	32967838	Paired	0.048845	0.956017	RF	84.56	Whole blood	No
SRX9635618	30931168	Paired	0.0794432	0.935703	RF	62.72	Whole blood	No
SRX9635619	23319178	Paired	0.0511312	0.953876	RF	84.48	Whole blood	No
SRX9635620	33499231	Paired	0.0724742	0.939932	RF	72.4	Whole blood	No
SRX9635621	42058347	Paired	0.0589234	0.950515	RF	78.39	Whole blood	No
SRX9635622	31455065	Paired	0.0730077	0.941012	RF	73.87	Whole blood	No
SRX9635623	27116909	Paired	0.0675491	0.946516	RF	82.02	Whole blood	No
SRX9635624	33510470	Paired	0.0714709	0.941526	RF	71.1	Whole blood	No
SRX9635627	47185533	Paired	0.071976	0.941195	RF	62.83	Whole blood	No
SRX9635628	38410182	Paired	0.0688036	0.942483	RF	70.29	Whole blood	No
SRX9635629	28100638	Paired	0.0772838	0.938301	RF	63.6	Whole blood	No
SRX8241172	49110005	Paired	0.0293846	0.970071	RF	82.95	Brain	No
SRX8241173	60111218	Paired	0.0348369	0.964877	RF	72.51	Brain	No
SRX8241174	65720931	Paired	0.0328096	0.966903	RF	82.36	Brain	No

SRX8241175	64753481	Paired	0.0321375	0.967249	RF	79.87	Brain	No
SRX8241176	49634715	Paired	0.0312915	0.969014	RF	82.15	Brain	No
SRX8241177	66042764	Paired	0.0329261	0.966813	RF	80.09	Brain	No
SRX8241178	69213315	Paired	0.0284455	0.97155	RF	85.97	Brain	No
SRX8241179	70201967	Paired	0.0311336	0.968849	RF	77.77	Brain	No
SRX9635630	41622075	Paired	0.0742363	0.9395	RF	67.31	Whole blood	No
SRX9635631	33579124	Paired	0.089596	0.928916	RF	65.53	Whole blood	No
SRX9635632	35740949	Paired	0.026617	0.972072	RF	90.23	Lymph node	No
SRX9635633	36393263	Paired	0.0399074	0.958055	RF	90.33	Lymph node	No
SRX9635634	25514658	Paired	0.0213084	0.97804	RF	89.23	Lymph node	No
SRX9635635	28866403	Paired	0.0225223	0.977573	RF	91.54	Lymph node	No
SRX9635636	31331086	Paired	0.0293404	0.969555	RF	91.64	Lymph node	No
SRX9635637	43078647	Paired	0.0219731	0.977135	RF	90.47	Lymph node	No
SRX9635638	35700724	Paired	0.0218294	0.975964	RF	90.29	Lymph node	No
SRX9635639	30498865	Paired	0.0277677	0.972618	RF	91.69	Lymph node	No
SRX8241180	70943893	Paired	0.0391787	0.960267	RF	79.51	Brain	No
SRX8241181	51257222	Paired	0.0309159	0.968919	RF	81.07	Brain	No
SRX8241182	53883033	Paired	0.0313167	0.968549	RF	82.12	Brain	No
SRX8241183	76018411	Paired	0.0296134	0.970515	RF	81.71	Brain	No
SRX8241184	78498013	Paired	0.0335275	0.967199	RF	84.08	Brain	No
SRX8241185	68271311	Paired	0.0424245	0.957164	RF	78.39	Brain	No
SRX8241186	72913986	Paired	0.0424075	0.95757	RF	73.17	Brain	No
SRX8241187	83703296	Paired	0.028458	0.971796	RF	84.49	Brain	No
SRX8241188	73376693	Paired	0.0346973	0.96516	RF	85.85	Brain	No
SRX8241189	69809576	Paired	0.0296149	0.970293	RF	84.15	Brain	No
SRX9635640	17507089	Paired	0.0366133	0.96215	RF	90.97	Lymph node	No
SRX9635641	34906357	Paired	0.079024	0.936448	RF	70.67	Whole blood	No
SRX9635642	41808921	Paired	0.023186	0.976205	RF	89.36	Lymph node	No
SRX9635643	33254031	Paired	0.0257532	0.973754	RF	91.15	Lymph node	No
SRX9635644	28834476	Paired	0.0259084	0.974017	RF	89.16	Lymph node	No
SRX9635645	26777510	Paired	0.0243513	0.975351	RF	89.43	Lymph node	No
SRX9635646	21397426	Paired	0.0305013	0.969151	RF	90.57	Lymph node	No
SRX9635647	26828252	Paired	0.0230527	0.975779	RF	89.93	Lymph node	No
SRX9635648	34587926	Paired	0.021001	0.979501	RF	92.25	Lymph node	No
SRX9635649	25683254	Paired	0.0233326	0.974366	RF	89.89	Lymph node	No
ERX1886700	31823363	Paired	0.0727526	0.885979	RF	84.1	Large intestine	No
ERX1886701	110107372	Paired	0.0627277	0.905062	RF	83.91	Small intestine	No
ERX1886702	38263798	Paired	0.0615148	0.890522	RF	87.28	Small intestine	No
SRX8241190	56578362	Paired	0.0391077	0.96124	RF	81.06	Brain	No
ERX1886704	31263836	Paired	0.0936424	0.866314	RF	89.31	Brain	No
SRX8241191	62670760	Paired	0.0263838	0.973613	RF	82.85	Brain	No
SRX8241192	62617967	Paired	0.0288852	0.971503	RF	84.51	Brain	No
ERX1886706	30777825	Paired	0.0991842	0.875375	RF	83.27	Large intestine	No
SRX8241193	72352318	Paired	0.0304198	0.969467	RF	81.48	Brain	No
SRX8241194	59735773	Paired	0.0290788	0.971172	RF	87.57	Brain	No
ERX1886708	19963693	Paired	0.10364	0.865968	RF	88.8	Brain	No
SRX8241195	61186936	Paired	0.0289302	0.97125	RF	86.52	Brain	No
ERX1886709	28815834	Paired	0.107506	0.862264	RF	89.09	Brain	No
SRX9635650	35055674	Paired	0.0211999	0.978435	RF	90.58	Lymph node	No
SRX9635651	34596399	Paired	0.0273473	0.972171	RF	89.61	Lymph node	No
SRX9635652	30816383	Paired	0.0427208	0.958718	RF	81.74	Whole blood	No
SRX9635653	24900797	Paired	0.0243031	0.975798	RF	90.39	Lymph node	No
SRX9635654	34901910	Paired	0.0288022	0.971322	RF	91.18	Lymph node	No
SRX9635655	40435873	Paired	0.0251564	0.974707	RF	90.51	Lymph node	No
SRX9635656	23557999	Paired	0.0261033	0.972983	RF	90.61	Lymph node	No
SRX9635657	34119175	Paired	0.0233807	0.977161	RF	91.08	Lymph node	No
SRX9635658	36405682	Paired	0.048769	0.947208	RF	90.22	Lymph node	No
SRX9635659	29586336	Paired	0.0518346	0.941817	RF	89.34	Lymph node	No

SRX2390468	53014578	Paired	0.0232148	0.972779	RF	83.03	IPEC-J2	No
ERX779606	45140397	Paired	0.0254372	0.975781	RF	85.9	Adipose	No
ERX779607	44880588	Paired	0.0236839	0.977846	RF	86.42	Heart	No
ERX779608	45567478	Paired	0.0269438	0.974095	RF	86.49	Kidney	No
ERX779609	50333518	Paired	0.021472	0.980556	RF	84.37	Liver	No
SRX5588517	45207464	Paired	0.0204143	0.979297	RF	78.36	Small intestine	No
SRX5588518	27100801	Paired	0.0210353	0.97902	RF	83.15	Small intestine	No
SRX5588519	28145797	Paired	0.0217375	0.976902	RF	85.43	Small intestine	No
ERX1886710	17348112	Paired	0.0895527	0.886769	RF	86.65	Brain	No
ERX1886711	21830197	Paired	0.0973714	0.879266	RF	86.77	Brain	No
ERX1886712	28340080	Paired	0.0818556	0.863014	RF	82.84	Brain	No
ERX1886713	31276416	Paired	0.0967864	0.861137	RF	85.62	Brain	No
ERX1886714	30277445	Paired	0.110419	0.862963	RF	89.66	Brain	No
ERX1886715	25589842	Paired	0.101759	0.876237	RF	88.64	Brain	No
ERX1886716	35168045	Paired	0.097607	0.867858	RF	86.98	Brain	No
ERX1886717	21285637	Paired	0.104161	0.865672	RF	86.87	Brain	No
ERX1886718	20569673	Paired	0.0924445	0.85779	RF	87.45	Brain	No
ERX1886719	26893263	Paired	0.0844264	0.867083	RF	86.14	Brain	No
SRX9635660	22578966	Paired	0.0501051	0.939498	RF	84.85	Lymph node	No
SRX9635661	28179321	Paired	0.0479441	0.946363	RF	88	Lymph node	No
SRX9635662	21667188	Paired	0.0469817	0.952213	RF	88.81	Lymph node	No
SRX9635663	32948860	Paired	0.0846898	0.936303	RF	72.03	Whole blood	No
SRX9635664	25189570	Paired	0.05098	0.945095	RF	91.13	Lymph node	No
SRX9635665	15404182	Paired	0.0490507	0.925571	RF	84.41	Lymph node	No
SRX9635666	22253117	Paired	0.0531186	0.938882	RF	89.54	Lymph node	No
SRX9635667	32587165	Paired	0.0426408	0.961084	RF	88.55	Whole blood	No
SRX2390474	53011063	Paired	0.0243054	0.971473	RF	84.16	IPEC-J2	No
SRX9635668	42641615	Paired	0.0650842	0.944775	RF	75.37	Whole blood	No
SRX9635669	45921077	Paired	0.0521583	0.954209	RF	81.45	Whole blood	No
ERX779610	40121094	Paired	0.0305745	0.970643	RF	80.29	Lung	No
ERX779612	46283609	Paired	0.015587	0.98635	RF	85.42	Muscle	No
ERX779613	37468410	Paired	0.0326983	0.968324	RF	70.47	Spleen	No
SRX5588520	22945128	Paired	0.0222048	0.977432	RF	78.1	Small intestine	No
SRX5588521	29692137	Paired	0.023177	0.975673	RF	85.28	Small intestine	No
SRX5588522	31778485	Paired	0.0208519	0.977593	RF	80.94	Small intestine	No
SRX5588523	22960341	Paired	0.0223984	0.977344	RF	84.43	Small intestine	No
SRX5588524	20591465	Paired	0.0216639	0.976661	RF	78.32	Small intestine	No
SRX5588525	22847924	Paired	0.0246587	0.974806	RF	83.57	Small intestine	No
SRX5588526	21468415	Paired	0.0232707	0.976555	RF	83.25	Small intestine	No
SRX5588527	22266548	Paired	0.0333818	0.967568	RF	79.19	Small intestine	No
SRX5588528	38329828	Paired	0.0229997	0.976989	RF	82.69	Small intestine	No
SRX5588529	23958967	Paired	0.030594	0.969975	RF	83.98	Small intestine	No
ERX1886720	26545382	Paired	0.0782941	0.877297	RF	84.23	Brain	No
ERX1886721	20235444	Paired	0.107586	0.867601	RF	84.37	Brain	No
ERX1886722	27853420	Paired	0.120158	0.850229	RF	86.67	Brain	No
ERX1886723	28341391	Paired	0.0947324	0.863642	RF	87.56	Brain	No
ERX1886724	30641282	Paired	0.107638	0.866815	RF	86.7	Brain	No
SRX2390481	52962201	Paired	0.0213912	0.973794	RF	81.74	IPEC-J2	No
SRX2390486	53153849	Paired	0.0282535	0.966607	RF	82.93	IPEC-J2	No
SRX5588530	40432692	Paired	0.0274463	0.972402	RF	86.41	Small intestine	No
SRX5588531	37095118	Paired	0.0249385	0.97545	RF	76.99	Small intestine	No
SRX5588532	24221020	Paired	0.0273834	0.97378	RF	79.77	Small intestine	No
SRX5588533	36981634	Paired	0.0223572	0.977135	RF	82.71	Small intestine	No
SRX5588534	22832460	Paired	0.032591	0.968467	RF	73.5	Small intestine	No
SRX5588535	22527217	Paired	0.0275022	0.972812	RF	80.91	Small intestine	No
SRX5588536	32714769	Paired	0.0228234	0.977582	RF	68.5	Small intestine	No
SRX5588537	23447533	Paired	0.0223532	0.978346	RF	74.86	Small intestine	No
SRX5588538	22290020	Paired	0.0226752	0.97801	RF	74.75	Small intestine	No

SRX5588539	30897012	Paired	0.0270234	0.972967	RF	70.13	Small intestine	No
SRX4345476	22436597	Paired	0.0273112	0.972642	RF	87.51	PBMCs	No
SRX4345477	23132842	Paired	0.0275846	0.972183	RF	87.31	PBMCs	No
SRX4345478	18611764	Paired	0.0268655	0.972445	RF	86.82	PBMCs	No
SRX4345479	24338159	Paired	0.0261036	0.973659	RF	87.51	PBMCs	No
SRX5588540	34503251	Paired	0.0221641	0.977764	RF	82.03	Small intestine	No
SRX5588541	21493532	Paired	0.0231178	0.977418	RF	81.3	Small intestine	No
SRX5588542	23408490	Paired	0.0253097	0.975574	RF	76.02	Small intestine	No
SRX5588543	40854164	Paired	0.0215096	0.977505	RF	82.11	Small intestine	No
SRX5588544	36721424	Paired	0.021138	0.978219	RF	80.14	Small intestine	No
SRX5588545	23915435	Paired	0.0210634	0.979205	RF	81.81	Small intestine	No
SRX5588546	23507466	Paired	0.0229876	0.977592	RF	82.84	Small intestine	No
SRX6828034	68570961	Paired	0.0419825	0.950501	RF	91.42	Muscle	No
SRX6828035	62358742	Paired	0.0365462	0.958426	RF	84.24	Muscle	No
SRX6828036	76409240	Paired	0.0348402	0.957103	RF	83.88	Brain	No
SRX6828037	85210648	Paired	0.035999	0.954152	RF	82.74	Brain	No
SRX6828038	77401764	Paired	0.0344782	0.958423	RF	83.04	Brain	No
SRX6828039	78023701	Paired	0.0367929	0.956696	RF	82.48	Brain	No
ERX3212527	38712447	Paired	0.0267949	0.97167	RF	88.66	Macrophage	No
ERX3212528	40195246	Paired	0.027294	0.970852	RF	88.15	Macrophage	No
ERX3212529	45925854	Paired	0.0279232	0.970275	RF	86.02	Macrophage	No
ERX1950927	29092056	Paired	0.0159141	0.985253	RF	92.59	Fibroblast	No
ERX1950928	31564781	Paired	0.017991	0.983471	RF	92.82	Fibroblast	No
ERX1950929	37770449	Paired	0.0150794	0.986007	RF	92.69	Fibroblast	No
SRX4345480	22384954	Paired	0.0277337	0.972118	RF	87.09	PBMCs	No
SRX4345481	19897519	Paired	0.0256576	0.974054	RF	87.34	PBMCs	No
SRX6828040	78938342	Paired	0.0370677	0.957035	RF	82.92	Brain	No
SRX6828041	108410244	Paired	0.0346521	0.959262	RF	81.87	Brain	No
SRX6828042	85937174	Paired	0.021087	0.974783	RF	91.42	Liver	No
SRX6828043	68651041	Paired	0.0206229	0.889239	RF	70.49	Liver	No
SRX6828044	63883388	Paired	0.0352346	0.957349	RF	84.1	Muscle	No
SRX6828045	64575317	Paired	0.0378903	0.958922	RF	84.95	Muscle	No
SRX6828046	65960751	Paired	0.0183052	0.979382	RF	82.13	Liver	No
SRX6828047	72769593	Paired	0.0224877	0.973842	RF	77.22	Liver	No
SRX6828048	62586656	Paired	0.036953	0.957902	RF	85.2	Brain	No
SRX6828049	92193981	Paired	0.0374226	0.942321	RF	81.24	Brain	No
ERX3212530	45202176	Paired	0.0301949	0.967814	RF	87.19	Macrophage	No
SRX9339800	40510866	Paired	0.979909	0.0214754	FR	85.07	Muscle	No
ERX3212531	36043020	Paired	0.02831	0.969539	RF	86.65	Macrophage	No
SRX9339801	57504715	Paired	0.978867	0.0224257	FR	86.56	Muscle	No
ERX3212532	43492045	Paired	0.0275229	0.970296	RF	86.84	Macrophage	No
SRX9339802	50578297	Paired	0.979856	0.0216163	FR	82.15	Muscle	No
ERX3212533	37664250	Paired	0.0365061	0.961494	RF	89.85	Macrophage	No
SRX9339803	56776524	Paired	0.976966	0.0235619	FR	78.2	Muscle	No
ERX3212534	44828782	Paired	0.0375426	0.960556	RF	89.32	Macrophage	No
SRX9339804	46971540	Paired	0.977738	0.0229942	FR	79.81	Muscle	No
ERX3212535	41812047	Paired	0.0353802	0.961807	RF	88.76	Macrophage	No
SRX9339805	46282346	Paired	0.975733	0.0247364	FR	81.04	Muscle	No
ERX3212536	44244962	Paired	0.0361513	0.961126	RF	89.41	Macrophage	No
ERX3212537	40687120	Paired	0.0370131	0.959689	RF	87.7	Macrophage	No
ERX3212538	47385866	Paired	0.0382052	0.95807	RF	87.07	Macrophage	No
ERX3212539	44036791	Paired	0.0310369	0.965836	RF	87.94	Macrophage	No
ERX1950930	37334853	Paired	0.0146597	0.986369	RF	91.16	Fibroblast	No
ERX1950931	34044359	Paired	0.0155645	0.985686	RF	92.23	Fibroblast	No
ERX1950932	38384919	Paired	0.0157037	0.985466	RF	91.04	Fibroblast	No
ERX1950933	38773809	Paired	0.0428947	0.958567	RF	91.58	Fibroblast	No
ERX1950934	32984782	Paired	0.0157237	0.985434	RF	90.96	Fibroblast	No
ERX1950935	30394090	Paired	0.0170607	0.984328	RF	92.92	Fibroblast	No

ERX1950936	32752481	Paired	0.019044	0.982803	RF	92.37	Fibroblast	No
ERX1950937	31688581	Paired	0.0519549	0.949541	RF	92.14	Fibroblast	No
ERX1950938	36605182	Paired	0.0195768	0.982027	RF	91.98	Fibroblast	No
ERX1950939	30468782	Paired	0.0179842	0.983489	RF	91.62	Fibroblast	No
ERX4193397	58782494	Paired	0.0362136	0.96066	RF	89.1	Large intestine	No
ERX4193398	53469420	Paired	0.0288221	0.968813	RF	86.63	Small intestine	No
ERX4193399	67206153	Paired	0.0370038	0.959396	RF	89.71	Small intestine	No
ERX3212540	41636581	Paired	0.0284088	0.969147	RF	87.32	Macrophage	No
ERX3212541	41297899	Paired	0.0288591	0.968004	RF	87.45	Macrophage	No
ERX3212542	44878857	Paired	0.0404324	0.954552	RF	89.04	Macrophage	No
ERX3212543	42811602	Paired	0.0291167	0.969048	RF	86.21	Macrophage	No
ERX3212544	40713588	Paired	0.031347	0.966456	RF	87.38	Macrophage	No
ERX3212545	46082774	Paired	0.05255	0.942994	RF	85.11	Macrophage	No
ERX3212546	42684218	Paired	0.0496473	0.946559	RF	84.72	Macrophage	No
ERX3212547	35967512	Paired	0.0442837	0.952113	RF	87.07	Macrophage	No
ERX3212548	38013954	Paired	0.0416799	0.955925	RF	85.89	Macrophage	No
ERX3212549	42713753	Paired	0.0406137	0.956758	RF	88.54	Macrophage	No
SRX8483900	53874100	Paired	0.0347689	0.965603	RF	86.38	Adipose	No
ERX3212550	46607846	Paired	0.0461043	0.95034	RF	87.01	Macrophage	No
SRX8483901	60214234	Paired	0.0359962	0.964534	RF	87.99	Muscle	No
SRX8483902	47117258	Paired	0.0479928	0.951301	RF	90.2	Muscle	No
SRX8483903	59950272	Paired	0.0385533	0.962151	RF	86.87	Muscle	No
SRX8483904	53730503	Paired	0.0388947	0.962363	RF	87.4	Muscle	No
SRX8483906	46787130	Paired	0.0328941	0.968252	RF	84.54	Muscle	No
SRX8483907	57852847	Paired	0.0361265	0.963443	RF	88.36	Ovary	No
SRX8483908	47632671	Paired	0.0376444	0.96235	RF	88.47	Ovary	No
SRX8483911	34378915	Paired	0.0406323	0.957487	RF	66.78	PK15	No
SRX8483912	62849993	Paired	0.0370728	0.96391	RF	85.47	Muscle	No
SRX3293175	21352255	Paired	0.0313552	0.966702	RF	89.1	Ovary	No
SRX8483913	61196388	Paired	0.0548095	0.945462	RF	88.37	Muscle	No
SRX3293176	10770623	Paired	0.0323308	0.960297	RF	76.71	Ovary	No
SRX8483914	45989131	Paired	0.0388313	0.963361	RF	85.45	Muscle	No
SRX3293177	19481582	Paired	0.0307785	0.964818	RF	87.08	Ovary	No
SRX3293178	11097718	Paired	0.030573	0.952963	RF	75.08	Ovary	No
SRX3293179	28273309	Paired	0.0309138	0.968131	RF	89.37	Ovary	No
SRX8483917	37788276	Paired	0.032981	0.967889	RF	82.55	Muscle	No
SRX8483918	43463467	Paired	0.178896	0.816818	RF	82.1	Muscle	No
SRX8483919	70011686	Paired	0.0285637	0.973354	RF	85.34	Muscle	No
SRX3973939	16044201	Paired	0.0238621	0.978553	RF	71.88	Liver	No
SRX3293180	12168563	Paired	0.0338464	0.961209	RF	77.78	Ovary	No
SRX3293181	18201778	Paired	0.0325755	0.9587	RF	86.23	Ovary	No
SRX3293182	8629903	Paired	0.036613	0.956573	RF	74.98	Ovary	No
SRX8483920	55595721	Paired	0.0362318	0.96475	RF	89.07	Muscle	No
SRX3293183	24050175	Paired	0.030316	0.968716	RF	89.49	Ovary	No
SRX8483921	57964113	Paired	0.03355	0.967805	RF	87.89	Muscle	No
SRX3293184	10095534	Paired	0.033688	0.95049	RF	75.23	Ovary	No
SRX8483922	54489492	Paired	0.0420599	0.958285	RF	84.58	Muscle	No
SRX3293185	12583360	Paired	0.0315932	0.961793	RF	77.03	Ovary	No
SRX8483923	38768802	Paired	0.0887846	0.909683	RF	83.41	Muscle	No
SRX3293186	22829149	Paired	0.0306025	0.967587	RF	89.04	Ovary	No
SRX8483924	55722655	Paired	0.0398127	0.960293	RF	87.58	Adipose	No
SRX3293187	9302087	Paired	0.0345846	0.961544	RF	76.75	Ovary	No
SRX8483925	49722203	Paired	0.0326037	0.967826	RF	81.12	Adipose	No
SRX3293188	22661608	Paired	0.0308436	0.967281	RF	89.63	Ovary	No
SRX3293189	8615439	Paired	0.0359133	0.955128	RF	74.02	Ovary	No
SRX10485861	62783663	Paired	0.0561285	0.944681	RF	86.71	Adipose	No
SRX10485862	58244901	Paired	0.0597248	0.941153	RF	85.29	Adipose	No
SRX10485863	43143159	Paired	0.0545095	0.946694	RF	87.67	Adipose	No

SRX3973940	22922770	Paired	0.0191452	0.983508	RF	64.64	Liver	No
SRX3973941	17078957	Paired	0.0201646	0.982082	RF	70.12	Liver	No
SRX3973942	18769539	Paired	0.0256915	0.976234	RF	74.04	Liver	No
SRX3973943	16663457	Paired	0.0235573	0.979476	RF	72.01	Liver	No
SRX3973944	17783809	Paired	0.0201025	0.98181	RF	69.64	Liver	No
SRX3973945	18012198	Paired	0.0206937	0.981756	RF	73.18	Liver	No
SRX3973946	20715183	Paired	0.0184681	0.983689	RF	73.46	Liver	No
SRX3973947	17538725	Paired	0.0207466	0.981121	RF	69.82	Liver	No
SRX3973948	18093123	Paired	0.0234155	0.979252	RF	69.27	Liver	No
SRX3973949	17144627	Paired	0.0278033	0.975245	RF	60.95	Liver	No
SRX3293190	25864404	Paired	0.0302883	0.968244	RF	89.42	Ovary	No
SRX3293191	9647203	Paired	0.0338726	0.955353	RF	76.71	Ovary	No
DRX002706	200157259	Single	0.983379	NULL	FR	84.34	Heart	No
SRX3293192	20863192	Paired	0.0299787	0.968065	RF	88.75	Ovary	No
SRX8483930	43660224	Paired	0.0555638	0.939408	RF	84.66	Muscle	No
SRX3293193	11799744	Paired	0.0289258	0.963599	RF	76.85	Ovary	No
SRX8483931	31171089	Paired	0.0965324	0.901986	RF	82.87	Muscle	No
SRX3293194	23054098	Paired	0.0269587	0.971891	RF	89.58	Ovary	No
SRX8483932	53131361	Paired	0.0281452	0.973071	RF	84.1	Muscle	No
SRX3293195	11242543	Paired	0.0357823	0.947477	RF	76.06	Ovary	No
SRX8483933	49487817	Paired	0.0349218	0.96603	RF	87.04	Muscle	No
SRX3293196	22146348	Paired	0.0337288	0.964223	RF	89.58	Ovary	No
SRX8483934	47763022	Paired	0.0366044	0.964305	RF	88.49	Heart	No
SRX3293197	10711944	Paired	0.0335729	0.959467	RF	76.65	Ovary	No
SRX8483935	53919081	Paired	0.0560312	0.944499	RF	88.5	Heart	No
SRX3293198	26519678	Paired	0.0304955	0.96787	RF	88.79	Ovary	No
SRX8483936	49168365	Paired	0.0346065	0.967256	RF	88.47	Heart	No
SRX3293199	10557025	Paired	0.0303821	0.961909	RF	76.77	Ovary	No
SRX8483937	55297351	Paired	0.0598214	0.939363	RF	87.74	Heart	No
SRX8483939	46060905	Paired	0.0933283	0.904679	RF	88.37	Heart	No
SRX3973950	20990190	Paired	0.0259263	0.97715	RF	63.62	Liver	No
SRX3973951	16939546	Paired	0.0212964	0.981125	RF	77.35	Liver	No
SRX3973952	17389845	Paired	0.0196997	0.982433	RF	74.65	Liver	No
SRX3973953	17092769	Paired	0.0238564	0.978488	RF	69.16	Liver	No
SRX3973955	17027715	Paired	0.0195196	0.983607	RF	78.79	Liver	No
SRX3973956	8570160	Paired	0.0346707	0.967873	RF	61.27	Liver	No
SRX5226835	4969036	Paired	0.879397	0.146438	FR	76.09	Muscle	No
SRX3973958	19357689	Paired	0.0321843	0.969048	RF	67.41	Liver	No
SRX3973959	17933835	Paired	0.0260305	0.976687	RF	81.2	Liver	No
SRX5226838	6302861	Paired	0.840871	0.192945	FR	78.73	Whole blood	No
SRX8483940	48657982	Paired	0.0509245	0.948453	RF	87.51	Heart	No
SRX8483941	34301817	Paired	0.17739	0.820036	RF	77.29	Muscle	No
SRX8483942	44352141	Paired	0.0251448	0.976064	RF	81.72	Muscle	No
SRX8483944	42995614	Paired	0.0249763	0.97796	RF	85.37	Muscle	No
SRX8483945	48839785	Paired	0.0412686	0.959818	RF	84.76	Muscle	No
SRX8483946	41687747	Paired	0.0334004	0.968987	RF	87.12	Muscle	No
SRX8483947	38800135	Paired	0.0330573	0.967869	RF	85.99	Small intestine	No
SRX8483948	63321837	Paired	0.0319592	0.969164	RF	84.4	Small intestine	No
SRX7277000	120258834	Single	0.0231762	NULL	RF	95.04	Testis	No
SRX7277001	194447687	Single	0.0278778	NULL	RF	94.38	Testis	No
SRX3973960	21452912	Paired	0.0169189	0.985271	RF	74.29	Liver	No
SRX7277002	74157957	Single	0.0226991	NULL	RF	94.5	Testis	No
SRX3973961	16767046	Paired	0.0182843	0.983463	RF	81.54	Liver	No
SRX7277003	75600627	Single	0.0209785	NULL	RF	94.62	Testis	No
SRX3973962	17349186	Paired	0.0194037	0.982858	RF	73.31	Liver	No
SRX7277004	77337164	Single	0.0219417	NULL	RF	94.57	Testis	No
SRX3973963	18848197	Paired	0.0154307	0.985757	RF	74.05	Liver	No
SRX7277005	86113392	Single	0.0235935	NULL	RF	94.37	Testis	No

SRX3973964	17593612	Paired	0.0176281	0.984605	RF	79.66	Liver	No
SRX7277006	71609641	Single	0.0276421	NULL	RF	94.09	Testis	No
SRX3973965	19426017	Paired	0.0177313	0.984517	RF	75.31	Liver	No
SRX3973966	19799139	Paired	0.0193676	0.983013	RF	74.25	Liver	No
SRX5226845	15215772	Paired	0.860564	0.18204	FR	63.38	Heart	No
SRX3973967	16413359	Paired	0.020835	0.981494	RF	80.49	Liver	No
SRX5226846	19970039	Paired	0.835962	0.192171	FR	71.49	Heart	No
SRX3973968	20294636	Paired	0.023132	0.979619	RF	67.55	Liver	No
SRX5226847	11049616	Paired	0.843532	0.169457	FR	73.67	Whole blood	No
SRX3973969	21013311	Paired	0.0271036	0.975507	RF	74.64	Liver	No
SRX8904510	27203651	Paired	0.0224313	0.978675	RF	89.61	Ovary	No
SRX8904511	36250669	Paired	0.0181939	0.981986	RF	90.84	Ovary	No
SRX8904512	37276400	Paired	0.0184333	0.982278	RF	89.53	Ovary	No
SRX8904513	29159289	Paired	0.022673	0.98034	RF	70.38	Ovary	No
SRX8483950	56869914	Paired	0.0305362	0.970233	RF	85.31	Small intestine	No
SRX8483951	49284185	Paired	0.0403706	0.959547	RF	87.9	Muscle	No
SRX8483952	47332766	Paired	0.0866456	0.908683	RF	87.82	Muscle	No
SRX8483953	48451449	Paired	0.049196	0.950411	RF	86.54	Muscle	No
SRX8483955	35226735	Paired	0.0324186	0.968502	RF	83.48	Muscle	No
SRX8483957	52913767	Paired	0.0317714	0.969735	RF	87.73	Spleen	No
SRX8483958	66878476	Paired	0.0449	0.955999	RF	88.09	Spleen	No
SRX8483959	44770489	Paired	0.0422505	0.95843	RF	87.41	Spleen	No
SRX3973970	17941780	Paired	0.0244735	0.977732	RF	63.88	Liver	No
SRX3973971	17396867	Paired	0.0217385	0.980573	RF	72.51	Liver	No
SRX3973972	14504363	Paired	0.0261521	0.976668	RF	62.06	Liver	No
SRX5226850	15495277	Paired	0.874001	0.13992	FR	69.75	Heart	No
SRX3973973	20133167	Paired	0.021237	0.980834	RF	77.78	Liver	No
SRX3973974	17621184	Paired	0.0244002	0.978484	RF	74.46	Liver	No
SRX5962140	13849996	Single	0.0842289	NULL	RF	61.93	Heart	No
SRX3973975	17727048	Paired	0.0212733	0.98116	RF	73.17	Liver	No
SRX5226853	16279335	Paired	0.873907	0.132718	FR	72.26	Whole blood	No
SRX3973976	16989773	Paired	0.0311536	0.971896	RF	65.59	Liver	No
SRX3973977	17349564	Paired	0.0195933	0.982577	RF	75.28	Liver	No
SRX3973978	16959264	Paired	0.0236576	0.978851	RF	68.61	Liver	No
SRX5226856	15520743	Paired	0.843352	0.194654	FR	75.05	Heart	No
SRX3973979	18100800	Paired	0.022139	0.980089	RF	76.6	Liver	No
SRX5226858	20476882	Paired	0.854982	0.196383	FR	74.18	Heart	No
SRX5226859	22628612	Paired	0.855318	0.174089	FR	75.55	Heart	No
SRX3174500	17364871	Paired	0.975776	0.0248155	FR	86.44	Muscle	No
SRX3174501	16409426	Paired	0.977517	0.0232297	FR	83.44	Muscle	No
SRX3174502	25615532	Paired	0.0184141	0.982378	RF	88.02	Muscle	No
SRX3174503	21638208	Paired	0.0235984	0.976828	RF	88.14	Muscle	No
SRX3174504	18174937	Paired	0.977677	0.0229501	FR	84.74	Muscle	No
SRX3174505	16619614	Paired	0.0216619	0.978934	RF	87.93	Muscle	No
SRX3174506	18135072	Paired	0.0221418	0.978352	RF	88.57	Muscle	No
SRX3174507	18350697	Paired	0.977378	0.0234085	FR	85.12	Muscle	No
SRX3174508	20172227	Paired	0.0163459	0.984423	RF	84.7	Muscle	No
SRX3174509	29305414	Paired	0.973913	0.0269341	FR	86.77	Muscle	No
SRX8483961	31865306	Paired	0.124304	0.872923	RF	77.78	Muscle	No
SRX8483962	39096064	Paired	0.036241	0.96408	RF	85.56	Muscle	No
SRX8483963	45954708	Paired	0.181319	0.81366	RF	82.55	Muscle	No
SRX8483964	49591234	Paired	0.0376642	0.962734	RF	88.21	Muscle	No
SRX8483965	56336912	Paired	0.0439767	0.956972	RF	85.73	Muscle	No
SRX5139548	38916242	Paired	0.0791644	0.96558	RF	65.6	Whole blood	No
SRX5139549	41487503	Paired	0.081949	0.964978	RF	64.89	Whole blood	No
SRX4962158	15723259	Paired	0.0253647	0.974802	RF	88.27	Liver	No
SRX4962159	14986338	Paired	0.022326	0.977651	RF	89.97	Liver	No
SRX3973980	17188698	Paired	0.0270533	0.976378	RF	61.68	Liver	No

SRX3973981	19826901	Paired	0.0286721	0.975066	RF	65.54	Liver	No
SRX5226860	10976248	Paired	0.841857	0.176179	FR	70.48	Whole blood	No
SRX3973982	17588335	Paired	0.0195488	0.98254	RF	76.15	Liver	No
SRX5226861	13617703	Paired	0.843416	0.18048	FR	73.48	Whole blood	No
SRX3973983	16886396	Paired	0.0227867	0.979274	RF	64.6	Liver	No
SRX3973984	14731350	Paired	0.0267689	0.97624	RF	69.41	Liver	No
SRX5962151	14111908	Single	0.0769878	NULL	RF	61.13	Heart	No
SRX5962152	13708201	Single	0.0787462	NULL	RF	60.72	Heart	No
SRX5226864	15067758	Paired	0.819736	0.197224	FR	75.81	Muscle	No
SRX5226865	14603379	Paired	0.840725	0.195503	FR	75.77	Heart	No
SRX3973987	30342376	Paired	0.0232488	0.976941	RF	83.28	Testis	No
SRX3973988	31031233	Paired	0.0256549	0.974718	RF	81.03	Testis	No
SRX5962155	13606424	Single	0.0780048	NULL	RF	60.7	Heart	No
SRX5226867	17817442	Paired	0.860734	0.17565	FR	66.19	Heart	No
SRX3973989	17980380	Paired	0.0286579	0.971424	RF	88.97	Testis	No
SRX5962156	13152861	Single	0.0833319	NULL	RF	64.93	Heart	No
SRX5962158	15520268	Single	0.0785501	NULL	RF	70.45	Heart	No
SRX5962159	15095373	Single	0.07767	NULL	RF	60.78	Heart	No
SRX3174510	9940219	Paired	0.0356938	0.964646	RF	85.97	Muscle	No
SRX3174512	19846324	Paired	0.0293758	0.9711	RF	84.05	Muscle	No
SRX3174513	19730703	Paired	0.973385	0.0270248	FR	83.41	Muscle	No
SRX3174514	11009635	Paired	0.968809	0.0317223	FR	81.53	Muscle	No
SRX3174516	22635275	Paired	0.974526	0.0260487	FR	84.66	Muscle	No
SRX3174517	17167452	Paired	0.0301044	0.970315	RF	81.56	Muscle	No
SRX3174518	13682469	Paired	0.0286225	0.971691	RF	85.32	Muscle	No
SRX3174519	24120918	Paired	0.0282913	0.971934	RF	85.96	Muscle	No
SRX6793304	118173783	Paired	0.0474412	0.941033	RF	81.26	Adipose	No
SRX6793305	106317663	Paired	0.0539055	0.926755	RF	80.7	Adipose	No
SRX6793306	94208983	Paired	0.0506789	0.93473	RF	77.13	Adipose	No
SRX6793307	99311061	Paired	0.0480167	0.938852	RF	75.3	Adipose	No
SRX6793308	89426119	Paired	0.0398183	0.95113	RF	77.23	Adipose	No
SRX6793309	103640001	Paired	0.0371527	0.954981	RF	78.76	Adipose	No
SRX5139550	39536475	Paired	0.0728989	0.967534	RF	62.48	Whole blood	No
SRX5139551	38601494	Paired	0.0739418	0.965226	RF	62.86	Whole blood	No
SRX5139552	40267091	Paired	0.063192	0.965269	RF	66.13	Whole blood	No
SRX5139553	41738894	Paired	0.062134	0.968245	RF	63.72	Whole blood	No
SRX5139554	39871920	Paired	0.065772	0.945467	RF	82.32	Whole blood	No
SRX5139555	40891281	Paired	0.0673007	0.943378	RF	83.29	Whole blood	No
SRX5139556	39132312	Paired	0.0610732	0.961916	RF	67.84	Whole blood	No
SRX4962160	14542698	Paired	0.0235741	0.976631	RF	88.01	Liver	No
SRX5139557	41399870	Paired	0.0646928	0.961563	RF	66.97	Whole blood	No
SRX4962161	14135940	Paired	0.0238368	0.976226	RF	89.78	Liver	No
SRX4962162	14534742	Paired	0.0215078	0.978118	RF	90.3	Liver	No
SRX4962163	14003610	Paired	0.023677	0.976294	RF	89.88	Liver	No
SRX4962164	13522938	Paired	0.02226	0.977942	RF	90.73	Liver	No
SRX4962165	14440609	Paired	0.0232798	0.976402	RF	90.34	Liver	No
SRX4962166	17345430	Paired	0.0227193	0.977227	RF	90.79	Liver	No
SRX4962167	13411902	Paired	0.0229557	0.977131	RF	90.71	Liver	No
SRX4962168	15476523	Paired	0.0235406	0.975994	RF	90.59	Liver	No
SRX4962169	15465459	Paired	0.0234386	0.976249	RF	90.18	Liver	No
SRX3973990	17348982	Paired	0.0273675	0.972655	RF	86.82	Testis	No
SRX3973991	31006610	Paired	0.0257981	0.975047	RF	87.77	Testis	No
SRX3973992	19408546	Paired	0.0255451	0.974473	RF	86.53	Testis	No
SRX3973993	14781535	Paired	0.0280275	0.972646	RF	83.34	Testis	No
SRX3973994	18862732	Paired	0.0289874	0.97056	RF	86.14	Testis	No
SRX3973995	18889704	Paired	0.0268497	0.973097	RF	83.19	Testis	No
SRX5226874	13391976	Paired	0.894002	0.127238	FR	69	Muscle	No
SRX3973996	14869451	Paired	0.0290348	0.971518	RF	89.13	Testis	No

SRX5226875	19053521	Paired	0.911039	0.10576	FR	65.1	Muscle	No
SRX3973997	19009764	Paired	0.0277294	0.971952	RF	85.72	Testis	No
SRX3973998	15044034	Paired	0.0272815	0.973015	RF	90.52	Testis	No
SRX3973999	16152936	Paired	0.0264701	0.973736	RF	87.6	Testis	No
SRX5962166	16075127	Single	0.0731645	NULL	RF	62.01	Heart	No
SRX5226879	14246509	Paired	0.879944	0.1371	FR	76.1	Muscle	No
SRX5962167	15624937	Single	0.063317	NULL	RF	62.31	Heart	No
SRX3174520	17404233	Paired	0.979066	0.0214761	FR	85.3	Muscle	No
SRX3174521	20182020	Paired	0.0260481	0.974356	RF	86.64	Muscle	No
SRX3174522	23087596	Paired	0.975672	0.0248959	FR	85.43	Muscle	No
SRX3174523	21006629	Paired	0.972804	0.0280781	FR	84.45	Muscle	No
SRX3174524	23725766	Paired	0.0252247	0.975653	RF	83.9	Muscle	No
SRX3174525	19481632	Paired	0.976287	0.024435	FR	87.45	Muscle	No
SRX3174526	20480927	Paired	0.0240803	0.976466	RF	85.93	Muscle	No
SRX3174527	24598088	Paired	0.976388	0.0244596	FR	88.96	Muscle	No
SRX3174528	32363232	Paired	0.0213277	0.979432	RF	89.35	Muscle	No
SRX3174529	22804007	Paired	0.974604	0.02603	FR	83.53	Muscle	No
SRX5577264	30314153	Paired	0.027403	0.972657	RF	88.88	Small intestine	No
SRX5577265	23368560	Paired	0.0247354	0.97569	RF	85.13	Small intestine	No
SRX5577266	27047825	Paired	0.0265845	0.973803	RF	87.68	Small intestine	No
SRX5577267	30429643	Paired	0.0280983	0.971629	RF	87.34	Small intestine	No
SRX5577268	27524768	Paired	0.0272439	0.972894	RF	86.58	Small intestine	No
SRX5577269	28839454	Paired	0.0268547	0.973503	RF	87.19	Small intestine	No
SRX6793310	92102184	Paired	0.038191	0.958843	RF	79.82	Adipose	No
SRX6793311	88005674	Paired	0.0515888	0.928862	RF	78.61	Adipose	No
SRX6793312	100930037	Paired	0.0520428	0.932702	RF	78.44	Adipose	No
SRX6793314	87120144	Paired	0.0458386	0.941389	RF	74.33	Adipose	No
SRX10293468	5786630	Paired	0.0375744	0.960032	RF	90.5	Placenta	No
SRX10293469	8712264	Paired	0.0327294	0.966856	RF	91.19	Placenta	No
SRX4962170	14380893	Paired	0.0217724	0.977683	RF	90.66	Liver	No
SRX4962171	15393141	Paired	0.0240867	0.975388	RF	90.58	Liver	No
SRX4962172	14313108	Paired	0.0257094	0.974747	RF	90.44	Liver	No
SRX4962173	14192857	Paired	0.0223487	0.978036	RF	90.76	Liver	No
SRX4962174	14218124	Paired	0.0257962	0.974164	RF	91.46	Liver	No
SRX4962175	13433325	Paired	0.0242621	0.976316	RF	90.72	Liver	No
SRX4962176	14535839	Paired	0.023063	0.976757	RF	91.23	Liver	No
SRX4962177	14411178	Paired	0.0229778	0.977644	RF	90.63	Liver	No
SRX4962178	16643500	Paired	0.0233313	0.977137	RF	91.56	Liver	No
SRX4962179	15544255	Paired	0.0221873	0.978323	RF	91.62	Liver	No
SRX5226881	11910433	Paired	0.858473	0.199011	FR	74.4	Heart	No
SRX5226882	13680920	Paired	0.86846	0.138499	FR	74.69	Whole blood	No
SRX3174531	9198550	Paired	0.0368454	0.963777	RF	86.6	Muscle	No
SRX3174532	14666334	Paired	0.970712	0.0297191	FR	80.99	Muscle	No
SRX3174533	15122459	Paired	0.0275865	0.97293	RF	79.32	Muscle	No
SRX3174534	8251463	Paired	0.0304394	0.969887	RF	81.42	Muscle	No
SRX5577270	27004776	Paired	0.0252126	0.975283	RF	86.75	Small intestine	No
SRX3174536	14396439	Paired	0.0293504	0.97112	RF	76.33	Muscle	No
SRX5577271	23851183	Paired	0.0260598	0.97308	RF	83.52	Small intestine	No
SRX5577272	27687521	Paired	0.0240365	0.976255	RF	85.85	Small intestine	No
SRX3174538	19383464	Paired	0.0278488	0.972563	RF	78.53	Muscle	No
SRX5577273	28380925	Paired	0.02346	0.977043	RF	78.21	Small intestine	No
SRX5577274	35853348	Paired	0.023355	0.977476	RF	81.38	Small intestine	No
SRX5577275	34436908	Paired	0.0231306	0.977286	RF	85.32	Small intestine	No
SRX5577276	32064394	Paired	0.019794	0.980352	RF	81.77	Small intestine	No
SRX5577277	35878589	Paired	0.0247109	0.975998	RF	83.81	Small intestine	No
SRX5577278	36278388	Paired	0.0224314	0.977784	RF	85.07	Small intestine	No
SRX5577279	34793072	Paired	0.0237286	0.976212	RF	86.41	Small intestine	No
SRX10293470	10956907	Paired	0.0354196	0.962195	RF	90.07	Placenta	No

SRX10293471	7870982	Paired	0.0363602	0.963255	RF	89.79	Placenta	No
SRX10293472	9187853	Paired	0.033041	0.964639	RF	90.93	Placenta	No
SRX10293473	6759303	Paired	0.0359095	0.962234	RF	90.42	Placenta	No
SRX10293474	9802127	Paired	0.031796	0.967194	RF	91.25	Placenta	No
SRX10293475	7369387	Paired	0.034838	0.965218	RF	90.64	Placenta	No
SRX10293476	10984195	Paired	0.0345143	0.964417	RF	88.76	Placenta	No
SRX10293477	7935627	Paired	0.0359138	0.964152	RF	86.16	Placenta	No
SRX4962180	15442106	Paired	0.0227979	0.977237	RF	92.25	Liver	No
SRX4962181	13886063	Paired	0.0210446	0.979099	RF	92.23	Liver	No
SRX4962182	16140036	Paired	0.0228502	0.977061	RF	90.71	Liver	No
SRX4962183	15771292	Paired	0.0241676	0.9767	RF	90.94	Liver	No
SRX4962184	17448280	Paired	0.023546	0.976653	RF	91.51	Liver	No
SRX4962185	13667299	Paired	0.0246465	0.975253	RF	89.6	Liver	No
SRX4962186	14040850	Paired	0.0222972	0.978734	RF	91.18	Liver	No
SRX4962187	16152171	Paired	0.0237047	0.976313	RF	91.36	Liver	No
SRX4962188	16176816	Paired	0.0227935	0.977445	RF	91.28	Liver	No
SRX4962189	15191538	Paired	0.0220358	0.978036	RF	91.02	Liver	No
SRX3174540	19814486	Paired	0.0320846	0.967747	RF	79.34	Muscle	No
SRX3174541	41712747	Paired	0.974158	0.0259246	FR	85.43	Muscle	No
SRX3174542	34130545	Paired	0.0254254	0.97505	RF	85.38	Muscle	No
SRX3174544	32192460	Paired	0.975861	0.0249338	FR	87.3	Muscle	No
SRX3174545	16112267	Paired	0.028994	0.971324	RF	82.89	Muscle	No
SRX5577280	35728107	Paired	0.0229085	0.977661	RF	76.47	Small intestine	No
SRX3174546	14831105	Paired	0.970815	0.0298974	FR	80.72	Muscle	No
SRX5577281	33679033	Paired	0.0237598	0.97666	RF	84.01	Small intestine	No
SRX5577282	34842511	Paired	0.0237875	0.976992	RF	83.42	Small intestine	No
SRX3174548	68609524	Paired	0.972836	0.0276896	FR	80.76	Muscle	No
SRX5577283	33987345	Paired	0.0241565	0.976392	RF	85.91	Small intestine	No
SRX3174549	8991187	Paired	0.974262	0.0282566	FR	75.37	Muscle	No
SRX5577284	36191866	Paired	0.0217294	0.978916	RF	83.31	Small intestine	No
SRX5577285	33159457	Paired	0.025816	0.975048	RF	85.02	Small intestine	No
SRX5577286	36107450	Paired	0.0236742	0.976478	RF	85.2	Small intestine	No
ERX5304229	42574257	Paired	0.0274655	0.969838	RF	90.01	T cell	No
SRX5577287	33011391	Paired	0.0245857	0.975614	RF	87.57	Small intestine	No
SRX5577288	27755664	Paired	0.022173	0.97864	RF	83.91	Small intestine	No
SRX5577289	23592693	Paired	0.0255779	0.975255	RF	84.52	Small intestine	No
ERX4738404	98619995	Paired	0.0223417	0.977776	RF	90.78	Muscle	No
ERX4738405	87049891	Paired	0.0251852	0.97486	RF	89.89	Muscle	No
ERX4738406	83811687	Paired	0.0225106	0.977234	RF	88.26	Muscle	No
ERX4738407	88544151	Paired	0.021377	0.979094	RF	90.73	Muscle	No
ERX4738408	96286941	Paired	0.0236947	0.976439	RF	88.82	Muscle	No
ERX4738409	75685504	Paired	0.0239464	0.976487	RF	89.57	Muscle	No
ERX768396	62549813	Paired	0.0168695	0.98481	RF	84.25	Muscle	No
ERX768397	66032358	Paired	0.0170321	0.985164	RF	90.92	Muscle	No
ERX768398	61070277	Paired	0.0171972	0.984444	RF	90.55	Muscle	No
ERX768399	64275055	Paired	0.0188476	0.98272	RF	86.07	Muscle	No
SRX3174550	15780932	Paired	0.0307809	0.96945	RF	81.06	Muscle	No
SRX3174551	24596535	Paired	0.973223	0.0272167	FR	87.54	Muscle	No
ERX5304230	50040601	Paired	0.0271882	0.965604	RF	88.85	T cell	No
ERX5304231	46130595	Paired	0.0291499	0.961559	RF	90.09	T cell	No
SRX3174553	17957958	Paired	0.0211109	0.97965	RF	88.06	Muscle	No
ERX5304232	34107355	Paired	0.0256258	0.962797	RF	90.34	T cell	No
SRX3174554	19546564	Paired	0.978365	0.0223603	FR	86.84	Muscle	No
SRX3174555	22473515	Paired	0.0263039	0.97429	RF	88.36	Muscle	No
SRX5577290	33965662	Paired	0.0212633	0.97883	RF	84.87	Small intestine	No
ERX5304234	39552058	Paired	0.0256902	0.966471	RF	90.32	T cell	No
SRX3174556	21037151	Paired	0.975372	0.0252539	FR	85.77	Muscle	No
SRX5577291	32699744	Paired	0.024887	0.975496	RF	88.32	Small intestine	No

SRX9302854	28138212	Paired	0.0247949	0.967914	RF	89.2	Liver	No
SRX3174557	18194438	Paired	0.980071	0.0207526	FR	84.23	Muscle	No
SRX5577292	27659812	Paired	0.023658	0.977026	RF	84.51	Small intestine	No
SRX9302855	40041592	Paired	0.0335442	0.947509	RF	85.19	Liver	No
SRX3174558	16868357	Paired	0.0221684	0.978464	RF	85.36	Muscle	No
SRX5577293	27339404	Paired	0.0231147	0.977524	RF	82.98	Small intestine	No
SRX9302856	56353725	Paired	0.0255689	0.95812	RF	85.35	Liver	No
ERX5304237	47625935	Paired	0.0323998	0.959866	RF	83.29	T cell	No
SRX3174559	18558192	Paired	0.0239709	0.976755	RF	85.59	Muscle	No
SRX9302857	43935251	Paired	0.0225248	0.95118	RF	85.25	Liver	No
ERX5304238	52473243	Paired	0.0242501	0.966736	RF	87.43	T cell	No
SRX9302858	28581164	Paired	0.0209565	0.970929	RF	88.68	Liver	No
ERX5304239	30992582	Paired	0.0311379	0.917677	RF	82.56	T cell	No
ERX4738410	82148779	Paired	0.0261851	0.97322	RF	89.3	Muscle	No
SRX9302859	45231062	Paired	0.0212833	0.97197	RF	89.15	Liver	No
ERX4738411	78472973	Paired	0.0236404	0.976422	RF	91.4	Muscle	No
ERX4738412	89848607	Paired	0.0214691	0.978376	RF	89.87	Muscle	No
ERX4738413	93986195	Paired	0.02808	0.972284	RF	90.56	Muscle	No
ERX4738414	82831607	Paired	0.0220449	0.977742	RF	90.57	Muscle	No
ERX4738415	96385571	Paired	0.0265168	0.97322	RF	88.18	Muscle	No
ERX4738416	74746950	Paired	0.0230287	0.976742	RF	90.81	Muscle	No
ERX4738417	83300613	Paired	0.0241753	0.976212	RF	90.87	Muscle	No
ERX4738418	75074993	Paired	0.0222585	0.977776	RF	91.11	Muscle	No
ERX4738419	64276905	Paired	0.0226763	0.976615	RF	90.36	Muscle	No
SRX2754925	16097828	Paired	0.117123	0.870424	RF	88.83	Brain	No
SRX2754927	21987724	Paired	0.148642	0.836775	RF	88.21	Brain	No
SRX5558516	27499937	Paired	0.0255079	0.974441	RF	91.8	Brain	No
SRX5558517	28226597	Paired	0.0266187	0.97331	RF	89.02	Brain	No
SRX5558518	26850802	Paired	0.026425	0.973284	RF	91.17	Brain	No
SRX5558519	28180779	Paired	0.0259784	0.974065	RF	91.35	Brain	No
SRX3174560	23132998	Paired	0.97534	0.0253609	FR	85.92	Muscle	No
SRX3174561	12658010	Paired	0.0332799	0.966659	RF	74.63	Muscle	No
SRX3174562	17515575	Paired	0.972226	0.0286224	FR	83.79	Muscle	No
ERX5304240	40063841	Paired	0.034636	0.958111	RF	88.29	T cell	No
SRX9302860	50878531	Paired	0.0221006	0.971709	RF	89.68	Liver	No
SRX3174563	19492902	Paired	0.972934	0.0277869	FR	86.11	Muscle	No
SRX9302861	35151963	Paired	0.0193394	0.972386	RF	87.25	Liver	No
SRX3174564	28338905	Paired	0.0324076	0.96807	RF	86.85	Muscle	No
SRX9302862	28936858	Paired	0.0208561	0.976049	RF	89.86	Liver	No
SRX3174565	20325962	Paired	0.034598	0.965736	RF	88.32	Muscle	No
SRX9302863	33020703	Paired	0.0206261	0.974153	RF	91	Liver	No
SRX9302864	31067191	Paired	0.0252263	0.965966	RF	86.35	Liver	No
SRX3174567	14138580	Paired	0.969399	0.0310848	FR	81.87	Muscle	No
SRX9302865	34557137	Paired	0.0244141	0.967822	RF	90.45	Liver	No
SRX3174568	18208565	Paired	0.0252071	0.975226	RF	84.02	Muscle	No
SRX9302866	32468665	Paired	0.0237817	0.96561	RF	88.35	Liver	No
SRX3174569	16258370	Paired	0.967877	0.0325732	FR	74.74	Muscle	No
SRX9302867	29018091	Paired	0.0244104	0.971405	RF	87.51	Liver	No
SRX9302868	49966481	Paired	0.021461	0.972382	RF	89.78	Liver	No
ERX4738420	95055858	Paired	0.0211201	0.978641	RF	90.69	Muscle	No
SRX9302869	33786171	Paired	0.0288705	0.965365	RF	88.91	Liver	No
ERX4738421	94428499	Paired	0.0233269	0.976545	RF	90.29	Muscle	No
ERX4738422	112052220	Paired	0.0205586	0.980056	RF	88.97	Muscle	No
ERX4738423	97252607	Paired	0.0190742	0.981754	RF	88.68	Muscle	No
ERX4738424	118206632	Paired	0.0175347	0.984089	RF	88.23	Muscle	No
ERX4738425	76979250	Paired	0.0167535	0.984877	RF	89.2	Muscle	No
ERX4738426	105513697	Paired	0.0182107	0.983312	RF	86.62	Muscle	No
ERX4738427	72159164	Paired	0.0174023	0.983441	RF	88.13	Muscle	No

ERX4738428	114107366	Paired	0.015104	0.986613	RF	89.72	Muscle	No
ERX4738429	80962133	Paired	0.0188895	0.982427	RF	88.84	Muscle	No
SRX5124334	31130328	Paired	0.0296324	0.962734	RF	83.21	Liver	No
SRX5124335	30743706	Paired	0.0281576	0.963386	RF	83.66	Liver	No
SRX5124336	37981583	Paired	0.0347894	0.964848	RF	82.81	Liver	No
SRX5124337	36886619	Paired	0.0352782	0.961668	RF	85.97	Liver	No
ERX1532973	25946401	Paired	0.02041	0.980588	RF	86.99	Liver	No
ERX1532974	28555187	Paired	0.0186841	0.982217	RF	90.42	Liver	No
ERX1532975	27498753	Paired	0.0208861	0.979836	RF	87.29	Liver	No
ERX1532976	29829599	Paired	0.018396	0.982424	RF	90.66	Liver	No
ERX1532977	21375194	Paired	0.0231728	0.977591	RF	83.78	Liver	No
ERX1532978	26835951	Paired	0.0196916	0.981274	RF	90.4	Liver	No
SRX9002163	40185835	Paired	0.0481739	0.949815	RF	89.85	Adipose	No
SRX9002164	38840045	Paired	0.0530013	0.945073	RF	90.01	Adipose	No
SRX9002168	40021140	Paired	0.0351195	0.963513	RF	88.52	Adipose	No
SRX5558520	29257145	Paired	0.0248167	0.974971	RF	90.11	Brain	No
SRX5558521	29463602	Paired	0.0246832	0.975185	RF	90.03	Brain	No
SRX4196830	32378151	Paired	0.0319428	0.970353	RF	90.23	Heart	No
SRX5558522	26812396	Paired	0.0250136	0.97462	RF	92.17	Brain	No
SRX4196831	26806376	Paired	0.0336232	0.969064	RF	87.92	Heart	No
SRX5558523	28413628	Paired	0.0255971	0.9743	RF	90.21	Brain	No
SRX4196832	30232412	Paired	0.0343691	0.966836	RF	89.19	Heart	No
SRX5558524	28510511	Paired	0.0235471	0.975914	RF	89.13	Brain	No
SRX4196833	30326846	Paired	0.0290379	0.973004	RF	90.26	Heart	No
SRX5558525	27662217	Paired	0.0246717	0.974949	RF	91.2	Brain	No
SRX4196834	29490806	Paired	0.0336864	0.968997	RF	87.6	Heart	No
SRX5558526	35751152	Paired	0.0243467	0.975698	RF	89.77	Brain	No
SRX4196835	29200368	Paired	0.0307279	0.97121	RF	88.64	Heart	No
SRX5558527	37991666	Paired	0.0250475	0.975026	RF	88.11	Brain	No
SRX4196836	24971027	Paired	0.0327158	0.969216	RF	88.12	Heart	No
SRX5558528	38359400	Paired	0.0239577	0.976153	RF	91.05	Brain	No
SRX4196837	45605346	Paired	0.0536034	0.952173	RF	86.18	Heart	No
SRX5558529	38411273	Paired	0.0256865	0.974433	RF	89.68	Brain	No
SRX4196838	32697081	Paired	0.0284923	0.973266	RF	92.14	Heart	No
SRX4196839	31271619	Paired	0.0295405	0.972694	RF	91.5	Heart	No
SRX3174570	16934518	Paired	0.0283933	0.971965	RF	77.74	Muscle	No
SRX9302870	27988794	Paired	0.0232909	0.966213	RF	87.63	Liver	No
SRX3174573	20393094	Paired	0.029493	0.971002	RF	81.14	Muscle	No
SRX9302871	25724851	Paired	0.0233197	0.96481	RF	80.68	Liver	No
SRX3174574	16584991	Paired	0.0303055	0.970867	RF	83.76	Muscle	No
SRX9302872	30610471	Paired	0.037211	0.938151	RF	69.65	Liver	No
SRX9302873	38958530	Paired	0.0260154	0.958104	RF	86	Liver	No
SRX3174576	17149859	Paired	0.0299579	0.970713	RF	85.52	Muscle	No
SRX9302874	26313778	Paired	0.0227258	0.968825	RF	89.34	Liver	No
SRX3174577	20477707	Paired	0.0262881	0.974121	RF	83.17	Muscle	No
SRX9302875	32296739	Paired	0.0226728	0.970626	RF	90.82	Liver	No
SRX9302876	31154916	Paired	0.0241384	0.964455	RF	89.32	Liver	No
SRX9302877	31267591	Paired	0.0251915	0.958192	RF	86.58	Liver	No
SRX9302878	25750948	Paired	0.0276778	0.971623	RF	85.74	Small intestine	No
SRX9302879	24742309	Paired	0.0291615	0.961204	RF	83.57	Small intestine	No
SRX5558530	35683046	Paired	0.0239639	0.976119	RF	86.47	Brain	No
SRX4196840	25218845	Paired	0.0379307	0.964857	RF	89.62	Heart	No
SRX5558531	33991024	Paired	0.025653	0.974506	RF	87.6	Brain	No
SRX4196841	23685611	Paired	0.0323441	0.969813	RF	90.4	Heart	No
SRX5558532	35541403	Paired	0.0251243	0.97494	RF	90.64	Brain	No
SRX4196842	29749910	Paired	0.0546868	0.946477	RF	89.47	Heart	No
SRX5558533	40373969	Paired	0.0254463	0.974636	RF	90.32	Brain	No
SRX4196843	19210597	Paired	0.0338218	0.96191	RF	88.21	Heart	No

SRX5558534	33988790	Paired	0.0252114	0.974659	RF	90.4	Brain	No
SRX4196844	20058056	Paired	0.0528616	0.942162	RF	88.81	Heart	No
SRX5558535	38852315	Paired	0.025941	0.973911	RF	86.61	Brain	No
SRX4196845	18200789	Paired	0.0358901	0.952561	RF	87.79	Heart	No
SRX5558536	27834294	Paired	0.0262719	0.973705	RF	90.66	Brain	No
SRX4196846	26281280	Paired	0.0375839	0.963863	RF	87.69	Heart	No
SRX5888900	60374187	Paired	0.0225141	0.976425	RF	77.45	Lung	No
SRX5558537	26245092	Paired	0.0262654	0.973755	RF	91.57	Brain	No
SRX4196847	25141172	Paired	0.0439341	0.95754	RF	86.8	Heart	No
SRX5888901	57122794	Paired	0.0242318	0.97501	RF	83.89	Lung	No
SRX5558538	27238001	Paired	0.0255619	0.974434	RF	92.09	Brain	No
SRX4196848	25630809	Paired	0.0434496	0.958523	RF	89.46	Heart	No
SRX5888902	48071402	Paired	0.028168	0.97095	RF	85.47	Lung	No
SRX5558539	28606919	Paired	0.0268225	0.973264	RF	90.82	Brain	No
SRX4196849	23137784	Paired	0.0462373	0.954802	RF	89.44	Heart	No
SRX5888903	48758408	Paired	0.0330774	0.965939	RF	85.54	Lung	No
SRX5888904	56045201	Paired	0.031841	0.967494	RF	84.19	Lung	No
SRX5888905	60857429	Paired	0.0271067	0.972153	RF	83.42	Lung	No
SRX9302880	25276402	Paired	0.0292119	0.966676	RF	87.11	Small intestine	No
SRX9302881	29931995	Paired	0.0287468	0.966853	RF	84.25	Small intestine	No
SRX9302882	26524664	Paired	0.0286999	0.969073	RF	85.69	Small intestine	No
SRX9302883	30629199	Paired	0.0291405	0.969131	RF	84.48	Small intestine	No
SRX9302884	25960874	Paired	0.0284503	0.97101	RF	86.88	Small intestine	No
SRX9302885	31315365	Paired	0.0287473	0.970614	RF	86.27	Small intestine	No
SRX8484400	50269134	Paired	0.189098	0.804004	RF	79.72	Muscle	No
SRX9302886	24693955	Paired	0.0286815	0.970024	RF	85.84	Small intestine	No
SRX8484401	38704509	Paired	0.144893	0.852052	RF	81.12	Muscle	No
SRX9302887	28837358	Paired	0.0272873	0.971108	RF	86.24	Small intestine	No
SRX8484403	35258788	Paired	0.0350998	0.965475	RF	83.33	Muscle	No
SRX8484405	37726852	Paired	0.164645	0.832761	RF	78.04	Muscle	No
ERX3774289	53705667	Paired	0.0152249	0.985533	RF	93.81	Ovary	No
SRX8484407	41125486	Paired	0.030297	0.970377	RF	84.32	Muscle	No
SRX8484409	35844933	Paired	0.150436	0.841513	RF	69.74	Muscle	No
SRX5568599	33929705	Paired	0.021223	0.978433	RF	84.62	Small intestine	No
SRX5055425	71453618	Paired	0.0446057	0.954257	RF	87.95	Testis	No
SRX10464088	53647126	Paired	0.0306528	0.969179	RF	85.55	Macrophage	No
SRX5055426	54831329	Paired	0.0363709	0.962916	RF	84.46	Testis	No
SRX10464089	44369666	Paired	0.0319854	0.967842	RF	83.21	Macrophage	No
SRX5055427	70236577	Paired	0.0407461	0.958185	RF	87.05	Testis	No
SRX5558540	34052420	Paired	0.0246592	0.975237	RF	88.36	Brain	No
SRX4196850	25789345	Paired	0.0340605	0.967293	RF	87.89	Heart	No
SRX5558541	31081064	Paired	0.0258163	0.974225	RF	89.64	Brain	No
SRX4196851	28449681	Paired	0.0353847	0.966578	RF	89.13	Heart	No
SRX5558542	36893734	Paired	0.0238128	0.976208	RF	87.68	Brain	No
SRX4196852	23679535	Paired	0.0419297	0.960221	RF	89.23	Heart	No
SRX5558543	36084571	Paired	0.0256866	0.974485	RF	87.6	Brain	No
SRX4196853	25119875	Paired	0.0416713	0.9604	RF	89.47	Heart	No
SRX5558544	37045297	Paired	0.0251821	0.974729	RF	90.33	Brain	No
SRX4196854	22648447	Paired	0.041112	0.959866	RF	88.17	Heart	No
SRX5558545	32262936	Paired	0.0258688	0.974221	RF	90.03	Brain	No
SRX4196855	29270368	Paired	0.0382167	0.963869	RF	90.78	Heart	No
SRX4196856	32722291	Paired	0.0320804	0.969131	RF	90.25	Heart	No
SRX4196857	28505259	Paired	0.0404982	0.96062	RF	91.48	Heart	No
SRX4196858	23921046	Paired	0.0558402	0.944177	RF	89.43	Heart	No
SRX4196859	34224656	Paired	0.0340258	0.968238	RF	89.07	Heart	No
ERX3774290	61177412	Paired	0.015148	0.985664	RF	92.83	Ovary	No
ERX3774291	58891965	Paired	0.0152223	0.985598	RF	93.22	Ovary	No
ERX3774292	56386168	Paired	0.0150859	0.985729	RF	93.3	Ovary	No

ERX3774293	52765772	Paired	0.015454	0.985369	RF	93.35	Ovary	No
ERX3774294	53349594	Paired	0.0154317	0.985349	RF	93.13	Ovary	No
ERX3774295	56469199	Paired	0.01536	0.985508	RF	93.65	Ovary	No
ERX3774296	54925739	Paired	0.015639	0.98523	RF	92.93	Ovary	No
ERX3774297	59764343	Paired	0.01525	0.985609	RF	92.87	Ovary	No
ERX3774298	54981272	Paired	0.0157799	0.985074	RF	93.21	Ovary	No
SRX10464090	56734294	Paired	0.0319848	0.968014	RF	87.8	Macrophage	No
SRX10464091	52151748	Paired	0.0298815	0.969812	RF	88.34	Macrophage	No
SRX10464092	57181590	Paired	0.0288555	0.971163	RF	84.89	Macrophage	No
SRX5055430	75923579	Paired	0.0349599	0.964504	RF	85.69	Testis	No
SRX10464093	57708644	Paired	0.0294935	0.970469	RF	85.61	Macrophage	No
SRX5055431	72183799	Paired	0.0398286	0.959117	RF	84.77	Testis	No
SRX2783300	8780162	Single	0.0247978	NULL	RF	94.5	Fibroblast	No
SRX10464094	47693105	Paired	0.0309724	0.968904	RF	86.34	Macrophage	No
SRX2783301	7819279	Single	0.0261917	NULL	RF	94.45	Fibroblast	No
SRX10464095	52846536	Paired	0.0293413	0.970604	RF	85.31	Macrophage	No
SRX2783302	8151458	Single	0.0265801	NULL	RF	93.98	Fibroblast	No
SRX10464096	51890732	Paired	0.0267162	0.973147	RF	78.67	Macrophage	No
SRX2783303	11513775	Single	0.0228139	NULL	RF	94.42	Fibroblast	No
SRX2783304	8012385	Single	0.0258147	NULL	RF	94.12	Fibroblast	No
SRX2783305	7551625	Single	0.0253194	NULL	RF	93.85	Fibroblast	No
SRX2783306	7243729	Single	0.0252238	NULL	RF	93.69	Fibroblast	No
SRX2783307	13262154	Single	0.02181	NULL	RF	94.7	Fibroblast	No
SRX4196860	26793578	Paired	0.0304136	0.971068	RF	89.9	Heart	No
SRX4196861	26743182	Paired	0.0301644	0.970417	RF	89.35	Heart	No
SRX4196862	10424867	Paired	0.0369343	0.879888	RF	79.05	Heart	No
SRX4196863	25116600	Paired	0.0337468	0.966595	RF	91.25	Heart	No
SRX4196864	29324996	Paired	0.029837	0.971263	RF	91.8	Heart	No
SRX4196865	22327416	Paired	0.038511	0.959037	RF	89.24	Heart	No
ERX2262900	25046873	Single	0.022792	NULL	RF	93.49	Early embryo	No
ERX2262901	28087897	Single	0.0241291	NULL	RF	94.36	Early embryo	No
ERX2262902	29758485	Single	0.0238118	NULL	RF	92.94	Early embryo	No
ERX2262903	22652524	Single	0.0301622	NULL	RF	93.31	Early embryo	No
ERX2262904	24472845	Single	0.0279702	NULL	RF	93.92	Early embryo	No
ERX2262905	27817146	Single	0.0236287	NULL	RF	94.26	Early embryo	No
ERX2262906	25180633	Single	0.0256126	NULL	RF	93.67	Early embryo	No
ERX2262907	23880909	Single	0.0222601	NULL	RF	94.12	Early embryo	No
ERX2262908	24947719	Single	0.022448	NULL	RF	94.81	Early embryo	No
ERX2262909	27027861	Single	0.0221022	NULL	RF	90.54	Early embryo	No
ERX2262910	23894744	Single	0.027805	NULL	RF	93.73	Early embryo	No
ERX2262911	22764753	Single	0.0298788	NULL	RF	92.32	Early embryo	No
ERX2262912	25210082	Single	0.0245647	NULL	RF	94.36	Early embryo	No
ERX2262913	28644723	Single	0.0219037	NULL	RF	94.09	Early embryo	No
ERX2262914	22656147	Single	0.0212006	NULL	RF	94.35	Early embryo	No
ERX1403346	43525084	Paired	0.0439357	0.954822	RF	74.31	Liver	No
SRX8937321	30954283	Single	0.0217119	NULL	RF	92.9	Brain	No
SRX8937322	47298104	Single	0.0237186	NULL	RF	93.03	Brain	No
SRX8937323	29285505	Single	0.0225908	NULL	RF	90.56	Heart	No
SRX8937324	45119988	Single	0.0199033	NULL	RF	94.33	Kidney	No
SRX8937325	33297322	Single	0.0174437	NULL	RF	92.79	Liver	No
SRX8937326	36317472	Single	0.0162991	NULL	RF	93.3	Ovary	No
SRX8937327	41015609	Single	0.0231683	NULL	RF	92.75	Brain	No
SRX8937328	33453789	Single	0.0243666	NULL	RF	94.15	Brain	No
SRX8937329	29005106	Single	0.021881	NULL	RF	93.3	Heart	No
SRX12627739	21834756	Paired	0.126719	0.872159	RF	83.47	Brain	No
ERX2292139	16027800	Paired	0.0321362	0.968821	RF	93.47	Brain	No
SRX8937331	39039928	Single	0.0212143	NULL	RF	92.74	Kidney	No
SRX8937332	21798518	Single	0.0189067	NULL	RF	91.09	Liver	No

SRX10654846	41824072	Paired	0.0274171	0.963125	RF	88.46	Early embryo	No
SRX10654847	45003901	Paired	0.0306082	0.965915	RF	88.24	Early embryo	No
SRX10654848	56063784	Paired	0.0337834	0.960876	RF	89.76	Early embryo	No
SRX10654849	42475092	Paired	0.0378632	0.951479	RF	86.7	Early embryo	No
SRX12627740	21947177	Paired	0.127901	0.870934	RF	83.53	Brain	No
SRX12627741	22084349	Paired	0.125614	0.873012	RF	84.1	Brain	No
SRX12627742	22652653	Paired	0.108339	0.890855	RF	83.31	Brain	No
ERX3183036	15159700	Paired	0.0412184	0.95826	RF	87.61	Skin	No
SRX12627743	20134887	Paired	0.121002	0.8779	RF	85.55	Brain	No
ERX2292140	16331620	Paired	0.0322191	0.96837	RF	93.03	Brain	No
ERX3183037	28483118	Paired	0.0332063	0.966775	RF	81.32	Skin	No
SRX12627744	23316102	Paired	0.122025	0.876779	RF	84.59	Brain	No
ERX2292141	15677697	Paired	0.0322961	0.968612	RF	93.56	Brain	No
ERX3183038	24469736	Paired	0.0248186	0.975647	RF	90.42	Skin	No
ERX2292142	13902464	Paired	0.0320313	0.968502	RF	92.58	Brain	No
ERX3183039	19587953	Paired	0.0305352	0.969848	RF	90.42	Skin	No
ERX2292143	13507045	Paired	0.0319169	0.968861	RF	92.99	Brain	No
ERX2292144	13069847	Paired	0.0323727	0.968455	RF	92.99	Brain	No
ERX2292145	18830372	Paired	0.0338899	0.967324	RF	92.51	Brain	No
ERX2292146	18610047	Paired	0.0340585	0.967175	RF	92.53	Brain	No
ERX2292147	19210826	Paired	0.0344038	0.966473	RF	92.13	Brain	No
ERX2292148	10293543	Paired	0.0441388	0.957202	RF	91.05	Brain	No
ERX2292149	10521579	Paired	0.0432805	0.958203	RF	91.65	Brain	No
ERX2937932	33032483	Paired	0.0205597	0.979933	RF	91.85	Ovary	No
ERX2937933	23476998	Paired	0.0203265	0.980322	RF	91.97	Ovary	No
ERX2937934	29760492	Paired	0.0208213	0.979701	RF	90.6	Ovary	No
ERX2937935	22822535	Paired	0.0208184	0.979848	RF	91.92	Ovary	No
ERX2937936	24250216	Paired	0.0204937	0.980136	RF	91.87	Ovary	No
ERX2937937	32873371	Paired	0.019257	0.981369	RF	92.76	Ovary	No
ERX2937938	31128612	Paired	0.0192764	0.981361	RF	90.22	Ovary	No
ERX2937939	27884061	Paired	0.0200785	0.980564	RF	92.82	Ovary	No
SRX10654850	51084287	Paired	0.0231318	0.970086	RF	92.17	Early embryo	No
SRX10654853	44770044	Paired	0.0335951	0.95317	RF	81.87	Early embryo	No
SRX4561241	61331676	Paired	0.0171075	0.983207	RF	70.7	Uterus	No
SRX10654854	47893961	Paired	0.0312286	0.962116	RF	92.33	Early embryo	No
SRX4561242	61442807	Paired	0.0163345	0.983875	RF	85.47	Uterus	No
SRX10654855	40874198	Paired	0.0352571	0.954744	RF	86.21	Early embryo	No
SRX10654856	44137269	Paired	0.0403425	0.952254	RF	86.72	Early embryo	No
SRX10654857	41249737	Paired	0.0303694	0.960605	RF	89.13	Early embryo	No
SRX4561245	61699443	Paired	0.0185179	0.981064	RF	89	Uterus	No
SRX10654858	52614334	Paired	0.03372	0.956611	RF	91.69	Early embryo	No
SRX4561246	61125705	Paired	0.0181779	0.980877	RF	86.5	Uterus	No
SRX10654859	44446739	Paired	0.037005	0.961603	RF	91.45	Early embryo	No
ERX2292150	10835268	Paired	0.0429904	0.958633	RF	91.87	Brain	No
ERX2292151	23641283	Paired	0.0325245	0.968184	RF	92.13	Brain	No
ERX2292152	23092408	Paired	0.0322201	0.968836	RF	92.45	Brain	No
ERX2292153	22640669	Paired	0.0324614	0.968598	RF	92.52	Brain	No
ERX2292154	15581012	Paired	0.0308335	0.969837	RF	92.08	Brain	No
ERX2292155	14920767	Paired	0.0306385	0.970084	RF	92.43	Brain	No
ERX2292156	15015341	Paired	0.0307754	0.970141	RF	92.47	Brain	No
ERX2292157	22396087	Paired	0.0333346	0.96726	RF	92.59	Brain	No
ERX2292158	21615707	Paired	0.0329855	0.967863	RF	92.97	Brain	No
ERX2292159	21458146	Paired	0.0332822	0.967563	RF	93.03	Brain	No
ERX2937940	33022695	Paired	0.0193408	0.981207	RF	92.38	Ovary	No
ERX2937941	35576346	Paired	0.0189315	0.981593	RF	92.52	Ovary	No
ERX2937942	28270874	Paired	0.0202747	0.980307	RF	92.15	Ovary	No
ERX2937943	30082332	Paired	0.0198595	0.980681	RF	92.17	Ovary	No
ERX5200977	19201711	Paired	0.0241779	0.976487	RF	90.85	Uterus	No

ERX5200978	18182150	Paired	0.0179311	0.982641	RF	83.14	Uterus	No
ERX5200979	20492556	Paired	0.022861	0.976813	RF	90.16	Uterus	No
SRX10654860	46913321	Paired	0.0311861	0.960111	RF	89.05	Early embryo	No
SRX10654861	43582712	Paired	0.0316069	0.955163	RF	89.59	Early embryo	No
SRX10654862	41559708	Paired	0.0319842	0.960529	RF	91.83	Early embryo	No
SRX10654863	52816776	Paired	0.0372608	0.953535	RF	91.07	Early embryo	No
SRX4561251	61225690	Paired	0.0165318	0.983149	RF	83.85	Uterus	No
SRX10654864	38808797	Paired	0.0318884	0.958275	RF	88.17	Early embryo	No
SRX6885006	12122384	Paired	0.0624412	0.934679	RF	75.72	Brain	No
SRX4561252	61102272	Paired	0.0175692	0.98247	RF	75.43	Uterus	No
SRX10654865	39165281	Paired	0.0329013	0.95822	RF	86.82	Early embryo	No
SRX6885007	18300029	Paired	0.0564451	0.939116	RF	73.46	Brain	No
SRX10654866	44297628	Paired	0.0304897	0.965083	RF	86.2	Early embryo	No
SRX6885008	9915520	Paired	0.0631042	0.934138	RF	71.02	Brain	No
SRX10654867	44071288	Paired	0.0317518	0.962739	RF	89.12	Early embryo	No
SRX6885009	12791729	Paired	0.0703159	0.92625	RF	79.54	Brain	No
SRX10654868	43018631	Paired	0.0296347	0.959272	RF	89.35	Early embryo	No
SRX10654869	43183356	Paired	0.0375191	0.954238	RF	90.15	Early embryo	No
ERX2292160	18356465	Paired	0.0330567	0.96799	RF	93.74	Brain	No
ERX2292161	18492803	Paired	0.0328936	0.967982	RF	93.16	Brain	No
ERX2292162	18470488	Paired	0.0328481	0.968266	RF	93.68	Brain	No
ERX2292163	12946000	Paired	0.0365327	0.963941	RF	92.57	Brain	No
ERX2292164	12788050	Paired	0.0362875	0.964537	RF	92.89	Brain	No
ERX2292165	12775426	Paired	0.0365782	0.964239	RF	92.94	Brain	No
ERX2292166	23083779	Paired	0.0349122	0.966422	RF	92.43	Brain	No
ERX2292167	23206253	Paired	0.0352241	0.965931	RF	92.09	Brain	No
ERX2292168	22358053	Paired	0.0349704	0.966458	RF	92.51	Brain	No
ERX2292169	11525315	Paired	0.0360624	0.964925	RF	92.55	Brain	No
ERX5200980	20095776	Paired	0.0200997	0.980033	RF	75.36	Uterus	No
ERX5200981	24755693	Paired	0.0202682	0.979855	RF	72.01	Uterus	No
ERX5200982	19755514	Paired	0.01902	0.981262	RF	74.43	Uterus	No
SRX6885010	15125356	Paired	0.0665535	0.930271	RF	74.3	Brain	No
SRX6885011	14509692	Paired	0.0657304	0.930557	RF	72.89	Brain	No
SRX10654870	48178654	Paired	0.0357653	0.960363	RF	90.3	Early embryo	No
SRX6885012	14959490	Paired	0.0669346	0.92945	RF	80.52	Brain	No
SRX10654871	46178779	Paired	0.0360394	0.960096	RF	89.43	Early embryo	No
SRX6885013	10087626	Paired	0.0557536	0.941765	RF	77.94	Brain	No
SRX6885014	10227296	Paired	0.0684312	0.926422	RF	77.97	Brain	No
SRX6885015	10368977	Paired	0.0718872	0.923835	RF	69.12	Brain	No
SRX10654874	40682879	Paired	0.0295203	0.963205	RF	88.1	Early embryo	No
SRX6885016	13919475	Paired	0.0639931	0.932724	RF	78.57	Brain	No
SRX10654875	43938074	Paired	0.0517363	0.926058	RF	81.22	Early embryo	No
SRX6885017	13773619	Paired	0.0698173	0.925807	RF	81.14	Brain	No
SRX10654876	41486662	Paired	0.027264	0.969201	RF	91.19	Early embryo	No
SRX6885018	13464440	Paired	0.0665895	0.929485	RF	76.02	Brain	No
SRX10654877	41126778	Paired	0.0389625	0.955877	RF	90.4	Early embryo	No
SRX6885019	7717313	Paired	0.0778838	0.913224	RF	71.14	Brain	No
SRX10654878	42238319	Paired	0.0470527	0.943417	RF	84.24	Early embryo	No
SRX10654879	42053457	Paired	0.0318304	0.962353	RF	86.46	Early embryo	No
ERX2292170	11415871	Paired	0.0363975	0.964536	RF	92.59	Brain	No
ERX2292171	11605998	Paired	0.0365857	0.964112	RF	92.19	Brain	No
ERX2292172	18745533	Paired	0.0331841	0.967453	RF	93.25	Brain	No
ERX2292173	18329222	Paired	0.0331842	0.967632	RF	93.8	Brain	No
ERX2292174	18649908	Paired	0.0327928	0.968059	RF	93.71	Brain	No
ERX2292175	16004846	Paired	0.0366314	0.964309	RF	93.54	Brain	No
SRX6832212	22081490	Paired	0.0257225	0.97091	RF	84.19	Heart	No
ERX2292176	15953953	Paired	0.0371653	0.963573	RF	93.06	Brain	No
SRX6832213	26010910	Paired	0.0230884	0.973905	RF	83.79	Heart	No

ERX2292177	16373439	Paired	0.0367124	0.964232	RF	93.66	Brain	No
SRX6832214	25525418	Paired	0.0211908	0.979772	RF	78.81	Heart	No
ERX2292178	16372493	Paired	0.0386278	0.961993	RF	93.3	Brain	No
SRX6832215	26561001	Paired	0.0218349	0.978934	RF	77.78	Heart	No
ERX2292179	16720730	Paired	0.0379335	0.963016	RF	93.82	Brain	No
SRX6832216	29615971	Paired	0.0204605	0.980388	RF	77.57	Heart	No
SRX10348000	28662873	Single	0.0256715	NULL	RF	92.17	Skin	No
SRX6832217	25061496	Paired	0.0212119	0.979725	RF	77.49	Heart	No
SRX6832218	44439576	Paired	0.0216365	0.976588	RF	87.33	Heart	No
SRX10348002	32937189	Single	0.0239064	NULL	RF	93.62	Skin	No
SRX6832219	41151035	Paired	0.0227277	0.977023	RF	85.59	Heart	No
SRX10348003	26524038	Single	0.0257723	NULL	RF	92.79	Skin	No
SRX10348004	30480219	Single	0.0274044	NULL	RF	91.82	Skin	No
SRX10348005	32173885	Single	0.0276126	NULL	RF	92.15	Skin	No
ERX4795766	119527311	Paired	0.037235	0.962104	RF	87.86	Liver	No
SRX10348006	18828742	Single	0.0291411	NULL	RF	90.94	Skin	No
ERX4795767	111736310	Paired	0.0356461	0.962264	RF	88.42	Liver	No
SRX10348007	32663451	Single	0.0265254	NULL	RF	92.41	Skin	No
ERX4795768	102337603	Paired	0.0371427	0.959068	RF	88.43	Liver	No
SRX10348008	31176669	Single	0.0245246	NULL	RF	92.73	Skin	No
ERX4795769	115735509	Paired	0.0362887	0.964681	RF	88.02	Liver	No
SRX10348009	34945391	Single	0.0282668	NULL	RF	91.75	Skin	No
SRX6885020	16402344	Paired	0.0519549	0.945977	RF	73.01	Brain	No
SRX6885021	12793166	Paired	0.060364	0.937402	RF	70.72	Brain	No
SRX6885022	13248674	Paired	0.0598925	0.937348	RF	73.26	Brain	No
SRX10654880	40539652	Paired	0.0350447	0.961265	RF	90.03	Early embryo	No
SRX6885023	12269714	Paired	0.063008	0.933821	RF	72.44	Brain	No
SRX10654881	54770200	Paired	0.033801	0.960675	RF	92.35	Early embryo	No
SRX6885024	12296856	Paired	0.0607164	0.936012	RF	71.38	Brain	No
SRX10654882	42327194	Paired	0.0341262	0.958718	RF	90.73	Early embryo	No
SRX6885025	13745775	Paired	0.0612463	0.935473	RF	72.81	Brain	No
SRX6885026	12978429	Paired	0.0610859	0.93594	RF	74.81	Brain	No
SRX6885027	13557136	Paired	0.0512655	0.946399	RF	69.05	Brain	No
SRX10654885	41837472	Paired	0.0329291	0.962116	RF	88.1	Early embryo	No
SRX6885028	13005997	Paired	0.065056	0.930981	RF	67.25	Brain	No
SRX10654886	53284861	Paired	0.038698	0.950829	RF	87.51	Early embryo	No
SRX6885029	10747285	Paired	0.0666701	0.925601	RF	76.33	Brain	No
SRX10654887	41898956	Paired	0.0266887	0.968821	RF	89.64	Early embryo	No
SRX10654888	57820979	Paired	0.0331441	0.960634	RF	88.81	Early embryo	No
SRX10654889	43406764	Paired	0.0345965	0.96136	RF	92.08	Early embryo	No
ERX2292180	19199080	Paired	0.0376228	0.963341	RF	93.87	Brain	No
ERX2292181	22751289	Paired	0.0291681	0.971543	RF	92.5	Brain	No
ERX2292182	21358861	Paired	0.0293394	0.971613	RF	92.89	Brain	No
SRX6832220	42266380	Paired	0.0204758	0.967316	RF	83.54	Heart	No
ERX2292183	21822934	Paired	0.0291273	0.971823	RF	92.86	Brain	No
SRX8573500	39565300	Paired	0.0277433	0.973408	RF	65.29	Milk	No
SRX6832221	59182747	Paired	0.0213966	0.978915	RF	85.5	Heart	No
ERX2292184	13686526	Paired	0.0295841	0.971385	RF	93.75	Brain	No
SRX8573501	18592010	Paired	0.0302397	0.971088	RF	68.01	Milk	No
SRX6832222	34634832	Paired	0.0233482	0.975457	RF	77.94	Heart	No
ERX2292185	13058748	Paired	0.0295565	0.971039	RF	93.1	Brain	No
SRX6832223	24189663	Paired	0.0243809	0.973779	RF	85.39	Heart	No
ERX2292186	13485333	Paired	0.0295628	0.971312	RF	93.65	Brain	No
SRX6832224	30809004	Paired	0.0240054	0.975568	RF	85.71	Heart	No
ERX2292187	20937738	Paired	0.0317532	0.969181	RF	92.38	Brain	No
SRX6832225	27072685	Paired	0.0223837	0.977034	RF	85.63	Heart	No
ERX2292188	20550401	Paired	0.0315449	0.969662	RF	92.75	Brain	No
SRX8573505	25521408	Paired	0.0383637	0.961284	RF	65.42	Milk	No

SRX6832226	19498562	Paired	0.0243581	0.932132	RF	80.3	Heart	No
SRX10348010	25313247	Single	0.029191	NULL	RF	90.91	Skin	No
ERX2292189	20325959	Paired	0.0317032	0.969558	RF	92.75	Brain	No
SRX8573506	21187580	Paired	0.0261888	0.974825	RF	64.58	Milk	No
SRX6832227	21189641	Paired	0.0224263	0.966697	RF	84.92	Heart	No
SRX10348011	32828640	Single	0.028149	NULL	RF	91.35	Skin	No
SRX8573507	19744221	Paired	0.0492187	0.950986	RF	76.67	Milk	No
SRX10348012	26352533	Single	0.0298759	NULL	RF	93.21	Skin	No
SRX8573508	16639336	Paired	0.0344554	0.964477	RF	73.72	Milk	No
SRX10348013	23933411	Single	0.0287555	NULL	RF	92.96	Skin	No
SRX8573509	30809580	Paired	0.0357976	0.962078	RF	64.23	Milk	No
SRX10348014	29292769	Single	0.0283609	NULL	RF	91.09	Skin	No
SRX10348015	28827792	Single	0.0259965	NULL	RF	92.55	Skin	No
SRX10348016	24331887	Single	0.0286453	NULL	RF	92.51	Skin	No
SRX10348017	35189081	Single	0.0278633	NULL	RF	92.38	Skin	No
ERX4795778	148576752	Paired	0.0497417	0.934118	RF	90.43	Kidney	No
SRX10348018	30887709	Single	0.0249607	NULL	RF	92.56	Skin	No
ERX4795779	79000860	Paired	0.0539415	0.927498	RF	90.21	Kidney	No
SRX10348019	24981170	Single	0.0235489	NULL	RF	93.73	Skin	No
SRX4617963	43110896	Paired	0.0185824	0.979679	RF	83.87	Muscle	No
SRX4617964	42152681	Paired	0.0160615	0.985758	RF	83.03	Muscle	No
SRX4617965	42940745	Paired	0.0254833	0.973788	RF	87.14	Muscle	No
SRX4617966	43247573	Paired	0.0227066	0.977014	RF	86.21	Muscle	No
SRX4617967	43020113	Paired	0.022444	0.977124	RF	84.45	Muscle	No
SRX7438600	57773383	Paired	0.0224326	0.978635	RF	85.68	Muscle	No
ERX3441403	72936610	Paired	0.0319348	0.9676	RF	82.08	Muscle	No
SRX4617968	43045656	Paired	0.0199952	0.980287	RF	85.83	Muscle	No
SRX7438601	47946465	Paired	0.0470588	0.955029	RF	84.95	Spleen	No
SRX6885030	10618579	Paired	0.0659554	0.930823	RF	69.55	Brain	No
SRX7438602	38064113	Paired	0.0486209	0.952176	RF	86.73	Spleen	No
SRX6885031	12810909	Paired	0.0686998	0.927364	RF	69.9	Brain	No
SRX7438603	47513046	Paired	0.0212797	0.978909	RF	90.04	Liver	No
SRX10654890	41677317	Paired	0.031597	0.963407	RF	87.52	Early embryo	No
SRX6885032	11842808	Paired	0.0607968	0.935953	RF	76.35	Brain	No
SRX7438604	36486600	Paired	0.0458429	0.953017	RF	88.95	Brain	No
SRX10654891	50031987	Paired	0.0312016	0.964465	RF	90	Early embryo	No
SRX6885033	13537294	Paired	0.0682277	0.928052	RF	76.29	Brain	No
SRX7438605	49021378	Paired	0.0409318	0.958241	RF	88.79	Brain	No
SRX10654892	48311182	Paired	0.0388007	0.9526	RF	71.59	Early embryo	No
SRX6885034	10992008	Paired	0.0711364	0.925658	RF	68.15	Brain	No
SRX5098723	52003723	Paired	0.0219373	0.977726	RF	82.74	Brain	No
SRX7438606	52083962	Paired	0.0471995	0.951273	RF	87.94	Brain	No
SRX10654893	43024169	Paired	0.0325898	0.962376	RF	92.3	Early embryo	No
SRX6885035	13186743	Paired	0.0595746	0.936975	RF	74.24	Brain	No
SRX5098724	52689825	Paired	0.0388436	0.960368	RF	88.68	Brain	No
SRX7438607	44921617	Paired	0.0434671	0.955304	RF	87.3	Brain	No
SRX10654894	46019695	Paired	0.0307217	0.963804	RF	90.81	Early embryo	No
SRX6885036	9882971	Paired	0.0615968	0.935661	RF	74.04	Brain	No
SRX5098725	49483117	Paired	0.0355637	0.964101	RF	89.75	Brain	No
SRX7438608	36076938	Paired	0.0662115	0.931382	RF	86.05	Small intestine	No
SRX10654895	54650995	Paired	0.0389854	0.953643	RF	87.57	Early embryo	No
SRX6885037	12100822	Paired	0.0605533	0.936659	RF	71.73	Brain	No
SRX5098726	57141975	Paired	0.0403268	0.959097	RF	89.12	Brain	No
SRX7438609	36311891	Paired	0.0324175	0.966881	RF	88.05	Small intestine	No
SRX10654896	54335575	Paired	0.0428152	0.94316	RF	72.41	Early embryo	No
SRX6885038	13594730	Paired	0.0643286	0.932554	RF	72.26	Brain	No
SRX5098727	52363570	Paired	0.0233035	0.97642	RF	85.15	Brain	No
SRX10654897	47767153	Paired	0.0311664	0.967747	RF	90.73	Early embryo	No

SRX6885039	12746498	Paired	0.060563	0.936756	RF	73.47	Brain	No
SRX5098728	54496178	Paired	0.0469735	0.952142	RF	89.41	Brain	No
SRX10654898	42723839	Paired	0.0338574	0.949856	RF	89.02	Early embryo	No
SRX5098729	56801342	Paired	0.0453191	0.953955	RF	89.54	Brain	No
SRX10654899	44609080	Paired	0.0252644	0.967177	RF	91.1	Early embryo	No
ERX2292190	15195549	Paired	0.0350738	0.966187	RF	93.4	Brain	No
ERX2292191	14617768	Paired	0.0354427	0.965543	RF	92.78	Brain	No
ERX2292192	14854036	Paired	0.0351478	0.966118	RF	93.26	Brain	No
ERX2292193	13373494	Paired	0.0303824	0.970488	RF	93.95	Brain	No
SRX8573510	31120886	Paired	0.0294408	0.971403	RF	76.21	Milk	No
ERX2292194	13201184	Paired	0.0305533	0.970329	RF	94.01	Brain	No
SRX8573511	18431589	Paired	0.0316792	0.969411	RF	73.61	Milk	No
ERX2292195	13136068	Paired	0.03039	0.970178	RF	93.42	Brain	No
SRX8573512	19843482	Paired	0.0242048	0.976984	RF	72.27	Milk	No
ERX2292196	19233225	Paired	0.0325378	0.968423	RF	92.68	Brain	No
SRX8573513	32936676	Paired	0.0252791	0.976779	RF	74.8	Milk	No
ERX2292197	19641197	Paired	0.0327534	0.96805	RF	92.3	Brain	No
SRX8573514	19275831	Paired	0.0262936	0.975194	RF	67.21	Milk	No
ERX4795780	104746738	Paired	0.0449151	0.941353	RF	90.39	Kidney	No
ERX2292198	19500781	Paired	0.0323102	0.968664	RF	92.64	Brain	No
SRX8573515	19006561	Paired	0.0262645	0.975709	RF	72.31	Milk	No
ERX4795781	168620228	Paired	0.0438154	0.94233	RF	90.55	Kidney	No
ERX2292199	19000133	Paired	0.032225	0.968452	RF	92.14	Brain	No
SRX10348020	30193963	Single	0.0264273	NULL	RF	91.34	Skin	No
SRX8573516	41432751	Paired	0.0247935	0.97683	RF	71.38	Milk	No
ERX4795782	119696613	Paired	0.0321326	0.962834	RF	91.16	Brain	No
SRX10348021	29948897	Single	0.0279176	NULL	RF	91.48	Skin	No
SRX8573517	25491133	Paired	0.0363334	0.96425	RF	63.8	Milk	No
ERX4795783	101560885	Paired	0.0325807	0.961883	RF	90.94	Brain	No
SRX10348022	29996514	Single	0.0265932	NULL	RF	92.9	Skin	No
SRX8573518	29835372	Paired	0.0321853	0.967943	RF	60.85	Milk	No
ERX4795784	133069192	Paired	0.0329878	0.960159	RF	90.73	Brain	No
SRX10348023	26752654	Single	0.0261521	NULL	RF	92.67	Skin	No
ERX4795785	87609315	Paired	0.032925	0.962619	RF	91.13	Brain	No
SRX10348024	28846754	Single	0.0272618	NULL	RF	92.6	Skin	No
ERX4795786	115873279	Paired	0.0320702	0.963148	RF	91.19	Skin	No
SRX10348025	32981126	Single	0.0280954	NULL	RF	93.17	Skin	No
ERX4795787	113004598	Paired	0.031211	0.964173	RF	90.88	Skin	No
SRX10348026	31952377	Single	0.0261526	NULL	RF	94.04	Skin	No
ERX4795788	114297314	Paired	0.0314032	0.963796	RF	90.8	Skin	No
SRX10348027	31939161	Single	0.0262022	NULL	RF	93.6	Skin	No
ERX4795789	189319431	Paired	0.0294733	0.966519	RF	91.19	Skin	No
SRX10348028	31968681	Single	0.0274127	NULL	RF	92.91	Skin	No
SRX10348029	32394976	Single	0.0246397	NULL	RF	91.69	Skin	No
SRX8483646	48498798	Paired	0.0397674	0.960688	RF	88.55	Muscle	No
SRX8483648	34247191	Paired	0.15562	0.841628	RF	73.04	Muscle	No
SRX8483649	40216649	Paired	0.0367036	0.963726	RF	84.5	Muscle	No
SRX7438610	42028745	Paired	0.0441472	0.954205	RF	89.44	Kidney	No
SRX7438611	48599903	Paired	0.0320679	0.967989	RF	87.38	Kidney	No
SRX6885040	10608792	Paired	0.0591332	0.915156	RF	69.93	Brain	No
SRX7438612	39480030	Paired	0.0421011	0.957778	RF	89.64	Lung	No
SRX6885041	11781777	Paired	0.0635422	0.933446	RF	75.84	Brain	No
SRX5098730	56326652	Paired	0.0237367	0.976069	RF	82.98	Brain	No
SRX7438613	39941734	Paired	0.0483286	0.950777	RF	87.25	Lung	No
SRX6885042	17409067	Paired	0.0572964	0.939541	RF	74.75	Brain	No
SRX5098731	41304275	Paired	0.0565492	0.943699	RF	90.21	Brain	No
SRX7438614	42040277	Paired	0.0224743	0.97764	RF	91.12	Liver	No
SRX6885044	14901405	Paired	0.0646513	0.932938	RF	72.93	Placenta	No

SRX6885045	14780931	Paired	0.0663147	0.932247	RF	76.6		Placenta	No
SRX8573520	25811627	Paired	0.0395696	0.961278	RF	74.08		Milk	No
SRX8573521	35774535	Paired	0.0434794	0.956271	RF	65		Milk	No
SRX8573522	14325645	Paired	0.0362536	0.965477	RF	80.47		Milk	No
SRX8573523	13342499	Paired	0.0335837	0.965989	RF	71.01		Milk	No
SRX8573524	24267849	Paired	0.0314272	0.970212	RF	67.63		Milk	No
SRX8573525	21799014	Paired	0.0305606	0.971215	RF	71.2		Milk	No
SRX10348030	20244817	Single	0.0286636	NULL	RF	93.95		Skin	No
SRX8573526	11710850	Paired	0.0415632	0.960072	RF	79		Milk	No
SRX10348031	26463729	Single	0.0269064	NULL	RF	92.85		Skin	No
SRX10348032	33796287	Single	0.0288503	NULL	RF	84.71		Skin	No
SRX10348033	24282598	Single	0.0262264	NULL	RF	92.29		Skin	No
ERX4795794	102939665	Paired	0.0317056	0.967724	RF	89.13		Liver	No
SRX8573529	21489212	Paired	0.0343543	0.966761	RF	80		Milk	No
SRX10348034	27353627	Single	0.0256068	NULL	RF	92.28		Skin	No
ERX4795795	100584256	Paired	0.0326325	0.96767	RF	88.79		Liver	No
SRX10348035	27960300	Single	0.0284824	NULL	RF	91.55		Skin	No
ERX4795796	130329820	Paired	0.0307712	0.971276	RF	87.69		Liver	No
SRX10348036	25579820	Single	0.0257426	NULL	RF	93.76		Skin	No
ERX4795797	189005330	Paired	0.0326585	0.969951	RF	87.36		Liver	No
SRX10348037	31313188	Single	0.0262891	NULL	RF	92.17		Skin	No
SRX10348038	27317709	Single	0.0274039	NULL	RF	92.29		Skin	No
SRX10348039	32174892	Single	0.0270002	NULL	RF	91.83		Skin	No
SRX8483652	53814341	Paired	0.0329415	0.968079	RF	86.52		Muscle	No
SRX8483653	48366791	Paired	0.0406064	0.960691	RF	87.1		Muscle	No
SRX8483654	54112731	Paired	0.0302798	0.971575	RF	84.48		Muscle	No
SRX8483655	46566757	Paired	0.0340581	0.967566	RF	84.45		Muscle	No
SRX8483656	48682997	Paired	0.0326767	0.968865	RF	88.06		Muscle	No
SRX8483657	43285850	Paired	0.13845	0.859075	RF	83.23		Muscle	No
SRX8483658	35239723	Paired	0.033348	0.966907	RF	83.8		Muscle	No
SRX6885051	13741694	Paired	0.0616758	0.934867	RF	81.04		Brain	No
SRX6885053	16607691	Paired	0.0445177	0.953622	RF	70.73		Placenta	No
SRX7438625	45979087	Paired	0.0274456	0.972883	RF	85.12		Muscle	No
SRX6885054	11807924	Paired	0.054955	0.93864	RF	61.78		Placenta	No
SRX6885056	11899413	Paired	0.069768	0.927857	RF	70.68		Placenta	No
SRX6885058	6632251	Paired	0.0883379	0.908298	RF	64.37		Placenta	No
SRX6885059	8115577	Paired	0.0669115	0.920292	RF	81.49		Brain	No
SRX8573530	20674396	Paired	0.0481812	0.94665	RF	68.67		Milk	No
SRX8573531	22975354	Paired	0.0368603	0.963435	RF	80.79		Milk	No
SRX1307213	38339192	Paired	0.934059	0.121036	FR	62.29		Heart	No
SRX8573532	19621763	Paired	0.0387923	0.958257	RF	70.75		Milk	No
SRX8573533	18806654	Paired	0.0341166	0.967228	RF	82.88		Milk	No
SRX8573534	16821261	Paired	0.032278	0.963375	RF	67.66		Milk	No
SRX8573535	16681768	Paired	0.0557108	0.933348	RF	66.85		Milk	No
SRX1307217	39057708	Paired	0.910109	0.113919	FR	68.18		Kidney	No
SRX10348040	29336417	Single	0.0271222	NULL	RF	91.97		Skin	No
SRX8573536	28325286	Paired	0.0281995	0.972593	RF	88.11		Milk	No
SRX1307218	41344468	Paired	0.93243	0.0741872	FR	68.69		Testis	No
SRX10348041	27231234	Single	0.0279117	NULL	RF	91.21		Skin	No
SRX8573537	46139914	Paired	0.0124909	0.986384	RF	85.61		Milk	No
SRX1307219	35614609	Paired	0.914761	0.058868	FR	86.05		Ovary	No
SRX10348042	28375991	Single	0.0288506	NULL	RF	89.92		Skin	No
SRX10348043	28141342	Single	0.0290342	NULL	RF	91.99		Skin	No
SRX8573539	18283400	Paired	0.0413783	0.954686	RF	81.88		Milk	No
SRX10348044	27121471	Single	0.0280474	NULL	RF	91.67		Skin	No
SRX10348045	27204398	Single	0.0265349	NULL	RF	92.21		Skin	No
SRX10348046	31760723	Single	0.025856	NULL	RF	88.15		Skin	No
SRX10348047	31024115	Single	0.0277347	NULL	RF	92.2		Skin	No

SRX10348048	32591672	Single	0.0269569	NULL	RF	93.66	Skin	No
SRX9862406	24440197	Paired	0.166055	0.837355	RF	61.02	Heart	No
SRX10348049	21910762	Single	0.0270568	NULL	RF	92.99	Skin	No
SRX8483660	38812654	Paired	0.175854	0.820996	RF	77.61	Muscle	No
SRX8483661	31696076	Paired	0.0379593	0.962385	RF	84.56	Muscle	No
SRX8483663	35795602	Paired	0.0347149	0.966302	RF	84.68	Muscle	No
SRX8483664	36975059	Paired	0.046674	0.951231	RF	89.81	Testis	No
SRX3326478	28188178	Paired	0.0166799	0.984447	RF	91.74	Uterus	No
SRX8483665	37638948	Paired	0.0382674	0.960179	RF	91.02	Testis	No
SRX3326479	27839498	Paired	0.0163281	0.984829	RF	92.02	Uterus	No
SRX8483666	36228131	Paired	0.0432452	0.954911	RF	90.88	Testis	No
SRX8483668	42554651	Paired	0.033767	0.967073	RF	81.5	Muscle	No
SRX2506212	26858004	Paired	0.163096	0.825262	RF	87.03	Heart	No
SRX2506213	22511071	Paired	0.142728	0.847675	RF	86.75	Lung	No
SRX2506214	23622827	Paired	0.156956	0.827417	RF	86.93	Heart	No
SRX2506215	22596272	Paired	0.166888	0.823485	RF	88.88	Lung	No
SRX2506218	21590506	Paired	0.180765	0.805209	RF	82.06	Lung	No
ERX3441431	69929075	Paired	0.0260664	0.973478	RF	83.97	Muscle	No
SRX6885060	4796810	Paired	0.0901386	0.81133	RF	73.43	Brain	No
SRX6885061	6186536	Paired	0.0881528	0.893374	RF	72.58	Brain	No
SRX9371994	19010008	Paired	0.0302241	0.970021	RF	88.64	Brain	No
SRX6885062	7502035	Paired	0.0759159	0.917217	RF	76.92	Brain	No
SRX9371995	21205989	Paired	0.0312435	0.970502	RF	87.43	Brain	No
SRX9371996	25874338	Paired	0.0313207	0.970057	RF	88.12	Brain	No
SRX9371997	27194590	Paired	0.03074	0.969821	RF	85.38	Brain	No
SRX7438636	48685313	Paired	0.0230103	0.977674	RF	88.43	Muscle	No
SRX9371998	23019813	Paired	0.031894	0.969793	RF	87.29	Brain	No
SRX9371999	22593730	Paired	0.0341723	0.967536	RF	88.92	Brain	No
SRX1307221	38214959	Paired	0.927112	0.0954565	FR	60.95	Muscle	No
SRX8573540	17155689	Paired	0.0292274	0.96889	RF	80.49	Milk	No
SRX1307223	36056603	Paired	0.948652	0.0984841	FR	62.03	Muscle	No
SRX8573542	26443442	Paired	0.0540958	0.945389	RF	61.95	Milk	No
SRX8573546	35300007	Paired	0.0234547	0.976373	RF	84.48	Milk	No
SRX10348051	26736296	Single	0.0268046	NULL	RF	92.94	Skin	No
SRX8573547	24780126	Paired	0.0447307	0.953725	RF	75.53	Milk	No
SRX10348052	33715469	Single	0.0254818	NULL	RF	92.1	Skin	No
SRX8573548	28138610	Paired	0.0400159	0.958917	RF	81.49	Milk	No
SRX10348053	28739251	Single	0.0273343	NULL	RF	93.04	Skin	No
SRX8573549	13321811	Paired	0.0506534	0.952375	RF	66.34	Milk	No
SRX10348054	28744739	Single	0.0269626	NULL	RF	93.85	Skin	No
SRX10348055	27742967	Single	0.029655	NULL	RF	92.81	Skin	No
SRX10348056	25285129	Single	0.0269435	NULL	RF	92.23	Skin	No
SRX10348057	34423115	Single	0.026653	NULL	RF	91.61	Skin	No
SRX10348058	29826545	Single	0.0313111	NULL	RF	89.49	Skin	No
SRX10348059	27773453	Single	0.0294044	NULL	RF	93.45	Skin	No
SRX3326480	25935164	Paired	0.0166186	0.984423	RF	91.72	Uterus	No
SRX3326481	23939693	Paired	0.0166617	0.984385	RF	92.05	Uterus	No
SRX3326482	24982696	Paired	0.0167766	0.984268	RF	91.34	Uterus	No
SRX3326483	26220095	Paired	0.0165508	0.984497	RF	91.71	Uterus	No
SRX8483670	47741448	Paired	0.188688	0.808466	RF	79.23	Muscle	No
SRX8483672	39964415	Paired	0.159204	0.83908	RF	78.54	Muscle	No
SRX8483676	38350081	Paired	0.0275289	0.973044	RF	79.7	Muscle	No
SRX8483678	52255881	Paired	0.0357983	0.964634	RF	84.86	Muscle	No
SRX8483679	48347363	Paired	0.0320938	0.969296	RF	86.89	Muscle	No
SRX9383754	98378347	Paired	0.0938245	0.905894	RF	89.8	Adipose	No
SRX2506229	22562206	Paired	0.155773	0.832202	RF	88.59	Lung	No
SRX7438647	31945736	Paired	0.0553745	0.942922	RF	68.81	Spleen	No
SRX3538558	105686608	Paired	0.0258551	0.94873	RF	67.68	IPEC-J2	No

SRX3538559	109790592	Paired	0.0183573	0.984051	RF	85.42	IPEC-J2	No
SRX7577433	47382863	Paired	0.122351	0.858576	RF	82.67	Pancreas	No
SRX7577434	43817614	Paired	0.129269	0.851049	RF	81.97	Pancreas	No
SRX7577436	66994997	Paired	0.124097	0.859411	RF	77.1	Pancreas	No
SRX7577438	45736806	Paired	0.153465	0.854232	RF	63.68	Pancreas	No
SRX8573550	12905879	Paired	0.0406982	0.958707	RF	67.75	Milk	No
SRX7577439	40341455	Paired	0.126686	0.863048	RF	79.44	Pancreas	No
SRX8573551	13157967	Paired	0.0541085	0.943508	RF	79.18	Milk	No
SRX8573552	14800483	Paired	0.0252145	0.969898	RF	91.92	Milk	No
SRX8573554	14406009	Paired	0.0719865	0.918343	RF	87.76	Milk	No
SRX8573555	16347400	Paired	0.026301	0.972916	RF	68.15	Milk	No
SRX10348060	30580766	Single	0.0266847	NULL	RF	93.03	Skin	No
SRX8573556	17835197	Paired	0.0381082	0.961512	RF	71.16	Milk	No
SRX10348061	24270693	Single	0.0303262	NULL	RF	92.64	Skin	No
SRX8573557	20511696	Paired	0.0453521	0.950321	RF	72.63	Milk	No
SRX10348062	28128638	Single	0.0296464	NULL	RF	93.33	Skin	No
SRX8573558	26001682	Paired	0.0288277	0.969518	RF	71.85	Milk	No
SRX10348063	30338666	Single	0.0269724	NULL	RF	93.05	Skin	No
SRX8573559	21519447	Paired	0.027455	0.965264	RF	62.37	Milk	No
SRX10348064	31690397	Single	0.0294057	NULL	RF	93.07	Skin	No
SRX10348065	28101179	Single	0.0297499	NULL	RF	93.37	Skin	No
SRX10348066	29301329	Single	0.0311746	NULL	RF	93.24	Skin	No
SRX10348067	25919536	Single	0.0297125	NULL	RF	88.14	Skin	No
SRX10348068	28604492	Single	0.0287719	NULL	RF	93.02	Skin	No
SRX10348069	26731779	Single	0.0285013	NULL	RF	93.52	Skin	No
SRX8483680	58606168	Paired	0.0350318	0.964788	RF	85.09	Adipose	No
SRX8483681	51083042	Paired	0.0326517	0.968213	RF	86.49	Adipose	No
SRX8483682	57709438	Paired	0.0371715	0.962491	RF	86.76	Adipose	No
SRX8483683	53546128	Paired	0.0369209	0.962748	RF	89.3	Uterus	No
SRX8483684	40305673	Paired	0.032192	0.967886	RF	85.15	Uterus	No
SRX8483686	43667459	Paired	0.0344736	0.965663	RF	88.52	Uterus	No
SRX8483687	30676215	Paired	0.177141	0.81946	RF	81.68	Muscle	No
SRX2506231	26596471	Paired	0.141711	0.842974	RF	87.66	Heart	No
SRX8483688	46139130	Paired	0.148751	0.846517	RF	82.26	Muscle	No
SRX2506232	24425763	Paired	0.186125	0.802909	RF	89.37	Lung	No
SRX8483689	50227678	Paired	0.139881	0.857934	RF	83.44	Muscle	No
SRX2506234	28885272	Paired	0.179473	0.803668	RF	85.35	Heart	No
SRX2506235	20242109	Paired	0.14695	0.841652	RF	88.97	Lung	No
SRX4581400	47631996	Paired	0.0155477	0.985031	RF	62.92	Muscle	No
SRX4581401	37476045	Paired	0.0217424	0.978131	RF	72.64	Muscle	No
SRX4581402	45799888	Paired	0.0248877	0.975965	RF	77.96	Muscle	No
SRX8213979	80303384	Paired	0.0267581	0.970604	RF	75.77	Liver	No
SRX4581403	43523684	Paired	0.0245836	0.975956	RF	69.36	Muscle	No
SRX4581404	49435880	Paired	0.0184694	0.981085	RF	65.19	Muscle	No
SRX4581405	47976698	Paired	0.0235927	0.976871	RF	69.68	Muscle	No
SRX4581406	52542349	Paired	0.0240622	0.975777	RF	72.25	Muscle	No
ERX3441450	125968745	Paired	0.0663508	0.92907	RF	76.95	Testis	No
SRX4581407	44636290	Paired	0.0248396	0.975242	RF	71.93	Muscle	No
SRX4581408	54706878	Paired	0.0250907	0.975158	RF	79.78	Muscle	No
SRX4581409	49506535	Paired	0.0276628	0.972842	RF	80.96	Muscle	No
SRX7438658	50208485	Paired	0.038421	0.96095	RF	90.6	Spleen	No
SRX7577440	40157950	Paired	0.11624	0.878424	RF	64.81	Pancreas	No
SRX7577441	56027111	Paired	0.112099	0.879338	RF	67.86	Pancreas	No
ERX2259428	50971494	Paired	0.039103	0.960751	RF	64.98	Adipose	No
SRX7577442	47209690	Paired	0.123187	0.870668	RF	67.66	Pancreas	No
SRX7577443	43787364	Paired	0.114637	0.873641	RF	67.45	Pancreas	No
SRX7577444	44059986	Paired	0.135409	0.858583	RF	79.75	Pancreas	No
SRX7577445	38359705	Paired	0.137975	0.860047	RF	74.57	Pancreas	No

SRX7577446	45400135	Paired	0.146364	0.843463	RF	78.99	Pancreas	No
SRX7577447	38065249	Paired	0.119493	0.864363	RF	80.17	Pancreas	No
SRX7577448	34098207	Paired	0.132334	0.855047	RF	79.41	Pancreas	No
SRX8573560	16884066	Paired	0.0615126	0.93125	RF	81.61	Milk	No
SRX7577449	55803344	Paired	0.114833	0.871447	RF	76.66	Pancreas	No
SRX8573561	22537514	Paired	0.0284759	0.967194	RF	72.65	Milk	No
SRX8573565	11963479	Paired	0.0341301	0.966581	RF	85.72	Milk	No
SRX10348070	27304123	Single	0.0324654	NULL	RF	91.81	Skin	No
SRX10348071	30865229	Single	0.0277431	NULL	RF	92.69	Skin	No
SRX8573567	17172032	Paired	0.0395148	0.961741	RF	84.33	Milk	No
SRX10348072	23863747	Single	0.0309298	NULL	RF	93.03	Skin	No
SRX8573568	13381298	Paired	0.0383329	0.963093	RF	73.19	Milk	No
SRX10348073	35384446	Single	0.0322221	NULL	RF	93.45	Skin	No
SRX8573569	21355682	Paired	0.03538	0.965522	RF	62.73	Milk	No
SRX10348075	27990294	Single	0.0275115	NULL	RF	92.42	Skin	No
SRX10348076	27827773	Single	0.0217126	NULL	RF	93.88	Skin	No
SRX10348077	28441684	Single	0.0279645	NULL	RF	89.71	Skin	No
SRX10348078	25068921	Single	0.0301967	NULL	RF	92.83	Skin	No
SRX10348079	28998712	Single	0.0303429	NULL	RF	91.83	Skin	No
SRX8483690	28443217	Paired	0.19175	0.805408	RF	78.59	Muscle	No
SRX8483691	53834832	Paired	0.0323304	0.968747	RF	83.15	Muscle	No
SRX8483692	40237815	Paired	0.191973	0.805961	RF	78.78	Muscle	No
SRX8483695	35320262	Paired	0.0360415	0.963836	RF	84	Muscle	No
SRX3347449	38886826	Paired	0.0559384	0.949835	RF	80.75	Spleen	No
SRX8213980	69931622	Paired	0.0238271	0.977083	RF	82.76	Liver	No
SRX8213981	86428694	Paired	0.0203466	0.980861	RF	88.15	Liver	No
SRX8213982	85476740	Paired	0.0230647	0.977083	RF	79.37	Liver	No
SRX8213983	80144198	Paired	0.0219566	0.978818	RF	81.81	Liver	No
SRX8213984	64172929	Paired	0.0250164	0.975536	RF	90.78	Liver	No
SRX8213985	83421888	Paired	0.0232313	0.977456	RF	88.3	Liver	No
SRX4581410	42269970	Paired	0.0310376	0.968958	RF	82.25	Muscle	No
SRX8213986	60275416	Paired	0.0215629	0.979259	RF	90.74	Liver	No
SRX4581411	50354287	Paired	0.0249668	0.975284	RF	81.06	Muscle	No
SRX8213987	64960850	Paired	0.0219797	0.979136	RF	89.3	Liver	No
SRX4581412	49779038	Paired	0.0252354	0.974816	RF	81.01	Muscle	No
SRX8213988	78051843	Paired	0.0183148	0.982629	RF	89.35	Liver	No
SRX4581413	53569911	Paired	0.0309322	0.969051	RF	79.12	Muscle	No
SRX4581415	53268756	Paired	0.028789	0.97121	RF	75.01	Muscle	No
SRX4581416	42991850	Paired	0.027789	0.971992	RF	80.39	Muscle	No
SRX4581417	49693682	Paired	0.024994	0.975136	RF	82.18	Muscle	No
SRX4581418	49170583	Paired	0.0247867	0.975251	RF	80.83	Muscle	No
SRX4581419	49681094	Paired	0.0238575	0.976196	RF	81.06	Muscle	No
SRX7438669	42317967	Paired	0.030796	0.968894	RF	81.02	Adipose	No
ERX2259430	57344717	Paired	0.0392446	0.96047	RF	61.97	Adipose	No
ERX2259431	42447019	Paired	0.0337368	0.966011	RF	61.04	Adipose	No
ERX2259432	84452390	Paired	0.0456848	0.953394	RF	65.02	Adipose	No
ERX2259433	40112122	Paired	0.034398	0.965745	RF	61.51	Adipose	No
ERX2259434	55492140	Paired	0.0448292	0.954472	RF	65.47	Adipose	No
ERX2259435	46878646	Paired	0.056678	0.942386	RF	64.16	Adipose	No
SRX7577450	65082735	Paired	0.120922	0.867908	RF	78.15	Pancreas	No
ERX2259436	47494837	Paired	0.0325388	0.967159	RF	63.74	Adipose	No
SRX7577451	42465336	Paired	0.119522	0.868564	RF	78.27	Pancreas	No
SRX7577452	37412325	Paired	0.122098	0.867862	RF	77.39	Pancreas	No
SRX7577453	36070370	Paired	0.142208	0.844054	RF	75.46	Pancreas	No
ERX2259439	50574532	Paired	0.0371918	0.962866	RF	61.54	Adipose	No
SRX7577454	30657071	Paired	0.129559	0.854131	RF	73.43	Pancreas	No
SRX7577455	37920730	Paired	0.114532	0.873084	RF	74.78	Pancreas	No
SRX7577456	37744710	Paired	0.143708	0.843748	RF	68.02	Pancreas	No

SRX7577457	41834574	Paired	0.145978	0.846539	RF	66.72	Pancreas	No
SRX7577458	40134064	Paired	0.139939	0.85391	RF	68.2	Pancreas	No
SRX8573570	11819575	Paired	0.0373546	0.964819	RF	75.19	Milk	No
SRX7577459	37023469	Paired	0.135443	0.859747	RF	78.88	Pancreas	No
SRX8573571	27752128	Paired	0.0342291	0.967391	RF	78.38	Milk	No
SRX8573572	19828348	Paired	0.0337615	0.967608	RF	78.21	Milk	No
SRX8573573	17711011	Paired	0.030545	0.970466	RF	67.59	Milk	No
SRX8573574	17573638	Paired	0.0478439	0.952031	RF	77.47	Milk	No
SRX8573575	38955090	Paired	0.0270145	0.976587	RF	82.87	Milk	No
SRX3347450	40359648	Paired	0.0643262	0.941966	RF	81.55	Spleen	No
SRX3347451	33188438	Paired	0.0652317	0.940299	RF	84.76	Spleen	No
SRX3347452	39712673	Paired	0.0558107	0.947471	RF	87.82	Spleen	No
SRX3347453	38592806	Paired	0.049415	0.954777	RF	87.85	Spleen	No
SRX3347454	40670227	Paired	0.0448359	0.954924	RF	88.46	Spleen	No
SRX3347455	43267958	Paired	0.0514677	0.958882	RF	86.64	Spleen	No
SRX3347456	33023786	Paired	0.0427121	0.956749	RF	88.7	Spleen	No
SRX3347457	39473288	Paired	0.0532145	0.95663	RF	87.09	Spleen	No
SRX3347458	35669351	Paired	0.0198058	0.981124	RF	74.15	Spleen	No
SRX3347459	34699649	Paired	0.0246906	0.977291	RF	77.2	Spleen	No
SRX4581420	50907502	Paired	0.022554	0.978232	RF	80.25	Muscle	No
SRX4581421	51956989	Paired	0.0257613	0.97405	RF	79.88	Muscle	No
SRX4581422	51881859	Paired	0.0239832	0.976533	RF	78.08	Muscle	No
SRX4581423	54282084	Paired	0.0272814	0.973349	RF	82	Muscle	No
SRX4581424	47431234	Paired	0.0232958	0.977316	RF	76.96	Muscle	No
SRX4581425	53523784	Paired	0.0254918	0.975213	RF	82.39	Muscle	No
SRX4581426	51111227	Paired	0.0255179	0.975171	RF	80.58	Muscle	No
SRX4581427	47818663	Paired	0.0245531	0.975756	RF	78.33	Muscle	No
SRX7438670	39983121	Paired	0.0304616	0.969268	RF	85.36	Adipose	No
SRX4581428	56067001	Paired	0.02437	0.976011	RF	81.93	Muscle	No
SRX7438671	34576189	Paired	0.0302724	0.969772	RF	84.02	Adipose	No
SRX4581429	52988079	Paired	0.0316577	0.96842	RF	83.13	Muscle	No
ERX2259440	44979230	Paired	0.0352628	0.964042	RF	68.63	Adipose	No
ERX2259441	49883571	Paired	0.0377927	0.961449	RF	65.64	Adipose	No
SRX11632200	7896062	Paired	0.0270629	0.973814	RF	86.61	Small intestine	No
SRX11632201	10819527	Paired	0.0245689	0.9762	RF	88.63	Small intestine	No
SRX4243191	52754033	Paired	0.0487407	0.952453	RF	84.98	Small intestine	No
ERX2259443	49617336	Paired	0.0407154	0.959119	RF	60.73	Adipose	No
SRX11632202	9309001	Paired	0.0247837	0.976236	RF	87.82	Small intestine	No
SRX4243192	69419568	Paired	0.0364927	0.964123	RF	83.82	Small intestine	No
SRX11632203	9420363	Paired	0.0253518	0.975821	RF	85.68	Small intestine	No
SRX4243193	66199090	Paired	0.936347	0.0631466	FR	82.8	Small intestine	No
SRX7577460	37912314	Paired	0.131225	0.866883	RF	76.05	Pancreas	No
SRX11632205	9221958	Paired	0.0268708	0.973891	RF	87.31	Small intestine	No
SRX7577461	39578051	Paired	0.122514	0.863662	RF	78.55	Pancreas	No
SRX11632206	9120182	Paired	0.0251158	0.975766	RF	87.42	Small intestine	No
SRX7577462	39994961	Paired	0.131543	0.854926	RF	79.86	Pancreas	No
SRX11632207	8950569	Paired	0.0256588	0.975104	RF	91.44	Small intestine	No
SRX7577463	37874712	Paired	0.121505	0.86671	RF	78.91	Pancreas	No
SRX11632208	5086187	Paired	0.0298972	0.971096	RF	87.32	Small intestine	No
SRX11632209	12817858	Paired	0.0237369	0.97713	RF	86.9	Small intestine	No
SRX7577464	62245659	Paired	0.119638	0.867655	RF	77.14	Pancreas	No
SRX7577465	57662005	Paired	0.11305	0.87716	RF	77.82	Pancreas	No
SRX7577466	37677904	Paired	0.159411	0.826053	RF	79.47	Pancreas	No
SRX7577467	38278962	Paired	0.146429	0.842735	RF	78.31	Pancreas	No
SRX7577468	40123358	Paired	0.143493	0.842194	RF	74.22	Pancreas	No
SRX7577469	58486559	Paired	0.130374	0.853233	RF	73.94	Pancreas	No
SRX3347460	32173674	Paired	0.0215464	0.980124	RF	74.58	Spleen	No
SRX3347461	52913767	Paired	0.0317655	0.969739	RF	87.73	Spleen	No

SRX3347462	66878476	Paired	0.044898	0.955978	RF	88.09	Spleen	No
SRX3347463	44770489	Paired	0.0422801	0.958403	RF	87.41	Spleen	No
SRX3347464	33526716	Paired	0.0234548	0.977372	RF	76.29	Spleen	No
SRX3347465	32335676	Paired	0.0259313	0.977374	RF	80.13	Spleen	No
SRX3347466	32190352	Paired	0.0287125	0.977013	RF	81.87	Spleen	No
SRX2506267	19524018	Paired	0.136708	0.851438	RF	88.59	Kidney	No
SRX2506268	24036114	Paired	0.129751	0.859804	RF	88.69	Kidney	No
SRX2506269	18487884	Paired	0.166533	0.822747	RF	87	Kidney	No
SRX4581430	49384852	Paired	0.025242	0.975544	RF	83.71	Muscle	No
SRX4581432	48124035	Paired	0.0259979	0.974223	RF	81.95	Muscle	No
SRX4581433	52233863	Paired	0.0330184	0.967519	RF	87.54	Muscle	No
SRX4581434	47660532	Paired	0.0382897	0.962123	RF	87.91	Muscle	No
SRX4581435	52015127	Paired	0.0372716	0.96335	RF	86.94	Muscle	No
SRX4581436	47817636	Paired	0.0249524	0.975674	RF	81.85	Muscle	No
SRX7438682	35902152	Paired	0.0341425	0.965128	RF	75.75	Adipose	No
SRX11632210	11379450	Paired	0.0249952	0.976408	RF	84.28	Small intestine	No
SRX5283393	22057762	Paired	0.0263052	0.97459	RF	89.83	Skin	No
SRX11632212	8369173	Paired	0.0272203	0.973694	RF	87.92	Small intestine	No
SRX5283394	28391165	Paired	0.0273019	0.973545	RF	86.9	Skin	No
SRX11632213	9694616	Paired	0.0250089	0.975989	RF	87.95	Small intestine	No
SRX5283395	20650397	Paired	0.0287728	0.971781	RF	89.75	Skin	No
SRX11632214	8994456	Paired	0.0246157	0.976331	RF	88.84	Small intestine	No
SRX5283396	25846143	Paired	0.0337806	0.967408	RF	87.71	Skin	No
SRX11632215	11059471	Paired	0.0245915	0.976363	RF	87.99	Small intestine	No
SRX5283397	24278730	Paired	0.0294074	0.971274	RF	89.22	Skin	No
SRX11632216	12260120	Paired	0.0279338	0.973108	RF	84.08	Small intestine	No
SRX2220518	38604984	Paired	0.0276807	0.97112	RF	84.04	Muscle	No
SRX5283398	22766102	Paired	0.0280749	0.972753	RF	89.97	Skin	No
SRX11632218	6877787	Paired	0.0300221	0.970915	RF	86.81	Small intestine	No
SRX11632219	20708274	Paired	0.0216436	0.979512	RF	87.39	Small intestine	No
SRX4740244	34085569	Paired	0.0252469	0.973728	RF	90.84	Adipose	No
SRX4740245	38447820	Paired	0.0277142	0.972591	RF	89.84	Lung	No
SRX4740246	34498063	Paired	0.0228499	0.977275	RF	91.1	Liver	No
SRX4740247	36192749	Paired	0.0334756	0.967258	RF	89.66	Heart	No
SRX4740248	36277368	Paired	0.02873	0.972439	RF	88.58	Heart	No
SRX4740249	34069270	Paired	0.0291508	0.972013	RF	90.75	Heart	No
SRX3677842	42161677	Paired	0.0275537	0.971889	RF	85.41	Placenta	No
SRX3677843	50601280	Paired	0.0235954	0.975859	RF	84.64	Placenta	No
SRX3677844	50161764	Paired	0.0251442	0.975	RF	82.56	Placenta	No
SRX3677845	58246770	Paired	0.0254698	0.973822	RF	86.45	Placenta	No
SRX3677846	45778561	Paired	0.0258954	0.972624	RF	76.71	Placenta	No
SRX3971090	25658269	Single	0.0248384	NULL	RF	94.47	Embryo	No
SRX3677847	50792555	Paired	0.026574	0.972803	RF	85.61	Placenta	No
SRX3971091	22110862	Single	0.0223859	NULL	RF	95.84	Embryo	No
SRX3677848	51761614	Paired	0.0428146	0.956746	RF	84.28	Placenta	No
SRX3971092	36247428	Single	0.0203807	NULL	RF	95.95	Embryo	No
SRX3677849	56935978	Paired	0.0427717	0.954797	RF	82.11	Placenta	No
SRX3971093	21498096	Single	0.0219197	NULL	RF	95.87	Embryo	No
SRX3971094	18253739	Single	0.0225716	NULL	RF	94.72	Embryo	No
SRX3971095	40257862	Single	0.0214958	NULL	RF	95.78	Embryo	No
SRX3971096	30455586	Single	0.0215073	NULL	RF	95.61	Embryo	No
SRX3971097	19284832	Single	0.0220195	NULL	RF	95.36	Embryo	No
SRX3971098	22214888	Single	0.0221881	NULL	RF	95.5	Embryo	No
SRX3971099	31374374	Single	0.0198849	NULL	RF	95.33	Embryo	No
SRX7438693	60214964	Paired	0.0307528	0.969557	RF	89.94	Heart	No
SRX4740250	34067601	Paired	0.0264689	0.973236	RF	89.04	Lung	No
SRX4740251	34064262	Paired	0.0303396	0.969725	RF	90.26	Lung	No
SRX4740252	38072234	Paired	0.0206554	0.97954	RF	91.98	Liver	No

SRX4740253	31916784	Paired	0.0239421	0.975928	RF	89.52	Liver	No
SRX4740256	41257126	Paired	0.0248097	0.974557	RF	92.68	Ovary	No
SRX4740258	43245908	Paired	0.0303616	0.969024	RF	92.13	Ovary	No
SRX4740259	32973641	Paired	0.029138	0.968319	RF	89.74	Ovary	No
SRX3677850	51121749	Paired	0.0401323	0.959601	RF	86.89	Placenta	No
SRX3677851	55816919	Paired	0.0333995	0.966195	RF	78.02	Placenta	No
SRX7730219	19766049	Paired	0.0280809	0.971201	RF	87.86	Heart	No
SRX4740260	51531731	Paired	0.0254133	0.973493	RF	91.72	Brain	No
SRX4740261	39056656	Paired	0.034453	0.964044	RF	91.01	Brain	No
SRX4740262	31454677	Paired	0.0263518	0.971856	RF	91.16	Brain	No
SRX4740263	40040846	Paired	0.0200749	0.980295	RF	91.16	Muscle	No
SRX4740264	37140281	Paired	0.0290319	0.971607	RF	90.44	Muscle	No
SRX4740265	35059612	Paired	0.0195522	0.980678	RF	89.91	Muscle	No
SRX4740266	34944000	Paired	0.0238644	0.976121	RF	92.19	Adipose	No
SRX4740267	37221505	Paired	0.0254316	0.974676	RF	92.15	Adipose	No
SRX7730220	25865664	Paired	0.0248753	0.974822	RF	89.57	Heart	No
SRX7730221	25688509	Paired	0.0279066	0.971778	RF	87.98	Heart	No
SRX7730222	21863965	Paired	0.024914	0.974056	RF	85.57	Heart	No
SRX7730223	22022124	Paired	0.0240867	0.974485	RF	87.78	Heart	No
SRX4059000	4521704	Paired	0.0457685	0.954134	RF	77.09	Testis	No
SRX7730224	21795931	Paired	0.0255487	0.974	RF	89.23	Heart	No
SRX4059001	5283264	Paired	0.0375613	0.961909	RF	81.02	Testis	No
SRX7730225	27444128	Paired	0.0231874	0.97734	RF	87.8	Heart	No
SRX4059002	6567665	Paired	0.0367958	0.963156	RF	76.67	Testis	No
SRX7730226	23202426	Paired	0.0262569	0.971804	RF	87.27	Heart	No
SRX4059003	22133528	Paired	0.036587	0.965324	RF	91.49	Testis	No
SRX7730227	24736039	Paired	0.0244632	0.975092	RF	88.14	Heart	No
SRX4059004	3767917	Paired	0.0517715	0.949897	RF	74.94	Testis	No
SRX4059005	33998286	Paired	0.0290209	0.971601	RF	92.66	Testis	No
SRX4059006	5985850	Paired	0.0388947	0.960942	RF	77.52	Testis	No
SRX4059007	5978790	Paired	0.0358125	0.965352	RF	76.73	Testis	No
SRX2506297	17186958	Paired	0.178219	0.81747	RF	88.66	Liver	No
SRX4059008	17045126	Paired	0.0310418	0.971232	RF	91.78	Testis	No
SRX2506298	18784561	Paired	0.188323	0.806148	RF	83.43	Spleen	No
SRX4059009	8599182	Paired	0.0323074	0.967992	RF	80.53	Testis	No
SRX4714931	13694270	Paired	0.0194735	0.981146	RF	86.69	Uterus	No
SRX4714932	14429042	Paired	0.0197082	0.981064	RF	88.6	Uterus	No
SRX4714933	10201695	Paired	0.0344016	0.96645	RF	89.49	Placenta	No
SRX4714934	16004879	Paired	0.0329395	0.967864	RF	89.1	Placenta	No
SRX4714935	13406346	Paired	0.020148	0.980283	RF	88.28	Uterus	No
SRX4714936	16600597	Paired	0.0224241	0.977932	RF	88.43	Uterus	No
SRX4714937	19025474	Paired	0.0318656	0.968965	RF	88.7	Placenta	No
SRX4714938	16865948	Paired	0.0334458	0.967558	RF	88.8	Placenta	No
SRX4714939	20432915	Paired	0.0170541	0.983497	RF	88.14	Uterus	No
SRX12192900	28921477	Paired	0.0287943	0.970177	RF	91.08	Brain	No
SRX12192901	32205866	Paired	0.0303626	0.968596	RF	91.24	Brain	No
SRX12192902	27763365	Paired	0.0288054	0.970555	RF	92.1	Brain	No
SRX12192903	28239932	Paired	0.0297517	0.970104	RF	93.07	Brain	No
SRX12192904	28161878	Paired	0.0322195	0.964096	RF	91.03	Brain	No
SRX12192905	26129460	Paired	0.0314401	0.967917	RF	91.45	Brain	No
SRX12192906	26482149	Paired	0.0290419	0.970914	RF	92.16	Brain	No
SRX12192907	27886964	Paired	0.0305897	0.969033	RF	93.25	Brain	No
ERX4488159	28873506	Paired	0.0305966	0.956794	RF	92.54	Lung	No
SRX4059010	6598926	Paired	0.0358618	0.964141	RF	77.94	Testis	No
SRX4059011	5345234	Paired	0.0387085	0.961028	RF	89.95	Testis	No
SRX4059012	8327085	Paired	0.0301255	0.970186	RF	83.23	Testis	No
SRX4059013	7759702	Paired	0.037229	0.962645	RF	78.47	Testis	No
SRX4059014	24786617	Paired	0.0307734	0.969575	RF	93.79	Testis	No

SRX4059015	5440641	Paired	0.0405208	0.95966	RF	83.38	Testis	No
SRX4059016	9425436	Paired	0.0292367	0.971493	RF	91.36	Testis	No
SRX4059017	10691806	Paired	0.0308857	0.969394	RF	83.84	Testis	No
SRX4059018	7152558	Paired	0.0343507	0.965725	RF	88.57	Testis	No
SRX4059019	23727248	Paired	0.0265557	0.973914	RF	93.23	Testis	No
SRX4714940	15768482	Paired	0.019646	0.981016	RF	90.12	Uterus	No
SRX4258008	67038926	Paired	0.917518	0.0804238	FR	83.5	Small intestine	No
SRX4258009	72252087	Paired	0.87912	0.11658	FR	82.66	Small intestine	No
SRX4714942	16542102	Paired	0.0265082	0.973959	RF	90.2	Uterus	No
SRX4714943	15354199	Paired	0.022277	0.978178	RF	90.34	Uterus	No
SRX4714944	16259613	Paired	0.0362362	0.964353	RF	89.92	Placenta	No
SRX4714945	16427544	Paired	0.0349312	0.96576	RF	90.46	Placenta	No
SRX4714946	18482889	Paired	0.0358357	0.964819	RF	89.88	Placenta	No
SRX4714947	15268200	Paired	0.0384639	0.962513	RF	88.96	Placenta	No
SRX4714948	13594101	Paired	0.0399287	0.960983	RF	88.92	Placenta	No
SRX4714949	14171151	Paired	0.0273975	0.972999	RF	89.64	Uterus	No
SRX10048357	38278400	Paired	0.110984	0.88421	RF	81.96	Muscle	No
SRX10048358	44886456	Paired	0.0930274	0.902115	RF	83.68	Muscle	No
SRX10048359	43413457	Paired	0.0941149	0.900639	RF	83.53	Muscle	No
ERX4488160	23476220	Paired	0.0336476	0.950062	RF	92.08	Lung	No
ERX4488161	26548797	Paired	0.0301449	0.961013	RF	92.55	Lung	No
ERX4488162	24289304	Paired	0.0312948	0.956956	RF	92.45	Lung	No
SRX4059020	6882352	Paired	0.0346865	0.965239	RF	88.25	Testis	No
SRX4059021	8966276	Paired	0.0317836	0.968499	RF	82.37	Testis	No
SRX4059022	5332671	Paired	0.037882	0.962417	RF	82.4	Testis	No
SRX4059023	28802790	Paired	0.0281019	0.972335	RF	93.51	Testis	No
SRX4059024	5157293	Paired	0.0443488	0.955558	RF	76.94	Testis	No
SRX4059025	6045734	Paired	0.0380776	0.962203	RF	87.95	Testis	No
SRX4059026	7219726	Paired	0.0353203	0.964478	RF	87.47	Testis	No
SRX4258010	61744529	Paired	0.902337	0.0969261	FR	85.85	Small intestine	No
SRX4714950	16292150	Paired	0.0252481	0.975041	RF	90.07	Uterus	No
SRX4714951	13043891	Paired	0.041167	0.959735	RF	88.73	Placenta	No
SRX10048360	42153113	Paired	0.105175	0.890644	RF	76.41	Muscle	No
SRX4714952	12962569	Paired	0.0394225	0.961519	RF	89.09	Placenta	No
SRX10048361	54079457	Paired	0.11206	0.882973	RF	70.99	Muscle	No
SRX4714953	11939086	Paired	0.0257433	0.97476	RF	90	Uterus	No
SRX10048362	42311258	Paired	0.107465	0.887635	RF	76.85	Muscle	No
SRX4714954	13469989	Paired	0.0236757	0.976737	RF	90.29	Uterus	No
SRX4714955	15722377	Paired	0.027592	0.972889	RF	91.15	Uterus	No
SRX4714956	18857951	Paired	0.0368648	0.963972	RF	89.32	Placenta	No
SRX4714957	18232162	Paired	0.0364136	0.96422	RF	89.3	Placenta	No
SRX4714958	15219204	Paired	0.0235527	0.976884	RF	91.68	Uterus	No
SRX4714959	15443857	Paired	0.0228518	0.977618	RF	91.11	Uterus	No
SRX4059031	15548166	Paired	0.0250292	0.968292	RF	83.9	PBMCs	No
SRX4059032	23374470	Paired	0.0209896	0.974593	RF	83.46	PBMCs	No
SRX4059033	15058618	Paired	0.0676049	0.968086	RF	66.06	PBMCs	No
SRX4059034	19220999	Paired	0.0236455	0.974408	RF	85.98	PBMCs	No
SRX4059035	15135025	Paired	0.0212804	0.972867	RF	88.29	Macrophage	No
SRX4059036	15912011	Paired	0.0227586	0.970415	RF	86.37	Macrophage	No
SRX4714960	14220032	Paired	0.0257062	0.974796	RF	90.51	Uterus	No
SRX4714961	18969214	Paired	0.0347292	0.965936	RF	90.43	Placenta	No
SRX4714962	17150994	Paired	0.0341853	0.966493	RF	90	Placenta	No
SRX4714963	14001639	Paired	0.0290384	0.971256	RF	88.54	Uterus	No
SRX4714964	16347478	Paired	0.0238055	0.976665	RF	90.79	Uterus	No
SRX4714965	12346937	Paired	0.0299684	0.971117	RF	90	Placenta	No
SRX4714966	12306004	Paired	0.0330526	0.967899	RF	88.17	Placenta	No
SRX4714967	15707987	Paired	0.0390524	0.961746	RF	89.72	Placenta	No
SRX4714968	14414761	Paired	0.0354852	0.965595	RF	91.26	Placenta	No

SRX4714969	16951651	Paired	0.0216708	0.978892	RF	90.91	Uterus	No
SRX6384210	38754418	Single	0.0265669	NULL	RF	91.82	Heart	No
SRX6384211	41481612	Single	0.0247686	NULL	RF	91.5	Heart	No
SRX6384212	40105547	Single	0.0247957	NULL	RF	90.95	Heart	No
SRX6384213	40473784	Single	0.024892	NULL	RF	91.95	Heart	No
SRX6384214	39412513	Single	0.0285759	NULL	RF	91.56	Heart	No
SRX6384215	38577207	Single	0.0278349	NULL	RF	91.05	Heart	No
SRX6384216	39089537	Single	0.026771	NULL	RF	91.33	Heart	No
SRX6384217	36543499	Single	0.0250007	NULL	RF	91.78	Heart	No
SRX6384218	40483699	Single	0.0275267	NULL	RF	91.16	Heart	No
SRX6384219	31125644	Single	0.0250879	NULL	RF	91.11	Heart	No
SRX4714970	17726228	Paired	0.0240372	0.976364	RF	91.27	Uterus	No
SRX4714971	15888422	Paired	0.0208616	0.979802	RF	91	Uterus	No
SRX4714972	10402611	Paired	0.0220835	0.978602	RF	90.59	Uterus	No
SRX4714973	16988858	Paired	0.0298443	0.970825	RF	87.71	Placenta	No
SRX4714974	17185666	Paired	0.0325006	0.968631	RF	90.19	Placenta	No
SRX4714975	16610107	Paired	0.0229063	0.977478	RF	90.59	Uterus	No
SRX4714976	14068022	Paired	0.0208401	0.979596	RF	89.38	Uterus	No
SRX4714977	15063015	Paired	0.0440876	0.955939	RF	89.97	Placenta	No
SRX4714978	13471930	Paired	0.0447046	0.955296	RF	89.25	Placenta	No
SRX4714979	17339366	Paired	0.0211846	0.979258	RF	90.94	Uterus	No
SRX6384220	36850849	Single	0.0291072	NULL	RF	91.24	Heart	No
SRX6384221	38558377	Single	0.0269623	NULL	RF	91.35	Heart	No
SRX3313023	43954391	Paired	0.0983563	0.896593	RF	88.46	Spleen	No
SRX6384222	37311236	Single	0.0320837	NULL	RF	90.18	Heart	No
SRX3313024	40563735	Paired	0.0891539	0.907573	RF	89.86	Spleen	No
SRX6384223	39308295	Single	0.0280566	NULL	RF	91.14	Heart	No
SRX3313025	49542382	Paired	0.0858299	0.912529	RF	90.26	Spleen	No
SRX6384224	46752058	Single	0.028358	NULL	RF	90.34	Heart	No
SRX3313026	47748956	Paired	0.117394	0.874575	RF	84.32	Spleen	No
SRX6384225	39668861	Single	0.0277955	NULL	RF	91.14	Heart	No
SRX3313027	46412096	Paired	0.0686788	0.931291	RF	89.75	Spleen	No
SRX3313029	43683196	Paired	0.0689396	0.928222	RF	88.55	Spleen	No
SRX4714980	13170690	Paired	0.026184	0.974121	RF	89.93	Uterus	No
SRX4714981	14808580	Paired	0.0341141	0.966732	RF	89.13	Placenta	No
SRX4714982	14187685	Paired	0.0342632	0.966815	RF	89.55	Placenta	No
SRX4714983	13841223	Paired	0.0214118	0.979162	RF	87.67	Uterus	No
SRX4714984	14089917	Paired	0.0200789	0.980577	RF	85.92	Uterus	No
SRX4714985	13002356	Paired	0.0398517	0.960962	RF	88.57	Placenta	No
SRX4714986	12279734	Paired	0.0435661	0.957266	RF	88.56	Placenta	No
SRX4714987	12130091	Paired	0.0283539	0.972074	RF	89.43	Uterus	No
SRX7675746	98040490	Paired	0.0357024	0.960095	RF	85.9	Muscle	No
SRX4714988	16186992	Paired	0.0235661	0.976973	RF	90.2	Uterus	No
SRX7675747	94140020	Paired	0.037772	0.958682	RF	84.88	Muscle	No
SRX4714989	17205956	Paired	0.0317775	0.969661	RF	91.71	Placenta	No
SRX7675748	93755287	Paired	0.0649127	0.926471	RF	84.23	Muscle	No
SRX7675749	92806668	Paired	0.0615696	0.930019	RF	83.6	Muscle	No
SRX3313030	45447585	Paired	0.0747751	0.921776	RF	83.67	Spleen	No
SRX3313031	49218439	Paired	0.066793	0.930098	RF	87.24	Spleen	No
SRX3313032	47977449	Paired	0.0694347	0.930502	RF	89.78	Spleen	No
SRX3313033	49347921	Paired	0.0603611	0.938263	RF	88.3	Spleen	No
SRX3313034	47502404	Paired	0.0699446	0.927526	RF	88.46	Spleen	No
SRX3313035	59826287	Paired	0.0638922	0.933558	RF	88.17	Spleen	No
SRX3313036	39767925	Paired	0.0572422	0.940693	RF	84.6	Spleen	No
SRX3313037	54186690	Paired	0.094123	0.902003	RF	90.17	Spleen	No
ERX3439283	15177872	Paired	0.0348688	0.945174	RF	69.38	Muscle	No
SRX4714990	18510122	Paired	0.0315968	0.969825	RF	90.71	Placenta	No
SRX4714991	16351988	Paired	0.0210604	0.979533	RF	90.18	Uterus	No

SRX7675750	89253407	Paired	0.0744246	0.914664	RF	83.25	Muscle	No
SRX4714992	14896928	Paired	0.0208995	0.979817	RF	90.84	Uterus	No
SRX7675751	90823124	Paired	0.0780868	0.91055	RF	83.74	Muscle	No
SRX4714993	8585239	Paired	0.0346882	0.966688	RF	91.14	Placenta	No
SRX7675752	87838717	Paired	0.0791026	0.90934	RF	80.02	Muscle	No
SRX4714994	15937263	Paired	0.0331129	0.96826	RF	89.82	Placenta	No
SRX7675753	95885378	Paired	0.0579937	0.934653	RF	85.24	Muscle	No
SRX7675754	98966038	Paired	0.0565412	0.936329	RF	85.04	Muscle	No
SRX7675755	99233712	Paired	0.0340984	0.962435	RF	83.35	Muscle	No
SRX7675756	109317565	Paired	0.0573195	0.934623	RF	83.78	Muscle	No
SRX7675757	104976839	Paired	0.0660557	0.923932	RF	85.38	Muscle	No
ERX4906669	146407221	Paired	0.0256629	0.972694	RF	89.09	Brain	No
SRX7365001	38473585	Single	0.100846	NULL	RF	61.97	Lung	No
SRX7365002	37308240	Single	0.0811454	NULL	RF	73.14	Lung	No
SRX7365003	66546330	Single	0.0895975	NULL	RF	80.32	Lung	No
SRX7365004	74502439	Single	0.064149	NULL	RF	78.44	Lung	No
SRX7365005	67780872	Single	0.0674752	NULL	RF	80.25	Lung	No
ERX4906670	90346114	Paired	0.0250185	0.972157	RF	87.33	Brain	No
ERX4906671	98985347	Paired	0.0247633	0.974627	RF	89.24	Brain	No
ERX4906672	101287166	Paired	0.0207686	0.977831	RF	90.65	Brain	No
ERX4906673	120588588	Paired	0.0198238	0.977627	RF	86.8	Small intestine	No
ERX4906674	100988151	Paired	0.020469	0.978249	RF	86.99	Small intestine	No
ERX4906675	110899660	Paired	0.0208179	0.977088	RF	85.56	Small intestine	No
ERX4906676	161674634	Paired	0.0186116	0.9806	RF	88.34	Small intestine	No
ERX4906677	108968427	Paired	0.0200399	0.979894	RF	88.87	Kidney	No
ERX4906678	115124153	Paired	0.0212758	0.977483	RF	89.7	Kidney	No
ERX4906679	118156832	Paired	0.0220694	0.979202	RF	88.18	Lung	No
SRX11916333	23151780	Paired	0.022249	0.977594	RF	91.85	Small intestine	No
SRX11916334	20563321	Paired	0.0215616	0.978581	RF	92.04	Small intestine	No
SRX11916335	23383511	Paired	0.0204963	0.980279	RF	91.43	Small intestine	No
SRX11916336	22823169	Paired	0.0200496	0.980224	RF	91.38	Small intestine	No
SRX11916337	22588399	Paired	0.0220521	0.978275	RF	87.89	Small intestine	No
SRX11916338	22422489	Paired	0.0222192	0.978477	RF	91.43	Small intestine	No
ERX4906680	128633814	Paired	0.0214612	0.978675	RF	88.18	Lung	No
ERX4906681	129338180	Paired	0.00972336	0.991905	RF	91.03	Muscle	No
ERX4906682	90413713	Paired	0.0096973	0.99081	RF	90.65	Muscle	No
ERX4906683	107417207	Paired	0.00874575	0.986834	RF	87.88	Muscle	No
ERX4906684	117386825	Paired	0.00890143	0.988065	RF	87.4	Muscle	No
ERX4906685	102926635	Paired	0.0178639	0.978019	RF	84.56	Small intestine	No
ERX4906686	121856321	Paired	0.0160479	0.981381	RF	82.87	Small intestine	No
ERX4906687	96095466	Paired	0.0141924	0.984499	RF	84.09	Small intestine	No
ERX4906688	117206423	Paired	0.0150082	0.97999	RF	82.83	Small intestine	No
ERX4906689	132065774	Paired	0.0237775	0.974769	RF	90.06	Testis	No
ERX4906690	92176646	Paired	0.0233843	0.972921	RF	89.21	Testis	No
ERX1886666	61680594	Paired	0.0873107	0.897509	RF	85.39	Brain	No
ERX1886667	59893288	Paired	0.0916552	0.891185	RF	88.97	Kidney	No
ERX1886668	64098080	Paired	0.101353	0.878909	RF	86.77	Heart	No
ERX1886669	39228316	Paired	0.0932389	0.874603	RF	82.5	Large intestine	No
ERX1886670	65398097	Paired	0.0890508	0.892828	RF	85.41	Large intestine	No
ERX585884	9339104	Single	0.0298705	NULL	RF	91.43	Muscle	No
ERX585885	8237219	Single	0.0363896	NULL	RF	91.03	Muscle	No
ERX585886	9155202	Single	0.0332988	NULL	RF	90.85	Muscle	No
ERX585887	9271112	Single	0.0315433	NULL	RF	90.01	Muscle	No
ERX585888	7634672	Single	0.0317907	NULL	RF	90.51	Muscle	No
ERX585889	9209355	Single	0.029729	NULL	RF	90.85	Muscle	No
ERX1886683	37687304	Paired	0.0812023	0.908294	RF	86.92	Spleen	No
SRX8483819	36443082	Paired	0.172386	0.825356	RF	77.39	Muscle	No
ERX1886691	42433618	Paired	0.0907794	0.891636	RF	84.04	Adipose	No

ERX1886692	37440667	Paired	0.0710627	0.912799	RF	85.86	Muscle	No
ERX1886693	17898943	Paired	0.0820199	0.881469	RF	84.47	Brain	No
ERX1886694	27678662	Paired	0.0782812	0.873183	RF	82.52	Large intestine	No
ERX1886695	16529565	Paired	0.07394	0.897178	RF	82.5	Small intestine	No
ERX1886696	26759520	Paired	0.0800317	0.889142	RF	82.93	Small intestine	No
SRX8483821	42306505	Paired	0.155534	0.841704	RF	81.33	Muscle	No
SRX8483822	48779081	Paired	0.0403011	0.959196	RF	80.71	Adipose	No
ERX1886698	28483549	Paired	0.0985016	0.853417	RF	86.89	Brain	No
SRX8483823	49363573	Paired	0.0314293	0.968622	RF	84.45	Adipose	No
ERX1886699	40372363	Paired	0.105115	0.875702	RF	86.93	Brain	No
SRX8483824	59548826	Paired	0.03421	0.966351	RF	86.37	Muscle	No
SRX8483825	58513830	Paired	0.0427231	0.957941	RF	88.16	Muscle	No
SRX8483828	41965288	Paired	0.0325172	0.968428	RF	82.14	Muscle	No
SRX8483829	41973982	Paired	0.162356	0.835443	RF	82.13	Muscle	No
SRX8483830	36656306	Paired	0.186	0.81082	RF	79.12	Muscle	No
SRX8483831	39727327	Paired	0.0247097	0.977386	RF	83.27	Muscle	No
SRX8483833	47752133	Paired	0.0484658	0.95111	RF	89.48	Brain	No
SRX8483834	48870466	Paired	0.0544574	0.944394	RF	89.55	Brain	No
SRX8483835	48909470	Paired	0.0477824	0.951384	RF	88.88	Brain	No
SRX8483836	47563774	Paired	0.0464978	0.952208	RF	89.06	Brain	No
SRX8483837	60231327	Paired	0.0568257	0.942245	RF	89.89	Brain	No
SRX8483839	57502597	Paired	0.0419562	0.957226	RF	89.31	Brain	No
SRX7276986	76971616	Single	0.0243251	NULL	RF	94.07	Testis	No
SRX7276987	107127989	Single	0.0218518	NULL	RF	94.33	Testis	No
SRX7276988	119092453	Single	0.0285347	NULL	RF	93.98	Testis	No
SRX7276989	87238872	Single	0.0268799	NULL	RF	93.91	Testis	No
SRX7438804	48821048	Paired	0.0385647	0.960819	RF	81.76	Heart	No
SRX4641300	5985850	Paired	0.0386494	0.960937	RF	77.52	Testis	No
SRX4641301	5978790	Paired	0.0358585	0.965334	RF	76.73	Testis	No
SRX4641302	17045126	Paired	0.0310312	0.971232	RF	91.78	Testis	No
SRX4641303	8599182	Paired	0.0323054	0.967974	RF	80.53	Testis	No
SRX4641304	6598926	Paired	0.035867	0.964226	RF	77.94	Testis	No
SRX4641305	5345234	Paired	0.0387364	0.960943	RF	89.95	Testis	No
SRX4641306	8327085	Paired	0.0301102	0.97013	RF	83.23	Testis	No
SRX4641307	7759702	Paired	0.0372406	0.962637	RF	78.47	Testis	No
SRX4641308	24786617	Paired	0.0307618	0.969603	RF	93.79	Testis	No
SRX4641309	5440641	Paired	0.0404244	0.959661	RF	83.38	Testis	No
SRX8483840	47864822	Paired	0.0337396	0.966279	RF	85.9	Large intestine	No
SRX8483841	53287103	Paired	0.0373692	0.963437	RF	86.2	Large intestine	No
SRX8483843	40546050	Paired	0.135102	0.86269	RF	71.93	Muscle	No
SRX8483844	29682861	Paired	0.160058	0.837484	RF	80.02	Muscle	No
SRX6419104	37603031	Paired	0.0727495	0.894191	RF	83.96	Ovary	No
SRX6419105	44166536	Paired	0.11025	0.880589	RF	85.36	Ovary	No
SRX7276990	94020057	Single	0.0248588	NULL	RF	94.01	Testis	No
SRX8483847	37300473	Paired	0.0412212	0.958947	RF	82.29	Muscle	No
SRX6419106	49905772	Paired	0.0797052	0.900629	RF	86.69	Ovary	No
SRX7276991	112771480	Single	0.0270545	NULL	RF	94.29	Testis	No
ERX1877981	36953933	Paired	0.0140729	0.987253	RF	92.72	Liver	No
SRX6419107	39079987	Paired	0.0557392	0.929947	RF	88.43	Ovary	No
SRX7276992	126616169	Single	0.0214559	NULL	RF	94.42	Testis	No
ERX1877982	29120800	Paired	0.0217536	0.980021	RF	84.98	Spleen	No
SRX6419108	37282333	Paired	0.051748	0.929246	RF	85.17	Ovary	No
SRX7276993	138901996	Single	0.0237459	NULL	RF	94.81	Testis	No
ERX1877983	33959341	Paired	0.0258139	0.975219	RF	83.87	Testis	No
SRX6419109	39291401	Paired	0.0492036	0.922909	RF	86.01	Ovary	No
SRX7276994	79458899	Single	0.0237232	NULL	RF	94.06	Testis	No
ERX1877984	40029118	Paired	0.0201923	0.980847	RF	94.99	Brain	No
SRX7276995	78309868	Single	0.0245755	NULL	RF	94.47	Testis	No

SRX7276996	70656344	Single	0.0261508	NULL	RF	93.92	Testis	No
SRX7276997	120645703	Single	0.0268174	NULL	RF	94.76	Testis	No
SRX7276998	84035721	Single	0.0244877	NULL	RF	94.27	Testis	No
SRX7276999	64626022	Single	0.0291895	NULL	RF	93.91	Testis	No
ERX1877989	28270072	Paired	0.020108	0.98035	RF	91.34	Brain	No
SRX4641310	9425436	Paired	0.0293025	0.971531	RF	91.36	Testis	No
SRX4641311	10691806	Paired	0.030868	0.969441	RF	83.84	Testis	No
SRX4641312	7152558	Paired	0.0344235	0.965736	RF	88.57	Testis	No
SRX4641313	23727248	Paired	0.0265804	0.973919	RF	93.23	Testis	No
SRX4641314	6882352	Paired	0.0346798	0.965209	RF	88.25	Testis	No
SRX4641315	8966276	Paired	0.0318087	0.968485	RF	82.37	Testis	No
SRX4641316	5332671	Paired	0.037881	0.962547	RF	82.4	Testis	No
SRX4641317	28802790	Paired	0.0281156	0.972359	RF	93.51	Testis	No
SRX4641318	5157293	Paired	0.0443732	0.955527	RF	76.94	Testis	No
SRX4641319	6045734	Paired	0.0380005	0.962142	RF	87.95	Testis	No
SRX8483851	40370966	Paired	0.0289067	0.972043	RF	84.01	Muscle	No
SRX6419110	41213408	Paired	0.0855746	0.886294	RF	85.67	Ovary	No
SRX6419111	38915329	Paired	0.0491797	0.928027	RF	84.68	Ovary	No
SRX8483853	54687023	Paired	0.0326221	0.968003	RF	86.87	Muscle	No
SRX6419112	55266523	Paired	0.0855158	0.89331	RF	85.59	Ovary	No
SRX8483854	48255538	Paired	0.0417381	0.958917	RF	85.94	Muscle	No
SRX6419113	38812747	Paired	0.089311	0.897191	RF	86.14	Ovary	No
SRX8483855	52944437	Paired	0.0425102	0.958317	RF	87.89	Muscle	No
SRX6419114	37056740	Paired	0.0673507	0.912451	RF	87.33	Ovary	No
SRX8483856	33892911	Paired	0.0901448	0.906896	RF	78.68	Muscle	No
SRX6419115	40976878	Paired	0.0805951	0.907398	RF	87.81	Ovary	No
ERX1877990	38103112	Paired	0.0151675	0.986677	RF	91.82	Muscle	No
SRX6419116	59743436	Paired	0.0884729	0.893216	RF	85.42	Ovary	No
ERX1877991	37001646	Paired	0.0183456	0.983933	RF	89.99	Liver	No
SRX6419117	43581819	Paired	0.0866475	0.899247	RF	87.2	Ovary	No
ERX1877992	32344419	Paired	0.023095	0.97897	RF	85.39	Spleen	No
SRX8483859	48133547	Paired	0.0375276	0.963189	RF	87.78	Muscle	No
SRX6419118	44296687	Paired	0.0991931	0.888222	RF	86.92	Ovary	No
ERX1877993	39281361	Paired	0.0249376	0.975595	RF	88.62	Testis	No
ERX1877994	39629537	Paired	0.019799	0.980846	RF	92.16	Brain	No
ERX1877995	39495279	Paired	0.0157611	0.985477	RF	92.71	Muscle	No
ERX1877997	44376396	Paired	0.0222743	0.978984	RF	92.23	Lung	No
ERX1877998	34128825	Paired	0.022938	0.97845	RF	92.74	Lymph node	No
SRX7691571	52030184	Paired	0.0194668	0.967273	RF	85.06	Heart	No
SRX7691572	37359505	Paired	0.0264779	0.965736	RF	83.22	Heart	No
SRX7691573	38757820	Paired	0.025697	0.969277	RF	84.94	Heart	No
SRX7691575	49026987	Paired	0.0250846	0.960725	RF	83.79	Heart	No
SRX7691576	62585991	Paired	0.0237205	0.962284	RF	85.67	Heart	No
SRX4641320	7219726	Paired	0.0352759	0.964404	RF	87.47	Testis	No
SRX7691577	48249085	Paired	0.022137	0.963896	RF	86.25	Heart	No
SRX7691578	59569356	Paired	0.0236242	0.965267	RF	87.19	Heart	No
SRX7691579	50797324	Paired	0.0262108	0.961882	RF	87.45	Heart	No
SRX9052703	17871850	Single	0.0275956	NULL	RF	92.95	Heart	No
SRX9052704	15308706	Single	0.0292512	NULL	RF	93.52	Heart	No
SRX9052705	17773031	Single	0.0267697	NULL	RF	93.36	Heart	No
SRX9052706	16164596	Single	0.0264865	NULL	RF	93.97	Heart	No
SRX9052707	20749092	Single	0.0299059	NULL	RF	92.26	Heart	No
SRX9052708	19587478	Single	0.0282316	NULL	RF	91.8	Heart	No
SRX9052709	20226519	Single	0.0289838	NULL	RF	92.13	Heart	No
SRX8483861	53197580	Paired	0.0373594	0.964212	RF	85.47	Muscle	No
SRX8483862	49806076	Paired	0.0320449	0.970392	RF	86.84	Muscle	No
SRX8483863	47347312	Paired	0.0504329	0.948789	RF	87.61	Adipose	No
SRX8483864	56179854	Paired	0.0360716	0.964812	RF	82.74	Adipose	No

SRX8483865	49203020	Paired	0.0356255	0.964656	RF	85.78	Adipose	No
SRX8483866	35501871	Paired	0.166814	0.830198	RF	78.54	Muscle	No
SRX8483867	40111396	Paired	0.183724	0.8113	RF	80.75	Muscle	No
SRX8483868	41757347	Paired	0.109691	0.888206	RF	79.53	Muscle	No
SRX8483869	43165840	Paired	0.0479155	0.950633	RF	67.38	Adipose	No
SRX3174410	18743404	Paired	0.972064	0.0288164	FR	81.6	Muscle	No
SRX3174411	21473932	Paired	0.973454	0.027258	FR	88.3	Muscle	No
SRX3174412	22262698	Paired	0.974974	0.025874	FR	87.76	Muscle	No
SRX3174413	22955071	Paired	0.976097	0.0246024	FR	88.49	Muscle	No
SRX3174414	20142745	Paired	0.0246477	0.975972	RF	87.87	Muscle	No
SRX3174415	22862102	Paired	0.97194	0.0290217	FR	87.28	Muscle	No
SRX3174416	20423554	Paired	0.975452	0.0254401	FR	79.82	Muscle	No
SRX3174417	31223011	Paired	0.972491	0.0281774	FR	78.63	Muscle	No
SRX7691580	38749555	Paired	0.0227551	0.966527	RF	81.16	Heart	No
SRX3174418	21269456	Paired	0.0266267	0.973999	RF	86.79	Muscle	No
SRX7691581	39788162	Paired	0.0263528	0.967539	RF	84.69	Heart	No
SRX3174419	30030596	Paired	0.976651	0.0239973	FR	80.23	Muscle	No
SRX7691582	39392587	Paired	0.0241935	0.97055	RF	85.23	Heart	No
SRX7691583	38871578	Paired	0.0279977	0.961227	RF	79.02	Heart	No
SRX7691584	44161639	Paired	0.0224797	0.964819	RF	85.49	Heart	No
SRX7691585	34994012	Paired	0.0261275	0.951198	RF	85.97	Heart	No
SRX7691586	50749649	Paired	0.0234892	0.964234	RF	83.26	Heart	No
SRX7691587	33901073	Paired	0.0279453	0.966737	RF	85.05	Heart	No
SRX7691588	37112047	Paired	0.0234321	0.972624	RF	87.32	Heart	No
SRX7691589	24428664	Paired	0.0250039	0.964608	RF	83.69	Heart	No
SRX9052710	17931603	Single	0.0308856	NULL	RF	92.42	Heart	No
SRX9052711	20063400	Single	0.0294791	NULL	RF	92.37	Heart	No
SRX9052712	15824139	Single	0.0297902	NULL	RF	92.22	Heart	No
SRX8483870	60622062	Paired	0.0319968	0.968645	RF	86.26	Adipose	No
SRX8483872	49550069	Paired	0.0330257	0.967158	RF	87.56	Adipose	No
SRX8483873	37064487	Paired	0.188255	0.807891	RF	78.78	Muscle	No
SRX8483874	33260822	Paired	0.0378517	0.962329	RF	80.44	Muscle	No
SRX8483876	55336816	Paired	0.0428075	0.956773	RF	89.68	Kidney	No
SRX9339796	46453739	Paired	0.973802	0.0253251	FR	79.01	Muscle	No
SRX8483877	53090046	Paired	0.0583116	0.941452	RF	89.49	Kidney	No
SRX9339797	42167488	Paired	0.97709	0.0240911	FR	83.38	Muscle	No
SRX8483878	50637192	Paired	0.0426046	0.956864	RF	89.15	Kidney	No
SRX9339798	45801682	Paired	0.979033	0.0218758	FR	83.49	Muscle	No
SRX8483879	64653637	Paired	0.0300024	0.972082	RF	85.74	Muscle	No
SRX9339799	49640428	Paired	0.978019	0.0222732	FR	88.58	Muscle	No
SRX3174420	27813409	Paired	0.025188	0.975321	RF	87.6	Muscle	No
SRX3174421	18646145	Paired	0.0277561	0.972674	RF	87.89	Muscle	No
SRX3174422	22201436	Paired	0.972313	0.028295	FR	86.58	Muscle	No
SRX3174424	19230722	Paired	0.970736	0.0296721	FR	86.09	Muscle	No
SRX3174425	6001272	Paired	0.963025	0.037164	FR	83.12	Muscle	No
SRX3174427	16551250	Paired	0.975539	0.0250848	FR	78.96	Muscle	No
SRX7691590	26166111	Paired	0.025295	0.962373	RF	84.14	Heart	No
SRX3174428	6307468	Paired	0.0337333	0.966688	RF	85.09	Muscle	No
SRX7691591	31792651	Paired	0.0246596	0.961983	RF	83.56	Heart	No
SRX8483880	66935101	Paired	0.0437093	0.956399	RF	88.58	Muscle	No
SRX8483881	58687518	Paired	0.020888	0.982271	RF	85.97	Muscle	No
SRX8483885	54518380	Paired	0.0391352	0.96164	RF	88.95	Heart	No
SRX8483886	46425697	Paired	0.0405731	0.960484	RF	83.96	Heart	No
SRX8483887	53307922	Paired	0.0519665	0.947744	RF	88.35	Heart	No
SRX8483888	55416095	Paired	0.0918157	0.905228	RF	88.44	Heart	No
SRX8483889	46847136	Paired	0.0779869	0.921715	RF	86.25	Heart	No
SRX3174430	19493241	Paired	0.973767	0.0267098	FR	86.57	Muscle	No
SRX3174431	13368361	Paired	0.0304089	0.970184	RF	84.37	Muscle	No

SRX3174432	15756224	Paired	0.0287084	0.971614	RF	81.77	Muscle	No
SRX3174433	17040269	Paired	0.0248241	0.975754	RF	86.25	Muscle	No
SRX3004257	6837561	Single	0.883912	NULL	FR	72.07	Adipose	No
SRX3004258	10873352	Single	0.901328	NULL	FR	83.89	Adipose	No
SRX3004259	16399754	Single	0.913466	NULL	FR	76.73	Adipose	No
ERX779590	37163323	Paired	0.0226034	0.978892	RF	85.49	Adipose	No
SRX4581431	53846744	Paired	0.0241158	0.976514	RF	80.3	Muscle	No
ERX779591	39819678	Paired	0.0221659	0.979635	RF	87.51	Heart	No
ERX779592	38481128	Paired	0.0233091	0.97809	RF	86.32	Kidney	No
SRX9775270	27363014	Single	0.0793566	NULL	RF	90	Muscle	No
ERX779593	42969965	Paired	0.0198803	0.982542	RF	84.38	Liver	No
SRX9775271	40846109	Single	0.0789611	NULL	RF	89.67	Muscle	No
ERX779594	32754301	Paired	0.0258884	0.975774	RF	78.82	Lung	No
ERX779596	39233680	Paired	0.0151616	0.986712	RF	85.55	Muscle	No
ERX779597	31221953	Paired	0.0292199	0.972175	RF	68.58	Spleen	No
ERX779598	22209302	Paired	0.0241047	0.977156	RF	95.23	Adipose	No
ERX779599	16372336	Paired	0.0260077	0.976	RF	90.96	Heart	No
SRX6885057	11197920	Paired	0.0745431	0.922676	RF	65.05	Placenta	No
SRX5055428	78929748	Paired	0.0355213	0.963756	RF	83.9	Testis	No
SRX9211725	701618117	Paired	0.0318728	0.967393	RF	93.91	Heart	No
SRX9383753	106431350	Paired	0.0873415	0.910978	RF	91.19	Adipose	No
SRX1465952	235952244	Paired	0.067803	0.92946	RF	78.99	Liver	No
SRX3004245	13997489	Single	0.91155	NULL	FR	84.03	Adipose	No
SRX3004246	12761503	Single	0.924193	NULL	FR	85.13	Adipose	No
SRX3004260	12337329	Single	0.915315	NULL	FR	80.27	Adipose	No
SRX3004247	11782591	Single	0.909283	NULL	FR	82.92	Adipose	No
SRX3004261	20736573	Single	0.908698	NULL	FR	76.13	Adipose	No
SRX3004248	12933511	Single	0.918334	NULL	FR	76.27	Adipose	No
SRX3004262	11545400	Single	0.906462	NULL	FR	82.13	Adipose	No
SRX3004249	8314663	Single	0.898922	NULL	FR	68.42	Adipose	No
SRX3004263	14374209	Single	0.887233	NULL	FR	69.19	Adipose	No
SRX3004264	11168653	Single	0.89521	NULL	FR	71.51	Adipose	No
SRX3004265	13158157	Single	0.925161	NULL	FR	86.66	Adipose	No
SRX3004267	11827157	Single	0.903157	NULL	FR	76.91	Adipose	No
ERX779600	23341004	Paired	0.0258158	0.97557	RF	95.68	Kidney	No
ERX779601	19906587	Paired	0.0222563	0.979436	RF	94.25	Liver	No
ERX779602	19996158	Paired	0.0261786	0.975217	RF	95	Lung	No
ERX779604	19212193	Paired	0.0169163	0.984349	RF	93.75	Muscle	No
ERX779605	14100364	Paired	0.030558	0.971033	RF	92.39	Spleen	No
SRX6885043	17346732	Paired	0.0590242	0.938929	RF	75.88	Placenta	No
SRX9775266	35184234	Single	0.111965	NULL	RF	89.57	Muscle	No
SRX9775267	39082244	Single	0.0610307	NULL	RF	89.97	Muscle	No
SRX6885046	14861554	Paired	0.0668791	0.930657	RF	70.92	Placenta	No
SRX9775268	24835992	Single	0.0767408	NULL	RF	88.83	Muscle	No
SRX6885047	11293272	Paired	0.0670696	0.928091	RF	68.17	Placenta	No
SRX9775269	28174360	Single	0.0898842	NULL	RF	89.3	Muscle	No
SRX6885048	15296257	Paired	0.0612969	0.937283	RF	75.22	Placenta	No
SRX3313028	42068468	Paired	0.0712926	0.925262	RF	83.21	Spleen	No
SRX3174487	50010890	Paired	0.0229613	0.977578	RF	85.35	Muscle	No
SRX10654851	21904582	Paired	0.0324102	0.963049	RF	91.45	Early embryo	No
SRX10654852	901456	Paired	0.104109	0.889378	RF	91.13	Early embryo	No
SRX2826810	38576228	Paired	0.0232952	0.976868	RF	84.85	Placenta	No
SRX3004250	11682684	Single	0.919159	NULL	FR	85.31	Adipose	No
SRX3004251	12513215	Single	0.911295	NULL	FR	76.52	Adipose	No
SRX3004252	11844270	Single	0.912931	NULL	FR	81.11	Adipose	No
SRX3004253	10735381	Single	0.887491	NULL	FR	75.6	Adipose	No
SRX3004254	14063943	Single	0.915345	NULL	FR	78.64	Adipose	No
SRX3004255	11321990	Single	0.910338	NULL	FR	65.02	Adipose	No

SRX3004256	11787007	Single	0.888156	NULL	FR	75.83	Adipose	No
ERX9241854	15606857	Paired	0.0208056	0.98048	RF	94.57	Muscle	No
ERX8133445	15722650	Paired	0.0239653	0.977096	RF	84.6	Liver	No
ERX10645162	22799966	Paired	0.0737429	0.925953	RF	90.36	Large intestine	No
ERX10645149	23596464	Paired	0.936392	0.0647162	FR	89.65	Small intestine	No
ERX9241855	17759060	Paired	0.0207956	0.980225	RF	94.29	Muscle	No
ERX8133446	17380805	Paired	0.0196797	0.981377	RF	82.33	Liver	No
ERX10645163	19601041	Paired	0.0975995	0.901901	RF	90.08	Large intestine	No
ERX9241856	16594613	Paired	0.0212785	0.979883	RF	94.51	Muscle	No
ERX8133447	17415584	Paired	0.0233975	0.977978	RF	73.28	Liver	No
ERX10645164	25839202	Paired	0.0806072	0.919529	RF	90.17	Large intestine	No
ERX9241857	16086739	Paired	0.0230272	0.977909	RF	94.08	Muscle	No
ERX8133448	15622126	Paired	0.0190556	0.981813	RF	87.59	Liver	No
ERX10645165	25899629	Paired	0.072252	0.927346	RF	90.26	Large intestine	No
ERX9241858	16232422	Paired	0.0222846	0.978636	RF	94.24	Muscle	No
ERX8133449	16342675	Paired	0.0193261	0.981814	RF	79.06	Liver	No
ERX10645166	26664421	Paired	0.913753	0.0860427	FR	89.34	Large intestine	No
ERX9241859	15034901	Paired	0.0228428	0.97841	RF	94.06	Muscle	No
ERX10645200	24920182	Paired	0.92801	0.072255	FR	90.09	Large intestine	No
ERX10645180	28851487	Paired	0.925464	0.0747688	FR	88.38	Small intestine	No
ERX10645167	29251970	Paired	0.0801921	0.919929	RF	89.66	Large intestine	No
ERX10645201	27222960	Paired	0.927124	0.0729609	FR	90.16	Large intestine	No
ERX10645181	23524051	Paired	0.924127	0.0770035	FR	89.27	Small intestine	No
ERX10645168	24110923	Paired	0.920373	0.0800071	FR	89.16	Large intestine	No
ERX10645202	32645021	Paired	0.919918	0.079841	FR	88.77	Large intestine	No
ERX10645182	25648491	Paired	0.927411	0.0733506	FR	88.74	Small intestine	No
ERX10645169	33654840	Paired	0.0863442	0.91405	RF	88.95	Large intestine	No
ERX10645203	31796146	Paired	0.926413	0.0734522	FR	89.67	Large intestine	No
ERX10645183	24996808	Paired	0.0770956	0.923813	RF	89.8	Small intestine	No
ERX10645204	23891404	Paired	0.0702125	0.930128	RF	90.43	Large intestine	No
ERX10645184	21631140	Paired	0.931582	0.0695779	FR	89.26	Small intestine	No
ERX10645185	22986128	Paired	0.919214	0.0810822	FR	89.72	Small intestine	No
ERX10645206	26597451	Paired	0.927796	0.0725339	FR	90.06	Large intestine	No
ERX10645186	21436037	Paired	0.0732909	0.927841	RF	88.71	Small intestine	No
ERX10645207	21809559	Paired	0.0920516	0.908588	RF	89.49	Large intestine	No
ERX10645187	23415684	Paired	0.0818388	0.918865	RF	89.66	Small intestine	No
ERX10645208	22911687	Paired	0.911326	0.0880573	FR	88.48	Large intestine	No
ERX10645188	27389539	Paired	0.0753613	0.924914	RF	90.04	Small intestine	No
ERX10645209	22518402	Paired	0.0769237	0.92311	RF	89.66	Large intestine	No
ERX10645189	20321406	Paired	0.0717621	0.92937	RF	90.2	Small intestine	No
ERX9241800	14985537	Paired	0.0302575	0.970694	RF	94.63	Muscle	No
ERX9241801	12809978	Paired	0.0304733	0.970216	RF	94.45	Muscle	No
ERX10645110	22935286	Paired	0.0938233	0.90769	RF	86.92	Small intestine	No
ERX9241803	15415152	Paired	0.0211367	0.980074	RF	94.51	Muscle	No
ERX10645111	25170919	Paired	0.110746	0.896691	RF	84.63	Small intestine	No
ERX9241804	14304219	Paired	0.0315139	0.969757	RF	94.36	Muscle	No
ERX10645112	23629155	Paired	0.922093	0.078266	FR	89.07	Large intestine	No
ERX9241805	13860721	Paired	0.0324035	0.968338	RF	94.12	Muscle	No
ERX10645113	24510345	Paired	0.903537	0.0966047	FR	87.1	Large intestine	No
ERX9241806	14000764	Paired	0.0302597	0.970625	RF	94.57	Muscle	No
ERX8133410	14867544	Paired	0.0228588	0.978186	RF	78.68	Liver	No
ERX8133390	19403429	Paired	0.0186773	0.982211	RF	87.06	Liver	No
ERX10645114	25251963	Paired	0.915664	0.0846415	FR	88.17	Large intestine	No
ERX9241820	19774107	Paired	0.0224322	0.978375	RF	93.89	Muscle	No
ERX9241807	14094256	Paired	0.029251	0.971827	RF	94.79	Muscle	No
ERX8133411	15351393	Paired	0.0220177	0.979206	RF	86.03	Liver	No
ERX8133391	15025002	Paired	0.0206657	0.980508	RF	83.92	Liver	No
ERX10645115	43141674	Paired	0.0723651	0.927805	RF	90.31	Large intestine	No

ERX9241821	16658554	Paired	0.0255931	0.975347	RF	94.34	Muscle	No
ERX9241808	15544721	Paired	0.018758	0.982325	RF	94.57	Muscle	No
ERX8133412	17239259	Paired	0.0185158	0.982533	RF	83.89	Liver	No
ERX8133392	16977923	Paired	0.0208762	0.980149	RF	85.34	Liver	No
ERX10645116	23283421	Paired	0.0944493	0.90654	RF	86.71	Small intestine	No
ERX9241822	17243209	Paired	0.0267806	0.973853	RF	94.33	Muscle	No
ERX9241809	14602691	Paired	0.0194814	0.981499	RF	94.44	Muscle	No
ERX8133413	15000009	Paired	0.0234089	0.977702	RF	83.15	Liver	No
ERX10645117	19670035	Paired	0.927127	0.0746509	FR	87.4	Small intestine	No
ERX10645130	23346214	Paired	0.100972	0.899018	RF	87.88	Large intestine	No
ERX9241823	17242820	Paired	0.0242216	0.976954	RF	93.9	Muscle	No
ERX8133414	15758739	Paired	0.0343975	0.965968	RF	73.73	Liver	No
ERX8133394	16400769	Paired	0.023401	0.977495	RF	83.9	Liver	No
ERX10645118	22735666	Paired	0.0724605	0.928518	RF	87.89	Small intestine	No
ERX10645131	23495860	Paired	0.0992629	0.900544	RF	88.64	Large intestine	No
ERX9241824	17583177	Paired	0.0214864	0.979853	RF	94.17	Muscle	No
ERX8133415	14798240	Paired	0.0267585	0.974733	RF	75.26	Liver	No
ERX8133395	15212018	Paired	0.0239657	0.977083	RF	84.86	Liver	No
ERX10645119	29725595	Paired	0.908053	0.0926038	FR	86.69	Small intestine	No
ERX10645132	27026481	Paired	0.92707	0.0731556	FR	89.09	Large intestine	No
ERX9241825	16779354	Paired	0.0233967	0.977975	RF	94.33	Muscle	No
ERX8133416	15810634	Paired	0.0199217	0.981509	RF	82.76	Liver	No
ERX8133396	18127219	Paired	0.0218199	0.979283	RF	80.32	Liver	No
ERX10645133	26022703	Paired	0.900329	0.0997526	FR	87.48	Large intestine	No
ERX9241826	16183186	Paired	0.0259504	0.974967	RF	94.07	Muscle	No
ERX8133430	13248898	Paired	0.0325477	0.968744	RF	76.43	Liver	No
ERX8133417	15142074	Paired	0.0212326	0.979787	RF	83.15	Liver	No
ERX8133397	17277683	Paired	0.0202556	0.980712	RF	85.05	Liver	No
ERX10645134	22997409	Paired	0.927971	0.0720145	FR	89.9	Large intestine	No
ERX9241840	16671009	Paired	0.0210199	0.980404	RF	94.04	Muscle	No
ERX9241827	19739954	Paired	0.0239306	0.977697	RF	68.21	Muscle	No
ERX8133431	17717917	Paired	0.022792	0.978556	RF	76.05	Liver	No
ERX8133418	14817240	Paired	0.0218705	0.979268	RF	83.06	Liver	No
ERX8133398	15151744	Paired	0.0246071	0.976832	RF	80.35	Liver	No
ERX10645135	23767116	Paired	0.0821477	0.917936	RF	89.11	Large intestine	No
ERX9241841	16065522	Paired	0.025777	0.97518	RF	94.43	Muscle	No
ERX9241828	16453702	Paired	0.0255499	0.975497	RF	94.09	Muscle	No
ERX8133432	13531424	Paired	0.0273158	0.973667	RF	75.49	Liver	No
ERX8133399	14720456	Paired	0.02172	0.979118	RF	82.74	Liver	No
ERX10645136	24479672	Paired	0.0705268	0.929473	RF	90.51	Large intestine	No
ERX9241842	15820619	Paired	0.0237962	0.977131	RF	93.88	Muscle	No
ERX9241829	15216172	Paired	0.0238483	0.977107	RF	93.79	Muscle	No
ERX8133433	14535424	Paired	0.0257909	0.975383	RF	74.55	Liver	No
ERX10645150	21996756	Paired	0.0748295	0.926692	RF	88.77	Small intestine	No
ERX10645137	26075393	Paired	0.0714879	0.928257	RF	89.88	Large intestine	No
ERX9241843	18314548	Paired	0.0259792	0.974813	RF	72.59	Muscle	No
ERX8133434	17551049	Paired	0.0244929	0.976754	RF	78.71	Liver	No
ERX10645151	22726293	Paired	0.0703362	0.931145	RF	89.12	Small intestine	No
ERX10645138	22891902	Paired	0.930593	0.0693954	FR	88.97	Large intestine	No
ERX9241844	16027696	Paired	0.0254894	0.975855	RF	94.31	Muscle	No
ERX8133435	13154813	Paired	0.0279261	0.972813	RF	63.67	Liver	No
ERX10645152	25407289	Paired	0.0721323	0.928735	RF	88.32	Small intestine	No
ERX10645139	30163190	Paired	0.903085	0.0978329	FR	87.13	Large intestine	No
ERX9241845	15724093	Paired	0.0247173	0.976492	RF	93.96	Muscle	No
ERX8133436	17764255	Paired	0.0236468	0.977611	RF	80.26	Liver	No
ERX10645153	23634970	Paired	0.934971	0.0663374	FR	89.85	Small intestine	No
ERX9241846	15157299	Paired	0.0223424	0.978608	RF	93.91	Muscle	No
ERX8133450	18659133	Paired	0.0196023	0.981356	RF	81.21	Liver	No

ERX8133437	14215474	Paired	0.0264484	0.974575	RF	81.24	Liver	No
ERX9241860	15583794	Paired	0.0237774	0.97721	RF	94.85	Muscle	No
ERX9241847	14938317	Paired	0.0241661	0.976696	RF	93.96	Muscle	No
ERX8133451	17906915	Paired	0.023574	0.977795	RF	77.55	Liver	No
ERX8133438	14926237	Paired	0.0229282	0.978225	RF	81.92	Liver	No
ERX10645155	23914514	Paired	0.924853	0.0762735	FR	88.75	Small intestine	No
ERX9241861	14718882	Paired	0.019418	0.981678	RF	94.43	Muscle	No
ERX9241848	16289436	Paired	0.0223969	0.978507	RF	94.11	Muscle	No
ERX8133439	15901713	Paired	0.0241727	0.977169	RF	78.81	Liver	No
ERX10645156	25507093	Paired	0.933375	0.0672161	FR	88.87	Small intestine	No
ERX9241862	15305702	Paired	0.0215625	0.979279	RF	94.53	Muscle	No
ERX9241849	14657323	Paired	0.0205876	0.980463	RF	94.29	Muscle	No
ERX10645170	26212168	Paired	0.0749349	0.925184	RF	90.34	Large intestine	No
ERX10645157	29827503	Paired	0.0639624	0.936722	RF	89.74	Small intestine	No
ERX9241863	15630332	Paired	0.0222069	0.978954	RF	94.33	Muscle	No
ERX10645171	23478661	Paired	0.0670174	0.932691	RF	90.67	Large intestine	No
ERX10645158	22688926	Paired	0.901135	0.0999676	FR	85.97	Small intestine	No
ERX10645172	27263391	Paired	0.923686	0.0760114	FR	89.94	Large intestine	No
ERX10645159	21591411	Paired	0.0738355	0.928482	RF	88.76	Small intestine	No
ERX10645173	29774834	Paired	0.932393	0.0671901	FR	90.6	Large intestine	No
ERX10645174	25963248	Paired	0.928676	0.0714235	FR	90.55	Large intestine	No
ERX10645175	24712309	Paired	0.919341	0.0808152	FR	89.76	Large intestine	No
ERX10645176	23192734	Paired	0.104214	0.897184	RF	88.99	Large intestine	No
ERX10645210	25198563	Paired	0.0716905	0.927958	RF	90.45	Large intestine	No
ERX10645190	23694398	Paired	0.923991	0.07616	FR	88.53	Small intestine	No
ERX10645177	23209656	Paired	0.100144	0.899275	RF	88.97	Large intestine	No
ERX10645211	22939373	Paired	0.919439	0.0804677	FR	88.91	Large intestine	No
ERX10645191	22205143	Paired	0.927303	0.0738708	FR	89.53	Small intestine	No
ERX10645178	21212001	Paired	0.900347	0.0993083	FR	88.58	Large intestine	No
ERX10645212	21841512	Paired	0.0867403	0.912768	RF	90.69	Large intestine	No
ERX10645192	23152095	Paired	0.0796463	0.921119	RF	88.93	Small intestine	No
ERX10645179	22474027	Paired	0.922044	0.077771	FR	89.63	Large intestine	No
ERX10645213	23112655	Paired	0.0856687	0.914784	RF	89.87	Large intestine	No
ERX10645193	29380962	Paired	0.0690639	0.931933	RF	90.01	Small intestine	No
ERX10645214	23078186	Paired	0.0797563	0.920027	RF	89.52	Large intestine	No
ERX10645194	27006857	Paired	0.927038	0.073269	FR	88.94	Small intestine	No
ERX10645215	28324536	Paired	0.931164	0.068627	FR	91.01	Large intestine	No
ERX10645195	20604685	Paired	0.931044	0.0698374	FR	89.61	Small intestine	No
ERX10645216	21983231	Paired	0.92632	0.0737489	FR	90.37	Large intestine	No
ERX10645196	27448229	Paired	0.927349	0.0730422	FR	89.45	Small intestine	No
ERX10645217	22338297	Paired	0.0798791	0.919523	RF	90.83	Large intestine	No
ERX10645197	26488593	Paired	0.92101	0.0801743	FR	90	Small intestine	No
ERX10645218	28572708	Paired	0.918601	0.081058	FR	90.03	Large intestine	No
ERX10645198	22583244	Paired	0.0770159	0.924419	RF	88.75	Small intestine	No
ERX10645219	25786017	Paired	0.0931343	0.906462	RF	89.12	Large intestine	No
ERX10645199	22899884	Paired	0.0894911	0.910859	RF	89.21	Small intestine	No
ERX10645100	23101309	Paired	0.0756867	0.925192	RF	87.02	Small intestine	No
ERX10645101	24433061	Paired	0.0748312	0.925999	RF	88.22	Small intestine	No
ERX10645102	24553409	Paired	0.917663	0.0836281	FR	86.69	Small intestine	No
ERX10645103	22922644	Paired	0.0947346	0.906389	RF	87.44	Small intestine	No
ERX8133400	16868849	Paired	0.0220494	0.978955	RF	85.16	Liver	No
ERX10645104	23175526	Paired	0.900206	0.10025	FR	86.45	Large intestine	No
ERX9241810	13859469	Paired	0.0299514	0.971203	RF	94.43	Muscle	No
ERX8133401	15720195	Paired	0.02249	0.978813	RF	79.47	Liver	No
ERX8133381	19009495	Paired	0.020902	0.980005	RF	83.69	Liver	No
ERX10645105	34689290	Paired	0.925091	0.0754727	FR	88.36	Large intestine	No
ERX8133402	17490054	Paired	0.0261068	0.974102	RF	69.25	Liver	No
ERX8133382	21545269	Paired	0.0220629	0.978858	RF	85.08	Liver	No

ERX10645106	32401643	Paired	0.087446	0.912998	RF	87.78	Large intestine	No
ERX9241812	14177317	Paired	0.0248323	0.976276	RF	94.37	Muscle	No
ERX9241792	14837113	Paired	0.0212257	0.979615	RF	82.95	Muscle	No
ERX8133403	15664288	Paired	0.0240648	0.976805	RF	78.19	Liver	No
ERX8133383	16589418	Paired	0.0218773	0.979104	RF	88.06	Liver	No
ERX10645107	26119662	Paired	0.922448	0.0776452	FR	89.22	Large intestine	No
ERX10645120	25155839	Paired	0.0836357	0.917661	RF	86.37	Small intestine	No
ERX9241813	13335724	Paired	0.0245671	0.976873	RF	94.38	Muscle	No
ERX9241793	16313241	Paired	0.0217793	0.978953	RF	94.65	Muscle	No
ERX8133404	19601907	Paired	0.0219857	0.979011	RF	79.74	Liver	No
ERX8133384	16200178	Paired	0.0204283	0.980671	RF	87.96	Liver	No
ERX10645108	29753389	Paired	0.0929927	0.908261	RF	86.95	Small intestine	No
ERX10645121	27785407	Paired	0.897547	0.103217	FR	85.66	Small intestine	No
ERX9241814	14973603	Paired	0.027099	0.974311	RF	94.15	Muscle	No
ERX9241794	14491308	Paired	0.028093	0.972771	RF	94.05	Muscle	No
ERX8133405	18435704	Paired	0.0242699	0.977069	RF	80.93	Liver	No
ERX8133385	16546976	Paired	0.0229224	0.978021	RF	85.96	Liver	No
ERX10645109	30741847	Paired	0.0723316	0.929102	RF	87.95	Small intestine	No
ERX10645122	29383055	Paired	0.0868147	0.914524	RF	87.96	Small intestine	No
ERX9241815	16922460	Paired	0.0273239	0.974009	RF	94.15	Muscle	No
ERX9241795	15974992	Paired	0.0313455	0.969364	RF	93.97	Muscle	No
ERX8133406	15809629	Paired	0.0225021	0.978469	RF	81.96	Liver	No
ERX8133386	18477419	Paired	0.0204487	0.980513	RF	84.39	Liver	No
ERX10645123	35539736	Paired	0.0765244	0.924418	RF	88.21	Small intestine	No
ERX9241816	19499778	Paired	0.0260647	0.975182	RF	93.65	Muscle	No
ERX8133420	13005850	Paired	0.0237969	0.977053	RF	83.43	Liver	No
ERX8133387	22304102	Paired	0.0186244	0.982381	RF	86.46	Liver	No
ERX10645124	24300351	Paired	0.921424	0.0797893	FR	87.89	Small intestine	No
ERX9241830	15143264	Paired	0.0256185	0.97559	RF	94.01	Muscle	No
ERX9241817	17812834	Paired	0.0244745	0.976543	RF	94.25	Muscle	No
ERX9241797	14533491	Paired	0.0340873	0.966741	RF	94.71	Muscle	No
ERX8133421	14104509	Paired	0.0224493	0.978579	RF	82.79	Liver	No
ERX8133408	15590045	Paired	0.0287665	0.972443	RF	65.02	Liver	No
ERX8133388	17669186	Paired	0.0199499	0.980922	RF	84.85	Liver	No
ERX10645125	24349310	Paired	0.0774192	0.923112	RF	87.99	Small intestine	No
ERX9241831	15677742	Paired	0.025169	0.976028	RF	93.44	Muscle	No
ERX9241818	16508699	Paired	0.0236123	0.977714	RF	93.98	Muscle	No
ERX9241798	14724069	Paired	0.0289189	0.972186	RF	94.27	Muscle	No
ERX8133422	14082811	Paired	0.02041	0.980563	RF	85.82	Liver	No
ERX8133409	17129096	Paired	0.022302	0.978701	RF	83.98	Liver	No
ERX8133389	11687464	Paired	0.0311958	0.970379	RF	80.55	Liver	No
ERX10645126	21818429	Paired	0.931371	0.069795	FR	88.39	Small intestine	No
ERX9241819	17961724	Paired	0.0230597	0.978046	RF	93.84	Muscle	No
ERX9241799	14343745	Paired	0.0251625	0.975665	RF	94.3	Muscle	No
ERX8133423	14098490	Paired	0.0211151	0.980111	RF	83.61	Liver	No
ERX10645140	23190020	Paired	0.0621679	0.938196	RF	90.62	Small intestine	No
ERX10645127	24810175	Paired	0.0773901	0.924437	RF	86.44	Small intestine	No
ERX9241833	13721915	Paired	0.025689	0.975471	RF	93.76	Muscle	No
ERX8133424	16119683	Paired	0.0202768	0.980905	RF	82.62	Liver	No
ERX10645141	29309217	Paired	0.0909118	0.910537	RF	88.15	Small intestine	No
ERX10645128	21950263	Paired	0.916827	0.0845391	FR	87.13	Large intestine	No
ERX9241834	13784651	Paired	0.024904	0.976352	RF	93.7	Muscle	No
ERX10645142	24630883	Paired	0.928931	0.0715261	FR	89.83	Small intestine	No
ERX10645129	23252492	Paired	0.0916929	0.908971	RF	88.27	Large intestine	No
ERX9241835	14006999	Paired	0.0259028	0.975237	RF	94.04	Muscle	No
ERX8133426	14338514	Paired	0.0198919	0.981063	RF	84.29	Liver	No
ERX10645143	24824504	Paired	0.0732406	0.927162	RF	89.7	Small intestine	No
ERX9241836	14514916	Paired	0.0237726	0.977495	RF	94.12	Muscle	No

ERX8133427	15111886	Paired	0.0227594	0.978786	RF	83.83	Liver	No
ERX10645144	26912617	Paired	0.0994176	0.901707	RF	87.81	Small intestine	No
ERX9241850	14719913	Paired	0.0193388	0.981584	RF	94.14	Muscle	No
ERX8133441	16431952	Paired	0.0195961	0.981381	RF	84.25	Liver	No
ERX10645145	32583958	Paired	0.905363	0.0953033	FR	87.7	Small intestine	No
ERX9241851	15616387	Paired	0.0223708	0.978414	RF	93.8	Muscle	No
ERX9241838	12970029	Paired	0.0235324	0.977639	RF	94.24	Muscle	No
ERX8133442	16541744	Paired	0.0220319	0.979105	RF	83.32	Liver	No
ERX8133429	14801777	Paired	0.0254194	0.975837	RF	82.72	Liver	No
ERX10645146	28696834	Paired	0.0999808	0.901488	RF	87.97	Small intestine	No
ERX9241852	14699905	Paired	0.0227046	0.978284	RF	94.18	Muscle	No
ERX9241839	13015029	Paired	0.0268427	0.973836	RF	94.36	Muscle	No
ERX8133443	16387997	Paired	0.0195243	0.981448	RF	83.67	Liver	No
ERX10645160	23316489	Paired	0.929285	0.0704008	FR	90.8	Large intestine	No
ERX10645147	24541988	Paired	0.927337	0.0731681	FR	89.09	Small intestine	No
ERX9241853	15514696	Paired	0.0232121	0.977654	RF	94.88	Muscle	No
ERX8133444	16171107	Paired	0.0214997	0.97969	RF	82.07	Liver	No
ERX10645161	23662494	Paired	0.0782154	0.921823	RF	90.83	Large intestine	No
ERX10645148	22245765	Paired	0.9382	0.0627107	FR	89.78	Small intestine	No
ERX10075980	35041869	Paired	0.967445	0.0318867	FR	79.44	Muscle	No
ERX10075981	40901577	Paired	0.968217	0.0313884	FR	76.17	Muscle	No
ERX10075940	25856405	Paired	0.968285	0.0309429	FR	77.1	Muscle	No
ERX10075982	43187775	Paired	0.965903	0.0327524	FR	77.68	Muscle	No
ERX10075941	26128509	Paired	0.962919	0.035913	FR	79.82	Muscle	No
ERX10075983	36735710	Paired	0.967361	0.03147	FR	81.06	Muscle	No
ERX10075942	29457590	Paired	0.968324	0.0308944	FR	75.85	Muscle	No
ERX10075984	31461118	Paired	0.966555	0.0321869	FR	77.26	Muscle	No
ERX10075943	31520675	Paired	0.965883	0.0322179	FR	76.91	Muscle	No
ERX10075985	38810883	Paired	0.966721	0.0325443	FR	77.37	Muscle	No
ERX10075944	29985945	Paired	0.968491	0.0304496	FR	78.01	Muscle	No
ERX10075986	38772837	Paired	0.968915	0.0306622	FR	75.25	Muscle	No
ERX10075945	32745587	Paired	0.967495	0.032936	FR	75.11	Muscle	No
ERX10076790	37382517	Paired	0.951824	0.0443948	FR	72.46	Small intestine	No
ERX10075987	41794966	Paired	0.97016	0.029725	FR	77.17	Muscle	No
ERX10075946	27144178	Paired	0.968495	0.0315232	FR	78.75	Muscle	No
ERX10076791	27230877	Paired	0.952389	0.0442648	FR	72.37	Small intestine	No
ERX10076750	49447722	Paired	0.957033	0.0381794	FR	72.75	Small intestine	No
ERX10075988	29452468	Paired	0.964327	0.0348106	FR	80.07	Muscle	No
ERX10075947	55391268	Paired	0.971412	0.0277482	FR	76.29	Muscle	No
ERX10076792	48334408	Paired	0.957259	0.0397269	FR	68.06	Small intestine	No
ERX10076751	43971382	Paired	0.962088	0.037578	FR	75.12	Small intestine	No
ERX10076710	26994933	Paired	0.947606	0.0499689	FR	75.37	Small intestine	No
ERX10075989	27604974	Paired	0.965959	0.0327241	FR	79.49	Muscle	No
ERX10075948	42481923	Paired	0.968073	0.0308513	FR	80.2	Muscle	No
ERX10076793	38258770	Paired	0.957095	0.0376166	FR	72.14	Small intestine	No
ERX10076752	39497805	Paired	0.957273	0.0398881	FR	71.36	Small intestine	No
ERX10076711	42278482	Paired	0.946149	0.0488575	FR	74.7	Small intestine	No
ERX10075949	27387465	Paired	0.966331	0.0312195	FR	80.88	Muscle	No
ERX10076794	48428083	Paired	0.960667	0.0379006	FR	73.21	Small intestine	No
ERX10076753	35959473	Paired	0.957825	0.0385033	FR	75.5	Small intestine	No
ERX10076712	46852206	Paired	0.952013	0.0467611	FR	76.84	Small intestine	No
ERX10076795	41002123	Paired	0.958162	0.039883	FR	75.79	Small intestine	No
ERX10076754	54465616	Paired	0.963007	0.0356649	FR	75.53	Small intestine	No
ERX10076713	49119564	Paired	0.927357	0.0656142	FR	69.65	Small intestine	No
ERX10076480	53567098	Paired	0.961239	0.038277	FR	71.9	Liver	No
ERX10076796	38788465	Paired	0.960133	0.0379945	FR	75.64	Small intestine	No
ERX10076755	35263254	Paired	0.955929	0.0396783	FR	74.81	Small intestine	No
ERX10076714	44007313	Paired	0.958521	0.0410537	FR	78.23	Small intestine	No

ERX10076481	51353195	Paired	0.955878	0.042505	FR	68.99	Liver	No
ERX10076440	43368783	Paired	0.964638	0.0324531	FR	82.1	Liver	No
ERX10076797	34189643	Paired	0.956969	0.0404683	FR	72.62	Small intestine	No
ERX10076756	36370781	Paired	0.955281	0.0414949	FR	70.95	Small intestine	No
ERX10076715	31520842	Paired	0.942881	0.0528364	FR	74.09	Small intestine	No
ERX10076482	37698206	Paired	0.961467	0.036555	FR	79.73	Liver	No
ERX10076441	32943466	Paired	0.958948	0.0369978	FR	80.77	Liver	No
ERX10076400	43952278	Paired	0.957347	0.0421148	FR	79.38	Liver	No
ERX10076798	27917817	Paired	0.959237	0.0385804	FR	74.39	Small intestine	No
ERX10076757	45985448	Paired	0.956184	0.0405763	FR	73.19	Small intestine	No
ERX10076716	38357040	Paired	0.947317	0.0521933	FR	75.49	Small intestine	No
ERX10076483	40729338	Paired	0.959842	0.0376934	FR	80.43	Liver	No
ERX10076442	37807593	Paired	0.956709	0.0397968	FR	71.86	Liver	No
ERX10076401	27546186	Paired	0.966108	0.0342349	FR	80.78	Liver	No
ERX10076799	36177011	Paired	0.957778	0.0399247	FR	73.45	Small intestine	No
ERX10076758	33366864	Paired	0.958202	0.0402928	FR	76.71	Small intestine	No
ERX10076717	40583484	Paired	0.951442	0.0476	FR	75.87	Small intestine	No
ERX10076484	36629929	Paired	0.953024	0.0445507	FR	78.03	Liver	No
ERX10076443	44151628	Paired	0.959699	0.0371676	FR	77.48	Liver	No
ERX10076402	32450114	Paired	0.955787	0.0424422	FR	75.95	Liver	No
ERX10076759	46989436	Paired	0.956467	0.0401008	FR	75.55	Small intestine	No
ERX10076718	35007386	Paired	0.95937	0.0423273	FR	72.94	Small intestine	No
ERX10076485	40450651	Paired	0.953702	0.0434141	FR	72.62	Liver	No
ERX10076444	33896992	Paired	0.955229	0.0412749	FR	66.45	Liver	No
ERX10076403	35852124	Paired	0.96669	0.0319463	FR	78.3	Liver	No
ERX10076170	52514476	Paired	0.970006	0.0298849	FR	71.52	Muscle	No
ERX10076719	33749749	Paired	0.947397	0.0488039	FR	72.42	Small intestine	No
ERX10076486	36826232	Paired	0.955018	0.0420766	FR	76.6	Liver	No
ERX10076445	40044916	Paired	0.964482	0.033837	FR	72.18	Liver	No
ERX10076404	39862996	Paired	0.96331	0.0359145	FR	78.59	Liver	No
ERX10076171	35350571	Paired	0.968402	0.0313343	FR	71.15	Muscle	No
ERX10076130	42064401	Paired	0.973077	0.0283196	FR	63.72	Muscle	No
ERX10076487	40648447	Paired	0.953218	0.0442372	FR	77.05	Liver	No
ERX10076446	34267275	Paired	0.959329	0.0376475	FR	71.17	Liver	No
ERX10076405	40964707	Paired	0.958418	0.0414362	FR	76.82	Liver	No
ERX10076131	82105009	Paired	0.975572	0.0251177	FR	71.11	Muscle	No
ERX10076172	54447381	Paired	0.970279	0.0307876	FR	69.77	Muscle	No
ERX10076488	45814712	Paired	0.953622	0.0452047	FR	74.26	Liver	No
ERX10076447	37681234	Paired	0.961471	0.0348697	FR	69.74	Liver	No
ERX10076406	31165101	Paired	0.969508	0.0303382	FR	81.91	Liver	No
ERX10076173	54998176	Paired	0.970576	0.0297845	FR	64.99	Muscle	No
ERX10076448	31909522	Paired	0.964154	0.0345658	FR	79.83	Liver	No
ERX10076407	53899038	Paired	0.962197	0.0369769	FR	80.83	Liver	No
ERX10076133	57826002	Paired	0.970528	0.029679	FR	60.85	Muscle	No
ERX10076174	40746222	Paired	0.967365	0.0325475	FR	69.5	Muscle	No
ERX10076449	38035093	Paired	0.961456	0.0353409	FR	79.49	Liver	No
ERX10076408	41254753	Paired	0.945548	0.0519956	FR	75.19	Liver	No
ERX10076134	50737576	Paired	0.974638	0.0261119	FR	61.8	Muscle	No
ERX10076175	57564275	Paired	0.967857	0.0314748	FR	61.74	Muscle	No
ERX10076409	42310342	Paired	0.972347	0.0277767	FR	82.44	Liver	No
ERX10076135	72488705	Paired	0.975989	0.0239669	FR	71.4	Muscle	No
ERX10076176	39657625	Paired	0.964729	0.0345252	FR	68.45	Muscle	No
ERX10076136	42430777	Paired	0.981056	0.019876	FR	81.76	Muscle	No
ERX10076177	39639912	Paired	0.969207	0.031111	FR	68.67	Muscle	No
ERX10076137	35889411	Paired	0.979604	0.0215289	FR	84.37	Muscle	No
ERX10076178	51777652	Paired	0.967942	0.0317089	FR	67.19	Muscle	No
ERX10076138	48959293	Paired	0.980199	0.0213681	FR	67.87	Muscle	No
ERX10076179	27630006	Paired	0.967795	0.0316476	FR	68.78	Muscle	No

ERX10076139	45781580	Paired	0.981344	0.0204368	FR	77.33	Muscle	No
ERX10076830	33357947	Paired	0.95337	0.0411749	FR	75.75	Small intestine	No
ERX10076831	41442530	Paired	0.953278	0.0423198	FR	76.38	Small intestine	No
ERX10076832	44887818	Paired	0.955074	0.0404854	FR	76.05	Small intestine	No
ERX10076833	55643678	Paired	0.952032	0.0414631	FR	73.64	Small intestine	No
ERX10076834	53463203	Paired	0.953827	0.0415562	FR	73.98	Small intestine	No
ERX10076560	51623514	Paired	0.958498	0.0398257	FR	76.53	Small intestine	No
ERX10076835	45980170	Paired	0.957827	0.0385992	FR	78.47	Small intestine	No
ERX10076561	44601773	Paired	0.93537	0.0566542	FR	76.65	Small intestine	No
ERX10076520	44547258	Paired	0.954792	0.0419339	FR	67.42	Liver	No
ERX10076836	26312152	Paired	0.963441	0.0356471	FR	77.28	Small intestine	No
ERX10076562	42365591	Paired	0.963245	0.0390487	FR	71.62	Small intestine	No
ERX10076521	61009572	Paired	0.950155	0.0455093	FR	70.84	Liver	No
ERX10076837	26523210	Paired	0.963508	0.0355535	FR	78.7	Small intestine	No
ERX10076563	71153471	Paired	0.966043	0.0336795	FR	73.83	Small intestine	No
ERX10076522	49294799	Paired	0.957778	0.0393264	FR	74.78	Liver	No
ERX10076838	27718566	Paired	0.964577	0.0338804	FR	81.44	Small intestine	No
ERX10076564	38808312	Paired	0.947297	0.0500614	FR	74.96	Small intestine	No
ERX10076523	48787736	Paired	0.956815	0.0404007	FR	73.26	Liver	No
ERX10076290	63589046	Paired	0.96397	0.0377653	FR	81.86	Liver	No
ERX10076839	26265264	Paired	0.963502	0.0355836	FR	81.09	Small intestine	No
ERX10076565	67347377	Paired	0.937676	0.0575088	FR	73.03	Small intestine	No
ERX10076524	40072510	Paired	0.955778	0.0413698	FR	73.68	Liver	No
ERX10076291	62450982	Paired	0.913735	0.0848674	FR	67.82	Liver	No
ERX10076250	84864996	Paired	0.961829	0.0389462	FR	80.98	Liver	No
ERX10076566	31869601	Paired	0.941019	0.0520069	FR	77.56	Small intestine	No
ERX10076525	39472705	Paired	0.961527	0.0365165	FR	76.15	Liver	No
ERX10076292	64668464	Paired	0.886115	0.104576	FR	62.41	Liver	No
ERX10076251	26456487	Paired	0.907451	0.0771644	FR	70.37	Liver	No
ERX10076210	40258998	Paired	0.968583	0.0311308	FR	61.46	Muscle	No
ERX10076567	42908341	Paired	0.944235	0.0514119	FR	72.28	Small intestine	No
ERX10076526	40771096	Paired	0.95578	0.0423483	FR	77.43	Liver	No
ERX10076293	30471620	Paired	0.933965	0.0638872	FR	73.42	Liver	No
ERX10076252	37420059	Paired	0.949746	0.0515139	FR	80.1	Liver	No
ERX10076211	49503684	Paired	0.967821	0.0320417	FR	68.44	Muscle	No
ERX10076568	29262402	Paired	0.905222	0.0649823	FR	64.11	Small intestine	No
ERX10076527	36597215	Paired	0.962394	0.0372703	FR	80.47	Liver	No
ERX10076294	41864964	Paired	0.922096	0.0737284	FR	71.89	Liver	No
ERX10076253	41895183	Paired	0.941127	0.0593252	FR	77.42	Liver	No
ERX10076212	42218183	Paired	0.968795	0.0301809	FR	64.69	Muscle	No
ERX10076569	28148380	Paired	0.919655	0.0692479	FR	68.35	Small intestine	No
ERX10076528	35970326	Paired	0.958115	0.0398005	FR	68.1	Liver	No
ERX10076295	42496744	Paired	0.949383	0.0516276	FR	77.49	Liver	No
ERX10076254	43756185	Paired	0.951913	0.0464455	FR	78.23	Liver	No
ERX10076213	40844222	Paired	0.967471	0.0316937	FR	61.14	Muscle	No
ERX10076529	47313119	Paired	0.95344	0.0427228	FR	71.26	Liver	No
ERX10076296	40327923	Paired	0.928318	0.0697048	FR	72.22	Liver	No
ERX10076255	39732414	Paired	0.952086	0.0485732	FR	80.17	Liver	No
ERX10076214	43787068	Paired	0.965818	0.0328062	FR	63.64	Muscle	No
ERX10076297	35679508	Paired	0.922518	0.0730864	FR	76.71	Liver	No
ERX10076256	31583778	Paired	0.907842	0.0673149	FR	66.44	Liver	No
ERX10076215	37421961	Paired	0.964091	0.0342929	FR	65.13	Muscle	No
ERX10076298	30305515	Paired	0.944815	0.0557592	FR	80.29	Liver	No
ERX10076258	67977242	Paired	0.927656	0.0645763	FR	72.59	Liver	No
ERX10076259	44964309	Paired	0.957019	0.0426419	FR	80.1	Liver	No
ERX10076219	33725067	Paired	0.969725	0.0295562	FR	68.37	Muscle	No
ERX10076680	39532966	Paired	0.959804	0.0411483	FR	73.62	Small intestine	No
ERX10076681	41690503	Paired	0.972215	0.0282735	FR	76.86	Small intestine	No

ERX10076640	32085368	Paired	0.926625	0.0641537	FR	65.41	Small intestine	No
ERX10076682	55298405	Paired	0.934504	0.0546137	FR	71.5	Small intestine	No
ERX10076641	47388552	Paired	0.956762	0.0410054	FR	75.01	Small intestine	No
ERX10076600	46759815	Paired	0.93912	0.0519499	FR	72.83	Small intestine	No
ERX10076683	45112881	Paired	0.916333	0.0684126	FR	69.17	Small intestine	No
ERX10076642	48885105	Paired	0.960619	0.0384575	FR	74.21	Small intestine	No
ERX10076601	41424756	Paired	0.951757	0.0459934	FR	76.79	Small intestine	No
ERX10076643	35217332	Paired	0.926462	0.0655726	FR	67.44	Small intestine	No
ERX10076602	45385828	Paired	0.950825	0.0451453	FR	72.55	Small intestine	No
ERX10076685	47101211	Paired	0.926816	0.062616	FR	71.49	Small intestine	No
ERX10076603	32757881	Paired	0.949658	0.0475698	FR	76.96	Small intestine	No
ERX10076370	29111002	Paired	0.961918	0.038577	FR	77.98	Liver	No
ERX10076686	41466322	Paired	0.940494	0.0559142	FR	70.65	Small intestine	No
ERX10076645	47545217	Paired	0.960114	0.0397772	FR	77.54	Small intestine	No
ERX10076604	36610538	Paired	0.96353	0.0363819	FR	75	Small intestine	No
ERX10076371	38141084	Paired	0.969432	0.0309947	FR	78.74	Liver	No
ERX10076330	33820977	Paired	0.959166	0.0419351	FR	79.2	Liver	No
ERX10076687	35788913	Paired	0.953663	0.0457578	FR	74.11	Small intestine	No
ERX10076646	42753716	Paired	0.956319	0.0422499	FR	78.56	Small intestine	No
ERX10076605	38167997	Paired	0.957987	0.0400371	FR	79.21	Small intestine	No
ERX10076372	47666824	Paired	0.967304	0.0342324	FR	74.77	Liver	No
ERX10076331	38091342	Paired	0.964907	0.0361516	FR	81.62	Liver	No
ERX10076688	48739485	Paired	0.95028	0.0449719	FR	75.73	Small intestine	No
ERX10076647	40706087	Paired	0.954409	0.0448274	FR	73.18	Small intestine	No
ERX10076606	56624127	Paired	0.961059	0.0382355	FR	74.39	Small intestine	No
ERX10076373	42329939	Paired	0.967823	0.0334196	FR	80.48	Liver	No
ERX10076332	33686089	Paired	0.968783	0.0323844	FR	82.29	Liver	No
ERX10076689	36470239	Paired	0.957756	0.0413706	FR	72.88	Small intestine	No
ERX10076648	36800309	Paired	0.968727	0.0317792	FR	77.97	Small intestine	No
ERX10076607	37647797	Paired	0.963794	0.0366635	FR	75.82	Small intestine	No
ERX10076374	37065508	Paired	0.96462	0.036547	FR	79.61	Liver	No
ERX10076333	28919976	Paired	0.968299	0.0330798	FR	85.25	Liver	No
ERX10076649	36863411	Paired	0.963709	0.036102	FR	78.39	Small intestine	No
ERX10076608	44565163	Paired	0.958699	0.0403052	FR	75.17	Small intestine	No
ERX10076375	36950897	Paired	0.962617	0.0380751	FR	81.29	Liver	No
ERX10076334	45459427	Paired	0.963112	0.0377929	FR	80.95	Liver	No
ERX10076060	43008406	Paired	0.97184	0.0280307	FR	67.7	Muscle	No
ERX10076609	47237110	Paired	0.945057	0.0473201	FR	75.87	Small intestine	No
ERX10076376	41699515	Paired	0.952124	0.0471679	FR	78.02	Liver	No
ERX10076335	30585934	Paired	0.943932	0.0531625	FR	78.28	Liver	No
ERX10076061	41104269	Paired	0.973221	0.0274455	FR	67.62	Muscle	No
ERX10076020	29163981	Paired	0.968046	0.0308604	FR	81.96	Muscle	No
ERX10076377	39471970	Paired	0.939811	0.0588764	FR	77.82	Liver	No
ERX10076336	28103138	Paired	0.960424	0.0405809	FR	80.68	Liver	No
ERX10076062	47271931	Paired	0.971534	0.0275532	FR	66.53	Muscle	No
ERX10076021	49864536	Paired	0.970454	0.0279916	FR	82.32	Muscle	No
ERX10076378	38713963	Paired	0.966047	0.0336701	FR	79.77	Liver	No
ERX10076337	25415241	Paired	0.957146	0.0416268	FR	80.1	Liver	No
ERX10076063	57744791	Paired	0.971826	0.0276628	FR	64.92	Muscle	No
ERX10076022	30021714	Paired	0.964623	0.033563	FR	81.98	Muscle	No
ERX10076379	50143725	Paired	0.969181	0.0305539	FR	82.45	Liver	No
ERX10076338	53225856	Paired	0.942954	0.0539133	FR	76.42	Liver	No
ERX10076064	41001052	Paired	0.972819	0.0282113	FR	68.05	Muscle	No
ERX10076023	44561482	Paired	0.969381	0.0290663	FR	81.8	Muscle	No
ERX10076339	36548665	Paired	0.937291	0.0618636	FR	77.07	Liver	No
ERX10076065	41445403	Paired	0.973644	0.0266934	FR	67.18	Muscle	No
ERX10076024	26301394	Paired	0.966154	0.0327458	FR	80.28	Muscle	No
ERX10076066	48729500	Paired	0.969806	0.0301348	FR	68.05	Muscle	No

ERX10076025	66980892	Paired	0.964996	0.0333415	FR	80.56	Muscle	No
ERX10076067	58303563	Paired	0.971461	0.0289778	FR	65.85	Muscle	No
ERX10076026	49881506	Paired	0.968322	0.0307429	FR	75.79	Muscle	No
ERX10076068	52929806	Paired	0.973277	0.0267082	FR	69.03	Muscle	No
ERX10076027	32255654	Paired	0.969326	0.0298036	FR	81.48	Muscle	No
ERX10076069	43629815	Paired	0.973435	0.0271878	FR	68.66	Muscle	No
ERX10076028	42285614	Paired	0.966587	0.0305895	FR	81.64	Muscle	No
ERX10076029	28722704	Paired	0.965882	0.0320652	FR	81.85	Muscle	No
ERX10075990	31945307	Paired	0.966981	0.0312691	FR	81.82	Muscle	No
ERX10075950	37325748	Paired	0.967037	0.0310042	FR	77.18	Muscle	No
ERX10075991	36439610	Paired	0.968263	0.0304442	FR	81.57	Muscle	No
ERX10075992	30580833	Paired	0.966315	0.0317442	FR	75.84	Muscle	No
ERX10075952	36078180	Paired	0.968942	0.0305278	FR	78.08	Muscle	No
ERX10075993	50099498	Paired	0.967143	0.030631	FR	75.94	Muscle	No
ERX10075953	35454134	Paired	0.968258	0.0311551	FR	77.81	Muscle	No
ERX10075994	36762298	Paired	0.968706	0.0304515	FR	79.48	Muscle	No
ERX10075954	35941751	Paired	0.966789	0.0327522	FR	75.36	Muscle	No
ERX10075995	38097695	Paired	0.967687	0.0311452	FR	79.81	Muscle	No
ERX10075955	30690936	Paired	0.970079	0.0292602	FR	76.77	Muscle	No
ERX10075996	26176899	Paired	0.967426	0.0315563	FR	76.87	Muscle	No
ERX10075956	37247150	Paired	0.968034	0.0307354	FR	76.16	Muscle	No
ERX10075997	35560096	Paired	0.9688	0.0299252	FR	80.35	Muscle	No
ERX10076760	48177206	Paired	0.949245	0.0445381	FR	75.32	Small intestine	No
ERX10075957	42278846	Paired	0.971429	0.0278692	FR	78.66	Muscle	No
ERX10075998	32720198	Paired	0.968856	0.0301714	FR	81.18	Muscle	No
ERX10076761	41598391	Paired	0.949646	0.0461599	FR	76.12	Small intestine	No
ERX10076720	38536825	Paired	0.966908	0.0332506	FR	77.43	Small intestine	No
ERX10075958	34305703	Paired	0.968474	0.0305449	FR	80.21	Muscle	No
ERX10075999	43554024	Paired	0.966657	0.032241	FR	79.32	Muscle	No
ERX10076762	43473208	Paired	0.964929	0.0346182	FR	77.86	Small intestine	No
ERX10076721	25901486	Paired	0.946459	0.0529125	FR	77.38	Small intestine	No
ERX10075959	32428457	Paired	0.960969	0.0378021	FR	77.31	Muscle	No
ERX10076763	51246616	Paired	0.947046	0.0465509	FR	77.82	Small intestine	No
ERX10076722	29829219	Paired	0.934891	0.0583998	FR	72.91	Small intestine	No
ERX10076764	33409107	Paired	0.959597	0.0384683	FR	71.78	Small intestine	No
ERX10076723	28554820	Paired	0.958012	0.0413213	FR	75.31	Small intestine	No
ERX10076490	50271831	Paired	0.955908	0.0416974	FR	74.95	Liver	No
ERX10076765	30953605	Paired	0.96059	0.0391483	FR	78.48	Small intestine	No
ERX10076724	34853034	Paired	0.924798	0.0660149	FR	71.98	Small intestine	No
ERX10076491	67294045	Paired	0.961638	0.0368973	FR	68.56	Liver	No
ERX10076450	46113801	Paired	0.95247	0.0425435	FR	62.08	Liver	No
ERX10076766	33545846	Paired	0.958494	0.0399012	FR	75.16	Small intestine	No
ERX10076725	38972700	Paired	0.9568	0.0424158	FR	78.41	Small intestine	No
ERX10076492	35396584	Paired	0.963278	0.0354357	FR	73.26	Liver	No
ERX10076451	34941495	Paired	0.962885	0.0357767	FR	75.6	Liver	No
ERX10076410	41225684	Paired	0.964981	0.0343464	FR	80.09	Liver	No
ERX10076726	43052008	Paired	0.948676	0.0488878	FR	79.05	Small intestine	No
ERX10076493	56279888	Paired	0.965128	0.0345708	FR	77.72	Liver	No
ERX10076452	36488170	Paired	0.959597	0.038229	FR	72.4	Liver	No
ERX10076411	38673284	Paired	0.961994	0.0358522	FR	77.55	Liver	No
ERX10076768	40733744	Paired	0.954977	0.0433225	FR	71.32	Small intestine	No
ERX10076727	38082523	Paired	0.940913	0.0560688	FR	73.62	Small intestine	No
ERX10076494	61448520	Paired	0.980294	0.0206721	FR	87.41	Liver	No
ERX10076453	41146993	Paired	0.962103	0.0354671	FR	71.14	Liver	No
ERX10076412	47853090	Paired	0.967021	0.0331298	FR	80.97	Liver	No
ERX10076769	33629380	Paired	0.956035	0.0411976	FR	76.18	Small intestine	No
ERX10076728	39576415	Paired	0.954156	0.0427241	FR	77.67	Small intestine	No
ERX10076495	57454107	Paired	0.959365	0.0403523	FR	73.03	Liver	No

ERX10076454	31650600	Paired	0.965755	0.0340775	FR	82.8	Liver	No
ERX10076413	34985668	Paired	0.963294	0.0361748	FR	81.96	Liver	No
ERX10076180	47182902	Paired	0.969363	0.0311855	FR	68.7	Muscle	No
ERX10076729	40341544	Paired	0.935024	0.0615913	FR	71.48	Small intestine	No
ERX10076496	54416845	Paired	0.943499	0.0522557	FR	70.85	Liver	No
ERX10076455	35540816	Paired	0.961102	0.0365683	FR	68.73	Liver	No
ERX10076414	32283314	Paired	0.966718	0.0326842	FR	83.28	Liver	No
ERX10076181	50501587	Paired	0.968858	0.0312124	FR	68.45	Muscle	No
ERX10076140	41601647	Paired	0.971952	0.0294694	FR	74.4	Muscle	No
ERX10076497	57945545	Paired	0.965042	0.0342462	FR	78.45	Liver	No
ERX10076456	49041636	Paired	0.965233	0.0339863	FR	80.38	Liver	No
ERX10076415	40823418	Paired	0.968431	0.0296316	FR	78.17	Liver	No
ERX10076182	59318105	Paired	0.970366	0.0293757	FR	69.73	Muscle	No
ERX10076100	60720629	Paired	0.972885	0.027939	FR	68.69	Muscle	No
ERX10076498	59180685	Paired	0.963289	0.0359968	FR	72.7	Liver	No
ERX10076457	37742694	Paired	0.968291	0.0313441	FR	83.37	Liver	No
ERX10076416	39676361	Paired	0.968224	0.0313124	FR	81.74	Liver	No
ERX10076183	53337492	Paired	0.968879	0.0303272	FR	66.48	Muscle	No
ERX10076142	62823211	Paired	0.970317	0.0287177	FR	61.46	Muscle	No
ERX10076101	66471809	Paired	0.97607	0.0246216	FR	69.94	Muscle	No
ERX10076499	65167212	Paired	0.953959	0.0442388	FR	63.05	Liver	No
ERX10076458	37426586	Paired	0.964848	0.0341843	FR	78.1	Liver	No
ERX10076417	35611297	Paired	0.955687	0.0423337	FR	81	Liver	No
ERX10076184	44436488	Paired	0.969441	0.0304134	FR	64.4	Muscle	No
ERX10076143	52869268	Paired	0.97161	0.0293892	FR	71.23	Muscle	No
ERX10076102	65435500	Paired	0.973518	0.0280005	FR	65.79	Muscle	No
ERX10076459	42158242	Paired	0.958804	0.0388474	FR	75.56	Liver	No
ERX10076418	38032558	Paired	0.957032	0.0413148	FR	79.64	Liver	No
ERX10076185	33360736	Paired	0.965234	0.0346271	FR	64.43	Muscle	No
ERX10076144	36302954	Paired	0.969537	0.0316371	FR	71.58	Muscle	No
ERX10076103	82723999	Paired	0.975595	0.0258694	FR	65.88	Muscle	No
ERX10076419	36189229	Paired	0.962368	0.0344641	FR	78.73	Liver	No
ERX10076186	43414458	Paired	0.968726	0.0299498	FR	72.45	Muscle	No
ERX10076145	36019128	Paired	0.967204	0.0337913	FR	70.07	Muscle	No
ERX10076104	52841985	Paired	0.974215	0.0273618	FR	64.75	Muscle	No
ERX10076187	39879467	Paired	0.954486	0.044211	FR	68.1	Muscle	No
ERX10076146	43556965	Paired	0.969407	0.0310524	FR	70.26	Muscle	No
ERX10076105	85000091	Paired	0.975348	0.0257196	FR	66.98	Muscle	No
ERX10076188	50216094	Paired	0.971077	0.0294549	FR	66.94	Muscle	No
ERX10076147	48894473	Paired	0.970336	0.0292133	FR	62.18	Muscle	No
ERX10076106	49295884	Paired	0.974969	0.0261859	FR	67.27	Muscle	No
ERX10076189	48254965	Paired	0.968293	0.0309758	FR	62.28	Muscle	No
ERX10076148	56518488	Paired	0.969155	0.0311951	FR	72.76	Muscle	No
ERX10076107	92942862	Paired	0.975332	0.0256396	FR	66.75	Muscle	No
ERX10076149	70429245	Paired	0.967904	0.032719	FR	71.19	Muscle	No
ERX10076108	57384790	Paired	0.97536	0.0254984	FR	69.41	Muscle	No
ERX10076109	80998480	Paired	0.972492	0.0283159	FR	65.34	Muscle	No
ERX10076800	43100483	Paired	0.952626	0.0427208	FR	70.46	Small intestine	No
ERX10076801	37323941	Paired	0.956014	0.0388732	FR	73.41	Small intestine	No
ERX10076802	46418002	Paired	0.959321	0.0383294	FR	74.59	Small intestine	No
ERX10076803	34489220	Paired	0.964005	0.035298	FR	74.46	Small intestine	No
ERX10076570	34136935	Paired	0.886145	0.0786087	FR	66	Small intestine	No
ERX10076804	33091391	Paired	0.960659	0.0384039	FR	74.66	Small intestine	No
ERX10076571	59548195	Paired	0.968117	0.0322883	FR	79.1	Small intestine	No
ERX10076530	60707586	Paired	0.949176	0.0472034	FR	66.43	Liver	No
ERX10076805	35291962	Paired	0.95835	0.0387023	FR	74.58	Small intestine	No
ERX10076572	40788855	Paired	0.949144	0.0470182	FR	74.08	Small intestine	No
ERX10076531	33781875	Paired	0.957521	0.0398628	FR	75.62	Liver	No

ERX10076806	31134530	Paired	0.957785	0.0389836	FR	77.43	Small intestine	No
ERX10076573	33567458	Paired	0.942552	0.0549047	FR	73.32	Small intestine	No
ERX10076532	39778943	Paired	0.957271	0.039692	FR	67.48	Liver	No
ERX10076807	36093238	Paired	0.952596	0.0429273	FR	73.57	Small intestine	No
ERX10076574	30965454	Paired	0.942624	0.0500585	FR	72.53	Small intestine	No
ERX10076533	42528065	Paired	0.951346	0.0442641	FR	66.15	Liver	No
ERX10076808	48582825	Paired	0.958053	0.0402064	FR	72.01	Small intestine	No
ERX10076575	34841730	Paired	0.95729	0.0414642	FR	79.52	Small intestine	No
ERX10076534	45703290	Paired	0.951449	0.0451248	FR	77.45	Liver	No
ERX10076260	42073127	Paired	0.952888	0.0465129	FR	79.29	Liver	No
ERX10076809	45875358	Paired	0.95691	0.0384328	FR	75.52	Small intestine	No
ERX10076576	35494553	Paired	0.921797	0.0682414	FR	69.65	Small intestine	No
ERX10076535	61843221	Paired	0.976663	0.0245117	FR	85.62	Liver	No
ERX10076261	42282704	Paired	0.949968	0.0507686	FR	77.95	Liver	No
ERX10076577	37945850	Paired	0.951643	0.0462803	FR	74.13	Small intestine	No
ERX10076536	36905189	Paired	0.97648	0.0241493	FR	82.38	Liver	No
ERX10076262	45674789	Paired	0.94909	0.0481673	FR	78.53	Liver	No
ERX10076578	34420997	Paired	0.958859	0.0401826	FR	75.19	Small intestine	No
ERX10076537	30479859	Paired	0.974224	0.0256645	FR	81.75	Liver	No
ERX10076263	41873082	Paired	0.952788	0.0454938	FR	77.79	Liver	No
ERX10076579	38251230	Paired	0.964512	0.0349164	FR	76.14	Small intestine	No
ERX10076538	26458868	Paired	0.973539	0.0267115	FR	81.37	Liver	No
ERX10076264	51325714	Paired	0.956709	0.0443951	FR	81.47	Liver	No
ERX10076223	34691893	Paired	0.970308	0.0293294	FR	72.39	Muscle	No
ERX10076539	25219106	Paired	0.97193	0.0276239	FR	83.44	Liver	No
ERX10076265	35234673	Paired	0.952089	0.0476074	FR	80.4	Liver	No
ERX10076224	36469234	Paired	0.967392	0.0313065	FR	68.13	Muscle	No
ERX10076266	53234410	Paired	0.963113	0.037865	FR	81.2	Liver	No
ERX10076225	28184672	Paired	0.965482	0.0335223	FR	62.61	Muscle	No
ERX10076267	43573489	Paired	0.938389	0.0531742	FR	72.02	Liver	No
ERX10076268	48568552	Paired	0.952083	0.0474566	FR	80.7	Liver	No
ERX10076269	45423609	Paired	0.949464	0.053872	FR	79.95	Liver	No
ERX10076229	49603475	Paired	0.966761	0.0317735	FR	60.04	Muscle	No
ERX10076690	31002441	Paired	0.924809	0.0642805	FR	72.2	Small intestine	No
ERX10076691	32877579	Paired	0.954686	0.0441452	FR	80.9	Small intestine	No
ERX10076650	27014819	Paired	0.944455	0.0475079	FR	69.97	Small intestine	No
ERX10076692	30829023	Paired	0.939132	0.0567478	FR	70.3	Small intestine	No
ERX10076651	26599527	Paired	0.947732	0.05225	FR	75.18	Small intestine	No
ERX10076610	61656843	Paired	0.968004	0.0323534	FR	73.81	Small intestine	No
ERX10076693	40315216	Paired	0.920109	0.0680787	FR	71.99	Small intestine	No
ERX10076652	41334389	Paired	0.945175	0.0491124	FR	73.39	Small intestine	No
ERX10076611	55963020	Paired	0.936193	0.053882	FR	72.96	Small intestine	No
ERX10076694	42839111	Paired	0.940871	0.0558022	FR	72.05	Small intestine	No
ERX10076653	37464399	Paired	0.911553	0.0733846	FR	70.49	Small intestine	No
ERX10076612	80569785	Paired	0.969357	0.0306972	FR	79.72	Small intestine	No
ERX10076695	39657508	Paired	0.961891	0.0379534	FR	80.47	Small intestine	No
ERX10076613	33491624	Paired	0.917608	0.061247	FR	69.47	Small intestine	No
ERX10076380	56386585	Paired	0.953267	0.0472896	FR	77.31	Liver	No
ERX10076696	27864020	Paired	0.954168	0.0480363	FR	72.33	Small intestine	No
ERX10076655	34184857	Paired	0.963661	0.0363878	FR	77.88	Small intestine	No
ERX10076381	61668686	Paired	0.966901	0.0334717	FR	81.24	Liver	No
ERX10076340	38879752	Paired	0.942494	0.0581943	FR	70.63	Liver	No
ERX10076697	41818698	Paired	0.942886	0.0553304	FR	73.75	Small intestine	No
ERX10076656	32457283	Paired	0.96317	0.0372656	FR	77.24	Small intestine	No
ERX10076615	36193825	Paired	0.956529	0.0428701	FR	75.97	Small intestine	No
ERX10076382	64337664	Paired	0.958682	0.0395935	FR	75.95	Liver	No
ERX10076341	49089591	Paired	0.964814	0.0366773	FR	77	Liver	No
ERX10076300	40722959	Paired	0.934172	0.0637832	FR	78.91	Liver	No

ERX10076698	29241589	Paired	0.927018	0.053117	FR	70.22	Small intestine	No
ERX10076657	42514223	Paired	0.952298	0.0441187	FR	73.89	Small intestine	No
ERX10076616	48039761	Paired	0.955235	0.042468	FR	76.7	Small intestine	No
ERX10076383	55554436	Paired	0.958888	0.0405951	FR	76.94	Liver	No
ERX10076342	42026748	Paired	0.970891	0.0313704	FR	77.68	Liver	No
ERX10076301	49356577	Paired	0.937223	0.0606296	FR	78.14	Liver	No
ERX10076699	41921939	Paired	0.940749	0.0554331	FR	70.63	Small intestine	No
ERX10076658	41612368	Paired	0.967965	0.0319107	FR	77.28	Small intestine	No
ERX10076617	31849301	Paired	0.953406	0.045763	FR	69.72	Small intestine	No
ERX10076384	56484497	Paired	0.964157	0.0363033	FR	80.31	Liver	No
ERX10076343	39520098	Paired	0.959995	0.0445646	FR	77.91	Liver	No
ERX10076302	46951995	Paired	0.934974	0.0634336	FR	75.44	Liver	No
ERX10076659	48094915	Paired	0.95466	0.0428753	FR	72.13	Small intestine	No
ERX10076618	33373812	Paired	0.955205	0.0442865	FR	73.86	Small intestine	No
ERX10076385	52509775	Paired	0.952852	0.0461569	FR	73.51	Liver	No
ERX10076344	42202353	Paired	0.963852	0.0386792	FR	79.16	Liver	No
ERX10076070	44226063	Paired	0.974695	0.0263958	FR	72.72	Muscle	No
ERX10076303	43368396	Paired	0.943638	0.0527612	FR	74.83	Liver	No
ERX10076619	42545573	Paired	0.951671	0.0456519	FR	75.17	Small intestine	No
ERX10076386	47175849	Paired	0.953149	0.0456251	FR	76.71	Liver	No
ERX10076345	46296199	Paired	0.960934	0.0423698	FR	78.78	Liver	No
ERX10076071	42065948	Paired	0.971808	0.0286554	FR	72.84	Muscle	No
ERX10076304	61424052	Paired	0.959081	0.0422383	FR	81.17	Liver	No
ERX10076030	29184615	Paired	0.959097	0.0392332	FR	78.58	Muscle	No
ERX10076387	61973191	Paired	0.941218	0.0562655	FR	79.95	Liver	No
ERX10076346	36012533	Paired	0.970353	0.0300726	FR	77.56	Liver	No
ERX10076072	27988819	Paired	0.974365	0.0267479	FR	69.81	Muscle	No
ERX10076031	41323668	Paired	0.966129	0.032422	FR	81.32	Muscle	No
ERX10076388	51169615	Paired	0.954738	0.0441454	FR	79.8	Liver	No
ERX10076347	45928655	Paired	0.96643	0.0350548	FR	75.02	Liver	No
ERX10076073	42639072	Paired	0.974295	0.0266667	FR	65.66	Muscle	No
ERX10076306	40820761	Paired	0.950843	0.0493404	FR	78.36	Liver	No
ERX10076032	28783374	Paired	0.966845	0.0325774	FR	74.5	Muscle	No
ERX10076389	37365204	Paired	0.941723	0.0565254	FR	75.22	Liver	No
ERX10076348	48929014	Paired	0.959434	0.0428452	FR	71.81	Liver	No
ERX10076074	50785804	Paired	0.973248	0.0272208	FR	68.65	Muscle	No
ERX10076307	46438739	Paired	0.949005	0.0497636	FR	79.89	Liver	No
ERX10076033	31540992	Paired	0.965302	0.0332865	FR	79.5	Muscle	No
ERX10076349	41247491	Paired	0.96537	0.0368113	FR	79.38	Liver	No
ERX10076075	52574485	Paired	0.971937	0.0284045	FR	69.48	Muscle	No
ERX10076308	56758520	Paired	0.968253	0.0329152	FR	84.7	Liver	No
ERX10076034	31192775	Paired	0.966475	0.0324704	FR	77.89	Muscle	No
ERX10076076	52243225	Paired	0.970643	0.029336	FR	68.21	Muscle	No
ERX10076309	61146951	Paired	0.965812	0.0344176	FR	79.65	Liver	No
ERX10076035	32153791	Paired	0.967733	0.031048	FR	77.96	Muscle	No
ERX10076077	29372632	Paired	0.974382	0.02678	FR	70.6	Muscle	No
ERX10076036	51899251	Paired	0.976747	0.0248012	FR	80.3	Muscle	No
ERX10076078	42904678	Paired	0.973456	0.0271488	FR	69.42	Muscle	No
ERX10076037	49912065	Paired	0.97708	0.0231949	FR	80.53	Muscle	No
ERX10076079	47060169	Paired	0.975904	0.0251271	FR	68.31	Muscle	No
ERX10076038	41746181	Paired	0.97227	0.0278275	FR	78.85	Muscle	No
ERX10076039	44574804	Paired	0.976185	0.0244737	FR	79.77	Muscle	No
ERX10075960	30838283	Paired	0.966674	0.0322548	FR	79.01	Muscle	No
ERX10075961	28806014	Paired	0.967861	0.0308288	FR	76.85	Muscle	No
ERX10075962	36115084	Paired	0.967274	0.0316019	FR	77.61	Muscle	No
ERX10075963	64773674	Paired	0.96636	0.0322952	FR	75.43	Muscle	No
ERX10075964	33212978	Paired	0.968445	0.0313014	FR	80.77	Muscle	No
ERX10075965	40885468	Paired	0.9678	0.0313822	FR	79.35	Muscle	No

ERX10075966	43998878	Paired	0.967324	0.0315547	FR	77.17	Muscle	No
ERX10076770	37674225	Paired	0.961547	0.0379639	FR	75.21	Small intestine	No
ERX10075967	44569586	Paired	0.969029	0.0303821	FR	78.05	Muscle	No
ERX10076771	31122496	Paired	0.962632	0.0362922	FR	77.5	Small intestine	No
ERX10076730	38379362	Paired	0.956896	0.0418952	FR	77.16	Small intestine	No
ERX10075968	47624380	Paired	0.965301	0.0334067	FR	75.57	Muscle	No
ERX10076772	39546264	Paired	0.958365	0.0393991	FR	75.88	Small intestine	No
ERX10076731	30634565	Paired	0.942237	0.0549595	FR	73.38	Small intestine	No
ERX10075969	45830738	Paired	0.969899	0.0299948	FR	77.36	Muscle	No
ERX10076773	46615860	Paired	0.94495	0.0493171	FR	78.12	Small intestine	No
ERX10076732	34282019	Paired	0.931776	0.0643985	FR	71.13	Small intestine	No
ERX10076774	50439179	Paired	0.953925	0.0419685	FR	74.4	Small intestine	No
ERX10076733	38581436	Paired	0.94283	0.0542267	FR	74.94	Small intestine	No
ERX10076775	35287212	Paired	0.958004	0.040077	FR	75.02	Small intestine	No
ERX10076734	38839427	Paired	0.939677	0.0554969	FR	72.56	Small intestine	No
ERX10076460	42828084	Paired	0.962721	0.0353636	FR	80.24	Liver	No
ERX10076776	49158608	Paired	0.940359	0.0518409	FR	74.13	Small intestine	No
ERX10076735	93401602	Paired	0.957411	0.0418148	FR	75.68	Small intestine	No
ERX10076461	36407598	Paired	0.96732	0.0318664	FR	84.18	Liver	No
ERX10076420	44250745	Paired	0.958521	0.0395722	FR	72.48	Liver	No
ERX10076777	47372238	Paired	0.955986	0.0409089	FR	73.64	Small intestine	No
ERX10076736	37100115	Paired	0.944691	0.0522561	FR	74.02	Small intestine	No
ERX10076462	35382318	Paired	0.954581	0.0424291	FR	73.18	Liver	No
ERX10076421	40530459	Paired	0.968336	0.0307348	FR	80.12	Liver	No
ERX10076778	104239941	Paired	0.958546	0.0398548	FR	75.89	Small intestine	No
ERX10076737	36989901	Paired	0.930513	0.0596702	FR	72.04	Small intestine	No
ERX10076463	40878366	Paired	0.9624	0.0360638	FR	80.88	Liver	No
ERX10076422	34875240	Paired	0.966962	0.0323923	FR	82.26	Liver	No
ERX10076779	39341588	Paired	0.962753	0.0361771	FR	73.9	Small intestine	No
ERX10076738	46011679	Paired	0.932596	0.0572543	FR	70.39	Small intestine	No
ERX10076464	51494630	Paired	0.96339	0.0351925	FR	75.39	Liver	No
ERX10076423	51982494	Paired	0.968102	0.0302082	FR	82.02	Liver	No
ERX10076739	49854448	Paired	0.960569	0.0368243	FR	75.71	Small intestine	No
ERX10076465	37415632	Paired	0.966635	0.0342784	FR	78.77	Liver	No
ERX10076424	45230571	Paired	0.96384	0.0357791	FR	78.86	Liver	No
ERX10076150	56357729	Paired	0.967262	0.0316	FR	70.16	Muscle	No
ERX10076191	34169791	Paired	0.968369	0.0310718	FR	69.83	Muscle	No
ERX10076466	47807548	Paired	0.949919	0.0469065	FR	73.33	Liver	No
ERX10076425	37883018	Paired	0.968379	0.0304455	FR	79.1	Liver	No
ERX10076151	68338436	Paired	0.969053	0.0308357	FR	69.96	Muscle	No
ERX10076110	81755671	Paired	0.97513	0.0270211	FR	67.95	Muscle	No
ERX10076467	51057500	Paired	0.944413	0.0535518	FR	64.8	Liver	No
ERX10076426	36584603	Paired	0.966399	0.0326735	FR	80.34	Liver	No
ERX10076111	63478497	Paired	0.976311	0.0250478	FR	67.08	Muscle	No
ERX10076468	40608297	Paired	0.964179	0.0359174	FR	77.06	Liver	No
ERX10076427	35966962	Paired	0.950672	0.044707	FR	76.37	Liver	No
ERX10076153	44526168	Paired	0.967428	0.0324829	FR	69.16	Muscle	No
ERX10076112	63033328	Paired	0.974529	0.0259238	FR	66.91	Muscle	No
ERX10076194	29811928	Paired	0.969811	0.030237	FR	68.89	Muscle	No
ERX10076469	41996200	Paired	0.962974	0.036536	FR	74.08	Liver	No
ERX10076428	41558047	Paired	0.962295	0.0365846	FR	78.64	Liver	No
ERX10076113	58457542	Paired	0.973671	0.0266124	FR	65.64	Muscle	No
ERX10076429	29296436	Paired	0.960317	0.0393389	FR	74.75	Liver	No
ERX10076155	75187974	Paired	0.969296	0.0304598	FR	67.84	Muscle	No
ERX10076114	83325192	Paired	0.973232	0.0266068	FR	66.12	Muscle	No
ERX10076156	39114721	Paired	0.964209	0.0345753	FR	71.25	Muscle	No
ERX10076115	73859109	Paired	0.97409	0.026712	FR	63.71	Muscle	No
ERX10076197	42759990	Paired	0.968076	0.0312947	FR	60.3	Muscle	No

ERX10076157	36740206	Paired	0.963774	0.035296	FR	62.5	Muscle	No
ERX10076116	45063834	Paired	0.971494	0.0294471	FR	64.21	Muscle	No
ERX10076158	35048050	Paired	0.963103	0.0350679	FR	60.77	Muscle	No
ERX10076117	54554512	Paired	0.973937	0.0271338	FR	69.35	Muscle	No
ERX10076199	47832940	Paired	0.96454	0.0337296	FR	60.78	Muscle	No
ERX10076118	80407466	Paired	0.974423	0.0258127	FR	65.37	Muscle	No
ERX10076159	36612925	Paired	0.965506	0.0331524	FR	68.18	Muscle	No
ERX10076119	85406527	Paired	0.975944	0.0248569	FR	76.04	Muscle	No
ERX10076810	48467386	Paired	0.961438	0.0370647	FR	74.39	Small intestine	No
ERX10076811	56842498	Paired	0.961135	0.0369585	FR	76.64	Small intestine	No
ERX10076812	39313183	Paired	0.955096	0.0411266	FR	73.45	Small intestine	No
ERX10076813	35205981	Paired	0.951405	0.0441871	FR	74.68	Small intestine	No
ERX10076580	34998713	Paired	0.937832	0.0570654	FR	70.57	Small intestine	No
ERX10076814	27384610	Paired	0.955113	0.0413218	FR	75.79	Small intestine	No
ERX10076581	35235920	Paired	0.954553	0.0432254	FR	76.91	Small intestine	No
ERX10076540	49072598	Paired	0.967848	0.03202	FR	76	Small intestine	No
ERX10076815	35377429	Paired	0.952599	0.0444143	FR	76.21	Small intestine	No
ERX10076582	36756733	Paired	0.928288	0.0643089	FR	72.68	Small intestine	No
ERX10076541	50167288	Paired	0.932175	0.054691	FR	71.9	Small intestine	No
ERX10076500	36176877	Paired	0.957497	0.0404395	FR	74.56	Liver	No
ERX10076816	55296298	Paired	0.943789	0.0481163	FR	70.7	Small intestine	No
ERX10076583	37425892	Paired	0.964946	0.0345792	FR	73.47	Small intestine	No
ERX10076542	59794343	Paired	0.939905	0.0530436	FR	68.57	Small intestine	No
ERX10076501	34989757	Paired	0.962042	0.0367369	FR	73.04	Liver	No
ERX10076817	52857562	Paired	0.946139	0.0469806	FR	75.87	Small intestine	No
ERX10076584	37372077	Paired	0.959509	0.0400758	FR	77.67	Small intestine	No
ERX10076543	49278658	Paired	0.9672	0.0326138	FR	76.31	Small intestine	No
ERX10076502	39885822	Paired	0.955344	0.0431798	FR	72.06	Liver	No
ERX10076818	41658421	Paired	0.949627	0.0457183	FR	75.86	Small intestine	No
ERX10076585	27829684	Paired	0.962005	0.0377916	FR	74.55	Small intestine	No
ERX10076544	46708377	Paired	0.961832	0.0387975	FR	74.65	Small intestine	No
ERX10076503	57699634	Paired	0.954153	0.0433764	FR	62.41	Liver	No
ERX10076270	73879431	Paired	0.960001	0.0422912	FR	79.56	Liver	No
ERX10076819	53492728	Paired	0.957028	0.0399887	FR	75.9	Small intestine	No
ERX10076586	43969147	Paired	0.946743	0.0490938	FR	73.13	Small intestine	No
ERX10076545	48816857	Paired	0.96009	0.038665	FR	81.1	Small intestine	No
ERX10076504	42924817	Paired	0.956257	0.0421118	FR	63.69	Liver	No
ERX10076271	47828349	Paired	0.958672	0.0418397	FR	78.61	Liver	No
ERX10076587	45381058	Paired	0.950221	0.0441118	FR	71.88	Small intestine	No
ERX10076546	27971855	Paired	0.94866	0.0510595	FR	72.18	Small intestine	No
ERX10076505	56132342	Paired	0.965392	0.0335445	FR	73.26	Liver	No
ERX10076272	39797271	Paired	0.880541	0.0989365	FR	65.24	Liver	No
ERX10076231	51535515	Paired	0.965294	0.0332978	FR	60.83	Muscle	No
ERX10076588	62857006	Paired	0.960547	0.0377136	FR	76.11	Small intestine	No
ERX10076547	40381514	Paired	0.940195	0.0553171	FR	75.66	Small intestine	No
ERX10076506	58529413	Paired	0.964622	0.0342268	FR	72.83	Liver	No
ERX10076273	51100475	Paired	0.952595	0.0475819	FR	78.24	Liver	No
ERX10076232	50298033	Paired	0.963969	0.034849	FR	63.8	Muscle	No
ERX10076589	52904864	Paired	0.948712	0.0452908	FR	78.14	Small intestine	No
ERX10076548	43461925	Paired	0.941395	0.0539424	FR	75.25	Small intestine	No
ERX10076507	65852950	Paired	0.955842	0.0422968	FR	68.57	Liver	No
ERX10076274	37149797	Paired	0.941732	0.0561358	FR	73.28	Liver	No
ERX10076233	50558420	Paired	0.968038	0.0310688	FR	68.79	Muscle	No
ERX10076549	29861488	Paired	0.959036	0.041506	FR	74.62	Small intestine	No
ERX10076508	36783071	Paired	0.957885	0.0411525	FR	71.03	Liver	No
ERX10076275	28656796	Paired	0.89045	0.0902347	FR	63.73	Liver	No
ERX10076234	52146040	Paired	0.970815	0.0283946	FR	67.8	Muscle	No
ERX10076509	51751133	Paired	0.963709	0.0350397	FR	74	Liver	No

ERX10076276	39181275	Paired	0.941872	0.0569636	FR	78	Liver	No
ERX10076235	50201546	Paired	0.968021	0.0304794	FR	66.76	Muscle	No
ERX10076277	44422735	Paired	0.952399	0.05026	FR	77.05	Liver	No
ERX10076236	65239817	Paired	0.976713	0.0237377	FR	66.15	Muscle	No
ERX10076278	37336732	Paired	0.935917	0.0649733	FR	75.53	Liver	No
ERX10076237	42995122	Paired	0.979212	0.02181	FR	75.68	Muscle	No
ERX10076279	39792368	Paired	0.953342	0.0468527	FR	76.05	Liver	No
ERX10076238	46438611	Paired	0.976773	0.0228904	FR	77.32	Muscle	No
ERX10076239	47607486	Paired	0.977923	0.022611	FR	62	Muscle	No
ERX10076660	48045772	Paired	0.93281	0.058721	FR	72.13	Small intestine	No
ERX10076661	44020622	Paired	0.948657	0.0491537	FR	70.17	Small intestine	No
ERX10076620	41980983	Paired	0.956252	0.0427422	FR	72.94	Small intestine	No
ERX10076662	54291246	Paired	0.956034	0.04194	FR	74.03	Small intestine	No
ERX10076621	42198713	Paired	0.969903	0.0305416	FR	73.04	Small intestine	No
ERX10076663	49274453	Paired	0.939748	0.0564926	FR	71.78	Small intestine	No
ERX10076622	45077973	Paired	0.954005	0.0453734	FR	71.83	Small intestine	No
ERX10076664	38922507	Paired	0.957538	0.0408623	FR	73.31	Small intestine	No
ERX10076623	38455689	Paired	0.947135	0.0494959	FR	72.1	Small intestine	No
ERX10076390	48916558	Paired	0.972444	0.0271795	FR	82.3	Liver	No
ERX10076665	42652410	Paired	0.907052	0.0768712	FR	65.92	Small intestine	No
ERX10076624	50046698	Paired	0.961252	0.0380495	FR	74.01	Small intestine	No
ERX10076391	45595064	Paired	0.960285	0.038325	FR	76.14	Liver	No
ERX10076350	43892052	Paired	0.966019	0.0355649	FR	77.37	Liver	No
ERX10076666	39224710	Paired	0.93772	0.0522389	FR	65.41	Small intestine	No
ERX10076625	28431255	Paired	0.955308	0.0435071	FR	75.91	Small intestine	No
ERX10076392	48016707	Paired	0.96361	0.0350124	FR	77.86	Liver	No
ERX10076351	43675743	Paired	0.951811	0.0508272	FR	74.77	Liver	No
ERX10076310	31284490	Paired	0.935232	0.0611494	FR	75.64	Liver	No
ERX10076667	42823681	Paired	0.955587	0.0427702	FR	75.59	Small intestine	No
ERX10076626	33708537	Paired	0.949174	0.0491671	FR	75.28	Small intestine	No
ERX10076393	41951350	Paired	0.967749	0.0321243	FR	81.2	Liver	No
ERX10076352	33871976	Paired	0.967461	0.0327545	FR	82.39	Liver	No
ERX10076311	49013671	Paired	0.970927	0.0303835	FR	84.1	Liver	No
ERX10076668	77369976	Paired	0.954181	0.0424817	FR	74.18	Small intestine	No
ERX10076627	45332129	Paired	0.961369	0.0378721	FR	79.2	Small intestine	No
ERX10076394	36880255	Paired	0.969788	0.0301347	FR	83.56	Liver	No
ERX10076353	33953942	Paired	0.957776	0.0416923	FR	78.6	Liver	No
ERX10076312	39619914	Paired	0.943911	0.0512818	FR	77.05	Liver	No
ERX10076669	73868758	Paired	0.955907	0.0409782	FR	77.74	Small intestine	No
ERX10076628	34830021	Paired	0.956224	0.0408611	FR	74.05	Small intestine	No
ERX10076395	42830419	Paired	0.966454	0.032397	FR	79.51	Liver	No
ERX10076354	30010640	Paired	0.955471	0.0419255	FR	73.52	Liver	No
ERX10076080	63231767	Paired	0.976299	0.0253609	FR	68.49	Muscle	No
ERX10076313	39851531	Paired	0.963357	0.0364754	FR	80.84	Liver	No
ERX10076396	51012913	Paired	0.971153	0.0287397	FR	80.84	Liver	No
ERX10076355	32911379	Paired	0.951398	0.0477724	FR	76.35	Liver	No
ERX10076081	39906286	Paired	0.974253	0.0272839	FR	68.33	Muscle	No
ERX10076314	35436617	Paired	0.95381	0.0431451	FR	78.66	Liver	No
ERX10076040	39947530	Paired	0.976141	0.025381	FR	74.51	Muscle	No
ERX10076397	49724763	Paired	0.954547	0.0458435	FR	78	Liver	No
ERX10076356	35922383	Paired	0.955197	0.0437702	FR	72.94	Liver	No
ERX10076082	48407359	Paired	0.971446	0.0285474	FR	69.21	Muscle	No
ERX10076315	42322076	Paired	0.966045	0.0341703	FR	83.04	Liver	No
ERX10076041	28459995	Paired	0.976288	0.0247628	FR	72.62	Muscle	No
ERX10076000	40149496	Paired	0.96134	0.0363243	FR	78.12	Muscle	No
ERX10076398	56702428	Paired	0.965094	0.0358946	FR	79.17	Liver	No
ERX10076357	41694166	Paired	0.962704	0.0369029	FR	76.45	Liver	No
ERX10076083	61005038	Paired	0.971121	0.0296407	FR	68.04	Muscle	No

ERX10076316	29906013	Paired	0.951238	0.0495989	FR	81.85	Liver	No
ERX10076042	42283771	Paired	0.973886	0.026564	FR	66.33	Muscle	No
ERX10076001	36455490	Paired	0.96249	0.0355003	FR	75.94	Muscle	No
ERX10076358	40522883	Paired	0.955409	0.0420677	FR	74.78	Liver	No
ERX10076084	49655486	Paired	0.972423	0.0275362	FR	69.3	Muscle	No
ERX10076317	100129455	Paired	0.962862	0.0360254	FR	80.12	Liver	No
ERX10076043	31553682	Paired	0.97361	0.0264005	FR	68.47	Muscle	No
ERX10076002	30355667	Paired	0.966797	0.0318663	FR	78.63	Muscle	No
ERX10076359	44980471	Paired	0.964468	0.0351854	FR	81.39	Liver	No
ERX10076085	34070694	Paired	0.973137	0.0271073	FR	68.51	Muscle	No
ERX10076318	102164395	Paired	0.968407	0.0313061	FR	81.86	Liver	No
ERX10076044	35903120	Paired	0.972367	0.0272156	FR	60.29	Muscle	No
ERX10076003	36379177	Paired	0.966549	0.0320267	FR	78.18	Muscle	No
ERX10076086	45456168	Paired	0.974031	0.0266949	FR	67.46	Muscle	No
ERX10076319	123684300	Paired	0.970154	0.0300453	FR	81.5	Liver	No
ERX10076045	31326526	Paired	0.972252	0.0281635	FR	69.15	Muscle	No
ERX10076004	32812440	Paired	0.966262	0.0326169	FR	77.59	Muscle	No
ERX10076087	53122880	Paired	0.97442	0.0264272	FR	66.98	Muscle	No
ERX10076046	38444832	Paired	0.972078	0.0275786	FR	66.29	Muscle	No
ERX10076005	49708708	Paired	0.966207	0.0323392	FR	82.06	Muscle	No
ERX10076047	39697030	Paired	0.971157	0.0283131	FR	64.51	Muscle	No
ERX10076006	36206040	Paired	0.967533	0.0309497	FR	80	Muscle	No
ERX10076089	41752739	Paired	0.975165	0.0266028	FR	68.19	Muscle	No
ERX10076048	46915177	Paired	0.97168	0.0279368	FR	65.04	Muscle	No
ERX10076007	39963142	Paired	0.970281	0.028132	FR	83.38	Muscle	No
ERX10076049	45229288	Paired	0.972426	0.0271141	FR	65.59	Muscle	No
ERX10076008	29649393	Paired	0.96535	0.0335275	FR	81.04	Muscle	No
ERX10076009	36582334	Paired	0.965513	0.0327782	FR	79.64	Muscle	No
ERX10075970	37934715	Paired	0.969395	0.0302175	FR	78.21	Muscle	No
ERX10075971	33700917	Paired	0.968783	0.0305768	FR	78.81	Muscle	No
ERX10075972	40333910	Paired	0.97083	0.0292432	FR	80.14	Muscle	No
ERX10075973	31606412	Paired	0.970149	0.0298129	FR	78.98	Muscle	No
ERX10075974	37147415	Paired	0.968483	0.0306806	FR	76.43	Muscle	No
ERX10075975	48663192	Paired	0.966858	0.032416	FR	79.61	Muscle	No
ERX10075976	39588416	Paired	0.96855	0.0304753	FR	78.58	Muscle	No
ERX10076780	35843506	Paired	0.963021	0.035807	FR	76.27	Small intestine	No
ERX10075977	37128681	Paired	0.968124	0.0310067	FR	79.68	Muscle	No
ERX10076781	35987787	Paired	0.958111	0.0400299	FR	74.83	Small intestine	No
ERX10076740	30918380	Paired	0.964437	0.0353199	FR	76.61	Small intestine	No
ERX10075978	36396600	Paired	0.968262	0.0306727	FR	77.07	Muscle	No
ERX10076782	31367809	Paired	0.953431	0.0437765	FR	76.13	Small intestine	No
ERX10076741	45353271	Paired	0.961818	0.0365515	FR	75.09	Small intestine	No
ERX10076700	38920341	Paired	0.936557	0.0603088	FR	73.52	Small intestine	No
ERX10075979	50814582	Paired	0.967715	0.0316208	FR	77.85	Muscle	No
ERX10076783	38683571	Paired	0.953945	0.0415592	FR	76.24	Small intestine	No
ERX10076742	31142345	Paired	0.963708	0.0362264	FR	78.71	Small intestine	No
ERX10076701	41162340	Paired	0.901519	0.0816201	FR	68.21	Small intestine	No
ERX10076784	51874217	Paired	0.956274	0.0404516	FR	74.49	Small intestine	No
ERX10076702	30811833	Paired	0.953607	0.0475169	FR	72.92	Small intestine	No
ERX10076785	51976113	Paired	0.955079	0.0416683	FR	75.85	Small intestine	No
ERX10076744	51387351	Paired	0.950976	0.0460212	FR	72.83	Small intestine	No
ERX10076703	43575043	Paired	0.952587	0.0457478	FR	75.4	Small intestine	No
ERX10076470	37572946	Paired	0.958767	0.040933	FR	74.82	Liver	No
ERX10076786	38973613	Paired	0.954672	0.0405791	FR	79.89	Small intestine	No
ERX10076745	49458729	Paired	0.952569	0.0420217	FR	72.46	Small intestine	No
ERX10076704	36153955	Paired	0.942476	0.0534683	FR	74.47	Small intestine	No
ERX10076471	43544914	Paired	0.960507	0.0392849	FR	79.37	Liver	No
ERX10076430	36655002	Paired	0.961453	0.0366552	FR	77.16	Liver	No

ERX10076787	30494333	Paired	0.962354	0.0367312	FR	75.22	Small intestine	No
ERX10076746	69164972	Paired	0.960826	0.0364992	FR	73.81	Small intestine	No
ERX10076705	38700005	Paired	0.955271	0.0465088	FR	75.11	Small intestine	No
ERX10076472	64010829	Paired	0.951339	0.0475524	FR	75.62	Liver	No
ERX10076431	36348564	Paired	0.962255	0.0355242	FR	75.26	Liver	No
ERX10076788	33756212	Paired	0.95894	0.039716	FR	71.35	Small intestine	No
ERX10076747	40229977	Paired	0.968124	0.0323023	FR	75.74	Small intestine	No
ERX10076706	27911258	Paired	0.922496	0.0609746	FR	70.51	Small intestine	No
ERX10076473	51437201	Paired	0.95229	0.0457821	FR	70.41	Liver	No
ERX10076432	40423526	Paired	0.964276	0.0344031	FR	75.85	Liver	No
ERX10076789	28543720	Paired	0.956294	0.040826	FR	74.66	Small intestine	No
ERX10076748	49988574	Paired	0.957266	0.0400229	FR	72.48	Small intestine	No
ERX10076707	31555213	Paired	0.957858	0.0438013	FR	75.29	Small intestine	No
ERX10076474	29263140	Paired	0.95022	0.0478042	FR	72.54	Liver	No
ERX10076433	40652553	Paired	0.94849	0.0489611	FR	78.53	Liver	No
ERX10076749	52123190	Paired	0.955307	0.0407039	FR	74.53	Small intestine	No
ERX10076708	25877955	Paired	0.921239	0.0687061	FR	70.49	Small intestine	No
ERX10076475	47640391	Paired	0.954241	0.043514	FR	74.03	Liver	No
ERX10076434	54215197	Paired	0.962556	0.0358894	FR	78.62	Liver	No
ERX10076160	107111191	Paired	0.967272	0.0323055	FR	70.48	Muscle	No
ERX10076709	37557687	Paired	0.941272	0.053737	FR	71.57	Small intestine	No
ERX10076476	40628109	Paired	0.949678	0.0473561	FR	76.33	Liver	No
ERX10076435	47036954	Paired	0.958197	0.0375649	FR	72.33	Liver	No
ERX10076161	44337804	Paired	0.964703	0.0336365	FR	68.62	Muscle	No
ERX10076120	54456152	Paired	0.974892	0.0261447	FR	66.37	Muscle	No
ERX10076477	36060937	Paired	0.940168	0.0548664	FR	68.36	Liver	No
ERX10076436	36704389	Paired	0.963081	0.0377664	FR	80.6	Liver	No
ERX10076121	122336488	Paired	0.97645	0.0244939	FR	68.93	Muscle	No
ERX10076478	30044173	Paired	0.943814	0.0537162	FR	75.76	Liver	No
ERX10076437	39200914	Paired	0.964887	0.0356539	FR	80.99	Liver	No
ERX10076163	26905613	Paired	0.965383	0.0338473	FR	65.08	Muscle	No
ERX10076122	49661543	Paired	0.974596	0.0268656	FR	68.13	Muscle	No
ERX10076479	35492160	Paired	0.951734	0.0458813	FR	74.39	Liver	No
ERX10076438	41613191	Paired	0.970376	0.0307751	FR	81.86	Liver	No
ERX10076164	46926392	Paired	0.969923	0.0297438	FR	69.07	Muscle	No
ERX10076123	65699219	Paired	0.9734	0.0272641	FR	66.91	Muscle	No
ERX10076439	31112373	Paired	0.968241	0.0326971	FR	81.93	Liver	No
ERX10076165	46084336	Paired	0.966733	0.0328396	FR	67.3	Muscle	No
ERX10076124	55649482	Paired	0.973296	0.0268784	FR	65.02	Muscle	No
ERX10076166	46719462	Paired	0.969891	0.0302169	FR	70.04	Muscle	No
ERX10076125	101424494	Paired	0.974748	0.025986	FR	64.56	Muscle	No
ERX10076167	39661263	Paired	0.967121	0.0325217	FR	71.7	Muscle	No
ERX10076126	50369035	Paired	0.974358	0.026587	FR	64.43	Muscle	No
ERX10076168	49454357	Paired	0.965443	0.0339936	FR	67.02	Muscle	No
ERX10076127	74744539	Paired	0.973686	0.0272998	FR	64.24	Muscle	No
ERX10076169	40629710	Paired	0.966873	0.0324533	FR	70.08	Muscle	No
ERX10076129	63964243	Paired	0.975616	0.0245606	FR	69.29	Muscle	No
ERX10076820	33466889	Paired	0.953355	0.0426219	FR	75.45	Small intestine	No
ERX10076821	32878486	Paired	0.946882	0.0461549	FR	75.1	Small intestine	No
ERX10076822	30186911	Paired	0.937851	0.0553291	FR	74.78	Small intestine	No
ERX10076823	43570590	Paired	0.9404	0.0504975	FR	75.27	Small intestine	No
ERX10076590	65802498	Paired	0.962384	0.0373632	FR	77.5	Small intestine	No
ERX10076824	44355435	Paired	0.959836	0.0377141	FR	75.75	Small intestine	No
ERX10076591	62691887	Paired	0.955206	0.0417962	FR	75.26	Small intestine	No
ERX10076550	61097057	Paired	0.94449	0.0564675	FR	66.4	Small intestine	No
ERX10076825	34569910	Paired	0.955634	0.0402686	FR	74.8	Small intestine	No
ERX10076592	57445886	Paired	0.96018	0.0385883	FR	76.61	Small intestine	No
ERX10076551	33990087	Paired	0.938042	0.0564865	FR	75.4	Small intestine	No

ERX10076510	52490052	Paired	0.962583	0.0362704	FR	73.68	Liver	No
ERX10076826	52701635	Paired	0.954792	0.0401684	FR	78.09	Small intestine	No
ERX10076593	36909688	Paired	0.935427	0.0563748	FR	72.38	Small intestine	No
ERX10076552	50953263	Paired	0.961839	0.0374845	FR	82.27	Small intestine	No
ERX10076511	60635375	Paired	0.950762	0.0452021	FR	68.91	Liver	No
ERX10076594	32586966	Paired	0.957247	0.0428231	FR	74.46	Small intestine	No
ERX10076553	37798494	Paired	0.939137	0.055251	FR	74.76	Small intestine	No
ERX10076512	37861622	Paired	0.960678	0.0380641	FR	76.38	Liver	No
ERX10076828	28245828	Paired	0.957343	0.0380805	FR	75.26	Small intestine	No
ERX10076595	76795244	Paired	0.968878	0.0313715	FR	74.13	Small intestine	No
ERX10076554	34092817	Paired	0.938631	0.0553382	FR	72.33	Small intestine	No
ERX10076513	33253386	Paired	0.964635	0.0354758	FR	76.36	Liver	No
ERX10076280	60014644	Paired	0.909939	0.0873929	FR	65.69	Liver	No
ERX10076829	32284073	Paired	0.944514	0.0490122	FR	74.45	Small intestine	No
ERX10076596	42538421	Paired	0.952188	0.0453456	FR	77.22	Small intestine	No
ERX10076514	46411239	Paired	0.955115	0.0409241	FR	72.85	Liver	No
ERX10076281	29809993	Paired	0.921878	0.0745064	FR	76.96	Liver	No
ERX10076597	34682284	Paired	0.949249	0.0494045	FR	75.65	Small intestine	No
ERX10076556	48788253	Paired	0.947003	0.0493142	FR	73.63	Small intestine	No
ERX10076515	51499798	Paired	0.949967	0.0461091	FR	72.68	Liver	No
ERX10076282	65877568	Paired	0.918946	0.0739087	FR	67.9	Liver	No
ERX10076241	48429566	Paired	0.951099	0.0485381	FR	81.32	Liver	No
ERX10076200	35049499	Paired	0.967965	0.0313369	FR	60.17	Muscle	No
ERX10076598	29414101	Paired	0.958427	0.0403714	FR	78.12	Small intestine	No
ERX10076516	38588514	Paired	0.909483	0.0776871	FR	69.02	Liver	No
ERX10076283	29709604	Paired	0.910278	0.0767255	FR	62.57	Liver	No
ERX10076242	36591741	Paired	0.963558	0.036571	FR	80.44	Liver	No
ERX10076201	41616198	Paired	0.970954	0.028386	FR	61.44	Muscle	No
ERX10076599	59215920	Paired	0.963026	0.0362974	FR	78.85	Small intestine	No
ERX10076558	32441703	Paired	0.943074	0.0464175	FR	74.02	Small intestine	No
ERX10076517	45489504	Paired	0.955619	0.0423136	FR	75.19	Liver	No
ERX10076284	41051384	Paired	0.903939	0.0807671	FR	70.48	Liver	No
ERX10076243	36383245	Paired	0.952297	0.0469994	FR	79.51	Liver	No
ERX10076559	62948878	Paired	0.962277	0.0368156	FR	75.33	Small intestine	No
ERX10076518	34567236	Paired	0.959328	0.0392486	FR	78.01	Liver	No
ERX10076285	37965117	Paired	0.93963	0.05748	FR	74.24	Liver	No
ERX10076244	32295098	Paired	0.95504	0.0458414	FR	77.67	Liver	No
ERX10076519	54967074	Paired	0.976037	0.0244685	FR	85.82	Liver	No
ERX10076286	45475622	Paired	0.942924	0.0578385	FR	78.42	Liver	No
ERX10076245	31296547	Paired	0.901135	0.0734941	FR	74.24	Liver	No
ERX10076204	45282567	Paired	0.967786	0.0315804	FR	60.04	Muscle	No
ERX10076287	45078022	Paired	0.959823	0.0411611	FR	82.53	Liver	No
ERX10076246	44223216	Paired	0.954246	0.0448599	FR	76.9	Liver	No
ERX10076288	49722568	Paired	0.950708	0.0489549	FR	78.21	Liver	No
ERX10076247	59551967	Paired	0.942655	0.0550842	FR	75.7	Liver	No
ERX10076248	37641314	Paired	0.936462	0.0612937	FR	75.56	Liver	No
ERX10076207	43513586	Paired	0.967044	0.0321709	FR	63.83	Muscle	No
ERX10076249	46828540	Paired	0.95933	0.0415996	FR	83.15	Liver	No
ERX10076208	38707787	Paired	0.966888	0.0317335	FR	60.25	Muscle	No
ERX10076209	48007567	Paired	0.968939	0.0301253	FR	60.53	Muscle	No
ERX10076670	62361751	Paired	0.89795	0.0787595	FR	69.75	Small intestine	No
ERX10076671	25471047	Paired	0.943885	0.0478567	FR	67.53	Small intestine	No
ERX10076630	46663349	Paired	0.971271	0.0289021	FR	71.99	Small intestine	No
ERX10076672	37761480	Paired	0.933426	0.0539736	FR	69.49	Small intestine	No
ERX10076631	41571943	Paired	0.881951	0.0756804	FR	63.3	Small intestine	No
ERX10076632	35181890	Paired	0.943986	0.0493887	FR	70.71	Small intestine	No
ERX10076674	57095448	Paired	0.943665	0.0551797	FR	71.19	Small intestine	No
ERX10076633	41383560	Paired	0.967497	0.0321865	FR	72.69	Small intestine	No

ERX10076675	33364242	Paired	0.968338	0.0321567	FR	73.99	Small intestine	No
ERX10076634	51614242	Paired	0.965363	0.0351937	FR	73.87	Small intestine	No
ERX10076360	40682720	Paired	0.971069	0.0286969	FR	82.35	Liver	No
ERX10076676	32134429	Paired	0.960871	0.0387724	FR	80.85	Small intestine	No
ERX10076635	27356618	Paired	0.876548	0.085208	FR	67.74	Small intestine	No
ERX10076361	34308155	Paired	0.967645	0.0317691	FR	82.04	Liver	No
ERX10076320	44945914	Paired	0.960516	0.0395917	FR	79.87	Liver	No
ERX10076677	44121474	Paired	0.936444	0.0567601	FR	69.89	Small intestine	No
ERX10076636	48679535	Paired	0.927119	0.0530599	FR	66.9	Small intestine	No
ERX10076362	30940313	Paired	0.962921	0.0352386	FR	80.38	Liver	No
ERX10076321	43025661	Paired	0.957493	0.0416839	FR	80.69	Liver	No
ERX10076678	55756427	Paired	0.931397	0.0605054	FR	72.98	Small intestine	No
ERX10076637	35061889	Paired	0.934674	0.0524033	FR	71.63	Small intestine	No
ERX10076363	48808271	Paired	0.963767	0.0345423	FR	78.02	Liver	No
ERX10076322	35090709	Paired	0.957963	0.0415169	FR	82.73	Liver	No
ERX10076679	51007113	Paired	0.931116	0.0598722	FR	72.63	Small intestine	No
ERX10076638	41646875	Paired	0.934382	0.0532356	FR	69.14	Small intestine	No
ERX10076364	33774656	Paired	0.957544	0.0438984	FR	78.45	Liver	No
ERX10076323	37050237	Paired	0.954652	0.0474104	FR	80.05	Liver	No
ERX10076090	37867835	Paired	0.974105	0.0276734	FR	65.09	Muscle	No
ERX10076639	29122870	Paired	0.895649	0.0747984	FR	63.74	Small intestine	No
ERX10076365	32512172	Paired	0.969425	0.0318401	FR	82.27	Liver	No
ERX10076324	43749302	Paired	0.956935	0.0439435	FR	82.3	Liver	No
ERX10076091	75694733	Paired	0.974811	0.0259685	FR	69.26	Muscle	No
ERX10076050	37298035	Paired	0.972331	0.0278148	FR	68.84	Muscle	No
ERX10076366	32219258	Paired	0.955373	0.0450374	FR	72.18	Liver	No
ERX10076325	43551768	Paired	0.957895	0.0428456	FR	80.68	Liver	No
ERX10076092	61801492	Paired	0.976364	0.0251373	FR	70.98	Muscle	No
ERX10076051	62185923	Paired	0.973132	0.026522	FR	67.12	Muscle	No
ERX10076010	45575903	Paired	0.969279	0.0301538	FR	82.16	Muscle	No
ERX10076367	35886963	Paired	0.956063	0.0449011	FR	75.77	Liver	No
ERX10076326	40145482	Paired	0.956373	0.045982	FR	78.91	Liver	No
ERX10076093	73485191	Paired	0.976048	0.0255087	FR	66.35	Muscle	No
ERX10076052	40814021	Paired	0.974907	0.0258144	FR	65.68	Muscle	No
ERX10076011	37949802	Paired	0.971418	0.0282843	FR	79.8	Muscle	No
ERX10076368	39936284	Paired	0.95243	0.0479812	FR	74.92	Liver	No
ERX10076327	40913219	Paired	0.958952	0.0399746	FR	80.01	Liver	No
ERX10076094	68627851	Paired	0.974386	0.0264118	FR	67.84	Muscle	No
ERX10076053	46669793	Paired	0.97183	0.0283421	FR	64.37	Muscle	No
ERX10076012	25443978	Paired	0.967687	0.0313069	FR	79.91	Muscle	No
ERX10076369	38235151	Paired	0.933021	0.0641286	FR	74.51	Liver	No
ERX10076328	26934024	Paired	0.967783	0.0330959	FR	80.93	Liver	No
ERX10076095	71326220	Paired	0.976004	0.0255329	FR	66.84	Muscle	No
ERX10076054	53282453	Paired	0.973262	0.02765	FR	67.86	Muscle	No
ERX10076013	79153608	Paired	0.970155	0.0288194	FR	81.68	Muscle	No
ERX10076096	90758093	Paired	0.977084	0.0237421	FR	67.42	Muscle	No
ERX10076055	28890275	Paired	0.971602	0.0288641	FR	66.95	Muscle	No
ERX10076014	29756741	Paired	0.969103	0.0297212	FR	81.8	Muscle	No
ERX10076097	54831940	Paired	0.975705	0.0257963	FR	66.86	Muscle	No
ERX10076056	44779232	Paired	0.974669	0.0258292	FR	68.98	Muscle	No
ERX10076015	25522792	Paired	0.966479	0.0319744	FR	79.52	Muscle	No
ERX10076098	44013455	Paired	0.97352	0.0287549	FR	68.77	Muscle	No
ERX10076057	47607497	Paired	0.973507	0.0276189	FR	66.16	Muscle	No
ERX10076016	42852639	Paired	0.961918	0.0358327	FR	76.81	Muscle	No
ERX10076099	84186080	Paired	0.975805	0.0253761	FR	65.09	Muscle	No
ERX10076058	54050693	Paired	0.974353	0.0259271	FR	67.03	Muscle	No
ERX10076017	32003016	Paired	0.968051	0.0309701	FR	77.2	Muscle	No
ERX10076059	48003997	Paired	0.972133	0.0279757	FR	68.39	Muscle	No

ERX10076018	32255365	Paired	0.970577	0.029214	FR	79.67	Muscle	No
ERX10076019	31427191	Paired	0.967412	0.031454	FR	80.18	Muscle	No
SRX16723082	4940662	Single	0.0359827	NULL	RF	94.36	Ovary	No
ERX8087584	58891965	Paired	0.0152108	0.985589	RF	93.22	Ovary	No
SRX16849390	38489938	Paired	0.0299902	0.971716	RF	82.33	Kidney	No
ERX8087585	59764343	Paired	0.0152342	0.985612	RF	92.87	Ovary	No
SRX16849391	35347887	Paired	0.0258105	0.975328	RF	79.39	Kidney	No
SRX16723084	8262129	Single	0.0305454	NULL	RF	93.38	Ovary	No
ERX8087586	56386168	Paired	0.0150863	0.985716	RF	93.3	Ovary	No
SRX16849392	36874344	Paired	0.0267197	0.975081	RF	88.81	Kidney	No
SRX16723085	3725947	Single	0.0421083	NULL	RF	93.78	Ovary	No
ERX8087587	54981272	Paired	0.0157724	0.985063	RF	93.21	Ovary	No
SRX16849393	37331107	Paired	0.0244878	0.976926	RF	85.65	Kidney	No
SRX16723086	7083094	Single	0.0323818	NULL	RF	94.33	Ovary	No
ERX8087588	52765772	Paired	0.0154524	0.985366	RF	93.35	Ovary	No
SRX16723087	6941514	Single	0.0348625	NULL	RF	93.62	Ovary	No
SRX16849395	38109767	Paired	0.02575	0.975813	RF	84.77	Kidney	No
SRX16723088	4700710	Single	0.0371303	NULL	RF	94.31	Ovary	No
SRX16849396	36140345	Paired	0.0239789	0.977487	RF	85.15	Kidney	No
SRX16723089	4257733	Single	0.954461	NULL	FR	92.36	Ovary	No
SRX16849397	40294221	Paired	0.0250767	0.97643	RF	87.68	Kidney	No
SRX16849398	37447688	Paired	0.0270006	0.974366	RF	79.11	Kidney	No
SRX16849399	36806385	Paired	0.0225684	0.978495	RF	77.33	Kidney	No
SRX15951990	48682254	Paired	0.117308	0.886498	RF	82.39	Muscle	No
SRX10629600	81517472	Paired	0.0928196	0.902564	RF	69.82	Macrophage	No
SRX10629601	82000220	Paired	0.124525	0.871755	RF	76.97	Brain	No
SRX10629602	83556117	Paired	0.118483	0.867289	RF	72.97	Brain	No
SRX10629603	61101564	Paired	0.134206	0.866112	RF	75.3	Brain	No
SRX10629604	86578642	Paired	0.135287	0.855247	RF	82.84	Brain	No
SRX10588950	49126594	Paired	0.100568	0.902756	RF	75.11	Liver	No
SRX10629605	67772465	Paired	0.118759	0.879299	RF	73.85	Brain	No
SRX10629606	82256778	Paired	0.120626	0.86656	RF	71.71	Brain	No
SRX10588952	40468096	Paired	0.0935054	0.91674	RF	79.91	Liver	No
SRX10629607	81790514	Paired	0.14083	0.857657	RF	74.47	Brain	No
SRX10588953	49527544	Paired	0.0890122	0.91554	RF	75.64	Liver	No
SRX10629608	83439774	Paired	0.121482	0.866875	RF	72.15	Brain	No
SRX10588954	32425395	Paired	0.093526	0.910608	RF	70.6	Liver	No
ERX9275360	25287441	Paired	0.0342071	0.965221	RF	86.02	Muscle	No
SRX10629609	61345449	Paired	0.13048	0.86745	RF	72.62	Brain	No
SRX10588955	44110796	Paired	0.100956	0.900652	RF	77.13	Liver	No
ERX9275361	24206621	Paired	0.0257299	0.975071	RF	87.61	Muscle	No
ERX9275320	29424493	Paired	0.0252758	0.97715	RF	87.37	Muscle	No
SRX10588956	45440270	Paired	0.081521	0.92797	RF	79.9	Liver	No
ERX9275362	27150518	Paired	0.025447	0.975071	RF	87.37	Muscle	No
ERX9275321	30904086	Paired	0.0256852	0.975725	RF	88.25	Muscle	No
SRX10588957	50382643	Paired	0.0820398	0.926647	RF	79.29	Liver	No
ERX9275363	25624171	Paired	0.0248614	0.977073	RF	85.61	Muscle	No
ERX9275322	32772382	Paired	0.0250936	0.977095	RF	87.79	Muscle	No
SRX10588958	39119789	Paired	0.0845371	0.923609	RF	79.31	Liver	No
ERX9275364	24260954	Paired	0.0243911	0.979089	RF	88.21	Muscle	No
ERX9275323	29002673	Paired	0.0336701	0.972402	RF	85.66	Muscle	No
ERX9275090	30862792	Paired	0.027846	0.973516	RF	86.61	Liver	No
ERX9275365	28072310	Paired	0.0252525	0.975819	RF	87.38	Muscle	No
ERX9275324	30310648	Paired	0.0223345	0.977844	RF	86.49	Muscle	No
ERX9275091	29067815	Paired	0.0259561	0.974978	RF	86.36	Liver	No
ERX9275366	22318422	Paired	0.0247972	0.97569	RF	87.56	Muscle	No
ERX9275325	32818541	Paired	0.0240306	0.978498	RF	88.67	Muscle	No
ERX9275092	27999739	Paired	0.0246947	0.976507	RF	86.69	Liver	No

ERX9275367	25643005	Paired	0.0293746	0.971015	RF	85.56	Muscle	No
ERX9275326	30843778	Paired	0.0316499	0.974019	RF	87.85	Muscle	No
ERX9275093	30036506	Paired	0.0230926	0.977992	RF	86.64	Liver	No
ERX9275368	24616518	Paired	0.0260645	0.97464	RF	87.77	Muscle	No
ERX9275327	28614276	Paired	0.0244249	0.974968	RF	87.18	Muscle	No
ERX9275094	29859343	Paired	0.0227279	0.978622	RF	86.18	Liver	No
ERX9275369	29080629	Paired	0.0251905	0.975354	RF	87.5	Muscle	No
ERX9275328	17427124	Paired	0.0466645	0.969066	RF	81.95	Muscle	No
ERX9275095	28831308	Paired	0.0318269	0.969117	RF	83.78	Liver	No
ERX9275329	25982385	Paired	0.0202027	0.979019	RF	88.61	Muscle	No
ERX9275096	30492350	Paired	0.0289134	0.97176	RF	85.58	Liver	No
ERX9275097	30318089	Paired	0.0353102	0.965797	RF	84.05	Liver	No
ERX9275098	25078706	Paired	0.0271653	0.973961	RF	85.78	Liver	No
ERX9275099	29363209	Paired	0.0266933	0.973792	RF	85.18	Liver	No
SRX11399720	75350964	Paired	0.05621	0.930475	RF	82.78	Uterus	No
SRX11399721	44938478	Paired	0.0464923	0.946556	RF	88.88	Uterus	No
SRX11399722	41785555	Paired	0.0503602	0.940958	RF	84.93	Uterus	No
SRX11399723	42622193	Paired	0.0282296	0.970666	RF	83.87	Uterus	No
SRX11399724	50864138	Paired	0.0337084	0.954488	RF	89.24	Uterus	No
SRX11399725	85839307	Paired	0.0399532	0.954276	RF	89.62	Uterus	No
SRX11399726	59630087	Paired	0.0420213	0.950451	RF	90.3	Uterus	No
SRX11399727	64527979	Paired	0.0417034	0.952693	RF	89.62	Uterus	No
SRX11399728	94041856	Paired	0.0411874	0.952379	RF	87.26	Uterus	No
SRX11399729	41291744	Paired	0.0563744	0.933358	RF	86.86	Uterus	No
ERX4132990	24009683	Paired	0.0299202	0.970648	RF	93.6	Brain	No
ERX4132991	23163672	Paired	0.0282092	0.972361	RF	93.02	Brain	No
ERX4132992	22498024	Paired	0.0258296	0.974852	RF	92.58	Brain	No
ERX4132993	23766988	Paired	0.0258006	0.974825	RF	92.68	Brain	No
ERX4132994	24597075	Paired	0.0296494	0.970942	RF	92.79	Brain	No
SRX14806470	26643851	Paired	0.121692	0.877269	RF	90.33	Liver	No
ERX4132995	23073442	Paired	0.026568	0.973972	RF	93.85	Brain	No
SRX14806471	28190595	Paired	0.12016	0.878507	RF	88.49	Large intestine	No
SRX14806430	27276637	Paired	0.129114	0.870798	RF	89.28	Small intestine	No
ERX4132996	22177631	Paired	0.0288478	0.971727	RF	92.82	Brain	No
SRX14806472	28673188	Paired	0.113036	0.886195	RF	86.66	Large intestine	No
SRX14806431	34699283	Paired	0.155778	0.842938	RF	88.77	Large intestine	No
ERX4132997	26247110	Paired	0.0252438	0.975359	RF	93.99	Brain	No
SRX14806473	23729069	Paired	0.135658	0.863393	RF	90.6	Small intestine	No
SRX14806432	24036671	Paired	0.0794376	0.920053	RF	88.88	Large intestine	No
ERX4132998	21504559	Paired	0.0258092	0.974903	RF	93.43	Brain	No
SRX14806474	28902904	Paired	0.119015	0.880834	RF	89.92	Small intestine	No
SRX14806433	28408362	Paired	0.13531	0.863525	RF	89.24	Small intestine	No
ERX4132999	22395647	Paired	0.0273637	0.973163	RF	93.47	Brain	No
SRX14806475	29005237	Paired	0.118926	0.879634	RF	89.64	Large intestine	No
SRX14806434	25021878	Paired	0.139893	0.859871	RF	88.89	Small intestine	No
SRX14806476	25391835	Paired	0.074603	0.925087	RF	88.26	Large intestine	No
SRX14806435	24465885	Paired	0.147764	0.849929	RF	89.49	Large intestine	No
SRX14806477	24614140	Paired	0.16061	0.837808	RF	88.44	Small intestine	No
SRX14806436	21242510	Paired	0.0832042	0.916581	RF	88.68	Large intestine	No
SRX14806478	29259961	Paired	0.13262	0.867641	RF	88.7	Small intestine	No
SRX14806437	26258308	Paired	0.141683	0.857317	RF	90.1	Liver	No
SRX14806479	27911699	Paired	0.145859	0.852473	RF	88.68	Large intestine	No
SRX14806438	25100527	Paired	0.130815	0.868008	RF	89.61	Small intestine	No
SRX14806439	25095176	Paired	0.142097	0.85527	RF	88.22	Large intestine	No
SRX12384060	42246790	Paired	0.0483454	0.960772	RF	84.59	Whole blood	No
SRX12384061	48608441	Paired	0.0657229	0.943457	RF	74.43	Whole blood	No
ERX4742290	18992530	Paired	0.0330871	0.967544	RF	92.24	PBMCs	No
ERX4742291	23069838	Paired	0.0454524	0.955207	RF	91.43	PBMCs	No

ERX4742250	21923613	Paired	0.0328021	0.967517	RF	93.11	PBMCs	No
ERX4742292	18383865	Paired	0.0334955	0.967162	RF	92.57	PBMCs	No
ERX4742251	21178462	Paired	0.0319029	0.968531	RF	93.04	PBMCs	No
ERX4742210	19777432	Paired	0.035329	0.965068	RF	93.55	PBMCs	No
ERX4742293	21751263	Paired	0.0325283	0.967947	RF	93.81	PBMCs	No
ERX4742252	17661104	Paired	0.0305703	0.969894	RF	91.23	PBMCs	No
ERX4742211	20423752	Paired	0.0325705	0.967808	RF	93.25	PBMCs	No
ERX4742294	23497173	Paired	0.0303934	0.970211	RF	93.27	PBMCs	No
ERX4742253	19849714	Paired	0.0277752	0.972677	RF	93.1	PBMCs	No
ERX4742212	30085410	Paired	0.0300001	0.970551	RF	91.83	PBMCs	No
ERX4742295	19216599	Paired	0.0313622	0.969369	RF	92.09	PBMCs	No
ERX4742254	18035588	Paired	0.0308955	0.969711	RF	92.61	PBMCs	No
ERX4742213	18628557	Paired	0.0318111	0.968654	RF	93.06	PBMCs	No
ERX4742296	22194123	Paired	0.0322358	0.968333	RF	92.69	PBMCs	No
ERX4742255	17193778	Paired	0.029179	0.971324	RF	92.07	PBMCs	No
ERX4742214	19160132	Paired	0.0321063	0.968422	RF	92.93	PBMCs	No
ERX4742297	21443365	Paired	0.0352042	0.965317	RF	93.05	PBMCs	No
ERX4742256	21961420	Paired	0.0364121	0.964057	RF	92.67	PBMCs	No
ERX4742215	18377002	Paired	0.0322447	0.968339	RF	92.3	PBMCs	No
ERX4742298	20433771	Paired	0.0328944	0.967605	RF	92.31	PBMCs	No
ERX4742257	21703530	Paired	0.0313946	0.968889	RF	92.94	PBMCs	No
ERX4742216	19532778	Paired	0.0347488	0.965678	RF	92.51	PBMCs	No
ERX4742299	18077610	Paired	0.0328659	0.9676	RF	91.98	PBMCs	No
ERX4742258	19636330	Paired	0.0437258	0.956977	RF	91.89	PBMCs	No
ERX4742217	19825538	Paired	0.0315866	0.968767	RF	93.16	PBMCs	No
SRX13373750	64028352	Paired	0.0283595	0.972194	RF	88.42	Macrophage	No
ERX4742259	20353929	Paired	0.0311306	0.969353	RF	93.4	PBMCs	No
ERX4742218	19160582	Paired	0.0337598	0.966803	RF	93.18	PBMCs	No
SRX13373751	67806034	Paired	0.0232626	0.97744	RF	88.5	Macrophage	No
SRX12142640	21505600	Paired	0.15443	0.83753	RF	71.03	PBMCs	No
ERX4742219	20232913	Paired	0.0325952	0.967953	RF	93.14	PBMCs	No
SRX13373752	72969507	Paired	0.0253577	0.975789	RF	89.8	Macrophage	No
SRX12142641	18942074	Paired	0.100123	0.89246	RF	67.55	PBMCs	No
SRX12142600	25281330	Paired	0.106183	0.883371	RF	67.69	PBMCs	No
SRX12142601	23470259	Paired	0.098916	0.890665	RF	66.92	PBMCs	No
SRX12142602	8364775	Paired	0.0853565	0.910889	RF	71.73	PBMCs	No
SRX12142644	26922822	Paired	0.116553	0.882339	RF	60.43	PBMCs	No
SRX12142645	19678839	Paired	0.0968969	0.896881	RF	64.94	PBMCs	No
SRX12142604	31734921	Paired	0.0918413	0.905387	RF	66.6	PBMCs	No
SRX12142646	9784580	Paired	0.0947231	0.898846	RF	66.84	PBMCs	No
SRX12142605	39626108	Paired	0.120231	0.880714	RF	60.56	PBMCs	No
SRX12142606	40444970	Paired	0.096223	0.901229	RF	62.66	PBMCs	No
SRX12142609	36068233	Paired	0.139215	0.854458	RF	61.96	PBMCs	No
SRX13507550	41930316	Paired	0.076493	0.923183	RF	81.72	Ovary	No
SRX13507551	37642410	Paired	0.0842752	0.915072	RF	81.91	Ovary	No
SRX13507552	39395081	Paired	0.0748205	0.924783	RF	82.44	Ovary	No
ERX9275170	26178724	Paired	0.0304804	0.976829	RF	83.9	Liver	No
SRX13507553	40109544	Paired	0.0778279	0.921735	RF	81.31	Ovary	No
ERX9275171	33690712	Paired	0.0273044	0.978662	RF	85.71	Liver	No
ERX9275130	26104450	Paired	0.0277653	0.976096	RF	87.12	Liver	No
ERX9275172	30143253	Paired	0.0274932	0.977715	RF	84.6	Liver	No
ERX9275131	23614000	Paired	0.0302283	0.972464	RF	87.09	Liver	No
ERX9275173	22415265	Paired	0.0266061	0.978989	RF	84.88	Liver	No
ERX9275132	24087317	Paired	0.024795	0.977686	RF	87.34	Liver	No
ERX9275174	22649724	Paired	0.0319692	0.972437	RF	86.59	Liver	No
ERX9275133	31555684	Paired	0.0255462	0.976895	RF	87.66	Liver	No
SRX15623530	18522862	Paired	0.0263418	0.972741	RF	85	Early embryo	No
ERX9275175	23280633	Paired	0.0325335	0.972022	RF	85.73	Liver	No

ERX9275134	29466965	Paired	0.0222782	0.979537	RF	88.06	Liver	No
SRX15623531	18650236	Paired	0.0306038	0.966271	RF	81.26	Early embryo	No
ERX9275176	16506922	Paired	0.0343214	0.970554	RF	86.77	Liver	No
ERX9275135	25820858	Paired	0.0234448	0.978498	RF	87.08	Liver	No
SRX15623532	19047196	Paired	0.0246598	0.974408	RF	83.75	Early embryo	No
ERX9275177	46042836	Paired	0.0250963	0.980299	RF	84.32	Liver	No
ERX9275136	27329522	Paired	0.0262576	0.978508	RF	87.13	Liver	No
SRX15623533	19048264	Paired	0.0271381	0.971532	RF	82.18	Early embryo	No
ERX9275178	29800721	Paired	0.0274129	0.977849	RF	84.97	Liver	No
ERX9275137	28486228	Paired	0.0221359	0.980274	RF	87.28	Liver	No
SRX15623534	18823171	Paired	0.0249977	0.974195	RF	85.09	Early embryo	No
ERX9275179	20291684	Paired	0.0393649	0.961408	RF	85.4	Liver	No
ERX9275138	33826359	Paired	0.0293608	0.974361	RF	85.98	Liver	No
SRX15623535	18994747	Paired	0.0258728	0.973294	RF	83.27	Early embryo	No
ERX9275139	33853337	Paired	0.0251664	0.977185	RF	87.38	Liver	No
SRX15623536	19679105	Paired	0.025961	0.973039	RF	84.29	Early embryo	No
SRX15623537	19548769	Paired	0.023893	0.975253	RF	85.52	Early embryo	No
SRX15623538	19000730	Paired	0.0234609	0.976051	RF	84.94	Early embryo	No
SRX11216680	43045151	Paired	0.131045	0.86888	RF	86.24	Small intestine	No
SRX11216681	53942231	Paired	0.0727961	0.924101	RF	78.66	Brain	No
ERX9900020	28420284	Paired	0.0471228	0.949652	RF	83.9	Brain	No
SRX11216682	43316882	Paired	0.132161	0.864947	RF	77.73	Heart	No
ERX9900021	32563515	Paired	0.0567626	0.940019	RF	87.42	Brain	No
SRX11216683	42071081	Paired	0.12257	0.875626	RF	69.08	Muscle	No
ERX9900022	26920624	Paired	0.0560153	0.939552	RF	87.19	Brain	No
SRX11216684	73727325	Paired	0.0911169	0.905451	RF	77.84	Brain	No
SRX11216686	44993214	Paired	0.0820487	0.917765	RF	74.51	Lung	No
SRX11216687	35753561	Paired	0.0828313	0.916089	RF	78.07	Liver	No
SRX11216688	40199351	Paired	0.104587	0.894098	RF	70.64	Adipose	No
SRX14241636	30514287	Single	0.0886415	NULL	RF	72.49	Large intestine	No
SRX14241637	56678461	Single	0.07786	NULL	RF	73.75	Large intestine	No
SRX14241638	37886797	Single	0.0799309	NULL	RF	70.86	Large intestine	No
SRX14241639	37180849	Single	0.081344	NULL	RF	74.16	Large intestine	No
SRX14748330	51732536	Single	0.0474046	NULL	RF	90.38	Small intestine	No
SRX13443410	48399551	Single	0.0360614	NULL	RF	88.02	Cartilage	No
SRX14748331	48219786	Single	0.0648239	NULL	RF	87.7	Small intestine	No
SRX13443411	41105417	Single	0.0323217	NULL	RF	88.33	Cartilage	No
SRX14748332	46068043	Single	0.0628827	NULL	RF	87.28	Small intestine	No
SRX13443412	39004068	Single	0.0397793	NULL	RF	76.02	Cartilage	No
SRX14748333	37536668	Single	0.0514647	NULL	RF	88.45	Small intestine	No
SRX13443413	39972797	Single	0.0345509	NULL	RF	76.64	Cartilage	No
SRX14748334	44806489	Single	0.0441921	NULL	RF	88.17	Small intestine	No
SRX13443414	50492329	Single	0.0299122	NULL	RF	88.49	Cartilage	No
SRX14748335	48380033	Single	0.0679494	NULL	RF	87.57	Small intestine	No
SRX13443415	52817376	Single	0.0333916	NULL	RF	66.59	Cartilage	No
SRX13443416	47272837	Single	0.0357489	NULL	RF	75.74	Cartilage	No
SRX13443417	48184813	Single	0.0350381	NULL	RF	79.69	Cartilage	No
SRX13443418	49969490	Single	0.034548	NULL	RF	86.65	Cartilage	No
SRX13443419	40665774	Single	0.0330129	NULL	RF	74.12	Cartilage	No
SRX11350443	76308484	Paired	0.0217297	0.978293	RF	92.42	Testis	No
SRX11350444	76337213	Paired	0.0215424	0.978561	RF	92.96	Testis	No
SRX11350445	75068132	Paired	0.021714	0.978426	RF	93.34	Testis	No
SRX11350446	74736536	Paired	0.0497655	0.948107	RF	92.95	Testis	No
SRX11350448	75294081	Paired	0.0448108	0.954091	RF	93.04	Testis	No
SRX11350449	74881025	Paired	0.0423836	0.95686	RF	93.64	Testis	No
SRX16019000	76284621	Paired	0.0669474	0.935645	RF	86.19	Muscle	No
SRX16019001	58933480	Paired	0.0669883	0.935731	RF	87.13	Muscle	No
SRX16019002	56757430	Paired	0.0667343	0.935164	RF	88.7	Muscle	No

SRX16019003	59017999	Paired	0.0615427	0.940113	RF	89.73	Muscle	No
SRX16019004	77152281	Paired	0.0602034	0.941689	RF	88.99	Muscle	No
SRX16019005	65475714	Paired	0.0565859	0.945557	RF	89.73	Muscle	No
SRX13459890	34836435	Paired	0.0263076	0.972449	RF	89.37	Cartilage	No
SRX16323980	45061678	Paired	0.0918579	0.915085	RF	76.17	Whole blood	No
SRX13459891	38110203	Paired	0.0272236	0.971583	RF	89.09	Cartilage	No
SRX16323981	39815287	Paired	0.0836223	0.919369	RF	83.79	Whole blood	No
SRX13459892	42432367	Paired	0.0259497	0.972825	RF	88.62	Cartilage	No
SRX16323982	49026390	Paired	0.109394	0.893429	RF	86.99	Whole blood	No
SRX13459893	36964468	Paired	0.0237984	0.975426	RF	91.33	Cartilage	No
SRX16323983	51514056	Paired	0.0983454	0.903087	RF	89.64	Whole blood	No
SRX13459894	34620908	Paired	0.0247624	0.97416	RF	89.6	Cartilage	No
SRX16323984	43931590	Paired	0.0875615	0.915341	RF	85.2	Whole blood	No
SRX13459895	37715053	Paired	0.0253352	0.97321	RF	90.26	Cartilage	No
SRX16323985	47783232	Paired	0.0984278	0.907127	RF	82.78	Whole blood	No
SRX13459896	33474226	Paired	0.0250997	0.97385	RF	87.68	Cartilage	No
ERX9275290	29176255	Paired	0.0281289	0.973459	RF	85.11	Muscle	No
SRX16323986	48183157	Paired	0.106954	0.894253	RF	90.67	Whole blood	No
SRX13459897	33637537	Paired	0.0249704	0.973121	RF	90.87	Cartilage	No
ERX9275291	28815300	Paired	0.0261059	0.975095	RF	86.85	Muscle	No
ERX9275250	60462355	Paired	0.0205365	0.983234	RF	84.66	Muscle	No
SRX16323987	51562875	Paired	0.0910201	0.913004	RF	84.38	Whole blood	No
SRX13459898	36619189	Paired	0.0263931	0.972379	RF	91.02	Cartilage	No
ERX9275292	27886399	Paired	0.0278646	0.973117	RF	86.34	Muscle	No
ERX9275251	23154848	Paired	0.0215518	0.980989	RF	76.96	Muscle	No
ERX9275210	26150441	Paired	0.0244479	0.979689	RF	84.85	Liver	No
SRX16323988	50778186	Paired	0.109187	0.891966	RF	89.07	Whole blood	No
SRX13459899	34315517	Paired	0.0217934	0.976963	RF	90.75	Cartilage	No
ERX9275293	36601844	Paired	0.0223608	0.979599	RF	86.47	Muscle	No
ERX9275252	24126815	Paired	0.0194051	0.983578	RF	82.34	Muscle	No
ERX9275211	24170091	Paired	0.0303132	0.974959	RF	83.81	Liver	No
SRX16323989	48346197	Paired	0.11983	0.881537	RF	86.75	Whole blood	No
SRX14418530	21715959	Paired	0.0464915	0.950448	RF	86	PBMCs	No
ERX9275294	35654775	Paired	0.0195763	0.982595	RF	86.17	Muscle	No
ERX9275253	26670762	Paired	0.0216079	0.980503	RF	84.78	Muscle	No
ERX9275212	26276123	Paired	0.028267	0.976278	RF	85.31	Liver	No
SRX14418531	29442511	Paired	0.0396846	0.957434	RF	86.89	PBMCs	No
SRX11011041	42322063	Paired	0.0515187	0.94709	RF	89.82	Adipose	No
ERX9275254	30431924	Paired	0.019777	0.982983	RF	85.81	Muscle	No
ERX9275213	24632306	Paired	0.0285369	0.975763	RF	86.18	Liver	No
SRX14418532	27780851	Paired	0.0444037	0.951501	RF	84.98	PBMCs	No
SRX14303977	39447568	Paired	0.0242369	0.977704	RF	86.13	Liver	No
ERX9275296	27742660	Paired	0.0272224	0.973794	RF	84.99	Muscle	No
ERX9275255	28253201	Paired	0.0203654	0.981193	RF	77.77	Muscle	No
ERX9275214	34790037	Paired	0.0282238	0.975683	RF	84.97	Liver	No
SRX14418533	22657895	Paired	0.0540675	0.942003	RF	83.11	PBMCs	No
SRX14303978	40751703	Paired	0.0216375	0.980598	RF	87.15	Liver	No
ERX9275297	21816805	Paired	0.0319532	0.974349	RF	83.41	Muscle	No
ERX9275256	41391334	Paired	0.0167928	0.983495	RF	86.9	Muscle	No
ERX9275215	28249242	Paired	0.0253625	0.979812	RF	85.59	Liver	No
SRX14303979	38994172	Paired	0.0226596	0.97924	RF	88.95	Liver	No
ERX9275298	27460186	Paired	0.021316	0.978586	RF	87.92	Muscle	No
ERX9275257	23322269	Paired	0.0202622	0.979903	RF	88.15	Muscle	No
ERX9275216	25347694	Paired	0.0261264	0.979791	RF	86.05	Liver	No
SRX14418535	15489255	Paired	0.0491705	0.948179	RF	84.35	PBMCs	No
SRX14410809	33609254	Paired	0.0418677	0.955693	RF	84.31	Brain	No
ERX9275299	22955768	Paired	0.0242954	0.976256	RF	86.33	Muscle	No
ERX9275258	24452665	Paired	0.024813	0.979632	RF	84.85	Muscle	No

ERX9275217	28164999	Paired	0.025874	0.979497	RF	84.7	Liver	No
SRX14418536	25339209	Paired	0.0414177	0.954345	RF	86.16	PBMCs	No
ERX9275259	19257539	Paired	0.0200962	0.981428	RF	84.76	Muscle	No
ERX9275218	22016542	Paired	0.0275072	0.977757	RF	84.31	Liver	No
ERX9275219	26449163	Paired	0.0275113	0.978042	RF	84.62	Liver	No
SRX11216721	39414475	Paired	0.130955	0.866711	RF	70.95	Adipose	No
ERX3981440	39342872	Paired	0.0635894	0.880626	RF	75.88	Heart	No
SRX11216723	41723074	Paired	0.157235	0.834965	RF	83.59	Heart	No
ERX3981441	34103315	Paired	0.0639948	0.888136	RF	78.36	Heart	No
SRX7443801	63436721	Paired	0.0242222	0.975939	RF	84.19	Muscle	No
ERX3981442	30944088	Paired	0.070627	0.878007	RF	77.81	Heart	No
SRX7443802	54050933	Paired	0.0293663	0.970027	RF	82.33	Muscle	No
ERX3981443	26724813	Paired	0.059668	0.894914	RF	78.07	Heart	No
SRX7443803	68849434	Paired	0.0242452	0.976329	RF	84.58	Muscle	No
ERX3981444	29059734	Paired	0.0567171	0.906209	RF	82.51	Heart	No
ERX2987510	18634594	Paired	0.0242019	0.97601	RF	90.75	Brain	No
SRX7443804	63072944	Paired	0.0252754	0.974734	RF	84.71	Muscle	No
ERX3981445	30133733	Paired	0.0581486	0.911913	RF	79.84	Heart	No
ERX2987511	17873659	Paired	0.0270106	0.973196	RF	87.35	Brain	No
SRX7443805	68653581	Paired	0.0233893	0.976371	RF	82.5	Muscle	No
ERX3981446	31701912	Paired	0.0571273	0.906485	RF	79.8	Heart	No
ERX2987512	13246714	Paired	0.0422435	0.9584	RF	90.87	Brain	No
SRX7443806	51275600	Paired	0.0262417	0.973452	RF	82.22	Muscle	No
ERX3981447	33895084	Paired	0.0641921	0.888372	RF	77.73	Heart	No
ERX2987513	12930503	Paired	0.0306083	0.969674	RF	93.36	Brain	No
ERX3981448	31760069	Paired	0.0661944	0.900843	RF	79.78	Heart	No
ERX2987514	32237370	Paired	0.0243831	0.975871	RF	92.76	Brain	No
ERX3981449	30436111	Paired	0.0613505	0.90304	RF	79.57	Heart	No
ERX2987515	25221219	Paired	0.0256027	0.974617	RF	92.88	Brain	No
ERX2987516	15805173	Paired	0.025281	0.974931	RF	89.79	Brain	No
ERX2987517	26283700	Paired	0.0232633	0.976948	RF	93.2	Brain	No
ERX2987518	31195161	Paired	0.0268769	0.97331	RF	92.91	Brain	No
ERX2987519	17709211	Paired	0.0266057	0.973679	RF	93.38	Brain	No
SRX16324011	44476240	Paired	0.104935	0.901787	RF	79.89	Whole blood	No
SRX11891700	50712878	Paired	0.0916059	0.912307	RF	63.87	T cell	No
SRX11891701	46464922	Paired	0.0978323	0.903862	RF	68.53	T cell	No
SRX11891702	53308909	Paired	0.0841617	0.914621	RF	66.28	T cell	No
SRX11891705	29639390	Paired	0.101709	0.906188	RF	65.49	T cell	No
SRX16723090	5829856	Single	0.0445548	NULL	RF	94.93	Uterus	No
SRX11891706	32065425	Paired	0.105476	0.89789	RF	62.79	T cell	No
SRX16723091	7880024	Single	0.0395658	NULL	RF	94.79	Uterus	No
SRX11891707	64253416	Paired	0.097009	0.900819	RF	71.78	T cell	No
SRX16723092	4747202	Single	0.0478092	NULL	RF	94.49	Uterus	No
SRX11891708	44092458	Paired	0.109886	0.890609	RF	63.2	T cell	No
SRX16723093	4804644	Single	0.946964	NULL	FR	93.87	Uterus	No
SRX16723094	6088550	Single	0.0415834	NULL	RF	94.96	Uterus	No
SRX16723095	6632103	Single	0.0402663	NULL	RF	94.67	Uterus	No
SRX16723096	6063519	Single	0.0425473	NULL	RF	93.97	Uterus	No
SRX14239277	3537862	Single	0.0603723	NULL	RF	90.2	Uterus	No
SRX14239278	762973	Single	0.860423	NULL	FR	86.78	Uterus	No
SRX14239279	4601930	Single	0.947904	NULL	FR	90.07	Uterus	No
SRX10629610	72342216	Paired	0.15148	0.84781	RF	73.34	Brain	No
SRX10629611	67802864	Paired	0.162407	0.835393	RF	80.84	Brain	No
SRX10629612	88202852	Paired	0.141952	0.848821	RF	82.61	Brain	No
SRX10629613	99347146	Paired	0.115189	0.876682	RF	76.59	Macrophage	No
ERX9275370	28538191	Paired	0.0258457	0.975523	RF	87.24	Muscle	No
ERX9275371	26265656	Paired	0.0261304	0.975019	RF	86.31	Muscle	No
ERX9275330	28745600	Paired	0.0263225	0.975304	RF	86.34	Muscle	No

ERX9275372	28324026	Paired	0.0264025	0.975816	RF	87.68	Muscle	No
ERX9275331	22835572	Paired	0.0270186	0.972867	RF	86.04	Muscle	No
ERX9275373	25167084	Paired	0.0323623	0.975463	RF	85.37	Muscle	No
ERX9275332	33345533	Paired	0.0242664	0.977193	RF	87.93	Muscle	No
ERX9275374	28299443	Paired	0.0247115	0.97675	RF	87.02	Muscle	No
ERX9275333	25210314	Paired	0.030054	0.972679	RF	85.42	Muscle	No
ERX9275375	36249016	Paired	0.0240267	0.976574	RF	88.1	Muscle	No
ERX9275334	26427333	Paired	0.026724	0.974857	RF	87.51	Muscle	No
ERX9275376	32521470	Paired	0.0267237	0.972564	RF	86.93	Muscle	No
ERX9275335	26298286	Paired	0.0243173	0.97634	RF	85.04	Muscle	No
ERX9275377	27271078	Paired	0.0287951	0.971332	RF	87.4	Muscle	No
ERX9275378	25737587	Paired	0.0218946	0.978764	RF	87.31	Muscle	No
ERX9275337	28766620	Paired	0.0300822	0.974794	RF	85.47	Muscle	No
ERX9275379	26106230	Paired	0.0266051	0.976096	RF	86.08	Liver	No
ERX9275338	25159560	Paired	0.0240804	0.976709	RF	87.5	Muscle	No
ERX9275339	32036963	Paired	0.0239225	0.977279	RF	88.12	Muscle	No
SRX11399730	44176780	Paired	0.0415209	0.950844	RF	89.39	Uterus	No
SRX14806480	27437929	Paired	0.078666	0.921056	RF	88.34	Large intestine	No
SRX14806481	24575877	Paired	0.131259	0.867887	RF	89.69	Liver	No
SRX14806440	25314032	Paired	0.0808065	0.918119	RF	88.32	Large intestine	No
SRX14806482	22842831	Paired	0.134861	0.863984	RF	90.68	Small intestine	No
SRX14806441	25883391	Paired	0.146449	0.853385	RF	88.4	Small intestine	No
SRX14806400	25501382	Paired	0.127721	0.871262	RF	90.44	Liver	No
SRX14806483	23065485	Paired	0.136731	0.863537	RF	87.74	Small intestine	No
SRX14806442	22787713	Paired	0.128517	0.870963	RF	89.4	Small intestine	No
SRX14806401	21440096	Paired	0.170775	0.828071	RF	87.47	Liver	No
SRX14806484	30202092	Paired	0.135625	0.863543	RF	89.02	Large intestine	No
SRX14806443	29179193	Paired	0.116232	0.881907	RF	88.67	Large intestine	No
SRX14806402	23298624	Paired	0.159993	0.83888	RF	88.37	Liver	No
SRX14806485	25517570	Paired	0.0730726	0.926616	RF	88.8	Large intestine	No
SRX14806444	24939463	Paired	0.0776169	0.921974	RF	88.07	Large intestine	No
SRX14806403	23959743	Paired	0.159165	0.838558	RF	88.34	Liver	No
SRX14806486	23836889	Paired	0.134993	0.863146	RF	90.25	Small intestine	No
SRX14806445	20253404	Paired	0.146769	0.852485	RF	87.88	Small intestine	No
SRX14806404	23980749	Paired	0.123908	0.875191	RF	91.26	Liver	No
SRX14806487	26354329	Paired	0.12892	0.870984	RF	89.18	Small intestine	No
SRX14806446	23354690	Paired	0.154046	0.844329	RF	87.29	Small intestine	No
SRX14806405	25781417	Paired	0.119166	0.880358	RF	90.21	Liver	No
SRX14806488	32893731	Paired	0.145495	0.85263	RF	89.34	Large intestine	No
SRX14806447	31920843	Paired	0.125274	0.872524	RF	88.59	Large intestine	No
SRX14806406	28091951	Paired	0.124529	0.875645	RF	90.63	Liver	No
SRX14806489	21506164	Paired	0.152891	0.844301	RF	89.93	Large intestine	No
SRX14806448	25179069	Paired	0.123441	0.875521	RF	90.59	Liver	No
SRX14806407	23181284	Paired	0.111672	0.888028	RF	90.8	Liver	No
SRX14806449	28164777	Paired	0.0842295	0.915415	RF	88.29	Large intestine	No
SRX14806408	21243164	Paired	0.177879	0.820978	RF	86.22	Small intestine	No
SRX14806409	21107950	Paired	0.143012	0.856249	RF	87.51	Small intestine	No
SRX16849400	42058328	Paired	0.0252686	0.975914	RF	84.1	Kidney	No
SRX16849401	38901718	Paired	0.0225959	0.978303	RF	80.46	Kidney	No
SRX16849402	37218941	Paired	0.0282446	0.973875	RF	85.25	Kidney	No
SRX16849403	37091131	Paired	0.0244705	0.977251	RF	86.34	Kidney	No
SRX16849404	37409747	Paired	0.0295713	0.972641	RF	84.81	Kidney	No
ERX4742260	23108670	Paired	0.0282302	0.972309	RF	91.56	PBMCs	No
ERX4742261	20594583	Paired	0.0289502	0.971486	RF	93.46	PBMCs	No
ERX4742220	21393490	Paired	0.0434858	0.95697	RF	88.22	PBMCs	No
ERX4742262	17788257	Paired	0.030833	0.969621	RF	92.87	PBMCs	No
ERX4742221	21003652	Paired	0.035085	0.965323	RF	93.68	PBMCs	No
ERX4742263	17550243	Paired	0.0293752	0.970729	RF	92.03	PBMCs	No

ERX4742222	18207727	Paired	0.0323244	0.968183	RF	92.33	PBMCs	No
ERX4742264	20309022	Paired	0.0307665	0.969717	RF	92.03	PBMCs	No
ERX4742223	34672471	Paired	0.0328817	0.967801	RF	92.47	PBMCs	No
ERX4742265	21070363	Paired	0.0282524	0.972206	RF	93.1	PBMCs	No
ERX4742224	18612529	Paired	0.0335215	0.966934	RF	92.49	PBMCs	No
ERX4742266	20332286	Paired	0.0314453	0.969065	RF	92.26	PBMCs	No
ERX4742225	27774935	Paired	0.0315524	0.969054	RF	92.19	PBMCs	No
ERX4742267	19001331	Paired	0.0305926	0.970172	RF	93.23	PBMCs	No
ERX4742226	23856066	Paired	0.0344036	0.966031	RF	93.49	PBMCs	No
ERX4742268	21217174	Paired	0.0301127	0.970317	RF	93.28	PBMCs	No
ERX4742227	19552156	Paired	0.0345205	0.965873	RF	91.79	PBMCs	No
ERX4742269	17534418	Paired	0.0387507	0.961644	RF	90.09	PBMCs	No
ERX4742228	19183155	Paired	0.03299	0.967525	RF	92.22	PBMCs	No
ERX4742229	22078034	Paired	0.0317973	0.968597	RF	92.5	PBMCs	No
SRX12142610	38215426	Paired	0.130559	0.864729	RF	64.91	PBMCs	No
SRX12142613	9605353	Paired	0.0830183	0.914563	RF	74.87	PBMCs	No
SRX12142657	8822232	Paired	0.0832946	0.914304	RF	70.3	PBMCs	No
SRX12142617	22383506	Paired	0.0881573	0.907717	RF	67.57	PBMCs	No
SRX12142618	23922835	Paired	0.0852447	0.910385	RF	69.05	PBMCs	No
SRX12142619	23590673	Paired	0.0670682	0.929311	RF	68.17	PBMCs	No
ERX9275180	28805979	Paired	0.0322722	0.976404	RF	82.8	Liver	No
ERX9275181	22378392	Paired	0.0337068	0.97292	RF	84.56	Liver	No
ERX9275140	23097968	Paired	0.0250752	0.977585	RF	87.76	Liver	No
ERX9275182	21519783	Paired	0.034143	0.970687	RF	86.06	Liver	No
ERX9275141	25445455	Paired	0.0302326	0.972184	RF	87.33	Liver	No
ERX9275100	42550915	Paired	0.0266103	0.974015	RF	85.17	Liver	No
ERX9275183	24306007	Paired	0.0310761	0.973248	RF	85.26	Liver	No
ERX9275142	27186543	Paired	0.0290737	0.972706	RF	87.77	Liver	No
ERX9275101	25051534	Paired	0.028508	0.972504	RF	85.61	Liver	No
ERX9275184	62734008	Paired	0.0255638	0.980108	RF	84.72	Liver	No
ERX9275143	20432865	Paired	0.0343478	0.971808	RF	85.24	Liver	No
ERX9275102	35838792	Paired	0.0274489	0.974408	RF	84.98	Liver	No
ERX9275185	31251009	Paired	0.0297387	0.974555	RF	85.9	Liver	No
ERX9275144	26220670	Paired	0.0261695	0.976083	RF	88.09	Liver	No
ERX6202487	20341021	Paired	0.0230404	0.978266	RF	85.62	Uterus	No
ERX9275103	29014101	Paired	0.0308848	0.969376	RF	84.88	Liver	No
ERX9275186	32007004	Paired	0.0270581	0.976932	RF	86.19	Liver	No
ERX9275145	24474431	Paired	0.0279241	0.978094	RF	84.11	Liver	No
ERX6202488	21632993	Paired	0.024286	0.976821	RF	85.88	Uterus	No
ERX9275104	28216088	Paired	0.0269935	0.97402	RF	85.47	Liver	No
ERX9275146	26960201	Paired	0.0266387	0.978955	RF	82.62	Liver	No
ERX6202489	19881219	Paired	0.0238304	0.977227	RF	85.07	Uterus	No
ERX9275105	25625545	Paired	0.0247394	0.976143	RF	85.67	Liver	No
ERX9275187	26092518	Paired	0.028003	0.975911	RF	86.53	Liver	No
ERX9275147	18574503	Paired	0.0455123	0.955233	RF	85.85	Liver	No
ERX9275106	19665299	Paired	0.0262944	0.974979	RF	86.25	Liver	No
ERX9275188	27624070	Paired	0.0342058	0.972677	RF	85.06	Liver	No
ERX9275148	24292690	Paired	0.0292192	0.976659	RF	84.08	Liver	No
ERX9275107	25895723	Paired	0.0262319	0.973891	RF	85.86	Liver	No
ERX9275189	22431318	Paired	0.0311334	0.973607	RF	86.59	Liver	No
ERX9275149	23714573	Paired	0.0273192	0.97861	RF	84.13	Liver	No
ERX9275108	35021439	Paired	0.0258754	0.974763	RF	85.87	Liver	No
ERX9275109	32115529	Paired	0.0250212	0.975439	RF	86.36	Liver	No
SRX17395010	23633614	Single	0.932484	NULL	FR	81.59	Small intestine	No
SRX17395011	34002619	Single	0.927834	NULL	FR	78.91	Small intestine	No
SRX17395013	19768366	Single	0.877883	NULL	FR	82.84	Small intestine	No
SRX17395014	36777782	Single	0.905556	NULL	FR	81.78	Small intestine	No
SRX11216690	53443713	Paired	0.103797	0.896211	RF	68.41	Small intestine	No

SRX17395015	13994960	Single	0.906066	NULL	FR	82.14	Small intestine	No
SRX11216691	43866544	Paired	0.0800689	0.918335	RF	81.07	Brain	No
SRX17395016	21677479	Single	0.929116	NULL	FR	82.7	Small intestine	No
SRX11216692	43182143	Paired	0.0949341	0.90425	RF	76.55	Heart	No
SRX17395017	18674018	Single	0.89127	NULL	FR	77.41	Small intestine	No
SRX11216693	42500758	Paired	0.0926351	0.907516	RF	80.44	Muscle	No
SRX17115220	23600309	Paired	0.0416414	0.907734	RF	85.97	Kidney	No
SRX14241641	24319522	Single	0.0830369	NULL	RF	72.85	Large intestine	No
SRX17395019	21502347	Single	0.86417	NULL	FR	70.79	Small intestine	No
SRX17115221	26638711	Paired	0.0422208	0.923935	RF	87.03	Kidney	No
SRX11216695	46261798	Paired	0.109544	0.889243	RF	72.92	Lung	No
SRX17115222	132172300	Paired	0.0542815	0.923321	RF	90.1	Kidney	No
SRX17115223	32134165	Paired	0.0492144	0.919312	RF	87.9	Kidney	No
SRX14241644	24537100	Single	0.0797905	NULL	RF	74.02	Large intestine	No
SRX11216697	49261187	Paired	0.116916	0.87959	RF	75.99	Adipose	No
SRX17115224	26815569	Paired	0.0400053	0.921007	RF	87.2	Kidney	No
SRX14241645	35578737	Single	0.0825398	NULL	RF	71.78	Large intestine	No
SRX11216698	44332262	Paired	0.0937327	0.907274	RF	86.22	Small intestine	No
ERX2987485	22118005	Paired	0.0268986	0.973309	RF	90.54	Brain	No
SRX14241649	64505859	Single	0.089637	NULL	RF	75.01	Large intestine	No
ERX2987486	18048011	Paired	0.025376	0.974834	RF	93.46	Brain	No
ERX2987487	13885858	Paired	0.039872	0.960302	RF	93.47	Brain	No
ERX2987488	35569516	Paired	0.0235044	0.976698	RF	93.06	Brain	No
ERX2987489	24152009	Paired	0.0244646	0.975944	RF	92.6	Brain	No
SRX13443420	43400114	Single	0.031767	NULL	RF	70.43	Cartilage	No
SRX13443421	43513777	Single	0.0303742	NULL	RF	72	Cartilage	No
SRX13443422	43222670	Single	0.0315989	NULL	RF	73.32	Cartilage	No
SRX13443423	39247492	Single	0.0351821	NULL	RF	84.74	Cartilage	No
SRX13443424	39132158	Single	0.0333265	NULL	RF	87.42	Cartilage	No
ERX4742300	22656969	Paired	0.0313236	0.969186	RF	93.25	PBMCs	No
ERX4742301	42597768	Paired	0.0333436	0.967204	RF	92.81	PBMCs	No
ERX4742302	17100317	Paired	0.0343876	0.966147	RF	92.8	PBMCs	No
ERX4742303	41975066	Paired	0.0285664	0.972024	RF	91.77	PBMCs	No
SRX10629583	69884632	Paired	0.143207	0.853329	RF	76.48	Brain	No
SRX16323990	39257612	Paired	0.109504	0.893026	RF	86.97	Whole blood	No
SRX10629584	70739314	Paired	0.113019	0.883996	RF	73.06	Brain	No
SRX10629585	78834618	Paired	0.143528	0.857592	RF	72.69	Brain	No
SRX10629586	79130918	Paired	0.121412	0.873087	RF	78.19	Brain	No
SRX10629587	82933534	Paired	0.105838	0.889701	RF	68.4	Brain	No
SRX14303980	39968750	Paired	0.0237871	0.977718	RF	88.81	Liver	No
SRX10629588	73647037	Paired	0.142351	0.856983	RF	77	Brain	No
SRX19802416	46298844	Paired	0.0308785	0.970053	RF	87.17	Cartilage	No
SRX14410810	42114752	Paired	0.0412383	0.956451	RF	82.87	Brain	No
SRX10629589	77098244	Paired	0.130836	0.8686	RF	77.19	Brain	No
SRX19802417	46701015	Paired	0.0338828	0.966024	RF	88.61	Cartilage	No
SRX16323955	45539409	Paired	0.0914604	0.911131	RF	85.23	Whole blood	No
SRX14410811	33183045	Paired	0.0375798	0.960333	RF	84.62	Brain	No
ERX9275260	41697108	Paired	0.0216631	0.980988	RF	86.96	Muscle	No
SRX19802418	44417616	Paired	0.0331458	0.970398	RF	83.3	Cartilage	No
SRX16323956	39151192	Paired	0.129527	0.873299	RF	84.89	Whole blood	No
ERX9275261	25369855	Paired	0.0215378	0.981576	RF	86.66	Muscle	No
ERX9275220	23084473	Paired	0.0286627	0.977937	RF	83.61	Liver	No
SRX19802419	48879658	Paired	0.044323	0.956703	RF	86.08	Cartilage	No
SRX16323957	51658016	Paired	0.103085	0.909483	RF	75.67	Whole blood	No
ERX9275262	23465182	Paired	0.0225192	0.980461	RF	86.94	Muscle	No
ERX9275221	25784043	Paired	0.0261928	0.979039	RF	83.09	Liver	No
SRX16323958	45893422	Paired	0.089523	0.911206	RF	89.65	Whole blood	No
ERX9275263	30546295	Paired	0.0198146	0.98235	RF	86.02	Muscle	No

ERX9275222	24485440	Paired	0.026395	0.978105	RF	83.54	Liver	No
SRX16323959	51705976	Paired	0.0932802	0.91181	RF	74.77	Whole blood	No
SRX11011051	40057640	Paired	0.0453001	0.953249	RF	87.75	Adipose	No
ERX9275264	32020733	Paired	0.0214237	0.980763	RF	85.83	Muscle	No
ERX9275223	34284872	Paired	0.0274046	0.978499	RF	83.8	Liver	No
ERX9275265	27261302	Paired	0.0213994	0.980195	RF	85.76	Muscle	No
ERX9275224	31374119	Paired	0.0369559	0.975013	RF	80.63	Liver	No
SRX14303988	39234284	Paired	0.0207231	0.979995	RF	88.49	Liver	No
ERX9275266	34449184	Paired	0.0188159	0.983188	RF	85.88	Muscle	No
ERX9275225	34943210	Paired	0.0282243	0.978653	RF	82.94	Liver	No
SRX14303989	39838031	Paired	0.0213969	0.979817	RF	88.3	Liver	No
ERX9275267	27748470	Paired	0.0210496	0.980833	RF	86.67	Muscle	No
ERX9275226	24673130	Paired	0.0262458	0.978886	RF	84.47	Liver	No
ERX9275268	24617361	Paired	0.0235148	0.979064	RF	80.27	Muscle	No
ERX9275227	40146970	Paired	0.0231695	0.980547	RF	85.24	Muscle	No
SRX11011056	39796294	Paired	0.0473636	0.951808	RF	88.96	Adipose	No
ERX9275269	27036564	Paired	0.0221018	0.98026	RF	86.56	Muscle	No
ERX9275228	26052355	Paired	0.0226813	0.980201	RF	86.03	Muscle	No
ERX9275229	23132407	Paired	0.0267914	0.977171	RF	85.79	Muscle	No
ERX3981450	24246012	Paired	0.0720398	0.869132	RF	76.54	Heart	No
ERX3981451	28942810	Paired	0.0691009	0.878846	RF	77.39	Heart	No
ERX3981452	33052120	Paired	0.0655351	0.874798	RF	76.2	Heart	No
ERX3981453	30744922	Paired	0.0702887	0.878678	RF	76.8	Heart	No
ERX2987520	21065392	Paired	0.0247384	0.975414	RF	92.47	Brain	No
ERX3981454	36535957	Paired	0.0679464	0.877052	RF	76.71	Heart	No
ERX2987521	20540143	Paired	0.0244422	0.975586	RF	93.46	Brain	No
ERX3981455	37036939	Paired	0.063086	0.886851	RF	77.93	Heart	No
ERX2987522	15190828	Paired	0.025044	0.975062	RF	93.19	Brain	No
ERX3981456	30934439	Paired	0.0680357	0.875255	RF	77	Heart	No
ERX2987523	15093795	Paired	0.0286484	0.971604	RF	93.11	Brain	No
ERX3981457	32365453	Paired	0.082963	0.854758	RF	75.95	Heart	No
ERX2987524	25183308	Paired	0.0243248	0.976012	RF	93.08	Brain	No
ERX3981458	31291135	Paired	0.0694106	0.8722	RF	75.67	Heart	No
ERX2987525	21523776	Paired	0.0247423	0.975518	RF	93.3	Brain	No
ERX3981459	35600094	Paired	0.069411	0.871912	RF	76.26	Heart	No
ERX2987526	24296730	Paired	0.0234523	0.976683	RF	93.26	Brain	No
ERX2987527	18679619	Paired	0.0246163	0.975615	RF	93.52	Brain	No
ERX2987528	30466561	Paired	0.0243874	0.975755	RF	93.48	Brain	No
ERX2987529	19950075	Paired	0.0342026	0.966004	RF	93.55	Brain	No
SRX16061017	32663995	Paired	0.0220186	0.97789	RF	85.91	Brain	No
SRX16061018	32185281	Paired	0.029802	0.969753	RF	84.68	Brain	No
SRX16061019	32007428	Paired	0.0263317	0.973547	RF	86.4	Brain	No
SRX16324022	41270231	Paired	0.0926072	0.914487	RF	83.88	Whole blood	No
SRX14806378	24450172	Paired	0.129735	0.870081	RF	90.71	Liver	No
SRX14806379	25963637	Paired	0.155579	0.843043	RF	88.2	Liver	No
ERX4133000	22516954	Paired	0.0271006	0.973418	RF	93.06	Brain	No
ERX4133001	23120683	Paired	0.026801	0.973813	RF	93.59	Brain	No
ERX4133002	23973003	Paired	0.027052	0.973522	RF	93.01	Brain	No
ERX4133003	19411090	Paired	0.0266233	0.974063	RF	93.18	Brain	No
ERX4133004	44743039	Paired	0.0246985	0.975916	RF	93.82	Brain	No
ERX4133005	22127277	Paired	0.0274527	0.973073	RF	92.55	Brain	No
ERX4133006	21825965	Paired	0.0303779	0.97021	RF	93.65	Brain	No
ERX4133007	23388046	Paired	0.0292496	0.971362	RF	93.15	Brain	No
ERX4133008	23670573	Paired	0.0273959	0.973134	RF	92.72	Brain	No
ERX4133009	23940541	Paired	0.0280655	0.972565	RF	93.6	Brain	No
SRX16723067	4103566	Single	0.0500601	NULL	RF	94.34	Uterus	No
SRX16723068	2117757	Single	0.0713235	NULL	RF	94.16	Uterus	No
SRX16723069	5947808	Single	0.0420178	NULL	RF	94	Uterus	No

SRX14239280	10908376	Single	0.95467	NULL	FR	90.04	Uterus	No
SRX14239281	16365421	Single	0.0361958	NULL	RF	88.07	Uterus	No
SRX14239283	3130260	Single	0.0532302	NULL	RF	93.23	Uterus	No
SRX14239284	2484100	Single	0.934349	NULL	FR	92.34	Uterus	No
SRX14239285	15368738	Single	0.0447912	NULL	RF	86.97	Uterus	No
SRX14239286	12450565	Single	0.941282	NULL	FR	84.42	Uterus	No
SRX14239287	9391412	Single	0.941023	NULL	FR	88.99	Uterus	No
SRX14239288	10855211	Single	0.0519563	NULL	RF	90.51	Uterus	No
SRX14239289	2372677	Single	0.925365	NULL	FR	86.85	Uterus	No
SRX13459900	34557094	Paired	0.0228233	0.976367	RF	87.23	Cartilage	No
SRX13459901	39934376	Paired	0.0214895	0.977898	RF	87.99	Cartilage	No
ERX9275380	33697268	Paired	0.0344514	0.967441	RF	86.01	Liver	No
ERX9275381	17907652	Paired	0.0351237	0.969254	RF	87.1	Liver	No
ERX9275340	28162741	Paired	0.0214326	0.977986	RF	88.16	Muscle	No
SRX10588935	42126584	Paired	0.07941	0.917257	RF	74.72	Liver	No
ERX9275382	24679201	Paired	0.0309844	0.97431	RF	86.44	Liver	No
ERX9275341	26984392	Paired	0.0378473	0.963526	RF	84.27	Muscle	No
ERX9275300	25917931	Paired	0.0261087	0.976596	RF	87.51	Muscle	No
SRX10588936	41284847	Paired	0.0900714	0.904532	RF	76.47	Liver	No
ERX9275342	28226599	Paired	0.0270345	0.974883	RF	87.72	Muscle	No
ERX9275301	24380718	Paired	0.0210403	0.978578	RF	89.24	Muscle	No
SRX10588937	40968195	Paired	0.0870182	0.90705	RF	70.04	Liver	No
ERX9275343	28623648	Paired	0.024361	0.977046	RF	88.71	Muscle	No
ERX9275302	24558009	Paired	0.0222763	0.978886	RF	87.11	Muscle	No
SRX10588938	40251761	Paired	0.0518211	0.947033	RF	79.61	Liver	No
ERX9275344	25085028	Paired	0.0251029	0.976252	RF	88.98	Muscle	No
ERX9275303	22765995	Paired	0.0221889	0.978816	RF	87.42	Muscle	No
SRX10588939	41092044	Paired	0.0909242	0.90448	RF	72.53	Liver	No
ERX9275345	39091853	Paired	0.0243721	0.977049	RF	87.86	Muscle	No
ERX9275304	30967625	Paired	0.0234706	0.977642	RF	88.54	Muscle	No
ERX9275071	24664402	Paired	0.0275132	0.975323	RF	86.68	Liver	No
ERX9275346	33568308	Paired	0.0232246	0.977986	RF	88.75	Muscle	No
ERX9275305	28819099	Paired	0.0229209	0.978978	RF	89.27	Muscle	No
ERX9275072	26627835	Paired	0.0250428	0.976585	RF	86.89	Liver	No
ERX9275347	38866788	Paired	0.0212456	0.979061	RF	88.73	Muscle	No
ERX9275306	27813648	Paired	0.0232368	0.97834	RF	88.04	Muscle	No
ERX9275073	26499448	Paired	0.025602	0.975813	RF	86.77	Liver	No
ERX9275348	40216785	Paired	0.0240817	0.977306	RF	87.84	Muscle	No
ERX9275307	24956090	Paired	0.0253668	0.975071	RF	84.41	Muscle	No
ERX9275074	28795454	Paired	0.0274699	0.974451	RF	85.98	Liver	No
ERX9275349	27591243	Paired	0.0239213	0.977799	RF	88	Muscle	No
ERX9275308	28281673	Paired	0.0247085	0.976022	RF	86.92	Muscle	No
ERX9275075	32671086	Paired	0.0248404	0.976556	RF	85.9	Liver	No
ERX9275309	24059474	Paired	0.0275407	0.971032	RF	85.85	Muscle	No
ERX9275076	33270723	Paired	0.0272511	0.974999	RF	85.92	Liver	No
ERX9275077	23803678	Paired	0.024557	0.977214	RF	86.21	Liver	No
ERX9275078	25737548	Paired	0.0237862	0.978229	RF	86.41	Liver	No
ERX9275079	27397981	Paired	0.0301225	0.970853	RF	85.55	Liver	No
SRX12844226	42364049	Paired	0.0452924	0.953185	RF	89.34	Adipose	No
SRX12844227	38951240	Paired	0.0349242	0.964371	RF	87.25	Adipose	No
SRX12844228	40820123	Paired	0.044413	0.954234	RF	88.42	Adipose	No
SRX12844229	41753968	Paired	0.0447068	0.953861	RF	85.84	Adipose	No
SRX14806490	23935502	Paired	0.131857	0.867676	RF	88.39	Small intestine	No
SRX14806491	27311979	Paired	0.133892	0.865302	RF	88.93	Small intestine	No
SRX14806450	21365689	Paired	0.144421	0.856108	RF	88.86	Small intestine	No
SRX14806451	22598329	Paired	0.129758	0.870207	RF	87.98	Small intestine	No
SRX14806410	20939125	Paired	0.140974	0.857276	RF	88.66	Large intestine	No
SRX14806411	33197843	Paired	0.0696472	0.929631	RF	89.73	Large intestine	No

ERX4132977	22637807	Paired	0.0265412	0.9741	RF	93.42	Brain	No
SRX14806453	27248128	Paired	0.07082	0.928847	RF	89.01	Large intestine	No
SRX14806412	23781655	Paired	0.131732	0.867033	RF	89.12	Small intestine	No
ERX4132978	21310271	Paired	0.0273556	0.973244	RF	93.54	Brain	No
SRX14806454	21415549	Paired	0.163839	0.835266	RF	87.6	Small intestine	No
SRX14806413	21938295	Paired	0.131982	0.867791	RF	88.12	Small intestine	No
ERX4132979	21159356	Paired	0.0272807	0.973288	RF	93.02	Brain	No
SRX14806455	25174504	Paired	0.142978	0.856621	RF	86.74	Small intestine	No
SRX14806414	30846556	Paired	0.12462	0.874295	RF	89.64	Large intestine	No
SRX14806456	23486861	Paired	0.160216	0.837991	RF	87.34	Large intestine	No
SRX14806415	26158865	Paired	0.1467	0.851714	RF	90.49	Liver	No
SRX14806457	20839424	Paired	0.140295	0.85893	RF	87.98	Small intestine	No
SRX14806416	24608789	Paired	0.111271	0.88799	RF	86.69	Large intestine	No
SRX14806458	29757096	Paired	0.126997	0.873442	RF	88.6	Small intestine	No
SRX14806417	25499795	Paired	0.125234	0.874386	RF	88.6	Small intestine	No
SRX14806459	26917113	Paired	0.112907	0.886364	RF	90.56	Liver	No
SRX14806418	25468472	Paired	0.130257	0.869791	RF	88.45	Small intestine	No
SRX14806419	23665867	Paired	0.175126	0.822693	RF	88.17	Large intestine	No
SRX10589007	52246408	Paired	0.0815929	0.913812	RF	66.74	Liver	No
SRX10589008	46140349	Paired	0.0849106	0.911769	RF	78.47	Liver	No
SRX10589009	42249984	Paired	0.0719248	0.925207	RF	74.77	Liver	No
ERX4742270	19838729	Paired	0.028687	0.971847	RF	92.75	PBMCs	No
ERX4742271	17290171	Paired	0.0318396	0.968827	RF	92.08	PBMCs	No
ERX4742230	18421623	Paired	0.0347624	0.965846	RF	92.9	PBMCs	No
ERX4742272	16842102	Paired	0.0344903	0.965885	RF	91.49	PBMCs	No
ERX4742231	26765214	Paired	0.0293701	0.971224	RF	92.26	PBMCs	No
ERX4742273	17427386	Paired	0.0300587	0.970558	RF	91.47	PBMCs	No
ERX4742232	20603156	Paired	0.034995	0.965388	RF	92.38	PBMCs	No
ERX4742274	20545855	Paired	0.0299506	0.970433	RF	92.67	PBMCs	No
ERX4742233	22071240	Paired	0.030874	0.969549	RF	93.05	PBMCs	No
ERX4742275	24327300	Paired	0.0310689	0.96948	RF	91.81	PBMCs	No
ERX4742234	19149666	Paired	0.035526	0.965053	RF	91.93	PBMCs	No
ERX4742276	17863265	Paired	0.0298724	0.970664	RF	91.76	PBMCs	No
ERX4742235	18894898	Paired	0.0323434	0.968194	RF	92.2	PBMCs	No
ERX4742277	21190345	Paired	0.0275846	0.972779	RF	92.95	PBMCs	No
ERX4742236	22220442	Paired	0.0292609	0.971161	RF	92.47	PBMCs	No
ERX4742278	16464842	Paired	0.0309488	0.969628	RF	92.29	PBMCs	No
ERX4742237	20663656	Paired	0.0292499	0.971189	RF	93.01	PBMCs	No
ERX4742279	16665020	Paired	0.0293114	0.971239	RF	91.93	PBMCs	No
ERX4742238	18547809	Paired	0.0307197	0.969731	RF	92.88	PBMCs	No
SRX12142660	21421113	Paired	0.0840944	0.910546	RF	65.89	PBMCs	No
ERX4742239	18201253	Paired	0.0286966	0.971905	RF	93.19	PBMCs	No
SRX12142661	21389166	Paired	0.0848833	0.914202	RF	67.5	PBMCs	No
SRX12142620	23919299	Paired	0.102937	0.889511	RF	62.06	PBMCs	No
SRX12142621	22260499	Paired	0.120808	0.870109	RF	61.02	PBMCs	No
SRX12142624	9733140	Paired	0.0869108	0.908915	RF	69.2	PBMCs	No
SRX12142625	22284016	Paired	0.10445	0.885625	RF	62.46	PBMCs	No
SRX12142626	19214438	Paired	0.137751	0.852246	RF	60.14	PBMCs	No
SRX12142627	22392205	Paired	0.138993	0.851094	RF	62.11	PBMCs	No
SRX12142628	20883104	Paired	0.0961151	0.89389	RF	60.02	PBMCs	No
ERX6202490	25190792	Paired	0.0219544	0.979266	RF	91.94	Uterus	No
ERX6202491	22936393	Paired	0.0202528	0.980842	RF	91.06	Uterus	No
ERX9275190	22187275	Paired	0.0298422	0.975295	RF	86.22	Liver	No
ERX6202492	21369159	Paired	0.0204124	0.980536	RF	91.36	Uterus	No
ERX9275191	31876375	Paired	0.0283445	0.97739	RF	83.29	Liver	No
ERX9275150	28044965	Paired	0.0264824	0.979515	RF	83.68	Liver	No
ERX6202493	20940468	Paired	0.0194408	0.982007	RF	87.51	Uterus	No
ERX9275192	40957537	Paired	0.0224624	0.981747	RF	84.94	Liver	No

ERX9275151	30586612	Paired	0.0404271	0.960035	RF	85.9	Liver	No
ERX6202494	21429388	Paired	0.0196357	0.981486	RF	88.45	Uterus	No
ERX9275110	33633207	Paired	0.02772	0.973161	RF	86.44	Liver	No
ERX9275193	29471717	Paired	0.0254681	0.979487	RF	85.83	Liver	No
ERX9275152	30740869	Paired	0.02637	0.979161	RF	84.63	Liver	No
ERX6202495	20715019	Paired	0.0199915	0.980993	RF	90.42	Uterus	No
ERX9275111	47246879	Paired	0.0275217	0.973517	RF	85.98	Liver	No
ERX9275194	26995864	Paired	0.0242758	0.980316	RF	86.29	Liver	No
ERX9275153	32319387	Paired	0.0265379	0.978802	RF	84.11	Liver	No
ERX6202496	23491729	Paired	0.0205155	0.980666	RF	92.69	Uterus	No
ERX9275112	34648178	Paired	0.025429	0.975338	RF	86.5	Liver	No
ERX9275195	30062145	Paired	0.0243186	0.98074	RF	85.26	Liver	No
ERX9275154	34199477	Paired	0.0284302	0.977882	RF	82.5	Liver	No
ERX6202497	20556673	Paired	0.0216229	0.979724	RF	92.1	Uterus	No
ERX9275113	25915381	Paired	0.0269827	0.973275	RF	86.05	Liver	No
ERX9275196	29561884	Paired	0.0307228	0.971042	RF	85.78	Liver	No
ERX9275155	28475809	Paired	0.0268385	0.979241	RF	84.87	Liver	No
ERX6202498	24859029	Paired	0.0206961	0.980562	RF	90.1	Uterus	No
ERX9275114	23152653	Paired	0.0272653	0.973133	RF	85.62	Liver	No
ERX9275197	24015955	Paired	0.030959	0.972448	RF	86.15	Liver	No
ERX9275156	27287423	Paired	0.0283863	0.976794	RF	84.63	Liver	No
ERX6202499	21265153	Paired	0.0204227	0.98116	RF	88.01	Uterus	No
ERX9275115	25565106	Paired	0.0301128	0.969388	RF	85.87	Liver	No
ERX9275198	38537249	Paired	0.0250806	0.979878	RF	85.37	Liver	No
ERX9275157	30477040	Paired	0.0276401	0.978562	RF	83.51	Liver	No
ERX9275116	28962637	Paired	0.025593	0.974074	RF	86.71	Liver	No
ERX9275199	25375806	Paired	0.0271243	0.978425	RF	85.07	Liver	No
ERX9275158	29399789	Paired	0.0266082	0.979255	RF	84.69	Liver	No
ERX9275117	27721725	Paired	0.0291775	0.970386	RF	86.33	Liver	No
ERX9275159	25602973	Paired	0.0284227	0.978148	RF	83.51	Liver	No
ERX9275118	25870634	Paired	0.0588863	0.940373	RF	79.33	Liver	No
ERX9275119	28979144	Paired	0.0284483	0.974452	RF	87.33	Liver	No
SRX17395020	4856884	Single	0.866565	NULL	FR	76.77	Small intestine	No
SRX17395021	27378452	Single	0.924118	NULL	FR	77.73	Small intestine	No
SRX11216664	39704475	Paired	0.116146	0.884518	RF	69.57	Muscle	No
SRX14241653	31137076	Single	0.0931422	NULL	RF	70.49	Large intestine	No
SRX11216665	37814244	Paired	0.117045	0.876875	RF	78.85	Brain	No
ERX2987490	20288608	Paired	0.0237272	0.976532	RF	93.47	Brain	No
SRX11216666	48856762	Paired	0.106914	0.892469	RF	76.18	Lung	No
ERX2987491	15640313	Paired	0.0257273	0.974534	RF	90.36	Brain	No
SRX14241655	25035682	Single	0.0836465	NULL	RF	70.81	Large intestine	No
SRX11216667	41924092	Paired	0.0825881	0.917459	RF	75.45	Liver	No
ERX2987492	30076251	Paired	0.0249185	0.97532	RF	89.68	Brain	No
SRX14241656	21678767	Single	0.0801983	NULL	RF	76.21	Large intestine	No
SRX11216668	41210961	Paired	0.0743536	0.923708	RF	73.09	Adipose	No
ERX2987493	17020169	Paired	0.0245415	0.975758	RF	93.22	Brain	No
SRX14241657	20851750	Single	0.0806694	NULL	RF	68.84	Large intestine	No
ERX2987494	30740438	Paired	0.0261042	0.974151	RF	93.27	Brain	No
ERX2987495	27177697	Paired	0.0241941	0.97597	RF	93.52	Brain	No
ERX2987496	23518159	Paired	0.0250235	0.975225	RF	93.28	Brain	No
ERX2987497	14354502	Paired	0.0262499	0.973894	RF	93.39	Brain	No
ERX9915175	30083022	Paired	0.036945	0.960188	RF	85.89	Brain	No
ERX2987498	25087733	Paired	0.0231779	0.976903	RF	93.43	Brain	No
ERX9915176	29698748	Paired	0.0399738	0.957155	RF	83.98	Brain	No
ERX2987499	22897912	Paired	0.0243433	0.975926	RF	93.38	Brain	No
ERX9915177	26976463	Paired	0.0409653	0.956566	RF	85.2	Brain	No
ERX9915178	26629384	Paired	0.0649363	0.927516	RF	86.23	Brain	No
ERX9915179	29529913	Paired	0.0432908	0.954327	RF	86.59	Brain	No

SRX14748312	46347070	Single	0.0821356	NULL	RF	85.23	Small intestine	No
SRX14748313	59040444	Single	0.0466129	NULL	RF	90.18	Small intestine	No
SRX14748314	67152106	Single	0.0480959	NULL	RF	88.8	Small intestine	No
SRX14748315	61030556	Single	0.0562805	NULL	RF	89.03	Small intestine	No
SRX14748316	49213726	Single	0.057756	NULL	RF	87.46	Small intestine	No
SRX14748317	62111752	Single	0.04746	NULL	RF	89.94	Small intestine	No
SRX14748318	55728902	Single	0.0490325	NULL	RF	89.74	Small intestine	No
SRX14748319	22220755	Single	0.0637027	NULL	RF	87.79	Small intestine	No
SRX19561480	58405421	Single	0.0654164	NULL	RF	67.29	PBMCs	No
SRX19561481	58712436	Single	0.0517088	NULL	RF	71.32	PBMCs	No
SRX19561482	53884793	Single	0.0576257	NULL	RF	73.93	PBMCs	No
SRX19561483	45608096	Single	0.0570354	NULL	RF	67.74	PBMCs	No
SRX19561484	48856612	Single	0.0582361	NULL	RF	78.09	PBMCs	No
SRX19561485	50698263	Single	0.0635576	NULL	RF	73.21	PBMCs	No
SRX19561486	66238187	Single	0.0523482	NULL	RF	77.7	PBMCs	No
SRX19561487	58027257	Single	0.0569856	NULL	RF	75.56	PBMCs	No
SRX19561489	51717726	Single	0.0631917	NULL	RF	68.94	PBMCs	No
SRX10629590	76039519	Paired	0.142033	0.855977	RF	78.36	Brain	No
SRX10629591	84670812	Paired	0.134352	0.863905	RF	76.09	Brain	No
SRX10629592	87202420	Paired	0.140766	0.851989	RF	80.48	Brain	No
SRX10629593	75063596	Paired	0.146954	0.847176	RF	78.79	Brain	No
SRX10629594	75393262	Paired	0.145934	0.848205	RF	80.62	Brain	No
SRX16323960	40028123	Paired	0.0919544	0.914729	RF	86.64	Whole blood	No
SRX10629595	97544256	Paired	0.130463	0.864514	RF	77.54	Brain	No
SRX16323961	40267422	Paired	0.0890114	0.914424	RF	84.7	Whole blood	No
SRX10629596	86701986	Paired	0.0969434	0.899589	RF	67.1	Macrophage	No
SRX19802424	48222471	Paired	0.0331625	0.96638	RF	86.17	Cartilage	No
SRX16323962	48133847	Paired	0.0937	0.911857	RF	79.19	Whole blood	No
SRX10629597	76790922	Paired	0.13311	0.864107	RF	73.73	Brain	No
SRX19802425	48832357	Paired	0.0442651	0.958739	RF	87.41	Cartilage	No
SRX16323963	41411485	Paired	0.0890774	0.915418	RF	87	Whole blood	No
SRX14303990	39777250	Paired	0.019897	0.981148	RF	87.88	Liver	No
SRX10629598	83459268	Paired	0.130878	0.863063	RF	80.14	Brain	No
SRX19802426	45581823	Paired	0.0349924	0.965552	RF	85.41	Cartilage	No
SRX16323964	39191464	Paired	0.0916121	0.91137	RF	89.07	Whole blood	No
SRX14303991	38765112	Paired	0.0200907	0.980694	RF	87.41	Liver	No
SRX10629599	76233505	Paired	0.115911	0.869615	RF	73	Brain	No
SRX19802427	47813818	Paired	0.0383572	0.963873	RF	81.46	Cartilage	No
SRX16323965	49624273	Paired	0.0912609	0.915843	RF	77.93	Whole blood	No
SRX14303992	40450668	Paired	0.0258001	0.975381	RF	87.88	Liver	No
ERX9275270	33675405	Paired	0.0270169	0.977135	RF	84.72	Muscle	No
SRX19802428	53520234	Paired	0.0283693	0.969764	RF	80.18	Cartilage	No
SRX16323966	44711384	Paired	0.0993827	0.902347	RF	86.52	Whole blood	No
SRX14303993	40569855	Paired	0.0272488	0.973559	RF	86.77	Liver	No
ERX9275271	32684895	Paired	0.026865	0.980313	RF	83.36	Muscle	No
ERX9275230	31843660	Paired	0.0219298	0.981776	RF	84.77	Muscle	No
SRX19802429	50471256	Paired	0.0284196	0.973142	RF	83.43	Cartilage	No
SRX16323967	39939482	Paired	0.103562	0.905531	RF	85.06	Whole blood	No
SRX14303994	39252291	Paired	0.0262729	0.9748	RF	88.21	Liver	No
SRX13459878	38722867	Paired	0.025871	0.97304	RF	90.25	Cartilage	No
ERX9275272	32590275	Paired	0.0237985	0.981716	RF	84.36	Muscle	No
ERX9275231	25243180	Paired	0.0227813	0.980427	RF	85.6	Muscle	No
SRX16323968	41707032	Paired	0.0990346	0.910539	RF	77.43	Whole blood	No
SRX14303995	38447320	Paired	0.0273253	0.973309	RF	88.2	Liver	No
SRX13459879	34566956	Paired	0.0260255	0.972711	RF	89.54	Cartilage	No
ERX9275273	31369011	Paired	0.0226992	0.980341	RF	86.22	Muscle	No
ERX9275232	29999156	Paired	0.0214354	0.981866	RF	86.38	Muscle	No
SRX16323969	51853015	Paired	0.0901744	0.92116	RF	65.92	Whole blood	No

ERX9275274	29039996	Paired	0.0225316	0.979276	RF	85.91	Muscle	No
ERX9275233	21908439	Paired	0.0232426	0.97939	RF	85.88	Muscle	No
ERX9275275	28231325	Paired	0.0225086	0.978983	RF	87.61	Muscle	No
ERX9275234	34411463	Paired	0.0191502	0.983808	RF	84.71	Muscle	No
SRX11011063	41350298	Paired	0.0515455	0.947245	RF	88.57	Adipose	No
ERX9275276	26344745	Paired	0.0231013	0.979282	RF	87.57	Muscle	No
ERX9275235	31064675	Paired	0.0210583	0.982359	RF	82.96	Muscle	No
SRX11011064	42134592	Paired	0.053007	0.945233	RF	88.95	Adipose	No
ERX9275277	35664591	Paired	0.0224941	0.976946	RF	88.59	Muscle	No
ERX9275236	27127421	Paired	0.0220306	0.981754	RF	84.88	Muscle	No
ERX9275278	36650802	Paired	0.0259767	0.97636	RF	86.89	Muscle	No
ERX9275237	22241656	Paired	0.0235586	0.979998	RF	85.26	Muscle	No
ERX9275279	34478664	Paired	0.0204528	0.982777	RF	86.92	Muscle	No
ERX9275238	26274144	Paired	0.0233035	0.979991	RF	84.99	Muscle	No
SRX11011067	39703779	Paired	0.0569014	0.941994	RF	89.99	Adipose	No
ERX9275239	21434914	Paired	0.0234277	0.978768	RF	84.67	Muscle	No
SRX14418519	16848109	Paired	0.0392374	0.956963	RF	86.8	PBMCs	No
SRX19452655	48040756	Paired	0.025482	0.974434	RF	84.27	Artery	No
SRX11216700	52230481	Paired	0.111035	0.883632	RF	80.28	Brain	No
SRX11216701	48159463	Paired	0.0935968	0.903191	RF	86.01	Brain	No
ERX3981460	31046544	Paired	0.0636732	0.890698	RF	76.59	Heart	No
SRX11216702	39400806	Paired	0.107208	0.886789	RF	85.33	Heart	No
ERX3981461	27319935	Paired	0.0880246	0.845376	RF	73.37	Heart	No
SRX11216703	34722722	Paired	0.164844	0.836586	RF	60.33	Muscle	No
ERX3981462	26682191	Paired	0.0694779	0.875423	RF	77.14	Heart	No
SRX11216704	48992590	Paired	0.0800543	0.919352	RF	78.33	Liver	No
ERX3981463	27095616	Paired	0.0714564	0.868151	RF	77.47	Heart	No
SRX11216705	47829280	Paired	0.108948	0.888733	RF	80.1	Lung	No
ERX3981464	28125047	Paired	0.0641541	0.887462	RF	77.17	Heart	No
ERX2987530	29884839	Paired	0.0245268	0.975838	RF	92.83	Brain	No
SRX11216706	40243327	Paired	0.0934692	0.906656	RF	89.26	Small intestine	No
ERX3981465	30663761	Paired	0.0660736	0.883222	RF	78.59	Heart	No
ERX2987531	18382632	Paired	0.0237504	0.976527	RF	89.49	Brain	No
ERX3981466	30515501	Paired	0.0678923	0.880009	RF	77.49	Heart	No
ERX2987532	25480827	Paired	0.0240363	0.976062	RF	92.74	Brain	No
ERX3981467	30047449	Paired	0.0673039	0.878177	RF	79.24	Heart	No
SRX11216709	48627741	Paired	0.0927835	0.902834	RF	82.33	Brain	No
SRX16061020	39590696	Paired	0.0372098	0.961058	RF	83.38	Brain	No
SRX16061021	38452704	Paired	0.0402643	0.958454	RF	84.16	Brain	No
SRX16061022	40874014	Paired	0.0339909	0.964761	RF	84.8	Brain	No
SRX14806380	28762785	Paired	0.176181	0.822043	RF	88.52	Liver	No
SRX14806381	23522190	Paired	0.136879	0.861001	RF	88.72	Large intestine	No
SRX14806382	23721092	Paired	0.135768	0.861981	RF	89.85	Large intestine	No
SRX14806383	22859024	Paired	0.18001	0.817628	RF	85.19	Small intestine	No
SRX14806384	25133345	Paired	0.132288	0.867952	RF	88.26	Small intestine	No
SRX14806385	25959446	Paired	0.146256	0.851843	RF	88.95	Large intestine	No
SRX14806386	22490856	Paired	0.170802	0.827128	RF	87.4	Large intestine	No
SRX14806387	20863429	Paired	0.180982	0.817793	RF	85.5	Small intestine	No
SRX16324033	40708970	Paired	0.10066	0.900855	RF	89.07	Whole blood	No
SRX14806388	28089971	Paired	0.120573	0.879763	RF	88.12	Small intestine	No
SRX14806389	22744371	Paired	0.170463	0.827562	RF	87.83	Large intestine	No
SRX16324038	48604482	Paired	0.0801697	0.922674	RF	88.66	Whole blood	No
ERX4133010	23012875	Paired	0.0274659	0.973071	RF	92.46	Brain	No
ERX4133011	22225457	Paired	0.0259799	0.97451	RF	92.33	Brain	No
ERX4133012	22294713	Paired	0.027894	0.972749	RF	92.49	Brain	No
ERX4133013	22264519	Paired	0.0273265	0.973216	RF	93.02	Brain	No
SRX8361278	24252186	Paired	0.0279356	0.963949	RF	87.85	Fibroblast	No
SRX16723070	6353744	Single	0.0406521	NULL	RF	94.21	Uterus	No

ERX4133014	23460378	Paired	0.0276698	0.972932	RF	92.64	Brain	No
SRX8361279	24163786	Paired	0.0275061	0.964761	RF	87.62	Fibroblast	No
SRX16723071	4165736	Single	0.0480381	NULL	RF	93.86	Uterus	No
ERX4133015	22714560	Paired	0.0266656	0.973924	RF	93.06	Brain	No
SRX16723072	3734793	Single	0.050605	NULL	RF	94.56	Uterus	No
ERX4133016	22200646	Paired	0.0303826	0.970303	RF	92.82	Brain	No
SRX16723073	680045	Single	0.118137	NULL	RF	94.45	Uterus	No
ERX4133017	19252907	Paired	0.0295455	0.971011	RF	92.11	Brain	No
SRX16723074	4210588	Single	0.0397147	NULL	RF	93.67	Ovary	No
ERX4133018	22036543	Paired	0.0275653	0.973034	RF	92.41	Brain	No
SRX16849382	65287871	Paired	0.0200511	0.98095	RF	87.97	Kidney	No
SRX16723075	4906432	Single	0.0373007	NULL	RF	94.21	Ovary	No
ERX4133019	23261888	Paired	0.027688	0.972917	RF	93.41	Brain	No
SRX16849383	33613573	Paired	0.0227508	0.977194	RF	85.28	Kidney	No
SRX16723076	4122280	Single	0.0404693	NULL	RF	93.94	Ovary	No
SRX16849384	35882031	Paired	0.0238153	0.978003	RF	89.08	Kidney	No
SRX16723077	4813920	Single	0.03843	NULL	RF	93.68	Ovary	No
ERX8087579	53349594	Paired	0.0154364	0.985344	RF	93.13	Ovary	No
SRX16849385	37375819	Paired	0.0222914	0.979159	RF	86.79	Kidney	No
SRX16723078	2895386	Single	0.0457363	NULL	RF	94.33	Ovary	No
SRX16849386	39342131	Paired	0.0220517	0.979338	RF	88.57	Kidney	No
SRX16723079	6863146	Single	0.044815	NULL	RF	93.98	Uterus	No
SRX16849387	37254437	Paired	0.0222752	0.979003	RF	88.19	Kidney	No
SRX16849388	38082889	Paired	0.0232519	0.978341	RF	88.26	Kidney	No
SRX16849389	38628544	Paired	0.0325422	0.970166	RF	85.64	Kidney	No
SRX14239290	3502718	Single	0.0657185	NULL	RF	88.82	Uterus	No
SRX14239291	20540150	Single	0.0359794	NULL	RF	87.71	Uterus	No
SRX14239292	1395770	Single	0.899204	NULL	FR	85.51	Uterus	No
SRX12142590	11262397	Paired	0.0874689	0.907805	RF	73.04	PBMCs	No
SRX12142591	8574652	Paired	0.104447	0.892146	RF	71.33	PBMCs	No
SRX12142593	18319734	Paired	0.0960831	0.907479	RF	71.09	PBMCs	No
SRX12142594	22682246	Paired	0.0725099	0.921108	RF	68.35	PBMCs	No
SRX12142595	18641356	Paired	0.0930921	0.898657	RF	67.74	PBMCs	No
SRX12142596	18467550	Paired	0.0915726	0.899695	RF	68.89	PBMCs	No
SRX12142597	15338656	Paired	0.0992254	0.891454	RF	61.86	PBMCs	No
SRX15951985	41867878	Paired	0.108879	0.894326	RF	81.39	Muscle	No
SRX12142598	31792694	Paired	0.0719967	0.921422	RF	65.03	PBMCs	No
SRX15951986	40653594	Paired	0.105186	0.899757	RF	81.96	Muscle	No
SRX12142599	25348522	Paired	0.11459	0.875748	RF	68.36	PBMCs	No
SRX15951987	48349704	Paired	0.123177	0.880311	RF	84.61	Muscle	No
SRX15951988	43514082	Paired	0.11441	0.888816	RF	81.83	Muscle	No
SRX10588941	35508615	Paired	0.0784037	0.918817	RF	74.67	Liver	No
SRX15951989	38272296	Paired	0.107794	0.895956	RF	78.99	Muscle	No
SRX10588942	43607063	Paired	0.0922354	0.902129	RF	73.07	Liver	No
SRX10588943	42134743	Paired	0.0892943	0.906327	RF	75.04	Liver	No
SRX10588944	52475629	Paired	0.0904535	0.903752	RF	76.83	Liver	No
ERX9275350	40330701	Paired	0.023073	0.978228	RF	88.16	Muscle	No
SRX10588945	47410875	Paired	0.0928695	0.902685	RF	74.78	Liver	No
ERX9275351	29342001	Paired	0.0255032	0.975489	RF	87.42	Muscle	No
ERX9275310	21338566	Paired	0.0238408	0.974102	RF	86.32	Muscle	No
SRX10588946	39625893	Paired	0.0756017	0.923662	RF	78.8	Liver	No
ERX9275352	33181637	Paired	0.023743	0.977566	RF	88.33	Muscle	No
ERX9275311	30999646	Paired	0.0245269	0.976673	RF	87.01	Muscle	No
SRX10588947	39261771	Paired	0.0957613	0.903096	RF	72.52	Liver	No
ERX9275353	22697897	Paired	0.0285968	0.974402	RF	87.58	Muscle	No
ERX9275312	37043569	Paired	0.0270715	0.973364	RF	84.89	Muscle	No
SRX10588948	38004146	Paired	0.0879718	0.909599	RF	73.88	Liver	No
ERX9275354	23257444	Paired	0.0333582	0.973206	RF	85.44	Muscle	No

ERX9275313	28469395	Paired	0.0272638	0.974066	RF	87.36	Muscle	No
ERX9275080	27857314	Paired	0.0259715	0.974909	RF	86.74	Liver	No
SRX10588949	43832803	Paired	0.0986375	0.904173	RF	73.11	Liver	No
ERX9275355	19561249	Paired	0.0875194	0.927556	RF	60.85	Muscle	No
ERX9275314	27230282	Paired	0.0244952	0.975058	RF	87.01	Muscle	No
ERX9275081	20349725	Paired	0.0310198	0.970958	RF	86.08	Liver	No
ERX9275356	24057873	Paired	0.0598961	0.948027	RF	73.63	Muscle	No
ERX9275315	28720081	Paired	0.0256339	0.975086	RF	85.25	Muscle	No
ERX9275082	20124356	Paired	0.0305938	0.971215	RF	85.6	Liver	No
ERX9275357	31224853	Paired	0.0310951	0.967131	RF	86.3	Muscle	No
ERX9275316	27242697	Paired	0.0238317	0.976502	RF	87.09	Muscle	No
ERX9275083	26715593	Paired	0.0260032	0.975741	RF	85.96	Liver	No
ERX9275358	30457810	Paired	0.0253931	0.975275	RF	85	Muscle	No
ERX9275317	32387050	Paired	0.0230659	0.978226	RF	88.54	Muscle	No
ERX9275084	25134391	Paired	0.0363449	0.965983	RF	86.13	Liver	No
ERX9275359	25512031	Paired	0.0245809	0.975977	RF	87.05	Muscle	No
ERX9275318	32087537	Paired	0.0236483	0.978204	RF	88.24	Muscle	No
ERX9275085	27923203	Paired	0.0296471	0.971745	RF	85.94	Liver	No
ERX9275319	27974215	Paired	0.0237914	0.977384	RF	88.64	Muscle	No
ERX9275086	29147254	Paired	0.0247511	0.976372	RF	86.08	Liver	No
ERX9275087	28903896	Paired	0.0304818	0.970784	RF	86.15	Liver	No
ERX9275088	28699871	Paired	0.0288691	0.971597	RF	86.22	Liver	No
ERX9275089	25378517	Paired	0.0272816	0.974152	RF	86.68	Liver	No
SRX11399719	64944343	Paired	0.0562403	0.933277	RF	87.61	Uterus	No
ERX4132980	23313062	Paired	0.0257051	0.974882	RF	93.52	Brain	No
SRX9515424	61203313	Single	0.0281992	NULL	RF	93.32	Muscle	No
ERX4132981	21922388	Paired	0.0268896	0.973719	RF	93.92	Brain	No
SRX9515425	58145144	Single	0.0300789	NULL	RF	93.24	Muscle	No
ERX4132982	22847488	Paired	0.0267444	0.97379	RF	93.48	Brain	No
SRX9515426	58082445	Single	0.0296286	NULL	RF	93.28	Muscle	No
ERX4132983	22927465	Paired	0.0325876	0.967958	RF	92.79	Brain	No
SRX9515427	51359202	Single	0.0291075	NULL	RF	93.09	Muscle	No
ERX4132984	24157486	Paired	0.0265858	0.973972	RF	92.61	Brain	No
SRX9515428	52410989	Single	0.029211	NULL	RF	93.13	Muscle	No
SRX14806460	30305494	Paired	0.123995	0.874705	RF	88.55	Large intestine	No
ERX4132985	22792065	Paired	0.0287287	0.971805	RF	92.53	Brain	No
SRX9515429	52855244	Single	0.0287348	NULL	RF	93.46	Muscle	No
SRX14806461	26813203	Paired	0.0741831	0.925723	RF	87.74	Large intestine	No
SRX14806420	33163092	Paired	0.105015	0.894711	RF	87.2	Large intestine	No
ERX4132986	23966522	Paired	0.0275207	0.973013	RF	93.08	Brain	No
SRX14806462	24955854	Paired	0.131648	0.868123	RF	89.89	Small intestine	No
SRX14806421	24388125	Paired	0.125392	0.873885	RF	89.89	Small intestine	No
ERX4132987	23948274	Paired	0.027798	0.972737	RF	92.87	Brain	No
SRX14806463	25391742	Paired	0.11002	0.88878	RF	89.36	Large intestine	No
SRX14806422	24677501	Paired	0.133436	0.866167	RF	88.66	Small intestine	No
ERX4132988	25047897	Paired	0.0264562	0.974086	RF	92.98	Brain	No
SRX14806464	24374607	Paired	0.0735771	0.925918	RF	89.34	Large intestine	No
SRX14806423	22934233	Paired	0.179708	0.81817	RF	87.8	Large intestine	No
ERX4132989	23114956	Paired	0.028099	0.972472	RF	93.22	Brain	No
SRX14806465	27586328	Paired	0.16947	0.828911	RF	86.73	Small intestine	No
SRX14806424	29765099	Paired	0.073487	0.926127	RF	89.23	Large intestine	No
SRX14806466	27861706	Paired	0.124319	0.875522	RF	89.06	Small intestine	No
SRX14806425	23984104	Paired	0.116962	0.882888	RF	89.37	Small intestine	No
SRX10589010	52032672	Paired	0.0746493	0.922152	RF	74.29	Liver	No
SRX14806467	29938121	Paired	0.113767	0.884572	RF	90.23	Large intestine	No
SRX14806426	24651787	Paired	0.12408	0.875036	RF	90.68	Liver	No
SRX10589011	41976920	Paired	0.0719949	0.925379	RF	72.67	Liver	No
SRX14806468	21329177	Paired	0.0968367	0.902713	RF	87.36	Large intestine	No

SRX14806427	29277530	Paired	0.129228	0.869196	RF	90.01	Large intestine	No
SRX14806469	28281259	Paired	0.144126	0.854949	RF	87.83	Small intestine	No
SRX14806428	23302573	Paired	0.074009	0.925631	RF	88.8	Large intestine	No
SRX14806429	23997364	Paired	0.136548	0.863347	RF	88.5	Small intestine	No
SRX12384054	37816195	Paired	0.0393841	0.96016	RF	88.76	Whole blood	No
SRX12384055	30797453	Paired	0.0423249	0.95602	RF	88.25	Whole blood	No
SRX12384056	39793134	Paired	0.0538301	0.948619	RF	84.19	Whole blood	No
SRX12384057	31537891	Paired	0.0418456	0.955994	RF	88.9	Whole blood	No
SRX12384058	55478251	Paired	0.0624135	0.948375	RF	76.53	Whole blood	No
ERX4742280	21722360	Paired	0.043369	0.957336	RF	91.78	PBMCs	No
ERX4742281	18097446	Paired	0.0408462	0.9596	RF	92.65	PBMCs	No
ERX4742240	21878849	Paired	0.029795	0.970669	RF	92.19	PBMCs	No
ERX4742282	21069044	Paired	0.0314916	0.96906	RF	93.13	PBMCs	No
ERX4742241	20323420	Paired	0.0289824	0.971579	RF	93.31	PBMCs	No
ERX4742283	19796706	Paired	0.0348764	0.965509	RF	92.47	PBMCs	No
ERX4742242	20283112	Paired	0.0319613	0.968569	RF	93.38	PBMCs	No
ERX4742284	19027151	Paired	0.0310758	0.969495	RF	92.22	PBMCs	No
ERX4742243	19410317	Paired	0.0298208	0.970706	RF	93.08	PBMCs	No
ERX4742285	21069598	Paired	0.0304149	0.970073	RF	93.56	PBMCs	No
ERX4742244	18172247	Paired	0.0313898	0.968991	RF	93.36	PBMCs	No
ERX4742286	18881658	Paired	0.0333021	0.967189	RF	91.54	PBMCs	No
ERX4742245	19123748	Paired	0.0330794	0.96741	RF	92.18	PBMCs	No
ERX4742287	17639227	Paired	0.0321872	0.968369	RF	92.35	PBMCs	No
ERX4742246	18652232	Paired	0.0291772	0.97131	RF	93.07	PBMCs	No
ERX4742288	19565066	Paired	0.036134	0.964292	RF	92.14	PBMCs	No
ERX4742247	16499074	Paired	0.0332797	0.967306	RF	91.83	PBMCs	No
ERX4742289	21135478	Paired	0.0316956	0.968826	RF	93.19	PBMCs	No
ERX4742248	17565733	Paired	0.0340755	0.966479	RF	92	PBMCs	No
ERX4742249	20466827	Paired	0.0295026	0.971134	RF	91.71	PBMCs	No
ERX4742208	20703186	Paired	0.0360413	0.964323	RF	93.48	PBMCs	No
ERX4742209	20616498	Paired	0.0451102	0.955538	RF	91.54	PBMCs	No
SRX12142633	26939949	Paired	0.11403	0.880921	RF	71.43	PBMCs	No
SRX12142634	24427927	Paired	0.141343	0.850831	RF	66.76	PBMCs	No
SRX12142635	9285012	Paired	0.107359	0.889104	RF	66.88	PBMCs	No
SRX12142636	17598375	Paired	0.185914	0.80954	RF	68.96	PBMCs	No
SRX12142637	21022224	Paired	0.0987971	0.895928	RF	72.78	PBMCs	No
SRX13373749	71736008	Paired	0.0272054	0.973902	RF	90.74	Macrophage	No
SRX12142638	26916152	Paired	0.0789057	0.915978	RF	61.86	PBMCs	No
SRX12142639	18518094	Paired	0.137409	0.854724	RF	66.49	PBMCs	No
ERX9275160	26194790	Paired	0.0264926	0.97853	RF	84.68	Liver	No
ERX9275161	28963731	Paired	0.0316142	0.976559	RF	80.64	Liver	No
ERX9275120	30417709	Paired	0.0230422	0.978864	RF	88.04	Liver	No
SRX13507544	38108510	Paired	0.0757922	0.924212	RF	80.98	Ovary	No
ERX9275162	24598570	Paired	0.0264715	0.978969	RF	82.71	Liver	No
ERX9275121	30683276	Paired	0.0253332	0.97691	RF	87.57	Liver	No
SRX13507545	39551718	Paired	0.0654251	0.934219	RF	83.17	Ovary	No
ERX9275163	25610754	Paired	0.0440647	0.957788	RF	85.82	Liver	No
ERX9275122	27919012	Paired	0.023019	0.978882	RF	87.76	Liver	No
SRX13507546	37421638	Paired	0.0837757	0.916055	RF	84.42	Ovary	No
ERX9275164	27052939	Paired	0.027505	0.977749	RF	85.2	Liver	No
ERX9275123	23466669	Paired	0.0296661	0.97382	RF	86.19	Liver	No
SRX13507547	42450543	Paired	0.0838736	0.915819	RF	85.53	Ovary	No
ERX9275165	25729200	Paired	0.0290104	0.977393	RF	82.85	Liver	No
ERX9275124	28876376	Paired	0.0269075	0.976816	RF	86.67	Liver	No
SRX13507548	43547322	Paired	0.0749343	0.925015	RF	82.79	Ovary	No
ERX9275166	26970431	Paired	0.0299597	0.978012	RF	83.25	Liver	No
ERX9275125	29047387	Paired	0.0222697	0.979943	RF	86.82	Liver	No
SRX13507549	40249813	Paired	0.0680576	0.932534	RF	84.64	Ovary	No

ERX9275167	29102100	Paired	0.0262762	0.979464	RF	84.55	Liver	No
ERX9275126	29495178	Paired	0.0236439	0.97847	RF	87.54	Liver	No
ERX9275168	30158033	Paired	0.0263792	0.978221	RF	84.79	Liver	No
ERX9275127	26860580	Paired	0.0255823	0.976358	RF	87.37	Liver	No
ERX9275169	27637607	Paired	0.0284246	0.97815	RF	84.15	Liver	No
ERX9275128	26520416	Paired	0.0231475	0.978851	RF	87.52	Liver	No
ERX9275129	28242661	Paired	0.0212316	0.980836	RF	86.76	Liver	No
SRX15623527	18177721	Paired	0.0273884	0.970813	RF	83.65	Early embryo	No
SRX15623528	19015566	Paired	0.0272023	0.972151	RF	86.34	Early embryo	No
SRX15623529	19230528	Paired	0.0250808	0.974192	RF	85.2	Early embryo	No
SRX11216670	42052623	Paired	0.0767579	0.923365	RF	72.04	Small intestine	No
SRX11216671	44299289	Paired	0.111874	0.886056	RF	74.77	Brain	No
SRX9105601	44364586	Paired	0.0286483	0.972597	RF	88	Muscle	No
SRX14241660	45888892	Single	0.0759374	NULL	RF	74.16	Large intestine	No
SRX11216672	49299440	Paired	0.114343	0.884517	RF	74.25	Heart	No
SRX9105602	47153285	Paired	0.0335841	0.968132	RF	88.83	Muscle	No
SRX11216673	57375769	Paired	0.0897929	0.911925	RF	73.36	Muscle	No
SRX9105603	45591104	Paired	0.0287292	0.973783	RF	89.61	Muscle	No
SRX9105604	56579987	Paired	0.0238669	0.978883	RF	90.54	Muscle	No
SRX14241663	61756705	Single	0.0905802	NULL	RF	72.76	Large intestine	No
SRX14241622	46245627	Single	0.0905145	NULL	RF	63.39	Large intestine	No
SRX11216675	39298382	Paired	0.105197	0.891626	RF	76.91	Brain	No
ERX9900014	29698748	Paired	0.0399086	0.957018	RF	83.98	Brain	No
SRX9105605	54171141	Paired	0.0245893	0.977965	RF	87.2	Muscle	No
SRX14241664	10455025	Single	0.0982544	NULL	RF	72.07	Large intestine	No
SRX14241623	35663010	Single	0.0801384	NULL	RF	63.98	Large intestine	No
SRX11216676	44940571	Paired	0.119248	0.880899	RF	80.1	Lung	No
ERX9900015	26976463	Paired	0.0409101	0.956527	RF	85.2	Brain	No
SRX9105606	46983509	Paired	0.0287636	0.973311	RF	89.57	Muscle	No
SRX14241665	30698300	Single	0.0900787	NULL	RF	63.89	Large intestine	No
SRX14241624	32759294	Single	0.0883086	NULL	RF	74.14	Large intestine	No
SRX11216677	50306602	Paired	0.0773018	0.923216	RF	77.85	Liver	No
ERX9900016	26629384	Paired	0.0649476	0.928338	RF	86.23	Brain	No
SRX9105607	47334548	Paired	0.0271789	0.975091	RF	89.83	Muscle	No
SRX11216678	41079609	Paired	0.0933931	0.904727	RF	77.36	Adipose	No
ERX9915180	27610544	Paired	0.0608248	0.934109	RF	89.24	Brain	No
ERX9900017	29529913	Paired	0.0432909	0.954271	RF	86.59	Brain	No
SRX9105608	40768413	Paired	0.0262695	0.97592	RF	90.2	Muscle	No
SRX14241667	27627841	Single	0.084803	NULL	RF	71.05	Large intestine	No
SRX14241626	30432247	Single	0.0816913	NULL	RF	74.5	Large intestine	No
ERX9915181	29500539	Paired	0.0547659	0.943439	RF	88.34	Brain	No
ERX9900018	27610544	Paired	0.060817	0.934235	RF	89.24	Brain	No
SRX9105609	47640782	Paired	0.0235076	0.978299	RF	90.06	Muscle	No
SRX14241627	27125154	Single	0.0782003	NULL	RF	73.49	Large intestine	No
ERX9915182	28420284	Paired	0.0471643	0.949827	RF	83.9	Brain	No
ERX9900019	29500539	Paired	0.0548195	0.943438	RF	88.34	Brain	No
SRX14241628	23981996	Single	0.0869161	NULL	RF	74.33	Large intestine	No
ERX9915183	32563515	Paired	0.0567945	0.940126	RF	87.42	Brain	No
ERX9915184	26920624	Paired	0.0560449	0.939866	RF	87.19	Brain	No
SRX16711689	28921578	Single	0.048716	NULL	RF	74.82	PK15	No
SRX14748320	59193499	Single	0.0400053	NULL	RF	88.41	Small intestine	No
SRX11003211	58877658	Single	0.0229777	NULL	RF	83.17	Fibroblast	No
SRX14748321	50883457	Single	0.0488561	NULL	RF	89.66	Small intestine	No
SRX13443401	42211345	Single	0.0415759	NULL	RF	82.32	Cartilage	No
SRX11003212	68536584	Single	0.020831	NULL	RF	81.91	Fibroblast	No
SRX14748322	51027698	Single	0.0600037	NULL	RF	88.35	Small intestine	No
SRX13443402	45102387	Single	0.0330322	NULL	RF	65.16	Cartilage	No
SRX11003213	55891414	Single	0.024463	NULL	RF	75.72	Fibroblast	No

SRX14748323	24358871	Single	0.0435778	NULL	RF	90.69	Small intestine	No
SRX13443403	40269347	Single	0.037923	NULL	RF	70.14	Cartilage	No
SRX11003214	59949301	Single	0.0248718	NULL	RF	85.71	Fibroblast	No
SRX14748324	45450994	Single	0.0724455	NULL	RF	89.99	Small intestine	No
SRX13443404	41515959	Single	0.038915	NULL	RF	89.13	Cartilage	No
SRX14748325	57249945	Single	0.0438537	NULL	RF	87.64	Small intestine	No
SRX13443405	41853691	Single	0.0363201	NULL	RF	83.03	Cartilage	No
SRX11003216	74765183	Single	0.0271325	NULL	RF	69.32	Fibroblast	No
SRX14748326	48345414	Single	0.0571568	NULL	RF	87.97	Small intestine	No
SRX13443406	38949280	Single	0.0361961	NULL	RF	84.36	Cartilage	No
SRX14748327	40905201	Single	0.0425113	NULL	RF	91.05	Small intestine	No
SRX13443407	37434957	Single	0.0418619	NULL	RF	84.91	Cartilage	No
SRX14748328	48392071	Single	0.0395798	NULL	RF	91.56	Small intestine	No
SRX13443408	41408225	Single	0.0315689	NULL	RF	78.74	Cartilage	No
SRX14748329	40707649	Single	0.0803903	NULL	RF	85.2	Small intestine	No
SRX13443409	39873778	Single	0.0395354	NULL	RF	76.24	Cartilage	No
SRX19561490	52294874	Single	0.0527376	NULL	RF	79.21	PBMCs	No
SRX19561491	52771717	Single	0.0570993	NULL	RF	72.34	PBMCs	No
SRX19561492	47601647	Single	0.0664928	NULL	RF	73.67	PBMCs	No
SRX19561493	52765146	Single	0.0622552	NULL	RF	75.88	PBMCs	No
SRX19561494	55013115	Single	0.0590519	NULL	RF	77.28	PBMCs	No
SRX19561495	46144585	Single	0.0689881	NULL	RF	75.96	PBMCs	No
SRX19561496	59449863	Single	0.0542456	NULL	RF	73.06	PBMCs	No
SRX19802430	51580837	Paired	0.0306412	0.971327	RF	85.1	Cartilage	No
SRX19802431	41979977	Paired	0.0426843	0.961189	RF	85.74	Cartilage	No
SRX13459880	35464849	Paired	0.0261326	0.972468	RF	87.32	Cartilage	No
SRX16323970	50899572	Paired	0.09987	0.907335	RF	66.17	Whole blood	No
SRX13459881	30275963	Paired	0.0246333	0.97466	RF	88.94	Cartilage	No
SRX16323971	42334641	Paired	0.0950132	0.909126	RF	78.48	Whole blood	No
SRX13459882	31836719	Paired	0.0238663	0.975446	RF	87.36	Cartilage	No
SRX16323972	48277592	Paired	0.0917973	0.91092	RF	81.09	Whole blood	No
SRX13459883	40622093	Paired	0.0225937	0.976368	RF	90.71	Cartilage	No
SRX16323973	46241432	Paired	0.0817984	0.924896	RF	76.93	Whole blood	No
SRX13459884	32567881	Paired	0.0246241	0.973296	RF	90.29	Cartilage	No
SRX16323974	51384154	Paired	0.0975198	0.907023	RF	69.4	Whole blood	No
SRX13459885	35203691	Paired	0.0258061	0.972937	RF	89.36	Cartilage	No
SRX16323975	44534068	Paired	0.0761522	0.926387	RF	85.42	Whole blood	No
SRX13459886	36934757	Paired	0.0246475	0.974099	RF	87.94	Cartilage	No
ERX9275280	29917042	Paired	0.0251271	0.979035	RF	86.36	Muscle	No
ERX6202500	24507346	Paired	0.0212974	0.980274	RF	88.46	Uterus	No
SRX16323976	52512629	Paired	0.0893897	0.914801	RF	80.4	Whole blood	No
SRX13459887	32162669	Paired	0.024012	0.97459	RF	90.26	Cartilage	No
ERX9275281	32547726	Paired	0.0199298	0.980475	RF	88.14	Muscle	No
ERX9275240	26689248	Paired	0.022689	0.981392	RF	84.83	Muscle	No
ERX6202501	29249201	Paired	0.0211247	0.980591	RF	91.94	Uterus	No
SRX16323977	40798308	Paired	0.08426	0.917863	RF	88.48	Whole blood	No
SRX13459888	31594867	Paired	0.0233895	0.975186	RF	87.51	Cartilage	No
ERX9275282	28304773	Paired	0.0205886	0.981706	RF	86.58	Muscle	No
ERX9275241	23685460	Paired	0.0213773	0.981868	RF	85.52	Muscle	No
ERX9275200	30069126	Paired	0.0245624	0.980523	RF	86.36	Liver	No
ERX6202502	26166174	Paired	0.0245337	0.976937	RF	90.53	Uterus	No
SRX16323978	49130991	Paired	0.14415	0.90631	RF	70.72	Whole blood	No
SRX13459889	38536314	Paired	0.0228878	0.975685	RF	90.99	Cartilage	No
ERX9275283	32522591	Paired	0.0203847	0.981435	RF	88.31	Muscle	No
ERX9275242	29304235	Paired	0.0207805	0.982199	RF	82.92	Muscle	No
ERX9275201	25489092	Paired	0.0265918	0.978768	RF	85.84	Liver	No
ERX6202503	27341986	Paired	0.0228905	0.97826	RF	90.91	Uterus	No
SRX16323979	47545313	Paired	0.100777	0.901919	RF	83.48	Whole blood	No

SRX14418520	16580385	Paired	0.0386344	0.958976	RF	87.33	PBMCs	No
ERX9275284	35192197	Paired	0.0232251	0.97898	RF	87.03	Muscle	No
ERX9275243	68695902	Paired	0.0226923	0.980784	RF	85.66	Muscle	No
ERX9275202	32836717	Paired	0.0240946	0.979836	RF	84.77	Liver	No
ERX6202504	30547752	Paired	0.024179	0.977318	RF	89.56	Uterus	No
SRX14418521	39579537	Paired	0.0370937	0.959868	RF	88.23	PBMCs	No
ERX9275285	44250850	Paired	0.0235562	0.978459	RF	86.75	Muscle	No
ERX9275244	51866286	Paired	0.0196665	0.98378	RF	83.41	Muscle	No
ERX9275203	27663923	Paired	0.0283792	0.976651	RF	81.66	Liver	No
ERX6202505	33423377	Paired	0.0231095	0.977942	RF	90.68	Uterus	No
SRX14418522	23301810	Paired	0.0390538	0.957909	RF	87.11	PBMCs	No
ERX9275286	28961471	Paired	0.0297778	0.970715	RF	87.33	Muscle	No
ERX9275245	51960087	Paired	0.0222887	0.981792	RF	84.51	Muscle	No
ERX9275204	31311964	Paired	0.0246467	0.980107	RF	82.16	Liver	No
ERX6202506	28341570	Paired	0.0211821	0.979775	RF	92.39	Uterus	No
SRX11011033	39914750	Paired	0.0356217	0.963814	RF	71.7	Adipose	No
ERX9275287	37958923	Paired	0.0232995	0.979021	RF	86.32	Muscle	No
ERX9275246	45181459	Paired	0.0204649	0.98325	RF	85.6	Muscle	No
ERX9275205	26900218	Paired	0.0261735	0.979336	RF	85.4	Liver	No
SRX14418524	20082897	Paired	0.0396322	0.956704	RF	86.77	PBMCs	No
ERX9275288	41861712	Paired	0.0235214	0.97944	RF	86.63	Muscle	No
ERX9275247	40803752	Paired	0.0212577	0.981825	RF	85.31	Muscle	No
ERX9275206	28197755	Paired	0.0254888	0.979414	RF	85.04	Liver	No
SRX14418525	14979417	Paired	0.0407394	0.954491	RF	86.26	PBMCs	No
ERX9275289	29755752	Paired	0.0352467	0.972196	RF	84.38	Muscle	No
ERX9275248	47685309	Paired	0.0210544	0.983468	RF	85.56	Muscle	No
ERX9275207	33890826	Paired	0.0256085	0.979139	RF	85.68	Liver	No
SRX14418526	22682572	Paired	0.0390034	0.957502	RF	86.92	PBMCs	No
ERX9275249	46561074	Paired	0.0202341	0.982915	RF	86.2	Muscle	No
ERX9275208	29045088	Paired	0.0244881	0.980776	RF	85.5	Liver	No
SRX14418527	25824493	Paired	0.0410558	0.956415	RF	87.12	PBMCs	No
ERX9275209	32408538	Paired	0.0257572	0.980204	RF	84.45	Liver	No
SRX19452660	49752778	Paired	0.0246003	0.975347	RF	85.93	Artery	No
SRX14418528	17736229	Paired	0.0413302	0.955369	RF	87.67	PBMCs	No
SRX14418529	14753189	Paired	0.0423164	0.95372	RF	86.32	PBMCs	No
SRX19452663	41137043	Paired	0.0280812	0.971733	RF	85.55	Artery	No
SRX11216710	41564816	Paired	0.0776485	0.919463	RF	84.61	Brain	No
SRX11216711	49324867	Paired	0.122247	0.875677	RF	75.35	Adipose	No
SRX11216712	45761600	Paired	0.093144	0.903901	RF	85.14	Heart	No
SRX11216714	43048587	Paired	0.13751	0.857274	RF	82.72	Brain	No
SRX11216715	52178441	Paired	0.0827916	0.915699	RF	79.52	Liver	No
SRX11216716	44081220	Paired	0.10735	0.890521	RF	68.33	Lung	No
ERX2987500	20614128	Paired	0.023384	0.976947	RF	93.18	Brain	No
SRX11216717	50867380	Paired	0.0999675	0.900908	RF	73.86	Muscle	No
ERX2987501	23764765	Paired	0.0225936	0.977788	RF	93.08	Brain	No
ERX2987502	23964063	Paired	0.0245379	0.975589	RF	93.22	Brain	No
ERX3981436	29542575	Paired	0.0695639	0.878071	RF	76.58	Heart	No
ERX2987503	21090687	Paired	0.0248026	0.975297	RF	93.4	Brain	No
ERX3981437	28613185	Paired	0.069405	0.870695	RF	77.21	Heart	No
ERX2987504	31408353	Paired	0.0221783	0.978074	RF	93.41	Brain	No
ERX3981438	36649820	Paired	0.0689591	0.879104	RF	76.29	Heart	No
ERX2987505	15385113	Paired	0.0475614	0.952656	RF	93.5	Brain	No
ERX3981439	31167301	Paired	0.0749627	0.864929	RF	76.32	Heart	No
ERX2987506	26877905	Paired	0.02553	0.97472	RF	93.02	Brain	No
ERX2987507	56184868	Paired	0.0209061	0.979336	RF	89.5	Brain	No
SRX14806390	21500695	Paired	0.17522	0.822263	RF	87.5	Large intestine	No
ERX2987508	22062099	Paired	0.0237897	0.976374	RF	92.96	Brain	No
SRX14806391	30891763	Paired	0.12855	0.871449	RF	90.65	Liver	No

ERX2987509	24373725	Paired	0.0242498	0.975846	RF	93.35	Brain	No
SRX14806392	31806521	Paired	0.127791	0.872768	RF	88.46	Small intestine	No
SRX14806393	26850442	Paired	0.168189	0.829997	RF	86.81	Large intestine	No
SRX14806394	27589313	Paired	0.134152	0.864392	RF	88.83	Large intestine	No
SRX14806395	20576098	Paired	0.134062	0.865215	RF	90.09	Liver	No
SRX16324000	39451390	Paired	0.0871417	0.915137	RF	88.5	Whole blood	No
SRX14806396	29346068	Paired	0.126757	0.872286	RF	88.57	Liver	No
SRX14806397	27942280	Paired	0.152775	0.846519	RF	88.67	Liver	No
SRX14806398	35833359	Paired	0.122522	0.877525	RF	90.51	Liver	No
SRX14806399	24465524	Paired	0.129973	0.870132	RF	89.32	Liver	No
SRX8361280	24150925	Paired	0.0314534	0.957332	RF	68.5	Fibroblast	No
SRX8361281	24536269	Paired	0.0325035	0.956344	RF	70.88	Fibroblast	No
SRX8361282	24697298	Paired	0.034852	0.952186	RF	68.42	Fibroblast	No
SRX8361283	24543103	Paired	0.0339288	0.957121	RF	71.27	Fibroblast	No
SRX8361284	24606043	Paired	0.02658	0.966289	RF	68.87	Fibroblast	No
ERX4133020	24020239	Paired	0.0258468	0.974694	RF	92.94	Brain	No
SRX8361285	24347815	Paired	0.0255025	0.967205	RF	75.77	Fibroblast	No
ERX4133021	21203439	Paired	0.0265087	0.974165	RF	93.6	Brain	No
SRX8361286	24453159	Paired	0.0258512	0.964065	RF	91.04	Fibroblast	No
ERX8087580	53705667	Paired	0.0152188	0.985529	RF	93.81	Ovary	No
ERX4133022	22030492	Paired	0.0258649	0.974833	RF	93.97	Brain	No
SRX8361287	24509828	Paired	0.0244361	0.972606	RF	91.31	Fibroblast	No
ERX8087581	56469199	Paired	0.0153378	0.985521	RF	93.65	Ovary	No
ERX4133023	23368216	Paired	0.0290389	0.971449	RF	93.32	Brain	No
ERX8087582	61177412	Paired	0.0151577	0.985683	RF	92.83	Ovary	No
ERX4133024	21325847	Paired	0.0270939	0.973431	RF	93.43	Brain	No
SRX16723081	4745491	Single	0.0376247	NULL	RF	94.45	Ovary	No
ERX8087583	54925739	Paired	0.0156472	0.985234	RF	92.93	Ovary	No
ERX9570707	24828323	Paired	0.0571774	0.943076	RF	93.37	Ovary	No
ERX9570708	23623207	Paired	0.049875	0.950807	RF	93.72	Ovary	No
SRX17937680	32737240	Paired	0.0223552	0.978936	RF	88.15	Kidney	No
ERX9570709	24154899	Paired	0.0460231	0.956201	RF	93.14	Ovary	No
SRX17937681	39847308	Paired	0.0234501	0.978006	RF	88.08	Kidney	No
SRX17937682	42523106	Paired	0.0312921	0.971509	RF	85.07	Kidney	No
SRX17937683	40974300	Paired	0.0263123	0.975134	RF	86.16	Kidney	No
SRX17937684	38163298	Paired	0.0232334	0.977773	RF	84.44	Kidney	No
SRX17937685	35953872	Paired	0.0236434	0.977759	RF	86.52	Kidney	No
SRX17937686	45924918	Paired	0.0318454	0.970459	RF	82.27	Kidney	No
SRX17937687	42452801	Paired	0.0260631	0.97528	RF	79.16	Kidney	No
SRX17389900	19581725	Paired	0.0438683	0.954639	RF	84.21	Spleen	No
SRX17389901	52565363	Paired	0.0374418	0.962205	RF	85.57	Spleen	No
SRX17389902	50511183	Paired	0.0417344	0.956915	RF	84.88	Spleen	No
SRX17389903	52039165	Paired	0.0504562	0.95128	RF	79.85	Spleen	No
SRX18363905	32872889	Paired	0.0199498	0.978359	RF	87.13	Liver	No
SRX17389904	52165239	Paired	0.0516976	0.953411	RF	82.23	Spleen	No
SRX18363906	37629851	Paired	0.0179075	0.981043	RF	87.86	Liver	No
SRX17389905	50638583	Paired	0.0478777	0.956152	RF	82.57	Spleen	No
SRX18363907	34311172	Paired	0.0194003	0.979578	RF	87.04	Liver	No
SRX17389906	53342797	Paired	0.0395607	0.958921	RF	85.64	Spleen	No
SRX18363908	32706921	Paired	0.0172179	0.981209	RF	87.27	Liver	No
SRX17389907	53416879	Paired	0.0396602	0.959124	RF	83.69	Spleen	No
SRX18363909	25354722	Paired	0.0205762	0.975687	RF	84.41	Liver	No
SRX17389908	50200864	Paired	0.0457938	0.952957	RF	82.77	Spleen	No
SRX17389909	51034864	Paired	0.0404403	0.958326	RF	84.88	Spleen	No
ERX9900013	30083022	Paired	0.036941	0.960255	RF	85.89	Brain	No
ERX9570710	23019505	Paired	0.0427874	0.957993	RF	93.39	Ovary	No
ERX9570711	23862219	Paired	0.0532696	0.947062	RF	93.48	Ovary	No
ERX9570712	22484253	Paired	0.0420177	0.959078	RF	93.72	Ovary	No

ERX9570693	21823773	Paired	0.0457024	0.95656	RF	92.54	Ovary	No
ERX9570694	24020043	Paired	0.0414754	0.9596	RF	93.27	Ovary	No
ERX9570695	23575073	Paired	0.0549136	0.945334	RF	93.38	Ovary	No
ERX9570696	20914911	Paired	0.0572733	0.943085	RF	93.51	Ovary	No
ERX9570697	25414072	Paired	0.0389761	0.962765	RF	93.1	Ovary	No
ERX9570698	22441351	Paired	0.045146	0.95526	RF	93.39	Ovary	No
ERX9570699	23376320	Paired	0.0417608	0.959734	RF	93.69	Ovary	No
SRX17937679	34218704	Paired	0.0225741	0.978454	RF	87.77	Kidney	No
SRX18363910	35357717	Paired	0.0172814	0.981556	RF	87.86	Liver	No
SRX18363911	29163775	Paired	0.0185466	0.980006	RF	87.14	Liver	No
SRX17389910	52576968	Paired	0.0492813	0.952123	RF	82.11	Spleen	No
SRX18363912	32298478	Paired	0.0205509	0.97755	RF	87.01	Liver	No
SRX17389911	50488621	Paired	0.0411922	0.960627	RF	85.11	Spleen	No
SRX18363913	34502703	Paired	0.0180259	0.980467	RF	87.32	Liver	No
SRX17389912	52897917	Paired	0.0411302	0.961772	RF	83.76	Spleen	No
SRX18363914	33809853	Paired	0.0189255	0.980235	RF	87.89	Liver	No
SRX17389913	50758441	Paired	0.0437065	0.955332	RF	83.07	Spleen	No
SRX18363915	31082579	Paired	0.0176139	0.980384	RF	86.59	Liver	No
SRX18363916	31905410	Paired	0.0204855	0.977079	RF	86.91	Liver	No
SRX17389899	52938558	Paired	0.0501322	0.945486	RF	80.94	Spleen	No
ERX9570700	20968803	Paired	0.0502448	0.950131	RF	93.59	Ovary	No
ERX9570701	24548039	Paired	0.051539	0.950285	RF	92.51	Ovary	No
ERX9570702	22986161	Paired	0.037921	0.96275	RF	93.19	Ovary	No
ERX9570703	23311540	Paired	0.0523469	0.948446	RF	93.35	Ovary	No
ERX9570704	22712730	Paired	0.0428915	0.958318	RF	93.66	Ovary	No
ERX9570705	20712122	Paired	0.0561559	0.946764	RF	92.8	Ovary	No
ERX9570706	23464710	Paired	0.0448079	0.956059	RF	93.44	Ovary	No
SRX13838667	24358532	Paired	0.0217185	0.979092	RF	83.85	Large intestine	No
SRX13838668	25705005	Paired	0.0193884	0.981469	RF	85.72	Large intestine	No
SRX14228400	28208540	Paired	0.081155	0.906925	RF	89.85	Heart	No
SRX13838669	25174277	Paired	0.0198192	0.98083	RF	85.69	Large intestine	No
SRX14228401	27334818	Paired	0.107412	0.879576	RF	87.6	Heart	No
ERX10011080	29346682	Paired	0.104373	0.894183	RF	89.99	Large intestine	No
SRX14228402	25254830	Paired	0.081676	0.910771	RF	89.79	Heart	No
ERX10011081	30110200	Paired	0.0892227	0.909679	RF	92.54	Large intestine	No
SRX14228403	23704106	Paired	0.0982117	0.886765	RF	87.24	Heart	No
ERX10011082	37356144	Paired	0.101996	0.896666	RF	91.3	Large intestine	No
SRX14228404	25604266	Paired	0.120648	0.85762	RF	86.3	Heart	No
ERX10011083	33731331	Paired	0.0898796	0.908992	RF	92.33	Large intestine	No
SRX14228405	25252345	Paired	0.107203	0.878199	RF	87.74	Heart	No
ERX10011084	30814519	Paired	0.100601	0.898102	RF	92.25	Large intestine	No
SRX14228406	16477672	Paired	0.072112	0.930078	RF	88.75	Heart	No
ERX10011085	30732469	Paired	0.0865863	0.912138	RF	90.77	Large intestine	No
SRX14228407	20612602	Paired	0.0532465	0.948739	RF	88.69	Heart	No
ERX10011086	31499261	Paired	0.0843847	0.914228	RF	90.4	Large intestine	No
SRX14228408	12343868	Paired	0.0496407	0.952193	RF	88.59	Heart	No
ERX10011087	33911733	Paired	0.106144	0.892595	RF	91.78	Large intestine	No
ERX10011088	31678183	Paired	0.0970405	0.901551	RF	90.99	Large intestine	No
ERX10011089	35094094	Paired	0.0984272	0.900506	RF	92.25	Large intestine	No
SRX7416750	26373134	Paired	0.0269623	0.977664	RF	86.58	Liver	No
SRX7416751	25257472	Paired	0.0250703	0.979007	RF	88.26	Liver	No
SRX7416752	23792183	Paired	0.0208613	0.98149	RF	87.65	Liver	No
SRX7416753	22042259	Paired	0.0247279	0.979456	RF	82.57	Liver	No
SRX7416754	18761289	Paired	0.0217137	0.982485	RF	81.98	Liver	No
SRX16728310	22799316	Paired	0.0208671	0.975529	RF	84.68	Hair	No
SRX16728311	28915538	Paired	0.0251156	0.970698	RF	89.62	Hair	No
SRX14852351	25258502	Paired	0.0271047	0.973385	RF	88.59	IPEC-J2	No
SRX16728312	31157189	Paired	0.0359236	0.957239	RF	87.47	Hair	No

SRX14852352	25317165	Paired	0.0268011	0.973597	RF	88.07	IPEC-J2	No
SRX16728313	32465995	Paired	0.0245257	0.970487	RF	88.31	Hair	No
SRX14852353	24686492	Paired	0.0274984	0.972873	RF	88.46	IPEC-J2	No
SRX16728314	33874385	Paired	0.0268597	0.97006	RF	89.04	Hair	No
SRX14852354	25097448	Paired	0.0270234	0.97346	RF	88.35	IPEC-J2	No
SRX16728315	34963438	Paired	0.0253529	0.971858	RF	87.89	Hair	No
SRX14852355	23911261	Paired	0.0272598	0.973105	RF	89.12	IPEC-J2	No
SRX16728316	33599093	Paired	0.02743	0.971579	RF	84.68	Hair	No
SRX14852356	24784400	Paired	0.0272519	0.973113	RF	89.25	IPEC-J2	No
SRX16728317	30610941	Paired	0.025786	0.973069	RF	83.15	Hair	No
SRX16728318	26020991	Paired	0.0254387	0.974891	RF	83.88	Hair	No
SRX16728319	39776477	Paired	0.037568	0.961114	RF	84.54	Hair	No
SRX9152410	17591818	Paired	0.860657	0.16487	FR	72	Pancreas	No
SRX9152411	14764709	Paired	0.857991	0.17808	FR	69.78	Pancreas	No
SRX9152412	18376634	Paired	0.862369	0.166412	FR	73.86	Pancreas	No
SRX5581731	31527705	Paired	0.0233552	0.977959	RF	92.17	3D4/31	No
SRX9152413	17313764	Paired	0.864936	0.165845	FR	71.38	Pancreas	No
SRX5581732	35024378	Paired	0.0225701	0.978608	RF	92.7	3D4/31	No
SRX9152414	16029501	Paired	0.862422	0.163493	FR	72.41	Pancreas	No
SRX5581733	31250947	Paired	0.0232781	0.97834	RF	92.66	3D4/31	No
SRX12162940	23702397	Paired	0.039255	0.961823	RF	90.28	Adipose	No
SRX9152415	16935920	Paired	0.872072	0.161415	FR	62.38	Pancreas	No
SRX5581734	36847073	Paired	0.0225995	0.978133	RF	92.26	3D4/31	No
SRX9152416	17231533	Paired	0.871534	0.160722	FR	70.25	Pancreas	No
SRX5581735	29000038	Paired	0.02186	0.979331	RF	92.45	3D4/31	No
SRX5581736	33653220	Paired	0.0238709	0.978435	RF	93.36	3D4/31	No
SRX9152418	14932211	Paired	0.870837	0.153506	FR	69.2	Pancreas	No
SRX5581737	38248373	Paired	0.0232983	0.977427	RF	91.59	3D4/31	No
SRX9152419	13043564	Paired	0.880963	0.149838	FR	60.71	Pancreas	No
SRX5581738	36185492	Paired	0.024322	0.97775	RF	92.01	3D4/31	No
SRX5581739	34507074	Paired	0.0247948	0.977465	RF	90.13	3D4/31	No
ERX7301061	100918224	Paired	0.0261228	0.97682	RF	89.79	Liver	No
ERX7301062	98967744	Paired	0.0279462	0.971425	RF	93.02	Brain	No
ERX7301063	97407236	Paired	0.0282709	0.970309	RF	93.11	Brain	No
ERX7301066	163253742	Paired	0.0286378	0.97083	RF	92.06	Brain	No
ERX7301067	132437628	Paired	0.0266957	0.973103	RF	92.26	Brain	No
ERX7301068	119798765	Paired	0.027137	0.972328	RF	92.48	Brain	No
ERX7301069	124520886	Paired	0.022952	0.976893	RF	91.3	Brain	No
SRX19271405	35005270	Paired	0.0311433	0.967226	RF	87.8	3D4/31	No
SRX19271406	37407859	Paired	0.0276812	0.969946	RF	88.88	3D4/31	No
SRX18280335	21838001	Paired	0.977813	0.0235838	FR	73.73	Small intestine	No
SRX18280336	29275631	Paired	0.979974	0.032197	FR	69.68	Small intestine	No
SRX18280337	24033004	Paired	0.978763	0.0232141	FR	76.66	Small intestine	No
SRX14127120	33672978	Paired	0.943911	0.0524622	FR	85.57	PBMCs	No
SRX18280338	30001075	Paired	0.975581	0.0313154	FR	73.43	Small intestine	No
SRX14127121	36102974	Paired	0.948482	0.0492487	FR	87.48	PBMCs	No
SRX18280339	29878446	Paired	0.977871	0.0336127	FR	73.31	Small intestine	No
SRX14127122	41994444	Paired	0.945582	0.0523836	FR	87.29	PBMCs	No
SRX14127123	37275356	Paired	0.947543	0.0497769	FR	87.4	PBMCs	No
SRX14127124	40704910	Paired	0.947025	0.0508192	FR	87.22	PBMCs	No
SRX14127125	32744406	Paired	0.946795	0.0508306	FR	86.89	PBMCs	No
SRX14127126	38410123	Paired	0.947847	0.049969	FR	86.76	PBMCs	No
SRX13144617	23866175	Paired	0.101105	0.898726	RF	83.9	Small intestine	No
SRX14127127	38120357	Paired	0.945367	0.0510666	FR	88.01	PBMCs	No
SRX13144618	21461384	Paired	0.118989	0.880456	RF	83.87	Small intestine	No
SRX14127128	38999789	Paired	0.946359	0.0506105	FR	86.26	PBMCs	No
SRX13144619	21984652	Paired	0.144673	0.854744	RF	82.35	Small intestine	No
SRX18968064	20968661	Paired	0.0367431	0.964136	RF	89.67	Small intestine	No

SRX18968065	23327917	Paired	0.0341423	0.966748	RF	89.75	Small intestine	No
SRX18968066	20995284	Paired	0.0273973	0.973795	RF	89.85	Small intestine	No
SRX18968067	22975344	Paired	0.0219796	0.979097	RF	89.82	Small intestine	No
SRX18968068	20484820	Paired	0.0227466	0.978475	RF	89.89	Small intestine	No
SRX18968069	20994141	Paired	0.0230495	0.978245	RF	90.01	Small intestine	No
SRX17708370	30542851	Paired	0.980815	0.0221295	FR	80.63	PBMCs	No
SRX17708371	20527310	Paired	0.979921	0.0217738	FR	81.57	PBMCs	No
SRX17708372	16266545	Paired	0.978474	0.0228105	FR	80.9	PBMCs	No
SRX17708373	12281930	Paired	0.974597	0.0282518	FR	82.44	PBMCs	No
SRX17708374	22791494	Paired	0.979392	0.0217483	FR	83.61	PBMCs	No
SRX17708375	27363174	Paired	0.979135	0.0219627	FR	81.85	PBMCs	No
SRX17708376	30059600	Paired	0.978834	0.023766	FR	83.03	PBMCs	No
SRX17708377	21500973	Paired	0.978193	0.0227448	FR	82.72	PBMCs	No
SRX17708378	20709041	Paired	0.974455	0.029703	FR	74.13	T cell	No
SRX14716177	23662551	Paired	0.041266	0.959201	RF	88.05	Macrophage	No
SRX17708379	25129380	Paired	0.97956	0.0225943	FR	78.89	T cell	No
SRX14716178	23295904	Paired	0.0369139	0.963577	RF	88.09	Macrophage	No
SRX14716179	23333568	Paired	0.0317326	0.968835	RF	87.73	Macrophage	No
SRX18427690	69016830	Paired	0.0247508	0.973526	RF	93.66	Ovary	No
SRX18427691	64784649	Paired	0.0249783	0.97283	RF	93.48	Ovary	No
SRX18427692	89858837	Paired	0.0249016	0.972843	RF	93.57	Ovary	No
SRX6430470	79703463	Paired	0.0280152	0.971453	RF	85.13	PK15	No
SRX10567279	47935910	Paired	0.0264156	0.968065	RF	68.24	Liver	No
SRX13276550	24198580	Paired	0.0188945	0.982229	RF	84.44	Large intestine	No
SRX13276551	17353995	Paired	0.026156	0.97423	RF	74.41	Brain	No
SRX13276510	18507060	Paired	0.0253185	0.97506	RF	82.98	Brain	No
SRX13276552	16809599	Paired	0.0209666	0.979987	RF	84.2	Large intestine	No
SRX13276511	23888832	Paired	0.0236898	0.976812	RF	85.76	Brain	No
SRX13276553	24991457	Paired	0.0187775	0.982433	RF	82.92	Large intestine	No
SRX13276512	15617439	Paired	0.0262268	0.97426	RF	84.92	Brain	No
SRX13276554	23858479	Paired	0.0188542	0.982105	RF	82.12	Large intestine	No
SRX13276513	16044994	Paired	0.0282076	0.972225	RF	83.3	Brain	No
SRX15447816	40336493	Paired	0.117287	0.881967	RF	60.26	Small intestine	No
SRX13276555	24528450	Paired	0.0193931	0.981698	RF	84.63	Large intestine	No
SRX13276514	17459667	Paired	0.0255408	0.97486	RF	83.39	Brain	No
SRX15447817	43580679	Paired	0.114899	0.885193	RF	70.25	Small intestine	No
SRX13276556	16567526	Paired	0.0281844	0.97244	RF	80.8	Brain	No
SRX13276515	24819954	Paired	0.0270439	0.973399	RF	83.12	Brain	No
SRX15447818	42460634	Paired	0.140541	0.859594	RF	77.04	Small intestine	No
SRX13276557	17487133	Paired	0.0231173	0.97748	RF	81.96	Brain	No
SRX13276516	20105718	Paired	0.0195765	0.981119	RF	83.01	Large intestine	No
SRX15447819	40789752	Paired	0.137665	0.862572	RF	72.21	Small intestine	No
SRX13328060	30214439	Paired	0.0422484	0.956974	RF	87.26	Macrophage	No
SRX13276558	19971404	Paired	0.0250107	0.975522	RF	81.25	Brain	No
SRX13276517	22578613	Paired	0.0217826	0.978887	RF	83.7	Large intestine	No
SRX13328061	34439071	Paired	0.0426549	0.956496	RF	86.38	Macrophage	No
SRX13276559	24655190	Paired	0.0228304	0.977562	RF	86.4	Brain	No
SRX13276518	18718707	Paired	0.0220034	0.978537	RF	82.74	Brain	No
SRX16121539	15507301	Paired	0.0314431	0.96495	RF	78.33	IPEC-J2	No
SRX13328062	34687649	Paired	0.0363504	0.963375	RF	88.47	Macrophage	No
SRX13276519	18108138	Paired	0.0212206	0.979522	RF	84.01	Large intestine	No
SRX13328063	32244202	Paired	0.0370507	0.962171	RF	88.32	Macrophage	No
SRX13328064	31187690	Paired	0.0373867	0.962429	RF	88.76	Macrophage	No
SRX19771840	17699206	Paired	0.0182858	0.982442	RF	84.62	IPEC-J2	No
SRX10971440	34352563	Paired	0.987506	0.0122744	FR	75.6	Testis	No
SRX19771841	20528695	Paired	0.0205021	0.980322	RF	85.1	IPEC-J2	No
SRX10971441	46486729	Paired	0.987741	0.0120495	FR	76.56	Testis	No
SRX19771842	17631998	Paired	0.0181108	0.982749	RF	86.66	IPEC-J2	No

SRX19771843	20224235	Paired	0.0219908	0.97985	RF	84.69	IPEC-J2	No
SRX19771844	20702233	Paired	0.0190783	0.981758	RF	85	IPEC-J2	No
SRX17708410	17033900	Paired	0.977532	0.0232403	FR	80.86	PBMCs	No
SRX17708411	21365068	Paired	0.980869	0.0207246	FR	81.1	PBMCs	No
SRX17708412	16893463	Paired	0.977522	0.0239038	FR	82.46	PBMCs	No
SRX17708413	23819524	Paired	0.981218	0.0197125	FR	81.9	T cell	No
SRX17708414	24288189	Paired	0.979269	0.0212219	FR	83.52	T cell	No
SRX16728280	31858106	Paired	0.0279052	0.970524	RF	83.23	Hair	No
SRX17708415	26240978	Paired	0.979452	0.0209904	FR	78.53	T cell	No
SRX16728281	26216607	Paired	0.0276689	0.97265	RF	82.37	Hair	No
SRX17708416	31209181	Paired	0.978898	0.0221422	FR	83.25	T cell	No
SRX16728282	33377059	Paired	0.0415516	0.956184	RF	87.61	Hair	No
SRX17708417	15343169	Paired	0.975475	0.0270969	FR	81.65	PBMCs	No
SRX16728283	27350894	Paired	0.0303375	0.968744	RF	87.73	Hair	No
SRX17708418	24623832	Paired	0.980941	0.0195441	FR	80.72	T cell	No
SRX16728284	29078355	Paired	0.0345453	0.964472	RF	86.97	Hair	No
SRX17708419	17041175	Paired	0.979047	0.0216465	FR	79.89	T cell	No
SRX16728285	27369022	Paired	0.0320674	0.965401	RF	87.59	Hair	No
SRX16728286	24208508	Paired	0.0329404	0.965529	RF	81.32	Hair	No
SRX16728287	28659861	Paired	0.0361325	0.962006	RF	87.53	Hair	No
SRX16728288	24666866	Paired	0.0281404	0.969949	RF	87.87	Hair	No
SRX16728289	34025155	Paired	0.0248749	0.972431	RF	87.29	Hair	No
SRX14985601	43768328	Paired	0.962631	0.0336077	FR	83.85	Brain	No
SRX14985602	45043195	Paired	0.962723	0.0335318	FR	85.27	Brain	No
SRX14985603	41206079	Paired	0.962178	0.0327518	FR	82.03	Brain	No
SRX14985604	52020207	Paired	0.961752	0.0327437	FR	83.14	Brain	No
SRX14985605	44016839	Paired	0.957886	0.0366634	FR	83.96	Brain	No
SRX10327890	9338724	Paired	0.0269447	0.975389	RF	91.7	Ovary	No
SRX14985606	50607403	Paired	0.961594	0.033052	FR	82.95	Brain	No
SRX10327891	9714691	Paired	0.0264208	0.975978	RF	91.71	Ovary	No
SRX14985607	36970815	Paired	0.960728	0.0340061	FR	82.21	Brain	No
SRX14985608	46734088	Paired	0.967213	0.0305279	FR	83.62	Brain	No
SRX14985609	43661817	Paired	0.95991	0.0350395	FR	82.53	Brain	No
SRX7521362	31117277	Single	0.0145803	NULL	RF	93.62	Artery	No
SRX7521363	35512382	Single	0.0143732	NULL	RF	93.19	Artery	No
SRX7521364	27358220	Single	0.0146819	NULL	RF	93.85	Artery	No
SRX7521365	32676056	Single	0.0142962	NULL	RF	94.03	Artery	No
SRX7521366	37995489	Single	0.0146039	NULL	RF	93.74	Artery	No
SRX7521367	41304291	Single	0.0137641	NULL	RF	94.08	Artery	No
SRX7521368	31678450	Single	0.0137928	NULL	RF	93.39	Artery	No
SRX7521369	31300926	Single	0.0145407	NULL	RF	94.59	Artery	No
SRX13838670	23990391	Paired	0.0205239	0.980238	RF	85.92	Large intestine	No
SRX13838671	20356459	Paired	0.01949	0.981279	RF	86.13	Large intestine	No
ERX10011090	36059900	Paired	0.0886287	0.910179	RF	91.85	Large intestine	No
ERX10011091	32828855	Paired	0.0833619	0.915891	RF	92.69	Large intestine	No
SRX19520218	53890704	Paired	0.0277024	0.972654	RF	85.09	Ovary	No
ERX10011092	34080281	Paired	0.080284	0.918247	RF	91.38	Large intestine	No
SRX19520219	59120959	Paired	0.0282258	0.972003	RF	81.59	Ovary	No
ERX10011093	34401280	Paired	0.092773	0.906115	RF	91.27	Large intestine	No
ERX10011094	31244493	Paired	0.0921113	0.90651	RF	90.64	Large intestine	No
ERX10011095	34010822	Paired	0.084751	0.914206	RF	92.14	Large intestine	No
ERX10011096	29649438	Paired	0.0975312	0.901451	RF	91.32	Large intestine	No
ERX10011097	29870636	Paired	0.0857599	0.912968	RF	91.58	Large intestine	No
ERX10011098	31898628	Paired	0.0971626	0.901563	RF	91.54	Large intestine	No
ERX10011099	29100986	Paired	0.0872094	0.91197	RF	91.58	Large intestine	No
SRX16728320	31861165	Paired	0.0288459	0.970232	RF	83.52	Hair	No
SRX18548237	46150508	Paired	0.0753591	0.926132	RF	86.3	Placenta	No
SRX18548238	49870327	Paired	0.0748803	0.926877	RF	87.88	Placenta	No

SRX18548239	47265668	Paired	0.0833638	0.918042	RF	87.96	Placenta	No
SRX10954800	26376486	Single	0.0212778	NULL	RF	92.93	Heart	No
SRX9152420	13930747	Paired	0.871047	0.165008	FR	68.24	Pancreas	No
SRX10954801	28041798	Single	0.0212338	NULL	RF	92.84	Heart	No
SRX9152421	13236348	Paired	0.862865	0.160572	FR	70.34	Pancreas	No
SRX5581740	30474051	Paired	0.0236064	0.977358	RF	91.62	3D4/31	No
SRX10954802	29262467	Single	0.0265748	NULL	RF	92.61	Heart	No
SRX5581741	34757431	Paired	0.0252396	0.976682	RF	91.6	3D4/31	No
SRX10954803	29186816	Single	0.0222198	NULL	RF	92.79	Heart	No
SRX5581742	33042979	Paired	0.0245351	0.976956	RF	91.65	3D4/31	No
SRX10954804	19229562	Single	0.0318647	NULL	RF	92.57	Heart	No
SRX10954805	27748942	Single	0.0221601	NULL	RF	91.41	Heart	No
ERX7301075	82968819	Paired	0.0231771	0.976667	RF	91.71	Muscle	No
ERX7301077	116657878	Paired	0.0245267	0.975316	RF	92.25	Muscle	No
ERX7301079	104296030	Paired	0.0258914	0.970993	RF	91.64	Placenta	No
SRX18280340	26076289	Paired	0.980951	0.0211233	FR	73.98	Small intestine	No
SRX18280341	25982391	Paired	0.976541	0.0253867	FR	72.8	Small intestine	No
SRX18280342	24380938	Paired	0.97717	0.0256468	FR	73.34	Small intestine	No
SRX18280343	23311614	Paired	0.974319	0.0301112	FR	73.24	Small intestine	No
SRX18280344	31794081	Paired	0.980972	0.0204078	FR	76.58	Small intestine	No
SRX18280345	22206498	Paired	0.97998	0.0220009	FR	76.92	Small intestine	No
SRX18280346	22498991	Paired	0.979292	0.025546	FR	75.67	Small intestine	No
SRX16999563	46496517	Paired	0.123664	0.879985	RF	85.57	Muscle	No
SRX13144620	20532044	Paired	0.143461	0.856118	RF	83.24	Small intestine	No
SRX16999564	63041191	Paired	0.122617	0.881141	RF	83.14	Muscle	No
SRX13144621	21222339	Paired	0.0947804	0.905036	RF	83.74	Small intestine	No
SRX14338631	28185094	Paired	0.128292	0.863079	RF	71.91	Artery	No
SRX13144622	20324266	Paired	0.156221	0.842415	RF	82.33	Small intestine	No
SRX13144623	21731426	Paired	0.148228	0.850065	RF	84.69	Small intestine	No
SRX13144624	21651350	Paired	0.15009	0.848684	RF	82.79	Small intestine	No
SRX13144625	22153390	Paired	0.127701	0.871218	RF	84.68	Small intestine	No
SRX17708380	21825907	Paired	0.979409	0.0235493	FR	81.63	T cell	No
SRX17708381	16231209	Paired	0.974182	0.0297508	FR	79.98	T cell	No
SRX14716180	24755173	Paired	0.0267981	0.973735	RF	87.67	Macrophage	No
SRX17708382	24972959	Paired	0.975621	0.0252494	FR	81.21	T cell	No
SRX14716181	23642061	Paired	0.0326636	0.967943	RF	87.92	Macrophage	No
SRX17708383	24054058	Paired	0.97646	0.0248488	FR	83.61	T cell	No
SRX14716182	23801409	Paired	0.0286948	0.971857	RF	87.65	Macrophage	No
SRX17708384	12624197	Paired	0.974524	0.0306584	FR	82.11	PBMCs	No
SRX17708385	20815439	Paired	0.975972	0.0250612	FR	84.46	T cell	No
SRX17708386	18760146	Paired	0.976804	0.0259491	FR	82.11	T cell	No
SRX17708387	15087706	Paired	0.979787	0.0206157	FR	75.7	PBMCs	No
SRX17708388	14317788	Paired	0.979289	0.0216335	FR	79.79	PBMCs	No
SRX17708389	17293442	Paired	0.98	0.0220335	FR	81.43	PBMCs	No
SRX10567280	47606339	Paired	0.0215447	0.974246	RF	71.22	Liver	No
SRX17672948	21939997	Paired	0.0154977	0.985105	RF	88.94	Adipose	No
SRX10567281	47179289	Paired	0.0199719	0.975293	RF	69.03	Liver	No
SRX17672949	21909622	Paired	0.0159835	0.984732	RF	88.85	Adipose	No
SRX10567282	48068502	Paired	0.0207411	0.97714	RF	78.5	Liver	No
SRX10567283	49226394	Paired	0.0214917	0.977106	RF	84.27	Liver	No
SRX10567284	47204459	Paired	0.0243902	0.971252	RF	70.09	Liver	No
SRX10567285	45705910	Paired	0.0294834	0.964518	RF	62.44	Muscle	No
SRX15447820	42425077	Paired	0.14559	0.854718	RF	77.93	Small intestine	No
SRX16121540	13788788	Paired	0.0311681	0.966594	RF	79.06	IPEC-J2	No
SRX13276520	22747951	Paired	0.0200944	0.980446	RF	79.44	Large intestine	No
SRX16121541	19625701	Paired	0.0291426	0.968992	RF	78.07	IPEC-J2	No
SRX13276521	20830991	Paired	0.0236283	0.976848	RF	79.86	Large intestine	No
SRX16121542	17768044	Paired	0.0293853	0.968453	RF	76.53	IPEC-J2	No

SRX13276522	22666257	Paired	0.0231517	0.977427	RF	79.1	Large intestine	No
SRX16121543	17142750	Paired	0.0293799	0.967514	RF	76.39	IPEC-J2	No
SRX13276523	21832622	Paired	0.0210732	0.979496	RF	83.06	Large intestine	No
SRX16121544	16167064	Paired	0.0259367	0.973886	RF	79.31	IPEC-J2	No
SRX13276524	16340419	Paired	0.0216229	0.978912	RF	81.44	Large intestine	No
SRX13276525	24722689	Paired	0.0203793	0.980181	RF	82.71	Large intestine	No
SRX13276526	24734263	Paired	0.0236044	0.977017	RF	84.23	Brain	No
SRX13276527	24684846	Paired	0.0243558	0.976166	RF	84.29	Brain	No
SRX13276528	19470065	Paired	0.0256513	0.974608	RF	78.71	Brain	No
SRX12569189	40590345	Paired	0.0341927	0.967482	RF	93.11	Small intestine	No
SRX13276529	19838148	Paired	0.0269972	0.973521	RF	82.55	Brain	No
SRX17287881	52252367	Paired	0.0453358	0.95385	RF	84.33	Spleen	No
SRX17287882	50106750	Paired	0.0488181	0.954777	RF	82.61	Spleen	No
SRX17287883	52572511	Paired	0.0530408	0.946884	RF	87.76	Spleen	No
SRX17287885	50189265	Paired	0.050035	0.951884	RF	84.27	Spleen	No
SRX17287886	52788346	Paired	0.0520319	0.94762	RF	87.72	Spleen	No
SRX17287887	49875489	Paired	0.0537113	0.946795	RF	84.59	Spleen	No
SRX17287888	50531144	Paired	0.056488	0.945967	RF	84.96	Spleen	No
SRX17287889	50392285	Paired	0.057394	0.939957	RF	87.76	Spleen	No
SRX17708420	20628218	Paired	0.980956	0.0209741	FR	81.66	T cell	No
SRX17708421	23052172	Paired	0.978986	0.021942	FR	82.52	T cell	No
SRX17708422	6522639	Paired	0.972273	0.0286695	FR	79.76	PBMCs	No
SRX17708423	18557190	Paired	0.975367	0.0262127	FR	82.94	T cell	No
SRX17708424	32134785	Paired	0.977843	0.0253358	FR	83.84	T cell	No
SRX16728290	30487505	Paired	0.0341416	0.964695	RF	87.37	Hair	No
SRX16728291	22875547	Paired	0.0273251	0.970729	RF	88.13	Hair	No
SRX16728292	26324993	Paired	0.0266417	0.970238	RF	88.89	Hair	No
SRX16728293	28177980	Paired	0.0371009	0.956178	RF	85.43	Hair	No
SRX16728294	24946334	Paired	0.0372484	0.956058	RF	85.96	Hair	No
SRX16728295	32021159	Paired	0.0355307	0.962912	RF	85.7	Hair	No
SRX16728296	30229122	Paired	0.0367384	0.958419	RF	86.57	Hair	No
SRX16728297	27214508	Paired	0.0360919	0.961012	RF	82.26	Hair	No
SRX16728298	26155855	Paired	0.0309447	0.965927	RF	86.48	Hair	No
SRX16728299	28539120	Paired	0.0250661	0.974134	RF	81.32	Hair	No
SRX14985610	34116265	Paired	0.961482	0.0333946	FR	83.37	Brain	No
SRX14985611	45463145	Paired	0.962494	0.032241	FR	82.82	Brain	No
SRX14985612	55884303	Paired	0.960141	0.034575	FR	82.06	Brain	No
SRX9152392	13792012	Paired	0.836612	0.171579	FR	60.43	Pancreas	No
SRX14985613	57052052	Paired	0.961473	0.0318247	FR	83.23	Brain	No
SRX14985614	58418090	Paired	0.962272	0.0332036	FR	82.53	Brain	No
SRX14985615	36187650	Paired	0.956015	0.0341952	FR	84	Brain	No
SRX14985616	42088381	Paired	0.962588	0.0317479	FR	82.5	Brain	No
SRX14985617	57401451	Paired	0.961761	0.0337828	FR	83.1	Brain	No
SRX14985618	34778596	Paired	0.955974	0.0446913	FR	86.45	Brain	No
SRX14664051	39811128	Paired	0.0151114	0.984976	RF	84.43	Muscle	No
SRX14985619	53443361	Paired	0.959267	0.0327009	FR	82.53	Brain	No
SRX14664052	36095436	Paired	0.0147448	0.986999	RF	84.28	Muscle	No
SRX14664053	60335987	Paired	0.0148461	0.985634	RF	85.69	Muscle	No
SRX14606351	26068689	Paired	0.021821	0.978678	RF	84.91	PK15	No
SRX14664054	43753870	Paired	0.0153059	0.985764	RF	86.13	Muscle	No
SRX14606352	26581402	Paired	0.0217781	0.978927	RF	84.68	PK15	No
SRX14664055	62306264	Paired	0.0154238	0.98575	RF	86.6	Muscle	No
SRX14606353	26385559	Paired	0.0219417	0.978714	RF	84.49	PK15	No
SRX7521370	30907638	Single	0.0140894	NULL	RF	94.83	Artery	No
SRX14664056	66951391	Paired	0.0153115	0.986282	RF	86.38	Muscle	No
SRX14606354	23126856	Paired	0.0267783	0.973074	RF	86.43	PK15	No
SRX7521371	28526859	Single	0.0144998	NULL	RF	93.06	Artery	No
SRX14606355	21272975	Paired	0.0290349	0.970937	RF	87.2	PK15	No

SRX14606356	22857689	Paired	0.0264146	0.973526	RF	87.31		PK15	No
SRX7521373	33092413	Single	0.014066	NULL	RF	94.17		Artery	No
SRX14606357	26383542	Paired	0.0218371	0.979101	RF	83.63		PK15	No
SRX14606358	23845536	Paired	0.022895	0.977612	RF	83.88		PK15	No
SRX14606359	24966295	Paired	0.0220445	0.978953	RF	83.34		PK15	No
SRX14112864	155928071	Paired	0.0211878	0.97324	RF	76		Muscle	No
SRX14112865	160654310	Paired	0.0183511	0.978581	RF	83.9		Muscle	No
SRX13202465	27281396	Single	0.979259	NULL	FR	70.71		PBMCs	No
SRX13202466	24695260	Single	0.979837	NULL	FR	75.58		PBMCs	No
SRX13202467	19673781	Single	0.978976	NULL	FR	76.11		PBMCs	No
SRX13202468	25715585	Single	0.980371	NULL	FR	76.98		PBMCs	No
SRX13202469	24542499	Single	0.980734	NULL	FR	68.8		PBMCs	No
SRX19520220	50442966	Paired	0.0270567	0.97337	RF	83.37		Ovary	No
SRX19520221	50237268	Paired	0.0342171	0.96541	RF	83.76		Ovary	No
SRX19520222	47993635	Paired	0.0360042	0.963646	RF	85.83		Ovary	No
SRX19520223	48590380	Paired	0.0352774	0.96457	RF	86.97		Ovary	No
ERX10011063	30814986	Paired	0.0882346	0.910816	RF	90.96		Large intestine	No
ERX10011064	34624356	Paired	0.102544	0.89601	RF	90.48		Large intestine	No
ERX10011065	35177066	Paired	0.0826189	0.916564	RF	91.57		Large intestine	No
ERX10011066	29187761	Paired	0.104976	0.893798	RF	90.95		Large intestine	No
ERX10011067	30287005	Paired	0.0849301	0.914421	RF	92.25		Large intestine	No
ERX10011068	31862037	Paired	0.107318	0.891279	RF	91.82		Large intestine	No
ERX10011069	32553159	Paired	0.0948474	0.904121	RF	91.85		Large intestine	No
SRX19901506	22778862	Paired	0.0195536	0.981172	RF	91.07		Macrophage	No
SRX19901507	24079572	Paired	0.0200127	0.980569	RF	90.87		Macrophage	No
SRX19901508	24021085	Paired	0.02042	0.980394	RF	92.23		Macrophage	No
SRX19901509	21527687	Paired	0.0209014	0.980087	RF	92.02		Macrophage	No
SRX18548240	45972294	Paired	0.109166	0.891221	RF	86.13		Placenta	No
SRX18548241	42417731	Paired	0.109003	0.890634	RF	83.47		Placenta	No
SRX18548242	45123114	Paired	0.0973458	0.902759	RF	88.1		Placenta	No
SRX18548243	46746036	Paired	0.0824546	0.919204	RF	86.88		Placenta	No
SRX18548244	48650017	Paired	0.0804753	0.921047	RF	87.43		Placenta	No
SRX18548245	44838086	Paired	0.0902103	0.911506	RF	86.23		Placenta	No
SRX12162923	23772995	Paired	0.0374518	0.963625	RF	90.59		Adipose	No
SRX12162924	21187691	Paired	0.0349735	0.966222	RF	88.88		Adipose	No
SRX12162925	26173313	Paired	0.0395946	0.961914	RF	90.31		Adipose	No
SRX12162926	23122730	Paired	0.0339888	0.967361	RF	90.34		Adipose	No
SRX12162927	24199806	Paired	0.0320118	0.969252	RF	90.3		Adipose	No
ERX7301081	96860417	Paired	0.0310025	0.94662	RF	89.55		Placenta	No
SRX12162928	21032356	Paired	0.0286857	0.972155	RF	89.9		Adipose	No
SRX12162929	21125860	Paired	0.0287219	0.972578	RF	90.32		Adipose	No
ERX7301083	144690912	Paired	0.0403968	0.960048	RF	90.05		Placenta	No
ERX7301085	102534978	Paired	0.0371156	0.965037	RF	87.92		Placenta	No
SRX16999573	57492440	Paired	0.092639	0.908898	RF	91.28		Muscle	No
SRX16999574	54060621	Paired	0.136362	0.867766	RF	82.7		Muscle	No
SRX16999575	48149900	Paired	0.141127	0.864432	RF	81.66		Muscle	No
SRX16999576	53528233	Paired	0.147996	0.857904	RF	72.27		Muscle	No
SRX16999577	47529736	Paired	0.139219	0.867146	RF	78.52		Muscle	No
SRX16999578	52261528	Paired	0.118733	0.885928	RF	83.23		Muscle	No
SRX16999579	53790584	Paired	0.127839	0.877967	RF	83.6		Muscle	No
ERX10011100	32520411	Paired	0.0939239	0.904898	RF	91.1		Large intestine	No
ERX10011101	33554623	Paired	0.0894985	0.909304	RF	90.97		Large intestine	No
SRX10783845	49628597	Single	0.015474	NULL	RF	95.61		Artery	No
ERX10011102	33127558	Paired	0.0897933	0.908445	RF	90.13		Large intestine	No
SRX10783846	38290515	Single	0.0172	NULL	RF	94.98		Artery	No
ERX10011103	31822065	Paired	0.0788068	0.920451	RF	92.2		Large intestine	No
SRX10783847	40294500	Single	0.0153889	NULL	RF	95.23		Artery	No
ERX10011104	31134156	Paired	0.0878125	0.910814	RF	91.44		Large intestine	No

SRX10783848	51774178	Single	0.0148519	NULL	RF	95.33	Artery	No
ERX10011105	32332565	Paired	0.0856606	0.913383	RF	92.14	Large intestine	No
SRX10783849	42716262	Single	0.0162438	NULL	RF	94.41	Artery	No
ERX10011106	38610057	Paired	0.086445	0.912237	RF	91.45	Large intestine	No
SRX14866548	24246605	Paired	0.0600075	0.94027	RF	89.31	IPEC-J2	No
SRX14866549	23230788	Paired	0.0507211	0.949608	RF	89.75	IPEC-J2	No
SRX17708390	16117476	Paired	0.97852	0.0228109	FR	76.91	PBMCs	No
SRX17708391	20607014	Paired	0.975931	0.025948	FR	79.69	PBMCs	No
SRX17708392	17046429	Paired	0.979205	0.0217791	FR	81.02	PBMCs	No
SRX17708393	21088779	Paired	0.977948	0.026137	FR	81.48	PBMCs	No
SRX17708394	19799617	Paired	0.977509	0.0284793	FR	80.11	PBMCs	No
SRX17708395	13516373	Paired	0.976131	0.0251885	FR	81.8	PBMCs	No
SRX17708396	15017639	Paired	0.97962	0.0212406	FR	81.51	T cell	No
SRX17708397	18081732	Paired	0.979899	0.0219084	FR	81.64	T cell	No
SRX17708398	12951397	Paired	0.978908	0.0233105	FR	81.88	T cell	No
SRX17708399	15542316	Paired	0.978839	0.0231647	FR	82.44	T cell	No
SRX13832262	30255895	Paired	0.0212541	0.978802	RF	83.47	Testis	No
SRX13832263	46277282	Paired	0.0191209	0.981067	RF	82.86	Testis	No
SRX13832264	34012505	Paired	0.0209666	0.979254	RF	81.48	Testis	No
SRX13832266	36287768	Paired	0.0200991	0.980075	RF	85.04	Testis	No
SRX17672950	22003673	Paired	0.0151758	0.98569	RF	89.53	Adipose	No
SRX17672951	22121153	Paired	0.0157796	0.985032	RF	92	Adipose	No
SRX17672952	22228859	Paired	0.0158787	0.984952	RF	91.97	Adipose	No
SRX17672953	22109334	Paired	0.0160283	0.984865	RF	92.07	Adipose	No
SRX17672954	21936806	Paired	0.0165215	0.984236	RF	89.17	Adipose	No
SRX17672955	22097885	Paired	0.016164	0.984682	RF	89.03	Adipose	No
SRX17672956	22103371	Paired	0.0166543	0.984244	RF	89.22	Adipose	No
SRX10367460	13528127	Paired	0.0291139	0.970939	RF	84.26	PK15	No
SRX10567291	48100972	Paired	0.0328613	0.960842	RF	68.53	Muscle	No
SRX10367461	17322209	Paired	0.0264906	0.973433	RF	78.48	PK15	No
SRX10567292	48470464	Paired	0.0306622	0.962146	RF	64.55	Muscle	No
SRX10367462	13805804	Paired	0.0261012	0.974535	RF	74.44	PK15	No
SRX11617236	46748001	Paired	0.117874	0.878374	RF	89.4	Testis	No
SRX10367463	19649505	Paired	0.0257581	0.974169	RF	78.73	PK15	No
SRX11617237	47810380	Paired	0.160205	0.835662	RF	92.27	Testis	No
SRX10567294	46223235	Paired	0.0283143	0.967104	RF	69.41	Muscle	No
SRX10367464	19914620	Paired	0.0258066	0.974235	RF	78.13	PK15	No
SRX11617238	43043596	Paired	0.154999	0.841225	RF	90.4	Testis	No
SRX10567295	45649529	Paired	0.0294824	0.962305	RF	60.15	Muscle	No
SRX10367465	19231584	Paired	0.0250813	0.974569	RF	77.2	PK15	No
SRX11617239	39481419	Paired	0.162388	0.833823	RF	90.28	Testis	No
SRX10367466	16911955	Paired	0.0234416	0.977195	RF	79.3	PK15	No
SRX10567297	48052052	Paired	0.0570868	0.93366	RF	72.65	Adipose	No
SRX10367467	12328571	Paired	0.0255452	0.975018	RF	77.74	PK15	No
SRX10567298	48242365	Paired	0.0573401	0.939172	RF	84.32	Adipose	No
SRX10367468	18294988	Paired	0.0222369	0.978428	RF	81.14	PK15	No
SRX10567299	49358692	Paired	0.0503888	0.941647	RF	69.77	Adipose	No
SRX10367469	17795666	Paired	0.0228124	0.977373	RF	85.57	PK15	No
SRX12569190	45047797	Paired	0.0333199	0.968074	RF	92.73	Small intestine	No
SRX13276530	15384166	Paired	0.0252273	0.975142	RF	86.06	Brain	No
SRX12569191	42075132	Paired	0.0409208	0.960438	RF	93.09	Small intestine	No
SRX13276531	24054924	Paired	0.0260164	0.97432	RF	83.62	Brain	No
SRX12569192	36516283	Paired	0.0435268	0.958085	RF	92.04	Small intestine	No
SRX13276532	18206436	Paired	0.0252911	0.975314	RF	83.72	Brain	No
SRX12569193	41067081	Paired	0.0351041	0.96616	RF	91.98	Small intestine	No
SRX13276533	17032004	Paired	0.0244986	0.975779	RF	80.02	Brain	No
SRX12569194	37768483	Paired	0.0430113	0.958102	RF	91.91	Small intestine	No
SRX13276534	18380654	Paired	0.0240164	0.976525	RF	84.6	Brain	No

SRX12569195	36347579	Paired	0.0448291	0.956043	RF	91.9	Small intestine	No
SRX13276535	23838562	Paired	0.0247772	0.975825	RF	83.72	Brain	No
SRX12569196	46481705	Paired	0.0457239	0.955064	RF	92.06	Small intestine	No
SRX13276536	21793677	Paired	0.0236664	0.976896	RF	84.71	Brain	No
SRX13276537	21232785	Paired	0.0237037	0.976648	RF	78.75	Brain	No
SRX13276538	22313454	Paired	0.0246901	0.975646	RF	82.37	Brain	No
SRX13276539	23789601	Paired	0.024863	0.975606	RF	83.79	Brain	No
SRX14228390	21888831	Paired	0.0338468	0.968762	RF	85.07	Heart	No
SRX14228391	13781988	Paired	0.0357844	0.966732	RF	88.68	Heart	No
SRX14228392	18113160	Paired	0.0346111	0.96835	RF	90.64	Heart	No
SRX14228393	18923010	Paired	0.0338833	0.968354	RF	87.11	Heart	No
SRX14228394	15179513	Paired	0.036048	0.966175	RF	87.56	Heart	No
SRX14228395	14723706	Paired	0.0388244	0.963538	RF	87.5	Heart	No
SRX14228396	18610995	Paired	0.0359721	0.966475	RF	90.31	Heart	No
SRX17287891	50150351	Paired	0.0438971	0.953562	RF	83.17	Spleen	No
SRX14228397	17452690	Paired	0.0392004	0.961296	RF	87.46	Heart	No
SRX17287892	51988506	Paired	0.039569	0.964376	RF	85	Spleen	No
SRX14228398	13354571	Paired	0.0373122	0.964548	RF	88.23	Heart	No
SRX14228399	13990173	Paired	0.0366496	0.965522	RF	87.72	Heart	No
ERX7562541	24489717	Paired	0.0276826	0.973658	RF	87.54	Uterus	No
ERX7562542	20263854	Paired	0.0376241	0.961053	RF	62.07	Uterus	No
ERX7562543	18770463	Paired	0.0257437	0.974247	RF	67.87	Uterus	No
ERX7562544	26689567	Paired	0.0323837	0.967739	RF	79.28	Uterus	No
ERX7562545	19339920	Paired	0.0294392	0.970459	RF	72.86	Uterus	No
ERX7562546	22325429	Paired	0.028463	0.972056	RF	78.88	Uterus	No
SRX14985620	52888841	Paired	0.965793	0.0310448	FR	83.1	Brain	No
SRX10327872	9424176	Paired	0.0267349	0.975043	RF	91.4	Ovary	No
SRX14606360	25548473	Paired	0.0224248	0.978504	RF	85.06	PK15	No
SRX10327873	9858956	Paired	0.0275418	0.975011	RF	91.21	Ovary	No
SRX14606361	22860638	Paired	0.0220342	0.978954	RF	85.39	PK15	No
SRX10327874	7686733	Paired	0.0289982	0.973562	RF	89.07	Ovary	No
SRX14606362	27004118	Paired	0.0213583	0.979621	RF	86.2	PK15	No
SRX10327875	10730872	Paired	0.0271435	0.974979	RF	92.49	Ovary	No
SRX10327876	10016214	Paired	0.0271931	0.974907	RF	91.86	Ovary	No
SRX10327877	12830144	Paired	0.0259404	0.976417	RF	92.23	Ovary	No
SRX10327878	8434642	Paired	0.0330711	0.968758	RF	82.44	Ovary	No
SRX10327879	15114886	Paired	0.0263022	0.975847	RF	90.75	Ovary	No
SRX14112870	114494228	Paired	0.0226731	0.973039	RF	79.06	Muscle	No
SRX14112871	121147775	Paired	0.0232836	0.971726	RF	73.06	Muscle	No
SRX14112872	150659679	Paired	0.0194406	0.979259	RF	80.93	Muscle	No
SRX14112873	148102859	Paired	0.024891	0.971213	RF	82.21	Muscle	No
SRX13202470	20516420	Single	0.981025	NULL	FR	76.37	PBMCs	No
SRX13202471	29759370	Single	0.980901	NULL	FR	70.49	PBMCs	No
SRX13202472	27146561	Single	0.980456	NULL	FR	66.08	PBMCs	No
SRX13202473	25512048	Single	0.981462	NULL	FR	75.12	PBMCs	No
SRX13202474	27382723	Single	0.981654	NULL	FR	71.05	PBMCs	No
SRX13202475	33217588	Single	0.981514	NULL	FR	66.68	PBMCs	No
SRX13202476	18628613	Single	0.980234	NULL	FR	70.16	PBMCs	No
SRX15414254	64198149	Paired	0.0179467	0.981522	RF	70.74	Adipose	No
SRX15414255	38697072	Paired	0.0334116	0.965482	RF	80.88	Adipose	No
SRX15414256	62854897	Paired	0.018755	0.980513	RF	74.81	Adipose	No
SRX15414257	68875180	Paired	0.0185896	0.98107	RF	74.87	Adipose	No
SRX15414258	75058025	Paired	0.0202688	0.979225	RF	74.48	Adipose	No
SRX15414259	64443878	Paired	0.0175318	0.982468	RF	78.31	Adipose	No
ERX10011070	34659659	Paired	0.0924705	0.906006	RF	91.85	Large intestine	No
ERX10011071	34414325	Paired	0.099804	0.899106	RF	90.73	Large intestine	No
ERX10011072	29829143	Paired	0.10525	0.893474	RF	90.29	Large intestine	No
ERX10011073	31923852	Paired	0.0945203	0.904385	RF	91.22	Large intestine	No

ERX10011074	31569593	Paired	0.0990949	0.899588	RF	91.73	Large intestine	No
SRX19901510	24410017	Paired	0.0238181	0.976821	RF	87.12	Macrophage	No
ERX10011075	29831154	Paired	0.092013	0.90692	RF	91.72	Large intestine	No
SRX19901511	23539597	Paired	0.0221398	0.978847	RF	89.26	Macrophage	No
ERX10011076	32665321	Paired	0.108406	0.89051	RF	91.76	Large intestine	No
SRX19901512	24903166	Paired	0.0194531	0.98148	RF	91.26	Macrophage	No
ERX10011077	35995870	Paired	0.0864636	0.912618	RF	91.78	Large intestine	No
SRX19901513	23370817	Paired	0.0188605	0.981705	RF	92.1	Macrophage	No
ERX10011078	32312248	Paired	0.101565	0.897141	RF	90.98	Large intestine	No
SRX19901514	24270655	Paired	0.0197731	0.98109	RF	92.41	Macrophage	No
ERX10011079	33119756	Paired	0.0904276	0.908511	RF	91.1	Large intestine	No
SRX19901515	24418374	Paired	0.021427	0.979638	RF	89.31	Macrophage	No
SRX19901516	21113395	Paired	0.0194224	0.981285	RF	91.4	Macrophage	No
SRX19901517	23673529	Paired	0.0184265	0.982208	RF	92.2	Macrophage	No
SRX7416743	23014186	Paired	0.0209191	0.982318	RF	86.73	Liver	No
SRX7416744	26188108	Paired	0.0230225	0.98038	RF	83.77	Liver	No
SRX7416745	25525729	Paired	0.0183736	0.984813	RF	86.04	Liver	No
SRX7416746	25675434	Paired	0.0205159	0.982133	RF	80.63	Liver	No
SRX7416747	19694644	Paired	0.0243432	0.979059	RF	82.27	Liver	No
SRX16728300	34476639	Paired	0.0225477	0.974462	RF	88.64	Hair	No
SRX7416748	27748770	Paired	0.0211824	0.982256	RF	82.77	Liver	No
SRX16728301	41745928	Paired	0.0230264	0.973744	RF	88.09	Hair	No
SRX7416749	24240068	Paired	0.0218643	0.982234	RF	85.31	Liver	No
SRX16728302	34612131	Paired	0.0237472	0.973044	RF	87.37	Hair	No
SRX16728303	27217502	Paired	0.0216966	0.974565	RF	87.71	Hair	No
SRX16728304	31993227	Paired	0.0200513	0.977372	RF	85.93	Hair	No
SRX16728305	35994176	Paired	0.0251234	0.970355	RF	81.03	Hair	No
SRX16728306	48929719	Paired	0.0254014	0.970396	RF	87.18	Hair	No
SRX16728307	33147411	Paired	0.0244738	0.971353	RF	86.87	Hair	No
SRX5807482	37974911	Paired	0.0359482	0.963248	RF	85.06	Liver	No
SRX16728308	52061056	Paired	0.0285875	0.970288	RF	82.33	Hair	No
SRX12014420	41325599	Paired	0.0260985	0.973972	RF	71.37	Adipose	No
SRX5807483	27466354	Paired	0.0345343	0.964703	RF	85.58	Liver	No
SRX16728309	40693698	Paired	0.020588	0.975673	RF	86.57	Hair	No
SRX12014421	38066572	Paired	0.030206	0.969497	RF	73.21	Adipose	No
SRX12014422	30845577	Paired	0.0328225	0.967483	RF	81.89	Adipose	No
SRX12014423	33284857	Paired	0.0325003	0.967546	RF	82.82	Adipose	No
SRX9152404	14237887	Paired	0.853131	0.181326	FR	72.9	Pancreas	No
SRX12162930	27698666	Paired	0.0383108	0.963034	RF	89.5	Adipose	No
SRX12162931	28047856	Paired	0.0299268	0.971312	RF	90.04	Adipose	No
SRX9152406	17522295	Paired	0.850587	0.182057	FR	73.78	Pancreas	No
SRX12162932	20747968	Paired	0.0302422	0.971052	RF	90.64	Adipose	No
SRX9152407	14263209	Paired	0.864518	0.170777	FR	73.32	Pancreas	No
SRX12162933	22745361	Paired	0.0310158	0.970276	RF	89	Adipose	No
SRX9152408	18917231	Paired	0.847886	0.178299	FR	66.67	Pancreas	No
SRX12162934	26917708	Paired	0.0202162	0.980656	RF	89.84	Adipose	No
SRX9152409	20638671	Paired	0.849151	0.183045	FR	73.71	Pancreas	No
SRX12162935	22777237	Paired	0.022169	0.979116	RF	90.51	Adipose	No
SRX12162936	21888945	Paired	0.0203823	0.981265	RF	90.37	Adipose	No
SRX12162937	27915689	Paired	0.0186286	0.98187	RF	90.57	Adipose	No
SRX12162938	22981271	Paired	0.0378446	0.9632	RF	89.48	Adipose	No
SRX12162939	24711954	Paired	0.0338665	0.967101	RF	90.25	Adipose	No
ERX7301055	105439503	Paired	0.0285248	0.97221	RF	89.39	Liver	No
ERX7301057	120922300	Paired	0.0334869	0.970665	RF	87.3	Liver	No
ERX7301059	203304344	Paired	0.02769	0.976451	RF	90.99	Liver	No
SRX13166670	374424135	Paired	0.0165659	0.98557	RF	83.13	Liver	No
SRX8564021	34863302	Paired	0.0687423	0.932177	RF	81.62	Spleen	No
SRX8564022	33804538	Paired	0.0618421	0.938746	RF	79.15	Spleen	No

SRX8564023	35685971	Paired	0.0754041	0.925075	RF	82.52	Spleen	No
SRX8564024	37354085	Paired	0.077324	0.923152	RF	81.92	Spleen	No
SRX8564025	37435918	Paired	0.0736093	0.926727	RF	73.77	Spleen	No
SRX8564026	35684821	Paired	0.0695961	0.930601	RF	71.21	Spleen	No
SRX8564027	36885235	Paired	0.0727309	0.927808	RF	83.05	Spleen	No
SRX14127113	30949172	Paired	0.948054	0.0504265	FR	88.19	PBMCs	No
SRX8564028	32787220	Paired	0.0847339	0.91499	RF	80.72	Spleen	No
SRX14127114	31172915	Paired	0.948263	0.0499716	FR	85.96	PBMCs	No
SRX8564029	30793867	Paired	0.076274	0.923831	RF	78.4	Spleen	No
SRX14127115	33717041	Paired	0.942436	0.0556176	FR	85.09	PBMCs	No
SRX14127116	36771523	Paired	0.944289	0.0534941	FR	86.06	PBMCs	No
SRX14127117	35786062	Paired	0.943246	0.055093	FR	85.55	PBMCs	No
SRX14127118	38570201	Paired	0.945078	0.0520248	FR	84.7	PBMCs	No
SRX10783850	43683312	Single	0.0152783	NULL	RF	94.2	Artery	No
SRX14127119	38665288	Paired	0.94483	0.0526403	FR	85.71	PBMCs	No
SRX10783851	46420204	Single	0.0155068	NULL	RF	95.34	Artery	No
SRX14866550	24966550	Paired	0.0511872	0.949202	RF	89.57	IPEC-J2	No
SRX10783852	41644379	Single	0.0164943	NULL	RF	94.24	Artery	No
SRX14866551	24257820	Paired	0.039509	0.960798	RF	90.33	IPEC-J2	No
SRX10783853	49838837	Single	0.015756	NULL	RF	94.57	Artery	No
SRX14866552	23469164	Paired	0.0491721	0.951194	RF	90.01	IPEC-J2	No
SRX10783854	46002014	Single	0.015675	NULL	RF	95.02	Artery	No
SRX14866553	23725750	Paired	0.0459878	0.954305	RF	89.89	IPEC-J2	No
SRX10783855	43650639	Single	0.0160133	NULL	RF	94.31	Artery	No
SRX10783856	41835450	Single	0.0152875	NULL	RF	94.8	Artery	No
SRX17708361	6608465	Paired	0.970262	0.0309922	FR	81.48	PBMCs	No
SRX17708362	9392454	Paired	0.974086	0.0278926	FR	82.02	PBMCs	No
SRX9059743	53526817	Paired	0.0578963	0.940456	RF	83.77	Adipose	No
SRX17708363	23313568	Paired	0.97926	0.022092	FR	83.09	T cell	No
SRX9059744	57026870	Paired	0.0482707	0.950212	RF	82.42	Adipose	No
SRX17708364	23707699	Paired	0.977663	0.0233672	FR	82.44	T cell	No
SRX9059745	53659702	Paired	0.0752826	0.924459	RF	88.08	Adipose	No
SRX17708365	17748878	Paired	0.976956	0.0232851	FR	83.21	T cell	No
SRX9059746	52927907	Paired	0.0750374	0.92469	RF	84.02	Adipose	No
SRX17708366	18971473	Paired	0.978013	0.0235559	FR	80.86	T cell	No
SRX9059747	54196631	Paired	0.0498788	0.949172	RF	87.69	Adipose	No
SRX17708367	17981104	Paired	0.980199	0.0197289	FR	84.23	T cell	No
SRX9059748	50726628	Paired	0.0201777	0.980922	RF	88.58	Adipose	No
SRX17708368	17600849	Paired	0.978737	0.02187	FR	77.74	T cell	No
SRX9059749	46748912	Paired	0.0267928	0.973165	RF	88.98	Adipose	No
SRX17708369	20782503	Paired	0.978846	0.0217003	FR	78.37	PBMCs	No
SRX18427681	75633334	Paired	0.0201495	0.979266	RF	93.44	Ovary	No
SRX18427682	70216413	Paired	0.0187282	0.981193	RF	92.77	Ovary	No
SRX18427683	99193925	Paired	0.0215973	0.977074	RF	93.76	Ovary	No
SRX18427684	91960797	Paired	0.0298083	0.967458	RF	93.77	Ovary	No
SRX18427685	57627789	Paired	0.0311655	0.96768	RF	93.46	Ovary	No
SRX18427686	69642344	Paired	0.027549	0.971371	RF	93.77	Ovary	No
SRX10171664	37616268	Paired	0.0168193	0.982784	RF	94.11	Fibroblast	No
SRX18427687	76168867	Paired	0.0278297	0.969829	RF	92.9	Ovary	No
SRX10171665	36000106	Paired	0.0206869	0.979104	RF	93.72	Fibroblast	No
SRX18427688	85894178	Paired	0.0244335	0.974966	RF	93.23	Ovary	No
SRX11617240	43400094	Paired	0.158234	0.838131	RF	90.01	Testis	No
SRX10171666	38378628	Paired	0.0173251	0.982734	RF	93.77	Fibroblast	No
SRX18427689	52470286	Paired	0.0267523	0.97082	RF	93.41	Ovary	No
SRX11617241	40019271	Paired	0.13147	0.864801	RF	89.49	Testis	No
SRX10171667	36908073	Paired	0.0177514	0.982352	RF	93.76	Fibroblast	No
SRX11617242	38538396	Paired	0.142719	0.853558	RF	90.19	Testis	No
SRX11617243	40906009	Paired	0.157969	0.838453	RF	89.23	Testis	No

SRX10367470	24545132	Paired	0.0201206	0.98053	RF	78.33	PK15	No
SRX11617244	45514291	Paired	0.146713	0.849105	RF	92.29	Testis	No
SRX10367471	20927708	Paired	0.0205646	0.980243	RF	79.22	PK15	No
SRX11617245	45196072	Paired	0.152307	0.844324	RF	91.75	Testis	No
SRX11617246	42707396	Paired	0.159944	0.836427	RF	89.96	Testis	No
SRX11617247	40136933	Paired	0.154344	0.841639	RF	89.3	Testis	No
SRX6430468	66491308	Paired	0.0292697	0.970124	RF	83.73	PK15	No
SRX6430469	85781105	Paired	0.0277943	0.971897	RF	83.26	PK15	No
SRX13276540	18269497	Paired	0.0246623	0.975832	RF	82.61	Brain	No
SRX13276541	22947759	Paired	0.0222278	0.978295	RF	83.18	Brain	No
SRX13276542	24991770	Paired	0.0236105	0.976797	RF	82.75	Brain	No
SRX13276543	22692410	Paired	0.0235555	0.976993	RF	85.01	Brain	No
SRX13276544	18652256	Paired	0.0247786	0.975645	RF	78.27	Brain	No
SRX13276545	25065913	Paired	0.0231793	0.977324	RF	81.31	Brain	No
SRX15447807	41926082	Paired	0.104496	0.894617	RF	63.02	Small intestine	No
SRX13276546	21171039	Paired	0.0201037	0.981044	RF	84.45	Large intestine	No
SRX15447808	46590492	Paired	0.130708	0.867913	RF	60.19	Small intestine	No
SRX13276547	18978992	Paired	0.0201426	0.981011	RF	84.05	Large intestine	No
SRX13276506	24450337	Paired	0.0246975	0.975889	RF	80.45	Brain	No
SRX13276548	23810189	Paired	0.0194057	0.981562	RF	83.3	Large intestine	No
SRX13276507	18561275	Paired	0.0236949	0.97681	RF	80.87	Brain	No
SRX13276549	19165561	Paired	0.0200912	0.980861	RF	84.44	Large intestine	No
SRX13276508	25226945	Paired	0.0247012	0.975658	RF	83.79	Brain	No
SRX13276509	17389442	Paired	0.0255446	0.974854	RF	79.19	Brain	No
SRX13328059	31760076	Paired	0.0398938	0.958209	RF	86.02	Macrophage	No
SRX11121522	42235050	Paired	0.0214462	0.979312	RF	79.72	Muscle	No
SRX11121523	50032822	Paired	0.0214303	0.979242	RF	77.25	Muscle	No
SRX11121524	48478198	Paired	0.0215049	0.978501	RF	69.14	Muscle	No
SRX11121525	53777839	Paired	0.0227376	0.977985	RF	75.42	Muscle	No
SRX11121526	41919454	Paired	0.0237807	0.977305	RF	78.21	Muscle	No
SRX11121527	48096824	Paired	0.0273011	0.9738	RF	77.7	Muscle	No
SRX11121528	43003661	Paired	0.0299351	0.970899	RF	81.71	Muscle	No
SRX11121529	47744473	Paired	0.0345656	0.965451	RF	81.64	Muscle	No
SRX19771835	18899728	Paired	0.0196094	0.980899	RF	87.74	IPEC-J2	No
SRX17708400	26252654	Paired	0.977382	0.0256491	FR	82.54	T cell	No
SRX19771836	18189334	Paired	0.0195084	0.981416	RF	87.37	IPEC-J2	No
SRX17708401	21877350	Paired	0.978113	0.0231001	FR	80.87	T cell	No
SRX19771837	17344156	Paired	0.0188308	0.981536	RF	86.22	IPEC-J2	No
SRX17708402	21010494	Paired	0.97842	0.0227915	FR	79.73	T cell	No
SRX19771838	19213706	Paired	0.0200746	0.980537	RF	84.31	IPEC-J2	No
SRX17708403	14198876	Paired	0.975196	0.026867	FR	76.78	T cell	No
SRX19771839	20653742	Paired	0.0204146	0.980128	RF	84.2	IPEC-J2	No
SRX17708404	18385725	Paired	0.978425	0.0230425	FR	79.48	PBMCs	No
SRX17708405	19249999	Paired	0.979658	0.0209009	FR	81.31	PBMCs	No
SRX17708406	14424757	Paired	0.978173	0.0234152	FR	82.92	PBMCs	No
SRX17708407	10579352	Paired	0.976807	0.0234881	FR	77.8	PBMCs	No
SRX17708408	16210023	Paired	0.978297	0.0230644	FR	82.56	PBMCs	No
SRX16728274	34726648	Paired	0.0343541	0.963711	RF	83.92	Hair	No
SRX17708409	15365299	Paired	0.978606	0.02171	FR	80.84	PBMCs	No
SRX16728275	27626882	Paired	0.026455	0.972627	RF	84.42	Hair	No
SRX16728276	35550680	Paired	0.0321932	0.966357	RF	84.7	Hair	No
SRX16728277	34212363	Paired	0.036555	0.961601	RF	83.53	Hair	No
SRX16728278	31933650	Paired	0.0296957	0.968662	RF	86.46	Hair	No
SRX16728279	32299682	Paired	0.0339494	0.964977	RF	85.55	Hair	No
SRX10954790	22692594	Single	0.0245658	NULL	RF	92.06	Heart	No
SRX10954791	25180062	Single	0.0219177	NULL	RF	93.17	Heart	No
SRX10954792	28241543	Single	0.0206832	NULL	RF	91.32	Heart	No
SRX10954793	28255186	Single	0.0206974	NULL	RF	91.09	Heart	No

SRX10954794	28210820	Single	0.022047	NULL	RF	92.43	Heart	No
SRX10954795	27751964	Single	0.022611	NULL	RF	92.43	Heart	No
SRX10327880	9019095	Paired	0.0323313	0.970657	RF	91.03	Ovary	No
SRX10954796	26232379	Single	0.021589	NULL	RF	91.8	Heart	No
SRX10327881	9203329	Paired	0.0307746	0.972134	RF	89.17	Ovary	No
SRX10954797	31108543	Single	0.0238564	NULL	RF	90.19	Heart	No
SRX10327882	9856896	Paired	0.0286558	0.973418	RF	92.16	Ovary	No
SRX10954798	27707588	Single	0.0240493	NULL	RF	91.62	Heart	No
SRX10327883	10249129	Paired	0.0270364	0.975259	RF	91.28	Ovary	No
SRX10954799	25372114	Single	0.0206646	NULL	RF	93.14	Heart	No
SRX10567300	49867359	Paired	0.0413587	0.954612	RF	82.26	Adipose	No
SRX10327884	10677029	Paired	0.0284917	0.973297	RF	92.33	Ovary	No
SRX10567301	48265073	Paired	0.0418202	0.95185	RF	73.69	Adipose	No
SRX10327885	14558129	Paired	0.0293672	0.972801	RF	91.83	Ovary	No
SRX10567302	50612020	Paired	0.0432457	0.951673	RF	79.41	Adipose	No
SRX10327886	8165295	Paired	0.0306173	0.972368	RF	90.65	Ovary	No
SRX10567303	48432967	Paired	0.0497668	0.944592	RF	75.68	Adipose	No
SRX10327887	7949617	Paired	0.0315967	0.970692	RF	91.73	Ovary	No
SRX10567304	48593625	Paired	0.0412066	0.95767	RF	91.55	Adipose	No
SRX10327888	8512558	Paired	0.0309527	0.971052	RF	92.02	Ovary	No
SRX10567305	49319910	Paired	0.0373022	0.961888	RF	89.65	Adipose	No
SRX10327889	7993215	Paired	0.0310013	0.971137	RF	91.42	Ovary	No
SRX10567306	48748306	Paired	0.0496345	0.941005	RF	69.67	Adipose	No
SRX10567307	49760397	Paired	0.0333151	0.963509	RF	78.73	Adipose	No
SRX10567308	50163853	Paired	0.039768	0.957129	RF	84.81	Adipose	No
SRX13838660	25284498	Paired	0.0204156	0.980495	RF	83.3	Large intestine	No
SRX13838661	25352808	Paired	0.0200152	0.980754	RF	84.34	Large intestine	No
SRX13838662	19877783	Paired	0.0179078	0.983099	RF	84.78	Large intestine	No
SRX13838663	23606124	Paired	0.0205158	0.980408	RF	86.66	Large intestine	No
SRX13838664	19799924	Paired	0.0204342	0.980532	RF	85.17	Large intestine	No
SRX13838665	21135389	Paired	0.0217301	0.978999	RF	84.63	Large intestine	No
SRX13838666	24487088	Paired	0.0209587	0.979874	RF	84.35	Large intestine	No
SRX17424958	23443841	Paired	0.0217747	0.978747	RF	91.97	PK15	No
SRX17424959	23843708	Paired	0.0209605	0.979273	RF	90.16	PK15	No
SRX19857310	36352917	Paired	0.0464456	0.95209	RF	72.04	Synovial membrane	No
SRX19857311	44055907	Paired	0.0570679	0.94113	RF	67.83	Synovial membrane	No
SRX19857312	40771249	Paired	0.0508192	0.947669	RF	68.69	Synovial membrane	No
SRX19857313	45675957	Paired	0.0549754	0.942437	RF	73.08	Synovial membrane	No
SRX19857314	35848875	Paired	0.0765614	0.921082	RF	75.47	Synovial membrane	No
SRX19857315	38408442	Paired	0.0533644	0.942304	RF	61.91	Synovial membrane	No
SRX20062580	39798911	Paired	0.0979134	0.867629	RF	73.81	Kidney	No
SRX20062581	32476590	Paired	0.0581838	0.919485	RF	69.6	Liver	No
SRX20062540	39234620	Paired	0.118577	0.836971	RF	69.04	Heart	No
SRX20062582	40298953	Paired	0.0555582	0.929618	RF	78.85	Liver	No
SRX20062541	29560495	Paired	0.0748928	0.916975	RF	75.16	Heart	No
SRX20062583	38508108	Paired	0.0500384	0.945906	RF	77.95	Liver	No
SRX20062584	42895034	Paired	0.0425159	0.944165	RF	84.06	Liver	No
SRX20062585	43337489	Paired	0.0567521	0.93156	RF	77.91	Liver	No
SRX20062587	33705151	Paired	0.0341145	0.957682	RF	82.49	Liver	No
SRX20062588	45386828	Paired	0.0590515	0.923868	RF	76.39	Liver	No
SRX20062589	35839005	Paired	0.0365854	0.952163	RF	85.42	Liver	No
SRX17655860	66691879	Paired	0.978792	0.0222572	FR	84.6	Muscle	No
SRX17655861	36323570	Paired	0.978891	0.0229777	FR	80.64	Muscle	No
SRX17655862	28278282	Paired	0.0206121	0.981917	RF	91.63	Muscle	No
SRX17655863	75376939	Paired	0.980349	0.0215074	FR	82.57	Muscle	No
SRX17655864	34051055	Paired	0.0176488	0.984457	RF	91.03	Muscle	No
SRX17655865	35843632	Paired	0.976138	0.0262895	FR	80.06	Muscle	No
SRX17655866	30714662	Paired	0.968079	0.0265	FR	82.79	Muscle	No

SRX17655825	64527608	Paired	0.977217	0.0229038	FR	83.09	Muscle	No
SRX17655867	36177429	Paired	0.972158	0.0267035	FR	82.61	Muscle	No
SRX17655826	63814783	Paired	0.97867	0.023314	FR	80.33	Muscle	No
SRX17655868	70648825	Paired	0.976818	0.0243843	FR	86.01	Muscle	No
SRX17655827	38371197	Paired	0.972805	0.0251246	FR	81.63	Muscle	No
SRX17655869	40886866	Paired	0.979029	0.0229174	FR	86.53	Muscle	No
SRX17655828	34349222	Paired	0.96607	0.0338611	FR	84.39	Muscle	No
SRX17655829	35279754	Paired	0.974848	0.0250266	FR	88.3	Muscle	No
SRX17655940	36406702	Paired	0.973619	0.0251418	FR	84.03	Muscle	No
SRX17655941	29800256	Paired	0.975706	0.0249444	FR	80.99	Muscle	No
SRX17655900	30681954	Paired	0.0176002	0.984421	RF	91.6	Muscle	No
SRX17655942	33405826	Paired	0.975067	0.025831	FR	81.35	Muscle	No
SRX17655901	31882878	Paired	0.972867	0.0257368	FR	80.97	Muscle	No
SRX17655943	38669547	Paired	0.971317	0.0280031	FR	84.19	Muscle	No
SRX17655902	70936447	Paired	0.977656	0.0236405	FR	84.15	Muscle	No
SRX17655944	34174256	Paired	0.97711	0.0249478	FR	86.95	Muscle	No
SRX17655903	30282321	Paired	0.973488	0.0261804	FR	83.32	Muscle	No
SRX17655945	28517426	Paired	0.974306	0.026281	FR	87.74	Muscle	No
SRX17655904	34432013	Paired	0.975891	0.0251556	FR	82.68	Muscle	No
SRX17655946	33962448	Paired	0.978219	0.0235957	FR	80.57	Muscle	No
SRX17655905	67874744	Paired	0.977398	0.0241885	FR	84.15	Muscle	No
SRX17655947	34710043	Paired	0.978623	0.0235117	FR	81.84	Muscle	No
SRX17655906	60849394	Paired	0.975447	0.0251067	FR	81.13	Muscle	No
SRX17655948	31350291	Paired	0.967492	0.027652	FR	83.36	Muscle	No
SRX17655907	30923277	Paired	0.970014	0.0285415	FR	79.72	Muscle	No
SRX17655949	36300734	Paired	0.979629	0.0222205	FR	84.95	Muscle	No
SRX17655908	80158529	Paired	0.979441	0.0218733	FR	84.31	Muscle	No
SRX17655909	76721619	Paired	0.976797	0.022496	FR	85.9	Muscle	No
SRX19857280	40225244	Paired	0.0541033	0.944118	RF	65.54	Synovial membrane	No
SRX19857281	34425382	Paired	0.0659857	0.932119	RF	74.38	Synovial membrane	No
SRX19857240	27441803	Paired	0.0578131	0.940824	RF	84.49	Cartilage	No
SRX19857282	37114950	Paired	0.0461033	0.953131	RF	64.67	Synovial membrane	No
SRX19857241	32786385	Paired	0.0479931	0.94883	RF	73.26	Cartilage	No
SRX19857283	28919926	Paired	0.0617258	0.936649	RF	68.37	Synovial membrane	No
SRX19857242	38946244	Paired	0.0516006	0.924256	RF	63.25	Cartilage	No
SRX19857284	33156312	Paired	0.0768427	0.921348	RF	76.15	Synovial membrane	No
SRX19857243	36528894	Paired	0.0537101	0.930196	RF	80.62	Cartilage	No
SRX19857285	36157114	Paired	0.0482739	0.949023	RF	62.85	Synovial membrane	No
SRX19857244	38559290	Paired	0.0560019	0.927121	RF	68.43	Cartilage	No
SRX19857286	37575860	Paired	0.0689007	0.927148	RF	72.5	Synovial membrane	No
SRX19857245	40448762	Paired	0.0707309	0.912499	RF	82.32	Cartilage	No
SRX19857287	34763057	Paired	0.0539421	0.944725	RF	71.51	Synovial membrane	No
SRX19857246	40374645	Paired	0.0651424	0.92247	RF	70.79	Cartilage	No
SRX19857288	33600267	Paired	0.0672414	0.926428	RF	74.48	Cartilage	No
SRX19857247	33202637	Paired	0.048254	0.940769	RF	81.48	Cartilage	No
SRX19857289	37226291	Paired	0.0562065	0.925862	RF	65.68	Cartilage	No
SRX19857248	41606196	Paired	0.0590819	0.922258	RF	71.05	Cartilage	No
SRX19857249	34353812	Paired	0.0500023	0.924718	RF	63.85	Cartilage	No
SRX17424960	24831014	Paired	0.0186848	0.981863	RF	89.17	PK15	No
SRX17424961	25047297	Paired	0.0210921	0.979573	RF	91.57	PK15	No
SRX17424962	23276943	Paired	0.0212872	0.979383	RF	91.58	PK15	No
SRX17424963	25003092	Paired	0.0203135	0.980361	RF	91.28	PK15	No
SRX20062554	38928089	Paired	0.0908521	0.898547	RF	80.48	Lung	No
SRX20062555	39271535	Paired	0.0873433	0.899558	RF	85.25	Lung	No
SRX20062557	31617615	Paired	0.0691319	0.908925	RF	79.71	Lung	No
SRX20062558	31969982	Paired	0.0759186	0.898927	RF	81.71	Lung	No
SRX20062559	32941189	Paired	0.0699832	0.909329	RF	81.01	Lung	No
SRX17655870	56766278	Paired	0.976837	0.0239581	FR	85.37	Muscle	No

SRX17655830	37843800	Paired	0.979216	0.0227279	FR	83.11	Muscle	No
SRX17655871	37384647	Paired	0.979775	0.0221278	FR	81.68	Muscle	No
SRX17655831	32519639	Paired	0.973496	0.0267015	FR	88.65	Muscle	No
SRX17655872	32474465	Paired	0.980736	0.0214742	FR	79.19	Muscle	No
SRX17655832	37052224	Paired	0.973815	0.0271861	FR	88.36	Muscle	No
SRX17655873	35809298	Paired	0.979387	0.0226087	FR	86.6	Muscle	No
SRX17655833	30942665	Paired	0.972183	0.0266421	FR	87.69	Muscle	No
SRX17655874	31851138	Paired	0.978029	0.0238576	FR	88.5	Muscle	No
SRX17655834	42401210	Paired	0.978876	0.0228417	FR	88.95	Muscle	No
SRX17655875	32039214	Paired	0.97883	0.0232727	FR	88.19	Muscle	No
SRX17655835	37778532	Paired	0.973724	0.0262391	FR	87.32	Muscle	No
SRX17655876	54912941	Paired	0.979394	0.0232562	FR	85.77	Muscle	No
SRX17655877	34627186	Paired	0.979974	0.0216725	FR	80.07	Muscle	No
SRX17655836	33266350	Paired	0.972866	0.0268986	FR	88.38	Muscle	No
SRX17655878	49931561	Paired	0.97856	0.0232247	FR	80.78	Muscle	No
SRX17655837	34261955	Paired	0.975146	0.025377	FR	88.3	Muscle	No
SRX19272721	38892612	Paired	0.0223104	0.977609	RF	88.99	Heart	No
SRX17655879	43330878	Paired	0.974642	0.0247588	FR	83.76	Muscle	No
SRX17655838	64933184	Paired	0.97916	0.0231956	FR	80.48	Muscle	No
SRX19272722	37209041	Paired	0.0213571	0.978484	RF	89.3	Heart	No
SRX17655839	41260846	Paired	0.965409	0.0349129	FR	83.08	Muscle	No
SRX19272723	38298941	Paired	0.0215339	0.978449	RF	88.74	Heart	No
SRX19272724	38291912	Paired	0.0228903	0.977481	RF	89.57	Heart	No
SRX19272725	37624029	Paired	0.0209785	0.979351	RF	89.91	Heart	No
SRX19272726	35903682	Paired	0.0209473	0.979378	RF	90.03	Heart	No
SRX19272727	37602332	Paired	0.0189983	0.981294	RF	89.63	Heart	No
SRX19272728	34895175	Paired	0.0217017	0.978906	RF	89.13	Heart	No
SRX17655950	35456381	Paired	0.97485	0.0253117	FR	81.81	Muscle	No
SRX17655951	34883292	Paired	0.977663	0.0248232	FR	79.53	Muscle	No
SRX17655910	91853028	Paired	0.978967	0.0217034	FR	83.96	Muscle	No
SRX17655952	58863369	Paired	0.980025	0.0218572	FR	81.43	Muscle	No
SRX17655911	33095737	Paired	0.97684	0.0244411	FR	83.06	Muscle	No
SRX17655953	46900589	Paired	0.973315	0.0255232	FR	81.87	Muscle	No
SRX17655912	36907678	Paired	0.977547	0.0232579	FR	83.14	Muscle	No
SRX17655913	24927229	Paired	0.0196923	0.982269	RF	90.6	Muscle	No
SRX17655914	26266160	Paired	0.0184864	0.983562	RF	91.4	Muscle	No
SRX17655915	36150014	Paired	0.971988	0.0246564	FR	81.98	Muscle	No
SRX17655916	66070065	Paired	0.978696	0.0219289	FR	83.28	Muscle	No
SRX17655917	30557143	Paired	0.975125	0.0256986	FR	78.25	Muscle	No
SRX17655918	35885973	Paired	0.018238	0.983854	RF	91.65	Muscle	No
SRX17655919	28411321	Paired	0.974605	0.0237314	FR	83.75	Muscle	No
SRX19857290	34494761	Paired	0.0431621	0.937832	RF	60.49	Cartilage	No
SRX19857250	33212373	Paired	0.0621215	0.935958	RF	75.79	Synovial membrane	No
SRX19857291	34231714	Paired	0.0494437	0.93932	RF	68.65	Cartilage	No
SRX19857251	20773676	Paired	0.0637605	0.934263	RF	72.93	Synovial membrane	No
SRX19857292	48112049	Paired	0.0444671	0.935943	RF	69.9	Cartilage	No
SRX19857252	27051877	Paired	0.070059	0.927079	RF	72.51	Synovial membrane	No
SRX19857293	30000865	Paired	0.0499584	0.9406	RF	72.68	Cartilage	No
SRX19857294	36724675	Paired	0.0504023	0.937596	RF	69.48	Cartilage	No
SRX19857254	26989541	Paired	0.0693629	0.928828	RF	71.96	Synovial membrane	No
SRX19857295	29729490	Paired	0.0478761	0.947184	RF	74.53	Cartilage	No
SRX19857296	33377886	Paired	0.0503106	0.9351	RF	71.19	Cartilage	No
SRX19857297	32708409	Paired	0.041367	0.949542	RF	66.39	Cartilage	No
SRX19857256	32974839	Paired	0.0554843	0.942155	RF	70.21	Synovial membrane	No
SRX19857298	45953105	Paired	0.0552389	0.930039	RF	69.12	Cartilage	No
SRX19857257	33141953	Paired	0.0506701	0.947129	RF	74.53	Synovial membrane	No
SRX19857299	30614932	Paired	0.0715647	0.919769	RF	70.61	Cartilage	No
SRX19857258	40521454	Paired	0.0548135	0.943966	RF	83.57	Synovial membrane	No

SRX19857259	44809406	Paired	0.0525923	0.945653	RF	71.31	Synovial membrane	No
SRX20062566	44651012	Paired	0.076365	0.882703	RF	82.7	Heart	No
SRX20062567	43387404	Paired	0.101629	0.864124	RF	79.09	Heart	No
SRX20062568	38012998	Paired	0.0914677	0.88112	RF	80.72	Heart	No
SRX20062569	39078871	Paired	0.0458232	0.938595	RF	81.7	Heart	No
SRX17655880	37866140	Paired	0.961353	0.0371587	FR	81.76	Muscle	No
SRX17655881	39355782	Paired	0.972725	0.0258746	FR	88	Muscle	No
SRX17655840	35835501	Paired	0.96262	0.0375605	FR	80.76	Muscle	No
SRX17655882	31167624	Paired	0.977912	0.0239259	FR	86.36	Muscle	No
SRX17655841	36041875	Paired	0.976258	0.0237772	FR	88.95	Muscle	No
SRX17655883	57931357	Paired	0.980412	0.0217331	FR	85.77	Muscle	No
SRX17655842	34814719	Paired	0.97311	0.0257732	FR	87.71	Muscle	No
SRX17655884	34436869	Paired	0.972842	0.0270351	FR	88.31	Muscle	No
SRX17655843	32207807	Paired	0.968394	0.0322587	FR	79.38	Muscle	No
SRX17655885	33732295	Paired	0.973649	0.0263623	FR	86.5	Muscle	No
SRX17655844	37405628	Paired	0.964339	0.0351661	FR	80.01	Muscle	No
SRX17655886	35504983	Paired	0.973631	0.0247222	FR	88.21	Muscle	No
SRX17655845	34957913	Paired	0.977332	0.0228286	FR	87.85	Muscle	No
SRX17655887	21250632	Paired	0.956711	0.042708	FR	82.49	Muscle	No
SRX17655846	31717135	Paired	0.966617	0.0342585	FR	79.73	Muscle	No
SRX17655888	38610500	Paired	0.972751	0.0274791	FR	88.23	Muscle	No
SRX17655847	36653895	Paired	0.977089	0.0237487	FR	88.69	Muscle	No
SRX17655889	44582642	Paired	0.96558	0.0345266	FR	80.49	Muscle	No
SRX17655848	34330037	Paired	0.973535	0.0274158	FR	88.73	Muscle	No
SRX17655849	27145691	Paired	0.0190177	0.983238	RF	84.53	Muscle	No
SRX7823401	25763854	Paired	0.0247562	0.977276	RF	85.7	Placenta	No
SRX7823402	17802187	Paired	0.0267133	0.974943	RF	86.18	Placenta	No
SRX7823403	18443480	Paired	0.0246551	0.97706	RF	88.81	Placenta	No
SRX7823404	23081625	Paired	0.0266953	0.974832	RF	87.38	Placenta	No
SRX20062603	28169309	Paired	0.0736642	0.89874	RF	77.05	Muscle	No
SRX7823405	33700319	Paired	0.0333962	0.968164	RF	88.83	Placenta	No
SRX7823406	33175928	Paired	0.0268504	0.97415	RF	89.86	Placenta	No
SRX20062605	45257412	Paired	0.0361993	0.940128	RF	80.28	Muscle	No
SRX7823407	31399524	Paired	0.0348194	0.966616	RF	89.05	Placenta	No
SRX20062606	31704215	Paired	0.055096	0.9261	RF	75.59	Muscle	No
SRX7823408	34571346	Paired	0.036403	0.965826	RF	88.73	Placenta	No
SRX20062607	45708788	Paired	0.0534149	0.921517	RF	84.07	Muscle	No
SRX7823409	30267975	Paired	0.0403109	0.961345	RF	89.77	Placenta	No
SRX20062608	40905389	Paired	0.0636684	0.915003	RF	77.54	Muscle	No
SRX20062609	37342635	Paired	0.0706569	0.903413	RF	77.5	Muscle	No
SRX17655920	77793789	Paired	0.978136	0.0225389	FR	87.46	Muscle	No
SRX17655921	24397246	Paired	0.0206741	0.981177	RF	90.77	Muscle	No
SRX17655922	37428163	Paired	0.973288	0.0246753	FR	80.33	Muscle	No
SRX17655923	70894177	Paired	0.976456	0.0229009	FR	86.55	Muscle	No
SRX17655924	50572220	Paired	0.977295	0.0226999	FR	81.06	Muscle	No
SRX17655925	29546722	Paired	0.97439	0.0258939	FR	82.75	Muscle	No
SRX17655926	37743183	Paired	0.974378	0.023872	FR	81.78	Muscle	No
SRX17655927	35152878	Paired	0.973447	0.0242733	FR	84.89	Muscle	No
SRX17655928	27518733	Paired	0.018794	0.983603	RF	90.2	Muscle	No
SRX17655929	33867776	Paired	0.972229	0.0277523	FR	87.72	Muscle	No
SRX17287890	48609836	Paired	0.0521344	0.948282	RF	85.27	Spleen	No
SRX19348781	18309912	Paired	0.0248771	0.975595	RF	71.26	Small intestine	No
SRX19348782	18935756	Paired	0.0248118	0.975726	RF	72.68	Small intestine	No
SRX19348783	19273859	Paired	0.0228949	0.97761	RF	74.27	Small intestine	No
SRX19348784	20055158	Paired	0.0271402	0.973467	RF	68.28	Small intestine	No
SRX19348785	18626637	Paired	0.0229584	0.977659	RF	75.1	Small intestine	No
SRX19348786	20797945	Paired	0.0247322	0.975597	RF	72.75	Small intestine	No
SRX19857260	41643866	Paired	0.0493865	0.949186	RF	73.7	Synovial membrane	No

SRX19857261	46224206	Paired	0.0665444	0.930494	RF	76.05		Synovial membrane	No
SRX19857220	38788640	Paired	0.0510131	0.941815	RF	69.48		Cartilage	No
SRX19857262	38765688	Paired	0.0486012	0.94093	RF	69.82		Cartilage	No
SRX19857221	30855077	Paired	0.0510519	0.939933	RF	71.34		Cartilage	No
SRX19857263	33061648	Paired	0.0523122	0.926552	RF	65.53		Cartilage	No
SRX19857222	40543289	Paired	0.046712	0.935791	RF	69.37		Cartilage	No
SRX19857264	19754240	Paired	0.0506698	0.92855	RF	66.2		Cartilage	No
SRX19857223	31362607	Paired	0.0504115	0.934342	RF	66.76		Cartilage	No
SRX19857265	45743378	Paired	0.0504545	0.939063	RF	69.44		Cartilage	No
SRX19857224	23422881	Paired	0.0841212	0.912812	RF	69.26		Synovial membrane	No
SRX19857266	39354452	Paired	0.0556279	0.936791	RF	71.83		Cartilage	No
SRX19857225	28020273	Paired	0.0547928	0.944014	RF	67.32		Synovial membrane	No
SRX19857267	36808781	Paired	0.0586504	0.928919	RF	67.92		Cartilage	No
SRX19857226	28691744	Paired	0.0608834	0.937303	RF	72.8		Synovial membrane	No
SRX19857268	37149165	Paired	0.0607397	0.926156	RF	71.41		Cartilage	No
SRX19857227	54615537	Paired	0.0476502	0.951559	RF	66.49		Synovial membrane	No
SRX19857269	37907647	Paired	0.0410859	0.931005	RF	62.86		Cartilage	No
SRX19857228	25553995	Paired	0.0539208	0.944915	RF	74.22		Synovial membrane	No
SRX19857229	24354498	Paired	0.0673876	0.93091	RF	80.7		Synovial membrane	No
SRX10509820	45423689	Paired	0.0438585	0.959948	RF	91.7		Spleen	No
SRX10509821	35859242	Paired	0.0430251	0.956173	RF	92.73		Spleen	No
SRX10509822	47192731	Paired	0.0418314	0.95756	RF	92.51		Spleen	No
SRX19857300	35347904	Paired	0.0519803	0.932916	RF	70.04		Cartilage	No
SRX19857301	32021683	Paired	0.0460409	0.941271	RF	70.01		Cartilage	No
SRX19857302	30767553	Paired	0.0578812	0.940329	RF	72.11		Synovial membrane	No
SRX19857303	33436944	Paired	0.0584747	0.939507	RF	73.33		Synovial membrane	No
SRX19857304	43296004	Paired	0.0533246	0.945181	RF	71.17		Synovial membrane	No
SRX19857305	41749354	Paired	0.0677921	0.929324	RF	77.53		Synovial membrane	No
SRX19857306	37350745	Paired	0.0634772	0.934216	RF	74.97		Synovial membrane	No
SRX19857307	40659143	Paired	0.0437463	0.95435	RF	62.29		Synovial membrane	No
SRX19857308	38504581	Paired	0.0507534	0.947661	RF	79.12		Synovial membrane	No
SRX19857309	32007522	Paired	0.0682808	0.928774	RF	70.67		Synovial membrane	No
SRX20062570	36571197	Paired	0.0636037	0.914668	RF	75.52		Heart	No
SRX20062571	45571309	Paired	0.0678159	0.906849	RF	77.58		Heart	No
SRX20062572	37730749	Paired	0.105298	0.888362	RF	78.47		Kidney	No
SRX20062573	34712804	Paired	0.164963	0.802008	RF	69.5		Kidney	No
SRX20062574	39080703	Paired	0.132961	0.815337	RF	66.52		Kidney	No
SRX20062575	44372104	Paired	0.0738777	0.904505	RF	85.65		Kidney	No
SRX20062576	44583945	Paired	0.0687955	0.906955	RF	86.32		Kidney	No
SRX20062577	45101699	Paired	0.0591644	0.918864	RF	85.49		Kidney	No
SRX20062578	39519281	Paired	0.0600917	0.92242	RF	86.42		Kidney	No
SRX20062579	32210031	Paired	0.0945093	0.878304	RF	78.03		Kidney	No
SRX20062539	33818859	Paired	0.109518	0.854923	RF	68.78		Heart	No
SRX17655890	73980200	Paired	0.976077	0.0244524	FR	82.38		Muscle	No
SRX17655891	37933732	Paired	0.972801	0.0269288	FR	75.02		Muscle	No
SRX17655850	36324279	Paired	0.976269	0.0249494	FR	88.9		Muscle	No
SRX17655892	29598888	Paired	0.975406	0.0233903	FR	81.32		Muscle	No
SRX17655851	40599625	Paired	0.975653	0.0250411	FR	83.9		Muscle	No
SRX17655893	36318309	Paired	0.966767	0.0337619	FR	76.31		Muscle	No
SRX17655852	44412736	Paired	0.980751	0.0210504	FR	88.2		Muscle	No
SRX17655894	29583263	Paired	0.0191648	0.983036	RF	90.9		Muscle	No
SRX17655853	31630514	Paired	0.97142	0.0282546	FR	87.89		Muscle	No
SRX17655895	63256195	Paired	0.978014	0.0231251	FR	87.92		Muscle	No
SRX17655854	33664256	Paired	0.965254	0.0341275	FR	79.43		Muscle	No
SRX17655896	35130451	Paired	0.972024	0.0266986	FR	79.01		Muscle	No
SRX17655855	33269609	Paired	0.972057	0.0261994	FR	87.28		Muscle	No
SRX17655897	43573292	Paired	0.975442	0.0260117	FR	82.24		Muscle	No
SRX17655856	31746554	Paired	0.969708	0.0275046	FR	86.89		Muscle	No

SRX17655898	66584182	Paired	0.98012	0.0213805	FR	85.34	Muscle	No
SRX17655857	42229717	Paired	0.976093	0.0245629	FR	86.88	Muscle	No
SRX17655899	41899123	Paired	0.976468	0.0228052	FR	78.31	Muscle	No
SRX17655858	37673428	Paired	0.974185	0.0250357	FR	88.8	Muscle	No
SRX17655859	83458385	Paired	0.977498	0.022673	FR	84.55	Muscle	No
SRX7823410	32274733	Paired	0.0365247	0.964723	RF	88.65	Placenta	No
SRX7823411	19879108	Paired	0.0275991	0.974816	RF	86.02	Placenta	No
SRX20062610	33262454	Paired	0.0676799	0.911878	RF	74.61	Muscle	No
SRX7823412	28727230	Paired	0.0438242	0.959076	RF	86.14	Placenta	No
SRX20062611	45894284	Paired	0.0980514	0.879358	RF	81.12	Spleen	No
SRX7823413	27823742	Paired	0.0382862	0.96479	RF	88.52	Placenta	No
SRX20062612	38214508	Paired	0.0794675	0.904296	RF	87.62	Spleen	No
SRX7823414	34249085	Paired	0.0317815	0.970416	RF	88.96	Placenta	No
SRX20062613	31521127	Paired	0.094781	0.885936	RF	81.35	Spleen	No
SRX7823415	31848744	Paired	0.0439945	0.959789	RF	89.05	Placenta	No
SRX20062614	34412868	Paired	0.129544	0.849723	RF	80.71	Spleen	No
SRX7823416	36812344	Paired	0.0386377	0.964449	RF	89.01	Placenta	No
SRX20062615	40971071	Paired	0.0919809	0.872835	RF	74.52	Spleen	No
SRX7823417	37278345	Paired	0.0366042	0.966286	RF	89.29	Placenta	No
SRX20062616	42774518	Paired	0.089375	0.892712	RF	83.56	Spleen	No
SRX7823418	16444312	Paired	0.0249271	0.976597	RF	87.53	Placenta	No
SRX20062617	32854693	Paired	0.0730372	0.901753	RF	76.89	Spleen	No
SRX20062618	39165855	Paired	0.0424482	0.945287	RF	83.13	Spleen	No
SRX20062619	36101090	Paired	0.0731624	0.90244	RF	76.5	Spleen	No
SRX17655930	34367987	Paired	0.975144	0.0256495	FR	81.93	Muscle	No
SRX17655931	37427581	Paired	0.974557	0.0244752	FR	87.5	Muscle	No
SRX17655932	41333530	Paired	0.970707	0.0273211	FR	81.24	Muscle	No
SRX17655933	30609477	Paired	0.972251	0.0268658	FR	84.11	Muscle	No
SRX17655934	25652246	Paired	0.0201044	0.983373	RF	90.73	Muscle	No
SRX17655935	33366921	Paired	0.966747	0.0298101	FR	85.28	Muscle	No
SRX17655936	28112885	Paired	0.0187748	0.983368	RF	91.31	Muscle	No
SRX17655937	40128349	Paired	0.974019	0.0270372	FR	83.01	Muscle	No
SRX17655938	40965448	Paired	0.974794	0.0257338	FR	82.88	Muscle	No
SRX17655939	43645815	Paired	0.972703	0.0251974	FR	82.71	Muscle	No
SRX19857270	31677944	Paired	0.0557063	0.931712	RF	64.41	Cartilage	No
SRX19857230	25632910	Paired	0.060341	0.938492	RF	73.4	Synovial membrane	No
SRX19857271	37640861	Paired	0.0583508	0.930746	RF	68.9	Cartilage	No
SRX19857231	30774385	Paired	0.0483515	0.949883	RF	63.88	Synovial membrane	No
SRX19857272	30532758	Paired	0.0503802	0.931744	RF	69.73	Cartilage	No
SRX19857232	25155010	Paired	0.0533059	0.943442	RF	71.63	Synovial membrane	No
SRX19857273	38641215	Paired	0.0566614	0.934036	RF	72.22	Cartilage	No
SRX19857233	30268223	Paired	0.0720374	0.924757	RF	71.91	Synovial membrane	No
SRX19857274	37129931	Paired	0.0516586	0.936431	RF	70.59	Cartilage	No
SRX19857234	30586890	Paired	0.0417018	0.956279	RF	72.16	Cartilage	No
SRX19857275	50498432	Paired	0.0565372	0.935135	RF	72.15	Cartilage	No
SRX19857235	35881975	Paired	0.0557292	0.941921	RF	73.32	Cartilage	No
SRX19857276	32227948	Paired	0.0465178	0.952912	RF	73.87	Synovial membrane	No
SRX19857236	37908774	Paired	0.0604311	0.937171	RF	83	Cartilage	No
SRX19857237	52688086	Paired	0.0568283	0.941037	RF	73.75	Cartilage	No
SRX19857278	35923123	Paired	0.0532058	0.944367	RF	69.84	Synovial membrane	No
SRX19857238	27459734	Paired	0.0557956	0.94203	RF	73.15	Cartilage	No
SRX19857279	34331346	Paired	0.0642433	0.932989	RF	73.98	Synovial membrane	No
SRX19857239	28203749	Paired	0.0518325	0.946381	RF	82.26	Cartilage	No
SRX9610840	21670954	Paired	0.0402187	0.956109	RF	81.98	Artery	No
SRX9610841	19862792	Paired	0.0475951	0.949057	RF	75.03	Artery	No
SRX9610842	17013475	Paired	0.0415277	0.955002	RF	67.56	Artery	No
SRX9610843	20766419	Paired	0.034041	0.963449	RF	80.11	Artery	No
SRX9610844	21520665	Paired	0.046549	0.949102	RF	80.07	Artery	No

SRX9610845	21549880	Paired	0.0427826	0.953194	RF	81.96	Artery	No
SRX9610846	21977457	Paired	0.0450399	0.951815	RF	84.86	Artery	No
SRX9610847	22581882	Paired	0.0384313	0.958595	RF	81.14	Artery	No
SRX9610848	20396368	Paired	0.0411151	0.95555	RF	81.79	Artery	No
SRX9610849	19479414	Paired	0.0379363	0.959103	RF	78.43	Artery	No
ERX3462600	19715479	Single	0.0197068	NULL	RF	91.82	Liver	No
ERX3462601	19204594	Single	0.0180596	NULL	RF	91.36	Liver	No
SRX3733871	43568258	Paired	0.0176026	0.985918	RF	91.99	Muscle	No
SRX3733872	45571675	Paired	0.982195	0.0212393	FR	88.9	Muscle	No
SRX3733873	45166817	Paired	0.985247	0.0169887	FR	88.17	Muscle	No
SRX3733874	45723237	Paired	0.984699	0.0185148	FR	87.16	Muscle	No
SRX3733875	29727828	Paired	0.0176775	0.984924	RF	90.18	Muscle	No
SRX3733876	50488329	Paired	0.0181819	0.984439	RF	87.27	Muscle	No
SRX3733877	22465715	Paired	0.0200878	0.982735	RF	90.03	Muscle	No
SRX3733878	60837618	Paired	0.986291	0.0166266	FR	89.78	Muscle	No
SRX3733879	44456747	Paired	0.9818	0.0207645	FR	86.86	Muscle	No
SRX9610850	20280574	Paired	0.0387367	0.958605	RF	77.92	Artery	No
SRX9610851	21401299	Paired	0.037762	0.958179	RF	82.67	Artery	No
SRX9610852	24733869	Paired	0.0378037	0.959395	RF	83.88	Artery	No
SRX5446266	26932293	Paired	0.0213131	0.979638	RF	88.71	Large intestine	No
SRX9610853	20631768	Paired	0.0352181	0.961544	RF	81.08	Artery	No
SRX5446267	28882886	Paired	0.0205454	0.980383	RF	88.26	Large intestine	No
SRX9610854	19823132	Paired	0.0486649	0.947636	RF	79.29	Artery	No
SRX5446268	28277780	Paired	0.0215459	0.979627	RF	87.98	Large intestine	No
SRX5446269	25476934	Paired	0.0206582	0.9802	RF	86.22	Large intestine	No
SRX3733880	56548258	Paired	0.0212382	0.981803	RF	85.46	Muscle	No
SRX3428331	17928728	Paired	0.021611	0.978985	RF	87.66	Small intestine	No
SRX3733881	50582270	Paired	0.0196232	0.982537	RF	82.13	Muscle	No
SRX3428332	19104937	Paired	0.0214349	0.97943	RF	87.47	Small intestine	No
SRX3733882	19143534	Paired	0.0189186	0.983659	RF	89.87	Muscle	No
SRX3428333	23178234	Paired	0.0204682	0.980468	RF	87.71	Small intestine	No
SRX3733883	33031277	Paired	0.983535	0.0192382	FR	89.44	Muscle	No
SRX3428334	26760627	Paired	0.0203401	0.980523	RF	85.36	Small intestine	No
SRX3733884	48000289	Paired	0.982667	0.0203947	FR	88.83	Muscle	No
SRX3428335	26119882	Paired	0.0203913	0.979593	RF	85.84	Small intestine	No
SRX3428336	21316648	Paired	0.023255	0.977798	RF	86.28	Small intestine	No
SRX3428337	19024989	Paired	0.0214847	0.979417	RF	83.59	Small intestine	No
SRX3428338	26923099	Paired	0.021215	0.980166	RF	85.07	Small intestine	No
SRX3428339	22210532	Paired	0.0212917	0.979113	RF	85.79	Small intestine	No
SRX5446270	24584079	Paired	0.0210908	0.979809	RF	87.66	Large intestine	No
SRX5446271	22442913	Paired	0.0213092	0.9795	RF	87.19	Large intestine	No
SRX5446272	19532853	Paired	0.023296	0.977739	RF	88.13	Large intestine	No
SRX5446273	22638300	Paired	0.0218743	0.97898	RF	88.82	Large intestine	No
SRX5446274	23030422	Paired	0.02145	0.979366	RF	89.48	Large intestine	No
SRX5446275	24558983	Paired	0.0208707	0.980078	RF	88.55	Large intestine	No
SRX5446276	25941528	Paired	0.0189395	0.981852	RF	86.87	Large intestine	No
SRX5446277	23621260	Paired	0.0220473	0.97862	RF	87.93	Large intestine	No
SRX5446278	19428351	Paired	0.0220857	0.978759	RF	87.58	Large intestine	No
SRX5446279	29369586	Paired	0.0199574	0.980467	RF	84.75	Large intestine	No
SRX3428340	23047109	Paired	0.022383	0.978531	RF	84.89	Small intestine	No
SRX3428341	22556893	Paired	0.0188832	0.981793	RF	86.97	Small intestine	No
SRX3428342	25776244	Paired	0.0189303	0.981991	RF	85.45	Small intestine	No
SRX2563700	41742018	Paired	0.0158904	0.984976	RF	89.71	Ovary	No
SRX5446280	27194545	Paired	0.0217356	0.979282	RF	87.91	Large intestine	No
SRX5446281	20679981	Paired	0.0207501	0.980223	RF	87.09	Large intestine	No
SRX5035691	12745450	Paired	0.0616498	0.937054	RF	63.04	Small intestine	No
SRX5035692	21035585	Paired	0.0321382	0.96973	RF	90.94	Small intestine	No
SRX5035693	21635307	Paired	0.0415094	0.963366	RF	87.99	Small intestine	No

SRX5035694	18054888	Paired	0.033012	0.969092	RF	91.07	Small intestine	No
SRX5035695	19133367	Paired	0.0332598	0.968821	RF	90.22	Small intestine	No
SRX5035696	22457908	Paired	0.0306415	0.970973	RF	91.66	Small intestine	No
SRX5035697	20513220	Paired	0.0328612	0.968946	RF	91.12	Small intestine	No
SRX5035698	17703298	Paired	0.0325233	0.969522	RF	89.72	Small intestine	No
SRX5035699	16291992	Paired	0.0329409	0.968422	RF	91.23	Small intestine	No
SRX1817148	10954449	Single	0.0261475	NULL	RF	91.63	Liver	No
SRX1817149	8780439	Single	0.0311862	NULL	RF	93	Liver	No
SRX1817150	8283311	Single	0.0345818	NULL	RF	92.34	Liver	No
SRX1817151	6963445	Single	0.0299943	NULL	RF	92.55	Liver	No
SRX1817152	8101345	Single	0.0286649	NULL	RF	93.1	Liver	No
SRX1817153	6968768	Single	0.0285369	NULL	RF	92.47	Liver	No
SRX4645767	126427593	Paired	0.0187827	0.981876	RF	83.82	Placenta	No
SRX4645768	121851646	Paired	0.0174136	0.984251	RF	88.35	Placenta	No
SRX4870082	30697732	Paired	0.0748947	0.924021	RF	90.14	Ovary	No
SRX4870083	34547680	Paired	0.045025	0.955126	RF	89.75	Ovary	No
SRX4870084	36358417	Paired	0.100781	0.896242	RF	89.85	Ovary	No
SRX4870085	30120871	Paired	0.124593	0.872345	RF	77.05	Ovary	No
SRX4870086	33933938	Paired	0.0578633	0.942292	RF	90.01	Ovary	No
SRX4870087	28442164	Paired	0.0390498	0.961171	RF	87.56	Ovary	No
SRX1522249	4347469	Single	0.931819	NULL	FR	88.66	Testis	No
SRX1522250	2740494	Single	0.920904	NULL	FR	87.96	Testis	No
SRX1522251	4186661	Single	0.933011	NULL	FR	88.98	Testis	No
SRX1522255	2359536	Single	0.915886	NULL	FR	90.14	Testis	No
SRX1522256	2302523	Single	0.888605	NULL	FR	86.42	Testis	No
SRX1522257	3238368	Single	0.91489	NULL	FR	88.25	Testis	No
SRX2327155	53294314	Paired	0.0303072	0.966202	RF	78.51	Macrophage	No
SRX4381383	69453850	Paired	0.0314794	0.969211	RF	86.05	Muscle	No
SRX4381384	59623669	Paired	0.0351289	0.963454	RF	76.56	Muscle	No
SRX4381385	67922603	Paired	0.0327776	0.967881	RF	80.28	Muscle	No
SRX4381386	80388544	Paired	0.0322715	0.967755	RF	86.61	Muscle	No
SRX4381387	52301566	Paired	0.0352604	0.964732	RF	81.56	Muscle	No
SRX4381388	76895782	Paired	0.0312382	0.96838	RF	83.93	Muscle	No
SRX4381389	64616960	Paired	0.0319175	0.967725	RF	79.17	Muscle	No
SRX4381390	67964970	Paired	0.0277188	0.972943	RF	88.38	Muscle	No
SRX4381391	58170217	Paired	0.0316988	0.968675	RF	84.91	Muscle	No
SRX4381392	71017309	Paired	0.0298288	0.970748	RF	87.37	Muscle	No
SRX4381393	59142199	Paired	0.0302903	0.970641	RF	87.79	Muscle	No
SRX4381394	78105347	Paired	0.0323297	0.967951	RF	87.66	Muscle	No
SRX8548919	35853490	Paired	0.0198828	0.98225	RF	86.91	Fibroblast	No
SRX2327191	53030161	Paired	0.0287871	0.967548	RF	68.49	Macrophage	No
SRX2327192	53098973	Paired	0.0291845	0.966914	RF	78.34	Macrophage	No
SRX2327193	53477189	Paired	0.0301716	0.965944	RF	68.41	Macrophage	No
SRX2327194	53296548	Paired	0.026343	0.969894	RF	71.81	Macrophage	No
SRX2327195	53253837	Paired	0.0279487	0.968678	RF	80.22	Macrophage	No
SRX2327196	53632997	Paired	0.0289143	0.967835	RF	77.79	Macrophage	No
SRX2327197	52877831	Paired	0.0271258	0.970236	RF	79.11	Macrophage	No
SRX4633123	70156587	Paired	0.050415	0.949036	RF	85.01	Testis	No
SRX4633124	46892909	Paired	0.0213276	0.980554	RF	79.62	Spleen	No
SRX4633125	47069056	Paired	0.0323053	0.97071	RF	84.3	Spleen	No
SRX4633128	50917536	Paired	0.0549327	0.945402	RF	90.43	Spleen	No
SRX9703863	43712719	Paired	0.0412851	0.958374	RF	83.1	Liver	No
SRX4633129	46629094	Paired	0.0689647	0.930725	RF	90.92	Spleen	No
SRX9703864	42057673	Paired	0.0378029	0.961438	RF	81.95	Liver	No
SRX9703865	38812489	Paired	0.045146	0.952554	RF	83.91	Liver	No
SRX9703866	38774814	Paired	0.0453824	0.94655	RF	83.74	Liver	No
SRX9703867	38158469	Paired	0.0368183	0.96039	RF	87.44	Liver	No
SRX9703868	44197941	Paired	0.041417	0.953334	RF	86.47	Liver	No

SRX9703869	44878071	Paired	0.0377916	0.960797	RF	86.79	Liver	No
SRX2863100	13577053	Paired	0.982063	0.0204348	FR	89.52	Muscle	No
SRX2863101	46469425	Paired	0.0171748	0.98575	RF	90.64	Muscle	No
SRX2863102	49756775	Paired	0.985389	0.016883	FR	87.44	Muscle	No
SRX4633130	38969416	Paired	0.0422421	0.958953	RF	90.76	Spleen	No
SRX2863103	5614916	Paired	0.0282993	0.973443	RF	81.61	Muscle	No
SRX4633131	49152250	Paired	0.0307935	0.976325	RF	77.79	Spleen	No
SRX2863104	52043974	Paired	0.0160764	0.986115	RF	89.41	Muscle	No
SRX4633132	56449135	Paired	0.0366972	0.962433	RF	84.45	Testis	No
SRX2408800	15697355	Paired	0.988358	0.0142748	FR	62.96	Muscle	No
SRX2863105	47141940	Paired	0.984409	0.0182304	FR	90.16	Muscle	No
SRX4633133	54569707	Paired	0.0354674	0.963894	RF	84.99	Testis	No
SRX2408801	15196538	Paired	0.988559	0.0143106	FR	62.48	Muscle	No
SRX2863106	43588738	Paired	0.0169003	0.985761	RF	89.77	Muscle	No
SRX2408802	22855911	Paired	0.99079	0.0108707	FR	66.97	Muscle	No
SRX2863107	56695715	Paired	0.0250657	0.978218	RF	85.43	Muscle	No
SRX2408803	17855295	Paired	0.989255	0.0127654	FR	62.4	Muscle	No
SRX2863108	41954982	Paired	0.979918	0.0235411	FR	90.38	Muscle	No
SRX9703870	40278672	Paired	0.0449685	0.951648	RF	87.06	Liver	No
SRX2863109	38427797	Paired	0.985111	0.0174628	FR	89.13	Muscle	No
SRX9703871	43365630	Paired	0.0337753	0.966706	RF	89.74	Liver	No
SRX9703872	38378002	Paired	0.0408222	0.958462	RF	81.69	Liver	No
SRX9703873	52620096	Paired	0.0293213	0.970933	RF	91.28	Liver	No
SRX9703874	51777694	Paired	0.0297111	0.969916	RF	91.01	Liver	No
SRX9703875	42466974	Paired	0.0282891	0.971588	RF	91.34	Liver	No
SRX9703876	36882357	Paired	0.029289	0.970251	RF	90.67	Liver	No
SRX9703877	50924193	Paired	0.0307642	0.96679	RF	90.29	Liver	No
SRX9703878	30324437	Paired	0.041432	0.954551	RF	87.91	Liver	No
SRX9703879	46253391	Paired	0.0344411	0.963683	RF	86.72	Liver	No
ERX2680016	111900507	Paired	0.0298983	0.971163	RF	89.94	T cell	No
ERX2680018	109608912	Paired	0.0262508	0.975849	RF	91.01	T cell	No
ERX2680019	221528568	Paired	0.0280288	0.973268	RF	90.77	T cell	No
SRX9274500	32708154	Paired	0.0317917	0.968475	RF	82.67	Adipose	No
SRX9274501	35203596	Paired	0.0420239	0.958986	RF	85.61	Adipose	No
SRX9274502	32730212	Paired	0.0394116	0.961082	RF	84.65	Adipose	No
SRX9274504	34172874	Paired	0.0340695	0.967755	RF	86.02	Muscle	No
SRX9274505	32211558	Paired	0.0329119	0.969338	RF	85.74	Muscle	No
SRX5230414	67836465	Single	0.0840587	NULL	RF	85.74	IPEC-J2	No
SRX5230415	49920815	Single	0.0801014	NULL	RF	86.02	IPEC-J2	No
SRX5230416	47279490	Single	0.0761302	NULL	RF	83.58	IPEC-J2	No
SRX5230417	22769360	Single	0.0806441	NULL	RF	81.94	IPEC-J2	No
SRX5230418	37968585	Single	0.0782934	NULL	RF	85.85	IPEC-J2	No
SRX5230419	34912775	Single	0.0730196	NULL	RF	83.72	IPEC-J2	No
SRX2863110	43115076	Paired	0.98547	0.0163605	FR	88.35	Muscle	No
SRX2863111	46987976	Paired	0.984798	0.0177471	FR	83.99	Muscle	No
SRX2863112	50660657	Paired	0.984349	0.0179567	FR	88.77	Muscle	No
SRX2863113	54803930	Paired	0.0175201	0.985043	RF	89.62	Muscle	No
SRX2863114	47415221	Paired	0.985416	0.0173462	FR	83.7	Muscle	No
SRX2863115	43778060	Paired	0.986918	0.0155193	FR	88.56	Muscle	No
SRX2863116	46394377	Paired	0.986272	0.016446	FR	89.07	Muscle	No
SRX2863117	49791233	Paired	0.985682	0.0167644	FR	88.18	Muscle	No
SRX2863118	45028329	Paired	0.0189381	0.983651	RF	88.6	Muscle	No
SRX9703880	38191729	Paired	0.0309515	0.968016	RF	83.23	Liver	No
SRX9703881	51282011	Paired	0.033329	0.966156	RF	81.05	Liver	No
SRX9703882	41606644	Paired	0.0349008	0.962488	RF	83.59	Liver	No
SRX9703883	46162759	Paired	0.0341039	0.964651	RF	87.29	Muscle	No
SRX9703884	47915910	Paired	0.032632	0.967073	RF	82.14	Muscle	No
SRX9703885	54856138	Paired	0.0336066	0.965491	RF	82.06	Muscle	No

SRX9703886	41078641	Paired	0.0374552	0.957777	RF	87.57	Muscle	No
SRX9703887	39633595	Paired	0.0340188	0.963461	RF	84.42	Muscle	No
SRX9703888	39760984	Paired	0.0394856	0.953583	RF	84.26	Muscle	No
SRX9703889	45567259	Paired	0.0367445	0.961607	RF	83.9	Muscle	No
ERX2680020	112380207	Paired	0.0291132	0.97198	RF	90.58	T cell	No
ERX2680021	112030481	Paired	0.0300751	0.970549	RF	91.04	T cell	No
ERX2680023	110635037	Paired	0.0163119	0.985955	RF	91.53	Liver	No
ERX2680024	112488463	Paired	0.0155404	0.98639	RF	90.71	Liver	No
ERX2680025	105144451	Paired	0.0155396	0.987087	RF	91.91	Liver	No
ERX2680026	109116256	Paired	0.0159727	0.986877	RF	91.35	Liver	No
SRX5230420	13790128	Single	0.105956	NULL	RF	84.78	IPEC-J2	No
SRX5230421	62727037	Single	0.0731333	NULL	RF	83.85	IPEC-J2	No
SRX5230422	11693063	Single	0.102213	NULL	RF	84.68	IPEC-J2	No
SRX5230423	32350519	Single	0.0751901	NULL	RF	84.24	IPEC-J2	No
SRX5230424	16595542	Single	0.077515	NULL	RF	83.48	IPEC-J2	No
SRX5230425	12819529	Single	0.077064	NULL	RF	84.21	IPEC-J2	No
SRX5230426	10797904	Single	0.0976019	NULL	RF	84.81	IPEC-J2	No
SRX5230427	24925038	Single	0.0975821	NULL	RF	84.47	IPEC-J2	No
SRX9703890	42599053	Paired	0.0355067	0.963115	RF	82.99	Muscle	No
SRX9703891	45549920	Paired	0.0362829	0.961023	RF	83.53	Muscle	No
SRX9703892	47058769	Paired	0.0346783	0.962859	RF	87.67	Muscle	No
SRX9703893	39763947	Paired	0.0287884	0.972615	RF	83.99	Muscle	No
SRX9703894	40256111	Paired	0.0296785	0.971192	RF	85.47	Muscle	No
SRX9703895	39914855	Paired	0.0332983	0.965403	RF	88.13	Muscle	No
SRX9703896	40658770	Paired	0.0297061	0.970638	RF	83.83	Muscle	No
SRX9703897	33672067	Paired	0.0296984	0.970966	RF	81.85	Muscle	No
SRX9703898	36233756	Paired	0.0303408	0.970563	RF	85.19	Muscle	No
SRX9703899	40048822	Paired	0.0294092	0.970861	RF	82.98	Muscle	No
ERX1958836	23885443	Paired	0.0325131	0.969138	RF	92.25	Whole blood	No
ERX1958837	18802572	Paired	0.0335361	0.968921	RF	92.04	Whole blood	No
ERX1958838	14190750	Paired	0.0360857	0.966361	RF	90.93	Whole blood	No
ERX1958839	16725852	Paired	0.0419593	0.961331	RF	90.35	Whole blood	No
SRX3373100	9208816	Paired	0.96698	0.0338566	FR	87.41	Lymph node	No
SRX3373101	10855174	Paired	0.967919	0.0328608	FR	85.95	Lymph node	No
SRX3373102	9772581	Paired	0.93945	0.0713857	FR	63.86	Whole blood	No
SRX4962217	61648346	Single	0.94439	NULL	FR	64.15	Liver	No
SRX4962218	57379365	Single	0.944379	NULL	FR	65.12	Liver	No
SRX4962219	50115873	Single	0.94144	NULL	FR	60.02	Liver	No
ERX1958840	15732426	Paired	0.0366951	0.965931	RF	91.68	Whole blood	No
ERX1958841	19587456	Paired	0.0329796	0.96867	RF	91.72	Whole blood	No
ERX1958842	17906953	Paired	0.0372365	0.964923	RF	91.02	Whole blood	No
ERX1958843	20272639	Paired	0.033806	0.967518	RF	91.81	Whole blood	No
ERX1958844	18557053	Paired	0.0286499	0.972335	RF	92.62	Whole blood	No
ERX1958845	15258550	Paired	0.0322214	0.969311	RF	91.73	Whole blood	No
ERX1958846	23055825	Paired	0.0300716	0.970852	RF	91.63	Whole blood	No
ERX1958847	18996133	Paired	0.0295076	0.972118	RF	91.63	Whole blood	No
ERX1958848	18989951	Paired	0.0321378	0.969652	RF	91.41	Whole blood	No
ERX1958849	22164040	Paired	0.0315771	0.969892	RF	92.06	Whole blood	No
SRX12132476	58264617	Paired	0.0376513	0.960103	RF	87.34	Muscle	No
SRX12132477	56239227	Paired	0.0359428	0.962475	RF	87.06	Muscle	No
SRX12132478	51815238	Paired	0.0349015	0.963993	RF	83.92	Muscle	No
SRX12132479	57539018	Paired	0.0407053	0.957474	RF	87.31	Muscle	No
SRX8610067	20445561	Paired	0.118848	0.877495	RF	63.38	Lymph node	No
SRX8912002	56407205	Paired	0.0252735	0.976246	RF	75.13	Muscle	No
SRX8912003	60834717	Paired	0.0247794	0.976865	RF	76.85	Muscle	No
SRX8912004	47955764	Paired	0.0255652	0.976105	RF	77.39	Muscle	No
SRX4962222	52934632	Single	0.951607	NULL	FR	60.33	Liver	No
SRX8912005	59515564	Paired	0.0300706	0.967577	RF	70.2	Muscle	No

SRX4962223	56964781	Single	0.946942	NULL	FR	64.13	Liver	No
SRX8912006	56928843	Paired	0.0257157	0.976423	RF	75.6	Muscle	No
SRX8912007	72552188	Paired	0.025516	0.976088	RF	73.02	Muscle	No
SRX8912008	57734115	Paired	0.0252987	0.975526	RF	73.57	Muscle	No
SRX8912009	59238048	Paired	0.0250541	0.977695	RF	75.47	Muscle	No
ERX1958850	16379282	Paired	0.0282399	0.972989	RF	92.3	Whole blood	No
ERX1958851	14354940	Paired	0.0309196	0.970309	RF	92.53	Whole blood	No
ERX1958852	18147151	Paired	0.0313752	0.969783	RF	90.34	Whole blood	No
ERX1958853	18450848	Paired	0.0271632	0.974059	RF	91.76	Whole blood	No
ERX1958854	20145798	Paired	0.0292386	0.972164	RF	90.47	Whole blood	No
ERX1958855	18286675	Paired	0.0297189	0.971092	RF	91.77	Whole blood	No
ERX1958856	22079890	Paired	0.0287062	0.972166	RF	92.02	Whole blood	No
ERX1958857	18568465	Paired	0.0300477	0.970475	RF	91.84	Whole blood	No
ERX1958858	19431646	Paired	0.0298258	0.971364	RF	91.67	Whole blood	No
ERX1958859	19328082	Paired	0.0280891	0.972749	RF	91.97	Whole blood	No
SRX12132480	59396004	Paired	0.037866	0.960514	RF	86.19	Muscle	No
SRX12132481	91264332	Paired	0.0402775	0.95775	RF	87.97	Muscle	No
SRX12132482	57809256	Paired	0.0389404	0.959108	RF	86.83	Muscle	No
SRX12132483	63420821	Paired	0.0449611	0.952705	RF	87.48	Muscle	No
SRX12132484	59399258	Paired	0.0411834	0.956515	RF	87.32	Muscle	No
SRX12132485	58229290	Paired	0.0373135	0.961264	RF	88.14	Muscle	No
SRX12132486	68325933	Paired	0.0405128	0.957184	RF	87.89	Muscle	No
SRX12132487	53971731	Paired	0.0311364	0.968114	RF	81.23	Muscle	No
SRX12132488	62253680	Paired	0.0403122	0.95755	RF	86.88	Muscle	No
SRX12132489	60695462	Paired	0.0370352	0.960799	RF	87.02	Muscle	No
SRX8610070	19081558	Paired	0.149509	0.847308	RF	63.59	Lymph node	No
SRX8610071	23413319	Paired	0.125234	0.870524	RF	63.37	Lymph node	No
SRX8610072	18904644	Paired	0.12725	0.868344	RF	65.86	Lymph node	No
SRX8912010	59701684	Paired	0.0268597	0.970032	RF	78.51	Muscle	No
SRX8912011	46853727	Paired	0.0265449	0.970237	RF	77.35	Muscle	No
SRX8912012	50389189	Paired	0.0263966	0.974258	RF	78.27	Muscle	No
SRX8912013	49901080	Paired	0.0293348	0.972386	RF	76.65	Muscle	No
ERX1958860	21225668	Paired	0.0319304	0.969642	RF	92.07	Whole blood	No
ERX1958861	23835584	Paired	0.038961	0.964287	RF	90.67	Whole blood	No
ERX1958862	17179099	Paired	0.0326258	0.968892	RF	91.66	Whole blood	No
ERX1958863	20198538	Paired	0.0332821	0.96843	RF	90.41	Whole blood	No
ERX1958864	16692043	Paired	0.0367053	0.965507	RF	91.94	Whole blood	No
ERX1958865	20397274	Paired	0.0352416	0.966468	RF	92.29	Whole blood	No
ERX1958866	18334551	Paired	0.0409606	0.962637	RF	88.54	Whole blood	No
ERX1958867	18612709	Paired	0.0330469	0.967719	RF	92.31	Whole blood	No
SRX12132490	51896496	Paired	0.0404897	0.957236	RF	84.59	Muscle	No
SRX12132491	70286396	Paired	0.0418414	0.955909	RF	87.21	Muscle	No
SRX12132492	48499960	Paired	0.0746182	0.918665	RF	83.51	Muscle	No
SRX12132493	73050281	Paired	0.0400479	0.95801	RF	87.73	Muscle	No
SRX12132494	66466491	Paired	0.0408775	0.956412	RF	85.08	Muscle	No
SRX12132495	58219605	Paired	0.0421277	0.955618	RF	87.67	Muscle	No
SRX12132496	61808633	Paired	0.040124	0.957437	RF	86.68	Muscle	No
SRX12132497	48822877	Paired	0.0393228	0.958697	RF	85.14	Muscle	No
SRX12132498	55144892	Paired	0.030401	0.969464	RF	82.69	Muscle	No
SRX12132499	82874944	Paired	0.0370325	0.96147	RF	88.12	Muscle	No
SRX3809417	77128012	Paired	0.121468	0.859153	RF	85.34	Fibroblast	No
SRX3809418	73284053	Paired	0.112128	0.873861	RF	88.94	Fibroblast	No
SRX1035351	4801875	Single	0.965985	NULL	FR	86.96	Muscle	No
SRX1035352	4001393	Single	0.963709	NULL	FR	88.15	Muscle	No
SRX1035353	5563149	Single	0.96923	NULL	FR	86.04	Muscle	No
SRX1035354	4361900	Single	0.964232	NULL	FR	86.78	Muscle	No
SRX1035355	4397647	Single	0.96692	NULL	FR	88.37	Muscle	No
SRX1035356	4541089	Single	0.966358	NULL	FR	87.61	Muscle	No

SRX1035357	4166926	Single	0.965915	NULL	FR	87.16	Muscle	No
SRX1035358	4335028	Single	0.966047	NULL	FR	85.8	Muscle	No
SRX1035359	4286655	Single	0.965636	NULL	FR	86.39	Muscle	No
SRX7051120	42152681	Paired	0.0160621	0.985766	RF	83.03	Muscle	No
SRX7051121	42940745	Paired	0.0255262	0.973783	RF	87.14	Muscle	No
SRX7051122	43110896	Paired	0.0186095	0.979666	RF	83.87	Muscle	No
SRX7051123	42564982	Paired	0.0170821	0.984112	RF	82.73	Muscle	No
SRX7051124	42848956	Paired	0.0240217	0.976118	RF	83.46	Muscle	No
SRX7051125	42264079	Paired	0.0150201	0.986239	RF	81.03	Muscle	No
SRX6415914	17748105	Paired	0.0165932	0.983811	RF	84.11	Small intestine	No
SRX6415915	16883461	Paired	0.014697	0.985494	RF	80.83	Small intestine	No
SRX6415916	20925845	Paired	0.0149911	0.98521	RF	82.33	Small intestine	No
SRX6415917	14044545	Paired	0.0166407	0.983857	RF	84.87	Small intestine	No
SRX6415918	18045189	Paired	0.0172259	0.983555	RF	85.4	Small intestine	No
SRX6415919	18429968	Paired	0.0149687	0.985417	RF	81.7	Small intestine	No
SRX3473560	24936697	Paired	0.98619	0.0190672	FR	74.51	Ovary	No
SRX3473561	24703232	Paired	0.987836	0.0155292	FR	74.56	Ovary	No
SRX6821327	28271495	Paired	0.0386499	0.965201	RF	89.18	T cell	No
SRX3808602	39199315	Paired	0.0299564	0.969117	RF	87.12	Muscle	No
SRX3808603	40608056	Paired	0.0327408	0.967683	RF	86.99	Muscle	No
SRX3808604	48922009	Paired	0.0266379	0.972648	RF	83.29	Muscle	No
SRX3808605	41949591	Paired	0.0306251	0.969548	RF	87.55	Muscle	No
SRX3808606	49332870	Paired	0.0302751	0.968836	RF	85.74	Muscle	No
SRX3808607	53092648	Paired	0.0299625	0.969358	RF	87.39	Muscle	No
SRX11555362	60580771	Paired	0.0223505	0.979452	RF	85.5	Muscle	No
SRX11555363	60765291	Paired	0.0232963	0.978981	RF	86.66	Muscle	No
SRX11555364	60656793	Paired	0.0219266	0.980556	RF	85.15	Muscle	No
SRX11555365	60664171	Paired	0.022624	0.979166	RF	87.54	Muscle	No
SRX11555366	60817250	Paired	0.024147	0.9768	RF	88.34	Muscle	No
SRX11555367	60587248	Paired	0.0253728	0.975896	RF	88.76	Muscle	No
SRX2327305	52918458	Paired	0.0287368	0.968132	RF	78	Macrophage	No
SRX2327306	53241797	Paired	0.0261101	0.971329	RF	78.94	Macrophage	No
SRX8418086	39701721	Paired	0.049997	0.973052	RF	73.14	PBMCs	No
SRX8418087	33530766	Paired	0.0432555	0.972731	RF	74.72	PBMCs	No
SRX8418088	32232857	Paired	0.045065	0.971643	RF	75.9	PBMCs	No
SRX8418089	41245328	Paired	0.0473533	0.974177	RF	71.44	PBMCs	No
SRX8363198	45812943	Paired	0.0468522	0.972224	RF	72.2	PBMCs	No
SRX8363199	44130483	Paired	0.0493975	0.970958	RF	71.86	PBMCs	No
ERX3013300	30775052	Single	0.0645086	NULL	RF	65.28	Whole blood	No
SRX7141523	23075418	Paired	0.0200475	0.980938	RF	89.43	Artery	No
SRX7141524	26736618	Paired	0.0202296	0.981044	RF	89.08	Artery	No
SRX7141525	22909841	Paired	0.0216659	0.979959	RF	88.83	Artery	No
SRX7141526	23354383	Paired	0.0182036	0.982695	RF	89.84	Artery	No
SRX7141527	22738529	Paired	0.0155181	0.985132	RF	89.19	Artery	No
SRX7141528	26859345	Paired	0.0193196	0.981071	RF	87.66	Artery	No
SRX7141529	24297416	Paired	0.0229987	0.978503	RF	87.88	Artery	No
SRX7101800	24674996	Paired	0.0358434	0.964526	RF	80.63	Liver	No
SRX7101801	15825652	Paired	0.0368368	0.963151	RF	81.86	Liver	No
SRX7101802	16589817	Paired	0.0357295	0.964808	RF	75.84	Liver	No
SRX7101803	17773131	Paired	0.0338421	0.966053	RF	76.48	Liver	No
SRX7101804	40544324	Paired	0.0404477	0.960399	RF	84.37	Liver	No
SRX7101805	11574656	Paired	0.0431262	0.956943	RF	85.52	Liver	No
SRX7101806	41633873	Paired	0.0373313	0.963574	RF	86.27	Liver	No
SRX7101807	15645581	Paired	0.0400578	0.959851	RF	78.3	Liver	No
SRX7101808	25404599	Paired	0.0365904	0.963312	RF	78.63	Liver	No
SRX7101809	32887252	Paired	0.0364583	0.962997	RF	77.72	Liver	No
SRX7141530	20051745	Paired	0.0243668	0.976733	RF	74.84	Artery	No
SRX7141531	21969470	Paired	0.0232072	0.978944	RF	85.88	Artery	No

ERX3013314	21372757	Single	0.0428259	NULL	RF	76.61		Whole blood	No
ERX3013315	23659509	Single	0.0649589	NULL	RF	62.46		Whole blood	No
ERX3013316	25222463	Single	0.0530321	NULL	RF	67.86		Whole blood	No
ERX3013317	25439537	Single	0.0483616	NULL	RF	64.19		Whole blood	No
SRX5712705	15987336	Paired	0.95741	0.0410037	FR	90.07		Brain	No
ERX3013318	36647462	Single	0.0538983	NULL	RF	69.2		Whole blood	No
SRX5712706	15805007	Paired	0.955531	0.0429522	FR	89.39		Brain	No
SRX5712707	17309315	Paired	0.957148	0.0414639	FR	89.61		Brain	No
SRX5712708	13953896	Paired	0.957304	0.0413569	FR	85.93		Brain	No
SRX5712709	17469970	Paired	0.958855	0.0402456	FR	90.07		Brain	No
SRX3594496	22976838	Single	0.864761	NULL	FR	69.91		Uterus	No
SRX7101810	37444542	Paired	0.038948	0.960652	RF	80.74		Liver	No
SRX3594498	20907586	Single	0.84533	NULL	FR	71.66		Uterus	No
SRX7101811	35394653	Paired	0.0360058	0.963677	RF	75.82		Liver	No
SRX3594499	23485608	Single	0.846519	NULL	FR	68.91		Uterus	No
SRX7101812	28370631	Paired	0.0389644	0.960608	RF	77.61		Liver	No
SRX7101813	35127104	Paired	0.0454785	0.953676	RF	78.94		Liver	No
SRX7101814	34394740	Paired	0.0353275	0.964049	RF	86.52		Liver	No
SRX7101815	17553960	Paired	0.046196	0.953036	RF	79.71		Liver	No
SRX7101816	23422006	Paired	0.0430034	0.955922	RF	82		Liver	No
SRX7101817	37073444	Paired	0.0384578	0.962054	RF	83.24		Liver	No
SRX7101818	18263922	Paired	0.0451476	0.955579	RF	77.11		Liver	No
SRX7101819	19265097	Paired	0.0434207	0.956457	RF	78.67		Liver	No
ERX3013321	34918192	Single	0.0451207	NULL	RF	71.84		Whole blood	No
ERX3013322	37010804	Single	0.0492311	NULL	RF	65.55		Whole blood	No
SRX5712710	15132385	Paired	0.957903	0.040788	FR	89.88		Brain	No
ERX3013323	33497559	Single	0.0777833	NULL	RF	60.34		Whole blood	No
ERX3013324	32177461	Single	0.0474229	NULL	RF	77.14		Whole blood	No
ERX3013325	33173001	Single	0.042411	NULL	RF	72.47		Whole blood	No
ERX3013328	30778081	Single	0.0534954	NULL	RF	70.36		Whole blood	No
ERX3013329	27760011	Single	0.0692468	NULL	RF	68.62		Whole blood	No
SRX9154178	72324691	Single	0.0173637	NULL	RF	92.69		Ovary	No
SRX5401591	64926671	Paired	0.0267838	0.973322	RF	88.17		Muscle	No
SRX9154179	77731443	Single	0.0225416	NULL	RF	91.13		Ovary	No
SRX6430407	31187228	Paired	0.0264051	0.973759	RF	79.13		Large intestine	No
SRX5401592	63301362	Paired	0.02827	0.971959	RF	86.99		Muscle	No
SRX6430408	30276161	Paired	0.0291568	0.97068	RF	80.52		Large intestine	No
SRX5401593	57117252	Paired	0.030809	0.968898	RF	87.22		Muscle	No
SRX6430409	37379071	Paired	0.0319271	0.967571	RF	80.58		Large intestine	No
SRX5401594	54876941	Paired	0.0280862	0.97199	RF	88.99		Muscle	No
SRX5401596	82417361	Paired	0.0552579	0.940975	RF	81.51		Muscle	No
SRX5401597	55515465	Paired	0.0918816	0.903933	RF	81.42		Muscle	No
SRX5401598	56127433	Paired	0.0918755	0.903831	RF	81.94		Muscle	No
SRX7101820	15516646	Paired	0.0349354	0.965283	RF	74.36		Liver	No
SRX5401599	54029830	Paired	0.0980139	0.897707	RF	81.72		Muscle	No
SRX7101821	21081014	Paired	0.0326449	0.967208	RF	75.82		Liver	No
SRX7101822	27771570	Paired	0.0360385	0.963851	RF	77.03		Liver	No
SRX7101823	16447811	Paired	0.0375652	0.962074	RF	79.84		Liver	No
ERX3013330	36601106	Single	0.084075	NULL	RF	61.38		Whole blood	No
ERX3013331	26377759	Single	0.0809794	NULL	RF	60.06		Whole blood	No
ERX3013332	4072529	Single	0.076196	NULL	RF	60.33		Whole blood	No
ERX3013333	34173509	Single	0.104269	NULL	RF	61.89		Whole blood	No
ERX3013336	39159241	Single	0.0690925	NULL	RF	61.57		Whole blood	No
SRX4634146	26305913	Paired	0.0535411	0.946722	RF	91.07		Muscle	No
SRX4634147	26402967	Paired	0.0570382	0.94232	RF	89.37		Muscle	No
SRX9154182	81582237	Single	0.0211258	NULL	RF	92.72		Ovary	No
SRX6430410	31520855	Paired	0.0326769	0.967061	RF	79.45		Large intestine	No
SRX4634148	25453373	Paired	0.0591489	0.939343	RF	89.17		Muscle	No

SRX9154183	65290782	Single	0.0188292	NULL	RF	93.11	Ovary	No
SRX6430411	41215106	Paired	0.027029	0.972806	RF	80.13	Large intestine	No
SRX4634149	28495518	Paired	0.0879114	0.908764	RF	86.38	Muscle	No
SRX9154184	83952515	Single	0.0195364	NULL	RF	93.05	Ovary	No
SRX6430412	41260474	Paired	0.0307245	0.969003	RF	80.51	Large intestine	No
SRX6430413	36123254	Paired	0.0364733	0.963271	RF	77.71	Large intestine	No
SRX6430414	36021193	Paired	0.0344214	0.96549	RF	77.49	Large intestine	No
SRX6430415	31099357	Paired	0.025336	0.974848	RF	81.03	Large intestine	No
SRX9154188	47699838	Single	0.0214692	NULL	RF	91.76	Ovary	No
SRX6430416	33898011	Paired	0.0243727	0.975633	RF	79.24	Large intestine	No
SRX9154189	86335029	Single	0.0231449	NULL	RF	91.13	Ovary	No
SRX6430417	36660769	Paired	0.0271342	0.972906	RF	80.23	Large intestine	No
SRX6430418	31071321	Paired	0.0283974	0.971836	RF	79.55	Large intestine	No
SRX6430419	30635144	Paired	0.02873	0.971877	RF	79.84	Large intestine	No
SRX2210928	39055995	Single	0.0438615	NULL	RF	64.39	Adipose	No
ERX3013347	33600046	Single	0.0773528	NULL	RF	63.18	Whole blood	No
ERX3013348	29792467	Single	0.0798191	NULL	RF	65.81	Whole blood	No
SRX2734522	17542647	Paired	0.963755	0.0561254	FR	76.52	Brain	No
SRX4634150	28141648	Paired	0.0591006	0.939421	RF	88.02	Muscle	No
SRX2734523	19459045	Paired	0.952715	0.0647917	FR	73.3	Brain	No
SRX4634151	26767970	Paired	0.0540807	0.945013	RF	87.37	Muscle	No
SRX2734524	16599543	Paired	0.962104	0.0670448	FR	73.51	Brain	No
SRX4634152	27848891	Paired	0.0828941	0.914181	RF	88.69	Muscle	No
SRX2734525	17798461	Paired	0.965333	0.0312811	FR	76.14	Brain	No
SRX4634153	25028726	Paired	0.0550008	0.943844	RF	87.9	Muscle	No
SRX2734526	39710493	Paired	0.985427	0.0157441	FR	66.34	Brain	No
SRX4634154	24535483	Paired	0.0762244	0.920376	RF	88.71	Muscle	No
SRX2734527	26785042	Paired	0.98182	0.018992	FR	66.26	Brain	No
SRX4634155	23326411	Paired	0.0611168	0.937096	RF	89.22	Muscle	No
SRX2734528	23234447	Paired	0.985355	0.0158901	FR	79.62	Brain	No
ERX4361643	33866957	Paired	0.0473704	0.951197	RF	87.27	Brain	No
SRX9154190	76343058	Single	0.0222768	NULL	RF	92.57	Ovary	No
SRX4634156	29809876	Paired	0.0797891	0.91613	RF	90.58	Muscle	No
ERX4361644	33447295	Paired	0.0371732	0.959147	RF	86.61	Brain	No
SRX9154191	77333644	Single	0.0210679	NULL	RF	91.93	Ovary	No
SRX4634157	28763170	Paired	0.0760196	0.919794	RF	89.71	Muscle	No
ERX4361645	30385306	Paired	0.0559728	0.942598	RF	88.55	Brain	No
SRX6430420	34088747	Paired	0.0310974	0.968892	RF	77.21	Large intestine	No
SRX4634158	23883991	Paired	0.0826506	0.913466	RF	89.77	Muscle	No
ERX4361646	37453597	Paired	0.0546576	0.943573	RF	78.37	Brain	No
SRX6430421	28001984	Paired	0.0277619	0.972498	RF	81.7	Large intestine	No
SRX4634159	26297862	Paired	0.0761677	0.920633	RF	90.03	Muscle	No
ERX4361647	32831792	Paired	0.0471054	0.951374	RF	86.08	Brain	No
SRX6430422	30556730	Paired	0.0296203	0.970628	RF	81.88	Large intestine	No
ERX4361648	32031257	Paired	0.0465043	0.952631	RF	89.29	Brain	No
SRX9154195	50902903	Single	0.0240702	NULL	RF	89.93	Ovary	No
SRX6430423	49455256	Paired	0.0309719	0.969084	RF	81.78	Large intestine	No
ERX4361649	31552614	Paired	0.0365405	0.960465	RF	85.76	Brain	No
SRX9154196	64569885	Single	0.0222794	NULL	RF	92.51	Ovary	No
SRX6430424	37075634	Paired	0.0321226	0.967646	RF	80.78	Large intestine	No
SRX9154197	98018090	Single	0.0224963	NULL	RF	91.35	Ovary	No
SRX6430425	39707187	Paired	0.0290914	0.971069	RF	79.47	Large intestine	No
SRX6430426	38122185	Paired	0.0289532	0.971161	RF	82.53	Large intestine	No
SRX6430427	44526006	Paired	0.0294217	0.970694	RF	80.22	Large intestine	No
SRX6430428	39700165	Paired	0.0301258	0.969899	RF	81.46	Large intestine	No
SRX6430429	43038928	Paired	0.032455	0.967542	RF	81.34	Large intestine	No
ERX3013350	25769651	Single	0.0463279	NULL	RF	79.29	Whole blood	No
ERX3013351	29969535	Single	0.0712639	NULL	RF	61.99	Whole blood	No

ERX3013353	25847643	Single	0.0449152	NULL	RF	67.35	Whole blood	No
ERX3013354	29971241	Single	0.062807	NULL	RF	60.63	Whole blood	No
ERX3013355	34663539	Single	0.0401994	NULL	RF	79.9	Whole blood	No
ERX3013356	30547815	Single	0.0591706	NULL	RF	65.54	Whole blood	No
ERX3013357	29916226	Single	0.0757777	NULL	RF	62.19	Whole blood	No
ERX3013358	44573936	Single	0.0589068	NULL	RF	76.5	Whole blood	No
ERX3013359	28322219	Single	0.0591254	NULL	RF	67.62	Whole blood	No
SRX4634160	24450331	Paired	0.0672015	0.929992	RF	89.85	Muscle	No
SRX4634161	29314706	Paired	0.0907008	0.904629	RF	89.91	Muscle	No
SRX4634162	27003123	Paired	0.126008	0.866639	RF	89.15	Muscle	No
ERX4361650	30838488	Paired	0.049767	0.949208	RF	91.3	Brain	No
SRX4634163	27121776	Paired	0.121563	0.872136	RF	89.58	Muscle	No
ERX4361651	34800230	Paired	0.0494405	0.948823	RF	90.35	Brain	No
SRX4634164	21697485	Paired	0.0961457	0.898642	RF	87.7	Muscle	No
SRX4634165	22890997	Paired	0.0906111	0.905722	RF	90.35	Muscle	No
SRX4634166	27903502	Paired	0.0911744	0.904235	RF	89.46	Muscle	No
SRX4634167	24063916	Paired	0.0797691	0.916457	RF	90.14	Muscle	No
SRX6430430	35434061	Paired	0.0329509	0.967044	RF	81.19	Large intestine	No
SRX4634168	25502300	Paired	0.156038	0.833134	RF	88.17	Muscle	No
SRX6430431	33232120	Paired	0.0250334	0.9752	RF	79.51	Large intestine	No
SRX4634169	22700652	Paired	0.0805283	0.915311	RF	88.9	Muscle	No
SRX6430432	32535411	Paired	0.027518	0.972428	RF	77.66	Large intestine	No
SRX6430433	27886255	Paired	0.0284285	0.971823	RF	81.75	Large intestine	No
SRX6430434	31749066	Paired	0.0275653	0.972562	RF	81.01	Large intestine	No
SRX6430435	30310180	Paired	0.0317027	0.968237	RF	81.47	Large intestine	No
SRX6430436	37953625	Paired	0.0286759	0.971458	RF	81.27	Large intestine	No
SRX6430437	46329229	Paired	0.0248552	0.975507	RF	81.09	Large intestine	No
SRX6430438	37973249	Paired	0.0289993	0.971092	RF	79.91	Large intestine	No
SRX6430439	40562057	Paired	0.0263473	0.973844	RF	81.93	Large intestine	No
ERX3013360	27181222	Single	0.0516107	NULL	RF	72.9	Whole blood	No
ERX3013361	30951916	Single	0.0623468	NULL	RF	66.73	Whole blood	No
ERX3013362	36675595	Single	0.0577964	NULL	RF	75.15	Whole blood	No
ERX3013363	34407159	Single	0.0608713	NULL	RF	63.99	Whole blood	No
ERX3013364	25529263	Single	0.0819887	NULL	RF	64.3	Whole blood	No
ERX3013365	25601181	Single	0.0630767	NULL	RF	71.85	Whole blood	No
ERX3013366	32962685	Single	0.0525291	NULL	RF	71.23	Whole blood	No
ERX3013367	36553206	Single	0.0472144	NULL	RF	79.71	Whole blood	No
ERX3013368	37002966	Single	0.0444122	NULL	RF	78.77	Whole blood	No
ERX3013369	32958019	Single	0.0366682	NULL	RF	81.07	Whole blood	No
SRX4634170	26449512	Paired	0.128447	0.864838	RF	88.56	Muscle	No
SRX4634171	27828367	Paired	0.122814	0.8694	RF	89.66	Muscle	No
SRX4634172	27289065	Paired	0.0953426	0.899722	RF	89.43	Muscle	No
SRX4634173	27781362	Paired	0.080796	0.915604	RF	86.24	Muscle	No
SRX4634174	25222904	Paired	0.0951084	0.899781	RF	87.58	Muscle	No
SRX4634175	25120631	Paired	0.0792875	0.916356	RF	87.23	Muscle	No
SRX4634176	25583670	Paired	0.086677	0.908823	RF	86.41	Muscle	No
SRX4634177	27151642	Paired	0.087991	0.907244	RF	85.65	Muscle	No
SRX6430440	46560806	Paired	0.0280641	0.972216	RF	80.66	Large intestine	No
SRX6430441	53345104	Paired	0.0280939	0.972116	RF	81.5	Large intestine	No
SRX6430442	28984864	Paired	0.0265534	0.973558	RF	81.71	Large intestine	No
SRX6430443	42137612	Paired	0.0295768	0.970523	RF	81.2	Large intestine	No
SRX6430444	32794075	Paired	0.0285131	0.971615	RF	82.23	Large intestine	No
SRX6430445	35146606	Paired	0.0286707	0.971392	RF	81.51	Large intestine	No
SRX6430446	43645685	Paired	0.028583	0.971627	RF	82.64	Large intestine	No
ERX3013370	29662017	Single	0.0556968	NULL	RF	64.27	Whole blood	No
ERX3013371	32163146	Single	0.0510285	NULL	RF	70.79	Whole blood	No
ERX3013373	28473615	Single	0.0356851	NULL	RF	85.12	Whole blood	No
ERX3013374	32902108	Single	0.0357407	NULL	RF	84.21	Whole blood	No

ERX3013376	33069823	Single	0.0592997	NULL	RF	66.94	Whole blood	No
ERX3013377	30549757	Single	0.0372371	NULL	RF	83.71	Whole blood	No
ERX3013378	27642536	Single	0.0478456	NULL	RF	81.99	Whole blood	No
ERX3013379	31719239	Single	0.0567902	NULL	RF	75.89	Whole blood	No
SRX2267648	26613935	Paired	0.0131204	0.987781	RF	87.4	Cartilage	No
ERX3013380	28152098	Single	0.0448902	NULL	RF	88.76	Whole blood	No
ERX3013381	31496363	Single	0.0324192	NULL	RF	91.65	Whole blood	No
ERX3013382	36862754	Single	0.0786848	NULL	RF	67.44	Whole blood	No
ERX3013383	31771849	Single	0.0750795	NULL	RF	63.55	Whole blood	No
ERX3013384	28775087	Single	0.0440562	NULL	RF	86.13	Whole blood	No
ERX3013385	30041099	Single	0.0694852	NULL	RF	73.07	Whole blood	No
ERX3013386	33913807	Single	0.0732743	NULL	RF	63.82	Whole blood	No
ERX3013387	26461351	Single	0.0630386	NULL	RF	73.69	Whole blood	No
ERX3013388	43577773	Single	0.0418195	NULL	RF	77.82	Whole blood	No
ERX3013389	42112177	Single	0.0327594	NULL	RF	62.68	Whole blood	No
SRX2425138	33184587	Paired	0.0439999	0.954744	RF	88.48	Liver	No
SRX2425139	32673143	Paired	0.0517804	0.950609	RF	89.09	Liver	No
SRX2267652	25298393	Paired	0.0119554	0.988765	RF	89.95	Cartilage	No
SRX2267658	26426109	Paired	0.0124568	0.988207	RF	91.36	Cartilage	No
ERX3013390	28446219	Single	0.0548847	NULL	RF	66.03	Whole blood	No
ERX3013393	34638571	Single	0.053693	NULL	RF	62.83	Whole blood	No
ERX3013394	28632754	Single	0.0618213	NULL	RF	60.86	Whole blood	No
ERX3013395	33755765	Single	0.0726456	NULL	RF	60.35	Whole blood	No
ERX3013396	32692077	Single	0.054805	NULL	RF	63.08	Whole blood	No
ERX3013397	32207897	Single	0.0589358	NULL	RF	65.09	Whole blood	No
ERX3013399	36439701	Single	0.0334462	NULL	RF	86.89	Whole blood	No
SRX2425140	31224800	Paired	0.0402505	0.9579	RF	91.04	Liver	No
SRX2267661	25340836	Paired	0.0126329	0.987848	RF	89.88	Cartilage	No
SRX2267663	23198856	Paired	0.0147641	0.985723	RF	92.18	Cartilage	No
SRX2267664	25903998	Paired	0.0128481	0.987594	RF	92.13	Cartilage	No
SRX2267666	24133336	Paired	0.0116033	0.98887	RF	90.79	Cartilage	No
SRX2267667	24995593	Paired	0.0104955	0.989913	RF	90.69	Cartilage	No
ERX1700230	18824313	Paired	0.0315046	0.969951	RF	91.38	Macrophage	No
ERX1700231	21215482	Paired	0.0283688	0.973016	RF	92.38	Macrophage	No
ERX1700232	19454995	Paired	0.0295569	0.971757	RF	91.28	Macrophage	No
SRX9171764	33165479	Paired	0.0201665	0.983242	RF	75.17	Macrophage	No
SRX9171765	33099891	Paired	0.019958	0.983501	RF	75.17	Macrophage	No
SRX9171766	32894885	Paired	0.0203336	0.98303	RF	75.42	Macrophage	No
SRX9171767	36708832	Paired	0.0186555	0.983112	RF	73.62	Macrophage	No
SRX9171768	46221488	Paired	0.0188778	0.982641	RF	73.8	Macrophage	No
SRX9171769	31874522	Paired	0.018558	0.982996	RF	73.93	Macrophage	No
ERX4406561	33866957	Paired	0.0473533	0.951213	RF	87.27	Brain	No
ERX4406562	33447295	Paired	0.0371576	0.959124	RF	86.61	Brain	No
ERX4406563	30385306	Paired	0.0560074	0.94254	RF	88.55	Brain	No
ERX4406564	37453597	Paired	0.0546437	0.94359	RF	78.37	Brain	No
ERX4406565	32831792	Paired	0.0470978	0.951407	RF	86.08	Brain	No
ERX4406566	32031257	Paired	0.0464902	0.952614	RF	89.29	Brain	No
ERX4406567	31552614	Paired	0.0365671	0.960443	RF	85.76	Brain	No
ERX4406568	30838488	Paired	0.0497653	0.949223	RF	91.3	Brain	No
ERX4406569	34800230	Paired	0.0494467	0.94882	RF	90.35	Brain	No
SRX3719254	19800604	Paired	0.0735217	0.926509	RF	82.24	Large intestine	No
SRX3719255	23525131	Paired	0.0718321	0.93059	RF	79.98	Large intestine	No
SRX3719256	18040658	Paired	0.0671883	0.935882	RF	82.8	Large intestine	No
SRX3719257	19881813	Paired	0.0896589	0.913879	RF	79.39	Large intestine	No
SRX3719258	25930033	Paired	0.0837061	0.918813	RF	83	Large intestine	No
SRX3719259	24017443	Paired	0.103717	0.900886	RF	76.84	Large intestine	No
ERX4406570	34901237	Paired	0.0372631	0.962725	RF	80.82	Brain	No
ERX4406571	41539314	Paired	0.0383816	0.96126	RF	83.75	Brain	No

ERX4406572	34382770	Paired	0.054439	0.945293	RF	83.87	Brain	No
ERX4406573	34772199	Paired	0.0408402	0.95874	RF	83.21	Brain	No
ERX4406574	39637470	Paired	0.0338209	0.966183	RF	80.14	Brain	No
ERX4406575	34561662	Paired	0.0326498	0.967412	RF	87.3	Brain	No
ERX4406576	31240744	Paired	0.0543834	0.94606	RF	88.45	Brain	No
ERX4406577	36660918	Paired	0.0397846	0.960243	RF	87.5	Brain	No
ERX4406578	38702289	Paired	0.0347973	0.965192	RF	85.02	Brain	No
ERX4406579	30697732	Paired	0.0749283	0.924009	RF	90.14	Ovary	No
SRX3719260	24991150	Paired	0.10018	0.901525	RF	80.46	Large intestine	No
SRX3719261	18750375	Paired	0.0467127	0.956116	RF	80.83	Large intestine	No
SRX3719262	22447086	Paired	0.0657616	0.939864	RF	81.71	Large intestine	No
SRX3719263	15876894	Paired	0.0625704	0.943403	RF	81.18	Large intestine	No
SRX3719264	28796453	Paired	0.0919531	0.910791	RF	83.39	Large intestine	No
SRX1469702	9872640	Single	0.881247	NULL	FR	64.6	3D4/31	No
SRX1469703	16560625	Single	0.882768	NULL	FR	67.93	3D4/31	No
SRX1469704	14586017	Single	0.853669	NULL	FR	67.37	3D4/31	No
SRX4881739	34398910	Paired	0.0210042	0.980472	RF	90.98	Lung	No
SRX1469707	8848436	Single	0.835472	NULL	FR	61.48	3D4/31	No
SRX6968960	41532970	Paired	0.0383603	0.96125	RF	83.76	Brain	No
SRX6968961	34369375	Paired	0.0544679	0.94527	RF	83.9	Brain	No
SRX6968962	31237133	Paired	0.054378	0.946095	RF	88.45	Brain	No
SRX6968963	36657193	Paired	0.0397708	0.96025	RF	87.51	Brain	No
SRX6968964	38697960	Paired	0.0347657	0.965272	RF	85.02	Brain	No
SRX6968965	34767474	Paired	0.040867	0.958763	RF	83.22	Brain	No
SRX6968966	39620833	Paired	0.0338296	0.96618	RF	80.17	Brain	No
SRX7817025	31977689	Paired	0.0737978	0.932888	RF	75.68	Whole blood	No
SRX6968967	34558148	Paired	0.032641	0.967388	RF	87.32	Brain	No
SRX6968968	34881780	Paired	0.0372706	0.962731	RF	80.86	Brain	No
SRX7817027	33395872	Paired	0.0825734	0.924463	RF	81.17	Whole blood	No
SRX7817028	30867911	Paired	0.0771454	0.925367	RF	87.28	Whole blood	No
SRX7817029	39621733	Paired	0.0597922	0.939989	RF	83.74	Whole blood	No
ERX4406580	36358417	Paired	0.100764	0.896312	RF	89.85	Ovary	No
ERX4406581	33933938	Paired	0.0578891	0.942319	RF	90.01	Ovary	No
ERX4406582	30120871	Paired	0.124556	0.872383	RF	77.05	Ovary	No
ERX4406583	28442164	Paired	0.0390577	0.96125	RF	87.56	Ovary	No
ERX4406584	34547680	Paired	0.0450206	0.955107	RF	89.75	Ovary	No
ERX4406585	30792796	Paired	0.033922	0.965799	RF	84.8	Ovary	No
ERX4406586	31621437	Paired	0.0340569	0.965661	RF	88.89	Ovary	No
ERX4406587	33835403	Paired	0.0343695	0.964471	RF	87.29	Ovary	No
SRX4881740	33623134	Paired	0.0230015	0.978488	RF	90.14	Lung	No
SRX4881741	33659422	Paired	0.0193948	0.982097	RF	90.76	Lung	No
SRX4881742	34138449	Paired	0.0188046	0.983092	RF	91.23	Lung	No
SRX1469710	7834483	Single	0.817097	NULL	FR	61.22	3D4/31	No
SRX4881743	34051915	Paired	0.0207819	0.980835	RF	90.48	Lung	No
SRX1469711	8977456	Single	0.900619	NULL	FR	65.02	3D4/31	No
SRX4881744	32878705	Paired	0.0202347	0.981455	RF	90.95	Lung	No
SRX1469712	17336995	Single	0.89259	NULL	FR	65.03	3D4/31	No
SRX4881745	34035139	Paired	0.0193284	0.982598	RF	91.19	Lung	No
SRX1469713	17136435	Single	0.877937	NULL	FR	68.45	3D4/31	No
SRX4881746	32953244	Paired	0.0197349	0.981929	RF	90.97	Lung	No
SRX4881747	33923390	Paired	0.0197771	0.982641	RF	88.04	Lung	No
SRX4881748	33393014	Paired	0.0225127	0.979446	RF	89.14	Lung	No
SRX1469716	8989032	Single	0.866237	NULL	FR	61.92	3D4/31	No
SRX4881749	33285728	Paired	0.0199717	0.982302	RF	84.14	Lung	No
SRX1469717	9442777	Single	0.836158	NULL	FR	61.77	3D4/31	No
SRX1469718	16721155	Single	0.888574	NULL	FR	66.1	3D4/31	No
SRX1469719	15060281	Single	0.862704	NULL	FR	67.08	3D4/31	No
SRX7817030	31333576	Paired	0.071305	0.93602	RF	77.82	Whole blood	No

SRX7817031	34735588	Paired	0.0730757	0.934373	RF	82.24	Whole blood	No
SRX7817032	35831071	Paired	0.0784013	0.926686	RF	85.6	Whole blood	No
SRX7817033	31328806	Paired	0.0784737	0.928793	RF	81.33	Whole blood	No
SRX7817034	33660806	Paired	0.078969	0.928284	RF	82.44	Whole blood	No
SRX7817035	36618908	Paired	0.0763074	0.926172	RF	85.05	Whole blood	No
SRX7817036	34951300	Paired	0.0798074	0.931687	RF	78.52	Whole blood	No
SRX4881750	33623008	Paired	0.0208774	0.980894	RF	81.2	Lung	No
SRX4881751	33241763	Paired	0.0199604	0.98213	RF	86.38	Lung	No
SRX4881752	33398685	Paired	0.0206661	0.981504	RF	84.2	Lung	No
SRX1469722	10390352	Single	0.803706	NULL	FR	61.84	3D4/31	No
SRX1469725	9267149	Single	0.850989	NULL	FR	63.4	3D4/31	No
SRX1469726	10271786	Single	0.899705	NULL	FR	62.34	3D4/31	No
SRX1469727	15681215	Single	0.91441	NULL	FR	69.33	3D4/31	No
SRX1469728	17666223	Single	0.878131	NULL	FR	69.05	3D4/31	No
SRX1469729	7534536	Single	0.846898	NULL	FR	63.67	3D4/31	No
SRX1469730	17748428	Single	0.893245	NULL	FR	67.78	3D4/31	No
SRX1469731	20977232	Single	0.8458	NULL	FR	68.09	3D4/31	No
SRX2835403	35663932	Paired	0.00976811	0.991037	RF	86.95	Muscle	No
SRX2835404	25069404	Paired	0.00990506	0.990874	RF	87.55	Muscle	No
SRX2835405	37934521	Paired	0.00974029	0.991094	RF	86.9	Muscle	No
SRX2835406	21840756	Paired	0.0120686	0.988743	RF	87.46	Muscle	No
SRX2835407	23909143	Paired	0.010967	0.989896	RF	84.7	Muscle	No
SRX2835408	32250539	Paired	0.009622	0.991022	RF	86.83	Muscle	No
SRX7211449	18027809	Paired	0.0281678	0.967069	RF	84.68	Adipose	No
SRX9914893	45388081	Paired	0.0406778	0.957665	RF	85.83	Testis	No
SRX9914895	45297585	Paired	0.0351976	0.963254	RF	82.99	Testis	No
SRX9914897	46031716	Paired	0.0327757	0.965992	RF	84.73	Testis	No
SRX3791896	17567397	Single	0.915531	NULL	FR	75.34	Cartilage	No
SRX3791897	7934106	Single	0.909832	NULL	FR	65.06	Cartilage	No
SRX3791898	14579063	Single	0.86975	NULL	FR	60.62	Cartilage	No
SRX4781393	55576978	Paired	0.0473836	0.952315	RF	60.59	IPEC-J2	No
SRX4781395	49451340	Paired	0.0372194	0.963079	RF	63.94	IPEC-J2	No
SRX7211450	17915325	Paired	0.0281158	0.967072	RF	84.67	Adipose	No
SRX7211451	21606874	Paired	0.0290279	0.966692	RF	78.99	Adipose	No
SRX7211452	21269170	Paired	0.0290817	0.966637	RF	79.02	Adipose	No
SRX7211453	18215903	Paired	0.0314448	0.964545	RF	80.48	Adipose	No
SRX7211454	18091602	Paired	0.0314824	0.964607	RF	80.53	Adipose	No
SRX7211455	18387437	Paired	0.0300064	0.967379	RF	81.33	Adipose	No
SRX7211456	18271999	Paired	0.0300845	0.967361	RF	81.34	Adipose	No
SRX7211457	26196922	Paired	0.0300388	0.963503	RF	82.64	Adipose	No
SRX7211458	26007856	Paired	0.030171	0.963602	RF	82.69	Adipose	No
SRX7211459	21605845	Paired	0.0300587	0.962916	RF	81.53	Adipose	No
SRX2184877	40781610	Paired	0.9716	0.02947	FR	72.77	Fibroblast	No
ERX3462538	19150033	Single	0.0228926	NULL	RF	91.3	Large intestine	No
ERX3462539	19205253	Single	0.0234905	NULL	RF	89.3	Large intestine	No
SRX8599850	56307403	Paired	0.0139173	0.986758	RF	88.45	Skin	No
SRX8599851	43121069	Paired	0.0177656	0.983012	RF	89.02	Skin	No
SRX8599856	47600026	Paired	0.0236316	0.977285	RF	87.24	Skin	No
SRX8599857	54473834	Paired	0.0203277	0.980655	RF	85.51	Skin	No
SRX8599858	48434509	Paired	0.0148465	0.985856	RF	84.59	Skin	No
SRX8599859	49859111	Paired	0.0149483	0.98557	RF	90.21	Skin	No
SRX7211460	21248805	Paired	0.0300563	0.962791	RF	81.51	Adipose	No
SRX7211461	19830719	Paired	0.031454	0.961102	RF	84.69	Adipose	No
SRX7211462	48137694	Paired	0.0374423	0.958802	RF	80.62	Adipose	No
SRX7211463	46545413	Paired	0.0341621	0.961825	RF	80.48	Adipose	No
SRX7211464	38052472	Paired	0.0350455	0.960422	RF	82.87	Adipose	No
SRX7211465	40622119	Paired	0.0340548	0.962167	RF	79.88	Adipose	No
SRX7211466	56386061	Paired	0.0337907	0.964264	RF	78.53	Adipose	No

SRX7211467	18100803	Paired	0.0358916	0.959722	RF	80.66	Adipose	No
SRX7211468	33703362	Paired	0.0345546	0.961549	RF	80.52	Adipose	No
SRX7211469	42881356	Paired	0.0349198	0.961206	RF	77.88	Adipose	No
SRX2888220	42386336	Paired	0.98432	0.0166713	FR	87.71	Ovary	No
SRX2888221	46183073	Paired	0.0154765	0.985478	RF	89.24	Ovary	No
SRX2888222	43751828	Paired	0.0164805	0.984434	RF	87.44	Ovary	No
ERX3462540	19255337	Single	0.0234809	NULL	RF	89.82	Large intestine	No
ERX3462541	18807455	Single	0.0241913	NULL	RF	90.1	Large intestine	No
ERX3462542	20360457	Single	0.022019	NULL	RF	91.92	Large intestine	No
ERX3462543	18889294	Single	0.0230543	NULL	RF	91.66	Large intestine	No
ERX3462544	19044761	Single	0.0226221	NULL	RF	92.34	Large intestine	No
ERX3462545	19411629	Single	0.0235937	NULL	RF	91.56	Large intestine	No
ERX3462546	21193021	Single	0.0242935	NULL	RF	88.32	Large intestine	No
ERX3462547	18922990	Single	0.0225214	NULL	RF	91.25	Large intestine	No
ERX3462548	19064688	Single	0.0230154	NULL	RF	91.9	Large intestine	No
ERX3462549	18581881	Single	0.0239465	NULL	RF	90.05	Large intestine	No
SRX7211470	49630426	Paired	0.0315793	0.965073	RF	76.79	Adipose	No
SRX7211471	57343641	Paired	0.0371427	0.956804	RF	76.86	Adipose	No
SRX7211472	19695003	Paired	0.0315448	0.961165	RF	84.73	Adipose	No
SRX7211473	34655313	Paired	0.0366249	0.959798	RF	77.36	Adipose	No
SRX7211474	20766208	Single	0.031068	NULL	RF	83.44	Adipose	No
SRX7211475	20408965	Paired	0.0307693	0.964356	RF	81.42	Adipose	No
SRX7211476	20296536	Paired	0.0290961	0.967181	RF	80.56	Adipose	No
SRX7211477	19935323	Paired	0.029071	0.967133	RF	80.59	Adipose	No
SRX7211478	20143794	Paired	0.0303773	0.963923	RF	81.57	Adipose	No
SRX7211479	19865463	Paired	0.0304048	0.963889	RF	81.53	Adipose	No
SRX4510381	18872197	Single	0.953286	NULL	FR	68.26	Whole blood	No
SRX4510382	16831817	Single	0.952257	NULL	FR	76.25	Whole blood	No
SRX4510383	8544725	Single	0.946316	NULL	FR	72.09	Whole blood	No
SRX4510384	15223097	Single	0.949715	NULL	FR	69.51	Whole blood	No
SRX2184898	27190814	Paired	0.970193	0.0318365	FR	72.5	Fibroblast	No
SRX2191437	40781610	Paired	0.971592	0.029454	FR	72.77	Fibroblast	No
SRX4510385	24151847	Single	0.952486	NULL	FR	61.59	Whole blood	No
SRX2191438	27190814	Paired	0.970214	0.0318609	FR	72.5	Fibroblast	No
SRX4510386	16582215	Single	0.954767	NULL	FR	69.96	Whole blood	No
SRX4510387	16507362	Single	0.957047	NULL	FR	72.82	Whole blood	No
SRX4510388	12447154	Single	0.953936	NULL	FR	68.77	Whole blood	No
SRX4510389	13808894	Single	0.951804	NULL	FR	72.08	Whole blood	No
SRX1091045	15895315	Paired	0.0256877	0.978299	RF	84.14	Muscle	No
SRX1091046	13382410	Paired	0.0171058	0.982604	RF	85.53	Muscle	No
ERX3462550	19690830	Single	0.024674	NULL	RF	91.44	Large intestine	No
ERX3462551	19728264	Single	0.022172	NULL	RF	91.04	Large intestine	No
SRX8418246	40002453	Paired	0.0395889	0.97506	RF	75.58	PBMCs	No
ERX3462552	19219659	Single	0.0259501	NULL	RF	91.66	Large intestine	No
SRX8418247	38513030	Paired	0.0404102	0.974081	RF	71.55	PBMCs	No
ERX3462553	19532436	Single	0.0244962	NULL	RF	92.45	Large intestine	No
SRX8418248	31998629	Paired	0.0435932	0.973971	RF	71.98	PBMCs	No
ERX3462554	22416052	Single	0.0228853	NULL	RF	91.19	Large intestine	No
SRX8418249	30745647	Paired	0.0454799	0.972883	RF	73.27	PBMCs	No
ERX3462555	19265172	Single	0.024278	NULL	RF	91.14	Large intestine	No
ERX3462556	19131146	Single	0.0233165	NULL	RF	91.9	Large intestine	No
ERX3462557	19383341	Single	0.0244216	NULL	RF	92.03	Large intestine	No
ERX3462558	19989553	Single	0.0237546	NULL	RF	92.02	Large intestine	No
ERX3462559	19619834	Single	0.0222568	NULL	RF	92.45	Large intestine	No
SRX4510390	25447739	Single	0.952154	NULL	FR	60.95	Whole blood	No
SRX4510392	18378177	Single	0.953817	NULL	FR	66.18	Whole blood	No
ERX3462561	19723888	Single	0.0228884	NULL	RF	92.2	Large intestine	No
ERX3462562	19150695	Single	0.0230727	NULL	RF	91.77	Large intestine	No

ERX3462563	19117430	Single	0.0235855	NULL	RF	92.29	Large intestine	No
ERX3462564	19708873	Single	0.0226236	NULL	RF	91.62	Large intestine	No
ERX3462565	19409417	Single	0.0239564	NULL	RF	92.24	Large intestine	No
ERX3462566	19587035	Single	0.0232593	NULL	RF	91.87	Large intestine	No
ERX3462567	19534606	Single	0.0223591	NULL	RF	92.28	Large intestine	No
ERX3462568	19850630	Single	0.0230888	NULL	RF	89.05	Large intestine	No
ERX3462569	19980420	Single	0.0225437	NULL	RF	91.89	Large intestine	No
SRX8413041	24640845	Paired	0.0347845	0.964289	RF	76.42	PBMCs	No
SRX8413043	30561960	Paired	0.0407561	0.957213	RF	77.32	PBMCs	No
SRX8413044	24067922	Paired	0.0347085	0.963674	RF	76.34	PBMCs	No
SRX9788368	40548463	Paired	0.0840007	0.916781	RF	90.68	Uterus	No
SRX9788369	39553589	Paired	0.070857	0.92979	RF	90.36	Uterus	No
SRX8098000	50964951	Paired	0.0428483	0.956431	RF	86.54	Adipose	No
SRX8098001	50736142	Paired	0.0268738	0.973003	RF	82.83	Adipose	No
SRX8098002	47882898	Paired	0.0389709	0.961581	RF	78.39	Muscle	No
SRX8098003	45771503	Paired	0.0414078	0.959077	RF	79.14	Muscle	No
SRX8098004	39824122	Paired	0.0379369	0.962529	RF	74.88	Muscle	No
SRX8098005	47918195	Paired	0.030197	0.970673	RF	79.5	Liver	No
SRX8098006	45549808	Paired	0.0349393	0.965344	RF	73.97	Liver	No
SRX8098007	45272444	Paired	0.0355403	0.964254	RF	78.05	Liver	No
SRX8098008	44387819	Paired	0.0950357	0.903439	RF	82.75	Lung	No
ERX3462570	18659093	Single	0.0177718	NULL	RF	89.99	Liver	No
SRX8098009	53554138	Paired	0.0375276	0.96552	RF	80.24	Adipose	No
ERX3462571	19729589	Single	0.0172689	NULL	RF	91.35	Liver	No
ERX3462572	19751117	Single	0.018729	NULL	RF	91.7	Liver	No
ERX3462573	18936994	Single	0.017759	NULL	RF	91.81	Liver	No
ERX3462574	19470416	Single	0.0168741	NULL	RF	92.21	Liver	No
ERX3462575	19106704	Single	0.0192567	NULL	RF	90.22	Liver	No
ERX3462576	19322900	Single	0.0190014	NULL	RF	90.85	Liver	No
ERX3462577	18891303	Single	0.0196583	NULL	RF	90.31	Liver	No
ERX3462578	19711704	Single	0.0165562	NULL	RF	92.06	Liver	No
ERX3462579	18586238	Single	0.0189435	NULL	RF	90.18	Liver	No
SRX8345904	59370120	Paired	0.0231431	0.977734	RF	91.03	Ovary	No
SRX8345905	65994969	Paired	0.0211596	0.979857	RF	89.78	Ovary	No
SRX9788370	46321801	Paired	0.0833822	0.917149	RF	91	Uterus	No
SRX9788371	46600403	Paired	0.067184	0.933772	RF	90.7	Uterus	No
SRX9788372	39607279	Paired	0.0968826	0.903624	RF	89.39	Uterus	No
SRX9788373	40697248	Paired	0.0908586	0.909355	RF	90.43	Uterus	No
SRX9788374	47233436	Paired	0.0872287	0.913102	RF	90.34	Uterus	No
SRX9788375	44078710	Paired	0.0899963	0.910432	RF	90.31	Uterus	No
SRX8098010	51251280	Paired	0.044583	0.956614	RF	79.15	Adipose	No
SRX8098011	53642941	Paired	0.0425871	0.956332	RF	76.03	Adipose	No
SRX8098012	45369475	Paired	0.0541044	0.945242	RF	82.55	Brain	No
SRX8098013	46855331	Paired	0.0444837	0.954738	RF	79.71	Brain	No
SRX8098014	45660409	Paired	0.0355575	0.964435	RF	77.64	Brain	No
SRX8098015	51849434	Paired	0.036229	0.963644	RF	79.07	Adipose	No
SRX8098016	49892823	Paired	0.038054	0.961952	RF	77.89	Adipose	No
SRX8098017	39299514	Paired	0.0383387	0.960949	RF	77.31	Adipose	No
SRX8098018	48055884	Paired	0.044704	0.955379	RF	75.63	Muscle	No
ERX3462580	19803071	Single	0.0171996	NULL	RF	91.65	Liver	No
ERX3462581	19469744	Single	0.0200879	NULL	RF	90.98	Liver	No
ERX3462582	19774268	Single	0.0169608	NULL	RF	91.62	Liver	No
ERX3462583	19324256	Single	0.0171048	NULL	RF	91.76	Liver	No
ERX3462584	19309117	Single	0.0182906	NULL	RF	90.05	Liver	No
ERX3462585	19656776	Single	0.0179207	NULL	RF	90.14	Liver	No
ERX3462586	19248790	Single	0.0177964	NULL	RF	91.86	Liver	No
ERX3462587	18736500	Single	0.0194214	NULL	RF	91.6	Liver	No
ERX3462588	19103874	Single	0.0188767	NULL	RF	92.02	Liver	No

ERX3462589	19864795	Single	0.0237614	NULL	RF	85.5	Liver	No
SRX8098020	58504767	Paired	0.0389579	0.961728	RF	70.74	Muscle	No
SRX8098021	57638672	Paired	0.0450347	0.955412	RF	74.89	Muscle	No
SRX8098022	48852008	Paired	0.046747	0.950687	RF	76.58	Brain	No
SRX8098023	49075589	Paired	0.0429438	0.954412	RF	80.46	Brain	No
SRX8098024	56779470	Paired	0.0430935	0.955161	RF	78.75	Brain	No
SRX8098025	48380434	Paired	0.0473754	0.952513	RF	74.99	Muscle	No
SRX8098026	55331810	Paired	0.0487318	0.9516	RF	76.02	Muscle	No
SRX8098027	52681313	Paired	0.0400981	0.960232	RF	78.17	Muscle	No
SRX8098028	48299903	Paired	0.0414705	0.957359	RF	80.04	Liver	No
ERX3462590	19700429	Single	0.0185176	NULL	RF	91.8	Liver	No
SRX8098029	48484399	Paired	0.0279218	0.972531	RF	74.98	Liver	No
ERX3462591	19247671	Single	0.0191051	NULL	RF	91.04	Liver	No
ERX3462592	19719944	Single	0.0199093	NULL	RF	89.89	Liver	No
ERX3462593	19663558	Single	0.0176642	NULL	RF	90.96	Liver	No
ERX3462594	18892042	Single	0.0183898	NULL	RF	90.25	Liver	No
ERX3462595	19631274	Single	0.0177665	NULL	RF	90.4	Liver	No
ERX3462596	19985621	Single	0.0186187	NULL	RF	90.97	Liver	No
ERX3462597	19956913	Single	0.0192809	NULL	RF	90.65	Liver	No
ERX3462598	19659504	Single	0.0188299	NULL	RF	90.93	Liver	No
ERX3462599	19968607	Single	0.0183119	NULL	RF	90.75	Liver	No
SRX3036013	12567425	Paired	0.0253766	0.974214	RF	83.83	Liver	No
SRX3036014	12682732	Paired	0.0227258	0.977326	RF	85.1	Liver	No
SRX3036015	19171351	Paired	0.0199737	0.980489	RF	90.86	Liver	No
SRX3036016	16198153	Paired	0.0200737	0.980018	RF	84.68	Liver	No
SRX3036017	14865659	Paired	0.021733	0.97808	RF	77.93	Liver	No
SRX3036018	16729831	Paired	0.0191517	0.980778	RF	85.78	Liver	No
SRX8098030	46502600	Paired	0.0852422	0.914822	RF	82.84	Spleen	No
SRX8098031	53773771	Paired	0.0346154	0.965621	RF	79.72	Liver	No
SRX8098032	48760199	Paired	0.0609632	0.937655	RF	82.35	Adipose	No
SRX8098033	50747184	Paired	0.0431991	0.956796	RF	76.13	Adipose	No
SRX8098034	54624579	Paired	0.0669862	0.931172	RF	79.31	Adipose	No
SRX8098035	45438184	Paired	0.0334991	0.966182	RF	71.86	Brain	No
SRX8098036	46038710	Paired	0.0404367	0.95884	RF	75.15	Brain	No
SRX8098037	49284227	Paired	0.0479483	0.951076	RF	75.56	Brain	No
SRX8098038	55022013	Paired	0.0395279	0.959993	RF	74.77	Adipose	No
SRX8098039	58105180	Paired	0.0397359	0.960334	RF	77.06	Adipose	No
SRX8098040	57891346	Paired	0.0382081	0.960881	RF	78.91	Adipose	No
SRX8098042	52099504	Paired	0.0451295	0.955555	RF	77.98	Muscle	No
SRX8098043	51604616	Paired	0.0434932	0.956577	RF	74.84	Muscle	No
SRX8098045	50372016	Paired	0.040268	0.960132	RF	74.97	Muscle	No
SRX8098046	54423012	Paired	0.0555083	0.942554	RF	75.63	Brain	No
SRX8098048	52429819	Paired	0.0436851	0.953946	RF	75.84	Brain	No
SRX8098049	46721897	Paired	0.0452742	0.952227	RF	79.11	Brain	No
SRX8098050	53404460	Paired	0.0476672	0.951521	RF	83.9	Muscle	No
SRX8098051	52822253	Paired	0.0383926	0.96168	RF	73.61	Muscle	No
SRX8098052	38717546	Paired	0.0847562	0.911476	RF	80.54	Kidney	No
SRX8098053	49948614	Paired	0.0439824	0.956095	RF	76.05	Muscle	No
SRX8098054	45168495	Paired	0.0308007	0.969282	RF	75.6	Liver	No
SRX8098056	50298836	Paired	0.0297579	0.970419	RF	74.91	Liver	No
SRX8098057	43649711	Paired	0.0282627	0.972252	RF	81.02	Liver	No
SRX8098058	59093824	Paired	0.0535327	0.945099	RF	80.84	Adipose	No
SRX8098059	48834667	Paired	0.0536526	0.945334	RF	83.42	Adipose	No
SRX8098060	49305013	Paired	0.0376998	0.961387	RF	72	Adipose	No
SRX8098061	49950673	Paired	0.0384209	0.961089	RF	77.65	Brain	No
SRX8098062	48590652	Paired	0.0355547	0.963914	RF	68.44	Brain	No
SRX8098064	45986511	Paired	0.0347602	0.965016	RF	76.86	Brain	No
SRX8098065	55199402	Paired	0.0464465	0.952888	RF	79.89	Adipose	No

SRX8098066	53471272	Paired	0.0282051	0.972232	RF	66.68	Adipose	No
SRX8098068	44585869	Paired	0.0403486	0.958287	RF	73.37	Adipose	No
SRX8098069	51958084	Paired	0.0465658	0.952791	RF	87.58	Muscle	No
SRX8189264	44434244	Paired	0.0536733	0.945477	RF	83.1	Testis	No
SRX8189265	52109200	Paired	0.10286	0.892837	RF	84.66	Testis	No
SRX8189267	38758977	Paired	0.175629	0.819232	RF	83.15	Testis	No
SRX8098070	52859108	Paired	0.0430598	0.956124	RF	88.4	Muscle	No
SRX8098071	47260074	Paired	0.0443315	0.956008	RF	86.07	Muscle	No
SRX8098072	46151294	Paired	0.0579579	0.940607	RF	87.93	Brain	No
SRX8098073	40491514	Paired	0.0541602	0.945261	RF	87.23	Brain	No
SRX8098074	50706962	Paired	0.0415995	0.959405	RF	76.57	Muscle	No
SRX8098075	46314277	Paired	0.0512324	0.946595	RF	88.94	Muscle	No
SRX8098076	46959401	Paired	0.0461464	0.950828	RF	89.89	Muscle	No
SRX8098077	46082183	Paired	0.0480741	0.951718	RF	86.11	Muscle	No
SRX8098078	44326008	Paired	0.0360114	0.962729	RF	88.79	Liver	No
SRX8098079	49419214	Paired	0.0299872	0.969191	RF	86.64	Liver	No
SRX1792839	9022575	Paired	0.958612	0.0394614	FR	80.86	Muscle	No
SRX5412700	51069296	Paired	0.0213548	0.97985	RF	76.57	Spleen	No
SRX5412701	52962304	Paired	0.0226998	0.978507	RF	75.74	Spleen	No
SRX5412702	50374411	Paired	0.0669018	0.933924	RF	89.9	Lymph node	No
SRX5412703	53598177	Paired	0.0569884	0.943963	RF	90.37	Lymph node	No
SRX5412704	50017041	Paired	0.0392052	0.961544	RF	87.84	Spleen	No
SRX5412705	58007652	Paired	0.0543236	0.946016	RF	90.04	Spleen	No
SRX5412706	46128798	Paired	0.0174044	0.983422	RF	70.8	Spleen	No
SRX5412707	47887945	Paired	0.0564001	0.944683	RF	87.39	Spleen	No
SRX8098080	48060946	Paired	0.0310214	0.968271	RF	87.14	Liver	No
SRX8098081	49313121	Paired	0.0545196	0.945012	RF	88.32	Adipose	No
SRX8098082	55582672	Paired	0.0597215	0.939634	RF	88.35	Adipose	No
SRX8098083	55300821	Paired	0.0614703	0.937856	RF	85.87	Brain	No
SRX8098084	57217718	Paired	0.0679722	0.931432	RF	85.81	Brain	No
SRX8098085	46084221	Paired	0.0394924	0.961261	RF	78.4	Muscle	No
SRX8098086	51365428	Paired	0.0593222	0.940624	RF	84.65	Brain	No
SRX8098087	57484682	Paired	0.0455697	0.953573	RF	84.32	Adipose	No
SRX8098088	53021980	Paired	0.051104	0.947757	RF	90.15	Adipose	No
SRX1792840	9252211	Paired	0.963663	0.0348454	FR	80.88	Muscle	No
SRX1792841	9956052	Paired	0.957335	0.036252	FR	80.78	Muscle	No
SRX1792842	10400806	Paired	0.96187	0.0357645	FR	80.88	Muscle	No
SRX1792843	9664258	Paired	0.965145	0.0319085	FR	81.63	Muscle	No
SRX1792844	10643326	Paired	0.962418	0.0348044	FR	81.19	Muscle	No
SRX8098090	48129096	Paired	0.0334089	0.966069	RF	83.6	Muscle	No
SRX8098091	45973752	Paired	0.0260713	0.97475	RF	86.31	Muscle	No
SRX8098092	47378454	Paired	0.0250467	0.975855	RF	86.85	Muscle	No
SRX8098093	48077520	Paired	0.0591494	0.939718	RF	83.57	Brain	No
SRX8098094	46302073	Paired	0.0614341	0.936581	RF	86.29	Brain	No
SRX8098095	56198876	Paired	0.0420143	0.954425	RF	88.28	Brain	No
SRX6071093	52517614	Paired	0.0209619	0.979647	RF	81.24	Macrophage	No
SRX6071094	46061696	Paired	0.0229494	0.977651	RF	81.97	Macrophage	No
SRX6071095	51713642	Paired	0.0221518	0.978374	RF	81.83	Macrophage	No
SRX6071096	48574230	Paired	0.0237059	0.976729	RF	83.06	Macrophage	No
SRX6071097	53912040	Paired	0.0192451	0.981247	RF	80	Macrophage	No
SRX6071098	57854246	Paired	0.0208313	0.979765	RF	81.09	Macrophage	No
SRX8144833	25698965	Paired	0.0273465	0.970369	RF	90.73	PBMCs	No
SRX8144834	22143672	Paired	0.0288656	0.967355	RF	90.71	PBMCs	No
SRX1136473	12960662	Paired	0.89127	0.103871	FR	84.63	Brain	No
SRX8144835	24488761	Paired	0.0296848	0.967344	RF	91.31	PBMCs	No
SRX1136474	10388249	Paired	0.848065	0.143938	FR	75.12	Brain	No
SRX8144836	28158403	Paired	0.029549	0.968626	RF	92	PBMCs	No
SRX1136475	12919626	Paired	0.878249	0.117775	FR	84.83	Brain	No

SRX8144837	24579256	Paired	0.0289614	0.968523	RF	92.01	PBMCs	No
SRX1136476	23545212	Paired	0.864077	0.129193	FR	85.29	Brain	No
SRX8144838	25235417	Paired	0.0298197	0.96937	RF	92.19	PBMCs	No
SRX1136477	26279388	Paired	0.857242	0.131472	FR	85.91	Brain	No
SRX8144839	30748841	Paired	0.026537	0.973092	RF	92.65	PBMCs	No
SRX1136478	4457551	Paired	0.810628	0.167992	FR	79.18	Brain	No
SRX1136479	3554078	Paired	0.82852	0.160706	FR	81.12	Brain	No
SRX3556732	22401171	Paired	0.0237971	0.977361	RF	88.59	Ovary	No
SRX3556733	18245208	Paired	0.0247646	0.976464	RF	89.17	Ovary	No
SRX3556734	19647669	Paired	0.0237754	0.977307	RF	87.96	Ovary	No
SRX3556735	19232638	Paired	0.0242223	0.976728	RF	88.7	Ovary	No
SRX3556736	21279430	Paired	0.0233352	0.977754	RF	88.9	Ovary	No
SRX3556737	19729003	Paired	0.0236471	0.977462	RF	89.54	Ovary	No
SRX3556738	22579040	Paired	0.0226815	0.978604	RF	89.98	Ovary	No
SRX3556739	23590241	Paired	0.0229708	0.978166	RF	84.23	Ovary	No
SRX5035700	20343881	Paired	0.0373277	0.962872	RF	90.19	Small intestine	No
SRX5035701	25681036	Paired	0.0298528	0.972482	RF	91.68	Small intestine	No
SRX5035702	23239963	Paired	0.0280866	0.973794	RF	90.54	Small intestine	No
SRX5035704	11924745	Paired	0.0387271	0.962444	RF	88.32	Small intestine	No
SRX5035705	16146236	Paired	0.0419558	0.957405	RF	83.51	Small intestine	No
SRX5035706	37955775	Paired	0.0314278	0.97038	RF	90.57	Small intestine	No
SRX5035707	36648208	Paired	0.0291643	0.973344	RF	91.67	Small intestine	No
SRX5035708	17419307	Paired	0.0370593	0.963985	RF	80.55	Small intestine	No
SRX5035709	37047006	Paired	0.028854	0.973005	RF	91.07	Small intestine	No
SRX8144840	26986575	Paired	0.0284795	0.971181	RF	92.73	PBMCs	No
SRX8144841	38020826	Paired	0.0274753	0.973056	RF	91.26	PBMCs	No
SRX8144842	28325252	Paired	0.0281439	0.97166	RF	92.79	PBMCs	No
SRX1136481	15093522	Paired	0.855535	0.122569	FR	81.84	Brain	No
SRX8144843	25266923	Paired	0.0291625	0.970993	RF	92.95	PBMCs	No
SRX8144844	26729346	Paired	0.0296002	0.969763	RF	92.38	PBMCs	No
SRX8144845	21772199	Paired	0.0296964	0.968953	RF	92.09	PBMCs	No
SRX8144846	23454450	Paired	0.0315659	0.966341	RF	91.18	PBMCs	No
SRX8144847	23270134	Paired	0.0270931	0.973412	RF	92.65	PBMCs	No
SRX8144848	27692758	Paired	0.0309159	0.968924	RF	92.35	PBMCs	No
SRX8144849	36538798	Paired	0.0320541	0.968227	RF	91.79	PBMCs	No
SRX5702486	69919529	Paired	0.0254706	0.974222	RF	80.08	Muscle	No
SRX5702487	72735982	Paired	0.0226272	0.977078	RF	81.69	Muscle	No
SRX5702488	66911753	Paired	0.0257996	0.973686	RF	77.46	Muscle	No
SRX5702489	62138574	Paired	0.0261244	0.973507	RF	80.17	Muscle	No
SRX3556740	23714494	Paired	0.0232119	0.977981	RF	89.86	Ovary	No
SRX3556741	22638358	Paired	0.02329	0.97791	RF	89.73	Ovary	No
SRX3556742	20329405	Paired	0.0240342	0.97733	RF	89.3	Ovary	No
SRX3556743	20805446	Paired	0.0239338	0.977249	RF	88.87	Ovary	No
SRX5035710	25676315	Paired	0.0335127	0.968414	RF	90.2	Small intestine	No
SRX5035711	16057186	Paired	0.0359786	0.965756	RF	89.09	Small intestine	No
SRX5035712	32528425	Paired	0.0262882	0.983689	RF	91.94	Small intestine	No
SRX5035713	10880036	Paired	0.0351041	0.966362	RF	89.88	Small intestine	No
SRX5035714	14872583	Paired	0.0333775	0.967531	RF	89.77	Small intestine	No
SRX5035715	16531679	Paired	0.0378183	0.96417	RF	90.13	Small intestine	No
SRX5035716	23144420	Paired	0.0354575	0.965337	RF	88.11	Small intestine	No
SRX5035717	18001305	Paired	0.0333233	0.968204	RF	89.83	Small intestine	No
SRX5035718	24640291	Paired	0.0386124	0.970819	RF	89.33	Small intestine	No
SRX5035719	23208026	Paired	0.0353062	0.966512	RF	89.98	Small intestine	No
SRX8144850	28872964	Paired	0.0329152	0.967325	RF	91.64	PBMCs	No
SRX8144851	31106321	Paired	0.0329588	0.967312	RF	90.49	PBMCs	No
SRX8144852	26873554	Paired	0.0389733	0.960147	RF	90.84	PBMCs	No
SRX6486509	19549522	Paired	0.0176754	0.982735	RF	89.39	Liver	No
SRX8144853	28726846	Paired	0.0390633	0.960728	RF	91.27	PBMCs	No

SRX5702490	54942791	Paired	0.0307327	0.968261	RF	80.51	Muscle	No
SRX8144854	39714573	Paired	0.0384551	0.961576	RF	92.25	PBMCs	No
SRX5702491	74186117	Paired	0.0278522	0.971726	RF	77.37	Muscle	No
SRX8144855	26758518	Paired	0.0296881	0.969286	RF	91.73	PBMCs	No
SRX8144856	30149263	Paired	0.0284846	0.971886	RF	91.72	PBMCs	No
SRX8144857	33146802	Paired	0.0259114	0.974669	RF	91.74	PBMCs	No
SRX8144858	23363575	Paired	0.0308817	0.965756	RF	90.58	PBMCs	No
SRX8144859	29340024	Paired	0.0282429	0.971274	RF	92.06	PBMCs	No
SRX5035720	13040597	Paired	0.037324	0.963868	RF	89.83	Small intestine	No
SRX5035721	15697550	Paired	0.0349023	0.966699	RF	89.81	Small intestine	No
SRX5035722	24355196	Paired	0.0327307	0.969211	RF	88.95	Small intestine	No
SRX5035723	19221077	Paired	0.0348891	0.966503	RF	90.11	Small intestine	No
SRX5035724	11750398	Paired	0.0404264	0.964977	RF	87.98	Small intestine	No
SRX5035725	20040706	Paired	0.0370464	0.96457	RF	88.84	Small intestine	No
SRX6486510	19509198	Paired	0.0205349	0.979502	RF	87.89	Liver	No
SRX6486511	17952866	Paired	0.0195939	0.980496	RF	86.77	Liver	No
SRX6486512	14865659	Paired	0.0220226	0.978071	RF	77.93	Liver	No
SRX6486513	16198153	Paired	0.0201392	0.980049	RF	84.68	Liver	No
SRX6486514	19171351	Paired	0.0196361	0.980482	RF	90.86	Liver	No
SRX2863080	47306383	Paired	0.0178386	0.984538	RF	87.41	Muscle	No
SRX2863081	52060319	Paired	0.0182458	0.984955	RF	87.35	Muscle	No
SRX2863082	49102472	Paired	0.0197837	0.984227	RF	89.16	Muscle	No
SRX2863083	57712915	Paired	0.986362	0.0157688	FR	87.07	Muscle	No
SRX2863084	47862277	Paired	0.986697	0.0161352	FR	89.29	Muscle	No
SRX2863085	6087267	Paired	0.97274	0.0284195	FR	80.31	Muscle	No
SRX2863086	41125531	Paired	0.0171236	0.985058	RF	91.23	Muscle	No
SRX2863087	50994230	Paired	0.0165499	0.985777	RF	89.9	Muscle	No
SRX2863088	43784390	Paired	0.0169741	0.98551	RF	91.3	Muscle	No
SRX2863089	47742863	Paired	0.984292	0.0175388	FR	78.56	Muscle	No
SRX3373055	8625008	Paired	0.966528	0.0347539	FR	84.45	Lymph node	No
SRX3373057	12680978	Paired	0.0339613	0.967131	RF	88.5	Lymph node	No
SRX3373058	9662573	Paired	0.969102	0.031906	FR	85.35	Lymph node	No
SRX3373059	10646420	Paired	0.969397	0.0315865	FR	87.23	Lymph node	No
SRX9274489	34098870	Paired	0.0171793	0.984225	RF	87.21	Liver	No
SRX2863090	57691184	Paired	0.98627	0.016087	FR	88.83	Muscle	No
SRX2863091	36656939	Paired	0.982892	0.019088	FR	87.15	Muscle	No
SRX2863092	48023831	Paired	0.0169264	0.985123	RF	89.29	Muscle	No
SRX2863093	43697355	Paired	0.0191211	0.983886	RF	88.78	Muscle	No
SRX2863094	45571675	Paired	0.982206	0.0211994	FR	88.9	Muscle	No
SRX2863095	52260419	Paired	0.982341	0.0206507	FR	89.63	Muscle	No
SRX2863096	53642082	Paired	0.984023	0.0178903	FR	88.86	Muscle	No
SRX2863097	46958602	Paired	0.0178597	0.984879	RF	89.09	Muscle	No
SRX2863098	37273579	Paired	0.984883	0.0181195	FR	89.95	Muscle	No
SRX2863099	48608537	Paired	0.985224	0.017704	FR	88.03	Muscle	No
SRX2408798	16043825	Paired	0.988839	0.0139527	FR	62.61	Muscle	No
SRX2408799	18090458	Paired	0.989144	0.0134438	FR	63.4	Muscle	No
SRX3373060	9645312	Paired	0.940208	0.0713231	FR	64.43	Whole blood	No
SRX3373061	8994353	Paired	0.0332125	0.967713	RF	87.84	Lymph node	No
SRX3373062	10188437	Paired	0.930536	0.0834996	FR	62.74	Whole blood	No
SRX3373063	11977210	Paired	0.0336433	0.967287	RF	88.17	Lymph node	No
SRX3373064	10773749	Paired	0.0308505	0.970035	RF	88.25	Lymph node	No
SRX3373065	10489093	Paired	0.968659	0.032409	FR	86.53	Lymph node	No
SRX3373067	10655185	Paired	0.967938	0.0330246	FR	85.97	Lymph node	No
SRX3373068	8878660	Paired	0.0338767	0.967121	RF	89.23	Lymph node	No
SRX9274490	33319291	Paired	0.0173378	0.983657	RF	86.39	Liver	No
SRX3373069	10717197	Paired	0.966619	0.0344196	FR	86.38	Lymph node	No
SRX9274499	33866597	Paired	0.0212395	0.979926	RF	84.28	Liver	No
SRX3373070	9937874	Paired	0.0346127	0.966451	RF	86.61	Lymph node	No

SRX3373071	10709958	Paired	0.0323249	0.968713	RF	88.18		Lymph node	No
SRX3373073	11162834	Paired	0.968211	0.0327347	FR	86.25		Lymph node	No
SRX3373074	9476515	Paired	0.967258	0.0336881	FR	87.64		Lymph node	No
SRX3373075	9300754	Paired	0.0330564	0.967806	RF	87.96		Lymph node	No
SRX3373076	10168465	Paired	0.0835953	0.930555	RF	61.2		Whole blood	No
SRX3373077	12502840	Paired	0.0334687	0.967436	RF	88.35		Lymph node	No
SRX3373078	11414607	Paired	0.970135	0.0306585	FR	86.3		Lymph node	No
SRX3373079	10833083	Paired	0.966491	0.0346064	FR	86.67		Lymph node	No
SRX5680321	43531796	Paired	0.0435754	0.955165	RF	83.95		Adipose	No
SRX5680322	41330213	Paired	0.0360837	0.96302	RF	85.03		Adipose	No
SRX5680323	38125830	Paired	0.0216585	0.977567	RF	84.23		Adipose	No
SRX5680324	40327662	Paired	0.021829	0.977323	RF	83.59		Adipose	No
SRX5680325	45965069	Paired	0.0177474	0.981352	RF	87.07		Adipose	No
SRX5680326	51850446	Paired	0.0179636	0.981492	RF	87.64		Adipose	No
SRX3373080	10548789	Paired	0.966588	0.0345276	FR	84.74		Lymph node	No
SRX3373081	10850169	Paired	0.0314997	0.969502	RF	89		Lymph node	No
SRX3373082	9703066	Paired	0.0717068	0.940539	RF	63.93		Whole blood	No
SRX3373083	8992403	Paired	0.96675	0.0345154	FR	84.77		Lymph node	No
SRX3373085	13266851	Paired	0.967232	0.0338818	FR	87.83		Lymph node	No
SRX3373086	9994441	Paired	0.96916	0.0317668	FR	85.61		Lymph node	No
SRX3373087	10299893	Paired	0.966391	0.0345805	FR	84.41		Lymph node	No
SRX3373088	10816393	Paired	0.96939	0.0314904	FR	87.22		Lymph node	No
SRX3373089	10465556	Paired	0.0323967	0.96854	RF	88.07		Lymph node	No
SRX5277051	26927086	Paired	0.0231177	0.976567	RF	88.4		Cartilage	No
SRX5277052	24726876	Paired	0.0242101	0.975448	RF	90.6		Cartilage	No
SRX5277053	23888263	Paired	0.0224783	0.977267	RF	87.77		Cartilage	No
SRX5277054	24505142	Paired	0.0243948	0.975095	RF	88.22		Cartilage	No
SRX5277055	20043393	Paired	0.0214571	0.976956	RF	87.01		Cartilage	No
SRX5277056	29579129	Paired	0.0322755	0.966643	RF	85.22		Cartilage	No
SRX5277057	24656225	Paired	0.0227418	0.975498	RF	88.98		Cartilage	No
SRX5277058	26312220	Paired	0.0359415	0.969034	RF	86.88		Cartilage	No
SRX5277059	23516201	Paired	0.0318226	0.973213	RF	87.01		Cartilage	No
SRX3764987	34404779	Paired	0.0208784	0.980096	RF	91.5		PK15	No
SRX3764988	35249931	Paired	0.0199454	0.980642	RF	90.59		PK15	No
SRX3764989	38241458	Paired	0.0213614	0.979714	RF	91.85		PK15	No
SRX3373091	9915811	Paired	0.0835771	0.929653	RF	63.32		Whole blood	No
SRX3373092	9055119	Paired	0.0333352	0.967524	RF	87.91		Lymph node	No
SRX3373093	10837421	Paired	0.0307952	0.969913	RF	88.31		Lymph node	No
SRX3373094	12625673	Paired	0.967352	0.0334688	FR	85.8		Lymph node	No
SRX3373095	9773901	Paired	0.968909	0.0318792	FR	85.23		Lymph node	No
SRX3373096	12763853	Paired	0.0340006	0.966932	RF	88.66		Lymph node	No
SRX3373098	8891579	Paired	0.966599	0.0344057	FR	84.38		Lymph node	No
SRX3373099	10636427	Paired	0.966357	0.0345575	FR	86.35		Lymph node	No
SRX3345113	29960751	Paired	0.986056	0.017034	FR	71.48		Ovary	No
SRX3764990	47735052	Paired	0.0220747	0.97902	RF	90.67		PK15	No
SRX3764991	40372078	Paired	0.0224477	0.97859	RF	91.74		PK15	No
SRX3764992	37999812	Paired	0.0222832	0.978746	RF	91.66		PK15	No
SRX8417108	36893511	Paired	0.0466203	0.972864	RF	76.31		PBMCs	No
SRX8417109	35543342	Paired	0.0480075	0.971659	RF	73		PBMCs	No
SRX8241341	21725246	Paired	0.0229724	0.976778	RF	86.64		PK15	No
SRX8241343	22483880	Paired	0.0231923	0.97676	RF	86.01		PK15	No
SRX8241344	23354083	Paired	0.0224808	0.977782	RF	84.53		PK15	No
SRX8241345	23257284	Paired	0.0234076	0.977229	RF	85.76		PK15	No
SRX8241346	24722968	Paired	0.0247175	0.975902	RF	84.79		PK15	No
SRX8241347	23283505	Paired	0.024184	0.976427	RF	86.38		PK15	No
ERX4362359	30697732	Paired	0.0749531	0.923986	RF	90.14		Ovary	No
SRX8413224	26151881	Paired	0.0309557	0.967951	RF	75.12		PBMCs	No
SRX8413225	25021389	Paired	0.0388187	0.959182	RF	80.5		PBMCs	No

SRX8413226	25801243	Paired	0.0389048	0.959704	RF	81.31	PBMCs	No
SRX8413227	25118262	Paired	0.0309875	0.967394	RF	73.95	PBMCs	No
SRX8417110	41545543	Paired	0.0471396	0.971163	RF	72.48	PBMCs	No
SRX8417111	40049308	Paired	0.0487335	0.969643	RF	73.06	PBMCs	No
ERX4362360	36358417	Paired	0.100807	0.896179	RF	89.85	Ovary	No
ERX4362361	33933938	Paired	0.0578929	0.942314	RF	90.01	Ovary	No
ERX4362362	30120871	Paired	0.124633	0.872399	RF	77.05	Ovary	No
ERX4362363	28442164	Paired	0.0390083	0.961224	RF	87.56	Ovary	No
ERX4362364	34547680	Paired	0.045033	0.955137	RF	89.75	Ovary	No
ERX4362365	30792796	Paired	0.0339549	0.965804	RF	84.8	Ovary	No
ERX4362366	31621437	Paired	0.0340828	0.965654	RF	88.89	Ovary	No
ERX4362367	33835403	Paired	0.034336	0.964491	RF	87.29	Ovary	No
SRX8418467	29369118	Paired	0.0394624	0.959449	RF	84.12	PBMCs	No
SRX8418468	30264850	Paired	0.0396894	0.959776	RF	83.24	PBMCs	No
SRX8418469	25639105	Paired	0.0323066	0.966778	RF	78.17	PBMCs	No
SRX9703900	36810043	Paired	0.0290177	0.971729	RF	82.95	Muscle	No
SRX9703901	45132834	Paired	0.0307213	0.969717	RF	88.2	Muscle	No
SRX9703902	46015706	Paired	0.0310373	0.969779	RF	85.53	Muscle	No
SRX8418470	26614634	Paired	0.0321819	0.967338	RF	78.05	PBMCs	No
SRX8413261	26911455	Paired	0.0291786	0.970149	RF	78.67	PBMCs	No
SRX8413262	36694063	Paired	0.0307723	0.968443	RF	66.76	PBMCs	No
SRX8413263	38016799	Paired	0.0306996	0.969165	RF	67.23	PBMCs	No
SRX8413264	26155078	Paired	0.0291822	0.96951	RF	78.27	PBMCs	No
SRX8363587	24980346	Paired	0.0398984	0.958607	RF	80.99	PBMCs	No
SRX8363588	26041275	Paired	0.0398934	0.959093	RF	81.29	PBMCs	No
ERX3013252	28056964	Single	0.0587368	NULL	RF	72.35	Whole blood	No
ERX3013253	30853377	Single	0.0628563	NULL	RF	63.08	Whole blood	No
ERX3013255	28194186	Single	0.0583943	NULL	RF	72.26	Whole blood	No
ERX3013256	32697108	Single	0.0454302	NULL	RF	67.37	Whole blood	No
ERX3013257	39390582	Single	0.0815883	NULL	RF	62.34	Whole blood	No
ERX3013258	35996185	Single	0.0603223	NULL	RF	71.39	Whole blood	No
ERX3013259	32912463	Single	0.0540155	NULL	RF	76.75	Whole blood	No
SRX9435556	29693359	Single	0.0453989	NULL	RF	92.18	Skin	No
SRX9435557	31650398	Single	0.0528845	NULL	RF	91.15	Skin	No
SRX9435558	32276269	Single	0.020902	NULL	RF	94.38	Skin	No
ERX3013266	32083034	Single	0.0523119	NULL	RF	67.97	Whole blood	No
ERX3013267	34464819	Single	0.0613289	NULL	RF	70.53	Whole blood	No
ERX3013268	23325357	Single	0.0459726	NULL	RF	69.3	Whole blood	No
SRX12132500	65889450	Paired	0.0387386	0.959925	RF	86.49	Muscle	No
SRX12132501	66693131	Paired	0.0365289	0.962424	RF	87.6	Muscle	No
SRX12132502	74304541	Paired	0.0393994	0.959678	RF	88.18	Muscle	No
SRX12132503	51902191	Paired	0.0400792	0.957122	RF	89.01	Muscle	No
SRX12132504	50451242	Paired	0.0410281	0.956909	RF	86.62	Muscle	No
SRX12132505	48871364	Paired	0.0383054	0.960164	RF	84.57	Muscle	No
SRX12132506	67657176	Paired	0.0389267	0.959688	RF	87.78	Muscle	No
SRX12132507	59218265	Paired	0.0370318	0.961431	RF	88.32	Muscle	No
SRX12132508	55959783	Paired	0.038848	0.959142	RF	85.97	Muscle	No
SRX12132509	61889937	Paired	0.0370368	0.961423	RF	87.51	Muscle	No
ERX3013272	29227993	Single	0.0649027	NULL	RF	64.8	Whole blood	No
ERX3013273	27472531	Single	0.0483852	NULL	RF	62.13	Whole blood	No
ERX3013274	29048615	Single	0.06376	NULL	RF	61.44	Whole blood	No
ERX3013279	28779296	Single	0.0558079	NULL	RF	69.49	Whole blood	No
SRX12132510	63953355	Paired	0.0382185	0.959894	RF	85.72	Muscle	No
SRX12132511	68018200	Paired	0.0404738	0.957631	RF	85.58	Muscle	No
SRX12132512	60177655	Paired	0.0400045	0.957787	RF	87.42	Muscle	No
SRX12132513	74587036	Paired	0.0375908	0.960758	RF	88.37	Muscle	No
SRX12132514	67736690	Paired	0.0353016	0.96339	RF	87.28	Muscle	No
SRX12132515	67314477	Paired	0.0420516	0.955503	RF	87.31	Muscle	No

SRX10957682	33674770	Paired	0.0463912	0.952547	RF	75.55	Artery	No
SRX10957683	29131356	Paired	0.0459455	0.955753	RF	82.89	Artery	No
SRX10957684	33183733	Paired	0.0368164	0.964125	RF	82.25	Artery	No
ERX3013280	39498784	Single	0.0515032	NULL	RF	65.4	Whole blood	No
SRX10957685	27684595	Paired	0.0483785	0.95223	RF	67.34	Artery	No
SRX10957686	20027948	Paired	0.0484179	0.953819	RF	87.04	Artery	No
SRX10957687	27969878	Paired	0.0329141	0.968106	RF	82.1	Artery	No
ERX3013283	28096104	Single	0.0632527	NULL	RF	63.3	Whole blood	No
SRX10957688	29236519	Paired	0.0276294	0.9727	RF	87.92	Artery	No
SRX10957689	31330305	Paired	0.036068	0.964129	RF	77.66	Artery	No
ERX3013285	30154635	Single	0.0665957	NULL	RF	64.24	Whole blood	No
ERX3013286	29302111	Single	0.051246	NULL	RF	60.75	Whole blood	No
SRX2327299	53450047	Paired	0.0265363	0.970634	RF	78.64	Macrophage	No
ERX2261182	11302510	Single	0.899299	NULL	FR	77.6	Uterus	No
ERX2261183	10199588	Single	0.88644	NULL	FR	78.75	Uterus	No
ERX2261184	13014902	Single	0.895076	NULL	FR	81.22	Uterus	No
ERX2261185	10491088	Single	0.881158	NULL	FR	75.73	Uterus	No
ERX2261186	11627819	Single	0.869186	NULL	FR	77.1	Uterus	No
ERX2261187	13502282	Single	0.892725	NULL	FR	81.36	Uterus	No
ERX2261188	12298419	Single	0.901753	NULL	FR	80.17	Uterus	No
ERX2261189	11769968	Single	0.881748	NULL	FR	79.05	Uterus	No
SRX9786442	32840064	Single	0.0968811	NULL	RF	82.75	Heart	No
SRX9786443	32887570	Single	0.101195	NULL	RF	81.3	Heart	No
SRX9786444	37023804	Single	0.0881555	NULL	RF	82.21	Heart	No
SRX9786445	22156678	Single	0.0903558	NULL	RF	77.98	Heart	No
SRX9786446	25415597	Single	0.0811753	NULL	RF	85.57	Heart	No
SRX9786447	34349286	Single	0.0811556	NULL	RF	83.17	Heart	No
SRX9786448	23770381	Single	0.0673885	NULL	RF	86.77	Heart	No
SRX10957690	34989298	Paired	0.0625955	0.935758	RF	77.72	Artery	No
SRX9786449	24589867	Single	0.0928994	NULL	RF	83.5	Heart	No
SRX10957691	36612936	Paired	0.0403805	0.95859	RF	78.9	Artery	No
SRX10957692	35639740	Paired	0.0398936	0.954494	RF	90.67	Artery	No
ERX3013292	39478266	Single	0.0499153	NULL	RF	66.02	Whole blood	No
ERX3013293	39189772	Single	0.0713861	NULL	RF	61.1	Whole blood	No
ERX2261190	11401151	Single	0.879641	NULL	FR	79.76	Uterus	No
ERX2261191	12345086	Single	0.895017	NULL	FR	80.02	Uterus	No
ERX2261192	11700158	Single	0.901407	NULL	FR	81.28	Uterus	No
ERX2261193	11077341	Single	0.879705	NULL	FR	80.37	Uterus	No
ERX2261194	12310681	Single	0.886491	NULL	FR	78.68	Uterus	No
ERX2261195	11642892	Single	0.893697	NULL	FR	82.07	Uterus	No
ERX2261196	11437602	Single	0.887666	NULL	FR	80.25	Uterus	No
ERX2261197	10551441	Single	0.876553	NULL	FR	77.26	Uterus	No
SRX7101794	46336399	Paired	0.0417054	0.958373	RF	79.02	Liver	No
SRX7101795	38394668	Paired	0.0368382	0.963216	RF	88.63	Liver	No
SRX9786450	33218908	Single	0.0964264	NULL	RF	80.73	Heart	No
SRX7101796	16708771	Paired	0.0432311	0.956966	RF	80.08	Liver	No
SRX9786451	22893993	Single	0.0862393	NULL	RF	85.68	Heart	No
SRX7101797	19678823	Paired	0.0409952	0.958711	RF	81.5	Liver	No
SRX9786452	33711197	Single	0.0699451	NULL	RF	84.56	Heart	No
SRX7101798	23809059	Paired	0.0362539	0.964551	RF	83.61	Liver	No
SRX9786453	24261848	Single	0.0685187	NULL	RF	86.33	Heart	No
SRX7101799	15272253	Paired	0.0371007	0.962998	RF	84.8	Liver	No
SRX9786454	35797530	Single	0.0884686	NULL	RF	81.52	Heart	No
SRX9786455	33748119	Single	0.0930245	NULL	RF	79.44	Heart	No
SRX9786456	23909864	Single	0.0786217	NULL	RF	85.18	Heart	No
SRX10176147	30023938	Paired	0.0363104	0.958814	RF	77.4	Heart	No
SRX10176148	34412582	Paired	0.041591	0.952847	RF	77.91	Heart	No
SRX10176149	34536378	Paired	0.0349547	0.960488	RF	78.53	Heart	No

SRX10176150	35092800	Paired	0.0537576	0.938099	RF	75.87	Heart	No
SRX10176151	32102089	Paired	0.0407787	0.954227	RF	82.34	Heart	No
SRX10176152	33443642	Paired	0.035947	0.959013	RF	80.27	Heart	No
SRX6769851	44606341	Paired	0.0161448	0.98425	RF	89.59	Ovary	No
SRX6769852	42944441	Paired	0.0201374	0.979578	RF	90.06	Ovary	No
SRX6769853	48919308	Paired	0.0211296	0.978852	RF	85.48	Ovary	No
SRX6769854	44721068	Paired	0.0190203	0.980811	RF	91.14	Ovary	No
SRX6769855	42640302	Paired	0.0151052	0.984971	RF	90.59	Ovary	No
SRX6769856	45330281	Paired	0.043666	0.955327	RF	87.2	Ovary	No
SRX1469600	8989032	Single	0.866277	NULL	FR	61.92	3D4/31	No
SRX1469601	9442777	Single	0.836122	NULL	FR	61.8	3D4/31	No
SRX1469602	16721155	Single	0.888598	NULL	FR	66.1	3D4/31	No
SRX1469603	15060281	Single	0.86268	NULL	FR	67.09	3D4/31	No
SRX1469606	10390352	Single	0.803928	NULL	FR	61.84	3D4/31	No
SRX1469609	9267149	Single	0.850964	NULL	FR	63.39	3D4/31	No
SRX10517380	23629367	Paired	0.0215564	0.979107	RF	94.7	Embryo	No
SRX10517381	25525035	Paired	0.0229067	0.978023	RF	94.45	Embryo	No
SRX10517382	26274217	Paired	0.02131	0.97955	RF	94.77	Embryo	No
SRX10517383	24479984	Paired	0.0212155	0.979442	RF	94.85	Embryo	No
SRX10517384	24661514	Paired	0.022763	0.978206	RF	94.31	Embryo	No
SRX10517385	25096558	Paired	0.0222045	0.978455	RF	94.4	Embryo	No
SRX1469610	10271786	Single	0.899704	NULL	FR	62.33	3D4/31	No
SRX1469611	15681215	Single	0.914507	NULL	FR	69.33	3D4/31	No
SRX1469612	17666223	Single	0.878029	NULL	FR	69.05	3D4/31	No
SRX4801285	45250977	Single	0.104524	NULL	RF	76.47	Brain	No
SRX4801286	20045154	Single	0.0722658	NULL	RF	77.42	Brain	No
SRX4801287	26795313	Single	0.103347	NULL	RF	73.25	Brain	No
SRX4801288	4853814	Single	0.114791	NULL	RF	70.78	Brain	No
SRX4801289	38837701	Single	0.0939645	NULL	RF	80.85	Brain	No
SRX3958358	32016777	Paired	0.985187	0.0161106	FR	93.01	IPEC-J2	No
SRX3958359	67169178	Paired	0.986293	0.0150439	FR	93.3	IPEC-J2	No
SRX4801290	30324431	Single	0.0858556	NULL	RF	74.63	Brain	No
SRX4801291	26782948	Single	0.0843067	NULL	RF	66.77	Brain	No
SRX8609221	47637814	Paired	0.0323439	0.967442	RF	91.12	Testis	No
SRX4801292	19550412	Single	0.0913817	NULL	RF	85.19	Brain	No
SRX8609222	39629624	Paired	0.0333825	0.966276	RF	91.38	Testis	No
SRX4801293	29403324	Single	0.0909987	NULL	RF	62.37	Brain	No
SRX8609223	45832010	Paired	0.0349661	0.96488	RF	91.52	Testis	No
SRX4801294	25297015	Single	0.0940785	NULL	RF	82.48	Brain	No
SRX8609224	39690471	Paired	0.0375746	0.962177	RF	91.45	Testis	No
SRX4801295	13986138	Single	0.0787208	NULL	RF	84.68	Brain	No
SRX8609225	39588979	Paired	0.0357897	0.963762	RF	91.39	Testis	No
SRX8609226	49453523	Paired	0.0361179	0.963352	RF	90.68	Testis	No
SRX3958360	40694258	Paired	0.985519	0.0156335	FR	92.08	IPEC-J2	No
SRX3958361	43978842	Paired	0.985331	0.0159305	FR	92.23	IPEC-J2	No
SRX3958364	31102618	Paired	0.98538	0.0159108	FR	93.44	IPEC-J2	No
SRX3958368	19706825	Paired	0.0178826	0.983448	RF	90.88	IPEC-J2	No
ERX2933209	16906281	Paired	0.0177331	0.982515	RF	89.62	Liver	No
SRX2900808	55192655	Single	0.89596	NULL	FR	63.17	Whole blood	No
SRX3958370	29451292	Paired	0.0170186	0.984155	RF	91.96	IPEC-J2	No
SRX3958371	32574352	Paired	0.0158206	0.985361	RF	94.31	IPEC-J2	No
SRX3958372	21626484	Paired	0.984258	0.017118	FR	92.83	IPEC-J2	No
ERX2933210	13206801	Paired	0.0198905	0.980268	RF	87.94	Liver	No
ERX2933211	16513025	Paired	0.0174596	0.982634	RF	88.49	Liver	No
ERX2933212	16388909	Paired	0.0183345	0.981935	RF	89.39	Liver	No
ERX2933213	15083460	Paired	0.019358	0.980709	RF	87.73	Liver	No
ERX2933214	17926824	Paired	0.0171126	0.983229	RF	89.49	Liver	No
ERX2933215	17340999	Paired	0.0198506	0.980493	RF	89.07	Liver	No

ERX2933216	15139181	Paired	0.0196126	0.980668	RF	88.99	Liver	No
ERX2933217	16323984	Paired	0.0170989	0.982955	RF	89.64	Liver	No
ERX2933218	15379510	Paired	0.0180575	0.982004	RF	89.18	Liver	No
ERX2933219	15707615	Paired	0.0178129	0.982213	RF	88.8	Liver	No
SRX3594502	15642896	Single	0.85977	NULL	FR	70.48	Uterus	No
SRX3594503	24723782	Single	0.887584	NULL	FR	72.04	Uterus	No
SRX3594504	21614971	Single	0.837491	NULL	FR	77.1	Uterus	No
SRX3594506	14347814	Single	0.863325	NULL	FR	73.01	Uterus	No
SRX3594507	23522041	Single	0.887473	NULL	FR	62.53	Uterus	No
SRX3594508	17270469	Single	0.862322	NULL	FR	61.9	Uterus	No
SRX3594509	18124821	Single	0.856255	NULL	FR	70.87	Uterus	No
SRX2900810	40509219	Single	0.905857	NULL	FR	73.95	Whole blood	No
SRX2900811	49641676	Single	0.900624	NULL	FR	72.87	Whole blood	No
SRX2900812	35838908	Single	0.909852	NULL	FR	72.76	Whole blood	No
SRX2900813	39415099	Single	0.912747	NULL	FR	72.88	Whole blood	No
SRX2900814	36212853	Single	0.90499	NULL	FR	72.74	Whole blood	No
SRX2900816	39126045	Single	0.916358	NULL	FR	69.27	Whole blood	No
SRX2900817	33114788	Single	0.90456	NULL	FR	73.04	Whole blood	No
SRX2900819	37448460	Single	0.912877	NULL	FR	64.14	Whole blood	No
SRX3958380	34213931	Paired	0.98433	0.0166029	FR	91.57	IPEC-J2	No
SRX3958381	32158335	Paired	0.0174606	0.983485	RF	91.87	IPEC-J2	No
SRX3958382	6148698	Paired	0.976944	0.0244514	FR	93.77	IPEC-J2	No
SRX3958385	27123333	Paired	0.98442	0.0166044	FR	91.98	IPEC-J2	No
ERX2933220	14379774	Paired	0.0187342	0.981458	RF	88.53	Liver	No
ERX2933221	12909822	Paired	0.0150648	0.985953	RF	85.16	Muscle	No
ERX2933222	15245078	Paired	0.0151638	0.985473	RF	87.51	Muscle	No
ERX2933223	15181016	Paired	0.0150487	0.985561	RF	87.11	Muscle	No
ERX2933224	17540685	Paired	0.0148347	0.985709	RF	88.87	Muscle	No
ERX2933225	14022823	Paired	0.0148428	0.985543	RF	84.8	Muscle	No
ERX2933226	15219808	Paired	0.0163127	0.983909	RF	85.63	Muscle	No
ERX2933227	15742490	Paired	0.0145456	0.987323	RF	88.18	Muscle	No
ERX2933228	17213151	Paired	0.012606	0.988507	RF	88.95	Muscle	No
ERX2933229	17654953	Paired	0.0119093	0.98933	RF	88.33	Muscle	No
SRX5401600	59667932	Paired	0.0284982	0.971871	RF	88.44	Muscle	No
SRX5401601	72692762	Paired	0.0531601	0.943739	RF	82.48	Muscle	No
SRX3594510	25509430	Single	0.816918	NULL	FR	71.59	Uterus	No
SRX3594511	8135180	Single	0.903182	NULL	FR	69.44	Uterus	No
SRX3594512	16178141	Single	0.857631	NULL	FR	62.68	Uterus	No
SRX3594513	18812398	Single	0.857212	NULL	FR	73.55	Uterus	No
SRX3594514	17095636	Single	0.882608	NULL	FR	74.14	Uterus	No
SRX3594516	19019123	Single	0.859322	NULL	FR	72.31	Uterus	No
SRX3594517	22744167	Single	0.820997	NULL	FR	74.56	Uterus	No
SRX2900820	47110550	Single	0.908888	NULL	FR	68.24	Whole blood	No
SRX2900821	34813559	Single	0.920175	NULL	FR	63.26	Whole blood	No
SRX2900822	39944561	Single	0.917722	NULL	FR	71.2	Whole blood	No
SRX2900823	46137078	Single	0.908752	NULL	FR	73.94	Whole blood	No
SRX2900824	37836811	Single	0.906323	NULL	FR	74.91	Whole blood	No
SRX2900825	33498328	Single	0.899779	NULL	FR	79.79	Whole blood	No
SRX2900826	37573607	Single	0.901468	NULL	FR	71.83	Whole blood	No
SRX2900827	39321936	Single	0.900603	NULL	FR	81.19	Whole blood	No
SRX2900828	29830930	Single	0.905741	NULL	FR	69.55	Whole blood	No
SRX2900829	24429405	Single	0.890783	NULL	FR	61.37	Whole blood	No
SRX8097960	45501931	Paired	0.0274407	0.972047	RF	82.76	Muscle	No
SRX8097961	61475220	Paired	0.0270072	0.973376	RF	89.59	Muscle	No
SRX8097962	52832401	Paired	0.0219174	0.978536	RF	85.4	Liver	No
SRX8097963	60736461	Paired	0.0139875	0.986372	RF	82.58	Liver	No
SRX8097964	50597634	Paired	0.0243677	0.975289	RF	86.83	Liver	No
SRX8097965	49157205	Paired	0.0503856	0.948658	RF	87.29	Adipose	No

SRX8097966	53056490	Paired	0.0479446	0.951577	RF	79.49	Adipose	No
SRX8097967	47750035	Paired	0.0455479	0.952918	RF	88.56	Adipose	No
SRX8097968	45606407	Paired	0.0652755	0.933209	RF	86.62	Brain	No
SRX8097969	56275162	Paired	0.0401823	0.958896	RF	81.09	Brain	No
ERX2933230	16260853	Paired	0.0154714	0.984885	RF	88.21	Muscle	No
ERX2933231	15797070	Paired	0.0125155	0.988315	RF	87.88	Muscle	No
ERX2933232	13862228	Paired	0.0154711	0.984922	RF	88.04	Muscle	No
ERX2933233	18172163	Paired	0.0180658	0.981962	RF	88.75	Liver	No
ERX2933234	14670052	Paired	0.020293	0.980088	RF	88.99	Liver	No
ERX2933235	14320866	Paired	0.0193795	0.98071	RF	86.61	Liver	No
ERX2933236	14174607	Paired	0.0196384	0.980446	RF	88.96	Liver	No
ERX2933237	15091846	Paired	0.0196328	0.980192	RF	86.96	Liver	No
ERX2933238	15738983	Paired	0.0175949	0.982694	RF	89.54	Liver	No
ERX2933239	13321845	Paired	0.0219482	0.977999	RF	88.15	Liver	No
SRX2900830	34540490	Single	0.906288	NULL	FR	64.63	Whole blood	No
SRX8097970	51548689	Paired	0.0674414	0.931576	RF	86.89	Brain	No
SRX8097971	45913150	Paired	0.0635945	0.935296	RF	82.39	Brain	No
SRX8097972	47499779	Paired	0.0510241	0.948211	RF	89.82	Adipose	No
SRX8097973	48134553	Paired	0.0484305	0.950429	RF	87.47	Adipose	No
SRX8097974	47243942	Paired	0.0411627	0.957329	RF	78.12	Adipose	No
SRX8097975	50819157	Paired	0.0259128	0.974884	RF	89.06	Muscle	No
SRX8097976	56562383	Paired	0.0297292	0.971582	RF	90.34	Muscle	No
SRX8097977	49164406	Paired	0.0323412	0.969135	RF	90.3	Muscle	No
SRX8097978	46407860	Paired	0.0497188	0.948616	RF	87.48	Brain	No
SRX8097979	44187345	Paired	0.0306835	0.966731	RF	82.72	Brain	No
ERX2933240	16852943	Paired	0.0192998	0.98083	RF	87.26	Liver	No
ERX2933241	14355796	Paired	0.0193188	0.980676	RF	88.81	Liver	No
ERX2933243	16915894	Paired	0.0189341	0.981254	RF	83.79	Liver	No
ERX2933244	14696756	Paired	0.0189114	0.981369	RF	85.34	Liver	No
ERX2933245	14659864	Paired	0.0147905	0.986234	RF	88.62	Muscle	No
ERX2933246	16181687	Paired	0.0148448	0.985436	RF	87.97	Muscle	No
ERX2933247	17358915	Paired	0.0153372	0.985152	RF	88.8	Muscle	No
ERX2933248	14280405	Paired	0.0139864	0.98662	RF	83.29	Muscle	No
ERX2933249	12397617	Paired	0.017115	0.983146	RF	87.23	Muscle	No
SRX9154202	84360406	Single	0.0245634	NULL	RF	92.15	Ovary	No
SRX9154203	67221734	Single	0.0233251	NULL	RF	90.49	Ovary	No
SRX9154204	50708082	Single	0.0203689	NULL	RF	91.4	Ovary	No
SRX6804821	71673569	Paired	0.0385112	0.960516	RF	89.22	Small intestine	No
SRX6804822	71201833	Paired	0.0377249	0.961123	RF	88.05	Small intestine	No
SRX6804823	69252823	Paired	0.0417347	0.956116	RF	87.96	Small intestine	No
SRX6804824	71051863	Paired	0.0397061	0.959092	RF	88.14	Small intestine	No
SRX6804825	65224667	Paired	0.0396147	0.958791	RF	88.74	Small intestine	No
SRX6804826	70927294	Paired	0.0422789	0.955513	RF	88.66	Small intestine	No
SRX6804827	70267212	Paired	0.0417564	0.956377	RF	87.99	Small intestine	No
SRX6804828	71028641	Paired	0.039751	0.95859	RF	86.97	Small intestine	No
SRX6804829	71012747	Paired	0.0410488	0.957421	RF	87.76	Small intestine	No
SRX8097980	50515995	Paired	0.043357	0.954245	RF	82.43	Brain	No
SRX8097981	53787602	Paired	0.035034	0.96364	RF	83.6	Brain	No
SRX8097982	55230452	Paired	0.0314163	0.969282	RF	89.74	Muscle	No
SRX8097983	50542111	Paired	0.0310349	0.969676	RF	91.78	Muscle	No
SRX8097984	48219753	Paired	0.0349795	0.965609	RF	91.56	Muscle	No
SRX8097985	45889509	Paired	0.0241777	0.975494	RF	85.51	Liver	No
SRX8097986	49414473	Paired	0.0267744	0.972987	RF	86.06	Liver	No
SRX8097987	46281427	Paired	0.0235535	0.976048	RF	87.8	Liver	No
SRX8097988	53274406	Paired	0.027468	0.973192	RF	79.67	Adipose	No
SRX8097989	46309028	Paired	0.0386935	0.961143	RF	84.21	Adipose	No
ERX2933250	16140518	Paired	0.0146368	0.985866	RF	86.85	Muscle	No
ERX2933251	15067247	Paired	0.0135432	0.987873	RF	86.49	Muscle	No

SRX11196336	21882065	Paired	0.0274492	0.973745	RF	84.91	Heart	No
ERX2933252	18117857	Paired	0.0120267	0.989253	RF	88.78	Muscle	No
SRX11196337	19770465	Paired	0.0243719	0.976513	RF	86.25	Heart	No
ERX2933253	14790265	Paired	0.0138955	0.987395	RF	87.69	Muscle	No
SRX11196338	18303407	Paired	0.0236919	0.977091	RF	85.68	Heart	No
ERX2933254	14755657	Paired	0.0161184	0.984207	RF	87.19	Muscle	No
SRX11196339	18472696	Paired	0.0245115	0.976741	RF	84.86	Heart	No
ERX2933255	16083553	Paired	0.0138181	0.986599	RF	88.39	Muscle	No
ERX2933256	15594776	Paired	0.0139699	0.986903	RF	89.06	Muscle	No
SRX9154210	67609497	Single	0.0195552	NULL	RF	93.12	Ovary	No
SRX9154211	72123130	Single	0.0191846	NULL	RF	93.7	Ovary	No
SRX9154212	91642836	Single	0.0189494	NULL	RF	93.29	Ovary	No
SRX6804830	71351483	Paired	0.034737	0.965111	RF	88.06	Small intestine	No
SRX6804831	71828754	Paired	0.037304	0.962152	RF	89.02	Small intestine	No
SRX8418613	28306830	Paired	0.0331167	0.966212	RF	80.77	PBMCs	No
SRX8418614	29940427	Paired	0.0329408	0.966746	RF	80.73	PBMCs	No
SRX8097990	52959066	Paired	0.0415793	0.958311	RF	89.16	Adipose	No
SRX8097991	47125934	Paired	0.0394487	0.959149	RF	80.82	Brain	No
SRX8097992	59595865	Paired	0.0849626	0.913644	RF	77.29	Uterus	No
SRX8097994	47857183	Paired	0.0394848	0.961199	RF	77.01	Muscle	No
SRX8097995	48587239	Paired	0.0291903	0.970904	RF	87.28	Muscle	No
SRX8097996	42870225	Paired	0.0669732	0.932294	RF	89.2	Brain	No
SRX11196340	21343508	Paired	0.0249468	0.975851	RF	85.1	Heart	No
SRX2173433	43687683	Paired	0.17127	0.830489	RF	80.43	Muscle	No
SRX8097997	45318518	Paired	0.0683127	0.930204	RF	90	Brain	No
SRX11196341	21356048	Paired	0.024305	0.976775	RF	85.19	Heart	No
SRX2173434	39152101	Paired	0.169503	0.831975	RF	82.05	Muscle	No
SRX8097998	55663025	Paired	0.065694	0.933387	RF	89.22	Brain	No
SRX11196342	19647995	Paired	0.0247834	0.976419	RF	86.31	Heart	No
SRX8097999	51761826	Paired	0.0303008	0.969256	RF	81.58	Adipose	No
SRX11196343	21104068	Paired	0.025533	0.975653	RF	85.26	Heart	No
SRX11196344	20026679	Paired	0.0244115	0.976532	RF	86.04	Heart	No
SRX11196345	20815224	Paired	0.025965	0.97457	RF	84.21	Heart	No
SRX11196346	22433166	Paired	0.0251376	0.975632	RF	83.42	Heart	No
SRX11196347	20741481	Paired	0.0228684	0.978075	RF	85.83	Heart	No
SRX11196348	18852221	Paired	0.0273084	0.973567	RF	84.85	Heart	No
SRX11196349	21494317	Paired	0.0241409	0.97714	RF	84.56	Heart	No
SRX11196350	21204883	Paired	0.0255275	0.97583	RF	85.2	Heart	No
SRX11196351	22538554	Paired	0.0227824	0.978022	RF	84.08	Heart	No
SRX11196352	22987011	Paired	0.0249571	0.976069	RF	85	Heart	No
SRX11196353	21338708	Paired	0.0250198	0.97601	RF	84.86	Heart	No
SRX11196354	17420373	Paired	0.025606	0.974689	RF	82.44	Heart	No
SRX11196355	20726728	Paired	0.0238395	0.977144	RF	85.14	Heart	No
SRX11196356	23765777	Paired	0.0248335	0.976374	RF	85.29	Heart	No
SRX11196357	21146698	Paired	0.0243361	0.976677	RF	85.66	Heart	No
SRX11196358	17467536	Paired	0.0254772	0.975528	RF	85.23	Heart	No
SRX11196359	19426292	Paired	0.0254719	0.975411	RF	84.98	Heart	No
SRX7855000	34580887	Paired	0.019006	0.981606	RF	92.11	Small intestine	No
SRX7855001	35085213	Paired	0.0198073	0.981044	RF	92.11	Small intestine	No
SRX7855002	36180843	Paired	0.0197312	0.981244	RF	92.17	Small intestine	No
SRX7855003	37963584	Paired	0.0218002	0.978419	RF	92.06	Small intestine	No
SRX7855004	35829253	Paired	0.020566	0.980065	RF	92.13	Small intestine	No
SRX7855005	33864588	Paired	0.0202455	0.980518	RF	92.13	Small intestine	No
SRX7855006	38124229	Paired	0.0224862	0.979261	RF	90	Small intestine	No
SRX7855007	33542887	Paired	0.0180592	0.982444	RF	92.01	Small intestine	No
SRX7855008	33409211	Paired	0.0180215	0.982699	RF	91.93	Small intestine	No
SRX7855009	33228984	Paired	0.0195456	0.981089	RF	92.13	Small intestine	No
SRX7855010	34681628	Paired	0.0189879	0.981835	RF	72.46	Small intestine	No

SRX7855011	34171642	Paired	0.0249961	0.97658	RF	75.09	Small intestine	No
SRX7855012	40120602	Paired	0.0185976	0.981944	RF	68.03	Small intestine	No
SRX7855013	32228052	Paired	0.0207208	0.9807	RF	90.3	Small intestine	No
SRX7855014	40154405	Paired	0.0202999	0.978982	RF	91.35	IPEC-J2	No
SRX7855015	39038269	Paired	0.0209039	0.979628	RF	92.79	IPEC-J2	No
SRX7855016	36424048	Paired	0.0220823	0.978118	RF	91.93	IPEC-J2	No
SRX8418644	29912060	Paired	0.0512287	0.971552	RF	74.92	PBMCs	No
SRX8418645	28781577	Paired	0.0537293	0.970208	RF	74.96	PBMCs	No
ERX3013403	26039712	Single	0.0465419	NULL	RF	66.86	Whole blood	No
ERX3013404	28341364	Single	0.0551512	NULL	RF	70.43	Whole blood	No
ERX3013406	30660953	Single	0.0457673	NULL	RF	72.34	Whole blood	No
ERX3013407	32323242	Single	0.0439314	NULL	RF	80.66	Whole blood	No
ERX3013408	31592361	Single	0.046098	NULL	RF	84.01	Whole blood	No
ERX3013410	32769169	Single	0.053744	NULL	RF	71.54	Whole blood	No
ERX3013411	33830819	Single	0.0587338	NULL	RF	60.67	Whole blood	No
ERX3013412	37530883	Single	0.0839934	NULL	RF	66.09	Whole blood	No
SRX8361118	70300579	Paired	0.0228563	0.97528	RF	91.34	Ovary	No
SRX8361119	60744033	Paired	0.0226197	0.974857	RF	89.07	Ovary	No
SRX8361120	70803358	Paired	0.0200224	0.978383	RF	91.11	Ovary	No
SRX8361121	60103938	Paired	0.0202199	0.978274	RF	88.44	Ovary	No
SRX9720712	138033091	Single	0.0282086	NULL	RF	94.25	Testis	No
SRX9720713	81771170	Single	0.0204684	NULL	RF	94.59	Testis	No
SRX9720714	134264312	Single	0.0216023	NULL	RF	94.46	Testis	No
SRX9720715	82809636	Single	0.0259321	NULL	RF	94.22	Testis	No
SRX9720716	92810304	Single	0.0235971	NULL	RF	94.82	Testis	No
SRX9720718	77933347	Single	0.0280205	NULL	RF	94.27	Testis	No
SRX9720719	82776764	Single	0.0239252	NULL	RF	94.53	Testis	No
SRX4488066	46821792	Paired	0.0490753	0.948514	RF	89.22	Lung	No
SRX4488067	43743269	Paired	0.0448517	0.951294	RF	88.84	Lung	No
SRX4488068	43377583	Paired	0.047938	0.951824	RF	88.34	Lung	No
SRX4488069	46270985	Paired	0.0493757	0.945788	RF	88.65	Lung	No
SRX2576695	45598534	Paired	0.984571	0.0163097	FR	88.22	Ovary	No
SRX9720720	67456449	Single	0.0280818	NULL	RF	94.3	Testis	No
SRX4488070	49369301	Paired	0.0453426	0.949615	RF	89.18	Lung	No
SRX4488071	47862018	Paired	0.0399767	0.956389	RF	89.41	Lung	No
SRX3098412	25821878	Paired	0.0205568	0.980293	RF	90.94	Brain	No
SRX3098413	25840964	Paired	0.0174975	0.983906	RF	91.92	Brain	No
SRX3098414	25723155	Paired	0.019609	0.981779	RF	92.46	Ovary	No
SRX3098415	25816397	Paired	0.0209246	0.980094	RF	93.44	Brain	No
SRX3098416	25891532	Paired	0.0177591	0.983821	RF	92.34	Brain	No
SRX3098417	25686139	Paired	0.0182304	0.983602	RF	92.7	Ovary	No
SRX8982584	76831091	Paired	0.0382095	0.961667	RF	86.95	Macrophage	No
SRX8982585	74199742	Paired	0.0378593	0.961932	RF	86.04	Macrophage	No
SRX8982586	73362312	Paired	0.0413694	0.958089	RF	90	Macrophage	No
SRX8982587	79308988	Paired	0.0303686	0.969821	RF	87.24	Macrophage	No
SRX8982588	65213907	Paired	0.0342186	0.965697	RF	89.62	Macrophage	No
SRX8982589	73697778	Paired	0.0461122	0.952672	RF	89.3	Macrophage	No
SRX7117136	54634760	Paired	0.0291222	0.970897	RF	89.53	IPEC-J2	No
SRX7117137	53519279	Paired	0.0234387	0.976433	RF	88.49	IPEC-J2	No
SRX7117138	44085709	Paired	0.0246314	0.975476	RF	89.11	IPEC-J2	No
SRX7117139	59632130	Paired	0.0243723	0.975686	RF	88.77	IPEC-J2	No
SRX8982590	69629563	Paired	0.0297965	0.970407	RF	83.71	Macrophage	No
SRX8982591	74133170	Paired	0.025937	0.974294	RF	83.83	Macrophage	No
SRX8982592	81927072	Paired	0.0264782	0.973877	RF	80.16	Macrophage	No
SRX7117140	53413010	Paired	0.0262499	0.973509	RF	89.7	IPEC-J2	No
SRX7117141	46784233	Paired	0.0246506	0.975236	RF	89.38	IPEC-J2	No
SRX6076650	46750662	Paired	0.0271717	0.972774	RF	90.58	Skin	No
SRX6076651	57600867	Paired	0.0316791	0.967991	RF	89.53	Skin	No

SRX3791900	17938898	Single	0.913948	NULL	FR	71.18	Cartilage	No
SRX3791901	16531785	Single	0.916784	NULL	FR	68.34	Cartilage	No
SRX6076653	61215504	Paired	0.0283879	0.970981	RF	88.06	Skin	No
SRX3791902	16436061	Single	0.805422	NULL	FR	64.77	Cartilage	No
SRX3791903	10986230	Single	0.89944	NULL	FR	71.65	Cartilage	No
SRX6076655	60925336	Paired	0.0255313	0.973814	RF	89.2	Skin	No
SRX6076656	49070497	Paired	0.0265292	0.972925	RF	90.42	Skin	No
SRX6076657	65192487	Paired	0.0262177	0.972537	RF	88.81	Skin	No
SRX6076658	55842782	Paired	0.029293	0.97026	RF	89.76	Skin	No
SRX6076659	42157645	Paired	0.0326342	0.96676	RF	89.61	Skin	No
SRX3679683	44465155	Paired	0.0536553	0.945465	RF	83.05	Testis	No
SRX3679684	52130049	Paired	0.102889	0.892877	RF	84.63	Testis	No
SRX3679686	38770226	Paired	0.175538	0.819161	RF	83.13	Testis	No
SRX6076662	47214659	Paired	0.0256833	0.973624	RF	90.03	Skin	No
SRX6076663	58755419	Paired	0.0237127	0.976095	RF	89.28	Skin	No
SRX6076664	54078521	Paired	0.0272898	0.97201	RF	89.4	Skin	No
SRX6076665	46634708	Paired	0.027907	0.971093	RF	88.18	Skin	No
SRX6076666	46111068	Paired	0.0348269	0.964792	RF	89.07	Skin	No
ERX5700812	28108881	Paired	0.0277718	0.973579	RF	91.39	Artery	No
ERX5700814	28200606	Paired	0.0229138	0.977962	RF	91.78	Artery	No
ERX5700816	32980410	Paired	0.0201975	0.981497	RF	91.8	Artery	No
ERX5700818	26805364	Paired	0.0215001	0.979362	RF	92.51	Artery	No
SRX6076671	50112118	Paired	0.0264635	0.972389	RF	89	Skin	No
SRX6076672	50468686	Paired	0.0244396	0.975288	RF	90.3	Skin	No
SRX6076673	49600777	Paired	0.0265232	0.97269	RF	87.67	Skin	No
SRX5728535	28203724	Paired	0.962238	0.0356154	FR	89.18	Brain	No
SRX5728536	35383739	Paired	0.963523	0.0353892	FR	89.25	Brain	No
SRX5728537	30574719	Paired	0.962945	0.034815	FR	89.21	Brain	No
ERX5700820	28450312	Paired	0.0221895	0.97868	RF	92.6	Artery	No
SRX5728538	39164731	Paired	0.964256	0.0346197	FR	89.72	Brain	No
SRX5728539	29248704	Paired	0.962881	0.0357537	FR	89.24	Brain	No
ERX5700823	35010869	Paired	0.0217993	0.979432	RF	91.97	Artery	No
ERX5700826	35609368	Paired	0.0234764	0.97768	RF	92.23	Artery	No
ERX5700829	30885966	Paired	0.0242928	0.976551	RF	92.11	Artery	No
SRX5728540	22880390	Paired	0.961509	0.0363055	FR	88.95	Brain	No
ERX5700832	32413808	Paired	0.0266318	0.974153	RF	92.18	Artery	No
ERX5700835	30413065	Paired	0.0247109	0.976195	RF	92.16	Artery	No
ERX5700838	34978381	Paired	0.0241093	0.97696	RF	91.88	Artery	No
SRX7278247	46003075	Paired	0.0672365	0.931679	RF	85.91	Uterus	No
SRX7278248	47975743	Paired	0.112818	0.884152	RF	83.05	Uterus	No
SRX3360331	89343900	Paired	0.0644912	0.928369	RF	89.51	Liver	No
SRX3360332	92422473	Paired	0.0660967	0.928081	RF	91.52	Liver	No
SRX3360333	93215404	Paired	0.0943704	0.894358	RF	89.55	Liver	No
SRX3360334	103908002	Paired	0.0988108	0.887063	RF	89.54	Liver	No
SRX3360335	93591229	Paired	0.0660641	0.925895	RF	90.35	Liver	No
SRX3360336	97379199	Paired	0.0663201	0.924595	RF	90.54	Liver	No
SRX2981753	10732047	Paired	0.119076	0.87755	RF	79.23	Lung	No
SRX2981754	10630621	Paired	0.119127	0.877496	RF	79.52	Lung	No
SRX2981755	11240340	Paired	0.877079	0.119836	FR	77.62	Lung	No
SRX2981756	10686143	Paired	0.119194	0.877678	RF	79.2	Lung	No
SRX9610832	21537702	Paired	0.0552165	0.93788	RF	79.82	Artery	No
SRX5412690	63321556	Paired	0.0297259	0.971482	RF	80.23	Lymph node	No
SRX9610833	20904801	Paired	0.0404661	0.955592	RF	78.96	Artery	No
SRX5412691	50613999	Paired	0.116251	0.88475	RF	82.25	Lymph node	No
SRX9610834	22981315	Paired	0.039286	0.957248	RF	82.69	Artery	No
SRX5412692	57737114	Paired	0.027731	0.974062	RF	80.65	Lymph node	No
SRX9610835	21509902	Paired	0.0642241	0.926508	RF	76.98	Artery	No
SRX5412693	62231889	Paired	0.0286458	0.973011	RF	77.79	Lymph node	No

SRX9610836	20965953	Paired	0.0435878	0.951512	RF	79.84	Artery	No
SRX5412694	71124484	Paired	0.0459347	0.955127	RF	81.13	Lymph node	No
SRX9610837	21134068	Paired	0.0452588	0.950171	RF	82.47	Artery	No
SRX5412695	63246084	Paired	0.0442662	0.957201	RF	85.99	Lymph node	No
SRX9610838	22957846	Paired	0.0463733	0.95028	RF	79	Artery	No
SRX5412696	56722649	Paired	0.0281657	0.972584	RF	85.25	Lymph node	No
SRX9610839	18532565	Paired	0.0272359	0.970368	RF	66.96	Artery	No
SRX5412697	53845761	Paired	0.0677818	0.932978	RF	87.6	Lymph node	No
SRX5412698	76518273	Paired	0.0633626	0.938219	RF	85.11	Lymph node	No
SRX5412699	43509724	Paired	0.0580951	0.942408	RF	90.79	Lymph node	No
SRX5260180	7907896	Paired	0.0351764	0.964037	RF	93.1	Muscle	No
SRX5260181	9294881	Paired	0.0334929	0.965754	RF	93.12	Muscle	No
SRX5260182	8941054	Paired	0.0342071	0.964816	RF	93.28	Muscle	No
SRX5260183	9438055	Paired	0.0344031	0.964701	RF	93.04	Muscle	No
SRX9274503	32067938	Paired	0.032406	0.969057	RF	84.91	Muscle	No
SRX5401595	66944288	Paired	0.0561315	0.93969	RF	82.26	Muscle	No
SRX9914894	23184402	Paired	0.0400105	0.95843	RF	84.74	Testis	No
SRX9914896	15537217	Paired	0.035318	0.963411	RF	84.65	Testis	No
SRX9914898	45395238	Paired	0.030509	0.96843	RF	85.03	Testis	No
SRX5260178	10410871	Paired	0.0375887	0.96128	RF	93.39	Muscle	No
SRX5260179	10481951	Paired	0.0341968	0.964977	RF	93.43	Muscle	No
SRX5401602	62025249	Paired	0.026099	0.973844	RF	86.86	Muscle	No
SRX8413042	24614663	Paired	0.0414999	0.955518	RF	76.82	PBMCs	No
SRX6604383	55075251	Paired	0.0264532	0.97281	RF	86.91	Ovary	No
SRX6604384	54581675	Paired	0.0234305	0.975387	RF	84.19	Ovary	No
P100_Adipose_rep1	52261994	Paired	-	-	RF	90.48	Adipose	No
P100_Adipose_rep2	51898981	Paired	-	-	RF	90.09	Adipose	No
P100_Brain_rep1	49712413	Paired	-	-	RF	88.3	Brain	No
P100_Brain_rep2	53061643	Paired	-	-	RF	88.29	Brain	No
P100_Heart_rep1	50680762	Paired	-	-	RF	91.12	Heart	No
P100_Heart_rep2	51221578	Paired	-	-	RF	90.79	Heart	No
P100_Kidney_rep1	50507406	Paired	-	-	RF	91.03	Kidney	No
P100_Kidney_rep2	51867697	Paired	-	-	RF	90.78	Kidney	No
P100_LInt_rep1	51351979	Paired	-	-	RF	90.03	Large intestine	No
P100_LInt_rep2	49997858	Paired	-	-	RF	88.91	Large intestine	No
P100_Liver_rep1	50600502	Paired	-	-	RF	90.21	Liver	No
P100_Liver_rep2	50788917	Paired	-	-	RF	90.86	Liver	No
P100_Lung_rep1	50876779	Paired	-	-	RF	90.21	Lung	No
P100_Lung_rep2	51223956	Paired	-	-	RF	90.3	Lung	No
P100_Muscle_rep1	50306553	Paired	-	-	RF	90.84	Muscle	No
P100_Muscle_rep2	50854528	Paired	-	-	RF	89.49	Muscle	No
P100_SInt_rep1	51399308	Paired	-	-	RF	89.15	Small intestine	No
P100_SInt_rep2	50619598	Paired	-	-	RF	89.95	Small intestine	No
P100_Spleen_rep1	50272221	Paired	-	-	RF	89.81	Spleen	No
P100_Spleen_rep2	57630379	Paired	-	-	RF	90.11	Spleen	No
P100_Testis_rep1	51061316	Paired	-	-	RF	90.89	Testis	No
P100_Testis_rep2	50434350	Paired	-	-	RF	89.17	Testis	No
P180_Adipose_rep1	52953411	Paired	-	-	RF	88.56	Adipose	No
P180_Adipose_rep2	51890675	Paired	-	-	RF	89.74	Adipose	No
P180_Brain_rep1	50843980	Paired	-	-	RF	85.14	Brain	No
P180_Brain_rep2	50086388	Paired	-	-	RF	87.86	Brain	No
P180_Heart_rep1	51258478	Paired	-	-	RF	89.93	Heart	No
P180_Heart_rep2	52079579	Paired	-	-	RF	88.71	Heart	No
P180_Kidney_rep1	50811168	Paired	-	-	RF	90.1	Kidney	No
P180_Kidney_rep2	50968545	Paired	-	-	RF	90.36	Kidney	No
P180_LInt_rep1	51711062	Paired	-	-	RF	87.2	Large intestine	No
P180_LInt_rep2	53303883	Paired	-	-	RF	88.21	Large intestine	No
P180_Liver_rep1	50121803	Paired	-	-	RF	89.33	Liver	No

P180_Liver_rep2	51865695	Paired	-	-	RF	90.39	Liver	No
P180_Lung_rep1	50694825	Paired	-	-	RF	89.7	Lung	No
P180_Lung_rep2	51150173	Paired	-	-	RF	90.17	Lung	No
P180_Muscle_rep1	51104520	Paired	-	-	RF	88.8	Muscle	No
P180_Muscle_rep2	51303885	Paired	-	-	RF	88.48	Muscle	No
P180_SInt_rep1	51558745	Paired	-	-	RF	87.52	Small intestine	No
P180_SInt_rep2	50466013	Paired	-	-	RF	89.68	Small intestine	No
P180_Spleen_rep1	50642007	Paired	-	-	RF	88.92	Spleen	No
P180_Spleen_rep2	50812025	Paired	-	-	RF	89.18	Spleen	No
P180_Testis_rep1	49914461	Paired	-	-	RF	90.09	Testis	No
P180_Testis_rep2	51112822	Paired	-	-	RF	89.65	Testis	No
P21_Adipose_rep1	54971819	Paired	-	-	RF	90.57	Adipose	No
P21_Adipose_rep2	50428601	Paired	-	-	RF	89.85	Adipose	No
P21_Brain_rep1	50895283	Paired	-	-	RF	89.26	Brain	No
P21_Brain_rep2	52393724	Paired	-	-	RF	89.06	Brain	No
P21_Heart_rep1	54775782	Paired	-	-	RF	90.68	Heart	No
P21_Heart_rep2	51144706	Paired	-	-	RF	90.71	Heart	No
P21_Kidney_rep1	54663791	Paired	-	-	RF	90.81	Kidney	No
P21_Kidney_rep2	51615655	Paired	-	-	RF	90.8	Kidney	No
P21_LInt_rep1	50345551	Paired	-	-	RF	88.7	Large intestine	No
P21_LInt_rep2	50393482	Paired	-	-	RF	90.56	Large intestine	No
P21_Liver_rep1	55003938	Paired	-	-	RF	90.71	Liver	No
P21_Liver_rep2	50726164	Paired	-	-	RF	90.94	Liver	No
P21_Lung_rep1	54824300	Paired	-	-	RF	90.97	Lung	No
P21_Lung_rep2	51504356	Paired	-	-	RF	90.81	Lung	No
P21_Muscle_rep1	55792299	Paired	-	-	RF	90.16	Muscle	No
P21_Muscle_rep2	51470924	Paired	-	-	RF	90.52	Muscle	No
P21_SInt_rep1	52227101	Paired	-	-	RF	89.9	Small intestine	No
P21_SInt_rep2	51152052	Paired	-	-	RF	90.46	Small intestine	No
P21_Spleen_rep1	55457106	Paired	-	-	RF	90.16	Spleen	No
P21_Spleen_rep2	51577489	Paired	-	-	RF	88.57	Spleen	No
P21_Testis_rep1	54594417	Paired	-	-	RF	90.51	Testis	No
P21_Testis_rep2	51074626	Paired	-	-	RF	90.55	Testis	No
P50_Adipose_rep1	51805197	Paired	-	-	RF	90.41	Adipose	No
P50_Adipose_rep2	50971770	Paired	-	-	RF	90.4	Adipose	No
P50_Brain_rep1	49905419	Paired	-	-	RF	89.06	Brain	No
P50_Brain_rep2	50135639	Paired	-	-	RF	87.83	Brain	No
P50_Heart_rep1	52627831	Paired	-	-	RF	90.73	Heart	No
P50_Heart_rep2	53945967	Paired	-	-	RF	91.26	Heart	No
P50_Kidney_rep1	51064114	Paired	-	-	RF	91.13	Kidney	No
P50_Kidney_rep2	50805369	Paired	-	-	RF	91.33	Kidney	No
P50_LInt_rep1	52096758	Paired	-	-	RF	89.63	Large intestine	No
P50_LInt_rep2	50618576	Paired	-	-	RF	90.31	Large intestine	No
P50_Liver_rep1	50372169	Paired	-	-	RF	90.84	Liver	No
P50_Liver_rep2	52752845	Paired	-	-	RF	90.79	Liver	No
P50_Lung_rep1	51106803	Paired	-	-	RF	90.61	Lung	No
P50_Lung_rep2	49577441	Paired	-	-	RF	89.93	Lung	No
P50_Muscle_rep1	50859675	Paired	-	-	RF	90.49	Muscle	No
P50_Muscle_rep2	50926395	Paired	-	-	RF	91.22	Muscle	No
P50_SInt_rep1	49797808	Paired	-	-	RF	90.13	Small intestine	No
P50_SInt_rep2	53505442	Paired	-	-	RF	89.93	Small intestine	No
P50_Spleen_rep1	50961753	Paired	-	-	RF	90.44	Spleen	No
P50_Spleen_rep2	50664051	Paired	-	-	RF	90.56	Spleen	No
P50_Testis_rep1	50430838	Paired	-	-	RF	90.61	Testis	No
P50_Testis_rep2	50806136	Paired	-	-	RF	90.98	Testis	No
S_PK15_rep1	60631841	Paired	-	-	RF	87.13	PK15	No
S_PK15_rep2	45840462	Paired	-	-	RF	87.24	PK15	No
