# Supplementary material for: Genome-wide identification of long non-coding RNAs in pigs and their roles in muscle development
Source: Anim Biosci. 2026 Jul 27;39(10):260444. doi: 10.5713/ab.260444 (PMC13662941; doi:10.5713/ab.260444)
Supplement: Supplementary file 4 [file ab-260444-Supplementary-4.pdf]

Supplement 4. Marker genes used for cell type annotation.

**Cell name**	**Cell marker**
HES1^{+} satellite cells	*PAX7, HES1*
TRIB1^{+} satellite cells	*PAX7, TRIB1*
Satellite stem cells	*PAX7, MYOD1*
MYL1^{+} myoblasts	*MYOG, MYL1*
MYMK^{+} myoblasts	*MYOG, MYMK*
I myofibers	*MYH7*
IIA myofibers	*MYH2*
IIX myofibers	*MYH1*
