# Supplementary material for: Genome-wide identification of long non-coding RNAs in pigs and their roles in muscle development
Source: Anim Biosci. 2026 Jul 27;39(10):260444. doi: 10.5713/ab.260444 (PMC13662941; doi:10.5713/ab.260444)
Supplement: Supplementary file 5 [file ab-260444-Supplementary-5.pdf]

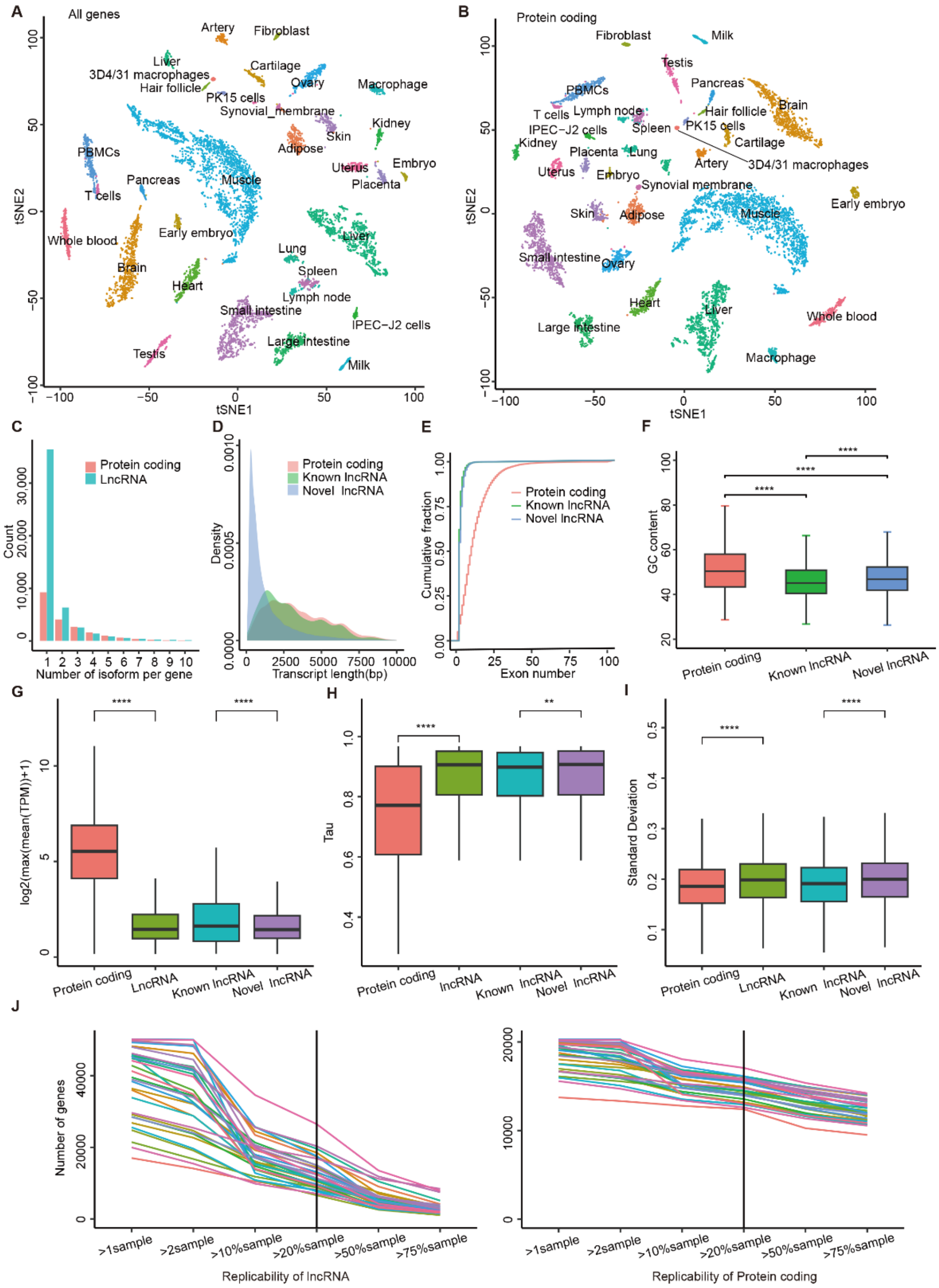

Supplement 5. Characteristics of lncRNA. (A) Tissue clustering based on all genes, (B) protein-coding genes. Comparisons between lncRNAs and protein-coding genes: transcript number (C), length (D), exon number (E); (F) GC content; (G) max expression; (H) tissue specificity; (I) expression SD. (J) Within-tissue reproducibility. $^{**}p < 0.01$, $^{****}p < 0.0001$. Significance was determined by Wilcoxon rank-sum test.
