# Supplementary material for: Genome-wide identification of long non-coding RNAs in pigs and their roles in muscle development
Source: Anim Biosci. 2026 Jul 27;39(10):260444. doi: 10.5713/ab.260444 (PMC13662941; doi:10.5713/ab.260444)
Supplement: Supplementary file 6 [file ab-260444-Supplementary-6.pdf]

**Supplement 6.** Percentage of eQTL linked lncRNA-mRNA pairs in the same TAD

| Percentage | Day | Resolution | Tissue |
|---|---|---|---|
| 0.558586 | P21 | 50000 | Adipose |
| 0.475855 | P21 | 50000 | Brain |
| 0.537371 | P21 | 50000 | Heart |
| 0.5 | P21 | 50000 | Kidney |
| 0.524993 | P21 | 50000 | Large_intestine |
| 0.581512 | P21 | 50000 | Liver |
| 0.593253 | P21 | 50000 | Lung |
| 0.562004 | P21 | 50000 | Muscle |
| 0.565381 | P21 | 50000 | Small_intestine |
| 0.602778 | P21 | 50000 | Spleen |
| 0.532968 | P21 | 50000 | Testis |
