# Supplementary material for: Genome-wide identification of long non-coding RNAs in pigs and their roles in muscle development
Source: Anim Biosci. 2026 Jul 27;39(10):260444. doi: 10.5713/ab.260444 (PMC13662941; doi:10.5713/ab.260444)
Supplement: Supplementary file 7 [file ab-260444-Supplementary-7.pdf]

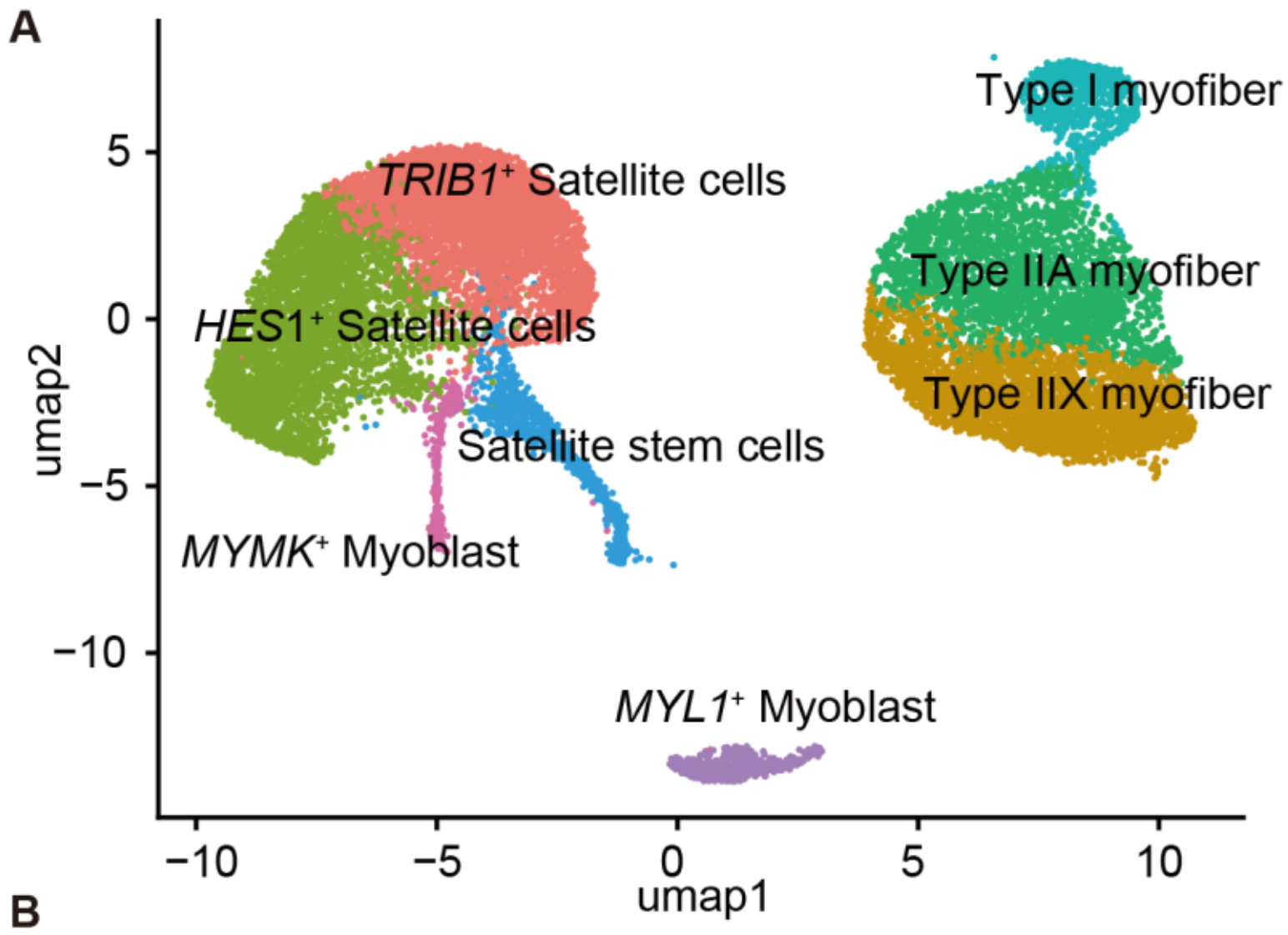

B

```
7 TCCAATTCCAGAGTAGATTGGGAGTAAG---AGAAGACAGACTTCAGTCTTAGAGAAATCAG 65 Human
  |||| |||||||| ||||| |||||  |   ||| || |||||  | |||||||||||| ||
7 TCCAGTTCCAGAGGAGATTAGGAGTTGGGTGAGAGGATAGACTCTAATCTTAGAGAAATAAG 68 Pig
```

Supplement 7. (A) Single-cell clustering of myogenic cell populations. (B) The results of BLASTN analysis of the second homologous exon of *MSTRG.33011* and *PRANCR*.
