# Supplementary material for: Genome-wide identification of long non-coding RNAs in pigs and their roles in muscle development
Source: Anim Biosci. 2026 Jul 27;39(10):260444. doi: 10.5713/ab.260444 (PMC13662941; doi:10.5713/ab.260444)
Supplement: Supplementary file 8 [file ab-260444-Supplementary-8.pdf]

Supplement 8. Differentially expressed genes across different cell types.

Gene	Cell name	logFC	logCPM	p_val	Gene id	Gene_biotype
CCBE1	*HES1*$^+$ Satellite cells	-5.09	6.21	0.000959	ENSSSCG00000004907	protein coding
ENSSSCG00000032436	*HES1*$^+$ Satellite cells	-4.21	5.17	0.0225	ENSSSCG00000032436	protein coding
MSTRG.22838	*HES1*$^+$ Satellite cells	-4.21	3.66	0.000371	MSTRG.22838	lncRNA
VAT1L	*HES1*$^+$ Satellite cells	-3.41	5.66	0.023	ENSSSCG00000002696	protein coding
ENSSSCG00000014565	*HES1*$^+$ Satellite cells	-3.33	5.56	0.0115	ENSSSCG00000014565	protein coding
IFI6	*HES1*$^+$ Satellite cells	-3.32	5.74	0.0425	ENSSSCG00000034570	protein coding
MPP3	*HES1*$^+$ Satellite cells	-3.3	6.92	9.88E-05	ENSSSCG00000017372	protein coding
ISG15	*HES1*$^+$ Satellite cells	-3.2	7.01	0.0298	ENSSSCG00000040575	protein coding
MSTRG.505	*HES1*$^+$ Satellite cells	-2.99	4.12	0.000154	MSTRG.505	lncRNA
CLCA2	*HES1*$^+$ Satellite cells	-2.98	3.28	0.00025	ENSSSCG00000006935	protein coding
IFITM3	*HES1*$^+$ Satellite cells	-2.86	7.02	0.00602	ENSSSCG00000038912	protein coding
SLC24A4	*HES1*$^+$ Satellite cells	-2.8	5.6	2.88E-06	ENSSSCG00000002450	protein coding
MSTRG.4840	*HES1*$^+$ Satellite cells	-2.78	4.41	9.75E-06	MSTRG.4840	lncRNA
ENSSSCG00000049704	*HES1*$^+$ Satellite cells	-2.75	3.8	0.00177	ENSSSCG00000049704	lncRNA
ENSSSCG00000009182	*HES1*$^+$ Satellite cells	-2.69	4.1	0.00583	ENSSSCG00000009182	protein coding
ALDH5A1	*HES1*$^+$ Satellite cells	-2.66	3.79	0.00452	ENSSSCG00000001090	protein coding
ANKFN1	*HES1*$^+$ Satellite cells	-2.66	5.02	0.0153	ENSSSCG00000017609	protein coding
TMEM100	*HES1*$^+$ Satellite cells	-2.63	7.06	4.68E-06	ENSSSCG00000017607	protein coding
ENSSSCG00000027725	*HES1*$^+$ Satellite cells	-2.52	3.54	0.000202	ENSSSCG00000027725	protein coding
EPHA1	*HES1*$^+$ Satellite cells	-2.44	3.46	0.0044	ENSSSCG00000016459	protein coding
MSTRG.39368	*HES1*$^+$ Satellite cells	-2.4	6.48	0.000601	MSTRG.39368	lncRNA
SERPINA1	*HES1*$^+$ Satellite cells	-2.4	3.74	0.000681	ENSSSCG00000002476	protein coding
MSTRG.27605	*HES1*$^+$ Satellite cells	-2.36	3.6	0.0041	MSTRG.27605	lncRNA
RAB6B	*HES1*$^+$ Satellite cells	-2.27	3.3	0.00261	ENSSSCG00000036503	protein coding
KCTD16	*HES1*$^+$ Satellite cells	-2.21	4.85	0.0038	ENSSSCG00000032826	protein coding
EIF2AK2	*HES1*$^+$ Satellite cells	-2.2	5.76	0.026	ENSSSCG00000008496	protein coding
MSTRG.34836	*HES1*$^+$ Satellite cells	-2.1	5.33	1.18E-05	MSTRG.34836	lncRNA
MSTRG.5305	*HES1*$^+$ Satellite cells	-2.1	4.55	0.00265	MSTRG.5305	lncRNA
ENSSSCG00000005216	*HES1*$^+$ Satellite cells	-2.07	4.24	0.024	ENSSSCG00000005216	protein coding
PNMT	*HES1*$^+$ Satellite cells	-2.02	6.63	9.24E-07	ENSSSCG00000017501	protein coding
FAM124A	*HES1*$^+$ Satellite cells	-1.89	4.41	0.00487	ENSSSCG00000029598	protein coding
XIRP2	*HES1*$^+$ Satellite cells	-1.89	4.46	0.0113	ENSSSCG00000015917	protein coding

ENSSSCG00000048185	*HES1*$^{+}$ Satellite cells	-1.88	3.44	0.0346	ENSSSCG00000048185	lncRNA
RIMBP2	*HES1*$^{+}$ Satellite cells	-1.87	3.54	0.000306	ENSSSCG00000009748	protein coding
MSTRG.6783	*HES1*$^{+}$ Satellite cells	-1.85	5.58	0.00557	MSTRG.6783	lncRNA
MSTRG.48476	*HES1*$^{+}$ Satellite cells	-1.82	4.18	0.000271	MSTRG.48476	lncRNA
MSTRG.27600	*HES1*$^{+}$ Satellite cells	-1.78	4.83	0.00199	MSTRG.27600	lncRNA
ENSSSCG00000049158	*HES1*$^{+}$ Satellite cells	-1.75	4.52	5.27E-05	ENSSSCG00000049158	protein coding
PLXDC1	*HES1*$^{+}$ Satellite cells	-1.74	4.66	0.000959	ENSSSCG00000017511	protein coding
DAPK1	*HES1*$^{+}$ Satellite cells	-1.73	6.83	0.00245	ENSSSCG00000010949	protein coding
MSTRG.32994	*HES1*$^{+}$ Satellite cells	-1.73	3.6	0.0111	MSTRG.32994	lncRNA
TAP1	*HES1*$^{+}$ Satellite cells	-1.73	3.67	0.0226	ENSSSCG00000025618	protein coding
MSTRG.42256	*HES1*$^{+}$ Satellite cells	-1.72	3.81	0.00217	MSTRG.42256	lncRNA
BOC	*HES1*$^{+}$ Satellite cells	-1.71	5.3	0.000593	ENSSSCG00000025826	protein coding
MSTRG.5303	*HES1*$^{+}$ Satellite cells	-1.68	3.49	0.0109	MSTRG.5303	lncRNA
SH3RF2	*HES1*$^{+}$ Satellite cells	-1.68	6.41	0.00364	ENSSSCG00000014410	protein coding
MSTRG.8361	*HES1*$^{+}$ Satellite cells	-1.67	3.8	0.00677	MSTRG.8361	lncRNA
ENSSSCG00000049152	*HES1*$^{+}$ Satellite cells	-1.64	3.84	0.0026	ENSSSCG00000049152	lncRNA
ADAMTS15	*HES1*$^{+}$ Satellite cells	-1.6	4.13	0.0128	ENSSSCG00000015250	protein coding
ENSSSCG00000042225	*HES1*$^{+}$ Satellite cells	-1.6	5.47	0.000107	ENSSSCG00000042225	lncRNA
PLXNA4	*HES1*$^{+}$ Satellite cells	-1.6	6.9	0.000398	ENSSSCG00000021562	protein coding
SLC22A3	*HES1*$^{+}$ Satellite cells	-1.6	3.86	0.0109	ENSSSCG00000004039	protein coding
MSTRG.1952	*HES1*$^{+}$ Satellite cells	-1.59	6.3	0.000789	MSTRG.1952	lncRNA
SYTL5	*HES1*$^{+}$ Satellite cells	-1.58	6.26	0.0329	ENSSSCG00000012233	protein coding
ENSSSCG00000017608	*HES1*$^{+}$ Satellite cells	-1.56	5.52	0.000491	ENSSSCG00000017608	protein coding
MFSD4B	*HES1*$^{+}$ Satellite cells	-1.55	3.94	0.000885	ENSSSCG00000041409	protein coding
KIAA0513	*HES1*$^{+}$ Satellite cells	-1.54	6.31	0.000229	ENSSSCG00000002666	protein coding
PDZRN3	*HES1*$^{+}$ Satellite cells	-1.54	6.39	0.00638	ENSSSCG00000011521	protein coding
ASPA	*HES1*$^{+}$ Satellite cells	-1.53	3.81	0.0159	ENSSSCG00000017861	protein coding
HR	*HES1*$^{+}$ Satellite cells	-1.53	3.85	0.0043	ENSSSCG00000009616	protein coding
ENSSSCG00000039821	*HES1*$^{+}$ Satellite cells	-1.51	3.56	0.00176	ENSSSCG00000039821	lncRNA
MSTRG.14188	*HES1*$^{+}$ Satellite cells	-1.5	3.34	0.0458	MSTRG.14188	lncRNA
NOL11	*HES1*$^{+}$ Satellite cells	-1.5	9.44	0.000772	ENSSSCG00000017274	protein coding
CRISPLD2	*HES1*$^{+}$ Satellite cells	-1.49	5.6	0.00056	ENSSSCG00000002669	protein coding
FIS1	*HES1*$^{+}$ Satellite cells	-1.49	3.52	0.0196	ENSSSCG00000038938	protein coding
ABCA8	*HES1*$^{+}$ Satellite cells	-1.48	7.12	0.0363	ENSSSCG00000026473	protein coding

MSTRG.24904	*HES1*$^+$ Satellite cells	-1.48	3.83	0.0015	MSTRG.24904	lncRNA
MSTRG.9705	*HES1*$^+$ Satellite cells	-1.47	4.55	0.00466	MSTRG.9705	lncRNA
AGT	*HES1*$^+$ Satellite cells	-1.45	7.67	0.0106	ENSSSCG00000010184	protein coding
MSTRG.34842	*HES1*$^+$ Satellite cells	-1.44	4	0.0193	MSTRG.34842	lncRNA
MSTRG.30373	*HES1*$^+$ Satellite cells	-1.43	4.04	0.0023	MSTRG.30373	lncRNA
MSTRG.506	*HES1*$^+$ Satellite cells	-1.43	7.13	0.000311	MSTRG.506	lncRNA
TRAPPC12	*HES1*$^+$ Satellite cells	-1.43	3.45	0.00482	ENSSSCG00000024665	protein coding
KLF15	*HES1*$^+$ Satellite cells	-1.42	5.72	0.00689	ENSSSCG00000036742	protein coding
LAMC2	*HES1*$^+$ Satellite cells	-1.42	3.48	0.00277	ENSSSCG00000015556	protein coding
MSTRG.508	*HES1*$^+$ Satellite cells	-1.42	3.37	0.034	MSTRG.508	lncRNA
MSTRG.3699	*HES1*$^+$ Satellite cells	-1.41	3.9	0.0147	MSTRG.3699	lncRNA
ENSSSCG00000004572	*HES1*$^+$ Satellite cells	-1.4	5.53	0.0226	ENSSSCG00000004572	protein coding
MSTRG.39366	*HES1*$^+$ Satellite cells	-1.4	4.15	0.0332	MSTRG.39366	lncRNA
MSTRG.43969	*HES1*$^+$ Satellite cells	-1.4	4.08	0.00231	MSTRG.43969	lncRNA
ENSSSCG00000051726	*HES1*$^+$ Satellite cells	-1.39	4.3	0.0148	ENSSSCG00000051726	lncRNA
MAMDC2	*HES1*$^+$ Satellite cells	-1.38	3.79	0.00358	ENSSSCG00000030655	protein coding
MAOB	*HES1*$^+$ Satellite cells	-1.38	3.72	0.00461	ENSSSCG00000023215	protein coding
MYBPH	*HES1*$^+$ Satellite cells	-1.38	5.04	0.0254	ENSSSCG00000030681	protein coding
PREX1	*HES1*$^+$ Satellite cells	-1.38	5.18	0.0015	ENSSSCG00000031118	protein coding
ZBTB16	*HES1*$^+$ Satellite cells	-1.38	9.21	0.0233	ENSSSCG00000030095	protein coding
SLCO2A1	*HES1*$^+$ Satellite cells	-1.37	6.32	0.00142	ENSSSCG00000011641	protein coding
HSPBAP1	*HES1*$^+$ Satellite cells	-1.34	4.75	0.00226	ENSSSCG00000011873	protein coding
BMP6	*HES1*$^+$ Satellite cells	-1.33	5.52	0.0105	ENSSSCG00000001027	protein coding
ENSSSCG00000040267	*HES1*$^+$ Satellite cells	-1.33	3.65	0.0203	ENSSSCG00000040267	lncRNA
MSTRG.20405	*HES1*$^+$ Satellite cells	-1.33	4.76	0.000317	MSTRG.20405	lncRNA
MSTRG.8595	*HES1*$^+$ Satellite cells	-1.33	3.42	0.00226	MSTRG.8595	lncRNA
AGPAT3	*HES1*$^+$ Satellite cells	-1.32	6.53	0.000203	ENSSSCG00000038081	protein coding
HSD11B1	*HES1*$^+$ Satellite cells	-1.32	7.56	0.0153	ENSSSCG00000015616	protein coding
RAPGEF1	*HES1*$^+$ Satellite cells	-1.32	5.81	0.000808	ENSSSCG00000005719	protein coding
CDKL5	*HES1*$^+$ Satellite cells	-1.3	4.94	0.00458	ENSSSCG00000012156	protein coding
ENSSSCG00000004387	*HES1*$^+$ Satellite cells	-1.3	8.6	0.00199	ENSSSCG00000004387	protein coding
PLCB4	*HES1*$^+$ Satellite cells	-1.3	7.09	0.000993	ENSSSCG00000007058	protein coding
PRR5L	*HES1*$^+$ Satellite cells	-1.3	4.29	0.00435	ENSSSCG00000013292	protein coding
TEX26	*HES1*$^+$ Satellite cells	-1.3	3.81	0.0354	ENSSSCG00000046261	protein coding

MSTRG.38909	*HES1*$^+$ Satellite cells	-1.29	3.99	0.0263	MSTRG.38909	lncRNA
MSTRG.7842	*HES1*$^+$ Satellite cells	-1.29	3.54	0.00195	MSTRG.7842	lncRNA
MSTRG.7023	*HES1*$^+$ Satellite cells	-1.28	4.13	0.00151	MSTRG.7023	lncRNA
ABCC1	*HES1*$^+$ Satellite cells	-1.27	3.65	0.0239	ENSSSCG00000007874	protein coding
MSTRG.32717	*HES1*$^+$ Satellite cells	-1.27	3.45	0.00466	MSTRG.32717	lncRNA
HLF	*HES1*$^+$ Satellite cells	-1.26	4.17	0.00249	ENSSSCG00000017604	protein coding
LRRC8D	*HES1*$^+$ Satellite cells	-1.26	6.48	0.0148	ENSSSCG00000038417	protein coding
MSTRG.14445	*HES1*$^+$ Satellite cells	-1.26	4.69	0.00716	MSTRG.14445	lncRNA
MSTRG.38316	*HES1*$^+$ Satellite cells	-1.26	3.66	0.0229	MSTRG.38316	lncRNA
MSTRG.24014	*HES1*$^+$ Satellite cells	-1.24	3.43	0.00414	MSTRG.24014	lncRNA
STK33	*HES1*$^+$ Satellite cells	-1.24	4.2	0.0455	ENSSSCG00000030206	protein coding
MSTRG.40676	*HES1*$^+$ Satellite cells	-1.23	4.59	0.000345	MSTRG.40676	lncRNA
MSTRG.46541	*HES1*$^+$ Satellite cells	-1.22	4.27	0.0335	MSTRG.46541	lncRNA
MSTRG.33951	*HES1*$^+$ Satellite cells	-1.21	3.62	0.00177	MSTRG.33951	lncRNA
TMPRSS7	*HES1*$^+$ Satellite cells	-1.21	6.13	0.00668	ENSSSCG00000025721	protein coding
ZCCHC24	*HES1*$^+$ Satellite cells	-1.21	7.04	0.000114	ENSSSCG00000033074	protein coding
ARRDC2	*HES1*$^+$ Satellite cells	-1.2	5.13	0.00246	ENSSSCG00000013893	protein coding
LRRK1	*HES1*$^+$ Satellite cells	-1.2	3.65	0.0128	ENSSSCG00000004823	protein coding
HDAC4	*HES1*$^+$ Satellite cells	-1.19	4.84	0.000365	ENSSSCG00000024777	protein coding
ANO4	*HES1*$^+$ Satellite cells	-1.18	4.35	0.0441	ENSSSCG00000000873	protein coding
MSTRG.42389	*HES1*$^+$ Satellite cells	-1.18	3.64	0.0229	MSTRG.42389	lncRNA
MSTRG.9790	*HES1*$^+$ Satellite cells	-1.17	4.13	0.00153	MSTRG.9790	lncRNA
MSTRG.43974	*HES1*$^+$ Satellite cells	-1.16	7.39	0.000706	MSTRG.43974	lncRNA
B2M	*HES1*$^+$ Satellite cells	-1.15	9.41	0.0293	ENSSSCG00000004687	protein coding
TBC1D8	*HES1*$^+$ Satellite cells	-1.15	8.41	0.000665	ENSSSCG00000008169	protein coding
LANCL3	*HES1*$^+$ Satellite cells	-1.14	4.93	0.000523	ENSSSCG00000037269	protein coding
MSTRG.11604	*HES1*$^+$ Satellite cells	-1.14	4.97	0.00249	MSTRG.11604	lncRNA
PHETA2	*HES1*$^+$ Satellite cells	-1.14	4.3	0.00369	ENSSSCG00000038492	protein coding
STC2	*HES1*$^+$ Satellite cells	-1.14	3.67	0.0155	ENSSSCG00000016983	protein coding
ASS1	*HES1*$^+$ Satellite cells	-1.13	4.21	0.0041	ENSSSCG00000005701	protein coding
CGNL1	*HES1*$^+$ Satellite cells	-1.13	5.33	0.016	ENSSSCG00000035038	protein coding
MSTRG.24951	*HES1*$^+$ Satellite cells	-1.12	3.53	0.00824	MSTRG.24951	lncRNA
NFATC2	*HES1*$^+$ Satellite cells	-1.12	5.91	0.00442	ENSSSCG00000007477	protein coding
ENSSSCG00000006682	*HES1*$^+$ Satellite cells	-1.11	3.98	0.00241	ENSSSCG00000006682	protein coding

ENSSSCG00000049039	*HES1*$^{+}$ Satellite cells	-1.11	4.02	0.0102	ENSSSCG00000049039	protein coding
FADS2	*HES1*$^{+}$ Satellite cells	-1.11	4.9	0.0037	ENSSSCG00000013072	protein coding
FKBP5	*HES1*$^{+}$ Satellite cells	-1.11	8.91	0.0176	ENSSSCG00000001549	protein coding
GRIN2B	*HES1*$^{+}$ Satellite cells	-1.11	4.12	0.0403	ENSSSCG00000000614	protein coding
MOB3B	*HES1*$^{+}$ Satellite cells	-1.11	3.88	0.00373	ENSSSCG00000038811	protein coding
MSTRG.30396	*HES1*$^{+}$ Satellite cells	-1.11	4.53	0.00211	MSTRG.30396	lncRNA
BTBD11	*HES1*$^{+}$ Satellite cells	-1.1	3.83	0.00417	ENSSSCG00000000162	protein coding
USP53	*HES1*$^{+}$ Satellite cells	-1.1	6.69	0.00257	ENSSSCG00000023174	protein coding
FGGY	*HES1*$^{+}$ Satellite cells	-1.09	5.07	0.00115	ENSSSCG00000003828	protein coding
MSTRG.42628	*HES1*$^{+}$ Satellite cells	-1.09	5.95	0.00096	MSTRG.42628	lncRNA
BSN	*HES1*$^{+}$ Satellite cells	-1.07	3.92	0.019	ENSSSCG00000011384	protein coding
ENSSSCG00000013726	*HES1*$^{+}$ Satellite cells	-1.07	3.72	0.00897	ENSSSCG00000013726	protein coding
KLHDC4	*HES1*$^{+}$ Satellite cells	-1.07	4.04	0.0078	ENSSSCG00000002652	protein coding
ZMIZ1	*HES1*$^{+}$ Satellite cells	-1.07	5.73	0.0014	ENSSSCG00000010329	protein coding
MSTRG.27599	*HES1*$^{+}$ Satellite cells	-1.06	5.23	0.00982	MSTRG.27599	lncRNA
MSTRG.29775	*HES1*$^{+}$ Satellite cells	-1.06	5.23	0.0155	MSTRG.29775	lncRNA
MSTRG.31110	*HES1*$^{+}$ Satellite cells	-1.06	4	0.00938	MSTRG.31110	lncRNA
MSTRG.32698	*HES1*$^{+}$ Satellite cells	-1.06	3.97	0.0229	MSTRG.32698	lncRNA
ENOX1	*HES1*$^{+}$ Satellite cells	-1.05	7.72	0.000178	ENSSSCG00000021818	protein coding
HMCN2	*HES1*$^{+}$ Satellite cells	-1.05	9.05	0.000905	ENSSSCG00000005699	protein coding
SLC25A25	*HES1*$^{+}$ Satellite cells	-1.05	4.44	0.0139	ENSSSCG00000005636	protein coding
LIFR	*HES1*$^{+}$ Satellite cells	-1.04	7.32	0.000415	ENSSSCG00000016850	protein coding
PLEKHM1	*HES1*$^{+}$ Satellite cells	-1.04	4.96	0.00576	ENSSSCG00000017325	protein coding
ENSSSCG00000004830	*HES1*$^{+}$ Satellite cells	-1.02	5.51	0.00225	ENSSSCG00000004830	protein coding
LDLRAD4	*HES1*$^{+}$ Satellite cells	-1.02	3.48	0.0336	ENSSSCG00000027417	protein coding
FAH	*HES1*$^{+}$ Satellite cells	-1.01	4.83	0.0235	ENSSSCG00000001780	protein coding
LDB2	*HES1*$^{+}$ Satellite cells	-1.01	5.34	0.00121	ENSSSCG00000029227	protein coding
MSTRG.15079	*HES1*$^{+}$ Satellite cells	-1.01	3.85	0.0193	MSTRG.15079	lncRNA
RANGAP1	*HES1*$^{+}$ Satellite cells	-1.01	3.42	0.0117	ENSSSCG00000028202	protein coding
SETBP1	*HES1*$^{+}$ Satellite cells	-0.999	6.41	0.00192	ENSSSCG00000004490	protein coding
STAT2	*HES1*$^{+}$ Satellite cells	-0.998	3.52	0.0449	ENSSSCG00000000396	protein coding
NRXN3	*HES1*$^{+}$ Satellite cells	-0.991	4.02	0.0319	ENSSSCG00000028942	protein coding
OSBPL11	*HES1*$^{+}$ Satellite cells	-0.988	7.12	0.0071	ENSSSCG00000026753	protein coding
MSTRG.35752	*HES1*$^{+}$ Satellite cells	-0.986	8.61	0.00301	MSTRG.35752	lncRNA

MSTRG.35160	*HES1*$^{+}$ Satellite cells	-0.984	4.54	0.00127	MSTRG.35160	lncRNA
NHS	*HES1*$^{+}$ Satellite cells	-0.973	4.17	0.0323	ENSSSCG00000012151	protein coding
IGF1	*HES1*$^{+}$ Satellite cells	-0.97	6.25	0.0322	ENSSSCG00000000857	protein coding
GPR156	*HES1*$^{+}$ Satellite cells	-0.969	4.03	0.00782	ENSSSCG00000011888	protein coding
KATNBL1	*HES1*$^{+}$ Satellite cells	-0.969	6.08	0.000632	ENSSSCG00000002245	protein coding
ENSSSCG00000035371	*HES1*$^{+}$ Satellite cells	-0.968	4.27	0.0065	ENSSSCG00000035371	protein coding
ENSSSCG00000039368	*HES1*$^{+}$ Satellite cells	-0.965	4.65	0.0025	ENSSSCG00000039368	protein coding
ZNF462	*HES1*$^{+}$ Satellite cells	-0.964	6.43	0.000725	ENSSSCG00000031592	protein coding
MSTRG.40810	*HES1*$^{+}$ Satellite cells	-0.959	3.8	0.0216	MSTRG.40810	lncRNA
RRM2B	*HES1*$^{+}$ Satellite cells	-0.959	3.84	0.0112	ENSSSCG00000036139	protein coding
SLC16A9	*HES1*$^{+}$ Satellite cells	-0.959	5.72	0.0493	ENSSSCG00000010210	protein coding
TXNIP	*HES1*$^{+}$ Satellite cells	-0.958	9.85	0.00768	ENSSSCG00000031262	protein coding
MAMLD1	*HES1*$^{+}$ Satellite cells	-0.956	4.02	0.0483	ENSSSCG00000012741	protein coding
ENSSSCG00000028635	*HES1*$^{+}$ Satellite cells	-0.955	5.31	0.00675	ENSSSCG00000028635	protein coding
MSTRG.11093	*HES1*$^{+}$ Satellite cells	-0.955	5.35	0.0347	MSTRG.11093	lncRNA
PDK4	*HES1*$^{+}$ Satellite cells	-0.953	9.46	0.00312	ENSSSCG00000015334	protein coding
RXRA	*HES1*$^{+}$ Satellite cells	-0.952	3.45	0.0257	ENSSSCG00000038220	protein coding
MSTRG.20294	*HES1*$^{+}$ Satellite cells	-0.947	5.04	0.0126	MSTRG.20294	lncRNA
ARHGEF28	*HES1*$^{+}$ Satellite cells	-0.945	4.01	0.0267	ENSSSCG00000028317	protein coding
MSTRG.3240	*HES1*$^{+}$ Satellite cells	-0.944	5.07	0.0215	MSTRG.3240	lncRNA
PMEPA1	*HES1*$^{+}$ Satellite cells	-0.944	3.55	0.0191	ENSSSCG00000034259	protein coding
CRLF3	*HES1*$^{+}$ Satellite cells	-0.943	4.55	0.00369	ENSSSCG00000017743	protein coding
ENSSSCG00000018039	*HES1*$^{+}$ Satellite cells	-0.941	4	0.00968	ENSSSCG00000018039	protein coding
LPIN1	*HES1*$^{+}$ Satellite cells	-0.941	4.53	0.0215	ENSSSCG00000008624	protein coding
ADAMTS20	*HES1*$^{+}$ Satellite cells	-0.94	8.32	0.000576	ENSSSCG00000000795	protein coding
PYGL	*HES1*$^{+}$ Satellite cells	-0.937	3.57	0.0162	ENSSSCG00000005024	protein coding
TRAPPC9	*HES1*$^{+}$ Satellite cells	-0.936	4.43	0.0058	ENSSSCG00000005934	protein coding
PPP2R2B	*HES1*$^{+}$ Satellite cells	-0.933	4.8	0.00845	ENSSSCG00000028976	protein coding
URB1	*HES1*$^{+}$ Satellite cells	-0.932	3.87	0.00626	ENSSSCG00000032024	protein coding
TRIM56	*HES1*$^{+}$ Satellite cells	-0.927	3.8	0.0253	ENSSSCG00000034347	protein coding
PRPF6	*HES1*$^{+}$ Satellite cells	-0.926	4.55	0.00391	ENSSSCG00000029119	protein coding
ARFGEF2	*HES1*$^{+}$ Satellite cells	-0.916	5.16	0.0016	ENSSSCG00000007460	protein coding
NTN1	*HES1*$^{+}$ Satellite cells	-0.916	7.61	0.00277	ENSSSCG00000017993	protein coding
ST3GAL1	*HES1*$^{+}$ Satellite cells	-0.914	5.2	0.0355	ENSSSCG00000005943	protein coding

RSPRY1	*HES1* $^{+}$ Satellite cells	-0.912	4.51	0.00295	ENSSSCG00000002820	protein coding
ENSSSCG00000044326	*HES1* $^{+}$ Satellite cells	-0.909	5.1	0.0121	ENSSSCG00000044326	lncRNA
ADCY7	*HES1* $^{+}$ Satellite cells	-0.907	4.92	0.00218	ENSSSCG00000039408	protein coding
MSTRG.24269	*HES1* $^{+}$ Satellite cells	-0.907	3.72	0.0141	MSTRG.24269	lncRNA
PGAP3	*HES1* $^{+}$ Satellite cells	-0.907	3.59	0.0168	ENSSSCG00000017502	protein coding
KCNQ1	*HES1* $^{+}$ Satellite cells	-0.906	5.2	0.022	ENSSSCG00000039556	protein coding
DDRGK1	*HES1* $^{+}$ Satellite cells	-0.903	5.63	0.00167	ENSSSCG00000007160	protein coding
MSTRG.30552	*HES1* $^{+}$ Satellite cells	-0.903	3.97	0.0252	MSTRG.30552	lncRNA
KLHL29	*HES1* $^{+}$ Satellite cells	-0.902	3.75	0.048	ENSSSCG00000008593	protein coding
MSTRG.3698	*HES1* $^{+}$ Satellite cells	-0.897	5.44	0.0155	MSTRG.3698	lncRNA
MSTRG.40219	*HES1* $^{+}$ Satellite cells	-0.897	4.2	0.0215	MSTRG.40219	lncRNA
MSTRG.36550	*HES1* $^{+}$ Satellite cells	-0.892	4.03	0.0315	MSTRG.36550	lncRNA
PRR12	*HES1* $^{+}$ Satellite cells	-0.89	3.58	0.0169	ENSSSCG00000032668	protein coding
DTX4	*HES1* $^{+}$ Satellite cells	-0.884	5.89	0.0025	ENSSSCG00000013145	protein coding
LRIG1	*HES1* $^{+}$ Satellite cells	-0.881	7.18	0.00362	ENSSSCG00000011499	protein coding
ZFAND5	*HES1* $^{+}$ Satellite cells	-0.88	8.57	0.0177	ENSSSCG00000030268	protein coding
ENSSSCG00000008898	*HES1* $^{+}$ Satellite cells	-0.875	8.56	0.0284	ENSSSCG00000008898	protein coding
KCNQ5	*HES1* $^{+}$ Satellite cells	-0.873	6.1	0.00432	ENSSSCG00000004281	protein coding
ARMC2	*HES1* $^{+}$ Satellite cells	-0.871	6.65	0.00812	ENSSSCG00000004388	protein coding
LONRF3	*HES1* $^{+}$ Satellite cells	-0.869	6.43	0.00848	ENSSSCG00000012631	protein coding
ABR	*HES1* $^{+}$ Satellite cells	-0.868	4.64	0.00992	ENSSSCG00000017804	protein coding
ENSSSCG00000009914	*HES1* $^{+}$ Satellite cells	-0.867	4.6	0.0197	ENSSSCG00000009914	protein coding
MSTRG.14344	*HES1* $^{+}$ Satellite cells	-0.867	4.05	0.00735	MSTRG.14344	lncRNA
MSTRG.35258	*HES1* $^{+}$ Satellite cells	-0.863	3.61	0.0153	MSTRG.35258	lncRNA
TTC39B	*HES1* $^{+}$ Satellite cells	-0.861	4.38	0.0102	ENSSSCG00000005186	protein coding
MSTRG.6817	*HES1* $^{+}$ Satellite cells	-0.859	5.16	0.0363	MSTRG.6817	lncRNA
PLXNA2	*HES1* $^{+}$ Satellite cells	-0.859	7.06	0.000825	ENSSSCG00000026412	protein coding
FRY	*HES1* $^{+}$ Satellite cells	-0.854	6.11	0.0224	ENSSSCG00000009338	protein coding
GLI2	*HES1* $^{+}$ Satellite cells	-0.851	8.7	0.00306	ENSSSCG00000015733	protein coding
PAK3	*HES1* $^{+}$ Satellite cells	-0.851	4.38	0.0196	ENSSSCG00000025306	protein coding
CNKSR2	*HES1* $^{+}$ Satellite cells	-0.849	6.27	0.00254	ENSSSCG00000012164	protein coding
ENSSSCG00000027115	*HES1* $^{+}$ Satellite cells	-0.848	5.76	0.00995	ENSSSCG00000027115	protein coding
MSTRG.26556	*HES1* $^{+}$ Satellite cells	-0.848	4.12	0.0373	MSTRG.26556	lncRNA
PPP1R10	*HES1* $^{+}$ Satellite cells	-0.848	6.8	0.00223	ENSSSCG00000001347	protein coding

HSD17B7	*HES1*$^+$ Satellite cells	-0.847	4.84	0.00708	ENSSSCG00000006337	protein coding
DDIT4	*HES1*$^+$ Satellite cells	-0.844	8.46	0.0026	ENSSSCG00000031888	protein coding
PI4KA	*HES1*$^+$ Satellite cells	-0.842	4.75	0.00469	ENSSSCG00000010092	protein coding
FANCI	*HES1*$^+$ Satellite cells	-0.841	3.5	0.0149	ENSSSCG00000001837	protein coding
MSTRG.21276	*HES1*$^+$ Satellite cells	-0.841	3.98	0.0231	MSTRG.21276	lncRNA
MSTRG.43970	*HES1*$^+$ Satellite cells	-0.841	4.57	0.0427	MSTRG.43970	lncRNA
MSTRG.19111	*HES1*$^+$ Satellite cells	-0.839	5.52	0.00346	MSTRG.19111	lncRNA
PHLDB1	*HES1*$^+$ Satellite cells	-0.834	5.32	0.00817	ENSSSCG00000024578	protein coding
ENSSSCG00000001229	*HES1*$^+$ Satellite cells	-0.832	7.81	0.00379	ENSSSCG00000001229	protein coding
MSTRG.19413	*HES1*$^+$ Satellite cells	-0.828	4.76	0.0301	MSTRG.19413	lncRNA
NCOR2	*HES1*$^+$ Satellite cells	-0.819	6.92	0.00189	ENSSSCG00000009761	protein coding
SEMA4B	*HES1*$^+$ Satellite cells	-0.819	5.67	0.00197	ENSSSCG00000031719	protein coding
SNX11	*HES1*$^+$ Satellite cells	-0.818	3.96	0.0399	ENSSSCG00000017528	protein coding
MSTRG.11522	*HES1*$^+$ Satellite cells	-0.816	3.89	0.0166	MSTRG.11522	lncRNA
AMOT	*HES1*$^+$ Satellite cells	-0.813	5.51	0.00154	ENSSSCG00000012591	protein coding
ENSSSCG00000050111	*HES1*$^+$ Satellite cells	-0.81	3.71	0.0465	ENSSSCG00000050111	lncRNA
MAP3K5	*HES1*$^+$ Satellite cells	-0.81	5.67	0.00664	ENSSSCG00000026041	protein coding
MSTRG.6795	*HES1*$^+$ Satellite cells	-0.81	3.78	0.0157	MSTRG.6795	lncRNA
BOD1L1	*HES1*$^+$ Satellite cells	-0.809	4.56	0.0167	ENSSSCG00000008735	protein coding
MSTRG.41031	*HES1*$^+$ Satellite cells	-0.807	5.23	0.0474	MSTRG.41031	lncRNA
PPRC1	*HES1*$^+$ Satellite cells	-0.807	4.17	0.0161	ENSSSCG00000010575	protein coding
NIBAN1	*HES1*$^+$ Satellite cells	-0.802	4.59	0.0272	ENSSSCG00000015567	protein coding
MFN2	*HES1*$^+$ Satellite cells	-0.801	4.24	0.0252	ENSSSCG00000003434	protein coding
ZKSCAN1	*HES1*$^+$ Satellite cells	-0.801	4.18	0.014	ENSSSCG00000031461	protein coding
TLN2	*HES1*$^+$ Satellite cells	-0.795	9.14	0.00823	ENSSSCG00000024088	protein coding
GOLGA1	*HES1*$^+$ Satellite cells	-0.792	4.4	0.0138	ENSSSCG00000005597	protein coding
MSTRG.22353	*HES1*$^+$ Satellite cells	-0.792	4.07	0.0306	MSTRG.22353	lncRNA
CACNB2	*HES1*$^+$ Satellite cells	-0.79	4.74	0.0215	ENSSSCG00000011040	protein coding
CHKA	*HES1*$^+$ Satellite cells	-0.79	4.61	0.0163	ENSSSCG00000012889	protein coding
SLC3A2	*HES1*$^+$ Satellite cells	-0.79	5.37	0.0103	ENSSSCG00000022404	protein coding
ELMOD1	*HES1*$^+$ Satellite cells	-0.787	3.73	0.0306	ENSSSCG00000015002	protein coding
INPPL1	*HES1*$^+$ Satellite cells	-0.785	4.41	0.0125	ENSSSCG00000014811	protein coding
SASH1	*HES1*$^+$ Satellite cells	-0.782	9.39	0.000979	ENSSSCG00000033175	protein coding
TSTD2	*HES1*$^+$ Satellite cells	-0.782	4.34	0.0405	ENSSSCG00000005367	protein coding

ZC3H7B	*HES1*$^+$ Satellite cells	-0.782	5.31	0.00415	ENSSSCG00000033036	protein coding
ENSSSCG00000004082	*HES1*$^+$ Satellite cells	-0.781	6.76	0.00778	ENSSSCG00000004082	protein coding
ZFP28	*HES1*$^+$ Satellite cells	-0.781	3.99	0.0201	ENSSSCG00000027774	protein coding
SESN1	*HES1*$^+$ Satellite cells	-0.78	8.7	0.00405	ENSSSCG00000004390	protein coding
DENND1B	*HES1*$^+$ Satellite cells	-0.779	4.06	0.0416	ENSSSCG00000010900	protein coding
LHFPL2	*HES1*$^+$ Satellite cells	-0.779	7.45	0.000805	ENSSSCG00000028536	protein coding
SNTB1	*HES1*$^+$ Satellite cells	-0.779	6.54	0.0151	ENSSSCG00000005994	protein coding
CHST8	*HES1*$^+$ Satellite cells	-0.777	4.83	0.0356	ENSSSCG00000035222	protein coding
GAP43	*HES1*$^+$ Satellite cells	-0.776	4.68	0.014	ENSSSCG00000031244	protein coding
LRMDA	*HES1*$^+$ Satellite cells	-0.776	8.15	0.0189	ENSSSCG00000034308	protein coding
ENSSSCG00000011859	*HES1*$^+$ Satellite cells	-0.773	4.97	0.0128	ENSSSCG00000011859	protein coding
MAN1C1	*HES1*$^+$ Satellite cells	-0.773	4.74	0.0457	ENSSSCG00000023235	protein coding
MSTRG.5748	*HES1*$^+$ Satellite cells	-0.772	4.09	0.016	MSTRG.5748	lncRNA
PLCG1	*HES1*$^+$ Satellite cells	-0.77	4.28	0.0169	ENSSSCG00000007356	protein coding
TRIM4	*HES1*$^+$ Satellite cells	-0.769	3.81	0.0201	ENSSSCG00000007637	protein coding
MSTRG.8115	*HES1*$^+$ Satellite cells	-0.768	6.01	0.00911	MSTRG.8115	lncRNA
ATP1A4	*HES1*$^+$ Satellite cells	-0.767	5.42	0.0228	ENSSSCG00000006391	protein coding
KIAA1217	*HES1*$^+$ Satellite cells	-0.767	5.07	0.0244	ENSSSCG00000011075	protein coding
ATP9A	*HES1*$^+$ Satellite cells	-0.766	5.74	0.00758	ENSSSCG00000007478	protein coding
MSTRG.2298	*HES1*$^+$ Satellite cells	-0.766	3.9	0.0472	MSTRG.2298	lncRNA
SNED1	*HES1*$^+$ Satellite cells	-0.761	5.5	0.00619	ENSSSCG00000016381	protein coding
ENSSSCG00000035856	*HES1*$^+$ Satellite cells	-0.758	4.08	0.0367	ENSSSCG00000035856	protein coding
ACTB	*HES1*$^+$ Satellite cells	-0.754	4.49	0.00811	ENSSSCG00000007585	protein coding
RIPK1	*HES1*$^+$ Satellite cells	-0.751	5.57	0.00894	ENSSSCG00000001009	protein coding
C14orf93	*HES1*$^+$ Satellite cells	-0.748	4.12	0.0172	ENSSSCG00000031452	protein coding
ENSSSCG00000004415	*HES1*$^+$ Satellite cells	-0.748	3.93	0.0432	ENSSSCG00000004415	protein coding
MSTRG.16410	*HES1*$^+$ Satellite cells	-0.747	4.68	0.0281	MSTRG.16410	lncRNA
TOR3A	*HES1*$^+$ Satellite cells	-0.747	4.6	0.00623	ENSSSCG00000015525	protein coding
SNAP47	*HES1*$^+$ Satellite cells	-0.744	3.77	0.0315	ENSSSCG00000013984	protein coding
OSBPL5	*HES1*$^+$ Satellite cells	-0.742	4.18	0.0385	ENSSSCG00000012862	protein coding
UTP23	*HES1*$^+$ Satellite cells	-0.742	3.92	0.0355	ENSSSCG00000030493	protein coding
POLM	*HES1*$^+$ Satellite cells	-0.741	4.57	0.025	ENSSSCG00000016755	protein coding
SLC25A20	*HES1*$^+$ Satellite cells	-0.735	5	0.0245	ENSSSCG00000011366	protein coding
MSTRG.48113	*HES1*$^+$ Satellite cells	-0.734	4.09	0.0335	MSTRG.48113	lncRNA

CLMP	*HES1*$^{+}$ Satellite cells	-0.733	5.66	0.0207	ENSSSCG00000022961	protein coding
PAAF1	*HES1*$^{+}$ Satellite cells	-0.733	5	0.00738	ENSSSCG00000014831	protein coding
SACS	*HES1*$^{+}$ Satellite cells	-0.733	3.54	0.0329	ENSSSCG00000035493	protein coding
EIF4G1	*HES1*$^{+}$ Satellite cells	-0.731	4.52	0.0128	ENSSSCG00000030255	protein coding
ANKRD54	*HES1*$^{+}$ Satellite cells	-0.726	4.52	0.014	ENSSSCG00000000120	protein coding
ERCC3	*HES1*$^{+}$ Satellite cells	-0.724	4.4	0.0443	ENSSSCG00000015721	protein coding
METTL16	*HES1*$^{+}$ Satellite cells	-0.724	4.37	0.0165	ENSSSCG00000017832	protein coding
REV3L	*HES1*$^{+}$ Satellite cells	-0.724	7.9	0.0221	ENSSSCG00000025703	protein coding
TGS1	*HES1*$^{+}$ Satellite cells	-0.722	4.77	0.0117	ENSSSCG00000006251	protein coding
RASL11B	*HES1*$^{+}$ Satellite cells	-0.718	6.42	0.00128	ENSSSCG00000008835	protein coding
MAPK10	*HES1*$^{+}$ Satellite cells	-0.712	6.14	0.0361	ENSSSCG00000009228	protein coding
PARVA	*HES1*$^{+}$ Satellite cells	-0.709	6.72	0.00594	ENSSSCG00000028827	protein coding
MSTRG.20211	*HES1*$^{+}$ Satellite cells	-0.708	3.73	0.0246	MSTRG.20211	lncRNA
CYP2E1	*HES1*$^{+}$ Satellite cells	-0.707	3.68	0.0487	ENSSSCG00000010780	protein coding
RRAGD	*HES1*$^{+}$ Satellite cells	-0.706	5.13	0.0135	ENSSSCG00000004318	protein coding
ECE1	*HES1*$^{+}$ Satellite cells	-0.704	4.87	0.0435	ENSSSCG00000003513	protein coding
KCNN3	*HES1*$^{+}$ Satellite cells	-0.702	7.33	0.0369	ENSSSCG00000006542	protein coding
MTMR1	*HES1*$^{+}$ Satellite cells	-0.701	5.05	0.0425	ENSSSCG00000012743	protein coding
SRSF7	*HES1*$^{+}$ Satellite cells	-0.701	7.69	0.00362	ENSSSCG00000008484	protein coding
CXCL12	*HES1*$^{+}$ Satellite cells	-0.699	7.6	0.0321	ENSSSCG00000034973	protein coding
COL14A1	*HES1*$^{+}$ Satellite cells	-0.698	9.22	0.0206	ENSSSCG00000005997	protein coding
ADAMTS9	*HES1*$^{+}$ Satellite cells	-0.697	7.31	0.0198	ENSSSCG00000011496	protein coding
KDM5C	*HES1*$^{+}$ Satellite cells	-0.697	5.05	0.00947	ENSSSCG00000012322	protein coding
SART3	*HES1*$^{+}$ Satellite cells	-0.697	4.75	0.00578	ENSSSCG00000009946	protein coding
HDHD2	*HES1*$^{+}$ Satellite cells	-0.696	4.76	0.0327	ENSSSCG00000005771	protein coding
TMEM107	*HES1*$^{+}$ Satellite cells	-0.695	6.33	0.0228	ENSSSCG00000027528	protein coding
ENSSSCG00000047749	*HES1*$^{+}$ Satellite cells	-0.694	4.45	0.0225	ENSSSCG00000047749	protein coding
RHOJ	*HES1*$^{+}$ Satellite cells	-0.694	6.19	0.0163	ENSSSCG00000022804	protein coding
SEMA4D	*HES1*$^{+}$ Satellite cells	-0.694	5.86	0.00537	ENSSSCG00000009584	protein coding
ESRRG	*HES1*$^{+}$ Satellite cells	-0.693	4.26	0.0374	ENSSSCG00000010814	protein coding
APBB2	*HES1*$^{+}$ Satellite cells	-0.691	8.5	0.0153	ENSSSCG00000022168	protein coding
MBD1	*HES1*$^{+}$ Satellite cells	-0.69	4.47	0.0197	ENSSSCG00000004516	protein coding
IL6R	*HES1*$^{+}$ Satellite cells	-0.689	5.92	0.0448	ENSSSCG00000027628	protein coding
ENSSSCG00000050854	*HES1*$^{+}$ Satellite cells	-0.688	5.98	0.00527	ENSSSCG00000050854	lncRNA

CPT1A	*HES1*$^{+}$ Satellite cells	-0.686	5.37	0.0382	ENSSSCG00000012880	protein coding
MSTRG.10446	*HES1*$^{+}$ Satellite cells	-0.686	4.08	0.0404	MSTRG.10446	lncRNA
ENSSSCG00000033340	*HES1*$^{+}$ Satellite cells	-0.684	3.77	0.0433	ENSSSCG00000033340	protein coding
GCN1	*HES1*$^{+}$ Satellite cells	-0.684	5.06	0.0106	ENSSSCG00000009898	protein coding
DUSP29	*HES1*$^{+}$ Satellite cells	-0.683	4.01	0.0325	ENSSSCG00000010317	protein coding
FOXO1	*HES1*$^{+}$ Satellite cells	-0.681	7.25	0.0309	ENSSSCG00000009370	protein coding
MYH14	*HES1*$^{+}$ Satellite cells	-0.681	7.15	0.00172	ENSSSCG00000027407	protein coding
INTS3	*HES1*$^{+}$ Satellite cells	-0.68	4.32	0.0265	ENSSSCG00000006571	protein coding
EGFR	*HES1*$^{+}$ Satellite cells	-0.679	7.49	0.00637	ENSSSCG00000022126	protein coding
POLR3A	*HES1*$^{+}$ Satellite cells	-0.677	4.08	0.0321	ENSSSCG00000010327	protein coding
SEMA6C	*HES1*$^{+}$ Satellite cells	-0.676	4.01	0.028	ENSSSCG00000006635	protein coding
PRPF8	*HES1*$^{+}$ Satellite cells	-0.675	5.49	0.00628	ENSSSCG00000017817	protein coding
USF3	*HES1*$^{+}$ Satellite cells	-0.673	4.39	0.0209	ENSSSCG00000011918	protein coding
FAM13A	*HES1*$^{+}$ Satellite cells	-0.669	6.87	0.00258	ENSSSCG00000026196	protein coding
DHX37	*HES1*$^{+}$ Satellite cells	-0.668	3.8	0.0397	ENSSSCG00000009758	protein coding
ZNF217	*HES1*$^{+}$ Satellite cells	-0.668	3.99	0.0495	ENSSSCG00000007484	protein coding
ENSSSCG00000000107	*HES1*$^{+}$ Satellite cells	-0.666	4.32	0.0277	ENSSSCG00000000107	protein coding
FOSB	*HES1*$^{+}$ Satellite cells	-0.666	10.2	0.0176	ENSSSCG00000031616	protein coding
NRCAM	*HES1*$^{+}$ Satellite cells	-0.666	6.93	0.00565	ENSSSCG00000015445	protein coding
ENSSSCG00000016119	*HES1*$^{+}$ Satellite cells	-0.665	8.1	0.0201	ENSSSCG00000016119	protein coding
MSTRG.36167	*HES1*$^{+}$ Satellite cells	-0.665	3.59	0.0469	MSTRG.36167	lncRNA
PDE3A	*HES1*$^{+}$ Satellite cells	-0.665	8.12	0.0174	ENSSSCG00000028662	protein coding
ENSSSCG00000005241	*HES1*$^{+}$ Satellite cells	-0.663	4.83	0.0148	ENSSSCG00000005241	protein coding
MSTRG.30836	*HES1*$^{+}$ Satellite cells	-0.662	6.67	0.0223	MSTRG.30836	lncRNA
PPTC7	*HES1*$^{+}$ Satellite cells	-0.661	4.17	0.0322	ENSSSCG00000009825	protein coding
CEP68	*HES1*$^{+}$ Satellite cells	-0.66	3.9	0.0391	ENSSSCG00000008360	protein coding
KLF13	*HES1*$^{+}$ Satellite cells	-0.66	6.57	0.039	ENSSSCG00000026006	protein coding
ENSSSCG00000002003	*HES1*$^{+}$ Satellite cells	-0.657	4.63	0.0307	ENSSSCG00000002003	protein coding
OSGIN1	*HES1*$^{+}$ Satellite cells	-0.653	5.52	0.0185	ENSSSCG00000032115	protein coding
SGCA	*HES1*$^{+}$ Satellite cells	-0.653	7.88	0.00324	ENSSSCG00000017583	protein coding
SNX9	*HES1*$^{+}$ Satellite cells	-0.653	5.32	0.0178	ENSSSCG00000030535	protein coding
ENSSSCG00000047993	*HES1*$^{+}$ Satellite cells	-0.65	5.55	0.0215	ENSSSCG00000047993	lncRNA
EXOSC10	*HES1*$^{+}$ Satellite cells	-0.65	4.56	0.0368	ENSSSCG00000003412	protein coding
FAM174B	*HES1*$^{+}$ Satellite cells	-0.648	5.37	0.0316	ENSSSCG00000002265	protein coding

PIK3R1	*HES1*$^+$ Satellite cells	-0.647	7.73	0.035	ENSSSCG00000016958	protein coding
FAM110A	*HES1*$^+$ Satellite cells	-0.645	4.14	0.0358	ENSSSCG00000007200	protein coding
KIAA0319L	*HES1*$^+$ Satellite cells	-0.645	5.2	0.00989	ENSSSCG00000037121	protein coding
MSTRG.29944	*HES1*$^+$ Satellite cells	-0.644	5.21	0.0438	MSTRG.29944	lncRNA
DENND2A	*HES1*$^+$ Satellite cells	-0.643	4.24	0.0318	ENSSSCG00000016497	protein coding
ENSSSCG00000014280	*HES1*$^+$ Satellite cells	-0.643	6.9	0.0398	ENSSSCG00000014280	protein coding
MSTRG.25857	*HES1*$^+$ Satellite cells	-0.643	4.33	0.0327	MSTRG.25857	lncRNA
BCAR3	*HES1*$^+$ Satellite cells	-0.641	6.27	0.0327	ENSSSCG00000006893	protein coding
CLUAP1	*HES1*$^+$ Satellite cells	-0.64	3.76	0.0478	ENSSSCG00000007955	protein coding
AGAP1	*HES1*$^+$ Satellite cells	-0.639	7.68	0.0149	ENSSSCG00000016317	protein coding
ENSSSCG00000001970	*HES1*$^+$ Satellite cells	-0.636	3.91	0.0315	ENSSSCG00000001970	protein coding
SLCO3A1	*HES1*$^+$ Satellite cells	-0.636	5.53	0.0232	ENSSSCG00000002263	protein coding
ABCF1	*HES1*$^+$ Satellite cells	-0.635	5.06	0.0188	ENSSSCG00000001346	protein coding
FOXRED2	*HES1*$^+$ Satellite cells	-0.635	5.62	0.0116	ENSSSCG00000000142	protein coding
MSTRG.14856	*HES1*$^+$ Satellite cells	-0.633	5.12	0.0399	MSTRG.14856	lncRNA
HSPG2	*HES1*$^+$ Satellite cells	-0.632	8.75	0.00437	ENSSSCG00000003514	protein coding
MPHOSPH10	*HES1*$^+$ Satellite cells	-0.632	4.35	0.0175	ENSSSCG00000004847	protein coding
SYNRG	*HES1*$^+$ Satellite cells	-0.632	5.02	0.0164	ENSSSCG00000026596	protein coding
ZNF536	*HES1*$^+$ Satellite cells	-0.632	5.91	0.0324	ENSSSCG00000029234	protein coding
PRRC2A	*HES1*$^+$ Satellite cells	-0.631	5.66	0.00835	ENSSSCG00000001409	protein coding
ENSSSCG00000005255	*HES1*$^+$ Satellite cells	-0.629	7.09	0.0141	ENSSSCG00000005255	protein coding
SZT2	*HES1*$^+$ Satellite cells	-0.629	4.38	0.0343	ENSSSCG00000003941	protein coding
EDA	*HES1*$^+$ Satellite cells	-0.627	5.28	0.0402	ENSSSCG00000021647	protein coding
CUEDC1	*HES1*$^+$ Satellite cells	-0.625	4.78	0.0197	ENSSSCG00000017624	protein coding
CCDC173	*HES1*$^+$ Satellite cells	-0.624	6.14	0.0316	ENSSSCG00000015935	protein coding
ITPR1	*HES1*$^+$ Satellite cells	-0.624	5.24	0.0142	ENSSSCG00000023437	protein coding
PIP5K1B	*HES1*$^+$ Satellite cells	-0.621	4.3	0.0357	ENSSSCG00000027161	protein coding
GSTO1	*HES1*$^+$ Satellite cells	-0.619	5.38	0.0474	ENSSSCG00000022351	protein coding
TCAF1	*HES1*$^+$ Satellite cells	-0.617	5.28	0.0206	ENSSSCG00000016453	protein coding
PRKCB	*HES1*$^+$ Satellite cells	-0.615	4.77	0.0376	ENSSSCG00000033674	protein coding
HSPB8	*HES1*$^+$ Satellite cells	-0.614	6.76	0.00671	ENSSSCG00000009844	protein coding
STAT5A	*HES1*$^+$ Satellite cells	-0.614	3.95	0.0388	ENSSSCG00000017402	protein coding
PRKDC	*HES1*$^+$ Satellite cells	-0.613	4.49	0.019	ENSSSCG00000006274	protein coding
MON1B	*HES1*$^+$ Satellite cells	-0.612	4.63	0.0271	ENSSSCG00000002701	protein coding

TMEM63A	*HES1*$^{+}$ Satellite cells	-0.612	4.34	0.0252	ENSSSCG00000010854	protein coding
FRMD4A	*HES1*$^{+}$ Satellite cells	-0.611	6.38	0.0343	ENSSSCG00000011056	protein coding
CCNL2	*HES1*$^{+}$ Satellite cells	-0.61	4.92	0.0313	ENSSSCG00000003354	protein coding
ZFHX3	*HES1*$^{+}$ Satellite cells	-0.61	4.33	0.0353	ENSSSCG00000002752	protein coding
ENSSSCG00000033497	*HES1*$^{+}$ Satellite cells	-0.609	7.81	0.00664	ENSSSCG00000033497	protein coding
ENSSSCG00000010034	*HES1*$^{+}$ Satellite cells	-0.607	4.1	0.0366	ENSSSCG00000010034	protein coding
SLC49A4	*HES1*$^{+}$ Satellite cells	-0.605	5.98	0.0388	ENSSSCG00000036766	protein coding
EEFSEC	*HES1*$^{+}$ Satellite cells	-0.604	4.9	0.0242	ENSSSCG00000011619	protein coding
TEAD3	*HES1*$^{+}$ Satellite cells	-0.604	6.06	0.0108	ENSSSCG00000001544	protein coding
CHD8	*HES1*$^{+}$ Satellite cells	-0.603	4.51	0.0422	ENSSSCG00000002127	protein coding
CACHD1	*HES1*$^{+}$ Satellite cells	-0.601	6.01	0.0394	ENSSSCG00000004829	protein coding
IGF2R	*HES1*$^{+}$ Satellite cells	-0.598	7.15	0.0472	ENSSSCG00000004044	protein coding
KCNMA1	*HES1*$^{+}$ Satellite cells	-0.598	5.43	0.0462	ENSSSCG00000010325	protein coding
POR	*HES1*$^{+}$ Satellite cells	-0.598	6.09	0.0182	ENSSSCG00000038320	protein coding
FGFR1	*HES1*$^{+}$ Satellite cells	-0.597	5.92	0.0485	ENSSSCG00000015815	protein coding
SMC1A	*HES1*$^{+}$ Satellite cells	-0.597	5.68	0.015	ENSSSCG00000012325	protein coding
ENSSSCG00000041731	*HES1*$^{+}$ Satellite cells	-0.596	4.99	0.0397	ENSSSCG00000041731	protein coding
SPIDR	*HES1*$^{+}$ Satellite cells	-0.595	7.3	0.0288	ENSSSCG00000006277	protein coding
MAST2	*HES1*$^{+}$ Satellite cells	-0.593	4.83	0.0116	ENSSSCG00000003913	protein coding
GNL2	*HES1*$^{+}$ Satellite cells	-0.592	4.65	0.0484	ENSSSCG00000031631	protein coding
MSTRG.9565	*HES1*$^{+}$ Satellite cells	-0.591	5.06	0.0193	MSTRG.9565	lncRNA
CWC25	*HES1*$^{+}$ Satellite cells	-0.588	4.59	0.023	ENSSSCG00000021478	protein coding
ENSSSCG00000002259	*HES1*$^{+}$ Satellite cells	-0.587	8.12	0.0146	ENSSSCG00000002259	protein coding
KDM5A	*HES1*$^{+}$ Satellite cells	-0.582	5.48	0.0125	ENSSSCG00000029464	protein coding
TXLNA	*HES1*$^{+}$ Satellite cells	-0.582	5.01	0.0283	ENSSSCG00000003617	protein coding
MDC1	*HES1*$^{+}$ Satellite cells	-0.581	3.94	0.048	ENSSSCG00000001362	protein coding
ENSSSCG00000002260	*HES1*$^{+}$ Satellite cells	-0.58	6.28	0.00504	ENSSSCG00000002260	protein coding
KLF4	*HES1*$^{+}$ Satellite cells	-0.577	8.01	0.0248	ENSSSCG00000005437	protein coding
ENSSSCG00000017165	*HES1*$^{+}$ Satellite cells	-0.574	4.98	0.0308	ENSSSCG00000017165	protein coding
FOXK2	*HES1*$^{+}$ Satellite cells	-0.573	4.09	0.0469	ENSSSCG00000017125	protein coding
LEMD2	*HES1*$^{+}$ Satellite cells	-0.573	4.45	0.0283	ENSSSCG00000001520	protein coding
COL4A2	*HES1*$^{+}$ Satellite cells	-0.568	7.99	0.00793	ENSSSCG00000009545	protein coding
ENSSSCG00000031810	*HES1*$^{+}$ Satellite cells	-0.568	4.64	0.0377	ENSSSCG00000031810	protein coding
NRF1	*HES1*$^{+}$ Satellite cells	-0.563	5.93	0.0194	ENSSSCG00000016566	protein coding

ADAM17	*HES1*$^{+}$ Satellite cells	-0.562	5.2	0.0499	ENSSSCG00000008641	protein coding
JSRP1	*HES1*$^{+}$ Satellite cells	-0.56	7.54	0.00547	ENSSSCG00000031899	protein coding
AP4S1	*HES1*$^{+}$ Satellite cells	-0.559	4.87	0.0363	ENSSSCG00000001972	protein coding
ZCCHC14	*HES1*$^{+}$ Satellite cells	-0.559	5.53	0.0106	ENSSSCG00000002656	protein coding
MTOR	*HES1*$^{+}$ Satellite cells	-0.556	4.82	0.0212	ENSSSCG00000003413	protein coding
NUP214	*HES1*$^{+}$ Satellite cells	-0.556	5.04	0.0264	ENSSSCG00000005711	protein coding
SYNE3	*HES1*$^{+}$ Satellite cells	-0.555	5.57	0.0177	ENSSSCG00000002495	protein coding
MSTRG.36142	*HES1*$^{+}$ Satellite cells	-0.554	4.08	0.0434	MSTRG.36142	lncRNA
MSTRG.21028	*HES1*$^{+}$ Satellite cells	-0.551	4.98	0.0299	MSTRG.21028	lncRNA
CCDC6	*HES1*$^{+}$ Satellite cells	-0.55	6.56	0.0141	ENSSSCG00000010211	protein coding
MTRR	*HES1*$^{+}$ Satellite cells	-0.55	4.94	0.0248	ENSSSCG00000017100	protein coding
EIF2B3	*HES1*$^{+}$ Satellite cells	-0.549	5.82	0.0472	ENSSSCG00000029808	protein coding
ENSSSCG00000039972	*HES1*$^{+}$ Satellite cells	-0.549	4.66	0.0302	ENSSSCG00000039972	protein coding
GOLGA3	*HES1*$^{+}$ Satellite cells	-0.548	4.25	0.0494	ENSSSCG00000009733	protein coding
ULK2	*HES1*$^{+}$ Satellite cells	-0.546	5.83	0.0295	ENSSSCG00000018045	protein coding
DUSP10	*HES1*$^{+}$ Satellite cells	-0.545	6.38	0.0273	ENSSSCG00000010831	protein coding
GCC2	*HES1*$^{+}$ Satellite cells	-0.545	4.68	0.0465	ENSSSCG00000008138	protein coding
TRIP11	*HES1*$^{+}$ Satellite cells	-0.543	5.65	0.0133	ENSSSCG00000002445	protein coding
MSTRG.27279	*HES1*$^{+}$ Satellite cells	-0.541	5.8	0.0353	MSTRG.27279	lncRNA
DDX23	*HES1*$^{+}$ Satellite cells	-0.54	5.06	0.0358	ENSSSCG00000000176	protein coding
UTP20	*HES1*$^{+}$ Satellite cells	-0.54	4.74	0.0452	ENSSSCG00000000869	protein coding
DDC	*HES1*$^{+}$ Satellite cells	-0.539	5.98	0.0193	ENSSSCG00000022009	protein coding
NAV3	*HES1*$^{+}$ Satellite cells	-0.535	7.1	0.0209	ENSSSCG00000029249	protein coding
AKAP6	*HES1*$^{+}$ Satellite cells	-0.534	5.08	0.037	ENSSSCG00000024517	protein coding
ENSSSCG00000010440	*HES1*$^{+}$ Satellite cells	-0.532	6.24	0.00813	ENSSSCG00000010440	protein coding
ENSSSCG00000011162	*HES1*$^{+}$ Satellite cells	-0.532	5.03	0.0259	ENSSSCG00000011162	protein coding
INPP4A	*HES1*$^{+}$ Satellite cells	-0.53	4.88	0.0296	ENSSSCG00000008190	protein coding
CTNNBL1	*HES1*$^{+}$ Satellite cells	-0.528	6.47	0.0483	ENSSSCG00000007337	protein coding
MSTRG.16606	*HES1*$^{+}$ Satellite cells	-0.528	4.51	0.0444	MSTRG.16606	lncRNA
RNF216	*HES1*$^{+}$ Satellite cells	-0.528	5.79	0.0194	ENSSSCG00000021285	protein coding
ENSSSCG00000043464	*HES1*$^{+}$ Satellite cells	-0.527	5.49	0.0219	ENSSSCG00000043464	lncRNA
EIF2AK4	*HES1*$^{+}$ Satellite cells	-0.525	5.77	0.0172	ENSSSCG00000039061	protein coding
AMOTL1	*HES1*$^{+}$ Satellite cells	-0.523	6.76	0.00945	ENSSSCG00000014960	protein coding
C1orf21	*HES1*$^{+}$ Satellite cells	-0.521	6.82	0.0322	ENSSSCG00000015565	protein coding

ARHGAP10	*HES1*$^{+}$ Satellite cells	-0.518	8.99	0.0227	ENSSSCG00000009029	protein coding
GIGYF2	*HES1*$^{+}$ Satellite cells	-0.515	5.56	0.0338	ENSSSCG00000016291	protein coding
MKNK1	*HES1*$^{+}$ Satellite cells	-0.515	5.02	0.0262	ENSSSCG00000003898	protein coding
RASA3	*HES1*$^{+}$ Satellite cells	-0.514	5.04	0.0333	ENSSSCG00000009567	protein coding
RNF149	*HES1*$^{+}$ Satellite cells	-0.513	5.33	0.0229	ENSSSCG00000008168	protein coding
STX18	*HES1*$^{+}$ Satellite cells	-0.513	5.35	0.0357	ENSSSCG00000038731	protein coding
ZNF407	*HES1*$^{+}$ Satellite cells	-0.51	5.26	0.0332	ENSSSCG00000033971	protein coding
DDX46	*HES1*$^{+}$ Satellite cells	-0.509	6.05	0.0288	ENSSSCG00000026644	protein coding
TOP3A	*HES1*$^{+}$ Satellite cells	-0.508	5.77	0.0291	ENSSSCG00000035839	protein coding
RPTOR	*HES1*$^{+}$ Satellite cells	-0.507	5.69	0.02	ENSSSCG00000017139	protein coding
LRP6	*HES1*$^{+}$ Satellite cells	-0.506	7.15	0.0222	ENSSSCG00000000625	protein coding
CTIF	*HES1*$^{+}$ Satellite cells	-0.503	7.14	0.043	ENSSSCG00000004506	protein coding
NFIX	*HES1*$^{+}$ Satellite cells	-0.502	7.28	0.0144	ENSSSCG00000013742	protein coding
ENSSSCG00000021744	*HES1*$^{+}$ Satellite cells	-0.501	5.92	0.0284	ENSSSCG00000021744	protein coding
SETD7	*HES1*$^{+}$ Satellite cells	-0.501	5.89	0.0376	ENSSSCG00000030396	protein coding
ACLY	*HES1*$^{+}$ Satellite cells	-0.5	4.81	0.0372	ENSSSCG00000017421	protein coding
C21orf91	*HES1*$^{+}$ Satellite cells	-0.496	6.48	0.0214	ENSSSCG00000033025	protein coding
MRPL9	*HES1*$^{+}$ Satellite cells	-0.493	5.78	0.0477	ENSSSCG00000028343	protein coding
HLTF	*HES1*$^{+}$ Satellite cells	-0.492	5.03	0.0483	ENSSSCG00000025293	protein coding
CREM	*HES1*$^{+}$ Satellite cells	-0.491	5.25	0.0454	ENSSSCG00000011106	protein coding
IKBIP	*HES1*$^{+}$ Satellite cells	-0.49	5.55	0.0209	ENSSSCG00000038772	protein coding
USF1	*HES1*$^{+}$ Satellite cells	-0.488	5.23	0.0459	ENSSSCG00000006371	protein coding
ENSSSCG00000040854	*HES1*$^{+}$ Satellite cells	-0.484	6.55	0.0384	ENSSSCG00000040854	protein coding
FYCO1	*HES1*$^{+}$ Satellite cells	-0.484	5.24	0.0455	ENSSSCG00000011317	protein coding
ABL2	*HES1*$^{+}$ Satellite cells	-0.482	5	0.043	ENSSSCG00000024674	protein coding
ENSSSCG00000000697	*HES1*$^{+}$ Satellite cells	-0.482	5.75	0.019	ENSSSCG00000000697	protein coding
MSTRG.14908	*HES1*$^{+}$ Satellite cells	-0.482	6.69	0.0388	MSTRG.14908	lncRNA
BANP	*HES1*$^{+}$ Satellite cells	-0.481	4.76	0.0442	ENSSSCG00000020678	protein coding
TLN1	*HES1*$^{+}$ Satellite cells	-0.481	5.47	0.0465	ENSSSCG00000005317	protein coding
CYRIA	*HES1*$^{+}$ Satellite cells	-0.48	7.31	0.0169	ENSSSCG00000008617	protein coding
MICU2	*HES1*$^{+}$ Satellite cells	-0.48	6.25	0.0282	ENSSSCG00000024667	protein coding
NLK	*HES1*$^{+}$ Satellite cells	-0.479	4.76	0.0414	ENSSSCG00000017756	protein coding
TBL2	*HES1*$^{+}$ Satellite cells	-0.479	5.09	0.0264	ENSSSCG00000007709	protein coding
R3HDM1	*HES1*$^{+}$ Satellite cells	-0.478	6.31	0.0181	ENSSSCG00000015692	protein coding

ENSSSCG00000038594	*HES1* + Satellite cells	-0.477	6.93	0.0351	ENSSSCG00000038594	protein coding
KDM4B	*HES1* + Satellite cells	-0.477	5.04	0.0438	ENSSSCG00000024570	protein coding
SESTD1	*HES1* + Satellite cells	-0.477	7.73	0.0472	ENSSSCG00000016005	protein coding
PLOD1	*HES1* + Satellite cells	-0.475	6.8	0.0299	ENSSSCG00000003433	protein coding
USP7	*HES1* + Satellite cells	-0.47	5.25	0.0336	ENSSSCG00000007907	protein coding
ENSSSCG00000016330	*HES1* + Satellite cells	-0.468	6.31	0.0235	ENSSSCG00000016330	protein coding
REEP3	*HES1* + Satellite cells	-0.468	6.47	0.0492	ENSSSCG00000036476	protein coding
PRPF3	*HES1* + Satellite cells	-0.467	5.6	0.0314	ENSSSCG00000030119	protein coding
ENSSSCG00000050244	*HES1* + Satellite cells	-0.464	6.48	0.0342	ENSSSCG00000050244	protein coding
XPO6	*HES1* + Satellite cells	-0.459	5.14	0.0315	ENSSSCG00000007812	protein coding
PPP2R1B	*HES1* + Satellite cells	-0.458	5.13	0.0384	ENSSSCG00000029538	protein coding
RANBP3	*HES1* + Satellite cells	-0.457	5.87	0.0257	ENSSSCG00000013533	protein coding
HECTD1	*HES1* + Satellite cells	-0.452	6.62	0.0194	ENSSSCG00000001971	protein coding
DCTPP1	*HES1* + Satellite cells	-0.451	6.58	0.0363	ENSSSCG00000007795	protein coding
ARID1B	*HES1* + Satellite cells	-0.448	7.31	0.0247	ENSSSCG00000004064	protein coding
NEK9	*HES1* + Satellite cells	-0.445	5.06	0.0417	ENSSSCG00000002381	protein coding
IDH2	*HES1* + Satellite cells	-0.441	7	0.0419	ENSSSCG00000001852	protein coding
VPS53	*HES1* + Satellite cells	-0.437	5.34	0.045	ENSSSCG00000017812	protein coding
ARHGAP21	*HES1* + Satellite cells	-0.426	7.35	0.0301	ENSSSCG00000011074	protein coding
PRRC2C	*HES1* + Satellite cells	-0.425	7.35	0.0431	ENSSSCG00000015480	protein coding
USP40	*HES1* + Satellite cells	-0.425	5.54	0.0409	ENSSSCG00000016306	protein coding
DAG1	*HES1* + Satellite cells	-0.422	9.63	0.044	ENSSSCG00000034357	protein coding
CFAP20	*HES1* + Satellite cells	-0.421	6.49	0.0239	ENSSSCG00000002805	protein coding
ENSSSCG00000044700	*HES1* + Satellite cells	-0.419	8.8	0.0432	ENSSSCG00000044700	protein coding
DOCK1	*HES1* + Satellite cells	-0.414	7.11	0.0307	ENSSSCG00000035045	protein coding
ENSSSCG00000034868	*HES1* + Satellite cells	-0.412	6.54	0.0352	ENSSSCG00000034868	protein coding
TTC17	*HES1* + Satellite cells	-0.408	6.19	0.0449	ENSSSCG00000013284	protein coding
NAA20	*HES1* + Satellite cells	-0.405	7.07	0.0316	ENSSSCG00000028132	protein coding
CHD6	*HES1* + Satellite cells	-0.397	6.64	0.0364	ENSSSCG00000007360	protein coding
SBNO2	*HES1* + Satellite cells	-0.391	6.04	0.0471	ENSSSCG00000030042	protein coding
GNL3	*HES1* + Satellite cells	-0.384	5.92	0.045	ENSSSCG00000011449	protein coding
HEATR3	*HES1* + Satellite cells	-0.372	6.2	0.0418	ENSSSCG00000040887	protein coding
FAM126A	*HES1* + Satellite cells	0.364	6.19	0.0497	ENSSSCG00000015386	protein coding
EIF1B	*HES1* + Satellite cells	0.374	7.17	0.0405	ENSSSCG00000011270	protein coding

ANKRD28	*HES1*$^+$ Satellite cells	0.381	6.06	0.0484	ENSSSCG00000011194	protein coding
COPS2	*HES1*$^+$ Satellite cells	0.39	6.67	0.0465	ENSSSCG00000004652	protein coding
NUDT21	*HES1*$^+$ Satellite cells	0.391	6.45	0.0419	ENSSSCG00000034521	protein coding
SRP19	*HES1*$^+$ Satellite cells	0.391	7.51	0.0394	ENSSSCG00000026230	protein coding
ENSSSCG00000024019	*HES1*$^+$ Satellite cells	0.393	6.53	0.0336	ENSSSCG00000024019	protein coding
IGF2BP3	*HES1*$^+$ Satellite cells	0.393	6.06	0.039	ENSSSCG00000036695	protein coding
HP1BP3	*HES1*$^+$ Satellite cells	0.397	6.18	0.0359	ENSSSCG00000003511	protein coding
SSBP2	*HES1*$^+$ Satellite cells	0.397	8.22	0.0382	ENSSSCG00000035757	protein coding
ENSSSCG00000033368	*HES1*$^+$ Satellite cells	0.398	8.59	0.0389	ENSSSCG00000033368	protein coding
C4orf3	*HES1*$^+$ Satellite cells	0.399	7.36	0.0456	ENSSSCG00000031773	protein coding
ENSSSCG00000004970	*HES1*$^+$ Satellite cells	0.399	13.2	0.0363	ENSSSCG00000004970	protein coding
ENSSSCG00000008451	*HES1*$^+$ Satellite cells	0.4	6.71	0.0467	ENSSSCG00000008451	protein coding
TCEA1	*HES1*$^+$ Satellite cells	0.4	6.38	0.03	ENSSSCG00000006261	protein coding
TM2D1	*HES1*$^+$ Satellite cells	0.4	6.62	0.0342	ENSSSCG00000038861	protein coding
ENSSSCG00000046054	*HES1*$^+$ Satellite cells	0.403	8.35	0.0328	ENSSSCG00000046054	lncRNA
ARL8B	*HES1*$^+$ Satellite cells	0.407	5.95	0.0403	ENSSSCG00000011535	protein coding
ENSSSCG00000003903	*HES1*$^+$ Satellite cells	0.41	8.49	0.0465	ENSSSCG00000003903	protein coding
H2AZ2	*HES1*$^+$ Satellite cells	0.418	8.01	0.0483	ENSSSCG00000016738	protein coding
ENSSSCG00000038364	*HES1*$^+$ Satellite cells	0.421	7.06	0.0381	ENSSSCG00000038364	protein coding
RIOK3	*HES1*$^+$ Satellite cells	0.421	6.46	0.0475	ENSSSCG00000029518	protein coding
UBE2D3	*HES1*$^+$ Satellite cells	0.421	7.89	0.0411	ENSSSCG00000035622	protein coding
MMADHC	*HES1*$^+$ Satellite cells	0.423	6.44	0.0379	ENSSSCG00000028646	protein coding
HERPUD2	*HES1*$^+$ Satellite cells	0.424	6.09	0.0341	ENSSSCG00000027389	protein coding
MSTRG.21070	*HES1*$^+$ Satellite cells	0.424	5.94	0.03	MSTRG.21070	lncRNA
RPS29	*HES1*$^+$ Satellite cells	0.424	12	0.0414	ENSSSCG00000038507	protein coding
VBP1	*HES1*$^+$ Satellite cells	0.426	5.82	0.0483	ENSSSCG00000012811	protein coding
DERL1	*HES1*$^+$ Satellite cells	0.427	6.64	0.0388	ENSSSCG00000033690	protein coding
ENSSSCG00000012427	*HES1*$^+$ Satellite cells	0.428	12	0.0329	ENSSSCG00000012427	protein coding
PAIP2	*HES1*$^+$ Satellite cells	0.43	8.4	0.0273	ENSSSCG00000026606	protein coding
DPYD	*HES1*$^+$ Satellite cells	0.433	6.94	0.0284	ENSSSCG00000006878	protein coding
EPS15	*HES1*$^+$ Satellite cells	0.438	7.52	0.0224	ENSSSCG00000003872	protein coding
TRIB1	*HES1*$^+$ Satellite cells	0.439	6.65	0.022	ENSSSCG00000038500	protein coding
SYPL1	*HES1*$^+$ Satellite cells	0.44	6.79	0.0316	ENSSSCG00000015434	protein coding
METAP2	*HES1*$^+$ Satellite cells	0.441	6.53	0.028	ENSSSCG00000030608	protein coding

NIPSNAP2	*HES1*$^{+}$ Satellite cells	0.441	6.09	0.0214	ENSSSCG00000036659	protein coding
RPL37	*HES1*$^{+}$ Satellite cells	0.442	12.2	0.0316	ENSSSCG00000033326	protein coding
SLC35F5	*HES1*$^{+}$ Satellite cells	0.442	5.28	0.0405	ENSSSCG00000015709	protein coding
HNRNPDL	*HES1*$^{+}$ Satellite cells	0.444	9.04	0.0203	ENSSSCG00000037245	protein coding
ZC2HC1A	*HES1*$^{+}$ Satellite cells	0.444	5.91	0.0382	ENSSSCG00000006162	protein coding
CLDND1	*HES1*$^{+}$ Satellite cells	0.448	5.62	0.043	ENSSSCG00000011980	protein coding
EIF2S2	*HES1*$^{+}$ Satellite cells	0.448	7.22	0.0302	ENSSSCG00000026617	protein coding
VGLL3	*HES1*$^{+}$ Satellite cells	0.448	6.01	0.0407	ENSSSCG00000032398	protein coding
UBC	*HES1*$^{+}$ Satellite cells	0.449	10.4	0.0283	ENSSSCG00000029226	protein coding
HNRNPA0	*HES1*$^{+}$ Satellite cells	0.451	7.71	0.0455	ENSSSCG00000042630	protein coding
SUFU	*HES1*$^{+}$ Satellite cells	0.451	7.74	0.0326	ENSSSCG00000010586	protein coding
TOMM20	*HES1*$^{+}$ Satellite cells	0.451	7.02	0.02	ENSSSCG00000010158	protein coding
MSTRG.30002	*HES1*$^{+}$ Satellite cells	0.452	8.97	0.0378	MSTRG.30002	lncRNA
PTBP3	*HES1*$^{+}$ Satellite cells	0.453	6.48	0.0256	ENSSSCG00000005466	protein coding
LSM2	*HES1*$^{+}$ Satellite cells	0.455	5.96	0.0397	ENSSSCG00000039021	protein coding
UBALD1	*HES1*$^{+}$ Satellite cells	0.457	5.58	0.0387	ENSSSCG00000023603	protein coding
TAF12	*HES1*$^{+}$ Satellite cells	0.458	5.15	0.0382	ENSSSCG00000027530	protein coding
HSD11B1L	*HES1*$^{+}$ Satellite cells	0.46	11.8	0.0266	ENSSSCG00000030546	protein coding
ENSSSCG00000031838	*HES1*$^{+}$ Satellite cells	0.462	12.5	0.0227	ENSSSCG00000031838	protein coding
CNOT6	*HES1*$^{+}$ Satellite cells	0.463	5.68	0.0401	ENSSSCG00000014009	protein coding
GNAI3	*HES1*$^{+}$ Satellite cells	0.463	6.06	0.0253	ENSSSCG00000006824	protein coding
MSTRG.180	*HES1*$^{+}$ Satellite cells	0.463	7.48	0.0326	MSTRG.180	lncRNA
EIF3M	*HES1*$^{+}$ Satellite cells	0.464	7.12	0.049	ENSSSCG00000013315	protein coding
PMP22	*HES1*$^{+}$ Satellite cells	0.465	7.84	0.0282	ENSSSCG00000037832	protein coding
RPL7	*HES1*$^{+}$ Satellite cells	0.465	12.1	0.0177	ENSSSCG00000032111	protein coding
UBE2E3	*HES1*$^{+}$ Satellite cells	0.465	6.99	0.0289	ENSSSCG00000033987	protein coding
ST13	*HES1*$^{+}$ Satellite cells	0.466	8.33	0.0226	ENSSSCG00000000071	protein coding
EIF1AX	*HES1*$^{+}$ Satellite cells	0.467	6.42	0.0305	ENSSSCG00000032223	protein coding
ENSSSCG00000027778	*HES1*$^{+}$ Satellite cells	0.468	6.2	0.0401	ENSSSCG00000027778	protein coding
ARPC3	*HES1*$^{+}$ Satellite cells	0.469	6.89	0.0291	ENSSSCG00000009821	protein coding
BTF3L4	*HES1*$^{+}$ Satellite cells	0.469	6.56	0.0197	ENSSSCG00000038762	protein coding
CLPX	*HES1*$^{+}$ Satellite cells	0.47	6.07	0.0421	ENSSSCG00000004927	protein coding
CHMP5	*HES1*$^{+}$ Satellite cells	0.473	6.3	0.0279	ENSSSCG00000010995	protein coding
DSTN	*HES1*$^{+}$ Satellite cells	0.473	6.96	0.0444	ENSSSCG00000007085	protein coding

KARS1	*HES1*$^{+}$ Satellite cells	0.473	8.67	0.0425	ENSSSCG00000002704	protein coding
KCNAB3	*HES1*$^{+}$ Satellite cells	0.474	5.86	0.0378	ENSSSCG00000037973	protein coding
ADAM10	*HES1*$^{+}$ Satellite cells	0.477	6.25	0.0404	ENSSSCG00000004595	protein coding
CBLB	*HES1*$^{+}$ Satellite cells	0.477	6.5	0.0498	ENSSSCG00000022486	protein coding
ACTL6A	*HES1*$^{+}$ Satellite cells	0.478	4.71	0.0494	ENSSSCG00000011762	protein coding
GOLGA7	*HES1*$^{+}$ Satellite cells	0.478	5.99	0.0171	ENSSSCG00000020715	protein coding
NPM1	*HES1*$^{+}$ Satellite cells	0.479	9.37	0.0351	ENSSSCG00000016998	protein coding
SMNDC1	*HES1*$^{+}$ Satellite cells	0.479	5.71	0.0324	ENSSSCG00000030507	protein coding
RB1	*HES1*$^{+}$ Satellite cells	0.48	6.32	0.0406	ENSSSCG00000009401	protein coding
B3GNTL1	*HES1*$^{+}$ Satellite cells	0.482	5.1	0.0443	ENSSSCG00000027229	protein coding
NOL7	*HES1*$^{+}$ Satellite cells	0.482	7.01	0.0378	ENSSSCG00000040816	protein coding
TYW3	*HES1*$^{+}$ Satellite cells	0.482	4.53	0.0408	ENSSSCG00000003779	protein coding
ZNHIT1	*HES1*$^{+}$ Satellite cells	0.482	6.9	0.0141	ENSSSCG00000033982	protein coding
UGDH	*HES1*$^{+}$ Satellite cells	0.484	6.65	0.048	ENSSSCG00000030150	protein coding
NAP1L1	*HES1*$^{+}$ Satellite cells	0.488	7.84	0.0363	ENSSSCG00000000522	protein coding
ABI1	*HES1*$^{+}$ Satellite cells	0.49	5.71	0.0331	ENSSSCG00000024127	protein coding
OSBPL9	*HES1*$^{+}$ Satellite cells	0.491	6.41	0.025	ENSSSCG00000021292	protein coding
SPART	*HES1*$^{+}$ Satellite cells	0.494	5.52	0.0226	ENSSSCG00000024635	protein coding
IAH1	*HES1*$^{+}$ Satellite cells	0.495	5.81	0.0413	ENSSSCG00000008640	protein coding
LACTB2	*HES1*$^{+}$ Satellite cells	0.495	5.75	0.0189	ENSSSCG00000006192	protein coding
C1QBP	*HES1*$^{+}$ Satellite cells	0.497	6.93	0.0309	ENSSSCG00000035048	protein coding
RPL38	*HES1*$^{+}$ Satellite cells	0.497	11.2	0.0273	ENSSSCG00000017239	protein coding
PFDN4	*HES1*$^{+}$ Satellite cells	0.498	6.13	0.0378	ENSSSCG00000007487	protein coding
SEC61G	*HES1*$^{+}$ Satellite cells	0.498	8.53	0.0387	ENSSSCG00000035434	protein coding
TMEM159	*HES1*$^{+}$ Satellite cells	0.498	5.33	0.0273	ENSSSCG00000031876	protein coding
CHIC2	*HES1*$^{+}$ Satellite cells	0.499	7.17	0.0259	ENSSSCG00000032645	protein coding
SUMF1	*HES1*$^{+}$ Satellite cells	0.5	6.48	0.0202	ENSSSCG00000011532	protein coding
RPL15	*HES1*$^{+}$ Satellite cells	0.501	11.1	0.0245	ENSSSCG00000037030	protein coding
SUMO1	*HES1*$^{+}$ Satellite cells	0.501	6.98	0.0145	ENSSSCG00000035842	protein coding
ENSSSCG00000007088	*HES1*$^{+}$ Satellite cells	0.502	6.37	0.0203	ENSSSCG00000007088	protein coding
ENSSSCG00000012452	*HES1*$^{+}$ Satellite cells	0.504	5.94	0.0187	ENSSSCG00000012452	protein coding
PM20D2	*HES1*$^{+}$ Satellite cells	0.504	5.23	0.0218	ENSSSCG00000004314	protein coding
POLR2I	*HES1*$^{+}$ Satellite cells	0.505	7	0.0133	ENSSSCG00000039196	protein coding
PTP4A1	*HES1*$^{+}$ Satellite cells	0.505	5.32	0.0328	ENSSSCG00000025941	protein coding

RASSF3	*HES1*$^+$ Satellite cells	0.505	7.67	0.0281	ENSSSCG00000039060	protein coding
SRP9	*HES1*$^+$ Satellite cells	0.505	5.02	0.03	ENSSSCG00000031664	protein coding
ZBED5	*HES1*$^+$ Satellite cells	0.505	5.65	0.0342	ENSSSCG00000033411	protein coding
POLB	*HES1*$^+$ Satellite cells	0.506	5.04	0.0418	ENSSSCG00000007031	protein coding
GPX4	*HES1*$^+$ Satellite cells	0.507	7.04	0.0241	ENSSSCG00000024052	protein coding
LCORL	*HES1*$^+$ Satellite cells	0.509	5.43	0.0319	ENSSSCG00000008748	protein coding
PNRC2	*HES1*$^+$ Satellite cells	0.513	7.19	0.0251	ENSSSCG00000023326	protein coding
RNF150	*HES1*$^+$ Satellite cells	0.513	7.51	0.0396	ENSSSCG00000009053	protein coding
ATP6AP2	*HES1*$^+$ Satellite cells	0.515	6.36	0.0255	ENSSSCG00000012247	protein coding
GNG10	*HES1*$^+$ Satellite cells	0.515	7.84	0.0116	ENSSSCG00000046439	protein coding
SPAG8	*HES1*$^+$ Satellite cells	0.515	6.5	0.017	ENSSSCG00000025842	protein coding
BTF3	*HES1*$^+$ Satellite cells	0.516	10.1	0.0365	ENSSSCG00000014071	protein coding
CCNC	*HES1*$^+$ Satellite cells	0.516	5.82	0.0234	ENSSSCG00000004353	protein coding
DST	*HES1*$^+$ Satellite cells	0.516	5.85	0.0415	ENSSSCG00000001499	protein coding
RBM3	*HES1*$^+$ Satellite cells	0.517	8.26	0.018	ENSSSCG00000021591	protein coding
RAB11A	*HES1*$^+$ Satellite cells	0.518	5.71	0.0442	ENSSSCG00000032312	protein coding
RPS15A	*HES1*$^+$ Satellite cells	0.518	12.1	0.0164	ENSSSCG00000035768	protein coding
RPS3A	*HES1*$^+$ Satellite cells	0.518	12.1	0.0134	ENSSSCG00000009019	protein coding
CAMLG	*HES1*$^+$ Satellite cells	0.522	5.02	0.0204	ENSSSCG00000038955	protein coding
DENR	*HES1*$^+$ Satellite cells	0.524	6.33	0.0124	ENSSSCG00000009788	protein coding
GTF3C6	*HES1*$^+$ Satellite cells	0.524	4.95	0.0361	ENSSSCG00000004393	protein coding
RPA3	*HES1*$^+$ Satellite cells	0.525	6.21	0.0333	ENSSSCG00000023886	protein coding
ARPC5	*HES1*$^+$ Satellite cells	0.526	6.33	0.0407	ENSSSCG00000015560	protein coding
H2AJ	*HES1*$^+$ Satellite cells	0.526	5.65	0.0458	ENSSSCG00000039307	protein coding
MSTRG.12822	*HES1*$^+$ Satellite cells	0.526	6.56	0.0358	MSTRG.12822	lncRNA
NIPSNAP3A	*HES1*$^+$ Satellite cells	0.526	6.57	0.0109	ENSSSCG00000005424	protein coding
RTN4	*HES1*$^+$ Satellite cells	0.53	7.34	0.019	ENSSSCG00000032007	protein coding
DEPDC1B	*HES1*$^+$ Satellite cells	0.533	4.9	0.0234	ENSSSCG00000035012	protein coding
RSRP1	*HES1*$^+$ Satellite cells	0.534	6.18	0.0346	ENSSSCG00000034449	protein coding
MSTRG.39789	*HES1*$^+$ Satellite cells	0.535	5.92	0.0232	MSTRG.39789	lncRNA
ATIC	*HES1*$^+$ Satellite cells	0.537	4.79	0.0236	ENSSSCG00000016173	protein coding
CYLD	*HES1*$^+$ Satellite cells	0.538	4.71	0.0306	ENSSSCG00000040286	protein coding
CCT8	*HES1*$^+$ Satellite cells	0.539	7.05	0.0131	ENSSSCG00000026761	protein coding
IVNS1ABP	*HES1*$^+$ Satellite cells	0.539	5.88	0.0271	ENSSSCG00000015570	protein coding

ACBD5	*HES1* $^{+}$ Satellite cells	0.54	5.77	0.0392	ENSSSCG00000011064	protein coding
TBPL1	*HES1* $^{+}$ Satellite cells	0.54	5.8	0.014	ENSSSCG00000022387	protein coding
ENSSSCG00000039544	*HES1* $^{+}$ Satellite cells	0.541	11.9	0.0118	ENSSSCG00000039544	protein coding
CAPZA2	*HES1* $^{+}$ Satellite cells	0.542	7.75	0.015	ENSSSCG00000028113	protein coding
SLC12A2	*HES1* $^{+}$ Satellite cells	0.542	4.94	0.044	ENSSSCG00000014255	protein coding
CD164	*HES1* $^{+}$ Satellite cells	0.543	7.7	0.0121	ENSSSCG00000004414	protein coding
COMMD9	*HES1* $^{+}$ Satellite cells	0.544	5.02	0.0293	ENSSSCG00000013293	protein coding
RNF11	*HES1* $^{+}$ Satellite cells	0.547	6.11	0.0185	ENSSSCG00000031172	protein coding
CAPRIN1	*HES1* $^{+}$ Satellite cells	0.548	6.4	0.00992	ENSSSCG00000013305	protein coding
MSL3	*HES1* $^{+}$ Satellite cells	0.548	5.08	0.0247	ENSSSCG00000012114	protein coding
SPAST	*HES1* $^{+}$ Satellite cells	0.549	4.22	0.0441	ENSSSCG00000008517	protein coding
COX7A2L	*HES1* $^{+}$ Satellite cells	0.55	8.13	0.012	ENSSSCG00000008466	protein coding
DUSP16	*HES1* $^{+}$ Satellite cells	0.55	5.85	0.0343	ENSSSCG00000032097	protein coding
ENSSSCG00000000097	*HES1* $^{+}$ Satellite cells	0.554	6.46	0.0312	ENSSSCG00000000097	protein coding
ENSSSCG00000026064	*HES1* $^{+}$ Satellite cells	0.555	6.91	0.0062	ENSSSCG00000026064	protein coding
MGMT	*HES1* $^{+}$ Satellite cells	0.555	5.81	0.0424	ENSSSCG00000010756	protein coding
RPS28	*HES1* $^{+}$ Satellite cells	0.555	12.2	0.0114	ENSSSCG00000013597	protein coding
HES1	*HES1* $^{+}$ Satellite cells	0.556	9.13	0.0221	ENSSSCG00000031356	protein coding
NDFIP2	*HES1* $^{+}$ Satellite cells	0.556	5.4	0.0304	ENSSSCG00000009480	protein coding
CCT4	*HES1* $^{+}$ Satellite cells	0.559	7.48	0.00646	ENSSSCG00000008377	protein coding
YAP1	*HES1* $^{+}$ Satellite cells	0.559	6.04	0.0233	ENSSSCG00000031429	protein coding
HMGB2	*HES1* $^{+}$ Satellite cells	0.56	6.94	0.0333	ENSSSCG00000009704	protein coding
NFU1	*HES1* $^{+}$ Satellite cells	0.56	5.27	0.0226	ENSSSCG00000025007	protein coding
ZBTB10	*HES1* $^{+}$ Satellite cells	0.56	5.92	0.0341	ENSSSCG00000006155	protein coding
SEC22B	*HES1* $^{+}$ Satellite cells	0.561	5.55	0.0477	ENSSSCG00000020964	protein coding
PRKCSH	*HES1* $^{+}$ Satellite cells	0.562	6.69	0.0413	ENSSSCG00000013627	protein coding
APOOL	*HES1* $^{+}$ Satellite cells	0.563	4.72	0.0378	ENSSSCG00000012459	protein coding
STOM	*HES1* $^{+}$ Satellite cells	0.563	5.15	0.0458	ENSSSCG00000036639	protein coding
ZNF704	*HES1* $^{+}$ Satellite cells	0.563	6.65	0.0331	ENSSSCG00000022989	protein coding
ATP6V1G1	*HES1* $^{+}$ Satellite cells	0.565	7.52	0.00949	ENSSSCG00000032835	protein coding
MSTRG.37309	*HES1* $^{+}$ Satellite cells	0.565	4.67	0.0446	MSTRG.37309	lncRNA
ENSSSCG00000011764	*HES1* $^{+}$ Satellite cells	0.566	7.18	0.00789	ENSSSCG00000011764	protein coding
SPATA1	*HES1* $^{+}$ Satellite cells	0.566	4.25	0.029	ENSSSCG00000029882	protein coding
ENSSSCG00000009772	*HES1* $^{+}$ Satellite cells	0.567	8.05	0.0323	ENSSSCG00000009772	protein coding

TSPAN6	*HES1*$^{+}$ Satellite cells	0.567	6.14	0.00741	ENSSSCG00000012481	protein coding
ARMC10	*HES1*$^{+}$ Satellite cells	0.569	5.91	0.0276	ENSSSCG00000028444	protein coding
C19orf53	*HES1*$^{+}$ Satellite cells	0.57	6.41	0.011	ENSSSCG00000013757	protein coding
C5orf15	*HES1*$^{+}$ Satellite cells	0.57	6.07	0.0116	ENSSSCG00000032323	protein coding
MSTRG.28593	*HES1*$^{+}$ Satellite cells	0.57	4.56	0.0405	MSTRG.28593	lncRNA
ENSSSCG00000033734	*HES1*$^{+}$ Satellite cells	0.572	6.85	0.00789	ENSSSCG00000033734	protein coding
CDC42	*HES1*$^{+}$ Satellite cells	0.573	8.23	0.00905	ENSSSCG00000003520	protein coding
ENSSSCG00000020725	*HES1*$^{+}$ Satellite cells	0.573	11.9	0.00972	ENSSSCG00000020725	protein coding
GRSF1	*HES1*$^{+}$ Satellite cells	0.573	5.44	0.0424	ENSSSCG00000008940	protein coding
RTRAF	*HES1*$^{+}$ Satellite cells	0.573	8.12	0.0163	ENSSSCG00000005029	protein coding
TANC1	*HES1*$^{+}$ Satellite cells	0.574	6.62	0.0167	ENSSSCG00000015880	protein coding
PKIA	*HES1*$^{+}$ Satellite cells	0.575	7.6	0.0167	ENSSSCG00000006163	protein coding
ERRFI1	*HES1*$^{+}$ Satellite cells	0.577	6.01	0.0262	ENSSSCG00000035774	protein coding
DMAC2	*HES1*$^{+}$ Satellite cells	0.578	6.7	0.0113	ENSSSCG00000032711	protein coding
ENSSSCG00000012178	*HES1*$^{+}$ Satellite cells	0.578	7.39	0.0284	ENSSSCG00000012178	protein coding
HDAC2	*HES1*$^{+}$ Satellite cells	0.578	6.92	0.00694	ENSSSCG00000004432	protein coding
GATAD2A	*HES1*$^{+}$ Satellite cells	0.583	6.51	0.0495	ENSSSCG00000013938	protein coding
DCTN2	*HES1*$^{+}$ Satellite cells	0.584	7.19	0.0434	ENSSSCG00000000444	protein coding
MSTRG.15925	*HES1*$^{+}$ Satellite cells	0.585	4.03	0.0477	MSTRG.15925	lncRNA
MSTRG.1135	*HES1*$^{+}$ Satellite cells	0.588	7.46	0.0122	MSTRG.1135	lncRNA
CZIB	*HES1*$^{+}$ Satellite cells	0.59	5.58	0.0375	ENSSSCG00000003850	protein coding
STAU2	*HES1*$^{+}$ Satellite cells	0.59	5.21	0.035	ENSSSCG00000006179	protein coding
TMEM50B	*HES1*$^{+}$ Satellite cells	0.59	5.64	0.0395	ENSSSCG00000034624	protein coding
TMEM126B	*HES1*$^{+}$ Satellite cells	0.592	4.67	0.0417	ENSSSCG00000014905	protein coding
MSTRG.21044	*HES1*$^{+}$ Satellite cells	0.593	4.2	0.049	MSTRG.21044	lncRNA
SCAMP1	*HES1*$^{+}$ Satellite cells	0.596	6.27	0.0175	ENSSSCG00000034795	protein coding
DHRS7B	*HES1*$^{+}$ Satellite cells	0.598	5.6	0.0115	ENSSSCG00000031802	protein coding
RIDA	*HES1*$^{+}$ Satellite cells	0.599	5.41	0.0217	ENSSSCG00000006079	protein coding
MSTRG.15926	*HES1*$^{+}$ Satellite cells	0.6	5.9	0.0279	MSTRG.15926	lncRNA
BNIP3L	*HES1*$^{+}$ Satellite cells	0.601	6.11	0.0166	ENSSSCG00000009658	protein coding
CMTM8	*HES1*$^{+}$ Satellite cells	0.601	5.19	0.0325	ENSSSCG00000022618	protein coding
CRK	*HES1*$^{+}$ Satellite cells	0.601	9.06	0.0116	ENSSSCG00000038989	protein coding
NDUFAF2	*HES1*$^{+}$ Satellite cells	0.601	4.11	0.0474	ENSSSCG00000027763	protein coding
RNF170	*HES1*$^{+}$ Satellite cells	0.603	3.93	0.0432	ENSSSCG00000036001	protein coding

RAD51B	*HES1*$^+$ Satellite cells	0.604	6.33	0.00411	ENSSSCG00000028877	protein coding
MSTRG.20719	*HES1*$^+$ Satellite cells	0.606	4.27	0.0318	MSTRG.20719	lncRNA
ENSSSCG00000000288	*HES1*$^+$ Satellite cells	0.607	9.37	0.0291	ENSSSCG00000000288	protein coding
PLPP1	*HES1*$^+$ Satellite cells	0.607	8	0.00483	ENSSSCG00000016912	protein coding
ENSSSCG00000028423	*HES1*$^+$ Satellite cells	0.608	6.9	0.00808	ENSSSCG00000028423	protein coding
FEM1C	*HES1*$^+$ Satellite cells	0.608	5.21	0.0352	ENSSSCG00000014217	protein coding
FLAD1	*HES1*$^+$ Satellite cells	0.608	5.75	0.0317	ENSSSCG00000006538	protein coding
PPP1CC	*HES1*$^+$ Satellite cells	0.608	7.5	0.0166	ENSSSCG00000009828	protein coding
SETD9	*HES1*$^+$ Satellite cells	0.609	3.99	0.0493	ENSSSCG00000016919	protein coding
BMPR1B	*HES1*$^+$ Satellite cells	0.612	4.08	0.042	ENSSSCG00000029621	protein coding
ARF6	*HES1*$^+$ Satellite cells	0.615	6.63	0.0159	ENSSSCG00000005012	protein coding
ENSSSCG00000037595	*HES1*$^+$ Satellite cells	0.616	5.87	0.0304	ENSSSCG00000037595	protein coding
RCBTB2	*HES1*$^+$ Satellite cells	0.617	4.77	0.0397	ENSSSCG00000009400	protein coding
ZFAND2B	*HES1*$^+$ Satellite cells	0.62	4.17	0.039	ENSSSCG00000016208	protein coding
RAB6A	*HES1*$^+$ Satellite cells	0.621	6.1	0.008	ENSSSCG00000014828	protein coding
CDK5RAP3	*HES1*$^+$ Satellite cells	0.627	4.59	0.0141	ENSSSCG00000017524	protein coding
SKP2	*HES1*$^+$ Satellite cells	0.628	5	0.00814	ENSSSCG00000016835	protein coding
C10orf143	*HES1*$^+$ Satellite cells	0.629	5.12	0.0253	ENSSSCG00000040426	protein coding
REEP5	*HES1*$^+$ Satellite cells	0.629	8.1	0.0204	ENSSSCG00000022048	protein coding
RFC4	*HES1*$^+$ Satellite cells	0.629	4.84	0.0234	ENSSSCG00000011804	protein coding
ENSSSCG00000033733	*HES1*$^+$ Satellite cells	0.631	7.86	0.0141	ENSSSCG00000033733	protein coding
MSTRG.24294	*HES1*$^+$ Satellite cells	0.631	4.16	0.0267	MSTRG.24294	lncRNA
DNAJB4	*HES1*$^+$ Satellite cells	0.632	7.84	0.0178	ENSSSCG00000003766	protein coding
MFF	*HES1*$^+$ Satellite cells	0.632	6.81	0.00247	ENSSSCG00000031572	protein coding
STX2	*HES1*$^+$ Satellite cells	0.633	4.71	0.0307	ENSSSCG00000009747	protein coding
BAG3	*HES1*$^+$ Satellite cells	0.634	7.83	0.0251	ENSSSCG00000010686	protein coding
GEMIN6	*HES1*$^+$ Satellite cells	0.634	4.89	0.0303	ENSSSCG00000008482	protein coding
IFT46	*HES1*$^+$ Satellite cells	0.634	5.57	0.0437	ENSSSCG00000029649	protein coding
PPT1	*HES1*$^+$ Satellite cells	0.636	5.12	0.0266	ENSSSCG00000036673	protein coding
SLC10A7	*HES1*$^+$ Satellite cells	0.639	4.23	0.0289	ENSSSCG00000009035	protein coding
ENSSSCG00000012061	*HES1*$^+$ Satellite cells	0.64	5.38	0.0311	ENSSSCG00000012061	protein coding
PDK1	*HES1*$^+$ Satellite cells	0.64	4.01	0.0362	ENSSSCG00000015958	protein coding
RAP1GDS1	*HES1*$^+$ Satellite cells	0.64	5.44	0.0111	ENSSSCG00000022353	protein coding
GSTA4	*HES1*$^+$ Satellite cells	0.641	5.53	0.0114	ENSSSCG00000002627	protein coding

FBXL3	*HES1*$^{+}$ Satellite cells	0.643	4.7	0.0145	ENSSSCG00000009472	protein coding
EFS	*HES1*$^{+}$ Satellite cells	0.645	4.25	0.0401	ENSSSCG00000002026	protein coding
KLF10	*HES1*$^{+}$ Satellite cells	0.645	8.04	0.013	ENSSSCG00000027480	protein coding
APIP	*HES1*$^{+}$ Satellite cells	0.648	5.41	0.00881	ENSSSCG00000013299	protein coding
ENSSSCG00000009125	*HES1*$^{+}$ Satellite cells	0.648	8.54	0.00999	ENSSSCG00000009125	protein coding
DLGAP4	*HES1*$^{+}$ Satellite cells	0.651	4.07	0.0496	ENSSSCG00000007317	protein coding
ENSSSCG00000000359	*HES1*$^{+}$ Satellite cells	0.651	7.94	0.0127	ENSSSCG00000000359	protein coding
CTNNA3	*HES1*$^{+}$ Satellite cells	0.652	4.32	0.0193	ENSSSCG00000036480	protein coding
YPEL3	*HES1*$^{+}$ Satellite cells	0.652	6.96	0.011	ENSSSCG00000035430	protein coding
SELENOH	*HES1*$^{+}$ Satellite cells	0.654	6.25	0.00704	ENSSSCG00000032364	protein coding
ENSSSCG00000007705	*HES1*$^{+}$ Satellite cells	0.657	4.67	0.0324	ENSSSCG00000007705	protein coding
C16orf87	*HES1*$^{+}$ Satellite cells	0.659	4.4	0.0337	ENSSSCG00000022333	protein coding
IPO5	*HES1*$^{+}$ Satellite cells	0.66	6.46	0.00691	ENSSSCG00000009509	protein coding
MSTRG.49539	*HES1*$^{+}$ Satellite cells	0.66	6.78	0.0362	MSTRG.49539	lncRNA
JAM3	*HES1*$^{+}$ Satellite cells	0.662	6.43	0.0063	ENSSSCG00000015256	protein coding
TAGLN	*HES1*$^{+}$ Satellite cells	0.663	6.58	0.0459	ENSSSCG00000015073	protein coding
TFDP1	*HES1*$^{+}$ Satellite cells	0.664	4.35	0.0377	ENSSSCG00000009560	protein coding
ENSSSCG00000001927	*HES1*$^{+}$ Satellite cells	0.666	5.5	0.00904	ENSSSCG00000001927	protein coding
MSTRG.2535	*HES1*$^{+}$ Satellite cells	0.666	4.59	0.0302	MSTRG.2535	lncRNA
NQO2	*HES1*$^{+}$ Satellite cells	0.666	4.8	0.0222	ENSSSCG00000020758	protein coding
SAMD4A	*HES1*$^{+}$ Satellite cells	0.666	7.05	0.032	ENSSSCG00000023408	protein coding
COL1A2	*HES1*$^{+}$ Satellite cells	0.668	10.1	0.0336	ENSSSCG00000015326	protein coding
ENSSSCG00000002351	*HES1*$^{+}$ Satellite cells	0.668	4.66	0.0201	ENSSSCG00000002351	protein coding
FUOM	*HES1*$^{+}$ Satellite cells	0.674	4.68	0.0202	ENSSSCG00000010791	protein coding
WLS	*HES1*$^{+}$ Satellite cells	0.677	5.56	0.0148	ENSSSCG00000003795	protein coding
CCN1	*HES1*$^{+}$ Satellite cells	0.678	7.97	0.00627	ENSSSCG00000006940	protein coding
PLAAT3	*HES1*$^{+}$ Satellite cells	0.678	4.72	0.03	ENSSSCG00000026914	protein coding
GUSB	*HES1*$^{+}$ Satellite cells	0.679	5.12	0.0309	ENSSSCG00000007739	protein coding
AIMP1	*HES1*$^{+}$ Satellite cells	0.68	7.66	0.00706	ENSSSCG00000024168	protein coding
CCNI	*HES1*$^{+}$ Satellite cells	0.68	7.49	0.0136	ENSSSCG00000008987	protein coding
RHOBTB1	*HES1*$^{+}$ Satellite cells	0.68	4.12	0.0258	ENSSSCG00000028327	protein coding
ACTR10	*HES1*$^{+}$ Satellite cells	0.681	6.5	0.00255	ENSSSCG00000022299	protein coding
MTUS1	*HES1*$^{+}$ Satellite cells	0.682	5.63	0.0224	ENSSSCG00000040013	protein coding
PON2	*HES1*$^{+}$ Satellite cells	0.682	6.41	0.0191	ENSSSCG00000015331	protein coding

BCAP29	*HES1* $^{+}$ Satellite cells	0.683	5.14	0.0109	ENSSSCG00000022869	protein coding
BLOC1S2	*HES1* $^{+}$ Satellite cells	0.687	4.37	0.0138	ENSSSCG00000010551	protein coding
LITAF	*HES1* $^{+}$ Satellite cells	0.687	5.74	0.0244	ENSSSCG00000032381	protein coding
THUMPD2	*HES1* $^{+}$ Satellite cells	0.689	5.3	0.00906	ENSSSCG00000008470	protein coding
ENSSSCG00000005440	*HES1* $^{+}$ Satellite cells	0.693	4.17	0.048	ENSSSCG00000005440	protein coding
UBXN8	*HES1* $^{+}$ Satellite cells	0.694	4.99	0.0231	ENSSSCG00000015843	protein coding
DRAP1	*HES1* $^{+}$ Satellite cells	0.695	6.25	0.0118	ENSSSCG00000012965	protein coding
ENSSSCG00000022925	*HES1* $^{+}$ Satellite cells	0.695	4.12	0.021	ENSSSCG00000022925	protein coding
MSTRG.35782	*HES1* $^{+}$ Satellite cells	0.696	4.99	0.0383	MSTRG.35782	lncRNA
ENSSSCG00000044542	*HES1* $^{+}$ Satellite cells	0.698	4.63	0.0161	ENSSSCG00000044542	protein coding
FAM177A1	*HES1* $^{+}$ Satellite cells	0.704	4.62	0.0161	ENSSSCG00000034753	protein coding
ENSSSCG00000015023	*HES1* $^{+}$ Satellite cells	0.705	4.17	0.029	ENSSSCG00000015023	protein coding
TMEM168	*HES1* $^{+}$ Satellite cells	0.707	4.13	0.0194	ENSSSCG00000016643	protein coding
EZH2	*HES1* $^{+}$ Satellite cells	0.708	5.71	0.00799	ENSSSCG00000028840	protein coding
GALK2	*HES1* $^{+}$ Satellite cells	0.708	5.52	0.0133	ENSSSCG00000004651	protein coding
SAV1	*HES1* $^{+}$ Satellite cells	0.708	4.59	0.026	ENSSSCG00000005021	protein coding
ANXA2	*HES1* $^{+}$ Satellite cells	0.71	8.89	0.00749	ENSSSCG00000004578	protein coding
ID2	*HES1* $^{+}$ Satellite cells	0.711	5.07	0.025	ENSSSCG00000008645	protein coding
MSTRG.161	*HES1* $^{+}$ Satellite cells	0.711	6.88	0.0127	MSTRG.161	lncRNA
MRPL39	*HES1* $^{+}$ Satellite cells	0.714	5.9	0.00396	ENSSSCG00000012021	protein coding
PMAIP1	*HES1* $^{+}$ Satellite cells	0.714	4.78	0.0208	ENSSSCG00000026454	protein coding
NXT2	*HES1* $^{+}$ Satellite cells	0.716	3.88	0.0461	ENSSSCG00000012581	protein coding
TMX1	*HES1* $^{+}$ Satellite cells	0.716	5.67	0.0373	ENSSSCG00000038422	protein coding
ING2	*HES1* $^{+}$ Satellite cells	0.722	5.22	0.0301	ENSSSCG00000024555	protein coding
ENSSSCG00000006395	*HES1* $^{+}$ Satellite cells	0.724	6.87	0.0216	ENSSSCG00000006395	protein coding
FAP	*HES1* $^{+}$ Satellite cells	0.725	5.81	0.00594	ENSSSCG00000015896	protein coding
MSTRG.12119	*HES1* $^{+}$ Satellite cells	0.725	4.26	0.0456	MSTRG.12119	lncRNA
SLC22A17	*HES1* $^{+}$ Satellite cells	0.727	4.37	0.02	ENSSSCG00000002025	protein coding
ENSSSCG00000040056	*HES1* $^{+}$ Satellite cells	0.73	4.13	0.034	ENSSSCG00000040056	protein coding
MSTRG.14929	*HES1* $^{+}$ Satellite cells	0.73	4.61	0.0198	MSTRG.14929	lncRNA
TOLLIP	*HES1* $^{+}$ Satellite cells	0.73	6.05	0.0123	ENSSSCG00000033033	protein coding
ZFP36L1	*HES1* $^{+}$ Satellite cells	0.733	8.41	0.00179	ENSSSCG00000028304	protein coding
S100A11	*HES1* $^{+}$ Satellite cells	0.735	8.5	0.00655	ENSSSCG00000006610	protein coding
ENSSSCG00000016679	*HES1* $^{+}$ Satellite cells	0.736	3.97	0.0287	ENSSSCG00000016679	protein coding

COX7C	*HES1* + Satellite cells	0.737	9.42	0.0125	ENSSSCG00000040325	protein coding
CCDC167	*HES1* + Satellite cells	0.738	4.97	0.0158	ENSSSCG00000036178	protein coding
FGFR1OP2	*HES1* + Satellite cells	0.738	4.19	0.0152	ENSSSCG00000000554	protein coding
MSTRG.7260	*HES1* + Satellite cells	0.739	4.49	0.0442	MSTRG.7260	lncRNA
MSTRG.28741	*HES1* + Satellite cells	0.74	4.19	0.0241	MSTRG.28741	lncRNA
PROS1	*HES1* + Satellite cells	0.74	5.39	0.0291	ENSSSCG00000025463	protein coding
MSTRG.12708	*HES1* + Satellite cells	0.744	4.47	0.0135	MSTRG.12708	lncRNA
MSTRG.22342	*HES1* + Satellite cells	0.744	5.88	0.0405	MSTRG.22342	lncRNA
MTHFD2	*HES1* + Satellite cells	0.744	3.88	0.0468	ENSSSCG00000008289	protein coding
ZNF277	*HES1* + Satellite cells	0.744	6.94	0.00678	ENSSSCG00000025910	protein coding
RBM38	*HES1* + Satellite cells	0.746	4.88	0.0115	ENSSSCG00000007504	protein coding
ENSSSCG00000004307	*HES1* + Satellite cells	0.749	6.48	0.00228	ENSSSCG00000004307	protein coding
ENSSSCG00000014197	*HES1* + Satellite cells	0.749	3.9	0.0312	ENSSSCG00000014197	protein coding
POC1B	*HES1* + Satellite cells	0.751	5.1	0.00502	ENSSSCG00000000920	protein coding
FDFT1	*HES1* + Satellite cells	0.753	5.36	0.00853	ENSSSCG00000026044	protein coding
PDRG1	*HES1* + Satellite cells	0.753	4.42	0.0368	ENSSSCG00000007237	protein coding
CETN3	*HES1* + Satellite cells	0.754	6.44	0.00541	ENSSSCG00000014151	protein coding
FOLR2	*HES1* + Satellite cells	0.76	4.92	0.023	ENSSSCG00000014812	protein coding
MSTRG.38999	*HES1* + Satellite cells	0.76	5.54	0.0173	MSTRG.38999	lncRNA
ENSSSCG00000030585	*HES1* + Satellite cells	0.764	4	0.0371	ENSSSCG00000030585	protein coding
MYF5	*HES1* + Satellite cells	0.765	9.16	0.00359	ENSSSCG00000000937	protein coding
TBCA	*HES1* + Satellite cells	0.766	8.03	0.0146	ENSSSCG00000014100	protein coding
MSTRG.48550	*HES1* + Satellite cells	0.771	3.98	0.0365	MSTRG.48550	lncRNA
TFPI2	*HES1* + Satellite cells	0.772	6.51	0.0205	ENSSSCG00000015322	protein coding
CHURC1	*HES1* + Satellite cells	0.774	4.96	0.0136	ENSSSCG00000022147	protein coding
ENSSSCG00000005135	*HES1* + Satellite cells	0.775	4.34	0.0441	ENSSSCG00000005135	protein coding
MSTRG.17830	*HES1* + Satellite cells	0.775	4.24	0.0372	MSTRG.17830	lncRNA
MSTRG.30533	*HES1* + Satellite cells	0.776	4.18	0.0282	MSTRG.30533	lncRNA
ENSSSCG00000029857	*HES1* + Satellite cells	0.777	7.17	0.00692	ENSSSCG00000029857	protein coding
MSTRG.17788	*HES1* + Satellite cells	0.777	6.56	0.00935	MSTRG.17788	lncRNA
MSTRG.28032	*HES1* + Satellite cells	0.779	3.87	0.0385	MSTRG.28032	lncRNA
NT5C3A	*HES1* + Satellite cells	0.78	4.75	0.0124	ENSSSCG00000022912	protein coding
RPL36A-HNRNPH2	*HES1* + Satellite cells	0.78	10.9	0.0022	ENSSSCG00000039046	protein coding
LBR	*HES1* + Satellite cells	0.781	4.35	0.0198	ENSSSCG00000032397	protein coding

ENSSSCG00000032235	*HES1*$^{+}$ Satellite cells	0.782	5.85	0.0131	ENSSSCG00000032235	protein coding
DEPP1	*HES1*$^{+}$ Satellite cells	0.79	4.59	0.0462	ENSSSCG00000022256	protein coding
MSTRG.33764	*HES1*$^{+}$ Satellite cells	0.79	4.93	0.0152	MSTRG.33764	lncRNA
PDPN	*HES1*$^{+}$ Satellite cells	0.791	5.53	0.0112	ENSSSCG00000003451	protein coding
MSTRG.43169	*HES1*$^{+}$ Satellite cells	0.792	5.72	0.00366	MSTRG.43169	lncRNA
FADS3	*HES1*$^{+}$ Satellite cells	0.793	4.46	0.0491	ENSSSCG00000013073	protein coding
MSTRG.39337	*HES1*$^{+}$ Satellite cells	0.793	4.97	0.0259	MSTRG.39337	lncRNA
ACAT2	*HES1*$^{+}$ Satellite cells	0.795	4.95	0.00815	ENSSSCG00000004049	protein coding
ZNF672	*HES1*$^{+}$ Satellite cells	0.796	4.38	0.0372	ENSSSCG00000013978	protein coding
ACSS2	*HES1*$^{+}$ Satellite cells	0.797	4.51	0.0378	ENSSSCG00000007286	protein coding
RNF2	*HES1*$^{+}$ Satellite cells	0.797	5.31	0.00483	ENSSSCG00000015568	protein coding
MSTRG.5629	*HES1*$^{+}$ Satellite cells	0.799	4.87	0.00472	MSTRG.5629	lncRNA
TXNDC17	*HES1*$^{+}$ Satellite cells	0.799	6.51	0.00363	ENSSSCG00000035820	protein coding
ENSSSCG00000004324	*HES1*$^{+}$ Satellite cells	0.8	4.92	0.00389	ENSSSCG00000004324	lncRNA
LGALS1	*HES1*$^{+}$ Satellite cells	0.8	11.3	0.0233	ENSSSCG00000033854	protein coding
ARPC1B	*HES1*$^{+}$ Satellite cells	0.801	4.74	0.00744	ENSSSCG00000007625	protein coding
CDC42EP5	*HES1*$^{+}$ Satellite cells	0.803	7.03	0.0342	ENSSSCG00000039922	protein coding
MSTRG.17842	*HES1*$^{+}$ Satellite cells	0.805	4.62	0.0227	MSTRG.17842	lncRNA
ACSL3	*HES1*$^{+}$ Satellite cells	0.806	5.04	0.0199	ENSSSCG00000016223	protein coding
VGLL2	*HES1*$^{+}$ Satellite cells	0.806	4.82	0.0314	ENSSSCG00000004255	protein coding
TWF1	*HES1*$^{+}$ Satellite cells	0.813	4.29	0.0125	ENSSSCG00000000798	protein coding
MSTRG.3891	*HES1*$^{+}$ Satellite cells	0.815	4.17	0.0117	MSTRG.3891	lncRNA
DPP4	*HES1*$^{+}$ Satellite cells	0.817	5.92	0.00986	ENSSSCG00000015894	protein coding
NUP54	*HES1*$^{+}$ Satellite cells	0.817	5.01	0.0071	ENSSSCG00000008979	protein coding
MRPS9	*HES1*$^{+}$ Satellite cells	0.818	5.04	0.0134	ENSSSCG00000028020	protein coding
GMFB	*HES1*$^{+}$ Satellite cells	0.824	4.48	0.0164	ENSSSCG00000026294	protein coding
MSTRG.6594	*HES1*$^{+}$ Satellite cells	0.825	7.78	0.0184	MSTRG.6594	lncRNA
PLP2	*HES1*$^{+}$ Satellite cells	0.825	6.08	0.0129	ENSSSCG00000012294	protein coding
MSTRG.36649	*HES1*$^{+}$ Satellite cells	0.826	4.04	0.0422	MSTRG.36649	lncRNA
GPX1	*HES1*$^{+}$ Satellite cells	0.831	8.12	0.0121	ENSSSCG00000033727	protein coding
CCNG2	*HES1*$^{+}$ Satellite cells	0.832	5.72	0.0161	ENSSSCG00000008988	protein coding
TMEM59	*HES1*$^{+}$ Satellite cells	0.832	7.99	0.00272	ENSSSCG00000034261	protein coding
EIF2A	*HES1*$^{+}$ Satellite cells	0.834	6.28	0.00963	ENSSSCG00000011710	protein coding
ATG12	*HES1*$^{+}$ Satellite cells	0.835	5.15	0.00196	ENSSSCG00000033912	protein coding

BTG1	*HES1*$^{+}$ Satellite cells	0.836	9.58	0.0193	ENSSSCG00000035790	protein coding
TRIM13	*HES1*$^{+}$ Satellite cells	0.837	4.29	0.00622	ENSSSCG00000009389	protein coding
MSTRG.36323	*HES1*$^{+}$ Satellite cells	0.838	5.59	0.00808	MSTRG.36323	lncRNA
SNRPB2	*HES1*$^{+}$ Satellite cells	0.839	6.66	0.002	ENSSSCG00000037602	protein coding
PIM3	*HES1*$^{+}$ Satellite cells	0.84	5.06	0.0344	ENSSSCG00000032936	protein coding
SELENBP1	*HES1*$^{+}$ Satellite cells	0.841	6.03	0.0115	ENSSSCG00000006624	protein coding
MSTRG.25390	*HES1*$^{+}$ Satellite cells	0.842	4.4	0.00649	MSTRG.25390	lncRNA
ENSSSCG00000030849	*HES1*$^{+}$ Satellite cells	0.843	11.8	0.00116	ENSSSCG00000030849	protein coding
TWSG1	*HES1*$^{+}$ Satellite cells	0.846	6.08	0.0102	ENSSSCG00000039663	protein coding
CHCHD5	*HES1*$^{+}$ Satellite cells	0.85	5.19	0.00246	ENSSSCG00000008094	protein coding
STMN1	*HES1*$^{+}$ Satellite cells	0.85	6.68	0.0122	ENSSSCG00000026257	protein coding
CBX3	*HES1*$^{+}$ Satellite cells	0.852	6.77	0.000934	ENSSSCG00000016711	protein coding
ENSSSCG00000032768	*HES1*$^{+}$ Satellite cells	0.857	4.5	0.0287	ENSSSCG00000032768	protein coding
KIF20B	*HES1*$^{+}$ Satellite cells	0.86	4	0.00796	ENSSSCG00000010457	protein coding
MSTRG.17417	*HES1*$^{+}$ Satellite cells	0.866	3.89	0.0118	MSTRG.17417	lncRNA
S100A6	*HES1*$^{+}$ Satellite cells	0.866	6.57	0.0368	ENSSSCG00000021084	protein coding
ISOC1	*HES1*$^{+}$ Satellite cells	0.867	5.25	0.0297	ENSSSCG00000014258	protein coding
GLMP	*HES1*$^{+}$ Satellite cells	0.868	4.47	0.021	ENSSSCG00000006488	protein coding
SCLY	*HES1*$^{+}$ Satellite cells	0.874	5.08	0.0237	ENSSSCG00000016334	protein coding
CYP39A1	*HES1*$^{+}$ Satellite cells	0.875	4.53	0.0152	ENSSSCG00000001718	protein coding
IDI1	*HES1*$^{+}$ Satellite cells	0.876	5.18	0.0286	ENSSSCG00000029066	protein coding
ST8SIA4	*HES1*$^{+}$ Satellite cells	0.878	4.24	0.0087	ENSSSCG00000035539	protein coding
FAM227B	*HES1*$^{+}$ Satellite cells	0.88	4.72	0.0225	ENSSSCG00000004648	protein coding
ARMCX2	*HES1*$^{+}$ Satellite cells	0.887	4.31	0.022	ENSSSCG00000012510	protein coding
TPD52	*HES1*$^{+}$ Satellite cells	0.887	6.81	0.00176	ENSSSCG00000006156	protein coding
MSTRG.25438	*HES1*$^{+}$ Satellite cells	0.892	4.69	0.0311	MSTRG.25438	lncRNA
ATP6V1D	*HES1*$^{+}$ Satellite cells	0.902	4.24	0.00566	ENSSSCG00000033278	protein coding
ENSSSCG00000050347	*HES1*$^{+}$ Satellite cells	0.902	3.86	0.0157	ENSSSCG00000050347	lncRNA
SELENOM	*HES1*$^{+}$ Satellite cells	0.903	5.5	0.042	ENSSSCG00000029781	protein coding
ADD3	*HES1*$^{+}$ Satellite cells	0.905	4.71	0.0074	ENSSSCG00000010621	protein coding
MSTRG.11643	*HES1*$^{+}$ Satellite cells	0.912	4.83	0.00676	MSTRG.11643	lncRNA
RNFT1	*HES1*$^{+}$ Satellite cells	0.912	5.07	0.0092	ENSSSCG00000017671	protein coding
ABHD10	*HES1*$^{+}$ Satellite cells	0.919	3.97	0.0335	ENSSSCG00000034450	protein coding
MSTRG.41885	*HES1*$^{+}$ Satellite cells	0.921	3.9	0.0432	MSTRG.41885	lncRNA

PRCP	*HES1*$^{+}$ Satellite cells	0.921	3.91	0.00884	ENSSSCG00000014899	protein coding
TCP11L1	*HES1*$^{+}$ Satellite cells	0.927	4.23	0.012	ENSSSCG00000030326	protein coding
ASAH1	*HES1*$^{+}$ Satellite cells	0.929	4.47	0.0223	ENSSSCG00000006995	protein coding
HSPH1	*HES1*$^{+}$ Satellite cells	0.932	8.14	0.0187	ENSSSCG00000009334	protein coding
MSTRG.11148	*HES1*$^{+}$ Satellite cells	0.935	5.51	0.0153	MSTRG.11148	lncRNA
PHLDA2	*HES1*$^{+}$ Satellite cells	0.937	5.62	0.0097	ENSSSCG00000021597	protein coding
YPEL5	*HES1*$^{+}$ Satellite cells	0.94	5.42	0.000751	ENSSSCG00000008529	protein coding
SRPX2	*HES1*$^{+}$ Satellite cells	0.945	3.82	0.0092	ENSSSCG00000012482	protein coding
TUBB6	*HES1*$^{+}$ Satellite cells	0.95	4.45	0.0118	ENSSSCG00000038992	protein coding
ENSSSCG00000016823	*HES1*$^{+}$ Satellite cells	0.961	8.28	0.00612	ENSSSCG00000016823	protein coding
TENT5C	*HES1*$^{+}$ Satellite cells	0.967	5.13	0.0405	ENSSSCG00000006729	protein coding
MSTRG.23990	*HES1*$^{+}$ Satellite cells	0.985	4.07	0.03	MSTRG.23990	lncRNA
GPR176	*HES1*$^{+}$ Satellite cells	0.986	4.76	0.0159	ENSSSCG00000004787	protein coding
SLC38A5	*HES1*$^{+}$ Satellite cells	0.99	4.41	0.00422	ENSSSCG00000025981	protein coding
AP1S2	*HES1*$^{+}$ Satellite cells	0.993	5.74	0.00644	ENSSSCG00000036074	protein coding
HOXA10	*HES1*$^{+}$ Satellite cells	1	4.39	0.0106	ENSSSCG00000021204	protein coding
NRAS	*HES1*$^{+}$ Satellite cells	1	4.8	0.00231	ENSSSCG00000032477	protein coding
CCNG1	*HES1*$^{+}$ Satellite cells	1.01	7.5	0.000434	ENSSSCG00000017024	protein coding
MSTRG.8502	*HES1*$^{+}$ Satellite cells	1.01	4.21	0.0112	MSTRG.8502	lncRNA
NRXN1	*HES1*$^{+}$ Satellite cells	1.01	4.03	0.0449	ENSSSCG00000026180	protein coding
SCN7A	*HES1*$^{+}$ Satellite cells	1.01	5.38	0.00181	ENSSSCG00000015914	protein coding
SLC25A40	*HES1*$^{+}$ Satellite cells	1.01	3.85	0.00735	ENSSSCG00000026177	protein coding
TUFT1	*HES1*$^{+}$ Satellite cells	1.01	4.99	0.0152	ENSSSCG00000006620	protein coding
BTBD3	*HES1*$^{+}$ Satellite cells	1.02	3.92	0.00242	ENSSSCG00000020853	protein coding
CFL2	*HES1*$^{+}$ Satellite cells	1.02	7.45	0.000303	ENSSSCG00000001959	protein coding
MSTRG.32310	*HES1*$^{+}$ Satellite cells	1.02	5.11	0.0319	MSTRG.32310	lncRNA
MSTRG.44098	*HES1*$^{+}$ Satellite cells	1.02	4.41	0.028	MSTRG.44098	lncRNA
MSTRG.45936	*HES1*$^{+}$ Satellite cells	1.02	3.9	0.0289	MSTRG.45936	lncRNA
PLCB1	*HES1*$^{+}$ Satellite cells	1.02	4.72	0.0473	ENSSSCG00000007056	protein coding
ITGA2	*HES1*$^{+}$ Satellite cells	1.03	4.04	0.00463	ENSSSCG00000016887	protein coding
MSTRG.28657	*HES1*$^{+}$ Satellite cells	1.03	4.4	0.00497	MSTRG.28657	lncRNA
IMMP2L	*HES1*$^{+}$ Satellite cells	1.04	7.58	0.00696	ENSSSCG00000016650	protein coding
MFGE8	*HES1*$^{+}$ Satellite cells	1.04	4.59	0.0162	ENSSSCG00000001834	protein coding
SLC43A3	*HES1*$^{+}$ Satellite cells	1.04	5.13	0.0135	ENSSSCG00000033998	protein coding

TMEM109	*HES1*$^{+}$ Satellite cells	1.04	5.05	0.0275	ENSSSCG00000013110	protein coding
HACE1	*HES1*$^{+}$ Satellite cells	1.05	4.43	0.00361	ENSSSCG00000004364	protein coding
LARP6	*HES1*$^{+}$ Satellite cells	1.05	4.76	0.00384	ENSSSCG00000004974	protein coding
TIMP1	*HES1*$^{+}$ Satellite cells	1.05	7.02	0.000453	ENSSSCG00000012277	protein coding
LRRTM3	*HES1*$^{+}$ Satellite cells	1.06	5.4	0.019	ENSSSCG00000010232	protein coding
MSTRG.27683	*HES1*$^{+}$ Satellite cells	1.07	4.96	0.000595	MSTRG.27683	lncRNA
NR4A1	*HES1*$^{+}$ Satellite cells	1.07	5.93	0.000727	ENSSSCG00000031321	protein coding
TMSB10	*HES1*$^{+}$ Satellite cells	1.07	9.53	0.00401	ENSSSCG00000008245	protein coding
MAT2B	*HES1*$^{+}$ Satellite cells	1.08	4.98	0.00268	ENSSSCG00000021229	protein coding
MGLL	*HES1*$^{+}$ Satellite cells	1.08	3.85	0.00604	ENSSSCG00000024134	protein coding
MSTRG.11887	*HES1*$^{+}$ Satellite cells	1.08	5.32	0.00984	MSTRG.11887	lncRNA
MSTRG.17090	*HES1*$^{+}$ Satellite cells	1.08	4.42	0.0125	MSTRG.17090	lncRNA
MSTRG.39844	*HES1*$^{+}$ Satellite cells	1.08	3.9	0.00707	MSTRG.39844	lncRNA
MSTRG.18663	*HES1*$^{+}$ Satellite cells	1.09	4.83	0.00599	MSTRG.18663	lncRNA
MSTRG.37107	*HES1*$^{+}$ Satellite cells	1.09	4.22	0.00188	MSTRG.37107	lncRNA
MSTRG.9045	*HES1*$^{+}$ Satellite cells	1.09	3.88	0.00523	MSTRG.9045	lncRNA
MSTRG.26567	*HES1*$^{+}$ Satellite cells	1.12	3.89	0.00328	MSTRG.26567	lncRNA
DAB2	*HES1*$^{+}$ Satellite cells	1.15	4.22	0.0125	ENSSSCG00000016857	protein coding
C15orf40	*HES1*$^{+}$ Satellite cells	1.16	5.84	0.00023	ENSSSCG00000030131	protein coding
MSTRG.3582	*HES1*$^{+}$ Satellite cells	1.17	3.97	0.00104	MSTRG.3582	lncRNA
XRCC6	*HES1*$^{+}$ Satellite cells	1.2	4.69	0.000496	ENSSSCG00000000059	protein coding
MSTRG.27392	*HES1*$^{+}$ Satellite cells	1.21	4.54	0.0112	MSTRG.27392	lncRNA
MSTRG.8677	*HES1*$^{+}$ Satellite cells	1.23	4.79	0.0139	MSTRG.8677	lncRNA
DACH1	*HES1*$^{+}$ Satellite cells	1.26	4.92	0.0292	ENSSSCG00000009457	protein coding
DNAJB1	*HES1*$^{+}$ Satellite cells	1.27	9.79	0.00306	ENSSSCG00000013784	protein coding
INSIG1	*HES1*$^{+}$ Satellite cells	1.27	3.81	0.00801	ENSSSCG00000016420	protein coding
SELENOW	*HES1*$^{+}$ Satellite cells	1.29	6.57	0.000147	ENSSSCG00000034313	protein coding
CTHRC1	*HES1*$^{+}$ Satellite cells	1.3	3.75	0.000934	ENSSSCG00000006051	protein coding
ENSSSCG00000031288	*HES1*$^{+}$ Satellite cells	1.3	3.94	0.00274	ENSSSCG00000031288	protein coding
MSTRG.4983	*HES1*$^{+}$ Satellite cells	1.3	4.46	0.000458	MSTRG.4983	lncRNA
MSTRG.7224	*HES1*$^{+}$ Satellite cells	1.3	4.61	0.0026	MSTRG.7224	lncRNA
ENSSSCG00000029160	*HES1*$^{+}$ Satellite cells	1.31	11.5	0.00756	ENSSSCG00000029160	protein coding
MSTRG.9241	*HES1*$^{+}$ Satellite cells	1.33	5.74	0.0221	MSTRG.9241	lncRNA
MSTRG.17815	*HES1*$^{+}$ Satellite cells	1.34	5.32	0.0211	MSTRG.17815	lncRNA

MSTRG.26379	*HES1*$^+$ Satellite cells	1.35	3.99	0.00609	MSTRG.26379	lncRNA
MSTRG.3143	*HES1*$^+$ Satellite cells	1.36	4.49	0.00439	MSTRG.3143	lncRNA
CKS2	*HES1*$^+$ Satellite cells	1.37	4.47	0.00142	ENSSSCG00000021161	protein coding
ENSSSCG00000045666	*HES1*$^+$ Satellite cells	1.37	4.97	0.00313	ENSSSCG00000045666	lncRNA
MSTRG.13235	*HES1*$^+$ Satellite cells	1.39	4.98	0.0261	MSTRG.13235	lncRNA
ENSSSCG00000028695	*HES1*$^+$ Satellite cells	1.4	5.25	0.0033	ENSSSCG00000028695	lncRNA
SELENOP	*HES1*$^+$ Satellite cells	1.43	8.56	3.64E-05	ENSSSCG00000021208	protein coding
MSTRG.3599	*HES1*$^+$ Satellite cells	1.44	4.94	0.00591	MSTRG.3599	lncRNA
HSPA6	*HES1*$^+$ Satellite cells	1.45	6.11	0.0161	ENSSSCG00000047932	protein coding
MSTRG.27574	*HES1*$^+$ Satellite cells	1.45	4.29	0.000226	MSTRG.27574	lncRNA
APOD	*HES1*$^+$ Satellite cells	1.48	5	0.0267	ENSSSCG00000011831	protein coding
BGN	*HES1*$^+$ Satellite cells	1.5	4.68	0.0425	ENSSSCG00000012766	protein coding
MSTRG.16544	*HES1*$^+$ Satellite cells	1.51	3.92	0.00963	MSTRG.16544	lncRNA
MSTRG.8600	*HES1*$^+$ Satellite cells	1.51	5.36	0.0456	MSTRG.8600	lncRNA
NUDT15	*HES1*$^+$ Satellite cells	1.51	3.73	0.00102	ENSSSCG00000027551	protein coding
MSTRG.32351	*HES1*$^+$ Satellite cells	1.55	5.29	0.00537	MSTRG.32351	lncRNA
CXCL2	*HES1*$^+$ Satellite cells	1.58	5.73	0.000568	ENSSSCG00000008959	protein coding
NFE2L3	*HES1*$^+$ Satellite cells	1.59	3.6	0.00186	ENSSSCG00000026894	protein coding
MSTRG.43168	*HES1*$^+$ Satellite cells	1.65	3.91	0.000691	MSTRG.43168	lncRNA
SLC10A1	*HES1*$^+$ Satellite cells	1.67	6.05	0.00328	ENSSSCG00000002313	protein coding
ZNF385B	*HES1*$^+$ Satellite cells	1.71	4.84	0.00272	ENSSSCG00000023181	protein coding
NUPR1	*HES1*$^+$ Satellite cells	1.74	4.85	0.00508	ENSSSCG00000040162	protein coding
MSTRG.39989	*HES1*$^+$ Satellite cells	1.77	4.83	0.0196	MSTRG.39989	lncRNA
PHGDH	*HES1*$^+$ Satellite cells	1.84	5	0.0233	ENSSSCG00000006717	protein coding
CD36	*HES1*$^+$ Satellite cells	1.89	3.71	0.00138	ENSSSCG00000015405	protein coding
MSTRG.38467	*HES1*$^+$ Satellite cells	1.93	8.57	0.0256	MSTRG.38467	lncRNA
MSTRG.882	*HES1*$^+$ Satellite cells	2	4.52	0.0283	MSTRG.882	lncRNA
VCAM1	*HES1*$^+$ Satellite cells	2.06	3.42	0.00135	ENSSSCG00000006862	protein coding
ENSSSCG00000042577	*HES1*$^+$ Satellite cells	2.21	4.03	0.000238	ENSSSCG00000042577	protein coding
MSTRG.23839	*HES1*$^+$ Satellite cells	2.21	3.96	0.00119	MSTRG.23839	lncRNA
MSTRG.8106	*HES1*$^+$ Satellite cells	2.23	3.99	6.84E-05	MSTRG.8106	lncRNA
ENSSSCG00000037324	*HES1*$^+$ Satellite cells	2.3	3.56	0.00233	ENSSSCG00000037324	lncRNA
MSTRG.19885	*HES1*$^+$ Satellite cells	2.94	3.88	0.00211	MSTRG.19885	lncRNA
PSAT1	*HES1*$^+$ Satellite cells	3.16	5.47	0.00145	ENSSSCG00000005287	protein coding

ENSSSCG00000029003	*HES1*$^{+}$ Satellite cells	3.57	4.24	0.00955	ENSSSCG00000029003	protein coding
MSTRG.29989	*HES1*$^{+}$ Satellite cells	3.84	5.91	0.0131	MSTRG.29989	lncRNA
MSTRG.38853	*HES1*$^{+}$ Satellite cells	3.88	8.4	0.0122	MSTRG.38853	lncRNA
MSTRG.43887	*HES1*$^{+}$ Satellite cells	4.07	3.44	6.93E-06	MSTRG.43887	lncRNA
MSTRG.11119	*HES1*$^{+}$ Satellite cells	4.1	5.3	0.00278	MSTRG.11119	lncRNA
CCDC43	I myofibers	-2.4	4.97	0.0071	ENSSSCG00000017352	protein coding
ENSSSCG00000002953	I myofibers	-2.17	4.96	0.0117	ENSSSCG00000002953	protein coding
SLC25A25	I myofibers	-1.86	5.19	0.0161	ENSSSCG00000005636	protein coding
MSTRG.45632	I myofibers	-1.81	4.85	0.0285	MSTRG.45632	lncRNA
PDK4	I myofibers	-1.45	9.3	0.00285	ENSSSCG00000015334	protein coding
RPF1	I myofibers	-1.44	5.22	0.0165	ENSSSCG00000022145	protein coding
TAF11	I myofibers	-1.4	5.3	0.0278	ENSSSCG00000001533	protein coding
MSTRG.21028	I myofibers	-1.38	6.46	0.00533	MSTRG.21028	lncRNA
DRG2	I myofibers	-1.36	4.77	0.0282	ENSSSCG00000036328	protein coding
SORD	I myofibers	-1.35	5.81	0.00546	ENSSSCG00000004679	protein coding
NRAP	I myofibers	-1.29	6.63	0.00292	ENSSSCG00000010640	protein coding
TXNIP	I myofibers	-1.25	8.63	0.00169	ENSSSCG00000031262	protein coding
NSFL1C	I myofibers	-1.23	5.37	0.0261	ENSSSCG00000007187	protein coding
IP6K3	I myofibers	-1.22	5.65	0.0244	ENSSSCG00000039272	protein coding
TRIM63	I myofibers	-1.22	6.54	0.00718	ENSSSCG00000027684	protein coding
ACO2	I myofibers	-1.19	9.22	0.00947	ENSSSCG00000000064	protein coding
CMYA5	I myofibers	-1.19	7.49	0.00418	ENSSSCG00000026098	protein coding
PDHX	I myofibers	-1.16	5.77	0.0321	ENSSSCG00000013298	protein coding
PLGRKT	I myofibers	-1.15	5.93	0.023	ENSSSCG00000005212	protein coding
NEB	I myofibers	-1.12	8.35	0.00287	ENSSSCG00000016397	protein coding
USP13	I myofibers	-1.11	5.99	0.0309	ENSSSCG00000011765	protein coding
SYNGR1	I myofibers	-1.09	5.97	0.0137	ENSSSCG00000020873	protein coding
MSTRG.18287	I myofibers	-1.08	5.63	0.0259	MSTRG.18287	lncRNA
HADHB	I myofibers	-1.06	7.72	0.0344	ENSSSCG00000008569	protein coding
FILIP1	I myofibers	-1.03	5.2	0.0473	ENSSSCG00000004479	protein coding
PPP5C	I myofibers	-1.03	6.04	0.0236	ENSSSCG00000003098	protein coding
ENSSSCG00000020686	I myofibers	-0.989	7.44	0.0135	ENSSSCG00000020686	protein coding
BNIP3	I myofibers	-0.985	7.44	0.00318	ENSSSCG00000024388	protein coding
ENSSSCG00000002351	I myofibers	-0.944	7.15	0.00462	ENSSSCG00000002351	protein coding
ARPP21	I myofibers	-0.941	6.32	0.0488	ENSSSCG00000011238	protein coding
ATP2A1	I myofibers	-0.923	7.56	0.024	ENSSSCG00000007805	protein coding
PFKM	I myofibers	-0.913	7.8	0.0246	ENSSSCG00000021129	protein coding
C16orf91	I myofibers	-0.896	5.82	0.0312	ENSSSCG00000038882	protein coding

ALAS1	I myofibers	-0.895	6.01	0.0313	ENSSSCG00000011437	protein coding
DUSP23	I myofibers	-0.888	6.46	0.0412	ENSSSCG00000033585	protein coding
PITPNA	I myofibers	-0.864	5.53	0.0481	ENSSSCG00000025248	protein coding
ENSSSCG00000037251	I myofibers	-0.836	7.06	0.0145	ENSSSCG00000037251	protein coding
OLA1	I myofibers	-0.834	6.4	0.0468	ENSSSCG00000015963	protein coding
MRPS14	I myofibers	-0.827	5.58	0.0492	ENSSSCG00000031435	protein coding
CFAP298-TCP10L	I myofibers	-0.824	5.82	0.0402	ENSSSCG00000040827	protein coding
NDUFS2	I myofibers	-0.772	7.69	0.0208	ENSSSCG00000006358	protein coding
MRPL51	I myofibers	-0.75	7.35	0.0423	ENSSSCG00000039662	protein coding
NAA20	I myofibers	-0.723	8.01	0.0211	ENSSSCG00000028132	protein coding
ENSSSCG00000033700	I myofibers	-0.713	6.95	0.0436	ENSSSCG00000033700	protein coding
IDH3A	I myofibers	-0.698	8.13	0.0415	ENSSSCG00000032498	protein coding
NDUFA1	I myofibers	-0.698	7.92	0.0168	ENSSSCG00000023277	protein coding
EIF3G	I myofibers	-0.688	8.28	0.0302	ENSSSCG00000013661	protein coding
ENSSSCG00000012046	I myofibers	-0.685	7.01	0.0497	ENSSSCG00000012046	protein coding
TPT1	I myofibers	-0.672	12.8	0.0234	ENSSSCG00000040929	protein coding
ENSSSCG00000027746	I myofibers	-0.667	8.56	0.0226	ENSSSCG00000027746	protein coding
ENSSSCG00000012961	I myofibers	-0.633	8.46	0.0302	ENSSSCG00000012961	protein coding
GLRX3	I myofibers	-0.596	7.45	0.0297	ENSSSCG00000010759	protein coding
YWHAH	I myofibers	-0.589	7.21	0.0419	ENSSSCG00000010035	protein coding
ATP5F1A	I myofibers	-0.549	10.3	0.0332	ENSSSCG00000004499	protein coding
TCAP	I myofibers	-0.537	12.9	0.0383	ENSSSCG00000017500	protein coding
RPS12	I myofibers	0.449	12.9	0.0483	ENSSSCG00000004177	protein coding
ENSSSCG00000031838	I myofibers	0.489	11.3	0.0381	ENSSSCG00000031838	protein coding
RPL36A-HNRNPH2	I myofibers	0.617	10.8	0.013	ENSSSCG00000039046	protein coding
PKIA	I myofibers	0.628	8.5	0.0421	ENSSSCG00000006163	protein coding
MAP2K2	I myofibers	0.636	6.9	0.0276	ENSSSCG00000027127	protein coding
CFL2	I myofibers	0.646	10.2	0.0135	ENSSSCG00000001959	protein coding
MSRB1	I myofibers	0.656	7.4	0.038	ENSSSCG00000032932	protein coding
MYO5A	I myofibers	0.668	7.01	0.0274	ENSSSCG00000004620	protein coding
CCNG1	I myofibers	0.676	9.71	0.043	ENSSSCG00000017024	protein coding
SOD2	I myofibers	0.678	7.91	0.0333	ENSSSCG00000040317	protein coding
CD79A	I myofibers	0.681	7.33	0.0446	ENSSSCG00000033684	protein coding
ENSSSCG00000030849	I myofibers	0.681	12.1	0.00826	ENSSSCG00000030849	protein coding
ATF4	I myofibers	0.703	8.66	0.0286	ENSSSCG00000000084	protein coding
MORF4L2	I myofibers	0.71	7.06	0.0426	ENSSSCG00000012534	protein coding
DNAJB4	I myofibers	0.714	6.66	0.0279	ENSSSCG00000003766	protein coding
PPP1CB	I myofibers	0.724	7.43	0.0169	ENSSSCG00000008540	protein coding

NAA50	I myofibers	0.745	6.46	0.0253	ENSSSCG00000011917	protein coding
ENSSSCG00000033744	I myofibers	0.751	6.59	0.0421	ENSSSCG00000033744	protein coding
ROMO1	I myofibers	0.767	7.51	0.0299	ENSSSCG00000007308	protein coding
FHL3	I myofibers	0.781	8.18	0.00766	ENSSSCG00000003644	protein coding
ITM2B	I myofibers	0.783	7.54	0.0401	ENSSSCG00000009403	protein coding
SCOC	I myofibers	0.786	5.65	0.0439	ENSSSCG00000038550	protein coding
DMAC2	I myofibers	0.793	6.35	0.0329	ENSSSCG00000032711	protein coding
MSTRG.49539	I myofibers	0.814	6.12	0.0324	MSTRG.49539	lncRNA
MCU	I myofibers	0.819	6.72	0.0124	ENSSSCG00000010290	protein coding
ARF6	I myofibers	0.826	6.23	0.036	ENSSSCG00000005012	protein coding
PTP4A2	I myofibers	0.826	7.78	0.044	ENSSSCG00000039885	protein coding
TRMT112	I myofibers	0.85	6.94	0.0135	ENSSSCG00000013029	protein coding
ENSSSCG00000039249	I myofibers	0.858	6.38	0.0489	ENSSSCG00000039249	protein coding
CD36	I myofibers	0.863	6.38	0.0191	ENSSSCG00000015405	protein coding
IMP3	I myofibers	0.867	5.47	0.0434	ENSSSCG00000042572	protein coding
CFL1	I myofibers	0.871	6.67	0.0146	ENSSSCG00000012974	protein coding
COPS2	I myofibers	0.883	6.14	0.0158	ENSSSCG00000004652	protein coding
ENSSSCG00000026981	I myofibers	0.928	6.42	0.0329	ENSSSCG00000026981	protein coding
SPARC	I myofibers	0.941	7.52	0.0273	ENSSSCG00000017082	protein coding
RNF157	I myofibers	0.981	5.17	0.0366	ENSSSCG00000017186	protein coding
RAB6A	I myofibers	1.01	5.02	0.0394	ENSSSCG00000014828	protein coding
MSTRG.1890	I myofibers	1.02	4.93	0.0385	MSTRG.1890	lncRNA
FYTTD1	I myofibers	1.04	5.04	0.0352	ENSSSCG00000027139	protein coding
HOXD8	I myofibers	1.07	5.86	0.0445	ENSSSCG00000040349	protein coding
AMD1	I myofibers	1.08	5.32	0.0344	ENSSSCG00000004392	protein coding
SYPL1	I myofibers	1.09	5.24	0.026	ENSSSCG00000015434	protein coding
SELENOH	I myofibers	1.12	5.96	0.00588	ENSSSCG00000032364	protein coding
UBALD1	I myofibers	1.13	5.84	0.0202	ENSSSCG00000023603	protein coding
VAPA	I myofibers	1.14	7.92	0.00499	ENSSSCG00000038465	protein coding
FOXN3	I myofibers	1.15	4.74	0.0401	ENSSSCG00000002429	protein coding
MSTRG.38012	I myofibers	1.15	6.61	0.0454	MSTRG.38012	lncRNA
ENSSSCG00000038359	I myofibers	1.16	6.76	0.00588	ENSSSCG00000038359	protein coding
LRRC39	I myofibers	1.18	9.47	0.000487	ENSSSCG00000006867	protein coding
PPIB	I myofibers	1.26	5.25	0.0229	ENSSSCG00000034137	protein coding
ENSSSCG00000026064	I myofibers	1.27	5.27	0.0157	ENSSSCG00000026064	protein coding
MSTRG.1135	I myofibers	1.27	6.06	0.0439	MSTRG.1135	lncRNA
ZFP36L2	I myofibers	1.27	5.18	0.015	ENSSSCG00000038549	protein coding
MSTRG.24239	I myofibers	1.31	5.42	0.0296	MSTRG.24239	lncRNA

RPL22L1	I myofibers	1.33	7.2	0.0204	ENSSSCG00000036114	protein coding
ENSSSCG00000012119	I myofibers	1.35	7.54	0.0219	ENSSSCG00000012119	protein coding
TRAM1	I myofibers	1.35	5	0.048	ENSSSCG00000006193	protein coding
SARS1	I myofibers	1.36	6.48	0.0276	ENSSSCG00000006835	protein coding
S100A11	I myofibers	1.37	5.8	0.00515	ENSSSCG00000006610	protein coding
ENSSSCG00000039894	I myofibers	1.38	5.26	0.025	ENSSSCG00000039894	protein coding
MAP2K1	I myofibers	1.38	5.6	0.0196	ENSSSCG00000004943	protein coding
SLC25A33	I myofibers	1.38	5.14	0.0266	ENSSSCG00000038006	protein coding
BTG1	I myofibers	1.41	6.19	0.000985	ENSSSCG00000035790	protein coding
CST3	I myofibers	1.41	5.87	0.0129	ENSSSCG00000037360	protein coding
S100A10	I myofibers	1.41	6.1	0.00301	ENSSSCG00000006612	protein coding
CRK	I myofibers	1.44	8.11	0.00259	ENSSSCG00000038989	protein coding
RNF11	I myofibers	1.45	5.82	0.00394	ENSSSCG00000031172	protein coding
VIM	I myofibers	1.45	6.24	0.00698	ENSSSCG00000011033	protein coding
CRYZ	I myofibers	1.46	4.93	0.0157	ENSSSCG00000003780	protein coding
RORA	I myofibers	1.49	4.75	0.0235	ENSSSCG00000004576	protein coding
MSTRG.48395	I myofibers	1.57	5.07	0.0283	MSTRG.48395	lncRNA
MSH4	I myofibers	1.59	6.14	0.0133	ENSSSCG00000003775	protein coding
ENSSSCG00000044700	I myofibers	1.61	5.49	0.0276	ENSSSCG00000044700	protein coding
GADD45B	I myofibers	1.62	5.15	0.00232	ENSSSCG00000022689	protein coding
COL1A2	I myofibers	1.63	8.34	0.023	ENSSSCG00000015326	protein coding
ENSSSCG00000029160	I myofibers	1.63	7.7	0.00066	ENSSSCG00000029160	protein coding
MSTRG.43875	I myofibers	1.64	6.82	0.012	MSTRG.43875	lncRNA
HSPA5	I myofibers	1.66	5.95	0.0107	ENSSSCG00000005601	protein coding
MAPKAP1	I myofibers	1.71	5.43	0.0173	ENSSSCG00000029169	protein coding
MSTRG.41285	I myofibers	1.71	5.16	0.00581	MSTRG.41285	lncRNA
SELENOP	I myofibers	1.74	6.42	0.00646	ENSSSCG00000021208	protein coding
SERPINF1	I myofibers	1.74	5.47	0.042	ENSSSCG00000017818	protein coding
C15orf40	I myofibers	1.75	6.23	0.00152	ENSSSCG00000030131	protein coding
MSTRG.45976	I myofibers	1.77	5.08	0.00532	MSTRG.45976	lncRNA
MSTRG.38467	I myofibers	1.82	8.53	0.0332	MSTRG.38467	lncRNA
NGLY1	I myofibers	1.91	5.36	0.0018	ENSSSCG00000011214	protein coding
TCF12	I myofibers	1.93	4.75	0.0186	ENSSSCG00000004600	protein coding
RRAD	I myofibers	1.94	4.84	0.0364	ENSSSCG00000036060	protein coding
TMSB10	I myofibers	1.94	6.91	0.00343	ENSSSCG00000008245	protein coding
SPARCL1	I myofibers	2.08	4.73	0.00195	ENSSSCG00000009222	protein coding
SELENOW	I myofibers	2.14	8.37	4.26E-05	ENSSSCG00000034313	protein coding
AUTS2	I myofibers	2.15	4.82	0.0103	ENSSSCG00000007727	protein coding

COL6A3	I myofibers	2.19	5.6	0.0328	ENSSSCG00000027331	protein coding
ANXA1	I myofibers	2.25	5.15	0.00859	ENSSSCG00000005267	protein coding
ARNT	I myofibers	2.26	4.2	0.0195	ENSSSCG00000006646	protein coding
GPX1	I myofibers	2.26	7.29	3.99E-05	ENSSSCG00000033727	protein coding
COL4A2	I myofibers	2.27	4.62	0.00621	ENSSSCG00000009545	protein coding
OGN	I myofibers	2.28	6.04	0.0413	ENSSSCG00000034607	protein coding
APOE	I myofibers	2.43	5.61	0.0336	ENSSSCG00000003088	protein coding
SERPINH1	I myofibers	2.44	5.06	0.0169	ENSSSCG00000039468	protein coding
MSTRG.23946	I myofibers	2.57	9.59	0.000565	MSTRG.23946	lncRNA
GPC3	I myofibers	2.58	5.14	0.00235	ENSSSCG00000012680	protein coding
ITGB1	I myofibers	2.63	4.49	0.00534	ENSSSCG00000011101	protein coding
TGFBR3	I myofibers	2.65	4.11	0.0244	ENSSSCG00000006911	protein coding
LUM	I myofibers	2.68	7.56	0.00421	ENSSSCG00000000916	protein coding
MYL9	I myofibers	2.81	6.13	0.000279	ENSSSCG00000036402	protein coding
MSTRG.9222	I myofibers	2.88	5.07	0.0112	MSTRG.9222	lncRNA
RNASE4	I myofibers	3.03	5.17	0.000148	ENSSSCG00000031538	protein coding
DNAJB1	I myofibers	3.34	5.82	2.03E-05	ENSSSCG00000013784	protein coding
MSTRG.25755	IIA myofibers	-2.62	4.12	0.00542	MSTRG.25755	lncRNA
FIS1	IIA myofibers	-2.45	3.74	0.00774	ENSSSCG00000038938	protein coding
ENAH	IIA myofibers	-2.42	4.73	0.00382	ENSSSCG00000038646	protein coding
XIRP2	IIA myofibers	-2.15	8.37	0.00113	ENSSSCG00000015917	protein coding
MYBPH	IIA myofibers	-2.03	9.87	0.00116	ENSSSCG00000030681	protein coding
MSTRG.49025	IIA myofibers	-1.85	4.13	0.0353	MSTRG.49025	lncRNA
NRAP	IIA myofibers	-1.84	6.61	0.000126	ENSSSCG00000010640	protein coding
C14orf93	IIA myofibers	-1.8	3.65	0.0249	ENSSSCG00000031452	protein coding
GADD45G	IIA myofibers	-1.77	6.49	0.00882	ENSSSCG00000035249	protein coding
TOB2	IIA myofibers	-1.58	3.76	0.023	ENSSSCG00000037958	protein coding
ENSSSCG00000036052	IIA myofibers	-1.5	5.49	0.00201	ENSSSCG00000036052	protein coding
IP6K3	IIA myofibers	-1.5	5.34	0.00122	ENSSSCG00000039272	protein coding
MSTRG.38177	IIA myofibers	-1.49	3.77	0.0214	MSTRG.38177	lncRNA
WDR37	IIA myofibers	-1.49	3.94	0.00676	ENSSSCG00000029602	protein coding
CHCHD4	IIA myofibers	-1.46	4.88	0.00159	ENSSSCG00000011604	protein coding
EMG1	IIA myofibers	-1.42	6.38	0.00141	ENSSSCG00000026812	protein coding
TAF15	IIA myofibers	-1.39	3.83	0.0123	ENSSSCG00000017707	protein coding
BLOC1S2	IIA myofibers	-1.37	4.11	0.0149	ENSSSCG00000010551	protein coding
MAVS	IIA myofibers	-1.34	4.15	0.0151	ENSSSCG00000007143	protein coding
TMEM69	IIA myofibers	-1.33	3.61	0.0234	ENSSSCG00000034035	protein coding
PRRC2C	IIA myofibers	-1.32	3.99	0.037	ENSSSCG00000015480	protein coding

MED27	IIA myofibers	-1.28	3.75	0.0317	ENSSSCG00000005720	protein coding
SRRT	IIA myofibers	-1.28	3.57	0.0273	ENSSSCG00000024789	protein coding
PLEKHF1	IIA myofibers	-1.27	4.84	0.0353	ENSSSCG00000002852	protein coding
ENSSSCG00000023091	IIA myofibers	-1.24	4.93	0.00449	ENSSSCG00000023091	protein coding
NEB	IIA myofibers	-1.23	8.61	0.0187	ENSSSCG00000016397	protein coding
DNPH1	IIA myofibers	-1.22	4.71	0.0457	ENSSSCG00000026568	protein coding
TRIM63	IIA myofibers	-1.21	6.78	0.00231	ENSSSCG00000027684	protein coding
NOC4L	IIA myofibers	-1.2	3.6	0.0359	ENSSSCG00000009739	protein coding
SERPINB6	IIA myofibers	-1.19	4.26	0.0189	ENSSSCG00000038404	protein coding
CFAP36	IIA myofibers	-1.18	4.05	0.0354	ENSSSCG00000008400	protein coding
MRPL9	IIA myofibers	-1.16	6.24	0.00818	ENSSSCG00000028343	protein coding
EIF2B3	IIA myofibers	-1.15	4.25	0.0107	ENSSSCG00000029808	protein coding
DNAJC11	IIA myofibers	-1.13	4.35	0.0303	ENSSSCG00000003384	protein coding
VPS72	IIA myofibers	-1.13	5.18	0.0192	ENSSSCG00000031869	protein coding
TCAF1	IIA myofibers	-1.09	5.12	0.0313	ENSSSCG00000016453	protein coding
PMPCB	IIA myofibers	-1.07	4.74	0.0385	ENSSSCG00000015422	protein coding
USP5	IIA myofibers	-1.07	4.98	0.0204	ENSSSCG00000000684	protein coding
CIAO3	IIA myofibers	-1.05	4.31	0.037	ENSSSCG00000008002	protein coding
HCFC1R1	IIA myofibers	-1.05	6.46	0.0336	ENSSSCG00000027252	protein coding
LONP1	IIA myofibers	-1.05	4.44	0.02	ENSSSCG00000027128	protein coding
PPIG	IIA myofibers	-1.05	4.35	0.0139	ENSSSCG00000031123	protein coding
SRSF7	IIA myofibers	-1.04	5.75	0.0312	ENSSSCG00000008484	protein coding
CCDC85B	IIA myofibers	-1.02	5.59	0.0112	ENSSSCG00000012968	protein coding
PRELID1	IIA myofibers	-1	4.32	0.0332	ENSSSCG00000031182	protein coding
DDX19B	IIA myofibers	-0.999	4.4	0.0328	ENSSSCG00000002722	protein coding
ENSSSCG00000025203	IIA myofibers	-0.995	4.27	0.0299	ENSSSCG00000025203	protein coding
FLYWCH2	IIA myofibers	-0.992	4.09	0.0303	ENSSSCG00000022630	protein coding
IFRD2	IIA myofibers	-0.972	5.36	0.0101	ENSSSCG00000011402	protein coding
SLC25A39	IIA myofibers	-0.968	4.43	0.0352	ENSSSCG00000030167	protein coding
DUSP23	IIA myofibers	-0.959	6.43	0.00946	ENSSSCG00000033585	protein coding
KIF5B	IIA myofibers	-0.959	5.68	0.018	ENSSSCG00000011027	protein coding
LEO1	IIA myofibers	-0.952	3.95	0.0418	ENSSSCG00000004625	protein coding
THUMPD1	IIA myofibers	-0.946	5.46	0.0112	ENSSSCG00000024748	protein coding
CLIC5	IIA myofibers	-0.945	4.98	0.0458	ENSSSCG00000037416	protein coding
CRYAB	IIA myofibers	-0.927	10.9	0.0122	ENSSSCG00000036724	protein coding
PNPO	IIA myofibers	-0.921	4.81	0.0489	ENSSSCG00000032782	protein coding
TRIM55	IIA myofibers	-0.919	5.22	0.0204	ENSSSCG00000006216	protein coding
PDE4DIP	IIA myofibers	-0.907	6.38	0.0176	ENSSSCG00000024481	protein coding

PTGES3	IIA myofibers	-0.907	4.81	0.0418	ENSSSCG00000000406	protein coding
TXNIP	IIA myofibers	-0.895	8.87	0.00817	ENSSSCG00000031262	protein coding
PSMC1	IIA myofibers	-0.888	5.91	0.0486	ENSSSCG00000002433	protein coding
CEBPZ	IIA myofibers	-0.887	4.7	0.0351	ENSSSCG00000008493	protein coding
DHX29	IIA myofibers	-0.886	5.51	0.0352	ENSSSCG00000016907	protein coding
TUBG1	IIA myofibers	-0.88	6.14	0.00829	ENSSSCG00000036106	protein coding
ENSSSCG00000040571	IIA myofibers	-0.865	7.16	0.0158	ENSSSCG00000040571	protein coding
PARVB	IIA myofibers	-0.859	6.25	0.0067	ENSSSCG00000000024	protein coding
MRTO4	IIA myofibers	-0.845	5.26	0.0488	ENSSSCG00000034814	protein coding
LIN37	IIA myofibers	-0.828	4.93	0.0474	ENSSSCG00000002898	protein coding
NCL	IIA myofibers	-0.816	5.62	0.0319	ENSSSCG00000016275	protein coding
TAF11	IIA myofibers	-0.816	5.16	0.0276	ENSSSCG00000001533	protein coding
MRPS7	IIA myofibers	-0.808	6.29	0.0367	ENSSSCG00000035134	protein coding
WASHC3	IIA myofibers	-0.803	5.25	0.0392	ENSSSCG00000036075	protein coding
ATP1B1	IIA myofibers	-0.802	5.71	0.0306	ENSSSCG00000006296	protein coding
ENSSSCG00000035544	IIA myofibers	-0.785	7.27	0.0163	ENSSSCG00000035544	protein coding
ENSSSCG00000020706	IIA myofibers	-0.783	5.45	0.0319	ENSSSCG00000020706	protein coding
C18orf21	IIA myofibers	-0.745	6.18	0.0398	ENSSSCG00000030002	protein coding
H3-3A	IIA myofibers	-0.744	6.53	0.036	ENSSSCG00000023971	protein coding
SRP68	IIA myofibers	-0.743	4.9	0.046	ENSSSCG00000017191	protein coding
FKBP4	IIA myofibers	-0.736	7.67	0.0103	ENSSSCG00000000743	protein coding
CCDC59	IIA myofibers	-0.734	5.31	0.0457	ENSSSCG00000038987	protein coding
AKR1B1	IIA myofibers	-0.726	6.71	0.0496	ENSSSCG00000040608	protein coding
DLD	IIA myofibers	-0.7	7.32	0.0474	ENSSSCG00000015443	protein coding
EIF4H	IIA myofibers	-0.692	6.47	0.0317	ENSSSCG00000025966	protein coding
ENSSSCG00000037979	IIA myofibers	-0.686	5.72	0.042	ENSSSCG00000037979	protein coding
NAA20	IIA myofibers	-0.678	8.08	0.0179	ENSSSCG00000028132	protein coding
MAP4	IIA myofibers	-0.675	6.05	0.0407	ENSSSCG00000029251	protein coding
PFN1	IIA myofibers	-0.674	7.22	0.0499	ENSSSCG00000017907	protein coding
TCAP	IIA myofibers	-0.646	12.6	0.0335	ENSSSCG00000017500	protein coding
ENSSSCG00000032067	IIA myofibers	-0.64	7.01	0.0419	ENSSSCG00000032067	protein coding
MRPL18	IIA myofibers	-0.637	6.35	0.0319	ENSSSCG00000004047	protein coding
MRPL51	IIA myofibers	-0.636	7.4	0.034	ENSSSCG00000039662	protein coding
RNF7	IIA myofibers	-0.629	7.16	0.0498	ENSSSCG00000029774	protein coding
ILF2	IIA myofibers	-0.62	6.15	0.0464	ENSSSCG00000028553	protein coding
TPT1	IIA myofibers	-0.618	12.8	0.0153	ENSSSCG00000040929	protein coding
TIMM17A	IIA myofibers	-0.617	6.15	0.0399	ENSSSCG00000037431	protein coding
MRPL24	IIA myofibers	-0.605	7.29	0.0338	ENSSSCG00000006469	protein coding

MRPL12	IIA myofibers	-0.592	8.47	0.0484	ENSSSCG00000033148	protein coding
ENSSSCG00000027746	IIA myofibers	-0.582	8.45	0.0321	ENSSSCG00000027746	protein coding
ATP2A1	IIA myofibers	-0.579	9.18	0.0376	ENSSSCG00000007805	protein coding
ENSSSCG00000037143	IIA myofibers	-0.558	6.73	0.0395	ENSSSCG00000037143	protein coding
MRPL13	IIA myofibers	-0.554	6.63	0.0444	ENSSSCG00000005996	protein coding
ENSSSCG00000012961	IIA myofibers	-0.547	8.68	0.0495	ENSSSCG00000012961	protein coding
ENSSSCG00000022757	IIA myofibers	-0.522	8.02	0.0464	ENSSSCG00000022757	lncRNA
RPS3	IIA myofibers	-0.475	11.3	0.0446	ENSSSCG00000014855	protein coding
STRADB	IIA myofibers	0.547	7.37	0.0487	ENSSSCG00000031271	protein coding
NAA50	IIA myofibers	0.57	6.51	0.0471	ENSSSCG00000011917	protein coding
ENSSSCG00000030849	IIA myofibers	0.639	11.9	0.00805	ENSSSCG00000030849	protein coding
ST13	IIA myofibers	0.649	7.89	0.0279	ENSSSCG00000000071	protein coding
CAPZA2	IIA myofibers	0.656	8.84	0.0245	ENSSSCG00000028113	protein coding
ENSSSCG00000039249	IIA myofibers	0.656	6.38	0.0466	ENSSSCG00000039249	protein coding
GPD1	IIA myofibers	0.665	8.49	0.0487	ENSSSCG00000000214	protein coding
DSTN	IIA myofibers	0.689	6.34	0.0198	ENSSSCG00000007085	protein coding
CHMP4B	IIA myofibers	0.691	6.38	0.0345	ENSSSCG00000007272	protein coding
SMIM4	IIA myofibers	0.694	6.75	0.0277	ENSSSCG00000034529	protein coding
ENSSSCG00000007188	IIA myofibers	0.695	7.52	0.0155	ENSSSCG00000007188	protein coding
PPP1CB	IIA myofibers	0.702	7.81	0.0132	ENSSSCG00000008540	protein coding
EPN1	IIA myofibers	0.709	5.98	0.0417	ENSSSCG00000039670	protein coding
ATF4	IIA myofibers	0.737	8.02	0.0113	ENSSSCG00000000084	protein coding
DYM	IIA myofibers	0.737	4.45	0.0487	ENSSSCG00000004508	protein coding
TPGS1	IIA myofibers	0.749	5.37	0.0395	ENSSSCG00000036434	protein coding
YAF2	IIA myofibers	0.754	5.2	0.047	ENSSSCG00000031775	protein coding
IVNS1ABP	IIA myofibers	0.756	6.75	0.0352	ENSSSCG00000015570	protein coding
PPP1R3A	IIA myofibers	0.762	6.3	0.0352	ENSSSCG00000016640	protein coding
CBX3	IIA myofibers	0.763	6.04	0.0186	ENSSSCG00000016711	protein coding
RAB6A	IIA myofibers	0.771	5.31	0.0467	ENSSSCG00000014828	protein coding
COPS2	IIA myofibers	0.807	6.05	0.0406	ENSSSCG00000004652	protein coding
RER1	IIA myofibers	0.835	4.15	0.0493	ENSSSCG00000026626	protein coding
TRAPPC13	IIA myofibers	0.839	4.98	0.0277	ENSSSCG00000016949	protein coding
MSTRG.43875	IIA myofibers	0.847	6.37	0.0385	MSTRG.43875	lncRNA
PPP2R2A	IIA myofibers	0.856	5.34	0.0208	ENSSSCG00000009657	protein coding
CCNG1	IIA myofibers	0.861	9.34	0.0101	ENSSSCG00000017024	protein coding
MCCC1	IIA myofibers	0.869	5.6	0.03	ENSSSCG00000025942	protein coding
ENSSSCG00000022906	IIA myofibers	0.873	5.07	0.0245	ENSSSCG00000022906	protein coding
IMPA1	IIA myofibers	0.888	4.83	0.0234	ENSSSCG00000033791	protein coding

ENSSSCG00000038991	IIA myofibers	0.92	4.08	0.0416	ENSSSCG00000038991	protein coding
ENSSSCG00000008451	IIA myofibers	0.925	6.27	0.0136	ENSSSCG00000008451	protein coding
DNAJC10	IIA myofibers	0.93	4.73	0.0192	ENSSSCG00000016017	protein coding
THOC2	IIA myofibers	0.937	4.02	0.0396	ENSSSCG00000012639	protein coding
ENSSSCG00000022176	IIA myofibers	0.958	4.77	0.0493	ENSSSCG00000022176	protein coding
KLHL24	IIA myofibers	0.961	4.93	0.0153	ENSSSCG00000011775	protein coding
SARS1	IIA myofibers	0.967	6.52	0.0328	ENSSSCG00000006835	protein coding
TIMP1	IIA myofibers	0.969	6.04	0.00494	ENSSSCG00000012277	protein coding
EMP3	IIA myofibers	0.982	4.8	0.0162	ENSSSCG00000023377	protein coding
HIBCH	IIA myofibers	1.01	4.03	0.0297	ENSSSCG00000016049	protein coding
RBM38	IIA myofibers	1.01	6.65	0.0314	ENSSSCG00000007504	protein coding
PSME1	IIA myofibers	1.02	5.05	0.0473	ENSSSCG00000002006	protein coding
CTDSP1	IIA myofibers	1.03	4.25	0.0149	ENSSSCG00000036938	protein coding
CARNMT1	IIA myofibers	1.05	5.34	0.0187	ENSSSCG00000005271	protein coding
CD164	IIA myofibers	1.05	5.1	0.0159	ENSSSCG00000004414	protein coding
CD36	IIA myofibers	1.05	6.19	0.0049	ENSSSCG00000015405	protein coding
SPARCL1	IIA myofibers	1.05	4.91	0.0378	ENSSSCG00000009222	protein coding
MSH4	IIA myofibers	1.07	6.07	0.0373	ENSSSCG00000003775	protein coding
CITED2	IIA myofibers	1.1	5.54	0.012	ENSSSCG00000004142	protein coding
ENSSSCG00000008137	IIA myofibers	1.1	3.96	0.0193	ENSSSCG00000008137	protein coding
GADL1	IIA myofibers	1.1	6.85	0.0126	ENSSSCG00000038607	protein coding
LAPTM4A	IIA myofibers	1.14	5.32	0.0418	ENSSSCG00000008602	protein coding
C15orf40	IIA myofibers	1.15	5.8	0.0104	ENSSSCG00000030131	protein coding
SMCO1	IIA myofibers	1.15	4.52	0.0348	ENSSSCG00000031614	protein coding
BTG1	IIA myofibers	1.18	6.32	0.0116	ENSSSCG00000035790	protein coding
ENSSSCG00000016768	IIA myofibers	1.18	4.52	0.0194	ENSSSCG00000016768	protein coding
ENSSSCG00000012119	IIA myofibers	1.19	7.55	0.0334	ENSSSCG00000012119	protein coding
MSTRG.23946	IIA myofibers	1.21	8.84	0.0473	MSTRG.23946	lncRNA
LRRC39	IIA myofibers	1.23	8.96	0.00129	ENSSSCG00000006867	protein coding
KPNA3	IIA myofibers	1.24	5.67	0.00281	ENSSSCG00000024125	protein coding
LACTB2	IIA myofibers	1.24	6.16	0.00288	ENSSSCG00000006192	protein coding
SLC40A1	IIA myofibers	1.26	3.85	0.0304	ENSSSCG00000027157	protein coding
SELENBP1	IIA myofibers	1.31	5.05	0.00416	ENSSSCG00000006624	protein coding
HSPH1	IIA myofibers	1.34	3.88	0.0434	ENSSSCG00000009334	protein coding
TMED10	IIA myofibers	1.37	3.85	0.0351	ENSSSCG00000035684	protein coding
SELENOH	IIA myofibers	1.38	6.21	0.000444	ENSSSCG00000032364	protein coding
DDX3X	IIA myofibers	1.43	4.55	0.00559	ENSSSCG00000012252	protein coding
CLIC4	IIA myofibers	1.46	4.8	0.0418	ENSSSCG00000038994	protein coding

FDFT1	IIA myofibers	1.49	4.74	0.0221	ENSSSCG00000026044	protein coding
NRAS	IIA myofibers	1.49	3.91	0.0132	ENSSSCG00000032477	protein coding
PSPH	IIA myofibers	1.54	3.94	0.0391	ENSSSCG00000007748	protein coding
GPX1	IIA myofibers	1.58	6.92	0.00134	ENSSSCG00000033727	protein coding
TMSB10	IIA myofibers	1.59	6.88	0.0143	ENSSSCG00000008245	protein coding
MSTRG.8412	IIA myofibers	1.61	4.44	0.00654	MSTRG.8412	lncRNA
MSTRG.25593	IIA myofibers	1.62	4.97	0.0116	MSTRG.25593	lncRNA
OPTN	IIA myofibers	1.68	4.51	0.00291	ENSSSCG00000023247	protein coding
AUTS2	IIA myofibers	1.7	4.32	0.0267	ENSSSCG00000007727	protein coding
YPEL3	IIA myofibers	1.72	4.29	0.00661	ENSSSCG00000035430	protein coding
CA3	IIA myofibers	1.74	7.49	0.0273	ENSSSCG00000006141	protein coding
NT5C3A	IIA myofibers	1.76	4.19	0.00303	ENSSSCG00000022912	protein coding
SELENOM	IIA myofibers	1.76	5.16	0.00592	ENSSSCG00000029781	protein coding
TRAM1	IIA myofibers	1.8	3.87	0.00744	ENSSSCG00000006193	protein coding
GPX3	IIA myofibers	1.82	3.96	0.0205	ENSSSCG00000036438	protein coding
MYL9	IIA myofibers	1.9	5.79	0.00157	ENSSSCG00000036402	protein coding
LUM	IIA myofibers	1.92	7.65	0.0424	ENSSSCG00000000916	protein coding
SELENOP	IIA myofibers	1.96	6.61	0.00153	ENSSSCG00000021208	protein coding
PENK	IIA myofibers	1.99	4.26	0.0467	ENSSSCG00000006243	protein coding
BGN	IIA myofibers	2.1	4.35	0.0374	ENSSSCG00000012766	protein coding
MSTRG.17300	IIA myofibers	2.18	4.34	0.0118	MSTRG.17300	lncRNA
CAPN6	IIA myofibers	2.19	3.57	0.0498	ENSSSCG00000012584	protein coding
MSTRG.30964	IIA myofibers	2.25	4.61	0.000198	MSTRG.30964	lncRNA
TPM4	IIA myofibers	2.32	4.27	0.00566	ENSSSCG00000013849	protein coding
ASPN	IIA myofibers	2.33	3.91	0.0307	ENSSSCG00000008072	protein coding
DNAJB1	IIA myofibers	2.34	5.63	0.00726	ENSSSCG00000013784	protein coding
NUPR1	IIA myofibers	2.34	4.21	0.0335	ENSSSCG00000040162	protein coding
SELENOW	IIA myofibers	2.36	8.45	0.000119	ENSSSCG00000034313	protein coding
MSTRG.9222	IIA myofibers	2.38	4.94	0.0433	MSTRG.9222	lncRNA
OGN	IIA myofibers	2.38	6.03	0.0167	ENSSSCG00000034607	protein coding
CDK14	IIA myofibers	2.39	3.59	0.0257	ENSSSCG00000015307	protein coding
APOD	IIA myofibers	2.45	5.03	0.000409	ENSSSCG00000011831	protein coding
PGM1	IIA myofibers	2.94	5.78	0.0359	ENSSSCG00000003812	protein coding
MSTRG.37393	IIA myofibers	3.25	4	0.000689	MSTRG.37393	lncRNA
MSTRG.25755	IIX myofibers	-2.4	4.3	0.00831	MSTRG.25755	lncRNA
FIS1	IIX myofibers	-1.98	3.78	0.0106	ENSSSCG00000038938	protein coding
MSTRG.17458	IIX myofibers	-1.9	4.63	0.0332	MSTRG.17458	lncRNA
XIRP2	IIX myofibers	-1.88	8.27	0.000313	ENSSSCG00000015917	protein coding

PMPCB	IIX myofibers	-1.8	4.78	0.00691	ENSSSCG00000015422	protein coding
ENSSSCG00000045624	IIX myofibers	-1.78	6.32	0.0187	ENSSSCG00000045624	lncRNA
GRWD1	IIX myofibers	-1.62	4.82	0.0131	ENSSSCG00000003134	protein coding
TNNT1	IIX myofibers	-1.55	6.79	0.041	ENSSSCG00000025353	protein coding
ENAH	IIX myofibers	-1.5	5.31	0.0282	ENSSSCG00000038646	protein coding
WDR18	IIX myofibers	-1.45	4.37	0.00428	ENSSSCG00000013423	protein coding
GPATCH4	IIX myofibers	-1.38	4.84	0.003	ENSSSCG00000006481	protein coding
PNPO	IIX myofibers	-1.38	4.82	0.0114	ENSSSCG00000032782	protein coding
USP13	IIX myofibers	-1.37	5.26	0.0352	ENSSSCG00000011765	protein coding
GUCD1	IIX myofibers	-1.35	3.85	0.0445	ENSSSCG00000010059	protein coding
C5orf63	IIX myofibers	-1.33	4.14	0.0293	ENSSSCG00000014250	protein coding
DDRGK1	IIX myofibers	-1.33	4.72	0.00806	ENSSSCG00000007160	protein coding
PPP1R3B	IIX myofibers	-1.33	6.5	0.0119	ENSSSCG00000022797	protein coding
SRSF7	IIX myofibers	-1.3	5.91	0.0103	ENSSSCG00000008484	protein coding
EIF2B3	IIX myofibers	-1.21	4.56	0.0132	ENSSSCG00000029808	protein coding
ENSSSCG00000040571	IIX myofibers	-1.21	6.72	0.00687	ENSSSCG00000040571	protein coding
MAGOHB	IIX myofibers	-1.2	4.36	0.0361	ENSSSCG00000000635	protein coding
KIF5B	IIX myofibers	-1.18	5.59	0.00333	ENSSSCG00000011027	protein coding
CHCHD4	IIX myofibers	-1.15	4.62	0.0166	ENSSSCG00000011604	protein coding
ENSSSCG00000016982	IIX myofibers	-1.15	4.04	0.0305	ENSSSCG00000016982	protein coding
PPIH	IIX myofibers	-1.15	4.65	0.0178	ENSSSCG00000003964	protein coding
METTL21A	IIX myofibers	-1.14	3.84	0.0413	ENSSSCG00000036466	protein coding
MSTRG.35464	IIX myofibers	-1.13	4.63	0.0164	MSTRG.35464	lncRNA
NOP56	IIX myofibers	-1.12	5.31	0.0149	ENSSSCG00000027676	protein coding
MSI2	IIX myofibers	-1.09	3.73	0.0479	ENSSSCG00000038505	protein coding
PTDSS1	IIX myofibers	-1.04	5.82	0.0474	ENSSSCG00000006089	protein coding
TRIM63	IIX myofibers	-1.03	7.17	0.0169	ENSSSCG00000027684	protein coding
GNL3	IIX myofibers	-1.01	5.98	0.0335	ENSSSCG00000011449	protein coding
TSR2	IIX myofibers	-1.01	4.38	0.035	ENSSSCG00000012334	protein coding
ELOA	IIX myofibers	-1	4.35	0.0341	ENSSSCG00000025440	protein coding
CSRP3	IIX myofibers	-0.998	10.5	0.0431	ENSSSCG00000013354	protein coding
CCDC85B	IIX myofibers	-0.996	5.82	0.0297	ENSSSCG00000012968	protein coding
HCFC1R1	IIX myofibers	-0.996	6.64	0.042	ENSSSCG00000027252	protein coding
IFRD2	IIX myofibers	-0.995	5.45	0.0206	ENSSSCG00000011402	protein coding
USE1	IIX myofibers	-0.983	4.45	0.0262	ENSSSCG00000013875	protein coding
SHARPIN	IIX myofibers	-0.981	4.94	0.0436	ENSSSCG00000005914	protein coding
TFPT	IIX myofibers	-0.968	4.1	0.0497	ENSSSCG00000003262	protein coding
VARS1	IIX myofibers	-0.955	5.04	0.0318	ENSSSCG00000038950	protein coding

BABAM2	IIX myofibers	-0.949	4.48	0.0275	ENSSSCG00000026465	protein coding
DNAJC11	IIX myofibers	-0.943	4.26	0.0415	ENSSSCG00000003384	protein coding
ENSSSCG00000023091	IIX myofibers	-0.931	5.21	0.015	ENSSSCG00000023091	protein coding
NAA20	IIX myofibers	-0.929	7.99	0.00488	ENSSSCG00000028132	protein coding
GSTO1	IIX myofibers	-0.925	5.84	0.0435	ENSSSCG00000022351	protein coding
MRPS7	IIX myofibers	-0.92	6.42	0.0136	ENSSSCG00000035134	protein coding
MYBPH	IIX myofibers	-0.914	10.7	0.0153	ENSSSCG00000030681	protein coding
SRPRA	IIX myofibers	-0.9	4.43	0.042	ENSSSCG00000015227	protein coding
ARRDC3	IIX myofibers	-0.885	4.54	0.0458	ENSSSCG00000014156	protein coding
TUBA8	IIX myofibers	-0.869	6.13	0.0248	ENSSSCG00000027190	protein coding
NSFL1C	IIX myofibers	-0.858	5.78	0.0161	ENSSSCG00000007187	protein coding
PPP5C	IIX myofibers	-0.852	5.37	0.0426	ENSSSCG00000003098	protein coding
EIPR1	IIX myofibers	-0.847	5.68	0.0208	ENSSSCG00000028755	protein coding
DCTPP1	IIX myofibers	-0.836	5.98	0.0376	ENSSSCG00000007795	protein coding
ENSSSCG00000037979	IIX myofibers	-0.82	5.89	0.0488	ENSSSCG00000037979	protein coding
CHCHD1	IIX myofibers	-0.813	6.45	0.0215	ENSSSCG00000010308	protein coding
TUBA4A	IIX myofibers	-0.81	9.95	0.0261	ENSSSCG00000016216	protein coding
PTPMT1	IIX myofibers	-0.809	5.69	0.0325	ENSSSCG00000013226	protein coding
THUMPD1	IIX myofibers	-0.808	5.41	0.0327	ENSSSCG00000024748	protein coding
CCDC59	IIX myofibers	-0.805	5.37	0.0307	ENSSSCG00000038987	protein coding
CRYAB	IIX myofibers	-0.799	10.8	0.0249	ENSSSCG00000036724	protein coding
SLC35A4	IIX myofibers	-0.794	6.02	0.0233	ENSSSCG00000014367	protein coding
MTG1	IIX myofibers	-0.792	5.72	0.0231	ENSSSCG00000029088	protein coding
RFXANK	IIX myofibers	-0.792	5.73	0.0471	ENSSSCG00000036310	protein coding
ENSSSCG00000032067	IIX myofibers	-0.785	6.49	0.0344	ENSSSCG00000032067	protein coding
ENSSSCG00000012879	IIX myofibers	-0.782	6.69	0.0348	ENSSSCG00000012879	protein coding
ENSSSCG00000031608	IIX myofibers	-0.736	9.85	0.0176	ENSSSCG00000031608	protein coding
ENSSSCG00000027746	IIX myofibers	-0.726	8.08	0.0202	ENSSSCG00000027746	protein coding
MRPS31	IIX myofibers	-0.716	5.12	0.0418	ENSSSCG00000009372	protein coding
COPS8	IIX myofibers	-0.713	6.45	0.045	ENSSSCG00000016323	protein coding
TBRG4	IIX myofibers	-0.713	6.09	0.0473	ENSSSCG00000016732	protein coding
BCCIP	IIX myofibers	-0.663	6.29	0.047	ENSSSCG00000010743	protein coding
MRPL27	IIX myofibers	-0.651	6.59	0.0458	ENSSSCG00000023783	protein coding
TPT1	IIX myofibers	-0.646	12.9	0.0327	ENSSSCG00000040929	protein coding
RPS3	IIX myofibers	-0.629	11.4	0.0218	ENSSSCG00000014855	protein coding
PSMD4	IIX myofibers	-0.608	7.08	0.0364	ENSSSCG00000006628	protein coding
ENSSSCG00000030849	IIX myofibers	0.527	11.9	0.0404	ENSSSCG00000030849	protein coding
LGALS1	IIX myofibers	0.676	10.6	0.047	ENSSSCG00000033854	protein coding

CAPZA2	IIX myofibers	0.686	9.06	0.0237	ENSSSCG00000028113	protein coding
NIT1	IIX myofibers	0.706	5.33	0.0464	ENSSSCG00000037793	protein coding
MSTRG.39186	IIX myofibers	0.729	7.45	0.0271	MSTRG.39186	lncRNA
ZFAND6	IIX myofibers	0.735	6.77	0.0309	ENSSSCG00000001779	protein coding
ATF4	IIX myofibers	0.744	8.13	0.0424	ENSSSCG00000000084	protein coding
SUPT4H1	IIX myofibers	0.756	5.19	0.0376	ENSSSCG00000034752	protein coding
MRPL39	IIX myofibers	0.76	6.22	0.0435	ENSSSCG00000012021	protein coding
HNRNPA0	IIX myofibers	0.771	5.97	0.0201	ENSSSCG00000042630	protein coding
USP15	IIX myofibers	0.775	4.95	0.0353	ENSSSCG00000000457	protein coding
ROMO1	IIX myofibers	0.78	7.42	0.0421	ENSSSCG00000007308	protein coding
SUMO1	IIX myofibers	0.788	6.26	0.0174	ENSSSCG00000035842	protein coding
PPP1CB	IIX myofibers	0.792	7.65	0.0161	ENSSSCG00000008540	protein coding
MSTRG.21597	IIX myofibers	0.804	4.69	0.0479	MSTRG.21597	lncRNA
ZBED5	IIX myofibers	0.804	4.28	0.0435	ENSSSCG00000033411	protein coding
GPD1	IIX myofibers	0.807	8.54	0.0469	ENSSSCG00000000214	protein coding
CHMP4B	IIX myofibers	0.816	6.62	0.0254	ENSSSCG00000007272	protein coding
RILPL1	IIX myofibers	0.819	5.7	0.0289	ENSSSCG00000009773	protein coding
C15orf40	IIX myofibers	0.82	5.55	0.0142	ENSSSCG00000030131	protein coding
BZW1	IIX myofibers	0.838	6.66	0.0225	ENSSSCG00000016094	protein coding
TMBIM6	IIX myofibers	0.851	4.8	0.0489	ENSSSCG00000029652	protein coding
CTDSP1	IIX myofibers	0.869	4.48	0.0466	ENSSSCG00000036938	protein coding
CTSV	IIX myofibers	0.886	4.52	0.0496	ENSSSCG00000011171	protein coding
CD164	IIX myofibers	0.906	5.6	0.0161	ENSSSCG00000004414	protein coding
ENSSSCG00000012916	IIX myofibers	0.929	5.83	0.0253	ENSSSCG00000012916	protein coding
UBALD1	IIX myofibers	0.932	6.17	0.0427	ENSSSCG00000023603	protein coding
ZNF277	IIX myofibers	0.937	5.02	0.022	ENSSSCG00000025910	protein coding
DNAJB4	IIX myofibers	0.94	6.54	0.00977	ENSSSCG00000003766	protein coding
PPP1R3A	IIX myofibers	0.942	6.55	0.0102	ENSSSCG00000016640	protein coding
TST	IIX myofibers	0.943	6.07	0.0358	ENSSSCG00000000133	protein coding
ENSSSCG00000005768	IIX myofibers	0.956	5.06	0.0478	ENSSSCG00000005768	protein coding
TSC22D3	IIX myofibers	0.968	6.83	0.0185	ENSSSCG00000030241	protein coding
CBX3	IIX myofibers	0.972	5.85	0.00918	ENSSSCG00000016711	protein coding
RNF11	IIX myofibers	0.982	5.49	0.0377	ENSSSCG00000031172	protein coding
DDX3X	IIX myofibers	0.994	5.1	0.04	ENSSSCG00000012252	protein coding
RORA	IIX myofibers	1.02	4.8	0.0431	ENSSSCG00000004576	protein coding
ENSSSCG00000032235	IIX myofibers	1.05	4.82	0.043	ENSSSCG00000032235	protein coding
COX6A1	IIX myofibers	1.07	5.1	0.0382	ENSSSCG00000039265	protein coding
ENSSSCG00000039331	IIX myofibers	1.07	5.08	0.0383	ENSSSCG00000039331	protein coding

SESN1	IIX myofibers	1.08	6.7	0.0282	ENSSSCG00000004390	protein coding
ATF2	IIX myofibers	1.1	4.58	0.0399	ENSSSCG00000038076	protein coding
CD36	IIX myofibers	1.1	5.99	0.02	ENSSSCG00000015405	protein coding
NUDT15	IIX myofibers	1.11	5.36	0.00805	ENSSSCG00000027551	protein coding
SYPL1	IIX myofibers	1.11	5.92	0.0286	ENSSSCG00000015434	protein coding
DMD	IIX myofibers	1.12	4.73	0.0225	ENSSSCG00000028148	protein coding
MYL9	IIX myofibers	1.12	6.78	0.033	ENSSSCG00000036402	protein coding
ARF6	IIX myofibers	1.14	5.76	0.00395	ENSSSCG00000005012	protein coding
YAF2	IIX myofibers	1.14	5.52	0.00728	ENSSSCG00000031775	protein coding
KPNA3	IIX myofibers	1.16	5.94	0.0172	ENSSSCG00000024125	protein coding
PCMTD1	IIX myofibers	1.16	4.66	0.025	ENSSSCG00000006267	protein coding
ATP6V0B	IIX myofibers	1.17	4.43	0.0491	ENSSSCG00000039267	protein coding
CRK	IIX myofibers	1.19	8.28	0.00891	ENSSSCG00000038989	protein coding
MSTRG.32961	IIX myofibers	1.2	4.13	0.0275	MSTRG.32961	lncRNA
HSF2	IIX myofibers	1.21	4.72	0.0302	ENSSSCG00000004238	protein coding
LACTB2	IIX myofibers	1.21	5.85	0.0232	ENSSSCG00000006192	protein coding
TCF12	IIX myofibers	1.23	4.42	0.049	ENSSSCG00000004600	protein coding
BTG1	IIX myofibers	1.24	6.63	0.0229	ENSSSCG00000035790	protein coding
MSTRG.32627	IIX myofibers	1.25	4.26	0.012	MSTRG.32627	lncRNA
ENSSSCG00000008451	IIX myofibers	1.26	6.16	0.00852	ENSSSCG00000008451	protein coding
PPM1A	IIX myofibers	1.26	4.91	0.00608	ENSSSCG00000005084	protein coding
PLPP1	IIX myofibers	1.27	4.2	0.0216	ENSSSCG00000016912	protein coding
CARNMT1	IIX myofibers	1.3	4.68	0.00502	ENSSSCG00000005271	protein coding
ENSSSCG00000038991	IIX myofibers	1.31	4.39	0.0185	ENSSSCG00000038991	protein coding
RNF34	IIX myofibers	1.31	5.15	0.0364	ENSSSCG00000037811	protein coding
TMEM59	IIX myofibers	1.31	4.47	0.0226	ENSSSCG00000034261	protein coding
KLHL24	IIX myofibers	1.32	4.79	0.0392	ENSSSCG00000011775	protein coding
TM9SF2	IIX myofibers	1.32	4.36	0.00747	ENSSSCG00000031808	protein coding
MSTRG.43875	IIX myofibers	1.35	7.43	0.0227	MSTRG.43875	lncRNA
SELENOH	IIX myofibers	1.35	6.27	0.00155	ENSSSCG00000032364	protein coding
ENSSSCG00000040056	IIX myofibers	1.36	6.33	0.00934	ENSSSCG00000040056	protein coding
DDIT4L	IIX myofibers	1.4	7.37	0.0139	ENSSSCG00000025240	protein coding
AP2B1	IIX myofibers	1.42	4.66	0.007	ENSSSCG00000017712	protein coding
LHFPL6	IIX myofibers	1.43	4.88	0.0356	ENSSSCG00000029855	protein coding
CITED2	IIX myofibers	1.45	5.58	0.00331	ENSSSCG00000004142	protein coding
EMP1	IIX myofibers	1.46	4.33	0.0375	ENSSSCG00000036723	protein coding
HDAC5	IIX myofibers	1.46	4.14	0.00973	ENSSSCG00000017366	protein coding
ANXA2	IIX myofibers	1.47	6.15	0.0195	ENSSSCG00000004578	protein coding

RBMS1	IIX myofibers	1.47	4.58	0.0369	ENSSSCG00000015888	protein coding
GPX1	IIX myofibers	1.48	7.08	0.000787	ENSSSCG00000033727	protein coding
IER3	IIX myofibers	1.5	4.32	0.0296	ENSSSCG00000027607	protein coding
MSTRG.1412	IIX myofibers	1.54	4.42	0.0141	MSTRG.1412	lncRNA
SELENOP	IIX myofibers	1.56	6.53	0.0134	ENSSSCG00000021208	protein coding
TPM4	IIX myofibers	1.56	4.55	0.0423	ENSSSCG00000013849	protein coding
YPEL5	IIX myofibers	1.57	4.26	0.00248	ENSSSCG00000008529	protein coding
S100A6	IIX myofibers	1.61	4.92	0.0407	ENSSSCG00000021084	protein coding
FDFT1	IIX myofibers	1.63	4.97	0.00672	ENSSSCG00000026044	protein coding
SELENBP1	IIX myofibers	1.67	4.7	0.0303	ENSSSCG00000006624	protein coding
ENSSSCG00000029160	IIX myofibers	1.75	7.56	0.00198	ENSSSCG00000029160	protein coding
MSTRG.25664	IIX myofibers	1.76	5.76	0.0394	MSTRG.25664	lncRNA
ZEB2	IIX myofibers	1.77	4.19	0.0267	ENSSSCG00000021343	protein coding
LAMP1	IIX myofibers	1.84	4.45	0.00288	ENSSSCG00000009555	protein coding
SELENOM	IIX myofibers	1.85	4.61	0.0171	ENSSSCG00000029781	protein coding
CISH	IIX myofibers	1.89	5.63	0.0366	ENSSSCG00000040581	protein coding
CLIC4	IIX myofibers	1.93	5.25	0.0111	ENSSSCG00000038994	protein coding
MSTRG.23946	IIX myofibers	1.93	9.76	0.0295	MSTRG.23946	lncRNA
NRAS	IIX myofibers	1.93	4.43	0.00248	ENSSSCG00000032477	protein coding
TMSB10	IIX myofibers	1.93	7.64	0.00694	ENSSSCG00000008245	protein coding
TGFBR3	IIX myofibers	1.98	4.29	0.0429	ENSSSCG00000006911	protein coding
ASNS	IIX myofibers	2	5.47	0.0453	ENSSSCG00000015340	protein coding
MED13L	IIX myofibers	2	4.43	0.0176	ENSSSCG00000009864	protein coding
CAPN6	IIX myofibers	2.15	4.23	0.0475	ENSSSCG00000012584	protein coding
P3H2	IIX myofibers	2.21	4.11	0.0189	ENSSSCG00000011813	protein coding
SELENOW	IIX myofibers	2.34	8.12	0.000448	ENSSSCG00000034313	protein coding
YPEL3	IIX myofibers	2.38	4.91	0.00209	ENSSSCG00000035430	protein coding
DNAJB1	IIX myofibers	2.39	5.68	0.00252	ENSSSCG00000013784	protein coding
APOD	IIX myofibers	2.4	5.5	0.00245	ENSSSCG00000011831	protein coding
MSTRG.30964	IIX myofibers	2.41	4.54	0.00297	MSTRG.30964	lncRNA
PGM1	IIX myofibers	2.81	6.21	0.035	ENSSSCG00000003812	protein coding
TNNT2	IIX myofibers	3.09	6	3.91E-05	ENSSSCG00000023031	protein coding
MSTRG.882	IIX myofibers	4.11	4.42	0.000628	MSTRG.882	lncRNA
MSTRG.8458	*MYL1*$^{+}$ Myoblasts	-7.68	6.93	2.33E-05	MSTRG.8458	lncRNA
MSTRG.11093	*MYL1*$^{+}$ Myoblasts	-3.93	5.45	0.000135	MSTRG.11093	lncRNA
NRSN1	*MYL1*$^{+}$ Myoblasts	-3.71	5.52	0.00121	ENSSSCG00000021696	protein coding
MSTRG.31305	*MYL1*$^{+}$ Myoblasts	-3.52	6.09	2.39E-05	MSTRG.31305	lncRNA
MSTRG.39086	*MYL1*$^{+}$ Myoblasts	-3.51	8.55	0.00144	MSTRG.39086	lncRNA

FNDC1	*MYL1*$^{+}$ Myoblasts	-3.33	5.41	0.000367	ENSSSCG00000004052	protein coding
MSTRG.26550	*MYL1*$^{+}$ Myoblasts	-3.28	4.46	0.0063	MSTRG.26550	lncRNA
DIO1	*MYL1*$^{+}$ Myoblasts	-3.01	6.66	0.0115	ENSSSCG00000028896	protein coding
TNNT1	*MYL1*$^{+}$ Myoblasts	-2.96	7.78	0.00397	ENSSSCG00000025353	protein coding
PLXNA4	*MYL1*$^{+}$ Myoblasts	-2.94	5.43	0.000224	ENSSSCG00000021562	protein coding
MSTRG.37468	*MYL1*$^{+}$ Myoblasts	-2.91	4.74	0.0087	MSTRG.37468	lncRNA
ENSSSCG00000029441	*MYL1*$^{+}$ Myoblasts	-2.88	6.4	0.0178	ENSSSCG00000029441	protein coding
MT1A	*MYL1*$^{+}$ Myoblasts	-2.76	6.59	0.00211	ENSSSCG00000023684	protein coding
ACTN3	*MYL1*$^{+}$ Myoblasts	-2.7	6.05	0.0323	ENSSSCG00000038323	protein coding
ENSSSCG00000031608	*MYL1*$^{+}$ Myoblasts	-2.56	7.47	0.00392	ENSSSCG00000031608	protein coding
PDE7B	*MYL1*$^{+}$ Myoblasts	-2.56	6.54	0.00981	ENSSSCG00000004165	protein coding
MSTRG.38018	*MYL1*$^{+}$ Myoblasts	-2.48	5.22	0.00446	MSTRG.38018	lncRNA
MYH7	*MYL1*$^{+}$ Myoblasts	-2.45	6.16	0.0297	ENSSSCG00000002029	protein coding
MSTRG.39368	*MYL1*$^{+}$ Myoblasts	-2.37	6.57	0.000269	MSTRG.39368	lncRNA
MSTRG.28323	*MYL1*$^{+}$ Myoblasts	-2.29	6.17	0.000345	MSTRG.28323	lncRNA
ENSSSCG00000038561	*MYL1*$^{+}$ Myoblasts	-2.2	5.51	0.00322	ENSSSCG00000038561	protein coding
MSTRG.13203	*MYL1*$^{+}$ Myoblasts	-2.1	5.2	0.0034	MSTRG.13203	lncRNA
C1orf21	*MYL1*$^{+}$ Myoblasts	-2.09	7.06	0.00771	ENSSSCG00000015565	protein coding
MYBPC2	*MYL1*$^{+}$ Myoblasts	-2.06	6.56	0.0442	ENSSSCG00000003218	protein coding
MSTRG.40588	*MYL1*$^{+}$ Myoblasts	-2.04	4.97	0.048	MSTRG.40588	lncRNA
PANK4	*MYL1*$^{+}$ Myoblasts	-2.03	5.5	0.00601	ENSSSCG00000027299	protein coding
MSTRG.30373	*MYL1*$^{+}$ Myoblasts	-1.98	6.29	0.00125	MSTRG.30373	lncRNA
JUNB	*MYL1*$^{+}$ Myoblasts	-1.94	5.9	0.00604	ENSSSCG00000013735	protein coding
TSTD2	*MYL1*$^{+}$ Myoblasts	-1.89	5.06	0.00557	ENSSSCG00000005367	protein coding
PEBP4	*MYL1*$^{+}$ Myoblasts	-1.87	6.28	0.044	ENSSSCG00000009631	protein coding
ENSSSCG00000042639	*MYL1*$^{+}$ Myoblasts	-1.85	4.69	0.0232	ENSSSCG00000042639	lncRNA
EXOG	*MYL1*$^{+}$ Myoblasts	-1.83	4.57	0.0268	ENSSSCG00000011258	protein coding
MSTRG.6783	*MYL1*$^{+}$ Myoblasts	-1.83	5.68	0.0015	MSTRG.6783	lncRNA
MSTRG.39706	*MYL1*$^{+}$ Myoblasts	-1.81	8.28	0.0037	MSTRG.39706	lncRNA
ANKRD39	*MYL1*$^{+}$ Myoblasts	-1.77	8.34	0.00985	ENSSSCG00000035533	protein coding
MSTRG.21573	*MYL1*$^{+}$ Myoblasts	-1.77	5.37	0.0167	MSTRG.21573	lncRNA
NRAP	*MYL1*$^{+}$ Myoblasts	-1.76	7.11	0.028	ENSSSCG00000010640	protein coding
DHRS7C	*MYL1*$^{+}$ Myoblasts	-1.74	5.28	0.0366	ENSSSCG00000017996	protein coding
CACNA1S	*MYL1*$^{+}$ Myoblasts	-1.73	6.42	0.0123	ENSSSCG00000024784	protein coding

MPP3	*MYL1* $^{+}$ Myoblasts	-1.7	6.37	0.000698	ENSSSCG00000017372	protein coding
ZC3HAV1	*MYL1* $^{+}$ Myoblasts	-1.68	5.02	0.0104	ENSSSCG00000016512	protein coding
MKKS	*MYL1* $^{+}$ Myoblasts	-1.66	5.25	0.00495	ENSSSCG00000007064	protein coding
MSTRG.1952	*MYL1* $^{+}$ Myoblasts	-1.65	6.01	0.00422	MSTRG.1952	lncRNA
MSTRG.41928	*MYL1* $^{+}$ Myoblasts	-1.65	4.89	0.021	MSTRG.41928	lncRNA
PPP1R3B	*MYL1* $^{+}$ Myoblasts	-1.65	6.03	0.0128	ENSSSCG00000022797	protein coding
GSTM3	*MYL1* $^{+}$ Myoblasts	-1.64	8.93	0.01	ENSSSCG00000006821	protein coding
ENSSSCG00000015747	*MYL1* $^{+}$ Myoblasts	-1.61	6.98	0.0117	ENSSSCG00000015747	protein coding
LDHA	*MYL1* $^{+}$ Myoblasts	-1.61	8.56	0.0215	ENSSSCG00000013366	protein coding
DPF3	*MYL1* $^{+}$ Myoblasts	-1.6	5.74	0.0243	ENSSSCG00000002333	protein coding
PHKB	*MYL1* $^{+}$ Myoblasts	-1.6	6.39	0.00365	ENSSSCG00000002844	protein coding
ZBTB16	*MYL1* $^{+}$ Myoblasts	-1.6	9.08	0.0379	ENSSSCG00000030095	protein coding
CEP63	*MYL1* $^{+}$ Myoblasts	-1.58	5.14	0.0111	ENSSSCG00000011645	protein coding
ENSSSCG00000035105	*MYL1* $^{+}$ Myoblasts	-1.58	5.75	0.00337	ENSSSCG00000035105	protein coding
MSTRG.31110	*MYL1* $^{+}$ Myoblasts	-1.58	5.84	0.00121	MSTRG.31110	lncRNA
MSTRG.6897	*MYL1* $^{+}$ Myoblasts	-1.58	5.05	0.0218	MSTRG.6897	lncRNA
TPM3	*MYL1* $^{+}$ Myoblasts	-1.58	7.9	0.0204	ENSSSCG00000006556	protein coding
MSTRG.17458	*MYL1* $^{+}$ Myoblasts	-1.56	6.06	0.0391	MSTRG.17458	lncRNA
MSTRG.14079	*MYL1* $^{+}$ Myoblasts	-1.54	4.75	0.0374	MSTRG.14079	lncRNA
PDE4D	*MYL1* $^{+}$ Myoblasts	-1.52	7.36	0.00642	ENSSSCG00000016929	protein coding
CCDC60	*MYL1* $^{+}$ Myoblasts	-1.51	5.86	0.00758	ENSSSCG00000009842	protein coding
MYOT	*MYL1* $^{+}$ Myoblasts	-1.5	7.45	0.0182	ENSSSCG00000014324	protein coding
PHYH	*MYL1* $^{+}$ Myoblasts	-1.5	6.45	0.00662	ENSSSCG00000039244	protein coding
AANAT	*MYL1* $^{+}$ Myoblasts	-1.49	5.42	0.0065	ENSSSCG00000017176	protein coding
PPARGC1A	*MYL1* $^{+}$ Myoblasts	-1.49	6.01	0.0159	ENSSSCG00000029275	protein coding
MSTRG.9438	*MYL1* $^{+}$ Myoblasts	-1.48	4.82	0.0411	MSTRG.9438	lncRNA
BABAM2	*MYL1* $^{+}$ Myoblasts	-1.46	5.05	0.0247	ENSSSCG00000026465	protein coding
MSTRG.19743	*MYL1* $^{+}$ Myoblasts	-1.44	6.43	0.00345	MSTRG.19743	lncRNA
ZNF462	*MYL1* $^{+}$ Myoblasts	-1.44	5.38	0.0193	ENSSSCG00000031592	protein coding
FLYWCH2	*MYL1* $^{+}$ Myoblasts	-1.42	5.03	0.0214	ENSSSCG00000022630	protein coding
ARRDC2	*MYL1* $^{+}$ Myoblasts	-1.41	5.95	0.00662	ENSSSCG00000013893	protein coding
ENSSSCG00000040885	*MYL1* $^{+}$ Myoblasts	-1.41	5.29	0.0346	ENSSSCG00000040885	protein coding
CASQ1	*MYL1* $^{+}$ Myoblasts	-1.4	8.6	0.0134	ENSSSCG00000006390	protein coding
CDIN1	*MYL1* $^{+}$ Myoblasts	-1.39	5.43	0.02	ENSSSCG00000026900	protein coding

MSTRG.42783	*MYL1* + Myoblasts	-1.39	4.76	0.0459	MSTRG.42783	lncRNA
ABLIM2	*MYL1* + Myoblasts	-1.37	6.92	0.00125	ENSSSCG00000008721	protein coding
ASB8	*MYL1* + Myoblasts	-1.37	4.93	0.0472	ENSSSCG00000021024	protein coding
GREB1L	*MYL1* + Myoblasts	-1.37	5.08	0.0264	ENSSSCG00000003699	protein coding
MSTRG.28397	*MYL1* + Myoblasts	-1.37	4.94	0.0378	MSTRG.28397	lncRNA
MSTRG.9628	*MYL1* + Myoblasts	-1.36	6.58	0.00843	MSTRG.9628	lncRNA
PLEKHH3	*MYL1* + Myoblasts	-1.35	5.66	0.00597	ENSSSCG00000017391	protein coding
TSC22D1	*MYL1* + Myoblasts	-1.35	6.08	0.00503	ENSSSCG00000009422	protein coding
AMPD1	*MYL1* + Myoblasts	-1.34	7.05	0.0265	ENSSSCG00000006754	protein coding
MSTRG.24116	*MYL1* + Myoblasts	-1.34	6.3	0.00942	MSTRG.24116	lncRNA
USP39	*MYL1* + Myoblasts	-1.34	4.91	0.0355	ENSSSCG00000030435	protein coding
SEMA5A	*MYL1* + Myoblasts	-1.32	5.12	0.0319	ENSSSCG00000017095	protein coding
UQCC1	*MYL1* + Myoblasts	-1.31	6.07	0.0365	ENSSSCG00000038237	protein coding
CDC37L1	*MYL1* + Myoblasts	-1.3	5.59	0.02	ENSSSCG00000005219	protein coding
TBC1D1	*MYL1* + Myoblasts	-1.3	5.6	0.0112	ENSSSCG00000028983	protein coding
TAP1	*MYL1* + Myoblasts	-1.29	5.86	0.0035	ENSSSCG00000025618	protein coding
CASZ1	*MYL1* + Myoblasts	-1.27	6.17	0.0118	ENSSSCG00000003407	protein coding
CKM	*MYL1* + Myoblasts	-1.26	11.6	0.0312	ENSSSCG00000036132	protein coding
ADAMTS12	*MYL1* + Myoblasts	-1.25	5.61	0.0195	ENSSSCG00000016816	protein coding
RPP25L	*MYL1* + Myoblasts	-1.24	5.09	0.0302	ENSSSCG00000010973	protein coding
ZNF385D	*MYL1* + Myoblasts	-1.23	6.7	0.0123	ENSSSCG00000011208	protein coding
CEBPZ	*MYL1* + Myoblasts	-1.21	5.14	0.031	ENSSSCG00000008493	protein coding
DHDH	*MYL1* + Myoblasts	-1.21	5.64	0.00853	ENSSSCG00000003144	protein coding
FYCO1	*MYL1* + Myoblasts	-1.21	6.48	0.0218	ENSSSCG00000011317	protein coding
ENSSSCG00000001930	*MYL1* + Myoblasts	-1.2	9.17	0.0117	ENSSSCG00000001930	protein coding
PTCD3	*MYL1* + Myoblasts	-1.2	5.19	0.0273	ENSSSCG00000008225	protein coding
MSTRG.17518	*MYL1* + Myoblasts	-1.19	5.85	0.0192	MSTRG.17518	lncRNA
SYNPO2L	*MYL1* + Myoblasts	-1.18	6.43	0.00907	ENSSSCG00000010303	protein coding
MSTRG.3642	*MYL1* + Myoblasts	-1.17	5.78	0.00817	MSTRG.3642	lncRNA
ACSL1	*MYL1* + Myoblasts	-1.16	7.55	0.00663	ENSSSCG00000015784	protein coding
ATP2A1	*MYL1* + Myoblasts	-1.16	9.9	0.0175	ENSSSCG00000007805	protein coding
CUEDC1	*MYL1* + Myoblasts	-1.16	5.3	0.0304	ENSSSCG00000017624	protein coding
ENSSSCG00000039740	*MYL1* + Myoblasts	-1.16	5.37	0.04	ENSSSCG00000039740	protein coding
GAPDH	*MYL1* + Myoblasts	-1.16	11.2	0.0376	ENSSSCG00000000694	protein coding

MSTRG.42628	*MYL1* + Myoblasts	-1.14	6.95	0.005	MSTRG.42628	lncRNA
ZCCHC24	*MYL1* + Myoblasts	-1.14	5.29	0.0389	ENSSSCG00000033074	protein coding
ACOT4	*MYL1* + Myoblasts	-1.13	6.69	0.00331	ENSSSCG00000002349	protein coding
PKNOX2	*MYL1* + Myoblasts	-1.13	5.85	0.0135	ENSSSCG00000015211	protein coding
SLC25A20	*MYL1* + Myoblasts	-1.13	5.39	0.035	ENSSSCG00000011366	protein coding
C16orf91	*MYL1* + Myoblasts	-1.12	5.68	0.028	ENSSSCG00000038882	protein coding
CEP95	*MYL1* + Myoblasts	-1.12	5.22	0.0336	ENSSSCG00000028925	protein coding
DDX23	*MYL1* + Myoblasts	-1.12	5.16	0.0352	ENSSSCG00000000176	protein coding
DNAJC11	*MYL1* + Myoblasts	-1.12	5.33	0.0334	ENSSSCG00000003384	protein coding
EPHX1	*MYL1* + Myoblasts	-1.12	7.43	0.00465	ENSSSCG00000010853	protein coding
ENSSSCG00000026816	*MYL1* + Myoblasts	-1.11	6.79	0.0173	ENSSSCG00000026816	protein coding
MBNL1	*MYL1* + Myoblasts	-1.11	7.66	0.0262	ENSSSCG00000011720	protein coding
MGARP	*MYL1* + Myoblasts	-1.11	5.96	0.0458	ENSSSCG00000009062	protein coding
MSTRG.38699	*MYL1* + Myoblasts	-1.11	5.21	0.0356	MSTRG.38699	lncRNA
RCAN2	*MYL1* + Myoblasts	-1.11	6.22	0.0217	ENSSSCG00000001716	protein coding
TPK1	*MYL1* + Myoblasts	-1.11	6.15	0.0472	ENSSSCG00000015462	protein coding
VLDLR	*MYL1* + Myoblasts	-1.1	5.31	0.0372	ENSSSCG00000005229	protein coding
FBL	*MYL1* + Myoblasts	-1.09	5.66	0.017	ENSSSCG00000030510	protein coding
ANGPTL4	*MYL1* + Myoblasts	-1.08	7.64	0.0243	ENSSSCG00000013599	protein coding
ASXL2	*MYL1* + Myoblasts	-1.08	5.62	0.0197	ENSSSCG00000008575	protein coding
CEBPD	*MYL1* + Myoblasts	-1.08	9.15	0.0494	ENSSSCG00000032367	protein coding
SRL	*MYL1* + Myoblasts	-1.08	8.33	0.0156	ENSSSCG00000007949	protein coding
TXNIP	*MYL1* + Myoblasts	-1.07	9.63	0.00167	ENSSSCG00000031262	protein coding
PDK4	*MYL1* + Myoblasts	-1.06	9.58	0.00246	ENSSSCG00000015334	protein coding
RRAGA	*MYL1* + Myoblasts	-1.06	5.64	0.0221	ENSSSCG00000005174	protein coding
CDC42EP3	*MYL1* + Myoblasts	-1.05	5.47	0.0345	ENSSSCG00000021359	protein coding
HJV	*MYL1* + Myoblasts	-1.05	6.73	0.0453	ENSSSCG00000035429	protein coding
H6PD	*MYL1* + Myoblasts	-1.01	6.13	0.044	ENSSSCG00000034502	protein coding
MYPN	*MYL1* + Myoblasts	-1.01	6.96	0.0493	ENSSSCG00000029311	protein coding
MLIP	*MYL1* + Myoblasts	-1	9.23	0.0218	ENSSSCG00000001485	protein coding
RPL3L	*MYL1* + Myoblasts	-1	6.67	0.0142	ENSSSCG00000032657	protein coding
SDF2	*MYL1* + Myoblasts	-0.995	5.69	0.0354	ENSSSCG00000017772	protein coding
TUBA4A	*MYL1* + Myoblasts	-0.989	8.78	0.0274	ENSSSCG00000016216	protein coding
PTDSS1	*MYL1* + Myoblasts	-0.978	5.64	0.0482	ENSSSCG00000006089	protein coding

UBAC2	*MYL1* $^+$ Myoblasts	-0.966	5.84	0.0179	ENSSSCG00000009516	protein coding
ENO1	*MYL1* $^+$ Myoblasts	-0.956	7.07	0.0142	ENSSSCG00000022343	protein coding
EYA1	*MYL1* $^+$ Myoblasts	-0.955	5.68	0.0303	ENSSSCG00000040728	protein coding
SDHC	*MYL1* $^+$ Myoblasts	-0.954	6.75	0.0205	ENSSSCG00000030318	protein coding
PDE4DIP	*MYL1* $^+$ Myoblasts	-0.951	8.84	0.0173	ENSSSCG00000024481	protein coding
MT3	*MYL1* $^+$ Myoblasts	-0.949	6.11	0.0275	ENSSSCG00000024305	protein coding
TRIP12	*MYL1* $^+$ Myoblasts	-0.943	5.5	0.0374	ENSSSCG00000024373	protein coding
DDIT4	*MYL1* $^+$ Myoblasts	-0.941	7.64	0.00656	ENSSSCG00000031888	protein coding
ENSSSCG00000036052	*MYL1* $^+$ Myoblasts	-0.936	7.88	0.0202	ENSSSCG00000036052	protein coding
ENSSSCG00000017791	*MYL1* $^+$ Myoblasts	-0.929	6.21	0.0337	ENSSSCG00000017791	protein coding
ENSSSCG00000031152	*MYL1* $^+$ Myoblasts	-0.923	6.7	0.0199	ENSSSCG00000031152	protein coding
GTF2I	*MYL1* $^+$ Myoblasts	-0.912	5.73	0.0327	ENSSSCG00000007721	protein coding
ENSSSCG00000003348	*MYL1* $^+$ Myoblasts	-0.91	6.13	0.0197	ENSSSCG00000003348	protein coding
SMARCA2	*MYL1* $^+$ Myoblasts	-0.909	6.7	0.0147	ENSSSCG00000005232	protein coding
NFYC	*MYL1* $^+$ Myoblasts	-0.904	6.45	0.0251	ENSSSCG00000003976	protein coding
BYSL	*MYL1* $^+$ Myoblasts	-0.895	5.64	0.049	ENSSSCG00000026752	protein coding
ASCC1	*MYL1* $^+$ Myoblasts	-0.893	6.23	0.0192	ENSSSCG00000023130	protein coding
FAM204A	*MYL1* $^+$ Myoblasts	-0.884	6.01	0.0397	ENSSSCG00000010674	protein coding
LIMCH1	*MYL1* $^+$ Myoblasts	-0.883	7.62	0.0169	ENSSSCG00000008799	protein coding
PIK3R1	*MYL1* $^+$ Myoblasts	-0.881	7.83	0.0195	ENSSSCG00000016958	protein coding
SH3KBP1	*MYL1* $^+$ Myoblasts	-0.877	6.09	0.0408	ENSSSCG00000012160	protein coding
TRIM72	*MYL1* $^+$ Myoblasts	-0.875	7.02	0.013	ENSSSCG00000007757	protein coding
NDUFS2	*MYL1* $^+$ Myoblasts	-0.868	6.91	0.033	ENSSSCG00000006358	protein coding
MSTRG.12210	*MYL1* $^+$ Myoblasts	-0.861	5.72	0.0485	MSTRG.12210	lncRNA
TIMM17A	*MYL1* $^+$ Myoblasts	-0.86	5.81	0.0436	ENSSSCG00000037431	protein coding
ALDH3B2	*MYL1* $^+$ Myoblasts	-0.857	6.81	0.0313	ENSSSCG00000026349	protein coding
MSTRG.14209	*MYL1* $^+$ Myoblasts	-0.853	6.19	0.039	MSTRG.14209	lncRNA
ENSSSCG00000000298	*MYL1* $^+$ Myoblasts	-0.847	7.31	0.0239	ENSSSCG00000000298	protein coding
SGCD	*MYL1* $^+$ Myoblasts	-0.835	9.17	0.0181	ENSSSCG00000017063	protein coding
ENSSSCG00000034314	*MYL1* $^+$ Myoblasts	-0.83	6.32	0.0245	ENSSSCG00000034314	protein coding
BFAR	*MYL1* $^+$ Myoblasts	-0.828	6.79	0.0291	ENSSSCG00000037673	protein coding
STAC3	*MYL1* $^+$ Myoblasts	-0.823	7.62	0.0466	ENSSSCG00000029471	protein coding
COQ4	*MYL1* $^+$ Myoblasts	-0.82	6.28	0.0411	ENSSSCG00000005646	protein coding
PLXNA2	*MYL1* $^+$ Myoblasts	-0.82	7.54	0.0274	ENSSSCG00000026412	protein coding

GYS1	*MYL1* + Myoblasts	-0.818	6.11	0.0421	ENSSSCG00000003154	protein coding
UQCRC1	*MYL1* + Myoblasts	-0.811	7.84	0.0319	ENSSSCG00000011356	protein coding
ATP6V0A1	*MYL1* + Myoblasts	-0.795	6.61	0.029	ENSSSCG00000017401	protein coding
APMAP	*MYL1* + Myoblasts	-0.787	6.41	0.0307	ENSSSCG00000036286	protein coding
MACROD1	*MYL1* + Myoblasts	-0.781	6.88	0.028	ENSSSCG00000013043	protein coding
MSTRG.45033	*MYL1* + Myoblasts	-0.763	9.5	0.0372	MSTRG.45033	lncRNA
SLC8A3	*MYL1* + Myoblasts	-0.758	8.57	0.0284	ENSSSCG00000002315	protein coding
PSMA5	*MYL1* + Myoblasts	-0.757	6.83	0.0259	ENSSSCG00000006830	protein coding
ZFP36	*MYL1* + Myoblasts	-0.757	9.24	0.0246	ENSSSCG00000028056	protein coding
ENSSSCG00000040571	*MYL1* + Myoblasts	-0.756	6.51	0.0396	ENSSSCG00000040571	protein coding
NDUFAB1	*MYL1* + Myoblasts	-0.754	8.13	0.0468	ENSSSCG00000010795	protein coding
SMYD3	*MYL1* + Myoblasts	-0.751	6.55	0.0354	ENSSSCG00000022034	protein coding
ATP5MC3	*MYL1* + Myoblasts	-0.75	8.81	0.0497	ENSSSCG00000015973	protein coding
MSTRG.48485	*MYL1* + Myoblasts	-0.747	6.14	0.045	MSTRG.48485	lncRNA
RBMS1	*MYL1* + Myoblasts	-0.741	6.76	0.0308	ENSSSCG00000015888	protein coding
SLMAP	*MYL1* + Myoblasts	-0.741	7.45	0.0498	ENSSSCG00000038873	protein coding
TCP1	*MYL1* + Myoblasts	-0.733	7.45	0.0331	ENSSSCG00000004048	protein coding
ENSSSCG00000024791	*MYL1* + Myoblasts	-0.724	7.34	0.031	ENSSSCG00000024791	protein coding
GGNBP2	*MYL1* + Myoblasts	-0.717	6.32	0.041	ENSSSCG00000017688	protein coding
ENSSSCG00000040854	*MYL1* + Myoblasts	-0.715	6.87	0.0424	ENSSSCG00000040854	protein coding
PPFIA4	*MYL1* + Myoblasts	-0.713	7.52	0.0392	ENSSSCG00000015474	protein coding
KCNN3	*MYL1* + Myoblasts	-0.711	7.97	0.0403	ENSSSCG00000006542	protein coding
GADD45G	*MYL1* + Myoblasts	-0.709	7.62	0.0285	ENSSSCG00000035249	protein coding
FKBP4	*MYL1* + Myoblasts	-0.708	7.34	0.0327	ENSSSCG00000000743	protein coding
OSBPL11	*MYL1* + Myoblasts	-0.704	7.33	0.0371	ENSSSCG00000026753	protein coding
USP2	*MYL1* + Myoblasts	-0.674	7.3	0.0336	ENSSSCG00000015120	protein coding
SELENOF	*MYL1* + Myoblasts	-0.659	7.45	0.0491	ENSSSCG00000030125	protein coding
THSD4	*MYL1* + Myoblasts	-0.652	7.55	0.0474	ENSSSCG00000004980	protein coding
MSTRG.23946	*MYL1* + Myoblasts	0.48	16.4	0.0149	MSTRG.23946	lncRNA
PAIP2	*MYL1* + Myoblasts	0.65	7.77	0.0464	ENSSSCG00000026606	protein coding
MSTRG.161	*MYL1* + Myoblasts	0.669	7.19	0.0432	MSTRG.161	lncRNA
S100A11	*MYL1* + Myoblasts	0.669	8.49	0.0476	ENSSSCG00000006610	protein coding
THRAP3	*MYL1* + Myoblasts	0.67	7.43	0.04	ENSSSCG00000029954	protein coding
KMT5B	*MYL1* + Myoblasts	0.692	7.06	0.0487	ENSSSCG00000031459	protein coding

ACTR2	*MYL1* $^{+}$ Myoblasts	0.704	6.47	0.0443	ENSSSCG00000008358	protein coding
DSTN	*MYL1* $^{+}$ Myoblasts	0.714	7.41	0.0378	ENSSSCG00000007085	protein coding
CRK	*MYL1* $^{+}$ Myoblasts	0.715	9.38	0.042	ENSSSCG00000038989	protein coding
LDLRAD3	*MYL1* $^{+}$ Myoblasts	0.741	7.19	0.0487	ENSSSCG00000013294	protein coding
PGRMC1	*MYL1* $^{+}$ Myoblasts	0.745	6.44	0.0357	ENSSSCG00000012625	protein coding
MSTRG.39186	*MYL1* $^{+}$ Myoblasts	0.76	8.18	0.0255	MSTRG.39186	lncRNA
BMP7	*MYL1* $^{+}$ Myoblasts	0.763	6.06	0.0479	ENSSSCG00000007501	protein coding
SERTAD2	*MYL1* $^{+}$ Myoblasts	0.766	6.36	0.0473	ENSSSCG00000037002	protein coding
HIVEP2	*MYL1* $^{+}$ Myoblasts	0.767	8.44	0.0197	ENSSSCG00000004138	protein coding
IL34	*MYL1* $^{+}$ Myoblasts	0.774	7.36	0.0491	ENSSSCG00000040629	protein coding
POSTN	*MYL1* $^{+}$ Myoblasts	0.774	7.29	0.0434	ENSSSCG00000009361	protein coding
IER3	*MYL1* $^{+}$ Myoblasts	0.784	8.07	0.0214	ENSSSCG00000027607	protein coding
CAST	*MYL1* $^{+}$ Myoblasts	0.799	6.37	0.0431	ENSSSCG00000014170	protein coding
RNF144B	*MYL1* $^{+}$ Myoblasts	0.808	7.14	0.0438	ENSSSCG00000001076	protein coding
NKAIN2	*MYL1* $^{+}$ Myoblasts	0.809	7.99	0.0475	ENSSSCG00000036607	protein coding
KDM2A	*MYL1* $^{+}$ Myoblasts	0.81	6.84	0.0311	ENSSSCG00000029637	protein coding
ORC4	*MYL1* $^{+}$ Myoblasts	0.817	6.03	0.037	ENSSSCG00000015668	protein coding
HSPH1	*MYL1* $^{+}$ Myoblasts	0.823	6.08	0.039	ENSSSCG00000009334	protein coding
TMSB10	*MYL1* $^{+}$ Myoblasts	0.834	11.7	0.0355	ENSSSCG00000008245	protein coding
MSTRG.22101	*MYL1* $^{+}$ Myoblasts	0.84	6.89	0.0298	MSTRG.22101	lncRNA
ARF5	*MYL1* $^{+}$ Myoblasts	0.842	6.14	0.0298	ENSSSCG00000016593	protein coding
ABTB2	*MYL1* $^{+}$ Myoblasts	0.858	7	0.0215	ENSSSCG00000013303	protein coding
SNX4	*MYL1* $^{+}$ Myoblasts	0.86	5.98	0.0483	ENSSSCG00000036828	protein coding
PDE5A	*MYL1* $^{+}$ Myoblasts	0.862	5.83	0.0393	ENSSSCG00000009106	protein coding
WASF2	*MYL1* $^{+}$ Myoblasts	0.863	6.5	0.0463	ENSSSCG00000003577	protein coding
MSTRG.22894	*MYL1* $^{+}$ Myoblasts	0.87	5.91	0.045	MSTRG.22894	lncRNA
ADGRL2	*MYL1* $^{+}$ Myoblasts	0.876	5.9	0.0442	ENSSSCG00000003761	protein coding
ZFP36L1	*MYL1* $^{+}$ Myoblasts	0.879	7.21	0.0134	ENSSSCG00000028304	protein coding
UBAP2L	*MYL1* $^{+}$ Myoblasts	0.882	6.47	0.0183	ENSSSCG00000006553	protein coding
TPD52	*MYL1* $^{+}$ Myoblasts	0.887	7.91	0.0406	ENSSSCG00000006156	protein coding
CETN3	*MYL1* $^{+}$ Myoblasts	0.889	5.87	0.0384	ENSSSCG00000014151	protein coding
SELENOP	*MYL1* $^{+}$ Myoblasts	0.89	7.88	0.0215	ENSSSCG00000021208	protein coding
MLLT3	*MYL1* $^{+}$ Myoblasts	0.891	7.19	0.0272	ENSSSCG00000005166	protein coding
CLIC4	*MYL1* $^{+}$ Myoblasts	0.896	9.14	0.0261	ENSSSCG00000038994	protein coding

NAPA	*MYL1* + Myoblasts	0.916	6.61	0.0163	ENSSSCG00000003120	protein coding
YPEL5	*MYL1* + Myoblasts	0.921	5.97	0.0337	ENSSSCG00000008529	protein coding
MSH4	*MYL1* + Myoblasts	0.929	6.29	0.0253	ENSSSCG00000003775	protein coding
MSTRG.1412	*MYL1* + Myoblasts	0.933	7.42	0.0193	MSTRG.1412	lncRNA
LRIG2	*MYL1* + Myoblasts	0.94	5.55	0.0443	ENSSSCG00000034581	protein coding
TMEM41B	*MYL1* + Myoblasts	0.945	6.16	0.0208	ENSSSCG00000014579	protein coding
MSTRG.3973	*MYL1* + Myoblasts	0.951	7.32	0.00985	MSTRG.3973	lncRNA
SCLY	*MYL1* + Myoblasts	0.96	6.32	0.017	ENSSSCG00000016334	protein coding
CRIM1	*MYL1* + Myoblasts	0.971	6.68	0.0293	ENSSSCG00000008504	protein coding
CBX3	*MYL1* + Myoblasts	0.975	6.61	0.0192	ENSSSCG00000016711	protein coding
CYP7B1	*MYL1* + Myoblasts	0.976	8.01	0.0349	ENSSSCG00000022092	protein coding
RSF1	*MYL1* + Myoblasts	0.976	6.47	0.0183	ENSSSCG00000014882	protein coding
ENSSSCG00000035293	*MYL1* + Myoblasts	0.983	10.8	0.0265	ENSSSCG00000035293	protein coding
TNNT2	*MYL1* + Myoblasts	0.983	9.95	0.00612	ENSSSCG00000023031	protein coding
CLPX	*MYL1* + Myoblasts	0.986	6.22	0.0219	ENSSSCG00000004927	protein coding
NR4A1	*MYL1* + Myoblasts	0.988	8.11	0.0118	ENSSSCG00000031321	protein coding
RND3	*MYL1* + Myoblasts	0.99	6.62	0.0257	ENSSSCG00000040445	protein coding
GYG2	*MYL1* + Myoblasts	0.991	7.75	0.0243	ENSSSCG00000038044	protein coding
HDAC9	*MYL1* + Myoblasts	0.993	7	0.0286	ENSSSCG00000015368	protein coding
ARL15	*MYL1* + Myoblasts	0.996	6.46	0.0357	ENSSSCG00000016894	protein coding
PCCB	*MYL1* + Myoblasts	0.998	5.53	0.0314	ENSSSCG00000011653	protein coding
OSER1	*MYL1* + Myoblasts	1.01	6.54	0.0231	ENSSSCG00000022714	protein coding
GALK2	*MYL1* + Myoblasts	1.04	7.31	0.016	ENSSSCG00000004651	protein coding
MED13	*MYL1* + Myoblasts	1.05	5.94	0.0291	ENSSSCG00000017672	protein coding
MYO10	*MYL1* + Myoblasts	1.06	6.59	0.0256	ENSSSCG00000016794	protein coding
SPART	*MYL1* + Myoblasts	1.06	5.66	0.0438	ENSSSCG00000024635	protein coding
MSTRG.20986	*MYL1* + Myoblasts	1.07	6.82	0.0115	MSTRG.20986	lncRNA
HBEGF	*MYL1* + Myoblasts	1.09	6.91	0.0358	ENSSSCG00000014362	protein coding
GALP	*MYL1* + Myoblasts	1.1	6.17	0.0119	ENSSSCG00000003318	protein coding
PCDH9	*MYL1* + Myoblasts	1.1	5.63	0.0201	ENSSSCG00000038842	protein coding
MSTRG.26543	*MYL1* + Myoblasts	1.12	5.6	0.0394	MSTRG.26543	lncRNA
MSTRG.42550	*MYL1* + Myoblasts	1.14	6.06	0.00922	MSTRG.42550	lncRNA
DPYD	*MYL1* + Myoblasts	1.15	6.42	0.0149	ENSSSCG00000006878	protein coding
LRCH1	*MYL1* + Myoblasts	1.15	6.86	0.007	ENSSSCG00000009408	protein coding

DENND4A	*MYL1* $^+$ Myoblasts	1.16	6.4	0.0188	ENSSSCG00000004938	protein coding
SELENOW	*MYL1* $^+$ Myoblasts	1.16	7.94	0.00989	ENSSSCG00000034313	protein coding
KHDRBS3	*MYL1* $^+$ Myoblasts	1.17	6.12	0.0189	ENSSSCG00000005941	protein coding
BAIAP2L2	*MYL1* $^+$ Myoblasts	1.19	6.22	0.0267	ENSSSCG00000000111	protein coding
ITGA4	*MYL1* $^+$ Myoblasts	1.19	6.59	0.0264	ENSSSCG00000016009	protein coding
RICTOR	*MYL1* $^+$ Myoblasts	1.19	6.35	0.00634	ENSSSCG00000016853	protein coding
OXCT1	*MYL1* $^+$ Myoblasts	1.2	5.58	0.0371	ENSSSCG00000016863	protein coding
PLPPR1	*MYL1* $^+$ Myoblasts	1.2	6.53	0.0092	ENSSSCG00000005393	protein coding
ERBB4	*MYL1* $^+$ Myoblasts	1.21	6.07	0.0256	ENSSSCG00000016160	protein coding
FYN	*MYL1* $^+$ Myoblasts	1.21	5.72	0.0391	ENSSSCG00000004421	protein coding
BCAS3	*MYL1* $^+$ Myoblasts	1.23	6.63	0.0196	ENSSSCG00000017676	protein coding
ZSWIM6	*MYL1* $^+$ Myoblasts	1.23	7.13	0.0044	ENSSSCG00000025194	protein coding
DNAJB1	*MYL1* $^+$ Myoblasts	1.24	7.69	0.00319	ENSSSCG00000013784	protein coding
GNAI1	*MYL1* $^+$ Myoblasts	1.24	5.07	0.0427	ENSSSCG00000015407	protein coding
NEDD4L	*MYL1* $^+$ Myoblasts	1.25	5.77	0.00825	ENSSSCG00000004919	protein coding
MSTRG.43155	*MYL1* $^+$ Myoblasts	1.26	5.94	0.0324	MSTRG.43155	lncRNA
TBC1D17	*MYL1* $^+$ Myoblasts	1.27	5.46	0.0328	ENSSSCG00000003193	protein coding
ENSSSCG00000033566	*MYL1* $^+$ Myoblasts	1.28	5.35	0.0286	ENSSSCG00000033566	protein coding
GABPB1	*MYL1* $^+$ Myoblasts	1.28	5.43	0.0149	ENSSSCG00000004645	protein coding
RUSC2	*MYL1* $^+$ Myoblasts	1.29	5.49	0.0263	ENSSSCG00000005308	protein coding
SINHCAF	*MYL1* $^+$ Myoblasts	1.29	5.3	0.0391	ENSSSCG00000037732	protein coding
AP1S2	*MYL1* $^+$ Myoblasts	1.3	5.3	0.0189	ENSSSCG00000036074	protein coding
MSTRG.10825	*MYL1* $^+$ Myoblasts	1.3	5.89	0.00548	MSTRG.10825	lncRNA
KDM1A	*MYL1* $^+$ Myoblasts	1.31	5.36	0.0176	ENSSSCG00000003531	protein coding
LBR	*MYL1* $^+$ Myoblasts	1.31	5.07	0.0379	ENSSSCG00000032397	protein coding
DNAJC10	*MYL1* $^+$ Myoblasts	1.32	5.21	0.0232	ENSSSCG00000016017	protein coding
PELI2	*MYL1* $^+$ Myoblasts	1.33	7.58	0.0126	ENSSSCG00000033800	protein coding
UHRF2	*MYL1* $^+$ Myoblasts	1.36	5.38	0.0206	ENSSSCG00000005201	protein coding
ADD1	*MYL1* $^+$ Myoblasts	1.38	6.08	0.0123	ENSSSCG00000008692	protein coding
MSTRG.14609	*MYL1* $^+$ Myoblasts	1.38	5.69	0.0162	MSTRG.14609	lncRNA
MSTRG.29048	*MYL1* $^+$ Myoblasts	1.38	5.48	0.0194	MSTRG.29048	lncRNA
OSR2	*MYL1* $^+$ Myoblasts	1.38	5.61	0.0102	ENSSSCG00000006073	protein coding
LITAF	*MYL1* $^+$ Myoblasts	1.39	7.02	0.002	ENSSSCG00000032381	protein coding
STXBP5	*MYL1* $^+$ Myoblasts	1.39	5.48	0.015	ENSSSCG00000004113	protein coding

MSTRG.20753	*MYL1* + Myoblasts	1.4	5.07	0.0278	MSTRG.20753	lncRNA
NRAS	*MYL1* + Myoblasts	1.4	6.09	0.00261	ENSSSCG00000032477	protein coding
MSTRG.3966	*MYL1* + Myoblasts	1.41	7.04	0.008	MSTRG.3966	lncRNA
ENSSSCG00000028481	*MYL1* + Myoblasts	1.42	5.67	0.0388	ENSSSCG00000028481	protein coding
SMURF1	*MYL1* + Myoblasts	1.43	4.97	0.0398	ENSSSCG00000030153	protein coding
SPATS2	*MYL1* + Myoblasts	1.45	4.96	0.045	ENSSSCG00000000199	protein coding
PCDH7	*MYL1* + Myoblasts	1.47	8.23	0.00446	ENSSSCG00000008765	protein coding
LCA5	*MYL1* + Myoblasts	1.48	5.59	0.022	ENSSSCG00000004469	protein coding
MSTRG.11272	*MYL1* + Myoblasts	1.49	5.5	0.0448	MSTRG.11272	lncRNA
AHCYL2	*MYL1* + Myoblasts	1.52	5.65	0.00975	ENSSSCG00000016568	protein coding
ECHDC1	*MYL1* + Myoblasts	1.53	5.04	0.0253	ENSSSCG00000004216	protein coding
SELENOM	*MYL1* + Myoblasts	1.54	5.88	0.0026	ENSSSCG00000029781	protein coding
MSTRG.41682	*MYL1* + Myoblasts	1.55	6.4	0.0122	MSTRG.41682	lncRNA
MSTRG.32912	*MYL1* + Myoblasts	1.57	5.86	0.00467	MSTRG.32912	lncRNA
MSTRG.16378	*MYL1* + Myoblasts	1.58	5.33	0.0201	MSTRG.16378	lncRNA
MSTRG.31494	*MYL1* + Myoblasts	1.59	4.91	0.0325	MSTRG.31494	lncRNA
NAB2	*MYL1* + Myoblasts	1.6	5.68	0.0233	ENSSSCG00000000411	protein coding
PCCA	*MYL1* + Myoblasts	1.61	5.53	0.00612	ENSSSCG00000009522	protein coding
RASGEF1B	*MYL1* + Myoblasts	1.61	5.81	0.00234	ENSSSCG00000029507	protein coding
MSTRG.34696	*MYL1* + Myoblasts	1.62	5	0.0254	MSTRG.34696	lncRNA
TSPO	*MYL1* + Myoblasts	1.63	5.29	0.00744	ENSSSCG00000000033	protein coding
ENSSSCG00000029160	*MYL1* + Myoblasts	1.64	9.65	0.00122	ENSSSCG00000029160	protein coding
ENSSSCG00000017446	*MYL1* + Myoblasts	1.66	6.48	0.00573	ENSSSCG00000017446	protein coding
MSTRG.3599	*MYL1* + Myoblasts	1.66	5.17	0.0203	MSTRG.3599	lncRNA
GAB1	*MYL1* + Myoblasts	1.67	6.63	0.000782	ENSSSCG00000009048	protein coding
LARP6	*MYL1* + Myoblasts	1.68	4.92	0.0352	ENSSSCG00000004974	protein coding
ELL2	*MYL1* + Myoblasts	1.69	5.76	0.0322	ENSSSCG00000014168	protein coding
ENSSSCG00000036013	*MYL1* + Myoblasts	1.73	6.26	0.00232	ENSSSCG00000036013	protein coding
PAWR	*MYL1* + Myoblasts	1.73	5.76	0.00728	ENSSSCG00000036560	protein coding
HSPA13	*MYL1* + Myoblasts	1.74	4.94	0.0169	ENSSSCG00000012007	protein coding
MSTRG.11887	*MYL1* + Myoblasts	1.77	5.2	0.00619	MSTRG.11887	lncRNA
TP53BP1	*MYL1* + Myoblasts	1.77	4.91	0.022	ENSSSCG00000031949	protein coding
MSTRG.6594	*MYL1* + Myoblasts	1.78	5.07	0.0126	MSTRG.6594	lncRNA
SELENBP1	*MYL1* + Myoblasts	1.79	5.84	0.0149	ENSSSCG00000006624	protein coding

STMN1	*MYL1*$^{+}$ Myoblasts	1.79	5.62	0.00189	ENSSSCG00000026257	protein coding
DAB2	*MYL1*$^{+}$ Myoblasts	1.8	5.39	0.0267	ENSSSCG00000016857	protein coding
ID2	*MYL1*$^{+}$ Myoblasts	1.93	5.72	0.0148	ENSSSCG00000008645	protein coding
SLC10A1	*MYL1*$^{+}$ Myoblasts	1.97	5.32	0.0405	ENSSSCG00000002313	protein coding
MAGI3	*MYL1*$^{+}$ Myoblasts	2.04	5.99	0.000508	ENSSSCG00000006767	protein coding
MSTRG.26207	*MYL1*$^{+}$ Myoblasts	2.06	4.94	0.0344	MSTRG.26207	lncRNA
NDRG1	*MYL1*$^{+}$ Myoblasts	2.08	4.97	0.0104	ENSSSCG00000005944	protein coding
GAB2	*MYL1*$^{+}$ Myoblasts	2.16	5.91	0.00024	ENSSSCG00000014891	protein coding
WWC2	*MYL1*$^{+}$ Myoblasts	2.17	4.65	0.0247	ENSSSCG00000021731	protein coding
RTKN2	*MYL1*$^{+}$ Myoblasts	2.38	5.03	0.00248	ENSSSCG00000039707	protein coding
MSTRG.25664	*MYL1*$^{+}$ Myoblasts	2.41	5.91	0.00843	MSTRG.25664	lncRNA
MSTRG.34841	*MYL1*$^{+}$ Myoblasts	2.42	5.24	0.019	MSTRG.34841	lncRNA
MSTRG.38853	*MYL1*$^{+}$ Myoblasts	2.53	7.51	0.0479	MSTRG.38853	lncRNA
REPS1	*MYL1*$^{+}$ Myoblasts	2.71	6.79	0.0236	ENSSSCG00000004146	protein coding
MIPOL1	*MYL1*$^{+}$ Myoblasts	2.86	4.91	0.00198	ENSSSCG00000039714	protein coding
APOA1	*MYL1*$^{+}$ Myoblasts	3.09	4.75	0.00646	ENSSSCG00000030921	protein coding
MSTRG.22838	*MYMK*$^{+}$ Myoblasts	-5.21	6.11	2.28E-05	MSTRG.22838	lncRNA
PROSER2	*MYMK*$^{+}$ Myoblasts	-3.41	5.17	1.11E-05	ENSSSCG00000022247	protein coding
TNNT1	*MYMK*$^{+}$ Myoblasts	-3.3	7.59	1.60E-05	ENSSSCG00000025353	protein coding
MSTRG.39368	*MYMK*$^{+}$ Myoblasts	-3.03	5.07	0.00041	MSTRG.39368	lncRNA
ENSSSCG00000015632	*MYMK*$^{+}$ Myoblasts	-2.7	5.29	0.000234	ENSSSCG00000015632	protein coding
YIPF1	*MYMK*$^{+}$ Myoblasts	-2.29	6.03	0.00149	ENSSSCG00000028506	protein coding
MSTRG.4840	*MYMK*$^{+}$ Myoblasts	-2.21	5.43	0.000693	MSTRG.4840	lncRNA
ARPP21	*MYMK*$^{+}$ Myoblasts	-1.99	5.53	0.000261	ENSSSCG00000011238	protein coding
MYL2	*MYMK*$^{+}$ Myoblasts	-1.99	6.61	0.034	ENSSSCG00000039710	protein coding
TNNT2	*MYMK*$^{+}$ Myoblasts	-1.82	6.19	0.00254	ENSSSCG00000023031	protein coding
MSTRG.10446	*MYMK*$^{+}$ Myoblasts	-1.8	5.17	0.00313	MSTRG.10446	lncRNA
BTBD11	*MYMK*$^{+}$ Myoblasts	-1.76	4.76	0.00446	ENSSSCG00000000162	protein coding
ZBTB16	*MYMK*$^{+}$ Myoblasts	-1.73	7.67	0.000887	ENSSSCG00000030095	protein coding
EDA	*MYMK*$^{+}$ Myoblasts	-1.71	4.99	0.00894	ENSSSCG00000021647	protein coding
MSTRG.29775	*MYMK*$^{+}$ Myoblasts	-1.67	5.03	0.00181	MSTRG.29775	lncRNA
LMOD2	*MYMK*$^{+}$ Myoblasts	-1.65	5.77	0.00496	ENSSSCG00000016605	protein coding
MEF2C	*MYMK*$^{+}$ Myoblasts	-1.64	5.31	0.00715	ENSSSCG00000014149	protein coding
DUSP4	*MYMK*$^{+}$ Myoblasts	-1.63	6.64	0.000532	ENSSSCG00000015850	protein coding

ORC3	*MYMK*$^+$ Myoblasts	-1.6	4.77	0.00812	ENSSSCG00000004306	protein coding
ENSSSCG00000036052	*MYMK*$^+$ Myoblasts	-1.57	5.09	0.00989	ENSSSCG00000036052	protein coding
PDE1C	*MYMK*$^+$ Myoblasts	-1.52	6.23	0.0341	ENSSSCG00000016668	protein coding
QTRT1	*MYMK*$^+$ Myoblasts	-1.5	5.22	0.0152	ENSSSCG00000013637	protein coding
MTR	*MYMK*$^+$ Myoblasts	-1.49	5.07	0.00447	ENSSSCG00000010143	protein coding
OTUD4	*MYMK*$^+$ Myoblasts	-1.46	5.04	0.0405	ENSSSCG00000009042	protein coding
MCFD2	*MYMK*$^+$ Myoblasts	-1.45	5.84	0.0127	ENSSSCG00000021325	protein coding
MSTRG.30004	*MYMK*$^+$ Myoblasts	-1.42	5.13	0.0158	MSTRG.30004	lncRNA
LPGAT1	*MYMK*$^+$ Myoblasts	-1.41	6.94	0.00654	ENSSSCG00000015603	protein coding
PRTG	*MYMK*$^+$ Myoblasts	-1.41	5.94	0.032	ENSSSCG00000035798	protein coding
ENSSSCG00000036224	*MYMK*$^+$ Myoblasts	-1.4	5.64	0.033	ENSSSCG00000036224	protein coding
HES6	*MYMK*$^+$ Myoblasts	-1.4	6.33	0.00653	ENSSSCG00000016342	protein coding
EIF4G1	*MYMK*$^+$ Myoblasts	-1.38	5.16	0.00392	ENSSSCG00000030255	protein coding
SLC3A2	*MYMK*$^+$ Myoblasts	-1.36	5.66	0.00582	ENSSSCG00000022404	protein coding
FKBP5	*MYMK*$^+$ Myoblasts	-1.35	7.87	0.00222	ENSSSCG00000001549	protein coding
MYO18B	*MYMK*$^+$ Myoblasts	-1.34	6.72	0.00509	ENSSSCG00000009957	protein coding
FOXO6	*MYMK*$^+$ Myoblasts	-1.33	5.43	0.00623	ENSSSCG00000035074	protein coding
TM7SF2	*MYMK*$^+$ Myoblasts	-1.33	5.79	0.00504	ENSSSCG00000013004	protein coding
BCAM	*MYMK*$^+$ Myoblasts	-1.32	6.46	0.00133	ENSSSCG00000020657	protein coding
MAP1A	*MYMK*$^+$ Myoblasts	-1.3	6.21	0.00605	ENSSSCG00000004705	protein coding
RETREG3	*MYMK*$^+$ Myoblasts	-1.29	4.75	0.0244	ENSSSCG00000017395	protein coding
KLF2	*MYMK*$^+$ Myoblasts	-1.28	8.14	0.0146	ENSSSCG00000038911	protein coding
LIMS2	*MYMK*$^+$ Myoblasts	-1.28	5.3	0.0274	ENSSSCG00000015862	protein coding
ELAC2	*MYMK*$^+$ Myoblasts	-1.26	5.04	0.0453	ENSSSCG00000028465	protein coding
TUBA4A	*MYMK*$^+$ Myoblasts	-1.26	5.37	0.0332	ENSSSCG00000016216	protein coding
PRKCA	*MYMK*$^+$ Myoblasts	-1.25	5.51	0.0101	ENSSSCG00000017268	protein coding
ATP6V0D1	*MYMK*$^+$ Myoblasts	-1.23	6.4	0.00577	ENSSSCG00000002776	protein coding
MSTRG.20970	*MYMK*$^+$ Myoblasts	-1.23	5.07	0.0127	MSTRG.20970	lncRNA
ENSSSCG00000017165	*MYMK*$^+$ Myoblasts	-1.22	5.1	0.037	ENSSSCG00000017165	protein coding
MGARP	*MYMK*$^+$ Myoblasts	-1.22	6.33	0.00545	ENSSSCG00000009062	protein coding
CPT1B	*MYMK*$^+$ Myoblasts	-1.21	6.17	0.0215	ENSSSCG00000022364	protein coding
MEF2D	*MYMK*$^+$ Myoblasts	-1.21	5.14	0.024	ENSSSCG00000006482	protein coding
MFAP5	*MYMK*$^+$ Myoblasts	-1.2	7.68	0.00197	ENSSSCG00000031712	protein coding
POFUT1	*MYMK*$^+$ Myoblasts	-1.2	5.22	0.0122	ENSSSCG00000007244	protein coding

SEC14L1	*MYMK*$^{+}$	Myoblasts	-1.19	5.39	0.00688	ENSSSCG00000017169	protein coding
SORD	*MYMK*$^{+}$	Myoblasts	-1.19	5.64	0.00861	ENSSSCG00000004679	protein coding
MRPL34	*MYMK*$^{+}$	Myoblasts	-1.17	5.17	0.0346	ENSSSCG00000039295	protein coding
MSTRG.32618	*MYMK*$^{+}$	Myoblasts	-1.17	5.44	0.0172	MSTRG.32618	lncRNA
GLI2	*MYMK*$^{+}$	Myoblasts	-1.15	7.6	0.00214	ENSSSCG00000015733	protein coding
MSTRG.6686	*MYMK*$^{+}$	Myoblasts	-1.14	5.06	0.0249	MSTRG.6686	lncRNA
PRKAR1B	*MYMK*$^{+}$	Myoblasts	-1.14	5.83	0.0105	ENSSSCG00000007542	protein coding
NMT1	*MYMK*$^{+}$	Myoblasts	-1.13	5.52	0.0084	ENSSSCG00000017339	protein coding
ENSSSCG00000000145	*MYMK*$^{+}$	Myoblasts	-1.1	5.09	0.037	ENSSSCG00000000145	protein coding
MSTRG.30567	*MYMK*$^{+}$	Myoblasts	-1.1	5.72	0.0459	MSTRG.30567	lncRNA
C16orf91	*MYMK*$^{+}$	Myoblasts	-1.08	5.65	0.0109	ENSSSCG00000038882	protein coding
CBFA2T2	*MYMK*$^{+}$	Myoblasts	-1.08	5.72	0.0333	ENSSSCG00000027410	protein coding
ENSSSCG00000039740	*MYMK*$^{+}$	Myoblasts	-1.07	5.88	0.0398	ENSSSCG00000039740	protein coding
MKKS	*MYMK*$^{+}$	Myoblasts	-1.07	4.9	0.0406	ENSSSCG00000007064	protein coding
AGPAT3	*MYMK*$^{+}$	Myoblasts	-1.06	5.44	0.0292	ENSSSCG00000038081	protein coding
RRP9	*MYMK*$^{+}$	Myoblasts	-1.06	5.29	0.0252	ENSSSCG00000023177	protein coding
HECTD3	*MYMK*$^{+}$	Myoblasts	-1.05	4.82	0.0412	ENSSSCG00000003923	protein coding
MRPS18C	*MYMK*$^{+}$	Myoblasts	-1.05	5.8	0.011	ENSSSCG00000038042	protein coding
MTG1	*MYMK*$^{+}$	Myoblasts	-1.05	5.1	0.0225	ENSSSCG00000029088	protein coding
DUSP23	*MYMK*$^{+}$	Myoblasts	-1.04	5.25	0.0347	ENSSSCG00000033585	protein coding
SLC5A10	*MYMK*$^{+}$	Myoblasts	-1.04	6.44	0.00766	ENSSSCG00000018049	protein coding
SRL	*MYMK*$^{+}$	Myoblasts	-1.04	7.54	0.00862	ENSSSCG00000007949	protein coding
EXOSC4	*MYMK*$^{+}$	Myoblasts	-1.03	5.42	0.0276	ENSSSCG00000005925	protein coding
MYLPF	*MYMK*$^{+}$	Myoblasts	-1.03	10.2	0.00493	ENSSSCG00000007799	protein coding
AKR1B1	*MYMK*$^{+}$	Myoblasts	-1.02	9.65	0.00499	ENSSSCG00000040608	protein coding
DYSF	*MYMK*$^{+}$	Myoblasts	-1.02	6.2	0.0284	ENSSSCG00000008312	protein coding
FRAS1	*MYMK*$^{+}$	Myoblasts	-1.02	6.23	0.0377	ENSSSCG00000008991	protein coding
POLR3D	*MYMK*$^{+}$	Myoblasts	-1.02	5.63	0.0286	ENSSSCG00000009622	protein coding
RRAGD	*MYMK*$^{+}$	Myoblasts	-1.02	5.99	0.0152	ENSSSCG00000004318	protein coding
AGAP3	*MYMK*$^{+}$	Myoblasts	-1.01	5.66	0.0172	ENSSSCG00000025610	protein coding
ID1	*MYMK*$^{+}$	Myoblasts	-1.01	6.23	0.00836	ENSSSCG00000037016	protein coding
SLC20A2	*MYMK*$^{+}$	Myoblasts	-1	5.59	0.00995	ENSSSCG00000007027	protein coding
OSBPL11	*MYMK*$^{+}$	Myoblasts	-0.993	6.03	0.0206	ENSSSCG00000026753	protein coding
ENSSSCG00000000194	*MYMK*$^{+}$	Myoblasts	-0.991	5.73	0.0201	ENSSSCG00000000194	protein coding

RRP12	*MYMK*$^{+}$ Myoblasts	-0.99	4.95	0.0435	ENSSSCG00000010514	protein coding
STAC3	*MYMK*$^{+}$ Myoblasts	-0.989	6.55	0.0362	ENSSSCG00000029471	protein coding
PDCL3	*MYMK*$^{+}$ Myoblasts	-0.975	5.82	0.0079	ENSSSCG00000033885	protein coding
SMARCA4	*MYMK*$^{+}$ Myoblasts	-0.975	5.94	0.0302	ENSSSCG00000013629	protein coding
ENSSSCG00000025224	*MYMK*$^{+}$ Myoblasts	-0.973	6.63	0.0242	ENSSSCG00000025224	protein coding
ZCCHC24	*MYMK*$^{+}$ Myoblasts	-0.971	5.29	0.0207	ENSSSCG00000033074	protein coding
ACTC1	*MYMK*$^{+}$ Myoblasts	-0.967	12.1	9.66E-05	ENSSSCG00000004803	protein coding
CAMK2A	*MYMK*$^{+}$ Myoblasts	-0.967	6.27	0.0215	ENSSSCG00000014443	protein coding
SPECC1	*MYMK*$^{+}$ Myoblasts	-0.963	5.66	0.0214	ENSSSCG00000034364	protein coding
CCDC28B	*MYMK*$^{+}$ Myoblasts	-0.962	6	0.0176	ENSSSCG00000031514	protein coding
TTC7B	*MYMK*$^{+}$ Myoblasts	-0.961	6.74	0.00393	ENSSSCG00000002436	protein coding
CAVIN2	*MYMK*$^{+}$ Myoblasts	-0.948	6.24	0.0327	ENSSSCG00000026618	protein coding
ENSSSCG00000009543	*MYMK*$^{+}$ Myoblasts	-0.946	8.32	0.00596	ENSSSCG00000009543	protein coding
UCK2	*MYMK*$^{+}$ Myoblasts	-0.938	5.28	0.0463	ENSSSCG00000039867	protein coding
TEAD4	*MYMK*$^{+}$ Myoblasts	-0.935	6.8	0.00443	ENSSSCG00000000736	protein coding
GPATCH4	*MYMK*$^{+}$ Myoblasts	-0.932	5.81	0.0146	ENSSSCG00000006481	protein coding
ATP2A1	*MYMK*$^{+}$ Myoblasts	-0.927	8.45	0.0458	ENSSSCG00000007805	protein coding
CPT1A	*MYMK*$^{+}$ Myoblasts	-0.927	5.17	0.0338	ENSSSCG00000012880	protein coding
MSTRG.49624	*MYMK*$^{+}$ Myoblasts	-0.921	6.9	0.0404	MSTRG.49624	lncRNA
MSN	*MYMK*$^{+}$ Myoblasts	-0.919	5.92	0.0311	ENSSSCG00000012365	protein coding
AMBRA1	*MYMK*$^{+}$ Myoblasts	-0.916	5.78	0.0254	ENSSSCG00000013262	protein coding
JMJD6	*MYMK*$^{+}$ Myoblasts	-0.915	5.32	0.0495	ENSSSCG00000035923	protein coding
SLC25A4	*MYMK*$^{+}$ Myoblasts	-0.914	8.68	0.00238	ENSSSCG00000015790	protein coding
MARS1	*MYMK*$^{+}$ Myoblasts	-0.905	5.97	0.0199	ENSSSCG00000023318	protein coding
ACO2	*MYMK*$^{+}$ Myoblasts	-0.904	6.89	0.00924	ENSSSCG00000000064	protein coding
GSTO1	*MYMK*$^{+}$ Myoblasts	-0.904	6	0.0121	ENSSSCG00000022351	protein coding
PFN1	*MYMK*$^{+}$ Myoblasts	-0.904	9.86	0.00993	ENSSSCG00000017907	protein coding
POP4	*MYMK*$^{+}$ Myoblasts	-0.901	5.29	0.0487	ENSSSCG00000002854	protein coding
ARPC4	*MYMK*$^{+}$ Myoblasts	-0.884	7.58	0.00248	ENSSSCG00000011552	protein coding
FAM50A	*MYMK*$^{+}$ Myoblasts	-0.881	5.52	0.0483	ENSSSCG00000025303	protein coding
RSBN1	*MYMK*$^{+}$ Myoblasts	-0.881	5.55	0.0222	ENSSSCG00000030154	protein coding
SNRNP200	*MYMK*$^{+}$ Myoblasts	-0.877	5.45	0.0337	ENSSSCG00000008127	protein coding
PIK3R1	*MYMK*$^{+}$ Myoblasts	-0.87	7.44	0.00242	ENSSSCG00000016958	protein coding
NCL	*MYMK*$^{+}$ Myoblasts	-0.867	7.62	0.00256	ENSSSCG00000016275	protein coding

PPP1CA	*MYMK*$^{+}$ Myoblasts	-0.863	6.09	0.00947	ENSSSCG00000012913	protein coding
TOB2	*MYMK*$^{+}$ Myoblasts	-0.857	6.46	0.038	ENSSSCG00000037958	protein coding
GLCCI1	*MYMK*$^{+}$ Myoblasts	-0.854	7.92	0.0199	ENSSSCG00000015345	protein coding
RNF217	*MYMK*$^{+}$ Myoblasts	-0.853	6.63	0.00492	ENSSSCG00000021149	protein coding
MRPS23	*MYMK*$^{+}$ Myoblasts	-0.844	5.98	0.0206	ENSSSCG00000035193	protein coding
CHST7	*MYMK*$^{+}$ Myoblasts	-0.839	5.52	0.0329	ENSSSCG00000012265	protein coding
ANKRD10	*MYMK*$^{+}$ Myoblasts	-0.836	6.67	0.00985	ENSSSCG00000033732	protein coding
NFATC3	*MYMK*$^{+}$ Myoblasts	-0.835	6.71	0.018	ENSSSCG00000029578	protein coding
PSMB3	*MYMK*$^{+}$ Myoblasts	-0.828	6.99	0.0219	ENSSSCG00000037606	protein coding
ADI1	*MYMK*$^{+}$ Myoblasts	-0.827	6.46	0.0214	ENSSSCG00000029161	protein coding
ABHD16A	*MYMK*$^{+}$ Myoblasts	-0.826	6.03	0.0322	ENSSSCG00000034923	protein coding
KIAA0513	*MYMK*$^{+}$ Myoblasts	-0.825	7.42	0.0155	ENSSSCG00000002666	protein coding
ENSSSCG00000041267	*MYMK*$^{+}$ Myoblasts	-0.822	6.04	0.0383	ENSSSCG00000041267	lncRNA
MSTRG.30425	*MYMK*$^{+}$ Myoblasts	-0.82	7.16	0.0179	MSTRG.30425	lncRNA
TRMT6	*MYMK*$^{+}$ Myoblasts	-0.813	5.75	0.0337	ENSSSCG00000007046	protein coding
EXOC3	*MYMK*$^{+}$ Myoblasts	-0.804	5.51	0.0456	ENSSSCG00000039607	protein coding
HARS1	*MYMK*$^{+}$ Myoblasts	-0.804	5.36	0.041	ENSSSCG00000014375	protein coding
TSPAN15	*MYMK*$^{+}$ Myoblasts	-0.801	8.95	0.0046	ENSSSCG00000036152	protein coding
ANKS1A	*MYMK*$^{+}$ Myoblasts	-0.797	5.91	0.0414	ENSSSCG00000001534	protein coding
ENSSSCG00000031903	*MYMK*$^{+}$ Myoblasts	-0.794	9.06	0.0456	ENSSSCG00000031903	protein coding
DRG2	*MYMK*$^{+}$ Myoblasts	-0.79	6.23	0.0294	ENSSSCG00000036328	protein coding
ARHGAP21	*MYMK*$^{+}$ Myoblasts	-0.78	7.37	0.0107	ENSSSCG00000011074	protein coding
UNC45B	*MYMK*$^{+}$ Myoblasts	-0.779	6.03	0.0367	ENSSSCG00000017717	protein coding
AGAP1	*MYMK*$^{+}$ Myoblasts	-0.775	7.42	0.0252	ENSSSCG00000016317	protein coding
SPATA5	*MYMK*$^{+}$ Myoblasts	-0.772	6.27	0.0361	ENSSSCG00000009084	protein coding
XPO4	*MYMK*$^{+}$ Myoblasts	-0.77	7.69	0.00715	ENSSSCG00000009276	protein coding
KLHDC8B	*MYMK*$^{+}$ Myoblasts	-0.768	6.14	0.0347	ENSSSCG00000011379	protein coding
RRP36	*MYMK*$^{+}$ Myoblasts	-0.765	6.08	0.0185	ENSSSCG00000001656	protein coding
AHCTF1	*MYMK*$^{+}$ Myoblasts	-0.759	6.45	0.0231	ENSSSCG00000010863	protein coding
NVL	*MYMK*$^{+}$ Myoblasts	-0.756	5.81	0.0355	ENSSSCG00000026705	protein coding
NAA20	*MYMK*$^{+}$ Myoblasts	-0.753	7.26	0.00796	ENSSSCG00000028132	protein coding
NOP2	*MYMK*$^{+}$ Myoblasts	-0.749	5.68	0.0406	ENSSSCG00000000696	protein coding
OSGIN1	*MYMK*$^{+}$ Myoblasts	-0.745	6.29	0.0453	ENSSSCG00000032115	protein coding
RFTN1	*MYMK*$^{+}$ Myoblasts	-0.745	8.29	0.0398	ENSSSCG00000011198	protein coding

MACF1	*MYMK*$^+$ Myoblasts	-0.743	6.75	0.0179	ENSSSCG00000003654	protein coding
CAV3	*MYMK*$^+$ Myoblasts	-0.742	6.94	0.0302	ENSSSCG00000040643	protein coding
PRPF19	*MYMK*$^+$ Myoblasts	-0.741	6.12	0.0291	ENSSSCG00000013107	protein coding
NDUFA6	*MYMK*$^+$ Myoblasts	-0.737	7.22	0.0105	ENSSSCG00000000045	protein coding
CDIP1	*MYMK*$^+$ Myoblasts	-0.731	5.97	0.031	ENSSSCG00000007941	protein coding
NDUFAB1	*MYMK*$^+$ Myoblasts	-0.729	8.02	0.00417	ENSSSCG00000010795	protein coding
NES	*MYMK*$^+$ Myoblasts	-0.72	6.84	0.0144	ENSSSCG00000006474	protein coding
C1orf74	*MYMK*$^+$ Myoblasts	-0.71	7.86	0.0169	ENSSSCG00000015613	protein coding
SEMA4D	*MYMK*$^+$ Myoblasts	-0.708	7.59	0.029	ENSSSCG00000009584	protein coding
DRG1	*MYMK*$^+$ Myoblasts	-0.706	6.71	0.0363	ENSSSCG00000010029	protein coding
MSTRG.9119	*MYMK*$^+$ Myoblasts	-0.7	5.56	0.0486	MSTRG.9119	lncRNA
ACTN2	*MYMK*$^+$ Myoblasts	-0.696	9.68	0.0309	ENSSSCG00000010144	protein coding
NAPA	*MYMK*$^+$ Myoblasts	-0.693	7.46	0.0239	ENSSSCG00000003120	protein coding
DGKZ	*MYMK*$^+$ Myoblasts	-0.69	6.91	0.0354	ENSSSCG00000013259	protein coding
CKM	*MYMK*$^+$ Myoblasts	-0.687	9.54	0.0242	ENSSSCG00000036132	protein coding
NEB	*MYMK*$^+$ Myoblasts	-0.685	6.99	0.035	ENSSSCG00000016397	protein coding
DDR1	*MYMK*$^+$ Myoblasts	-0.684	5.83	0.0482	ENSSSCG00000001382	protein coding
WDR43	*MYMK*$^+$ Myoblasts	-0.684	6.57	0.0291	ENSSSCG00000008534	protein coding
ACADM	*MYMK*$^+$ Myoblasts	-0.681	6.67	0.0305	ENSSSCG00000003776	protein coding
AMH	*MYMK*$^+$ Myoblasts	-0.681	6.16	0.044	ENSSSCG00000035812	protein coding
DIP2C	*MYMK*$^+$ Myoblasts	-0.681	6.59	0.034	ENSSSCG00000011163	protein coding
BNIP3	*MYMK*$^+$ Myoblasts	-0.675	7.6	0.0138	ENSSSCG00000024388	protein coding
ENSSSCG00000004387	*MYMK*$^+$ Myoblasts	-0.674	7.51	0.0355	ENSSSCG00000004387	protein coding
SH3PXD2A	*MYMK*$^+$ Myoblasts	-0.671	7.59	0.0261	ENSSSCG00000010604	protein coding
ANGPTL4	*MYMK*$^+$ Myoblasts	-0.661	8.38	0.0431	ENSSSCG00000013599	protein coding
ISYNA1	*MYMK*$^+$ Myoblasts	-0.66	7.73	0.0469	ENSSSCG00000040524	protein coding
KIF21A	*MYMK*$^+$ Myoblasts	-0.659	7.18	0.0462	ENSSSCG00000000779	protein coding
ENSSSCG00000000530	*MYMK*$^+$ Myoblasts	-0.657	7.55	0.0102	ENSSSCG00000000530	protein coding
PSD3	*MYMK*$^+$ Myoblasts	-0.644	6.95	0.027	ENSSSCG00000007034	protein coding
SGCD	*MYMK*$^+$ Myoblasts	-0.641	8.68	0.0319	ENSSSCG00000017063	protein coding
TMED3	*MYMK*$^+$ Myoblasts	-0.638	8.03	0.0239	ENSSSCG00000001773	protein coding
TRAK1	*MYMK*$^+$ Myoblasts	-0.635	6.9	0.0485	ENSSSCG00000011278	protein coding
EIF4E	*MYMK*$^+$ Myoblasts	-0.634	6.7	0.0284	ENSSSCG00000028420	protein coding
CUX1	*MYMK*$^+$ Myoblasts	-0.63	8.46	0.0219	ENSSSCG00000007681	protein coding

CYCS	*MYMK*$^+$ Myoblasts	-0.626	8.59	0.034	ENSSSCG00000016714	protein coding
FERMT2	*MYMK*$^+$ Myoblasts	-0.625	6.75	0.0363	ENSSSCG00000005041	protein coding
ENSSSCG00000020686	*MYMK*$^+$ Myoblasts	-0.624	7.13	0.0301	ENSSSCG00000020686	protein coding
CHCHD1	*MYMK*$^+$ Myoblasts	-0.623	7.02	0.021	ENSSSCG00000010308	protein coding
BCAR3	*MYMK*$^+$ Myoblasts	-0.622	6.31	0.0442	ENSSSCG00000006893	protein coding
ENSSSCG00000034868	*MYMK*$^+$ Myoblasts	-0.617	6.5	0.0458	ENSSSCG00000034868	protein coding
NEXN	*MYMK*$^+$ Myoblasts	-0.613	7.33	0.0341	ENSSSCG00000003768	protein coding
NFIC	*MYMK*$^+$ Myoblasts	-0.612	8.85	0.0118	ENSSSCG00000036537	protein coding
TNNI2	*MYMK*$^+$ Myoblasts	-0.612	9.5	0.0409	ENSSSCG00000033260	protein coding
SRSF7	*MYMK*$^+$ Myoblasts	-0.607	7.58	0.0209	ENSSSCG00000008484	protein coding
ENSSSCG00000014031	*MYMK*$^+$ Myoblasts	-0.604	7.05	0.0334	ENSSSCG00000014031	protein coding
ILF2	*MYMK*$^+$ Myoblasts	-0.596	7.84	0.0176	ENSSSCG00000028553	protein coding
PTPRA	*MYMK*$^+$ Myoblasts	-0.592	6.56	0.0496	ENSSSCG00000007166	protein coding
TIMM8B	*MYMK*$^+$ Myoblasts	-0.592	7.66	0.0261	ENSSSCG00000023599	protein coding
FLII	*MYMK*$^+$ Myoblasts	-0.589	6.54	0.0476	ENSSSCG00000039324	protein coding
PAK2	*MYMK*$^+$ Myoblasts	-0.581	7.12	0.0475	ENSSSCG00000011843	protein coding
RUVBL1	*MYMK*$^+$ Myoblasts	-0.58	7.54	0.0216	ENSSSCG00000011620	protein coding
CALD1	*MYMK*$^+$ Myoblasts	-0.577	8.83	0.0279	ENSSSCG00000016535	protein coding
SUN1	*MYMK*$^+$ Myoblasts	-0.575	7.13	0.0461	ENSSSCG00000007544	protein coding
COPS3	*MYMK*$^+$ Myoblasts	-0.572	8.77	0.0289	ENSSSCG00000018051	protein coding
PSMB4	*MYMK*$^+$ Myoblasts	-0.57	8.19	0.0164	ENSSSCG00000034984	protein coding
MSTRG.41285	*MYMK*$^+$ Myoblasts	-0.566	10.9	0.0177	MSTRG.41285	lncRNA
CAMTA2	*MYMK*$^+$ Myoblasts	-0.563	7.02	0.0357	ENSSSCG00000017902	protein coding
ERBIN	*MYMK*$^+$ Myoblasts	-0.555	8.03	0.0443	ENSSSCG00000016954	protein coding
FRY	*MYMK*$^+$ Myoblasts	-0.554	8.76	0.044	ENSSSCG00000009338	protein coding
ADAMTS9	*MYMK*$^+$ Myoblasts	-0.551	6.85	0.0464	ENSSSCG00000011496	protein coding
PDCD5	*MYMK*$^+$ Myoblasts	-0.55	7.44	0.0303	ENSSSCG00000002860	protein coding
PSMD4	*MYMK*$^+$ Myoblasts	-0.544	6.79	0.042	ENSSSCG00000006628	protein coding
SGCA	*MYMK*$^+$ Myoblasts	-0.544	7.24	0.0285	ENSSSCG00000017583	protein coding
COX5A	*MYMK*$^+$ Myoblasts	-0.542	8.44	0.0227	ENSSSCG00000024316	protein coding
POLR2E	*MYMK*$^+$ Myoblasts	-0.537	7.78	0.0376	ENSSSCG00000021472	protein coding
ENSSSCG00000032166	*MYMK*$^+$ Myoblasts	-0.529	8.76	0.0234	ENSSSCG00000032166	protein coding
NDUFA4	*MYMK*$^+$ Myoblasts	-0.52	9.3	0.0286	ENSSSCG00000024313	protein coding
PCBP4	*MYMK*$^+$ Myoblasts	-0.506	8.18	0.034	ENSSSCG00000028446	protein coding

MAP4	*MYMK*$^{+}$ Myoblasts	-0.492	8.36	0.0419	ENSSSCG00000029251	protein coding
ATP5PF	*MYMK*$^{+}$ Myoblasts	-0.489	9.04	0.036	ENSSSCG00000038399	protein coding
GAPDH	*MYMK*$^{+}$ Myoblasts	-0.489	11.5	0.0235	ENSSSCG00000000694	protein coding
ERH	*MYMK*$^{+}$ Myoblasts	-0.466	8.47	0.0492	ENSSSCG00000002307	protein coding
SERHL2	*MYMK*$^{+}$ Myoblasts	-0.465	10.1	0.0472	ENSSSCG00000034350	protein coding
RPS3	*MYMK*$^{+}$ Myoblasts	-0.399	12.2	0.0499	ENSSSCG00000014855	protein coding
RPS15A	*MYMK*$^{+}$ Myoblasts	0.33	12.4	0.0458	ENSSSCG00000035768	protein coding
MYO1E	*MYMK*$^{+}$ Myoblasts	0.483	8.64	0.0448	ENSSSCG00000004587	protein coding
PKIA	*MYMK*$^{+}$ Myoblasts	0.485	8.43	0.0387	ENSSSCG00000006163	protein coding
IPO5	*MYMK*$^{+}$ Myoblasts	0.494	7.51	0.0475	ENSSSCG00000009509	protein coding
CHIC2	*MYMK*$^{+}$ Myoblasts	0.498	8.18	0.0337	ENSSSCG00000032645	protein coding
PPP1CC	*MYMK*$^{+}$ Myoblasts	0.501	7.82	0.0493	ENSSSCG00000009828	protein coding
ENSSSCG00000028423	*MYMK*$^{+}$ Myoblasts	0.503	7.56	0.044	ENSSSCG00000028423	protein coding
PABPC1	*MYMK*$^{+}$ Myoblasts	0.515	7.65	0.0491	ENSSSCG00000006063	protein coding
UBE2H	*MYMK*$^{+}$ Myoblasts	0.526	7.72	0.0452	ENSSSCG00000016564	protein coding
SEC62	*MYMK*$^{+}$ Myoblasts	0.531	7.2	0.0395	ENSSSCG00000029608	protein coding
CLIC4	*MYMK*$^{+}$ Myoblasts	0.532	8.63	0.0324	ENSSSCG00000038994	protein coding
RUNX1	*MYMK*$^{+}$ Myoblasts	0.538	9.04	0.0283	ENSSSCG00000035537	protein coding
ENSSSCG00000009772	*MYMK*$^{+}$ Myoblasts	0.545	7.81	0.036	ENSSSCG00000009772	protein coding
MED13L	*MYMK*$^{+}$ Myoblasts	0.545	8.43	0.0265	ENSSSCG00000009864	protein coding
CAPZA2	*MYMK*$^{+}$ Myoblasts	0.546	7.91	0.037	ENSSSCG00000028113	protein coding
ARF4	*MYMK*$^{+}$ Myoblasts	0.547	7.78	0.0277	ENSSSCG00000031791	protein coding
NOL7	*MYMK*$^{+}$ Myoblasts	0.555	7.28	0.035	ENSSSCG00000040816	protein coding
ENSSSCG00000000576	*MYMK*$^{+}$ Myoblasts	0.559	8.29	0.0419	ENSSSCG00000000576	protein coding
ENSSSCG00000024019	*MYMK*$^{+}$ Myoblasts	0.564	6.71	0.0463	ENSSSCG00000024019	protein coding
RIOK3	*MYMK*$^{+}$ Myoblasts	0.564	6.49	0.0448	ENSSSCG00000029518	protein coding
ENSSSCG00000030849	*MYMK*$^{+}$ Myoblasts	0.574	12.2	0.00308	ENSSSCG00000030849	protein coding
RPL22L1	*MYMK*$^{+}$ Myoblasts	0.576	9.61	0.0288	ENSSSCG00000036114	protein coding
ANXA5	*MYMK*$^{+}$ Myoblasts	0.577	7.27	0.04	ENSSSCG00000009097	protein coding
ENSSSCG00000029857	*MYMK*$^{+}$ Myoblasts	0.586	7.44	0.0345	ENSSSCG00000029857	protein coding
ZNF277	*MYMK*$^{+}$ Myoblasts	0.589	7.25	0.0302	ENSSSCG00000025910	protein coding
XBP1	*MYMK*$^{+}$ Myoblasts	0.59	7.63	0.0267	ENSSSCG00000030996	protein coding
PTMA	*MYMK*$^{+}$ Myoblasts	0.606	11	0.00282	ENSSSCG00000037274	protein coding
CCNL1	*MYMK*$^{+}$ Myoblasts	0.61	6.99	0.0474	ENSSSCG00000029352	protein coding

ENSSSCG00000012119	*MYMK*$^{+}$ Myoblasts	0.613	10.1	0.0322	ENSSSCG00000012119	protein coding
HIVEP2	*MYMK*$^{+}$ Myoblasts	0.619	7.99	0.0249	ENSSSCG00000004138	protein coding
ENSSSCG00000036483	*MYMK*$^{+}$ Myoblasts	0.62	6.2	0.0456	ENSSSCG00000036483	protein coding
CCNG2	*MYMK*$^{+}$ Myoblasts	0.622	7.35	0.0191	ENSSSCG00000008988	protein coding
MTFR1L	*MYMK*$^{+}$ Myoblasts	0.625	6.99	0.0222	ENSSSCG00000003553	protein coding
BAZ2B	*MYMK*$^{+}$ Myoblasts	0.629	7.46	0.0267	ENSSSCG00000015882	protein coding
ENSSSCG00000007088	*MYMK*$^{+}$ Myoblasts	0.643	6.89	0.0255	ENSSSCG00000007088	protein coding
ENSSSCG00000012178	*MYMK*$^{+}$ Myoblasts	0.648	8	0.0131	ENSSSCG00000012178	protein coding
ENSSSCG00000029761	*MYMK*$^{+}$ Myoblasts	0.65	6.19	0.0312	ENSSSCG00000029761	protein coding
UBE2E3	*MYMK*$^{+}$ Myoblasts	0.65	7.41	0.0174	ENSSSCG00000033987	protein coding
ENSSSCG00000033733	*MYMK*$^{+}$ Myoblasts	0.651	8.19	0.0229	ENSSSCG00000033733	protein coding
NFIA	*MYMK*$^{+}$ Myoblasts	0.651	8.22	0.0368	ENSSSCG00000023243	protein coding
ENSSSCG00000038991	*MYMK*$^{+}$ Myoblasts	0.66	6.44	0.0449	ENSSSCG00000038991	protein coding
DISP1	*MYMK*$^{+}$ Myoblasts	0.663	7.6	0.0135	ENSSSCG00000035479	protein coding
SSBP2	*MYMK*$^{+}$ Myoblasts	0.667	7.76	0.013	ENSSSCG00000035757	protein coding
MAN2A1	*MYMK*$^{+}$ Myoblasts	0.668	7.23	0.021	ENSSSCG00000014195	protein coding
CYB5A	*MYMK*$^{+}$ Myoblasts	0.673	6.34	0.0407	ENSSSCG00000004875	protein coding
ENSSSCG00000005453	*MYMK*$^{+}$ Myoblasts	0.674	8.85	0.0274	ENSSSCG00000005453	protein coding
C4orf3	*MYMK*$^{+}$ Myoblasts	0.678	7.26	0.0218	ENSSSCG00000031773	protein coding
TBC1D15	*MYMK*$^{+}$ Myoblasts	0.679	6.7	0.0347	ENSSSCG00000000512	protein coding
SLC43A3	*MYMK*$^{+}$ Myoblasts	0.686	6.57	0.0458	ENSSSCG00000033998	protein coding
ENSSSCG00000004307	*MYMK*$^{+}$ Myoblasts	0.687	7.89	0.0165	ENSSSCG00000004307	protein coding
FBXL7	*MYMK*$^{+}$ Myoblasts	0.687	6.25	0.0278	ENSSSCG00000028239	protein coding
ELL	*MYMK*$^{+}$ Myoblasts	0.689	6.13	0.044	ENSSSCG00000040181	protein coding
ENSSSCG00000004598	*MYMK*$^{+}$ Myoblasts	0.689	6.13	0.0309	ENSSSCG00000004598	protein coding
DNAJB4	*MYMK*$^{+}$ Myoblasts	0.695	7.77	0.0244	ENSSSCG00000003766	protein coding
PNRC2	*MYMK*$^{+}$ Myoblasts	0.709	6.85	0.0365	ENSSSCG00000023326	protein coding
SELENOH	*MYMK*$^{+}$ Myoblasts	0.713	7.06	0.0111	ENSSSCG00000032364	protein coding
PEAK1	*MYMK*$^{+}$ Myoblasts	0.715	6.24	0.0318	ENSSSCG00000001869	protein coding
PPP1R9A	*MYMK*$^{+}$ Myoblasts	0.72	6.29	0.0253	ENSSSCG00000015329	protein coding
ENSSSCG00000000274	*MYMK*$^{+}$ Myoblasts	0.725	8.46	0.0489	ENSSSCG00000000274	protein coding
P4HA1	*MYMK*$^{+}$ Myoblasts	0.725	6.19	0.0402	ENSSSCG00000010292	protein coding
FBXW7	*MYMK*$^{+}$ Myoblasts	0.726	6.54	0.0431	ENSSSCG00000020879	protein coding
MSTRG.12822	*MYMK*$^{+}$ Myoblasts	0.727	6.75	0.0454	MSTRG.12822	lncRNA

UBC	*MYMK*$^{+}$ Myoblasts	0.733	9.72	0.00438	ENSSSCG00000029226	protein coding
PRKCSH	*MYMK*$^{+}$ Myoblasts	0.736	6.95	0.0445	ENSSSCG00000013627	protein coding
AP2B1	*MYMK*$^{+}$ Myoblasts	0.739	6.32	0.0165	ENSSSCG00000017712	protein coding
MSTRG.3891	*MYMK*$^{+}$ Myoblasts	0.74	6.07	0.0415	MSTRG.3891	lncRNA
ZFP36L1	*MYMK*$^{+}$ Myoblasts	0.74	8.07	0.0137	ENSSSCG00000028304	protein coding
GFM1	*MYMK*$^{+}$ Myoblasts	0.746	6.91	0.0208	ENSSSCG00000025651	protein coding
CBLB	*MYMK*$^{+}$ Myoblasts	0.75	6.44	0.0169	ENSSSCG00000022486	protein coding
YWHAZ	*MYMK*$^{+}$ Myoblasts	0.752	7.47	0.00566	ENSSSCG00000006062	protein coding
RNF169	*MYMK*$^{+}$ Myoblasts	0.754	6.77	0.015	ENSSSCG00000021822	protein coding
SYPL1	*MYMK*$^{+}$ Myoblasts	0.754	7.02	0.00795	ENSSSCG00000015434	protein coding
COL19A1	*MYMK*$^{+}$ Myoblasts	0.766	5.61	0.0434	ENSSSCG00000044472	protein coding
MSTRG.1135	*MYMK*$^{+}$ Myoblasts	0.766	7.62	0.00794	MSTRG.1135	lncRNA
PNRC1	*MYMK*$^{+}$ Myoblasts	0.767	8.08	0.00431	ENSSSCG00000039953	protein coding
ANP32E	*MYMK*$^{+}$ Myoblasts	0.772	6.32	0.0167	ENSSSCG00000035209	protein coding
CST3	*MYMK*$^{+}$ Myoblasts	0.772	8.34	0.00691	ENSSSCG00000037360	protein coding
ARPC5	*MYMK*$^{+}$ Myoblasts	0.776	5.55	0.0447	ENSSSCG00000015560	protein coding
LHFPL6	*MYMK*$^{+}$ Myoblasts	0.776	6.28	0.0373	ENSSSCG00000029855	protein coding
ENSSSCG00000022176	*MYMK*$^{+}$ Myoblasts	0.781	8.07	0.0432	ENSSSCG00000022176	protein coding
MSTRG.41997	*MYMK*$^{+}$ Myoblasts	0.784	5.92	0.0207	MSTRG.41997	lncRNA
AP2S1	*MYMK*$^{+}$ Myoblasts	0.785	6.79	0.011	ENSSSCG00000003106	protein coding
NR4A1	*MYMK*$^{+}$ Myoblasts	0.787	6.88	0.0491	ENSSSCG00000031321	protein coding
MSTRG.36822	*MYMK*$^{+}$ Myoblasts	0.788	7.19	0.0106	MSTRG.36822	lncRNA
ARHGAP35	*MYMK*$^{+}$ Myoblasts	0.79	5.53	0.0384	ENSSSCG00000003107	protein coding
TBC1D12	*MYMK*$^{+}$ Myoblasts	0.79	5.67	0.0347	ENSSSCG00000010485	protein coding
MICU1	*MYMK*$^{+}$ Myoblasts	0.791	7.17	0.00474	ENSSSCG00000010289	protein coding
POLR1C	*MYMK*$^{+}$ Myoblasts	0.791	5.59	0.0349	ENSSSCG00000028007	protein coding
BTG3	*MYMK*$^{+}$ Myoblasts	0.794	6.83	0.0351	ENSSSCG00000012014	protein coding
CCNC	*MYMK*$^{+}$ Myoblasts	0.794	6.2	0.0121	ENSSSCG00000004353	protein coding
GPX4	*MYMK*$^{+}$ Myoblasts	0.795	7.72	0.00304	ENSSSCG00000024052	protein coding
AP3B1	*MYMK*$^{+}$ Myoblasts	0.796	5.95	0.042	ENSSSCG00000014101	protein coding
CRK	*MYMK*$^{+}$ Myoblasts	0.797	9.56	0.00505	ENSSSCG00000038989	protein coding
SPPL2A	*MYMK*$^{+}$ Myoblasts	0.8	5.51	0.0302	ENSSSCG00000004635	protein coding
HSPB1	*MYMK*$^{+}$ Myoblasts	0.803	8.86	0.0176	ENSSSCG00000039962	protein coding
ADORA1	*MYMK*$^{+}$ Myoblasts	0.824	6.03	0.0461	ENSSSCG00000021059	protein coding

SERPING1	*MYMK*$^+$ Myoblasts	0.825	5.51	0.0474	ENSSSCG00000013181	protein coding
RNF11	*MYMK*$^+$ Myoblasts	0.826	6.06	0.0367	ENSSSCG00000031172	protein coding
PLEKHA5	*MYMK*$^+$ Myoblasts	0.828	6.26	0.0248	ENSSSCG00000000588	protein coding
ENSSSCG00000028035	*MYMK*$^+$ Myoblasts	0.831	6.14	0.0275	ENSSSCG00000028035	protein coding
MEIS1	*MYMK*$^+$ Myoblasts	0.839	5.89	0.0239	ENSSSCG00000039161	protein coding
HSPA5	*MYMK*$^+$ Myoblasts	0.844	10.8	0.00589	ENSSSCG00000005601	protein coding
SAT1	*MYMK*$^+$ Myoblasts	0.85	5.45	0.0338	ENSSSCG00000012173	protein coding
DESI2	*MYMK*$^+$ Myoblasts	0.859	5.85	0.0406	ENSSSCG00000010876	protein coding
EPS15	*MYMK*$^+$ Myoblasts	0.86	6.57	0.0168	ENSSSCG00000003872	protein coding
MAPRE2	*MYMK*$^+$ Myoblasts	0.871	5.5	0.0258	ENSSSCG00000003738	protein coding
YPEL5	*MYMK*$^+$ Myoblasts	0.871	5.44	0.0397	ENSSSCG00000008529	protein coding
EPB41L2	*MYMK*$^+$ Myoblasts	0.873	7.64	0.00286	ENSSSCG00000004199	protein coding
ENSSSCG00000007322	*MYMK*$^+$ Myoblasts	0.876	5.71	0.0276	ENSSSCG00000007322	protein coding
GCNT1	*MYMK*$^+$ Myoblasts	0.878	6.18	0.0102	ENSSSCG00000005277	protein coding
PTPN9	*MYMK*$^+$ Myoblasts	0.88	5.44	0.0244	ENSSSCG00000001878	protein coding
ENSSSCG00000033734	*MYMK*$^+$ Myoblasts	0.891	6.38	0.0299	ENSSSCG00000033734	protein coding
SNRNP25	*MYMK*$^+$ Myoblasts	0.91	6.63	0.00464	ENSSSCG00000007962	protein coding
RASSF8	*MYMK*$^+$ Myoblasts	0.912	5.78	0.0336	ENSSSCG00000000559	protein coding
MSTRG.3217	*MYMK*$^+$ Myoblasts	0.913	5.75	0.0208	MSTRG.3217	lncRNA
ENSSSCG00000039523	*MYMK*$^+$ Myoblasts	0.921	5.27	0.028	ENSSSCG00000039523	protein coding
ENSSSCG00000037595	*MYMK*$^+$ Myoblasts	0.937	5.81	0.0109	ENSSSCG00000037595	protein coding
CETN3	*MYMK*$^+$ Myoblasts	0.942	6.01	0.0114	ENSSSCG00000014151	protein coding
ZFPM2	*MYMK*$^+$ Myoblasts	0.947	6.48	0.0142	ENSSSCG00000006038	protein coding
ARMC10	*MYMK*$^+$ Myoblasts	0.948	5.82	0.00726	ENSSSCG00000028444	protein coding
TMSB10	*MYMK*$^+$ Myoblasts	0.949	9.89	0.0237	ENSSSCG00000008245	protein coding
COL5A2	*MYMK*$^+$ Myoblasts	0.956	6.92	0.00456	ENSSSCG00000016035	protein coding
SLC1A5	*MYMK*$^+$ Myoblasts	0.956	5.7	0.0256	ENSSSCG00000003105	protein coding
PAFAH1B2	*MYMK*$^+$ Myoblasts	0.958	5.53	0.0455	ENSSSCG00000024027	protein coding
IREB2	*MYMK*$^+$ Myoblasts	0.964	5.09	0.032	ENSSSCG00000001756	protein coding
LRIG2	*MYMK*$^+$ Myoblasts	0.969	5.82	0.00836	ENSSSCG00000034581	protein coding
ENSSSCG00000005478	*MYMK*$^+$ Myoblasts	0.971	5.25	0.0296	ENSSSCG00000005478	protein coding
CCDC173	*MYMK*$^+$ Myoblasts	0.972	5.84	0.034	ENSSSCG00000015935	protein coding
MSTRG.20753	*MYMK*$^+$ Myoblasts	0.972	5.97	0.0382	MSTRG.20753	lncRNA
XRCC6	*MYMK*$^+$ Myoblasts	0.976	5.53	0.0255	ENSSSCG00000000059	protein coding

ZYX	*MYMK*$^+$ Myoblasts	0.976	6.67	0.00917	ENSSSCG00000016461	protein coding
MSTRG.30533	*MYMK*$^+$ Myoblasts	0.979	6.68	0.0106	MSTRG.30533	lncRNA
SLC39A14	*MYMK*$^+$ Myoblasts	0.982	4.97	0.0475	ENSSSCG00000032622	protein coding
TSPO	*MYMK*$^+$ Myoblasts	0.984	5.58	0.0472	ENSSSCG00000000033	protein coding
KLHL24	*MYMK*$^+$ Myoblasts	0.986	6.07	0.0117	ENSSSCG00000011775	protein coding
OSBPL6	*MYMK*$^+$ Myoblasts	0.989	6.43	0.0269	ENSSSCG00000023133	protein coding
SLC35A1	*MYMK*$^+$ Myoblasts	0.993	5.25	0.0446	ENSSSCG00000004304	protein coding
CHURC1	*MYMK*$^+$ Myoblasts	0.995	5.58	0.0224	ENSSSCG00000022147	protein coding
MAP2K1	*MYMK*$^+$ Myoblasts	0.997	5.3	0.0391	ENSSSCG00000004943	protein coding
GIPC1	*MYMK*$^+$ Myoblasts	1	5.29	0.029	ENSSSCG00000032842	protein coding
LITAF	*MYMK*$^+$ Myoblasts	1	6.19	0.0228	ENSSSCG00000032381	protein coding
ARMCX3	*MYMK*$^+$ Myoblasts	1.01	5.53	0.0119	ENSSSCG00000040281	protein coding
CASP6	*MYMK*$^+$ Myoblasts	1.01	5.45	0.0298	ENSSSCG00000009140	protein coding
SLC38A6	*MYMK*$^+$ Myoblasts	1.01	5.46	0.0179	ENSSSCG00000005092	protein coding
WSB1	*MYMK*$^+$ Myoblasts	1.01	5.99	0.0367	ENSSSCG00000017752	protein coding
CCNG1	*MYMK*$^+$ Myoblasts	1.02	7.76	0.00101	ENSSSCG00000017024	protein coding
EAPP	*MYMK*$^+$ Myoblasts	1.02	5.42	0.0139	ENSSSCG00000001960	protein coding
MSTRG.799	*MYMK*$^+$ Myoblasts	1.02	5.21	0.0402	MSTRG.799	lncRNA
PIK3C3	*MYMK*$^+$ Myoblasts	1.02	5.12	0.0494	ENSSSCG00000003749	protein coding
TRPS1	*MYMK*$^+$ Myoblasts	1.02	6.56	0.00283	ENSSSCG00000006018	protein coding
COL1A2	*MYMK*$^+$ Myoblasts	1.03	8.63	0.034	ENSSSCG00000015326	protein coding
GADD45B	*MYMK*$^+$ Myoblasts	1.03	6.06	0.0246	ENSSSCG00000022689	protein coding
TNFAIP8	*MYMK*$^+$ Myoblasts	1.03	5.25	0.0168	ENSSSCG00000040617	protein coding
BTG1	*MYMK*$^+$ Myoblasts	1.04	9.92	0.0051	ENSSSCG00000035790	protein coding
METTL23	*MYMK*$^+$ Myoblasts	1.04	5.33	0.0143	ENSSSCG00000023139	protein coding
RFTN2	*MYMK*$^+$ Myoblasts	1.04	5.73	0.0117	ENSSSCG00000037483	protein coding
SEC22B	*MYMK*$^+$ Myoblasts	1.04	5.5	0.00992	ENSSSCG00000020964	protein coding
SGCE	*MYMK*$^+$ Myoblasts	1.04	6.11	0.0169	ENSSSCG00000015328	protein coding
ADGRB3	*MYMK*$^+$ Myoblasts	1.05	5.53	0.0248	ENSSSCG00000004270	protein coding
ARL13B	*MYMK*$^+$ Myoblasts	1.05	5.98	0.00785	ENSSSCG00000027571	protein coding
NBR1	*MYMK*$^+$ Myoblasts	1.05	5.99	0.013	ENSSSCG00000030211	protein coding
RBMS3	*MYMK*$^+$ Myoblasts	1.05	7.15	0.0065	ENSSSCG00000036381	protein coding
NBEA	*MYMK*$^+$ Myoblasts	1.06	5.25	0.0199	ENSSSCG00000034348	protein coding
SPARCL1	*MYMK*$^+$ Myoblasts	1.07	6.84	0.0268	ENSSSCG00000009222	protein coding

CITED2	*MYMK*$^+$ Myoblasts	1.09	6.93	0.00247	ENSSSCG00000004142	protein coding
MAN1A2	*MYMK*$^+$ Myoblasts	1.09	5.48	0.00626	ENSSSCG00000006730	protein coding
BIRC2	*MYMK*$^+$ Myoblasts	1.1	7.18	0.000716	ENSSSCG00000027529	protein coding
MEST	*MYMK*$^+$ Myoblasts	1.1	6.21	0.0342	ENSSSCG00000016554	protein coding
SERPINH1	*MYMK*$^+$ Myoblasts	1.1	7.9	0.0129	ENSSSCG00000039468	protein coding
WDR61	*MYMK*$^+$ Myoblasts	1.1	5.48	0.00951	ENSSSCG00000001757	protein coding
EMC2	*MYMK*$^+$ Myoblasts	1.11	5.57	0.00539	ENSSSCG00000006032	protein coding
PLAC9	*MYMK*$^+$ Myoblasts	1.11	6.13	0.014	ENSSSCG00000010332	protein coding
TGFBR3	*MYMK*$^+$ Myoblasts	1.11	5.58	0.00649	ENSSSCG00000006911	protein coding
DHX40	*MYMK*$^+$ Myoblasts	1.12	5.64	0.0129	ENSSSCG00000017665	protein coding
GOLM2	*MYMK*$^+$ Myoblasts	1.13	5.42	0.00947	ENSSSCG00000023996	protein coding
MSTRG.161	*MYMK*$^+$ Myoblasts	1.13	7.53	0.00121	MSTRG.161	lncRNA
COMMD8	*MYMK*$^+$ Myoblasts	1.15	4.87	0.0229	ENSSSCG00000033373	protein coding
PDLIM4	*MYMK*$^+$ Myoblasts	1.15	6.13	0.0215	ENSSSCG00000014274	protein coding
MSTRG.3205	*MYMK*$^+$ Myoblasts	1.16	4.94	0.0206	MSTRG.3205	lncRNA
ARID5B	*MYMK*$^+$ Myoblasts	1.17	6	0.0114	ENSSSCG00000010219	protein coding
MSTRG.6594	*MYMK*$^+$ Myoblasts	1.17	7.4	0.0283	MSTRG.6594	lncRNA
MATN2	*MYMK*$^+$ Myoblasts	1.18	6.22	0.0442	ENSSSCG00000006082	protein coding
BAG3	*MYMK*$^+$ Myoblasts	1.19	7.75	0.00298	ENSSSCG00000010686	protein coding
MSC	*MYMK*$^+$ Myoblasts	1.19	5.42	0.0475	ENSSSCG00000006187	protein coding
PHTF2	*MYMK*$^+$ Myoblasts	1.2	5.35	0.024	ENSSSCG00000015410	protein coding
PLXDC2	*MYMK*$^+$ Myoblasts	1.21	5.87	0.00502	ENSSSCG00000032444	protein coding
RFX2	*MYMK*$^+$ Myoblasts	1.21	5.5	0.0284	ENSSSCG00000035403	protein coding
PAICS	*MYMK*$^+$ Myoblasts	1.22	5.48	0.0062	ENSSSCG00000008901	protein coding
UBE2E2	*MYMK*$^+$ Myoblasts	1.22	5.3	0.0178	ENSSSCG00000031392	protein coding
CCNT2	*MYMK*$^+$ Myoblasts	1.23	5.05	0.019	ENSSSCG00000015697	protein coding
IGF2BP2	*MYMK*$^+$ Myoblasts	1.23	5.69	0.0126	ENSSSCG00000011795	protein coding
MSTRG.13235	*MYMK*$^+$ Myoblasts	1.23	5.02	0.0304	MSTRG.13235	lncRNA
PTS	*MYMK*$^+$ Myoblasts	1.23	5.86	0.0315	ENSSSCG00000015040	protein coding
TAGLN	*MYMK*$^+$ Myoblasts	1.26	6.22	0.00742	ENSSSCG00000015073	protein coding
MSTRG.44547	*MYMK*$^+$ Myoblasts	1.27	6.02	0.00353	MSTRG.44547	lncRNA
GAB1	*MYMK*$^+$ Myoblasts	1.28	6.29	0.00163	ENSSSCG00000009048	protein coding
ENSSSCG00000022128	*MYMK*$^+$ Myoblasts	1.29	5.37	0.0053	ENSSSCG00000022128	protein coding
PTPN12	*MYMK*$^+$ Myoblasts	1.3	5.61	0.00275	ENSSSCG00000015411	protein coding

SUMF1	*MYMK*$^+$ Myoblasts	1.3	5.49	0.00765	ENSSSCG00000011532	protein coding
BCAS1	*MYMK*$^+$ Myoblasts	1.31	5.39	0.0447	ENSSSCG00000007485	protein coding
GPC3	*MYMK*$^+$ Myoblasts	1.31	7.63	0.00698	ENSSSCG00000012680	protein coding
PHGDH	*MYMK*$^+$ Myoblasts	1.32	5.87	0.0245	ENSSSCG00000006717	protein coding
ENSSSCG00000035364	*MYMK*$^+$ Myoblasts	1.33	5.96	0.00819	ENSSSCG00000035364	protein coding
CDC42EP5	*MYMK*$^+$ Myoblasts	1.36	6.07	0.00907	ENSSSCG00000039922	protein coding
NFKBIA	*MYMK*$^+$ Myoblasts	1.36	5.91	0.0162	ENSSSCG00000001952	protein coding
ENSSSCG00000027777	*MYMK*$^+$ Myoblasts	1.37	5.45	0.00242	ENSSSCG00000027777	protein coding
TIMP1	*MYMK*$^+$ Myoblasts	1.37	5.61	0.00474	ENSSSCG00000012277	protein coding
KANSL1L	*MYMK*$^+$ Myoblasts	1.38	5.22	0.0165	ENSSSCG00000022830	protein coding
MSTRG.20986	*MYMK*$^+$ Myoblasts	1.38	4.97	0.0134	MSTRG.20986	lncRNA
GUSB	*MYMK*$^+$ Myoblasts	1.39	5.07	0.0264	ENSSSCG00000007739	protein coding
VPS37A	*MYMK*$^+$ Myoblasts	1.39	5.35	0.00781	ENSSSCG00000006984	protein coding
ADAM9	*MYMK*$^+$ Myoblasts	1.4	5.47	0.00542	ENSSSCG00000015808	protein coding
MSTRG.17788	*MYMK*$^+$ Myoblasts	1.41	5.75	0.00162	MSTRG.17788	lncRNA
CREB3L2	*MYMK*$^+$ Myoblasts	1.42	5.4	0.00403	ENSSSCG00000016520	protein coding
HSPH1	*MYMK*$^+$ Myoblasts	1.42	7.86	0.00412	ENSSSCG00000009334	protein coding
RRAD	*MYMK*$^+$ Myoblasts	1.42	6.94	0.0287	ENSSSCG00000036060	protein coding
MSANTD2	*MYMK*$^+$ Myoblasts	1.43	5	0.00724	ENSSSCG00000015201	protein coding
SCN7A	*MYMK*$^+$ Myoblasts	1.48	5.12	0.0366	ENSSSCG00000015914	protein coding
SELENOW	*MYMK*$^+$ Myoblasts	1.49	7.02	0.000316	ENSSSCG00000034313	protein coding
DNAJB1	*MYMK*$^+$ Myoblasts	1.5	8.83	0.00477	ENSSSCG00000013784	protein coding
LUM	*MYMK*$^+$ Myoblasts	1.5	7.27	0.0178	ENSSSCG00000000916	protein coding
TPM4	*MYMK*$^+$ Myoblasts	1.51	7.01	0.00495	ENSSSCG00000013849	protein coding
CYBRD1	*MYMK*$^+$ Myoblasts	1.53	4.91	0.0065	ENSSSCG00000025049	protein coding
CORO1B	*MYMK*$^+$ Myoblasts	1.56	5.19	0.00458	ENSSSCG00000012920	protein coding
MAGI3	*MYMK*$^+$ Myoblasts	1.57	6.21	0.000825	ENSSSCG00000006767	protein coding
SLIT2	*MYMK*$^+$ Myoblasts	1.58	5.52	0.0157	ENSSSCG00000008749	protein coding
BRD8	*MYMK*$^+$ Myoblasts	1.59	5.11	0.00756	ENSSSCG00000014327	protein coding
BCKDHB	*MYMK*$^+$ Myoblasts	1.6	5.42	0.00234	ENSSSCG00000004465	protein coding
ARHGAP28	*MYMK*$^+$ Myoblasts	1.61	5.32	0.00489	ENSSSCG00000021612	protein coding
PDCD4	*MYMK*$^+$ Myoblasts	1.63	5.44	0.00142	ENSSSCG00000010627	protein coding
GPX1	*MYMK*$^+$ Myoblasts	1.66	6.39	0.00142	ENSSSCG00000033727	protein coding
GPX3	*MYMK*$^+$ Myoblasts	1.68	5.65	0.00218	ENSSSCG00000036438	protein coding

ENSSSCG00000012452	*MYMK*$^{+}$ Myoblasts	1.74	5.1	0.000939	ENSSSCG00000012452	protein coding
ENSSSCG00000006395	*MYMK*$^{+}$ Myoblasts	1.77	5.95	0.00487	ENSSSCG00000006395	protein coding
MSTRG.32627	*MYMK*$^{+}$ Myoblasts	1.8	5.26	0.00469	MSTRG.32627	lncRNA
SELENOP	*MYMK*$^{+}$ Myoblasts	1.82	7.85	0.000134	ENSSSCG00000021208	protein coding
MSTRG.38467	*MYMK*$^{+}$ Myoblasts	1.88	8.9	0.0175	MSTRG.38467	lncRNA
PARM1	*MYMK*$^{+}$ Myoblasts	1.88	5.41	0.00484	ENSSSCG00000008966	protein coding
ENSSSCG00000016823	*MYMK*$^{+}$ Myoblasts	1.97	6.76	0.00148	ENSSSCG00000016823	protein coding
ENSSSCG00000029160	*MYMK*$^{+}$ Myoblasts	2.08	10.6	0.0074	ENSSSCG00000029160	protein coding
MSTRG.33023	*MYMK*$^{+}$ Myoblasts	2.08	5.22	0.00562	MSTRG.33023	lncRNA
CKS2	*MYMK*$^{+}$ Myoblasts	2.1	6.08	0.000272	ENSSSCG00000021161	protein coding
MSTRG.1341	*MYMK*$^{+}$ Myoblasts	2.34	5.46	0.00214	MSTRG.1341	lncRNA
ENSSSCG00000036880	*MYMK*$^{+}$ Myoblasts	2.4	4.58	0.000844	ENSSSCG00000036880	lncRNA
ENSSSCG00000037324	*MYMK*$^{+}$ Myoblasts	2.53	5.06	0.000271	ENSSSCG00000037324	lncRNA
MSTRG.38853	*MYMK*$^{+}$ Myoblasts	2.69	7.53	0.00688	MSTRG.38853	lncRNA
MYF5	*MYMK*$^{+}$ Myoblasts	3.09	6.05	0.000598	ENSSSCG00000000937	protein coding
ENSSSCG00000014565	Satellite stem cells	-3.33	5	0.00864	ENSSSCG00000014565	protein coding
MPP3	Satellite stem cells	-3.12	4.99	3.23E-05	ENSSSCG00000017372	protein coding
IFITM3	Satellite stem cells	-2.99	6.7	0.00708	ENSSSCG00000038912	protein coding
MSTRG.23873	Satellite stem cells	-2.93	3.8	0.00153	MSTRG.23873	lncRNA
KIF22	Satellite stem cells	-2.64	6.01	4.63E-06	ENSSSCG00000029509	protein coding
DLL4	Satellite stem cells	-2.56	3.88	0.000303	ENSSSCG00000004755	protein coding
PNMT	Satellite stem cells	-2.52	3.36	0.000564	ENSSSCG00000017501	protein coding
MSTRG.29485	Satellite stem cells	-2.5	3.8	0.000991	MSTRG.29485	lncRNA
MSTRG.6894	Satellite stem cells	-2.5	3.24	0.000505	MSTRG.6894	lncRNA
MSTRG.4840	Satellite stem cells	-2.44	4.26	9.51E-05	MSTRG.4840	lncRNA
USP18	Satellite stem cells	-2.39	4.77	0.0275	ENSSSCG00000000774	protein coding
ENSSSCG00000004572	Satellite stem cells	-2.37	5.46	0.000355	ENSSSCG00000004572	protein coding
BDKRB2	Satellite stem cells	-2.22	3.03	0.00214	ENSSSCG00000027030	protein coding
MSTRG.22838	Satellite stem cells	-2.19	4.26	0.00177	MSTRG.22838	lncRNA
MSTRG.31726	Satellite stem cells	-2.05	3.87	0.000364	MSTRG.31726	lncRNA
RGR	Satellite stem cells	-1.97	3.89	0.00264	ENSSSCG00000010350	protein coding
TMEM100	Satellite stem cells	-1.93	4.12	0.00939	ENSSSCG00000017607	protein coding
TRIM21	Satellite stem cells	-1.92	3.38	0.0188	ENSSSCG00000014780	protein coding
MSTRG.45291	Satellite stem cells	-1.86	3.25	0.00265	MSTRG.45291	lncRNA
MSTRG.39706	Satellite stem cells	-1.84	7.14	0.000543	MSTRG.39706	lncRNA
UVSSA	Satellite stem cells	-1.83	3.13	0.0071	ENSSSCG00000037035	protein coding

TMEM108	Satellite stem cells	-1.78	3.72	0.0042	ENSSSCG00000011635	protein coding
MSTRG.30373	Satellite stem cells	-1.76	3.45	0.00502	MSTRG.30373	lncRNA
MSTRG.17351	Satellite stem cells	-1.75	3.07	0.00367	MSTRG.17351	lncRNA
H1-4	Satellite stem cells	-1.74	3.84	0.00414	ENSSSCG00000033672	protein coding
MSTRG.40859	Satellite stem cells	-1.72	3.67	0.00398	MSTRG.40859	lncRNA
SPECC1	Satellite stem cells	-1.71	4.98	0.00019	ENSSSCG00000034364	protein coding
BSN	Satellite stem cells	-1.66	3.77	0.00233	ENSSSCG00000011384	protein coding
MSTRG.26439	Satellite stem cells	-1.66	3.13	0.00567	MSTRG.26439	lncRNA
C5orf22	Satellite stem cells	-1.62	3.49	0.00361	ENSSSCG00000016808	protein coding
MSTRG.34836	Satellite stem cells	-1.62	4.42	0.000772	MSTRG.34836	lncRNA
ENSSSCG00000025593	Satellite stem cells	-1.61	4.36	0.0211	ENSSSCG00000025593	protein coding
RGS16	Satellite stem cells	-1.57	3.58	0.0187	ENSSSCG00000015550	protein coding
SH3RF2	Satellite stem cells	-1.57	4.29	0.000795	ENSSSCG00000014410	protein coding
GSE1	Satellite stem cells	-1.55	5.24	0.000256	ENSSSCG00000002664	protein coding
CAVIN2	Satellite stem cells	-1.54	5.85	0.000299	ENSSSCG00000026618	protein coding
MSTRG.32994	Satellite stem cells	-1.54	3.38	0.00846	MSTRG.32994	lncRNA
MSTRG.25569	Satellite stem cells	-1.51	3.15	0.0141	MSTRG.25569	lncRNA
TESK1	Satellite stem cells	-1.51	3.35	0.0149	ENSSSCG00000005310	protein coding
EIF2AK2	Satellite stem cells	-1.49	5.6	0.0429	ENSSSCG00000008496	protein coding
MSTRG.14445	Satellite stem cells	-1.48	5.07	0.00441	MSTRG.14445	lncRNA
UCP2	Satellite stem cells	-1.48	4.81	0.000981	ENSSSCG00000014833	protein coding
MSTRG.38316	Satellite stem cells	-1.46	3.8	0.016	MSTRG.38316	lncRNA
SERPINA1	Satellite stem cells	-1.46	3.96	0.00193	ENSSSCG00000002476	protein coding
POLR1B	Satellite stem cells	-1.43	3.1	0.0204	ENSSSCG00000008098	protein coding
C14orf93	Satellite stem cells	-1.42	4.13	0.00466	ENSSSCG00000031452	protein coding
DUSP4	Satellite stem cells	-1.41	3.51	0.0128	ENSSSCG00000015850	protein coding
SH3GL2	Satellite stem cells	-1.41	3.74	0.00298	ENSSSCG00000005177	protein coding
TSTD2	Satellite stem cells	-1.41	3.91	0.00331	ENSSSCG00000005367	protein coding
MSTRG.43915	Satellite stem cells	-1.39	3.16	0.0203	MSTRG.43915	lncRNA
PDGFB	Satellite stem cells	-1.39	4.21	0.00366	ENSSSCG00000033327	protein coding
TNNT1	Satellite stem cells	-1.39	6.5	0.0499	ENSSSCG00000025353	protein coding
MYOG	Satellite stem cells	-1.38	6.01	0.00256	ENSSSCG00000034654	protein coding
ARPP21	Satellite stem cells	-1.36	4.2	0.00737	ENSSSCG00000011238	protein coding
MSTRG.22353	Satellite stem cells	-1.36	3.11	0.0166	MSTRG.22353	lncRNA
HK3	Satellite stem cells	-1.35	3.86	0.0374	ENSSSCG00000014048	protein coding
MAPT	Satellite stem cells	-1.35	3.9	0.0211	ENSSSCG00000017311	protein coding
MSTRG.39368	Satellite stem cells	-1.33	4.49	0.00848	MSTRG.39368	lncRNA
NSMAF	Satellite stem cells	-1.33	3.59	0.0201	ENSSSCG00000006236	protein coding

PITPNM2	Satellite stem cells	-1.33	4.09	0.0095	ENSSSCG00000009781	protein coding
SERPINB6	Satellite stem cells	-1.32	5.29	0.0024	ENSSSCG00000038404	protein coding
FBN3	Satellite stem cells	-1.3	6.46	0.000933	ENSSSCG00000013594	protein coding
MSTRG.11644	Satellite stem cells	-1.3	3.67	0.0285	MSTRG.11644	lncRNA
SVIL	Satellite stem cells	-1.3	4.98	0.0026	ENSSSCG00000011022	protein coding
MORN4	Satellite stem cells	-1.29	3.47	0.0235	ENSSSCG00000036954	protein coding
RBP4	Satellite stem cells	-1.29	4.36	0.0262	ENSSSCG00000010479	protein coding
ENSSSCG00000024009	Satellite stem cells	-1.27	3.47	0.0311	ENSSSCG00000024009	protein coding
KIAA0513	Satellite stem cells	-1.26	6.79	0.000656	ENSSSCG00000002666	protein coding
KSR1	Satellite stem cells	-1.26	3.6	0.0304	ENSSSCG00000017753	protein coding
MFSD4B	Satellite stem cells	-1.26	3.26	0.0176	ENSSSCG00000041409	protein coding
MSTRG.42062	Satellite stem cells	-1.26	3.9	0.00716	MSTRG.42062	lncRNA
MYBPH	Satellite stem cells	-1.26	4.74	0.0199	ENSSSCG00000030681	protein coding
SASS6	Satellite stem cells	-1.26	4.77	0.0162	ENSSSCG00000028821	protein coding
ZBTB16	Satellite stem cells	-1.26	7.71	0.011	ENSSSCG00000030095	protein coding
HR	Satellite stem cells	-1.25	4.26	0.0138	ENSSSCG00000009616	protein coding
FANCA	Satellite stem cells	-1.24	4.11	0.007	ENSSSCG00000024687	protein coding
MSTRG.33951	Satellite stem cells	-1.23	4.09	0.00755	MSTRG.33951	lncRNA
MSTRG.6312	Satellite stem cells	-1.23	3.4	0.0161	MSTRG.6312	lncRNA
ENSSSCG00000051726	Satellite stem cells	-1.22	3.84	0.0403	ENSSSCG00000051726	lncRNA
MSTRG.27930	Satellite stem cells	-1.22	3.59	0.0277	MSTRG.27930	lncRNA
MSTRG.42807	Satellite stem cells	-1.22	3.27	0.0145	MSTRG.42807	lncRNA
ZNF624	Satellite stem cells	-1.22	3.26	0.0315	ENSSSCG00000025254	protein coding
AGT	Satellite stem cells	-1.21	5.78	0.00861	ENSSSCG00000010184	protein coding
ANO1	Satellite stem cells	-1.21	4.11	0.0106	ENSSSCG00000024158	protein coding
B2M	Satellite stem cells	-1.21	9.63	0.0345	ENSSSCG00000004687	protein coding
MSTRG.14028	Satellite stem cells	-1.21	3.33	0.0198	MSTRG.14028	lncRNA
PSME2	Satellite stem cells	-1.21	7.08	0.0409	ENSSSCG00000002004	protein coding
CAMKK1	Satellite stem cells	-1.2	3.12	0.03	ENSSSCG00000017873	protein coding
DNAAF5	Satellite stem cells	-1.2	3.28	0.03	ENSSSCG00000007543	protein coding
ENSSSCG00000031608	Satellite stem cells	-1.2	4.29	0.0483	ENSSSCG00000031608	protein coding
ESRRG	Satellite stem cells	-1.2	4.2	0.00844	ENSSSCG00000010814	protein coding
MSTRG.16133	Satellite stem cells	-1.2	3.58	0.0423	MSTRG.16133	lncRNA
DUOXA2	Satellite stem cells	-1.19	3.43	0.0395	ENSSSCG00000004676	protein coding
PROSER2	Satellite stem cells	-1.19	3.59	0.0139	ENSSSCG00000022247	protein coding
TMCO4	Satellite stem cells	-1.19	3.41	0.0176	ENSSSCG00000027489	protein coding
MSTRG.25856	Satellite stem cells	-1.18	5.38	0.0117	MSTRG.25856	lncRNA
PLXNA1	Satellite stem cells	-1.18	3.32	0.031	ENSSSCG00000024001	protein coding

MSTRG.17703	Satellite stem cells	-1.17	5.23	0.0123	MSTRG.17703	lncRNA
NTF3	Satellite stem cells	-1.17	5.03	0.00109	ENSSSCG00000033544	protein coding
PDE12	Satellite stem cells	-1.17	4.07	0.00915	ENSSSCG00000011465	protein coding
PIAS4	Satellite stem cells	-1.17	3.32	0.024	ENSSSCG00000022066	protein coding
PSMB10	Satellite stem cells	-1.17	5.39	0.0414	ENSSSCG00000031781	protein coding
LANCL3	Satellite stem cells	-1.16	3.8	0.0232	ENSSSCG00000037269	protein coding
TUBGCP5	Satellite stem cells	-1.16	3.96	0.0172	ENSSSCG00000027105	protein coding
TMEFF2	Satellite stem cells	-1.15	6.34	0.00748	ENSSSCG00000016064	protein coding
KLF15	Satellite stem cells	-1.14	3.9	0.0442	ENSSSCG00000036742	protein coding
MSTRG.42217	Satellite stem cells	-1.14	3.57	0.0395	MSTRG.42217	lncRNA
PHRF1	Satellite stem cells	-1.14	3.35	0.0255	ENSSSCG00000029723	protein coding
NADK	Satellite stem cells	-1.12	3.81	0.0258	ENSSSCG00000038967	protein coding
SERINC2	Satellite stem cells	-1.12	6.76	0.0042	ENSSSCG00000023585	protein coding
SNX8	Satellite stem cells	-1.12	4.6	0.00917	ENSSSCG00000040977	protein coding
ENSSSCG00000006510	Satellite stem cells	-1.11	4.41	0.0141	ENSSSCG00000006510	protein coding
MSTRG.14448	Satellite stem cells	-1.11	5.21	0.000992	MSTRG.14448	lncRNA
MSTRG.29775	Satellite stem cells	-1.11	5.37	0.0026	MSTRG.29775	lncRNA
LRRC17	Satellite stem cells	-1.09	5.3	0.00819	ENSSSCG00000036452	protein coding
MSTRG.16656	Satellite stem cells	-1.09	3.66	0.0298	MSTRG.16656	lncRNA
TCAF1	Satellite stem cells	-1.09	5.16	0.00543	ENSSSCG00000016453	protein coding
CD44	Satellite stem cells	-1.07	3.92	0.00794	ENSSSCG00000013297	protein coding
ENSSSCG00000001341	Satellite stem cells	-1.07	5.42	0.0354	ENSSSCG00000001341	protein coding
ENSSSCG00000009543	Satellite stem cells	-1.07	4.51	0.0336	ENSSSCG00000009543	protein coding
HIST1H3E	Satellite stem cells	-1.07	3.37	0.043	ENSSSCG00000036727	protein coding
KCNQ1	Satellite stem cells	-1.07	4.13	0.0236	ENSSSCG00000039556	protein coding
MSTRG.19111	Satellite stem cells	-1.07	4.06	0.00823	MSTRG.19111	lncRNA
MSTRG.24014	Satellite stem cells	-1.07	3.44	0.0214	MSTRG.24014	lncRNA
SEMA4D	Satellite stem cells	-1.07	6.64	0.0022	ENSSSCG00000009584	protein coding
SYVN1	Satellite stem cells	-1.07	3.71	0.0155	ENSSSCG00000027057	protein coding
ACTC1	Satellite stem cells	-1.05	8.56	0.0399	ENSSSCG00000004803	protein coding
ATOH8	Satellite stem cells	-1.05	3.79	0.0421	ENSSSCG00000008230	protein coding
ENSSSCG00000003352	Satellite stem cells	-1.05	4.37	0.00576	ENSSSCG00000003352	protein coding
MFAP5	Satellite stem cells	-1.05	7.63	0.00686	ENSSSCG00000031712	protein coding
SENP7	Satellite stem cells	-1.05	3.47	0.023	ENSSSCG00000021791	protein coding
FEZ2	Satellite stem cells	-1.04	4.42	0.0245	ENSSSCG00000008503	protein coding
GRIA3	Satellite stem cells	-1.04	4.04	0.0239	ENSSSCG00000012638	protein coding
PLEKHO1	Satellite stem cells	-1.04	3.7	0.0468	ENSSSCG00000022784	protein coding
TARBP1	Satellite stem cells	-1.04	3.33	0.0476	ENSSSCG00000010160	protein coding

DLGAP2	Satellite stem cells	-1.03	4.31	0.0482	ENSSSCG00000015746	protein coding
FAM124A	Satellite stem cells	-1.03	3.53	0.0394	ENSSSCG00000029598	protein coding
MSTRG.49025	Satellite stem cells	-1.03	4.11	0.0477	MSTRG.49025	lncRNA
MSTRG.49624	Satellite stem cells	-1.03	6.31	0.0116	MSTRG.49624	lncRNA
ZCCHC24	Satellite stem cells	-1.03	5.56	0.00659	ENSSSCG00000033074	protein coding
MSTRG.25554	Satellite stem cells	-1.02	4.5	0.00483	MSTRG.25554	lncRNA
NUP210	Satellite stem cells	-1.02	4.69	0.0216	ENSSSCG00000011610	protein coding
BAALC	Satellite stem cells	-1.01	4.79	0.00496	ENSSSCG00000006053	protein coding
RASSF2	Satellite stem cells	-1.01	4.96	0.00254	ENSSSCG00000032601	protein coding
ADCY7	Satellite stem cells	-1	5.19	0.00283	ENSSSCG00000039408	protein coding
ARHGAP26	Satellite stem cells	-1	3.38	0.0498	ENSSSCG00000014399	protein coding
ENSSSCG00000023627	Satellite stem cells	-1	3.62	0.0309	ENSSSCG00000023627	lncRNA
MED12L	Satellite stem cells	-0.994	5	0.0126	ENSSSCG00000011714	protein coding
SERHL2	Satellite stem cells	-0.994	7.82	0.00392	ENSSSCG00000034350	protein coding
PDZRN3	Satellite stem cells	-0.984	6.45	0.013	ENSSSCG00000011521	protein coding
GNAI2	Satellite stem cells	-0.983	4.07	0.0114	ENSSSCG00000011399	protein coding
CEACAM16	Satellite stem cells	-0.977	4.1	0.0167	ENSSSCG00000003081	protein coding
ORC2	Satellite stem cells	-0.97	3.62	0.041	ENSSSCG00000016098	protein coding
FBRSL1	Satellite stem cells	-0.964	4.57	0.0117	ENSSSCG00000023569	protein coding
ABHD15	Satellite stem cells	-0.962	4.97	0.0238	ENSSSCG00000017789	protein coding
DAPK1	Satellite stem cells	-0.959	5.79	0.0265	ENSSSCG00000010949	protein coding
HPCAL1	Satellite stem cells	-0.953	5.15	0.00998	ENSSSCG00000003663	protein coding
WDR54	Satellite stem cells	-0.952	3.78	0.0233	ENSSSCG00000008282	protein coding
MTSS1	Satellite stem cells	-0.946	4.75	0.0183	ENSSSCG00000005975	protein coding
SETDB1	Satellite stem cells	-0.944	3.94	0.0404	ENSSSCG00000006645	protein coding
MSTRG.8483	Satellite stem cells	-0.94	4.29	0.0485	MSTRG.8483	lncRNA
PLCG1	Satellite stem cells	-0.94	4.21	0.0262	ENSSSCG00000007356	protein coding
CSPG5	Satellite stem cells	-0.933	3.36	0.0382	ENSSSCG00000039224	protein coding
MSTRG.29959	Satellite stem cells	-0.933	4.93	0.00516	MSTRG.29959	lncRNA
WTIP	Satellite stem cells	-0.93	4.25	0.0141	ENSSSCG00000039166	protein coding
ACOT11	Satellite stem cells	-0.929	4.24	0.0134	ENSSSCG00000037559	protein coding
PFN1	Satellite stem cells	-0.928	10.3	0.0043	ENSSSCG00000017907	protein coding
MSTRG.48556	Satellite stem cells	-0.924	4.89	0.0117	MSTRG.48556	lncRNA
HSPBAP1	Satellite stem cells	-0.923	3.94	0.0386	ENSSSCG00000011873	protein coding
ACTB	Satellite stem cells	-0.91	4.49	0.0121	ENSSSCG00000007585	protein coding
ENSSSCG00000042756	Satellite stem cells	-0.909	3.72	0.0479	ENSSSCG00000042756	protein coding
KAZN	Satellite stem cells	-0.907	4.57	0.0166	ENSSSCG00000037676	protein coding
ACTN2	Satellite stem cells	-0.904	6.24	0.0213	ENSSSCG00000010144	protein coding

MSTRG.24269	Satellite stem cells	-0.9	3.46	0.0404	MSTRG.24269	lncRNA
UBE2Q1	Satellite stem cells	-0.898	4.31	0.0456	ENSSSCG00000006544	protein coding
ISYNA1	Satellite stem cells	-0.897	6.83	0.00493	ENSSSCG00000040524	protein coding
BCAM	Satellite stem cells	-0.896	4.53	0.0391	ENSSSCG00000020657	protein coding
DISC1	Satellite stem cells	-0.89	4.4	0.0208	ENSSSCG00000010170	protein coding
BCAR3	Satellite stem cells	-0.883	5.99	0.0044	ENSSSCG00000006893	protein coding
EIF4G1	Satellite stem cells	-0.881	5.03	0.0135	ENSSSCG00000030255	protein coding
EMG1	Satellite stem cells	-0.878	6.59	0.0112	ENSSSCG00000026812	protein coding
ARHGAP31	Satellite stem cells	-0.877	5.72	0.0264	ENSSSCG00000026224	protein coding
GALK1	Satellite stem cells	-0.876	4.99	0.0233	ENSSSCG00000017203	protein coding
EDRF1	Satellite stem cells	-0.872	4.03	0.0254	ENSSSCG00000010741	protein coding
OTUD4	Satellite stem cells	-0.868	4.84	0.0177	ENSSSCG00000009042	protein coding
ENSSSCG00000010860	Satellite stem cells	-0.867	3.85	0.0493	ENSSSCG00000010860	protein coding
MSTRG.1952	Satellite stem cells	-0.866	4.8	0.0409	MSTRG.1952	lncRNA
TJAP1	Satellite stem cells	-0.863	3.9	0.0479	ENSSSCG00000001688	protein coding
EBF4	Satellite stem cells	-0.854	4.32	0.0338	ENSSSCG00000007171	protein coding
SIRT5	Satellite stem cells	-0.852	5.03	0.0172	ENSSSCG00000031129	protein coding
ENSSSCG00000015083	Satellite stem cells	-0.851	6.58	0.00153	ENSSSCG00000015083	protein coding
MSTRG.8245	Satellite stem cells	-0.85	3.87	0.0384	MSTRG.8245	lncRNA
ENSSSCG00000038708	Satellite stem cells	-0.849	4.64	0.0168	ENSSSCG00000038708	protein coding
FAM20B	Satellite stem cells	-0.844	4.34	0.0415	ENSSSCG00000015524	protein coding
GLCCI1	Satellite stem cells	-0.838	7.15	0.00769	ENSSSCG00000015345	protein coding
MCAM	Satellite stem cells	-0.838	6.38	0.00247	ENSSSCG00000015116	protein coding
MSTRG.29958	Satellite stem cells	-0.836	4.85	0.00681	MSTRG.29958	lncRNA
FOSB	Satellite stem cells	-0.834	8.92	0.00179	ENSSSCG00000031616	protein coding
ZNF207	Satellite stem cells	-0.831	4.86	0.00989	ENSSSCG00000017732	protein coding
SETX	Satellite stem cells	-0.83	4.84	0.0142	ENSSSCG00000005724	protein coding
ELOA	Satellite stem cells	-0.828	4.76	0.0163	ENSSSCG00000025440	protein coding
DBN1	Satellite stem cells	-0.827	6.49	0.00958	ENSSSCG00000040706	protein coding
ENSSSCG00000042225	Satellite stem cells	-0.823	5.18	0.0074	ENSSSCG00000042225	lncRNA
SRSF7	Satellite stem cells	-0.82	8.02	0.00248	ENSSSCG00000008484	protein coding
MSTRG.29555	Satellite stem cells	-0.819	6.35	0.0168	MSTRG.29555	lncRNA
CNKSR2	Satellite stem cells	-0.818	6.33	0.0287	ENSSSCG00000012164	protein coding
MSTRG.43974	Satellite stem cells	-0.816	6.02	0.00581	MSTRG.43974	lncRNA
TAPT1	Satellite stem cells	-0.816	4.67	0.0311	ENSSSCG00000021828	protein coding
OGFRL1	Satellite stem cells	-0.815	4.44	0.031	ENSSSCG00000004279	protein coding
PIP5K1B	Satellite stem cells	-0.807	6.17	0.0112	ENSSSCG00000027161	protein coding
KLF13	Satellite stem cells	-0.803	6.38	0.035	ENSSSCG00000026006	protein coding

ZMIZ1	Satellite stem cells	-0.802	5.09	0.00573	ENSSSCG00000010329	protein coding
ENSSSCG00000001229	Satellite stem cells	-0.801	8.03	0.0168	ENSSSCG00000001229	protein coding
GLI2	Satellite stem cells	-0.8	7.71	0.012	ENSSSCG00000015733	protein coding
ZNF710	Satellite stem cells	-0.796	3.87	0.041	ENSSSCG00000021361	protein coding
AGPAT3	Satellite stem cells	-0.794	5.85	0.00647	ENSSSCG00000038081	protein coding
MSTRG.30004	Satellite stem cells	-0.792	5.01	0.024	MSTRG.30004	lncRNA
ARID1A	Satellite stem cells	-0.791	4.17	0.0352	ENSSSCG00000003559	protein coding
ENSSSCG00000011859	Satellite stem cells	-0.79	4.6	0.0155	ENSSSCG00000011859	protein coding
ENSSSCG00000004830	Satellite stem cells	-0.787	5.85	0.0282	ENSSSCG00000004830	protein coding
HS6ST1	Satellite stem cells	-0.786	4.07	0.0449	ENSSSCG00000022197	protein coding
TBRG4	Satellite stem cells	-0.786	5.52	0.00741	ENSSSCG00000016732	protein coding
MSTRG.14856	Satellite stem cells	-0.783	4.33	0.0298	MSTRG.14856	lncRNA
STX18	Satellite stem cells	-0.783	5.45	0.0301	ENSSSCG00000038731	protein coding
TMEM63A	Satellite stem cells	-0.78	4.09	0.049	ENSSSCG00000010854	protein coding
FLNC	Satellite stem cells	-0.777	6.5	0.00246	ENSSSCG00000016578	protein coding
NUP133	Satellite stem cells	-0.777	5.07	0.00874	ENSSSCG00000010189	protein coding
TOR3A	Satellite stem cells	-0.777	5.76	0.00766	ENSSSCG00000015525	protein coding
NOB1	Satellite stem cells	-0.776	5.36	0.0215	ENSSSCG00000002753	protein coding
KDM4B	Satellite stem cells	-0.775	4	0.0487	ENSSSCG00000024570	protein coding
FKBP5	Satellite stem cells	-0.773	7.8	0.0338	ENSSSCG00000001549	protein coding
ENSSSCG00000032469	Satellite stem cells	-0.771	4.32	0.0388	ENSSSCG00000032469	protein coding
HEATR6	Satellite stem cells	-0.771	4.57	0.0307	ENSSSCG00000038478	protein coding
GGA2	Satellite stem cells	-0.769	4.26	0.0433	ENSSSCG00000010798	protein coding
CLSPN	Satellite stem cells	-0.767	5.91	0.0295	ENSSSCG00000003628	protein coding
POR	Satellite stem cells	-0.765	5.73	0.0239	ENSSSCG00000038320	protein coding
TUBA8	Satellite stem cells	-0.76	5.23	0.0158	ENSSSCG00000027190	protein coding
TSPAN15	Satellite stem cells	-0.759	7.13	0.0393	ENSSSCG00000036152	protein coding
PDE1C	Satellite stem cells	-0.758	6.54	0.0205	ENSSSCG00000016668	protein coding
RBL1	Satellite stem cells	-0.754	5.01	0.0462	ENSSSCG00000007331	protein coding
RPTOR	Satellite stem cells	-0.754	5.43	0.0208	ENSSSCG00000017139	protein coding
TSPAN14	Satellite stem cells	-0.754	5.14	0.0107	ENSSSCG00000010341	protein coding
SGCD	Satellite stem cells	-0.75	7.97	0.00675	ENSSSCG00000017063	protein coding
ATP6V0D1	Satellite stem cells	-0.744	4.9	0.0449	ENSSSCG00000002776	protein coding
DST	Satellite stem cells	-0.744	7.76	0.0155	ENSSSCG00000001499	protein coding
MAD1L1	Satellite stem cells	-0.741	5.06	0.0241	ENSSSCG00000038766	protein coding
ESS2	Satellite stem cells	-0.736	4.39	0.0341	ENSSSCG00000010114	protein coding
GSTO1	Satellite stem cells	-0.733	6.31	0.0295	ENSSSCG00000022351	protein coding
MAN2A2	Satellite stem cells	-0.733	4.43	0.0395	ENSSSCG00000001819	protein coding

BARD1	Satellite stem cells	-0.731	5.33	0.0269	ENSSSCG00000016170	protein coding
TLN2	Satellite stem cells	-0.731	8.19	0.00551	ENSSSCG00000024088	protein coding
ENSSSCG00000004387	Satellite stem cells	-0.729	7.42	0.0106	ENSSSCG00000004387	protein coding
KIF21A	Satellite stem cells	-0.722	6.24	0.026	ENSSSCG00000000779	protein coding
PLXNA2	Satellite stem cells	-0.719	6.94	0.00826	ENSSSCG00000026412	protein coding
CDH4	Satellite stem cells	-0.718	5.14	0.0176	ENSSSCG00000025784	protein coding
CNP	Satellite stem cells	-0.717	6	0.00724	ENSSSCG00000017420	protein coding
NOP2	Satellite stem cells	-0.717	5.19	0.0203	ENSSSCG00000000696	protein coding
CLPB	Satellite stem cells	-0.715	4.9	0.022	ENSSSCG00000036887	protein coding
CHKA	Satellite stem cells	-0.714	4.56	0.0234	ENSSSCG00000012889	protein coding
PIK3R1	Satellite stem cells	-0.712	7.03	0.0161	ENSSSCG00000016958	protein coding
PPRC1	Satellite stem cells	-0.712	4.92	0.0224	ENSSSCG00000010575	protein coding
STRIP2	Satellite stem cells	-0.71	5.46	0.0385	ENSSSCG00000016567	protein coding
ARFGAP2	Satellite stem cells	-0.709	5.21	0.0271	ENSSSCG00000013245	protein coding
DDX47	Satellite stem cells	-0.708	4.96	0.0458	ENSSSCG00000033149	protein coding
NOL11	Satellite stem cells	-0.708	7.53	0.0242	ENSSSCG00000017274	protein coding
GTF3C2	Satellite stem cells	-0.705	4.2	0.0414	ENSSSCG00000032798	protein coding
EIF2B3	Satellite stem cells	-0.7	5.65	0.0391	ENSSSCG00000029808	protein coding
NCL	Satellite stem cells	-0.695	7.67	0.00603	ENSSSCG00000016275	protein coding
DCAF7	Satellite stem cells	-0.694	5.16	0.0339	ENSSSCG00000017294	protein coding
PRKCB	Satellite stem cells	-0.693	6.19	0.00848	ENSSSCG00000033674	protein coding
RET	Satellite stem cells	-0.693	6.61	0.0333	ENSSSCG00000010199	protein coding
PAXBP1	Satellite stem cells	-0.688	4.97	0.0342	ENSSSCG00000012037	protein coding
SEPTIN9	Satellite stem cells	-0.687	5.51	0.02	ENSSSCG00000017168	protein coding
EP400	Satellite stem cells	-0.686	4.84	0.0352	ENSSSCG00000028018	protein coding
CCNQ	Satellite stem cells	-0.684	4.59	0.0318	ENSSSCG00000012770	protein coding
ENSSSCG00000040571	Satellite stem cells	-0.683	5.96	0.0106	ENSSSCG00000040571	protein coding
SLC29A1	Satellite stem cells	-0.68	6.76	0.0111	ENSSSCG00000001700	protein coding
MTBP	Satellite stem cells	-0.674	5.52	0.0343	ENSSSCG00000005995	protein coding
ENSSSCG00000027115	Satellite stem cells	-0.672	5.42	0.0219	ENSSSCG00000027115	protein coding
TCERG1	Satellite stem cells	-0.67	6.08	0.00762	ENSSSCG00000014416	protein coding
DDX18	Satellite stem cells	-0.669	5.3	0.0491	ENSSSCG00000015712	protein coding
ENSSSCG00000006725	Satellite stem cells	-0.666	5.58	0.0427	ENSSSCG00000006725	protein coding
SMO	Satellite stem cells	-0.665	6.92	0.00658	ENSSSCG00000016569	protein coding
PHLDB1	Satellite stem cells	-0.664	5.16	0.0248	ENSSSCG00000024578	protein coding
NAA60	Satellite stem cells	-0.661	5.45	0.0188	ENSSSCG00000021065	protein coding
HES6	Satellite stem cells	-0.66	5.14	0.0222	ENSSSCG00000016342	protein coding
LMNB2	Satellite stem cells	-0.659	6.78	0.0405	ENSSSCG00000026784	protein coding

MMP16	Satellite stem cells	-0.656	5.34	0.0396	ENSSSCG00000006132	protein coding
AKR1B1	Satellite stem cells	-0.653	7.85	0.00597	ENSSSCG00000040608	protein coding
NOL9	Satellite stem cells	-0.65	4.33	0.0489	ENSSSCG00000030253	protein coding
RASSF4	Satellite stem cells	-0.65	7.33	0.00754	ENSSSCG00000029662	protein coding
CPT1A	Satellite stem cells	-0.649	5.45	0.0432	ENSSSCG00000012880	protein coding
ILF2	Satellite stem cells	-0.649	7.92	0.00742	ENSSSCG00000028553	protein coding
ARMC2	Satellite stem cells	-0.644	5.24	0.044	ENSSSCG00000004388	protein coding
ENSSSCG00000009914	Satellite stem cells	-0.644	4.99	0.0327	ENSSSCG00000009914	protein coding
ENSSSCG00000006928	Satellite stem cells	-0.641	4.99	0.0214	ENSSSCG00000006928	protein coding
ASH2L	Satellite stem cells	-0.636	5.13	0.0375	ENSSSCG00000021898	protein coding
CDH15	Satellite stem cells	-0.631	7.33	0.0328	ENSSSCG00000002642	protein coding
ENSSSCG00000040701	Satellite stem cells	-0.63	7.28	0.0146	ENSSSCG00000040701	protein coding
MACF1	Satellite stem cells	-0.629	6.47	0.048	ENSSSCG00000003654	protein coding
ESF1	Satellite stem cells	-0.626	5.93	0.0155	ENSSSCG00000007077	protein coding
TUBB2B	Satellite stem cells	-0.626	8.52	0.0152	ENSSSCG00000033655	protein coding
ENSSSCG00000016119	Satellite stem cells	-0.625	7.29	0.0138	ENSSSCG00000016119	protein coding
TPR	Satellite stem cells	-0.625	6.45	0.0382	ENSSSCG00000015576	protein coding
ZCCHC14	Satellite stem cells	-0.625	5.17	0.0291	ENSSSCG00000002656	protein coding
TRAK1	Satellite stem cells	-0.617	6.08	0.0429	ENSSSCG00000011278	protein coding
SRRM2	Satellite stem cells	-0.613	6.92	0.0319	ENSSSCG00000023304	protein coding
KLHL21	Satellite stem cells	-0.612	4.95	0.0344	ENSSSCG00000003379	protein coding
PFKFB3	Satellite stem cells	-0.612	4.83	0.0318	ENSSSCG00000011133	protein coding
OSBPL11	Satellite stem cells	-0.611	6.52	0.0285	ENSSSCG00000026753	protein coding
WNT9A	Satellite stem cells	-0.609	4.75	0.0452	ENSSSCG00000024671	protein coding
RRAGD	Satellite stem cells	-0.608	5.62	0.0314	ENSSSCG00000004318	protein coding
EEFSEC	Satellite stem cells	-0.607	5.1	0.0444	ENSSSCG00000011619	protein coding
FGFR1	Satellite stem cells	-0.601	6.46	0.0128	ENSSSCG00000015815	protein coding
SRRT	Satellite stem cells	-0.595	6.01	0.0237	ENSSSCG00000024789	protein coding
FANCI	Satellite stem cells	-0.589	5.68	0.0278	ENSSSCG00000001837	protein coding
FOXO1	Satellite stem cells	-0.587	5.96	0.0293	ENSSSCG00000009370	protein coding
PLCB4	Satellite stem cells	-0.582	5.96	0.0339	ENSSSCG00000007058	protein coding
NFIC	Satellite stem cells	-0.581	9.08	0.00776	ENSSSCG00000036537	protein coding
PTPA	Satellite stem cells	-0.577	5.73	0.0277	ENSSSCG00000005672	protein coding
MRPL51	Satellite stem cells	-0.576	6.12	0.0258	ENSSSCG00000039662	protein coding
EBF3	Satellite stem cells	-0.574	5.75	0.0428	ENSSSCG00000010757	protein coding
RASA3	Satellite stem cells	-0.574	5.23	0.036	ENSSSCG00000009567	protein coding
ADSL	Satellite stem cells	-0.573	5.37	0.0384	ENSSSCG00000000077	protein coding
DENND1A	Satellite stem cells	-0.572	6.67	0.0119	ENSSSCG00000005585	protein coding

EIF2AK4	Satellite stem cells	-0.571	5.84	0.031	ENSSSCG00000039061	protein coding
UBR4	Satellite stem cells	-0.569	6.39	0.0338	ENSSSCG00000003488	protein coding
CLMP	Satellite stem cells	-0.565	5.6	0.0255	ENSSSCG00000022961	protein coding
SMARCA4	Satellite stem cells	-0.565	6.05	0.0352	ENSSSCG00000013629	protein coding
C16orf91	Satellite stem cells	-0.561	5.3	0.0461	ENSSSCG00000038882	protein coding
MICAL3	Satellite stem cells	-0.549	5.79	0.0444	ENSSSCG00000000770	protein coding
CLIP1	Satellite stem cells	-0.544	5.48	0.0312	ENSSSCG00000009793	protein coding
EDA	Satellite stem cells	-0.544	6.12	0.0368	ENSSSCG00000021647	protein coding
CCDC59	Satellite stem cells	-0.543	6.04	0.0474	ENSSSCG00000038987	protein coding
PALM2AKAP2	Satellite stem cells	-0.542	8.57	0.0278	ENSSSCG00000033120	protein coding
TBC1D8	Satellite stem cells	-0.526	7.51	0.0259	ENSSSCG00000008169	protein coding
XPO5	Satellite stem cells	-0.526	5.68	0.035	ENSSSCG00000001679	protein coding
ENOX1	Satellite stem cells	-0.525	7.12	0.0251	ENSSSCG00000021818	protein coding
RNF19B	Satellite stem cells	-0.521	5.39	0.0456	ENSSSCG00000025206	protein coding
NDUFAB1	Satellite stem cells	-0.507	7.91	0.0176	ENSSSCG00000010795	protein coding
POFUT1	Satellite stem cells	-0.502	5.71	0.0403	ENSSSCG00000007244	protein coding
RPL7L1	Satellite stem cells	-0.498	6.62	0.0373	ENSSSCG00000026472	protein coding
C20orf27	Satellite stem cells	-0.494	6.22	0.0407	ENSSSCG00000035666	protein coding
ARHGAP21	Satellite stem cells	-0.482	7.61	0.0325	ENSSSCG00000011074	protein coding
INO80	Satellite stem cells	-0.482	6.08	0.0417	ENSSSCG00000004753	protein coding
TXNIP	Satellite stem cells	-0.474	8.07	0.0361	ENSSSCG00000031262	protein coding
HSPG2	Satellite stem cells	-0.457	8.49	0.0389	ENSSSCG00000003514	protein coding
NEXN	Satellite stem cells	-0.441	6.91	0.0388	ENSSSCG00000003768	protein coding
MSTRG.41285	Satellite stem cells	-0.44	10.1	0.0424	MSTRG.41285	lncRNA
DDX21	Satellite stem cells	-0.433	6.98	0.0498	ENSSSCG00000010247	protein coding
NAA20	Satellite stem cells	-0.425	7.32	0.0394	ENSSSCG00000028132	protein coding
SUN1	Satellite stem cells	-0.422	6.92	0.049	ENSSSCG00000007544	protein coding
ENSSSCG00000004970	Satellite stem cells	0.287	13.3	0.044	ENSSSCG00000004970	protein coding
ENSSSCG00000039544	Satellite stem cells	0.304	11.8	0.0446	ENSSSCG00000039544	protein coding
RPL7	Satellite stem cells	0.323	12.1	0.0226	ENSSSCG00000032111	protein coding
ENSSSCG00000031838	Satellite stem cells	0.335	12.3	0.0327	ENSSSCG00000031838	protein coding
RPL37	Satellite stem cells	0.337	12.1	0.0376	ENSSSCG00000033326	protein coding
ENSSSCG00000012427	Satellite stem cells	0.355	11.9	0.0142	ENSSSCG00000012427	protein coding
HNRNPDL	Satellite stem cells	0.376	9.33	0.0444	ENSSSCG00000037245	protein coding
COX7A2L	Satellite stem cells	0.396	8.23	0.047	ENSSSCG00000008466	protein coding
RPS15A	Satellite stem cells	0.397	12.2	0.0135	ENSSSCG00000035768	protein coding
RPS3A	Satellite stem cells	0.414	12.2	0.0142	ENSSSCG00000009019	protein coding
ENSSSCG00000020725	Satellite stem cells	0.417	12.1	0.00786	ENSSSCG00000020725	protein coding

MSTRG.30002	Satellite stem cells	0.42	9.19	0.0325	MSTRG.30002	lncRNA
PAIP2	Satellite stem cells	0.425	8.23	0.0327	ENSSSCG00000026606	protein coding
CDC42	Satellite stem cells	0.444	8.14	0.0258	ENSSSCG00000003520	protein coding
TPM4	Satellite stem cells	0.454	8.93	0.0467	ENSSSCG00000013849	protein coding
DERL1	Satellite stem cells	0.461	6.35	0.0431	ENSSSCG00000033690	protein coding
HBP1	Satellite stem cells	0.466	6.78	0.0345	ENSSSCG00000035673	protein coding
RPS28	Satellite stem cells	0.466	12.1	0.0135	ENSSSCG00000013597	protein coding
UCHL5	Satellite stem cells	0.468	6.6	0.0387	ENSSSCG00000010802	protein coding
C5orf15	Satellite stem cells	0.475	6.25	0.0334	ENSSSCG00000032323	protein coding
LAPTM4A	Satellite stem cells	0.478	8.23	0.0301	ENSSSCG00000008602	protein coding
RCAN1	Satellite stem cells	0.489	8.42	0.042	ENSSSCG00000012050	protein coding
TBC1D15	Satellite stem cells	0.492	6.71	0.0335	ENSSSCG00000000512	protein coding
PTMA	Satellite stem cells	0.495	12.6	0.00566	ENSSSCG00000037274	protein coding
SYPL1	Satellite stem cells	0.495	6.8	0.0263	ENSSSCG00000015434	protein coding
CD164	Satellite stem cells	0.499	7.54	0.0447	ENSSSCG00000004414	protein coding
ARHGAP28	Satellite stem cells	0.5	5.85	0.0394	ENSSSCG00000021612	protein coding
ENSSSCG00000037509	Satellite stem cells	0.503	6.16	0.0305	ENSSSCG00000037509	protein coding
PRTFDC1	Satellite stem cells	0.505	7.54	0.0176	ENSSSCG00000028135	protein coding
ENSSSCG00000029857	Satellite stem cells	0.511	7.27	0.0226	ENSSSCG00000029857	protein coding
RPL36A-HNRNPH2	Satellite stem cells	0.515	11.1	0.00551	ENSSSCG00000039046	protein coding
CHMP4B	Satellite stem cells	0.516	6.13	0.0363	ENSSSCG00000007272	protein coding
MSTRG.39186	Satellite stem cells	0.523	9.12	0.0321	MSTRG.39186	lncRNA
AIMP1	Satellite stem cells	0.524	7.59	0.0193	ENSSSCG00000024168	protein coding
NDRG1	Satellite stem cells	0.524	5.73	0.0498	ENSSSCG00000005944	protein coding
MMADHC	Satellite stem cells	0.525	6.35	0.0341	ENSSSCG00000028646	protein coding
PLEKHA5	Satellite stem cells	0.529	6.13	0.0229	ENSSSCG00000000588	protein coding
COX7C	Satellite stem cells	0.531	9.44	0.0403	ENSSSCG00000040325	protein coding
SNRNP27	Satellite stem cells	0.532	6.98	0.0228	ENSSSCG00000025674	protein coding
MRPL39	Satellite stem cells	0.533	6.36	0.041	ENSSSCG00000012021	protein coding
RAB18	Satellite stem cells	0.535	6.54	0.0266	ENSSSCG00000028655	protein coding
SPAG8	Satellite stem cells	0.536	6.58	0.0398	ENSSSCG00000025842	protein coding
ENSSSCG00000012382	Satellite stem cells	0.54	5.67	0.0466	ENSSSCG00000012382	protein coding
CRK	Satellite stem cells	0.541	9.39	0.0137	ENSSSCG00000038989	protein coding
SELENOH	Satellite stem cells	0.543	6.97	0.0251	ENSSSCG00000032364	protein coding
UBE2B	Satellite stem cells	0.547	7.18	0.0375	ENSSSCG00000038953	protein coding
CLCN5	Satellite stem cells	0.548	7.72	0.0426	ENSSSCG00000023026	protein coding
SNX14	Satellite stem cells	0.553	4.96	0.0491	ENSSSCG00000004293	protein coding
ACADL	Satellite stem cells	0.557	6.18	0.0328	ENSSSCG00000016156	protein coding

FBXL5	Satellite stem cells	0.56	5.76	0.0329	ENSSSCG00000008740	protein coding
VPS51	Satellite stem cells	0.562	5.28	0.0387	ENSSSCG00000013005	protein coding
MFF	Satellite stem cells	0.564	6.83	0.029	ENSSSCG00000031572	protein coding
PNRC1	Satellite stem cells	0.57	7.9	0.02	ENSSSCG00000039953	protein coding
RASA1	Satellite stem cells	0.573	6.09	0.0239	ENSSSCG00000014146	protein coding
SELENBP1	Satellite stem cells	0.578	5.45	0.0407	ENSSSCG00000006624	protein coding
CETN3	Satellite stem cells	0.583	6.23	0.0292	ENSSSCG00000014151	protein coding
GPX4	Satellite stem cells	0.585	7.17	0.0136	ENSSSCG00000024052	protein coding
DNAJB4	Satellite stem cells	0.587	7.29	0.0231	ENSSSCG00000003766	protein coding
TBCA	Satellite stem cells	0.594	8.28	0.0154	ENSSSCG00000014100	protein coding
CLCN3	Satellite stem cells	0.595	4.88	0.0423	ENSSSCG00000009713	protein coding
SLC38A6	Satellite stem cells	0.595	5.22	0.0251	ENSSSCG00000005092	protein coding
ITGAV	Satellite stem cells	0.596	6.61	0.0206	ENSSSCG00000016027	protein coding
IAH1	Satellite stem cells	0.598	5.96	0.0253	ENSSSCG00000008640	protein coding
NRAS	Satellite stem cells	0.598	5.2	0.0424	ENSSSCG00000032477	protein coding
SNX25	Satellite stem cells	0.603	5.89	0.0257	ENSSSCG00000015789	protein coding
COL19A1	Satellite stem cells	0.604	6.3	0.0308	ENSSSCG00000044472	protein coding
P3H2	Satellite stem cells	0.608	6.76	0.0348	ENSSSCG00000011813	protein coding
ENSSSCG00000033744	Satellite stem cells	0.621	6.61	0.0493	ENSSSCG00000033744	protein coding
ENSSSCG00000046054	Satellite stem cells	0.621	7.72	0.0256	ENSSSCG00000046054	lncRNA
ENSSSCG00000030849	Satellite stem cells	0.622	11.9	0.000974	ENSSSCG00000030849	protein coding
MSTRG.3217	Satellite stem cells	0.623	5.99	0.0209	MSTRG.3217	lncRNA
EMP1	Satellite stem cells	0.628	7.51	0.00533	ENSSSCG00000036723	protein coding
MSTRG.17288	Satellite stem cells	0.628	5.76	0.0258	MSTRG.17288	lncRNA
ZFP36L1	Satellite stem cells	0.629	7.8	0.00644	ENSSSCG00000028304	protein coding
ZNHIT1	Satellite stem cells	0.629	6.75	0.0273	ENSSSCG00000033982	protein coding
CITED2	Satellite stem cells	0.633	6.63	0.0487	ENSSSCG00000004142	protein coding
SUMO1	Satellite stem cells	0.633	7.41	0.00787	ENSSSCG00000035842	protein coding
TPGS2	Satellite stem cells	0.633	4.83	0.0448	ENSSSCG00000026953	protein coding
ZNF277	Satellite stem cells	0.634	6.45	0.039	ENSSSCG00000025910	protein coding
KAT8	Satellite stem cells	0.638	5.43	0.0223	ENSSSCG00000031247	protein coding
PDCD4	Satellite stem cells	0.638	5.97	0.0253	ENSSSCG00000010627	protein coding
C19orf53	Satellite stem cells	0.642	6.63	0.0453	ENSSSCG00000013757	protein coding
GNAL	Satellite stem cells	0.642	5.14	0.0323	ENSSSCG00000022163	protein coding
RUNX1	Satellite stem cells	0.642	9	0.00745	ENSSSCG00000035537	protein coding
SLC43A2	Satellite stem cells	0.642	5.89	0.0318	ENSSSCG00000024108	protein coding
MSTRG.180	Satellite stem cells	0.643	6.97	0.0474	MSTRG.180	lncRNA
DLD	Satellite stem cells	0.646	6.4	0.014	ENSSSCG00000015443	protein coding

TRMT1	Satellite stem cells	0.649	4.81	0.0378	ENSSSCG00000013744	protein coding
STON2	Satellite stem cells	0.655	6.18	0.0207	ENSSSCG00000002412	protein coding
MSTRG.1135	Satellite stem cells	0.656	7.02	0.0149	MSTRG.1135	lncRNA
GCNT1	Satellite stem cells	0.658	5.01	0.0311	ENSSSCG00000005277	protein coding
ENSSSCG00000022128	Satellite stem cells	0.659	4.67	0.0432	ENSSSCG00000022128	protein coding
PDPN	Satellite stem cells	0.664	6.26	0.0322	ENSSSCG00000003451	protein coding
TRIB1	Satellite stem cells	0.666	5.97	0.0188	ENSSSCG00000038500	protein coding
UMAD1	Satellite stem cells	0.668	4.35	0.0464	ENSSSCG00000036169	protein coding
MSTRG.41067	Satellite stem cells	0.672	5.5	0.0279	MSTRG.41067	lncRNA
BNIP3L	Satellite stem cells	0.675	5.81	0.0241	ENSSSCG00000009658	protein coding
CHURC1	Satellite stem cells	0.676	4.93	0.0335	ENSSSCG00000022147	protein coding
GABARAPL1	Satellite stem cells	0.676	6.09	0.0201	ENSSSCG00000000645	protein coding
SLC31A1	Satellite stem cells	0.676	4.93	0.0246	ENSSSCG00000034278	protein coding
MSTRG.18928	Satellite stem cells	0.678	4.86	0.0409	MSTRG.18928	lncRNA
MSTRG.33764	Satellite stem cells	0.68	4.64	0.0499	MSTRG.33764	lncRNA
UAP1	Satellite stem cells	0.682	5.43	0.0154	ENSSSCG00000006340	protein coding
PAQR8	Satellite stem cells	0.683	5.4	0.014	ENSSSCG00000001750	protein coding
BIRC2	Satellite stem cells	0.692	7.74	0.0147	ENSSSCG00000027529	protein coding
ENSSSCG00000006395	Satellite stem cells	0.694	8.02	0.0201	ENSSSCG00000006395	protein coding
LITAF	Satellite stem cells	0.695	6.33	0.00849	ENSSSCG00000032381	protein coding
HIVEP2	Satellite stem cells	0.702	7.97	0.0067	ENSSSCG00000004138	protein coding
GIPC1	Satellite stem cells	0.705	5.87	0.0169	ENSSSCG00000032842	protein coding
TSHZ1	Satellite stem cells	0.705	5.45	0.0094	ENSSSCG00000004861	protein coding
WSB1	Satellite stem cells	0.705	6.36	0.0101	ENSSSCG00000017752	protein coding
RETREG2	Satellite stem cells	0.707	4.61	0.0291	ENSSSCG00000016207	protein coding
WIPI1	Satellite stem cells	0.712	5.26	0.0269	ENSSSCG00000017260	protein coding
UBXN8	Satellite stem cells	0.714	5.17	0.0138	ENSSSCG00000015843	protein coding
CHSY3	Satellite stem cells	0.717	4.55	0.0264	ENSSSCG00000035069	protein coding
ARMCX3	Satellite stem cells	0.718	4.84	0.0423	ENSSSCG00000040281	protein coding
MSTRG.25395	Satellite stem cells	0.72	4.71	0.0264	MSTRG.25395	lncRNA
GLT8D1	Satellite stem cells	0.721	5.68	0.0109	ENSSSCG00000011448	protein coding
RANBP6	Satellite stem cells	0.721	5.79	0.0438	ENSSSCG00000005204	protein coding
ATG12	Satellite stem cells	0.725	5.19	0.0269	ENSSSCG00000033912	protein coding
JDP2	Satellite stem cells	0.726	5.38	0.0432	ENSSSCG00000035895	protein coding
CLDND1	Satellite stem cells	0.727	5.41	0.0252	ENSSSCG00000011980	protein coding
ERRFI1	Satellite stem cells	0.73	5.4	0.0229	ENSSSCG00000035774	protein coding
EIF2A	Satellite stem cells	0.731	6.23	0.0253	ENSSSCG00000011710	protein coding
CACNA2D1	Satellite stem cells	0.733	6.08	0.0387	ENSSSCG00000015402	protein coding

MSTRG.4110	Satellite stem cells	0.733	5.69	0.018	MSTRG.4110	lncRNA
HCFC2	Satellite stem cells	0.734	4.23	0.0475	ENSSSCG00000000846	protein coding
GPANK1	Satellite stem cells	0.737	4.23	0.036	ENSSSCG00000001413	protein coding
PPT1	Satellite stem cells	0.738	4.79	0.0246	ENSSSCG00000036673	protein coding
CSNK1G3	Satellite stem cells	0.741	4.77	0.0499	ENSSSCG00000014240	protein coding
METTL23	Satellite stem cells	0.742	5.07	0.0312	ENSSSCG00000023139	protein coding
SERINC1	Satellite stem cells	0.743	5.65	0.0177	ENSSSCG00000004237	protein coding
ENSSSCG00000038633	Satellite stem cells	0.747	4.83	0.0284	ENSSSCG00000038633	protein coding
FBXL20	Satellite stem cells	0.747	5.43	0.0156	ENSSSCG00000017506	protein coding
MSTRG.40649	Satellite stem cells	0.755	4.41	0.0314	MSTRG.40649	lncRNA
RNPEP	Satellite stem cells	0.763	5.01	0.0172	ENSSSCG00000034151	protein coding
PRDX5	Satellite stem cells	0.77	5.84	0.0492	ENSSSCG00000013030	protein coding
STX12	Satellite stem cells	0.775	5.48	0.0124	ENSSSCG00000024927	protein coding
NR1H2	Satellite stem cells	0.776	4.88	0.0267	ENSSSCG00000003211	protein coding
CTSF	Satellite stem cells	0.778	5.57	0.00935	ENSSSCG00000038918	protein coding
FUOM	Satellite stem cells	0.78	4.32	0.0397	ENSSSCG00000010791	protein coding
TRPS1	Satellite stem cells	0.781	6.87	0.00287	ENSSSCG00000006018	protein coding
MSTRG.25817	Satellite stem cells	0.782	5.03	0.0164	MSTRG.25817	lncRNA
BTG1	Satellite stem cells	0.784	9.15	0.0197	ENSSSCG00000035790	protein coding
SLC8A1	Satellite stem cells	0.79	5.89	0.0153	ENSSSCG00000036418	protein coding
PNKP	Satellite stem cells	0.796	4.51	0.0496	ENSSSCG00000003195	protein coding
MSTRG.27683	Satellite stem cells	0.799	4.73	0.0278	MSTRG.27683	lncRNA
SAMD4A	Satellite stem cells	0.801	7.34	0.00649	ENSSSCG00000023408	protein coding
DENND10	Satellite stem cells	0.802	4.63	0.0395	ENSSSCG00000010680	protein coding
RBAK	Satellite stem cells	0.807	3.91	0.0401	ENSSSCG00000008668	protein coding
HSPA13	Satellite stem cells	0.81	5.52	0.00708	ENSSSCG00000012007	protein coding
ENSSSCG00000032946	Satellite stem cells	0.816	5.04	0.0404	ENSSSCG00000032946	protein coding
MKRN1	Satellite stem cells	0.818	5.01	0.0112	ENSSSCG00000016498	protein coding
HELZ	Satellite stem cells	0.824	4.63	0.0112	ENSSSCG00000017272	protein coding
SCN7A	Satellite stem cells	0.826	5.24	0.00554	ENSSSCG00000015914	protein coding
MAML2	Satellite stem cells	0.829	6.19	0.00855	ENSSSCG00000034879	protein coding
TMEM117	Satellite stem cells	0.83	4.19	0.0452	ENSSSCG00000000801	protein coding
MSTRG.38999	Satellite stem cells	0.833	5.68	0.0108	MSTRG.38999	lncRNA
RUNX1T1	Satellite stem cells	0.833	5.64	0.0168	ENSSSCG00000006115	protein coding
CASP6	Satellite stem cells	0.836	5.85	0.0193	ENSSSCG00000009140	protein coding
IL1R1	Satellite stem cells	0.838	6.32	0.0335	ENSSSCG00000008162	protein coding
ATXN1	Satellite stem cells	0.842	5.96	0.00484	ENSSSCG00000001065	protein coding
DPYD	Satellite stem cells	0.842	6.91	0.00858	ENSSSCG00000006878	protein coding

KCTD6	Satellite stem cells	0.843	4.19	0.0204	ENSSSCG00000022131	protein coding
FAM171A1	Satellite stem cells	0.844	4.57	0.0147	ENSSSCG00000011047	protein coding
OXCT1	Satellite stem cells	0.844	4.93	0.0249	ENSSSCG00000016863	protein coding
FAP	Satellite stem cells	0.845	4.96	0.0264	ENSSSCG00000015896	protein coding
PIM3	Satellite stem cells	0.85	4.82	0.0471	ENSSSCG00000032936	protein coding
FBXL4	Satellite stem cells	0.851	3.91	0.0303	ENSSSCG00000004347	protein coding
ZNF622	Satellite stem cells	0.853	4.92	0.013	ENSSSCG00000016793	protein coding
ATP6AP2	Satellite stem cells	0.854	5.81	0.0277	ENSSSCG00000012247	protein coding
C15orf40	Satellite stem cells	0.855	5.79	0.0309	ENSSSCG00000030131	protein coding
INSIG1	Satellite stem cells	0.856	4.46	0.0341	ENSSSCG00000016420	protein coding
MSTRG.3143	Satellite stem cells	0.86	4	0.0438	MSTRG.3143	lncRNA
ATP6V1D	Satellite stem cells	0.865	4.38	0.026	ENSSSCG00000033278	protein coding
CCNG1	Satellite stem cells	0.869	7.5	0.00162	ENSSSCG00000017024	protein coding
ENSSSCG00000004666	Satellite stem cells	0.877	4.58	0.0404	ENSSSCG00000004666	protein coding
RBM12B	Satellite stem cells	0.878	4.55	0.0116	ENSSSCG00000006110	protein coding
MSTRG.10109	Satellite stem cells	0.887	4.04	0.046	MSTRG.10109	lncRNA
ZSWIM4	Satellite stem cells	0.889	4.13	0.0322	ENSSSCG00000013758	protein coding
ENSSSCG00000038991	Satellite stem cells	0.891	5.19	0.00332	ENSSSCG00000038991	protein coding
GSTA4	Satellite stem cells	0.891	6.27	0.00918	ENSSSCG00000002627	protein coding
RNF227	Satellite stem cells	0.898	3.81	0.0401	ENSSSCG00000023166	protein coding
MAPK4	Satellite stem cells	0.902	4.34	0.0145	ENSSSCG00000004520	protein coding
PM20D2	Satellite stem cells	0.904	4.92	0.00445	ENSSSCG00000004314	protein coding
MSTRG.31090	Satellite stem cells	0.905	4	0.0417	MSTRG.31090	lncRNA
DNAJA4	Satellite stem cells	0.907	3.57	0.0486	ENSSSCG00000037646	protein coding
ID2	Satellite stem cells	0.911	4.27	0.0115	ENSSSCG00000008645	protein coding
MSTRG.21090	Satellite stem cells	0.911	4.19	0.0488	MSTRG.21090	lncRNA
PDGFRL	Satellite stem cells	0.918	6	0.0182	ENSSSCG00000006988	protein coding
KBTBD3	Satellite stem cells	0.919	3.53	0.0342	ENSSSCG00000014999	protein coding
MSTRG.22894	Satellite stem cells	0.928	5.01	0.0101	MSTRG.22894	lncRNA
TMEM59	Satellite stem cells	0.928	7.22	0.0019	ENSSSCG00000034261	protein coding
HSPH1	Satellite stem cells	0.93	7.96	0.0268	ENSSSCG00000009334	protein coding
MSTRG.1869	Satellite stem cells	0.932	4.1	0.0317	MSTRG.1869	lncRNA
NFKBIE	Satellite stem cells	0.935	4.26	0.0117	ENSSSCG00000001703	protein coding
MDFIC	Satellite stem cells	0.937	6.58	0.0028	ENSSSCG00000021885	protein coding
MSTRG.11691	Satellite stem cells	0.937	4.14	0.0183	MSTRG.11691	lncRNA
RABEPK	Satellite stem cells	0.939	3.59	0.0471	ENSSSCG00000005599	protein coding
XRCC6	Satellite stem cells	0.941	5.1	0.0123	ENSSSCG00000000059	protein coding
MSTRG.45174	Satellite stem cells	0.943	4.44	0.0334	MSTRG.45174	lncRNA

LAMA4	Satellite stem cells	0.95	5.06	0.0147	ENSSSCG00000004425	protein coding
ALDH2	Satellite stem cells	0.956	3.9	0.0346	ENSSSCG00000009889	protein coding
NTN4	Satellite stem cells	0.959	4.38	0.0289	ENSSSCG00000000896	protein coding
AKTIP	Satellite stem cells	0.962	3.74	0.0461	ENSSSCG00000026181	protein coding
HERPUD2	Satellite stem cells	0.964	5.86	0.00324	ENSSSCG00000027389	protein coding
IFT46	Satellite stem cells	0.966	4.98	0.0233	ENSSSCG00000029649	protein coding
STX11	Satellite stem cells	0.969	6.07	0.00288	ENSSSCG00000004125	protein coding
RETREG1	Satellite stem cells	0.975	5.76	0.00852	ENSSSCG00000016792	protein coding
LURAP1L	Satellite stem cells	0.981	4.36	0.0159	ENSSSCG00000036360	protein coding
TFPI2	Satellite stem cells	0.993	5.81	0.00607	ENSSSCG00000015322	protein coding
ZNF672	Satellite stem cells	0.997	3.76	0.0436	ENSSSCG00000013978	protein coding
C16orf87	Satellite stem cells	1	4.41	0.0217	ENSSSCG00000022333	protein coding
IMMP2L	Satellite stem cells	1	8.3	0.0213	ENSSSCG00000016650	protein coding
CD302	Satellite stem cells	1.01	4.2	0.0164	ENSSSCG00000036050	protein coding
ELL2	Satellite stem cells	1.01	5.26	0.00266	ENSSSCG00000014168	protein coding
FAM177A1	Satellite stem cells	1.01	4.67	0.00452	ENSSSCG00000034753	protein coding
HEXIM2	Satellite stem cells	1.01	4.28	0.0109	ENSSSCG00000017335	protein coding
KCNJ13	Satellite stem cells	1.01	3.68	0.0478	ENSSSCG00000025038	protein coding
ENSSSCG00000016823	Satellite stem cells	1.02	7.3	0.00114	ENSSSCG00000016823	protein coding
MSTRG.11887	Satellite stem cells	1.02	4.54	0.0406	MSTRG.11887	lncRNA
MSTRG.6594	Satellite stem cells	1.02	7.19	0.0124	MSTRG.6594	lncRNA
PNPLA2	Satellite stem cells	1.02	4.8	0.0337	ENSSSCG00000012841	protein coding
COL1A2	Satellite stem cells	1.04	9.49	0.014	ENSSSCG00000015326	protein coding
HAO2	Satellite stem cells	1.04	3.54	0.0321	ENSSSCG00000006720	protein coding
MSTRG.32419	Satellite stem cells	1.04	3.6	0.0372	MSTRG.32419	lncRNA
CBLB	Satellite stem cells	1.05	6.12	0.00072	ENSSSCG00000022486	protein coding
MSTRG.10768	Satellite stem cells	1.05	3.62	0.0434	MSTRG.10768	lncRNA
QSOX1	Satellite stem cells	1.05	4	0.0238	ENSSSCG00000015535	protein coding
TANC1	Satellite stem cells	1.06	6.37	0.00163	ENSSSCG00000015880	protein coding
TUFT1	Satellite stem cells	1.06	4.64	0.019	ENSSSCG00000006620	protein coding
KLC4	Satellite stem cells	1.07	3.16	0.0306	ENSSSCG00000001659	protein coding
MSTRG.33011	Satellite stem cells	1.07	5.06	0.00178	MSTRG.33011	lncRNA
MSTRG.36323	Satellite stem cells	1.07	3.68	0.0493	MSTRG.36323	lncRNA
ENSSSCG00000039731	Satellite stem cells	1.08	4.16	0.0139	ENSSSCG00000039731	protein coding
GPX3	Satellite stem cells	1.08	6.34	0.015	ENSSSCG00000036438	protein coding
RIDA	Satellite stem cells	1.08	4.75	0.00253	ENSSSCG00000006079	protein coding
UBL7	Satellite stem cells	1.08	4.89	0.0204	ENSSSCG00000001907	protein coding
MSTRG.10687	Satellite stem cells	1.09	3.58	0.0334	MSTRG.10687	lncRNA

MSTRG.10660	Satellite stem cells	1.1	4.57	0.0304	MSTRG.10660	lncRNA
TNFAIP6	Satellite stem cells	1.1	5.02	0.0328	ENSSSCG00000023716	protein coding
ARID5B	Satellite stem cells	1.11	6.44	0.000658	ENSSSCG00000010219	protein coding
MSTRG.44698	Satellite stem cells	1.11	4.52	0.0273	MSTRG.44698	lncRNA
MSTRG.40007	Satellite stem cells	1.12	4.38	0.00723	MSTRG.40007	lncRNA
RNASE4	Satellite stem cells	1.12	6.04	0.00306	ENSSSCG00000031538	protein coding
YPEL3	Satellite stem cells	1.12	5.88	0.00176	ENSSSCG00000035430	protein coding
CDH20	Satellite stem cells	1.13	4.14	0.0073	ENSSSCG00000004903	protein coding
MAP3K1	Satellite stem cells	1.13	3.62	0.0186	ENSSSCG00000016918	protein coding
SELENOW	Satellite stem cells	1.13	7.77	0.000134	ENSSSCG00000034313	protein coding
SMARCA1	Satellite stem cells	1.13	4.09	0.0221	ENSSSCG00000012649	protein coding
PDE4B	Satellite stem cells	1.14	4.35	0.00469	ENSSSCG00000003805	protein coding
MSTRG.23990	Satellite stem cells	1.15	3.81	0.0223	MSTRG.23990	lncRNA
MSTRG.31456	Satellite stem cells	1.15	3.2	0.0211	MSTRG.31456	lncRNA
MSTRG.31639	Satellite stem cells	1.16	4	0.035	MSTRG.31639	lncRNA
NUDT7	Satellite stem cells	1.16	3.18	0.027	ENSSSCG00000002697	protein coding
BORCS8	Satellite stem cells	1.17	3.63	0.0104	ENSSSCG00000044084	protein coding
PCDH17	Satellite stem cells	1.17	3.47	0.0366	ENSSSCG00000009446	protein coding
RASD1	Satellite stem cells	1.17	4.53	0.00401	ENSSSCG00000034044	protein coding
SESN3	Satellite stem cells	1.17	4.12	0.01	ENSSSCG00000037015	protein coding
ARVCF	Satellite stem cells	1.18	3.87	0.013	ENSSSCG00000010133	protein coding
FAM227B	Satellite stem cells	1.18	3.92	0.00986	ENSSSCG00000004648	protein coding
HMGCR	Satellite stem cells	1.18	3.37	0.0204	ENSSSCG00000014080	protein coding
MSTRG.23419	Satellite stem cells	1.18	4.33	0.0177	MSTRG.23419	lncRNA
MSTRG.32310	Satellite stem cells	1.18	4.83	0.0421	MSTRG.32310	lncRNA
ARMCX2	Satellite stem cells	1.19	4.68	0.0074	ENSSSCG00000012510	protein coding
ENSSSCG00000042577	Satellite stem cells	1.19	3.7	0.0418	ENSSSCG00000042577	protein coding
GALNS	Satellite stem cells	1.19	3.62	0.042	ENSSSCG00000002645	protein coding
MSTRG.11460	Satellite stem cells	1.19	4.26	0.0144	MSTRG.11460	lncRNA
MSTRG.9045	Satellite stem cells	1.2	4.08	0.0257	MSTRG.9045	lncRNA
MSMO1	Satellite stem cells	1.21	4.35	0.00641	ENSSSCG00000008857	protein coding
RASGEF1B	Satellite stem cells	1.21	5.11	0.0285	ENSSSCG00000029507	protein coding
MSTRG.27442	Satellite stem cells	1.23	3.55	0.026	MSTRG.27442	lncRNA
ZNF773	Satellite stem cells	1.23	3.19	0.0347	ENSSSCG00000039978	protein coding
JAZF1	Satellite stem cells	1.24	3.76	0.0384	ENSSSCG00000016691	protein coding
SLC40A1	Satellite stem cells	1.24	5.93	0.00366	ENSSSCG00000027157	protein coding
ENPP1	Satellite stem cells	1.25	3.35	0.0483	ENSSSCG00000004193	protein coding
MSTRG.17788	Satellite stem cells	1.25	5.3	0.000986	MSTRG.17788	lncRNA

MSTRG.18663	Satellite stem cells	1.25	4.65	0.00156	MSTRG.18663	lncRNA
MSTRG.25659	Satellite stem cells	1.25	3.72	0.015	MSTRG.25659	lncRNA
MSTRG.28701	Satellite stem cells	1.25	3.65	0.00851	MSTRG.28701	lncRNA
FAM161B	Satellite stem cells	1.26	3.08	0.0181	ENSSSCG00000002353	protein coding
ENSSSCG00000028481	Satellite stem cells	1.27	3.58	0.0211	ENSSSCG00000028481	protein coding
MAT2B	Satellite stem cells	1.27	4.62	0.00337	ENSSSCG00000021229	protein coding
MSTRG.17509	Satellite stem cells	1.28	3.31	0.0169	MSTRG.17509	lncRNA
MSTRG.8793	Satellite stem cells	1.28	4.12	0.0194	MSTRG.8793	lncRNA
SERPINE2	Satellite stem cells	1.28	4.68	0.0135	ENSSSCG00000016233	protein coding
SGK1	Satellite stem cells	1.28	3.31	0.0127	ENSSSCG00000021443	protein coding
MSTRG.42632	Satellite stem cells	1.29	5.58	0.0369	MSTRG.42632	lncRNA
AGTR1	Satellite stem cells	1.3	4.04	0.0192	ENSSSCG00000011695	protein coding
BGN	Satellite stem cells	1.3	4.52	0.0122	ENSSSCG00000012766	protein coding
ABI3BP	Satellite stem cells	1.33	4.55	0.0133	ENSSSCG00000011959	protein coding
MSTRG.46283	Satellite stem cells	1.33	3.69	0.0283	MSTRG.46283	lncRNA
NUDT15	Satellite stem cells	1.33	3.91	0.00489	ENSSSCG00000027551	protein coding
CCSER1	Satellite stem cells	1.35	3.59	0.0311	ENSSSCG00000022986	protein coding
ENSSSCG00000017446	Satellite stem cells	1.36	3.87	0.0355	ENSSSCG00000017446	protein coding
MSTRG.1400	Satellite stem cells	1.36	3.87	0.0184	MSTRG.1400	lncRNA
MSTRG.9241	Satellite stem cells	1.37	4.4	0.0163	MSTRG.9241	lncRNA
TIMP1	Satellite stem cells	1.37	6.47	0.000165	ENSSSCG00000012277	protein coding
YPEL5	Satellite stem cells	1.37	4.63	0.000623	ENSSSCG00000008529	protein coding
GCAT	Satellite stem cells	1.38	3.58	0.00658	ENSSSCG00000029945	protein coding
CCL2	Satellite stem cells	1.4	4.14	0.00692	ENSSSCG00000017723	protein coding
ENSSSCG00000022925	Satellite stem cells	1.4	4	0.0172	ENSSSCG00000022925	protein coding
ENSSSCG00000044542	Satellite stem cells	1.41	4.76	0.000968	ENSSSCG00000044542	protein coding
MSTRG.161	Satellite stem cells	1.41	6.44	0.000196	MSTRG.161	lncRNA
PCK2	Satellite stem cells	1.41	4.76	0.0291	ENSSSCG00000002009	protein coding
EBF2	Satellite stem cells	1.45	3.56	0.0362	ENSSSCG00000009655	protein coding
PID1	Satellite stem cells	1.45	4	0.0346	ENSSSCG00000035058	protein coding
MSTRG.32351	Satellite stem cells	1.46	5.28	0.00233	MSTRG.32351	lncRNA
APOD	Satellite stem cells	1.47	5.24	0.0138	ENSSSCG00000011831	protein coding
MSTRG.9345	Satellite stem cells	1.49	3.02	0.0309	MSTRG.9345	lncRNA
SELENOP	Satellite stem cells	1.49	7.45	0.000202	ENSSSCG00000021208	protein coding
MSTRG.18957	Satellite stem cells	1.5	4.18	0.0218	MSTRG.18957	lncRNA
ENSSSCG00000041684	Satellite stem cells	1.54	3.48	0.0191	ENSSSCG00000041684	lncRNA
GPX1	Satellite stem cells	1.54	7.54	0.01	ENSSSCG00000033727	protein coding
ENSSSCG00000039222	Satellite stem cells	1.55	3.97	0.00187	ENSSSCG00000039222	protein coding

MSTRG.23839	Satellite stem cells	1.55	3.73	0.0174	MSTRG.23839	lncRNA
PHGDH	Satellite stem cells	1.55	7.7	0.0203	ENSSSCG00000006717	protein coding
MSTRG.14868	Satellite stem cells	1.56	2.97	0.00879	MSTRG.14868	lncRNA
RBM48	Satellite stem cells	1.56	3.74	0.00288	ENSSSCG00000015315	protein coding
EFNA1	Satellite stem cells	1.57	4.28	0.00291	ENSSSCG00000006530	protein coding
SLC10A1	Satellite stem cells	1.58	4.4	0.000797	ENSSSCG00000002313	protein coding
IL1RAPL2	Satellite stem cells	1.61	3.56	0.0132	ENSSSCG00000012545	protein coding
NRXN1	Satellite stem cells	1.61	4.02	0.00629	ENSSSCG00000026180	protein coding
MSTRG.13235	Satellite stem cells	1.65	4.84	0.00364	MSTRG.13235	lncRNA
DACH1	Satellite stem cells	1.67	3.56	0.0103	ENSSSCG00000009457	protein coding
EGLN3	Satellite stem cells	1.69	3.26	0.0097	ENSSSCG00000001963	protein coding
MSTRG.17815	Satellite stem cells	1.7	4.68	0.0046	MSTRG.17815	lncRNA
MSTRG.25877	Satellite stem cells	1.72	3.71	0.00913	MSTRG.25877	lncRNA
MSTRG.28657	Satellite stem cells	1.75	3.96	0.00312	MSTRG.28657	lncRNA
ENSSSCG00000045666	Satellite stem cells	1.77	4.44	0.000243	ENSSSCG00000045666	lncRNA
MSTRG.8600	Satellite stem cells	1.77	5.02	0.0209	MSTRG.8600	lncRNA
MSTRG.22342	Satellite stem cells	1.78	4.44	0.00127	MSTRG.22342	lncRNA
SELENOM	Satellite stem cells	1.79	4.44	0.0176	ENSSSCG00000029781	protein coding
MSTRG.38467	Satellite stem cells	1.83	8.56	0.0195	MSTRG.38467	lncRNA
HEY1	Satellite stem cells	1.87	3.74	0.00245	ENSSSCG00000006159	protein coding
NTRK2	Satellite stem cells	1.87	3.23	0.0215	ENSSSCG00000010959	protein coding
ERC2	Satellite stem cells	1.89	3.06	0.00487	ENSSSCG00000024417	protein coding
MSTRG.22177	Satellite stem cells	1.89	3.32	0.0378	MSTRG.22177	lncRNA
PLAAT3	Satellite stem cells	1.92	3.62	0.00823	ENSSSCG00000026914	protein coding
LUM	Satellite stem cells	1.93	7.02	0.0242	ENSSSCG00000000916	protein coding
MSTRG.26712	Satellite stem cells	1.96	2.93	0.0143	MSTRG.26712	lncRNA
MSTRG.27574	Satellite stem cells	1.99	3.68	0.013	MSTRG.27574	lncRNA
MSTRG.13087	Satellite stem cells	2	3.87	0.00579	MSTRG.13087	lncRNA
MSTRG.28362	Satellite stem cells	2	4.57	0.0405	MSTRG.28362	lncRNA
RRAD	Satellite stem cells	2.02	3.75	0.00217	ENSSSCG00000036060	protein coding
EGFL8	Satellite stem cells	2.03	3.24	0.00422	ENSSSCG00000001434	protein coding
TNFAIP3	Satellite stem cells	2.06	5.14	0.00526	ENSSSCG00000004154	protein coding
MYF5	Satellite stem cells	2.07	7.38	1.64E-05	ENSSSCG00000000937	protein coding
NRXN3	Satellite stem cells	2.07	3.56	0.00317	ENSSSCG00000028942	protein coding
ASNS	Satellite stem cells	2.09	6.09	0.0252	ENSSSCG00000015340	protein coding
NFE2L3	Satellite stem cells	2.15	3.87	0.000718	ENSSSCG00000026894	protein coding
MSTRG.39844	Satellite stem cells	2.16	3.14	0.000953	MSTRG.39844	lncRNA
PLCB1	Satellite stem cells	2.18	4.8	0.000383	ENSSSCG00000007056	protein coding

HOXB2	Satellite stem cells	2.19	3.22	0.00872	ENSSSCG00000017533	protein coding
NUPR1	Satellite stem cells	2.21	4.4	0.00853	ENSSSCG00000040162	protein coding
OPTN	Satellite stem cells	2.35	2.97	0.00311	ENSSSCG00000023247	protein coding
ENSSSCG00000037324	Satellite stem cells	2.5	3.89	0.000192	ENSSSCG00000037324	lncRNA
MSTRG.882	Satellite stem cells	2.71	4.7	0.0251	MSTRG.882	lncRNA
MSTRG.38853	Satellite stem cells	3.2	7.16	0.00745	MSTRG.38853	lncRNA
PSAT1	Satellite stem cells	3.27	7.42	0.00628	ENSSSCG00000005287	protein coding
MSTRG.29989	Satellite stem cells	3.9	5.71	0.0108	MSTRG.29989	lncRNA
MSTRG.210	*TRIB1*$^+$ Satellite cells	-4.84	5.07	0.00231	MSTRG.210	lncRNA
ENSSSCG00000032436	*TRIB1*$^+$ Satellite cells	-4.57	5.21	0.0183	ENSSSCG00000032436	protein coding
CCBE1	*TRIB1*$^+$ Satellite cells	-4.4	6.09	0.00114	ENSSSCG00000004907	protein coding
EPSTI1	*TRIB1*$^+$ Satellite cells	-4.22	4.39	0.0043	ENSSSCG00000037572	protein coding
IFI6	*TRIB1*$^+$ Satellite cells	-4.12	6.06	0.0189	ENSSSCG00000034570	protein coding
ISG15	*TRIB1*$^+$ Satellite cells	-3.94	7.63	0.0288	ENSSSCG00000040575	protein coding
MSTRG.4840	*TRIB1*$^+$ Satellite cells	-3.55	4.14	1.03E-05	MSTRG.4840	lncRNA
CLCA2	*TRIB1*$^+$ Satellite cells	-3.31	3.27	0.00104	ENSSSCG00000006935	protein coding
ENSSSCG00000014565	*TRIB1*$^+$ Satellite cells	-3.24	5.67	0.0167	ENSSSCG00000014565	protein coding
MPP3	*TRIB1*$^+$ Satellite cells	-3.24	6.13	8.18E-05	ENSSSCG00000017372	protein coding
MSTRG.22838	*TRIB1*$^+$ Satellite cells	-3.2	3.84	0.00268	MSTRG.22838	lncRNA
MX1	*TRIB1*$^+$ Satellite cells	-3.2	5.86	0.0345	ENSSSCG00000012077	protein coding
TMEM100	*TRIB1*$^+$ Satellite cells	-3.01	7.22	8.18E-06	ENSSSCG00000017607	protein coding
MSTRG.5319	*TRIB1*$^+$ Satellite cells	-2.92	3.03	0.00492	MSTRG.5319	lncRNA
IFITM3	*TRIB1*$^+$ Satellite cells	-2.83	7.3	0.0058	ENSSSCG00000038912	protein coding
MSTRG.34616	*TRIB1*$^+$ Satellite cells	-2.67	4.92	0.0446	MSTRG.34616	lncRNA
SLC24A4	*TRIB1*$^+$ Satellite cells	-2.66	5.01	8.87E-06	ENSSSCG00000002450	protein coding
SERPINA1	*TRIB1*$^+$ Satellite cells	-2.65	3.68	0.00142	ENSSSCG00000002476	protein coding
ENSSSCG00000017146	*TRIB1*$^+$ Satellite cells	-2.6	3.73	0.0179	ENSSSCG00000017146	protein coding
GRXCR2	*TRIB1*$^+$ Satellite cells	-2.54	4.54	0.0178	ENSSSCG00000040306	protein coding
ANKFN1	*TRIB1*$^+$ Satellite cells	-2.5	4.38	0.021	ENSSSCG00000017609	protein coding
PNMT	*TRIB1*$^+$ Satellite cells	-2.48	5.51	2.26E-06	ENSSSCG00000017501	protein coding
EIF2AK2	*TRIB1*$^+$ Satellite cells	-2.37	6.03	0.017	ENSSSCG00000008496	protein coding
SH3RF2	*TRIB1*$^+$ Satellite cells	-2.34	5.88	0.00104	ENSSSCG00000014410	protein coding
MSTRG.41034	*TRIB1*$^+$ Satellite cells	-2.28	4.55	0.0105	MSTRG.41034	lncRNA
RAB6B	*TRIB1*$^+$ Satellite cells	-2.24	3.12	0.00104	ENSSSCG00000036503	protein coding
ENSSSCG00000004572	*TRIB1*$^+$ Satellite cells	-2.23	4.38	0.00188	ENSSSCG00000004572	protein coding

MSTRG.39368	*TRIB1* + Satellite cells	-2.23	5.41	0.000284	MSTRG.39368	lncRNA
ENSSSCG00000009182	*TRIB1* + Satellite cells	-2.2	3.89	0.0409	ENSSSCG00000009182	protein coding
ENSSSCG00000005216	*TRIB1* + Satellite cells	-2.11	3.91	0.00902	ENSSSCG00000005216	protein coding
MSTRG.6894	*TRIB1* + Satellite cells	-2.07	2.91	0.00456	MSTRG.6894	lncRNA
MSTRG.14188	*TRIB1* + Satellite cells	-2.03	3.23	0.00254	MSTRG.14188	lncRNA
ENSSSCG00000049704	*TRIB1* + Satellite cells	-1.95	3.13	0.0285	ENSSSCG00000049704	lncRNA
ENSSSCG00000027725	*TRIB1* + Satellite cells	-1.92	3.22	0.00496	ENSSSCG00000027725	protein coding
ENSSSCG00000017608	*TRIB1* + Satellite cells	-1.9	4.93	0.000404	ENSSSCG00000017608	protein coding
MSTRG.32994	*TRIB1* + Satellite cells	-1.9	3.26	0.0149	MSTRG.32994	lncRNA
MSTRG.42256	*TRIB1* + Satellite cells	-1.9	3.25	0.000687	MSTRG.42256	lncRNA
UCP2	*TRIB1* + Satellite cells	-1.9	3.15	0.0146	ENSSSCG00000014833	protein coding
MSTRG.5303	*TRIB1* + Satellite cells	-1.88	3.79	0.0025	MSTRG.5303	lncRNA
MSTRG.5305	*TRIB1* + Satellite cells	-1.87	4.45	0.0026	MSTRG.5305	lncRNA
DDIT4	*TRIB1* + Satellite cells	-1.86	6.37	0.000357	ENSSSCG00000031888	protein coding
KLF15	*TRIB1* + Satellite cells	-1.85	5.14	0.00126	ENSSSCG00000036742	protein coding
MSTRG.34836	*TRIB1* + Satellite cells	-1.82	4.75	0.000227	MSTRG.34836	lncRNA
MSTRG.8361	*TRIB1* + Satellite cells	-1.79	3.52	0.0122	MSTRG.8361	lncRNA
ENSSSCG00000012101	*TRIB1* + Satellite cells	-1.76	3.43	0.00537	ENSSSCG00000012101	protein coding
STK33	*TRIB1* + Satellite cells	-1.76	4.81	0.0054	ENSSSCG00000030206	protein coding
MSTRG.19874	*TRIB1* + Satellite cells	-1.75	3.08	0.00152	MSTRG.19874	lncRNA
ASPA	*TRIB1* + Satellite cells	-1.74	3.37	0.00931	ENSSSCG00000017861	protein coding
TRAPPC12	*TRIB1* + Satellite cells	-1.73	3.06	0.000454	ENSSSCG00000024665	protein coding
ZBTB16	*TRIB1* + Satellite cells	-1.73	8.85	0.0146	ENSSSCG00000030095	protein coding
PLXNA4	*TRIB1* + Satellite cells	-1.71	6.7	0.000405	ENSSSCG00000021562	protein coding
KIAA0513	*TRIB1* + Satellite cells	-1.69	6	0.000148	ENSSSCG00000002666	protein coding
FAM124A	*TRIB1* + Satellite cells	-1.67	4.14	0.0065	ENSSSCG00000029598	protein coding
MSTRG.29485	*TRIB1* + Satellite cells	-1.67	3.58	0.0087	MSTRG.29485	lncRNA
CAVIN2	*TRIB1* + Satellite cells	-1.64	3.81	0.00113	ENSSSCG00000026618	protein coding
DAPK1	*TRIB1* + Satellite cells	-1.64	6.91	0.00332	ENSSSCG00000010949	protein coding
HEATR1	*TRIB1* + Satellite cells	-1.63	3.21	0.000399	ENSSSCG00000010145	protein coding
ADAMTS15	*TRIB1* + Satellite cells	-1.62	3.7	0.00265	ENSSSCG00000015250	protein coding
CHRDL2	*TRIB1* + Satellite cells	-1.62	5.05	0.0453	ENSSSCG00000014843	protein coding
ARRDC2	*TRIB1* + Satellite cells	-1.61	3.7	0.00402	ENSSSCG00000013893	protein coding
MSTRG.9705	*TRIB1* + Satellite cells	-1.58	4.17	0.00616	MSTRG.9705	lncRNA

BOC	*TRIB1* + Satellite cells	-1.57	5.05	0.000335	ENSSSCG00000025826	protein coding
ENSSSCG00000051726	*TRIB1* + Satellite cells	-1.57	3.82	0.000752	ENSSSCG00000051726	lncRNA
MSTRG.11093	*TRIB1* + Satellite cells	-1.55	3.6	0.00591	MSTRG.11093	lncRNA
MSTRG.38911	*TRIB1* + Satellite cells	-1.52	3.2	0.000843	MSTRG.38911	lncRNA
MSTRG.505	*TRIB1* + Satellite cells	-1.51	4.45	0.00204	MSTRG.505	lncRNA
MYBPH	*TRIB1* + Satellite cells	-1.5	4.83	0.012	ENSSSCG00000030681	protein coding
MYO7A	*TRIB1* + Satellite cells	-1.5	3.06	0.00942	ENSSSCG00000014876	protein coding
SRF	*TRIB1* + Satellite cells	-1.5	3.46	0.00174	ENSSSCG00000001661	protein coding
ZNFX1	*TRIB1* + Satellite cells	-1.5	5.13	0.0174	ENSSSCG00000030108	protein coding
FBN3	*TRIB1* + Satellite cells	-1.49	3.71	0.0118	ENSSSCG00000013594	protein coding
MSTRG.43969	*TRIB1* + Satellite cells	-1.49	4.2	0.000472	MSTRG.43969	lncRNA
ABCA8	*TRIB1* + Satellite cells	-1.48	6.35	0.0334	ENSSSCG00000026473	protein coding
DDIT3	*TRIB1* + Satellite cells	-1.48	4.77	0.00392	ENSSSCG00000044553	protein coding
HLF	*TRIB1* + Satellite cells	-1.48	3.97	0.000278	ENSSSCG00000017604	protein coding
MSTRG.6783	*TRIB1* + Satellite cells	-1.47	4.57	0.0131	MSTRG.6783	lncRNA
ZC3HAV1	*TRIB1* + Satellite cells	-1.47	4.28	0.0294	ENSSSCG00000016512	protein coding
MSTRG.19359	*TRIB1* + Satellite cells	-1.46	3.5	0.0169	MSTRG.19359	lncRNA
PDZRN3	*TRIB1* + Satellite cells	-1.45	5.96	0.00425	ENSSSCG00000011521	protein coding
BCL9L	*TRIB1* + Satellite cells	-1.43	3.45	0.00119	ENSSSCG00000015099	protein coding
MSTRG.25856	*TRIB1* + Satellite cells	-1.42	5.16	0.00547	MSTRG.25856	lncRNA
ZCCHC24	*TRIB1* + Satellite cells	-1.42	6.7	0.000111	ENSSSCG00000033074	protein coding
KCTD16	*TRIB1* + Satellite cells	-1.41	4.54	0.0458	ENSSSCG00000032826	protein coding
MSTRG.40676	*TRIB1* + Satellite cells	-1.41	3.77	0.000342	MSTRG.40676	lncRNA
ANO4	*TRIB1* + Satellite cells	-1.39	3.7	0.0192	ENSSSCG00000000873	protein coding
HSPBAP1	*TRIB1* + Satellite cells	-1.39	4.48	0.00114	ENSSSCG00000011873	protein coding
TXNIP	*TRIB1* + Satellite cells	-1.39	9.18	0.00135	ENSSSCG00000031262	protein coding
MSTRG.6521	*TRIB1* + Satellite cells	-1.38	3.64	0.00049	MSTRG.6521	lncRNA
ZFP36	*TRIB1* + Satellite cells	-1.38	9.83	0.00052	ENSSSCG00000028056	protein coding
CDKL5	*TRIB1* + Satellite cells	-1.37	4.34	0.00268	ENSSSCG00000012156	protein coding
PLXDC1	*TRIB1* + Satellite cells	-1.37	4.39	0.0194	ENSSSCG00000017511	protein coding
AGT	*TRIB1* + Satellite cells	-1.36	7.52	0.0149	ENSSSCG00000010184	protein coding
ABCA1	*TRIB1* + Satellite cells	-1.34	4.06	0.0163	ENSSSCG00000005423	protein coding
MSTRG.29775	*TRIB1* + Satellite cells	-1.34	4.9	0.000356	MSTRG.29775	lncRNA
PER1	*TRIB1* + Satellite cells	-1.34	7.02	0.00886	ENSSSCG00000017983	protein coding

CRISPLD2	*TRIB1*⁺ Satellite cells	-1.32	5.51	0.0011	ENSSSCG00000002669	protein coding
MFSD4B	*TRIB1*⁺ Satellite cells	-1.32	3.62	0.00417	ENSSSCG00000041409	protein coding
BMP6	*TRIB1*⁺ Satellite cells	-1.31	4.99	0.0127	ENSSSCG00000001027	protein coding
FIS1	*TRIB1*⁺ Satellite cells	-1.31	3.63	0.0384	ENSSSCG00000038938	protein coding
ENSSSCG00000004387	*TRIB1*⁺ Satellite cells	-1.3	8.41	0.000376	ENSSSCG00000004387	protein coding
MSTRG.41574	*TRIB1*⁺ Satellite cells	-1.3	3.66	0.00294	MSTRG.41574	lncRNA
SLCO2A1	*TRIB1*⁺ Satellite cells	-1.3	5.61	0.00244	ENSSSCG00000011641	protein coding
MSTRG.30373	*TRIB1*⁺ Satellite cells	-1.29	3.94	0.0248	MSTRG.30373	lncRNA
MSTRG.35160	*TRIB1*⁺ Satellite cells	-1.29	3.33	0.004	MSTRG.35160	lncRNA
MSTRG.49025	*TRIB1*⁺ Satellite cells	-1.29	4.27	0.046	MSTRG.49025	lncRNA
FBXO39	*TRIB1*⁺ Satellite cells	-1.28	3.63	0.0414	ENSSSCG00000017886	protein coding
FKBP5	*TRIB1*⁺ Satellite cells	-1.28	8.35	0.0099	ENSSSCG00000001549	protein coding
MSTRG.14445	*TRIB1*⁺ Satellite cells	-1.27	4.39	0.00599	MSTRG.14445	lncRNA
FAH	*TRIB1*⁺ Satellite cells	-1.26	4.74	0.00306	ENSSSCG00000001780	protein coding
MSTRG.1952	*TRIB1*⁺ Satellite cells	-1.26	4.95	0.00379	MSTRG.1952	lncRNA
MSTRG.27600	*TRIB1*⁺ Satellite cells	-1.26	4.57	0.00204	MSTRG.27600	lncRNA
NOL11	*TRIB1*⁺ Satellite cells	-1.26	9.54	0.0107	ENSSSCG00000017274	protein coding
PEAR1	*TRIB1*⁺ Satellite cells	-1.26	3.65	0.0242	ENSSSCG00000006463	protein coding
ENSSSCG00000006510	*TRIB1*⁺ Satellite cells	-1.24	3.74	0.00861	ENSSSCG00000006510	protein coding
ENSSSCG00000008898	*TRIB1*⁺ Satellite cells	-1.24	8.53	0.00864	ENSSSCG00000008898	protein coding
XIRP2	*TRIB1*⁺ Satellite cells	-1.24	4.33	0.0356	ENSSSCG00000015917	protein coding
ABCC1	*TRIB1*⁺ Satellite cells	-1.23	3.33	0.0133	ENSSSCG00000007874	protein coding
LPIN1	*TRIB1*⁺ Satellite cells	-1.23	4.31	0.00357	ENSSSCG00000008624	protein coding
MSTRG.20896	*TRIB1*⁺ Satellite cells	-1.23	3.1	0.0202	MSTRG.20896	lncRNA
MSTRG.24094	*TRIB1*⁺ Satellite cells	-1.23	4.23	0.00112	MSTRG.24094	lncRNA
MSTRG.14711	*TRIB1*⁺ Satellite cells	-1.22	3.72	0.00949	MSTRG.14711	lncRNA
ENSSSCG00000009914	*TRIB1*⁺ Satellite cells	-1.21	4.69	0.000638	ENSSSCG00000009914	protein coding
MSTRG.48810	*TRIB1*⁺ Satellite cells	-1.21	3.29	0.00453	MSTRG.48810	lncRNA
ENSSSCG00000049158	*TRIB1*⁺ Satellite cells	-1.19	5.21	0.00301	ENSSSCG00000049158	protein coding
MOB3B	*TRIB1*⁺ Satellite cells	-1.19	3.32	0.0212	ENSSSCG00000038811	protein coding
SLC22A3	*TRIB1*⁺ Satellite cells	-1.19	3.44	0.0178	ENSSSCG00000004039	protein coding
ENSSSCG00000011859	*TRIB1*⁺ Satellite cells	-1.18	4.86	0.00104	ENSSSCG00000011859	protein coding
LANCL3	*TRIB1*⁺ Satellite cells	-1.18	4.64	0.00142	ENSSSCG00000037269	protein coding
PLCB4	*TRIB1*⁺ Satellite cells	-1.18	6.93	0.00135	ENSSSCG00000007058	protein coding

MSTRG.714	*TRIB1*[+] Satellite cells	-1.17	3.19	0.0287	MSTRG.714	lncRNA
CFLAR	*TRIB1*[+] Satellite cells	-1.16	5.12	0.0128	ENSSSCG00000016101	protein coding
CRLF3	*TRIB1*[+] Satellite cells	-1.16	3.89	0.00195	ENSSSCG00000017743	protein coding
MSTRG.3699	*TRIB1*[+] Satellite cells	-1.16	4.36	0.00513	MSTRG.3699	lncRNA
MSTRG.48476	*TRIB1*[+] Satellite cells	-1.16	3.49	0.0375	MSTRG.48476	lncRNA
PHETA2	*TRIB1*[+] Satellite cells	-1.16	4.37	0.001	ENSSSCG00000038492	protein coding
PIK3R1	*TRIB1*[+] Satellite cells	-1.16	7.02	0.00508	ENSSSCG00000016958	protein coding
EPHA4	*TRIB1*[+] Satellite cells	-1.15	6.46	0.0229	ENSSSCG00000016230	protein coding
KLHDC4	*TRIB1*[+] Satellite cells	-1.15	3.83	0.0019	ENSSSCG00000002652	protein coding
KLHL29	*TRIB1*[+] Satellite cells	-1.15	3.53	0.00673	ENSSSCG00000008593	protein coding
NOD2	*TRIB1*[+] Satellite cells	-1.15	3.36	0.00605	ENSSSCG00000038073	protein coding
TEX26	*TRIB1*[+] Satellite cells	-1.15	4.4	0.048	ENSSSCG00000046261	protein coding
TSTD2	*TRIB1*[+] Satellite cells	-1.14	3.85	0.00422	ENSSSCG00000005367	protein coding
FOSB	*TRIB1*[+] Satellite cells	-1.13	10.5	0.000271	ENSSSCG00000031616	protein coding
GIT1	*TRIB1*[+] Satellite cells	-1.12	3.41	0.00347	ENSSSCG00000017787	protein coding
MSTRG.26865	*TRIB1*[+] Satellite cells	-1.12	3.29	0.00693	MSTRG.26865	lncRNA
ENSSSCG00000042225	*TRIB1*[+] Satellite cells	-1.11	5.82	0.000408	ENSSSCG00000042225	lncRNA
MEDAG	*TRIB1*[+] Satellite cells	-1.11	3.37	0.0215	ENSSSCG00000009331	protein coding
MSTRG.24014	*TRIB1*[+] Satellite cells	-1.1	3.35	0.00466	MSTRG.24014	lncRNA
ST3GAL1	*TRIB1*[+] Satellite cells	-1.1	5.53	0.00875	ENSSSCG00000005943	protein coding
ENSSSCG00000003741	*TRIB1*[+] Satellite cells	-1.09	3.51	0.00579	ENSSSCG00000003741	protein coding
GRIN2B	*TRIB1*[+] Satellite cells	-1.08	3.62	0.0267	ENSSSCG00000000614	protein coding
RASL11B	*TRIB1*[+] Satellite cells	-1.08	5.93	0.00178	ENSSSCG00000008835	protein coding
ENOX1	*TRIB1*[+] Satellite cells	-1.07	7.45	0.000677	ENSSSCG00000021818	protein coding
MSTRG.16410	*TRIB1*[+] Satellite cells	-1.07	4.62	0.0194	MSTRG.16410	lncRNA
ENSSSCG00000049152	*TRIB1*[+] Satellite cells	-1.06	4.16	0.0248	ENSSSCG00000049152	lncRNA
ENSSSCG00000050367	*TRIB1*[+] Satellite cells	-1.06	3.5	0.00354	ENSSSCG00000050367	lncRNA
FOS	*TRIB1*[+] Satellite cells	-1.06	11.7	0.0112	ENSSSCG00000002383	protein coding
MSTRG.7023	*TRIB1*[+] Satellite cells	-1.06	3.65	0.005	MSTRG.7023	lncRNA
PRR5L	*TRIB1*[+] Satellite cells	-1.06	4.03	0.0302	ENSSSCG00000013292	protein coding
MSTRG.7842	*TRIB1*[+] Satellite cells	-1.05	4.11	0.00185	MSTRG.7842	lncRNA
MSTRG.9790	*TRIB1*[+] Satellite cells	-1.05	3.72	0.0136	MSTRG.9790	lncRNA
ZKSCAN1	*TRIB1*[+] Satellite cells	-1.05	3.75	0.00235	ENSSSCG00000031461	protein coding
GNA14	*TRIB1*[+] Satellite cells	-1.04	3.92	0.0464	ENSSSCG00000005284	protein coding

MSTRG.46541	*TRIB1* + Satellite cells	-1.04	4.79	0.0402	MSTRG.46541	lncRNA
PLK3	*TRIB1* + Satellite cells	-1.04	4.47	0.00236	ENSSSCG00000003928	protein coding
SERPINB6	*TRIB1* + Satellite cells	-1.04	4.76	0.0213	ENSSSCG00000038404	protein coding
ELP1	*TRIB1* + Satellite cells	-1.03	3.21	0.0127	ENSSSCG00000005441	protein coding
MSTRG.6578	*TRIB1* + Satellite cells	-1.03	3.69	0.0051	MSTRG.6578	lncRNA
ENSSSCG00000001341	*TRIB1* + Satellite cells	-1.02	5.38	0.0439	ENSSSCG00000001341	protein coding
LRRC8D	*TRIB1* + Satellite cells	-1.02	6.48	0.0147	ENSSSCG00000038417	protein coding
MSTRG.30552	*TRIB1* + Satellite cells	-1.02	3.73	0.00854	MSTRG.30552	lncRNA
RAPGEF1	*TRIB1* + Satellite cells	-1.02	5.83	0.0108	ENSSSCG00000005719	protein coding
ABR	*TRIB1* + Satellite cells	-1.01	4.34	0.0118	ENSSSCG00000017804	protein coding
MSTRG.33988	*TRIB1* + Satellite cells	-1.01	3.51	0.0194	MSTRG.33988	lncRNA
URB1	*TRIB1* + Satellite cells	-1.01	3.41	0.0146	ENSSSCG00000032024	protein coding
POLM	*TRIB1* + Satellite cells	-1	4.42	0.00332	ENSSSCG00000016755	protein coding
MSTRG.17481	*TRIB1* + Satellite cells	-0.993	3.7	0.0233	MSTRG.17481	lncRNA
SLC3A2	*TRIB1* + Satellite cells	-0.993	5.48	0.00632	ENSSSCG00000022404	protein coding
ENSSSCG00000021041	*TRIB1* + Satellite cells	-0.988	3.78	0.033	ENSSSCG00000021041	protein coding
PLEKHM1	*TRIB1* + Satellite cells	-0.983	4.83	0.00139	ENSSSCG00000017325	protein coding
AGPAT3	*TRIB1* + Satellite cells	-0.978	6.48	0.0027	ENSSSCG00000038081	protein coding
MON1B	*TRIB1* + Satellite cells	-0.977	4.04	0.00852	ENSSSCG00000002701	protein coding
MSTRG.48953	*TRIB1* + Satellite cells	-0.975	3.48	0.0211	MSTRG.48953	lncRNA
UHRF1BP1	*TRIB1* + Satellite cells	-0.972	3.91	0.00401	ENSSSCG00000001532	protein coding
MSTRG.14344	*TRIB1* + Satellite cells	-0.971	3.41	0.013	MSTRG.14344	lncRNA
PPL	*TRIB1* + Satellite cells	-0.97	5.17	0.0107	ENSSSCG00000007927	protein coding
ENSSSCG00000021941	*TRIB1* + Satellite cells	-0.969	3.44	0.00608	ENSSSCG00000021941	protein coding
MSTRG.43974	*TRIB1* + Satellite cells	-0.966	6.88	0.016	MSTRG.43974	lncRNA
ENSSSCG00000039972	*TRIB1* + Satellite cells	-0.964	3.95	0.00816	ENSSSCG00000039972	protein coding
MSTRG.11604	*TRIB1* + Satellite cells	-0.964	4.78	0.00149	MSTRG.11604	lncRNA
MSTRG.30032	*TRIB1* + Satellite cells	-0.964	3.41	0.0125	MSTRG.30032	lncRNA
SMARCD2	*TRIB1* + Satellite cells	-0.964	4.12	0.00976	ENSSSCG00000041822	protein coding
ADAMTS20	*TRIB1* + Satellite cells	-0.961	7.67	0.0014	ENSSSCG00000000795	protein coding
EP400	*TRIB1* + Satellite cells	-0.959	5.08	0.00134	ENSSSCG00000028018	protein coding
MSTRG.35752	*TRIB1* + Satellite cells	-0.954	6.11	0.000609	MSTRG.35752	lncRNA
PREX1	*TRIB1* + Satellite cells	-0.954	5.02	0.00437	ENSSSCG00000031118	protein coding
ENSSSCG00000040825	*TRIB1* + Satellite cells	-0.947	3.8	0.0174	ENSSSCG00000040825	protein coding

EXOSC4	*TRIB1*$^+$ Satellite cells	-0.947	4.09	0.0297	ENSSSCG00000005925	protein coding
MLLT1	*TRIB1*$^+$ Satellite cells	-0.945	3.59	0.00798	ENSSSCG00000013534	protein coding
FADS2	*TRIB1*$^+$ Satellite cells	-0.944	4.83	0.013	ENSSSCG00000013072	protein coding
SLC35D1	*TRIB1*$^+$ Satellite cells	-0.944	3.56	0.0147	ENSSSCG00000032043	protein coding
ADCY7	*TRIB1*$^+$ Satellite cells	-0.943	4.82	0.00105	ENSSSCG00000039408	protein coding
F2RL2	*TRIB1*$^+$ Satellite cells	-0.943	3.41	0.0299	ENSSSCG00000037582	protein coding
MSTRG.19111	*TRIB1*$^+$ Satellite cells	-0.942	4.17	0.00461	MSTRG.19111	lncRNA
USP53	*TRIB1*$^+$ Satellite cells	-0.941	7.07	0.00165	ENSSSCG00000023174	protein coding
UTP23	*TRIB1*$^+$ Satellite cells	-0.939	3.61	0.021	ENSSSCG00000030493	protein coding
ZNF462	*TRIB1*$^+$ Satellite cells	-0.939	6.27	0.00829	ENSSSCG00000031592	protein coding
ARFGEF2	*TRIB1*$^+$ Satellite cells	-0.935	4.48	0.00448	ENSSSCG00000007460	protein coding
EGR1	*TRIB1*$^+$ Satellite cells	-0.931	9.8	0.00917	ENSSSCG00000014336	protein coding
TTC39B	*TRIB1*$^+$ Satellite cells	-0.92	4.2	0.0189	ENSSSCG00000005186	protein coding
DTX4	*TRIB1*$^+$ Satellite cells	-0.919	5.15	0.00466	ENSSSCG00000013145	protein coding
LRIG1	*TRIB1*$^+$ Satellite cells	-0.917	6.88	0.00631	ENSSSCG00000011499	protein coding
MSTRG.14583	*TRIB1*$^+$ Satellite cells	-0.915	4	0.0321	MSTRG.14583	lncRNA
CHKA	*TRIB1*$^+$ Satellite cells	-0.914	4.53	0.0117	ENSSSCG00000012889	protein coding
ECI2	*TRIB1*$^+$ Satellite cells	-0.913	5.18	0.00482	ENSSSCG00000001000	protein coding
GLCCI1	*TRIB1*$^+$ Satellite cells	-0.913	5.78	0.0171	ENSSSCG00000015345	protein coding
MAN1C1	*TRIB1*$^+$ Satellite cells	-0.909	4.49	0.00961	ENSSSCG00000023235	protein coding
MSTRG.506	*TRIB1*$^+$ Satellite cells	-0.907	7.48	0.00317	MSTRG.506	lncRNA
AMOT	*TRIB1*$^+$ Satellite cells	-0.906	4.99	0.00259	ENSSSCG00000012591	protein coding
KATNBL1	*TRIB1*$^+$ Satellite cells	-0.903	6.01	0.000658	ENSSSCG00000002245	protein coding
C14orf93	*TRIB1*$^+$ Satellite cells	-0.9	3.85	0.0159	ENSSSCG00000031452	protein coding
MSTRG.24764	*TRIB1*$^+$ Satellite cells	-0.895	4.35	0.0287	MSTRG.24764	lncRNA
MSTRG.18958	*TRIB1*$^+$ Satellite cells	-0.892	3.76	0.0366	MSTRG.18958	lncRNA
CPT1A	*TRIB1*$^+$ Satellite cells	-0.891	5.56	0.013	ENSSSCG00000012880	protein coding
ATP1A4	*TRIB1*$^+$ Satellite cells	-0.886	4.79	0.0153	ENSSSCG00000006391	protein coding
ENSSSCG00000014280	*TRIB1*$^+$ Satellite cells	-0.884	6.35	0.0431	ENSSSCG00000014280	protein coding
ELOA	*TRIB1*$^+$ Satellite cells	-0.881	4.14	0.00633	ENSSSCG00000025440	protein coding
ZNF696	*TRIB1*$^+$ Satellite cells	-0.88	3.29	0.0173	ENSSSCG00000006959	protein coding
PSME2	*TRIB1*$^+$ Satellite cells	-0.878	6.07	0.0255	ENSSSCG00000002004	protein coding
NPHP1	*TRIB1*$^+$ Satellite cells	-0.877	3.65	0.0138	ENSSSCG00000008111	protein coding
RRM2B	*TRIB1*$^+$ Satellite cells	-0.869	3.77	0.00836	ENSSSCG00000036139	protein coding

MSTRG.11009	*TRIB1*$^{+}$ Satellite cells	-0.866	3.83	0.0128	MSTRG.11009	lncRNA
MSTRG.6795	*TRIB1*$^{+}$ Satellite cells	-0.866	3.72	0.0306	MSTRG.6795	lncRNA
FGGY	*TRIB1*$^{+}$ Satellite cells	-0.865	4.87	0.00737	ENSSSCG00000003828	protein coding
MSTRG.3697	*TRIB1*$^{+}$ Satellite cells	-0.863	5.78	0.0219	MSTRG.3697	lncRNA
FOXRED2	*TRIB1*$^{+}$ Satellite cells	-0.862	5.33	0.00161	ENSSSCG00000000142	protein coding
ARMC2	*TRIB1*$^{+}$ Satellite cells	-0.859	5.73	0.00814	ENSSSCG00000004388	protein coding
MDC1	*TRIB1*$^{+}$ Satellite cells	-0.858	3.51	0.0274	ENSSSCG00000001362	protein coding
SNED1	*TRIB1*$^{+}$ Satellite cells	-0.858	5.66	0.016	ENSSSCG00000016381	protein coding
TRAF3IP2	*TRIB1*$^{+}$ Satellite cells	-0.858	3.48	0.0219	ENSSSCG00000004420	protein coding
N6AMT1	*TRIB1*$^{+}$ Satellite cells	-0.857	3.95	0.0432	ENSSSCG00000028724	protein coding
SNTB1	*TRIB1*$^{+}$ Satellite cells	-0.856	6.17	0.0112	ENSSSCG00000005994	protein coding
RRAGD	*TRIB1*$^{+}$ Satellite cells	-0.855	5.03	0.0126	ENSSSCG00000004318	protein coding
ZNF740	*TRIB1*$^{+}$ Satellite cells	-0.855	3.11	0.0401	ENSSSCG00000036564	protein coding
MSTRG.43970	*TRIB1*$^{+}$ Satellite cells	-0.854	4.3	0.0333	MSTRG.43970	lncRNA
NUP214	*TRIB1*$^{+}$ Satellite cells	-0.853	4.57	0.00334	ENSSSCG00000005711	protein coding
TBC1D8	*TRIB1*$^{+}$ Satellite cells	-0.847	8.35	0.00171	ENSSSCG00000008169	protein coding
CLEC14A	*TRIB1*$^{+}$ Satellite cells	-0.846	3.44	0.0254	ENSSSCG00000023760	protein coding
HMCN2	*TRIB1*$^{+}$ Satellite cells	-0.842	8.75	0.0025	ENSSSCG00000005699	protein coding
PRRC2A	*TRIB1*$^{+}$ Satellite cells	-0.841	5.62	0.00159	ENSSSCG00000001409	protein coding
RHOJ	*TRIB1*$^{+}$ Satellite cells	-0.839	6.27	0.0183	ENSSSCG00000022804	protein coding
INTS3	*TRIB1*$^{+}$ Satellite cells	-0.829	3.9	0.00835	ENSSSCG00000006571	protein coding
DDRGK1	*TRIB1*$^{+}$ Satellite cells	-0.828	5.6	0.000943	ENSSSCG00000007160	protein coding
SLC25A25	*TRIB1*$^{+}$ Satellite cells	-0.826	5.73	0.00382	ENSSSCG00000005636	protein coding
EMG1	*TRIB1*$^{+}$ Satellite cells	-0.819	5.98	0.00698	ENSSSCG00000026812	protein coding
POR	*TRIB1*$^{+}$ Satellite cells	-0.817	6.01	0.00277	ENSSSCG00000038320	protein coding
PAAF1	*TRIB1*$^{+}$ Satellite cells	-0.816	4.26	0.0144	ENSSSCG00000014831	protein coding
ZC3H7B	*TRIB1*$^{+}$ Satellite cells	-0.813	4.98	0.00194	ENSSSCG00000033036	protein coding
STX18	*TRIB1*$^{+}$ Satellite cells	-0.81	5.35	0.011	ENSSSCG00000038731	protein coding
ENSSSCG00000035371	*TRIB1*$^{+}$ Satellite cells	-0.808	4.09	0.0352	ENSSSCG00000035371	protein coding
ST3GAL4	*TRIB1*$^{+}$ Satellite cells	-0.808	6.14	0.0038	ENSSSCG00000015232	protein coding
CHST8	*TRIB1*$^{+}$ Satellite cells	-0.807	4.11	0.0163	ENSSSCG00000035222	protein coding
MSTRG.16606	*TRIB1*$^{+}$ Satellite cells	-0.806	5.37	0.0021	MSTRG.16606	lncRNA
ENSSSCG00000044326	*TRIB1*$^{+}$ Satellite cells	-0.805	4.13	0.0167	ENSSSCG00000044326	lncRNA
LRRK1	*TRIB1*$^{+}$ Satellite cells	-0.804	3.37	0.0311	ENSSSCG00000004823	protein coding

MSTRG.20405	*TRIB1* + Satellite cells	-0.803	4.41	0.0153	MSTRG.20405	lncRNA
PDSS1	*TRIB1* + Satellite cells	-0.803	3.53	0.0211	ENSSSCG00000011061	protein coding
PPARGC1A	*TRIB1* + Satellite cells	-0.802	6.57	0.0188	ENSSSCG00000029275	protein coding
SEMA4B	*TRIB1* + Satellite cells	-0.8	5.14	0.00625	ENSSSCG00000031719	protein coding
MLXIP	*TRIB1* + Satellite cells	-0.798	4.48	0.00735	ENSSSCG00000009794	protein coding
SLC7A1	*TRIB1* + Satellite cells	-0.798	4.24	0.0473	ENSSSCG00000029456	protein coding
MSTRG.43966	*TRIB1* + Satellite cells	-0.796	3.62	0.0277	MSTRG.43966	lncRNA
ENSSSCG00000041264	*TRIB1* + Satellite cells	-0.794	5.67	0.00787	ENSSSCG00000041264	protein coding
MLH3	*TRIB1* + Satellite cells	-0.792	3.42	0.0256	ENSSSCG00000002378	protein coding
PPP1R10	*TRIB1* + Satellite cells	-0.79	6.57	0.0018	ENSSSCG00000001347	protein coding
LTBP3	*TRIB1* + Satellite cells	-0.787	5.36	0.0194	ENSSSCG00000012985	protein coding
MSTRG.17861	*TRIB1* + Satellite cells	-0.786	4.14	0.0295	MSTRG.17861	lncRNA
SPRY4	*TRIB1* + Satellite cells	-0.784	3.91	0.0352	ENSSSCG00000036322	protein coding
SESN1	*TRIB1* + Satellite cells	-0.782	7.6	0.00863	ENSSSCG00000004390	protein coding
TTC39C	*TRIB1* + Satellite cells	-0.782	3.48	0.0307	ENSSSCG00000003710	protein coding
SLC41A3	*TRIB1* + Satellite cells	-0.78	3.31	0.0272	ENSSSCG00000001825	protein coding
MSTRG.3698	*TRIB1* + Satellite cells	-0.779	5.48	0.0161	MSTRG.3698	lncRNA
DDC	*TRIB1* + Satellite cells	-0.778	6.28	0.00269	ENSSSCG00000022009	protein coding
SETBP1	*TRIB1* + Satellite cells	-0.776	5.91	0.00799	ENSSSCG00000004490	protein coding
GLI2	*TRIB1* + Satellite cells	-0.775	8.44	0.00377	ENSSSCG00000015733	protein coding
PLXNA2	*TRIB1* + Satellite cells	-0.775	6.83	0.00322	ENSSSCG00000026412	protein coding
EIF4G1	*TRIB1* + Satellite cells	-0.773	4.75	0.00565	ENSSSCG00000030255	protein coding
FBXW4	*TRIB1* + Satellite cells	-0.773	5.16	0.0159	ENSSSCG00000010566	protein coding
ADGRE5	*TRIB1* + Satellite cells	-0.772	4.37	0.0304	ENSSSCG00000013775	protein coding
ENSSSCG00000028635	*TRIB1* + Satellite cells	-0.771	5.32	0.0241	ENSSSCG00000028635	protein coding
LIFR	*TRIB1* + Satellite cells	-0.763	7.48	0.00276	ENSSSCG00000016850	protein coding
CTTNBP2	*TRIB1* + Satellite cells	-0.76	3.47	0.031	ENSSSCG00000016625	protein coding
ELK4	*TRIB1* + Satellite cells	-0.756	3.79	0.0137	ENSSSCG00000025182	protein coding
ENSSSCG00000004082	*TRIB1* + Satellite cells	-0.756	6.62	0.00968	ENSSSCG00000004082	protein coding
LMNB2	*TRIB1* + Satellite cells	-0.756	3.92	0.0436	ENSSSCG00000026784	protein coding
FYCO1	*TRIB1* + Satellite cells	-0.754	5.52	0.0167	ENSSSCG00000011317	protein coding
NPLOC4	*TRIB1* + Satellite cells	-0.754	3.37	0.0215	ENSSSCG00000024235	protein coding
SEC14L1	*TRIB1* + Satellite cells	-0.753	4.97	0.00718	ENSSSCG00000017169	protein coding
TLN2	*TRIB1* + Satellite cells	-0.753	8.81	0.0224	ENSSSCG00000024088	protein coding

IL21R	*TRIB1*$^+$ Satellite cells	-0.751	3.49	0.0275	ENSSSCG00000007816	protein coding
SEMA4D	*TRIB1*$^+$ Satellite cells	-0.751	5.68	0.00201	ENSSSCG00000009584	protein coding
ZFAND5	*TRIB1*$^+$ Satellite cells	-0.75	8.68	0.012	ENSSSCG00000030268	protein coding
MLIP	*TRIB1*$^+$ Satellite cells	-0.749	6.06	0.0277	ENSSSCG00000001485	protein coding
MSTRG.10446	*TRIB1*$^+$ Satellite cells	-0.748	5.02	0.0217	MSTRG.10446	lncRNA
PRSS23	*TRIB1*$^+$ Satellite cells	-0.746	7.79	0.0204	ENSSSCG00000014921	protein coding
HDHD2	*TRIB1*$^+$ Satellite cells	-0.745	4.49	0.0242	ENSSSCG00000005771	protein coding
ADAMTS9	*TRIB1*$^+$ Satellite cells	-0.741	6.8	0.00902	ENSSSCG00000011496	protein coding
PRKCB	*TRIB1*$^+$ Satellite cells	-0.74	4.39	0.0302	ENSSSCG00000033674	protein coding
CNKSR2	*TRIB1*$^+$ Satellite cells	-0.738	6.39	0.0178	ENSSSCG00000012164	protein coding
NOP2	*TRIB1*$^+$ Satellite cells	-0.737	4.89	0.0229	ENSSSCG00000000696	protein coding
TLE3	*TRIB1*$^+$ Satellite cells	-0.733	5.24	0.00468	ENSSSCG00000004971	protein coding
TXLNA	*TRIB1*$^+$ Satellite cells	-0.733	4.92	0.00391	ENSSSCG00000003617	protein coding
ATP1B1	*TRIB1*$^+$ Satellite cells	-0.732	4.27	0.0373	ENSSSCG00000006296	protein coding
LRP5	*TRIB1*$^+$ Satellite cells	-0.732	5.48	0.00693	ENSSSCG00000012885	protein coding
PAK1IP1	*TRIB1*$^+$ Satellite cells	-0.728	5.03	0.00738	ENSSSCG00000001041	protein coding
SRSF7	*TRIB1*$^+$ Satellite cells	-0.727	7.74	0.00533	ENSSSCG00000008484	protein coding
TPR	*TRIB1*$^+$ Satellite cells	-0.726	5.65	0.0137	ENSSSCG00000015576	protein coding
UTP20	*TRIB1*$^+$ Satellite cells	-0.726	4.45	0.0209	ENSSSCG00000000869	protein coding
BAP1	*TRIB1*$^+$ Satellite cells	-0.724	3.78	0.0182	ENSSSCG00000011438	protein coding
MSTRG.17048	*TRIB1*$^+$ Satellite cells	-0.724	4.88	0.00783	MSTRG.17048	lncRNA
FOXO1	*TRIB1*$^+$ Satellite cells	-0.722	6.89	0.00345	ENSSSCG00000009370	protein coding
GAP43	*TRIB1*$^+$ Satellite cells	-0.719	3.93	0.0474	ENSSSCG00000031244	protein coding
RNF20	*TRIB1*$^+$ Satellite cells	-0.718	3.49	0.0388	ENSSSCG00000005399	protein coding
GOLGA1	*TRIB1*$^+$ Satellite cells	-0.717	3.7	0.0375	ENSSSCG00000005597	protein coding
POLR3E	*TRIB1*$^+$ Satellite cells	-0.717	3.94	0.0264	ENSSSCG00000007840	protein coding
LPCAT3	*TRIB1*$^+$ Satellite cells	-0.715	5.08	0.0106	ENSSSCG00000027205	protein coding
MSTRG.9304	*TRIB1*$^+$ Satellite cells	-0.713	5.67	0.0194	MSTRG.9304	lncRNA
PRKDC	*TRIB1*$^+$ Satellite cells	-0.712	4.11	0.0246	ENSSSCG00000006274	protein coding
GALNT10	*TRIB1*$^+$ Satellite cells	-0.711	3.94	0.0274	ENSSSCG00000017072	protein coding
DUSP23	*TRIB1*$^+$ Satellite cells	-0.708	4.19	0.0219	ENSSSCG00000033585	protein coding
PDCD11	*TRIB1*$^+$ Satellite cells	-0.708	3.36	0.0359	ENSSSCG00000010599	protein coding
CKAP5	*TRIB1*$^+$ Satellite cells	-0.707	3.91	0.0212	ENSSSCG00000013249	protein coding
TOR3A	*TRIB1*$^+$ Satellite cells	-0.706	4.49	0.0211	ENSSSCG00000015525	protein coding

ZCCHC14	*TRIB1* + Satellite cells	-0.706	5	0.0137	ENSSSCG00000002656	protein coding
ZMYND8	*TRIB1* + Satellite cells	-0.705	3.91	0.0462	ENSSSCG00000007454	protein coding
TIPARP	*TRIB1* + Satellite cells	-0.703	6.39	0.00224	ENSSSCG00000027646	protein coding
ARID1A	*TRIB1* + Satellite cells	-0.701	4.4	0.0171	ENSSSCG00000003559	protein coding
APBB2	*TRIB1* + Satellite cells	-0.7	8.22	0.0142	ENSSSCG00000022168	protein coding
ENSSSCG00000010860	*TRIB1* + Satellite cells	-0.697	4.37	0.0233	ENSSSCG00000010860	protein coding
MSTRG.32707	*TRIB1* + Satellite cells	-0.696	5.34	0.00943	MSTRG.32707	lncRNA
ENSSSCG00000005255	*TRIB1* + Satellite cells	-0.694	6.7	0.0059	ENSSSCG00000005255	protein coding
CLUH	*TRIB1* + Satellite cells	-0.693	3.69	0.0263	ENSSSCG00000017835	protein coding
COL14A1	*TRIB1* + Satellite cells	-0.688	9.16	0.0163	ENSSSCG00000005997	protein coding
MSTRG.38012	*TRIB1* + Satellite cells	-0.686	10.5	0.0494	MSTRG.38012	lncRNA
TNRC6C	*TRIB1* + Satellite cells	-0.685	5.55	0.00834	ENSSSCG00000034373	protein coding
EZH1	*TRIB1* + Satellite cells	-0.684	4.43	0.0351	ENSSSCG00000017382	protein coding
MSTRG.7583	*TRIB1* + Satellite cells	-0.684	3.44	0.0455	MSTRG.7583	lncRNA
ZNF274	*TRIB1* + Satellite cells	-0.684	3.96	0.0426	ENSSSCG00000003998	protein coding
MSTRG.36142	*TRIB1* + Satellite cells	-0.683	3.53	0.0417	MSTRG.36142	lncRNA
MRPL34	*TRIB1* + Satellite cells	-0.682	5.03	0.0329	ENSSSCG00000039295	protein coding
MSTRG.24601	*TRIB1* + Satellite cells	-0.682	5.2	0.0176	MSTRG.24601	lncRNA
DNMT1	*TRIB1* + Satellite cells	-0.681	4.87	0.00919	ENSSSCG00000013659	protein coding
SOCS3	*TRIB1* + Satellite cells	-0.681	6.46	0.0356	ENSSSCG00000036956	protein coding
CCNL2	*TRIB1* + Satellite cells	-0.68	4.78	0.0105	ENSSSCG00000003354	protein coding
ENSSSCG00000006682	*TRIB1* + Satellite cells	-0.679	3.59	0.0311	ENSSSCG00000006682	protein coding
USP24	*TRIB1* + Satellite cells	-0.678	5.4	0.00918	ENSSSCG00000029633	protein coding
KDM4B	*TRIB1* + Satellite cells	-0.677	4.92	0.00548	ENSSSCG00000024570	protein coding
LDB2	*TRIB1* + Satellite cells	-0.676	4.78	0.0215	ENSSSCG00000029227	protein coding
ARL4A	*TRIB1* + Satellite cells	-0.675	4.39	0.0157	ENSSSCG00000032709	protein coding
MTHFD1	*TRIB1* + Satellite cells	-0.672	4.81	0.0482	ENSSSCG00000002269	protein coding
PMVK	*TRIB1* + Satellite cells	-0.672	4.48	0.0253	ENSSSCG00000006535	protein coding
ENSSSCG00000010440	*TRIB1* + Satellite cells	-0.666	5.33	0.0297	ENSSSCG00000010440	protein coding
MSTRG.46047	*TRIB1* + Satellite cells	-0.663	4.82	0.0124	MSTRG.46047	lncRNA
MSTRG.30567	*TRIB1* + Satellite cells	-0.661	3.95	0.0477	MSTRG.30567	lncRNA
WDR92	*TRIB1* + Satellite cells	-0.661	4.25	0.0252	ENSSSCG00000008353	protein coding
NMT2	*TRIB1* + Satellite cells	-0.66	3.95	0.0471	ENSSSCG00000026499	protein coding
PARVA	*TRIB1* + Satellite cells	-0.659	6.68	0.00581	ENSSSCG00000028827	protein coding

ABCF1	*TRIB1*$^{+}$ Satellite cells	-0.658	4.53	0.00907	ENSSSCG00000001346	protein coding
ZNF710	*TRIB1*$^{+}$ Satellite cells	-0.658	3.5	0.0468	ENSSSCG00000021361	protein coding
ESF1	*TRIB1*$^{+}$ Satellite cells	-0.656	5.65	0.0184	ENSSSCG00000007077	protein coding
FPGS	*TRIB1*$^{+}$ Satellite cells	-0.656	3.81	0.0444	ENSSSCG00000005626	protein coding
MSTRG.16656	*TRIB1*$^{+}$ Satellite cells	-0.656	3.76	0.0454	MSTRG.16656	lncRNA
ZNRF3	*TRIB1*$^{+}$ Satellite cells	-0.655	4.09	0.0256	ENSSSCG00000009971	protein coding
LRMDA	*TRIB1*$^{+}$ Satellite cells	-0.654	7.97	0.0209	ENSSSCG00000034308	protein coding
MGA	*TRIB1*$^{+}$ Satellite cells	-0.654	4.73	0.042	ENSSSCG00000004739	protein coding
IFT172	*TRIB1*$^{+}$ Satellite cells	-0.653	4.54	0.0351	ENSSSCG00000026367	protein coding
POLR1G	*TRIB1*$^{+}$ Satellite cells	-0.653	4.41	0.0411	ENSSSCG00000020754	protein coding
GCN1	*TRIB1*$^{+}$ Satellite cells	-0.652	4.93	0.0151	ENSSSCG00000009898	protein coding
ZC3H18	*TRIB1*$^{+}$ Satellite cells	-0.652	4.21	0.0487	ENSSSCG00000035967	protein coding
ADPRS	*TRIB1*$^{+}$ Satellite cells	-0.65	4.17	0.0313	ENSSSCG00000003634	protein coding
TOE1	*TRIB1*$^{+}$ Satellite cells	-0.65	4.49	0.0345	ENSSSCG00000003918	protein coding
NAMPT	*TRIB1*$^{+}$ Satellite cells	-0.648	6.5	0.029	ENSSSCG00000015435	protein coding
ENSSSCG00000001229	*TRIB1*$^{+}$ Satellite cells	-0.644	7.94	0.00592	ENSSSCG00000001229	protein coding
IL1RAP	*TRIB1*$^{+}$ Satellite cells	-0.642	6.16	0.0113	ENSSSCG00000021206	protein coding
MLLT6	*TRIB1*$^{+}$ Satellite cells	-0.642	4.19	0.0344	ENSSSCG00000032478	protein coding
CDH4	*TRIB1*$^{+}$ Satellite cells	-0.641	5.67	0.0255	ENSSSCG00000025784	protein coding
SEC22C	*TRIB1*$^{+}$ Satellite cells	-0.641	3.73	0.0342	ENSSSCG00000034200	protein coding
MSTRG.49624	*TRIB1*$^{+}$ Satellite cells	-0.639	5.41	0.0424	MSTRG.49624	lncRNA
PRR14L	*TRIB1*$^{+}$ Satellite cells	-0.639	3.83	0.0335	ENSSSCG00000010033	protein coding
NCL	*TRIB1*$^{+}$ Satellite cells	-0.638	6.93	0.0133	ENSSSCG00000016275	protein coding
CACNB2	*TRIB1*$^{+}$ Satellite cells	-0.637	3.88	0.047	ENSSSCG00000011040	protein coding
MSTRG.30836	*TRIB1*$^{+}$ Satellite cells	-0.633	6.16	0.0386	MSTRG.30836	lncRNA
MAN2A2	*TRIB1*$^{+}$ Satellite cells	-0.632	4.42	0.0235	ENSSSCG00000001819	protein coding
TRIM28	*TRIB1*$^{+}$ Satellite cells	-0.632	5.24	0.0186	ENSSSCG00000037920	protein coding
KIFBP	*TRIB1*$^{+}$ Satellite cells	-0.631	4.49	0.0209	ENSSSCG00000010248	protein coding
GART	*TRIB1*$^{+}$ Satellite cells	-0.628	4.35	0.0184	ENSSSCG00000030677	protein coding
PLOD1	*TRIB1*$^{+}$ Satellite cells	-0.628	6.58	0.00784	ENSSSCG00000003433	protein coding
ZNF503	*TRIB1*$^{+}$ Satellite cells	-0.628	4.43	0.0299	ENSSSCG00000010322	protein coding
IKBIP	*TRIB1*$^{+}$ Satellite cells	-0.627	5.38	0.00989	ENSSSCG00000038772	protein coding
MSTRG.8115	*TRIB1*$^{+}$ Satellite cells	-0.624	5.76	0.0267	MSTRG.8115	lncRNA
ENSSSCG00000012264	*TRIB1*$^{+}$ Satellite cells	-0.623	3.86	0.0356	ENSSSCG00000012264	protein coding

TASOR2	*TRIB1* +	Satellite cells	-0.623	4.17	0.0416	ENSSSCG00000011136	protein coding
ENSSSCG00000004830	*TRIB1* +	Satellite cells	-0.619	6.51	0.0136	ENSSSCG00000004830	protein coding
C16orf91	*TRIB1* +	Satellite cells	-0.618	3.96	0.0492	ENSSSCG00000038882	protein coding
LIG3	*TRIB1* +	Satellite cells	-0.617	3.76	0.0405	ENSSSCG00000024770	protein coding
CORO1C	*TRIB1* +	Satellite cells	-0.615	7.77	0.00607	ENSSSCG00000009944	protein coding
LONRF3	*TRIB1* +	Satellite cells	-0.615	7.13	0.0116	ENSSSCG00000012631	protein coding
MCFD2	*TRIB1* +	Satellite cells	-0.615	5.28	0.0155	ENSSSCG00000021325	protein coding
ABCF3	*TRIB1* +	Satellite cells	-0.614	4.66	0.0194	ENSSSCG00000028856	protein coding
ZFAT	*TRIB1* +	Satellite cells	-0.611	4	0.0316	ENSSSCG00000030947	protein coding
MSTRG.3562	*TRIB1* +	Satellite cells	-0.61	4.32	0.045	MSTRG.3562	lncRNA
NUMA1	*TRIB1* +	Satellite cells	-0.61	5.3	0.00917	ENSSSCG00000014802	protein coding
GNAI2	*TRIB1* +	Satellite cells	-0.604	3.74	0.0426	ENSSSCG00000011399	protein coding
DNAJC14	*TRIB1* +	Satellite cells	-0.603	4.67	0.0166	ENSSSCG00000000366	protein coding
ENSSSCG00000000107	*TRIB1* +	Satellite cells	-0.602	4.62	0.0145	ENSSSCG00000000107	protein coding
PRPF8	*TRIB1* +	Satellite cells	-0.601	5.15	0.00752	ENSSSCG00000017817	protein coding
IFT27	*TRIB1* +	Satellite cells	-0.6	4.35	0.0318	ENSSSCG00000000139	protein coding
ENSSSCG00000016119	*TRIB1* +	Satellite cells	-0.598	8.56	0.0233	ENSSSCG00000016119	protein coding
RPRD2	*TRIB1* +	Satellite cells	-0.597	5.25	0.0254	ENSSSCG00000030291	protein coding
SETD7	*TRIB1* +	Satellite cells	-0.596	5.44	0.0483	ENSSSCG00000030396	protein coding
ADCY9	*TRIB1* +	Satellite cells	-0.593	5.39	0.0194	ENSSSCG00000007950	protein coding
AMOTL1	*TRIB1* +	Satellite cells	-0.593	6.67	0.018	ENSSSCG00000014960	protein coding
ENSSSCG00000013361	*TRIB1* +	Satellite cells	-0.593	5.8	0.0235	ENSSSCG00000013361	protein coding
C21orf91	*TRIB1* +	Satellite cells	-0.592	6.89	0.0314	ENSSSCG00000033025	protein coding
COL18A1	*TRIB1* +	Satellite cells	-0.592	6.34	0.0494	ENSSSCG00000038089	protein coding
ECE1	*TRIB1* +	Satellite cells	-0.59	4.64	0.049	ENSSSCG00000003513	protein coding
MSTRG.20211	*TRIB1* +	Satellite cells	-0.59	3.66	0.0429	MSTRG.20211	lncRNA
SNRNP70	*TRIB1* +	Satellite cells	-0.59	6.68	0.0153	ENSSSCG00000023792	protein coding
WHAMM	*TRIB1* +	Satellite cells	-0.59	4.58	0.0177	ENSSSCG00000001805	protein coding
HSPG2	*TRIB1* +	Satellite cells	-0.589	8.63	0.00683	ENSSSCG00000003514	protein coding
MRPL16	*TRIB1* +	Satellite cells	-0.586	4.51	0.02	ENSSSCG00000013118	protein coding
RPTOR	*TRIB1* +	Satellite cells	-0.586	5.63	0.0112	ENSSSCG00000017139	protein coding
MSTRG.9331	*TRIB1* +	Satellite cells	-0.585	4.61	0.032	MSTRG.9331	lncRNA
NAA20	*TRIB1* +	Satellite cells	-0.585	6.87	0.00903	ENSSSCG00000028132	protein coding
SZT2	*TRIB1* +	Satellite cells	-0.585	3.86	0.0474	ENSSSCG00000003941	protein coding

MSTRG.21931	*TRIB1*$^{+}$ Satellite cells	-0.58	5.71	0.00894	MSTRG.21931	lncRNA
MSTRG.2004	*TRIB1*$^{+}$ Satellite cells	-0.576	7.53	0.0394	MSTRG.2004	lncRNA
NVL	*TRIB1*$^{+}$ Satellite cells	-0.574	4.87	0.0302	ENSSSCG00000026705	protein coding
USP40	*TRIB1*$^{+}$ Satellite cells	-0.571	5.03	0.0455	ENSSSCG00000016306	protein coding
ENSSSCG00000034711	*TRIB1*$^{+}$ Satellite cells	-0.569	4.76	0.0266	ENSSSCG00000034711	protein coding
TNFSF13	*TRIB1*$^{+}$ Satellite cells	-0.569	4.79	0.0215	ENSSSCG00000036521	protein coding
CLTC	*TRIB1*$^{+}$ Satellite cells	-0.568	5.77	0.0425	ENSSSCG00000022659	protein coding
DNMT3A	*TRIB1*$^{+}$ Satellite cells	-0.566	5.09	0.017	ENSSSCG00000029413	protein coding
MSTRG.32453	*TRIB1*$^{+}$ Satellite cells	-0.566	5.07	0.041	MSTRG.32453	lncRNA
BUD23	*TRIB1*$^{+}$ Satellite cells	-0.565	6.73	0.046	ENSSSCG00000007713	protein coding
NECAP1	*TRIB1*$^{+}$ Satellite cells	-0.564	4.07	0.0429	ENSSSCG00000025179	protein coding
IARS2	*TRIB1*$^{+}$ Satellite cells	-0.563	5.25	0.043	ENSSSCG00000010823	protein coding
TBL2	*TRIB1*$^{+}$ Satellite cells	-0.563	4.78	0.0243	ENSSSCG00000007709	protein coding
ANO5	*TRIB1*$^{+}$ Satellite cells	-0.562	4.06	0.0415	ENSSSCG00000013344	protein coding
ACLY	*TRIB1*$^{+}$ Satellite cells	-0.557	4.87	0.0246	ENSSSCG00000017421	protein coding
WDR47	*TRIB1*$^{+}$ Satellite cells	-0.557	4.79	0.0476	ENSSSCG00000006840	protein coding
ENSSSCG00000050854	*TRIB1*$^{+}$ Satellite cells	-0.556	5.98	0.011	ENSSSCG00000050854	lncRNA
SLTM	*TRIB1*$^{+}$ Satellite cells	-0.556	5.39	0.0282	ENSSSCG00000004592	protein coding
CHCHD3	*TRIB1*$^{+}$ Satellite cells	-0.555	4.43	0.0418	ENSSSCG00000016545	protein coding
JSRP1	*TRIB1*$^{+}$ Satellite cells	-0.553	7.47	0.00791	ENSSSCG00000031899	protein coding
COL5A1	*TRIB1*$^{+}$ Satellite cells	-0.548	7.61	0.0496	ENSSSCG00000005751	protein coding
EIF3B	*TRIB1*$^{+}$ Satellite cells	-0.541	4.2	0.0458	ENSSSCG00000039390	protein coding
ENSSSCG00000050244	*TRIB1*$^{+}$ Satellite cells	-0.539	6.53	0.0176	ENSSSCG00000050244	protein coding
CGNL1	*TRIB1*$^{+}$ Satellite cells	-0.538	5.45	0.0264	ENSSSCG00000035038	protein coding
MTG1	*TRIB1*$^{+}$ Satellite cells	-0.538	4.33	0.0375	ENSSSCG00000029088	protein coding
FGFR1	*TRIB1*$^{+}$ Satellite cells	-0.536	6.11	0.0199	ENSSSCG00000015815	protein coding
MSTRG.48556	*TRIB1*$^{+}$ Satellite cells	-0.536	4.76	0.0332	MSTRG.48556	lncRNA
ANTXR2	*TRIB1*$^{+}$ Satellite cells	-0.534	6.11	0.0472	ENSSSCG00000009256	protein coding
SMC1A	*TRIB1*$^{+}$ Satellite cells	-0.531	5.71	0.026	ENSSSCG00000012325	protein coding
ZNF862	*TRIB1*$^{+}$ Satellite cells	-0.53	4.48	0.0376	ENSSSCG00000040257	protein coding
MYH14	*TRIB1*$^{+}$ Satellite cells	-0.528	7.22	0.0113	ENSSSCG00000027407	protein coding
PTCH1	*TRIB1*$^{+}$ Satellite cells	-0.528	4.5	0.041	ENSSSCG00000027312	protein coding
RRP36	*TRIB1*$^{+}$ Satellite cells	-0.528	5.79	0.0247	ENSSSCG00000001656	protein coding
GTPBP1	*TRIB1*$^{+}$ Satellite cells	-0.523	4.87	0.042	ENSSSCG00000000095	protein coding

ZC3H6	*TRIB1*$^{+}$ Satellite cells	-0.522	5.45	0.034	ENSSSCG00000008096	protein coding
LMBRD2	*TRIB1*$^{+}$ Satellite cells	-0.521	4.69	0.0239	ENSSSCG00000016834	protein coding
GNL2	*TRIB1*$^{+}$ Satellite cells	-0.52	4.53	0.0296	ENSSSCG00000031631	protein coding
ENSSSCG00000037376	*TRIB1*$^{+}$ Satellite cells	-0.519	6.33	0.0163	ENSSSCG00000037376	protein coding
SUN1	*TRIB1*$^{+}$ Satellite cells	-0.518	7.1	0.0102	ENSSSCG00000007544	protein coding
DDX18	*TRIB1*$^{+}$ Satellite cells	-0.516	4.9	0.0322	ENSSSCG00000015712	protein coding
ENSSSCG00000000145	*TRIB1*$^{+}$ Satellite cells	-0.514	5.41	0.0387	ENSSSCG00000000145	protein coding
ZFP90	*TRIB1*$^{+}$ Satellite cells	-0.514	4.68	0.0458	ENSSSCG00000032372	protein coding
DAG1	*TRIB1*$^{+}$ Satellite cells	-0.513	9.37	0.02	ENSSSCG00000034357	protein coding
ENSSSCG00000047749	*TRIB1*$^{+}$ Satellite cells	-0.511	4.34	0.0389	ENSSSCG00000047749	protein coding
CNP	*TRIB1*$^{+}$ Satellite cells	-0.509	5.21	0.0296	ENSSSCG00000017420	protein coding
AKAP8	*TRIB1*$^{+}$ Satellite cells	-0.507	4.87	0.0402	ENSSSCG00000013837	protein coding
GUCD1	*TRIB1*$^{+}$ Satellite cells	-0.506	5.44	0.0418	ENSSSCG00000010059	protein coding
ENSSSCG00000036213	*TRIB1*$^{+}$ Satellite cells	-0.502	4.92	0.0273	ENSSSCG00000036213	protein coding
SYNRG	*TRIB1*$^{+}$ Satellite cells	-0.502	4.71	0.0317	ENSSSCG00000026596	protein coding
ENSSSCG00000040571	*TRIB1*$^{+}$ Satellite cells	-0.5	5.75	0.0244	ENSSSCG00000040571	protein coding
CHD3	*TRIB1*$^{+}$ Satellite cells	-0.497	6.42	0.0411	ENSSSCG00000021363	protein coding
ENSSSCG00000043464	*TRIB1*$^{+}$ Satellite cells	-0.497	5.07	0.0429	ENSSSCG00000043464	lncRNA
ENSSSCG00000002259	*TRIB1*$^{+}$ Satellite cells	-0.496	8.37	0.0126	ENSSSCG00000002259	protein coding
RAB11FIP3	*TRIB1*$^{+}$ Satellite cells	-0.496	5.02	0.0368	ENSSSCG00000035854	protein coding
CUEDC1	*TRIB1*$^{+}$ Satellite cells	-0.492	4.73	0.0418	ENSSSCG00000017624	protein coding
MSTRG.29959	*TRIB1*$^{+}$ Satellite cells	-0.492	5.6	0.0423	MSTRG.29959	lncRNA
ENSSSCG00000004207	*TRIB1*$^{+}$ Satellite cells	-0.489	7.5	0.0499	ENSSSCG00000004207	protein coding
NFIX	*TRIB1*$^{+}$ Satellite cells	-0.489	6.99	0.0229	ENSSSCG00000013742	protein coding
PPP1R12B	*TRIB1*$^{+}$ Satellite cells	-0.489	5.12	0.0407	ENSSSCG00000010925	protein coding
REV3L	*TRIB1*$^{+}$ Satellite cells	-0.489	7.81	0.0344	ENSSSCG00000025703	protein coding
DDX42	*TRIB1*$^{+}$ Satellite cells	-0.488	4.82	0.0332	ENSSSCG00000017289	protein coding
MSTRG.28397	*TRIB1*$^{+}$ Satellite cells	-0.488	5.47	0.038	MSTRG.28397	lncRNA
DCTPP1	*TRIB1*$^{+}$ Satellite cells	-0.487	6.45	0.0141	ENSSSCG00000007795	protein coding
NRCAM	*TRIB1*$^{+}$ Satellite cells	-0.486	7.05	0.0266	ENSSSCG00000015445	protein coding
ZHX3	*TRIB1*$^{+}$ Satellite cells	-0.478	7.27	0.0313	ENSSSCG00000032566	protein coding
GLG1	*TRIB1*$^{+}$ Satellite cells	-0.477	6.87	0.0157	ENSSSCG00000030420	protein coding
SGCA	*TRIB1*$^{+}$ Satellite cells	-0.477	7.88	0.0163	ENSSSCG00000017583	protein coding
TRRAP	*TRIB1*$^{+}$ Satellite cells	-0.475	4.72	0.047	ENSSSCG00000007606	protein coding

NCOR2	*TRIB1* $^{+}$	Satellite cells	-0.473	7.02	0.0289	ENSSSCG00000009761	protein coding
ETV5	*TRIB1* $^{+}$	Satellite cells	-0.472	6.97	0.0377	ENSSSCG00000023229	protein coding
ENSSSCG00000033497	*TRIB1* $^{+}$	Satellite cells	-0.469	7.51	0.0339	ENSSSCG00000033497	protein coding
NOP56	*TRIB1* $^{+}$	Satellite cells	-0.468	6.16	0.0354	ENSSSCG00000027676	protein coding
RORA	*TRIB1* $^{+}$	Satellite cells	-0.463	9.57	0.0488	ENSSSCG00000004576	protein coding
SESTD1	*TRIB1* $^{+}$	Satellite cells	-0.463	7.53	0.0472	ENSSSCG00000016005	protein coding
TBRG4	*TRIB1* $^{+}$	Satellite cells	-0.461	5.05	0.0481	ENSSSCG00000016732	protein coding
FBRSL1	*TRIB1* $^{+}$	Satellite cells	-0.459	4.52	0.0493	ENSSSCG00000023569	protein coding
SNRPA1	*TRIB1* $^{+}$	Satellite cells	-0.453	5.88	0.0496	ENSSSCG00000005786	protein coding
DDX23	*TRIB1* $^{+}$	Satellite cells	-0.447	4.96	0.0478	ENSSSCG00000000176	protein coding
GNE	*TRIB1* $^{+}$	Satellite cells	-0.445	5.74	0.0338	ENSSSCG00000037468	protein coding
WASL	*TRIB1* $^{+}$	Satellite cells	-0.434	6.3	0.0331	ENSSSCG00000016604	protein coding
ZFC3H1	*TRIB1* $^{+}$	Satellite cells	-0.415	6.55	0.0475	ENSSSCG00000000509	protein coding
LRP6	*TRIB1* $^{+}$	Satellite cells	-0.409	7.33	0.0306	ENSSSCG00000000625	protein coding
VPS72	*TRIB1* $^{+}$	Satellite cells	-0.405	5.13	0.0497	ENSSSCG00000031869	protein coding
EIF4A3	*TRIB1* $^{+}$	Satellite cells	-0.404	5.98	0.0376	ENSSSCG00000017155	protein coding
ILKAP	*TRIB1* $^{+}$	Satellite cells	-0.402	5.84	0.0449	ENSSSCG00000016339	protein coding
DHX9	*TRIB1* $^{+}$	Satellite cells	-0.393	6.81	0.041	ENSSSCG00000026806	protein coding
ENSSSCG00000034868	*TRIB1* $^{+}$	Satellite cells	-0.39	6.41	0.0356	ENSSSCG00000034868	protein coding
IDH2	*TRIB1* $^{+}$	Satellite cells	-0.386	7.14	0.0381	ENSSSCG00000001852	protein coding
NBR1	*TRIB1* $^{+}$	Satellite cells	-0.384	6.18	0.0488	ENSSSCG00000030211	protein coding
NDUFAB1	*TRIB1* $^{+}$	Satellite cells	-0.361	7.12	0.0494	ENSSSCG00000010795	protein coding
LSM6	*TRIB1* $^{+}$	Satellite cells	0.375	6.3	0.0479	ENSSSCG00000035973	protein coding
RPLP0	*TRIB1* $^{+}$	Satellite cells	0.38	12.3	0.0443	ENSSSCG00000036014	protein coding
HNRNPDL	*TRIB1* $^{+}$	Satellite cells	0.382	9.39	0.043	ENSSSCG00000037245	protein coding
ENSSSCG00000004470	*TRIB1* $^{+}$	Satellite cells	0.384	7.81	0.0409	ENSSSCG00000004470	protein coding
PCDH7	*TRIB1* $^{+}$	Satellite cells	0.389	9.73	0.0449	ENSSSCG00000008765	protein coding
NFE2L2	*TRIB1* $^{+}$	Satellite cells	0.391	7.51	0.0461	ENSSSCG00000015987	protein coding
LAPTM4A	*TRIB1* $^{+}$	Satellite cells	0.394	9.09	0.0345	ENSSSCG00000008602	protein coding
TBL1XR1	*TRIB1* $^{+}$	Satellite cells	0.396	8.07	0.0471	ENSSSCG00000011760	protein coding
ENSSSCG00000046054	*TRIB1* $^{+}$	Satellite cells	0.397	8.23	0.0301	ENSSSCG00000046054	lncRNA
RPL37	*TRIB1* $^{+}$	Satellite cells	0.398	12.1	0.0445	ENSSSCG00000033326	protein coding
ENSSSCG00000026488	*TRIB1* $^{+}$	Satellite cells	0.4	6.3	0.0391	ENSSSCG00000026488	protein coding
TOMM20	*TRIB1* $^{+}$	Satellite cells	0.401	7	0.0287	ENSSSCG00000010158	protein coding

ZFP36L1	*TRIB1*$^{+}$ Satellite cells	0.404	9.07	0.0419	ENSSSCG00000028304	protein coding
RPL22	*TRIB1*$^{+}$ Satellite cells	0.405	10.8	0.0471	ENSSSCG00000003369	protein coding
RPS29	*TRIB1*$^{+}$ Satellite cells	0.405	12	0.0433	ENSSSCG00000038507	protein coding
ARL8B	*TRIB1*$^{+}$ Satellite cells	0.407	5.85	0.0453	ENSSSCG00000011535	protein coding
SUPT4H1	*TRIB1*$^{+}$ Satellite cells	0.409	6.95	0.0318	ENSSSCG00000034752	protein coding
ENSSSCG00000032748	*TRIB1*$^{+}$ Satellite cells	0.411	6.28	0.0326	ENSSSCG00000032748	protein coding
UQCRC2	*TRIB1*$^{+}$ Satellite cells	0.414	6.96	0.035	ENSSSCG00000007845	protein coding
ENSSSCG00000033368	*TRIB1*$^{+}$ Satellite cells	0.416	8.4	0.0329	ENSSSCG00000033368	protein coding
NAA50	*TRIB1*$^{+}$ Satellite cells	0.416	5.62	0.0393	ENSSSCG00000011917	protein coding
EIF1AX	*TRIB1*$^{+}$ Satellite cells	0.417	6.66	0.0283	ENSSSCG00000032223	protein coding
MGAT4A	*TRIB1*$^{+}$ Satellite cells	0.417	6.32	0.0447	ENSSSCG00000008188	protein coding
ENSSSCG00000012523	*TRIB1*$^{+}$ Satellite cells	0.418	7.31	0.0347	ENSSSCG00000012523	protein coding
UBE2E3	*TRIB1*$^{+}$ Satellite cells	0.42	7.01	0.0274	ENSSSCG00000033987	protein coding
RAB18	*TRIB1*$^{+}$ Satellite cells	0.421	6.59	0.0476	ENSSSCG00000028655	protein coding
MET	*TRIB1*$^{+}$ Satellite cells	0.422	5.89	0.0452	ENSSSCG00000024351	protein coding
MYO5A	*TRIB1*$^{+}$ Satellite cells	0.423	7.24	0.0275	ENSSSCG00000004620	protein coding
MSTRG.9736	*TRIB1*$^{+}$ Satellite cells	0.424	7.95	0.0297	MSTRG.9736	lncRNA
RPL10	*TRIB1*$^{+}$ Satellite cells	0.424	12.6	0.0482	ENSSSCG00000035997	protein coding
RSU1	*TRIB1*$^{+}$ Satellite cells	0.424	6.24	0.0414	ENSSSCG00000011042	protein coding
CCNL1	*TRIB1*$^{+}$ Satellite cells	0.425	8.36	0.0355	ENSSSCG00000029352	protein coding
SNRPA	*TRIB1*$^{+}$ Satellite cells	0.432	5.34	0.0418	ENSSSCG00000002998	protein coding
EIF3E	*TRIB1*$^{+}$ Satellite cells	0.434	7.92	0.0403	ENSSSCG00000006033	protein coding
ETV6	*TRIB1*$^{+}$ Satellite cells	0.434	6.31	0.0376	ENSSSCG00000024152	protein coding
MYO1B	*TRIB1*$^{+}$ Satellite cells	0.436	6.41	0.0478	ENSSSCG00000016061	protein coding
RB1	*TRIB1*$^{+}$ Satellite cells	0.436	6.52	0.0436	ENSSSCG00000009401	protein coding
ARHGAP28	*TRIB1*$^{+}$ Satellite cells	0.441	6.09	0.0389	ENSSSCG00000021612	protein coding
NPM1	*TRIB1*$^{+}$ Satellite cells	0.441	9.38	0.0463	ENSSSCG00000016998	protein coding
RBM18	*TRIB1*$^{+}$ Satellite cells	0.443	5.37	0.0383	ENSSSCG00000030089	protein coding
ELAVL1	*TRIB1*$^{+}$ Satellite cells	0.445	6.77	0.0258	ENSSSCG00000013592	protein coding
BCL10	*TRIB1*$^{+}$ Satellite cells	0.446	6.09	0.0316	ENSSSCG00000025691	protein coding
ENSSSCG00000008183	*TRIB1*$^{+}$ Satellite cells	0.446	6.19	0.0405	ENSSSCG00000008183	protein coding
SLC2A13	*TRIB1*$^{+}$ Satellite cells	0.447	5.6	0.0405	ENSSSCG00000000783	protein coding
TMEM30A	*TRIB1*$^{+}$ Satellite cells	0.447	6.69	0.0269	ENSSSCG00000033155	protein coding
TSHZ1	*TRIB1*$^{+}$ Satellite cells	0.448	5.59	0.0265	ENSSSCG00000004861	protein coding

ECM2	*TRIB1*$^{+}$ Satellite cells	0.449	6.62	0.0464	ENSSSCG00000028549	protein coding
PRPF38A	*TRIB1*$^{+}$ Satellite cells	0.449	6.16	0.0259	ENSSSCG00000028288	protein coding
REEP5	*TRIB1*$^{+}$ Satellite cells	0.45	8.06	0.0396	ENSSSCG00000022048	protein coding
COL19A1	*TRIB1*$^{+}$ Satellite cells	0.451	6.57	0.0483	ENSSSCG00000044472	protein coding
TMEM106B	*TRIB1*$^{+}$ Satellite cells	0.451	5.8	0.0428	ENSSSCG00000032740	protein coding
MSTRG.48763	*TRIB1*$^{+}$ Satellite cells	0.452	5.02	0.0405	MSTRG.48763	lncRNA
PNRC2	*TRIB1*$^{+}$ Satellite cells	0.453	7.47	0.026	ENSSSCG00000023326	protein coding
SERINC3	*TRIB1*$^{+}$ Satellite cells	0.453	6.97	0.0256	ENSSSCG00000039591	protein coding
FBXL5	*TRIB1*$^{+}$ Satellite cells	0.454	6.41	0.0498	ENSSSCG00000008740	protein coding
FEM1C	*TRIB1*$^{+}$ Satellite cells	0.458	5.58	0.0264	ENSSSCG00000014217	protein coding
PAIP2	*TRIB1*$^{+}$ Satellite cells	0.459	8.32	0.0276	ENSSSCG00000026606	protein coding
PFN2	*TRIB1*$^{+}$ Satellite cells	0.459	6.37	0.0292	ENSSSCG00000030260	protein coding
STRADB	*TRIB1*$^{+}$ Satellite cells	0.459	5.86	0.0265	ENSSSCG00000031271	protein coding
AP2S1	*TRIB1*$^{+}$ Satellite cells	0.46	6.3	0.0422	ENSSSCG00000003106	protein coding
ASCC3	*TRIB1*$^{+}$ Satellite cells	0.46	6.21	0.0385	ENSSSCG00000004358	protein coding
CDC42	*TRIB1*$^{+}$ Satellite cells	0.46	8.29	0.0202	ENSSSCG00000003520	protein coding
STX11	*TRIB1*$^{+}$ Satellite cells	0.46	7.76	0.0204	ENSSSCG00000004125	protein coding
CHIC2	*TRIB1*$^{+}$ Satellite cells	0.461	7.63	0.0325	ENSSSCG00000032645	protein coding
ENSSSCG00000024019	*TRIB1*$^{+}$ Satellite cells	0.461	6.41	0.0308	ENSSSCG00000024019	protein coding
TMEM50A	*TRIB1*$^{+}$ Satellite cells	0.462	7.28	0.0159	ENSSSCG00000024267	protein coding
CCT4	*TRIB1*$^{+}$ Satellite cells	0.463	7.39	0.0312	ENSSSCG00000008377	protein coding
ENSSSCG00000012427	*TRIB1*$^{+}$ Satellite cells	0.465	12.1	0.025	ENSSSCG00000012427	protein coding
BTG3	*TRIB1*$^{+}$ Satellite cells	0.466	7.35	0.0338	ENSSSCG00000012014	protein coding
EMP1	*TRIB1*$^{+}$ Satellite cells	0.466	9.99	0.0432	ENSSSCG00000036723	protein coding
MSTRG.40943	*TRIB1*$^{+}$ Satellite cells	0.466	5.19	0.0366	MSTRG.40943	lncRNA
HAT1	*TRIB1*$^{+}$ Satellite cells	0.467	5.08	0.0389	ENSSSCG00000015952	protein coding
CORO1B	*TRIB1*$^{+}$ Satellite cells	0.468	5.74	0.0332	ENSSSCG00000012920	protein coding
NIPSNAP3A	*TRIB1*$^{+}$ Satellite cells	0.468	6.43	0.0455	ENSSSCG00000005424	protein coding
YIPF4	*TRIB1*$^{+}$ Satellite cells	0.468	6.16	0.029	ENSSSCG00000035747	protein coding
MYNN	*TRIB1*$^{+}$ Satellite cells	0.469	5.4	0.0293	ENSSSCG00000026829	protein coding
RPL38	*TRIB1*$^{+}$ Satellite cells	0.469	11.1	0.0263	ENSSSCG00000017239	protein coding
ATXN1	*TRIB1*$^{+}$ Satellite cells	0.47	7.35	0.0264	ENSSSCG00000001065	protein coding
GPBP1	*TRIB1*$^{+}$ Satellite cells	0.471	7.13	0.025	ENSSSCG00000016922	protein coding
METAP2	*TRIB1*$^{+}$ Satellite cells	0.472	6.59	0.0207	ENSSSCG00000030608	protein coding

IAH1	*TRIB1*$^{+}$ Satellite cells	0.473	5.6	0.0248	ENSSSCG00000008640	protein coding
CLPX	*TRIB1*$^{+}$ Satellite cells	0.474	5.77	0.0399	ENSSSCG00000004927	protein coding
HIGD2A	*TRIB1*$^{+}$ Satellite cells	0.474	6.99	0.0197	ENSSSCG00000029179	protein coding
TMEM123	*TRIB1*$^{+}$ Satellite cells	0.476	6.77	0.034	ENSSSCG00000031170	protein coding
UCHL5	*TRIB1*$^{+}$ Satellite cells	0.478	5.85	0.0177	ENSSSCG00000010802	protein coding
ENSSSCG00000028423	*TRIB1*$^{+}$ Satellite cells	0.479	6.7	0.0171	ENSSSCG00000028423	protein coding
TMEM50B	*TRIB1*$^{+}$ Satellite cells	0.479	5.79	0.0331	ENSSSCG00000034624	protein coding
TNIK	*TRIB1*$^{+}$ Satellite cells	0.479	7.63	0.0393	ENSSSCG00000028905	protein coding
UBALD1	*TRIB1*$^{+}$ Satellite cells	0.479	6.77	0.0456	ENSSSCG00000023603	protein coding
ENSSSCG00000017971	*TRIB1*$^{+}$ Satellite cells	0.48	5.71	0.028	ENSSSCG00000017971	protein coding
PHC3	*TRIB1*$^{+}$ Satellite cells	0.48	4.96	0.0448	ENSSSCG00000030467	protein coding
MSTRG.11643	*TRIB1*$^{+}$ Satellite cells	0.481	4.96	0.0406	MSTRG.11643	lncRNA
TM2D1	*TRIB1*$^{+}$ Satellite cells	0.481	6.67	0.0186	ENSSSCG00000038861	protein coding
JAM3	*TRIB1*$^{+}$ Satellite cells	0.483	6.39	0.0208	ENSSSCG00000015256	protein coding
ATP6AP2	*TRIB1*$^{+}$ Satellite cells	0.484	6.49	0.0261	ENSSSCG00000012247	protein coding
ENSSSCG00000031838	*TRIB1*$^{+}$ Satellite cells	0.485	12.6	0.0167	ENSSSCG00000031838	protein coding
HSD11B1L	*TRIB1*$^{+}$ Satellite cells	0.485	11.8	0.02	ENSSSCG00000030546	protein coding
RAB6A	*TRIB1*$^{+}$ Satellite cells	0.487	6.29	0.0272	ENSSSCG00000014828	protein coding
RNF138	*TRIB1*$^{+}$ Satellite cells	0.487	6.09	0.0361	ENSSSCG00000003730	protein coding
STT3B	*TRIB1*$^{+}$ Satellite cells	0.487	6.7	0.0283	ENSSSCG00000011229	protein coding
CAMLG	*TRIB1*$^{+}$ Satellite cells	0.488	5.2	0.0255	ENSSSCG00000038955	protein coding
MSTRG.39837	*TRIB1*$^{+}$ Satellite cells	0.488	5.19	0.0494	MSTRG.39837	lncRNA
PROM1	*TRIB1*$^{+}$ Satellite cells	0.489	5.54	0.0338	ENSSSCG00000008745	protein coding
KIRREL1	*TRIB1*$^{+}$ Satellite cells	0.491	6.09	0.0302	ENSSSCG00000006453	protein coding
OSBPL9	*TRIB1*$^{+}$ Satellite cells	0.491	6.69	0.0427	ENSSSCG00000021292	protein coding
COQ10B	*TRIB1*$^{+}$ Satellite cells	0.493	7.57	0.0251	ENSSSCG00000016076	protein coding
RIOK3	*TRIB1*$^{+}$ Satellite cells	0.493	6.77	0.0448	ENSSSCG00000029518	protein coding
CMC1	*TRIB1*$^{+}$ Satellite cells	0.494	5.47	0.0462	ENSSSCG00000041813	protein coding
PBDC1	*TRIB1*$^{+}$ Satellite cells	0.494	5.83	0.0332	ENSSSCG00000030577	protein coding
SUMF1	*TRIB1*$^{+}$ Satellite cells	0.494	6.46	0.0289	ENSSSCG00000011532	protein coding
ATP6V1G1	*TRIB1*$^{+}$ Satellite cells	0.495	7.63	0.0215	ENSSSCG00000032835	protein coding
ENSSSCG00000004441	*TRIB1*$^{+}$ Satellite cells	0.495	4.64	0.0305	ENSSSCG00000004441	protein coding
ABCE1	*TRIB1*$^{+}$ Satellite cells	0.497	6.6	0.0127	ENSSSCG00000009043	protein coding
ENSSSCG00000039544	*TRIB1*$^{+}$ Satellite cells	0.497	11.9	0.0181	ENSSSCG00000039544	protein coding

PCMTD1	*TRIB1*$^+$ Satellite cells	0.497	6.05	0.0464	ENSSSCG00000006267	protein coding
TMEM41B	*TRIB1*$^+$ Satellite cells	0.497	5.63	0.0298	ENSSSCG00000014579	protein coding
CAPRIN1	*TRIB1*$^+$ Satellite cells	0.498	6.35	0.0129	ENSSSCG00000013305	protein coding
EXT1	*TRIB1*$^+$ Satellite cells	0.499	7.45	0.0387	ENSSSCG00000006009	protein coding
TXNDC17	*TRIB1*$^+$ Satellite cells	0.499	6.54	0.0397	ENSSSCG00000035820	protein coding
CETN3	*TRIB1*$^+$ Satellite cells	0.5	6.3	0.0263	ENSSSCG00000014151	protein coding
GNAI3	*TRIB1*$^+$ Satellite cells	0.5	5.99	0.0457	ENSSSCG00000006824	protein coding
HNRNPA0	*TRIB1*$^+$ Satellite cells	0.501	8.16	0.0223	ENSSSCG00000042630	protein coding
CNOT7	*TRIB1*$^+$ Satellite cells	0.503	5.47	0.0198	ENSSSCG00000006983	protein coding
ENSSSCG00000022331	*TRIB1*$^+$ Satellite cells	0.503	6.02	0.0346	ENSSSCG00000022331	protein coding
ENSSSCG00000040701	*TRIB1*$^+$ Satellite cells	0.503	5.43	0.0481	ENSSSCG00000040701	protein coding
RASAL2	*TRIB1*$^+$ Satellite cells	0.503	5.38	0.0326	ENSSSCG00000015519	protein coding
ZNF622	*TRIB1*$^+$ Satellite cells	0.503	5.22	0.0327	ENSSSCG00000016793	protein coding
CLDND1	*TRIB1*$^+$ Satellite cells	0.504	6.07	0.0176	ENSSSCG00000011980	protein coding
PLEKHA5	*TRIB1*$^+$ Satellite cells	0.504	7.03	0.0216	ENSSSCG00000000588	protein coding
RAD51B	*TRIB1*$^+$ Satellite cells	0.505	6.1	0.0437	ENSSSCG00000028877	protein coding
SPAG8	*TRIB1*$^+$ Satellite cells	0.505	6.39	0.0172	ENSSSCG00000025842	protein coding
ENSSSCG00000029761	*TRIB1*$^+$ Satellite cells	0.506	5.74	0.0483	ENSSSCG00000029761	protein coding
MCM7	*TRIB1*$^+$ Satellite cells	0.509	5.68	0.0186	ENSSSCG00000032180	protein coding
ENSSSCG00000001850	*TRIB1*$^+$ Satellite cells	0.51	4.71	0.0468	ENSSSCG00000001850	protein coding
ENSSSCG00000027905	*TRIB1*$^+$ Satellite cells	0.51	4.71	0.0484	ENSSSCG00000027905	protein coding
MAPK6	*TRIB1*$^+$ Satellite cells	0.51	5.73	0.0424	ENSSSCG00000004624	protein coding
SLC23A2	*TRIB1*$^+$ Satellite cells	0.51	5.75	0.0199	ENSSSCG00000026466	protein coding
DMD	*TRIB1*$^+$ Satellite cells	0.511	8.72	0.0208	ENSSSCG00000028148	protein coding
DCBLD1	*TRIB1*$^+$ Satellite cells	0.512	4.41	0.0355	ENSSSCG00000004254	protein coding
ORC4	*TRIB1*$^+$ Satellite cells	0.512	6.02	0.0393	ENSSSCG00000015668	protein coding
PPP2CA	*TRIB1*$^+$ Satellite cells	0.512	7.49	0.014	ENSSSCG00000014298	protein coding
RPL7	*TRIB1*$^+$ Satellite cells	0.514	12.1	0.0106	ENSSSCG00000032111	protein coding
CCT8	*TRIB1*$^+$ Satellite cells	0.516	7.13	0.015	ENSSSCG00000026761	protein coding
ARF4	*TRIB1*$^+$ Satellite cells	0.518	8.51	0.0132	ENSSSCG00000031791	protein coding
EML4	*TRIB1*$^+$ Satellite cells	0.518	6.59	0.0224	ENSSSCG00000008467	protein coding
LRRFIP2	*TRIB1*$^+$ Satellite cells	0.519	5.49	0.0409	ENSSSCG00000027738	protein coding
RPS28	*TRIB1*$^+$ Satellite cells	0.52	12.2	0.0146	ENSSSCG00000013597	protein coding
MSTRG.1412	*TRIB1*$^+$ Satellite cells	0.521	4.21	0.0421	MSTRG.1412	lncRNA

CD164	*TRIB1* $^{+}$ Satellite cells	0.523	7.6	0.00943	ENSSSCG00000004414	protein coding
SCAMP1	*TRIB1* $^{+}$ Satellite cells	0.523	6.04	0.0283	ENSSSCG00000034795	protein coding
NAP1L1	*TRIB1* $^{+}$ Satellite cells	0.525	7.92	0.0192	ENSSSCG00000000522	protein coding
NFU1	*TRIB1* $^{+}$ Satellite cells	0.526	5.24	0.0248	ENSSSCG00000025007	protein coding
ENSSSCG00000011196	*TRIB1* $^{+}$ Satellite cells	0.529	5.53	0.0321	ENSSSCG00000011196	lncRNA
RPP30	*TRIB1* $^{+}$ Satellite cells	0.53	4.98	0.0219	ENSSSCG00000010460	protein coding
VAPA	*TRIB1* $^{+}$ Satellite cells	0.53	7.16	0.04	ENSSSCG00000038465	protein coding
POLB	*TRIB1* $^{+}$ Satellite cells	0.531	4.96	0.0277	ENSSSCG00000007031	protein coding
RPS15A	*TRIB1* $^{+}$ Satellite cells	0.531	12.1	0.0137	ENSSSCG00000035768	protein coding
CDIPT	*TRIB1* $^{+}$ Satellite cells	0.533	5.26	0.0169	ENSSSCG00000027629	protein coding
RPL15	*TRIB1* $^{+}$ Satellite cells	0.533	11.1	0.0203	ENSSSCG00000037030	protein coding
CYB5A	*TRIB1* $^{+}$ Satellite cells	0.535	6.8	0.0119	ENSSSCG00000004875	protein coding
MRPL39	*TRIB1* $^{+}$ Satellite cells	0.535	5.73	0.0115	ENSSSCG00000012021	protein coding
ENSSSCG00000020725	*TRIB1* $^{+}$ Satellite cells	0.536	12	0.0127	ENSSSCG00000020725	protein coding
HSPA8	*TRIB1* $^{+}$ Satellite cells	0.536	9.83	0.0285	ENSSSCG00000015140	protein coding
PPP3R1	*TRIB1* $^{+}$ Satellite cells	0.536	4.83	0.0435	ENSSSCG00000036115	protein coding
SLC35F5	*TRIB1* $^{+}$ Satellite cells	0.536	5.28	0.0254	ENSSSCG00000015709	protein coding
UBE2D3	*TRIB1* $^{+}$ Satellite cells	0.536	8.25	0.00883	ENSSSCG00000035622	protein coding
SRP19	*TRIB1* $^{+}$ Satellite cells	0.537	7.41	0.00774	ENSSSCG00000026230	protein coding
RPA3	*TRIB1* $^{+}$ Satellite cells	0.538	6.27	0.0159	ENSSSCG00000023886	protein coding
SMNDC1	*TRIB1* $^{+}$ Satellite cells	0.539	5.92	0.0153	ENSSSCG00000030507	protein coding
ENSSSCG00000011764	*TRIB1* $^{+}$ Satellite cells	0.54	7.13	0.00994	ENSSSCG00000011764	protein coding
FNBP1L	*TRIB1* $^{+}$ Satellite cells	0.541	6.01	0.0271	ENSSSCG00000006894	protein coding
ACTR10	*TRIB1* $^{+}$ Satellite cells	0.542	6.41	0.0198	ENSSSCG00000022299	protein coding
RUNX1	*TRIB1* $^{+}$ Satellite cells	0.542	10.1	0.0168	ENSSSCG00000035537	protein coding
TMEM159	*TRIB1* $^{+}$ Satellite cells	0.542	5.27	0.0288	ENSSSCG00000031876	protein coding
ENSSSCG00000033744	*TRIB1* $^{+}$ Satellite cells	0.543	5.99	0.0456	ENSSSCG00000033744	protein coding
DOCK7	*TRIB1* $^{+}$ Satellite cells	0.544	5.12	0.0227	ENSSSCG00000003818	protein coding
ENSSSCG00000007088	*TRIB1* $^{+}$ Satellite cells	0.545	6.35	0.00738	ENSSSCG00000007088	protein coding
POLR2M	*TRIB1* $^{+}$ Satellite cells	0.546	5.14	0.0151	ENSSSCG00000030605	protein coding
GPX4	*TRIB1* $^{+}$ Satellite cells	0.549	6.97	0.0143	ENSSSCG00000024052	protein coding
MSTRG.14549	*TRIB1* $^{+}$ Satellite cells	0.549	5.46	0.0438	MSTRG.14549	lncRNA
SRP9	*TRIB1* $^{+}$ Satellite cells	0.556	4.84	0.0247	ENSSSCG00000031664	protein coding
ZFAND1	*TRIB1* $^{+}$ Satellite cells	0.556	4.83	0.0204	ENSSSCG00000024780	protein coding

GRIA4	*TRIB1*$^{+}$ Satellite cells	0.56	6.05	0.0288	ENSSSCG00000030565	protein coding
RAB11A	*TRIB1*$^{+}$ Satellite cells	0.56	5.82	0.0132	ENSSSCG00000032312	protein coding
RPS3A	*TRIB1*$^{+}$ Satellite cells	0.56	12.1	0.00861	ENSSSCG00000009019	protein coding
GPSM2	*TRIB1*$^{+}$ Satellite cells	0.561	5.47	0.0323	ENSSSCG00000021440	protein coding
MSTRG.6594	*TRIB1*$^{+}$ Satellite cells	0.561	7.06	0.0158	MSTRG.6594	lncRNA
NIPA2	*TRIB1*$^{+}$ Satellite cells	0.561	4.87	0.0231	ENSSSCG00000036898	protein coding
ST13	*TRIB1*$^{+}$ Satellite cells	0.561	8.19	0.0142	ENSSSCG00000000071	protein coding
CNOT2	*TRIB1*$^{+}$ Satellite cells	0.565	6.89	0.0157	ENSSSCG00000000502	protein coding
MSTRG.22894	*TRIB1*$^{+}$ Satellite cells	0.566	5	0.0236	MSTRG.22894	lncRNA
PGGT1B	*TRIB1*$^{+}$ Satellite cells	0.566	5.51	0.0245	ENSSSCG00000014215	protein coding
BTF3	*TRIB1*$^{+}$ Satellite cells	0.567	10.1	0.0285	ENSSSCG00000014071	protein coding
ENSSSCG00000033871	*TRIB1*$^{+}$ Satellite cells	0.567	4.36	0.0243	ENSSSCG00000033871	protein coding
CCN1	*TRIB1*$^{+}$ Satellite cells	0.568	8.76	0.039	ENSSSCG00000006940	protein coding
CRPPA	*TRIB1*$^{+}$ Satellite cells	0.57	4.98	0.0423	ENSSSCG00000015358	protein coding
MSTRG.1890	*TRIB1*$^{+}$ Satellite cells	0.57	5.79	0.0477	MSTRG.1890	lncRNA
PAICS	*TRIB1*$^{+}$ Satellite cells	0.57	4.77	0.0478	ENSSSCG00000008901	protein coding
RTRAF	*TRIB1*$^{+}$ Satellite cells	0.571	8.08	0.017	ENSSSCG00000005029	protein coding
SHISA4	*TRIB1*$^{+}$ Satellite cells	0.573	3.86	0.0472	ENSSSCG00000031981	protein coding
SLC4A4	*TRIB1*$^{+}$ Satellite cells	0.574	4.29	0.0483	ENSSSCG00000008943	protein coding
SNAP23	*TRIB1*$^{+}$ Satellite cells	0.574	5.47	0.0158	ENSSSCG00000038735	protein coding
SYPL1	*TRIB1*$^{+}$ Satellite cells	0.575	6.91	0.00628	ENSSSCG00000015434	protein coding
OPA1	*TRIB1*$^{+}$ Satellite cells	0.578	4.45	0.029	ENSSSCG00000011822	protein coding
PRKCSH	*TRIB1*$^{+}$ Satellite cells	0.578	6.58	0.0123	ENSSSCG00000013627	protein coding
SUMO1	*TRIB1*$^{+}$ Satellite cells	0.578	7	0.00684	ENSSSCG00000035842	protein coding
CACUL1	*TRIB1*$^{+}$ Satellite cells	0.579	4.51	0.0362	ENSSSCG00000038222	protein coding
MARCKS	*TRIB1*$^{+}$ Satellite cells	0.579	7.59	0.0494	ENSSSCG00000037803	protein coding
PPT1	*TRIB1*$^{+}$ Satellite cells	0.58	4.95	0.0276	ENSSSCG00000036673	protein coding
PSMD10	*TRIB1*$^{+}$ Satellite cells	0.58	3.75	0.0439	ENSSSCG00000033180	protein coding
MSTRG.31302	*TRIB1*$^{+}$ Satellite cells	0.582	4.44	0.0478	MSTRG.31302	lncRNA
CCDC50	*TRIB1*$^{+}$ Satellite cells	0.583	4.71	0.0314	ENSSSCG00000036423	protein coding
DEPDC1B	*TRIB1*$^{+}$ Satellite cells	0.583	5.67	0.0134	ENSSSCG00000035012	protein coding
ERGIC2	*TRIB1*$^{+}$ Satellite cells	0.584	5.68	0.00959	ENSSSCG00000000536	protein coding
C19orf53	*TRIB1*$^{+}$ Satellite cells	0.585	6.17	0.009	ENSSSCG00000013757	protein coding
EYA1	*TRIB1*$^{+}$ Satellite cells	0.585	7.32	0.00683	ENSSSCG00000040728	protein coding

CRLS1	*TRIB1*$^{+}$ Satellite cells	0.586	4.67	0.0204	ENSSSCG00000040202	protein coding
PON2	*TRIB1*$^{+}$ Satellite cells	0.586	6.28	0.00942	ENSSSCG00000015331	protein coding
SAV1	*TRIB1*$^{+}$ Satellite cells	0.586	4.95	0.038	ENSSSCG00000005021	protein coding
MSTRG.30002	*TRIB1*$^{+}$ Satellite cells	0.587	9.06	0.00878	MSTRG.30002	lncRNA
STMN1	*TRIB1*$^{+}$ Satellite cells	0.587	7.31	0.0412	ENSSSCG00000026257	protein coding
TMEM168	*TRIB1*$^{+}$ Satellite cells	0.587	4.28	0.0402	ENSSSCG00000016643	protein coding
ENSSSCG00000000359	*TRIB1*$^{+}$ Satellite cells	0.588	7.75	0.0161	ENSSSCG00000000359	protein coding
ENSSSCG00000004307	*TRIB1*$^{+}$ Satellite cells	0.588	6.83	0.00835	ENSSSCG00000004307	protein coding
ENSSSCG00000002446	*TRIB1*$^{+}$ Satellite cells	0.589	4.55	0.024	ENSSSCG00000002446	protein coding
CAPZA2	*TRIB1*$^{+}$ Satellite cells	0.59	7.93	0.00547	ENSSSCG00000028113	protein coding
CZIB	*TRIB1*$^{+}$ Satellite cells	0.59	5.41	0.0255	ENSSSCG00000003850	protein coding
POP7	*TRIB1*$^{+}$ Satellite cells	0.59	5.86	0.0462	ENSSSCG00000007672	protein coding
HMGN2	*TRIB1*$^{+}$ Satellite cells	0.591	4.04	0.0443	ENSSSCG00000003555	protein coding
GSK3B	*TRIB1*$^{+}$ Satellite cells	0.592	8.48	0.00717	ENSSSCG00000011889	protein coding
PDCD4	*TRIB1*$^{+}$ Satellite cells	0.595	7.28	0.0427	ENSSSCG00000010627	protein coding
ARID5A	*TRIB1*$^{+}$ Satellite cells	0.596	5.43	0.0233	ENSSSCG00000008123	protein coding
MSTRG.36818	*TRIB1*$^{+}$ Satellite cells	0.596	5.6	0.00916	MSTRG.36818	lncRNA
H2AFZ	*TRIB1*$^{+}$ Satellite cells	0.597	8.03	0.0232	ENSSSCG00000009178	protein coding
RBM38	*TRIB1*$^{+}$ Satellite cells	0.598	5.05	0.0425	ENSSSCG00000007504	protein coding
MAML2	*TRIB1*$^{+}$ Satellite cells	0.601	7.22	0.0493	ENSSSCG00000034879	protein coding
ENSSSCG00000033733	*TRIB1*$^{+}$ Satellite cells	0.607	7.85	0.0124	ENSSSCG00000033733	protein coding
PKIA	*TRIB1*$^{+}$ Satellite cells	0.608	7.53	0.00924	ENSSSCG00000006163	protein coding
MTPAP	*TRIB1*$^{+}$ Satellite cells	0.609	5.22	0.0172	ENSSSCG00000011018	protein coding
SEC22B	*TRIB1*$^{+}$ Satellite cells	0.61	5.56	0.0109	ENSSSCG00000020964	protein coding
LEPROTL1	*TRIB1*$^{+}$ Satellite cells	0.613	4.65	0.0287	ENSSSCG00000038085	protein coding
OGN	*TRIB1*$^{+}$ Satellite cells	0.614	8.39	0.0245	ENSSSCG00000034607	protein coding
WSB1	*TRIB1*$^{+}$ Satellite cells	0.615	7.54	0.0294	ENSSSCG00000017752	protein coding
HSDL2	*TRIB1*$^{+}$ Satellite cells	0.616	5.46	0.0494	ENSSSCG00000005467	protein coding
POPDC3	*TRIB1*$^{+}$ Satellite cells	0.616	6.85	0.00938	ENSSSCG00000004367	protein coding
RSRP1	*TRIB1*$^{+}$ Satellite cells	0.617	5.14	0.0359	ENSSSCG00000034449	protein coding
STOM	*TRIB1*$^{+}$ Satellite cells	0.617	4.97	0.0206	ENSSSCG00000036639	protein coding
YPEL3	*TRIB1*$^{+}$ Satellite cells	0.617	6.96	0.0127	ENSSSCG00000035430	protein coding
ENSSSCG00000000097	*TRIB1*$^{+}$ Satellite cells	0.618	6.34	0.0128	ENSSSCG00000000097	protein coding
SPINDOC	*TRIB1*$^{+}$ Satellite cells	0.618	4.44	0.0216	ENSSSCG00000013048	protein coding

PDK1	*TRIB1*$^{+}$ Satellite cells	0.619	4.05	0.0371	ENSSSCG00000015958	protein coding
HSPA13	*TRIB1*$^{+}$ Satellite cells	0.62	5.5	0.00629	ENSSSCG00000012007	protein coding
MSTRG.37673	*TRIB1*$^{+}$ Satellite cells	0.621	4.13	0.0281	MSTRG.37673	lncRNA
GPX1	*TRIB1*$^{+}$ Satellite cells	0.622	8.36	0.0473	ENSSSCG00000033727	protein coding
PTP4A1	*TRIB1*$^{+}$ Satellite cells	0.623	5.87	0.00584	ENSSSCG00000025941	protein coding
SELENOH	*TRIB1*$^{+}$ Satellite cells	0.625	6.04	0.00569	ENSSSCG00000032364	protein coding
UBE2E2	*TRIB1*$^{+}$ Satellite cells	0.629	6.25	0.0151	ENSSSCG00000031392	protein coding
ZNF704	*TRIB1*$^{+}$ Satellite cells	0.629	6.41	0.0244	ENSSSCG00000022989	protein coding
HDAC2	*TRIB1*$^{+}$ Satellite cells	0.63	6.76	0.00546	ENSSSCG00000004432	protein coding
ERCC8	*TRIB1*$^{+}$ Satellite cells	0.631	4.23	0.0306	ENSSSCG00000016934	protein coding
MSTRG.17788	*TRIB1*$^{+}$ Satellite cells	0.631	5.53	0.0218	MSTRG.17788	lncRNA
MSTRG.25390	*TRIB1*$^{+}$ Satellite cells	0.631	4.7	0.0324	MSTRG.25390	lncRNA
ARPC1B	*TRIB1*$^{+}$ Satellite cells	0.632	5.1	0.0282	ENSSSCG00000007625	protein coding
IFNGR1	*TRIB1*$^{+}$ Satellite cells	0.633	5.41	0.00835	ENSSSCG00000004156	protein coding
ATP11C	*TRIB1*$^{+}$ Satellite cells	0.634	5.76	0.0293	ENSSSCG00000012713	protein coding
DNMBP	*TRIB1*$^{+}$ Satellite cells	0.634	5.79	0.014	ENSSSCG00000010544	protein coding
KDM1B	*TRIB1*$^{+}$ Satellite cells	0.634	4.24	0.0211	ENSSSCG00000001074	protein coding
ARMC10	*TRIB1*$^{+}$ Satellite cells	0.638	5.78	0.0066	ENSSSCG00000028444	protein coding
ORMDL1	*TRIB1*$^{+}$ Satellite cells	0.639	5.27	0.0379	ENSSSCG00000016044	protein coding
SLC36A4	*TRIB1*$^{+}$ Satellite cells	0.64	4.39	0.0225	ENSSSCG00000024933	protein coding
MSTRG.15963	*TRIB1*$^{+}$ Satellite cells	0.642	7.28	0.0204	MSTRG.15963	lncRNA
RWDD4	*TRIB1*$^{+}$ Satellite cells	0.642	4.6	0.0116	ENSSSCG00000015776	protein coding
LGALS1	*TRIB1*$^{+}$ Satellite cells	0.643	11.4	0.0453	ENSSSCG00000033854	protein coding
TRAP1	*TRIB1*$^{+}$ Satellite cells	0.643	5.76	0.0116	ENSSSCG00000007952	protein coding
IPO5	*TRIB1*$^{+}$ Satellite cells	0.644	6.86	0.0146	ENSSSCG00000009509	protein coding
TRIM13	*TRIB1*$^{+}$ Satellite cells	0.644	4.05	0.038	ENSSSCG00000009389	protein coding
COPS2	*TRIB1*$^{+}$ Satellite cells	0.646	6.73	0.0105	ENSSSCG00000004652	protein coding
MSN	*TRIB1*$^{+}$ Satellite cells	0.646	4.85	0.0109	ENSSSCG00000012365	protein coding
OGFOD3	*TRIB1*$^{+}$ Satellite cells	0.647	5.09	0.0398	ENSSSCG00000032116	protein coding
ENSSSCG00000032768	*TRIB1*$^{+}$ Satellite cells	0.649	4.43	0.0318	ENSSSCG00000032768	protein coding
CDKN1A	*TRIB1*$^{+}$ Satellite cells	0.652	6.68	0.0152	ENSSSCG00000001565	protein coding
MSTRG.46283	*TRIB1*$^{+}$ Satellite cells	0.652	4.58	0.0447	MSTRG.46283	lncRNA
THUMPD2	*TRIB1*$^{+}$ Satellite cells	0.652	4.71	0.0203	ENSSSCG00000008470	protein coding
IFT22	*TRIB1*$^{+}$ Satellite cells	0.653	3.82	0.036	ENSSSCG00000046214	protein coding

TPM4	*TRIB1*$^{+}$ Satellite cells	0.654	8.7	0.0234	ENSSSCG00000013849	protein coding
COL1A2	*TRIB1*$^{+}$ Satellite cells	0.655	10.2	0.0226	ENSSSCG00000015326	protein coding
CTNNBIP1	*TRIB1*$^{+}$ Satellite cells	0.655	3.66	0.0475	ENSSSCG00000036868	protein coding
B9D2	*TRIB1*$^{+}$ Satellite cells	0.657	4.42	0.0272	ENSSSCG00000003013	protein coding
CASP3	*TRIB1*$^{+}$ Satellite cells	0.657	4.59	0.0377	ENSSSCG00000034012	protein coding
GNG10	*TRIB1*$^{+}$ Satellite cells	0.658	8.21	0.00506	ENSSSCG00000046439	protein coding
PMP22	*TRIB1*$^{+}$ Satellite cells	0.658	7.98	0.00481	ENSSSCG00000037832	protein coding
TUFT1	*TRIB1*$^{+}$ Satellite cells	0.658	5.1	0.0384	ENSSSCG00000006620	protein coding
CCDC142	*TRIB1*$^{+}$ Satellite cells	0.659	6.22	0.0464	ENSSSCG00000008270	protein coding
FARP1	*TRIB1*$^{+}$ Satellite cells	0.659	6.22	0.0254	ENSSSCG00000026863	protein coding
MSTRG.27683	*TRIB1*$^{+}$ Satellite cells	0.659	4.48	0.038	MSTRG.27683	lncRNA
ENSSSCG00000001927	*TRIB1*$^{+}$ Satellite cells	0.66	5.44	0.0119	ENSSSCG00000001927	protein coding
FLAD1	*TRIB1*$^{+}$ Satellite cells	0.66	5.73	0.00886	ENSSSCG00000006538	protein coding
COX7A2L	*TRIB1*$^{+}$ Satellite cells	0.661	8.16	0.00408	ENSSSCG00000008466	protein coding
NONO	*TRIB1*$^{+}$ Satellite cells	0.662	6.48	0.0436	ENSSSCG00000023245	protein coding
UBA6	*TRIB1*$^{+}$ Satellite cells	0.662	4.31	0.0204	ENSSSCG00000008924	protein coding
ACTA2	*TRIB1*$^{+}$ Satellite cells	0.664	8.18	0.0193	ENSSSCG00000010447	protein coding
ENSSSCG00000039473	*TRIB1*$^{+}$ Satellite cells	0.664	6.37	0.0386	ENSSSCG00000039473	protein coding
SERTAD2	*TRIB1*$^{+}$ Satellite cells	0.664	5.11	0.018	ENSSSCG00000037002	protein coding
CCND3	*TRIB1*$^{+}$ Satellite cells	0.666	6.53	0.0321	ENSSSCG00000032284	protein coding
ABI3BP	*TRIB1*$^{+}$ Satellite cells	0.668	5.44	0.0439	ENSSSCG00000011959	protein coding
DUSP28	*TRIB1*$^{+}$ Satellite cells	0.668	4.2	0.0264	ENSSSCG00000028180	protein coding
STK10	*TRIB1*$^{+}$ Satellite cells	0.668	4.74	0.0265	ENSSSCG00000022478	protein coding
IGF2BP3	*TRIB1*$^{+}$ Satellite cells	0.669	6.51	0.00561	ENSSSCG00000036695	protein coding
MSTRG.35782	*TRIB1*$^{+}$ Satellite cells	0.67	5	0.0338	MSTRG.35782	lncRNA
POC1B	*TRIB1*$^{+}$ Satellite cells	0.67	5.51	0.012	ENSSSCG00000000920	protein coding
TPD52	*TRIB1*$^{+}$ Satellite cells	0.67	6.69	0.00805	ENSSSCG00000006156	protein coding
PDK2	*TRIB1*$^{+}$ Satellite cells	0.673	5.01	0.0234	ENSSSCG00000027198	protein coding
KARS1	*TRIB1*$^{+}$ Satellite cells	0.674	8.74	0.0201	ENSSSCG00000002704	protein coding
CYP39A1	*TRIB1*$^{+}$ Satellite cells	0.675	4.35	0.0445	ENSSSCG00000001718	protein coding
IFT46	*TRIB1*$^{+}$ Satellite cells	0.676	5.27	0.0258	ENSSSCG00000029649	protein coding
CMTM8	*TRIB1*$^{+}$ Satellite cells	0.677	5.06	0.0274	ENSSSCG00000022618	protein coding
SNRPB2	*TRIB1*$^{+}$ Satellite cells	0.678	6.52	0.00747	ENSSSCG00000037602	protein coding
HUS1	*TRIB1*$^{+}$ Satellite cells	0.68	3.8	0.0436	ENSSSCG00000028523	protein coding

PPP1CC	*TRIB1*$^{+}$ Satellite cells	0.68	7.61	0.0106	ENSSSCG00000009828	protein coding
BNIP3L	*TRIB1*$^{+}$ Satellite cells	0.681	5.73	0.00748	ENSSSCG00000009658	protein coding
NDFIP2	*TRIB1*$^{+}$ Satellite cells	0.681	5.51	0.0107	ENSSSCG00000009480	protein coding
NFKB1	*TRIB1*$^{+}$ Satellite cells	0.683	6.51	0.0161	ENSSSCG00000030957	protein coding
MICU1	*TRIB1*$^{+}$ Satellite cells	0.684	7.51	0.0438	ENSSSCG00000010289	protein coding
AP1S2	*TRIB1*$^{+}$ Satellite cells	0.688	5.86	0.0213	ENSSSCG00000036074	protein coding
ENSSSCG00000039731	*TRIB1*$^{+}$ Satellite cells	0.689	5.41	0.0319	ENSSSCG00000039731	protein coding
GALK2	*TRIB1*$^{+}$ Satellite cells	0.689	5.07	0.0432	ENSSSCG00000004651	protein coding
RFC4	*TRIB1*$^{+}$ Satellite cells	0.689	4.94	0.0113	ENSSSCG00000011804	protein coding
COX7C	*TRIB1*$^{+}$ Satellite cells	0.692	9.41	0.0191	ENSSSCG00000040325	protein coding
MSTRG.10211	*TRIB1*$^{+}$ Satellite cells	0.693	3.76	0.0324	MSTRG.10211	lncRNA
ENSSSCG00000029857	*TRIB1*$^{+}$ Satellite cells	0.694	7.27	0.0192	ENSSSCG00000029857	protein coding
MSTRG.1135	*TRIB1*$^{+}$ Satellite cells	0.694	7.27	0.0051	MSTRG.1135	lncRNA
DPP4	*TRIB1*$^{+}$ Satellite cells	0.695	6.12	0.0185	ENSSSCG00000015894	protein coding
RGCC	*TRIB1*$^{+}$ Satellite cells	0.695	6.98	0.0175	ENSSSCG00000009434	protein coding
ZDHHC16	*TRIB1*$^{+}$ Satellite cells	0.695	3.94	0.0328	ENSSSCG00000010519	protein coding
ENSSSCG00000009772	*TRIB1*$^{+}$ Satellite cells	0.696	8.07	0.00677	ENSSSCG00000009772	protein coding
EZH2	*TRIB1*$^{+}$ Satellite cells	0.697	7.25	0.025	ENSSSCG00000028840	protein coding
CACNA2D1	*TRIB1*$^{+}$ Satellite cells	0.698	6.59	0.00521	ENSSSCG00000015402	protein coding
TMX1	*TRIB1*$^{+}$ Satellite cells	0.698	5.68	0.00943	ENSSSCG00000038422	protein coding
FAM114A2	*TRIB1*$^{+}$ Satellite cells	0.701	4.24	0.0395	ENSSSCG00000017073	protein coding
HBS1L	*TRIB1*$^{+}$ Satellite cells	0.701	5.41	0.00279	ENSSSCG00000004169	protein coding
RPA2	*TRIB1*$^{+}$ Satellite cells	0.703	4.48	0.0167	ENSSSCG00000003583	protein coding
DDX31	*TRIB1*$^{+}$ Satellite cells	0.705	4.3	0.0193	ENSSSCG00000005729	protein coding
KRAS	*TRIB1*$^{+}$ Satellite cells	0.709	5.47	0.00281	ENSSSCG00000026969	protein coding
AIMP1	*TRIB1*$^{+}$ Satellite cells	0.71	7.43	0.00283	ENSSSCG00000024168	protein coding
S100A11	*TRIB1*$^{+}$ Satellite cells	0.71	9.01	0.00409	ENSSSCG00000006610	protein coding
GRSF1	*TRIB1*$^{+}$ Satellite cells	0.711	5.39	0.0237	ENSSSCG00000008940	protein coding
ENSSSCG00000039331	*TRIB1*$^{+}$ Satellite cells	0.714	5.4	0.00733	ENSSSCG00000039331	protein coding
ENSSSCG00000012178	*TRIB1*$^{+}$ Satellite cells	0.716	7.49	0.0256	ENSSSCG00000012178	protein coding
HACE1	*TRIB1*$^{+}$ Satellite cells	0.716	4.05	0.0204	ENSSSCG00000004364	protein coding
NFKBIE	*TRIB1*$^{+}$ Satellite cells	0.719	4.17	0.0181	ENSSSCG00000001703	protein coding
WDR48	*TRIB1*$^{+}$ Satellite cells	0.719	4.87	0.0121	ENSSSCG00000011261	protein coding
CCNG2	*TRIB1*$^{+}$ Satellite cells	0.721	5.96	0.03	ENSSSCG00000008988	protein coding

PROS1	*TRIB1* + Satellite cells	0.721	5.75	0.00948	ENSSSCG00000025463	protein coding
CHCHD5	*TRIB1* + Satellite cells	0.722	4.88	0.0177	ENSSSCG00000008094	protein coding
TPGS2	*TRIB1* + Satellite cells	0.722	3.9	0.0291	ENSSSCG00000026953	protein coding
ARF6	*TRIB1* + Satellite cells	0.724	7.31	0.00299	ENSSSCG00000005012	protein coding
BMPR2	*TRIB1* + Satellite cells	0.725	7.84	0.00283	ENSSSCG00000016113	protein coding
ARHGAP29	*TRIB1* + Satellite cells	0.726	4.14	0.0177	ENSSSCG00000006889	protein coding
ENSSSCG00000012452	*TRIB1* + Satellite cells	0.726	6.01	0.00163	ENSSSCG00000012452	protein coding
PRDX5	*TRIB1* + Satellite cells	0.727	6.06	0.0154	ENSSSCG00000013030	protein coding
ASAH1	*TRIB1* + Satellite cells	0.729	4.34	0.0113	ENSSSCG00000006995	protein coding
FDFT1	*TRIB1* + Satellite cells	0.729	5.15	0.0149	ENSSSCG00000026044	protein coding
MSTRG.25012	*TRIB1* + Satellite cells	0.73	5.08	0.0214	MSTRG.25012	lncRNA
NUP54	*TRIB1* + Satellite cells	0.73	5.13	0.00615	ENSSSCG00000008979	protein coding
CRK	*TRIB1* + Satellite cells	0.732	9.19	0.0019	ENSSSCG00000038989	protein coding
FADS3	*TRIB1* + Satellite cells	0.733	4.36	0.0276	ENSSSCG00000013073	protein coding
CBLB	*TRIB1* + Satellite cells	0.736	6.7	0.00149	ENSSSCG00000022486	protein coding
RNFT1	*TRIB1* + Satellite cells	0.738	5.19	0.00678	ENSSSCG00000017671	protein coding
CDC42EP4	*TRIB1* + Satellite cells	0.739	5.42	0.00445	ENSSSCG00000017245	protein coding
MFF	*TRIB1* + Satellite cells	0.742	6.8	0.00119	ENSSSCG00000031572	protein coding
IFNGR2	*TRIB1* + Satellite cells	0.746	5.2	0.00463	ENSSSCG00000034378	protein coding
PDPN	*TRIB1* + Satellite cells	0.746	6.22	0.00918	ENSSSCG00000003451	protein coding
TSPAN6	*TRIB1* + Satellite cells	0.748	6.2	0.00114	ENSSSCG00000012481	protein coding
PLAAT3	*TRIB1* + Satellite cells	0.749	4.74	0.0191	ENSSSCG00000026914	protein coding
RPL36A-HNRNPH2	*TRIB1* + Satellite cells	0.751	10.9	0.00288	ENSSSCG00000039046	protein coding
TOM1L1	*TRIB1* + Satellite cells	0.751	5.02	0.0128	ENSSSCG00000017601	protein coding
ENSSSCG00000037597	*TRIB1* + Satellite cells	0.752	4.1	0.0126	ENSSSCG00000037597	protein coding
MYD88	*TRIB1* + Satellite cells	0.752	4.38	0.014	ENSSSCG00000011251	protein coding
CCNC	*TRIB1* + Satellite cells	0.753	5.89	0.00191	ENSSSCG00000004353	protein coding
FAP	*TRIB1* + Satellite cells	0.753	5.27	0.0164	ENSSSCG00000015896	protein coding
MSTRG.43169	*TRIB1* + Satellite cells	0.753	7.46	0.00435	MSTRG.43169	lncRNA
FOLR2	*TRIB1* + Satellite cells	0.758	4.71	0.0436	ENSSSCG00000014812	protein coding
NCOA4	*TRIB1* + Satellite cells	0.758	5.49	0.00564	ENSSSCG00000010399	protein coding
TBCA	*TRIB1* + Satellite cells	0.758	8.04	0.0101	ENSSSCG00000014100	protein coding
BCR	*TRIB1* + Satellite cells	0.759	4.59	0.048	ENSSSCG00000010052	protein coding
CEP126	*TRIB1* + Satellite cells	0.76	4.43	0.0338	ENSSSCG00000014979	protein coding

ATG5	*TRIB1*$^{+}$ Satellite cells	0.763	5.14	0.00713	ENSSSCG00000004370	protein coding
ENSSSCG00000032235	*TRIB1*$^{+}$ Satellite cells	0.764	5.91	0.00541	ENSSSCG00000032235	protein coding
ENSSSCG00000040828	*TRIB1*$^{+}$ Satellite cells	0.765	3.98	0.0179	ENSSSCG00000040828	protein coding
MSTRG.33764	*TRIB1*$^{+}$ Satellite cells	0.765	4.79	0.0291	MSTRG.33764	lncRNA
BIRC2	*TRIB1*$^{+}$ Satellite cells	0.766	7.94	0.008	ENSSSCG00000027529	protein coding
LYPLA1	*TRIB1*$^{+}$ Satellite cells	0.769	5.33	0.00681	ENSSSCG00000006260	protein coding
ADD3	*TRIB1*$^{+}$ Satellite cells	0.77	4.51	0.0164	ENSSSCG00000010621	protein coding
MDFIC	*TRIB1*$^{+}$ Satellite cells	0.771	8.37	0.00778	ENSSSCG00000021885	protein coding
NQO2	*TRIB1*$^{+}$ Satellite cells	0.772	4.87	0.0354	ENSSSCG00000020758	protein coding
DPYD	*TRIB1*$^{+}$ Satellite cells	0.776	7.42	0.00695	ENSSSCG00000006878	protein coding
TFPI2	*TRIB1*$^{+}$ Satellite cells	0.778	6.46	0.0163	ENSSSCG00000015322	protein coding
ENSSSCG00000033786	*TRIB1*$^{+}$ Satellite cells	0.781	4.63	0.00716	ENSSSCG00000033786	protein coding
RAB4A	*TRIB1*$^{+}$ Satellite cells	0.781	4.04	0.00888	ENSSSCG00000036039	protein coding
MSTRG.49539	*TRIB1*$^{+}$ Satellite cells	0.783	6.6	0.0171	MSTRG.49539	lncRNA
FGFR1OP2	*TRIB1*$^{+}$ Satellite cells	0.784	4.14	0.045	ENSSSCG00000000554	protein coding
PLP2	*TRIB1*$^{+}$ Satellite cells	0.784	6.44	0.00978	ENSSSCG00000012294	protein coding
ENSSSCG00000038805	*TRIB1*$^{+}$ Satellite cells	0.786	3.96	0.019	ENSSSCG00000038805	protein coding
HSPH1	*TRIB1*$^{+}$ Satellite cells	0.787	7.54	0.00263	ENSSSCG00000009334	protein coding
NR4A3	*TRIB1*$^{+}$ Satellite cells	0.788	3.81	0.0279	ENSSSCG00000005385	protein coding
YPEL5	*TRIB1*$^{+}$ Satellite cells	0.789	5.27	0.00206	ENSSSCG00000008529	protein coding
TOLLIP	*TRIB1*$^{+}$ Satellite cells	0.79	6.03	0.0125	ENSSSCG00000033033	protein coding
ANXA2	*TRIB1*$^{+}$ Satellite cells	0.791	9.41	0.0029	ENSSSCG00000004578	protein coding
FREM1	*TRIB1*$^{+}$ Satellite cells	0.792	5.05	0.0111	ENSSSCG00000039025	protein coding
MSTRG.37309	*TRIB1*$^{+}$ Satellite cells	0.792	4.86	0.00808	MSTRG.37309	lncRNA
IPMK	*TRIB1*$^{+}$ Satellite cells	0.795	3.81	0.0206	ENSSSCG00000040863	protein coding
MCU	*TRIB1*$^{+}$ Satellite cells	0.796	9.41	0.0146	ENSSSCG00000010290	protein coding
BMPR1B	*TRIB1*$^{+}$ Satellite cells	0.797	4.6	0.0243	ENSSSCG00000029621	protein coding
SPATA1	*TRIB1*$^{+}$ Satellite cells	0.797	3.67	0.0157	ENSSSCG00000029882	protein coding
ANKRD28	*TRIB1*$^{+}$ Satellite cells	0.8	6.6	0.00117	ENSSSCG00000011194	protein coding
TMEM109	*TRIB1*$^{+}$ Satellite cells	0.8	4.63	0.026	ENSSSCG00000013110	protein coding
CCNI	*TRIB1*$^{+}$ Satellite cells	0.803	7.5	0.00295	ENSSSCG00000008987	protein coding
EIF2A	*TRIB1*$^{+}$ Satellite cells	0.807	6.4	0.0122	ENSSSCG00000011710	protein coding
MGAT5	*TRIB1*$^{+}$ Satellite cells	0.807	6.8	0.0134	ENSSSCG00000015701	protein coding
RNF11	*TRIB1*$^{+}$ Satellite cells	0.81	6.42	0.00712	ENSSSCG00000031172	protein coding

CBX3	*TRIB1*$^+$ Satellite cells	0.812	6.77	0.00142	ENSSSCG00000016711	protein coding
TRAM1	*TRIB1*$^+$ Satellite cells	0.812	6.57	0.0295	ENSSSCG00000006193	protein coding
GLMP	*TRIB1*$^+$ Satellite cells	0.813	4.24	0.0167	ENSSSCG00000006488	protein coding
MSTRG.11148	*TRIB1*$^+$ Satellite cells	0.813	5.59	0.0462	MSTRG.11148	lncRNA
ARL6	*TRIB1*$^+$ Satellite cells	0.814	3.7	0.0219	ENSSSCG00000027278	protein coding
MYF5	*TRIB1*$^+$ Satellite cells	0.814	10	0.00141	ENSSSCG00000000937	protein coding
MSTRG.32273	*TRIB1*$^+$ Satellite cells	0.817	4.4	0.0279	MSTRG.32273	lncRNA
ENSSSCG00000030849	*TRIB1*$^+$ Satellite cells	0.822	11.8	0.001	ENSSSCG00000030849	protein coding
MSTRG.20636	*TRIB1*$^+$ Satellite cells	0.824	3.8	0.0201	MSTRG.20636	lncRNA
TMEM59	*TRIB1*$^+$ Satellite cells	0.824	8.07	0.00387	ENSSSCG00000034261	protein coding
ZNF277	*TRIB1*$^+$ Satellite cells	0.824	6.86	0.00331	ENSSSCG00000025910	protein coding
RIDA	*TRIB1*$^+$ Satellite cells	0.825	5.21	0.00212	ENSSSCG00000006079	protein coding
NT5C3A	*TRIB1*$^+$ Satellite cells	0.83	4.81	0.0021	ENSSSCG00000022912	protein coding
POT1	*TRIB1*$^+$ Satellite cells	0.83	3.72	0.0236	ENSSSCG00000016597	protein coding
RHOBTB1	*TRIB1*$^+$ Satellite cells	0.83	3.78	0.0473	ENSSSCG00000028327	protein coding
PRKRA	*TRIB1*$^+$ Satellite cells	0.832	4.09	0.0112	ENSSSCG00000038254	protein coding
RAD9A	*TRIB1*$^+$ Satellite cells	0.832	3.71	0.0203	ENSSSCG00000012914	protein coding
MSTRG.46073	*TRIB1*$^+$ Satellite cells	0.834	3.78	0.0196	MSTRG.46073	lncRNA
MSTRG.23990	*TRIB1*$^+$ Satellite cells	0.837	3.79	0.0236	MSTRG.23990	lncRNA
ENSSSCG00000039071	*TRIB1*$^+$ Satellite cells	0.841	6.22	0.0459	ENSSSCG00000039071	protein coding
EMILIN2	*TRIB1*$^+$ Satellite cells	0.842	6.7	0.00367	ENSSSCG00000003694	protein coding
GCNT1	*TRIB1*$^+$ Satellite cells	0.843	5.45	0.00401	ENSSSCG00000005277	protein coding
MSTRG.22327	*TRIB1*$^+$ Satellite cells	0.846	4.86	0.0121	MSTRG.22327	lncRNA
MSTRG.48570	*TRIB1*$^+$ Satellite cells	0.846	3.83	0.04	MSTRG.48570	lncRNA
CHURC1	*TRIB1*$^+$ Satellite cells	0.847	4.59	0.00396	ENSSSCG00000022147	protein coding
MSTRG.17842	*TRIB1*$^+$ Satellite cells	0.847	4.96	0.0256	MSTRG.17842	lncRNA
RASGEF1B	*TRIB1*$^+$ Satellite cells	0.857	5.32	0.0232	ENSSSCG00000029507	protein coding
MSTRG.10687	*TRIB1*$^+$ Satellite cells	0.858	4.02	0.0284	MSTRG.10687	lncRNA
GMFB	*TRIB1*$^+$ Satellite cells	0.859	4.44	0.0318	ENSSSCG00000026294	protein coding
CDC42EP5	*TRIB1*$^+$ Satellite cells	0.86	7.24	0.0278	ENSSSCG00000039922	protein coding
C15orf40	*TRIB1*$^+$ Satellite cells	0.863	5.67	0.00196	ENSSSCG00000030131	protein coding
IDI1	*TRIB1*$^+$ Satellite cells	0.863	5.07	0.0135	ENSSSCG00000029066	protein coding
SCLY	*TRIB1*$^+$ Satellite cells	0.863	4.86	0.0114	ENSSSCG00000016334	protein coding
ZSWIM4	*TRIB1*$^+$ Satellite cells	0.874	5.56	0.0116	ENSSSCG00000013758	protein coding

CFL2	*TRIB1*$^+$ Satellite cells	0.879	7.56	0.000633	ENSSSCG00000001959	protein coding
MSTRG.40007	*TRIB1*$^+$ Satellite cells	0.881	5.28	0.0103	MSTRG.40007	lncRNA
RNF150	*TRIB1*$^+$ Satellite cells	0.881	7.79	0.00478	ENSSSCG00000009053	protein coding
MSTRG.11887	*TRIB1*$^+$ Satellite cells	0.892	5.55	0.00974	MSTRG.11887	lncRNA
ENSSSCG00000037566	*TRIB1*$^+$ Satellite cells	0.897	5.26	0.0168	ENSSSCG00000037566	protein coding
MSTRG.31671	*TRIB1*$^+$ Satellite cells	0.897	4.82	0.00254	MSTRG.31671	lncRNA
MSTRG.15926	*TRIB1*$^+$ Satellite cells	0.9	6.26	0.00246	MSTRG.15926	lncRNA
ENSSSCG00000031288	*TRIB1*$^+$ Satellite cells	0.901	3.64	0.0168	ENSSSCG00000031288	protein coding
MSTRG.36323	*TRIB1*$^+$ Satellite cells	0.901	5.31	0.00531	MSTRG.36323	lncRNA
CLCN5	*TRIB1*$^+$ Satellite cells	0.909	8.42	0.00461	ENSSSCG00000023026	protein coding
AGTPBP1	*TRIB1*$^+$ Satellite cells	0.91	4.17	0.0205	ENSSSCG00000010957	protein coding
MSTRG.1625	*TRIB1*$^+$ Satellite cells	0.919	5.2	0.0275	MSTRG.1625	lncRNA
MFGE8	*TRIB1*$^+$ Satellite cells	0.92	4.77	0.00907	ENSSSCG00000001834	protein coding
TMSB10	*TRIB1*$^+$ Satellite cells	0.92	9.7	0.00788	ENSSSCG00000008245	protein coding
SLC43A3	*TRIB1*$^+$ Satellite cells	0.922	5.17	0.00844	ENSSSCG00000033998	protein coding
MSTRG.20986	*TRIB1*$^+$ Satellite cells	0.924	3.85	0.00772	MSTRG.20986	lncRNA
RAVER2	*TRIB1*$^+$ Satellite cells	0.924	4.03	0.00627	ENSSSCG00000025672	protein coding
TWSG1	*TRIB1*$^+$ Satellite cells	0.925	5.87	0.00471	ENSSSCG00000039663	protein coding
ENSSSCG00000037595	*TRIB1*$^+$ Satellite cells	0.928	6.1	0.000771	ENSSSCG00000037595	protein coding
SCN7A	*TRIB1*$^+$ Satellite cells	0.929	5.77	0.00452	ENSSSCG00000015914	protein coding
ENSSSCG00000004666	*TRIB1*$^+$ Satellite cells	0.93	3.97	0.0338	ENSSSCG00000004666	protein coding
CYP51A1	*TRIB1*$^+$ Satellite cells	0.933	4.56	0.0287	ENSSSCG00000015311	protein coding
ENSSSCG00000009208	*TRIB1*$^+$ Satellite cells	0.933	6.28	0.000763	ENSSSCG00000009208	protein coding
BAG2	*TRIB1*$^+$ Satellite cells	0.938	5.05	0.0132	ENSSSCG00000033526	protein coding
XRCC6	*TRIB1*$^+$ Satellite cells	0.941	4.52	0.0039	ENSSSCG00000000059	protein coding
DNAJB1	*TRIB1*$^+$ Satellite cells	0.948	8.44	0.00912	ENSSSCG00000013784	protein coding
MSTRG.11460	*TRIB1*$^+$ Satellite cells	0.949	4.2	0.0244	MSTRG.11460	lncRNA
MSTRG.27753	*TRIB1*$^+$ Satellite cells	0.95	4.49	0.00126	MSTRG.27753	lncRNA
SELENBP1	*TRIB1*$^+$ Satellite cells	0.953	6.02	0.0107	ENSSSCG00000006624	protein coding
VGLL2	*TRIB1*$^+$ Satellite cells	0.955	4.57	0.00619	ENSSSCG00000004255	protein coding
IL18	*TRIB1*$^+$ Satellite cells	0.957	3.96	0.0265	ENSSSCG00000015037	protein coding
MSTRG.10966	*TRIB1*$^+$ Satellite cells	0.957	4.11	0.0147	MSTRG.10966	lncRNA
MSTRG.44698	*TRIB1*$^+$ Satellite cells	0.962	5.5	0.00116	MSTRG.44698	lncRNA
FAM227B	*TRIB1*$^+$ Satellite cells	0.964	4.32	0.0024	ENSSSCG00000004648	protein coding

SELENOM	*TRIB1* +	Satellite cells	0.968	5.38	0.0289	ENSSSCG00000029781	protein coding
GLI3	*TRIB1* +	Satellite cells	0.969	4.28	0.0218	ENSSSCG00000016763	protein coding
MSTRG.8502	*TRIB1* +	Satellite cells	0.973	4.09	0.0033	MSTRG.8502	lncRNA
FJX1	*TRIB1* +	Satellite cells	0.974	3.98	0.0133	ENSSSCG00000025588	protein coding
LRRTM3	*TRIB1* +	Satellite cells	0.98	5.25	0.0217	ENSSSCG00000010232	protein coding
ELL2	*TRIB1* +	Satellite cells	0.984	6.87	0.000405	ENSSSCG00000014168	protein coding
MSTRG.43168	*TRIB1* +	Satellite cells	0.993	5.7	0.0197	MSTRG.43168	lncRNA
MSTRG.180	*TRIB1* +	Satellite cells	0.996	7.61	0.00113	MSTRG.180	lncRNA
IMMP2L	*TRIB1* +	Satellite cells	0.997	7.45	0.00954	ENSSSCG00000016650	protein coding
CTNNA3	*TRIB1* +	Satellite cells	1	4.03	0.0127	ENSSSCG00000036480	protein coding
P3H2	*TRIB1* +	Satellite cells	1.01	7.4	0.0113	ENSSSCG00000011813	protein coding
ENSSSCG00000009125	*TRIB1* +	Satellite cells	1.02	8.74	0.00112	ENSSSCG00000009125	protein coding
TANC1	*TRIB1* +	Satellite cells	1.02	7.65	0.00184	ENSSSCG00000015880	protein coding
BACH2	*TRIB1* +	Satellite cells	1.03	8.49	0.0315	ENSSSCG00000004332	protein coding
TUBB6	*TRIB1* +	Satellite cells	1.03	6.45	0.0062	ENSSSCG00000038992	protein coding
NEGR1	*TRIB1* +	Satellite cells	1.04	6.53	0.0224	ENSSSCG00000025085	protein coding
NRAS	*TRIB1* +	Satellite cells	1.04	4.74	0.00321	ENSSSCG00000032477	protein coding
MAT2B	*TRIB1* +	Satellite cells	1.05	4.72	0.0372	ENSSSCG00000021229	protein coding
MSTRG.4110	*TRIB1* +	Satellite cells	1.05	6.06	0.0025	MSTRG.4110	lncRNA
CCNG1	*TRIB1* +	Satellite cells	1.07	7.69	0.000106	ENSSSCG00000017024	protein coding
INPP1	*TRIB1* +	Satellite cells	1.07	4.47	0.00419	ENSSSCG00000016050	protein coding
ENSSSCG00000006395	*TRIB1* +	Satellite cells	1.08	8.03	0.00299	ENSSSCG00000006395	protein coding
ENSSSCG00000016823	*TRIB1* +	Satellite cells	1.08	8.06	0.00267	ENSSSCG00000016823	protein coding
GIPC1	*TRIB1* +	Satellite cells	1.08	4.9	0.0138	ENSSSCG00000032842	protein coding
MSTRG.32310	*TRIB1* +	Satellite cells	1.08	5.02	0.0406	MSTRG.32310	lncRNA
MSTRG.33247	*TRIB1* +	Satellite cells	1.08	3.58	0.0215	MSTRG.33247	lncRNA
MSTRG.42487	*TRIB1* +	Satellite cells	1.08	3.76	0.0121	MSTRG.42487	lncRNA
DUT	*TRIB1* +	Satellite cells	1.09	5.58	0.0291	ENSSSCG00000032141	protein coding
ENSSSCG00000044542	*TRIB1* +	Satellite cells	1.09	5.98	0.00596	ENSSSCG00000044542	protein coding
LARP6	*TRIB1* +	Satellite cells	1.09	5.18	0.000301	ENSSSCG00000004974	protein coding
MSTRG.3143	*TRIB1* +	Satellite cells	1.09	4.1	0.0387	MSTRG.3143	lncRNA
SAMD4A	*TRIB1* +	Satellite cells	1.09	8.81	0.00387	ENSSSCG00000023408	protein coding
MSTRG.45497	*TRIB1* +	Satellite cells	1.11	3.93	0.00461	MSTRG.45497	lncRNA
PDE4B	*TRIB1* +	Satellite cells	1.11	3.97	0.00488	ENSSSCG00000003805	protein coding

MSTRG.17830	$TRIB1^+$	Satellite cells	1.12	5.27	0.0183	MSTRG.17830	lncRNA
SERPINE2	$TRIB1^+$	Satellite cells	1.12	3.63	0.0278	ENSSSCG00000016233	protein coding
ATG12	$TRIB1^+$	Satellite cells	1.13	5	0.00162	ENSSSCG00000033912	protein coding
MSTRG.25438	$TRIB1^+$	Satellite cells	1.13	4.66	0.00226	MSTRG.25438	lncRNA
NUDT15	$TRIB1^+$	Satellite cells	1.13	3.53	0.00735	ENSSSCG00000027551	protein coding
SLC35E3	$TRIB1^+$	Satellite cells	1.13	3.54	0.00657	ENSSSCG00000000487	protein coding
ITGA2	$TRIB1^+$	Satellite cells	1.14	3.6	0.00681	ENSSSCG00000016887	protein coding
MSTRG.17815	$TRIB1^+$	Satellite cells	1.14	4.67	0.0351	MSTRG.17815	lncRNA
MSTRG.2262	$TRIB1^+$	Satellite cells	1.15	4.81	0.00165	MSTRG.2262	lncRNA
MSTRG.14019	$TRIB1^+$	Satellite cells	1.17	4.91	0.018	MSTRG.14019	lncRNA
MSTRG.17548	$TRIB1^+$	Satellite cells	1.17	4.44	0.00327	MSTRG.17548	lncRNA
MSTRG.3582	$TRIB1^+$	Satellite cells	1.17	4.28	0.00249	MSTRG.3582	lncRNA
DDAH2	$TRIB1^+$	Satellite cells	1.19	5.7	0.0357	ENSSSCG00000038514	protein coding
INSIG1	$TRIB1^+$	Satellite cells	1.19	4.37	0.0397	ENSSSCG00000016420	protein coding
KCNJ13	$TRIB1^+$	Satellite cells	1.2	4.09	0.00324	ENSSSCG00000025038	protein coding
MSTRG.28657	$TRIB1^+$	Satellite cells	1.21	4.28	0.0027	MSTRG.28657	lncRNA
MSTRG.31458	$TRIB1^+$	Satellite cells	1.21	4.7	0.0117	MSTRG.31458	lncRNA
SELENOW	$TRIB1^+$	Satellite cells	1.21	6.46	0.00011	ENSSSCG00000034313	protein coding
MSTRG.161	$TRIB1^+$	Satellite cells	1.22	7.67	0.00252	MSTRG.161	lncRNA
MSTRG.30533	$TRIB1^+$	Satellite cells	1.22	4.01	0.000677	MSTRG.30533	lncRNA
MSTRG.39844	$TRIB1^+$	Satellite cells	1.23	3.43	0.00533	MSTRG.39844	lncRNA
MSTRG.22342	$TRIB1^+$	Satellite cells	1.24	6.34	0.0121	MSTRG.22342	lncRNA
MSMO1	$TRIB1^+$	Satellite cells	1.25	5.16	0.0149	ENSSSCG00000008857	protein coding
TRIM16	$TRIB1^+$	Satellite cells	1.25	4.6	0.00177	ENSSSCG00000034049	protein coding
LITAF	$TRIB1^+$	Satellite cells	1.26	7.64	0.00116	ENSSSCG00000032381	protein coding
MSTRG.7224	$TRIB1^+$	Satellite cells	1.26	4.31	0.00253	MSTRG.7224	lncRNA
SLC10A1	$TRIB1^+$	Satellite cells	1.29	4.59	0.00733	ENSSSCG00000002313	protein coding
MSTRG.26567	$TRIB1^+$	Satellite cells	1.3	3.87	0.0016	MSTRG.26567	lncRNA
MSTRG.39442	$TRIB1^+$	Satellite cells	1.3	3.54	0.00381	MSTRG.39442	lncRNA
MSTRG.9045	$TRIB1^+$	Satellite cells	1.3	4.82	0.00458	MSTRG.9045	lncRNA
ANKRD44	$TRIB1^+$	Satellite cells	1.31	4.09	0.00345	ENSSSCG00000016074	protein coding
PLCB1	$TRIB1^+$	Satellite cells	1.32	4.86	0.0133	ENSSSCG00000007056	protein coding
ST8SIA4	$TRIB1^+$	Satellite cells	1.32	5.01	0.000607	ENSSSCG00000035539	protein coding
ENSSSCG00000021576	$TRIB1^+$	Satellite cells	1.34	3.84	0.0176	ENSSSCG00000021576	protein coding

CCL2	*TRIB1*$^{+}$ Satellite cells	1.35	5.45	0.000101	ENSSSCG00000017723	protein coding
ENSSSCG00000039009	*TRIB1*$^{+}$ Satellite cells	1.35	3.52	0.00566	ENSSSCG00000039009	protein coding
MSTRG.19258	*TRIB1*$^{+}$ Satellite cells	1.35	4.26	0.000846	MSTRG.19258	lncRNA
MSTRG.26379	*TRIB1*$^{+}$ Satellite cells	1.35	4.89	0.00281	MSTRG.26379	lncRNA
CCDC146	*TRIB1*$^{+}$ Satellite cells	1.37	4.08	0.0126	ENSSSCG00000015412	protein coding
SELENOP	*TRIB1*$^{+}$ Satellite cells	1.4	8.45	7.84E-05	ENSSSCG00000021208	protein coding
DAB2	*TRIB1*$^{+}$ Satellite cells	1.41	4.12	0.00263	ENSSSCG00000016857	protein coding
SGMS2	*TRIB1*$^{+}$ Satellite cells	1.41	6.16	9.43E-05	ENSSSCG00000009152	protein coding
ENSSSCG00000045666	*TRIB1*$^{+}$ Satellite cells	1.42	6.47	0.0032	ENSSSCG00000045666	lncRNA
MSTRG.18663	*TRIB1*$^{+}$ Satellite cells	1.42	5.05	0.000666	MSTRG.18663	lncRNA
ZNF385B	*TRIB1*$^{+}$ Satellite cells	1.42	4.48	0.0222	ENSSSCG00000023181	protein coding
MSTRG.4983	*TRIB1*$^{+}$ Satellite cells	1.43	4.38	0.000889	MSTRG.4983	lncRNA
SLC8A1	*TRIB1*$^{+}$ Satellite cells	1.44	4.92	0.019	ENSSSCG00000036418	protein coding
MSTRG.13060	*TRIB1*$^{+}$ Satellite cells	1.47	4.11	0.00655	MSTRG.13060	lncRNA
NUPR1	*TRIB1*$^{+}$ Satellite cells	1.47	4.69	0.00949	ENSSSCG00000040162	protein coding
PLAU	*TRIB1*$^{+}$ Satellite cells	1.47	5.8	0.00984	ENSSSCG00000010312	protein coding
ENSSSCG00000046277	*TRIB1*$^{+}$ Satellite cells	1.48	3.83	0.015	ENSSSCG00000046277	lncRNA
MSTRG.27444	*TRIB1*$^{+}$ Satellite cells	1.49	5.19	0.000601	MSTRG.27444	lncRNA
ENSSSCG00000028695	*TRIB1*$^{+}$ Satellite cells	1.5	5.14	0.00388	ENSSSCG00000028695	lncRNA
MSTRG.25395	*TRIB1*$^{+}$ Satellite cells	1.51	5.02	0.000817	MSTRG.25395	lncRNA
MSTRG.39989	*TRIB1*$^{+}$ Satellite cells	1.51	4.81	0.0462	MSTRG.39989	lncRNA
DACH1	*TRIB1*$^{+}$ Satellite cells	1.55	4.5	0.026	ENSSSCG00000009457	protein coding
FAM89A	*TRIB1*$^{+}$ Satellite cells	1.55	4.04	0.00774	ENSSSCG00000038765	protein coding
MSTRG.13235	*TRIB1*$^{+}$ Satellite cells	1.55	4.76	0.0226	MSTRG.13235	lncRNA
CXCL2	*TRIB1*$^{+}$ Satellite cells	1.59	6.65	0.00394	ENSSSCG00000008959	protein coding
ENSSSCG00000041954	*TRIB1*$^{+}$ Satellite cells	1.59	3.78	0.00072	ENSSSCG00000041954	protein coding
MSTRG.17825	*TRIB1*$^{+}$ Satellite cells	1.62	3.89	0.0109	MSTRG.17825	lncRNA
TIMP1	*TRIB1*$^{+}$ Satellite cells	1.62	8.59	5.89E-05	ENSSSCG00000012277	protein coding
SLCO5A1	*TRIB1*$^{+}$ Satellite cells	1.64	4.03	0.00636	ENSSSCG00000006196	protein coding
MSTRG.32351	*TRIB1*$^{+}$ Satellite cells	1.67	5.17	0.00196	MSTRG.32351	lncRNA
MSTRG.27574	*TRIB1*$^{+}$ Satellite cells	1.68	3.94	0.000463	MSTRG.27574	lncRNA
MSTRG.37096	*TRIB1*$^{+}$ Satellite cells	1.72	3.42	0.00473	MSTRG.37096	lncRNA
MSTRG.8106	*TRIB1*$^{+}$ Satellite cells	1.72	3.9	0.00288	MSTRG.8106	lncRNA
ENSSSCG00000042185	*TRIB1*$^{+}$ Satellite cells	1.77	3.25	0.00725	ENSSSCG00000042185	lncRNA

LUM	*TRIB1*$^{+}$ Satellite cells	1.77	7.44	0.0427	ENSSSCG00000000916	protein coding
APOD	*TRIB1*$^{+}$ Satellite cells	1.81	4.87	0.00417	ENSSSCG00000011831	protein coding
ENSSSCG00000029160	*TRIB1*$^{+}$ Satellite cells	1.82	9.98	0.0057	ENSSSCG00000029160	protein coding
MSTRG.41032	*TRIB1*$^{+}$ Satellite cells	1.85	3.31	0.0266	MSTRG.41032	lncRNA
PHGDH	*TRIB1*$^{+}$ Satellite cells	1.9	5.05	0.0331	ENSSSCG00000006717	protein coding
MSTRG.38467	*TRIB1*$^{+}$ Satellite cells	1.94	8.63	0.0276	MSTRG.38467	lncRNA
MSTRG.31456	*TRIB1*$^{+}$ Satellite cells	1.99	3.75	9.89E-05	MSTRG.31456	lncRNA
CD36	*TRIB1*$^{+}$ Satellite cells	2.02	3.73	0.000262	ENSSSCG00000015405	protein coding
MSTRG.42632	*TRIB1*$^{+}$ Satellite cells	2.04	6.39	0.0293	MSTRG.42632	lncRNA
MSTRG.41836	*TRIB1*$^{+}$ Satellite cells	2.2	3.21	0.000215	MSTRG.41836	lncRNA
ENSSSCG00000039222	*TRIB1*$^{+}$ Satellite cells	2.23	4.2	0.00266	ENSSSCG00000039222	protein coding
VCAM1	*TRIB1*$^{+}$ Satellite cells	2.27	3.49	0.00596	ENSSSCG00000006862	protein coding
MSTRG.28362	*TRIB1*$^{+}$ Satellite cells	2.3	4.89	0.0368	MSTRG.28362	lncRNA
MSTRG.8600	*TRIB1*$^{+}$ Satellite cells	2.3	5.46	0.0111	MSTRG.8600	lncRNA
MSTRG.882	*TRIB1*$^{+}$ Satellite cells	2.32	4.47	0.0396	MSTRG.882	lncRNA
ENSSSCG00000037324	*TRIB1*$^{+}$ Satellite cells	2.39	3.88	0.000372	ENSSSCG00000037324	lncRNA
MSTRG.43238	*TRIB1*$^{+}$ Satellite cells	2.42	3.37	0.0301	MSTRG.43238	lncRNA
CDCP1	*TRIB1*$^{+}$ Satellite cells	2.58	3.99	4.73E-05	ENSSSCG00000011298	protein coding
MSTRG.23839	*TRIB1*$^{+}$ Satellite cells	2.64	3.91	0.000129	MSTRG.23839	lncRNA
ENSSSCG00000029003	*TRIB1*$^{+}$ Satellite cells	3.35	4.23	0.0146	ENSSSCG00000029003	protein coding
PSAT1	*TRIB1*$^{+}$ Satellite cells	3.46	5.45	0.00147	ENSSSCG00000005287	protein coding
MSTRG.29989	*TRIB1*$^{+}$ Satellite cells	3.53	5.56	0.0149	MSTRG.29989	lncRNA
MSTRG.38853	*TRIB1*$^{+}$ Satellite cells	3.62	7.81	0.0102	MSTRG.38853	lncRNA
MGST2	*TRIB1*$^{+}$ Satellite cells	3.69	3.32	0.00095	ENSSSCG00000031736	protein coding
MSTRG.11119	*TRIB1*$^{+}$ Satellite cells	4.89	5.22	0.00232	MSTRG.11119	lncRNA
